# NKX6-3 in B-Cell Progenitor Differentiation and Leukemia

**DOI:** 10.3390/genes16101199

**Published:** 2025-10-14

**Authors:** Stefan Nagel, Corinna Meyer, Claudia Pommerenke

**Affiliations:** Department of Human and Animal Cell Lines, Leibniz-Institute DSMZ, 38124 Braunschweig, Germany

**Keywords:** B-ALL, ETS-code, GP5, NKL-code, TALE-code, TEAD, TGFBR2

## Abstract

Early B-cell development is primarily regulated at the transcriptional level and comprises the consecutive differentiation stages B-cell progenitor, pro-B-cell and pre-B-cell. These entities provide the cells of origin in B-cell precursor acute lymphoid leukemia (BCP-ALL) that show aberrations of developmental transcription factors (TFs), representing major oncogenic drivers. Analysis of physiological TFs in these developmental entities helps us to understand their normal and disturbed activities and regulatory connections. Here, we focused on NKL-subclass homeodomain TF NKX6-3, which is active in both normal B-cell progenitors and TCF3::PBX1 fusion gene-positive BCP-ALL cases. By performing siRNA-mediated knockdown and forced expression experiments in BCP-ALL model cell lines, we established a gene regulatory network for NKX6-3 together with TALE-class homeodomain TFs IRX1 and MEIS1, as well as ETS-TF SPIB. Importantly, NKX6-3 was activated by TCF3::PBX1, underlying their co-expression in BCP-ALL. Furthermore, comparative expression profiling analysis of public BCP-ALL patient data revealed TGFb-pathway in-hibitor CD109 as a downregulated target gene of NKX6-3. TGFb-signalling, in turn, enhanced NKX6-3 expression, indicating mutual activation. Finally, RNA-seq analysis of BCP-ALL cell line RCH-ACV after NKX6-3 knockdown revealed MPP7 as an upregulated target gene of both NKX6-3 and TCF3::PBX1, revealing a role for the HIPPO-pathway in B-cell progenitors and TCF3::PBX1-positive BCP-ALL. Collectively, our data introduce novel players and regulatory connections to normal and aberrant TF-networks in B-cell progenitors to serve as potential diagnostic markers or therapeutic targets.

## 1. Introduction

Mature B-cells are important players in the adaptive immune system via their expression of antigen-specific receptors or secreted antibodies. B-cell production occurs during the whole lifetime in primary and secondary lymphoid organs and is, accordingly, separated into early and late stages. In addition to the B-cell lineage, the common lymphoid progenitor (CLP) generates those for T-cells, NK-cells and innate lymphoid cells. The sequential early stages of B-cell development comprise B-cell progenitor (BCP), pro-B-cell and pre-B-cell, and are located in the bone marrow, while in the late stages, naïve B-cell and germinal centre (GC) B-cells populate the lymph nodes and spleen. Mature B-cells are represented by memory B-cells and plasma cells and remain in secondary lymphoid organs or travel in the peripheral blood stream.

Hematopoiesis including B-cell differentiation is primarily regulated at the transcriptional level [1,2]. Moreover, combinations of specific transcription factors (TFs) create regulatory networks, which orchestrate differentiation processes, including the co-regulatory B-cell “master” TFs EBF1, PAX5 and TCF3 [3,4,5]. PAX5 is a Paired (PRD)-class homeobox gene, which generally encode developmental TFs [6]. Additional homeobox genes involved in B-cell development are TALE-class members PBX1, MEIS1 and IRX1 and NKL-subclass members HLX and NKX6-3 [6,7,8,9,10]. To systematize TFs active in hematopoiesis including B-cell development, we have established the concept of “TF-codes”. These codes describe all members of selected TF-classes/subclasses expressed in developing and mature entities. Accordingly, we described the NKL-code for NKL-subclass homeodomain TFs, the TALE-code for TALE-class homeodomain TFs, and the ETS-code for ETS TFs [7,8,9,10,11]. For each developmental stage, these codes show the typically expressed TFs, thus serving as a platform to screen and evaluate physiological versus aberrant TF activities in both normal and malignant B-cell development [12].

Gene mutations and/or chromosomal aberrations may alter TF-encoding genes and disturb hematopoietic differentiation processes, resulting in developmental arrest at particular stages and consequently in the generation of leukemia/lymphoma [4,13,14,15]. Accordingly, B-cell precursor acute lymphoid leukemia (BCP-ALL) is derived from early stages of developing B-cells, including B-cell progenitor (BCP), pro-B-cells and pre-B-cells. Moreover, BCP-ALL falls into several subtypes, each with specific gene expression signatures, gene mutations and/or fusion genes [16,17,18,19]. The latter represent the most important diagnostic markers in the clinic [20]. Fusion genes are generated by specific chromosomal rearrangements, notably t(9;22)(q34;q11) with BCR::ABL1, t(1;19)(q23;p13) with TCF3::PBX1, t(17;19)(q22;p13) with TCF3::HLF, t(12;21)(p13;q22) with ETV6::RUNX1 and those targeting the gene KMT2A/MLL1 at 11p13, which is fused to different partners (KMT2A::r). Of note, one of the main activated oncogenic targets of KMT2A-fusion proteins is TALE-class homeobox gene MEIS1, whose physiological expression is restricted to pro-B-cells during B-cell development [21,22]. Thus, knowledge of normal and aberrant TF activities may illuminate both physiological B-cell development and the mechanisms of B-cell leukemogenesis.

Here, we analyzed the role of NKL homeobox gene NKX6-3 in normal and aberrant B-cell development. NKX6-3 is physiologically expressed in BCPs and associated with the TCF3-rearranged BCP-ALL subtype. Our data show that NKX6-3 creates a network with specific TFs active in early B-cell development. Additional downstream analyses indicated regulatory connections with the TGFb- and HIPPO-pathways, which may open novel diagnostic and therapeutic routes.

## 2. Materials and Methods

### 2.1. Bioinformatic Analyses

Datasets GSE79533 and GSE13576 containing array-based gene expression profiling data from pediatric BCP-ALL patients were obtained from the Gene Expression Omnibus (GEO, www.ncbi.nlm.nih.gov, accessed on 1 October 2025) [23,24]. In addition, we used the associated R-based online tool GEOR, generating the statistically most significant top 250 differently expressed genes, to compare two selected patient groups [25]. RNA-seq data for normal human tissues and cell lines were obtained from the Human Protein Atlas (www.proteinatlas.org, accessed on 1 April 2025) [26].

Direct TF binding was analyzed using ChIP-seq data for TCF3 from the ENCODE project (accessed on 1 April 2025, www.genome.gov). For the identification of potential NKX6-3 binding sites at the TCF3 locus, we used the CIS-BP database (www.cisbp.ccbr.utoronto.ca/index.php) and the UCSC genome browser (www.genome.cse.ucsc.edu), accessed on 1 April 2025 [27].

RNA-seq data from knockdown-treated cell line RCH-ACV were generated commercially. The RNA quality was determined using the Agilent 2100 Bioanalyzer (Agilent Technologies, Waldbronn, Germany), revealing RIN values of 10.0. Sample libraries for control and treated cells were prepared with the strand-specific cDNA library and sequenced 2 × 150 bp by Eurofins Genomics on the Illumina NovaSeq 6000 platform (INVIEW Transcriptome, Ebersberg, Germany) by aiming for a minimum of 30 M reads per sample with an insert size of >150 bp in order to reduce overlapping paired-end read sequences [28]. The obtained reads were 52.6 M for RCH-ACVsiCTR and 44.7 M for RCH-ACVsiNKX6-3. Additional quality control metrics are given in supplemented fastp reports (Figures S1 and S2). Trimming and quality control of the sequencing reads were performed using fastp [29], and quantification of the reads via salmon on the human reference genode GRCh38, version 42 [30,31]. Finally, data were analyzed with DESeq2 and R/Bioconductor [32]. The data are deposited at ArrayExpress (Available online: www.ebi.ac.uk/biostudies/arrayexpress, accessed on 3 July 2025) and available via E-MTAB-15340. Gene set annotation analysis was performed using the online tool DAVID v2025_1 (www.david.abcc.ncifcrf.gov) (accessed on 29 September 2025) [33].

### 2.2. Cell Lines and Treatments

Cell lines used in this study are held at DSMZ (Braunschweig, Germany). Information concerning cultivation, classification and karyotype is given on the website (www.DSMZ.de). All cell lines had been authenticated and tested negative for mycoplasma infection. Gene-specific siRNA oligonucleotides were used to repress gene expression levels with reference to AllStars Negative Control siRNA (siCTR) obtained from Qiagen (Hilden, Germany). Each gene was repressed, using two different siRNAs. For overexpression studies we used the following commercial cDNAs: NKX6-3, MEIS1 and SPIB. They were cloned into pCMV6 vectors and obtained from Origene (Wiesbaden, Germany). To target a regulatory binding site for NKX6-3, we used phosphorothioate (PTO)-modified oligonucleotide PTO-N1 5′-GCCTGGCCGCCTCATTAACTTTTTAA-3′ and the complementary oligonucleotide PTO-N2 5′-TTAAAAAGTTAATGAGGCGGCCAGGC-3′. As control we used the reported oligonucleotide No. 4 [34]: PTO-41 5′-TAGAAGCCCTAGCCAGGACTAGCACA-3′ and its complement PTO-42 5′-TGTGCTAGTCCTGGCTAGGGCTTCTA-3′. SiRNAs (100 pmol), expression vectors (2 µg) and PTOs (20 µmol) were transfected into 1 × 10^6^ cells by electroporation using the EPI-2500 impulse generator (Fischer, Heidelberg, Germany) at 350 V for 10 ms. After 20 h cultivation, electroporated cells were harvested. Cell lines were stimulated with 20 ng/mL TGFb (R&D Systems, Wiesbaden, Germany) for 20 h.

Functional tests were performed with the IncuCyte S3 Live-Cell Imaging Analysis System (Sartorius, Göttingen, Germany). Apoptosis was induced by treatment with 100 µM etoposide dissolved in DMSO (Sigma-Aldrich, Taufkirchen, Germany). Apoptotic cells were detected using the IncuCyte Caspase-3/7 Green Apoptosis Assay diluted at 1:2000 (Sartorius). Live-cell imaging experiments were performed in biological triplicates.

### 2.3. Polymerase Chain Reaction (PCR) Analyses

For the detection of fusion transcripts, we performed reverse transcription (RT) PCR, using oligonucleotides, as reported previously [35]. As the positive control we analyzed ETV6, using the following oligonucleotides: ETV6-for 5′-AGGCCAATTGACAGCAACAC-3′ and ETV6-rev 5′-TGCACATTATCCACGGATGG-3′. All oligonucleotides were obtained from Eurofins MWG (Ebersberg, Germany). PCR products were generated using taqpol (Qiagen) and thermocycler TGradient (Biometra, Göttingen, Germany), analyzed by gel electrophoresis and documented with the Azure c200 Gel Imaging System (Azure Biosystems, Dublin, CA, USA).

TRIzol reagent (Invitrogen, Darmstadt, Germany) or RNeasy Plus extraction kit (Qiagen) were used to extract total RNA from cultivated and treated cells. cDNA was synthesized using 1 µg RNA, random priming and Superscript II (Invitrogen). Real-time quantitative (RQ) PCR analysis was performed using the 7500 Real-time System and commercial buffer and primer sets (Applied Biosystems/Life Technologies, Darmstadt, Germany). For normalization of expression levels, we quantified the RNA transcripts of the TBP gene, which has been reported as a suitable reference [36,37]. The following primer sets were used: CD109 (Hs00370347_m1), GP5 (Hs03027242_s1), IRX1 (Hs00411782_m1), MEIS1 (Hs01017441_m1), MPP7 (Hs00399584_m1), NKX6-3 (Hs04190028_g1), PBX1 (Hs00231228_m1), SMAD4 (Hs00929647-m1), SPIB (Hs01548149_m1), TCF3 (Hs01012685_m1), TBP (Hs00427620_m1) and TGFBR2 (Hs00234253_m1).

For the quantification of genomic copy numbers for NKX6-3 we used the following oligonucleotides: NKX6-3-1 5′-CGAGTACAACAAGCCGCTGGACC-3′ and NKX6-3-2 5′-ACGGCGGGCGTCAGACGCTGTG-3′. The MEF2C locus was used as control: MEF2C-1 5′-AGAAGGCTTATGAGCTGAGC-3′ and MEF2C-2 5′-AGACTGGCATCTCGAAGTTG-3′. Genomic DNA was prepared using the Qiagen Gentra Puregene Kit (Qiagen).

Quantitative analyses were performed as biological replicates or triplicates as indicated in the figure legends, and measured in triplicate. Standard deviations are presented in the figures as error bars. Statistical significance was assessed by t-test (two-tailed), and the calculated *p*-values are indicated by asterisks (* *p* < 0.05, ** *p* < 0.01, *** *p* < 0.001, n.s. not significant).

### 2.4. Genomic Profiling Analysis

SNP-array-based genomic profiling allowed comprehensive detection of genomic copy number alterations. Genomic DNA of BCP-ALL cell lines was prepared by the Qiagen Gentra Puregene Kit (Qiagen). The procedure of labelling, hybridization and scanning of Cytoscan HD arrays was performed by the Genome Analytics Facility located at the Helmholtz Centre for Infection Research (Braunschweig, Germany), according to the manufacturer’s protocols (Affymetrix, High Wycombe, UK). The associated Chromosome Analysis Suite software version 3.1.0.15 (Affymetrix) was used to generate and illustrate the data.

### 2.5. Protein Analyses

Western blots were performed by the semi-dry method. Cell line protein lysates were prepared using a SIGMAFast protease inhibitor cocktail (Sigma-Aldrich). Extracted proteins were separated in SDS-gels, transferred onto nitrocellulose membranes (Bio-Rad, Munich, Germany) and blocked with 5% dry milk powder dissolved in phosphate-buffered-saline buffer (PBS). We used the following antibodies: alpha-Tubulin (Sigma-Aldrich, #T6199) and NKX6-3 (G-Biosciences; Taufkirchen, Germany #ITA3891). For loading control, blots were reversibly stained with Poinceau (Sigma-Aldrich) and detection of alpha-Tubulin (TUBA) was performed thereafter. Secondary antibodies were linked to peroxidase for detection by Western-Lightning-ECL (Perkin Elmer, Waltham, MA, USA). For documentation we used the digital system ChemoStar Imager (INTAS, Goettingen, Germany).

## 3. Results

### 3.1. Expression and Regulation of NKX6-3 in B-Cell Progenitors

TF-codes describe the normal expression patterns of selected TF groups in cognate hematopoietic entities. These codes are useful to guide analysis of regulatory connections between co-expressed TFs in developing B-cells [12]. Here, we focused on NKL-code member NKX6-3 because of its sharp restriction to BCPs within early B-cell development [10,12]. Furthermore, BCPs, in addition to pro-B-cells and pre-B-cells, are cells of origin for BCP-ALL. Thus, aberrant deregulation of NKX6-3 may contribute to the pathogenesis of BCP-ALL subsets.

**Figure 1:**
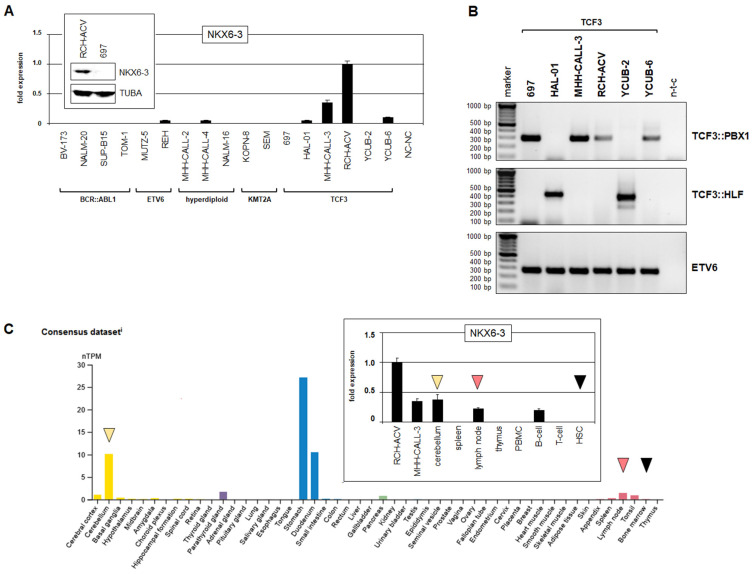
NKX6-3 expression in TCF3::PBX1-positive BCP-ALL. (**A**) RQ-PCR analysis of NKX6-3 in BCP-ALL cell lines, which are ranked according to reported subgroups BCR::ABL1, ETV6, hyperdiploid, KMT2A and TCF3 (above). The highest expression level was detected in RCH-ACV and set as 1. Western blot analysis confirmed NKX6-3 expression in RCH-ACV; TUBA served as loading control (insert). (**B**) RT-PCR analysis of TCF3-group BCP-ALL cell lines for fusion genes TCF3::PBX1 (above), TCF3::HLF (middle) and ETV6 (below), which served as positive control. (**C**) Public RNA-seq data (obtained from the Human Protein Atlas) for NKX6-3 in selected tissues. Arrowheads highlight the tissues cerebellum (yellow), lymph node (red) and bone marrow (black). RQ-PCR analysis of NKX6-3 in cell line RCH-ACV and selected primary tissues and cell types (insert). Arrowheads highlight cerebellum (yellow), lymph node (red) and hematopoietic stem cells (HSCs, black). PBMC: Peripheral blood mononuclear cells. The data were generated in triplicate (**A**,**B**) or in duplicate (**C**).

To identify TFs generating a regulatory network together with NKX6-3, we first assessed the suitability of BCP-ALL cell lines as models for the corresponding entities of early B-cell development. RQ-PCR, Western blot and RT-PCR analyses demonstrated that TCF3::PBX1-positive BCP-ALL cell lines RCH-ACV and MHH-CALL3 expressed prominent levels of NKX6-3 (Figure 1A,B). Public RNA-seq data from healthy donors showed NKX6-3 activity in lymph nodes and tonsils, absence in the bone marrow, and prominent expression levels in the cerebellum and the stomach (Figure 1C). Data from comparative RQ-PCR analysis of NKX6-3 expression in cell lines RCH-ACV and MHH-CALL3, together with primary cells derived from cerebellum and selected hematopoietic cells and tissues, confirmed the RNA-seq data and demonstrated elevated transcript levels in both cell lines (Figure 1C). Of note, these data correspond to the reported NKL-code [10,12], showing NKX6-3 expression in lymph nodes/GC B-cells and mature B-cells/plasma cells, and its absence in hematopoietic stem cells. Therefore, these data endorse BCP-ALL cell lines RCH-ACV and MHH-CALL3 as suitable models for NKX6-3 network analyses.

**Figure 2:**
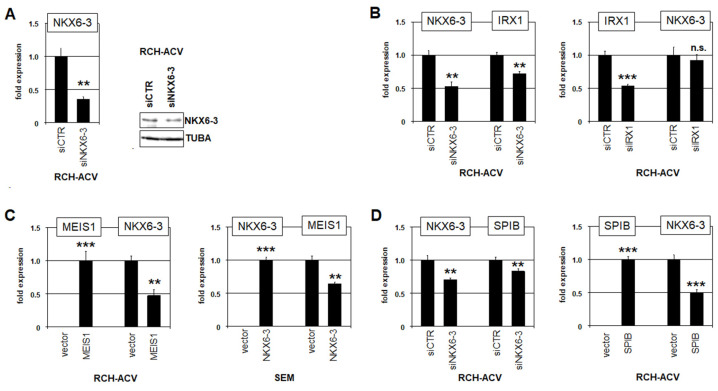
Regulatory connections of NKX6-3 with TALE-class homeobox genes IRX1 and MEIS1 and ETS-TF SPIB. (**A**) SiRNA-mediated knockdown of NKX6-3 in RCH-ACV was confirmed by RQ-PCR (left) and Western blot analysis (right). (**B**) RQ-PCR analysis of RCH-ACV after knockdown of NKX6-3 (left) and IRX1 (right). (**C**) RQ-PCR analysis of RCH-ACV after forced expression of MEIS1 (left) and of SEM after forced expression of NKX6-3 (right). (**D**) RQ-PCR analysis of RCH-ACV after knockdown of NKX6-3 (left) and forced expression of SPIB (right). The data were generated in triplicate. Statistical significance was assessed by t-test (two-tailed), and the calculated *p*-values are indicated by asterisks (** *p* < 0.01, *** *p* < 0.001, n.s. not significant).

We selected TFs for analysis of their potential regulatory connections with NKX6-3 according to established TF-codes, namely TALE-code members IRX1 and MEIS1 and ETS-code member SPIB. These TFs showed conspicuous activities in early B-cell development: IRX1 and MEIS1 expression is restricted to pro-B-cells, and SPIB expression begins in pro-B-cells and remains active in early B-cell development [12]. TF activities were suppressed via siRNA-mediated knockdowns and enhanced by gene expression constructs. The corresponding experiments were performed in NKX6-3-positive cell line RCH-ACV (Figure 2A) and MEIS1-positive cell line SEM, as described recently [12]. Gene activities within the treated cells were subsequently quantified at the transcript level by RQ-PCR. The data demonstrated that TALE-class homeobox gene IRX1 was activated by NKX6-3, while IRX1 showed no impact on NKX6-3 expression (Figure 2B). In contrast, NKX6-3 and MEIS1 were found to be mutually repressive (Figure 2C). Furthermore, ETS-TF SPIB inhibited NKX6-3 expression, while NKX6-3 activated SPIB (Figure 2D). Taken together, these results show that NKX6-3 is tightly connected with TFs active in the following pro-B-cell stage. Therefore, due to its unique expression in BCPs, NKX6-3 may play an important role in differentiation processes, mediating transition between these particular stages of early B-cell development.

### 3.2. NKX6-3 Is a Target Gene of TCF3::PBX1 in BCP-ALL

**Figure 3:**
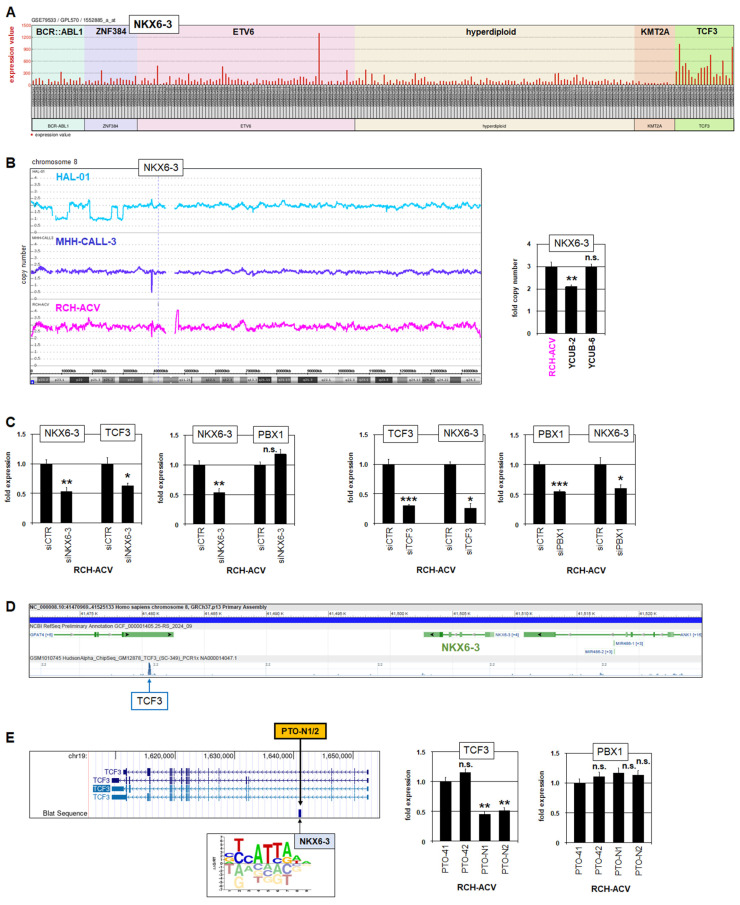
Expression and regulation of NKX6-3 in BCP-ALL. (**A**) Public gene expression profiling data for NKX6-3 from pediatric BCP-ALL patients (dataset GSE79533). The patients are ordered according to the subtypes BCR-ABL1, ZNF384, ETV6, hyperdiploid, KMT2A and TCF3. (**B**) Genomic copy numbers for chromosomes 8 using genomic profiling data from cell lines HAL-01, MHH-CALL3 and RCH-ACV (left). The NKX6-3 locus is indicated. RQ-PCR analysis of NKX6-3 copy numbers in RCH-ACV, YCUB-2 and YCUB-6 (right). (**C**) RQ-PCR analysis of NKX6-3, TCF3 and PBX1 transcripts in RCH-ACV after siRNA-mediated knockdowns. (**D**) Public ChIP-seq data for TCF3 (ENCODE project) showing binding of TCF3 at the locus of NKX6-3 in lymphoblastoid cell line GM12878. (**E**) A search for potential NKX6-3 binding sites at the locus of TCF3 revealed a site in intron 3 as indicated, using genome browser UCSC and data from the CIS-BP database (left). RQ-PCR analysis of TCF3 and PBX1 expression in RCH-ACV after performing enhancer inhibition using PTO-modified oligonucleotides targeting the indicated NKX6-3 binding site. PTO-41 and PTO-42 served as negative controls. The data were generated in triplicate (**B**,**C**) and duplicate (**E**). Statistical significance was assessed by t-test (two-tailed) and the calculated *p*-values are indicated by asterisks (* *p* < 0.05, ** *p* < 0.01, *** *p* < 0.001, n.s. not significant).

Interestingly, analysis of NKX6-3 expression in pediatric BCP-ALL patients using public gene expression profiling dataset GSE79533 showed prominent activity in most patients of the TCF3-subtype, while silent in the remaining subtypes (Figure 3A). This conspicuous expression pattern was confirmed in an additional dataset with pediatric BCP-ALL patients (Figure S3), corresponded to the cell line data (Figure 1A), and indicates both aberrant activation of NKX6-3 and its regulatory connection with fusion gene TCF3::PBX1.

Analysis of NKX6-3 copy numbers using genomic profiling data indicated a copy number gain in RCH-ACV, while cell lines MHH-CALL3 and HAL-01 showed wild type configurations (Figure 3B). NKX6-3 copy number gain in RCH-ACV was confirmed by RQ-PCR analysis, which also revealed a gain in the NKX6-3 weakly expressing cell line YCUB-6 (Figure 3B). Thus, copy number alterations may support NKX6-3 expression but did not represent a major mechanism of its deregulation.

SiRNA-mediated knockdown experiments targeting NKX6-3, TCF3 and PBX1 in TCF3::PBX1-positive RCH-ACV demonstrated that NKX6-3 activated TCF3 but not PBX1, while both TCF3 and PBX1 activated NKX6-3 (Figure 3C). These results were supported by public ChIP-seq data obtained from the ENCODE project [38], showing binding of TCF3 at the NKX6-3 locus (Figure 3D). Furthermore, we performed an enhancer-inhibition assay as we described for analysis of the transcriptional regulation of NKL homeobox gene NKX2-5 in T-cell leukemia [34]. Accordingly, electroporation of PTO-modified oligonucleotides into RCH-ACV cells, which target a potential binding site for NKX6-3 identified at the TCF3 locus, resulted in reduced TCF3 expression while sparing PBX1 (Figure 3E). Thus, NKX6-3 and TCF3 are mutual activators, probably reflecting the physiological context. Furthermore, fusion protein TCF3::PBX1 aberrantly activates NKX6-3, underlying their observed co-expression in TCF3-subtype BCP-ALL patients.

### 3.3. NKX6-3 Is Linked to TGFb-Signalling and the HIPPO-Pathway

**Figure 4:**
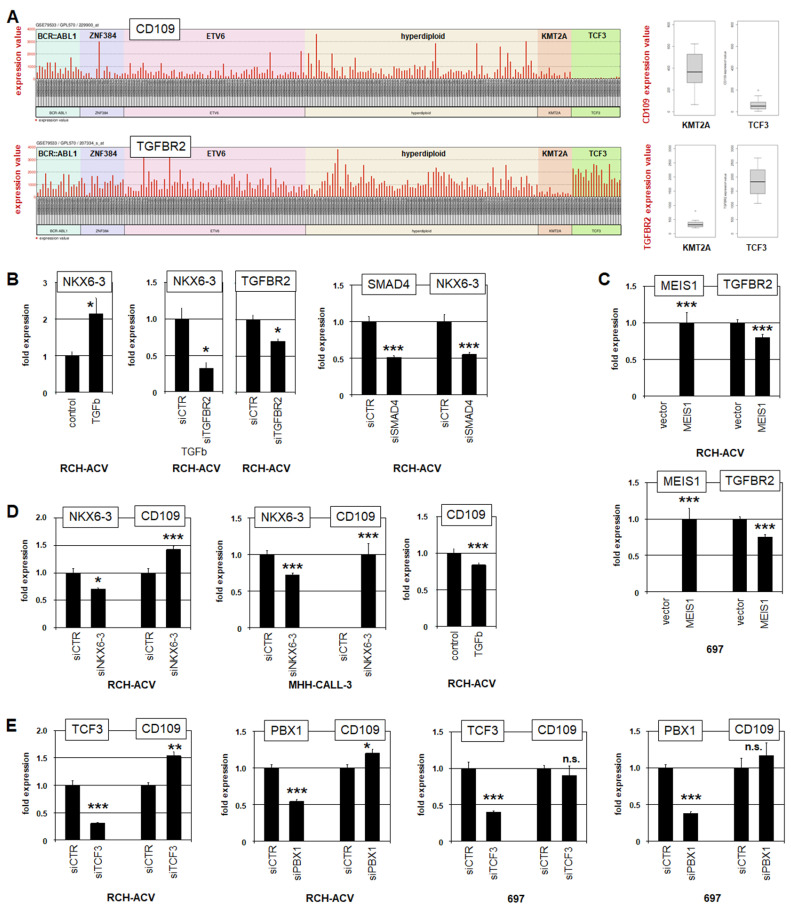
NKX6-3 inhibits CD109 and is activated by TGFb-signalling. (**A**) Expression of CD109 (above) and TGFBR2 (below) in BCP-ALL patients using public gene expression profiling dataset GSE79533. The patient samples are ordered according to the subtypes BCR-ABL1, ZNF384, ETV6, hyperdiploid, KMT2A and TCF3. Box plots (right) show statistical comparison of expression data for KMT2A and TCF3 subgroup patients. The *p*-values are 0.00015 for CD109 and 0.00001 for TGFBR2, showing highly significant differences. (**B**) RQ-PCR analysis of NKX6-3, TGFBR2 and SMAD4 in RCH-ACV after stimulation with TGFB (left) and siRNA-mediated knockdown of TGFBR2 (middle) and SMAD4 (right). (**C**) RQ-PCR analysis of MEIS1 and TGFBR2 in RCH-ACV (above) and 697 (below) after forced expression of MEIS1. (**D**) RQ-PCR analysis of NKX6-3 and CD109 in RCH-ACV (left) and MHH-CALL3 (middle) after siRNA-mediated knockdown of NKX6-3. RQ-PCR analysis of CD109 in RCH-ACV stimulated with TGFb (right). (**E**) RQ-PCR analysis of TCF3, PBX1,and CD109 in RCH-ACV (left) and 697 (right) after siRNA-mediated knockdown of TCF3 and PBX1. The data were generated in duplicate (**B**,**C**) and triplicate (**D**,**E**). Statistical significance was assessed by t-test (two-tailed) and the calculated *p*-values are indicated by asterisks (* *p* < 0.05, ** *p* < 0.01, *** *p* < 0.001, n.s. not significant).

To identify potential target genes of NKX6-3, we analyzed public BCP-ALL patient dataset GSE79533, using the associated online tool GEOR. We compared 11 patient samples from the TCF3-group expressing high NKX6-3 levels with 207 controls comprising the remaining samples and subtypes of this dataset. This inspection revealed the 250 most significantly differentially expressed genes for the analyzed groups (Table S1), including NKX6-3, PBX1, WNT16, CD99, CD109, GP5 and MPP7 (Figure S4). Corresponding analysis of dataset GSE13576 revealed significance for the same genes, confirming these results (Table S2, Figure S3). Of note, PBX1 corresponds to the presence of fusion gene TCF3::PBX1 in the TCF3-group, while WNT16 and CD99 have been recently reported to be associated with TCF3::PBX1 in BCP-ALL [39,40], endorsing this approach. Here, we focused on CD109, which encodes an inhibitor of TGFb-signalling [41] and showed downregulation in NKX6-3-positive TCF3-subtype patients (Figure 4A). TGFb-signalling via TGFBR2 and SMAD3 is reportedly inhibited in ETV6-subtype BCP-ALL patients, demonstrating the significance of this pathway in this malignancy [12,42]. In this setting, treatment of RCH-ACV cells with TGFb enhanced NKX6-3 expression, indicating an activating input for TGFb-signalling (Figure 4B). Furthermore, TGFb treatment combined with knockdown of TGFBR2, or knockdown of signalling cofactor SMAD4 without additional treatment inhibited NKX6-3 expression, indicating that TGFb-signalling is mediated by receptor TGFBR2 (Figure 4B). Thus, reduced CD109 may support NKX6-3 expression via enhanced TGFb-pathway activity.

Analysis of TGFBR2 in BCP-ALL patients showed reduced expression levels in the KMT2A-subtype group (Figure 4A). Consistent with this observation, forced expression of KMT2A target gene MEIS1 in RCH-ACV and 697 cells reduced TGFBR2 expression (Figure 4C). These results may well indicate that TGFb-signalling is active in BCPs while attenuated in pro-B-cells via MEIS1. To analyze the regulation of TGFb-inhibitor CD109, we performed siRNA-mediated knockdown of NKX6-3 in RCH-ACV and MHH-CALL3. These experiments showed that NKX6-3 inhibited CD109 expression (Figure 4D). Consistent with this finding, treatment of RCH-ACV cells with TGFb resulted in reduced expression of CD109 (Figure 4D). The potential impact of fusion protein TCF3::PBX1 was analyzed by knockdown of TCF3 and PBX1 in NKX6-3-positive RCH-ACV and NKX6-3-negative 697 cells. The results demonstrated significant inhibition of CD109 by TCF3 and PBX1 in RCH-ACV but not in 697 (Figure 4E). These data may suggest that the inhibitory input of TCF3::PBX1 was mediated via NKX6-3, which is absent in 697 cells. Thus, NKX6-3 supports its own expression by inhibition of TGFb-pathway inhibitor CD109. Moreover, analysis of potential NKX6-3 binding sites at the CD109 locus revealed a highly conserved NKX6-3-site within a 20 kb upstream regulatory region (Figure S5), suggesting direct regulation.

**Figure 5:**
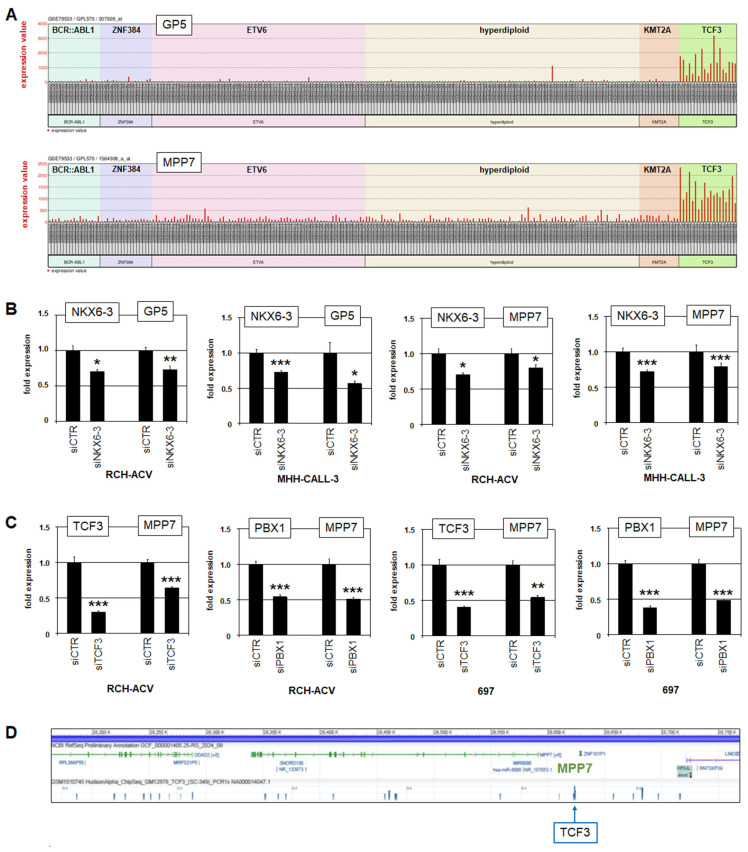
NKX6-3 and TCF3::PBX1 activate HIPPO-pathway component MPP7. (**A**) Expression of GP5 (above) and MPP7 (below) in BCP-ALL patients using public gene expression profiling dataset GSE79533. The patient samples are ordered according to the subtypes BCR-ABL1, ZNF384, ETV6, hyperdiploid, KMT2A and TCF3. (**B**) RQ-PCR analysis of NKX6-3, GP5 and MPP7 in RCH-ACV and MHH-CALL3 after siRNA-mediated knockdown of NKX6-3. (**C**) RQ-PCR analysis of TCF3, PBX1 and MPP7 in RCH-ACV (left) and 697 (right) after siRNA-mediated knockdown of TCF3 and PBX1. (**D**) Public ChIP-seq data for TCF3 (ENCODE project) showing binding of TCF3 at the locus of MPP7 in lymphoblastoid cell line GM12878. The data were generated in triplicate. Statistical significance was assessed by t-test (two-tailed) and the calculated *p*-values are indicated by asterisks (* *p* < 0.05, ** *p* < 0.01, *** *p* < 0.001).

To identify additional NKX6-3 targets, we performed RNA-seq analysis of RCH-ACV cells treated for siRNA-mediated knockdown of NKX6-3 (Table S3). Subsequent gene set annotation analysis of the top 500 differentially activated and downregulated genes indicated potential impacts of NKX6-3 on development and differentiation (including brain, B-cells and stem cells), immune response, and several pathways (including Calcium-, cAMP-, AKT- and JAK-STAT-signalling) (Figure S6). Thus, these data support a role for NKX6-3 in developmental processes and indicate regulation of signalling pathways. Interestingly, this approach revealed NKX6-3-activated genes GP5 and MPP7 among the top 20, which were also identified via comparative gene expression profiling analysis of BCP-ALL patients (Tables S1 and S2, Figures S3 and S4). Consistent with this picture, GP5 and MPP7 showed strong positive correlation with NKX6-3-expressing TCF3-subtype BCP-ALL patients (Figure 5A).

RQ-PCR analysis of GP5 and MPP7 after NKX6-3 knockdown in RCH-ACV and MHH-CALL-3 confirmed that NKX6-3 activated their expression (Figure 5B). GP5 encodes a glycoprotein which is associated with the VWF-receptor, and MPP7 a component of the HIPPO-pathway [43,44]. Recently, this pathway has been shown to play a role in BCP-ALL and may, therefore, be of heightened interest [40,45,46]. Therefore, as performed for CD109 (see above), we analyzed RCH-ACV and 697 cells after treatment for knockdown of TCF3 and PBX1. This approach showed that TCF3 and PBX1 activated MPP7 in both cell lines (Figure 5C). Thus, MPP7 is an activated target gene of both TCF3::PBX1 and NKX6-3. In conclusion, these data may indicate that HIPPO-pathway activation is physiologically conducted via NKX6-3 in BCPs and aberrantly via TCF3::PBX1 in BCP-ALL. Analysis of potential NKX6-3 binding sites at MPP7 locus revealed a highly conserved NKX6-3-site within a 20 kb upstream regulatory region (Figure S5), suggesting direct regulation. In addition, public ChIP-seq data obtained from the ENCODE project show binding of TCF3 at the locus of MPP7 [38] (Figure 3D), supporting direct regulation via fusion protein TCF3::PBX1.

**Figure 6:**
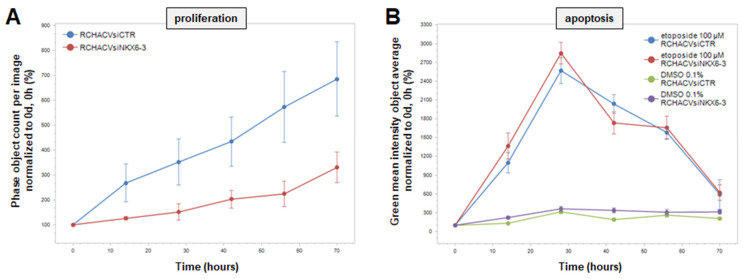
Functional analysis of NKX6-3 in RCH-ACV cells was performed after siRNA-mediated knockdown via live-cell imaging. (**A**) Proliferation of RCH-ACV cells significantly decreased when NKX6-3 was reduced. (**B**) Apoptosis was induced by adding etoposide, controls received the solvent DMSO. This treatment showed no difference to the control. The data were generated in triplicate; standard deviations are indicated by error bars.

Aberrantly activated HIPPO-pathway has been reported in cancer cells, supporting proliferation and survival [46]. To investigate these functional aspects in our model, we analyzed cell line RCH-ACV after treatment for siRNA-mediated knockdown of NKX6-3 via live-cell imaging. The data indicated that NKX6-3 mediated increased proliferation (Figure 6A). To analyze a potential role in cell survival we added apoptosis-inducer etoposide. However, this treatment elicited indistinguishable responses in knockdown and control cells (Figure 6B), indicating that NKX6-3 has no impact on the survival of early B-cells.

## 4. Discussion

**Figure 7:**
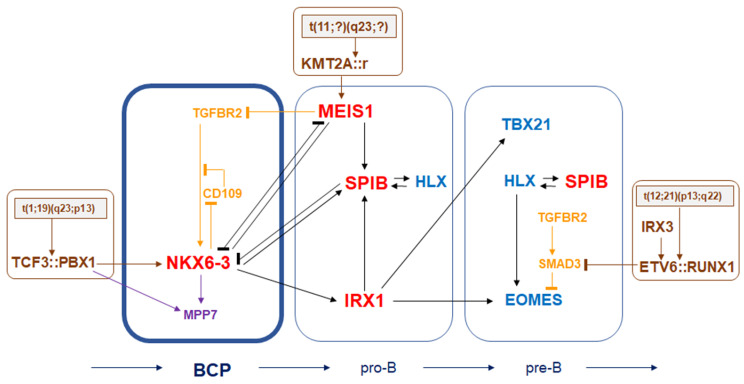
NKX6-3 is part of a regulatory network in B-cell progenitors (BCPs) and a target of the TCF3::PBX1-fusion in BCP-ALL. This diagram summarizes gene the regulatory data obtained from this study for the stage of BCPs and from our previous study for the stages of pro-B- and pre-B-cells [12]. Analyzed TFs (red) and target genes (TGFb-pathway members in orange, HIPPO-pathway member in purple), in addition to the selected fusion genes, TCF3::PBX1, KMT2A::r and ETV6::RUNX1 (brown) are indicated.

In this study we have shown that NKX6-3 is physiologically expressed in B-cell progenitors and that it plays a significant role in the mutual regulation of specific developmental TFs active in early B-cell differentiation. Furthermore, NKX6-3 is expressed in step with the formation of fusion gene TCF3::PBX1 in BCP-ALL patients and cell lines of the corresponding subtype, which acts as an activator. Downstream analyses of NKX6-3 revealed novel target genes, underlining the impact of TGFb- and HIPPO-pathways in normal and abnormal early B-cell development. The derived regulatory network of NKX6-3 is depicted in Figure 7 and discussed below.

NKX6-3 is physiologically expressed in BCPs, GC-B-cells and plasma cells, thus showing discrete activities in early and late B-cell development [10,12]. Moreover, NKX6-3 is normally expressed in developing neural crest cells, the hindbrain/cerebellum and the stomach/duodenum, demonstrating a restricted activity overall [47,48,49,50]. Aberrant downregulation of NKX6-3 causes gastric cancer, indicating tumour suppressor activity in this tissue type [49,50]. In contrast, NKX6-3 is highly expressed in TCF3-subtype BCP-ALL and GC-B-cell-derived diffuse large B-cell lymphoma subsets, contrastingly indicating oncogene activity in developing B-cells [10]. NKX6-3 expression in these two types of B-cell malignancies may result from defects in its downregulation, thus reflecting its aberrant maintenance in their cells of origin. This interpretation resembles the situation reported for aberrant activities of NKL-subclass homeobox genes in T-cell leukemia. In this lymphoid malignancy, NKL homeobox genes HHEX, HLX, NKX3-1, TLX2 and VENTX have to be downregulated after the DN-stage of T-cell development to terminate the differentiation process. Their aberrant maintenance or ectopic activation of other NKL-subclass members like NKX2-5, TLX1 or TLX3 support T-cell leukemogenesis [9,34].

NKX6-3 downregulation may be required to proceed from BCPs to the pro-B-cell stage and to complete early B-cell development. Furthermore, our data showed that MEIS1 and SPIB inhibit NKX6-3 expression while NKX6-3 activates IRX1 and SPIB. Of note, analysis of potential NKX6-3 binding sites at the loci of IRX1 and SPIB revealed highly conserved NKX6-3-sites (Figure S5), suggesting direct regulation. TALE-class homeobox genes MEIS1 and IRX1 are physiologically restricted to pro-B-cells, while ETS-gene SPIB is upregulated in this entity and remains active in the following stages of B-cell development [8,11]. To licence B-cell differentiation at these stages, NKX6-3 must be silenced, and MEIS1, IRX1 and SPIB upregulated. These genes may thus constitute a fundamental regulatory network at the transition from BCP to pro-B-cells. Accordingly, their deregulation is involved in the pathogenesis of BCP-ALL at a fundamental level. Hence, fusion protein TCF3::PBX1 activates NKX6-3; KMT2A fusions activate MEIS1, which in turn inhibits NKX6-3; IRX1 remains aberrantly active; IRX1 relatives IRX2 and IRX3 are ectopically expressed and SPIB is aberrantly downregulated [8,21,22,51]. Similarly, the reported regulatory network consisting of the TFs PAX5, EBF1 and TCF3 plays a fundamental role in B-cell development, while their genes are frequently mutated or deregulated in BCP-ALL [4,14,15,16,17]. Thus, specific TFs are involved in the regulation of developmental processes and prone to drive B-cell leukemogenesis when their activity is perturbed.

In addition to these TFs, we identified regulatory connections between NKX6-3 and the TGFb- and HIPPO-pathways. NKX6-3 downregulated expression of TGFb-pathway inhibitor CD109 while TGFb activated NKX6-3 expression via TGFBR2. Thus, TGFb-signalling may support BCPs via NKX6-3. In contrast, MEIS1 inhibits TGFBR2 expression probably in both normal pro-B-cells and tumour cells of the corresponding BCP-ALL subtype. This regulatory link may inhibit NKX6-3 expression indirectly and has also been reported in lung cancer cells [52]. Furthermore, TGFb-signalling inhibits EOMES in pre-B-cells via TGFBR2 and SMAD3. Aberrant inhibition of SMAD3 by fusion protein ETV6::RUNX1 or downregulation of TGFBR2 via genomic deletion activates the expression of EOMES, supporting a developmental arrest at this stage [12,42]. In conclusion, TGFBR2-mediated TGFb-signalling may, in turn, physiologically support B-cell differentiation at the stage of BCP via NKX6-3, be downregulated in pro-B-cells by MEIS1, and drive developmental progression at the stage of pre-B-cells via repression of EOMES.

MPP7 enhances the cooperation of HIPPO-pathway factor YAP/TAZ with YY1 in muscle stem cells [44]. Therefore, the identified NKX6-3 target gene MPP7 indicates an important role for the HIPPO-pathway in early B-cell development. This pathway activity is also required in plasma cells [53], suggesting activation by NKX6-3 in these mature B-cells as well. An oncogenic role for the HIPPO-pathway in BCP-ALL has been reported recently [45,54], further supporting our findings and interpretation. However, our functional assay revealed that NKX6-3 enhances proliferation but leaves open whether this is mediated by the activated HIPPO-pathway. Nevertheless, our gene set annotation analysis revealed additional signalling pathways potentially regulated by NKX6-3, indicating a much broader role of this gene in the orchestration of early B-cell development. In contrast, the identified NKX6-3 target gene GP5 encodes Glycoprotein 5, which is part of the receptor complex for VWF and mediates platelet adhesion and hemostasis [43]. Whether GP5 plays a physiological and/or pathological role in early B-cell development remains to be shown. Finally, public RNA-seq data from 93 leukemia cell lines for the identified NKX6-3 target genes show reduced activity of CD109 and overexpression of GP5 and MPP7 in RCH-ACV and MHH-CALL-3 (Figure S7), highlighting the suitability of these cell lines as models for their investigation in follow-up studies.

## 5. Conclusions

Our study underlines the role of NKL homeobox gene NKX6-3 in early B-cell development and TCF3::PBX1-positive BCP-ALL. The data may contribute to the understanding of early B-cell differentiation, implicating regulatory networks and introducing novel players which may serve as diagnostic markers and/or therapeutic targets in congruent BCP-ALL subtypes.

## Data Availability

The original data presented in the study are openly available via the indicated resources.

## Supplementary Materials

The following supporting information can be downloaded at https://www.mdpi.com/article/10.3390/genes16101199/s1, Figure S1: RNAseq QC-metric for RCH-ACVsiCTR; Figure S2: RNAseq QC-metric for RCH-ACVsiNKX6-3; Figure S3: Gene expression data of public dataset GSE13576; Figure S4: Gene expression data of public dataset GSE79533; Figure S5: Potential binding sites for NKX6-3; Figure S6: Gene set annotation analysis; Figure S7: Human Protein Atlas RNA-seq data for leukemia cell lines; Table S1: GEOR analysis of dataset GSE79533; Table S2: GEOR analysis of dataset GSE13576; Table S3: RNAseq data of RCH-ACV treated for NKX6-3 knockdown.
