# Supplementary material for: NKX6-3 in B-Cell Progenitor Differentiation and Leukemia
Source: Genes (Basel). 2025 Oct 14;16(10):1199. doi: 10.3390/genes16101199 (PMC12563489; doi:10.3390/genes16101199)
Supplement: Supplementary file 1 [file genes-16-01199-s001.zip › Supplemental Figures 3-7.pdf]

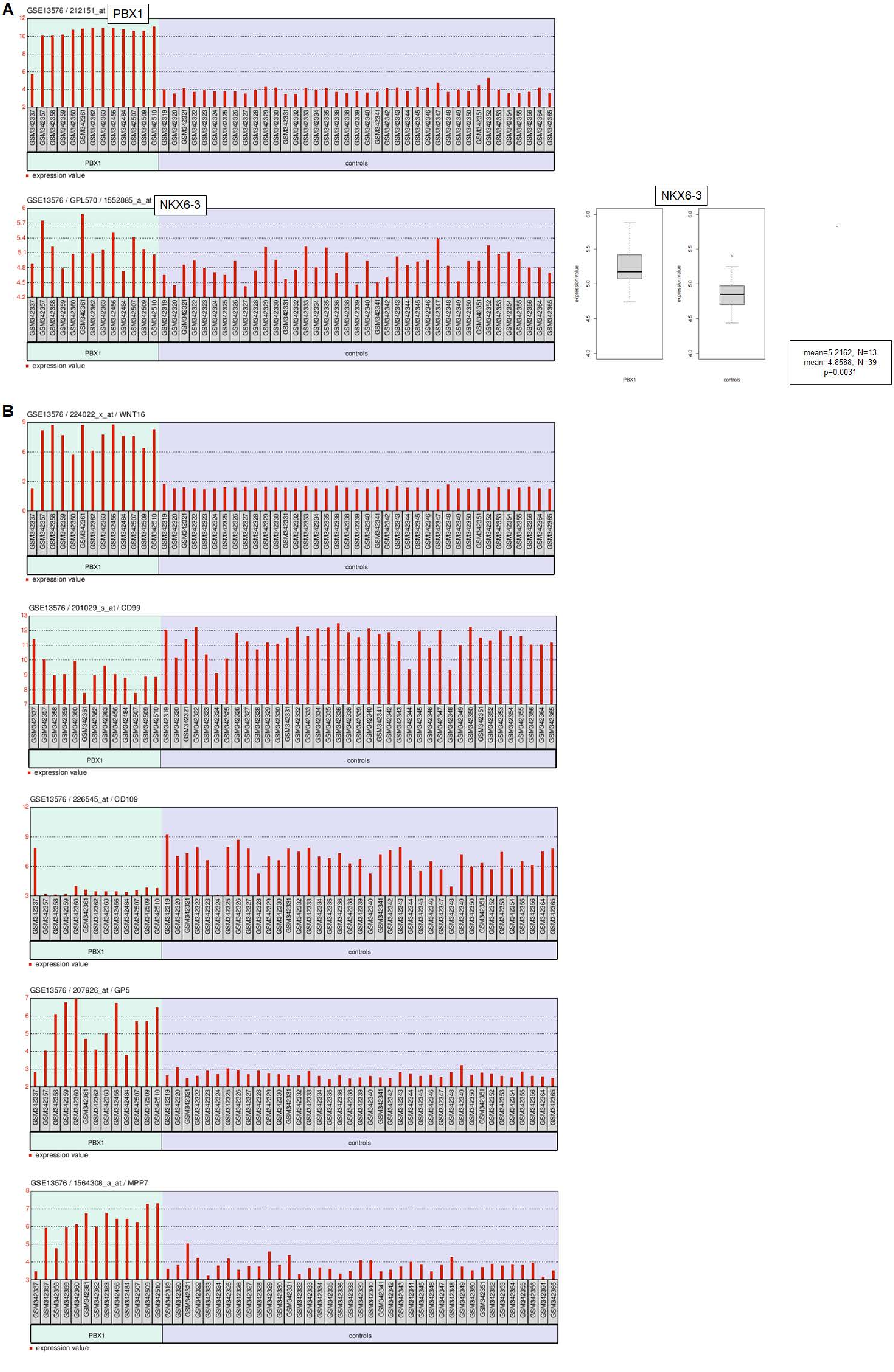

Figure S3: Gene expression profiling data for selected genes, obtained from dataset GSE13576 and selected pediatric BCP-ALL patients, expressing enhanced PBX1 (N=13, corresponding to fusion gene TCF3::PBX1) and 39 controls. (A) Expression data for PBX1 (above) and NKX6-3 (below). For NKX6-3 these data are illustrated as a boxplot and analyzed statistically (right). (B) GEOR analysis of these two patient groups revealed WNT16, CD99, CD109, GP5 and MPP7.

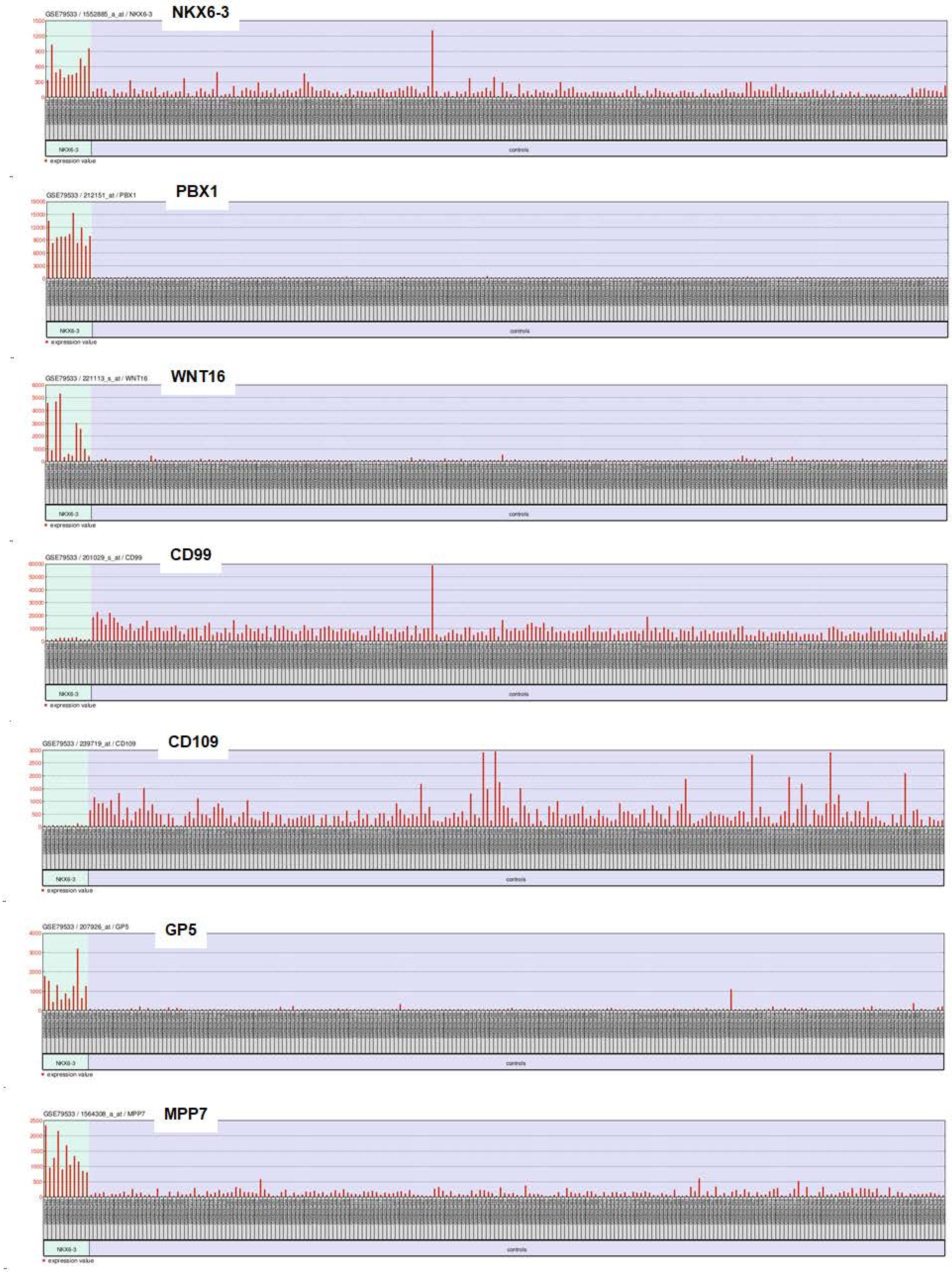

Figure S4: Gene expression profiling data for selected genes, identified by comparison of pediatric BCP-ALL patient groups (eleven NKX6-3 high versus 207 controls), using dataset GSE79533 and online tool GEOR.

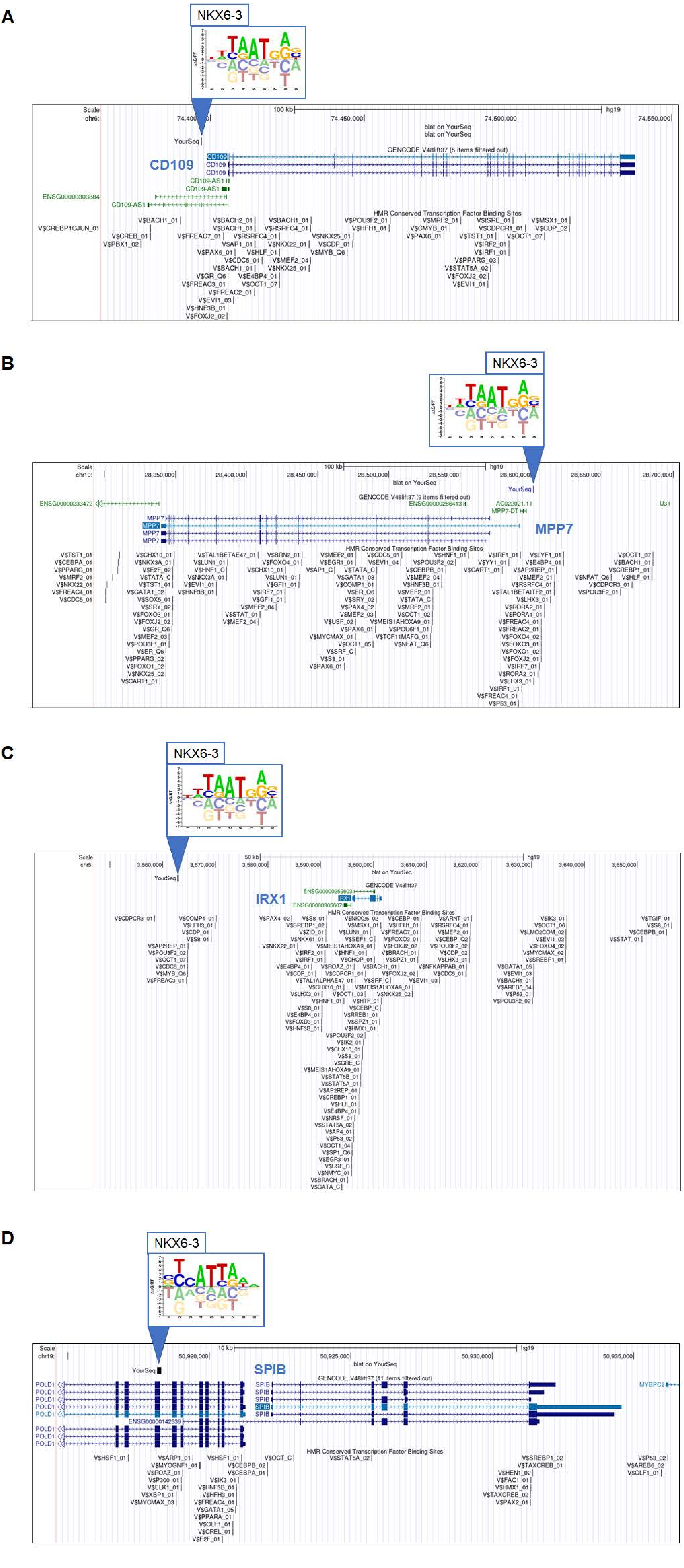

Figure S5: Potential binding sites for NKX6-3 using the CIS-BP Database and the UCSC genome browser for the loci of (A) CD109, (B) MPP7, (C) IRX1, and (D) SPIB.

NKX6-3 activated targets (top-500)

Sublist	Category	Term	RT	Genes	Count	%	P-Value	Benjamini
	KEGG_PATHWAY	Amphetamine addiction	RT		9	2,0	7,4E-5	2,0E-2
	KEGG_PATHWAY	Glutamatergic synapse	RT		10	2,2	5,8E-4	7,9E-2
	KEGG_PATHWAY	Insulin secretion	RT		7	1,6	8,1E-3	4,4E-1
	KEGG_PATHWAY	Serotonergic synapse	RT		8	1,8	9,0E-3	4,4E-1
	KEGG_PATHWAY	GABAergic synapse	RT		7	1,6	9,5E-3	4,4E-1
	KEGG_PATHWAY	Proximal tubule bicarbonate reclamation	RT		4	0,9	1,1E-2	4,4E-1
	KEGG_PATHWAY	Oxytocin signaling pathway	RT		9	2,0	1,4E-2	4,4E-1
	KEGG_PATHWAY	Circadian entrainment	RT		7	1,6	1,4E-2	4,4E-1
■	KEGG_PATHWAY	Calcium signaling pathway	RT		12	2,7	1,5E-2	4,4E-1
	KEGG_PATHWAY	Cocaine addiction	RT		5	1,1	1,7E-2	4,7E-1
	KEGG_PATHWAY	Vascular smooth muscle contraction	RT		8	1,8	2,0E-2	4,8E-1
	KEGG_PATHWAY	Retinol metabolism	RT		5	1,1	5,0E-2	1,0E0
	KEGG_PATHWAY	Nicotine addiction	RT		4	0,9	5,0E-2	1,0E0
	KEGG_PATHWAY	Dopaminergic synapse	RT		7	1,6	5,3E-2	1,0E0
	KEGG_PATHWAY	Type II diabetes mellitus	RT		4	0,9	7,0E-2	1,0E0
	KEGG_PATHWAY	Metabolic pathways	RT		41	9,2	7,2E-2	1,0E0
	KEGG_PATHWAY	Alcoholism	RT		8	1,8	9,0E-2	1,0E0
■	KEGG_PATHWAY	cAMP signaling pathway	RT		9	2,0	9,1E-2	1,0E0

Sublist	Category	Term	RT	Genes	Count	%	P-Value	Benjamini
	GOTERM_BP_DIRECT	cellular response to cAMP	RT		7	1,6	8,7E-4	1,0E0
	GOTERM_BP_DIRECT	digestive tract morphogenesis	RT		4	0,9	2,9E-3	1,0E0
	GOTERM_BP_DIRECT	extracellular matrix organization	RT		10	2,2	5,2E-3	1,0E0
	GOTERM_BP_DIRECT	calcium ion transmembrane transport	RT		9	2,0	5,3E-3	1,0E0
	GOTERM_BP_DIRECT	male gonad development	RT		8	1,8	5,8E-3	1,0E0
	GOTERM_BP_DIRECT	chemical synaptic transmission	RT		12	2,7	6,0E-3	1,0E0
	GOTERM_BP_DIRECT	regulation of synaptic plasticity	RT		6	1,3	6,5E-3	1,0E0
	GOTERM_BP_DIRECT	transmembrane transport	RT		12	2,7	9,7E-3	1,0E0
	GOTERM_BP_DIRECT	regulation of neurotransmitter secretion	RT		4	0,9	1,0E-2	1,0E0
	GOTERM_BP_DIRECT	catecholamine metabolic process	RT		3	0,7	1,3E-2	1,0E0
	GOTERM_BP_DIRECT	axon guidance	RT		10	2,2	1,3E-2	1,0E0
■	GOTERM_BP_DIRECT	developmental growth	RT		4	0,9	1,4E-2	1,0E0
■	GOTERM_BP_DIRECT	cell differentiation	RT		24	5,4	1,5E-2	1,0E0
	GOTERM_BP_DIRECT	sodium ion transport	RT		6	1,3	1,7E-2	1,0E0
	GOTERM_BP_DIRECT	monoatomic ion transport	RT		8	1,8	1,9E-2	1,0E0
■	GOTERM_BP_DIRECT	brain development	RT		10	2,2	2,2E-2	1,0E0
	GOTERM_BP_DIRECT	spermatogenesis	RT		18	4,0	2,7E-2	1,0E0
	GOTERM_BP_DIRECT	chondrocyte differentiation	RT		5	1,1	2,7E-2	1,0E0
	GOTERM_BP_DIRECT	cell adhesion	RT		19	4,2	2,8E-2	1,0E0
	GOTERM_BP_DIRECT	endodermal cell differentiation	RT		4	0,9	2,9E-2	1,0E0
	GOTERM_BP_DIRECT	startle response	RT		3	0,7	3,4E-2	1,0E0
	GOTERM_BP_DIRECT	sulfur compound metabolic process	RT		3	0,7	3,9E-2	1,0E0
	GOTERM_BP_DIRECT	parathyroid hormone secretion	RT		2	0,4	3,9E-2	1,0E0
	GOTERM_BP_DIRECT	dopamine metabolic process	RT		3	0,7	4,3E-2	1,0E0
	GOTERM_BP_DIRECT	limb morphogenesis	RT		3	0,7	4,3E-2	1,0E0
	GOTERM_BP_DIRECT	circadian regulation of gene expression	RT		5	1,1	4,5E-2	1,0E0
	GOTERM_BP_DIRECT	sensory perception of sound	RT		8	1,8	4,5E-2	1,0E0
	GOTERM_BP_DIRECT	endoplasmic reticulum organization	RT		4	0,9	4,6E-2	1,0E0
	GOTERM_BP_DIRECT	uterus development	RT		3	0,7	5,3E-2	1,0E0
	GOTERM_BP_DIRECT	protein sulfation	RT		2	0,4	5,8E-2	1,0E0
	GOTERM_BP_DIRECT	synaptic transmission, glutamatergic	RT		4	0,9	5,8E-2	1,0E0
	GOTERM_BP_DIRECT	long-term synaptic potentiation	RT		5	1,1	6,0E-2	1,0E0
	GOTERM_BP_DIRECT	water transport	RT		3	0,7	6,3E-2	1,0E0
	GOTERM_BP_DIRECT	negative regulation of fatty acid biosynthetic process	RT		3	0,7	6,3E-2	1,0E0
■	GOTERM_BP_DIRECT	central nervous system development	RT		7	1,6	6,4E-2	1,0E0
	GOTERM_BP_DIRECT	monoatomic ion transmembrane transport	RT		7	1,6	6,4E-2	1,0E0
	GOTERM_BP_DIRECT	lens development in camera-type eye	RT		4	0,9	6,5E-2	1,0E0
	GOTERM_BP_DIRECT	visual perception	RT		9	2,0	6,6E-2	1,0E0
	GOTERM_BP_DIRECT	response to xenobiotic stimulus	RT		10	2,2	6,6E-2	1,0E0
■	GOTERM_BP_DIRECT	immune response	RT		17	3,8	6,6E-2	1,0E0
	GOTERM_BP_DIRECT	neuron fate specification	RT		3	0,7	6,9E-2	1,0E0
	GOTERM_BP_DIRECT	neutral amino acid transport	RT		3	0,7	6,9E-2	1,0E0
	GOTERM_BP_DIRECT	morphogenesis of an epithelium	RT		3	0,7	6,9E-2	1,0E0
	GOTERM_BP_DIRECT	chloride transmembrane transport	RT		6	1,3	7,2E-2	1,0E0
	GOTERM_BP_DIRECT	epidermis development	RT		5	1,1	7,5E-2	1,0E0
	GOTERM_BP_DIRECT	glutamate biosynthetic process	RT		2	0,4	7,6E-2	1,0E0
■	GOTERM_BP_DIRECT	mature B cell differentiation involved in immune response	RT		2	0,4	7,6E-2	1,0E0
	GOTERM_BP_DIRECT	negative regulation of chondrocyte differentiation	RT		3	0,7	8,0E-2	1,0E0
■	GOTERM_BP_DIRECT	stem cell proliferation	RT		4	0,9	8,2E-2	1,0E0
	GOTERM_BP_DIRECT	cell surface receptor signaling pathway	RT		12	2,7	8,3E-2	1,0E0
	GOTERM_BP_DIRECT	neurogenesis	RT		5	1,1	8,7E-2	1,0E0
	GOTERM_BP_DIRECT	transport across blood-brain barrier	RT		5	1,1	8,7E-2	1,0E0
	GOTERM_BP_DIRECT	negative regulation of Wnt signaling pathway	RT		4	0,9	9,0E-2	1,0E0
	GOTERM_BP_DIRECT	sperm capacitation	RT		3	0,7	9,2E-2	1,0E0
	GOTERM_BP_DIRECT	ionotropic glutamate receptor signaling pathway	RT		3	0,7	9,2E-2	1,0E0
	GOTERM_BP_DIRECT	cell-cell adhesion	RT		8	1,8	9,3E-2	1,0E0
	GOTERM_BP_DIRECT	xenobiotic metabolic process	RT		6	1,3	9,3E-2	1,0E0
	GOTERM_BP_DIRECT	positive regulation of protein kinase A signaling	RT		2	0,4	9,5E-2	1,0E0
	GOTERM_BP_DIRECT	protein localization to adherens junction	RT		2	0,4	9,5E-2	1,0E0
	GOTERM_BP_DIRECT	CMP catabolic process	RT		2	0,4	9,5E-2	1,0E0
	GOTERM_BP_DIRECT	inner ear receptor cell differentiation	RT		2	0,4	9,5E-2	1,0E0
	GOTERM_BP_DIRECT	UMP catabolic process	RT		2	0,4	9,5E-2	1,0E0
■	GOTERM_BP_DIRECT	immune system development	RT		2	0,4	9,5E-2	1,0E0
	GOTERM_BP_DIRECT	skeletal muscle contraction	RT		3	0,7	9,8E-2	1,0E0
	GOTERM_BP_DIRECT	bile acid biosynthetic process	RT		3	0,7	9,8E-2	1,0E0
	GOTERM_BP_DIRECT	plasma membrane repair	RT		3	0,7	9,8E-2	1,0E0
	GOTERM_BP_DIRECT	non-canonical Wnt signaling pathway	RT		3	0,7	9,8E-2	1,0E0

NKX6-3 inhibited targets (top-500)

Sublist	Category	Term	RT	Genes	Count	%	P-Value	Benjamini
	KEGG_PATHWAY	Virion - Hepatitis viruses	RT		7	1,5	7,1E-4	1,9E-1
	KEGG_PATHWAY	Neuroactive ligand-receptor interaction	RT		19	4,2	1,9E-3	2,5E-1
	KEGG_PATHWAY	Cytokine-cytokine receptor interaction	RT		16	3,5	3,2E-3	2,8E-1
	KEGG_PATHWAY	Neutrophil extracellular trap formation	RT		11	2,4	1,3E-2	7,3E-1
	KEGG_PATHWAY	Cholinergic synapse	RT		8	1,8	1,6E-2	7,3E-1
	KEGG_PATHWAY	Breast cancer	RT		9	2,0	1,9E-2	7,3E-1
	KEGG_PATHWAY	Hormone signaling	RT		11	2,4	2,8E-2	7,3E-1
	KEGG_PATHWAY	Amoebiasis	RT		7	1,5	3,0E-2	7,3E-1
	KEGG_PATHWAY	Linoleic acid metabolism	RT		4	0,9	3,0E-2	7,3E-1
	KEGG_PATHWAY	Kaposi sarcoma-associated herpesvirus infection	RT		10	2,2	3,5E-2	7,3E-1
	KEGG_PATHWAY	Motor proteins	RT		10	2,2	3,6E-2	7,3E-1
	KEGG_PATHWAY	Tight junction	RT		9	2,0	4,0E-2	7,3E-1
■	KEGG_PATHWAY	Signaling pathways regulating pluripotency of stem cells	RT		8	1,8	4,6E-2	7,3E-1
	KEGG_PATHWAY	Arrhythmogenic right ventricular cardiomyopathy	RT		6	1,3	4,6E-2	7,3E-1
	KEGG_PATHWAY	Leukocyte transendothelial migration	RT		7	1,5	4,9E-2	7,3E-1
	KEGG_PATHWAY	Complement and coagulation cascades	RT		6	1,3	5,0E-2	7,3E-1
	KEGG_PATHWAY	GABAergic synapse	RT		6	1,3	5,2E-2	7,3E-1
	KEGG_PATHWAY	Phospholipase D signaling pathway	RT		8	1,8	5,3E-2	7,3E-1
	KEGG_PATHWAY	Arachidonic acid metabolism	RT		5	1,1	5,5E-2	7,3E-1
	KEGG_PATHWAY	Gastric cancer	RT		8	1,8	5,5E-2	7,3E-1
	KEGG_PATHWAY	Cushing syndrome	RT		8	1,8	6,3E-2	7,7E-1
■	KEGG_PATHWAY	Calcium signaling pathway	RT		11	2,4	6,4E-2	7,7E-1
■	KEGG_PATHWAY	PI3K-Akt signaling pathway	RT		14	3,1	6,9E-2	7,8E-1
	KEGG_PATHWAY	Hepatitis C	RT		8	1,8	7,0E-2	7,8E-1
	KEGG_PATHWAY	cGMP-PKG signaling pathway	RT		8	1,8	8,4E-2	8,6E-1
	KEGG_PATHWAY	Melanoma	RT		5	1,1	8,4E-2	8,6E-1
■	KEGG_PATHWAY	JAK-STAT signaling pathway	RT		8	1,8	8,8E-2	8,7E-1
	KEGG_PATHWAY	Pathogenic Escherichia coli infection	RT		9	2,0	9,1E-2	8,7E-1

Sublist	Category	Term	RT	Genes	Count	%	P-Value	Benjamini
	GOTERM_BP_DIRECT	axon guidance	RT		12	2,7	1,5E-3	1,0E0
	GOTERM_BP_DIRECT	calcium ion import across plasma membrane	RT		5	1,1	2,2E-3	1,0E0
	GOTERM_BP_DIRECT	proteolysis	RT		24	5,3	3,4E-3	1,0E0
	GOTERM_BP_DIRECT	bicellular tight junction assembly	RT		6	1,3	3,5E-3	1,0E0
	GOTERM_BP_DIRECT	sodium ion transmembrane transport	RT		9	2,0	5,1E-3	1,0E0
■	GOTERM_BP_DIRECT	immune response	RT		21	4,6	6,1E-3	1,0E0
	GOTERM_BP_DIRECT	purine nucleobase metabolic process	RT		3	0,7	7,9E-3	1,0E0
	GOTERM_BP_DIRECT	calcium-independent cell-cell adhesion via plasma membrane cell-adhesion molecules	RT		4	0,9	8,1E-3	1,0E0
	GOTERM_BP_DIRECT	regulation of cytosolic calcium ion concentration	RT		5	1,1	8,2E-3	1,0E0
	GOTERM_BP_DIRECT	potassium ion transport	RT		7	1,5	8,5E-3	1,0E0
	GOTERM_BP_DIRECT	positive regulation of stem cell proliferation	RT		5	1,1	9,7E-3	1,0E0
	GOTERM_BP_DIRECT	retina development in camera-type eye	RT		6	1,3	1,0E-2	1,0E0
	GOTERM_BP_DIRECT	negative regulation of platelet activation	RT		3	0,7	1,0E-2	1,0E0
	GOTERM_BP_DIRECT	keratinocyte differentiation	RT		6	1,3	1,1E-2	1,0E0
	GOTERM_BP_DIRECT	regulation of monoatomic ion transmembrane transport	RT		4	0,9	1,2E-2	1,0E0
	GOTERM_BP_DIRECT	potassium ion import across plasma membrane	RT		5	1,1	1,3E-2	1,0E0
	GOTERM_BP_DIRECT	cell adhesion	RT		20	4,4	1,8E-2	1,0E0
	GOTERM_BP_DIRECT	chemical synaptic transmission	RT		11	2,4	1,8E-2	1,0E0
	GOTERM_BP_DIRECT	sodium ion transport	RT		6	1,3	1,8E-2	1,0E0
	GOTERM_BP_DIRECT	acetylcholine receptor signaling pathway	RT		4	0,9	2,0E-2	1,0E0
	GOTERM_BP_DIRECT	wound healing	RT		6	1,3	2,0E-2	1,0E0
■	GOTERM_BP_DIRECT	nervous system development	RT		17	3,8	2,1E-2	1,0E0
	GOTERM_BP_DIRECT	monoatomic ion transport	RT		8	1,8	2,1E-2	1,0E0
	GOTERM_BP_DIRECT	cell surface receptor signaling pathway	RT		14	3,1	2,3E-2	1,0E0
	GOTERM_BP_DIRECT	epidermis development	RT		6	1,3	2,3E-2	1,0E0
	GOTERM_BP_DIRECT	G protein-coupled purinergic nucleotide receptor signaling pathway	RT		3	0,7	2,7E-2	1,0E0
	GOTERM_BP_DIRECT	regulation of cell adhesion	RT		5	1,1	2,7E-2	1,0E0
	GOTERM_BP_DIRECT	calcium-mediated signaling	RT		7	1,5	2,8E-2	1,0E0
	GOTERM_BP_DIRECT	associative learning	RT		4	0,9	3,2E-2	1,0E0
■	GOTERM_BP_DIRECT	signal transduction	RT		37	8,2	3,3E-2	1,0E0
	GOTERM_BP_DIRECT	neuron remodeling	RT		3	0,7	3,5E-2	1,0E0
	GOTERM_BP_DIRECT	regulation of macromolecule metabolic process	RT		3	0,7	3,5E-2	1,0E0
	GOTERM_BP_DIRECT	positive regulation of synapse assembly	RT		5	1,1	3,6E-2	1,0E0
	GOTERM_BP_DIRECT	blood coagulation, common pathway	RT		2	0,4	4,0E-2	1,0E0
	GOTERM_BP_DIRECT	positive regulation of activation of membrane attack complex	RT		2	0,4	4,0E-2	1,0E0
	GOTERM_BP_DIRECT	complement activation, alternative pathway	RT		3	0,7	4,0E-2	1,0E0
	GOTERM_BP_DIRECT	cellular response to interleukin-6	RT		3	0,7	4,0E-2	1,0E0
	GOTERM_BP_DIRECT	cellular response to lipopolysaccharide	RT		9	2,0	4,0E-2	1,0E0
	GOTERM_BP_DIRECT	dorsal/ventral pattern formation	RT		4	0,9	4,3E-2	1,0E0
	GOTERM_BP_DIRECT	cartilage development	RT		5	1,1	4,3E-2	1,0E0
	GOTERM_BP_DIRECT	positive regulation of angiogenesis	RT		8	1,8	4,4E-2	1,0E0
	GOTERM_BP_DIRECT	extracellular matrix organization	RT		8	1,8	4,7E-2	1,0E0
	GOTERM_BP_DIRECT	chondrocyte development	RT		3	0,7	5,0E-2	1,0E0
	GOTERM_BP_DIRECT	negative regulation of wound healing	RT		3	0,7	5,0E-2	1,0E0
	GOTERM_BP_DIRECT	protein localization to CENP-A containing chromatin	RT		3	0,7	5,0E-2	1,0E0
■	GOTERM_BP_DIRECT	inflammatory response	RT		15	3,3	5,3E-2	1,0E0
	GOTERM_BP_DIRECT	positive regulation of phosphatidylinositol 3-kinase/protein kinase B signal transduction	RT		9	2,0	5,3E-2	1,0E0
	GOTERM_BP_DIRECT	cilium movement	RT		4	0,9	5,5E-2	1,0E0
	GOTERM_BP_DIRECT	embryonic skeletal system morphogenesis	RT		4	0,9	5,5E-2	1,0E0
	GOTERM_BP_DIRECT	meiotic nuclear division	RT		2	0,4	5,9E-2	1,0E0
	GOTERM_BP_DIRECT	neural fold formation	RT		2	0,4	5,9E-2	1,0E0
	GOTERM_BP_DIRECT	positive regulation of chondrocyte differentiation	RT		3	0,7	6,0E-2	1,0E0
	GOTERM_BP_DIRECT	positive regulation of endocytosis	RT		3	0,7	6,0E-2	1,0E0
	GOTERM_BP_DIRECT	cellular response to ATP	RT		3	0,7	6,0E-2	1,0E0
■	GOTERM_BP_DIRECT	cell fate determination	RT		3	0,7	6,5E-2	1,0E0
	GOTERM_BP_DIRECT	regulation of cell migration	RT		6	1,3	6,6E-2	1,0E0
	GOTERM_BP_DIRECT	monoatomic ion transmembrane transport	RT		7	1,5	6,8E-2	1,0E0
	GOTERM_BP_DIRECT	regulation of cardiac conduction	RT		3	0,7	7,1E-2	1,0E0
	GOTERM_BP_DIRECT	negative regulation of fibroblast growth factor receptor signaling pathway	RT		3	0,7	7,1E-2	1,0E0
	GOTERM_BP_DIRECT	visual perception	RT		9	2,0	7,1E-2	1,0E0
	GOTERM_BP_DIRECT	negative regulation of T-helper 2 cell cytokine production	RT		2	0,4	7,8E-2	1,0E0
	GOTERM_BP_DIRECT	glossopharyngeal nerve morphogenesis	RT		2	0,4	7,8E-2	1,0E0
	GOTERM_BP_DIRECT	gamma-aminobutyric acid reuptake	RT		2	0,4	7,8E-2	1,0E0
	GOTERM_BP_DIRECT	renal D-glucose absorption	RT		2	0,4	7,8E-2	1,0E0
	GOTERM_BP_DIRECT	prolactin signaling pathway	RT		2	0,4	7,8E-2	1,0E0
	GOTERM_BP_DIRECT	fatty acid metabolic process	RT		6	1,3	7,9E-2	1,0E0
	GOTERM_BP_DIRECT	female pregnancy	RT		5	1,1	7,9E-2	1,0E0
	GOTERM_BP_DIRECT	G protein-coupled receptor signaling pathway	RT		26	5,8	8,1E-2	1,0E0
	GOTERM_BP_DIRECT	cardiac muscle cell action potential involved in contraction	RT		3	0,7	8,3E-2	1,0E0
	GOTERM_BP_DIRECT	synaptic transmission, cholinergic	RT		3	0,7	8,9E-2	1,0E0
	GOTERM_BP_DIRECT	adrenal gland development	RT		3	0,7	8,9E-2	1,0E0
	GOTERM_BP_DIRECT	cilium movement involved in cell motility	RT		3	0,7	8,9E-2	1,0E0
■	GOTERM_BP_DIRECT	BMP signaling pathway	RT		5	1,1	9,4E-2	1,0E0
	GOTERM_BP_DIRECT	adenylate cyclase-activating G protein-coupled receptor signaling pathway	RT		7	1,5	9,6E-2	1,0E0
	GOTERM_BP_DIRECT	Fc receptor signaling pathway	RT		2	0,4	9,6E-2	1,0E0
	GOTERM_BP_DIRECT	melanocyte proliferation	RT		2	0,4	9,6E-2	1,0E0
	GOTERM_BP_DIRECT	negative regulation of chondrocyte proliferation	RT		2	0,4	9,6E-2	1,0E0
	GOTERM_BP_DIRECT	calcium ion export across plasma membrane	RT		2	0,4	9,6E-2	1,0E0
	GOTERM_BP_DIRECT	positive regulation of meiotic nuclear division	RT		2	0,4	9,6E-2	1,0E0
	GOTERM_BP_DIRECT	phosphocreatine biosynthetic process	RT		2	0,4	9,6E-2	1,0E0

Figure S6: Gene set annotation analysis of RNA-seq data of the top-500 differentially expressed genes in RCH-ACV after treatment for NKX6-3 knockdown, using the online tool DAVID, revealing significant KEGG-pathways and GO-terms. Pathways/terms activated by NKX6-3 are shown on the left; selected are highlighted by red squares. Pathways/terms inhibited by NKX6-3 are shown on the right; selected are highlighted by green squares.

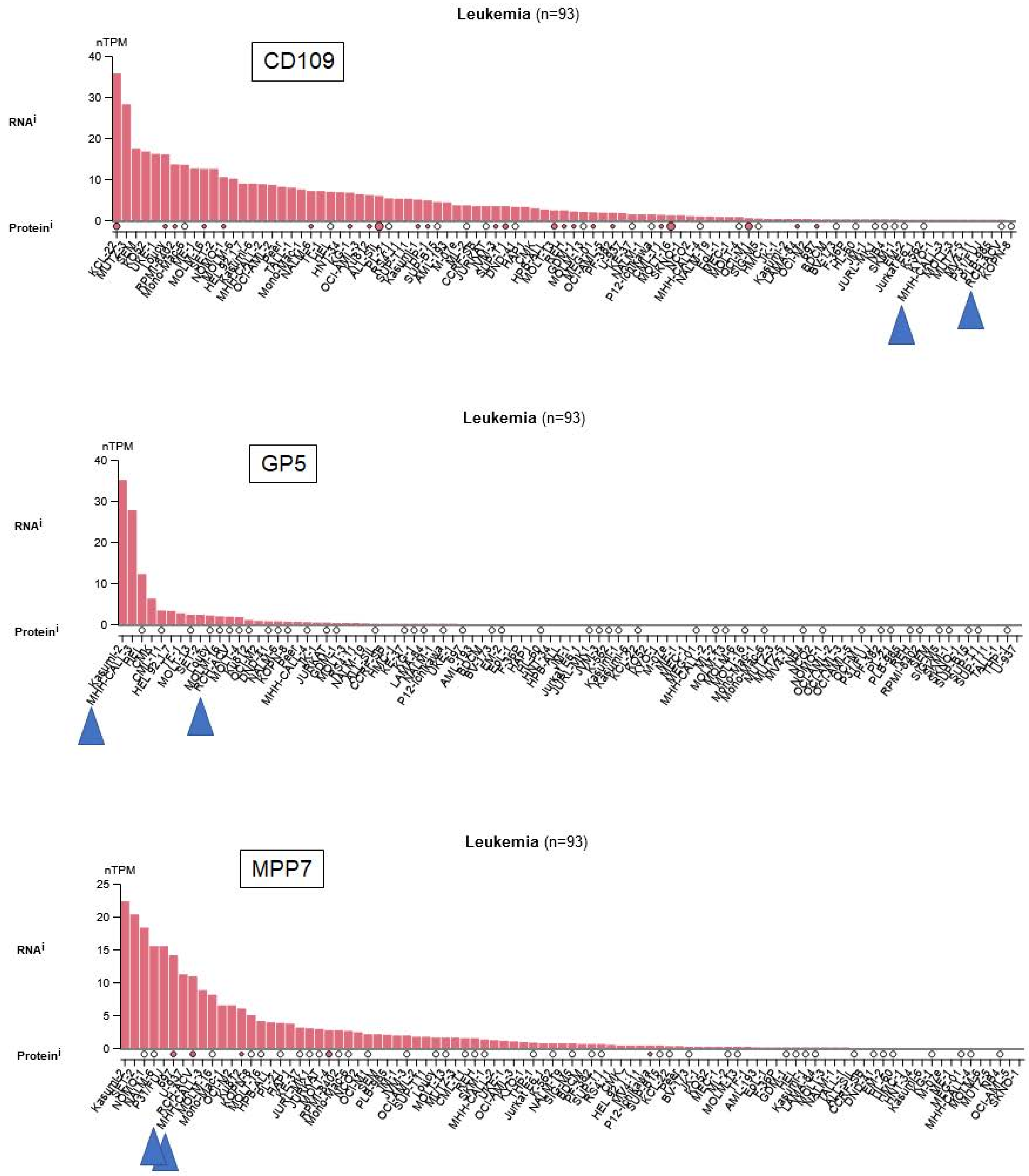

Figure S7: Gene expression values using RNA-seq data of CD109, GP5 and MPP7 for 93 leukemia cell lines, obtained from the public Human Protein Atlas. BCP-ALL cell lines RCH-ACV and MHH-CALL-3 are indicated by blue arrowheads.
