# Supplementary material for: NKX6-3 in B-Cell Progenitor Differentiation and Leukemia
Source: Genes (Basel). 2025 Oct 14;16(10):1199. doi: 10.3390/genes16101199 (PMC12563489; doi:10.3390/genes16101199)
Supplement: Supplementary file 1 [file genes-16-01199-s001.zip › Supplemental Tables 1-3.pdf]

Table S1: GEOR analysis of dataset GSE79533 (NKX6-3 high versus controls)

ID	P.Value	logFC	Gene.symbol	Gene.title
227441_s_at	5.61e-53	10.28	ANKS1B	ankyrin repeat and sterile alpha motif domain containing 1B
212151_at	2.32e-47	7.35	PBX1	PBX homeobox 1
223786_at	1.58e-46	7	CHST6	carbohydrate sulfotransferase 6
205253_at	2.40e-45	9.51	PBX1	PBX homeobox 1
227949_at	7.66e-43	7.94	PHACTR3	phosphatase and actin regulator 3
232289_at	3.76e-38	4.9	KCNJ12	ATP-sensitive inward rectifier potassium channel 12-like
203373_at	9.88e-38	-4.03	SOCS2	suppressor of cytokine signaling 2
231095_at	3.79e-36	4.65	LOC101928045	uncharacterized LOC101928045
231257_at	8.26e-35	5.95	TCERG1L	transcription elongation regulator 1 like
212148_at	2.23e-33	8.15	PBX1	PBX homeobox 1
1552960_at	1.87e-32	6.64	LRRC15	leucine rich repeat containing 15
204114_at	9.19e-32	3.8	NID2	nidogen 2
202796_at	4.25e-31	4.94	SYNPO	synaptopodin
213909_at	1.80e-30	5.52	LRRC15	leucine rich repeat containing 15
211913_s_at	3.02e-30	3.78	MERTK	MER proto-oncogene, tyrosine kinase
221585_at	4.93e-30	4.83	CACNG4	calcium voltage-gated channel auxiliary subunit gamma 4
240524_x_at	4.89e-29	5.1		
219989_s_at	5.57e-29	5.82	ANKS1B	ankyrin repeat and sterile alpha motif domain containing 1B
1552526_at	1.74e-28	5.2	FAM71C	family with sequence similarity 71 member C
227440_at	2.56e-28	5.75	ANKS1B	ankyrin repeat and sterile alpha motif domain containing 1B
235666_at	1.05e-27	4.44	ITGA8	integrin subunit alpha 8
205489_at	7.12e-27	4.47	CRYM	crystallin mu
214265_at	8.67e-27	5.94	ITGA8	integrin subunit alpha 8
234985_at	1.42e-26	6.41	LDLRAD3	low density lipoprotein receptor class A domain containing 3
207926_at	4.49e-26	4.53	GP5	glycoprotein V platelet
221113_s_at	4.89e-26	4.1	WNT16	Wnt family member 16
244338_at	5.32e-26	6.19		
210032_s_at	5.94e-26	6.08	SPAG6	sperm associated antigen 6
1564308_a_at	1.21e-25	3.31	MPP7	membrane palmitoylated protein 7
227676_at	1.42e-25	4.09	FAM3D	family with sequence similarity 3 member D
200953_s_at	4.23e-25	-3.47	CCND2	cyclin D2
217968_at	7.87e-25	3.06	TSSC1	tumor suppressing subtransferable candidate 1
224862_at	4.53e-24	-2	GNAQ	G protein subunit alpha q
243533_x_at	5.39e-24	6.26		
227439_at	5.43e-24	6.08	ANKS1B	ankyrin repeat and sterile alpha motif domain containing 1B
213005_s_at	5.87e-24	4.88	KANK1	KN motif and ankyrin repeat domains 1
233801_s_at	6.14e-24	4.89	SEMA6D	semaphorin 6D
220911_s_at	7.04e-24	4.1	NYNRIN	NYN domain and retroviral integrase containing
1562034_at	2.44e-23	4.39	LINC00163	long intergenic non-protein coding RNA 163
224998_at	4.48e-23	-3.49	CMTM4	CKLF like MARVEL transmembrane domain containing 4

235911_at	1.22e-22	3.93	MELTF	melanotransferrin
207110_at 12	1.27e-22	3.73	KCNJ12	ATP-sensitive inward rectifier potassium channel 12-like subfamily J member
205286_at	1.72e-22	6.22	TFAP2C	transcription factor AP-2 gamma
221021_s_at	2.08e-22	1.81	CTNNBL1	catenin beta like 1
225483_at	2.69e-22	2.7	VPS26B	VPS26, retromer complex component B
220373_at	5.78e-22	5.03	DCHS2	dachsous cadherin-related 2
203372_s_at	7.53e-22	-3.56	SOCS2	suppressor of cytokine signaling 2
206304_at	8.36e-22	4.35	MYBPH	myosin binding protein H
222936_s_at	1.10e-21	1.36	DESI2	desumoylating isopeptidase 2
208358_s_at	1.63e-21	5.2	UGT8	UDP glycosyltransferase 8
229258_at	1.67e-21	4.02	KIF12	kinesin family member 12
1554340_a_at	1.78e-21	4.42	DRAXIN	dorsal inhibitory axon guidance protein
226492_at	2.66e-21	5.03	SEMA6D	semaphorin 6D
206443_at	3.32e-21	5.4	RORB	RAR related orphan receptor B
1560066_at	8.97e-21	-6.66		
201029_s_at	1.08e-20	-1.93	CD99	CD99 molecule
210150_s_at	1.69e-20	3.67	LAMA5	laminin subunit alpha 5
232539_at	1.70e-20	-4.34	SOCS2	suppressor of cytokine signaling 2
1569122_at	2.02e-20	4.9	LOC105370697	uncharacterized LOC105370697
1556826_s_at	2.03e-20	4.42	DRAXIN	dorsal inhibitory axon guidance protein
1563000_at	2.12e-20	6.22		
205780_at	2.51e-20	4.23	BIK	BCL2 interacting killer
1555153_s_at	2.62e-20	-2.89	FCHO2	FCH domain only 2
202615_at	2.84e-20	-2.13	GNAQ	G protein subunit alpha q
224666_at	3.45e-20	2.49	NSMCE1	NSE1 homolog, SMC5-SMC6 complex component
235128_at	4.69e-20	4.34	SYNPO	synaptopodin
219489_s_at	9.00e-20	3.98	NXN	nucleoredoxin
239092_at	9.20e-20	6.19	ITGA8	integrin subunit alpha 8
207497_s_at	1.32e-19	3.72	MS4A2	membrane spanning 4-domains A2
234261_at	1.34e-19	4.26		
233882_s_at	1.70e-19	6.78	SEMA6D	semaphorin 6D
1562984_at	2.24e-19	3.78	LOC101928937	uncharacterized LOC101928937
228956_at	2.95e-19	5.61	UGT8	UDP glycosyltransferase 8
242764_at	3.16e-19	5.31	DCHS2	dachsous cadherin-related 2
220574_at	4.23e-19	4.55	SEMA6D	semaphorin 6D
211426_x_at	4.24e-19	-1.98	GNAQ	G protein subunit alpha q
211525_s_at	4.31e-19	5.4	GP5	glycoprotein V platelet
1564253_at	4.50e-19	4.11	LOC285766	uncharacterized LOC285766
240292_x_at	5.19e-19	7.16	ANKS1B	ankyrin repeat and sterile alpha motif domain containing 1B
1555626_a_at	5.54e-19	4.51	SLAMF1	signaling lymphocytic activation molecule family member 1
209760_at	7.59e-19	1.85	KIAA0922	KIAA0922
214432_at	7.75e-19	3.57	ATP1A3	ATPase Na+/K+ transporting subunit alpha 3

205287_s_at	8.07e-19	4.43	TFAP2C	transcription factor AP-2 gamma
1555536_at	8.66e-19	-3.04	ANTXR2	anthrax toxin receptor 2
223161_at	9.81e-19	-1.53	KIAA1147	KIAA1147
238451_at	1.21e-18	3.86	MPP7	membrane palmitoylated protein 7
213355_at	1.29e-18	-3.81	ST3GAL6ST3	beta-galactoside alpha-2,3-sialyltransferase 6
202178_at	1.36e-18	4.5	PRKCZ	protein kinase C zeta
224022_x_at	1.71e-18	6.31	WNT16	Wnt family member 16
206679_at	1.78e-18	4.67	APBA1	amyloid beta precursor protein binding family A member 1
206028_s_at	2.80e-18	3.94	MERTK	MER proto-oncogene, tyrosine kinase
223693_s_at	2.91e-18	3.31	RADIL	Rap associating with DIL domain
1558368_s_at	3.41e-18	3.64	DRAXIN	dorsal inhibitory axon guidance protein
210942_s_at	3.43e-18	-3.91	ST3GAL6ST3	beta-galactoside alpha-2,3-sialyltransferase 6
239719_at	3.48e-18	-3.2	CD109	CD109 molecule
222158_s_at	3.51e-18	1.54	DESI2	desumoylating isopeptidase 2
226828_s_at	4.28e-18	3.14	HEYL	hes related family bHLH transcription factor with YRPW motif-like
231737_at	5.02e-18	3.84	CACNG4	calcium voltage-gated channel auxiliary subunit gamma 4
202479_s_at	5.63e-18	3.48	TRIB2	tribbles pseudokinase 2
243526_at	6.09e-18	4.82	WDR86	WD repeat domain 86
231924_at	6.17e-18	-4.92	LINC00958	long intergenic non-protein coding RNA 958
210033_s_at	7.74e-18	5.95	SPAG6	sperm associated antigen 6
209570_s_at	8.00e-18	2.77	NSG1	neuron specific gene family member 1
205897_at	1.09e-17	5.08	NFATC4	nuclear factor of activated T-cells 4
206181_at	1.24e-17	4.2	SLAMF1	signaling lymphocytic activation molecule family member 1
213611_at	1.31e-17	4.35	AQP5	aquaporin 5
205663_at	1.36e-17	2.24	PCBP3	poly(rC) binding protein 3
230015_at	1.44e-17	-3.04	PRCD	progressive rod-cone degeneration
209569_x_at	1.70e-17	4.21	NSG1	neuron specific gene family member 1
211401_s_at	1.98e-17	3.9	FGFR2	fibroblast growth factor receptor 2
202242_at	2.06e-17	-5.29	TSPAN7	tetraspanin 7
1562945_at	2.08e-17	3.58	LOC105378130	uncharacterized LOC105378130
226631_at	2.71e-17	-1.25	METTL10	methyltransferase like 10
232950_s_at	3.91e-17	3.29	PITPNM2	phosphatidylinositol transfer protein membrane associated 2
208567_s_at 12	3.91e-17	3.46	KCNJ12	ATP-sensitive inward rectifier potassium channel 12-like subfamily J member
230067_at	4.15e-17	4.63	FAM124A	family with sequence similarity 124 member A
222592_s_at	4.42e-17	-1.78	ACSL5	acyl-CoA synthetase long-chain family member 5
230161_at	5.50e-17	-3.01		
224863_at	6.33e-17	-2.03	GNAQ	G protein subunit alpha q
239087_at	7.16e-17	3.91	ANKS4B	ankyrin repeat and sterile alpha motif domain containing 4B
220043_s_at	7.28e-17	4.35	MELTF	melanotransferrin
206999_at	7.53e-17	3.87	IL12RB2	interleukin 12 receptor subunit beta 2
239427_at	8.58e-17	4.4	SLAMF1	signaling lymphocytic activation molecule family member 1
227423_at	1.17e-16	2.4	LRRC28	leucine rich repeat containing 28

242385_at	1.36e-16	5.2	RORB	RAR related orphan receptor B
202289_s_at	1.41e-16	4.04	TACC2	transforming acidic coiled-coil containing protein 2
227806_at	1.75e-16	3.78	C16orf74	chromosome 16 open reading frame 74
231040_at	2.00e-16	6.65	RORB	RAR related orphan receptor B
206690_at	2.15e-16	4.25	ASIC2	acid sensing ion channel subunit 2
206312_at	2.33e-16	3.78	GUCY2C	guanylate cyclase 2C
1559732_at	2.41e-16	3.89	NUB1	negative regulator of ubiquitin like proteins 1
212789_at	2.59e-16	2.06	NCAPD3	non-SMC condensin II complex subunit D3
229900_at	2.59e-16	-3.34	CD109	CD109 molecule
229698_at	2.61e-16	-7.2	SHANK3	SH3 and multiple ankyrin repeat domains 3
224733_at	3.54e-16	-2.03	CMTM3	CKLF like MARVEL transmembrane domain containing 3
211382_s_at	3.57e-16	5.21	TACC2	transforming acidic coiled-coil containing protein 2
204202_at	3.80e-16	2.21	IQCE	IQ motif containing E
230519_at	3.91e-16	3.56	FAM124A	family with sequence similarity 124 member A
201579_at	4.15e-16	6	FAT1	FAT atypical cadherin 1
226796_at	4.32e-16	3.05	ABHD15	abhydrolase domain containing 15
1557326_at	4.81e-16	5.42		
211819_s_at	5.81e-16	3.64	SORBS1	sorbin and SH3 domain containing 1
228220_at	6.08e-16	-2.3	FCHO2	FCH domain only 2
226949_at	6.34e-16	1.36	GOLGA3	golgin A3
222258_s_at	8.08e-16	5.19	SH3BP4	SH3 domain binding protein 4
239657_x_at	8.57e-16	3.75	FOXO6	forkhead box O6
228249_at	8.76e-16	-2.83	C11orf74	chromosome 11 open reading frame 74
227923_at	8.94e-16	-6.74	SHANK3	SH3 and multiple ankyrin repeat domains 3
235209_at	9.03e-16	3.21	SBSPON	somatomedin B and thrombospondin type 1 domain containing
223162_s_at	9.34e-16	-1.73	KIAA1147	KIAA1147
230128_at	9.41e-16	3.23	CKAP2	cytoskeleton associated protein 2
229233_at	9.52e-16	6.26	NRG3	neuregulin 3
225389_at	1.08e-15	1.72	BTBD6	BTB domain containing 6
212589_at	1.23e-15	-3.05	RRAS2	related RAS viral (r-ras) oncogene homolog 2
229288_at	1.32e-15	-6.33	EPHA7	EPH receptor A7
1554629_at	1.40e-15	-6.57	EPHA7	EPH receptor A7
215283_at	1.50e-15	-1.64	LINC00667	long intergenic non-protein coding RNA 667
205477_s_at	1.54e-15	2.58	AMBP	alpha-1-microglobulin/bikunin precursor
241456_at	1.85e-15	3.4	FAM78B	family with sequence similarity 78 member B
238870_at	1.88e-15	4.12	KCNK9	potassium two pore domain channel subfamily K member 9
229247_at	2.04e-15	4.75	FBLN7	fibulin 7
226545_at	2.93e-15	-3.82	CD109	CD109 molecule
1552885_a_at	3.55e-15	2.33	NKX6-3	NK6 homeobox 3
220169_at	3.82e-15	-3.35	TMEM156	transmembrane protein 156
200952_s_at	5.13e-15	-3.24	CCND2	cyclin D2
238778_at	7.59e-15	3.54	MPP7	membrane palmitoylated protein 7
213335_s_at	7.82e-15	-2.7	ST3GAL6ST3	beta-galactoside alpha-2,3-sialyltransferase 6

220034_at	9.08e-15	-2.47	IRAK3	interleukin 1 receptor associated kinase 3
218980_at	9.55e-15	2.42	FHOD3	formin homology 2 domain containing 3
1554957_at	9.64e-15	3.46	LOC101928921	uncharacterized LOC101928921
226997_at	1.29e-14	3.56	ADAMTS12	ADAM metallopeptidase with thrombospondin type 1 motif 12
220117_at	1.54e-14	3.8	ZNF385D	zinc finger protein 385D
226192_at	1.65e-14	3.38	AR	androgen receptor
217124_at	1.86e-14	2.15	IQCE	IQ motif containing E
228160_at	1.91e-14	-2.24	LINC00667	long intergenic non-protein coding RNA 667
225540_at	1.92e-14	3.07	MAP2	microtubule associated protein 2
1565734_at	1.95e-14	-4.46		
1553446_at	1.97e-14	4.67	LOC100996634	transmembrane protein FLJ37396
226944_at	2.05e-14	2.29	HTRA3	HtrA serine peptidase 3
222513_s_at	2.15e-14	3.52	SORBS1	sorbin and SH3 domain containing 1
215764_x_at	2.31e-14	1.48	AP2A2	adaptor related protein complex 2 alpha 2 subunit
218627_at	2.41e-14	-2.09	DRAM1	DNA damage regulated autophagy modulator 1
241844_x_at	2.58e-14	-3.63	TMEM156	transmembrane protein 156
208591_s_at	2.93e-14	-2.56	PDE3B	phosphodiesterase 3B
225009_at	3.11e-14	-2.5	CMTM4	CKLF like MARVEL transmembrane domain containing 4
218949_s_at	3.52e-14	2.4	QRSL1	glutaminyl-tRNA synthase (glutamine-hydrolyzing)-like 1
204262_s_at	3.61e-14	3.04	PSEN2	presenilin 2
230834_at	3.86e-14	2.04		
210957_s_at	3.87e-14	3.91	AFF2	AF4/FMR2 family member 2
237495_at	4.13e-14	3.18	MPP7	membrane palmitoylated protein 7
204276_at	4.30e-14	-1.63	TK2	thymidine kinase 2, mitochondrial
206404_at	4.76e-14	3.49	FGF9	fibroblast growth factor 9
1556538_at	5.11e-14	2.63	MELTF	melanotransferrin
1559254_at	5.15e-14	2.95	PICSAR	P38 inhibited cutaneous squamous cell carcinoma associated lincRNA
1563802_at	5.19e-14	2.14	LINC01226	long intergenic non-protein coding RNA 1226
203865_s_at	5.32e-14	3.77	ADARB1	adenosine deaminase, RNA specific B1
226634_at	5.97e-14	-1.47	METTL10	methyltransferase like 10
213940_s_at	6.69e-14	-1.51	FNBP1	formin binding protein 1
224516_s_at	9.04e-14	3.31	CXXC5	CXXC finger protein 5
204562_at	9.43e-14	2.83	IRF4	interferon regulatory factor 4
1552511_a_at	9.96e-14	4.21	CPA6	carboxypeptidase A6
239650_at	1.04e-13	6.66	NCKAP5	NCK associated protein 5
239691_at	1.09e-13	5.01	C12orf77	chromosome 12 open reading frame 77
204438_at	1.15e-13	-4	MRC1	mannose receptor, C type 1
218322_s_at	1.23e-13	-1.98	ACSL5	acyl-CoA synthetase long-chain family member 5
228101_at	1.28e-13	3.73	APBA1	amyloid beta precursor protein binding family A member 1
1567219_at	1.40e-13	1.69		
1565735_at	1.46e-13	-2.82		
211702_s_at	1.56e-13	-1.09	USP32	ubiquitin specific peptidase 32
238804_at	1.61e-13	2.85		

222317_at	1.76e-13	-3.22	PDE3B	phosphodiesterase 3B
212735_at	1.76e-13	1.8	RUBCN	RUN and cysteine rich domain containing beclin 1 interacting protein
1560225_at	1.77e-13	4.14	CNR1	cannabinoid receptor 1
231259_s_at	1.84e-13	-3.4	CCND2	cyclin D2
209559_at	1.88e-13	2.41	HIP1R	huntingtin interacting protein 1 related
32625_at	1.96e-13	-4.18	NPR1	natriuretic peptide receptor 1
211323_s_at	2.06e-13	-1.92	ITPR1	inositol 1,4,5-trisphosphate receptor type 1
235914_at	2.24e-13	2.89	SYNPO	synaptopodin
227589_at	2.48e-13	2.46	PITPNC1	phosphatidylinositol transfer protein, cytoplasmic 1
222378_at	2.50e-13	-2.95		
244394_at	2.60e-13	3.04		
216364_s_at	2.62e-13	4.41	AFF2	AF4/FMR2 family member 2
225235_at	2.67e-13	2.33	TSPAN17	tetraspanin 17
214582_at	2.70e-13	-2.8	PDE3B	phosphodiesterase 3B
62987_r_at	3.02e-13	2.26	CACNG4	calcium voltage-gated channel auxiliary subunit gamma 4
1566647_s_at	3.16e-13	3.3	LINC01225	long intergenic non-protein coding RNA 1225
235246_at	3.23e-13	3.72	WDR86	WD repeat domain 86
212590_at	3.34e-13	-2.56	RRAS2	related RAS viral (r-ras) oncogene homolog 2
212288_at	3.89e-13	-2.11	FNBP1	formin binding protein 1
240727_s_at	4.10e-13	2.11		
224861_at	4.32e-13	-2.07	GNAQ	G protein subunit alpha q
1563494_at	4.45e-13	4.21		
231793_s_at	5.99e-13	2.91	CAMK2D	calcium/calmodulin dependent protein kinase II delta
244306_at	6.68e-13	5.03		
202478_at	6.89e-13	3.26	TRIB2	tribbles pseudokinase 2
44790_s_at	7.10e-13	-3.08	KIAA0226L	KIAA0226 like
228900_at	7.16e-13	-2.58	SPECC1	sperm antigen with calponin homology and coiled-coil domains 1
233144_s_at	7.57e-13	3.06	RASAL1	RAS protein activator like 1
242747_at	7.58e-13	5.72		
235210_s_at	7.59e-13	1.78	SBSPON	somatomedin B and thrombospondin type 1 domain containing
241013_at	7.61e-13	2.98	FAM124A	family with sequence similarity 124 member A
225598_at	8.23e-13	1.66	SLC45A4	solute carrier family 45 member 4
235721_at	8.57e-13	3.74	DTX3	deltex E3 ubiquitin ligase 3
205768_s_at	1.11e-12	2.87	SLC27A2	solute carrier family 27 member 2
209295_at	1.22e-12	-1.99	TNFRSF10B	TNF receptor superfamily member 10b
220567_at	1.22e-12	3.31	IKZF2	IKAROS family zinc finger 2
1564337_at	1.23e-12	3.87	LOC101929538	uncharacterized LOC101929538
1560180_at	1.30e-12	2.87		
205805_s_at	1.51e-12	5.04	ROR1	receptor tyrosine kinase like orphan receptor 1
234338_s_at	1.63e-12	2.6	ZBTB47	zinc finger and BTB domain containing 47

Table S2: GEOR analysis of dataset GSE13576 (PBX1 versus controls)

ID	P.Value	logFC	Gene.symbol	Gene.title
212151_at	6.38e-32	6.308	PBX1	PBX homeobox 1
212148_at	2.10e-30	7.067	PBX1	PBX homeobox 1
205253_at	1.25e-27	5.448	PBX1	PBX homeobox 1
203373_at	1.08e-25	-4.898	SOCS2	suppressor of cytokine signaling 2
227441_s_at	4.77e-25	4.28	ANKS1B	ankyrin repeat and sterile alpha motif domain containing 1B
203372_s_at	4.94e-25	-5.925	SOCS2	suppressor of cytokine signaling 2
227439_at	1.52e-24	4.008	ANKS1B	ankyrin repeat and sterile alpha motif domain containing 1B
231040_at	2.44e-24	4.027	RORB	RAR related orphan receptor B
242385_at	5.01e-24	4.156	RORB	RAR related orphan receptor B
224022_x_at	1.39e-23	4.847	WNT16	Wnt family member 16
227440_at	1.62e-21	3.118	ANKS1B	ankyrin repeat and sterile alpha motif domain containing 1B
210150_s_at	3.15e-21	2.312	LAMA5	laminin subunit alpha 5
229247_at	4.07e-21	3.12	FBLN7	fibulin 7
227949_at	4.21e-21	6.086	PHACTR3	phosphatase and actin regulator 3
207110_at	1.51e-20	3.29	KCNJ12	potassium voltage-gated channel subfamily J member 12
206028_s_at	1.15e-19	4.226	MERTK	MER proto-oncogene, tyrosine kinase
1557326_at	1.74e-19	2.311		
201579_at	3.00e-19	5.082	FAT1	FAT atypical cadherin 1
235911_at	4.62e-19	3.949	MELTF	melanotransferrin
1559315_s_at	5.91e-19	-3.78	SOCS2-AS1	SOCS2 antisense RNA 1
225235_at	2.72e-18	1.987	TSPAN17	tetraspanin 17
208358_s_at	3.35e-18	1.817	UGT8	UDP glycosyltransferase 8
209569_x_at	5.00e-18	3.113	NSG1	neuron specific gene family member 1
202178_at	8.26e-18	5.196	PRKCZ	protein kinase C zeta
209570_s_at	1.08e-17	3.149	NSG1	neuron specific gene family member 1
219989_s_at	1.23e-17	1.663	ANKS1B	ankyrin repeat and sterile alpha motif domain containing 1B
223562_at	1.40e-17	-2.408	PARVG	parvin gamma
231095_at	1.59e-17	3.514	LOC101928045	uncharacterized LOC101928045
230834_at	2.32e-17	1.913		
232289_at	4.26e-17	2.933	KCNJ12	potassium voltage-gated channel subfamily J member 12
235666_at	4.40e-17	3.336	ITGA8	integrin subunit alpha 8
207926_at	4.43e-17	2.582	GP5	glycoprotein V platelet
204114_at	4.68e-17	5.164	NID2	nidogen 2
1564308_a_at	4.90e-17	2.297	MPP7	membrane palmitoylated protein 7
227230_s_at	5.38e-17	2.247	KIAA1211	KIAA1211
213533_at	5.56e-17	1.788	NSG1	neuron specific gene family member 1
234985_at	8.13e-17	4.581	LDLRAD3	low density lipoprotein receptor class A domain containing 3
214265_at	9.52e-17	3.55	ITGA8	integrin subunit alpha 8
229770_at	1.22e-16	2.309	GLT1D1	glycosyltransferase 1 domain containing 1
206443_at	1.71e-16	1.915	RORB	RAR related orphan receptor B

223693_s_at	2.40e-16	3.204	RADIL	Rap associating with DIL domain
1559732_at	2.56e-16	2.367	NUB1	negative regulator of ubiquitin like proteins 1
232060_at	2.66e-16	2.404	ROR1	receptor tyrosine kinase like orphan receptor 1
243533_x_at	3.29e-16	2.648		
239657_x_at	5.04e-16	3.172	FOXO6	forkhead box O6
46665_at	1.76e-15	2.735	SEMA4C	semaphorin 4C
223786_at	1.86e-15	2.798	CHST6	carbohydrate sulfotransferase 6
208567_s_at	2.01e-15	2.396	KCNJ12	potassium voltage-gated channel subfamily J member 12
212873_at	2.92e-15	-1.182	ARHGAP45	Rho GTPase activating protein 45
221113_s_at	6.22e-15	2.209	WNT16	Wnt family member 16
227231_at	7.90e-15	1.45	KIAA1211	KIAA1211
228956_at	1.20e-14	2.511	UGT8	UDP glycosyltransferase 8
238778_at	1.43e-14	3.855	MPP7	membrane palmitoylated protein 7
237974_at	1.50e-14	3.25	MIR4454	microRNA 4454
222513_s_at	1.62e-14	2.424	SORBS1	sorbin and SH3 domain containing 1
236565_s_at	2.83e-14	2.272	LARP6	La ribonucleoprotein domain family member 6
223723_at	2.97e-14	2.085	MELTF	melanotransferrin
228262_at	3.18e-14	3.004	MAP7D2	MAP7 domain containing 2
205489_at	4.25e-14	2.445	CRYM	crystallin mu
206105_at	6.68e-14	0.74	AFF2	AF4/FMR2 family member 2
234261_at	7.87e-14	2.309		
231257_at	8.58e-14	3.603	TCERG1L	transcription elongation regulator 1 like
203865_s_at	1.20e-13	3.46	ADARB1	adenosine deaminase, RNA specific B1
219740_at	1.57e-13	3.021	VASH2	vasohibin 2
227909_at	1.77e-13	2.016	SMIM10L2B	small integral membrane protein 10 like 2B
216986_s_at	1.83e-13	1.091	IRF4	interferon regulatory factor 4
239427_at	2.01e-13	2.618	SLAMF1	signaling lymphocytic activation molecule family member 1
205769_at	2.32e-13	2.656	SLC27A2	solute carrier family 27 member 2
225483_at	2.50e-13	2.787	VPS26B	VPS26, retromer complex component B
202479_s_at	2.81e-13	3.417	TRIB2	tribbles pseudokinase 2
229113_s_at	3.13e-13	-1.377	FAAP20	Fanconi anemia core complex associated protein 20
227676_at	3.59e-13	2.032	FAM3D	family with sequence similarity 3 member D
240292_x_at	3.74e-13	1.421	ANKS1B	ankyrin repeat and sterile alpha motif domain containing 1B
203998_s_at	3.75e-13	3.617	SYT1	synaptotagmin 1
205768_s_at	4.15e-13	2.494	SLC27A2	solute carrier family 27 member 2
204014_at	4.50e-13	2.97	DUSP4	dual specificity phosphatase 4
233510_s_at	4.54e-13	-2.387	PARVG	parvin gamma
226192_at	4.77e-13	1.775	AR	androgen receptor
208788_at	5.48e-13	1.315	ELOVL5	ELOVL fatty acid elongase 5
238451_at	5.66e-13	1.703	MPP7	membrane palmitoylated protein 7
207237_at	6.23e-13	3.16	KCNA3	potassium voltage-gated channel subfamily A member 3
232950_s_at	6.24e-13	1.919	PITPNM2	phosphatidylinositol transfer protein membrane associated 2
238804_at	6.90e-13	1.479		

213909_at	7.01e-13	2.831	LRRC15	leucine rich repeat containing 15
216364_s_at	7.15e-13	0.976	AFF2	AF4/FMR2 family member 2
206181_at	9.59e-13	3.546	SLAMF1	signaling lymphocytic activation molecule family member 1
224733_at	1.00e-12	-2.511	CMTM3	CKLF like MARVEL transmembrane domain containing 3
1569122_at	1.05e-12	1.851	LOC105370697	uncharacterized LOC105370697
1552924_a_at	1.23e-12	2.29	PITPNM2	phosphatidylinositol transfer protein membrane associated 2
210613_s_at	1.25e-12	-1.984	SYNGR1	synaptogyrin 1
226961_at	1.30e-12	1.638	PRR15	proline rich 15
230306_at	1.33e-12	1.953	VPS26B	VPS26, retromer complex component B
1560109_s_at	1.48e-12	1.912	NUB1	negative regulator of ubiquitin like proteins 1
239650_at	1.57e-12	2.839	NCKAP5	NCK associated protein 5
205559_s_at	1.83e-12	1.651	PCSK5	proprotein convertase subtilisin/kexin type 5
209901_x_at	1.89e-12	-1.787	AIF1	allograft inflammatory factor 1
229258_at	1.92e-12	1.365	KIF12	kinesin family member 12
212538_at	2.07e-12	2.656	DOCK9	dedicator of cytokinesis 9
204425_at	2.24e-12	-1.337	ARHGAP4	Rho GTPase activating protein 4
243084_at	2.54e-12	0.925	CALD1	caldesmon 1
226949_at	3.02e-12	1.989	GOLGA3	golgin A3
215051_x_at	3.33e-12	-1.843	AIF1	allograft inflammatory factor 1
225855_at	3.38e-12	1.506	EPB41L5	erythrocyte membrane protein band 4.1 like 5
230486_at	3.52e-12	1.179		
221092_at	3.88e-12	1.506	IKZF3	IKAROS family zinc finger 3
226188_at	4.04e-12	1.039	LGALSL	galectin like
229997_at	4.48e-12	1.915	VANGL1	VANGL planar cell polarity protein 1
239082_at	6.56e-12	1.388	FZD3	frizzled class receptor 3
240725_at	7.83e-12	0.854		
213638_at	8.24e-12	1.956	PHACTR1	phosphatase and actin regulator 1
1555705_a_at	1.15e-11	-2.362	CMTM3	CKLF like MARVEL transmembrane domain containing 3
213050_at	1.19e-11	2.731	COBL	cordon-bleu WH2 repeat protein
211913_s_at	1.28e-11	1.599	MERTK	MER proto-oncogene, tyrosine kinase
205525_at	1.29e-11	1.268	CALD1	caldesmon 1
222158_s_at	1.36e-11	1.664	DESI2	desumoylating isopeptidase 2
243000_at	1.46e-11	-1.727	CDK6	cyclin dependent kinase 6
205477_s_at	1.56e-11	0.801	AMBP	alpha-1-microglobulin/bikunin precursor
235343_at	1.64e-11	3.106	VASH2	vasohibin 2
1564337_at	1.65e-11	1.017	LOC101929538	uncharacterized LOC101929538
229655_at	2.44e-11	3.158	FAM19A5	family with sequence similarity 19 member A5, C-C motif chemokine like
229459_at	2.46e-11	3.743	FAM19A5	family with sequence similarity 19 member A5, C-C motif chemokine like
239092_at	2.63e-11	1.911	ITGA8	integrin subunit alpha 8
1561135_at	2.99e-11	1.904		
242764_at	3.09e-11	1.274	DCHS2	dachsous cadherin-related 2
204262_s_at	3.63e-11	1.043	PSEN2	presenilin 2
237094_at	4.70e-11	3.209	FAM19A5	family with sequence similarity 19 member A5, C-C motif chemokine like

212552_at	5.14e-11	-1.538	HPCAL1	hippocalcin like 1
235171_at	5.15e-11	-2.765	LOC100505501	uncharacterized LOC100505501
202615_at	5.26e-11	-1.726	GNAQ	G protein subunit alpha q
233273_at	5.64e-11	1.28		
207661_s_at	6.02e-11	1.789	SH3PXD2A	SH3 and PX domains 2A
227030_at	6.48e-11	2.102	IKZF3	IKAROS family zinc finger 3
202771_at	7.70e-11	-1.975	PIEZO1	piezo type mechanosensitive ion channel component 1
229414_at	7.82e-11	1.738	PITPNC1	phosphatidylinositol transfer protein, cytoplasmic 1
214786_at	8.40e-11	2.512	MAP3K1	mitogen-activated protein kinase kinase kinase 1
202206_at	8.62e-11	3.875	ARL4C	ADP ribosylation factor like GTPase 4C
226197_at	9.04e-11	1.062	AR	androgen receptor
213358_at	9.80e-11	3.734	MTCL1	microtubule crosslinking factor 1
202796_at	1.20e-10	2.125	SYNPO	synaptopodin
201029_s_at	1.25e-10	-2.17	CD99	CD99 molecule
236677_at	1.31e-10	1.128	NGB	neuroglobin
232951_at	1.33e-10	2.039		
212845_at	1.44e-10	2.604	SAMD4A	sterile alpha motif domain containing 4A
231929_at	1.56e-10	3.239	IKZF2	IKAROS family zinc finger 2
224862_at	1.59e-10	-2.066	GNAQ	G protein subunit alpha q
201617_x_at	1.72e-10	1.863	CALD1	caldesmon 1
205893_at	1.87e-10	1.764	NLGN1	neuroligin 1
230487_at	1.99e-10	1.052	C6orf99	chromosome 6 open reading frame 99
212589_at	2.02e-10	-2.788	RRAS2	related RAS viral (r-ras) oncogene homolog 2
213095_x_at	2.05e-10	-1.561	AIF1	allograft inflammatory factor 1
212026_s_at	2.14e-10	-0.962	EXOC7	exocyst complex component 7
230519_at	2.25e-10	1.23	FAM124A	family with sequence similarity 124 member A
226223_at	2.26e-10	2.73	PAWR	pro-apoptotic WT1 regulator
212590_at	2.28e-10	-2.322	RRAS2	related RAS viral (r-ras) oncogene homolog 2
218322_s_at	2.30e-10	-1.945	ACSL5	acyl-CoA synthetase long-chain family member 5
202478_at	2.35e-10	3.502	TRIB2	tribbles pseudokinase 2
205159_at	2.50e-10	2.761	CSF2RB	colony stimulating factor 2 receptor beta common subunit
219039_at	2.50e-10	1.063	SEMA4C	semaphorin 4C
211373_s_at	2.51e-10	1.503	PSEN2	presenilin 2
230389_at	2.52e-10	-2.509	FNBP1	formin binding protein 1
206312_at	2.68e-10	1.016	GUCY2C	guanylate cyclase 2C
204430_s_at	2.84e-10	-2.545	SLC2A5	solute carrier family 2 member 5
232267_at	2.87e-10	2.37	ADGRD1	adhesion G protein-coupled receptor D1
236301_at	3.02e-10	1.019	IKZF3	IKAROS family zinc finger 3
226545_at	3.05e-10	-2.931	CD109	CD109 molecule
234799_at	3.10e-10	0.8	ADARB1	adenosine deaminase, RNA specific B1
235721_at	3.24e-10	1.869	DTX3	deltex E3 ubiquitin ligase 3
231340_at	3.31e-10	1.888		
225369_at	3.51e-10	2.044	ESAM	endothelial cell adhesion molecule

201616_s_at	3.57e-10	1.673	CALD1	caldesmon 1
213940_s_at	3.57e-10	-2.209	FNBP1	formin binding protein 1
212077_at	3.59e-10	3.936	CALD1	caldesmon 1
1556499_s_at	3.84e-10	2.79	COL1A1	collagen type I alpha 1 chain
217983_s_at	4.73e-10	-1.762	RNASET2	ribonuclease T2
212651_at	4.76e-10	2.154	RHOBTB1	Rho related BTB domain containing 1
222592_s_at	4.78e-10	-1.433	ACSL5	acyl-CoA synthetase long-chain family member 5
1554271_a_at	5.13e-10	1.373	CENPL	centromere protein L
204004_at	5.25e-10	2.133	PAWR	pro-apoptotic WT1 regulator
204562_at	5.36e-10	2.417	IRF4	interferon regulatory factor 4
219663_s_at	5.46e-10	1.256	TMEM121	transmembrane protein 121
217984_at	6.04e-10	-1.532	RNASET2	ribonuclease T2
212789_at	6.35e-10	1.653	NCAPD3	non-SMC condensin II complex subunit D3
1554080_at	6.90e-10	1.197	CNOT9	CCR4-NOT transcription complex subunit 9
236270_at	6.99e-10	1.655	NFATC4	nuclear factor of activated T-cells 4
204005_s_at	7.15e-10	1.869	PAWR	pro-apoptotic WT1 regulator
200696_s_at	8.20e-10	-2.174	GSN	gelsolin
211582_x_at	9.14e-10	-2.682	LST1	leukocyte specific transcript 1
225144_at	9.39e-10	2.014	BMPR2	bone morphogenetic protein receptor type 2
243526_at	9.98e-10	1.911	WDR86	WD repeat domain 86
220373_at	1.02e-09	1.101	DCHS2	dachsous cadherin-related 2
229072_at	1.03e-09	2.257	RAB30	RAB30, member RAS oncogene family
209691_s_at	1.04e-09	-1.739	DOK4	docking protein 4
202106_at	1.09e-09	1.754	GOLGA3	golgin A3
1553380_at	1.11e-09	2.168	PARP15	poly(ADP-ribose) polymerase family member 15
232539_at	1.16e-09	-2.957	SOCS2	suppressor of cytokine signaling 2
204993_at	1.17e-09	1.496	GNAZ	G protein subunit alpha z
218618_s_at	1.33e-09	1.806	FNDC3B	fibronectin type III domain containing 3B
214181_x_at	1.39e-09	-2.778	LST1	leukocyte specific transcript 1
215633_x_at	1.40e-09	-2.325	LST1	leukocyte specific transcript 1
221938_x_at	1.47e-09	-0.992	MED16	mediator complex subunit 16
213854_at	1.48e-09	-1.483	SYNGR1	synaptogyrin 1
241456_at	1.50e-09	1.177	FAM78B	family with sequence similarity 78 member B
227842_at	1.57e-09	0.828	RAB30	RAB30, member RAS oncogene family
219752_at	1.64e-09	2.298	RASAL1	RAS protein activator like 1
219489_s_at	1.72e-09	3.456	NXN	nucleoredoxin
239691_at	1.76e-09	2.468	C12orf77	chromosome 12 open reading frame 77
212288_at	1.78e-09	-2.688	FNBP1	formin binding protein 1
214574_x_at	1.92e-09	-2.472	LST1	leukocyte specific transcript 1
204015_s_at	2.05e-09	1.711	DUSP4	dual specificity phosphatase 4
228390_at	2.19e-09	2.497	RAB30	RAB30, member RAS oncogene family
213424_at	2.31e-09	0.331	KIAA0895	KIAA0895
1557740_a_at	2.35e-09	0.974		

225618_at	2.36e-09	-1.373	ARHGAP27	Rho GTPase activating protein 27
229233_at	2.39e-09	2.352	NRG3	neuregulin 3
219330_at	2.44e-09	1.042	VANGL1	VANGL planar cell polarity protein 1
211581_x_at	2.46e-09	-2.559	LST1	leukocyte specific transcript 1
226034_at	2.60e-09	2.224	DUSP4	dual specificity phosphatase 4
202275_at	2.60e-09	-1.395	G6PD	glucose-6-phosphate dehydrogenase
230067_at	2.64e-09	1.63	FAM124A	family with sequence similarity 124 member A
228740_at	2.75e-09	1.723		
1552496_a_at	2.90e-09	1.685	COBL	cordon-bleu WH2 repeat protein
213256_at	3.04e-09	1.719	MARCH3	membrane associated ring-CH-type finger 3
229336_at	3.12e-09	0.808	ST3GAL2ST3	beta-galactoside alpha-2,3-sialyltransferase 2
220911_s_at	3.16e-09	1.498	NYNRIN	NYN domain and retroviral integrase containing
236104_at	3.17e-09	0.592	HNRNPLL	heterogeneous nuclear ribonucleoprotein L like
234614_at	3.18e-09	0.996	FLI1	Fli-1 proto-oncogene, ETS transcription factor
235831_at	3.39e-09	0.621		
213005_s_at	3.51e-09	4.283	KANK1	KN motif and ankyrin repeat domains 1
204774_at	3.74e-09	-1.945	EVI2A	ecotropic viral integration site 2A
202741_at	3.77e-09	-2.845	PRKACB	protein kinase cAMP-activated catalytic subunit beta
218459_at	3.88e-09	1.374	TOR3A	torsin family 3 member A
226828_s_at	4.26e-09	0.632	HEYL	hes related family bHLH transcription factor with YRPW motif-like
201028_s_at	4.29e-09	-2.654	CD99	CD99 molecule
229492_at	4.38e-09	0.942	VANGL1	VANGL planar cell polarity protein 1
219271_at	4.41e-09	1.827	GALNT14	polypeptide N-acetylgalactosaminyltransferase 14
219279_at	4.74e-09	1.878	DOCK10	dedicator of cytokinesis 10
235287_at	4.76e-09	-1.676	CDK6	cyclin dependent kinase 6
219683_at	4.90e-09	1.009	FZD3	frizzled class receptor 3
214978_s_at	5.05e-09	1.445	PPFIA4	PTPRF interacting protein alpha 4
202421_at	5.06e-09	1.486	IGSF3	immunoglobulin superfamily member 3
230488_s_at	5.25e-09	0.857	DBH-AS1	DBH antisense RNA 1
223162_s_at	5.38e-09	-1.034	KIAA1147	KIAA1147
238649_at	5.74e-09	1.946	PITPNC1	phosphatidylinositol transfer protein, cytoplasmic 1
230509_at	5.81e-09	1.142	SNX22	sorting nexin 22
207996_s_at	5.83e-09	2.335	LDLRAD4	low density lipoprotein receptor class A domain containing 4
204529_s_at	5.87e-09	-2.455	TOX	thymocyte selection associated high mobility group box
211621_at	5.96e-09	0.753	AR	androgen receptor
224861_at	6.10e-09	-1.888	GNAQ	G protein subunit alpha q
227353_at	6.13e-09	-1.844	TMC8	transmembrane channel like 8
235952_at	6.34e-09	1.291	DGKH	diacylglycerol kinase eta

Table S3: RNA-seq data of RCH-ACV treated for NKX6-3 knockdown			
gene	RCH-ACV_siNKX6-3	RCH-ACV_siCTR	log2 Fold Chain
KLHL15	0,00	10,25	-10,25
PEDS1-UBE2V1	0,00	8,41	-8,41
BX470111.1	0,00	7,45	-7,45
AL359922.1	0,00	7,35	-7,35
GP5	0,00	7,23	-7,23
AMIGO3	0,00	7,13	-7,13
ANO5	0,00	6,92	-6,92
CHPF2	0,00	6,65	-6,65
AC008758.1	0,00	6,58	-6,58
AC135178.3	0,00	6,40	-6,40
AL022318.4	0,00	6,40	-6,40
MPP7	0,00	6,32	-6,32
FP565260.2	0,00	6,29	-6,29
PDCD6-AHRR	0,00	6,27	-6,27
CHURC1-FNTB	0,00	6,13	-6,13
AC009403.2	0,00	6,09	-6,09
SEPTIN1	0,00	6,07	-6,07
AC002310.4	0,00	6,05	-6,05
AL035461.3	0,00	6,03	-6,03
B4GALT6	0,00	5,97	-5,97
AMT	0,00	5,37	-5,37
AL136295.1	0,00	5,27	-5,27
AC048338.2	0,00	5,24	-5,24
GCNA	0,00	5,09	-5,09
ITGA7	0,00	5,05	-5,05
AC020909.1	0,00	5,01	-5,01
LRRC66	0,00	4,92	-4,92
ARHGAP8	0,00	4,92	-4,92
GET1-SH3BGR	0,00	4,92	-4,92
KLHL35	0,00	4,88	-4,88
MST1	0,00	4,79	-4,79
RGPD6	0,00	4,79	-4,79
PNMA3	0,00	4,79	-4,79
SEZ6L	0,00	4,53	-4,53
AC010197.2	0,00	4,53	-4,53
PKD1L1	0,00	4,53	-4,53
ZNF208	0,39	4,89	-4,50
AL049697.1	0,00	4,47	-4,47
PRDM6	0,87	5,30	-4,43
DNAH12	0,00	4,41	-4,41
AC091057.5	0,00	4,27	-4,27
AHR	0,00	4,13	-4,13
TRIM67	0,00	4,13	-4,13
FAM135B	0,00	4,13	-4,13
AC011462.1	0,00	4,13	-4,13
SASH1	1,35	5,41	-4,06
FNDC9	0,00	4,05	-4,05
KIAA0408	0,00	4,05	-4,05
CAMK2A	0,00	4,05	-4,05
HTR1F	0,00	3,97	-3,97
TNNI3K	0,00	3,97	-3,97
AC104532.1	0,00	3,97	-3,97
CHGB	0,00	3,88	-3,88

**PKLR**	0,00	3,88	-3,88
**RSPH10B**	0,00	3,88	-3,88
**AC068946.1**	0,00	3,88	-3,88
**TMEM249**	1,67	5,52	-3,85
**AFAP1**	5,55	9,40	-3,85
**SLC3A1**	0,76	4,60	-3,84
**SYN2**	1,05	4,86	-3,82
**GDPGP1**	0,00	3,79	-3,79
**PTPN5**	0,00	3,79	-3,79
**ADIRF**	0,00	3,79	-3,79
**F2RL2**	0,00	3,79	-3,79
**SLC38A3**	0,00	3,79	-3,79
**FCGR2B**	0,00	3,79	-3,79
**CACNA1S**	0,00	3,79	-3,79
**SLC45A2**	0,00	3,79	-3,79
**SPOCK3**	0,00	3,79	-3,79
**CNTN5**	0,00	3,69	-3,69
**NR0B1**	0,00	3,69	-3,69
**AP000350.4**	0,00	3,69	-3,69
**GOLGA8B**	5,32	8,91	-3,59
**TJP1**	0,00	3,59	-3,59
**DNAJC12**	0,00	3,59	-3,59
**ULBP2**	0,00	3,59	-3,59
**CHST4**	0,00	3,59	-3,59
**GLB1L3**	0,00	3,59	-3,59
**VCX3A**	0,00	3,59	-3,59
**INPP4B**	0,00	3,59	-3,59
**MIP**	0,00	3,59	-3,59
**MTNR1A**	0,00	3,59	-3,59
**GPC5**	0,00	3,59	-3,59
**PLEKHN1**	0,00	3,59	-3,59
**CBY3**	0,00	3,59	-3,59
**NXF2**	0,00	3,59	-3,59
**URGCP-MRPS24**	0,00	3,59	-3,59
**GREB1L**	0,00	3,47	-3,47
**HABP2**	0,00	3,47	-3,47
**NPR2**	0,00	3,47	-3,47
**RNF39**	0,00	3,47	-3,47
**EEF1E1-BLOC1S5**	0,00	3,47	-3,47
**ATP2C2**	0,00	3,47	-3,47
**ZNF407**	0,00	3,47	-3,47
**FOXL1**	1,89	5,35	-3,46
**SULT1A4**	6,50	9,89	-3,39
**BAIAP2**	1,10	4,45	-3,35
**SOX6**	0,00	3,35	-3,35
**SYPL2**	0,00	3,35	-3,35
**ITGAD**	0,00	3,35	-3,35
**CHST8**	0,00	3,35	-3,35
**RPL3L**	0,00	3,35	-3,35
**NXF3**	0,00	3,35	-3,35
**GAPT**	0,00	3,35	-3,35
**SYNE4**	0,00	3,35	-3,35
**MCIDAS**	0,00	3,35	-3,35
**AC007998.2**	0,00	3,35	-3,35
**FGF22**	0,00	3,35	-3,35
**CMA1**	0,00	3,35	-3,35

EFNB3	0,00	3,35	-3,35
PHF24	0,00	3,35	-3,35
HK3	0,00	3,35	-3,35
SLC26A11	0,00	3,35	-3,35
NWD1	0,00	3,35	-3,35
SBSN	0,00	3,35	-3,35
SP6	0,00	3,35	-3,35
TNFRSF9	1,37	4,72	-3,35
ANKRD29	1,38	4,70	-3,32
GATA3	1,30	4,59	-3,29
ASDURF	2,35	5,64	-3,29
ZNF233	1,34	4,62	-3,28
AP1M2	1,01	4,24	-3,23
TMEM181	0,00	3,21	-3,21
CPAMD8	0,00	3,21	-3,21
PRKN	0,00	3,21	-3,21
EYA2	0,00	3,21	-3,21
DRC1	0,00	3,21	-3,21
AC010463.1	0,00	3,21	-3,21
SYCP2L	0,00	3,21	-3,21
PRAP1	0,00	3,21	-3,21
POU3F2	0,00	3,21	-3,21
LILRB3	0,00	3,21	-3,21
TTLL13P	0,00	3,21	-3,21
CPEB1	0,00	3,21	-3,21
GFAP	0,00	3,21	-3,21
CASQ1	1,06	4,13	-3,07
CLNK	0,00	3,06	-3,06
ENOX1	0,00	3,06	-3,06
SAG	0,00	3,06	-3,06
GAD2	0,00	3,06	-3,06
CYP2C9	0,00	3,06	-3,06
GRB7	0,00	3,06	-3,06
ST18	0,00	3,06	-3,06
GPR183	0,00	3,06	-3,06
DAND5	0,00	3,06	-3,06
PRF1	0,00	3,06	-3,06
KREMEN1	0,00	3,06	-3,06
PEAK3	0,00	3,06	-3,06
VEPH1	0,00	3,06	-3,06
CYP3A7-CYP3A51P	0,00	3,06	-3,06
ZIC2	0,00	3,06	-3,06
APLP1	0,00	3,06	-3,06
PNCK	0,00	3,06	-3,06
TKTL2	0,00	3,06	-3,06
TEX29	0,00	3,06	-3,06
DMTN	0,00	3,06	-3,06
IQCF1	0,00	3,06	-3,06
KDF1	0,00	3,06	-3,06
GPR1	0,00	3,06	-3,06
MIXL1	0,00	3,06	-3,06
HBA2	0,00	3,06	-3,06
PCDHB11	0,00	3,06	-3,06
GTF2A1L	0,00	3,06	-3,06
AC008750.7	0,00	3,06	-3,06
AC005258.1	0,00	3,06	-3,06

**AL160272.2**	0,00	3,06	-3,06
**ADGRG2**	0,00	3,06	-3,06
**CCDC38**	1,73	4,75	-3,03
**GPR61**	1,67	4,64	-2,97
**FGF7**	2,60	5,54	-2,94
**NUAK1**	2,96	5,87	-2,91
**TMEM59L**	1,42	4,32	-2,90
**FAM149A**	0,00	2,89	-2,89
**CTXN2**	0,00	2,89	-2,89
**H2AB2**	0,00	2,89	-2,89
**SFRP5**	0,00	2,89	-2,89
**CHST5**	0,00	2,89	-2,89
**ABHD1**	0,00	2,89	-2,89
**FTCD**	0,00	2,89	-2,89
**TRPV6**	0,00	2,89	-2,89
**C3orf80**	0,00	2,89	-2,89
**AC012309.1**	0,00	2,89	-2,89
**TMEM255A**	0,00	2,89	-2,89
**ANKRD20A2P**	3,39	6,25	-2,86
**AC084337.2**	1,68	4,51	-2,84
**FUT3**	1,68	4,51	-2,82
**PCLO**	4,46	7,28	-2,82
**KIF25**	2,69	5,46	-2,78
**WHRN**	2,82	5,56	-2,74
**ENDOU**	1,06	3,79	-2,73
**OVOL1**	1,06	3,79	-2,73
**PLET1**	1,06	3,79	-2,73
**CACNA1C**	1,06	3,79	-2,73
**FO393400.1**	2,11	4,82	-2,71
**TENM1**	0,00	2,70	-2,70
**ANOS1**	0,00	2,70	-2,70
**TFAP2C**	0,00	2,70	-2,70
**FXYD3**	0,00	2,70	-2,70
**CCDC80**	0,00	2,70	-2,70
**OLFM2**	0,00	2,70	-2,70
**NKX3-2**	0,00	2,70	-2,70
**COLEC11**	0,00	2,70	-2,70
**TMPRSS13**	0,00	2,70	-2,70
**MARVELD3**	0,00	2,70	-2,70
**ZNF157**	0,00	2,70	-2,70
**M1AP**	0,00	2,70	-2,70
**PTCHD1**	0,00	2,70	-2,70
**ACTBL2**	0,00	2,70	-2,70
**FRMPD4**	0,00	2,70	-2,70
**ADAMTSL1**	0,00	2,70	-2,70
**TNFRSF18**	0,00	2,70	-2,70
**MYH6**	0,00	2,70	-2,70
**POU5F1B**	0,00	2,70	-2,70
**PCDHGB4**	0,00	2,70	-2,70
**TBC1D3K**	0,00	2,70	-2,70
**POTEB3**	0,00	2,70	-2,70
**FAM90A23P**	0,00	2,70	-2,70
**AC009879.3**	0,00	2,70	-2,70
**HSD17B2**	0,00	2,70	-2,70
**F11**	0,00	2,70	-2,70
**AQP8**	0,00	2,70	-2,70

**SYDE1**	0,00	2,70	-2,70
**MEIS3**	0,00	2,70	-2,70
**LILRB5**	0,00	2,70	-2,70
**IL9R**	0,00	2,70	-2,70
**MCF2L**	0,00	2,70	-2,70
**RNF112**	0,00	2,70	-2,70
**APOC1**	0,00	2,70	-2,70
**SLC41A2**	0,00	2,70	-2,70
**TGM7**	0,00	2,70	-2,70
**IP6K3**	0,00	2,70	-2,70
**DNAJC5G**	0,00	2,70	-2,70
**CMTM5**	0,00	2,70	-2,70
**CCDC103**	0,00	2,70	-2,70
**JSRP1**	0,00	2,70	-2,70
**ABLIM3**	0,00	2,70	-2,70
**SLC35D3**	0,00	2,70	-2,70
**C19orf67**	0,00	2,70	-2,70
**DOK6**	0,00	2,70	-2,70
**IGHV3-13**	0,00	2,70	-2,70
**UBE2QL1**	0,00	2,70	-2,70
**ZBED9**	0,00	2,70	-2,70
**AL136295.3**	0,00	2,70	-2,70
**AC140504.1**	0,00	2,70	-2,70
**AC091167.6**	0,00	2,70	-2,70
**GPR179**	0,00	2,70	-2,70
**TRBV7-2**	0,00	2,70	-2,70
**ATP1A4**	0,00	2,70	-2,70
**MPV17L**	0,00	2,70	-2,70
**STEAP2**	0,00	2,70	-2,70
**NPAS4**	0,00	2,70	-2,70
**GRM8**	0,00	2,70	-2,70
**PLAG1**	0,00	2,70	-2,70
**OR2V2**	0,00	2,70	-2,70
**GLUD2**	0,00	2,70	-2,70
**GRIN2A**	0,00	2,70	-2,70
**NHS**	0,00	2,70	-2,70
**HBE1**	0,00	2,70	-2,70
**IGKV1-8**	0,00	2,70	-2,70
**PI15**	1,58	4,26	-2,68
**ZNF609**	1,67	4,34	-2,67
**TRIM74**	2,71	5,38	-2,67
**SYT7**	3,26	5,90	-2,65
**SMIM2**	2,99	5,64	-2,64
**EML5**	5,87	8,51	-2,64
**FLVCR2**	3,19	5,83	-2,63
**JPH4**	1,06	3,69	-2,63
**IGHV4-39**	1,06	3,69	-2,63
**VWDE**	1,74	4,36	-2,62
**CXCR2**	4,41	7,02	-2,61
**SLCO2B1**	1,40	3,96	-2,56
**INSYN1**	0,38	2,93	-2,55
**DEPDC4**	2,39	4,94	-2,55
**DYNLT2**	2,09	4,64	-2,54
**SYT16**	1,67	4,20	-2,54
**GLYATL1B**	1,06	3,59	-2,52
**LAMB3**	1,32	3,84	-2,52

IGKV2-24	2,42	4,92	-2,50
MYO1A	2,09	4,58	-2,49
ABCB11	0,00	2,48	-2,48
NME8	0,00	2,48	-2,48
TGFBI	0,00	2,48	-2,48
COL21A1	0,00	2,48	-2,48
ANKRD1	0,00	2,48	-2,48
CES5A	0,00	2,48	-2,48
SIGLEC7	0,00	2,48	-2,48
CATSPERD	0,00	2,48	-2,48
TEX38	0,00	2,48	-2,48
IFNA14	0,00	2,48	-2,48
KRTAP5-8	0,00	2,48	-2,48
AC003002.3	0,00	2,48	-2,48
CCDC194	0,00	2,48	-2,48
SLC12A1	0,00	2,48	-2,48
CYP39A1	0,00	2,48	-2,48
TSPAN5	0,00	2,48	-2,48
WFIKKN2	0,00	2,48	-2,48
IGKV1-39	0,00	2,48	-2,48
MXRA7	8,18	10,66	-2,48
CA7	1,67	4,13	-2,46
PLEKHD1	1,67	4,13	-2,46
GLTPD2	1,67	4,13	-2,46
ZNF667	2,28	4,74	-2,45
AKAP14	0,79	3,21	-2,43
AC138811.2	7,52	9,95	-2,42
C1QTNF12	1,06	3,47	-2,41
TMCC1	6,45	8,85	-2,40
U2AF1	7,98	10,38	-2,40
H3C3	2,91	5,31	-2,39
GPR17	3,11	5,49	-2,38
IL23A	2,09	4,47	-2,37
AL355916.3	2,09	4,47	-2,37
MID2	2,96	5,32	-2,37
ADAMTS12	3,36	5,72	-2,36
GPX2	1,65	4,00	-2,35
GPR157	7,53	9,85	-2,32
S100A2	0,66	2,98	-2,32
MMP23B	2,87	5,18	-2,30
PPARGC1A	1,41	3,71	-2,30
ISM2	1,67	3,97	-2,30
PRDM16	1,67	3,97	-2,30
LRRN3	1,06	3,35	-2,28
C6orf132	3,57	5,86	-2,28
CTSW	1,68	3,96	-2,28
ABCA10	3,00	5,27	-2,28
SPATA17	0,96	3,23	-2,27
PLA2G4C	4,44	6,71	-2,27
ADAP2	4,12	6,38	-2,26
NKX6-3	2,91	5,16	-2,25
OR2AT4	1,77	4,00	-2,23
TMEM151B	3,00	5,23	-2,23
SCIN	0,00	2,22	-2,22
SOBP	0,00	2,22	-2,22
OTOF	0,00	2,22	-2,22

**MLPH**	0,00	2,22	-2,22
**CFAP74**	0,00	2,22	-2,22
**STC1**	0,00	2,22	-2,22
**SLC6A17**	0,00	2,22	-2,22
**ARX**	0,00	2,22	-2,22
**SOX30**	0,00	2,22	-2,22
**LAMC2**	0,00	2,22	-2,22
**FGF4**	0,00	2,22	-2,22
**HOXA9**	0,00	2,22	-2,22
**UPB1**	0,00	2,22	-2,22
**PDYN**	0,00	2,22	-2,22
**MYL9**	0,00	2,22	-2,22
**ACP5**	0,00	2,22	-2,22
**SFRP1**	0,00	2,22	-2,22
**DHDH**	0,00	2,22	-2,22
**ACTR3C**	0,00	2,22	-2,22
**CRTAM**	0,00	2,22	-2,22
**ACADL**	0,00	2,22	-2,22
**INSL6**	0,00	2,22	-2,22
**TBX4**	0,00	2,22	-2,22
**SPINK4**	0,00	2,22	-2,22
**BHLHE41**	0,00	2,22	-2,22
**GRIA3**	0,00	2,22	-2,22
**PRKCG**	0,00	2,22	-2,22
**CRB3**	0,00	2,22	-2,22
**MATN3**	0,00	2,22	-2,22
**KANK4**	0,00	2,22	-2,22
**GPR12**	0,00	2,22	-2,22
**SAA2**	0,00	2,22	-2,22
**BCO1**	0,00	2,22	-2,22
**LRRC49**	0,00	2,22	-2,22
**CDK15**	0,00	2,22	-2,22
**AC008764.1**	0,00	2,22	-2,22
**CACNG8**	0,00	2,22	-2,22
**PADI1**	0,00	2,22	-2,22
**DMRTA2**	0,00	2,22	-2,22
**CNTN4**	0,00	2,22	-2,22
**AWAT2**	0,00	2,22	-2,22
**SLC39A12**	0,00	2,22	-2,22
**MPZL2**	0,00	2,22	-2,22
**CCNO**	0,00	2,22	-2,22
**SHCBP1L**	0,00	2,22	-2,22
**FCRL3**	0,00	2,22	-2,22
**NFIA**	0,00	2,22	-2,22
**FAM71A**	0,00	2,22	-2,22
**GABRG1**	0,00	2,22	-2,22
**RBM47**	0,00	2,22	-2,22
**C4orf45**	0,00	2,22	-2,22
**SLC6A18**	0,00	2,22	-2,22
**AQP7**	0,00	2,22	-2,22
**SSX3**	0,00	2,22	-2,22
**TC2N**	0,00	2,22	-2,22
**SCNN1G**	0,00	2,22	-2,22
**ART5**	0,00	2,22	-2,22
**ACER1**	0,00	2,22	-2,22
**CYP7A1**	0,00	2,22	-2,22

**GSDMA**	0,00	2,22	-2,22
**BATF2**	0,00	2,22	-2,22
**ADAM29**	0,00	2,22	-2,22
**NPNT**	0,00	2,22	-2,22
**RAB40A**	0,00	2,22	-2,22
**FAM166C**	0,00	2,22	-2,22
**PRR18**	0,00	2,22	-2,22
**CAPS2**	0,00	2,22	-2,22
**UNC5C**	0,00	2,22	-2,22
**SLIT3**	0,00	2,22	-2,22
**PLK5**	0,00	2,22	-2,22
**POTEG**	0,00	2,22	-2,22
**CYP26C1**	0,00	2,22	-2,22
**NANOS3**	0,00	2,22	-2,22
**CLEC17A**	0,00	2,22	-2,22
**MORN2**	0,00	2,22	-2,22
**FZD9**	0,00	2,22	-2,22
**CATSPER4**	0,00	2,22	-2,22
**VHLL**	0,00	2,22	-2,22
**MAOA**	0,00	2,22	-2,22
**OR2T4**	0,00	2,22	-2,22
**SERPINA1**	0,00	2,22	-2,22
**LEKR1**	0,00	2,22	-2,22
**F5**	0,00	2,22	-2,22
**C1orf53**	0,00	2,22	-2,22
**OR6K3**	0,00	2,22	-2,22
**ERICH4**	0,00	2,22	-2,22
**RNASE13**	0,00	2,22	-2,22
**IGLV1-47**	0,00	2,22	-2,22
**CERS1**	0,00	2,22	-2,22
**PATE3**	0,00	2,22	-2,22
**TRIM39-RPP21**	0,00	2,22	-2,22
**RFPL4B**	0,00	2,22	-2,22
**MTRNR2L3**	0,00	2,22	-2,22
**KHDC1L**	0,00	2,22	-2,22
**CCL4L2**	0,00	2,22	-2,22
**OR51B2**	0,00	2,22	-2,22
**LMLN2**	0,00	2,22	-2,22
**OOSP3**	0,00	2,22	-2,22
**AL031847.1**	0,00	2,22	-2,22
**IL5RA**	0,00	2,22	-2,22
**RAB6C**	1,67	3,88	-2,22
**AXDND1**	1,14	3,34	-2,21
**SMIM6**	1,78	3,98	-2,20
**CFD**	2,09	4,27	-2,18
**DUSP15**	2,29	4,44	-2,15
**TMEM89**	1,06	3,21	-2,15
**THSD4**	2,69	4,83	-2,15
**MYCBPAP**	2,92	5,05	-2,13
**TNNC2**	1,67	3,79	-2,12
**C11orf52**	1,67	3,79	-2,12
**DRC3**	4,17	6,29	-2,11
**CHRNE**	3,41	5,52	-2,11
**GLI2**	1,88	3,99	-2,10
**CAPN12**	1,18	3,27	-2,09
**AC093512.2**	4,92	7,00	-2,08

PRSS23	2,33	4,39	-2,06
SOX10	2,68	4,73	-2,05
ABCC8	2,62	4,66	-2,05
C1QTNF3-AMACR	5,33	7,38	-2,05
GUCY1A2	2,42	4,47	-2,04
AC005726.1	4,32	6,36	-2,04
RCCD1	6,59	8,63	-2,04
GPR37	2,09	4,13	-2,04
PLAC9	2,09	4,13	-2,04
EPGN	2,63	4,66	-2,03
GIMAP1	4,20	6,22	-2,01
TMEM108	1,41	3,41	-2,00
MYOF	1,72	3,72	-2,00
CCDC173	1,06	3,06	-2,00
BIRC7	1,68	3,67	-1,99
H2BC4	2,42	4,41	-1,98
ADGRV1	3,31	5,29	-1,97
RORC	3,93	5,90	-1,97
ATP6V0E2	4,38	6,35	-1,97
AKAP6	5,63	7,59	-1,96
TBC1D28	1,55	3,50	-1,95
TPTEP2-CSNK1E	2,69	4,64	-1,95
UPP1	1,73	3,67	-1,94
GRIN3B	2,91	4,83	-1,92
PANX2	1,67	3,59	-1,92
DDC	1,67	3,59	-1,92
B3GNT7	1,67	3,59	-1,92
CABP4	1,67	3,59	-1,92
AC011479.1	1,67	3,59	-1,92
NEK11	3,56	5,47	-1,91
CD6	0,00	1,91	-1,91
SLC26A3	0,00	1,91	-1,91
RAB36	0,00	1,91	-1,91
SLC1A6	0,00	1,91	-1,91
GLS2	0,00	1,91	-1,91
GPNMB	0,00	1,91	-1,91
GBX2	0,00	1,91	-1,91
RNF212	0,00	1,91	-1,91
ODF4	0,00	1,91	-1,91
NLRP9	0,00	1,91	-1,91
GPAT2	0,00	1,91	-1,91
EYS	0,00	1,91	-1,91
EIF1AY	0,00	1,91	-1,91
AL512785.2	0,00	1,91	-1,91
CFAP97D2	0,00	1,91	-1,91
FAM90A8P	0,00	1,91	-1,91
YBX2	0,00	1,91	-1,91
CRTAC1	0,00	1,91	-1,91
GATA1	0,00	1,91	-1,91
RTBDN	0,00	1,91	-1,91
ADAM20	0,00	1,91	-1,91
PLA2G12B	0,00	1,91	-1,91
STAC2	0,00	1,91	-1,91
BHMT	0,00	1,91	-1,91
C16orf89	0,00	1,91	-1,91
CLEC3B	0,00	1,91	-1,91

**UCMA**	0,00	1,91	-1,91
**LPO**	0,00	1,91	-1,91
**TAS2R1**	0,00	1,91	-1,91
**EZHIP**	0,00	1,91	-1,91
**P2RX2**	0,00	1,91	-1,91
**CERKL**	0,00	1,91	-1,91
**TOMM20L**	0,00	1,91	-1,91
**IQANK1**	0,00	1,91	-1,91
**CFAP99**	0,00	1,91	-1,91
**IGLC7**	0,00	1,91	-1,91
**CSH2**	0,00	1,91	-1,91
**ZNF99**	0,00	1,91	-1,91
**AC008770.1**	0,00	1,91	-1,91
**AC004076.1**	0,00	1,91	-1,91
**AL160269.1**	0,00	1,91	-1,91
**AC097634.4**	0,00	1,91	-1,91
**ZBED6CL**	3,92	5,81	-1,89
**CFAP61**	1,32	3,18	-1,86
**FOXE3**	2,42	4,27	-1,85
**GPR84**	2,25	4,09	-1,84
**RNF224**	2,69	4,53	-1,84
**ISLR2**	1,71	3,54	-1,83
**SRA1**	1,06	2,89	-1,83
**AC138696.1**	4,12	5,94	-1,83
**MEP1B**	1,06	2,89	-1,83
**ASAH2**	3,35	5,16	-1,82
**FRMPD2**	1,67	3,47	-1,80
**POMGNT2**	8,12	9,93	-1,80
**ALKAL2**	3,26	5,07	-1,80
**CAPN11**	3,70	5,49	-1,79
**SLC6A16**	1,14	2,93	-1,79
**HSPA12A**	3,20	4,99	-1,79
**PCDHGA7**	2,62	4,41	-1,79
**TMOD1**	2,42	4,20	-1,78
**FNDC11**	1,15	2,92	-1,78
**SERPINE3**	2,28	4,05	-1,77
**APOBR**	3,28	5,05	-1,77
**FA2H**	2,91	4,68	-1,77
**PPP1R26**	3,24	5,01	-1,76
**SERPIND1**	1,15	2,91	-1,76
**BDNF**	2,15	3,90	-1,76
**C6orf52**	3,62	5,36	-1,74
**AL353671.1**	6,39	8,13	-1,74
**RAD51B**	5,88	7,61	-1,73
**AL355312.5**	3,43	5,16	-1,73
**OLFML2B**	2,86	4,59	-1,73
**FOXD2**	2,91	4,64	-1,72
**ZNF705A**	1,17	2,89	-1,72
**AMPH**	2,42	4,13	-1,71
**KCNE5**	2,42	4,13	-1,71
**LRRC75B**	2,64	4,33	-1,70
**DCAKD**	6,84	8,53	-1,69
**BAAT**	3,85	5,54	-1,69
**FAM98A**	9,86	11,54	-1,68
**ZNF112**	1,67	3,35	-1,68
**ZBTB32**	1,67	3,35	-1,68

**SNRPF**	1,67	3,35	-1,68
**SPARCL1**	1,67	3,35	-1,68
**LHFPL4**	1,67	3,35	-1,68
**AIRE**	1,67	3,35	-1,68
**TNFRSF10C**	1,67	3,35	-1,68
**AC008687.4**	1,67	3,35	-1,68
**USHBP1**	3,22	4,90	-1,68
**C16orf86**	5,33	7,01	-1,68
**AC127029.3**	2,91	4,58	-1,67
**C13orf46**	2,91	4,58	-1,67
**DPF1**	6,57	8,22	-1,65
**FITM1**	4,33	5,98	-1,65
**LCN12**	1,56	3,21	-1,65
**AL353588.1**	4,12	5,76	-1,64
**KCNMB4**	5,00	6,64	-1,64
**CRYBG2**	2,42	4,05	-1,63
**HMX3**	2,42	4,05	-1,63
**CALCRL**	4,53	6,16	-1,63
**NRL**	5,85	7,48	-1,63
**ZBTB7B**	7,68	9,30	-1,62
**SLC22A11**	2,30	3,93	-1,62
**ADPRH**	2,56	4,18	-1,62
**RAP1GAP**	4,81	6,43	-1,62
**AC004706.3**	5,58	7,20	-1,62
**MXD3**	8,07	9,69	-1,62
**PLA2G4B**	7,07	8,68	-1,61
**ALPG**	2,91	4,53	-1,61
**TNFSF12**	2,91	4,53	-1,61
**EIF2S3B**	3,70	5,31	-1,61
**GAS2**	3,15	4,75	-1,59
**ROM1**	5,34	6,93	-1,59
**FOSB**	4,13	5,71	-1,59
**WNT9B**	2,37	3,95	-1,58
**RNF123**	10,40	11,97	-1,57
**HAPLN2**	1,39	2,96	-1,57
**CRIP3**	3,36	4,93	-1,57
**KIRREL2**	4,41	5,97	-1,57
**INO80B-WBP1**	5,71	7,27	-1,56
**RASGRF1**	3,56	5,12	-1,56
**CAMK1**	4,30	5,85	-1,55
**AC104389.5**	2,91	4,47	-1,55
**CTTN**	2,42	3,97	-1,55
**MSI1**	2,42	3,97	-1,55
**INSYN2B**	2,42	3,97	-1,55
**CCND2**	1,67	3,21	-1,54
**KCNJ5**	1,67	3,21	-1,54
**MSGN1**	1,67	3,21	-1,54
**C1orf189**	1,67	3,21	-1,54
**FAM131C**	1,67	3,21	-1,54
**RBP3**	1,67	3,21	-1,54
**AL451007.3**	1,67	3,21	-1,54
**ACKR3**	4,43	5,97	-1,54
**IL6ST**	10,37	11,90	-1,53
**ATG101**	4,74	6,26	-1,52
**CELF3**	4,45	5,97	-1,51
**C3orf14**	2,26	3,77	-1,51

**LGSN**	1,46	2,97	-1,51
**FKBP2**	8,56	10,07	-1,50
**ATP4A**	0,00	1,50	-1,50
**NMNAT2**	0,00	1,50	-1,50
**NRG1**	0,00	1,50	-1,50
**LONRF2**	0,00	1,50	-1,50
**GXYLT2**	0,00	1,50	-1,50
**OTC**	0,00	1,50	-1,50
**STYK1**	0,00	1,50	-1,50
**COL17A1**	0,00	1,50	-1,50
**GAL**	0,00	1,50	-1,50
**MGLL**	0,00	1,50	-1,50
**SIGLEC1**	0,00	1,50	-1,50
**P2RX6**	0,00	1,50	-1,50
**UPK3A**	0,00	1,50	-1,50
**MLNR**	0,00	1,50	-1,50
**FOXF1**	0,00	1,50	-1,50
**SIGLEC8**	0,00	1,50	-1,50
**IL4**	0,00	1,50	-1,50
**SERPINI2**	0,00	1,50	-1,50
**SLC30A3**	0,00	1,50	-1,50
**HAO2**	0,00	1,50	-1,50
**TP53AIP1**	0,00	1,50	-1,50
**TNFSF11**	0,00	1,50	-1,50
**TMIGD3**	0,00	1,50	-1,50
**INHBA**	0,00	1,50	-1,50
**SSPN**	0,00	1,50	-1,50
**DNAH8**	0,00	1,50	-1,50
**ZNF835**	0,00	1,50	-1,50
**FOXJ1**	0,00	1,50	-1,50
**RHOXF2**	0,00	1,50	-1,50
**SDS**	0,00	1,50	-1,50
**ENPEP**	0,00	1,50	-1,50
**PNMT**	0,00	1,50	-1,50
**FAM71E1**	0,00	1,50	-1,50
**CPLX2**	0,00	1,50	-1,50
**GLYAT**	0,00	1,50	-1,50
**DRD2**	0,00	1,50	-1,50
**C20orf144**	0,00	1,50	-1,50
**SPATA4**	0,00	1,50	-1,50
**POU4F2**	0,00	1,50	-1,50
**DDX4**	0,00	1,50	-1,50
**LCN1**	0,00	1,50	-1,50
**NEURL3**	0,00	1,50	-1,50
**NUAK2**	0,00	1,50	-1,50
**RHO**	0,00	1,50	-1,50
**HEY1**	0,00	1,50	-1,50
**GFI1B**	0,00	1,50	-1,50
**MS4A8**	0,00	1,50	-1,50
**INSM2**	0,00	1,50	-1,50
**KCTD19**	0,00	1,50	-1,50
**FABP6**	0,00	1,50	-1,50
**HSPB2**	0,00	1,50	-1,50
**LGALS9B**	0,00	1,50	-1,50
**CYP4F22**	0,00	1,50	-1,50
**TAS1R1**	0,00	1,50	-1,50

**SEZ6L2**	0,00	1,50	-1,50
**BEND2**	0,00	1,50	-1,50
**C12orf54**	0,00	1,50	-1,50
**RNF186**	0,00	1,50	-1,50
**PNPLA1**	0,00	1,50	-1,50
**TMEM252**	0,00	1,50	-1,50
**LIPF**	0,00	1,50	-1,50
**RIPK4**	0,00	1,50	-1,50
**H3C13**	0,00	1,50	-1,50
**MACC1**	0,00	1,50	-1,50
**A3GALT2**	0,00	1,50	-1,50
**RALYL**	0,00	1,50	-1,50
**DMRTC1B**	0,00	1,50	-1,50
**CLCNKA**	0,00	1,50	-1,50
**GPR141**	0,00	1,50	-1,50
**TMEM72**	0,00	1,50	-1,50
**DMBT1**	0,00	1,50	-1,50
**PRSS45P**	0,00	1,50	-1,50
**FBLL1**	0,00	1,50	-1,50
**SFTA2**	0,00	1,50	-1,50
**CYP2A7**	0,00	1,50	-1,50
**ARHGEF15**	0,00	1,50	-1,50
**CFI**	0,00	1,50	-1,50
**ACOT6**	0,00	1,50	-1,50
**KRTAP5-2**	0,00	1,50	-1,50
**TRAV14DV4**	0,00	1,50	-1,50
**TSGA13**	0,00	1,50	-1,50
**CPLX3**	0,00	1,50	-1,50
**EFCAB9**	0,00	1,50	-1,50
**SPINK13**	0,00	1,50	-1,50
**OR52I2**	0,00	1,50	-1,50
**IRGM**	0,00	1,50	-1,50
**IGKV1D-43**	0,00	1,50	-1,50
**MARCOL**	0,00	1,50	-1,50
**ECSCR**	0,00	1,50	-1,50
**AP000721.1**	0,00	1,50	-1,50
**TRDV3**	0,00	1,50	-1,50
**AC010542.3**	0,00	1,50	-1,50
**SMIM32**	0,00	1,50	-1,50
**C8orf89**	0,00	1,50	-1,50
**GIMAP1-GIMAP5**	0,00	1,50	-1,50
**TMEM265**	0,00	1,50	-1,50
**AC013271.1**	0,00	1,50	-1,50
**AOC1**	0,00	1,50	-1,50
**PCDH11Y**	0,00	1,50	-1,50
**HRH3**	0,00	1,50	-1,50
**MSLN**	0,00	1,50	-1,50
**TFPI2**	0,00	1,50	-1,50
**CCL24**	0,00	1,50	-1,50
**SFRP4**	0,00	1,50	-1,50
**ELAVL2**	0,00	1,50	-1,50
**SLC6A12**	0,00	1,50	-1,50
**RERGL**	0,00	1,50	-1,50
**BTNL8**	0,00	1,50	-1,50
**IRX4**	0,00	1,50	-1,50
**UNC5A**	0,00	1,50	-1,50

**BCHE**	0,00	1,50	-1,50
**HES1**	0,00	1,50	-1,50
**KCNIP3**	0,00	1,50	-1,50
**GRB14**	0,00	1,50	-1,50
**TACR1**	0,00	1,50	-1,50
**RPE65**	0,00	1,50	-1,50
**NR5A2**	0,00	1,50	-1,50
**IGSF21**	0,00	1,50	-1,50
**CNNM1**	0,00	1,50	-1,50
**EQTN**	0,00	1,50	-1,50
**PDZRN3**	0,00	1,50	-1,50
**PAEP**	0,00	1,50	-1,50
**PLP1**	0,00	1,50	-1,50
**CTCFL**	0,00	1,50	-1,50
**C20orf173**	0,00	1,50	-1,50
**F10**	0,00	1,50	-1,50
**RFPL2**	0,00	1,50	-1,50
**TNNI2**	0,00	1,50	-1,50
**NR0B2**	0,00	1,50	-1,50
**LRRC9**	0,00	1,50	-1,50
**SFTPD**	0,00	1,50	-1,50
**KLRC1**	0,00	1,50	-1,50
**CTSL**	0,00	1,50	-1,50
**MRAP2**	0,00	1,50	-1,50
**IL10**	0,00	1,50	-1,50
**IL36A**	0,00	1,50	-1,50
**NXPE4**	0,00	1,50	-1,50
**CYP1B1**	0,00	1,50	-1,50
**BTBD16**	0,00	1,50	-1,50
**OIT3**	0,00	1,50	-1,50
**CDH13**	0,00	1,50	-1,50
**TRIM43B**	0,00	1,50	-1,50
**GALNT13**	0,00	1,50	-1,50
**CDH18**	0,00	1,50	-1,50
**PRSS35**	0,00	1,50	-1,50
**EPHA1**	0,00	1,50	-1,50
**CDKN2B**	0,00	1,50	-1,50
**HMCN2**	0,00	1,50	-1,50
**ZP1**	0,00	1,50	-1,50
**HMGA2**	0,00	1,50	-1,50
**CA10**	0,00	1,50	-1,50
**KCNS2**	0,00	1,50	-1,50
**CDCP2**	0,00	1,50	-1,50
**SHROOM4**	0,00	1,50	-1,50
**PAGE5**	0,00	1,50	-1,50
**THEM4**	0,00	1,50	-1,50
**FAM131B**	0,00	1,50	-1,50
**MEIOB**	0,00	1,50	-1,50
**PDZK1IP1**	0,00	1,50	-1,50
**BRINP3**	0,00	1,50	-1,50
**XIRP2**	0,00	1,50	-1,50
**EMCN**	0,00	1,50	-1,50
**FOXQ1**	0,00	1,50	-1,50
**CHMP4C**	0,00	1,50	-1,50
**FAT3**	0,00	1,50	-1,50
**SOHLH1**	0,00	1,50	-1,50

**PEX11A**	0,00	1,50	-1,50
**NYAP1**	0,00	1,50	-1,50
**CHRM1**	0,00	1,50	-1,50
**WFDC13**	0,00	1,50	-1,50
**BTNL3**	0,00	1,50	-1,50
**CXCL11**	0,00	1,50	-1,50
**SLC38A11**	0,00	1,50	-1,50
**VSTM2A**	0,00	1,50	-1,50
**GPR27**	0,00	1,50	-1,50
**FPR1**	0,00	1,50	-1,50
**ALK**	0,00	1,50	-1,50
**CSDC2**	0,00	1,50	-1,50
**CCR9**	0,00	1,50	-1,50
**SULT1B1**	0,00	1,50	-1,50
**PODN**	0,00	1,50	-1,50
**ENTHD1**	0,00	1,50	-1,50
**LRRTM4**	0,00	1,50	-1,50
**KCNA2**	0,00	1,50	-1,50
**CAVIN1**	0,00	1,50	-1,50
**C2orf73**	0,00	1,50	-1,50
**DYNAP**	0,00	1,50	-1,50
**C14orf39**	0,00	1,50	-1,50
**LBX2**	0,00	1,50	-1,50
**ZNF648**	0,00	1,50	-1,50
**CSNK1A1L**	0,00	1,50	-1,50
**TSPYL5**	0,00	1,50	-1,50
**BHLHE22**	0,00	1,50	-1,50
**OR56B2P**	0,00	1,50	-1,50
**MAB21L2**	0,00	1,50	-1,50
**GPR88**	0,00	1,50	-1,50
**KCNIP1**	0,00	1,50	-1,50
**GPR39**	0,00	1,50	-1,50
**TMPRSS2**	0,00	1,50	-1,50
**OR2V1**	0,00	1,50	-1,50
**TRDN**	0,00	1,50	-1,50
**LIN28B**	0,00	1,50	-1,50
**LRRC74B**	0,00	1,50	-1,50
**POTEE**	0,00	1,50	-1,50
**SLC24A5**	0,00	1,50	-1,50
**GJB5**	0,00	1,50	-1,50
**FOXR2**	0,00	1,50	-1,50
**BNIP5**	0,00	1,50	-1,50
**SERPINA3**	0,00	1,50	-1,50
**NEK5**	0,00	1,50	-1,50
**ZSCAN5B**	0,00	1,50	-1,50
**FCGR1B**	0,00	1,50	-1,50
**CCT8L2**	0,00	1,50	-1,50
**BRINP2**	0,00	1,50	-1,50
**LRRC73**	0,00	1,50	-1,50
**COL15A1**	0,00	1,50	-1,50
**LY6G6C**	0,00	1,50	-1,50
**OR2H1**	0,00	1,50	-1,50
**SPINK9**	0,00	1,50	-1,50
**KRTAP10-2**	0,00	1,50	-1,50
**IGLV5-45**	0,00	1,50	-1,50
**IGLV1-40**	0,00	1,50	-1,50

**TRBV4-1**	0,00	1,50	-1,50
**IFNA7**	0,00	1,50	-1,50
**NOTO**	0,00	1,50	-1,50
**KRTAP10-4**	0,00	1,50	-1,50
**RPTN**	0,00	1,50	-1,50
**PSG3**	0,00	1,50	-1,50
**XKR9**	0,00	1,50	-1,50
**APOC4-APOC2**	0,00	1,50	-1,50
**ORM2**	0,00	1,50	-1,50
**NUTM2E**	0,00	1,50	-1,50
**PGA3**	0,00	1,50	-1,50
**IFNA4**	0,00	1,50	-1,50
**MEIOSIN**	0,00	1,50	-1,50
**PNMA2**	0,00	1,50	-1,50
**APELA**	0,00	1,50	-1,50
**PCDHA10**	0,00	1,50	-1,50
**AL033529.1**	0,00	1,50	-1,50
**LYPD8**	0,00	1,50	-1,50
**AC092073.1**	0,00	1,50	-1,50
**AC010646.1**	0,00	1,50	-1,50
**HEATR9**	0,00	1,50	-1,50
**AC104109.3**	0,00	1,50	-1,50
**AC073283.3**	0,00	1,50	-1,50
**PWWP4**	0,00	1,50	-1,50
**SMIM41**	0,00	1,50	-1,50
**TMTC1**	0,00	1,50	-1,50
**TTBK1**	0,00	1,50	-1,50
**CDHR1**	0,00	1,50	-1,50
**BDKRB2**	0,00	1,50	-1,50
**GRK1**	0,00	1,50	-1,50
**AC025165.3**	4,45	5,94	-1,50
**H3C11**	2,09	3,59	-1,49
**C16orf46**	4,12	5,61	-1,49
**PLEKHH2**	4,37	5,86	-1,49
**ABCG4**	1,86	3,34	-1,48
**ADORA2A**	3,50	4,98	-1,48
**FAM98C**	3,77	5,25	-1,48
**LTC4S**	2,42	3,88	-1,46
**AC018512.1**	2,42	3,88	-1,46
**HHIP**	5,25	6,71	-1,46
**TMEM217**	4,29	5,75	-1,46
**FNDC5**	4,11	5,57	-1,46
**CHCHD5**	5,91	7,35	-1,44
**ZNF630**	5,05	6,48	-1,43
**SEZ6**	4,11	5,54	-1,43
**MAGED4B**	5,69	7,11	-1,42
**KLB**	3,82	5,24	-1,42
**NRXN3**	2,92	4,34	-1,42
**SLC7A10**	1,06	2,48	-1,42
**DCDC2C**	1,06	2,48	-1,42
**AL512506.3**	1,06	2,48	-1,42
**PRDM11**	1,15	2,57	-1,42
**SPAG4**	3,72	5,14	-1,42
**CADM1**	2,95	4,36	-1,41
**GCNT4**	3,28	4,69	-1,41
**SLC4A4**	3,83	5,23	-1,40

**CDH26**	5,96	7,35	-1,39
**ALX4**	1,67	3,06	-1,39
**WFDC1**	1,67	3,06	-1,39
**PRDM12**	1,67	3,06	-1,39
**CNGA4**	1,67	3,06	-1,39
**SLC10A6**	1,67	3,06	-1,39
**P4HA3**	1,67	3,06	-1,39
**SHANK2**	1,67	3,06	-1,39
**CPLX1**	1,67	3,06	-1,39
**PIFO**	1,67	3,06	-1,39
**H2AC21**	1,67	3,06	-1,39
**FAM177B**	1,67	3,06	-1,39
**PVALEF**	1,67	3,06	-1,39
**AL121899.2**	1,67	3,06	-1,39
**AC093884.1**	1,67	3,06	-1,39
**SLC22A18AS**	3,21	4,60	-1,39
**TNFRSF10A**	4,82	6,20	-1,38
**ITIH3**	4,87	6,25	-1,38
**RUNDC1**	2,09	3,47	-1,38
**KLHL10**	0,98	2,36	-1,38
**RAB26**	4,81	6,19	-1,38
**FAM222B**	0,98	2,35	-1,37
**C17orf97**	4,37	5,74	-1,37
**EDN1**	2,42	3,79	-1,37
**KDR**	2,42	3,79	-1,37
**CNR2**	2,42	3,79	-1,37
**SSX5**	2,42	3,79	-1,37
**C5AR1**	2,42	3,79	-1,37
**RPEL1**	2,42	3,79	-1,37
**FOXH1**	2,69	4,05	-1,36
**AC008012.1**	7,07	8,43	-1,36
**ZSCAN4**	2,91	4,27	-1,36
**PHLDB3**	5,10	6,46	-1,36
**C3AR1**	3,14	4,49	-1,36
**C9orf131**	3,33	4,68	-1,35
**GRHL1**	4,58	5,93	-1,35
**SLITRK6**	4,02	5,37	-1,35
**TNS4**	1,81	3,16	-1,35
**OXCT2**	4,37	5,71	-1,35
**MYO15A**	2,54	3,88	-1,34
**AL121845.3**	8,16	9,50	-1,34
**POLR2M**	11,82	13,16	-1,33
**AC092587.1**	5,74	7,07	-1,33
**SKOR1**	3,37	4,70	-1,33
**SBF1**	9,46	10,78	-1,33
**ABCA4**	4,51	5,83	-1,32
**STXBP6**	4,01	5,33	-1,32
**BOLA2B**	8,63	9,95	-1,32
**SSC4D**	4,29	5,61	-1,32
**SNCAIP**	1,00	2,32	-1,32
**DCAF1**	7,68	9,00	-1,32
**TTC28**	4,55	5,86	-1,31
**FST**	1,00	2,31	-1,31
**TNXB**	7,85	9,16	-1,31
**AC009163.5**	3,57	4,88	-1,31
**AC005520.1**	4,76	6,07	-1,31

**EPS8L2**	5,76	7,06	-1,30
**CDH24**	7,91	9,21	-1,30
**RDH5**	4,37	5,66	-1,29
**RPA4**	2,91	4,20	-1,29
**IGFLR1**	7,05	8,34	-1,29
**ADAMTSL4**	3,73	5,01	-1,28
**ZSWIM5**	4,02	5,31	-1,28
**CATSPERZ**	1,78	3,06	-1,28
**CLMP**	2,42	3,69	-1,27
**ZSCAN20**	2,42	3,69	-1,27
**ARHGEF16**	2,42	3,69	-1,27
**IL17RD**	2,42	3,69	-1,27
**IGHG4**	2,42	3,69	-1,27
**CYSRT1**	4,95	6,22	-1,27
**HS3ST1**	3,57	4,83	-1,26
**IGDCC4**	3,57	4,83	-1,26
**AC005943.1**	8,45	9,71	-1,26
**POC1B-GALNT4**	8,13	9,38	-1,26
**TGM1**	5,60	6,86	-1,26
**NOX1**	3,43	4,69	-1,25
**MMP9**	2,09	3,35	-1,25
**ACOT12**	2,09	3,35	-1,25
**C3orf20**	1,57	2,82	-1,25
**RYR2**	1,03	2,28	-1,25
**TP73**	3,28	4,53	-1,24
**ESYT3**	3,28	4,53	-1,24
**RAB27B**	3,28	4,53	-1,24
**OR2A7**	3,98	5,23	-1,24
**DYDC2**	6,06	7,30	-1,24
**CCDC40**	2,41	3,65	-1,24
**DNAI3**	3,11	4,34	-1,23
**TAS2R43**	3,11	4,34	-1,23
**KLHL31**	4,20	5,43	-1,23
**PRH2**	4,14	5,37	-1,23
**PROM1**	1,49	2,71	-1,23
**DDX43**	1,67	2,89	-1,22
**SPEF1**	1,67	2,89	-1,22
**C7orf57**	1,67	2,89	-1,22
**CHRNA2**	4,12	5,34	-1,22
**LKAAEAR1**	2,91	4,13	-1,22
**C3orf56**	2,91	4,13	-1,22
**KIF19**	2,91	4,13	-1,22
**SMPD3**	5,67	6,89	-1,22
**TSHR**	4,13	5,34	-1,22
**SPATA48**	4,02	5,24	-1,21
**MAST4**	6,37	7,58	-1,21
**SYT17**	4,24	5,44	-1,21
**CC2D2B**	4,63	5,83	-1,20
**SYT5**	3,92	5,12	-1,20
**SOX15**	4,63	5,83	-1,20
**SAPCD1**	6,78	7,98	-1,20
**KBTBD11-OT1**	5,65	6,84	-1,20
**NPAS3**	4,91	6,11	-1,20
**CEP131**	9,70	10,89	-1,20
**SLC25A44**	6,63	7,83	-1,19
**TMEM154**	4,34	5,53	-1,19

**TMEM51**	4,36	5,56	-1,19
**SLAMF6**	3,28	4,47	-1,19
**FBXO32**	6,19	7,38	-1,18
**TBC1D3I**	4,93	6,11	-1,18
**CYB5RL**	7,60	8,77	-1,18
**CAMK2B**	2,22	3,40	-1,18
**SLC22A4**	5,03	6,20	-1,17
**EPHA10**	1,39	2,56	-1,17
**PCDHGA3**	4,82	5,99	-1,17
**FAM184B**	3,57	4,74	-1,17
**AC078927.1**	2,42	3,59	-1,16
**THBS2**	2,42	3,59	-1,16
**IRAK3**	2,42	3,59	-1,16
**EPHB1**	2,42	3,59	-1,16
**KLF15**	2,42	3,59	-1,16
**PDE7B**	2,42	3,59	-1,16
**FKBP6**	1,06	2,22	-1,16
**SGCG**	1,06	2,22	-1,16
**BTN1A1**	1,06	2,22	-1,16
**OR1J1**	1,06	2,22	-1,16
**OR51B5**	1,06	2,22	-1,16
**TMEM88**	1,06	2,22	-1,16
**APOF**	1,06	2,22	-1,16
**NOXO1**	1,06	2,22	-1,16
**IGHV8-51-1**	1,06	2,22	-1,16
**CYP2A6**	1,06	2,22	-1,16
**CCL3L3**	1,06	2,22	-1,16
**AC024598.1**	1,06	2,22	-1,16
**SHISA9**	3,62	4,77	-1,16
**NAALADL2**	5,58	6,73	-1,16
**TTC25**	4,40	5,55	-1,15
**IL20RB**	4,60	5,75	-1,15
**MAP3K7CL**	5,37	6,52	-1,15
**NAV3**	3,10	4,25	-1,15
**BFSP1**	5,07	6,22	-1,14
**KLHDC9**	3,44	4,58	-1,14
**LRRC24**	4,02	5,16	-1,14
**AC007192.1**	7,33	8,47	-1,14
**AL355987.3**	3,70	4,83	-1,14
**CXADR**	3,61	4,75	-1,13
**CRACD**	4,81	5,94	-1,13
**GAL3ST3**	3,92	5,05	-1,13
**SLC25A18**	2,96	4,08	-1,13
**PTGES3L-AARSD1**	4,84	5,96	-1,13
**CHADL**	4,65	5,77	-1,12
**HAVCR2**	4,64	5,76	-1,12
**DAPK2**	2,09	3,21	-1,12
**GYPB**	0,96	2,07	-1,11
**AC010326.2**	5,21	6,32	-1,11
**BMPR1A**	5,59	6,70	-1,11
**EIF4E3**	5,36	6,47	-1,11
**ZNF628**	8,61	9,72	-1,11
**TCEAL3**	7,81	8,92	-1,11
**TAS2R5**	5,07	6,18	-1,11
**CATSPERG**	4,02	5,13	-1,11
**IGFBP6**	2,67	3,77	-1,10

**AC092647.5**	5,64	6,75	-1,10
**SEMA6D**	6,20	7,30	-1,10
**INSRR**	3,11	4,20	-1,10
**MSANTD1**	4,31	5,40	-1,10
**AC069288.1**	6,97	8,06	-1,09
**UMPS**	12,10	13,19	-1,09
**RSPH10B2**	4,28	5,37	-1,09
**LCA5L**	5,34	6,43	-1,09
**GRK5**	5,33	6,42	-1,09
**FBXO33**	4,61	5,69	-1,09
**STK19**	7,88	8,97	-1,09
**CCDC9**	9,31	10,39	-1,08
**FBXO24**	4,05	5,14	-1,08
**NPAS2**	5,79	6,87	-1,08
**CCT6B**	5,47	6,55	-1,08
**ARL6IP4**	10,34	11,42	-1,08
**CLU**	7,83	8,91	-1,08
**SLC5A9**	1,65	2,72	-1,07
**C1S**	5,90	6,97	-1,07
**GNB3**	5,16	6,24	-1,07
**TDRD6**	3,82	4,88	-1,07
**KCNJ4**	3,57	4,64	-1,06
**OR2A42**	3,57	4,64	-1,06
**CFAP45**	3,57	4,64	-1,06
**STX1A**	5,18	6,25	-1,06
**AGBL4**	3,28	4,34	-1,06
**TGFB2**	3,92	4,98	-1,06
**HHLA2**	3,72	4,78	-1,06
**OR1L8**	2,91	3,97	-1,06
**SNAI2**	2,91	3,97	-1,06
**CHRNA9**	2,91	3,97	-1,06
**NLGN3**	6,66	7,72	-1,05
**TNNT1**	7,85	8,90	-1,05
**TLE3**	2,36	3,41	-1,05
**C17orf107**	2,42	3,47	-1,05
**FMOD**	4,12	5,16	-1,05
**TAF15**	12,57	13,62	-1,05
**ZGLP1**	5,77	6,81	-1,04
**AC068896.1**	4,51	5,55	-1,04
**ARMC9**	8,24	9,29	-1,04
**ANKDD1B**	2,15	3,19	-1,04
**GPT**	3,70	4,74	-1,04
**HLA-C**	9,44	10,48	-1,04
**BAIAP3**	4,82	5,86	-1,04
**NHSL2**	3,03	4,06	-1,04
**ACVRL1**	1,11	2,15	-1,03
**FAM47E**	2,72	3,76	-1,03
**DSPP**	1,67	2,70	-1,03
**SMCO2**	1,67	2,70	-1,03
**TRIM31**	1,67	2,70	-1,03
**TTC39A**	1,67	2,70	-1,03
**TBX15**	1,67	2,70	-1,03
**ASPA**	1,67	2,70	-1,03
**DLX4**	1,67	2,70	-1,03
**VSTM2L**	1,67	2,70	-1,03
**KCTD14**	1,67	2,70	-1,03

**TMEM163**	1,67	2,70	-1,03
**MAP6**	1,67	2,70	-1,03
**EGR3**	1,67	2,70	-1,03
**GPR62**	1,67	2,70	-1,03
**HEPHL1**	1,67	2,70	-1,03
**POU3F1**	1,67	2,70	-1,03
**C21orf62**	1,67	2,70	-1,03
**IGHV7-81**	1,67	2,70	-1,03
**TAS2R46**	1,67	2,70	-1,03
**TSNAX-DISC1**	1,67	2,70	-1,03
**IGHV4-4**	1,67	2,70	-1,03
**CKLF-CMTM1**	5,62	6,65	-1,03
**AL139011.2**	3,30	4,33	-1,03
**SGSM1**	2,84	3,87	-1,03
**SDSL**	1,83	2,86	-1,03
**AMDHD1**	5,61	6,64	-1,03
**GPRASP1**	6,21	7,23	-1,03
**SLC2A14**	3,93	4,95	-1,02
**CA5A**	1,12	2,14	-1,02
**GLOD4**	6,45	7,47	-1,02
**SLC7A9**	3,44	4,46	-1,02
**PTMS**	3,82	4,83	-1,02
**HAS1**	3,82	4,83	-1,02
**POSTN**	1,68	2,69	-1,02
**CCDC17**	3,89	4,89	-1,01
**ZNF385D**	6,35	7,35	-1,01
**DNAJB12**	10,27	11,28	-1,01
**ABCG8**	2,69	3,69	-1,00
**GRAMD1B**	7,26	8,26	-1,00
**ARHGEF4**	4,70	5,70	-1,00
**C19orf81**	5,68	6,67	-1,00
**ANKRD23**	7,13	8,12	-1,00
**ZSCAN18**	10,92	11,91	-0,99
**ATOH7**	3,28	4,27	-0,99
**AC091551.1**	3,28	4,27	-0,99
**TBX1**	2,70	3,69	-0,99
**AL360181.3**	4,39	5,38	-0,99
**HCN2**	3,70	4,69	-0,99
**CBLC**	2,15	3,14	-0,99
**FAM53A**	6,08	7,06	-0,99
**LST1**	3,77	4,76	-0,99
**MARS1**	13,74	14,73	-0,98
**SRRM3**	4,50	5,48	-0,98
**SYTL2**	6,83	7,81	-0,98
**C19orf44**	8,62	9,60	-0,98
**GPR156**	2,65	3,63	-0,98
**SH3D21**	6,90	7,87	-0,98
**EFEMP2**	4,04	5,02	-0,98
**MCCC2**	10,10	11,08	-0,98
**POPDC3**	4,86	5,83	-0,97
**SPRN**	4,37	5,34	-0,97
**DCC**	4,49	5,46	-0,97
**TMC5**	2,91	3,88	-0,97
**TSGA10IP**	2,91	3,88	-0,97
**MKRN2OS**	2,91	3,88	-0,97
**AP000311.1**	7,54	8,51	-0,97

**PLCD4**	5,11	6,08	-0,97
**IHH**	2,09	3,06	-0,97
**OR2C1**	2,09	3,06	-0,97
**AC113348.1**	2,09	3,06	-0,97
**SEMA4B**	8,22	9,18	-0,97
**CRB1**	1,70	2,66	-0,96
**PPFIA3**	8,82	9,78	-0,96
**WDR97**	5,00	5,97	-0,96
**ZSCAN5A**	7,49	8,45	-0,96
**RGS9**	3,51	4,47	-0,96
**PFN4**	4,15	5,11	-0,96
**ABCF3**	5,55	6,51	-0,96
**C1QTNF7**	1,15	2,10	-0,96
**MFAP4**	4,51	5,46	-0,95
**RAB39A**	4,51	5,46	-0,95
**ARL2-SNX15**	4,51	5,46	-0,95
**ITGAM**	3,57	4,53	-0,95
**TMEM216**	3,57	4,53	-0,95
**MIA-RAB4B**	3,57	4,53	-0,95
**EPSTI1**	3,57	4,52	-0,95
**AL135905.2**	12,24	13,19	-0,95
**SEPTIN4**	5,14	6,09	-0,95
**LGI4**	5,26	6,21	-0,95
**GHRL**	4,38	5,32	-0,94
**SLN**	1,35	2,29	-0,94
**UFSP1**	5,90	6,84	-0,94
**TRPC5**	0,00	0,94	-0,94
**BOC**	0,00	0,94	-0,94
**SIM2**	0,00	0,94	-0,94
**OR10A2**	0,00	0,94	-0,94
**FRRS1L**	0,00	0,94	-0,94
**DNAH9**	0,00	0,94	-0,94
**KCNQ2**	0,00	0,94	-0,94
**SLC5A5**	0,00	0,94	-0,94
**FSCN3**	0,00	0,94	-0,94
**RLN1**	0,00	0,94	-0,94
**MYCT1**	0,00	0,94	-0,94
**ARR3**	0,00	0,94	-0,94
**CSRP3**	0,00	0,94	-0,94
**SNTG1**	0,00	0,94	-0,94
**RSPO2**	0,00	0,94	-0,94
**NPHS1**	0,00	0,94	-0,94
**HPGDS**	0,00	0,94	-0,94
**GOLT1A**	0,00	0,94	-0,94
**C11orf87**	0,00	0,94	-0,94
**SPATC1**	0,00	0,94	-0,94
**KRT12**	0,00	0,94	-0,94
**ENO4**	0,00	0,94	-0,94
**CCDC9B**	0,00	0,94	-0,94
**RTP2**	0,00	0,94	-0,94
**C22orf42**	0,00	0,94	-0,94
**IGLV7-46**	0,00	0,94	-0,94
**TRGV4**	0,00	0,94	-0,94
**TRAV26-1**	0,00	0,94	-0,94
**TRDC**	0,00	0,94	-0,94
**IGHV3-73**	0,00	0,94	-0,94

**WEE2**	0,00	0,94	-0,94
**IGHV3-72**	0,00	0,94	-0,94
**PHGR1**	0,00	0,94	-0,94
**KDM4F**	0,00	0,94	-0,94
**AL133410.3**	0,00	0,94	-0,94
**FAM90A22P**	0,00	0,94	-0,94
**PROKR2**	0,00	0,94	-0,94
**CCL17**	0,00	0,94	-0,94
**ART4**	0,00	0,94	-0,94
**CD80**	0,00	0,94	-0,94
**AKR1D1**	0,00	0,94	-0,94
**CEACAM8**	0,00	0,94	-0,94
**SPDEF**	0,00	0,94	-0,94
**SSX1**	0,00	0,94	-0,94
**RFPL1**	0,00	0,94	-0,94
**KCP**	0,00	0,94	-0,94
**SLC22A14**	0,00	0,94	-0,94
**SLC5A12**	0,00	0,94	-0,94
**ATP6V1G3**	0,00	0,94	-0,94
**ACMSD**	0,00	0,94	-0,94
**ART3**	0,00	0,94	-0,94
**PGLYRP3**	0,00	0,94	-0,94
**IL31RA**	0,00	0,94	-0,94
**GLOD5**	0,00	0,94	-0,94
**OR2T33**	0,00	0,94	-0,94
**ZAR1**	0,00	0,94	-0,94
**GAST**	0,00	0,94	-0,94
**SELENOV**	0,00	0,94	-0,94
**KIR2DL4**	0,00	0,94	-0,94
**ZNF679**	0,00	0,94	-0,94
**RAET1G**	0,00	0,94	-0,94
**OR2J3**	0,00	0,94	-0,94
**COL6A6**	0,00	0,94	-0,94
**TRAV23DV6**	0,00	0,94	-0,94
**IGHA2**	0,00	0,94	-0,94
**IGHA1**	0,00	0,94	-0,94
**IGHV3-23**	0,00	0,94	-0,94
**AC011005.1**	0,00	0,94	-0,94
**GAGE12F**	0,00	0,94	-0,94
**BGLAP**	0,00	0,94	-0,94
**ZNF723**	0,00	0,94	-0,94
**AL590132.1**	0,00	0,94	-0,94
**RPL36A-HNRNPH2**	10,72	11,65	-0,94
**NUTM2A**	7,01	7,95	-0,94
**HOXA2**	3,70	4,64	-0,94
**ZNF837**	7,65	8,58	-0,93
**SLC2A10**	6,44	7,37	-0,93
**CDHR5**	2,11	3,04	-0,93
**USP40**	5,22	6,15	-0,93
**PODNL1**	4,86	5,78	-0,93
**INHBB**	5,44	6,37	-0,93
**CACNA1I**	2,42	3,35	-0,93
**ITGA1**	2,42	3,35	-0,93
**RASL10A**	2,42	3,35	-0,93
**KRTAP5-7**	2,42	3,35	-0,93
**UBE2L5**	1,50	2,43	-0,93

**GJC2**	3,28	4,20	-0,92
**ARC**	3,82	4,74	-0,92
**DRAP1**	11,40	12,32	-0,92
**ADHFE1**	4,05	4,98	-0,92
**GRM2**	2,11	3,04	-0,92
**MED19**	5,43	6,36	-0,92
**RNF103-CHMP3**	6,17	7,09	-0,92
**CFAP126**	5,03	5,94	-0,92
**SNX24**	6,72	7,64	-0,92
**RGS1**	5,63	6,55	-0,91
**RBFOX3**	3,83	4,75	-0,91
**AL441992.2**	7,86	8,77	-0,91
**OTP**	3,92	4,83	-0,91
**AL138752.2**	3,92	4,83	-0,91
**COL4A1**	5,74	6,65	-0,91
**PYROXD2**	5,29	6,20	-0,91
**CYTIP**	4,64	5,55	-0,91
**CDKL1**	9,16	10,06	-0,91
**SLC47A1**	2,43	3,34	-0,90
**ANXA1**	4,02	4,92	-0,90
**AC023055.1**	7,17	8,07	-0,90
**PILRA**	4,87	5,77	-0,90
**CBX7**	6,74	7,64	-0,90
**CETP**	4,05	4,94	-0,90
**GPR68**	5,41	6,30	-0,90
**GALNT17**	7,06	7,95	-0,90
**POPDC2**	4,52	5,42	-0,89
**SHC3**	4,12	5,01	-0,89
**EDDM13**	4,05	4,94	-0,89
**DDT**	8,42	9,31	-0,89
**DHRS3**	4,78	5,67	-0,89
**DNLZ**	9,12	10,01	-0,89
**EGF**	4,54	5,43	-0,89
**VTN**	4,11	5,00	-0,89
**ABTB2**	6,77	7,65	-0,89
**POU5F1**	4,82	5,71	-0,88
**CFAP57**	2,47	3,36	-0,88
**SAXO2**	4,06	4,94	-0,88
**CAMK4**	9,02	9,90	-0,88
**AL162231.3**	3,70	4,58	-0,88
**NOD2**	3,93	4,81	-0,88
**BRICD5**	5,61	6,49	-0,88
**GFRA1**	2,91	3,79	-0,88
**KCNJ10**	2,91	3,79	-0,88
**LTB**	2,91	3,79	-0,88
**SYBU**	2,91	3,79	-0,88
**RAMP2**	3,23	4,11	-0,88
**CLPSL2**	1,52	2,40	-0,87
**AL662899.2**	7,13	8,00	-0,87
**GOLGA3**	5,74	6,61	-0,87
**IQUB**	1,53	2,39	-0,87
**CLEC18A**	4,31	5,18	-0,87
**TMEM231**	8,93	9,80	-0,87
**MYRFL**	3,88	4,74	-0,87
**CCDC102A**	7,82	8,69	-0,86
**AC009070.1**	4,03	4,90	-0,86

**PRKG1**	5,24	6,10	-0,86
**SLC12A5**	7,88	8,74	-0,86
**LHX6**	5,28	6,14	-0,86
**C4orf48**	6,21	7,07	-0,86
**SPIB**	6,93	7,79	-0,86
**NR4A2**	5,98	6,83	-0,85
**CROCC**	9,35	10,21	-0,85
**ALKBH6**	7,00	7,85	-0,85
**H4-16**	5,03	5,88	-0,85
**MZT2B**	11,96	12,81	-0,85
**CASKIN1**	5,37	6,22	-0,85
**AURKAIP1**	8,43	9,28	-0,85
**MIEF2**	8,15	9,00	-0,85
**GPR55**	5,39	6,24	-0,85
**RBP1**	4,89	5,74	-0,85
**FAM169A**	3,34	4,19	-0,85
**CCDC12**	6,83	7,68	-0,85
**DUSP9**	5,86	6,70	-0,85
**HLA-DQB1**	7,23	8,07	-0,85
**TNK1**	4,93	5,78	-0,85
**GAREM1**	7,05	7,90	-0,85
**BBC3**	7,91	8,76	-0,85
**AC126283.1**	1,06	1,91	-0,85
**PLEKHG3**	6,78	7,63	-0,84
**OCA2**	2,93	3,77	-0,84
**PTPRH**	1,75	2,60	-0,84
**TXNRD3**	3,39	4,23	-0,84
**C9orf116**	4,52	5,36	-0,84
**INHBE**	6,09	6,93	-0,84
**SURF2**	9,35	10,19	-0,84
**KCNMA1**	6,20	7,04	-0,84
**H4C14**	6,47	7,30	-0,84
**HSPA6**	7,75	8,59	-0,83
**CPLANE2**	6,68	7,51	-0,83
**STKLD1**	3,57	4,41	-0,83
**NPB**	3,48	4,31	-0,83
**AC026954.2**	7,72	8,55	-0,83
**TMEM178A**	2,72	3,55	-0,83
**KLHL26**	9,27	10,10	-0,83
**ZCWPW2**	5,43	6,25	-0,83
**TUBB1**	5,48	6,30	-0,83
**ANKRD7**	3,49	4,31	-0,82
**SYCE1L**	6,38	7,20	-0,82
**CDC42EP3**	7,23	8,04	-0,82
**LYSMD4**	8,17	8,99	-0,82
**GCOM1**	1,67	2,48	-0,81
**A1CF**	1,67	2,48	-0,81
**RAMACL**	1,67	2,48	-0,81
**CD2BP2**	11,92	12,74	-0,81
**MRPL41**	9,23	10,05	-0,81
**FBXO15**	5,12	5,94	-0,81
**TMIGD2**	6,31	7,13	-0,81
**LMNTD2**	5,77	6,58	-0,81
**AC091167.7**	4,93	5,74	-0,81
**COLCA2**	1,21	2,02	-0,81
**DNPH1**	9,73	10,54	-0,81

**LYPD3**	4,12	4,92	-0,81
**CORT**	5,03	5,83	-0,81
**COL7A1**	6,76	7,56	-0,80
**ECEL1**	1,55	2,36	-0,80
**RAPSN**	1,08	1,88	-0,80
**PLEKHA4**	6,46	7,27	-0,80
**TLE2**	3,84	4,64	-0,80
**GDAP1L1**	4,06	4,86	-0,80
**MSTN**	4,37	5,16	-0,80
**KCNK5**	4,37	5,16	-0,80
**MICALL2**	5,96	6,76	-0,80
**COX6B2**	2,09	2,89	-0,80
**BRME1**	4,98	5,78	-0,80
**CA12**	2,51	3,31	-0,80
**MATN4**	3,46	4,25	-0,80
**MYH11**	5,90	6,70	-0,79
**AC040162.1**	5,93	6,72	-0,79
**NSUN4**	10,35	11,14	-0,79
**GRAMD2A**	2,42	3,21	-0,79
**PAPPA**	2,42	3,21	-0,79
**LDB3**	2,42	3,21	-0,79
**MPPED1**	2,42	3,21	-0,79
**LCN8**	2,42	3,21	-0,79
**HES4**	1,56	2,35	-0,79
**DNAJB2**	8,25	9,04	-0,79
**LILRA1**	2,69	3,47	-0,78
**KIF4B**	2,69	3,47	-0,78
**PNPLA3**	4,36	5,14	-0,78
**PPP1R32**	5,29	6,07	-0,78
**MAB21L1**	2,91	3,69	-0,78
**PIPOX**	2,91	3,69	-0,78
**PRR29**	8,32	9,09	-0,78
**BIK**	6,66	7,44	-0,78
**SPACA6**	6,14	6,92	-0,78
**SNPH**	5,37	6,14	-0,78
**LINC00634**	5,44	6,22	-0,78
**HEPACAM2**	2,36	3,13	-0,77
**TRIM69**	6,45	7,23	-0,77
**IRX5**	3,28	4,05	-0,77
**NOVA2**	8,06	8,84	-0,77
**RND1**	6,31	7,08	-0,77
**LITAFD**	2,43	3,20	-0,77
**ELMO3**	8,10	8,87	-0,77
**SLC39A8**	11,12	11,89	-0,77
**TMEM182**	7,52	8,29	-0,77
**ZAN**	4,77	5,54	-0,77
**FMO4**	3,82	4,58	-0,77
**TCL1B**	3,82	4,58	-0,77
**FOXD4L4**	3,92	4,69	-0,77
**FUT7**	4,12	4,88	-0,76
**AC091959.1**	8,51	9,28	-0,76
**ZNF474**	4,37	5,13	-0,76
**NFIB**	6,50	7,26	-0,76
**DMGDH**	4,23	4,99	-0,76
**PGF**	5,69	6,45	-0,76
**PPP2R3A**	7,29	8,05	-0,76

**SART1**	12,02	12,77	-0,76
**AHNAK**	6,19	6,94	-0,76
**MTARC1**	8,05	8,81	-0,75
**SLC26A1**	7,01	7,77	-0,75
**ODAPH**	2,62	3,37	-0,75
**DOCK5**	7,40	8,15	-0,75
**MON1A**	4,30	5,04	-0,75
**ABLIM2**	4,58	5,33	-0,75
**TBX3**	5,57	6,31	-0,75
**AC135050.2**	6,02	6,76	-0,75
**HSPA1A**	11,23	11,97	-0,75
**AP001931.2**	9,17	9,92	-0,74
**RHBDF1**	4,29	5,04	-0,74
**CCN1**	2,29	3,03	-0,74
**TMEM45A**	4,62	5,36	-0,74
**DCAF15**	7,52	8,26	-0,74
**FAM163B**	6,79	7,54	-0,74
**NR6A1**	6,22	6,95	-0,74
**PHEX**	5,25	5,99	-0,74
**PKDREJ**	5,16	5,90	-0,74
**MYLK2**	6,85	7,58	-0,73
**PROX1**	6,20	6,94	-0,73
**YPEL1**	11,19	11,92	-0,73
**ST20-MTHFS**	5,74	6,47	-0,73
**BTBD18**	4,82	5,55	-0,73
**ELAC1**	8,92	9,66	-0,73
**SLC7A8**	3,57	4,30	-0,73
**EXOC3L2**	5,82	6,55	-0,73
**PPP1R12C**	11,74	12,46	-0,73
**CFAP54**	1,25	1,97	-0,72
**CAV1**	4,29	5,01	-0,72
**SLC22A13**	2,21	2,93	-0,72
**H3C12**	4,20	4,92	-0,72
**C7orf50**	10,44	11,16	-0,72
**AL157935.2**	9,86	10,58	-0,72
**AL022238.3**	9,48	10,20	-0,72
**C15orf65**	5,37	6,09	-0,72
**MYCL**	4,87	5,59	-0,72
**TFPT**	9,19	9,91	-0,72
**ACTL10**	5,95	6,67	-0,72
**CLEC4A**	4,69	5,41	-0,72
**NEFH**	4,02	4,74	-0,72
**IFNA21**	4,02	4,74	-0,72
**EFHB**	4,02	4,74	-0,72
**FXYD2**	4,72	5,43	-0,72
**CFAP77**	4,11	4,82	-0,72
**FAM20A**	3,69	4,40	-0,71
**MBLAC1**	5,40	6,11	-0,71
**AL136295.5**	9,40	10,11	-0,71
**PCDH17**	3,82	4,53	-0,71
**RCN3**	3,82	4,53	-0,71
**POU6F1**	7,70	8,41	-0,71
**SYN3**	5,03	5,74	-0,71
**ZMYND15**	1,12	1,83	-0,71
**CBSL**	6,10	6,81	-0,71
**RPP25**	7,57	8,27	-0,71

**CA3**	3,70	4,41	-0,71
**NUDT10**	5,21	5,92	-0,71
**TCEAL4**	10,04	10,75	-0,71
**LLCFC1**	1,60	2,30	-0,71
**AC090360.1**	6,97	7,67	-0,70
**MLIP**	5,74	6,44	-0,70
**CCDC124**	11,77	12,47	-0,70
**CALML6**	4,34	5,04	-0,70
**DNMT3A**	12,52	13,22	-0,70
**AMER2**	3,57	4,27	-0,70
**MS4A4E**	3,57	4,27	-0,70
**C4orf36**	4,45	5,16	-0,70
**NHLH1**	4,76	5,46	-0,70
**ESRP2**	4,13	4,83	-0,70
**ARHGAP23**	11,15	11,85	-0,70
**C3orf49**	3,05	3,74	-0,70
**SRGAP2B**	9,85	10,55	-0,70
**MPZL3**	4,34	5,03	-0,70
**DDRGK1**	10,00	10,70	-0,70
**ETV2**	3,92	4,61	-0,69
**RNASE10**	4,49	5,18	-0,69
**CDKN2A**	8,21	8,90	-0,69
**NT5DC4**	3,28	3,97	-0,69
**NCF1**	3,28	3,97	-0,69
**SHISA3**	3,28	3,97	-0,69
**ZNF80**	3,28	3,97	-0,69
**KIRREL3**	1,61	2,30	-0,69
**PVRIG**	5,55	6,24	-0,69
**PPP1R35**	9,82	10,50	-0,69
**TRAPPC5**	9,66	10,34	-0,69
**GRIK4**	2,23	2,91	-0,69
**HRH2**	8,65	9,34	-0,69
**CRISPLD2**	5,27	5,96	-0,69
**RTKN**	8,83	9,51	-0,68
**HARBI1**	7,75	8,43	-0,68
**ADAMTS4**	3,11	3,79	-0,68
**TMED6**	4,98	5,66	-0,68
**INO80B**	8,79	9,47	-0,68
**CCDC102B**	5,13	5,81	-0,68
**ALOX12B**	3,36	4,04	-0,68
**KCNMB2**	1,73	2,41	-0,68
**SLC34A3**	2,96	3,64	-0,68
**AP001453.3**	5,34	6,02	-0,67
**LRRC17**	3,59	4,26	-0,67
**SEMA3B**	6,21	6,88	-0,67
**PROCA1**	8,39	9,07	-0,67
**HES6**	10,88	11,56	-0,67
**JDP2**	9,27	9,94	-0,67
**LYZ**	2,91	3,59	-0,67
**HPCAL4**	2,91	3,59	-0,67
**CNGA1**	2,91	3,59	-0,67
**PRPH**	2,91	3,59	-0,67
**CPO**	2,91	3,59	-0,67
**C10orf95**	7,30	7,97	-0,67
**PKIB**	2,99	3,66	-0,67
**LRRC31**	1,62	2,29	-0,67

**DYNC1I1**	6,57	7,24	-0,67
**TMEM225B**	4,06	4,73	-0,67
**PIP4K2A**	10,71	11,38	-0,67
**F13B**	2,07	2,73	-0,67
**MTRNR2L4**	5,16	5,83	-0,67
**TTC36**	3,72	4,38	-0,67
**ZBTB3**	8,99	9,65	-0,67
**DEUP1**	1,62	2,28	-0,66
**CAPN9**	3,81	4,47	-0,66
**INAFM1**	7,01	7,66	-0,66
**TBCEL-TECTA**	2,69	3,35	-0,66
**TRDMT1**	10,58	11,24	-0,66
**SDHAF1**	8,35	9,00	-0,66
**CAPN8**	1,62	2,28	-0,65
**ACRBP**	5,77	6,43	-0,65
**FIGNL2**	5,79	6,45	-0,65
**USP42**	5,83	6,48	-0,65
**PMVK**	10,78	11,43	-0,65
**CYP2D7**	3,82	4,47	-0,65
**ARRDC2**	10,58	11,23	-0,65
**UQCC3**	10,07	10,72	-0,65
**MAP3K6**	8,37	9,02	-0,65
**PNPT1**	13,11	13,76	-0,65
**NPHP3**	9,05	9,70	-0,65
**AC055839.2**	6,75	7,40	-0,65
**COL9A2**	7,79	8,44	-0,65
**CCDC85B**	11,84	12,48	-0,65
**QPCTL**	9,56	10,21	-0,65
**CES4A**	7,07	7,72	-0,65
**BICRA**	7,85	8,50	-0,65
**IMP3**	6,82	7,47	-0,65
**CYB5R2**	2,44	3,08	-0,64
**FAM228A**	2,67	3,31	-0,64
**DOCK1**	6,56	7,20	-0,64
**EXT1**	6,71	7,35	-0,64
**SLC4A5**	7,15	7,80	-0,64
**CIRBP**	13,41	14,05	-0,64
**PSMG4**	10,83	11,47	-0,64
**CLDN5**	5,01	5,65	-0,64
**ZNF746**	10,49	11,13	-0,64
**AL358113.1**	9,20	9,84	-0,64
**COL23A1**	3,85	4,49	-0,64
**RASGRP4**	2,42	3,06	-0,64
**HES2**	5,35	5,99	-0,64
**CIDEC**	2,42	3,06	-0,64
**GCM1**	2,42	3,06	-0,64
**GBP2**	2,42	3,06	-0,64
**LRRN4CL**	2,42	3,06	-0,64
**S100A14**	2,42	3,06	-0,64
**SCRT1**	2,42	3,06	-0,64
**OR52B6**	2,42	3,06	-0,64
**GRIP1**	9,85	10,49	-0,64
**FBXO17**	2,56	3,20	-0,64
**CCAR2**	12,15	12,79	-0,64
**COL25A1**	3,80	4,43	-0,64
**KATNAL1**	10,65	11,29	-0,64

**HGS**	12,17	12,80	-0,64
**GADD45GIP1**	11,62	12,26	-0,64
**FERMT2**	4,31	4,94	-0,64
**ENAH**	10,41	11,05	-0,63
**PCDHGC3**	10,75	11,38	-0,63
**PCDHB9**	5,74	6,37	-0,63
**EHBP1L1**	12,25	12,89	-0,63
**CABP7**	3,57	4,20	-0,63
**SOWAHC**	4,20	4,83	-0,63
**FUT6**	1,75	2,38	-0,63
**AP002373.1**	7,04	7,67	-0,63
**ZNF784**	8,22	8,85	-0,63
**MZT2A**	11,30	11,93	-0,63
**DPP7**	12,59	13,22	-0,63
**CCL2**	2,43	3,05	-0,63
**AC020915.4**	8,45	9,07	-0,63
**DDN**	10,72	11,35	-0,63
**C19orf73**	5,44	6,07	-0,63
**EFCAB3**	4,93	5,55	-0,62
**MDGA1**	8,60	9,22	-0,62
**EXOG**	10,67	11,29	-0,62
**TSPEAR**	4,12	4,74	-0,62
**PDE2A**	4,54	5,16	-0,62
**ZNF517**	7,04	7,66	-0,62
**TRIM9**	7,71	8,33	-0,62
**PIN1**	11,20	11,82	-0,62
**ANK1**	8,76	9,38	-0,62
**ARMCX5-GPRASP2**	6,05	6,67	-0,62
**DNAAF1**	2,39	3,01	-0,62
**GALNTL6**	6,11	6,73	-0,62
**TSR2**	11,24	11,86	-0,62
**EXOC3L1**	5,86	6,48	-0,61
**GPR37L1**	4,82	5,43	-0,61
**PET100**	10,80	11,41	-0,61
**ZNF836**	6,16	6,77	-0,61
**CTSG**	7,91	8,52	-0,61
**RIMBP3B**	7,64	8,25	-0,61
**PANK4**	10,03	10,64	-0,61
**TRPV5**	3,23	3,84	-0,61
**TMEM134**	8,58	9,19	-0,61
**ULK4**	8,35	8,96	-0,61
**SCRN2**	8,80	9,41	-0,61
**ARHGEF38**	2,09	2,70	-0,61
**RDH12**	2,09	2,70	-0,61
**SLC6A20**	2,09	2,70	-0,61
**ONECUT1**	2,09	2,70	-0,61
**SYCE1**	2,09	2,70	-0,61
**LIN52**	2,09	2,70	-0,61
**FRMD5**	6,39	7,00	-0,61
**CEP250**	11,91	12,52	-0,61
**STK32C**	6,94	7,55	-0,61
**AMPD3**	8,80	9,41	-0,61
**MRPS26**	11,33	11,94	-0,61
**FAM207A**	10,80	11,41	-0,61
**AC108488.2**	8,49	9,09	-0,60
**SHOC1**	3,28	3,88	-0,60

**AQP2**	3,28	3,88	-0,60
**H2BC10**	3,28	3,88	-0,60
**RPAP1**	11,55	12,16	-0,60
**STXBP5L**	9,31	9,91	-0,60
**FRG2C**	4,37	4,97	-0,60
**ABHD14A-ACY1**	6,58	7,19	-0,60
**PRSS27**	4,70	5,31	-0,60
**PGPEP1**	7,38	7,98	-0,60
**FBP1**	6,25	6,85	-0,60
**C1orf35**	10,21	10,81	-0,60
**PPP1R9B**	12,21	12,81	-0,60
**PFDN6**	10,87	11,47	-0,60
**NAB2**	9,79	10,39	-0,60
**RAPGEF3**	6,31	6,91	-0,60
**HCLS1**	12,90	13,50	-0,60
**MYT1**	5,14	5,74	-0,60
**FOXD4L1**	5,37	5,97	-0,60
**CSK**	9,67	10,27	-0,60
**TRPC4**	2,03	2,63	-0,60
**AKAP3**	5,88	6,48	-0,60
**CDAN1**	11,02	11,61	-0,60
**AC107871.1**	6,72	7,32	-0,59
**GRIN3A**	4,64	5,24	-0,59
**SEPTIN10**	5,31	5,90	-0,59
**MOV10**	10,02	10,61	-0,59
**AL669918.1**	8,55	9,14	-0,59
**HS6ST2**	9,46	10,05	-0,59
**CC2D2A**	3,75	4,34	-0,59
**GARNL3**	7,23	7,82	-0,59
**CNTNAP2**	5,12	5,71	-0,59
**ARL10**	10,66	11,26	-0,59
**PIP4P1**	10,44	11,03	-0,59
**SPC24**	7,86	8,45	-0,59
**NXPH3**	4,87	5,46	-0,59
**CDADC1**	9,59	10,18	-0,59
**AC242842.3**	5,90	6,49	-0,59
**CHPF**	8,62	9,21	-0,59
**PEX10**	9,63	10,22	-0,59
**NR1H3**	8,03	8,62	-0,59
**SIX5**	8,67	9,26	-0,59
**DDX41**	12,56	13,15	-0,59
**CPXM2**	5,07	5,66	-0,59
**STIMATE-MUSTN1**	6,69	7,28	-0,59
**CHST12**	11,38	11,97	-0,59
**Z83844.2**	8,21	8,80	-0,58
**DLC1**	6,91	7,49	-0,58
**AC096887.1**	2,90	3,49	-0,58
**CEP63**	6,47	7,06	-0,58
**NME3**	7,70	8,28	-0,58
**AC017083.3**	6,08	6,66	-0,58
**SWSAP1**	7,87	8,45	-0,58
**CARD9**	6,57	7,16	-0,58
**C22orf15**	6,73	7,31	-0,58
**BORCS6**	8,98	9,56	-0,58
**PNOC**	3,45	4,03	-0,58
**PPAN**	11,44	12,02	-0,58

**ASB14**	6,23	6,81	-0,58
**ARHGAP22**	6,46	7,04	-0,58
**TEN1**	8,69	9,27	-0,58
**PAM**	9,03	9,61	-0,58
**PIGV**	6,94	7,52	-0,58
**CREBRF**	8,94	9,51	-0,58
**VSTM4**	4,51	5,09	-0,58
**LCT**	5,20	5,78	-0,58
**VSIG2**	3,93	4,51	-0,58
**RNASEH2C**	11,96	12,54	-0,58
**TBKBP1**	8,72	9,30	-0,58
**DUSP23**	6,88	7,46	-0,58
**PFDN2**	12,31	12,89	-0,58
**PACSIN3**	11,17	11,75	-0,58
**PRRG2**	4,88	5,45	-0,58
**RDM1**	6,52	7,10	-0,58
**BCL2L2**	10,25	10,82	-0,58
**COL9A3**	7,70	8,27	-0,57
**TMEM255B**	5,54	6,12	-0,57
**FOXD4L6**	4,12	4,69	-0,57
**KLHDC7B**	4,99	5,56	-0,57
**SCX**	6,27	6,84	-0,57
**ZNF775**	8,67	9,24	-0,57
**REX1BD**	9,66	10,23	-0,57
**RCAN2**	4,36	4,93	-0,57
**AC025165.6**	6,08	6,65	-0,57
**C8G**	3,58	4,15	-0,57
**ZNF670-ZNF695**	6,53	7,10	-0,57
**AMBRA1**	10,36	10,93	-0,57
**AC015813.2**	9,82	10,39	-0,57
**SLC25A34**	6,48	7,05	-0,57
**KDM6A**	11,55	12,11	-0,57
**TRGV3**	5,12	5,69	-0,57
**PCSK7**	11,21	11,77	-0,57
**OGFR**	11,67	12,24	-0,57
**SCAF1**	12,12	12,69	-0,57
**RAB6B**	6,56	7,12	-0,57
**TMEM86B**	7,70	8,26	-0,56
**AC010422.3**	7,82	8,38	-0,56
**RASL10B**	10,96	11,52	-0,56
**TBXAS1**	6,79	7,35	-0,56
**ZBTB22**	8,41	8,97	-0,56
**FSTL4**	3,66	4,23	-0,56
**SLC12A8**	5,61	6,17	-0,56
**MAPK8IP1**	6,10	6,66	-0,56
**SLFNL1**	8,44	9,00	-0,56
**GJA4**	6,01	6,57	-0,56
**CDC42EP1**	2,91	3,47	-0,56
**WDR73**	11,23	11,79	-0,56
**ADH6**	2,12	2,68	-0,56
**RNF220**	12,22	12,78	-0,56
**DNAJC6**	8,72	9,27	-0,56
**SLC25A16**	10,45	11,00	-0,56
**ARHGAP6**	1,67	2,22	-0,56
**MYBPC2**	1,67	2,22	-0,56
**ZDHHC15**	1,67	2,22	-0,56

**ZBTB16**	1,67	2,22	-0,56
**PLA2G2D**	1,67	2,22	-0,56
**TEX11**	1,67	2,22	-0,56
**FIBCD1**	1,67	2,22	-0,56
**COL2A1**	1,67	2,22	-0,56
**OCSTAMP**	1,67	2,22	-0,56
**ADORA1**	1,67	2,22	-0,56
**FBLN2**	1,67	2,22	-0,56
**C15orf48**	1,67	2,22	-0,56
**TCP10L2**	1,67	2,22	-0,56
**C19orf33**	1,67	2,22	-0,56
**LRP1B**	1,67	2,22	-0,56
**BFSP2**	1,67	2,22	-0,56
**NWD2**	1,67	2,22	-0,56
**ABRA**	1,67	2,22	-0,56
**P2RY2**	1,67	2,22	-0,56
**OR5AN1**	1,67	2,22	-0,56
**TDRP**	1,67	2,22	-0,56
**TMEM132E**	1,67	2,22	-0,56
**ADGRG3**	1,67	2,22	-0,56
**OR10H1**	1,67	2,22	-0,56
**C1QL4**	1,67	2,22	-0,56
**OR7D2**	1,67	2,22	-0,56
**H4C6**	1,67	2,22	-0,56
**ASB16**	7,67	8,23	-0,55
**PORCN**	7,62	8,18	-0,55
**ZSCAN2**	10,84	11,40	-0,55
**PTRH1**	8,18	8,74	-0,55
**HAGHL**	8,47	9,03	-0,55
**MRM1**	10,43	10,99	-0,55
**PAOX**	8,26	8,81	-0,55
**ZGPAT**	9,99	10,54	-0,55
**IRF2BP1**	11,23	11,78	-0,55
**AC010980.1**	5,61	6,16	-0,55
**SLC66A1**	8,07	8,62	-0,55
**TMEM150A**	6,89	7,44	-0,55
**CNTF**	6,50	7,05	-0,55
**GOLGA7**	11,18	11,73	-0,55
**SLC23A3**	4,36	4,91	-0,55
**CYTH2**	11,77	12,32	-0,55
**LIPT2**	8,21	8,75	-0,55
**CLEC11A**	12,60	13,14	-0,55
**SLX1B**	10,51	11,06	-0,55
**PIN4**	9,94	10,48	-0,55
**ACTA1**	4,29	4,83	-0,55
**PAQR9**	4,29	4,83	-0,55
**BATF3**	8,24	8,79	-0,55
**IRF9**	8,19	8,74	-0,55
**FAM240C**	4,56	5,11	-0,55
**MZB1**	12,14	12,68	-0,55
**CCDC183**	4,45	5,00	-0,55
**DHRS12**	6,61	7,16	-0,54
**AC010422.8**	10,43	10,98	-0,54
**MVD**	10,52	11,07	-0,54
**DRD4**	3,92	4,47	-0,54
**DUSP26**	5,44	5,99	-0,54

**AC011511.1**	5,44	5,99	-0,54
**MIB2**	9,63	10,17	-0,54
**FCSK**	9,18	9,72	-0,54
**CAPS**	7,75	8,29	-0,54
**ZNF264**	10,82	11,36	-0,54
**VWA5B2**	5,70	6,24	-0,54
**AKIP1**	10,53	11,07	-0,54
**TTN**	8,74	9,28	-0,54
**PLB1**	5,99	6,53	-0,54
**ARL6IP1**	14,29	14,83	-0,54
**MRC1**	6,05	6,59	-0,54
**TTLL2**	1,90	2,44	-0,54
**RAB11FIP4**	11,71	12,25	-0,54
**VWA5A**	5,90	6,43	-0,54
**B9D2**	7,79	8,33	-0,54
**MRAS**	6,43	6,97	-0,54
**GADD45B**	8,71	9,25	-0,54
**TPGS1**	9,20	9,74	-0,54
**GLIPR1**	7,01	7,54	-0,54
**PDAP1**	12,94	13,47	-0,54
**GLI4**	9,96	10,50	-0,54
**FAM20C**	8,49	9,02	-0,54
**NPW**	8,57	9,11	-0,54
**IGLC2**	7,12	7,66	-0,54
**PLPP7**	5,13	5,66	-0,53
**SLC25A26**	8,94	9,48	-0,53
**IFI27L2**	8,22	8,76	-0,53
**FHDC1**	4,20	4,74	-0,53
**MAN1C1**	6,52	7,05	-0,53
**ITPKA**	7,90	8,44	-0,53
**SIRT3**	9,52	10,05	-0,53
**CREB3L3**	3,88	4,42	-0,53
**GRAMD4**	12,02	12,56	-0,53
**IQCH**	5,96	6,49	-0,53
**FFAR1**	6,63	7,16	-0,53
**SLC9A1**	10,24	10,77	-0,53
**UNC5B**	7,49	8,02	-0,53
**EFR3B**	5,32	5,85	-0,53
**BEST2**	2,30	2,83	-0,53
**PBXIP1**	11,18	11,71	-0,53
**PTX4**	4,87	5,40	-0,53
**FAAP20**	10,28	10,81	-0,53
**NKAIN3**	6,54	7,07	-0,53
**HSD17B6**	7,32	7,84	-0,53
**POR**	11,11	11,64	-0,53
**HEATR3**	10,34	10,87	-0,53
**TBL1X**	10,22	10,75	-0,53
**TNIP2**	10,69	11,21	-0,53
**ATP8A2**	7,69	8,21	-0,53
**RBM11**	3,82	4,34	-0,53
**B3GALT4**	6,21	6,74	-0,53
**TRGV9**	5,03	5,55	-0,52
**VDR**	8,48	9,01	-0,52
**FAM72C**	6,27	6,80	-0,52
**VWA1**	8,60	9,13	-0,52
**LARP6**	9,43	9,95	-0,52

**ARMCX5**	8,64	9,17	-0,52
**PRADC1**	9,37	9,89	-0,52
**AC068580.4**	5,08	5,60	-0,52
**LMTK3**	6,39	6,92	-0,52
**CYP3A43**	1,90	2,43	-0,52
**SLC52A1**	2,69	3,21	-0,52
**IGHV3-53**	2,69	3,21	-0,52
**TGFBR3L**	2,69	3,21	-0,52
**AF196969.1**	2,69	3,21	-0,52
**BCL7C**	9,81	10,33	-0,52
**C8orf82**	9,56	10,08	-0,52
**SDF2L1**	10,98	11,50	-0,52
**NCR3**	2,40	2,92	-0,52
**EFNA3**	6,77	7,29	-0,52
**IRAK2**	6,90	7,42	-0,52
**UPK3BL2**	9,36	9,88	-0,52
**YPEL4**	6,02	6,54	-0,52
**SPAG7**	10,74	11,26	-0,52
**NUGGC**	5,29	5,81	-0,52
**INHBC**	5,64	6,16	-0,52
**CCDC137**	11,47	11,98	-0,52
**PPM1N**	5,55	6,07	-0,52
**FMN2**	5,41	5,92	-0,52
**DISP3**	6,48	7,00	-0,52
**GIPR**	3,53	4,05	-0,52
**TSSK6**	6,29	6,81	-0,52
**GPR135**	4,37	4,88	-0,51
**TUBG2**	7,83	8,34	-0,51
**GATAD2A**	12,49	13,00	-0,51
**DAPK3**	10,01	10,53	-0,51
**TYMP**	5,16	5,67	-0,51
**TACR2**	2,38	2,89	-0,51
**MMP15**	5,25	5,76	-0,51
**TIMP3**	4,58	5,09	-0,51
**KCNF1**	3,28	3,79	-0,51
**CEBPD**	3,28	3,79	-0,51
**PRR23D2**	3,28	3,79	-0,51
**PRAM1**	5,37	5,88	-0,51
**CCR6**	5,88	6,39	-0,51
**MMP24**	4,76	5,27	-0,51
**GREM2**	8,21	8,72	-0,51
**CLTB**	11,10	11,61	-0,51
**GPC2**	9,88	10,39	-0,51
**AL163636.1**	4,93	5,43	-0,51
**C7orf25**	5,48	5,99	-0,51
**SLC9A5**	7,15	7,65	-0,51
**GP1BA**	6,78	7,29	-0,51
**ARFGAP1**	11,91	12,41	-0,50
**ARHGAP45**	12,52	13,03	-0,50
**EPOP**	10,96	11,47	-0,50
**CBX8**	8,82	9,33	-0,50
**LAMTOR4**	9,25	9,75	-0,50
**KLK1**	6,94	7,45	-0,50
**DAAM2**	6,83	7,34	-0,50
**OTUD7B**	5,93	6,43	-0,50
**EPS8**	8,20	8,71	-0,50

**RASGEF1C**	4,02	4,53	-0,50
**SCG2**	6,00	6,51	-0,50
**CTU1**	9,21	9,71	-0,50
**CC2D1A**	10,86	11,36	-0,50
**RAF1**	12,19	12,69	-0,50
**ARHGAP4**	11,74	12,24	-0,50
**TMC2**	6,08	6,58	-0,50
**AC074143.1**	6,14	6,65	-0,50
**RAB11FIP3**	12,05	12,55	-0,50
**PPP1R15A**	10,30	10,80	-0,50
**UBXN1**	11,94	12,44	-0,50
**POLD4**	8,44	8,94	-0,50
**RDH10**	9,10	9,60	-0,50
**NPEPL1**	9,31	9,81	-0,50
**SAMD1**	12,10	12,60	-0,50
**NTRK1**	6,44	6,94	-0,50
**GALNT18**	7,69	8,19	-0,50
**LRRC37A3**	8,13	8,63	-0,50
**RAB30**	8,58	9,07	-0,50
**NUDC**	13,16	13,66	-0,50
**ADAMTS2**	3,38	3,87	-0,50
**CHCHD6**	9,06	9,56	-0,50
**FAM71D**	5,07	5,56	-0,50
**TMEM160**	10,20	10,70	-0,50
**SCARA5**	1,92	2,41	-0,50
**HNRNPA3**	16,30	16,79	-0,49
**DEF8**	11,46	11,96	-0,49
**MOK**	7,14	7,63	-0,49
**C17orf100**	5,79	6,29	-0,49
**SPIRE2**	5,93	6,42	-0,49
**KREMEN2**	7,84	8,34	-0,49
**DEDD2**	9,66	10,15	-0,49
**B3GAT2**	6,83	7,32	-0,49
**NFE2L1**	12,63	13,12	-0,49
**NOP2**	12,60	13,10	-0,49
**IDNK**	7,90	8,39	-0,49
**PRICKLE4**	5,99	6,48	-0,49
**RCC1**	12,91	13,40	-0,49
**LMNB2**	14,03	14,52	-0,49
**SPIN3**	3,97	4,46	-0,49
**C15orf40**	10,99	11,48	-0,49
**CCDC136**	10,23	10,72	-0,49
**MICA**	9,16	9,65	-0,49
**SPDYE1**	6,00	6,49	-0,49
**PIEZO2**	6,42	6,91	-0,49
**MYEF2**	10,47	10,95	-0,49
**ASB2**	4,11	4,60	-0,49
**CADM4**	10,26	10,75	-0,49
**ACTL8**	4,98	5,46	-0,49
**SUN3**	2,50	2,98	-0,49
**UBE2G1**	10,59	11,08	-0,48
**DOCK9**	8,03	8,51	-0,48
**METRN**	9,02	9,50	-0,48
**MTG2**	10,48	10,96	-0,48
**RRS1**	12,27	12,75	-0,48
**ZNF41**	8,67	9,16	-0,48

**TMEFF1**	9,23	9,71	-0,48
**TEDC1**	9,64	10,12	-0,48
**ZNF783**	10,46	10,94	-0,48
**RNF222**	3,57	4,05	-0,48
**PRPF40B**	8,71	9,19	-0,48
**JMJD7**	7,79	8,27	-0,48
**C6orf47**	9,48	9,96	-0,48
**HSD17B1**	5,84	6,32	-0,48
**PRDX5**	11,51	11,99	-0,48
**CACNB4**	6,11	6,58	-0,48
**OR2W3**	5,21	5,69	-0,48
**SLC4A3**	3,11	3,59	-0,48
**REM2**	4,63	5,10	-0,48
**CLDND2**	3,95	4,43	-0,48
**MYL6B**	11,39	11,87	-0,48
**TMEM52**	6,98	7,46	-0,48
**POLR2F**	11,59	12,07	-0,48
**LTK**	6,98	7,45	-0,48
**ATG9B**	7,53	8,01	-0,48
**TRNP1**	4,70	5,18	-0,47
**VGF**	6,14	6,62	-0,47
**MICOS13**	9,49	9,97	-0,47
**B3GNT9**	6,08	6,55	-0,47
**EFHD2**	12,27	12,74	-0,47
**CYP27B1**	6,89	7,36	-0,47
**DAPK1**	5,67	6,15	-0,47
**ENTPD7**	6,91	7,38	-0,47
**PPCDC**	8,74	9,22	-0,47
**KRI1**	11,86	12,33	-0,47
**EXO5**	8,28	8,75	-0,47
**MMP16**	5,41	5,88	-0,47
**JCAD**	7,40	7,87	-0,47
**INKA1**	9,50	9,97	-0,47
**TBX6**	6,62	7,09	-0,47
**KLHL5**	10,27	10,74	-0,47
**NRXN2**	6,30	6,77	-0,47
**DNAJC22**	2,42	2,89	-0,47
**ARL4A**	2,42	2,89	-0,47
**ADAMTS8**	2,42	2,89	-0,47
**GRPR**	2,42	2,89	-0,47
**ANGPTL7**	2,42	2,89	-0,47
**INSM1**	2,42	2,89	-0,47
**NPIPB15**	2,42	2,89	-0,47
**CFB**	2,42	2,89	-0,47
**TG**	4,12	4,59	-0,47
**TRAV29DV5**	5,77	6,24	-0,47
**HLA-G**	3,54	4,01	-0,47
**TRAPPC2L**	11,02	11,49	-0,47
**ENHO**	4,37	4,83	-0,47
**PTPRC**	4,86	5,32	-0,47
**LRRC45**	10,79	11,26	-0,47
**MEGF6**	11,03	11,50	-0,47
**PDZRN4**	6,54	7,01	-0,47
**ROMO1**	10,55	11,02	-0,47
**HINT2**	8,87	9,34	-0,47
**KIF1A**	2,42	2,89	-0,47

NKAIN1	5,90	6,37	-0,47
TMEM63B	9,14	9,61	-0,47
ARHGEF40	9,75	10,22	-0,47
GTPBP6	11,35	11,82	-0,47
FCHSD1	10,18	10,65	-0,47
HSPB6	4,12	4,58	-0,47
FIZ1	10,43	10,90	-0,47
FBRS	12,03	12,50	-0,47
PHF21B	4,92	5,38	-0,47
TINCR	6,17	6,64	-0,47
MTM1	9,61	10,08	-0,47
ARRDC1	10,00	10,46	-0,46
HIC1	7,04	7,51	-0,46
MAGIX	5,35	5,82	-0,46
GDPD5	9,40	9,86	-0,46
MAFK	11,15	11,61	-0,46
GPATCH3	9,89	10,35	-0,46
GAR1	10,83	11,29	-0,46
ZNF747	9,97	10,43	-0,46
MYO15B	11,31	11,77	-0,46
PHF8	10,53	10,99	-0,46
RPL21	13,70	14,16	-0,46
ZNF442	6,42	6,89	-0,46
CENPX	11,18	11,64	-0,46
EGR2	6,85	7,31	-0,46
VPREB1	12,40	12,86	-0,46
IGSF11	1,93	2,39	-0,46
LCAT	7,20	7,67	-0,46
GJD3	6,81	7,27	-0,46
STAC3	7,60	8,06	-0,46
ELMOD1	4,24	4,70	-0,46
ANO8	9,53	9,99	-0,46
WFIKKN1	3,82	4,27	-0,46
GPR45	3,82	4,27	-0,46
FAM209B	3,82	4,27	-0,46
ERP29	12,44	12,90	-0,46
GLMP	4,97	5,43	-0,46
FGFBP3	8,78	9,24	-0,46
DNAH1	8,17	8,63	-0,46
HNRNPH3	14,19	14,65	-0,46
ALDH16A1	11,26	11,72	-0,46
IPO4	12,47	12,93	-0,46
SYP	6,23	6,69	-0,46
ATP13A4	5,46	5,92	-0,46
ADRA2C	6,27	6,73	-0,46
GAREM2	9,23	9,69	-0,46
PRRT4	6,40	6,86	-0,46
GOLGB1	11,83	12,29	-0,46
USP19	9,51	9,96	-0,46
UBE2S	13,40	13,85	-0,46
ZNF444	10,70	11,16	-0,46
NNAT	4,23	4,69	-0,46
REEP1	4,98	5,43	-0,46
ALG1L2	4,98	5,43	-0,46
RHOT2	12,52	12,97	-0,46
PIP5K1B	7,20	7,65	-0,46

**ARFRP1**	10,80	11,25	-0,45
**HSPA1B**	11,88	12,34	-0,45
**ALKBH2**	9,35	9,80	-0,45
**SLC25A25**	10,87	11,32	-0,45
**TMEM39A**	10,90	11,35	-0,45
**TMEM143**	8,66	9,11	-0,45
**CBR1**	10,75	11,20	-0,45
**ASRGL1**	7,65	8,10	-0,45
**CCDC189**	7,27	7,72	-0,45
**C2orf92**	6,18	6,63	-0,45
**FAM122C**	8,69	9,14	-0,45
**EMILIN1**	12,78	13,24	-0,45
**ELMOD3**	9,61	10,06	-0,45
**MANSC4**	3,43	3,88	-0,45
**FAM219A**	11,00	11,45	-0,45
**TMC4**	4,23	4,68	-0,45
**RAI1**	11,87	12,32	-0,45
**CDH11**	4,14	4,59	-0,45
**GRAPL**	6,22	6,67	-0,45
**TMEM185A**	8,97	9,42	-0,45
**RPS6KA4**	11,09	11,54	-0,45
**TLCD4-RWDD3**	6,79	7,24	-0,45
**SPIN2A**	5,30	5,74	-0,45
**ZSWIM8**	10,84	11,28	-0,45
**ZNF787**	10,05	10,49	-0,45
**CEP295NL**	4,64	5,09	-0,45
**WDR83**	9,48	9,93	-0,45
**FGFR3**	3,55	4,00	-0,45
**ESRRA**	10,06	10,51	-0,45
**FBXO44**	9,58	10,02	-0,45
**TAC3**	1,37	1,82	-0,45
**PPP1R37**	10,21	10,65	-0,45
**LSR**	8,02	8,46	-0,45
**ALG12**	10,72	11,16	-0,45
**GASK1A**	1,84	2,29	-0,45
**KCNMB3**	7,27	7,71	-0,45
**DLG4**	11,07	11,52	-0,45
**IFRD2**	8,23	8,68	-0,45
**MRPL53**	10,13	10,57	-0,45
**PXMP4**	6,67	7,12	-0,45
**GNPTG**	8,85	9,30	-0,44
**PPP1R14A**	10,24	10,68	-0,44
**TRMT61A**	11,04	11,48	-0,44
**ZNF799**	7,32	7,76	-0,44
**REXO4**	11,64	12,09	-0,44
**PSMD9**	11,75	12,19	-0,44
**ATP2A1**	7,75	8,19	-0,44
**BAD**	9,92	10,36	-0,44
**HMG20B**	11,59	12,04	-0,44
**SLC51A**	4,38	4,83	-0,44
**RGS3**	8,78	9,22	-0,44
**UBXN11**	9,60	10,04	-0,44
**ST8SIA6**	5,64	6,09	-0,44
**FILIP1**	6,12	6,56	-0,44
**AC003005.1**	6,48	6,92	-0,44
**PDF**	10,75	11,19	-0,44

**EXOSC4**	8,92	9,36	-0,44
**SGMS2**	1,06	1,50	-0,44
**AC005551.1**	1,06	1,50	-0,44
**DKK4**	1,06	1,50	-0,44
**IL37**	1,06	1,50	-0,44
**PPP1R1A**	1,06	1,50	-0,44
**PDE6H**	1,06	1,50	-0,44
**TRIM50**	1,06	1,50	-0,44
**CT55**	1,06	1,50	-0,44
**SLC26A5**	1,06	1,50	-0,44
**SYT9**	1,06	1,50	-0,44
**GDPD4**	1,06	1,50	-0,44
**SLC9A4**	1,06	1,50	-0,44
**TLCD2**	1,06	1,50	-0,44
**THEM5**	1,06	1,50	-0,44
**IFITM5**	1,06	1,50	-0,44
**MINDY4B**	1,06	1,50	-0,44
**AL365205.1**	9,35	9,79	-0,44
**CHAC1**	8,56	9,00	-0,44
**NGFR**	6,60	7,04	-0,44
**ALX3**	6,60	7,04	-0,44
**EIF5A2**	7,47	7,91	-0,44
**FAM155A**	4,76	5,20	-0,44
**TRAPPC6A**	9,64	10,07	-0,44
**ERBB2**	7,02	7,45	-0,44
**MSH5**	10,95	11,38	-0,44
**ARHGAP9**	10,08	10,51	-0,44
**TMEM139**	5,03	5,46	-0,44
**TP53I13**	11,00	11,44	-0,44
**AL391650.1**	6,64	7,08	-0,44
**SOCS7**	11,68	12,12	-0,44
**AC114490.1**	7,77	8,20	-0,44
**ZC2HC1C**	5,02	5,45	-0,44
**MYLK4**	7,90	8,34	-0,44
**C17orf67**	8,10	8,53	-0,43
**STAT4**	5,25	5,68	-0,43
**DPM3**	9,36	9,79	-0,43
**ADM2**	8,70	9,13	-0,43
**FXR2**	11,24	11,68	-0,43
**RSPO4**	2,91	3,35	-0,43
**TFCP2L1**	2,91	3,35	-0,43
**VSNL1**	2,91	3,35	-0,43
**PET117**	8,83	9,26	-0,43
**POLR2L**	11,88	12,31	-0,43
**ZNF720**	7,58	8,01	-0,43
**SLC44A4**	3,70	4,13	-0,43
**CACNA2D2**	4,20	4,64	-0,43
**ECT2L**	4,20	4,64	-0,43
**HEXD**	10,07	10,50	-0,43
**LIMD2**	11,22	11,66	-0,43
**WDR74**	11,80	12,23	-0,43
**LAD1**	3,35	3,78	-0,43
**CDC34**	11,14	11,57	-0,43
**KBTBD13**	5,82	6,25	-0,43
**PCED1B**	5,41	5,84	-0,43
**ICAM3**	7,46	7,89	-0,43

**PNKP**	10,41	10,84	-0,43
**NFKBIB**	10,37	10,80	-0,43
**EDF1**	12,73	13,16	-0,43
**NDUFS6**	12,22	12,65	-0,43
**TPK1**	6,94	7,36	-0,43
**THEMIS2**	8,77	9,19	-0,43
**AP002748.5**	6,23	6,66	-0,43
**TIFAB**	6,98	7,41	-0,43
**SLC5A10**	6,63	7,06	-0,43
**CDK11A**	10,82	11,25	-0,43
**CTU2**	10,99	11,42	-0,43
**FUS**	15,10	15,52	-0,43
**NCOR2**	12,98	13,41	-0,43
**MAP3K12**	9,59	10,02	-0,42
**NELFE**	11,70	12,12	-0,42
**DOK4**	9,00	9,42	-0,42
**TSPAN11**	4,45	4,88	-0,42
**LDHD**	1,07	1,49	-0,42
**ZNF781**	7,20	7,62	-0,42
**SUMF1**	8,78	9,20	-0,42
**MRPL24**	11,85	12,27	-0,42
**MAMDC4**	8,49	8,91	-0,42
**ZSWIM9**	8,79	9,21	-0,42
**ZNF646**	11,67	12,09	-0,42
**LCN10**	9,40	9,82	-0,42
**LIPG**	4,37	4,79	-0,42
**AC021087.5**	8,09	8,52	-0,42
**RAB38**	7,10	7,52	-0,42
**EMC10**	8,20	8,62	-0,42
**ARSG**	7,67	8,09	-0,42
**PLEKHH3**	7,67	8,09	-0,42
**FARP1**	10,21	10,64	-0,42
**BEND6**	8,14	8,56	-0,42
**NME1-NME2**	9,05	9,47	-0,42
**SKAP1**	6,08	6,50	-0,42
**TUBB2A**	8,62	9,04	-0,42
**TIGIT**	1,58	2,00	-0,42
**GPATCH2L**	12,54	12,96	-0,42
**FRMD4B**	11,69	12,11	-0,42
**GTF2E1**	10,76	11,18	-0,42
**FOSL1**	7,76	8,18	-0,42
**SLC39A7**	11,61	12,03	-0,42
**ATP5ME**	11,50	11,92	-0,42
**NPM2**	5,90	6,31	-0,42
**GDF1**	3,92	4,34	-0,42
**SCARF2**	3,92	4,34	-0,42
**PCP2**	2,60	3,02	-0,42
**TRADD**	9,25	9,67	-0,42
**PTGES2**	11,65	12,07	-0,42
**PNKD**	9,98	10,40	-0,42
**LYPD5**	5,91	6,32	-0,42
**COL16A1**	6,43	6,84	-0,42
**METRNL**	9,37	9,79	-0,42
**CLASRP**	11,46	11,88	-0,42
**MYLK**	9,87	10,28	-0,42
**GPX3**	6,41	6,83	-0,42

CD302	6,18	6,59	-0,42
ASCC2	12,11	12,53	-0,42
ORMDL2	10,04	10,46	-0,42
DOHH	10,07	10,49	-0,42
TUBB3	11,14	11,56	-0,41
RTL8B	5,16	5,58	-0,41
NDUFB1	10,60	11,01	-0,41
KRT10	10,59	11,01	-0,41
PDE6B	8,32	8,73	-0,41
ZNF316	10,98	11,39	-0,41
ZC3H10	10,18	10,59	-0,41
ACSF3	10,87	11,28	-0,41
ADAMTS13	7,15	7,56	-0,41
SLC12A9	11,29	11,71	-0,41
TP53BP1	12,76	13,17	-0,41
RASA4	12,05	12,46	-0,41
TAL2	3,28	3,69	-0,41
BBS5	8,33	8,74	-0,41
PES1	13,26	13,67	-0,41
WNT2B	6,53	6,94	-0,41
SNX32	8,01	8,42	-0,41
C15orf61	9,33	9,75	-0,41
SURF6	12,09	12,51	-0,41
RMDN3	10,43	10,85	-0,41
JAG1	7,87	8,28	-0,41
ITGA3	5,25	5,66	-0,41
FAM81A	9,41	9,82	-0,41
MGAT3	7,64	8,05	-0,41
TLCD3A	9,87	10,28	-0,41
PROB1	10,89	11,30	-0,41
ZNF408	9,54	9,95	-0,41
SLA	6,35	6,76	-0,41
INF2	12,56	12,97	-0,41
AXIN1	11,63	12,04	-0,41
C2CD6	4,12	4,53	-0,41
NUDT1	11,20	11,61	-0,41
SHISA6	4,30	4,71	-0,41
LYRM7	12,13	12,54	-0,41
ATAD3B	11,96	12,37	-0,41
MPIG6B	3,87	4,28	-0,41
RABEP2	11,04	11,44	-0,41
IRF5	7,13	7,54	-0,41
GSDMD	11,69	12,10	-0,41
RBM43	8,46	8,87	-0,41
NAT14	9,53	9,94	-0,41
OSMR	3,14	3,55	-0,41
PNRC1	10,18	10,59	-0,41
SLC25A29	12,78	13,19	-0,41
DHRS4	10,57	10,98	-0,41
TNFSF13	8,00	8,40	-0,41
SPRY3	6,89	7,29	-0,40
TMA7	13,11	13,51	-0,40
SENP3-EIF4A1	11,06	11,47	-0,40
AZIN2	6,31	6,71	-0,40
LRFN1	9,47	9,87	-0,40
NINL	11,65	12,06	-0,40

**AC009690.1**	7,80	8,20	-0,40
**NOL12**	11,37	11,77	-0,40
**UROC1**	2,54	2,94	-0,40
**MT2A**	9,34	9,74	-0,40
**SEMA3E**	4,76	5,16	-0,40
**AL358472.6**	8,01	8,41	-0,40
**CGN**	8,22	8,62	-0,40
**PMFBP1**	7,62	8,02	-0,40
**RUVBL2**	13,30	13,70	-0,40
**ALMS1**	11,18	11,58	-0,40
**SLC2A4**	6,22	6,62	-0,40
**MTFP1**	11,17	11,58	-0,40
**BCL9L**	6,79	7,19	-0,40
**CBLN3**	6,63	7,03	-0,40
**RBM17**	13,12	13,52	-0,40
**PRELID3A**	9,85	10,25	-0,40
**TNNI3**	4,83	5,23	-0,40
**ELL**	10,10	10,50	-0,40
**SNRNP35**	9,38	9,78	-0,40
**PHRF1**	12,34	12,74	-0,40
**ZHX3**	8,79	9,19	-0,40
**FCRL5**	4,19	4,59	-0,40
**RIOX1**	11,34	11,74	-0,40
**ABCG2**	5,77	6,17	-0,40
**AL590560.2**	3,57	3,97	-0,40
**AC027796.3**	5,21	5,61	-0,40
**SH2B1**	11,46	11,86	-0,40
**HNRNPAB**	14,72	15,12	-0,40
**ODF2**	9,84	10,24	-0,40
**A1BG**	9,50	9,89	-0,40
**RBKS**	6,19	6,59	-0,40
**SLC39A11**	7,90	8,30	-0,40
**NCAM2**	6,83	7,23	-0,40
**KDM8**	8,74	9,14	-0,40
**RTP5**	6,44	6,83	-0,40
**NEXMIF**	4,40	4,80	-0,40
**RXRB**	11,27	11,66	-0,40
**TRIM16**	9,53	9,93	-0,40
**SYNC**	10,38	10,77	-0,40
**KCNK7**	4,81	5,20	-0,40
**LARGE2**	9,99	10,39	-0,40
**RNF121**	9,38	9,77	-0,40
**NEK10**	5,15	5,54	-0,40
**KMT2B**	11,81	12,21	-0,40
**CIART**	5,85	6,25	-0,40
**AGER**	7,97	8,37	-0,39
**SNX9**	6,83	7,22	-0,39
**MTURN**	10,00	10,40	-0,39
**CD163L1**	7,39	7,78	-0,39
**PHLDB1**	7,88	8,27	-0,39
**BIRC2**	9,30	9,69	-0,39
**FBLN7**	9,18	9,58	-0,39
**RAB20**	8,33	8,72	-0,39
**LSM7**	12,46	12,85	-0,39
**ITPKB**	7,75	8,14	-0,39
**ATP6V1G2-DDX39B**	9,82	10,21	-0,39

**MTCH1**	12,66	13,06	-0,39
**TRIR**	12,95	13,34	-0,39
**TRIM3**	8,34	8,74	-0,39
**AIG1**	8,25	8,65	-0,39
**ZNF598**	12,35	12,74	-0,39
**GIGYF1**	12,83	13,23	-0,39
**NOTCH2NLB**	6,46	6,85	-0,39
**AL121594.1**	9,49	9,88	-0,39
**CHTF18**	11,82	12,21	-0,39
**UBE2M**	11,66	12,05	-0,39
**NOP53**	14,00	14,39	-0,39
**FAM110D**	8,04	8,43	-0,39
**MIDEAS**	9,94	10,33	-0,39
**C6orf136**	9,62	10,01	-0,39
**PICK1**	8,53	8,92	-0,39
**LSM4**	12,77	13,17	-0,39
**COQ8B**	9,72	10,11	-0,39
**MDK**	13,56	13,95	-0,39
**WDR91**	10,27	10,66	-0,39
**CD82**	8,92	9,31	-0,39
**BOK**	5,44	5,83	-0,39
**ARMCX2**	6,12	6,51	-0,39
**KCNG1**	10,60	10,99	-0,39
**GTF2H2**	11,48	11,87	-0,39
**FNDC8**	3,82	4,20	-0,39
**FBXL14**	9,85	10,24	-0,39
**TAF4**	2,09	2,48	-0,39
**CAGE1**	2,09	2,48	-0,39
**ZNF573**	8,04	8,42	-0,39
**TPRN**	10,00	10,39	-0,39
**SMIM29**	7,24	7,63	-0,39
**HOXA6**	6,23	6,62	-0,39
**NOC2L**	13,38	13,77	-0,39
**INO80C**	10,47	10,86	-0,39
**MAST1**	7,34	7,73	-0,39
**OXER1**	6,60	6,99	-0,39
**ZNF563**	6,83	7,22	-0,39
**ZNF552**	9,12	9,51	-0,39
**SOX12**	12,31	12,70	-0,39
**TMPRSS3**	7,90	8,29	-0,39
**TMEM238**	6,79	7,18	-0,39
**DDTL**	8,15	8,53	-0,39
**EPHA4**	8,78	9,16	-0,39
**CAPN10**	10,31	10,70	-0,39
**ISY1-RAB43**	9,93	10,32	-0,39
**ZNF613**	9,49	9,88	-0,39
**FRMD8**	11,57	11,96	-0,38
**ZNF593**	10,40	10,79	-0,38
**ENGASE**	10,77	11,16	-0,38
**TMEM250**	10,77	11,15	-0,38
**AC020765.6**	8,46	8,85	-0,38
**AKAP17A**	11,91	12,29	-0,38
**LRRC46**	6,16	6,55	-0,38
**C1QTNF6**	6,45	6,84	-0,38
**PODXL2**	9,90	10,28	-0,38
**LAX1**	7,34	7,72	-0,38

**SNAI1**	8,69	9,08	-0,38
**TMPRSS9**	7,45	7,84	-0,38
**SPTBN5**	4,02	4,41	-0,38
**IGHV1-24**	4,02	4,41	-0,38
**SH3TC1**	9,40	9,79	-0,38
**LENG1**	8,58	8,96	-0,38
**FZR1**	11,31	11,70	-0,38
**RPUSD2**	10,52	10,90	-0,38
**PURA**	11,38	11,77	-0,38
**ZNF585A**	9,51	9,89	-0,38
**LCTL**	6,36	6,74	-0,38
**METTL22**	10,88	11,26	-0,38
**LRRC3**	9,18	9,56	-0,38
**COL5A2**	6,95	7,33	-0,38
**GTPBP3**	11,65	12,03	-0,38
**NBL1**	7,06	7,44	-0,38
**H2BC11**	5,69	6,07	-0,38
**COX7A2**	9,74	10,12	-0,38
**GPD1**	6,69	7,07	-0,38
**OSBPL7**	10,07	10,45	-0,38
**ZFYVE1**	9,97	10,35	-0,38
**PKMYT1**	12,21	12,59	-0,38
**CHRNA1**	6,23	6,61	-0,38
**MAFF**	6,56	6,94	-0,38
**RPL36**	13,57	13,95	-0,38
**PHETA2**	8,86	9,24	-0,38
**AC073896.1**	9,57	9,95	-0,38
**TRAF2**	10,97	11,34	-0,38
**FUOM**	9,24	9,61	-0,38
**IZUMO4**	3,54	3,92	-0,38
**DGKG**	8,11	8,48	-0,38
**CRYBB2**	8,47	8,85	-0,38
**PEX11G**	4,20	4,58	-0,38
**RPP21**	9,77	10,15	-0,38
**TIGD5**	10,35	10,73	-0,38
**ZBTB12**	9,64	10,02	-0,38
**CSAD**	9,19	9,57	-0,38
**RUFY1**	11,36	11,74	-0,38
**ENPP7**	4,36	4,74	-0,38
**APOLD1**	9,40	9,77	-0,38
**ADRM1**	12,64	13,02	-0,38
**MLANA**	4,80	5,18	-0,38
**ARHGDIA**	14,48	14,85	-0,38
**DCTN3**	10,20	10,58	-0,38
**NAV2**	8,19	8,56	-0,38
**PEX19**	12,02	12,39	-0,38
**ZNF83**	9,49	9,87	-0,38
**CCDC107**	9,50	9,87	-0,37
**RILPL2**	10,97	11,34	-0,37
**TMC8**	9,66	10,03	-0,37
**RING1**	10,48	10,86	-0,37
**ALG10B**	11,49	11,86	-0,37
**NUDT9**	10,42	10,80	-0,37
**ZNF528**	10,12	10,50	-0,37
**UCN**	5,61	5,99	-0,37
**FURIN**	10,72	11,09	-0,37

**EEF1D**	14,77	15,14	-0,37
**TEX22**	6,92	7,30	-0,37
**SPAG8**	4,32	4,70	-0,37
**FAM50A**	10,24	10,61	-0,37
**AC006059.2**	9,15	9,53	-0,37
**SPTB**	4,37	4,74	-0,37
**FAM89B**	10,98	11,36	-0,37
**CDK3**	8,50	8,87	-0,37
**PAF1**	11,50	11,88	-0,37
**RELL2**	8,88	9,25	-0,37
**CKM**	2,69	3,06	-0,37
**RFX8**	2,69	3,06	-0,37
**IGHG2**	2,69	3,06	-0,37
**ADRA2B**	2,69	3,06	-0,37
**AL662884.4**	2,69	3,06	-0,37
**CAPN15**	12,49	12,86	-0,37
**C8orf58**	9,07	9,44	-0,37
**FN3KRP**	10,56	10,93	-0,37
**PRSS57**	7,77	8,14	-0,37
**OARD1**	10,51	10,88	-0,37
**PHLDA3**	10,49	10,86	-0,37
**BTN2A2**	10,09	10,46	-0,37
**IGSF8**	10,88	11,25	-0,37
**ZSWIM1**	9,80	10,17	-0,37
**HLA-DPB1**	11,82	12,19	-0,37
**INPP5K**	10,20	10,57	-0,37
**LMAN2**	12,38	12,75	-0,37
**TSR3**	11,11	11,48	-0,37
**NAA38**	9,42	9,79	-0,37
**MCF2**	6,39	6,76	-0,37
**ARNTL2**	6,75	7,12	-0,37
**POLR1G**	11,54	11,90	-0,37
**ZCCHC18**	8,27	8,63	-0,37
**TCHP**	11,57	11,94	-0,37
**SETD6**	10,52	10,89	-0,37
**AGPAT2**	10,65	11,02	-0,37
**FHAD1**	3,40	3,76	-0,37
**NUDT8**	7,91	8,28	-0,37
**CCDC78**	6,91	7,28	-0,37
**AC026464.4**	11,27	11,64	-0,37
**PLA2G6**	9,99	10,36	-0,37
**STMN3**	13,12	13,49	-0,37
**CBX2**	12,55	12,91	-0,37
**FIS1**	11,22	11,59	-0,37
**CFAP410**	10,10	10,46	-0,36
**B9D1**	7,41	7,78	-0,36
**HAAO**	7,69	8,06	-0,36
**EPN1**	12,39	12,75	-0,36
**DGCR6L**	10,19	10,55	-0,36
**FAM43A**	10,78	11,14	-0,36
**RBM42**	12,18	12,55	-0,36
**RPS27AP5**	3,11	3,47	-0,36
**AC000093.1**	8,71	9,07	-0,36
**MAGEF1**	10,09	10,45	-0,36
**PTPA**	12,52	12,88	-0,36
**IER3**	8,64	9,01	-0,36

**BHMT2**	1,98	2,34	-0,36
**KHK**	9,12	9,48	-0,36
**MON1B**	12,74	13,10	-0,36
**EVI5L**	9,29	9,65	-0,36
**PHF12**	12,19	12,55	-0,36
**KIF16B**	11,53	11,89	-0,36
**LCN6**	10,91	11,27	-0,36
**SNRNP70**	13,81	14,17	-0,36
**SLC27A4**	11,25	11,62	-0,36
**PRKRIP1**	11,17	11,53	-0,36
**ADCY5**	6,77	7,13	-0,36
**FDXR**	9,67	10,03	-0,36
**HAP1**	9,43	9,79	-0,36
**MEX3D**	10,51	10,87	-0,36
**ADAMTS9**	9,03	9,39	-0,36
**MCOLN2**	3,40	3,76	-0,36
**CCNA1**	4,98	5,34	-0,36
**C1QL1**	7,52	7,88	-0,36
**ANPEP**	5,16	5,52	-0,36
**CENPB**	12,07	12,42	-0,36
**IGLV3-1**	3,43	3,79	-0,36
**TPM2**	10,09	10,45	-0,36
**RUSF1**	11,48	11,84	-0,36
**TRIM41**	10,78	11,14	-0,36
**DNAH17**	7,26	7,61	-0,36
**IKBKE**	9,94	10,30	-0,36
**CDC37**	13,14	13,50	-0,36
**B3GAT3**	10,47	10,83	-0,36
**LSM14A**	13,76	14,12	-0,36
**RBFA**	11,35	11,70	-0,36
**MCRIP1**	11,12	11,47	-0,36
**ARMC5**	9,96	10,32	-0,36
**SLC25A42**	9,63	9,99	-0,36
**TCOF1**	13,84	14,19	-0,36
**GPATCH4**	12,89	13,25	-0,36
**ALDH4A1**	11,02	11,38	-0,36
**NUP62CL**	4,47	4,82	-0,36
**ILF3**	15,90	16,25	-0,36
**CFAP157**	6,01	6,36	-0,36
**TRIP6**	11,54	11,89	-0,36
**MYDGF**	11,47	11,83	-0,35
**SNCA**	9,77	10,13	-0,35
**NADK**	12,30	12,65	-0,35
**UBE2E2**	10,30	10,65	-0,35
**RASGRP3**	4,26	4,61	-0,35
**MRPS34**	12,16	12,51	-0,35
**ATP5IF1**	11,77	12,13	-0,35
**PLPP1**	7,42	7,77	-0,35
**KCNJ11**	6,91	7,27	-0,35
**USP38**	9,94	10,29	-0,35
**SPNS3**	7,71	8,06	-0,35
**TMEM39B**	10,71	11,07	-0,35
**VASH1**	11,13	11,48	-0,35
**TRIM26**	11,74	12,09	-0,35
**MRPS25**	12,95	13,30	-0,35
**GPR108**	8,66	9,01	-0,35

**ACE**	3,92	4,27	-0,35
**LILRA4**	3,92	4,27	-0,35
**ACACB**	8,84	9,19	-0,35
**TXK**	8,08	8,43	-0,35
**CBR3**	5,68	6,03	-0,35
**PYCR1**	12,84	13,19	-0,35
**NT5M**	8,00	8,35	-0,35
**OBSCN**	11,26	11,61	-0,35
**FLOT1**	12,41	12,76	-0,35
**ASAH2B**	7,96	8,31	-0,35
**NCL**	17,08	17,43	-0,35
**VPS37C**	9,51	9,86	-0,35
**AGAP6**	8,51	8,86	-0,35
**PABPC1L**	9,90	10,25	-0,35
**MYBL1**	5,90	6,25	-0,35
**PTCD1**	10,30	10,65	-0,35
**EPHX2**	8,12	8,47	-0,35
**SMKR1**	7,24	7,59	-0,35
**ZNF821**	9,13	9,48	-0,35
**RPH3AL**	5,36	5,71	-0,35
**BBS1**	9,93	10,28	-0,35
**IFI6**	9,16	9,50	-0,35
**B3GNT5**	7,97	8,31	-0,35
**EIF4H**	14,85	15,20	-0,35
**GPRC5C**	3,92	4,27	-0,35
**MPL**	4,44	4,79	-0,35
**LMBR1**	11,30	11,64	-0,35
**DDIT4**	9,83	10,18	-0,35
**TOP3B**	11,71	12,05	-0,35
**TAS2R14**	7,03	7,38	-0,35
**SPATA2L**	8,38	8,73	-0,35
**SPHK2**	10,49	10,84	-0,35
**PRR12**	12,13	12,47	-0,35
**FUCA2**	6,82	7,17	-0,35
**HLA-DRB5**	11,16	11,50	-0,35
**ANGPT2**	8,28	8,63	-0,35
**YPEL3**	9,29	9,63	-0,35
**HNRNPA0**	14,56	14,91	-0,34
**KIAA0895**	7,87	8,22	-0,34
**PGGHG**	11,22	11,56	-0,34
**RRP9**	11,82	12,16	-0,34
**S1PR2**	8,65	9,00	-0,34
**REEP6**	6,75	7,10	-0,34
**CDH4**	6,23	6,57	-0,34
**GNAO1**	5,15	5,49	-0,34
**CLDN20**	5,21	5,55	-0,34
**ANKRD35**	5,21	5,55	-0,34
**HDGF**	15,72	16,06	-0,34
**FMO5**	7,21	7,56	-0,34
**SAMD12**	2,57	2,91	-0,34
**H2AC6**	7,18	7,52	-0,34
**APPL2**	10,19	10,54	-0,34
**ATF5**	12,74	13,08	-0,34
**CDC42EP2**	6,67	7,02	-0,34
**SPNS2**	8,44	8,78	-0,34
**PLPP3**	7,18	7,52	-0,34

**ROBO3**	7,82	8,17	-0,34
**DYNLL2**	13,25	13,59	-0,34
**RASL12**	7,01	7,35	-0,34
**VPS13D**	12,12	12,47	-0,34
**MECP2**	12,09	12,43	-0,34
**ZNF491**	8,76	9,10	-0,34
**ECH1**	12,23	12,57	-0,34
**TMEM254**	9,91	10,25	-0,34
**CDC42BPB**	9,23	9,57	-0,34
**DXO**	10,06	10,41	-0,34
**TOMM40**	13,57	13,91	-0,34
**CYB5R1**	10,31	10,65	-0,34
**CRB2**	9,30	9,64	-0,34
**TBC1D25**	9,98	10,32	-0,34
**C1orf122**	9,50	9,84	-0,34
**FAM3A**	10,09	10,43	-0,34
**PPM1F**	11,85	12,19	-0,34
**TRIM73**	8,82	9,16	-0,34
**RTN4RL2**	7,77	8,11	-0,34
**NOL4**	7,12	7,46	-0,34
**C11orf95**	11,89	12,23	-0,34
**TMEM63C**	10,54	10,87	-0,34
**IGFBP7**	12,59	12,92	-0,34
**MAD1L1**	11,24	11,58	-0,34
**ACADVL**	13,09	13,43	-0,34
**ISY1**	11,75	12,09	-0,34
**ANKRD13B**	11,69	12,03	-0,34
**SPSB3**	10,38	10,71	-0,34
**DUS1L**	12,60	12,94	-0,34
**FAM193B**	11,72	12,06	-0,34
**LUC7L**	12,60	12,94	-0,34
**LGALS1**	9,50	9,84	-0,34
**EMD**	11,29	11,62	-0,34
**PDLIM5**	9,31	9,65	-0,34
**SLC46A1**	10,12	10,46	-0,34
**TAOK2**	12,14	12,48	-0,34
**ARIH2**	12,92	13,25	-0,34
**MMS22L**	9,20	9,54	-0,34
**ANKRD54**	10,50	10,84	-0,33
**ZNF524**	8,50	8,84	-0,33
**FLNA**	14,71	15,04	-0,33
**TRIM45**	8,89	9,23	-0,33
**SLC25A41**	5,81	6,15	-0,33
**SLC26A9**	8,60	8,93	-0,33
**GALNT8**	6,53	6,87	-0,33
**UBXN6**	10,88	11,21	-0,33
**MELTF**	11,11	11,44	-0,33
**ELOF1**	11,12	11,45	-0,33
**FKBP8**	12,49	12,82	-0,33
**IER5**	11,71	12,04	-0,33
**ZNF785**	11,16	11,49	-0,33
**AC106886.5**	12,36	12,70	-0,33
**HNRNPUL2**	13,69	14,02	-0,33
**KDM4D**	6,70	7,03	-0,33
**H2BC5**	5,74	6,07	-0,33
**ARMC7**	9,28	9,61	-0,33

**HLA-DRB1**	11,64	11,97	-0,33
**ADRB1**	5,68	6,01	-0,33
**TNRC6B**	12,13	12,46	-0,33
**KLHL29**	6,03	6,36	-0,33
**BLOC1S1**	8,51	8,84	-0,33
**POLRMT**	12,19	12,52	-0,33
**KDM5C**	13,05	13,38	-0,33
**DHX38**	12,68	13,01	-0,33
**C9orf153**	4,10	4,43	-0,33
**ZNF341**	8,65	8,98	-0,33
**LRRC14**	11,92	12,25	-0,33
**AC011448.1**	6,74	7,07	-0,33
**MAPK8IP3**	11,74	12,07	-0,33
**TARBP2**	10,64	10,97	-0,33
**HRAS**	10,63	10,96	-0,33
**CCHCR1**	10,62	10,95	-0,33
**C11orf98**	11,65	11,98	-0,33
**ARRDC5**	5,18	5,51	-0,33
**LUC7L3**	14,58	14,90	-0,33
**PIANP**	6,55	6,88	-0,33
**OPTN**	8,17	8,49	-0,33
**BRMS1**	11,81	12,14	-0,33
**HSPBP1**	11,37	11,70	-0,33
**CYP26A1**	2,64	2,97	-0,33
**BAG1**	12,27	12,60	-0,33
**SLC9A8**	10,15	10,48	-0,33
**HNRNPA1P48**	4,87	5,20	-0,33
**CRAMP1**	11,71	12,04	-0,33
**IGSF9B**	8,72	9,05	-0,33
**SPINDOC**	12,59	12,91	-0,33
**MLC1**	6,99	7,32	-0,33
**IGHV3-30**	8,89	9,21	-0,33
**PDRG1**	10,96	11,29	-0,33
**RGPD3**	6,21	6,54	-0,33
**MED26**	9,96	10,29	-0,33
**APC2**	6,52	6,85	-0,33
**TEDC2**	10,68	11,00	-0,33
**TESK2**	6,72	7,05	-0,33
**DMC1**	8,25	8,58	-0,33
**AC099489.1**	7,16	7,49	-0,32
**ATP2A3**	11,88	12,20	-0,32
**EHD4**	8,23	8,56	-0,32
**CYBB**	4,51	4,83	-0,32
**GPANK1**	9,78	10,10	-0,32
**ERICH1**	10,34	10,66	-0,32
**RPS29**	14,00	14,33	-0,32
**KIFC2**	9,35	9,67	-0,32
**INPPL1**	13,40	13,72	-0,32
**RNF5**	11,43	11,76	-0,32
**AL121900.1**	8,11	8,44	-0,32
**FAM167B**	4,37	4,69	-0,32
**DHRS4L2**	10,47	10,79	-0,32
**APCDD1**	10,84	11,16	-0,32
**ADAMTS10**	10,34	10,66	-0,32
**MUTYH**	10,67	11,00	-0,32
**TNFRSF25**	4,13	4,45	-0,32

**KIF24**	8,53	8,85	-0,32
**LY6K**	5,62	5,94	-0,32
**EIF3A**	15,05	15,37	-0,32
**ZNF311**	5,77	6,09	-0,32
**FCMR**	9,46	9,78	-0,32
**RAB27A**	10,18	10,50	-0,32
**SF3A1**	13,86	14,19	-0,32
**MT1F**	8,55	8,87	-0,32
**DIO2**	5,25	5,58	-0,32
**ITGB8**	4,20	4,53	-0,32
**FAM181B**	4,20	4,53	-0,32
**ADGRG5**	4,20	4,53	-0,32
**AP1S1**	11,40	11,72	-0,32
**ITGB1BP1**	12,36	12,68	-0,32
**SLC9A9**	5,83	6,16	-0,32
**DCAF8**	12,71	13,03	-0,32
**PRMT2**	10,42	10,74	-0,32
**COLGALT2**	5,17	5,49	-0,32
**TMEM205**	10,00	10,32	-0,32
**HEXIM2**	7,48	7,80	-0,32
**SLC9A3**	10,51	10,83	-0,32
**RNF166**	10,02	10,34	-0,32
**FUZ**	9,38	9,70	-0,32
**TOX4**	12,28	12,60	-0,32
**BAIAP2L2**	6,47	6,78	-0,32
**THEM6**	10,76	11,08	-0,32
**NAA80**	8,26	8,58	-0,32
**ABCA12**	4,02	4,34	-0,32
**ZNF579**	10,12	10,44	-0,32
**RALGDS**	12,06	12,38	-0,32
**CABP1**	3,07	3,39	-0,32
**RCAN3**	5,74	6,06	-0,32
**SLC2A8**	8,53	8,85	-0,32
**AL139260.3**	8,10	8,42	-0,32
**ITPR1**	10,71	11,03	-0,32
**NAPRT**	11,07	11,39	-0,32
**JPH2**	7,78	8,09	-0,32
**NT5C**	9,91	10,23	-0,32
**MSH5-SAPCD1**	10,48	10,80	-0,32
**DEK**	15,01	15,33	-0,32
**PBDC1**	10,39	10,70	-0,32
**RASIP1**	9,72	10,04	-0,32
**ZNF136**	10,59	10,91	-0,32
**SLC25A6**	14,72	15,04	-0,32
**GOLGA7B**	3,33	3,64	-0,32
**CCDC51**	10,22	10,54	-0,32
**SAP25**	8,50	8,81	-0,32
**GRN**	11,49	11,80	-0,32
**LRRC4B**	3,82	4,13	-0,32
**HOOK1**	3,82	4,13	-0,32
**TSLP**	3,82	4,13	-0,32
**TAS2R31**	3,82	4,13	-0,32
**SLC25A22**	11,32	11,63	-0,32
**SMIM11B**	7,35	7,67	-0,32
**MYO9B**	12,78	13,10	-0,32
**RP9**	10,05	10,36	-0,32

**ZNF76**	10,96	11,27	-0,32
**RBM10**	12,54	12,86	-0,32
**PNN**	14,67	14,99	-0,32
**DMAC2L**	9,89	10,21	-0,32
**HDAC7**	12,83	13,14	-0,32
**IER2**	11,55	11,86	-0,31
**PARP10**	11,36	11,68	-0,31
**SAFB**	14,05	14,36	-0,31
**JUNB**	10,53	10,84	-0,31
**CNTNAP3B**	3,50	3,82	-0,31
**CRYGS**	6,33	6,65	-0,31
**MADCAM1**	4,91	5,22	-0,31
**ZNF276**	10,54	10,85	-0,31
**FAM214B**	8,89	9,20	-0,31
**GPR3**	5,85	6,16	-0,31
**SMCO4**	9,38	9,70	-0,31
**MAP7D1**	11,93	12,25	-0,31
**UQCC2**	10,02	10,34	-0,31
**PRKAG1**	10,44	10,75	-0,31
**KNOP1**	12,59	12,90	-0,31
**TFCP2**	10,64	10,96	-0,31
**BRF1**	11,99	12,30	-0,31
**RRBP1**	12,58	12,89	-0,31
**MEX3A**	12,68	12,99	-0,31
**GUCY2C**	3,57	3,88	-0,31
**SLC2A12**	3,57	3,88	-0,31
**PMCH**	3,57	3,88	-0,31
**IGHV4-61**	3,57	3,88	-0,31
**ZNF404**	6,61	6,92	-0,31
**HDHD5**	12,17	12,48	-0,31
**MT-ND3**	14,83	15,14	-0,31
**EML3**	11,30	11,61	-0,31
**CNKSR1**	8,08	8,39	-0,31
**AC010522.1**	7,03	7,34	-0,31
**NRP2**	11,43	11,74	-0,31
**PIP4P2**	9,57	9,88	-0,31
**TULP3**	10,69	11,00	-0,31
**PUS1**	12,17	12,48	-0,31
**TCP11L2**	5,77	6,09	-0,31
**SLC29A3**	9,24	9,55	-0,31
**ZRSR2**	9,94	10,25	-0,31
**ETFBKMT**	7,17	7,48	-0,31
**ZFPL1**	10,35	10,66	-0,31
**FAM155B**	7,52	7,83	-0,31
**EXOSC1**	11,00	11,31	-0,31
**PAXX**	10,51	10,82	-0,31
**AMOT**	11,10	11,41	-0,31
**PWP2**	8,30	8,61	-0,31
**FRY**	6,52	6,83	-0,31
**POMT2**	9,07	9,38	-0,31
**ZNF771**	10,58	10,89	-0,31
**NUMBL**	9,66	9,97	-0,31
**KIF7**	9,11	9,42	-0,31
**TOP1MT**	12,53	12,84	-0,31
**DPH1**	10,89	11,20	-0,31
**DMAC1**	11,16	11,47	-0,31

**NBPF10**	10,07	10,37	-0,31
**UBASH3A**	2,98	3,28	-0,31
**MRPS7**	11,99	12,30	-0,31
**FES**	10,14	10,44	-0,31
**CFAP94**	6,26	6,56	-0,31
**MRPL23**	11,52	11,83	-0,31
**CNPY4**	9,15	9,45	-0,31
**ABHD14A**	9,03	9,34	-0,31
**BOLA3**	10,24	10,54	-0,31
**ZMAT2**	12,15	12,46	-0,31
**UPF3A**	11,54	11,84	-0,31
**RPL26L1**	11,15	11,46	-0,31
**NPIPA2**	9,57	9,88	-0,31
**NPIPB13**	10,60	10,91	-0,31
**RGS14**	10,36	10,67	-0,31
**RPL39L**	10,12	10,42	-0,31
**CNPY3**	12,36	12,67	-0,31
**DHX37**	12,54	12,85	-0,31
**ZSCAN25**	10,60	10,91	-0,31
**YJEFN3**	9,35	9,66	-0,31
**BMP7**	3,28	3,59	-0,31
**KRT80**	3,28	3,59	-0,31
**PDE4C**	3,28	3,59	-0,31
**FAM107A**	3,28	3,59	-0,31
**MISP**	3,28	3,59	-0,31
**FAM110C**	3,28	3,59	-0,31
**BMF**	9,18	9,49	-0,31
**ELK1**	11,20	11,51	-0,31
**FTSJ3**	13,04	13,34	-0,31
**ELP5**	6,82	7,13	-0,31
**KIDINS220**	12,10	12,41	-0,31
**DISP2**	6,78	7,09	-0,31
**OTOGL**	5,31	5,61	-0,31
**TBCK**	10,99	11,29	-0,31
**IRF3**	11,49	11,79	-0,31
**BSCL2**	8,27	8,58	-0,31
**MAGT1**	8,37	8,67	-0,31
**ATF4**	10,93	11,24	-0,31
**IDH3A**	12,15	12,45	-0,31
**DBNL**	12,75	13,05	-0,31
**KCNK3**	6,23	6,54	-0,31
**TAFA2**	4,66	4,96	-0,30
**ZNF169**	9,25	9,56	-0,30
**SHMT2**	13,62	13,92	-0,30
**ANAPC2**	11,52	11,82	-0,30
**CBWD3**	10,82	11,13	-0,30
**IL17D**	9,13	9,43	-0,30
**MYH3**	8,73	9,03	-0,30
**TRIM56**	13,25	13,56	-0,30
**RPS10-NUDT3**	11,08	11,39	-0,30
**TXLNB**	7,86	8,16	-0,30
**NPIPB3**	13,19	13,50	-0,30
**SLC32A1**	5,33	5,63	-0,30
**PCOLCE**	7,07	7,37	-0,30
**KEAP1**	12,84	13,15	-0,30
**TSPAN12**	5,77	6,07	-0,30

**AK9**	7,23	7,53	-0,30
**OXLD1**	9,38	9,68	-0,30
**FKBP9**	4,58	4,88	-0,30
**CDH15**	4,58	4,88	-0,30
**HSFX1**	4,58	4,88	-0,30
**IMPACT**	6,85	7,15	-0,30
**DVL3**	13,04	13,34	-0,30
**NAGPA**	9,55	9,85	-0,30
**EIF5AL1**	6,37	6,67	-0,30
**ARID5A**	11,60	11,91	-0,30
**LIN7A**	5,25	5,55	-0,30
**DEGS2**	5,25	5,55	-0,30
**GNRH1**	7,83	8,13	-0,30
**NDUFB7**	11,67	11,97	-0,30
**ABHD16B**	7,91	8,21	-0,30
**PTPRCAP**	10,84	11,14	-0,30
**CD151**	9,89	10,19	-0,30
**IGFN1**	5,76	6,06	-0,30
**PGP**	11,24	11,54	-0,30
**ZNF71**	10,53	10,83	-0,30
**ANKRD34A**	7,24	7,54	-0,30
**CXXC1**	12,20	12,50	-0,30
**ANAPC11**	11,84	12,14	-0,30
**WDR6**	13,83	14,13	-0,30
**HABP4**	8,32	8,62	-0,30
**MED22**	11,85	12,15	-0,30
**NUDT14**	9,34	9,64	-0,30
**SFPQ**	15,94	16,24	-0,30
**DGKQ**	10,60	10,90	-0,30
**PTK7**	12,30	12,60	-0,30
**PLAUR**	10,21	10,51	-0,30
**ADCY6**	11,78	12,08	-0,30
**RANGAP1**	13,45	13,75	-0,30
**TRAF7**	13,00	13,30	-0,30
**PELP1**	12,78	13,07	-0,30
**TP53INP2**	10,08	10,38	-0,30
**CARD11**	9,65	9,94	-0,30
**RGS12**	11,84	12,13	-0,30
**TAPBPL**	9,32	9,62	-0,30
**TENT5C**	7,52	7,81	-0,30
**ZNF296**	10,04	10,34	-0,30
**LRRC27**	8,35	8,64	-0,30
**HOMER3**	10,92	11,21	-0,30
**ST20**	7,53	7,82	-0,30
**CFAP20DC**	7,83	8,12	-0,30
**ACAP3**	11,42	11,72	-0,30
**GCNT3**	2,91	3,21	-0,30
**C11orf42**	2,91	3,21	-0,30
**USP17L1**	2,91	3,21	-0,30
**DLEU7**	5,67	5,97	-0,30
**ZNF451**	12,18	12,48	-0,30
**FMC1-LUC7L2**	11,87	12,17	-0,30
**SP2**	11,18	11,48	-0,30
**ZNF548**	11,23	11,52	-0,30
**BUD23**	12,14	12,44	-0,30
**TSSC4**	10,50	10,80	-0,30

KIAA0895L	11,16	11,46	-0,30
PIGQ	11,16	11,45	-0,30
TTC31	10,62	10,91	-0,30
PBX2	12,46	12,75	-0,30
COX16	11,58	11,88	-0,29
MRPL4	12,57	12,86	-0,29
IRS1	10,63	10,92	-0,29
RECQL4	12,65	12,94	-0,29
MRC2	10,32	10,62	-0,29
APTX	11,26	11,56	-0,29
TUBB6	12,46	12,75	-0,29
UNC119	10,85	11,14	-0,29
MBD3	13,25	13,54	-0,29
TEX14	5,11	5,40	-0,29
ACIN1	13,53	13,83	-0,29
HDAC6	11,68	11,97	-0,29
TBC1D30	8,52	8,82	-0,29
PRRT2	10,72	11,01	-0,29
OS9	12,59	12,88	-0,29
SPDYA	5,95	6,25	-0,29
LASP1	12,31	12,61	-0,29
NAGK	10,26	10,55	-0,29
SSRP1	15,04	15,34	-0,29
MAP2K7	11,59	11,88	-0,29
TMEM256-PLSCR3	4,52	4,81	-0,29
CORO7	12,21	12,51	-0,29
RAB4B	9,69	9,99	-0,29
R3HCC1L	10,91	11,20	-0,29
CEBPA	9,15	9,45	-0,29
NTNG2	9,18	9,47	-0,29
LONP1	13,07	13,36	-0,29
ZNF500	10,25	10,55	-0,29
TRH	4,36	4,65	-0,29
MLLT6	13,98	14,28	-0,29
HEXIM1	10,55	10,84	-0,29
AMDHD2	10,38	10,67	-0,29
FHL2	4,87	5,16	-0,29
CILP2	4,87	5,16	-0,29
FRS3	8,05	8,34	-0,29
MAP3K11	12,27	12,56	-0,29
PMP22	6,89	7,18	-0,29
CCS	9,68	9,97	-0,29
B3GALT6	11,50	11,79	-0,29
MRPL36	10,98	11,27	-0,29
TNKS1BP1	11,34	11,63	-0,29
UNKL	11,40	11,69	-0,29
THSD7A	8,60	8,88	-0,29
MYBBP1A	13,36	13,65	-0,29
CNFN	5,68	5,97	-0,29
SELENOO	11,09	11,38	-0,29
DPYSL4	10,52	10,81	-0,29
DEF6	11,33	11,62	-0,29
USP5	12,97	13,26	-0,29
CFDP1	11,76	12,05	-0,29
ZNF703	10,87	11,16	-0,29
USP6	6,59	6,88	-0,29

**GIT1**	13,38	13,67	-0,29
**CCDC24**	7,57	7,86	-0,29
**SKIV2L**	11,54	11,83	-0,29
**PAGR1**	11,61	11,90	-0,29
**OGFOD2**	10,03	10,32	-0,29
**PDIA2**	6,33	6,62	-0,29
**IFT20**	9,26	9,55	-0,29
**PDE4A**	10,92	11,20	-0,29
**CLUH**	13,94	14,23	-0,29
**TSPO2**	0,86	1,15	-0,29
**DNAJB5**	8,68	8,97	-0,29
**APOBEC3H**	6,24	6,53	-0,29
**PDE1B**	8,28	8,57	-0,29
**MLF2**	12,25	12,54	-0,29
**ASMTL**	10,82	11,10	-0,29
**PCSK4**	7,03	7,32	-0,29
**SMOC1**	4,64	4,93	-0,29
**ZNF513**	8,73	9,02	-0,29
**PRMT7**	10,81	11,10	-0,29
**TADA3**	8,01	8,29	-0,29
**TCEANC**	8,13	8,41	-0,29
**TBC1D3B**	5,55	5,83	-0,29
**RAB24**	9,24	9,52	-0,29
**SLC12A4**	9,95	10,24	-0,29
**SNX22**	12,03	12,32	-0,29
**PEX5**	11,71	12,00	-0,29
**LENG9**	9,43	9,72	-0,29
**PARP3**	8,56	8,84	-0,29
**SSTR2**	6,46	6,75	-0,29
**RAB41**	2,99	3,27	-0,29
**SLC39A1**	11,80	12,08	-0,29
**TAB1**	11,14	11,43	-0,29
**ZFAND2B**	9,58	9,86	-0,29
**TRAF4**	12,17	12,45	-0,29
**DHRS7B**	6,83	7,11	-0,29
**COL5A3**	5,55	5,83	-0,29
**MRPL51**	12,51	12,79	-0,29
**PACSIN1**	6,24	6,53	-0,28
**DNAJB13**	3,02	3,30	-0,28
**PGD**	13,90	14,19	-0,28
**ANP32B**	14,87	15,16	-0,28
**C15orf39**	11,65	11,93	-0,28
**DTX4**	9,53	9,82	-0,28
**AC008764.4**	7,90	8,19	-0,28
**BSPRY**	9,98	10,27	-0,28
**RHPN1**	10,83	11,11	-0,28
**GPSM1**	12,69	12,97	-0,28
**MRPL12**	12,92	13,20	-0,28
**COL6A3**	8,20	8,48	-0,28
**L1CAM**	6,90	7,18	-0,28
**GNG7**	11,98	12,26	-0,28
**ZNF174**	9,85	10,14	-0,28
**NUBP2**	11,80	12,08	-0,28
**KCNA5**	7,80	8,09	-0,28
**CD70**	9,34	9,62	-0,28
**SMIM26**	10,21	10,50	-0,28

**NDRG2**	9,50	9,79	-0,28
**RHOF**	10,27	10,55	-0,28
**TIMM44**	11,97	12,25	-0,28
**TYSND1**	11,28	11,56	-0,28
**VPS41**	11,91	12,20	-0,28
**ITGA10**	7,29	7,57	-0,28
**FOS**	6,21	6,49	-0,28
**DFFA**	12,58	12,86	-0,28
**NSMCE1**	10,72	11,00	-0,28
**FKBP14**	9,73	10,01	-0,28
**QDPR**	11,18	11,46	-0,28
**PEA15**	11,78	12,06	-0,28
**TMEM104**	9,78	10,07	-0,28
**TENT5B**	4,64	4,92	-0,28
**THOC6**	11,87	12,15	-0,28
**TMEM79**	8,65	8,93	-0,28
**POP7**	11,42	11,70	-0,28
**MAP3K19**	4,79	5,07	-0,28
**SLC66A2**	11,54	11,82	-0,28
**NCAPD3**	10,29	10,57	-0,28
**PCBP4**	10,84	11,12	-0,28
**POLE4**	10,01	10,30	-0,28
**CDKL2**	4,13	4,41	-0,28
**FICD**	8,03	8,31	-0,28
**CMPK1**	9,54	9,82	-0,28
**P2RY11**	9,95	10,23	-0,28
**DIPK1A**	8,88	9,16	-0,28
**BAHD1**	11,89	12,18	-0,28
**CXorf56**	10,96	11,24	-0,28
**PCDHB2**	2,42	2,70	-0,28
**ANGPTL1**	2,42	2,70	-0,28
**KRT7**	2,42	2,70	-0,28
**EPN3**	2,42	2,70	-0,28
**NID1**	2,42	2,70	-0,28
**GDPD2**	2,42	2,70	-0,28
**GIPC2**	2,42	2,70	-0,28
**SECTM1**	2,42	2,70	-0,28
**TRIM43**	2,42	2,70	-0,28
**CHODL**	2,42	2,70	-0,28
**CRYBB1**	2,42	2,70	-0,28
**EDAR**	2,42	2,70	-0,28
**NECTIN4**	2,42	2,70	-0,28
**COL26A1**	2,42	2,70	-0,28
**SDC3**	2,42	2,70	-0,28
**CSMD3**	2,42	2,70	-0,28
**RFLNA**	2,42	2,70	-0,28
**PCDHGA4**	2,42	2,70	-0,28
**TNFRSF6B**	6,86	7,14	-0,28
**BCKDHA**	10,63	10,91	-0,28
**ZNHIT2**	9,11	9,39	-0,28
**PRMT9**	10,09	10,37	-0,28
**CDKN1A**	12,68	12,96	-0,28
**FANCE**	10,83	11,11	-0,28
**MCTP2**	7,55	7,83	-0,28
**AC068631.2**	9,43	9,70	-0,28
**WDR46**	12,75	13,03	-0,28

**SRR**	10,02	10,30	-0,28
**SLC52A2**	12,07	12,35	-0,28
**FKBPL**	8,60	8,88	-0,28
**ATOX1**	10,19	10,47	-0,28
**IGF2BP2**	7,09	7,36	-0,28
**LACTB**	9,63	9,91	-0,28
**PEX16**	10,13	10,41	-0,28
**PIK3R3**	12,15	12,43	-0,28
**MEF2D**	13,77	14,05	-0,28
**MRPS33**	10,69	10,97	-0,28
**HMGN5**	10,74	11,02	-0,28
**C1orf50**	8,98	9,26	-0,28
**TLCD3B**	6,41	6,68	-0,28
**ELFN2**	11,79	12,07	-0,28
**GGN**	3,78	4,06	-0,28
**ZFP69B**	8,86	9,13	-0,28
**AGPAT4**	9,70	9,98	-0,28
**TBRG1**	11,40	11,67	-0,28
**CDCA4**	12,25	12,53	-0,28
**HS6ST1**	11,77	12,05	-0,28
**EPS8L1**	8,45	8,72	-0,28
**CDK11B**	12,06	12,34	-0,28
**MRPL2**	11,24	11,51	-0,28
**TAF3**	11,30	11,58	-0,28
**SAR1B**	11,46	11,74	-0,28
**QTRT1**	11,43	11,71	-0,28
**YKT6**	12,77	13,04	-0,28
**GAS2L1**	5,59	5,86	-0,28
**ABTB1**	7,95	8,22	-0,28
**MINK1**	11,28	11,55	-0,28
**TMEM132A**	10,64	10,92	-0,28
**FARSA**	12,98	13,26	-0,27
**AC012651.1**	10,51	10,78	-0,27
**PYCARD**	10,36	10,63	-0,27
**GRK6**	12,94	13,21	-0,27
**TLNRD1**	12,00	12,27	-0,27
**DPYSL3**	8,86	9,14	-0,27
**LPAR2**	9,64	9,92	-0,27
**MRPL49**	11,82	12,09	-0,27
**NOG**	12,22	12,49	-0,27
**FRAT1**	8,73	9,01	-0,27
**MFSD13A**	9,96	10,24	-0,27
**MEGF8**	11,00	11,27	-0,27
**AC009133.6**	10,73	11,00	-0,27
**KCTD15**	10,34	10,62	-0,27
**E2F1**	12,75	13,02	-0,27
**CBX4**	11,30	11,57	-0,27
**TMEM258**	11,77	12,04	-0,27
**AMH**	6,46	6,74	-0,27
**SHB**	6,46	6,74	-0,27
**GANC**	8,52	8,79	-0,27
**RPL38**	14,48	14,75	-0,27
**PIDD1**	11,04	11,32	-0,27
**SYNGR3**	5,25	5,52	-0,27
**EDARADD**	5,25	5,52	-0,27
**MARCKSL1**	14,99	15,26	-0,27

**PUSL1**	9,97	10,24	-0,27
**LMNA**	5,15	5,43	-0,27
**TRMT2A**	11,47	11,75	-0,27
**TNFRSF17**	3,70	3,97	-0,27
**KCTD16**	3,70	3,97	-0,27
**CTXND1**	3,70	3,97	-0,27
**ILDR1**	3,70	3,97	-0,27
**ARHGEF1**	12,62	12,89	-0,27
**CSKMT**	8,81	9,09	-0,27
**GRIN1**	7,37	7,64	-0,27
**CD79A**	13,03	13,31	-0,27
**SEM1**	9,81	10,08	-0,27
**ZNF480**	12,44	12,71	-0,27
**STC2**	11,67	11,94	-0,27
**SERTAD1**	8,57	8,84	-0,27
**ENOX2**	8,99	9,26	-0,27
**NAT8L**	9,41	9,68	-0,27
**CPXM1**	13,09	13,36	-0,27
**SAP30**	10,75	11,02	-0,27
**CUEDC2**	11,99	12,27	-0,27
**KCNN4**	8,33	8,60	-0,27
**ECHDC2**	9,47	9,74	-0,27
**HDGFL2**	11,53	11,80	-0,27
**FLG2**	4,37	4,64	-0,27
**TTC26**	10,31	10,58	-0,27
**JUN**	9,74	10,01	-0,27
**SSNA1**	11,57	11,84	-0,27
**SLC4A2**	12,29	12,56	-0,27
**GIPC1**	10,54	10,81	-0,27
**SNRNP25**	11,91	12,18	-0,27
**USF1**	12,28	12,55	-0,27
**ERFE**	8,58	8,85	-0,27
**GALK1**	11,24	11,51	-0,27
**EWSR1**	14,75	15,02	-0,27
**TIMM10**	11,83	12,10	-0,27
**LYAR**	12,33	12,60	-0,27
**TMCO4**	10,19	10,46	-0,27
**ZNF687**	11,42	11,69	-0,27
**IFT122**	10,43	10,70	-0,27
**PLEC**	9,76	10,03	-0,27
**LRRC69**	6,08	6,35	-0,27
**SREBF1**	12,46	12,73	-0,27
**SMARCA4**	14,87	15,14	-0,27
**UBTF**	14,40	14,67	-0,27
**CARMIL2**	11,61	11,88	-0,27
**TNFSF9**	8,67	8,93	-0,27
**AC134669.1**	6,58	6,85	-0,27
**ID4**	8,25	8,52	-0,27
**COX5B**	12,46	12,73	-0,27
**PCDH7**	3,04	3,30	-0,27
**NHLRC4**	7,63	7,90	-0,27
**ZBTB11**	9,07	9,34	-0,27
**TSEN54**	11,71	11,98	-0,27
**ATP5MC2**	13,90	14,17	-0,27
**ARMC4**	7,89	8,16	-0,27
**GABBR1**	10,88	11,15	-0,27

**ZC3H7B**	13,60	13,87	-0,27
**LAMTOR2**	10,96	11,23	-0,27
**HSPE1-MOB4**	8,65	8,92	-0,27
**VPS72**	12,44	12,71	-0,27
**RSRC2**	13,09	13,36	-0,27
**SHROOM1**	4,99	5,25	-0,27
**ZDHHC24**	10,47	10,74	-0,27
**CCDC187**	9,14	9,41	-0,27
**MIIP**	10,51	10,78	-0,27
**FLII**	12,94	13,20	-0,27
**ZNF584**	9,83	10,10	-0,27
**CENPV**	9,41	9,68	-0,26
**SRC**	10,94	11,21	-0,26
**JUND**	11,58	11,84	-0,26
**PRG2**	6,52	6,78	-0,26
**KDM4B**	11,06	11,33	-0,26
**LETM2**	9,23	9,50	-0,26
**INPP5E**	10,70	10,96	-0,26
**GPKOW**	10,35	10,62	-0,26
**ATP6V0A1**	6,53	6,79	-0,26
**CDH3**	7,98	8,25	-0,26
**ZCCHC10**	11,53	11,79	-0,26
**KHSRP**	15,07	15,34	-0,26
**SORBS3**	11,90	12,17	-0,26
**SYMPK**	12,95	13,21	-0,26
**SLC17A5**	8,99	9,25	-0,26
**ACOT8**	9,67	9,94	-0,26
**FAM222A**	8,29	8,55	-0,26
**POGZ**	13,37	13,63	-0,26
**FASTK**	10,83	11,10	-0,26
**TRABD**	13,09	13,35	-0,26
**FJX1**	12,41	12,67	-0,26
**ACTA2**	6,61	6,87	-0,26
**SLC38A5**	12,73	13,00	-0,26
**TWF2**	11,85	12,11	-0,26
**GOLGA6L10**	7,56	7,82	-0,26
**EFNB1**	10,02	10,28	-0,26
**SDR39U1**	10,38	10,64	-0,26
**MYADML2**	4,20	4,47	-0,26
**CAMK2N1**	4,20	4,47	-0,26
**KDM6B**	11,88	12,14	-0,26
**NIPAL3**	9,46	9,73	-0,26
**VPS9D1**	8,00	8,27	-0,26
**IGFBP4**	12,32	12,59	-0,26
**PLXNB1**	12,68	12,94	-0,26
**HNRNPM**	15,68	15,94	-0,26
**METTL26**	11,39	11,66	-0,26
**C1orf216**	10,76	11,02	-0,26
**EFCAB2**	8,25	8,51	-0,26
**RPUSD1**	11,40	11,67	-0,26
**SGF29**	10,40	10,67	-0,26
**PIM3**	11,55	11,81	-0,26
**PLEKHJ1**	11,93	12,20	-0,26
**GAS1**	8,19	8,45	-0,26
**NCDN**	10,74	11,00	-0,26
**PRR7**	9,31	9,57	-0,26

**HNRNPUL1**	14,95	15,21	-0,26
**DNTTIP1**	10,48	10,74	-0,26
**AATF**	12,52	12,78	-0,26
**FERMT3**	13,28	13,54	-0,26
**STPG3**	2,88	3,14	-0,26
**TNIK**	6,48	6,74	-0,26
**ACYP2**	8,54	8,80	-0,26
**BTBD9**	9,55	9,81	-0,26
**TTLL12**	13,43	13,69	-0,26
**CRYM**	4,98	5,24	-0,26
**LAMP3**	10,04	10,30	-0,26
**SEC14L2**	8,35	8,61	-0,26
**CNPPD1**	11,81	12,07	-0,26
**SPNS1**	10,82	11,08	-0,26
**YIPF2**	9,89	10,15	-0,26
**MYO3A**	8,41	8,67	-0,26
**PCMT1**	10,98	11,24	-0,26
**PPDPF**	11,95	12,21	-0,26
**BOP1**	13,70	13,96	-0,26
**TEX264**	10,38	10,64	-0,26
**CRELD2**	11,73	11,99	-0,26
**PDCD4**	11,14	11,39	-0,26
**BAG6**	13,43	13,68	-0,26
**YJU2**	10,41	10,67	-0,26
**KCNC1**	5,88	6,14	-0,26
**NOC4L**	11,30	11,56	-0,26
**DAB1**	8,11	8,36	-0,26
**ZFYVE16**	11,36	11,62	-0,26
**HAGH**	9,91	10,17	-0,26
**AC073508.2**	9,73	9,99	-0,26
**CCDC57**	9,09	9,35	-0,26
**MOB3A**	10,30	10,55	-0,26
**GRWD1**	12,75	13,01	-0,26
**NPIPB8**	4,39	4,65	-0,26
**RBMX2**	10,65	10,90	-0,26
**DPP3**	10,39	10,65	-0,26
**BOLA1**	9,32	9,57	-0,26
**MVP**	8,03	8,29	-0,26
**RPS19BP1**	11,63	11,88	-0,26
**PRKAR1B**	10,09	10,35	-0,26
**HSD17B10**	12,32	12,58	-0,26
**MADD**	12,27	12,53	-0,26
**APOBEC3B**	7,26	7,52	-0,26
**EID1**	13,26	13,52	-0,26
**PPP6R1**	13,75	14,01	-0,26
**MMS19**	11,95	12,21	-0,26
**TTLL3**	10,72	10,97	-0,26
**RASSF7**	9,14	9,40	-0,26
**MCRIP2**	10,64	10,90	-0,26
**AKAP13**	12,22	12,48	-0,26
**LPAR1**	3,50	3,75	-0,26
**ZBTB7A**	11,95	12,21	-0,26
**ZNF48**	11,05	11,30	-0,26
**DDX11**	13,12	13,38	-0,26
**NLRP2**	7,34	7,60	-0,25
**CFAP44**	10,22	10,48	-0,25

**NBPF19**	11,60	11,85	-0,25
**PSMB6**	12,64	12,90	-0,25
**ETFB**	11,25	11,50	-0,25
**ZNF189**	10,70	10,95	-0,25
**SH3BP5L**	11,69	11,94	-0,25
**PDLIM2**	9,26	9,51	-0,25
**SET**	15,68	15,93	-0,25
**ORAI1**	10,42	10,67	-0,25
**LSM14B**	12,79	13,04	-0,25
**ALKBH4**	9,11	9,36	-0,25
**TBCB**	12,47	12,73	-0,25
**CLCF1**	4,87	5,13	-0,25
**LDLRAD2**	7,57	7,82	-0,25
**STAT2**	10,85	11,10	-0,25
**BATF**	6,21	6,46	-0,25
**NLRP1**	10,58	10,84	-0,25
**TRIM52**	10,83	11,09	-0,25
**MGMT**	10,92	11,18	-0,25
**AKR7A2**	11,47	11,72	-0,25
**CHN1**	8,16	8,41	-0,25
**RPL37**	15,21	15,46	-0,25
**FBXW5**	11,90	12,16	-0,25
**HUNK**	10,91	11,16	-0,25
**C12orf57**	13,20	13,45	-0,25
**AP001273.2**	8,86	9,12	-0,25
**SPACA9**	7,43	7,69	-0,25
**POLR2I**	11,07	11,32	-0,25
**CCDC86**	12,38	12,63	-0,25
**GIMAP4**	4,02	4,27	-0,25
**C14orf28**	7,46	7,71	-0,25
**ARMCX3**	11,58	11,83	-0,25
**TARS3**	9,61	9,87	-0,25
**CITED4**	10,37	10,62	-0,25
**CDIPT**	11,19	11,44	-0,25
**LRWD1**	10,93	11,18	-0,25
**C2orf74**	7,98	8,23	-0,25
**KCNH8**	6,05	6,30	-0,25
**IL12RB1**	7,19	7,44	-0,25
**BEX4**	10,36	10,61	-0,25
**TRIM35**	11,32	11,57	-0,25
**BABAM1**	11,80	12,05	-0,25
**CLN6**	11,58	11,83	-0,25
**DDAH2**	10,94	11,19	-0,25
**ANKRD39**	10,17	10,42	-0,25
**CYBA**	13,94	14,19	-0,25
**GRTP1**	6,36	6,61	-0,25
**VPS18**	11,61	11,86	-0,25
**NTHL1**	10,97	11,22	-0,25
**CLHC1**	8,26	8,51	-0,25
**VARS2**	11,87	12,12	-0,25
**ARF3**	13,75	14,00	-0,25
**IRX1**	10,95	11,20	-0,25
**CSRP2**	11,72	11,97	-0,25
**RPL35**	14,96	15,21	-0,25
**DDX49**	12,02	12,27	-0,25
**SUGP1**	10,98	11,23	-0,25

**PALM**	11,96	12,21	-0,25
**EIF1AD**	11,39	11,63	-0,25
**FAM228B**	7,09	7,33	-0,25
**TESK1**	10,78	11,03	-0,25
**PPP1R14B**	13,58	13,83	-0,25
**SLC35C2**	11,38	11,62	-0,25
**MYH9**	15,78	16,03	-0,25
**CMYA5**	4,44	4,69	-0,25
**CASP6**	10,54	10,79	-0,25
**NINJ2**	7,09	7,34	-0,25
**ANTKMT**	8,50	8,75	-0,25
**PTMA**	17,87	18,12	-0,25
**MRPL28**	12,21	12,45	-0,25
**SLIRP**	11,66	11,91	-0,25
**MRPS36**	9,98	10,23	-0,25
**ACBD6**	11,94	12,19	-0,25
**WDR55**	9,49	9,73	-0,25
**DUS3L**	11,87	12,12	-0,25
**ZNF526**	10,62	10,87	-0,25
**HCFC1R1**	9,95	10,20	-0,25
**SRPRB**	12,13	12,38	-0,25
**MAGOH**	12,38	12,62	-0,25
**ZNF428**	11,58	11,82	-0,25
**NOP16**	12,18	12,43	-0,25
**CASZ1**	9,60	9,85	-0,25
**HLA-F**	9,64	9,88	-0,25
**GPR83**	6,43	6,67	-0,25
**BAHCC1**	13,95	14,19	-0,25
**C11orf49**	9,82	10,06	-0,25
**TCF3**	15,01	15,25	-0,25
**BLOC1S3**	9,81	10,06	-0,25
**GORASP1**	10,68	10,93	-0,25
**SARDH**	9,61	9,86	-0,25
**EMC9**	9,26	9,50	-0,25
**GPRIN1**	11,96	12,21	-0,24
**FAM200B**	9,87	10,12	-0,24
**BTG2**	10,75	11,00	-0,24
**FAM174C**	10,50	10,75	-0,24
**CREM**	9,62	9,87	-0,24
**FBXL8**	7,97	8,22	-0,24
**DBN1**	14,29	14,54	-0,24
**C19orf25**	10,68	10,92	-0,24
**SLC27A3**	10,20	10,44	-0,24
**SHANK3**	10,41	10,65	-0,24
**CPNE1**	12,44	12,69	-0,24
**USP50**	2,53	2,78	-0,24
**SNRPD3**	13,70	13,95	-0,24
**DENND2D**	9,53	9,77	-0,24
**GHR**	4,97	5,21	-0,24
**LPCAT4**	10,51	10,76	-0,24
**PYCR3**	11,22	11,46	-0,24
**TONSL**	13,20	13,44	-0,24
**FITM2**	9,56	9,81	-0,24
**MCUR1**	9,71	9,95	-0,24
**POLR1B**	13,13	13,37	-0,24
**GRIPAP1**	11,26	11,50	-0,24

SUPT5H	13,15	13,40	-0,24
TEX9	7,03	7,27	-0,24
RSPH1	1,47	1,71	-0,24
MOB2	9,15	9,39	-0,24
DEXI	11,35	11,59	-0,24
FBXW4	10,06	10,31	-0,24
SERBP1	15,93	16,18	-0,24
SMAGP	7,92	8,17	-0,24
HLA-A	15,46	15,70	-0,24
GALNT1	10,82	11,07	-0,24
UBAP2L	14,51	14,75	-0,24
INA	7,70	7,94	-0,24
ABHD8	9,16	9,40	-0,24
LTO1	10,44	10,68	-0,24
HPS6	10,82	11,06	-0,24
ENDOG	10,33	10,58	-0,24
U2AF2	14,62	14,86	-0,24
NR2C2AP	10,71	10,95	-0,24
CHST3	5,90	6,14	-0,24
SESN3	7,82	8,06	-0,24
ANAPC15	10,57	10,81	-0,24
KCNJ14	8,29	8,53	-0,24
IMMP1L	9,67	9,91	-0,24
C11orf68	10,18	10,42	-0,24
RPLP2	15,35	15,60	-0,24
ZNF606	9,01	9,25	-0,24
SPTBN2	13,41	13,65	-0,24
SLC35B2	10,96	11,20	-0,24
SLC45A3	9,10	9,34	-0,24
EFCAB13	7,69	7,93	-0,24
CLIP2	11,07	11,31	-0,24
PLOD3	9,87	10,11	-0,24
IFFO1	10,21	10,45	-0,24
CNP	12,70	12,94	-0,24
CNDP2	13,67	13,90	-0,24
FBXO46	10,55	10,78	-0,24
PLEKHG6	1,67	1,91	-0,24
PCDHB4	1,67	1,91	-0,24
LHB	1,67	1,91	-0,24
CATIP	1,67	1,91	-0,24
FBLIM1	1,67	1,91	-0,24
OVCH2	1,67	1,91	-0,24
FAM229A	1,67	1,91	-0,24
IGKV1OR2-108	1,67	1,91	-0,24
WASHC1	10,94	11,18	-0,24
MVK	9,63	9,87	-0,24
SMIM8	9,33	9,56	-0,24
RPL17	13,39	13,63	-0,24
BORCS8	10,02	10,26	-0,24
TMOD4	4,56	4,80	-0,24
NDUFAF8	11,00	11,24	-0,24
ID2	7,95	8,19	-0,24
CLDN11	3,11	3,35	-0,24
ARMH2	3,11	3,35	-0,24
E4F1	11,02	11,26	-0,24
PLG	3,82	4,05	-0,24

**DNALI1**	3,82	4,05	-0,24
**TEX35**	3,82	4,05	-0,24
**HAUS5**	11,52	11,76	-0,24
**SLC6A4**	4,29	4,53	-0,24
**PROM2**	5,69	5,93	-0,24
**MBOAT4**	4,64	4,88	-0,24
**ATG7**	9,43	9,67	-0,24
**IFT27**	10,28	10,52	-0,24
**H1-10**	13,71	13,95	-0,24
**SERPINH1**	11,77	12,01	-0,24
**RPUSD4**	12,08	12,31	-0,24
**AMN**	7,43	7,66	-0,24
**ORMDL3**	10,97	11,20	-0,24
**CHMP6**	10,07	10,31	-0,24
**CBX1**	13,73	13,97	-0,24
**ITGB4**	6,83	7,07	-0,24
**RUSC2**	9,68	9,92	-0,24
**NDUFA1**	11,76	12,00	-0,24
**PARVA**	2,69	2,93	-0,24
**IHO1**	3,77	4,01	-0,24
**SLC17A9**	12,25	12,49	-0,24
**FOXRED2**	11,93	12,17	-0,24
**USP2**	8,84	9,08	-0,24
**NDUFA13**	12,59	12,83	-0,24
**POLG2**	10,94	11,18	-0,24
**GFOD2**	11,33	11,57	-0,24
**HELZ2**	7,07	7,30	-0,24
**CHRNA7**	9,88	10,11	-0,24
**PYM1**	10,54	10,77	-0,24
**USP27X**	8,71	8,95	-0,24
**USF2**	12,34	12,58	-0,24
**LDLRAP1**	11,21	11,45	-0,24
**MLLT10**	11,02	11,26	-0,24
**PPM1M**	9,31	9,55	-0,24
**BRD9**	12,05	12,28	-0,24
**TTC16**	7,16	7,39	-0,24
**IGF1**	2,92	3,16	-0,24
**TMEM170B**	9,40	9,64	-0,24
**TBC1D24**	11,92	12,15	-0,24
**BTBD6**	10,44	10,68	-0,24
**SRRM2**	15,93	16,17	-0,24
**NPY**	8,97	9,21	-0,24
**ROGDI**	8,59	8,83	-0,24
**EML1**	6,70	6,93	-0,24
**CLDN15**	9,72	9,96	-0,24
**MYO1G**	9,60	9,83	-0,24
**SPPL2B**	12,37	12,61	-0,24
**PPIP5K1**	11,70	11,93	-0,24
**ARL13B**	10,37	10,60	-0,24
**SPINT1**	10,53	10,77	-0,24
**PSMF1**	12,63	12,86	-0,23
**RASGRP2**	12,32	12,55	-0,23
**UQCRQ**	12,13	12,36	-0,23
**NLRP12**	6,88	7,11	-0,23
**TMEM234**	8,05	8,28	-0,23
**SUPT4H1**	12,31	12,54	-0,23

**OPLAH**	5,93	6,16	-0,23
**NISCH**	12,23	12,46	-0,23
**ADCY7**	9,99	10,22	-0,23
**SRSF9**	13,86	14,10	-0,23
**CSRP1**	12,47	12,71	-0,23
**PLS1**	8,07	8,30	-0,23
**ARHGAP30**	13,05	13,29	-0,23
**FUT1**	7,27	7,51	-0,23
**SMIM10L2A**	6,68	6,91	-0,23
**NOL3**	8,66	8,89	-0,23
**PRRC2A**	14,91	15,14	-0,23
**OPA3**	11,03	11,27	-0,23
**HIC2**	11,21	11,44	-0,23
**APOL3**	11,29	11,52	-0,23
**COX17**	10,16	10,39	-0,23
**ARPC4-TTLL3**	8,05	8,29	-0,23
**MZF1**	9,71	9,95	-0,23
**BNIP3L**	11,57	11,80	-0,23
**CACNG4**	11,59	11,82	-0,23
**ATAD3A**	12,37	12,60	-0,23
**ZNF629**	12,07	12,30	-0,23
**KIF5C**	9,00	9,23	-0,23
**KIF1C**	11,65	11,88	-0,23
**PGAP3**	9,13	9,37	-0,23
**MAPK8IP2**	5,29	5,52	-0,23
**CRIP1**	10,25	10,49	-0,23
**GTF2H4**	10,99	11,22	-0,23
**SNX15**	10,05	10,28	-0,23
**RTL10**	11,99	12,22	-0,23
**TUBB4B**	14,86	15,09	-0,23
**RIPK3**	8,21	8,44	-0,23
**ACSBG2**	4,90	5,14	-0,23
**CYB5D1**	10,55	10,78	-0,23
**MAP3K10**	9,40	9,63	-0,23
**RNF113A**	10,75	10,98	-0,23
**DECR2**	10,12	10,35	-0,23
**PRDM8**	8,95	9,18	-0,23
**TET1**	6,12	6,35	-0,23
**THUMPD3**	12,05	12,28	-0,23
**ZFP41**	10,63	10,86	-0,23
**CUX2**	5,07	5,31	-0,23
**IKBKG**	10,25	10,48	-0,23
**ACY1**	9,80	10,04	-0,23
**CCDC34**	11,37	11,60	-0,23
**PPP1R18**	13,02	13,26	-0,23
**ERGIC1**	13,77	14,00	-0,23
**DOK3**	13,71	13,94	-0,23
**PDGFA**	6,57	6,80	-0,23
**TBC1D22A**	10,66	10,89	-0,23
**NRARP**	9,06	9,29	-0,23
**UIMC1**	11,65	11,88	-0,23
**DZIP1L**	5,83	6,06	-0,23
**H4C11**	6,48	6,71	-0,23
**RPS21**	14,27	14,50	-0,23
**SCRIB**	13,51	13,74	-0,23
**FOXP4**	11,08	11,31	-0,23

**PFKL**	13,16	13,39	-0,23
**SLC38A10**	11,83	12,06	-0,23
**LY6G5B**	10,57	10,80	-0,23
**LRPAP1**	11,91	12,14	-0,23
**UBTD1**	6,29	6,52	-0,23
**ACYP1**	9,87	10,10	-0,23
**TCEA2**	8,81	9,04	-0,23
**GPS2**	11,40	11,63	-0,23
**LRRC14B**	12,89	13,12	-0,23
**SRF**	12,05	12,28	-0,23
**MBNL2**	8,72	8,95	-0,23
**ZDHHC14**	8,65	8,88	-0,23
**TOR4A**	8,29	8,52	-0,23
**TLE5**	14,21	14,44	-0,23
**MRPL14**	11,79	12,02	-0,23
**ZNF222**	7,95	8,18	-0,23
**DUSP19**	7,83	8,06	-0,23
**MCTP1**	2,05	2,27	-0,23
**EHD3**	8,26	8,49	-0,23
**IGHV3-33**	7,73	7,96	-0,23
**MXD4**	10,19	10,42	-0,23
**FAM72D**	10,58	10,81	-0,23
**IFI27**	4,19	4,42	-0,23
**CGRRF1**	8,81	9,04	-0,23
**SMG5**	13,59	13,82	-0,23
**NPR1**	6,08	6,30	-0,23
**SIRT7**	11,08	11,31	-0,23
**SEMA6A**	7,32	7,54	-0,23
**RNF40**	12,99	13,22	-0,23
**ZNF324**	9,99	10,22	-0,23
**RAB11B**	11,79	12,01	-0,23
**ZNF816-ZNF321P**	8,40	8,63	-0,23
**MYPOP**	9,73	9,95	-0,23
**CREB3L4**	10,84	11,07	-0,23
**KLHDC4**	11,95	12,18	-0,23
**RIN2**	9,87	10,09	-0,23
**CCDC121**	7,76	7,98	-0,23
**GNA15**	11,91	12,14	-0,23
**COL27A1**	12,53	12,75	-0,23
**CHAF1A**	13,13	13,36	-0,23
**RPS27**	15,45	15,67	-0,23
**OAZ2**	11,77	12,00	-0,23
**STK25**	12,90	13,12	-0,23
**TMEM44**	10,04	10,26	-0,23
**NDUFV1**	13,18	13,41	-0,23
**NME4**	12,99	13,22	-0,23
**TMEM259**	12,68	12,91	-0,23
**FLYWCH2**	10,21	10,43	-0,23
**HDAC5**	12,76	12,99	-0,23
**GATB**	10,65	10,88	-0,23
**TCAP**	4,12	4,34	-0,23
**EZR**	13,65	13,88	-0,23
**BANF1**	13,38	13,61	-0,22
**PLAGL1**	8,70	8,93	-0,22
**ABCA5**	9,36	9,58	-0,22
**DOLPP1**	10,65	10,88	-0,22

**ACTR1B**	11,37	11,59	-0,22
**EXOSC7**	11,53	11,75	-0,22
**COG4**	12,01	12,23	-0,22
**NT5DC1**	11,23	11,45	-0,22
**RAB1B**	12,91	13,13	-0,22
**CLDN12**	7,77	8,00	-0,22
**WNK3**	4,98	5,20	-0,22
**CENPS**	10,71	10,93	-0,22
**CIB2**	7,78	8,00	-0,22
**AEBP2**	12,16	12,38	-0,22
**TBCEL**	10,12	10,34	-0,22
**ZNF425**	8,71	8,93	-0,22
**HNRNPA2B1**	17,01	17,24	-0,22
**NCS1**	10,83	11,05	-0,22
**EMC4**	11,90	12,12	-0,22
**HOXA1**	3,08	3,30	-0,22
**NR1I3**	4,29	4,52	-0,22
**TIMM17B**	11,36	11,59	-0,22
**MRM3**	10,65	10,87	-0,22
**PAK1**	12,72	12,94	-0,22
**CTDSP1**	12,12	12,35	-0,22
**ZFP90**	11,25	11,47	-0,22
**CCDC97**	11,65	11,87	-0,22
**PHYHIP**	3,95	4,17	-0,22
**SUFU**	10,68	10,90	-0,22
**AMPD2**	12,12	12,35	-0,22
**HLA-DOA**	11,24	11,46	-0,22
**LETM1**	12,35	12,57	-0,22
**ARHGEF18**	12,58	12,80	-0,22
**FBF1**	9,21	9,43	-0,22
**TAF7L**	4,73	4,95	-0,22
**TMEM42**	8,49	8,72	-0,22
**PPM1G**	13,93	14,16	-0,22
**MICAL1**	13,26	13,48	-0,22
**AKAP8**	12,10	12,32	-0,22
**LYL1**	10,38	10,61	-0,22
**PLD3**	10,24	10,47	-0,22
**CES2**	11,69	11,91	-0,22
**ALAD**	10,58	10,80	-0,22
**COQ7**	10,52	10,74	-0,22
**HSPA2**	4,70	4,92	-0,22
**TMSB10**	15,52	15,74	-0,22
**DCTPP1**	12,98	13,21	-0,22
**STRIP1**	11,40	11,62	-0,22
**GAS8**	10,40	10,62	-0,22
**MGAT1**	12,44	12,66	-0,22
**AKT2**	13,00	13,22	-0,22
**DDA1**	11,59	11,81	-0,22
**RALY**	13,80	14,02	-0,22
**LARP1**	13,48	13,70	-0,22
**RRP7A**	13,42	13,64	-0,22
**AVEN**	9,98	10,19	-0,22
**NDUFA3**	11,06	11,28	-0,22
**HSPA12B**	8,13	8,35	-0,22
**C12orf76**	3,57	3,79	-0,22
**MTRNR2L1**	3,57	3,79	-0,22

**AL133500.1**	3,57	3,79	-0,22
**SMAD6**	3,57	3,79	-0,22
**MAP11**	10,05	10,27	-0,22
**PLCB3**	11,44	11,66	-0,22
**PKD1**	14,32	14,54	-0,22
**KLHL33**	11,38	11,60	-0,22
**SAFB2**	12,55	12,76	-0,22
**CNTNAP1**	8,88	9,10	-0,22
**XRCC1**	11,52	11,74	-0,22
**RPL37A**	15,72	15,94	-0,22
**MACROH2A2**	12,37	12,59	-0,22
**PRPF6**	13,25	13,47	-0,22
**FBXO28**	11,82	12,04	-0,22
**NUBP1**	10,92	11,13	-0,22
**MTUS2**	5,85	6,07	-0,22
**AC073610.1**	5,85	6,07	-0,22
**RPL22**	15,64	15,86	-0,22
**F2RL3**	8,73	8,95	-0,22
**AIF1L**	9,27	9,49	-0,22
**TMIE**	7,56	7,77	-0,22
**NGRN**	11,80	12,02	-0,22
**PLCG1**	13,14	13,36	-0,22
**CCDC28B**	9,60	9,82	-0,22
**IKBKB**	12,68	12,90	-0,22
**SERPINF2**	5,49	5,70	-0,22
**PSMC3**	13,74	13,95	-0,22
**ATG16L1**	10,73	10,95	-0,22
**TBCE**	10,19	10,41	-0,22
**PRPF31**	12,56	12,77	-0,22
**FAM102A**	10,41	10,63	-0,22
**SERGEF**	9,57	9,78	-0,22
**RAB40C**	10,00	10,21	-0,22
**SMIM15**	11,38	11,60	-0,22
**PIF1**	10,10	10,31	-0,22
**RAD51D**	11,94	12,16	-0,22
**CD19**	13,41	13,63	-0,22
**PCDHB13**	7,11	7,33	-0,22
**TMEM18**	11,36	11,58	-0,22
**LMF2**	11,49	11,71	-0,22
**LY6E**	13,11	13,33	-0,22
**AARS2**	11,77	11,99	-0,22
**BPNT1**	11,56	11,77	-0,22
**ALDH1L2**	10,33	10,55	-0,22
**TMED9**	12,60	12,82	-0,22
**MVB12A**	10,14	10,35	-0,22
**CTDP1**	11,31	11,52	-0,22
**DENND4B**	12,87	13,08	-0,22
**POLR3K**	12,31	12,53	-0,22
**PNMA6A**	6,66	6,88	-0,22
**OSCP1**	6,41	6,63	-0,22
**ZNF808**	10,63	10,84	-0,22
**CALM3**	14,48	14,69	-0,22
**SELENOH**	12,21	12,42	-0,22
**APEX1**	14,91	15,12	-0,22
**DCTD**	12,87	13,09	-0,22
**JOSD2**	6,95	7,17	-0,21

**C9orf16**	10,84	11,06	-0,21
**DNPEP**	12,58	12,80	-0,21
**GFUS**	12,32	12,54	-0,21
**TOMM7**	11,88	12,09	-0,21
**SPR**	10,25	10,47	-0,21
**TNIP1**	11,82	12,04	-0,21
**CERS3**	2,45	2,67	-0,21
**LSM10**	10,51	10,72	-0,21
**DGCR8**	12,92	13,13	-0,21
**P2RX5**	9,55	9,76	-0,21
**POLD1**	13,10	13,31	-0,21
**SLC38A7**	10,31	10,53	-0,21
**SHISAL2A**	8,29	8,50	-0,21
**UPF3B**	11,85	12,07	-0,21
**SLU7**	12,05	12,26	-0,21
**NDUFB3**	11,60	11,81	-0,21
**PARD6G**	8,75	8,96	-0,21
**PLPBP**	13,00	13,21	-0,21
**ATXN2L**	13,80	14,02	-0,21
**PACSIN2**	12,49	12,70	-0,21
**ABR**	3,65	3,86	-0,21
**DYRK2**	12,89	13,11	-0,21
**RPL39**	14,99	15,20	-0,21
**TRAFD1**	11,82	12,03	-0,21
**ZNF343**	10,61	10,82	-0,21
**PCDHB14**	8,07	8,28	-0,21
**MTHFD2L**	6,63	6,84	-0,21
**TRIM11**	11,53	11,75	-0,21
**HMBS**	11,34	11,55	-0,21
**RAB9B**	9,38	9,59	-0,21
**C2CD2L**	9,81	10,02	-0,21
**IFITM2**	13,07	13,28	-0,21
**RNASEK**	11,32	11,53	-0,21
**MED11**	9,01	9,23	-0,21
**CDC26**	10,74	10,95	-0,21
**RELT**	11,52	11,73	-0,21
**MAN2B2**	10,72	10,93	-0,21
**RGMA**	11,36	11,58	-0,21
**LAS1L**	13,08	13,29	-0,21
**TLR9**	7,30	7,51	-0,21
**H2AX**	9,95	10,16	-0,21
**TBC1D10A**	9,27	9,48	-0,21
**USP20**	10,36	10,58	-0,21
**SAPCD2**	11,89	12,10	-0,21
**MMACHC**	10,33	10,54	-0,21
**NUMA1**	14,61	14,82	-0,21
**ARSA**	9,83	10,04	-0,21
**GNAT2**	4,04	4,25	-0,21
**PTBP1**	15,46	15,67	-0,21
**CYHR1**	11,04	11,25	-0,21
**SEC11C**	11,18	11,39	-0,21
**IFI27L1**	8,16	8,37	-0,21
**STK11**	12,19	12,40	-0,21
**AMN1**	8,73	8,94	-0,21
**PIWIL4**	6,35	6,56	-0,21
**NDUFS3**	12,37	12,58	-0,21

**AC024592.3**	5,18	5,39	-0,21
**IRAG1**	5,95	6,16	-0,21
**ILVBL**	10,79	10,99	-0,21
**GDPD1**	8,72	8,93	-0,21
**NUDT11**	11,80	12,01	-0,21
**ATP5MC1**	12,76	12,97	-0,21
**CHST14**	10,34	10,55	-0,21
**GMPPB**	11,61	11,82	-0,21
**ATP5F1D**	11,87	12,08	-0,21
**ZNF750**	3,92	4,13	-0,21
**COPS9**	10,89	11,10	-0,21
**XPNPEP3**	10,98	11,19	-0,21
**TIGD6**	8,38	8,59	-0,21
**KCNK12**	10,65	10,85	-0,21
**TRMT1**	12,24	12,44	-0,21
**PHF2**	12,06	12,27	-0,21
**SEMA4G**	7,83	8,04	-0,21
**AP5Z1**	10,85	11,05	-0,21
**KTI12**	10,60	10,81	-0,21
**CAMKK1**	8,92	9,13	-0,21
**EID2**	10,28	10,49	-0,21
**NCAPH2**	12,26	12,47	-0,21
**ARFGEF3**	5,48	5,69	-0,21
**PRKD2**	10,22	10,43	-0,21
**AHCTF1**	13,81	14,02	-0,21
**PCTP**	10,15	10,36	-0,21
**MEPCE**	12,38	12,59	-0,21
**GSK3A**	12,23	12,43	-0,21
**MEA1**	11,34	11,54	-0,21
**ABHD17A**	11,22	11,42	-0,21
**STAT6**	13,22	13,42	-0,21
**SBNO2**	11,50	11,71	-0,21
**ZNF362**	11,35	11,55	-0,21
**DGKZ**	12,67	12,87	-0,21
**TTC5**	10,86	11,07	-0,21
**SPATA24**	8,48	8,68	-0,21
**AC007326.4**	7,18	7,38	-0,21
**TMEM80**	7,91	8,12	-0,21
**TWNK**	12,26	12,47	-0,21
**ZNF443**	8,83	9,03	-0,21
**LZTS2**	11,95	12,16	-0,21
**TRIM46**	9,81	10,02	-0,21
**APRT**	12,69	12,89	-0,21
**COX11**	11,37	11,58	-0,21
**TMEM140**	9,80	10,01	-0,21
**TMC6**	12,34	12,55	-0,21
**NXT1**	11,27	11,47	-0,21
**MRPL52**	11,20	11,40	-0,21
**FAU**	14,51	14,71	-0,21
**CBFB**	14,15	14,35	-0,21
**RAB13**	10,38	10,59	-0,21
**C20orf27**	12,21	12,42	-0,21
**CHMP4B**	12,15	12,36	-0,21
**TGFB1**	11,97	12,17	-0,21
**DIABLO**	11,59	11,79	-0,21
**HMGA1**	15,72	15,93	-0,21

**NUDCD2**	11,34	11,55	-0,21
**TAF6L**	10,53	10,74	-0,20
**FMR1**	9,20	9,40	-0,20
**ZDHHC2**	12,75	12,95	-0,20
**FBXO22**	12,14	12,35	-0,20
**SPON2**	6,44	6,64	-0,20
**PER1**	11,32	11,53	-0,20
**ZNF487**	6,21	6,41	-0,20
**RBM4B**	11,27	11,48	-0,20
**NTN1**	5,90	6,11	-0,20
**PSEN1**	11,14	11,35	-0,20
**DGCR2**	12,00	12,21	-0,20
**ICOSLG**	10,27	10,48	-0,20
**PHKA1**	2,69	2,89	-0,20
**AL928654.3**	2,69	2,89	-0,20
**ZNF2**	9,09	9,29	-0,20
**RPL36AL**	13,19	13,39	-0,20
**TIMM8B**	11,42	11,62	-0,20
**MATR3**	13,98	14,18	-0,20
**PTS**	9,80	10,00	-0,20
**KLHDC1**	6,53	6,73	-0,20
**SIGMAR1**	12,52	12,73	-0,20
**SCART1**	6,39	6,59	-0,20
**PHF5A**	11,90	12,11	-0,20
**AC006538.2**	7,44	7,64	-0,20
**PSMB10**	12,48	12,68	-0,20
**STK17A**	11,25	11,46	-0,20
**SPATA9**	5,71	5,92	-0,20
**BSDC1**	11,46	11,66	-0,20
**TACO1**	10,64	10,84	-0,20
**PAFAH1B3**	11,52	11,72	-0,20
**LYRM9**	7,03	7,23	-0,20
**ZFAND4**	8,47	8,67	-0,20
**MTLN**	9,14	9,34	-0,20
**NFATC4**	11,30	11,50	-0,20
**VPS37D**	7,17	7,37	-0,20
**MRPL34**	11,00	11,20	-0,20
**ARMH1**	7,69	7,89	-0,20
**ITGB3**	7,83	8,03	-0,20
**ARID3A**	9,94	10,14	-0,20
**HSBP1**	11,12	11,32	-0,20
**SIPA1**	12,06	12,26	-0,20
**BMP6**	4,93	5,13	-0,20
**H2AC17**	4,93	5,13	-0,20
**TRMT44**	9,71	9,91	-0,20
**VCPKMT**	9,86	10,07	-0,20
**MTFR1L**	10,82	11,02	-0,20
**PTPMT1**	11,65	11,85	-0,20
**ZNF622**	11,16	11,36	-0,20
**PAIP2B**	7,34	7,54	-0,20
**MAFG**	12,78	12,98	-0,20
**GAS7**	11,67	11,87	-0,20
**ALYREF**	14,04	14,24	-0,20
**MANF**	12,60	12,80	-0,20
**CHD3**	14,66	14,86	-0,20
**AUTS2**	14,14	14,34	-0,20

**NEURL4**	11,46	11,66	-0,20
**PPT2**	10,31	10,51	-0,20
**UBFD1**	12,91	13,11	-0,20
**RAB15**	10,90	11,10	-0,20
**RCOR2**	10,80	11,00	-0,20
**SEPSECS**	10,56	10,76	-0,20
**MFN2**	13,00	13,20	-0,20
**OSBPL1A**	11,22	11,41	-0,20
**EPHB4**	12,23	12,43	-0,20
**AXIN2**	8,83	9,03	-0,20
**EHMT2**	13,18	13,38	-0,20
**WWOX**	9,97	10,17	-0,20
**UBAC1**	11,56	11,76	-0,20
**QPRT**	10,80	11,00	-0,20
**ZBTB42**	8,80	9,00	-0,20
**STK40**	11,84	12,03	-0,20
**ADAMTS7**	11,42	11,62	-0,20
**ZNF282**	11,47	11,67	-0,20
**TRIM65**	12,16	12,36	-0,20
**UHRF1**	16,06	16,26	-0,20
**LDB1**	13,14	13,34	-0,20
**SLC25A51**	9,21	9,40	-0,20
**TNFRSF14**	7,00	7,19	-0,20
**TIMM13**	12,69	12,89	-0,20
**ZNF778**	10,10	10,30	-0,20
**MLYCD**	10,15	10,35	-0,20
**KHNYN**	12,53	12,73	-0,20
**IDH2**	14,80	14,99	-0,20
**NOS3**	9,25	9,45	-0,20
**CLEC16A**	11,60	11,80	-0,20
**UBAP1**	11,56	11,76	-0,20
**EBNA1BP2**	13,60	13,79	-0,20
**CPTP**	9,62	9,81	-0,20
**ATP6V1FNB**	5,93	6,13	-0,20
**SYNE3**	12,97	13,17	-0,20
**AKAP8L**	11,63	11,82	-0,20
**BMP8A**	6,99	7,19	-0,20
**RAB8B**	13,13	13,33	-0,20
**CD72**	10,89	11,09	-0,20
**ZNF432**	10,65	10,84	-0,20
**SNURF**	7,23	7,43	-0,20
**BAX**	12,41	12,61	-0,20
**SH2D4B**	10,24	10,44	-0,20
**CLDN23**	4,44	4,64	-0,20
**CLEC14A**	11,75	11,94	-0,20
**CCDC22**	10,44	10,64	-0,20
**SYVN1**	12,55	12,74	-0,20
**CTDNEP1**	13,11	13,30	-0,20
**RPL17-C18orf32**	11,21	11,41	-0,20
**NOP9**	12,63	12,83	-0,20
**NSDHL**	10,39	10,58	-0,20
**RASSF10**	10,35	10,54	-0,19
**SLC16A13**	8,05	8,24	-0,19
**PEDS1**	11,15	11,35	-0,19
**BCL3**	8,59	8,78	-0,19
**AP2M1**	13,62	13,82	-0,19

**TBC1D3L**	9,81	10,01	-0,19
**LENG8**	14,17	14,37	-0,19
**SOX11**	13,29	13,49	-0,19
**RDH13**	9,78	9,98	-0,19
**TECPR1**	10,50	10,70	-0,19
**UPF1**	13,52	13,71	-0,19
**TAF10**	11,12	11,32	-0,19
**PSMD3**	13,71	13,90	-0,19
**MAP1S**	11,89	12,08	-0,19
**PJVK**	7,77	7,96	-0,19
**LSS**	11,93	12,12	-0,19
**BHLHA15**	7,28	7,48	-0,19
**C2orf81**	5,33	5,52	-0,19
**B4GALNT3**	7,80	8,00	-0,19
**CAPN14**	6,31	6,51	-0,19
**MAP4**	13,75	13,94	-0,19
**CBS**	11,18	11,37	-0,19
**PAM16**	10,53	10,73	-0,19
**RABL3**	11,55	11,75	-0,19
**TNPO1**	12,64	12,83	-0,19
**COMMD4**	11,41	11,60	-0,19
**FBXL19**	12,45	12,64	-0,19
**ZFYVE27**	11,24	11,43	-0,19
**ATG16L2**	10,68	10,88	-0,19
**TXNDC17**	11,30	11,50	-0,19
**ELAPOR2**	8,89	9,09	-0,19
**MCM5**	13,99	14,18	-0,19
**GAK**	12,33	12,52	-0,19
**HCN3**	8,87	9,06	-0,19
**SAT2**	10,13	10,32	-0,19
**PDP2**	12,29	12,48	-0,19
**RAP1GAP2**	12,86	13,05	-0,19
**GOLIM4**	12,55	12,75	-0,19
**LAMTOR1**	11,18	11,37	-0,19
**GET3**	11,88	12,08	-0,19
**AP1M1**	12,07	12,26	-0,19
**MED25**	11,89	12,08	-0,19
**TUSC2**	10,94	11,13	-0,19
**SETD9**	9,13	9,32	-0,19
**DNAJC30**	9,93	10,13	-0,19
**DHX58**	8,73	8,92	-0,19
**R3HCC1**	12,04	12,23	-0,19
**ZNF101**	11,26	11,45	-0,19
**STAB2**	3,48	3,67	-0,19
**RPL41**	16,42	16,61	-0,19
**KDSR**	11,60	11,80	-0,19
**EIF3G**	13,56	13,75	-0,19
**CIAO3**	12,37	12,57	-0,19
**CPNE9**	3,41	3,61	-0,19
**NUDT22**	10,18	10,37	-0,19
**NPHP3-ACAD11**	8,89	9,08	-0,19
**SARM1**	11,08	11,27	-0,19
**ADAMTS18**	3,28	3,47	-0,19
**PSTK**	8,09	8,29	-0,19
**ZNF543**	8,86	9,06	-0,19
**FLAD1**	12,25	12,44	-0,19

**DNM2**	13,11	13,30	-0,19
**TIMP1**	7,05	7,24	-0,19
**TNFRSF1B**	9,04	9,23	-0,19
**TSTD1**	10,59	10,78	-0,19
**VAV2**	11,21	11,40	-0,19
**SLFN14**	6,27	6,46	-0,19
**AUH**	9,31	9,50	-0,19
**NDUFB10**	12,61	12,80	-0,19
**ARPC5**	13,92	14,11	-0,19
**TAFA5**	6,13	6,32	-0,19
**RPS17**	15,90	16,09	-0,19
**PUF60**	13,95	14,14	-0,19
**NKD2**	10,04	10,23	-0,19
**KLHL18**	11,33	11,52	-0,19
**TEX261**	12,45	12,64	-0,19
**CACNB3**	11,53	11,72	-0,19
**PLEKHM2**	11,97	12,16	-0,19
**SAC3D1**	10,57	10,76	-0,19
**HM13**	12,27	12,46	-0,19
**SDK2**	8,99	9,18	-0,19
**POMZP3**	6,64	6,83	-0,19
**PIP5K1C**	11,29	11,48	-0,19
**SLC7A5**	14,91	15,10	-0,19
**CHRFAM7A**	6,72	6,91	-0,19
**AFAP1L1**	10,19	10,38	-0,19
**SMC1A**	14,62	14,81	-0,19
**RPL11**	16,10	16,29	-0,19
**CASKIN2**	11,65	11,84	-0,19
**TAF1C**	12,30	12,49	-0,19
**ZNF133**	8,94	9,13	-0,19
**SYCE2**	8,02	8,21	-0,19
**URGCP**	11,43	11,62	-0,19
**CMC1**	10,31	10,50	-0,19
**SPRING1**	12,17	12,36	-0,19
**NTN3**	6,03	6,22	-0,19
**TMEM201**	12,12	12,31	-0,19
**TCIRG1**	12,39	12,58	-0,19
**P2RX1**	11,74	11,93	-0,19
**ZNF263**	12,21	12,40	-0,19
**PRX**	7,85	8,04	-0,19
**DCAF5**	11,63	11,82	-0,19
**NR1D1**	9,59	9,78	-0,19
**SIK1**	10,32	10,51	-0,19
**HARS1**	12,25	12,43	-0,19
**BRI3**	10,80	10,99	-0,19
**JAG2**	12,67	12,86	-0,19
**NUTM2D**	8,85	9,04	-0,19
**ATE1**	10,60	10,78	-0,19
**TAZ**	10,64	10,83	-0,19
**DENR**	13,45	13,64	-0,19
**GTF3C5**	12,71	12,90	-0,19
**TRIM58**	10,20	10,39	-0,19
**H2AC8**	5,64	5,83	-0,19
**TAP2**	12,80	12,99	-0,19
**ZNF516**	11,26	11,45	-0,19
**AC026464.6**	9,35	9,54	-0,19

OGFOD1	12,74	12,93	-0,19
BTN3A3	10,63	10,82	-0,19
TSKU	10,06	10,25	-0,19
RAB3IP	11,06	11,25	-0,19
GSTK1	11,93	12,11	-0,19
ADAM11	7,60	7,79	-0,19
KYAT1	9,66	9,85	-0,19
TTYH3	14,00	14,19	-0,19
C10orf143	8,42	8,61	-0,19
GLYCTK	9,24	9,43	-0,19
CCDC81	8,16	8,35	-0,19
KIAA2013	11,90	12,09	-0,19
FMC1	9,16	9,35	-0,19
FOXA2	5,04	5,23	-0,19
KRR1	12,82	13,01	-0,19
LRRC59	13,81	14,00	-0,19
RPS6KB2	11,41	11,60	-0,19
KBTBD8	10,25	10,43	-0,19
RAB19	1,69	1,88	-0,19
SAMD4A	11,08	11,26	-0,19
RPS28	14,50	14,69	-0,19
ADGRL1	11,12	11,31	-0,19
EIF2B4	11,23	11,42	-0,19
C21orf58	11,76	11,95	-0,19
CD28	5,12	5,31	-0,19
SLFN12	5,82	6,01	-0,19
STRC	7,17	7,36	-0,19
COX19	11,72	11,90	-0,19
LIN37	8,56	8,74	-0,19
LINGO3	8,75	8,93	-0,19
CDK5R1	10,69	10,88	-0,19
VPS26C	11,45	11,63	-0,19
HNRNPC	16,16	16,35	-0,19
RBM24	4,96	5,15	-0,19
ARAP1	12,04	12,22	-0,19
SLC2A4RG	11,12	11,31	-0,19
PLIN4	7,40	7,58	-0,19
KLRG1	6,84	7,03	-0,19
XPC	11,30	11,48	-0,19
ABCB9	10,26	10,44	-0,18
PQBP1	11,47	11,66	-0,18
AEN	12,41	12,59	-0,18
DCAF11	12,03	12,22	-0,18
TMEM101	9,76	9,94	-0,18
CIAO2B	11,80	11,98	-0,18
DTNB	9,73	9,91	-0,18
C1orf131	11,71	11,89	-0,18
HECTD1	11,58	11,76	-0,18
JMJD8	11,85	12,03	-0,18
MAN1B1	8,98	9,16	-0,18
SESN2	10,24	10,42	-0,18
CCDC106	10,10	10,29	-0,18
AL136295.4	7,82	8,00	-0,18
DKK1	6,41	6,59	-0,18
ICA1L	9,71	9,89	-0,18
RPS19	15,84	16,03	-0,18

**ZBED6**	11,54	11,73	-0,18
**PNPLA8**	10,18	10,37	-0,18
**ECD**	11,71	11,89	-0,18
**ACAA1**	11,33	11,51	-0,18
**SHISA5**	12,17	12,36	-0,18
**INTS6**	12,54	12,72	-0,18
**PIM1**	8,99	9,17	-0,18
**MKX**	8,53	8,71	-0,18
**COX6B1**	13,01	13,20	-0,18
**STK10**	12,71	12,89	-0,18
**RGL2**	10,56	10,74	-0,18
**PRPF38B**	13,59	13,77	-0,18
**DPH2**	12,12	12,30	-0,18
**SLC1A4**	12,40	12,59	-0,18
**VAV1**	12,53	12,71	-0,18
**IGLL1**	14,72	14,90	-0,18
**CA5B**	9,24	9,42	-0,18
**HLF**	3,49	3,67	-0,18
**DALRD3**	9,98	10,17	-0,18
**ZNF717**	8,29	8,48	-0,18
**OSBPL6**	7,09	7,28	-0,18
**ATP5F1E**	12,49	12,67	-0,18
**FBXO31**	11,71	11,89	-0,18
**RPGR**	10,25	10,43	-0,18
**PLBD2**	10,45	10,64	-0,18
**THBS4**	7,44	7,62	-0,18
**GUCD1**	12,72	12,90	-0,18
**HTATSF1**	13,03	13,21	-0,18
**LRGUK**	6,44	6,62	-0,18
**NELFA**	11,96	12,14	-0,18
**CHID1**	11,36	11,54	-0,18
**NDUFS7**	12,06	12,24	-0,18
**ZBTB14**	11,11	11,30	-0,18
**NBPF12**	11,50	11,68	-0,18
**DNAI4**	7,33	7,51	-0,18
**MPLKIP**	11,46	11,65	-0,18
**NAGLU**	9,84	10,02	-0,18
**LIME1**	9,28	9,46	-0,18
**LMO4**	11,64	11,83	-0,18
**DVL2**	11,87	12,05	-0,18
**FLT4**	4,02	4,20	-0,18
**AC079741.2**	4,02	4,20	-0,18
**ORC5**	11,25	11,43	-0,18
**U2AF1L5**	13,81	13,99	-0,18
**RGS4**	5,48	5,66	-0,18
**TMEM147**	11,38	11,56	-0,18
**ARMC6**	12,19	12,37	-0,18
**MYO18A**	14,42	14,60	-0,18
**LXN**	8,80	8,98	-0,18
**VPS33B**	9,13	9,32	-0,18
**RBM15B**	13,70	13,88	-0,18
**SLC35A4**	10,12	10,30	-0,18
**LIMK2**	11,97	12,15	-0,18
**MRPL21**	11,03	11,21	-0,18
**RAB5IF**	12,28	12,46	-0,18
**DNAJC17**	9,42	9,60	-0,18

**NPIPB12**	11,71	11,89	-0,18
**SLC25A28**	10,51	10,69	-0,18
**C17orf49**	10,96	11,14	-0,18
**PNPLA7**	8,24	8,42	-0,18
**TMEM187**	7,95	8,13	-0,18
**ARMH3**	11,04	11,23	-0,18
**UBQLN4**	13,15	13,33	-0,18
**MTSS2**	12,23	12,41	-0,18
**GNAS**	15,02	15,20	-0,18
**MPEG1**	5,68	5,86	-0,18
**PGAM5**	12,95	13,13	-0,18
**INTS1**	13,58	13,76	-0,18
**FAM209A**	4,29	4,47	-0,18
**REEP4**	11,68	11,86	-0,18
**TACC3**	13,64	13,82	-0,18
**MRGBP**	11,22	11,40	-0,18
**UBE2Z**	12,29	12,47	-0,18
**GRPEL1**	12,21	12,39	-0,18
**FARS2**	10,02	10,20	-0,18
**LRTOMT**	7,27	7,45	-0,18
**RNF187**	13,24	13,41	-0,18
**PPIL6**	6,16	6,34	-0,18
**PRKCA**	10,47	10,65	-0,18
**LRFN4**	11,22	11,40	-0,18
**DLEC1**	5,68	5,86	-0,18
**MKNK2**	13,45	13,63	-0,18
**RANGRF**	11,06	11,24	-0,18
**ASL**	9,23	9,41	-0,18
**MED29**	11,76	11,94	-0,18
**CDK9**	12,47	12,65	-0,18
**SCAMP4**	11,02	11,20	-0,18
**CDC42EP4**	9,16	9,34	-0,18
**IGSF9**	10,60	10,78	-0,18
**MIF**	14,55	14,73	-0,18
**DDX27**	12,76	12,94	-0,18
**SMARCB1**	13,82	13,99	-0,18
**ANKRD11**	14,35	14,53	-0,18
**HSD17B8**	7,94	8,12	-0,18
**SLC5A11**	4,22	4,39	-0,18
**GOLGA6L4**	7,96	8,14	-0,18
**ORC3**	10,34	10,51	-0,18
**TMPRSS6**	4,51	4,69	-0,18
**DEAF1**	11,63	11,81	-0,18
**NRIP2**	7,76	7,94	-0,18
**GMEB1**	10,86	11,04	-0,18
**CEACAM21**	9,70	9,88	-0,18
**FZD8**	8,88	9,06	-0,18
**SOAT1**	12,15	12,32	-0,18
**PCYT1A**	11,83	12,01	-0,18
**HSPE1**	14,51	14,69	-0,18
**TMEM221**	7,88	8,06	-0,18
**ATP13A2**	10,98	11,16	-0,18
**RBM8A**	14,30	14,47	-0,18
**ISG15**	9,28	9,46	-0,18
**H2AZ2**	14,42	14,59	-0,18
**DOK1**	9,87	10,04	-0,18

**NTMT1**	10,72	10,89	-0,18
**MAN2C1**	11,37	11,54	-0,18
**ARL9**	9,79	9,97	-0,18
**TMEM92**	4,70	4,88	-0,18
**RBBP8**	12,48	12,66	-0,18
**KIF13B**	11,76	11,93	-0,18
**DYNC2I1**	10,94	11,11	-0,18
**HYAL2**	10,87	11,04	-0,18
**TRMT112**	12,90	13,07	-0,18
**RNF167**	11,81	11,99	-0,18
**VPS4A**	12,73	12,91	-0,18
**TEP1**	12,30	12,48	-0,18
**IFI35**	9,00	9,18	-0,18
**KLHL25**	10,46	10,63	-0,18
**CCDC116**	5,19	5,36	-0,18
**PARP8**	10,71	10,89	-0,18
**CENPT**	11,47	11,64	-0,18
**NBEAL2**	13,14	13,32	-0,18
**ING2**	9,76	9,94	-0,18
**CAPN5**	7,80	7,98	-0,18
**RNH1**	12,08	12,25	-0,18
**NRG4**	6,27	6,45	-0,18
**MED8**	10,99	11,17	-0,18
**CPNE7**	12,01	12,18	-0,18
**CRACR2A**	11,61	11,78	-0,18
**THAP3**	8,30	8,48	-0,18
**SWI5**	9,31	9,49	-0,18
**FAM78B**	9,70	9,87	-0,18
**IBA57**	10,64	10,81	-0,18
**NFKB2**	10,01	10,18	-0,18
**RELB**	8,28	8,46	-0,18
**IL18BP**	8,03	8,20	-0,18
**TMEM91**	8,14	8,31	-0,17
**MVB12B**	10,31	10,49	-0,17
**RPS23**	16,28	16,46	-0,17
**SH3GL1**	11,17	11,35	-0,17
**HLX**	10,72	10,90	-0,17
**TRIOBP**	11,31	11,48	-0,17
**C19orf12**	10,77	10,95	-0,17
**SPRYD3**	10,58	10,75	-0,17
**FKRP**	10,39	10,56	-0,17
**ZNF79**	9,39	9,57	-0,17
**ATG4D**	11,00	11,18	-0,17
**HTRA2**	11,02	11,19	-0,17
**ZNF707**	10,04	10,21	-0,17
**HIRIP3**	11,35	11,52	-0,17
**SNRPG**	13,11	13,29	-0,17
**TRMO**	9,68	9,85	-0,17
**THRAP3**	14,34	14,52	-0,17
**CYP26B1**	2,61	2,78	-0,17
**ZP3**	8,68	8,85	-0,17
**PKD2**	12,79	12,97	-0,17
**PHGDH**	14,70	14,87	-0,17
**AKNA**	12,65	12,82	-0,17
**RIC8A**	12,02	12,19	-0,17
**PTPN18**	12,17	12,35	-0,17

**GET4**	11,64	11,81	-0,17
**CORO1A**	13,97	14,15	-0,17
**ENC1**	11,44	11,61	-0,17
**ZNF34**	7,89	8,06	-0,17
**ANAPC7**	11,53	11,71	-0,17
**ATG2A**	10,94	11,11	-0,17
**ENSA**	13,69	13,86	-0,17
**P2RX5-TAX1BP3**	8,78	8,95	-0,17
**FGD2**	8,15	8,32	-0,17
**RIPK1**	11,17	11,35	-0,17
**CHTOP**	12,98	13,15	-0,17
**RPS16**	16,02	16,19	-0,17
**CELF6**	8,38	8,55	-0,17
**DOLK**	9,36	9,53	-0,17
**BLZF1**	10,13	10,30	-0,17
**FPGS**	13,30	13,48	-0,17
**ZNF773**	9,80	9,97	-0,17
**CDKN2C**	9,99	10,16	-0,17
**CYC1**	13,66	13,83	-0,17
**VEGFA**	11,02	11,19	-0,17
**WWC1**	9,71	9,88	-0,17
**RNF20**	12,47	12,65	-0,17
**NCK2**	11,63	11,80	-0,17
**PEX12**	8,57	8,74	-0,17
**GSTO2**	5,96	6,13	-0,17
**CCR7**	9,96	10,13	-0,17
**ZFHX2**	8,38	8,55	-0,17
**CALR**	16,15	16,32	-0,17
**MLLT1**	12,91	13,08	-0,17
**SEC61G**	11,80	11,97	-0,17
**UQCRH**	13,63	13,80	-0,17
**EIF5A**	15,50	15,67	-0,17
**RNF125**	11,25	11,42	-0,17
**ZNF175**	9,16	9,33	-0,17
**SNX8**	11,46	11,63	-0,17
**TUBA3D**	6,35	6,52	-0,17
**KCTD5**	11,81	11,98	-0,17
**FGFRL1**	11,75	11,92	-0,17
**RRP12**	12,48	12,65	-0,17
**GSK3B**	10,86	11,03	-0,17
**GRIK3**	5,85	6,02	-0,17
**FBXO10**	9,30	9,47	-0,17
**WDR45B**	11,86	12,03	-0,17
**ABCF2**	12,58	12,75	-0,17
**ARSD**	11,46	11,63	-0,17
**CHMP1A**	12,30	12,47	-0,17
**ZNF668**	10,21	10,38	-0,17
**CDKN2D**	10,19	10,36	-0,17
**UGDH**	12,10	12,27	-0,17
**KIAA0930**	12,99	13,16	-0,17
**TAPBP**	13,08	13,25	-0,17
**CCDC25**	12,30	12,46	-0,17
**DPP9**	12,41	12,58	-0,17
**ILRUN**	12,44	12,61	-0,17
**PLPP5**	11,20	11,37	-0,17
**LGALS3**	4,51	4,68	-0,17

SCN4A	9,38	9,55	-0,17
INAVA	6,94	7,11	-0,17
XRCC3	12,00	12,17	-0,17
RBM14	13,68	13,85	-0,17
KAT6B	12,05	12,21	-0,17
WNT9A	7,49	7,66	-0,17
CIZ1	13,12	13,29	-0,17
MAP4K1	11,55	11,72	-0,17
NDUFA4	12,82	12,99	-0,17
AMY2B	9,66	9,83	-0,17
SMAD4	11,82	11,99	-0,17
SCAND1	11,73	11,89	-0,17
ADGRB2	9,31	9,48	-0,17
TSC22D4	13,13	13,30	-0,17
MNT	11,27	11,44	-0,17
AP3D1	12,95	13,12	-0,17
RETREG2	11,93	12,10	-0,17
MAP2K1	11,34	11,51	-0,17
FUCA1	9,49	9,66	-0,17
SFSWAP	12,19	12,36	-0,17
PSMG3	10,37	10,54	-0,17
MRPL20	12,48	12,64	-0,17
HSF1	12,89	13,05	-0,17
NT5C3B	11,71	11,88	-0,17
TPTE	4,63	4,80	-0,17
ZC3H13	13,29	13,46	-0,17
MPZ	8,36	8,53	-0,17
TECR	12,51	12,68	-0,17
XXYLT1	10,94	11,11	-0,17
IQCK	8,93	9,10	-0,17
RIMS3	11,33	11,49	-0,17
EME1	10,28	10,45	-0,17
MYL6	14,50	14,66	-0,17
CCDC134	11,27	11,43	-0,17
AFG3L2	13,31	13,48	-0,17
SMG9	11,30	11,47	-0,17
ZNF284	8,58	8,75	-0,17
DHODH	10,98	11,15	-0,17
SLCO4A1	11,09	11,26	-0,17
ZNF878	8,33	8,49	-0,17
KCTD7	10,23	10,40	-0,17
FOSL2	11,89	12,06	-0,17
AARSD1	11,39	11,56	-0,17
C11orf96	8,96	9,12	-0,17
CSRNP2	11,34	11,50	-0,17
PRKCSH	13,14	13,31	-0,17
ZNF704	13,41	13,57	-0,17
SRRM5	7,18	7,34	-0,17
PRMT1	14,45	14,62	-0,17
PMF1	11,78	11,95	-0,17
PPIL2	11,86	12,03	-0,17
ATP7B	5,05	5,22	-0,17
USE1	9,55	9,72	-0,17
WT1	7,02	7,19	-0,17
DLGAP3	7,59	7,75	-0,17
MFSD12	10,47	10,63	-0,17

**ATP5MF**	12,71	12,88	-0,17
**HYOU1**	14,42	14,59	-0,17
**SNF8**	11,80	11,97	-0,17
**MANEAL**	11,82	11,98	-0,17
**TBC1D10B**	12,36	12,52	-0,17
**CFAP298**	12,01	12,17	-0,17
**TRAPPC12**	11,04	11,20	-0,17
**RFLNB**	8,93	9,09	-0,17
**ZFYVE21**	9,60	9,76	-0,17
**H1-2**	8,13	8,29	-0,17
**LFNG**	9,89	10,05	-0,17
**TMEM176B**	2,72	2,89	-0,17
**TMEM222**	10,99	11,15	-0,17
**IRX2**	13,47	13,64	-0,17
**AGRN**	12,32	12,48	-0,17
**LEAP2**	6,19	6,35	-0,17
**TFR2**	10,33	10,49	-0,17
**DACT1**	10,84	11,01	-0,17
**ADCK5**	9,87	10,03	-0,17
**EBF3**	7,24	7,41	-0,17
**ZC3H18**	12,85	13,01	-0,17
**MPPED2**	10,58	10,75	-0,17
**DCAF4**	10,70	10,86	-0,17
**FIBP**	12,03	12,19	-0,17
**ESS2**	10,67	10,84	-0,16
**RBM19**	12,09	12,26	-0,16
**SRSF4**	13,25	13,41	-0,16
**P3H1**	11,40	11,56	-0,16
**COX6A1**	13,40	13,57	-0,16
**UBL5**	12,71	12,88	-0,16
**TF**	7,99	8,16	-0,16
**LMO2**	11,06	11,23	-0,16
**STXBP4**	10,39	10,56	-0,16
**ZNF740**	12,00	12,16	-0,16
**EPN2**	5,74	5,90	-0,16
**CYP4F3**	5,74	5,90	-0,16
**DBP**	9,91	10,07	-0,16
**SLC5A6**	12,77	12,93	-0,16
**NKAIN4**	9,90	10,06	-0,16
**MPG**	11,03	11,19	-0,16
**BIN1**	12,78	12,95	-0,16
**SPHK1**	6,60	6,77	-0,16
**MRPS9**	12,14	12,30	-0,16
**MFAP1**	12,23	12,39	-0,16
**RNF44**	11,83	11,99	-0,16
**PINK1**	8,96	9,13	-0,16
**IFIT2**	7,37	7,54	-0,16
**PHETA1**	6,83	6,99	-0,16
**COA8**	10,16	10,32	-0,16
**STOML2**	13,31	13,47	-0,16
**FOXK1**	12,76	12,93	-0,16
**PKNOX1**	11,45	11,61	-0,16
**RHBDD3**	9,95	10,12	-0,16
**DNAJC11**	12,99	13,16	-0,16
**TIMM22**	11,01	11,17	-0,16
**ANP32A**	14,88	15,04	-0,16

**CYREN**	11,17	11,33	-0,16
**PITPNA**	12,89	13,06	-0,16
**CNNM4**	10,24	10,41	-0,16
**CHCHD3**	13,14	13,30	-0,16
**ZNF395**	13,32	13,48	-0,16
**ZEB1**	8,66	8,82	-0,16
**MAP3K3**	11,17	11,34	-0,16
**NPM3**	11,75	11,91	-0,16
**ZNF830**	10,75	10,91	-0,16
**CCN3**	6,90	7,07	-0,16
**CLSPN**	13,00	13,16	-0,16
**FKBP7**	8,95	9,12	-0,16
**PCDHGA11**	8,01	8,17	-0,16
**GATD3B**	11,47	11,64	-0,16
**ARPC5L**	12,23	12,39	-0,16
**NR3C2**	4,93	5,09	-0,16
**CACTIN**	11,25	11,41	-0,16
**SPRYD4**	10,55	10,71	-0,16
**PCED1A**	9,76	9,92	-0,16
**NUTM2G**	7,40	7,56	-0,16
**ENKUR**	2,24	2,40	-0,16
**FOXD4L5**	4,76	4,92	-0,16
**DAZAP1**	14,51	14,67	-0,16
**KLHL17**	9,40	9,57	-0,16
**PRKCZ**	12,22	12,38	-0,16
**HOOK2**	9,67	9,83	-0,16
**PHYKPL**	9,74	9,91	-0,16
**CD22**	13,70	13,86	-0,16
**GPATCH1**	10,62	10,78	-0,16
**NME1**	14,45	14,61	-0,16
**MALSU1**	10,83	10,99	-0,16
**TICAM2**	6,39	6,55	-0,16
**CRIP2**	8,10	8,26	-0,16
**NIBAN3**	11,80	11,96	-0,16
**NDUFA2**	11,77	11,93	-0,16
**MYRIP**	8,82	8,98	-0,16
**UST**	8,53	8,69	-0,16
**CLCC1**	10,37	10,54	-0,16
**OST4**	11,95	12,11	-0,16
**NAIF1**	9,64	9,80	-0,16
**TRIM7**	7,14	7,30	-0,16
**ZNF777**	10,95	11,11	-0,16
**TRAF3**	11,97	12,13	-0,16
**SUOX**	7,95	8,11	-0,16
**UGT8**	11,02	11,18	-0,16
**NAA10**	11,96	12,12	-0,16
**RRNAD1**	9,14	9,30	-0,16
**MTA2**	14,11	14,27	-0,16
**SRRT**	14,05	14,21	-0,16
**UTP23**	12,45	12,61	-0,16
**ALKBH7**	10,17	10,33	-0,16
**ERH**	14,05	14,21	-0,16
**FKBP11**	11,14	11,30	-0,16
**EBP**	11,81	11,97	-0,16
**GRK4**	8,79	8,95	-0,16
**PIMREG**	11,42	11,58	-0,16

**EFNB2**	13,41	13,57	-0,16
**DOT1L**	13,50	13,66	-0,16
**ZNF346**	11,27	11,43	-0,16
**NEIL1**	8,50	8,66	-0,16
**TNFRSF8**	10,84	11,00	-0,16
**CELF1**	12,61	12,77	-0,16
**RPS18**	16,48	16,64	-0,16
**NSRP1**	12,00	12,16	-0,16
**PYCR2**	13,13	13,29	-0,16
**CD99**	13,01	13,17	-0,16
**NME2**	14,83	14,99	-0,16
**ARMC12**	5,24	5,40	-0,16
**SLTM**	14,11	14,26	-0,16
**AAAS**	12,27	12,43	-0,16
**MRPL54**	10,26	10,42	-0,16
**MKNK1**	10,58	10,73	-0,16
**RBM3**	15,33	15,49	-0,16
**KRTCAP2**	11,23	11,39	-0,16
**STX4**	10,24	10,40	-0,16
**CASC3**	13,52	13,68	-0,16
**NMRK1**	8,66	8,81	-0,16
**ARNT2**	11,00	11,16	-0,16
**IMP4**	12,83	12,99	-0,16
**SUPT7L**	11,70	11,86	-0,16
**EHMT1**	13,01	13,17	-0,16
**NCBP2AS2**	10,66	10,82	-0,16
**SUSD1**	10,04	10,19	-0,16
**PTGER2**	11,31	11,47	-0,16
**SNRPD2**	13,88	14,03	-0,16
**PPP4C**	12,65	12,81	-0,16
**CREB3L1**	6,41	6,56	-0,16
**GPR150**	6,41	6,56	-0,16
**TDRKH**	9,34	9,50	-0,16
**PEX6**	11,26	11,42	-0,16
**PMEL**	5,75	5,91	-0,16
**DHRS2**	3,82	3,97	-0,16
**GNG3**	3,82	3,97	-0,16
**DDX51**	12,12	12,27	-0,16
**GLIPR2**	10,59	10,75	-0,16
**RAD23A**	12,65	12,81	-0,16
**KLHDC3**	13,04	13,20	-0,16
**TELO2**	12,51	12,67	-0,16
**NDUFAF2**	11,22	11,37	-0,16
**PAQR4**	11,87	12,03	-0,16
**RBCK1**	11,38	11,54	-0,16
**SLC2A11**	7,87	8,02	-0,16
**DUSP2**	10,37	10,53	-0,16
**PNMA1**	9,85	10,00	-0,16
**CEP164**	10,63	10,79	-0,16
**LRCH1**	11,76	11,91	-0,16
**SVBP**	9,61	9,76	-0,16
**MUS81**	11,29	11,45	-0,16
**GPR132**	10,02	10,18	-0,15
**SUGP2**	13,99	14,15	-0,15
**MRFAP1**	13,85	14,01	-0,15
**HIF1AN**	12,36	12,51	-0,15

**ZC4H2**	11,02	11,18	-0,15
**MAP6D1**	8,11	8,27	-0,15
**TCF19**	12,46	12,61	-0,15
**FCHSD2**	11,76	11,92	-0,15
**STRN4**	12,50	12,66	-0,15
**ZNF540**	6,99	7,15	-0,15
**ZNF780A**	10,19	10,34	-0,15
**ZNF710**	11,05	11,21	-0,15
**TSPAN17**	11,97	12,12	-0,15
**DSE**	9,80	9,95	-0,15
**RBM28**	13,04	13,19	-0,15
**MFSD6**	10,49	10,64	-0,15
**TXNRD2**	10,34	10,49	-0,15
**CMTR1**	12,20	12,36	-0,15
**PXMP2**	11,01	11,17	-0,15
**CMTM2**	7,58	7,74	-0,15
**ABCF1**	13,64	13,79	-0,15
**RETREG3**	11,14	11,29	-0,15
**TESC**	7,14	7,30	-0,15
**STOM**	10,57	10,72	-0,15
**UBIAD1**	11,49	11,65	-0,15
**ZNF567**	9,82	9,97	-0,15
**RGS5**	6,94	7,10	-0,15
**XAB2**	12,07	12,23	-0,15
**TTC24**	11,18	11,33	-0,15
**FEM1A**	12,50	12,65	-0,15
**SLC7A6**	12,99	13,14	-0,15
**EBPL**	11,71	11,87	-0,15
**SELENOW**	9,42	9,57	-0,15
**UBE3A**	13,76	13,91	-0,15
**SLC16A8**	3,43	3,59	-0,15
**LGALS3BP**	12,28	12,43	-0,15
**LRP3**	11,99	12,14	-0,15
**FABP5**	15,04	15,20	-0,15
**EIF4EBP3**	8,46	8,61	-0,15
**RAVER1**	13,06	13,21	-0,15
**GLRX5**	13,03	13,18	-0,15
**DLL3**	8,96	9,11	-0,15
**STARD5**	9,04	9,19	-0,15
**TUFM**	14,36	14,51	-0,15
**ZNF467**	9,07	9,22	-0,15
**S1PR4**	11,82	11,97	-0,15
**HLA-DQA2**	9,26	9,41	-0,15
**TMEM38B**	9,83	9,99	-0,15
**DNAJC4**	8,89	9,04	-0,15
**ATAD1**	11,93	12,08	-0,15
**MOAP1**	10,90	11,05	-0,15
**ZNF672**	12,15	12,30	-0,15
**DUSP7**	11,48	11,63	-0,15
**SRPK3**	7,02	7,17	-0,15
**CYBC1**	12,62	12,77	-0,15
**TAX1BP1**	12,01	12,16	-0,15
**PPARD**	10,43	10,58	-0,15
**FBXW9**	9,39	9,54	-0,15
**MAN2A2**	12,49	12,64	-0,15
**ERVK3-1**	11,66	11,81	-0,15

**ACAP1**	9,74	9,89	-0,15
**MFSD5**	10,33	10,48	-0,15
**PYGB**	12,86	13,01	-0,15
**YIPF3**	11,10	11,25	-0,15
**CARMIL3**	7,24	7,39	-0,15
**KLC1**	12,74	12,89	-0,15
**ENKD1**	9,68	9,83	-0,15
**ACSS1**	11,94	12,10	-0,15
**TIAF1**	10,92	11,07	-0,15
**MID1IP1**	12,35	12,50	-0,15
**ATG10**	10,17	10,32	-0,15
**ZNF497**	9,20	9,35	-0,15
**LRFN2**	9,40	9,55	-0,15
**SLC2A6**	9,52	9,67	-0,15
**DUS4L-BCAP29**	9,31	9,46	-0,15
**NFU1**	10,41	10,56	-0,15
**DCXR**	11,20	11,35	-0,15
**CBLB**	10,65	10,80	-0,15
**PRR3**	11,18	11,33	-0,15
**ILKAP**	11,64	11,79	-0,15
**DHX34**	11,23	11,38	-0,15
**BIN3**	11,40	11,55	-0,15
**AL132780.3**	8,80	8,95	-0,15
**SPCS2**	13,46	13,61	-0,15
**HNRNPUL2-BSCL2**	12,42	12,57	-0,15
**COQ3**	9,39	9,54	-0,15
**CHERP**	13,31	13,46	-0,15
**BTD**	9,08	9,22	-0,15
**ST6GALNAC4**	11,06	11,21	-0,15
**CCDC96**	6,19	6,34	-0,15
**R3HDM4**	11,28	11,43	-0,15
**PDHA1**	13,27	13,42	-0,15
**TSPYL4**	12,32	12,47	-0,15
**MAPK11**	9,70	9,85	-0,15
**XYLT2**	11,00	11,15	-0,15
**HIGD2A**	11,62	11,77	-0,15
**SMG6**	11,90	12,05	-0,15
**CD160**	6,06	6,21	-0,15
**DCTN1**	13,41	13,55	-0,15
**TMEM128**	8,40	8,54	-0,15
**STK36**	9,47	9,61	-0,15
**SENP3**	12,22	12,37	-0,15
**ING5**	12,46	12,61	-0,15
**CD180**	5,61	5,76	-0,15
**KLHL24**	11,09	11,24	-0,15
**TNFRSF1A**	11,05	11,19	-0,15
**RANBP3**	12,62	12,76	-0,15
**FZD1**	10,26	10,40	-0,15
**TCERG1L**	11,39	11,53	-0,15
**RRP1**	11,92	12,07	-0,15
**RNASET2**	11,02	11,16	-0,15
**COL11A2**	6,63	6,78	-0,15
**AUP1**	12,36	12,51	-0,15
**CSTF2T**	12,54	12,69	-0,15
**RUBCN**	11,40	11,54	-0,15
**ALS2CL**	8,08	8,23	-0,15

**NUDT18**	9,08	9,23	-0,15
**CRTC2**	11,20	11,35	-0,15
**WASH6P**	11,77	11,92	-0,15
**ZCCHC4**	10,31	10,46	-0,15
**STK16**	8,98	9,13	-0,15
**TCF25**	12,63	12,78	-0,15
**DPF2**	12,76	12,90	-0,15
**RAB5C**	12,66	12,81	-0,15
**NFKBIL1**	8,65	8,80	-0,15
**ANO9**	7,88	8,03	-0,15
**ZNF148**	12,38	12,52	-0,15
**ITGAE**	9,75	9,90	-0,15
**CCDC59**	11,71	11,86	-0,15
**SCAP**	12,44	12,58	-0,15
**SERF2**	14,40	14,55	-0,15
**ADCY1**	10,01	10,16	-0,15
**ANKAR**	6,63	6,78	-0,15
**PDXP**	11,87	12,02	-0,15
**PAQR5**	2,91	3,06	-0,15
**TNNT2**	2,91	3,06	-0,15
**OMD**	2,91	3,06	-0,15
**STEAP4**	2,91	3,06	-0,15
**MYBPC3**	2,91	3,06	-0,15
**PNLDC1**	2,91	3,06	-0,15
**OR56B1**	2,91	3,06	-0,15
**ERVFRD-1**	2,91	3,06	-0,15
**SLC22A15**	2,91	3,06	-0,15
**CAMK2N2**	2,91	3,06	-0,15
**WDR72**	2,91	3,06	-0,15
**GBP4**	9,96	10,11	-0,15
**ZNF696**	10,73	10,88	-0,15
**CHD9**	12,97	13,12	-0,15
**C12orf75**	10,24	10,39	-0,15
**MAF1**	12,66	12,80	-0,15
**HYKK**	5,59	5,74	-0,15
**WDR5**	13,16	13,30	-0,15
**STRIP2**	8,27	8,41	-0,15
**UBE2O**	12,55	12,70	-0,15
**RNF31**	11,13	11,28	-0,15
**FRRS1**	6,28	6,43	-0,15
**SIPA1L2**	7,21	7,35	-0,15
**INO80E**	12,17	12,32	-0,15
**COMMD3-BMI1**	9,51	9,65	-0,15
**MMP21**	6,27	6,41	-0,15
**CHCHD1**	11,17	11,31	-0,15
**PHTF1**	11,35	11,49	-0,15
**SPINT2**	11,71	11,85	-0,15
**CNOT7**	14,14	14,29	-0,15
**FOXD4**	4,64	4,79	-0,15
**BAMBI**	11,95	12,10	-0,15
**PRKAR1A**	13,33	13,48	-0,15
**WIZ**	12,70	12,85	-0,15
**CTC1**	11,10	11,25	-0,15
**ZEB2**	14,24	14,38	-0,15
**UBE2D4**	10,57	10,72	-0,15
**POLR3D**	12,65	12,80	-0,15

**NR1H2**	11,12	11,27	-0,15
**DMWD**	9,04	9,19	-0,15
**CATSPER3**	5,90	6,05	-0,15
**VAMP2**	11,45	11,60	-0,14
**RBM38**	12,59	12,74	-0,14
**SHARPIN**	11,21	11,36	-0,14
**WWP2**	12,03	12,17	-0,14
**ZNF511**	11,09	11,23	-0,14
**MARK4**	11,39	11,53	-0,14
**FADD**	10,96	11,10	-0,14
**CEP170**	13,38	13,53	-0,14
**CCDC115**	10,74	10,89	-0,14
**GGA1**	11,53	11,67	-0,14
**TSC2**	12,31	12,45	-0,14
**GRAP**	10,55	10,69	-0,14
**DGKA**	10,46	10,60	-0,14
**TEF**	9,82	9,97	-0,14
**PIAS3**	10,82	10,97	-0,14
**LPCAT3**	11,30	11,44	-0,14
**MRPS11**	11,64	11,78	-0,14
**AC013477.1**	7,28	7,42	-0,14
**CDT1**	12,82	12,96	-0,14
**KIF18B**	12,44	12,58	-0,14
**ZFP62**	11,48	11,62	-0,14
**NPIPB9**	8,70	8,85	-0,14
**KIF21A**	10,75	10,89	-0,14
**SLC22A5**	8,93	9,07	-0,14
**PTPN6**	13,29	13,43	-0,14
**DHX16**	11,98	12,13	-0,14
**NME9**	5,44	5,58	-0,14
**SELENOK**	11,41	11,56	-0,14
**HLA-E**	13,40	13,55	-0,14
**COX7C**	13,54	13,69	-0,14
**TRIM21**	10,90	11,04	-0,14
**PCDHB15**	5,29	5,43	-0,14
**TFIP11**	12,99	13,14	-0,14
**HCFC1**	14,53	14,67	-0,14
**MAP1A**	13,84	13,99	-0,14
**SOX4**	14,96	15,11	-0,14
**GPR89B**	10,30	10,44	-0,14
**PKDCC**	12,52	12,66	-0,14
**FUBP3**	12,63	12,77	-0,14
**EPHB6**	8,88	9,02	-0,14
**LZTR1**	11,76	11,90	-0,14
**COLGALT1**	13,38	13,52	-0,14
**SLC41A3**	10,48	10,62	-0,14
**SUN1**	12,67	12,81	-0,14
**OSER1**	11,06	11,20	-0,14
**ADCY3**	11,68	11,82	-0,14
**RTL6**	12,42	12,56	-0,14
**BCAP31**	13,28	13,42	-0,14
**RPL32**	15,47	15,61	-0,14
**RPP25L**	10,34	10,48	-0,14
**NDUFB8**	12,64	12,78	-0,14
**GNAI2**	13,73	13,87	-0,14
**CFL1**	15,66	15,80	-0,14

**FBL**	14,13	14,27	-0,14
**PLK3**	10,22	10,37	-0,14
**PFKM**	13,50	13,64	-0,14
**C18orf21**	10,12	10,26	-0,14
**TKFC**	9,96	10,10	-0,14
**ZNF347**	11,31	11,45	-0,14
**TIMELESS**	13,59	13,73	-0,14
**ARVCF**	10,03	10,17	-0,14
**BRD1**	12,05	12,19	-0,14
**SEPTIN5**	9,90	10,04	-0,14
**ABCC9**	6,53	6,67	-0,14
**COPE**	12,87	13,01	-0,14
**TUBGCP6**	11,70	11,84	-0,14
**TANC2**	10,23	10,37	-0,14
**CAPN1**	13,20	13,34	-0,14
**PTDSS2**	10,76	10,90	-0,14
**CDK7**	10,42	10,56	-0,14
**BST2**	11,28	11,42	-0,14
**PITPNM1**	10,87	11,01	-0,14
**XKR4**	11,02	11,16	-0,14
**KIF3C**	10,59	10,73	-0,14
**REEP2**	6,72	6,86	-0,14
**ALKBH1**	10,42	10,56	-0,14
**E2F4**	13,20	13,34	-0,14
**SRSF5**	14,80	14,94	-0,14
**SNRPA**	13,26	13,40	-0,14
**IL17RE**	7,12	7,26	-0,14
**TERT**	13,46	13,60	-0,14
**CENATAC**	9,28	9,42	-0,14
**SLC41A1**	12,47	12,60	-0,14
**KCNA3**	9,83	9,97	-0,14
**MCM7**	15,56	15,70	-0,14
**GZMA**	7,83	7,97	-0,14
**ARRB2**	12,28	12,42	-0,14
**UBXN8**	10,93	11,06	-0,14
**RNF126**	11,22	11,36	-0,14
**SARS1**	13,02	13,16	-0,14
**OAF**	10,83	10,97	-0,14
**POP1**	12,01	12,15	-0,14
**HMGB2**	15,83	15,97	-0,14
**SLC8B1**	8,30	8,44	-0,14
**LRCH4**	11,36	11,49	-0,14
**ZNF219**	10,51	10,65	-0,14
**BICD2**	11,84	11,97	-0,14
**LHFPL6**	9,64	9,77	-0,14
**ZNF345**	9,29	9,43	-0,14
**ING4**	10,21	10,34	-0,14
**CBLL1**	12,37	12,51	-0,14
**SETDB1**	10,85	10,99	-0,14
**BSN**	7,69	7,83	-0,14
**CHMP2A**	11,09	11,23	-0,14
**EPC2**	11,64	11,78	-0,14
**SLC29A2**	11,25	11,39	-0,14
**STAMBPL1**	8,99	9,13	-0,14
**KMT5C**	10,07	10,21	-0,14
**TMEM115**	10,47	10,61	-0,14

**CPEB2**	7,30	7,44	-0,14
**NDUFA9**	11,61	11,75	-0,14
**THAP11**	12,05	12,19	-0,14
**ZC3HAV1L**	9,86	10,00	-0,14
**NKX3-1**	9,32	9,46	-0,14
**MDC1**	13,43	13,57	-0,14
**UGT3A2**	12,37	12,51	-0,14
**CCNI2**	4,20	4,34	-0,14
**PEX26**	12,38	12,51	-0,14
**SEMA6C**	12,74	12,88	-0,14
**RNF157**	4,69	4,83	-0,14
**SHQ1**	11,28	11,42	-0,14
**SNCB**	7,52	7,66	-0,14
**PHF23**	11,46	11,59	-0,14
**SQSTM1**	12,96	13,10	-0,14
**NDUFS5**	13,13	13,26	-0,14
**TIMM29**	10,07	10,21	-0,14
**LRRK1**	10,39	10,52	-0,14
**ICAM1**	6,75	6,89	-0,14
**ZCCHC24**	9,62	9,76	-0,14
**CIC**	12,29	12,42	-0,14
**MAGED2**	11,23	11,36	-0,14
**PLEKHO2**	10,26	10,40	-0,14
**CLBA1**	9,07	9,21	-0,14
**B4GALT2**	12,30	12,43	-0,14
**CASK**	10,34	10,48	-0,14
**MIS18A**	12,10	12,24	-0,14
**PGGT1B**	11,29	11,43	-0,14
**GPBAR1**	3,16	3,30	-0,14
**CTXN1**	9,94	10,08	-0,14
**ARHGAP27**	10,40	10,53	-0,14
**PSMB5**	12,60	12,73	-0,14
**PPP5C**	13,03	13,17	-0,14
**VASP**	10,73	10,87	-0,14
**HPS1**	11,75	11,89	-0,14
**UBAP2**	12,26	12,39	-0,14
**JPT1**	13,45	13,59	-0,14
**PPP1R10**	12,33	12,47	-0,14
**CDCA5**	13,45	13,58	-0,14
**ADAM19**	9,03	9,17	-0,14
**ANKZF1**	10,53	10,67	-0,14
**TRIL**	8,40	8,53	-0,14
**HNRNPD**	15,60	15,74	-0,14
**GBA2**	10,47	10,61	-0,14
**ACD**	11,13	11,27	-0,14
**HAUS8**	10,81	10,95	-0,14
**ZNF223**	6,67	6,81	-0,14
**MAD2L2**	13,09	13,22	-0,13
**PCDH12**	7,93	8,06	-0,13
**MDP1**	9,08	9,21	-0,13
**SH3RF3**	10,14	10,27	-0,13
**BCR**	13,79	13,93	-0,13
**CCDC184**	7,10	7,23	-0,13
**KLHL36**	11,70	11,83	-0,13
**PSMD2**	14,56	14,70	-0,13
**C11orf1**	8,78	8,92	-0,13

TBK1	11,39	11,53	-0,13
POLE	14,16	14,30	-0,13
NDUFAF3	11,62	11,75	-0,13
AP000781.2	5,37	5,51	-0,13
GUCA1B	7,21	7,34	-0,13
OSM	5,15	5,28	-0,13
ZMAT3	11,84	11,97	-0,13
FAM120AOS	11,68	11,81	-0,13
TSACC	5,54	5,67	-0,13
TMEM109	12,98	13,11	-0,13
SLC25A39	13,16	13,29	-0,13
PRXL2B	9,60	9,74	-0,13
AIFM2	9,74	9,87	-0,13
NCOR1	12,11	12,24	-0,13
TVP23A	7,27	7,41	-0,13
SRM	14,02	14,16	-0,13
GAL3ST4	8,90	9,03	-0,13
PRCC	12,81	12,94	-0,13
MORN3	6,44	6,58	-0,13
ISOC2	11,55	11,69	-0,13
GPR137	9,70	9,83	-0,13
IGLL5	10,52	10,65	-0,13
KLF13	12,98	13,11	-0,13
PLCB2	10,54	10,67	-0,13
YBX1	17,01	17,14	-0,13
TK2	8,78	8,91	-0,13
TMEM214	11,70	11,83	-0,13
ZNF575	5,46	5,59	-0,13
ALG3	11,74	11,87	-0,13
C9orf43	6,00	6,13	-0,13
COX8A	12,46	12,59	-0,13
DUSP28	9,60	9,74	-0,13
KPNA1	13,39	13,52	-0,13
RCC1L	11,57	11,70	-0,13
NELFB	12,54	12,68	-0,13
RBM25	14,40	14,53	-0,13
COQ4	10,90	11,04	-0,13
TMA16	12,38	12,51	-0,13
JMJD6	11,08	11,21	-0,13
FAM53C	12,39	12,52	-0,13
NATD1	8,70	8,84	-0,13
MYBL2	14,71	14,84	-0,13
CHMP7	12,25	12,39	-0,13
SLC44A3	9,74	9,87	-0,13
ANKRD52	12,96	13,09	-0,13
PUS10	9,86	9,99	-0,13
PINX1	12,38	12,51	-0,13
OGDH	12,94	13,07	-0,13
REPS2	4,76	4,89	-0,13
NHP2	13,83	13,96	-0,13
KCNB1	2,26	2,39	-0,13
SIAH1	11,76	11,89	-0,13
PFN1	16,03	16,16	-0,13
EIF3C	15,90	16,03	-0,13
BCL2L1	11,88	12,01	-0,13
ADCK2	11,06	11,19	-0,13

**GTPBP1**	12,25	12,39	-0,13
**ZNF865**	10,69	10,82	-0,13
**CXorf65**	3,92	4,05	-0,13
**WDR4**	11,45	11,58	-0,13
**VAT1**	11,10	11,23	-0,13
**AC007040.2**	7,63	7,76	-0,13
**CC2D1B**	11,04	11,17	-0,13
**BCOR**	12,59	12,73	-0,13
**AIF1**	6,08	6,21	-0,13
**ZNF143**	11,25	11,38	-0,13
**RASEF**	10,57	10,70	-0,13
**C12orf73**	9,50	9,63	-0,13
**PHPT1**	11,02	11,15	-0,13
**GMFG**	13,59	13,72	-0,13
**LIF**	7,04	7,17	-0,13
**GORASP2**	12,84	12,97	-0,13
**CERS2**	13,69	13,82	-0,13
**PYGO2**	12,57	12,71	-0,13
**HMGB3**	13,56	13,69	-0,13
**POLDIP3**	13,25	13,38	-0,13
**OPN1SW**	2,09	2,22	-0,13
**S100P**	2,09	2,22	-0,13
**ATP4B**	2,09	2,22	-0,13
**IFITM10**	2,09	2,22	-0,13
**OR10G4**	2,09	2,22	-0,13
**PRR33**	2,09	2,22	-0,13
**PTPN9**	11,85	11,98	-0,13
**TBL3**	12,53	12,66	-0,13
**UBE2R2**	12,98	13,11	-0,13
**CHCHD4**	10,91	11,04	-0,13
**ZNF135**	10,39	10,52	-0,13
**KIN**	10,68	10,81	-0,13
**CERCAM**	10,34	10,47	-0,13
**KATNB1**	11,93	12,06	-0,13
**C1orf74**	9,89	10,02	-0,13
**RPL35A**	15,01	15,14	-0,13
**ZNF671**	10,14	10,27	-0,13
**DAXX**	12,60	12,73	-0,13
**NEK9**	13,23	13,36	-0,13
**ITPRIP**	11,17	11,30	-0,13
**S1PR5**	7,73	7,86	-0,13
**GP6**	3,68	3,81	-0,13
**STAG3**	9,87	10,00	-0,13
**TUBD1**	10,06	10,19	-0,13
**PLXNB2**	13,87	14,00	-0,13
**NECTIN1**	11,72	11,85	-0,13
**MLXIP**	13,41	13,54	-0,13
**ANKRD12**	12,11	12,24	-0,13
**AL031777.2**	5,86	5,99	-0,13
**PSKH1**	11,06	11,19	-0,13
**MAPKAPK5**	13,12	13,24	-0,13
**AC098484.3**	7,48	7,61	-0,13
**TBC1D7**	10,87	11,00	-0,13
**POLR2E**	13,57	13,70	-0,13
**HAUS2**	12,14	12,26	-0,13
**EPS15L1**	11,80	11,93	-0,13

**CCNF**	12,74	12,87	-0,13
**VARS1**	14,05	14,18	-0,13
**USP15**	12,14	12,27	-0,13
**MECR**	10,79	10,92	-0,13
**WBP1**	10,26	10,39	-0,13
**PSRC1**	10,59	10,71	-0,13
**RPS26**	14,57	14,70	-0,13
**TMEM54**	9,49	9,62	-0,13
**XKR8**	9,38	9,51	-0,13
**LSM6**	12,07	12,20	-0,13
**ARL16**	11,24	11,37	-0,13
**SCNM1**	11,48	11,61	-0,13
**CBWD6**	9,35	9,48	-0,13
**TMEM179B**	9,70	9,83	-0,13
**RTN4RL1**	9,58	9,70	-0,13
**MLST8**	11,67	11,79	-0,13
**MYO5C**	11,85	11,98	-0,13
**ZNF619**	9,58	9,71	-0,13
**PRR14**	9,99	10,12	-0,13
**SCO2**	9,42	9,55	-0,13
**TMSB15A**	12,31	12,44	-0,13
**SLC43A3**	13,85	13,98	-0,13
**RHOV**	7,72	7,84	-0,13
**JADE2**	9,29	9,42	-0,13
**HERPUD2**	10,50	10,63	-0,13
**KMT5A**	12,68	12,81	-0,13
**MAPKBP1**	11,03	11,16	-0,13
**PRAG1**	11,99	12,12	-0,13
**NDUFB11**	11,70	11,83	-0,13
**CD247**	9,31	9,44	-0,13
**COL18A1**	8,35	8,48	-0,13
**TMCO6**	9,62	9,74	-0,13
**PCYT1B**	9,05	9,17	-0,13
**ZFP2**	7,27	7,39	-0,13
**CLDN4**	3,02	3,15	-0,13
**TRAV27**	9,86	9,99	-0,13
**CDC20B**	1,21	1,34	-0,13
**RAB33B**	9,65	9,78	-0,13
**ZBED1**	12,80	12,93	-0,13
**HS2ST1**	12,12	12,24	-0,13
**VKORC1**	11,12	11,24	-0,13
**ATP6V1F**	11,89	12,02	-0,13
**DYNC1LI2**	12,30	12,42	-0,13
**PHC2**	13,27	13,40	-0,13
**INTS11**	12,26	12,39	-0,13
**NONO**	15,92	16,04	-0,13
**C11orf58**	14,27	14,40	-0,13
**GLDC**	11,60	11,73	-0,13
**EIF6**	12,71	12,83	-0,13
**DHX30**	13,59	13,71	-0,13
**PHACTR2**	10,37	10,49	-0,13
**LRPPRC**	11,54	11,66	-0,13
**XKR6**	4,51	4,64	-0,13
**TMEM272**	4,51	4,64	-0,13
**HUS1**	10,55	10,68	-0,13
**ZDHHC12**	9,65	9,78	-0,13

**NDUFC1**	11,17	11,30	-0,13
**FBXL2**	6,25	6,38	-0,13
**KCNQ4**	9,75	9,88	-0,13
**CDK5RAP3**	12,64	12,77	-0,13
**CCM2**	10,89	11,02	-0,13
**PTK2B**	14,56	14,68	-0,13
**KIRREL1**	11,68	11,80	-0,12
**SLC27A5**	8,83	8,95	-0,12
**CDK4**	14,15	14,28	-0,12
**SEMA4C**	11,18	11,31	-0,12
**NUCB1**	11,83	11,96	-0,12
**HINFP**	10,12	10,24	-0,12
**DNAJA3**	12,94	13,06	-0,12
**SNRPB**	14,70	14,83	-0,12
**ARNTL**	10,42	10,55	-0,12
**RASA4B**	10,12	10,24	-0,12
**SNIP1**	11,17	11,30	-0,12
**AIFM1**	12,62	12,75	-0,12
**SMYD4**	11,00	11,12	-0,12
**TFB1M**	9,63	9,76	-0,12
**NAT9**	10,94	11,06	-0,12
**DECR1**	12,78	12,90	-0,12
**SLC25A1**	12,25	12,38	-0,12
**RHOG**	10,82	10,95	-0,12
**GEMIN8**	9,53	9,65	-0,12
**NR2F6**	9,85	9,98	-0,12
**SF3B2**	14,73	14,85	-0,12
**GCSH**	12,13	12,25	-0,12
**HFE**	7,85	7,97	-0,12
**SMG8**	11,31	11,44	-0,12
**ZNF574**	11,56	11,68	-0,12
**DNASE2**	9,45	9,57	-0,12
**SIL1**	9,63	9,76	-0,12
**SPN**	14,55	14,67	-0,12
**CD320**	12,22	12,34	-0,12
**ALDH1A1**	6,81	6,93	-0,12
**SEC62**	13,22	13,35	-0,12
**G6PD**	11,83	11,96	-0,12
**ZBTB47**	10,74	10,86	-0,12
**C2CD2**	9,83	9,95	-0,12
**DGLUCY**	11,92	12,05	-0,12
**CRY2**	10,56	10,69	-0,12
**NEK4**	12,04	12,16	-0,12
**NANS**	11,77	11,89	-0,12
**TXLNA**	13,24	13,36	-0,12
**SPSB2**	8,19	8,31	-0,12
**KAT8**	11,64	11,77	-0,12
**HERC3**	10,40	10,52	-0,12
**CGREF1**	8,40	8,52	-0,12
**KLC2**	10,72	10,85	-0,12
**BRAT1**	11,90	12,02	-0,12
**ZNF688**	8,94	9,07	-0,12
**MMAB**	11,35	11,48	-0,12
**NCSTN**	12,66	12,79	-0,12
**SDF4**	12,37	12,49	-0,12
**ARGLU1**	13,65	13,78	-0,12

**NCLN**	13,21	13,33	-0,12
**TBC1D9B**	13,27	13,39	-0,12
**ELP6**	11,60	11,72	-0,12
**CDC45**	12,41	12,53	-0,12
**MRPS21**	12,48	12,60	-0,12
**HOXA5**	8,21	8,33	-0,12
**CALU**	13,31	13,43	-0,12
**CLCN2**	9,99	10,11	-0,12
**PMPCA**	12,80	12,93	-0,12
**SSH3**	9,19	9,31	-0,12
**ARL2**	10,73	10,85	-0,12
**TRIM47**	8,84	8,96	-0,12
**TKT**	14,92	15,04	-0,12
**RPA1**	14,17	14,29	-0,12
**THY1**	8,73	8,86	-0,12
**LAT2**	14,04	14,16	-0,12
**SNAPC5**	10,34	10,46	-0,12
**DGAT2**	9,05	9,17	-0,12
**SEMA6B**	6,08	6,20	-0,12
**MRPL33**	9,92	10,05	-0,12
**CCDC142**	9,99	10,11	-0,12
**VAMP5**	11,06	11,18	-0,12
**SBK1**	11,94	12,06	-0,12
**POLR3GL**	10,15	10,27	-0,12
**SPSB1**	9,25	9,37	-0,12
**TMEM17**	6,96	7,08	-0,12
**IER3IP1**	12,03	12,15	-0,12
**RTP4**	8,48	8,60	-0,12
**NTSR1**	8,70	8,82	-0,12
**FIP1L1**	12,61	12,73	-0,12
**CDK5RAP1**	11,45	11,57	-0,12
**TMEM11**	10,74	10,86	-0,12
**PTRHD1**	9,82	9,94	-0,12
**DACT3**	5,78	5,90	-0,12
**AQP5**	10,12	10,24	-0,12
**SNN**	12,36	12,48	-0,12
**ABHD6**	9,07	9,19	-0,12
**SLC12A3**	4,06	4,18	-0,12
**PGLS**	11,39	11,51	-0,12
**NFE2L3**	10,47	10,59	-0,12
**CKLF**	10,98	11,11	-0,12
**ANKRD13D**	11,12	11,24	-0,12
**SREBF2**	13,98	14,10	-0,12
**TOR2A**	10,16	10,28	-0,12
**RCC2**	15,65	15,77	-0,12
**ECE1**	8,98	9,10	-0,12
**DNAJC25-GNG10**	8,69	8,81	-0,12
**EIF3J**	13,52	13,64	-0,12
**C2orf76**	8,77	8,89	-0,12
**KLLN**	7,67	7,79	-0,12
**SIPA1L1**	9,79	9,91	-0,12
**LEMD2**	11,17	11,29	-0,12
**DENND2B**	5,80	5,92	-0,12
**GYS1**	11,85	11,97	-0,12
**AC244197.3**	9,48	9,60	-0,12
**PLEKHO1**	13,06	13,17	-0,12

**ZNF384**	11,81	11,93	-0,12
**MRPS5**	12,13	12,25	-0,12
**DDX23**	13,47	13,59	-0,12
**BORCS7**	10,91	11,03	-0,12
**NDUFA8**	11,54	11,66	-0,12
**SIVA1**	12,43	12,55	-0,12
**DIPK1B**	11,58	11,70	-0,12
**TPPP**	6,97	7,09	-0,12
**SOCS1**	4,29	4,41	-0,12
**RETREG1**	11,07	11,19	-0,12
**ATN1**	12,39	12,51	-0,12
**IMPDH1**	12,68	12,80	-0,12
**RAB37**	5,76	5,88	-0,12
**ZER1**	10,70	10,82	-0,12
**PSMC5**	13,26	13,38	-0,12
**UBE2I**	14,47	14,59	-0,12
**KIFC3**	10,16	10,27	-0,12
**CHAF1B**	12,15	12,27	-0,12
**ABT1**	11,83	11,95	-0,12
**FNTB**	10,35	10,46	-0,12
**DPH7**	10,80	10,92	-0,12
**ZNF576**	9,95	10,06	-0,12
**SEPTIN9**	15,10	15,22	-0,12
**C1orf159**	10,69	10,81	-0,12
**MRPS24**	11,73	11,85	-0,12
**PLCD1**	7,29	7,41	-0,12
**ZMYM4**	13,31	13,43	-0,12
**NPLOC4**	13,23	13,35	-0,12
**SLC39A4**	9,97	10,09	-0,12
**TOR1AIP2**	12,47	12,58	-0,12
**GPR75**	8,28	8,40	-0,12
**SMARCA2**	13,56	13,68	-0,12
**GUK1**	12,70	12,81	-0,12
**SLC29A1**	13,07	13,19	-0,12
**ATP1A3**	13,26	13,38	-0,12
**GDF11**	12,93	13,04	-0,12
**TTC9C**	10,77	10,89	-0,12
**SMTN**	10,18	10,30	-0,12
**AL355987.1**	8,27	8,38	-0,12
**ARAF**	11,24	11,36	-0,12
**CCDC7**	7,38	7,49	-0,12
**PLCXD2**	9,35	9,46	-0,12
**NF2**	11,96	12,08	-0,12
**UNK**	12,08	12,19	-0,12
**ERI1**	12,46	12,57	-0,12
**MTX3**	2,75	2,87	-0,12
**SMARCC2**	13,55	13,66	-0,12
**FTCDNL1**	6,59	6,70	-0,12
**ZNF684**	9,25	9,36	-0,12
**EPB41L4B**	7,75	7,87	-0,12
**MBD6**	11,62	11,73	-0,12
**PLEKHG2**	12,48	12,59	-0,12
**MPP3**	7,76	7,88	-0,12
**ULK2**	10,49	10,61	-0,12
**NMRAL1**	11,22	11,34	-0,12
**PSMD8**	13,40	13,52	-0,12

**GPAA1**	12,26	12,38	-0,12
**HAS3**	6,85	6,97	-0,12
**SYTL1**	9,69	9,81	-0,12
**GTF3C3**	10,64	10,76	-0,12
**PALD1**	12,31	12,43	-0,12
**FAM177A1**	9,34	9,46	-0,12
**PSPN**	8,20	8,32	-0,12
**YRDC**	11,45	11,57	-0,12
**INTS14**	11,22	11,33	-0,12
**PA2G4**	15,55	15,67	-0,12
**COPZ1**	13,12	13,23	-0,12
**ARF5**	12,66	12,78	-0,12
**TMEM129**	10,20	10,32	-0,12
**CCDC171**	8,60	8,72	-0,12
**UNC93B1**	11,90	12,02	-0,12
**IRF7**	9,22	9,33	-0,11
**YDJC**	10,72	10,83	-0,11
**KLHL7**	11,22	11,34	-0,11
**FRMD6**	5,92	6,04	-0,11
**KCTD21**	7,81	7,92	-0,11
**RAB3IL1**	5,12	5,23	-0,11
**SEC13**	13,00	13,12	-0,11
**MFAP3**	10,99	11,11	-0,11
**ZBTB4**	10,75	10,87	-0,11
**DNHD1**	10,45	10,57	-0,11
**ZNF20**	8,68	8,80	-0,11
**CMTM3**	11,91	12,02	-0,11
**C9orf78**	12,56	12,67	-0,11
**PPIB**	14,17	14,29	-0,11
**PIH1D1**	11,81	11,93	-0,11
**VLDLR**	11,60	11,71	-0,11
**UCK1**	10,98	11,10	-0,11
**CDCA3**	11,67	11,79	-0,11
**HERC3**	7,26	7,38	-0,11
**ENO3**	9,21	9,33	-0,11
**ZMAT5**	9,76	9,88	-0,11
**LRRC47**	11,70	11,81	-0,11
**COX4I1**	14,46	14,58	-0,11
**GLYR1**	13,43	13,55	-0,11
**TMEM164**	11,44	11,56	-0,11
**ETV3**	11,96	12,07	-0,11
**OVCA2**	9,66	9,78	-0,11
**VMAC**	8,13	8,24	-0,11
**DHFR2**	9,67	9,78	-0,11
**PLIN3**	12,95	13,06	-0,11
**KLHL3**	8,94	9,06	-0,11
**EXOSC3**	12,19	12,30	-0,11
**NIPSNAP1**	13,06	13,18	-0,11
**ING1**	11,36	11,47	-0,11
**DDX55**	12,35	12,47	-0,11
**ZCCHC3**	12,14	12,26	-0,11
**MPHOSPH8**	12,76	12,87	-0,11
**TUBG1**	12,33	12,44	-0,11
**GFOD1**	10,64	10,76	-0,11
**KIAA1328**	9,47	9,59	-0,11
**CEBPE**	6,27	6,39	-0,11

**MEF2C**	15,68	15,79	-0,11
**KLC4**	9,46	9,57	-0,11
**RABL2B**	10,58	10,69	-0,11
**DTX1**	10,67	10,78	-0,11
**TRIM25**	13,67	13,78	-0,11
**AC013489.1**	7,44	7,55	-0,11
**ABCA7**	9,13	9,24	-0,11
**DCHS1**	10,36	10,47	-0,11
**RPL34**	14,53	14,64	-0,11
**CMBL**	7,16	7,27	-0,11
**RPRD1B**	12,10	12,21	-0,11
**VPS37B**	12,78	12,89	-0,11
**HAUS4**	11,75	11,86	-0,11
**NHEJ1**	11,05	11,17	-0,11
**EIF4A3**	13,39	13,51	-0,11
**RTN4R**	11,19	11,30	-0,11
**WARS1**	12,15	12,26	-0,11
**VAMP1**	11,76	11,88	-0,11
**COL8A2**	8,43	8,54	-0,11
**ZNF3**	12,13	12,24	-0,11
**COX20**	11,32	11,43	-0,11
**HDDC3**	10,15	10,27	-0,11
**SLC10A5**	5,29	5,40	-0,11
**KCTD1**	10,80	10,92	-0,11
**E2F2**	14,25	14,37	-0,11
**TOM1**	10,12	10,23	-0,11
**FYN**	12,85	12,96	-0,11
**CRCP**	12,16	12,27	-0,11
**CCDC92**	9,85	9,97	-0,11
**PRDM15**	11,08	11,19	-0,11
**FOXN2**	12,05	12,16	-0,11
**MTRR**	11,80	11,92	-0,11
**CLCN6**	11,07	11,19	-0,11
**NDE1**	11,98	12,09	-0,11
**CSNK2A1**	14,26	14,37	-0,11
**EIF3K**	13,65	13,76	-0,11
**RPL27A**	15,68	15,79	-0,11
**D2HGDH**	11,08	11,20	-0,11
**HYI**	9,63	9,74	-0,11
**STIP1**	15,10	15,21	-0,11
**MSTO1**	12,29	12,40	-0,11
**KIF17**	4,98	5,09	-0,11
**EEF1AKNMT**	12,52	12,63	-0,11
**EGLN2**	11,94	12,05	-0,11
**UNC45A**	12,69	12,80	-0,11
**NRAP**	4,98	5,09	-0,11
**GSTP1**	14,97	15,08	-0,11
**FUNDC2**	11,65	11,76	-0,11
**TPD52**	13,15	13,27	-0,11
**FBRSL1**	14,17	14,28	-0,11
**AMFR**	12,16	12,27	-0,11
**UTP3**	12,10	12,21	-0,11
**CWF19L1**	11,82	11,93	-0,11
**TAP1**	11,96	12,07	-0,11
**NDUFS8**	11,61	11,73	-0,11
**NSFL1C**	12,04	12,15	-0,11

**KIFC1**	13,17	13,29	-0,11
**H3-3B**	15,54	15,65	-0,11
**SRP9**	15,13	15,25	-0,11
**IL7**	4,58	4,69	-0,11
**CD68**	7,01	7,12	-0,11
**IRF2BPL**	9,84	9,95	-0,11
**DVL1**	11,01	11,12	-0,11
**TMSB4X**	16,13	16,24	-0,11
**GALNT2**	14,75	14,86	-0,11
**IFT88**	10,08	10,20	-0,11
**PIAS4**	10,93	11,04	-0,11
**NOMO2**	11,19	11,30	-0,11
**RCSD1**	13,66	13,77	-0,11
**IL3RA**	8,02	8,13	-0,11
**ANKS3**	9,21	9,32	-0,11
**RPLP1**	16,18	16,29	-0,11
**NSUN5**	12,23	12,34	-0,11
**BLOC1S4**	10,02	10,13	-0,11
**ZNF212**	10,60	10,71	-0,11
**TSEN34**	10,58	10,69	-0,11
**FGFR2**	7,70	7,81	-0,11
**LZTFL1**	11,14	11,25	-0,11
**PLEKHG1**	8,64	8,75	-0,11
**RFX5**	12,95	13,06	-0,11
**SLC16A14**	9,61	9,72	-0,11
**FAM171A2**	9,76	9,87	-0,11
**AC018362.3**	5,68	5,79	-0,11
**ZNF420**	10,34	10,45	-0,11
**EFCAB11**	8,91	9,02	-0,11
**DOC2A**	9,33	9,44	-0,11
**KAT5**	11,30	11,41	-0,11
**KRBOX4**	9,87	9,98	-0,11
**MTMR8**	7,39	7,50	-0,11
**VPS16**	11,41	11,52	-0,11
**PSMC3IP**	10,07	10,18	-0,11
**ZBED3**	12,12	12,23	-0,11
**NKIRAS2**	11,77	11,88	-0,11
**RABAC1**	10,84	10,95	-0,11
**DGKI**	9,66	9,77	-0,11
**LSAMP**	5,03	5,14	-0,11
**AC068946.2**	7,96	8,07	-0,11
**PGBD4**	9,41	9,52	-0,11
**REXO1**	12,17	12,27	-0,11
**SF3A2**	13,31	13,42	-0,11
**NDST1**	12,41	12,52	-0,11
**PILRB**	10,85	10,96	-0,11
**PRNP**	12,40	12,51	-0,11
**PIM2**	10,51	10,62	-0,11
**PIP5K1A**	12,00	12,11	-0,11
**NOSIP**	11,54	11,65	-0,11
**GALM**	10,09	10,20	-0,11
**NANOS1**	5,44	5,55	-0,11
**SEMA4F**	9,32	9,43	-0,11
**RAB35**	11,84	11,95	-0,11
**FAHD2A**	11,03	11,14	-0,11
**ZNF358**	10,73	10,84	-0,11

**MCM2**	14,46	14,57	-0,11
**ENTPD6**	11,53	11,64	-0,11
**OPRD1**	4,02	4,13	-0,11
**MAT1A**	4,02	4,13	-0,11
**STXBP2**	11,55	11,66	-0,11
**ZNF333**	10,45	10,56	-0,11
**MROH6**	8,18	8,29	-0,11
**EVL**	12,92	13,03	-0,11
**TTLL1**	9,27	9,38	-0,11
**ADA**	12,83	12,94	-0,11
**NUP37**	11,20	11,30	-0,11
**BUD13**	11,78	11,89	-0,11
**SCYL1**	12,02	12,13	-0,11
**THAP7**	10,30	10,41	-0,11
**HSCB**	8,57	8,67	-0,11
**DBNDD2**	3,73	3,83	-0,11
**MTHFS**	9,17	9,28	-0,11
**UBN1**	13,08	13,18	-0,11
**ANKLE1**	12,32	12,43	-0,11
**AIP**	12,10	12,21	-0,11
**GPT2**	12,60	12,71	-0,11
**CDC73**	12,75	12,86	-0,11
**GOLGA8A**	13,48	13,59	-0,11
**ENTR1**	11,84	11,95	-0,11
**C9orf64**	6,55	6,66	-0,11
**NAPA**	12,04	12,15	-0,11
**SYS1**	9,79	9,90	-0,11
**MTCL1**	15,27	15,38	-0,11
**CHCHD7**	12,66	12,76	-0,11
**WDR90**	11,82	11,93	-0,11
**MT1H**	11,66	11,77	-0,11
**CREB3**	10,10	10,21	-0,11
**PLAC8L1**	6,38	6,49	-0,11
**RPS15**	15,08	15,19	-0,11
**EEF2**	17,30	17,40	-0,11
**TXN2**	11,82	11,92	-0,11
**DNAJB1**	13,21	13,32	-0,11
**SCN9A**	6,23	6,34	-0,11
**RSAD1**	11,88	11,99	-0,11
**SHKBP1**	12,61	12,72	-0,11
**SPRY1**	9,38	9,49	-0,11
**SPATA20**	7,11	7,22	-0,11
**AKR1B1**	14,32	14,43	-0,11
**SARS2**	10,50	10,61	-0,11
**JAK3**	10,51	10,62	-0,11
**EXOC7**	12,29	12,39	-0,11
**BCL7A**	13,46	13,57	-0,11
**ABCC5**	12,10	12,20	-0,11
**NLGN2**	11,26	11,37	-0,11
**TOR3A**	12,27	12,38	-0,11
**PDCD5**	12,39	12,49	-0,11
**WDR81**	11,21	11,31	-0,11
**MED9**	10,48	10,59	-0,11
**CAMTA1**	11,07	11,18	-0,11
**DDX28**	11,03	11,14	-0,11
**CINP**	10,83	10,93	-0,11

CBX6	12,57	12,68	-0,11
AL034430.2	6,74	6,84	-0,11
CSPG4	8,11	8,21	-0,11
EEF1AKMT1	9,57	9,68	-0,11
CABIN1	12,48	12,59	-0,11
DCUN1D2	10,98	11,08	-0,11
SNAPIN	11,05	11,15	-0,11
DDX60	9,36	9,46	-0,11
ZFP82	11,39	11,50	-0,11
PDPN	8,12	8,23	-0,11
SNUPN	11,44	11,54	-0,11
NOL7	12,61	12,72	-0,11
NUDT16	9,28	9,39	-0,11
CCDC88B	8,39	8,50	-0,11
PRKCD	11,51	11,61	-0,11
GDI1	12,70	12,81	-0,11
DNAJC5	12,76	12,86	-0,11
PRR5	11,25	11,36	-0,10
MRPL55	10,65	10,75	-0,10
SOX7	8,27	8,37	-0,10
MSN	14,85	14,95	-0,10
FDFT1	14,16	14,27	-0,10
ZMYND19	11,95	12,06	-0,10
IQCN	5,30	5,41	-0,10
IDUA	8,91	9,01	-0,10
JOSD1	12,48	12,58	-0,10
TADA2A	11,69	11,80	-0,10
ITPRIPL1	11,92	12,02	-0,10
WDR18	12,04	12,15	-0,10
NLE1	12,13	12,23	-0,10
SIGLEC15	4,95	5,06	-0,10
BEX3	12,95	13,05	-0,10
TMEM8B	9,28	9,38	-0,10
PTOV1	12,80	12,90	-0,10
AHDC1	11,35	11,46	-0,10
MICAL2	7,73	7,83	-0,10
RTF2	12,52	12,63	-0,10
PELI3	10,33	10,43	-0,10
TNFAIP3	12,46	12,56	-0,10
PGBD2	10,31	10,41	-0,10
ZNF677	9,42	9,53	-0,10
ZNF790	9,83	9,94	-0,10
TMEM121B	7,95	8,05	-0,10
NIBAN2	10,28	10,39	-0,10
TOMM22	13,23	13,33	-0,10
PRPSAP1	11,11	11,22	-0,10
LCK	9,97	10,07	-0,10
UBL7	11,20	11,30	-0,10
TMEM267	10,18	10,29	-0,10
S1PR1	7,15	7,26	-0,10
STK35	12,34	12,45	-0,10
RAB17	11,53	11,63	-0,10
ARFGAP2	12,76	12,86	-0,10
PTGER4	11,39	11,49	-0,10
AC073111.3	8,51	8,62	-0,10
HDGFL3	13,35	13,45	-0,10

**SETD1B**	12,19	12,29	-0,10
**ZNF577**	8,07	8,17	-0,10
**PTGIR**	7,32	7,42	-0,10
**BRCA1**	13,45	13,56	-0,10
**NOP14**	13,91	14,01	-0,10
**BCL10**	10,66	10,76	-0,10
**ADD2**	13,53	13,63	-0,10
**C1QTNF9B**	5,01	5,11	-0,10
**TPD52L2**	13,14	13,24	-0,10
**METTL17**	12,07	12,18	-0,10
**ZNF714**	11,88	11,98	-0,10
**SNAP47**	11,31	11,42	-0,10
**ENTPD5**	9,77	9,88	-0,10
**RFPL3**	6,00	6,11	-0,10
**METAP2**	13,79	13,89	-0,10
**RPS3**	16,88	16,98	-0,10
**ITGB2**	10,43	10,54	-0,10
**SPEM1**	3,11	3,21	-0,10
**TRGV2**	3,11	3,21	-0,10
**CARS2**	11,95	12,05	-0,10
**RNF215**	8,66	8,77	-0,10
**L3MBTL1**	9,29	9,39	-0,10
**SLX4**	10,08	10,18	-0,10
**WDR1**	14,58	14,68	-0,10
**ULK1**	12,41	12,51	-0,10
**ZNF512B**	12,04	12,14	-0,10
**PPIL3**	10,05	10,15	-0,10
**IWS1**	12,75	12,85	-0,10
**TIMM10B**	11,71	11,82	-0,10
**BID**	11,93	12,04	-0,10
**PLA2G15**	9,63	9,73	-0,10
**RNF165**	10,81	10,91	-0,10
**GMIP**	11,51	11,61	-0,10
**C19orf53**	11,77	11,87	-0,10
**TRMT12**	7,21	7,31	-0,10
**ZFP64**	11,14	11,24	-0,10
**PLCH2**	4,56	4,66	-0,10
**SLC39A3**	11,19	11,29	-0,10
**AP4S1**	9,16	9,26	-0,10
**TMX4**	13,08	13,18	-0,10
**SCML4**	7,78	7,88	-0,10
**MOCS3**	10,07	10,17	-0,10
**EIF4G3**	11,40	11,50	-0,10
**IL2RG**	10,59	10,70	-0,10
**ALDH1B1**	11,67	11,77	-0,10
**COQ8A**	12,19	12,29	-0,10
**HHAT**	8,60	8,70	-0,10
**FGD3**	11,58	11,68	-0,10
**CCDC15**	8,85	8,96	-0,10
**CEP72**	10,11	10,21	-0,10
**BCL6**	13,02	13,12	-0,10
**HP1BP3**	14,56	14,66	-0,10
**TP53I11**	12,78	12,89	-0,10
**LRRC26**	9,65	9,75	-0,10
**STING1**	9,42	9,52	-0,10
**TMEM9B**	9,96	10,06	-0,10

**TSPAN4**	8,63	8,73	-0,10
**GTF3C6**	12,65	12,75	-0,10
**MINDY4**	7,88	7,99	-0,10
**BASP1**	10,30	10,40	-0,10
**RPL27**	15,20	15,30	-0,10
**POLM**	10,93	11,03	-0,10
**ASPHD1**	6,68	6,78	-0,10
**CDC25B**	12,58	12,68	-0,10
**NEDD4L**	10,30	10,40	-0,10
**RAC3**	10,18	10,28	-0,10
**CASP8**	10,81	10,91	-0,10
**MC1R**	8,17	8,27	-0,10
**IL24**	3,41	3,51	-0,10
**GOLGA1**	9,48	9,58	-0,10
**CENPM**	11,67	11,77	-0,10
**DYRK3**	10,92	11,02	-0,10
**TRMT10B**	10,25	10,35	-0,10
**TENM2**	9,93	10,03	-0,10
**PPP1R8**	12,85	12,95	-0,10
**TNK2**	11,63	11,73	-0,10
**CCDC93**	12,38	12,48	-0,10
**CDCA8**	12,91	13,01	-0,10
**ACOT7**	13,13	13,23	-0,10
**NUDCD3**	12,43	12,53	-0,10
**RPS9**	15,87	15,97	-0,10
**ERMAP**	9,14	9,24	-0,10
**APBA3**	9,35	9,45	-0,10
**RNASEH2A**	12,14	12,24	-0,10
**DNAJC9**	13,16	13,26	-0,10
**DBF4B**	11,17	11,27	-0,10
**ZNF681**	11,25	11,35	-0,10
**GLIS2**	9,15	9,25	-0,10
**DEDD**	11,57	11,67	-0,10
**POLR3E**	12,57	12,67	-0,10
**CBY1**	6,15	6,25	-0,10
**SF1**	15,12	15,22	-0,10
**CACFD1**	9,62	9,72	-0,10
**ARSB**	10,93	11,03	-0,10
**MGST2**	7,52	7,62	-0,10
**BSG**	14,32	14,42	-0,10
**TPCN2**	9,71	9,81	-0,10
**TDRD12**	3,44	3,53	-0,10
**TSGA10**	6,77	6,86	-0,10
**KDM2B**	13,34	13,43	-0,10
**LIPC**	6,78	6,88	-0,10
**PKN1**	13,64	13,74	-0,10
**CTSZ**	11,16	11,26	-0,10
**ANKFY1**	12,54	12,64	-0,10
**PFDN5**	12,86	12,96	-0,10
**TTF2**	13,38	13,48	-0,10
**TMEM98**	11,46	11,56	-0,10
**MCM4**	15,96	16,05	-0,10
**DCTN2**	12,05	12,15	-0,10
**CCPG1**	10,83	10,93	-0,10
**SGK2**	4,49	4,58	-0,10
**TMEM106C**	12,79	12,89	-0,10

**AC022826.2**	6,27	6,37	-0,10
**FBXO41**	12,98	13,07	-0,10
**ADAM10**	13,96	14,06	-0,10
**QSOX1**	12,06	12,15	-0,10
**GNAQ**	10,25	10,34	-0,10
**EIF4E2**	12,36	12,46	-0,10
**NCOA5**	13,05	13,15	-0,10
**SLC1A5**	14,11	14,21	-0,10
**LSM12**	12,78	12,88	-0,10
**BAZ2B**	10,33	10,43	-0,10
**LYPLA2**	12,07	12,16	-0,10
**CCNQ**	10,40	10,50	-0,10
**RPL31**	15,57	15,67	-0,10
**RPL3**	17,28	17,38	-0,10
**FAM83E**	6,03	6,13	-0,10
**C6orf163**	6,11	6,20	-0,10
**WDR13**	10,88	10,98	-0,10
**TAX1BP3**	11,65	11,75	-0,10
**FAM168B**	13,61	13,71	-0,10
**ENY2**	13,69	13,79	-0,10
**NDUFA10**	13,21	13,31	-0,10
**POLR2A**	14,76	14,86	-0,10
**CTIF**	9,22	9,32	-0,10
**MARVELD1**	12,62	12,71	-0,10
**GPALPP1**	11,78	11,87	-0,10
**RPIA**	12,44	12,54	-0,10
**GPS1**	11,09	11,19	-0,10
**B3GAT1**	5,77	5,87	-0,10
**BMP1**	11,13	11,22	-0,10
**RPS25**	15,61	15,70	-0,10
**CSNK1E**	13,42	13,52	-0,10
**TXNL1**	12,83	12,93	-0,10
**OSCAR**	8,04	8,14	-0,10
**CD274**	5,74	5,83	-0,10
**CLN8**	11,48	11,58	-0,10
**POLL**	8,75	8,84	-0,10
**MRPL43**	9,40	9,50	-0,10
**ZSWIM6**	11,21	11,30	-0,10
**EML2**	9,16	9,26	-0,10
**ACSF2**	9,11	9,20	-0,10
**APLP2**	12,58	12,67	-0,10
**ST3GAL2**	11,36	11,45	-0,10
**FAM110A**	9,49	9,58	-0,10
**LAMTOR5**	12,27	12,37	-0,10
**KSR2**	3,85	3,94	-0,10
**TANGO2**	10,65	10,74	-0,10
**ZC3H4**	12,76	12,85	-0,10
**TPRA1**	10,48	10,57	-0,10
**ZNF503**	7,42	7,51	-0,10
**PXN**	10,61	10,71	-0,10
**MORF4L1**	14,62	14,72	-0,10
**RAB8A**	13,01	13,11	-0,10
**TJP2**	11,92	12,01	-0,10
**SIRT2**	11,11	11,20	-0,10
**IGFBP2**	11,87	11,97	-0,10
**H2BC12**	9,08	9,17	-0,10

**WDPCP**	9,71	9,81	-0,10
**AKT3**	12,31	12,40	-0,10
**ASB6**	11,48	11,58	-0,10
**HECTD3**	10,75	10,85	-0,10
**KLHL2**	11,51	11,61	-0,10
**TGDS**	10,41	10,51	-0,10
**CD63**	12,57	12,67	-0,10
**SLCO5A1**	11,69	11,78	-0,10
**RGS2**	8,44	8,53	-0,09
**TSNARE1**	6,45	6,55	-0,09
**FBLN1**	12,53	12,62	-0,09
**SCRN1**	5,85	5,94	-0,09
**DNER**	5,85	5,94	-0,09
**ISG20L2**	12,78	12,88	-0,09
**NLRX1**	10,43	10,52	-0,09
**UTP15**	11,75	11,85	-0,09
**WASF3**	7,45	7,54	-0,09
**PREB**	12,26	12,36	-0,09
**RPL19**	16,44	16,54	-0,09
**YPEL2**	7,84	7,93	-0,09
**NBR1**	11,15	11,24	-0,09
**GNL2**	13,47	13,56	-0,09
**TMEM184B**	11,19	11,29	-0,09
**ANKRD61**	6,74	6,83	-0,09
**OSBP2**	7,56	7,65	-0,09
**FOXM1**	13,20	13,30	-0,09
**SMIM12**	11,45	11,55	-0,09
**HK1**	12,52	12,62	-0,09
**PALB2**	11,32	11,41	-0,09
**FAM214A**	9,83	9,92	-0,09
**TRAF1**	7,77	7,87	-0,09
**CCDC130**	10,98	11,07	-0,09
**FAM186B**	7,78	7,87	-0,09
**HLA-B**	15,58	15,67	-0,09
**B3GLCT**	10,09	10,18	-0,09
**THAP9**	9,90	9,99	-0,09
**SMIM7**	11,64	11,73	-0,09
**NUB1**	12,04	12,13	-0,09
**EBF4**	8,39	8,48	-0,09
**ARHGEF17**	9,41	9,50	-0,09
**SH2D3C**	10,98	11,08	-0,09
**SATB1**	11,88	11,98	-0,09
**BECN1**	11,85	11,95	-0,09
**MSH3**	11,22	11,31	-0,09
**GTF2F1**	12,26	12,36	-0,09
**POLDIP2**	13,09	13,18	-0,09
**FEN1**	14,08	14,18	-0,09
**OTULINL**	4,87	4,97	-0,09
**CHRNA6**	4,87	4,97	-0,09
**PFDN4**	11,06	11,16	-0,09
**ATP5MG**	13,15	13,25	-0,09
**PDLIM7**	11,53	11,62	-0,09
**PLCXD1**	12,22	12,31	-0,09
**DESI1**	11,47	11,56	-0,09
**ZNF846**	7,93	8,02	-0,09
**TUBB4A**	11,90	11,99	-0,09

**H1-3**	3,70	3,79	-0,09
**MSRB2**	9,38	9,47	-0,09
**SIRT6**	10,22	10,32	-0,09
**PFKP**	13,28	13,38	-0,09
**EXOC4**	11,93	12,02	-0,09
**MRPL18**	12,20	12,29	-0,09
**HNRNPF**	15,28	15,37	-0,09
**SNX11**	10,21	10,30	-0,09
**GNB1**	15,18	15,27	-0,09
**NUDT16L1**	10,75	10,85	-0,09
**CLSTN3**	10,84	10,93	-0,09
**ZMYM5**	10,25	10,34	-0,09
**ZNF580**	10,55	10,64	-0,09
**OXNAD1**	10,45	10,54	-0,09
**RABGGTA**	10,08	10,17	-0,09
**EDC4**	13,24	13,33	-0,09
**KIAA0753**	10,99	11,09	-0,09
**SF3B5**	12,42	12,51	-0,09
**PPIG**	13,48	13,57	-0,09
**APH1A**	13,42	13,51	-0,09
**FMNL3**	11,28	11,37	-0,09
**SUPT6H**	13,14	13,23	-0,09
**RIC8B**	10,91	11,00	-0,09
**HIVEP1**	10,28	10,38	-0,09
**NCF4**	12,43	12,52	-0,09
**SPAG5**	13,18	13,27	-0,09
**CAND2**	9,29	9,38	-0,09
**LRP12**	11,21	11,30	-0,09
**MAU2**	12,30	12,39	-0,09
**NEK8**	9,12	9,21	-0,09
**PHLDB2**	10,98	11,07	-0,09
**FLI1**	14,36	14,45	-0,09
**POLR1H**	11,61	11,70	-0,09
**TRMT13**	9,10	9,19	-0,09
**GNB2**	13,50	13,59	-0,09
**SH2D2A**	11,28	11,38	-0,09
**NDUFA11**	12,68	12,77	-0,09
**AC022400.5**	9,55	9,64	-0,09
**ZSCAN22**	8,69	8,78	-0,09
**MSANTD4**	11,29	11,38	-0,09
**MPHOSPH6**	11,93	12,02	-0,09
**RSPH9**	5,07	5,16	-0,09
**POLD2**	13,57	13,66	-0,09
**KANK1**	10,67	10,76	-0,09
**FBXL12**	10,10	10,19	-0,09
**DCUN1D5**	12,17	12,26	-0,09
**UNG**	13,00	13,09	-0,09
**LTB4R2**	8,71	8,80	-0,09
**CHMP4A**	11,60	11,69	-0,09
**KANSL3**	12,90	12,99	-0,09
**GLA**	10,78	10,87	-0,09
**RPF1**	11,97	12,06	-0,09
**CCBE1**	4,00	4,09	-0,09
**RPP14**	11,74	11,83	-0,09
**ANKRD37**	7,59	7,68	-0,09
**AARS1**	14,45	14,54	-0,09

**SRPRA**	13,14	13,23	-0,09
**TMEM63A**	11,28	11,37	-0,09
**DMAP1**	11,00	11,09	-0,09
**CCDC167**	10,90	10,98	-0,09
**TBCA**	13,32	13,41	-0,09
**ZNF239**	5,99	6,08	-0,09
**UXT**	11,55	11,64	-0,09
**POLE3**	14,18	14,27	-0,09
**SWAP70**	11,48	11,57	-0,09
**GBP3**	8,96	9,04	-0,09
**UQCR11**	12,16	12,25	-0,09
**UNC13B**	10,46	10,54	-0,09
**MPO**	7,55	7,64	-0,09
**MPP2**	8,46	8,54	-0,09
**KANK3**	5,25	5,34	-0,09
**RGS16**	10,90	10,99	-0,09
**ABCD1**	11,01	11,10	-0,09
**ATP6AP2**	12,07	12,16	-0,09
**EGFL7**	11,20	11,29	-0,09
**SPG7**	12,74	12,83	-0,09
**TAF13**	9,25	9,33	-0,09
**FAM120C**	11,03	11,12	-0,09
**GLI3**	11,27	11,36	-0,09
**AC093323.1**	11,04	11,12	-0,09
**SMN2**	12,48	12,57	-0,09
**CCDC88C**	12,16	12,25	-0,09
**FAM120A**	14,37	14,46	-0,09
**DERL2**	11,51	11,60	-0,09
**XRRA1**	9,52	9,61	-0,09
**GPX4**	13,05	13,13	-0,09
**EHD1**	12,20	12,29	-0,09
**ACTR3**	14,47	14,56	-0,09
**CLTA**	13,75	13,83	-0,09
**ERO1A**	12,52	12,60	-0,09
**MYO19**	12,50	12,59	-0,09
**FAM118A**	11,20	11,29	-0,09
**LRSAM1**	9,97	10,05	-0,09
**ZNF816**	10,42	10,51	-0,09
**USP25**	12,04	12,13	-0,09
**CARHSP1**	13,41	13,49	-0,09
**DGAT1**	11,46	11,55	-0,09
**MACROD1**	9,90	9,99	-0,09
**SYNPO**	12,19	12,27	-0,09
**WAS**	12,29	12,38	-0,09
**RNF181**	10,47	10,56	-0,09
**ZNF280D**	11,28	11,37	-0,09
**QSOX2**	12,37	12,46	-0,09
**IFITM3**	9,91	9,99	-0,09
**DBI**	10,35	10,44	-0,09
**IL27RA**	8,87	8,96	-0,09
**ERI3**	11,77	11,86	-0,09
**CNBD2**	6,57	6,65	-0,09
**NOP56**	14,92	15,01	-0,09
**C1GALT1C1**	9,48	9,56	-0,09
**PDCD7**	12,49	12,58	-0,09
**PSMA7**	14,10	14,18	-0,09

**SGTB**	10,14	10,22	-0,09
**C4orf33**	8,55	8,63	-0,09
**RALB**	9,65	9,74	-0,09
**ATP9B**	10,52	10,60	-0,09
**TICAM1**	9,79	9,87	-0,09
**GAMT**	11,93	12,02	-0,09
**TMEM121**	10,87	10,96	-0,09
**PARP1**	16,58	16,67	-0,09
**SSBP1**	13,33	13,41	-0,09
**ARHGDIB**	15,91	15,99	-0,09
**RABL6**	13,74	13,83	-0,09
**SETDB2**	10,87	10,96	-0,09
**RPS14**	16,38	16,46	-0,09
**TMEM33**	13,13	13,22	-0,09
**RPS6KC1**	9,70	9,79	-0,09
**ZMIZ1**	14,17	14,25	-0,09
**NDUFAF6**	11,56	11,64	-0,09
**ATP6V0A2**	12,49	12,58	-0,09
**ENPP2**	4,44	4,53	-0,09
**PDCL**	11,68	11,77	-0,09
**GMPR2**	11,67	11,76	-0,09
**ANGEL1**	11,62	11,70	-0,09
**C16orf91**	9,92	10,00	-0,09
**NDUFB2**	12,14	12,22	-0,09
**ABHD10**	11,81	11,89	-0,09
**DTYMK**	12,20	12,29	-0,09
**PODXL**	12,14	12,23	-0,09
**MYO1C**	10,09	10,17	-0,09
**FADS3**	11,56	11,65	-0,09
**ALDOA**	16,09	16,17	-0,09
**SNX12**	11,14	11,22	-0,09
**ARPC4**	13,41	13,50	-0,09
**RABGEF1**	11,11	11,20	-0,09
**CALCOCO1**	9,79	9,88	-0,08
**NRGN**	9,58	9,67	-0,08
**PRPF39**	12,32	12,40	-0,08
**APBA1**	13,57	13,66	-0,08
**NNT**	13,97	14,05	-0,08
**OGDHL**	8,57	8,65	-0,08
**GFI1**	10,79	10,87	-0,08
**MTMR7**	8,37	8,46	-0,08
**ZMIZ2**	12,28	12,36	-0,08
**AKR1A1**	13,59	13,67	-0,08
**CDK2AP2**	12,20	12,29	-0,08
**SYNGR2**	11,70	11,79	-0,08
**PLEKHA8**	11,30	11,38	-0,08
**SLC10A3**	9,65	9,74	-0,08
**AKIRIN2**	10,32	10,41	-0,08
**MEN1**	12,01	12,09	-0,08
**MMP11**	10,88	10,96	-0,08
**KAT2A**	12,77	12,85	-0,08
**IFITM1**	10,47	10,55	-0,08
**RPL30**	16,02	16,10	-0,08
**DENND1A**	10,98	11,07	-0,08
**TRMT10A**	9,85	9,94	-0,08
**GYPC**	12,58	12,66	-0,08

**TTC32**	8,65	8,73	-0,08
**TEX52**	4,70	4,79	-0,08
**HNRNPA1**	17,97	18,05	-0,08
**ZNF331**	12,02	12,10	-0,08
**CWC25**	11,95	12,03	-0,08
**IPO13**	11,51	11,60	-0,08
**ARHGEF10L**	10,08	10,17	-0,08
**GP9**	7,37	7,45	-0,08
**BYSL**	12,27	12,36	-0,08
**TMED3**	11,79	11,87	-0,08
**SLC7A1**	13,81	13,89	-0,08
**RGL4**	9,95	10,03	-0,08
**KCTD11**	7,15	7,23	-0,08
**ATF6B**	12,61	12,70	-0,08
**PHF1**	10,11	10,20	-0,08
**CBFA2T2**	11,14	11,22	-0,08
**ISG20**	7,77	7,85	-0,08
**SIGIRR**	10,16	10,24	-0,08
**CRK**	12,09	12,17	-0,08
**SOX13**	3,39	3,47	-0,08
**FAM76A**	10,37	10,45	-0,08
**DDX39A**	14,02	14,10	-0,08
**MAP4K4**	13,71	13,79	-0,08
**POLR1C**	11,86	11,95	-0,08
**POGK**	13,69	13,78	-0,08
**CARS1**	11,92	12,00	-0,08
**TIPRL**	12,58	12,67	-0,08
**SPA17**	9,52	9,60	-0,08
**TRIT1**	10,94	11,02	-0,08
**MT-ND2**	17,59	17,68	-0,08
**TMEM107**	9,38	9,46	-0,08
**PLTP**	9,75	9,84	-0,08
**ULK3**	11,44	11,52	-0,08
**PRPF19**	14,32	14,41	-0,08
**GBGT1**	11,56	11,64	-0,08
**PNP**	14,00	14,08	-0,08
**PCBP1**	15,24	15,33	-0,08
**RUVBL1**	13,39	13,47	-0,08
**MRPL17**	11,86	11,94	-0,08
**MSL1**	13,53	13,61	-0,08
**HMGB1**	17,58	17,66	-0,08
**TIGD2**	10,06	10,14	-0,08
**PGM2**	12,95	13,03	-0,08
**AGMAT**	9,57	9,65	-0,08
**ZDHHC18**	11,44	11,52	-0,08
**SND1**	14,39	14,47	-0,08
**PARD3B**	7,63	7,71	-0,08
**ELP4**	10,97	11,05	-0,08
**TP53I3**	7,05	7,13	-0,08
**EIF2B5**	12,92	13,00	-0,08
**PIGZ**	7,51	7,59	-0,08
**SGCB**	11,13	11,21	-0,08
**ELOVL6**	12,52	12,60	-0,08
**PDZD11**	10,28	10,36	-0,08
**RFTN1**	12,55	12,63	-0,08
**KIAA1522**	8,96	9,04	-0,08

**DNAJA4**	5,29	5,37	-0,08
**SLC35F2**	12,34	12,42	-0,08
**MRPL38**	11,86	11,94	-0,08
**AAMP**	12,59	12,67	-0,08
**WRAP73**	10,68	10,76	-0,08
**AL031315.1**	5,49	5,57	-0,08
**MAZ**	15,00	15,08	-0,08
**TLR7**	7,65	7,73	-0,08
**ERF**	11,58	11,66	-0,08
**SIAH2**	12,62	12,70	-0,08
**SHFL**	10,34	10,42	-0,08
**NEDD8**	12,70	12,78	-0,08
**FOXO3B**	10,46	10,54	-0,08
**ANKMY1**	10,74	10,82	-0,08
**PSME2**	13,64	13,72	-0,08
**ZNF496**	11,76	11,84	-0,08
**SDHB**	12,32	12,39	-0,08
**KLHL21**	11,90	11,98	-0,08
**RPL23**	16,06	16,14	-0,08
**ITPK1**	11,79	11,87	-0,08
**MRFAP1L1**	12,41	12,49	-0,08
**PCIF1**	11,63	11,71	-0,08
**PSMB7**	13,48	13,56	-0,08
**PAQR6**	7,96	8,04	-0,08
**MYOZ3**	9,15	9,23	-0,08
**PCK2**	11,68	11,76	-0,08
**CCDC61**	9,22	9,30	-0,08
**SF3B6**	12,17	12,25	-0,08
**SVIL**	11,24	11,32	-0,08
**NABP2**	12,44	12,52	-0,08
**MEMO1**	12,25	12,33	-0,08
**HINT1**	13,98	14,06	-0,08
**FRAT2**	10,93	11,00	-0,08
**TMEM97**	12,72	12,80	-0,08
**ZNF488**	8,45	8,52	-0,08
**MKRN1**	12,16	12,24	-0,08
**TSC22D3**	10,02	10,10	-0,08
**LSM1**	11,79	11,87	-0,08
**PIGS**	11,46	11,54	-0,08
**CANX**	15,89	15,97	-0,08
**STK33**	7,52	7,60	-0,08
**PLGLB1**	9,59	9,67	-0,08
**ERCC1**	12,58	12,66	-0,08
**OAZ1**	15,26	15,34	-0,08
**BNC2**	11,13	11,21	-0,08
**PRR13**	13,24	13,32	-0,08
**SLFN5**	10,87	10,95	-0,08
**HMOX2**	11,54	11,62	-0,08
**EMG1**	12,55	12,63	-0,08
**RASSF1**	11,32	11,40	-0,08
**KXD1**	12,16	12,24	-0,08
**PARS2**	9,06	9,14	-0,08
**ALKBH8**	11,08	11,16	-0,08
**STAP2**	8,26	8,34	-0,08
**PIGU**	11,09	11,16	-0,08
**CDC42BPG**	6,21	6,29	-0,08

**PTPRN2**	6,21	6,29	-0,08
**SYNCRIP**	14,77	14,84	-0,08
**FZD2**	10,72	10,80	-0,08
**GEMIN2**	9,26	9,34	-0,08
**VPS25**	10,53	10,61	-0,08
**BDH1**	11,81	11,89	-0,08
**KCNJ2**	10,56	10,64	-0,08
**PRXL2A**	12,84	12,92	-0,08
**ATP5MPL**	12,18	12,26	-0,08
**COMMD6**	10,65	10,72	-0,08
**PHB2**	14,52	14,59	-0,08
**DUSP1**	11,74	11,81	-0,08
**RAP2A**	12,09	12,17	-0,08
**KCNH4**	6,86	6,94	-0,08
**TIMM23B**	10,37	10,45	-0,08
**EPB41L1**	9,16	9,23	-0,08
**L3HYPDH**	9,20	9,28	-0,08
**IMMP2L**	9,97	10,05	-0,08
**LIMD1**	12,99	13,07	-0,08
**TSPOAP1**	11,79	11,87	-0,08
**RNF152**	10,15	10,23	-0,08
**MRPS12**	11,59	11,66	-0,08
**PPP1CA**	13,76	13,84	-0,08
**JMJD4**	11,10	11,17	-0,08
**ZFAT**	9,83	9,90	-0,08
**CUTA**	12,64	12,72	-0,08
**GNAL**	8,00	8,08	-0,08
**MAML3**	11,63	11,71	-0,08
**TSR1**	13,74	13,82	-0,08
**EEF1AKMT2**	9,38	9,45	-0,08
**DTX3**	9,51	9,59	-0,08
**ARPC1B**	12,78	12,86	-0,08
**CORO1B**	10,83	10,91	-0,08
**FBXL6**	10,64	10,71	-0,08
**CHMP3**	11,71	11,79	-0,08
**CHKB**	9,72	9,80	-0,08
**NENF**	10,95	11,03	-0,08
**PTPRJ**	10,42	10,50	-0,08
**ALPK1**	7,31	7,39	-0,08
**GCDH**	11,65	11,73	-0,08
**UFC1**	12,96	13,03	-0,08
**CCDC85C**	11,80	11,88	-0,08
**EIF5B**	14,48	14,55	-0,08
**RPL24**	15,74	15,81	-0,08
**RPL13**	16,56	16,64	-0,08
**PRR19**	6,02	6,10	-0,08
**UBR4**	13,95	14,03	-0,08
**UTP25**	11,92	11,99	-0,08
**PLD6**	11,62	11,69	-0,08
**ATP2B1**	12,44	12,52	-0,08
**BCS1L**	11,55	11,62	-0,08
**RPS20**	16,47	16,54	-0,08
**ALOX15**	4,05	4,13	-0,08
**RPL18A**	16,38	16,45	-0,08
**CPSF4**	11,33	11,40	-0,08
**DNAJC21**	12,68	12,75	-0,08

**TOMM40L**	10,47	10,55	-0,08
**COTL1**	11,72	11,79	-0,08
**STX8**	10,24	10,32	-0,08
**MCM9**	11,28	11,35	-0,08
**ABCB6**	10,30	10,38	-0,08
**POLH**	12,64	12,72	-0,08
**SDHC**	12,59	12,67	-0,08
**SNAPC2**	9,17	9,24	-0,07
**C12orf43**	11,23	11,31	-0,07
**SUSD4**	5,71	5,78	-0,07
**DNAL4**	9,04	9,11	-0,07
**LPCAT1**	13,64	13,72	-0,07
**JUP**	13,97	14,04	-0,07
**NDUFA12**	12,07	12,15	-0,07
**SRSF6**	15,20	15,27	-0,07
**AK2**	14,45	14,52	-0,07
**FNDC10**	8,97	9,05	-0,07
**ABI2**	12,79	12,86	-0,07
**PPIP5K2**	13,13	13,20	-0,07
**RPA3**	10,95	11,03	-0,07
**CPSF7**	13,63	13,71	-0,07
**CALML4**	9,80	9,88	-0,07
**ZNF768**	11,11	11,18	-0,07
**SNRPC**	13,07	13,14	-0,07
**CCDC71**	10,36	10,44	-0,07
**NBPF14**	12,29	12,36	-0,07
**NOB1**	12,83	12,91	-0,07
**DLGAP4**	11,25	11,32	-0,07
**PSMA3**	13,62	13,69	-0,07
**BRD8**	12,52	12,60	-0,07
**NOMO1**	13,18	13,26	-0,07
**ZZEF1**	12,79	12,86	-0,07
**SZRD1**	13,61	13,68	-0,07
**FAM131A**	9,26	9,33	-0,07
**SHROOM3**	11,05	11,12	-0,07
**BMP4**	7,55	7,62	-0,07
**PPARGC1B**	11,84	11,91	-0,07
**GNG5**	12,04	12,11	-0,07
**PPP2R3B**	11,05	11,13	-0,07
**GYPE**	8,45	8,53	-0,07
**BAZ2A**	13,32	13,39	-0,07
**ARL4D**	8,02	8,09	-0,07
**ZCCHC2**	11,34	11,41	-0,07
**DDX19A**	12,80	12,87	-0,07
**CARM1**	13,21	13,29	-0,07
**HAX1**	12,45	12,52	-0,07
**KBTBD2**	11,81	11,88	-0,07
**CHD1L**	13,47	13,54	-0,07
**PCGF1**	9,12	9,20	-0,07
**LNX2**	10,61	10,68	-0,07
**SLC35E2B**	13,00	13,07	-0,07
**DNAH11**	5,16	5,24	-0,07
**LIG1**	13,24	13,32	-0,07
**BDH2**	11,22	11,29	-0,07
**CYP2D6**	6,73	6,80	-0,07
**MRPL27**	11,15	11,22	-0,07

**TRMT2B**	11,34	11,41	-0,07
**ACO2**	13,14	13,21	-0,07
**ZWINT**	13,65	13,72	-0,07
**CCDC87**	4,76	4,83	-0,07
**SNX1**	13,37	13,44	-0,07
**TMEM223**	9,56	9,63	-0,07
**CCDC127**	10,83	10,90	-0,07
**HK2**	13,19	13,26	-0,07
**ARHGAP42**	10,61	10,68	-0,07
**ABITRAM**	9,97	10,04	-0,07
**C8orf44-SGK3**	7,26	7,33	-0,07
**SCLY**	11,32	11,39	-0,07
**CACNA2D4**	12,63	12,70	-0,07
**STYXL1**	10,42	10,49	-0,07
**PSMC2**	13,34	13,41	-0,07
**GANAB**	14,60	14,67	-0,07
**NOLC1**	14,99	15,06	-0,07
**EXOSC2**	11,81	11,88	-0,07
**TPI1**	14,43	14,50	-0,07
**KCTD10**	12,14	12,21	-0,07
**IRAK1**	13,79	13,86	-0,07
**ISCU**	11,29	11,36	-0,07
**RREB1**	11,61	11,68	-0,07
**NDRG4**	6,62	6,69	-0,07
**ZNF423**	11,34	11,41	-0,07
**WASF2**	13,83	13,90	-0,07
**VTI1B**	12,22	12,29	-0,07
**RBM39**	14,75	14,82	-0,07
**ADPRS**	11,13	11,20	-0,07
**MFNG**	12,53	12,60	-0,07
**ELF2**	12,05	12,12	-0,07
**TSPYL1**	12,24	12,31	-0,07
**ZNF197**	11,08	11,15	-0,07
**SGSM2**	11,85	11,92	-0,07
**ASXL3**	7,78	7,85	-0,07
**RGS19**	11,11	11,18	-0,07
**BAK1**	11,01	11,08	-0,07
**MEGF9**	11,41	11,48	-0,07
**FAM126A**	11,88	11,95	-0,07
**SCAMP2**	11,85	11,92	-0,07
**DUSP4**	12,00	12,07	-0,07
**PDXK**	10,58	10,65	-0,07
**GPSM3**	11,23	11,30	-0,07
**CAPN3**	11,91	11,98	-0,07
**SEC16A**	12,88	12,95	-0,07
**RGP1**	10,27	10,34	-0,07
**DUSP5**	3,82	3,88	-0,07
**NODAL**	3,82	3,88	-0,07
**SVIP**	12,10	12,17	-0,07
**CYP21A2**	5,17	5,24	-0,07
**SPOUT1**	11,81	11,88	-0,07
**GALT**	9,65	9,72	-0,07
**ARRDC3**	11,19	11,26	-0,07
**CYB5R3**	7,55	7,62	-0,07
**TAGLN2**	15,34	15,41	-0,07
**SLC35A2**	10,51	10,58	-0,07

**ZNF546**	9,71	9,78	-0,07
**NUP62**	14,32	14,39	-0,07
**PGM1**	11,85	11,92	-0,07
**CAPZB**	13,66	13,73	-0,07
**SCAF4**	13,07	13,14	-0,07
**GPR137B**	7,56	7,63	-0,07
**SPATC1L**	8,84	8,91	-0,07
**PIGO**	10,96	11,02	-0,07
**USP39**	13,08	13,14	-0,07
**GTF3C1**	13,16	13,23	-0,07
**USP54**	9,87	9,94	-0,07
**BCL2L12**	11,36	11,43	-0,07
**MRTO4**	13,63	13,70	-0,07
**TNFRSF13C**	11,86	11,93	-0,07
**ZKSCAN4**	9,92	9,99	-0,07
**FSD1**	9,92	9,98	-0,07
**KANSL2**	11,68	11,75	-0,07
**PARK7**	13,90	13,97	-0,07
**MBD2**	12,72	12,79	-0,07
**PRPF8**	15,73	15,80	-0,07
**RNF26**	11,83	11,90	-0,07
**HMGN1**	15,38	15,45	-0,07
**LEPROTL1**	12,91	12,98	-0,07
**IFRD1**	11,05	11,12	-0,07
**WBP4**	11,08	11,15	-0,07
**TOP3A**	12,76	12,83	-0,07
**MAPK12**	11,32	11,39	-0,07
**SCO1**	12,53	12,59	-0,07
**ATP5MC3**	14,31	14,37	-0,07
**DGKD**	13,59	13,66	-0,07
**PAK4**	10,73	10,80	-0,07
**LRRTM1**	2,77	2,84	-0,07
**NSMCE3**	11,26	11,33	-0,07
**KDELR1**	12,60	12,66	-0,07
**ACOT1**	6,73	6,80	-0,07
**AKAP1**	13,28	13,34	-0,07
**HSPB1**	13,84	13,91	-0,07
**CCDC174**	10,94	11,01	-0,07
**ISCA2**	10,55	10,62	-0,07
**CHTF8**	12,10	12,17	-0,07
**MN1**	5,11	5,17	-0,07
**NDUFC2**	12,60	12,67	-0,07
**SETMAR**	10,21	10,27	-0,07
**METAP1D**	10,18	10,25	-0,07
**DELE1**	11,58	11,64	-0,07
**ALDH3A2**	11,57	11,64	-0,07
**TIPIN**	11,36	11,42	-0,07
**CAPG**	12,19	12,25	-0,07
**C5orf58**	3,28	3,35	-0,07
**SERTAD4**	3,28	3,35	-0,07
**IL1A**	3,28	3,35	-0,07
**DIO1**	3,28	3,35	-0,07
**PTGER3**	3,28	3,35	-0,07
**RAB25**	3,28	3,35	-0,07
**VGLL3**	3,28	3,35	-0,07
**DYNLT2B**	9,60	9,66	-0,07

**MRTFA**	11,54	11,60	-0,07
**MST1R**	5,26	5,33	-0,07
**RPS12**	16,25	16,32	-0,07
**MYG1**	11,28	11,35	-0,07
**UTP14A**	12,79	12,85	-0,07
**MT1X**	14,49	14,56	-0,07
**RRAGA**	11,70	11,77	-0,07
**FDPS**	13,79	13,86	-0,07
**ADARB1**	12,80	12,86	-0,07
**VASN**	5,64	5,71	-0,07
**DDHD2**	12,78	12,84	-0,07
**WWC2**	8,67	8,74	-0,07
**ATP6V1H**	11,80	11,86	-0,07
**UVSSA**	10,63	10,70	-0,07
**BCL11B**	10,82	10,88	-0,07
**KDM1A**	14,15	14,22	-0,07
**ATP6V0C**	12,52	12,59	-0,07
**CDK10**	11,46	11,52	-0,07
**RANBP1**	14,83	14,90	-0,07
**ZNF544**	11,66	11,73	-0,07
**RPL8**	16,91	16,97	-0,07
**SF3B4**	13,36	13,42	-0,07
**ANKRD13A**	12,30	12,37	-0,07
**DNAJC14**	12,06	12,12	-0,07
**SHANK1**	12,53	12,60	-0,07
**VPS28**	12,35	12,42	-0,07
**RHBDD2**	10,62	10,68	-0,07
**SLC37A4**	11,44	11,50	-0,07
**ZXDA**	9,22	9,29	-0,07
**CEP85**	12,47	12,54	-0,07
**WBP1L**	11,10	11,16	-0,07
**TRAPPC13**	11,08	11,14	-0,07
**HENMT1**	10,39	10,46	-0,07
**SRCAP**	13,47	13,54	-0,07
**NTN5**	3,36	3,42	-0,07
**CDK16**	12,28	12,35	-0,07
**ERCC2**	10,62	10,68	-0,07
**BPNT2**	13,84	13,91	-0,07
**RTL8A**	10,16	10,22	-0,07
**TM2D2**	11,63	11,70	-0,07
**DENND3**	12,32	12,38	-0,07
**JRK**	12,94	13,01	-0,07
**SFMBT2**	12,36	12,43	-0,07
**WDR75**	13,01	13,08	-0,07
**RFXANK**	11,46	11,52	-0,07
**PKM**	16,64	16,71	-0,07
**DIRAS1**	11,97	12,04	-0,07
**PPIF**	13,51	13,58	-0,07
**KIAA0825**	7,06	7,12	-0,07
**OGG1**	8,90	8,97	-0,07
**MUL1**	10,66	10,72	-0,07
**GPATCH8**	12,93	13,00	-0,07
**MED7**	10,33	10,39	-0,06
**UBL4A**	12,03	12,09	-0,06
**ALOX5**	7,89	7,95	-0,06
**GABRR2**	6,19	6,25	-0,06

**FAM160B2**	11,26	11,33	-0,06
**CACNB1**	9,83	9,89	-0,06
**ELAVL1**	14,38	14,44	-0,06
**TSPYL2**	10,84	10,91	-0,06
**LMF1**	9,59	9,65	-0,06
**HEATR5A**	9,81	9,88	-0,06
**NTAN1**	10,67	10,74	-0,06
**NOP10**	11,78	11,84	-0,06
**RIPK2**	10,99	11,06	-0,06
**IL12A**	6,09	6,15	-0,06
**SPECC1**	9,75	9,82	-0,06
**HELLS**	13,48	13,55	-0,06
**PTGDR2**	5,21	5,27	-0,06
**TRAF3IP2**	13,28	13,34	-0,06
**BVES**	10,07	10,14	-0,06
**MRPS23**	12,19	12,25	-0,06
**MRPL37**	13,08	13,14	-0,06
**RPS11**	16,64	16,71	-0,06
**ZDHHC8**	10,78	10,85	-0,06
**TRIM4**	11,17	11,23	-0,06
**SP110**	11,14	11,20	-0,06
**SOD2**	13,24	13,30	-0,06
**KLHL6**	11,62	11,68	-0,06
**RNASEL**	11,17	11,23	-0,06
**BCAT2**	11,45	11,51	-0,06
**TNRC6C**	10,42	10,48	-0,06
**LRRC8A**	11,37	11,43	-0,06
**OSBP**	12,55	12,62	-0,06
**TGIF2**	12,49	12,55	-0,06
**CRAT**	10,39	10,46	-0,06
**PSMD4**	13,72	13,79	-0,06
**PIGB**	9,46	9,52	-0,06
**PROSER2**	9,60	9,67	-0,06
**PSMB9**	12,41	12,47	-0,06
**STX10**	11,08	11,14	-0,06
**TUT1**	10,57	10,64	-0,06
**APOL1**	9,74	9,80	-0,06
**NSL1**	12,14	12,20	-0,06
**OXSR1**	12,57	12,63	-0,06
**CD47**	13,45	13,51	-0,06
**PPP2R1A**	14,36	14,43	-0,06
**TTC38**	7,07	7,13	-0,06
**ZNF880**	10,28	10,34	-0,06
**HEXB**	11,60	11,66	-0,06
**UBL3**	10,73	10,79	-0,06
**FAM161B**	7,42	7,48	-0,06
**SLC25A13**	11,46	11,52	-0,06
**CTNNAL1**	12,29	12,36	-0,06
**PLXNA3**	11,63	11,69	-0,06
**CRIPT**	10,22	10,28	-0,06
**TUBB**	17,79	17,86	-0,06
**PRKAB1**	10,92	10,98	-0,06
**PAWR**	12,42	12,48	-0,06
**MTCP1**	8,00	8,06	-0,06
**ARID3B**	9,17	9,23	-0,06
**LCMT1**	10,74	10,80	-0,06

FAM78A	5,37	5,43	-0,06
PDCD2L	10,03	10,09	-0,06
ZCCHC17	11,40	11,46	-0,06
SUV39H1	11,52	11,58	-0,06
NETO2	12,64	12,71	-0,06
NECAP2	11,41	11,47	-0,06
ARID4B	13,06	13,12	-0,06
NAP1L5	7,24	7,30	-0,06
MTERF4	11,14	11,20	-0,06
SMCR8	11,88	11,94	-0,06
GRAMD1A	12,51	12,57	-0,06
CELSR3	8,70	8,76	-0,06
ITFG2	11,29	11,36	-0,06
RRAGC	10,95	11,01	-0,06
LYRM4	11,51	11,57	-0,06
SPTLC3	8,76	8,82	-0,06
DNAJB9	10,30	10,36	-0,06
RBM6	13,36	13,42	-0,06
MICALL1	11,57	11,63	-0,06
PON2	9,53	9,60	-0,06
AP4M1	10,91	10,97	-0,06
TNNC1	2,42	2,48	-0,06
H2AC12	2,42	2,48	-0,06
DCST2	2,42	2,48	-0,06
TMEM241	9,92	9,98	-0,06
MRPL32	12,26	12,32	-0,06
RPS24	16,01	16,07	-0,06
WDR45	9,89	9,95	-0,06
TMEM185B	12,13	12,19	-0,06
SCARF1	11,06	11,12	-0,06
C6orf226	6,60	6,66	-0,06
TADA2B	11,16	11,22	-0,06
SRSF1	16,20	16,26	-0,06
NEU1	9,71	9,77	-0,06
FAM53B	11,14	11,20	-0,06
CDIP1	11,74	11,80	-0,06
SLC15A3	8,07	8,13	-0,06
GAB3	10,06	10,12	-0,06
GLB1L2	9,99	10,05	-0,06
UBA52	14,95	15,01	-0,06
MAPKAPK2	14,00	14,06	-0,06
HACL1	10,54	10,60	-0,06
FAM136A	12,62	12,68	-0,06
ZBTB43	10,39	10,45	-0,06
HYPK	9,41	9,47	-0,06
MSS51	8,06	8,12	-0,06
FSTL1	12,03	12,09	-0,06
MPC2	11,54	11,60	-0,06
PPIH	12,77	12,83	-0,06
PDK2	9,69	9,75	-0,06
SEC24C	13,44	13,50	-0,06
HECW1	10,26	10,32	-0,06
ZNF19	7,69	7,75	-0,06
ZFYVE19	10,03	10,09	-0,06
ASB1	11,02	11,08	-0,06
TEAD4	11,48	11,54	-0,06

**MRRF**	11,43	11,49	-0,06
**RPTOR**	11,78	11,84	-0,06
**NDUFA7**	10,60	10,66	-0,06
**PDXDC1**	13,11	13,17	-0,06
**CMSS1**	12,60	12,65	-0,06
**DRICH1**	6,46	6,52	-0,06
**MAN2B1**	12,15	12,21	-0,06
**TCEAL1**	9,27	9,33	-0,06
**FAAH**	10,94	11,00	-0,06
**NRG3**	11,03	11,09	-0,06
**COG3**	11,29	11,35	-0,06
**SLC24A1**	9,26	9,32	-0,06
**KHDRBS1**	14,88	14,94	-0,06
**UBALD1**	9,81	9,87	-0,06
**DYNC1LI1**	12,55	12,61	-0,06
**CHST11**	12,29	12,35	-0,06
**CDC123**	13,53	13,59	-0,06
**DNAJA1**	15,38	15,44	-0,06
**GRK2**	15,22	15,28	-0,06
**TALDO1**	13,39	13,45	-0,06
**POMGNT1**	10,54	10,60	-0,06
**APMAP**	12,21	12,27	-0,06
**PSMB8**	12,89	12,95	-0,06
**CCNG2**	11,46	11,52	-0,06
**IGHD**	12,55	12,61	-0,06
**BAP1**	12,89	12,94	-0,06
**FKBP4**	14,03	14,09	-0,06
**QRICH2**	8,25	8,31	-0,06
**MOSPD1**	9,40	9,46	-0,06
**SLC25A46**	12,79	12,85	-0,06
**YPEL5**	10,26	10,32	-0,06
**PNISR**	14,03	14,09	-0,06
**MT1G**	13,20	13,26	-0,06
**DNAAF2**	10,65	10,71	-0,06
**CYB561A3**	11,65	11,71	-0,06
**HIBCH**	11,20	11,26	-0,06
**RMND5B**	10,09	10,15	-0,06
**OSBPL5**	8,89	8,95	-0,06
**ZNRF2**	10,95	11,01	-0,06
**PTPRF**	12,29	12,34	-0,06
**NBPF1**	11,34	11,40	-0,06
**CLPTM1**	12,11	12,17	-0,06
**JTB**	13,18	13,24	-0,06
**TRIM8**	12,32	12,38	-0,06
**CAD**	14,01	14,07	-0,06
**BUD31**	12,42	12,48	-0,06
**TERF2IP**	12,83	12,89	-0,06
**ZNF202**	11,50	11,56	-0,06
**ZNF213**	9,45	9,51	-0,06
**MRPL15**	13,09	13,14	-0,06
**TRMU**	10,79	10,85	-0,06
**EIF2AK3**	11,37	11,43	-0,06
**VIM**	16,46	16,52	-0,06
**TAS1R3**	5,93	5,99	-0,06
**CLIC1**	13,82	13,88	-0,06
**ATP5PD**	12,22	12,28	-0,06

**PRR22**	8,99	9,05	-0,06
**DNMBP**	10,53	10,59	-0,06
**CMTM4**	10,22	10,28	-0,06
**TBC1D20**	11,53	11,59	-0,06
**CMPK2**	9,55	9,61	-0,06
**MXRA8**	4,92	4,98	-0,06
**ATG4C**	10,36	10,41	-0,06
**SNW1**	13,18	13,24	-0,06
**CLUAP1**	10,63	10,69	-0,06
**YIPF1**	9,59	9,65	-0,06
**NFKB1**	13,47	13,53	-0,06
**GGCX**	10,48	10,54	-0,06
**VGLL4**	12,30	12,36	-0,06
**NT5DC2**	13,79	13,85	-0,06
**CDK5RAP2**	12,66	12,71	-0,06
**MRPL9**	12,79	12,85	-0,06
**FAM219B**	11,11	11,17	-0,06
**LEO1**	12,33	12,39	-0,06
**CUL9**	11,04	11,10	-0,06
**CCNE1**	10,70	10,76	-0,06
**SLC15A4**	12,28	12,34	-0,06
**GTF2H3**	11,98	12,04	-0,06
**CCAR1**	14,03	14,08	-0,06
**ITGB3BP**	11,18	11,24	-0,06
**TMEM170A**	12,34	12,40	-0,06
**GABPB2**	11,45	11,51	-0,06
**FOXRED1**	11,09	11,15	-0,06
**LILRB4**	11,00	11,05	-0,06
**UBXN4**	13,84	13,89	-0,06
**LACC1**	9,42	9,48	-0,06
**GALNT14**	12,35	12,40	-0,06
**ZNF429**	10,54	10,60	-0,06
**GNB5**	10,91	10,97	-0,06
**PPP2R2C**	5,25	5,31	-0,06
**GPI**	14,76	14,81	-0,06
**AURKB**	13,10	13,15	-0,06
**IGSF3**	11,59	11,65	-0,06
**GSTM1**	12,61	12,66	-0,06
**RALGAPA1**	11,21	11,27	-0,06
**PRC1**	12,75	12,81	-0,06
**PCYT2**	11,54	11,59	-0,06
**SMIM24**	9,66	9,71	-0,06
**LRRC20**	9,70	9,76	-0,06
**CKAP4**	13,06	13,11	-0,06
**PTPN23**	11,39	11,44	-0,06
**PIGG**	10,24	10,30	-0,06
**PDSS2**	10,43	10,49	-0,06
**ZNF250**	11,17	11,23	-0,06
**MIEF1**	12,91	12,96	-0,06
**ZBTB8A**	9,91	9,96	-0,06
**YIF1B**	11,24	11,29	-0,06
**SC5D**	11,14	11,19	-0,06
**FKBP1A**	14,34	14,39	-0,06
**LOXL3**	6,51	6,56	-0,06
**PPIL4**	11,53	11,59	-0,06
**DMD**	9,81	9,87	-0,06

**UQCRB**	13,92	13,98	-0,06
**EIF3B**	14,98	15,03	-0,06
**SLFN11**	13,39	13,45	-0,06
**TMEM256**	10,53	10,59	-0,06
**NAP1L4**	13,68	13,73	-0,05
**RSRC1**	11,88	11,94	-0,05
**MLH1**	12,04	12,09	-0,05
**BRD7**	13,64	13,70	-0,05
**USP11**	12,38	12,44	-0,05
**CCDC182**	5,71	5,76	-0,05
**TMLHE**	10,25	10,30	-0,05
**AADAT**	11,45	11,51	-0,05
**STAT3**	11,63	11,69	-0,05
**TIMM23**	12,66	12,71	-0,05
**MRPL46**	10,74	10,80	-0,05
**NICN1**	8,39	8,44	-0,05
**RELA**	12,48	12,54	-0,05
**BICD1**	11,15	11,20	-0,05
**ENO1**	17,03	17,08	-0,05
**HEBP1**	9,80	9,85	-0,05
**COMMD9**	10,97	11,02	-0,05
**AC068533.4**	4,29	4,34	-0,05
**SIN3B**	12,15	12,20	-0,05
**RPP38**	10,58	10,63	-0,05
**TRAP1**	13,95	14,01	-0,05
**ATP1A1**	14,47	14,52	-0,05
**SMNDC1**	12,15	12,20	-0,05
**PIP4K2B**	13,16	13,21	-0,05
**BCL2L11**	12,35	12,40	-0,05
**EFNA4**	9,63	9,68	-0,05
**CZIB**	11,21	11,26	-0,05
**RSU1**	12,63	12,68	-0,05
**NDUFB6**	11,64	11,69	-0,05
**SHPK**	11,11	11,17	-0,05
**TMEM251**	9,76	9,81	-0,05
**PRICKLE3**	9,52	9,57	-0,05
**RHNO1**	11,57	11,62	-0,05
**UBE2D1**	11,40	11,46	-0,05
**CPT1B**	10,29	10,35	-0,05
**SFXN2**	11,99	12,04	-0,05
**PHF14**	13,45	13,50	-0,05
**DNMT1**	14,85	14,90	-0,05
**MRM2**	11,45	11,51	-0,05
**FBXO7**	12,89	12,95	-0,05
**IFIT1**	8,24	8,29	-0,05
**MRPS2**	12,29	12,34	-0,05
**WBP2**	11,30	11,35	-0,05
**SCAMP3**	12,39	12,45	-0,05
**IGBP1**	11,59	11,65	-0,05
**CISD3**	11,05	11,10	-0,05
**ANKRD42**	8,42	8,47	-0,05
**ANXA2R**	8,87	8,92	-0,05
**TPGS2**	13,17	13,23	-0,05
**DIDO1**	13,26	13,32	-0,05
**RNF25**	10,10	10,15	-0,05
**BTBD2**	12,75	12,81	-0,05

**TRIM62**	9,65	9,71	-0,05
**SALL4**	10,19	10,24	-0,05
**TMEM168**	11,27	11,32	-0,05
**ZDHHC3**	12,01	12,07	-0,05
**FBXL18**	10,88	10,94	-0,05
**CCZ1B**	12,24	12,29	-0,05
**RPL7**	17,50	17,56	-0,05
**ELOB**	12,55	12,60	-0,05
**FAH**	9,99	10,04	-0,05
**HTRA3**	13,21	13,27	-0,05
**N4BP3**	9,92	9,98	-0,05
**UBOX5**	9,74	9,79	-0,05
**VPS51**	12,27	12,32	-0,05
**MUC20**	9,06	9,12	-0,05
**SPATA5**	10,48	10,53	-0,05
**RRAGD**	11,25	11,30	-0,05
**REPIN1**	12,82	12,87	-0,05
**HSPH1**	14,97	15,02	-0,05
**GMDS**	11,09	11,15	-0,05
**BAZ1B**	14,72	14,77	-0,05
**CD79B**	12,66	12,71	-0,05
**CENPW**	11,54	11,60	-0,05
**TLR4**	8,37	8,42	-0,05
**C1orf52**	11,06	11,11	-0,05
**CCT7**	15,48	15,53	-0,05
**CTSD**	11,54	11,59	-0,05
**SLC22A16**	10,22	10,27	-0,05
**GRB2**	13,78	13,83	-0,05
**AP5B1**	12,00	12,06	-0,05
**COX18**	10,83	10,88	-0,05
**TMED2**	14,10	14,15	-0,05
**WDR19**	10,70	10,75	-0,05
**ZBTB37**	11,31	11,36	-0,05
**SLC36A4**	11,10	11,16	-0,05
**COA3**	11,15	11,20	-0,05
**AP2S1**	12,18	12,23	-0,05
**HINT3**	10,75	10,80	-0,05
**RBM33**	12,94	12,99	-0,05
**CCNK**	12,82	12,88	-0,05
**JARID2**	12,96	13,01	-0,05
**BRD4**	13,54	13,59	-0,05
**CTRL**	7,46	7,51	-0,05
**COX6C**	13,47	13,52	-0,05
**PPT2-EGFL8**	8,05	8,10	-0,05
**PRPF3**	13,13	13,18	-0,05
**CTNNBL1**	12,50	12,55	-0,05
**C3orf33**	7,39	7,44	-0,05
**KDM2A**	13,67	13,72	-0,05
**PSMC1**	13,88	13,94	-0,05
**ALDH7A1**	11,83	11,88	-0,05
**GTF2IRD1**	11,27	11,32	-0,05
**NUDT5**	13,13	13,18	-0,05
**FAM117A**	11,47	11,52	-0,05
**GHDC**	9,91	9,96	-0,05
**LGALS9**	12,53	12,58	-0,05
**CSF2RB**	11,83	11,88	-0,05

**CEBPG**	13,13	13,18	-0,05
**NOS1**	9,09	9,14	-0,05
**TRPV2**	8,86	8,91	-0,05
**URM1**	12,38	12,43	-0,05
**NPIPA1**	11,95	12,00	-0,05
**AMMECR1L**	11,91	11,96	-0,05
**TYK2**	12,39	12,44	-0,05
**PSMG1**	12,43	12,48	-0,05
**DRAXIN**	13,52	13,57	-0,05
**VANGL1**	10,97	11,02	-0,05
**KIF12**	7,80	7,85	-0,05
**TRIM36**	5,95	6,00	-0,05
**SRP68**	13,04	13,09	-0,05
**NOSTRIN**	6,31	6,36	-0,05
**UBR5**	14,72	14,77	-0,05
**POU4F1**	6,51	6,56	-0,05
**NUFIP2**	14,16	14,21	-0,05
**LCMT2**	10,66	10,70	-0,05
**CFAP53**	5,74	5,79	-0,05
**AC091167.2**	5,74	5,79	-0,05
**NUDT7**	7,26	7,31	-0,05
**CLK2**	12,18	12,23	-0,05
**YIF1A**	11,04	11,09	-0,05
**ZNF14**	10,04	10,09	-0,05
**MBOAT7**	11,75	11,80	-0,05
**ACER2**	8,12	8,17	-0,05
**SAE1**	14,32	14,37	-0,05
**KIF21B**	13,06	13,11	-0,05
**PWWP3A**	11,75	11,79	-0,05
**MASP2**	7,47	7,52	-0,05
**MRPS17**	11,17	11,21	-0,05
**STAT5B**	12,94	12,99	-0,05
**LUZP1**	12,30	12,35	-0,05
**MAX**	12,97	13,01	-0,05
**CMTM7**	11,67	11,72	-0,05
**TRIM28**	15,73	15,78	-0,05
**NOL9**	12,28	12,33	-0,05
**CWF19L2**	3,92	3,97	-0,05
**PGAM4**	3,92	3,97	-0,05
**TMEM86A**	6,66	6,71	-0,05
**GAPDH**	17,68	17,73	-0,05
**FLT1**	11,91	11,95	-0,05
**P4HB**	14,49	14,54	-0,05
**ZNF18**	9,87	9,92	-0,05
**EIF2B1**	12,68	12,73	-0,05
**KLHL30**	6,97	7,02	-0,05
**HIKESHI**	10,84	10,89	-0,05
**KAZALD1**	11,00	11,05	-0,05
**TERF2**	12,40	12,45	-0,05
**SLC2A3**	11,07	11,12	-0,05
**KIF22**	13,34	13,38	-0,05
**EMC8**	11,80	11,85	-0,05
**COX5A**	13,44	13,49	-0,05
**GFER**	10,33	10,38	-0,05
**PSMD13**	13,42	13,47	-0,05
**LTBR**	5,98	6,03	-0,05

**ZNF558**	9,97	10,02	-0,05
**ID3**	13,72	13,77	-0,05
**GMPPA**	10,07	10,12	-0,05
**CSRNP1**	10,25	10,30	-0,05
**RWDD2A**	7,69	7,74	-0,05
**RPS6KB1**	12,25	12,30	-0,05
**ZNF664**	14,01	14,06	-0,05
**PATZ1**	12,71	12,76	-0,05
**RIOK2**	11,93	11,98	-0,05
**ATXN1**	7,27	7,32	-0,05
**AGAP9**	10,09	10,14	-0,05
**PGS1**	10,69	10,73	-0,05
**CHCHD2**	14,80	14,85	-0,05
**AP1B1**	12,87	12,92	-0,05
**NADSYN1**	11,67	11,72	-0,05
**ERLIN1**	12,30	12,35	-0,05
**SMARCD2**	13,91	13,96	-0,05
**FGF11**	7,12	7,16	-0,05
**SEC61A2**	9,58	9,63	-0,05
**ADIPOR1**	12,34	12,38	-0,05
**HDAC8**	10,64	10,69	-0,05
**USP7**	14,60	14,65	-0,05
**MTMR14**	12,09	12,14	-0,05
**TMEM186**	10,23	10,27	-0,05
**MED10**	11,54	11,58	-0,05
**ZFP36L1**	13,81	13,85	-0,05
**FN1**	7,95	7,99	-0,05
**H3C6**	5,61	5,66	-0,05
**NBAS**	11,83	11,87	-0,05
**CREBZF**	13,55	13,60	-0,05
**SNRPD1**	13,74	13,79	-0,05
**MTOR**	13,50	13,55	-0,05
**PRMT5**	13,78	13,83	-0,05
**MRPL11**	12,15	12,20	-0,05
**ZPR1**	12,35	12,40	-0,05
**CTSK**	6,70	6,74	-0,05
**AP5S1**	9,63	9,68	-0,05
**MTA1**	13,65	13,70	-0,05
**PPFIBP2**	9,14	9,18	-0,05
**ARL8A**	11,19	11,24	-0,05
**NEO1**	11,43	11,48	-0,05
**EIF2S2**	13,85	13,90	-0,05
**USB1**	11,84	11,89	-0,05
**CPSF1**	13,98	14,02	-0,05
**TMEM219**	10,79	10,84	-0,05
**HYLS1**	10,73	10,78	-0,05
**CD84**	10,83	10,88	-0,05
**IQSEC1**	12,82	12,86	-0,05
**ANXA4**	11,43	11,47	-0,05
**POLR2J3**	10,71	10,75	-0,05
**TRIM32**	11,10	11,15	-0,05
**DDOST**	13,64	13,69	-0,05
**AKT1**	12,46	12,50	-0,05
**HDHD2**	11,24	11,29	-0,05
**AEBP1**	14,03	14,07	-0,05
**TUBA1B**	17,89	17,94	-0,05

**NOL6**	13,24	13,29	-0,05
**ST3GAL3**	10,90	10,95	-0,05
**MYC**	15,35	15,39	-0,05
**GALE**	10,28	10,32	-0,05
**ASF1B**	12,87	12,92	-0,05
**JADE1**	13,10	13,14	-0,05
**PDCL3**	11,57	11,62	-0,05
**ZMYM3**	12,64	12,68	-0,05
**ARID1A**	14,39	14,44	-0,05
**CARF**	9,74	9,79	-0,05
**PEBP1**	14,67	14,71	-0,05
**ACAD9**	12,34	12,38	-0,05
**ZNF689**	11,13	11,17	-0,05
**OFD1**	13,09	13,14	-0,05
**DKC1**	14,51	14,55	-0,05
**EDC3**	11,97	12,01	-0,05
**CHORDC1**	13,42	13,47	-0,05
**GMEB2**	11,64	11,69	-0,05
**CCND3**	15,67	15,71	-0,04
**THNSL1**	10,51	10,56	-0,04
**FGFR1**	12,41	12,45	-0,04
**EPB41L4A**	10,27	10,32	-0,04
**TPM1**	9,55	9,59	-0,04
**CELSR2**	12,15	12,20	-0,04
**TFEB**	11,31	11,36	-0,04
**CPD**	11,87	11,92	-0,04
**ZNF691**	9,73	9,77	-0,04
**TUT7**	11,11	11,15	-0,04
**RAB43**	9,98	10,03	-0,04
**TXNDC15**	11,32	11,36	-0,04
**SH2B3**	11,60	11,65	-0,04
**ZNF77**	9,70	9,74	-0,04
**HGFAC**	5,20	5,24	-0,04
**ZC3H12D**	11,07	11,11	-0,04
**PABPC4**	14,55	14,60	-0,04
**SLC29A4**	9,54	9,58	-0,04
**RHBDF2**	10,31	10,35	-0,04
**MYD88**	13,01	13,06	-0,04
**GLRX**	12,49	12,54	-0,04
**SMYD5**	10,77	10,81	-0,04
**EXOC3**	11,49	11,54	-0,04
**TMEM50B**	12,28	12,32	-0,04
**BTN3A2**	11,73	11,78	-0,04
**POLR1A**	13,68	13,72	-0,04
**RAB11A**	13,11	13,15	-0,04
**ZSCAN16**	9,13	9,18	-0,04
**ATP6V0D1**	12,17	12,21	-0,04
**YLPM1**	13,70	13,75	-0,04
**ERP27**	7,96	8,01	-0,04
**SIK2**	11,99	12,03	-0,04
**MRPS18C**	11,95	12,00	-0,04
**THAP8**	7,91	7,96	-0,04
**FTL**	15,02	15,07	-0,04
**ATAT1**	10,98	11,02	-0,04
**ZNF287**	9,80	9,85	-0,04
**PDCD6**	12,56	12,60	-0,04

**DERPC**	10,46	10,51	-0,04
**DHRS7**	10,54	10,58	-0,04
**UBA1**	14,94	14,99	-0,04
**SNU13**	13,56	13,60	-0,04
**TBP**	11,43	11,47	-0,04
**RPS15A**	15,73	15,77	-0,04
**C4orf3**	12,37	12,41	-0,04
**SP140L**	10,95	10,99	-0,04
**CACHD1**	11,47	11,51	-0,04
**CCDC77**	10,92	10,96	-0,04
**CCDC69**	13,18	13,22	-0,04
**TIGD1**	10,37	10,42	-0,04
**TMEM209**	11,82	11,87	-0,04
**SMC1B**	5,43	5,47	-0,04
**TRAPPC1**	11,91	11,95	-0,04
**CPSF2**	13,52	13,56	-0,04
**ACTR1A**	12,88	12,92	-0,04
**SURF4**	13,32	13,36	-0,04
**PARL**	11,65	11,69	-0,04
**TMEM60**	9,80	9,84	-0,04
**PHLPP2**	11,16	11,20	-0,04
**LAIR1**	11,97	12,01	-0,04
**MREG**	10,40	10,44	-0,04
**UQCRC2**	14,17	14,21	-0,04
**USP4**	12,87	12,91	-0,04
**SEMA3F**	9,68	9,73	-0,04
**MAP2K5**	10,42	10,47	-0,04
**DDX5**	16,34	16,39	-0,04
**SDE2**	12,24	12,28	-0,04
**ATXN1L**	12,13	12,17	-0,04
**VAC14**	11,62	11,67	-0,04
**MRNIP**	11,14	11,18	-0,04
**GLUL**	14,76	14,80	-0,04
**TM7SF3**	12,93	12,97	-0,04
**ASH1L**	12,82	12,86	-0,04
**INPP5D**	12,99	13,03	-0,04
**SMOX**	9,20	9,25	-0,04
**F12**	8,05	8,10	-0,04
**ALDH2**	6,92	6,96	-0,04
**AL031708.1**	7,82	7,87	-0,04
**PAX5**	15,44	15,48	-0,04
**FAM172A**	10,79	10,83	-0,04
**LANCL2**	10,89	10,93	-0,04
**ZFP30**	11,14	11,19	-0,04
**TBPL1**	10,96	11,00	-0,04
**GUSB**	12,12	12,16	-0,04
**EFL1**	11,81	11,85	-0,04
**LGR5**	7,37	7,41	-0,04
**YTHDF3**	13,70	13,74	-0,04
**DHRSX**	10,46	10,50	-0,04
**WDR7**	10,88	10,92	-0,04
**QARS1**	13,61	13,65	-0,04
**PPM1H**	8,64	8,68	-0,04
**CRMP1**	13,81	13,85	-0,04
**GPAT3**	6,09	6,13	-0,04
**FLNC**	7,42	7,46	-0,04

**ERLEC1**	11,13	11,17	-0,04
**AMOTL1**	13,15	13,19	-0,04
**TBRG4**	12,58	12,62	-0,04
**MAPK9**	12,08	12,12	-0,04
**OTUD1**	11,47	11,51	-0,04
**TSFM**	11,83	11,87	-0,04
**DIS3L**	12,80	12,84	-0,04
**LLGL1**	11,89	11,93	-0,04
**TCF20**	12,70	12,74	-0,04
**TSC22D1**	11,97	12,01	-0,04
**RADIL**	10,68	10,72	-0,04
**GALNT11**	8,51	8,55	-0,04
**QTRT2**	12,57	12,61	-0,04
**RAB1A**	12,89	12,93	-0,04
**LTBP2**	10,73	10,77	-0,04
**UCKL1**	11,49	11,53	-0,04
**CNOT1**	15,17	15,21	-0,04
**DRG2**	11,46	11,50	-0,04
**LRRTM2**	4,37	4,41	-0,04
**H1-4**	4,37	4,41	-0,04
**AC010615.4**	4,37	4,41	-0,04
**DERL3**	10,00	10,04	-0,04
**IAH1**	11,21	11,25	-0,04
**MLEC**	14,71	14,75	-0,04
**LRRC70**	6,41	6,45	-0,04
**ALKBH5**	12,98	13,02	-0,04
**FAM168A**	12,55	12,59	-0,04
**ACLY**	14,99	15,03	-0,04
**ABCB8**	11,02	11,06	-0,04
**TMEM74**	8,07	8,11	-0,04
**AAR2**	11,74	11,78	-0,04
**TUBA1C**	14,49	14,53	-0,04
**ZBTB39**	12,13	12,17	-0,04
**AL445524.2**	6,23	6,27	-0,04
**PKN3**	11,23	11,27	-0,04
**TOX**	8,72	8,76	-0,04
**TOMM6**	13,23	13,27	-0,04
**PHB**	14,26	14,30	-0,04
**PCNT**	12,58	12,62	-0,04
**SDHAF3**	9,60	9,64	-0,04
**MT-ND5**	17,15	17,19	-0,04
**LMNB1**	15,56	15,60	-0,04
**OTUD3**	12,00	12,04	-0,04
**DDX42**	14,09	14,13	-0,04
**ILF2**	15,60	15,64	-0,04
**TMED4**	12,47	12,51	-0,04
**DAZAP2**	13,43	13,47	-0,04
**MEIS2**	2,14	2,18	-0,04
**MATN2**	4,40	4,44	-0,04
**PITHD1**	12,42	12,46	-0,04
**ZNF749**	10,31	10,35	-0,04
**MAPKAPK3**	11,86	11,90	-0,04
**MFSD3**	9,57	9,61	-0,04
**PSME3IP1**	12,99	13,03	-0,04
**COPS6**	12,91	12,95	-0,04
**SNX13**	11,50	11,54	-0,04

**FBXO48**	8,71	8,75	-0,04
**PCYOX1**	11,26	11,30	-0,04
**SLC4A1AP**	11,63	11,67	-0,04
**ADGRE5**	10,74	10,77	-0,04
**CNTROB**	11,78	11,82	-0,04
**ADH5**	12,40	12,44	-0,04
**ANKH**	12,65	12,69	-0,04
**PRMT3**	12,22	12,26	-0,04
**IP6K1**	12,12	12,16	-0,04
**PTPN1**	12,46	12,50	-0,04
**ANKRD27**	12,46	12,50	-0,04
**SNTA1**	11,64	11,67	-0,04
**STUB1**	11,42	11,45	-0,04
**PCCB**	12,38	12,42	-0,04
**STN1**	9,96	10,00	-0,04
**GAA**	10,86	10,90	-0,04
**DNAJB14**	12,26	12,29	-0,04
**EPG5**	11,74	11,77	-0,04
**CAP1**	14,33	14,37	-0,04
**CACYBP**	14,56	14,60	-0,04
**CENPP**	11,22	11,26	-0,04
**DIAPH1**	15,00	15,04	-0,04
**MED14**	11,97	12,01	-0,04
**PRDX2**	13,74	13,78	-0,04
**PPEF2**	3,78	3,82	-0,04
**FASN**	15,22	15,25	-0,04
**DENND1C**	9,52	9,56	-0,04
**KIAA2026**	10,99	11,03	-0,04
**RRP36**	11,40	11,44	-0,04
**CDC42SE1**	13,09	13,13	-0,04
**KIF15**	13,20	13,24	-0,04
**ZDBF2**	9,93	9,97	-0,04
**PPP1R3E**	10,36	10,40	-0,04
**GDAP1**	10,10	10,14	-0,04
**LITAF**	9,48	9,51	-0,04
**ZNF324B**	9,28	9,32	-0,04
**ANKDD1A**	9,07	9,10	-0,04
**RPL36A**	15,15	15,19	-0,04
**IDH1**	12,18	12,21	-0,04
**NIF3L1**	10,31	10,34	-0,04
**WIPF2**	11,66	11,70	-0,04
**MTRF1**	10,10	10,14	-0,04
**MACIR**	11,52	11,56	-0,04
**LAIR2**	4,26	4,30	-0,04
**YARS1**	14,36	14,40	-0,04
**HGH1**	11,96	12,00	-0,04
**STEAP3**	10,13	10,16	-0,04
**RPL14**	16,44	16,47	-0,04
**OMA1**	10,49	10,53	-0,04
**C6orf89**	11,73	11,76	-0,04
**YARS2**	11,35	11,38	-0,04
**NCKIPSD**	10,63	10,66	-0,04
**CEP57L1**	11,25	11,28	-0,04
**ADGRE1**	4,41	4,44	-0,04
**ECI1**	11,10	11,13	-0,04
**C2orf68**	11,80	11,83	-0,04

**JKAMP**	10,85	10,89	-0,04
**SMPD2**	9,50	9,53	-0,04
**ELAVL4**	5,62	5,65	-0,04
**SOD1**	14,23	14,27	-0,04
**ZNF280C**	10,65	10,69	-0,04
**GINS4**	13,30	13,34	-0,04
**MEX3B**	12,02	12,05	-0,04
**CDC42EP5**	7,15	7,18	-0,04
**TMBIM6**	14,48	14,51	-0,04
**TRUB2**	12,33	12,36	-0,04
**ACTR2**	15,80	15,84	-0,04
**NKD1**	6,86	6,90	-0,04
**ZDHHC11**	8,86	8,90	-0,04
**LRTM2**	9,73	9,76	-0,04
**NSUN7**	5,28	5,31	-0,04
**SAMM50**	12,22	12,25	-0,04
**WDR41**	11,99	12,03	-0,04
**GNB1L**	9,45	9,49	-0,04
**MARK2**	11,91	11,95	-0,04
**RUSC1**	11,03	11,06	-0,04
**TJAP1**	11,52	11,55	-0,04
**PSAP**	13,51	13,54	-0,04
**HOMEZ**	10,18	10,21	-0,04
**ZNF565**	8,28	8,31	-0,04
**AP003108.2**	9,17	9,20	-0,04
**EOLA2**	10,30	10,34	-0,04
**CYB5R4**	10,33	10,36	-0,04
**PRPSAP2**	11,64	11,67	-0,04
**LARP1B**	11,40	11,43	-0,04
**NFIC**	12,62	12,66	-0,03
**IK**	13,40	13,43	-0,03
**STARD7**	14,37	14,40	-0,03
**PTP4A3**	12,16	12,19	-0,03
**CLCN7**	12,65	12,69	-0,03
**PSPH**	11,13	11,16	-0,03
**ZNF800**	11,69	11,73	-0,03
**CDC27**	13,25	13,29	-0,03
**VCP**	14,18	14,21	-0,03
**LTBP3**	10,84	10,87	-0,03
**FAM193A**	12,03	12,07	-0,03
**APP**	14,52	14,55	-0,03
**INTS8**	13,64	13,68	-0,03
**TOB2**	12,07	12,11	-0,03
**NEURL1B**	14,32	14,35	-0,03
**RBM5**	13,52	13,55	-0,03
**SYNE2**	13,35	13,38	-0,03
**STK32B**	10,98	11,02	-0,03
**ANTXR2**	8,96	8,99	-0,03
**DAD1**	12,17	12,20	-0,03
**HLA-DRA**	14,94	14,97	-0,03
**AP000812.4**	6,69	6,73	-0,03
**PLEKHA5**	11,19	11,22	-0,03
**BBS7**	10,57	10,61	-0,03
**CTSH**	11,56	11,59	-0,03
**MAVS**	13,41	13,44	-0,03
**NCBP3**	13,09	13,12	-0,03

**KLF16**	11,74	11,77	-0,03
**LRRC4**	12,74	12,78	-0,03
**COA7**	12,84	12,87	-0,03
**ATXN7L3B**	13,75	13,78	-0,03
**ARV1**	10,43	10,47	-0,03
**PTPRS**	9,82	9,86	-0,03
**PFAS**	13,56	13,60	-0,03
**ZBTB20**	9,70	9,73	-0,03
**ABHD14B**	11,92	11,95	-0,03
**EEF1AKMT4**	9,56	9,60	-0,03
**PINLYP**	7,09	7,12	-0,03
**SLC36A1**	10,27	10,31	-0,03
**SNRPN**	12,79	12,83	-0,03
**KLHL11**	11,17	11,20	-0,03
**TPM3**	15,64	15,67	-0,03
**AC138969.1**	12,88	12,92	-0,03
**UBE2Q1**	12,82	12,85	-0,03
**RPL18**	15,89	15,92	-0,03
**SLC25A4**	10,96	10,99	-0,03
**CKAP5**	14,77	14,80	-0,03
**CSF1R**	11,01	11,05	-0,03
**SNX16**	10,87	10,90	-0,03
**SIGLEC9**	6,98	7,01	-0,03
**RPL10**	16,92	16,95	-0,03
**EIF2B2**	10,98	11,01	-0,03
**SEC61A1**	14,02	14,06	-0,03
**ZGRF1**	12,02	12,05	-0,03
**DARS1**	13,74	13,77	-0,03
**ZNF587B**	11,75	11,79	-0,03
**ARMCX6**	10,49	10,52	-0,03
**FANCG**	11,77	11,80	-0,03
**PIGW**	11,54	11,57	-0,03
**HHEX**	10,57	10,61	-0,03
**LIAS**	10,48	10,52	-0,03
**DDX56**	12,99	13,02	-0,03
**MTX1**	11,40	11,43	-0,03
**RABIF**	10,67	10,70	-0,03
**CWC15**	12,20	12,23	-0,03
**KCNIP2**	5,32	5,35	-0,03
**ECSIT**	11,19	11,22	-0,03
**TESPA1**	8,83	8,86	-0,03
**KIAA1549L**	6,15	6,18	-0,03
**GID8**	12,99	13,02	-0,03
**OSBPL3**	11,49	11,52	-0,03
**RNF32**	6,86	6,89	-0,03
**RBBP7**	13,95	13,98	-0,03
**SNAPC1**	10,55	10,58	-0,03
**SHMT1**	11,97	12,00	-0,03
**AGBL5**	11,55	11,58	-0,03
**THOP1**	13,09	13,12	-0,03
**PHAX**	12,04	12,07	-0,03
**SFXN5**	9,22	9,25	-0,03
**A2M**	4,98	5,01	-0,03
**RTL3**	4,98	5,01	-0,03
**SBDS**	11,13	11,16	-0,03
**PPP4R2**	13,34	13,37	-0,03

**LTBP4**	11,51	11,54	-0,03
**PSME1**	14,25	14,28	-0,03
**ZNF335**	11,30	11,34	-0,03
**CRYBG3**	10,37	10,40	-0,03
**THAP4**	12,02	12,05	-0,03
**MARCKS**	12,85	12,88	-0,03
**PSMB4**	14,13	14,16	-0,03
**PARP11**	10,60	10,63	-0,03
**ZDHHC16**	11,17	11,20	-0,03
**FBXO42**	11,33	11,36	-0,03
**BTN2A1**	11,29	11,33	-0,03
**RTL5**	5,86	5,89	-0,03
**RAB9A**	10,82	10,85	-0,03
**PDCD2**	12,95	12,99	-0,03
**IQCC**	9,21	9,24	-0,03
**EDA**	6,87	6,90	-0,03
**TTC39C**	11,61	11,64	-0,03
**ZNF416**	9,78	9,81	-0,03
**KDM4A**	12,32	12,35	-0,03
**ELAC2**	13,35	13,38	-0,03
**IGHM**	17,10	17,13	-0,03
**ZC3H11B**	4,02	4,05	-0,03
**PJA1**	10,42	10,45	-0,03
**ATP5MD**	12,41	12,44	-0,03
**ITPA**	11,79	11,82	-0,03
**TMBIM1**	9,79	9,82	-0,03
**UBE2C**	12,57	12,60	-0,03
**THRA**	11,42	11,45	-0,03
**NGDN**	11,88	11,91	-0,03
**INTS3**	13,17	13,20	-0,03
**POM121**	11,83	11,86	-0,03
**TAF9**	11,69	11,72	-0,03
**NAA40**	12,13	12,16	-0,03
**MANBA**	10,54	10,57	-0,03
**XPO7**	13,89	13,92	-0,03
**MFHAS1**	13,34	13,37	-0,03
**TMTC4**	12,09	12,12	-0,03
**FLCN**	10,26	10,29	-0,03
**RPRD1A**	13,06	13,09	-0,03
**INTS5**	11,75	11,78	-0,03
**RAC2**	12,26	12,29	-0,03
**PNO1**	11,92	11,95	-0,03
**EIF4EBP1**	13,32	13,35	-0,03
**NFATC2IP**	11,04	11,07	-0,03
**TOMM5**	12,74	12,77	-0,03
**COA4**	11,94	11,97	-0,03
**PPIA**	16,77	16,80	-0,03
**ATP13A1**	12,38	12,41	-0,03
**RPL6**	16,79	16,82	-0,03
**NXF1**	13,37	13,40	-0,03
**SCN3B**	5,23	5,26	-0,03
**CAMTA2**	11,29	11,32	-0,03
**LRP8**	13,21	13,24	-0,03
**FDX1**	11,67	11,70	-0,03
**CDKAL1**	11,32	11,35	-0,03
**ATL2**	12,63	12,66	-0,03

**FARP2**	11,74	11,77	-0,03
**CLPP**	12,12	12,15	-0,03
**ZNF518B**	11,22	11,25	-0,03
**ATF7IP**	10,48	10,51	-0,03
**CTBP1**	14,14	14,17	-0,03
**TSTD2**	11,59	11,62	-0,03
**ANKRD9**	10,21	10,24	-0,03
**RPUSD3**	11,44	11,47	-0,03
**ZRANB2**	14,35	14,38	-0,03
**MXD1**	7,95	7,98	-0,03
**NOCT**	9,66	9,69	-0,03
**MTERF2**	10,13	10,16	-0,03
**PGAP2**	10,97	11,00	-0,03
**HNRNPH1**	16,12	16,15	-0,03
**MTG1**	11,76	11,79	-0,03
**TSPAN3**	13,44	13,47	-0,03
**MCUB**	11,40	11,43	-0,03
**PSMC4**	12,96	12,99	-0,03
**AFG1L**	8,77	8,80	-0,03
**ZKSCAN5**	10,95	10,98	-0,03
**AHSA1**	13,87	13,90	-0,03
**SLC12A7**	12,26	12,29	-0,03
**PPP1R11**	11,64	11,66	-0,03
**TSPAN14**	13,77	13,80	-0,03
**TEFM**	10,10	10,13	-0,03
**FAIM**	11,89	11,91	-0,03
**SCARA3**	9,64	9,67	-0,03
**TM7SF2**	8,77	8,80	-0,03
**ZNF195**	12,31	12,33	-0,03
**KAZN**	10,83	10,85	-0,03
**PFKFB3**	11,91	11,93	-0,03
**HMGN2**	16,51	16,54	-0,03
**CEP68**	12,07	12,10	-0,03
**CNOT6L**	10,82	10,85	-0,03
**GSS**	12,09	12,12	-0,03
**STIL**	11,39	11,41	-0,03
**CDKN1C**	6,44	6,47	-0,03
**DNAJC8**	13,82	13,85	-0,03
**ASB7**	10,44	10,46	-0,03
**TANGO6**	10,99	11,01	-0,03
**STRADA**	11,64	11,67	-0,03
**RAD9A**	10,64	10,66	-0,03
**MARCHF9**	9,19	9,22	-0,03
**PRKACA**	11,97	11,99	-0,03
**CNNM3**	11,22	11,25	-0,03
**WIPI2**	12,85	12,88	-0,03
**DOCK8**	10,01	10,04	-0,03
**IL17RC**	8,72	8,75	-0,03
**RTN4**	14,08	14,11	-0,03
**UBE2H**	11,57	11,60	-0,03
**HSP90AB1**	17,93	17,95	-0,03
**ZNRF1**	12,03	12,06	-0,03
**L3MBTL2**	12,18	12,21	-0,03
**CCDC188**	6,10	6,13	-0,03
**HMGCS1**	13,03	13,05	-0,03
**TUBA1A**	13,73	13,76	-0,03

**ACTN4**	13,86	13,89	-0,03
**ACP1**	13,55	13,58	-0,03
**BRD3**	12,84	12,87	-0,03
**ANKS1A**	11,51	11,53	-0,03
**ST7**	10,23	10,26	-0,03
**MPV17**	11,63	11,65	-0,03
**FAM156B**	9,33	9,36	-0,03
**ZNF446**	9,15	9,18	-0,03
**MRPL1**	11,77	11,79	-0,03
**COPS7A**	10,83	10,86	-0,03
**ZNF232**	11,59	11,61	-0,03
**C1RL**	9,09	9,12	-0,03
**PLSCR1**	10,35	10,38	-0,03
**SHCBP1**	12,78	12,80	-0,03
**SYNM**	8,74	8,76	-0,03
**MRPS6**	12,02	12,04	-0,03
**WDR3**	13,33	13,35	-0,03
**MAK**	5,43	5,46	-0,03
**PIGBOS1**	9,62	9,64	-0,03
**EAPP**	11,26	11,28	-0,03
**MEGF10**	5,58	5,61	-0,03
**TRIM27**	13,21	13,24	-0,03
**CHMP5**	11,35	11,38	-0,03
**NDUFAB1**	12,76	12,78	-0,03
**SLC45A1**	6,21	6,24	-0,03
**ALDH1A2**	2,15	2,17	-0,03
**AP3B2**	7,81	7,84	-0,03
**COBL**	9,66	9,69	-0,03
**TRIM71**	8,12	8,14	-0,03
**ZNF140**	11,14	11,17	-0,03
**VSIG10**	12,33	12,35	-0,03
**SKP2**	13,66	13,69	-0,03
**CTNS**	10,79	10,82	-0,03
**FNBP4**	13,43	13,45	-0,03
**MPST**	11,35	11,38	-0,03
**LEF1**	15,92	15,94	-0,03
**CYCS**	14,83	14,86	-0,03
**TMEM242**	9,82	9,85	-0,03
**SMIM14**	11,03	11,06	-0,03
**NOP58**	14,42	14,45	-0,03
**MTF1**	11,28	11,30	-0,03
**LRR1**	11,76	11,78	-0,03
**ECHS1**	12,42	12,45	-0,03
**TULP4**	11,41	11,43	-0,03
**GATAD1**	10,92	10,95	-0,03
**AAMDC**	7,24	7,26	-0,03
**CD37**	6,84	6,86	-0,03
**RRP1B**	13,90	13,93	-0,02
**STAU1**	13,21	13,24	-0,02
**EYA3**	12,05	12,08	-0,02
**SUMO3**	13,56	13,58	-0,02
**B4GALT5**	12,55	12,57	-0,02
**LATS2**	9,40	9,43	-0,02
**SENP2**	11,86	11,88	-0,02
**PHF13**	11,01	11,04	-0,02
**PPP6R3**	13,53	13,55	-0,02

**KAT14**	10,83	10,86	-0,02
**JPT2**	14,10	14,12	-0,02
**ZNF786**	10,30	10,32	-0,02
**NCBP2**	13,83	13,86	-0,02
**ELF4**	11,40	11,42	-0,02
**C12orf29**	11,51	11,53	-0,02
**MCRS1**	12,21	12,23	-0,02
**TIMM50**	12,98	13,00	-0,02
**LDLRAD4**	13,80	13,82	-0,02
**CKS2**	13,01	13,03	-0,02
**TIMP2**	12,75	12,78	-0,02
**SPATS2L**	11,90	11,92	-0,02
**PPIE**	11,13	11,15	-0,02
**TINF2**	11,54	11,56	-0,02
**RAB3D**	7,50	7,52	-0,02
**SPCS1**	12,13	12,16	-0,02
**LRP5**	13,09	13,12	-0,02
**TSPAN9**	10,03	10,06	-0,02
**DENND4C**	11,93	11,96	-0,02
**SPI1**	11,11	11,14	-0,02
**RELL1**	9,00	9,02	-0,02
**ZNF354B**	10,37	10,40	-0,02
**PDE6D**	11,30	11,33	-0,02
**MSANTD2**	10,41	10,43	-0,02
**SFMBT1**	10,43	10,45	-0,02
**AC018523.2**	6,50	6,52	-0,02
**LYRM1**	10,77	10,79	-0,02
**CPB2**	4,01	4,04	-0,02
**PPP2R5E**	12,71	12,73	-0,02
**RPS27A**	15,73	15,75	-0,02
**KCTD12**	12,22	12,24	-0,02
**RXRA**	11,47	11,49	-0,02
**HSPA4L**	8,93	8,95	-0,02
**TFDP1**	14,60	14,62	-0,02
**PANK2**	11,00	11,03	-0,02
**MAGEH1**	10,22	10,24	-0,02
**TIRAP**	9,29	9,31	-0,02
**MRPL19**	12,74	12,76	-0,02
**ATG13**	11,74	11,76	-0,02
**SYNE1**	11,41	11,44	-0,02
**IP6K2**	12,73	12,75	-0,02
**SMYD2**	12,43	12,45	-0,02
**PPP4R1**	12,75	12,78	-0,02
**AC013394.1**	9,61	9,63	-0,02
**SART3**	13,20	13,22	-0,02
**SNRPA1**	12,89	12,91	-0,02
**TBL1XR1**	14,92	14,95	-0,02
**RPS8**	16,55	16,57	-0,02
**NCF2**	8,33	8,35	-0,02
**MORC2**	12,45	12,47	-0,02
**ZNF160**	11,16	11,18	-0,02
**CHRAC1**	12,50	12,52	-0,02
**SNRNP200**	15,30	15,33	-0,02
**SLC39A13**	10,05	10,07	-0,02
**SASH3**	12,57	12,59	-0,02
**ARHGAP35**	13,20	13,23	-0,02

**ALAS1**	11,36	11,38	-0,02
**MCM3**	14,66	14,69	-0,02
**SLC20A1**	13,43	13,45	-0,02
**RNF185**	11,20	11,22	-0,02
**CENPO**	12,35	12,37	-0,02
**THYN1**	11,49	11,52	-0,02
**PITPNB**	13,31	13,33	-0,02
**C9orf40**	11,72	11,74	-0,02
**PRRC2C**	15,11	15,14	-0,02
**NSMCE2**	11,23	11,25	-0,02
**OXSM**	9,92	9,94	-0,02
**MFGE8**	10,36	10,38	-0,02
**CNOT3**	12,44	12,46	-0,02
**ATRIP**	10,93	10,95	-0,02
**PUM1**	13,55	13,57	-0,02
**MRPS15**	12,08	12,10	-0,02
**SELENOS**	10,78	10,80	-0,02
**IPO11**	12,43	12,45	-0,02
**INCA1**	6,00	6,02	-0,02
**GALK2**	10,20	10,22	-0,02
**TOR1B**	10,02	10,05	-0,02
**ZNF527**	9,77	9,79	-0,02
**C1orf198**	10,83	10,85	-0,02
**GINS3**	12,08	12,10	-0,02
**TECTA**	6,35	6,37	-0,02
**B4GALT3**	11,55	11,57	-0,02
**GEMIN4**	12,82	12,84	-0,02
**RPL28**	15,78	15,80	-0,02
**LPAR6**	9,52	9,54	-0,02
**MAEA**	12,46	12,48	-0,02
**CASP3**	12,89	12,92	-0,02
**DYSF**	9,45	9,47	-0,02
**SELENOI**	13,34	13,37	-0,02
**REEP5**	12,09	12,11	-0,02
**DDX50**	12,51	12,53	-0,02
**TRAK2**	11,73	11,75	-0,02
**TXNRD1**	15,28	15,30	-0,02
**GTPBP4**	13,76	13,79	-0,02
**FAM221A**	9,36	9,38	-0,02
**DLG3**	11,91	11,93	-0,02
**HECTD2**	10,22	10,25	-0,02
**PSMG2**	12,33	12,35	-0,02
**FAM174A**	7,51	7,53	-0,02
**TMEM70**	12,05	12,08	-0,02
**ARFIP2**	11,51	11,53	-0,02
**C2orf16**	7,55	7,57	-0,02
**NDUFA6**	12,46	12,49	-0,02
**NEGR1**	8,35	8,37	-0,02
**RAB11FIP5**	11,05	11,07	-0,02
**EPHB3**	9,38	9,40	-0,02
**PLCE1**	9,59	9,61	-0,02
**DHCR7**	11,57	11,59	-0,02
**MMP19**	8,02	8,04	-0,02
**NUTF2**	13,67	13,69	-0,02
**GSN**	11,89	11,91	-0,02
**PPP3CC**	12,27	12,30	-0,02

**KLK14**	8,52	8,54	-0,02
**GBA**	11,33	11,35	-0,02
**FP565260.1**	12,12	12,14	-0,02
**MAGED1**	14,56	14,58	-0,02
**NPR3**	3,69	3,71	-0,02
**SERINC2**	6,68	6,70	-0,02
**USP22**	15,04	15,06	-0,02
**GABARAP**	12,94	12,96	-0,02
**ATXN7L3**	12,18	12,20	-0,02
**NSA2**	13,48	13,50	-0,02
**MT-CO3**	18,27	18,29	-0,02
**BRIP1**	9,54	9,57	-0,02
**STK38L**	11,12	11,14	-0,02
**IDH3B**	12,53	12,55	-0,02
**SPRY4**	10,26	10,28	-0,02
**TRPM4**	6,10	6,12	-0,02
**GTF2B**	11,46	11,48	-0,02
**CRTAP**	12,99	13,01	-0,02
**AR**	11,19	11,21	-0,02
**LAT**	8,14	8,16	-0,02
**TEPSIN**	10,04	10,06	-0,02
**BRCC3**	11,61	11,63	-0,02
**RAD51**	11,71	11,73	-0,02
**EIF4A1**	16,24	16,26	-0,02
**RILP**	8,96	8,98	-0,02
**GPC1**	8,49	8,51	-0,02
**PAXIP1**	12,35	12,37	-0,02
**ACACA**	13,76	13,78	-0,02
**SPRY2**	11,86	11,88	-0,02
**POM121C**	13,16	13,18	-0,02
**MEAK7**	10,11	10,13	-0,02
**CYB5A**	11,31	11,33	-0,02
**DDX10**	12,35	12,37	-0,02
**NUDT4**	13,19	13,21	-0,02
**BCLAF1**	14,90	14,92	-0,02
**ATP9A**	10,12	10,14	-0,02
**PHLPP1**	12,62	12,64	-0,02
**CTSA**	11,62	11,64	-0,02
**GAB2**	12,14	12,16	-0,02
**ZC3HC1**	11,12	11,14	-0,02
**SLC33A1**	11,46	11,48	-0,02
**NPC2**	10,73	10,75	-0,02
**SCARB1**	13,49	13,51	-0,02
**BUB3**	14,67	14,69	-0,02
**MRPS28**	11,36	11,38	-0,02
**CDV3**	14,25	14,26	-0,02
**GGA3**	11,98	12,00	-0,02
**KPNB1**	15,99	16,01	-0,02
**SF3A3**	14,10	14,12	-0,02
**TSPAN15**	7,99	8,01	-0,02
**CDR2L**	7,99	8,01	-0,02
**ZNF525**	11,46	11,48	-0,02
**PNPLA2**	11,31	11,33	-0,02
**YEATS2**	13,54	13,56	-0,02
**ACAA2**	13,03	13,05	-0,02
**APEX2**	11,54	11,56	-0,02

**GLUD1**	12,77	12,79	-0,02
**ZNF7**	10,98	11,00	-0,02
**RFC2**	12,68	12,69	-0,02
**TMEM64**	11,49	11,51	-0,02
**STARD9**	11,35	11,37	-0,02
**SFXN4**	11,76	11,77	-0,02
**MTHFD1L**	13,17	13,18	-0,02
**CEP70**	10,93	10,94	-0,02
**ANKRD16**	9,66	9,68	-0,02
**RNF208**	8,68	8,70	-0,02
**DYNLL1**	13,87	13,89	-0,02
**PHACTR3**	14,41	14,43	-0,02
**OTUD6B**	12,13	12,15	-0,02
**STX5**	10,85	10,86	-0,02
**STS**	11,18	11,19	-0,02
**FGD1**	9,44	9,45	-0,02
**SERINC3**	12,48	12,49	-0,02
**RPS7**	15,87	15,88	-0,02
**HGSNAT**	11,50	11,52	-0,02
**CTSV**	6,37	6,39	-0,02
**UPK3BL1**	10,85	10,86	-0,02
**CCDC62**	7,19	7,20	-0,02
**PSENEN**	10,76	10,77	-0,02
**RNPS1**	14,58	14,60	-0,02
**GPSM2**	10,89	10,91	-0,02
**CPNE8**	8,92	8,94	-0,02
**TBC1D4**	13,19	13,21	-0,02
**SV2A**	11,77	11,78	-0,02
**BRD2**	14,44	14,46	-0,02
**MED15**	12,33	12,35	-0,02
**MYOM1**	7,45	7,46	-0,02
**MED24**	13,00	13,02	-0,02
**PRR5L**	12,01	12,02	-0,02
**TMEM94**	13,04	13,06	-0,02
**OTUB1**	12,64	12,65	-0,02
**MDH2**	13,43	13,45	-0,02
**PRELID1**	13,35	13,36	-0,02
**MGRN1**	12,11	12,13	-0,02
**HIP1**	10,49	10,51	-0,02
**DHX57**	10,98	10,99	-0,02
**MALT1**	12,79	12,81	-0,02
**EEF1B2**	15,42	15,44	-0,02
**NFKBID**	7,77	7,79	-0,02
**UBE2F**	10,23	10,25	-0,02
**AGPAT3**	11,46	11,48	-0,02
**TBCC**	10,61	10,62	-0,02
**FAM217B**	12,13	12,15	-0,02
**TRPC4AP**	12,39	12,41	-0,02
**RSKR**	10,84	10,86	-0,02
**AKAP12**	15,54	15,56	-0,02
**MCM3AP**	12,99	13,01	-0,02
**KLHL28**	10,56	10,57	-0,02
**FADS1**	14,10	14,12	-0,02
**TFB2M**	11,74	11,76	-0,02
**RFX1**	10,38	10,39	-0,02
**PRPS2**	12,72	12,74	-0,02

PGRMC2	12,00	12,01	-0,02
NDUFS4	10,32	10,34	-0,02
UTP18	12,98	13,00	-0,02
CDR2	11,25	11,27	-0,02
RBM4	13,63	13,65	-0,02
GM2A	11,79	11,81	-0,02
ADSS1	5,71	5,72	-0,02
TP53RK	12,09	12,10	-0,02
RPAIN	11,50	11,51	-0,02
NFAM1	7,36	7,38	-0,02
MCAT	10,79	10,81	-0,02
MRPL22	11,53	11,55	-0,02
ZNF462	10,29	10,30	-0,02
ATL1	4,03	4,05	-0,02
PASK	12,06	12,07	-0,02
RBM45	10,62	10,63	-0,02
FGD4	5,48	5,49	-0,02
PSD2	5,48	5,49	-0,02
ARHGEF7	12,93	12,95	-0,02
ZNF74	10,78	10,80	-0,02
PI4KA	13,68	13,69	-0,01
COX7B	12,71	12,72	-0,01
EOMES	4,51	4,53	-0,01
PGAM2	4,51	4,53	-0,01
MAFB	4,51	4,53	-0,01
IGF2R	13,44	13,46	-0,01
GALNT10	10,94	10,95	-0,01
SFRP2	4,12	4,13	-0,01
TCTEX1D4	4,12	4,13	-0,01
NFKBIE	10,23	10,24	-0,01
TIMM21	11,80	11,81	-0,01
SAMHD1	7,40	7,41	-0,01
RCBTB2	11,73	11,74	-0,01
MLX	11,87	11,89	-0,01
EIPR1	11,99	12,00	-0,01
ZC3H15	13,61	13,62	-0,01
PSME3	14,31	14,32	-0,01
PRIMA1	3,57	3,59	-0,01
HJURP	12,78	12,80	-0,01
HIRA	12,66	12,67	-0,01
EEF1AKMT3	9,40	9,41	-0,01
VDAC3	14,23	14,25	-0,01
GTPBP2	11,29	11,31	-0,01
NDUFS2	12,77	12,78	-0,01
UBE2G2	13,72	13,73	-0,01
ARHGEF2	13,76	13,77	-0,01
ADD1	13,24	13,25	-0,01
NAXE	12,49	12,51	-0,01
THAP5	10,87	10,88	-0,01
EML4	12,40	12,41	-0,01
AHCY	14,10	14,11	-0,01
COMMD5	10,22	10,24	-0,01
TOE1	11,06	11,08	-0,01
ATF7	11,97	11,98	-0,01
EIF4B	16,08	16,09	-0,01
NAAA	11,36	11,37	-0,01

**NCAPD2**	14,65	14,66	-0,01
**STIM1**	10,06	10,07	-0,01
**CHST6**	13,80	13,81	-0,01
**NOS2**	10,09	10,10	-0,01
**URI1**	12,67	12,69	-0,01
**ASCC1**	10,96	10,97	-0,01
**MYLIP**	10,40	10,41	-0,01
**HR**	12,61	12,63	-0,01
**POU2AF1**	12,20	12,22	-0,01
**MRPL57**	11,85	11,86	-0,01
**DUT**	14,33	14,34	-0,01
**NVL**	12,22	12,24	-0,01
**CTR9**	12,36	12,37	-0,01
**TTC21A**	2,69	2,70	-0,01
**NXPE2**	2,69	2,70	-0,01
**PRXL2C**	9,63	9,64	-0,01
**PDCD1**	10,06	10,07	-0,01
**TRIM66**	5,22	5,23	-0,01
**EIF3D**	14,55	14,56	-0,01
**PTPRZ1**	7,21	7,22	-0,01
**CYFIP2**	14,81	14,82	-0,01
**ATF1**	11,48	11,49	-0,01
**BLVRA**	10,13	10,14	-0,01
**MTUS1**	7,62	7,63	-0,01
**AUNIP**	10,25	10,26	-0,01
**SLC3A2**	13,76	13,77	-0,01
**SLC25A19**	11,81	11,83	-0,01
**KIAA1549**	11,24	11,25	-0,01
**PDE8B**	7,13	7,15	-0,01
**ABL1**	13,47	13,48	-0,01
**HRNR**	8,73	8,75	-0,01
**RDH14**	10,57	10,58	-0,01
**ARHGAP17**	13,19	13,20	-0,01
**SLC25A37**	13,30	13,31	-0,01
**SLC25A23**	11,46	11,47	-0,01
**PI4KB**	12,25	12,26	-0,01
**RUFY2**	10,15	10,16	-0,01
**KHDC1**	8,82	8,83	-0,01
**MT-ATP8**	15,50	15,52	-0,01
**STAT1**	12,48	12,49	-0,01
**CFAP20**	12,13	12,14	-0,01
**PITPNC1**	12,65	12,66	-0,01
**GSTCD**	10,95	10,96	-0,01
**IGSF6**	6,19	6,20	-0,01
**P2RY8**	13,38	13,39	-0,01
**ZNHIT6**	12,18	12,19	-0,01
**EED**	12,06	12,08	-0,01
**ANAPC13**	11,17	11,18	-0,01
**ZNF121**	13,07	13,08	-0,01
**CD96**	11,75	11,76	-0,01
**RAD52**	10,34	10,35	-0,01
**PLEKHA2**	13,69	13,71	-0,01
**HNRNPL**	15,66	15,68	-0,01
**EIF4G1**	15,36	15,37	-0,01
**COPA**	14,36	14,37	-0,01
**RPL12**	15,57	15,58	-0,01

**ANKHD1**	13,33	13,34	-0,01
**BMP8B**	8,74	8,75	-0,01
**MMGT1**	11,54	11,55	-0,01
**NRROS**	11,81	11,82	-0,01
**CEP89**	10,91	10,92	-0,01
**DIP2A**	11,44	11,45	-0,01
**CS**	14,54	14,55	-0,01
**DEPP1**	10,41	10,42	-0,01
**BTN3A1**	11,00	11,02	-0,01
**LSM2**	12,31	12,32	-0,01
**UQCRFS1**	12,96	12,97	-0,01
**MCCC1**	11,60	11,62	-0,01
**ATP6V1E1**	12,22	12,23	-0,01
**CBWD2**	11,78	11,79	-0,01
**IZUMO1**	3,69	3,70	-0,01
**UBE2E1**	13,24	13,25	-0,01
**ABCB7**	11,63	11,64	-0,01
**IL6R**	7,40	7,41	-0,01
**SCAMP5**	8,18	8,19	-0,01
**ZNF875**	10,49	10,50	-0,01
**YAP1**	10,06	10,07	-0,01
**MFSD2A**	10,29	10,30	-0,01
**ANKLE2**	13,60	13,61	-0,01
**GLT1D1**	12,24	12,25	-0,01
**MKRN2**	11,78	11,79	-0,01
**POLG**	12,86	12,87	-0,01
**ARID1B**	13,74	13,75	-0,01
**SEPHS2**	12,83	12,84	-0,01
**RASSF5**	13,55	13,56	-0,01
**MPP6**	12,35	12,36	-0,01
**PLCG2**	12,34	12,35	-0,01
**DNAJC25**	10,21	10,22	-0,01
**RAPGEF1**	13,32	13,33	-0,01
**CEBPB**	9,74	9,75	-0,01
**DCUN1D4**	11,79	11,80	-0,01
**CCDC71L**	10,09	10,10	-0,01
**LSM5**	12,21	12,22	-0,01
**TRIQK**	9,64	9,65	-0,01
**LLPH**	12,53	12,54	-0,01
**GLRX2**	11,19	11,20	-0,01
**KLF6**	12,21	12,22	-0,01
**TACC2**	9,46	9,47	-0,01
**SREK1**	13,67	13,68	-0,01
**TTYH2**	9,32	9,33	-0,01
**ESYT1**	13,50	13,51	-0,01
**MT-ND6**	15,20	15,21	-0,01
**SLC25A10**	11,06	11,07	-0,01
**MANBAL**	8,83	8,84	-0,01
**CYP2C8**	2,45	2,46	-0,01
**TRA2B**	14,94	14,95	-0,01
**PMM1**	8,64	8,65	-0,01
**BLOC1S5**	11,13	11,14	-0,01
**TRAPPC6B**	10,85	10,86	-0,01
**ATP6V1C2**	10,09	10,09	-0,01
**RRAGB**	8,93	8,94	-0,01
**CALCOCO2**	12,59	12,60	-0,01

**C22orf39**	11,76	11,77	-0,01
**FAAP100**	11,77	11,78	-0,01
**RAB3GAP1**	12,14	12,15	-0,01
**SLC11A2**	11,77	11,78	-0,01
**SLC22A18**	7,47	7,48	-0,01
**WRNIP1**	12,26	12,27	-0,01
**CHST2**	5,33	5,34	-0,01
**GATD1**	12,37	12,38	-0,01
**MACO1**	11,67	11,68	-0,01
**TTC7A**	14,08	14,09	-0,01
**TNPO2**	12,98	12,99	-0,01
**FRA10AC1**	10,59	10,60	-0,01
**APC**	12,45	12,46	-0,01
**BTF3**	15,37	15,37	-0,01
**SLC16A4**	7,76	7,77	-0,01
**CYFIP1**	13,85	13,86	-0,01
**ELMO1**	11,94	11,95	-0,01
**PLK2**	10,93	10,94	-0,01
**NOM1**	12,35	12,36	-0,01
**SPOPL**	11,00	11,00	-0,01
**CASP7**	11,46	11,47	-0,01
**FIGNL1**	12,35	12,36	-0,01
**EDEM2**	10,22	10,23	-0,01
**NRBP2**	9,70	9,70	-0,01
**PRRG4**	8,70	8,71	-0,01
**LAP3**	13,59	13,60	-0,01
**ACOT9**	10,45	10,46	-0,01
**PDCD11**	13,85	13,86	-0,01
**CLSTN1**	13,46	13,47	-0,01
**GPR18**	5,90	5,91	-0,01
**LRCH3**	11,83	11,84	-0,01
**UCHL1**	13,66	13,66	-0,01
**SLC2A1**	11,57	11,58	-0,01
**NRDE2**	11,39	11,40	-0,01
**VMP1**	11,96	11,96	-0,01
**RPL29**	15,81	15,81	-0,01
**DERA**	11,65	11,66	-0,01
**TMEM106B**	12,14	12,14	-0,01
**UBE2L6**	12,13	12,14	-0,01
**CAPNS1**	12,96	12,96	-0,01
**CDC25C**	9,61	9,62	-0,01
**UMAD1**	8,63	8,63	-0,01
**YWHAE**	16,05	16,05	-0,01
**KIF2C**	13,00	13,00	-0,01
**PEPD**	11,93	11,94	-0,01
**KCNH2**	6,97	6,98	-0,01
**AP002495.1**	7,01	7,02	-0,01
**DUSP14**	10,88	10,89	-0,01
**EPB41L2**	14,25	14,26	-0,01
**ZNF66**	10,33	10,34	-0,01
**ARPC2**	13,95	13,96	-0,01
**HMBOX1**	11,42	11,42	-0,01
**PNPLA6**	10,90	10,90	-0,01
**DUSP18**	8,56	8,57	-0,01
**UTP11**	12,38	12,39	-0,01
**L3MBTL3**	11,40	11,41	-0,01

**FSTL3**	8,69	8,70	-0,01
**RPL23A**	16,14	16,15	-0,01
**TRIM13**	12,65	12,65	-0,01
**EZH1**	11,65	11,66	-0,01
**GRHPR**	12,08	12,09	-0,01
**TYW1**	11,13	11,14	-0,01
**RAD23B**	14,41	14,42	-0,01
**GTF2A2**	12,59	12,60	-0,01
**MAP2K2**	12,69	12,70	-0,01
**MTBP**	11,33	11,34	-0,01
**ASXL1**	13,25	13,26	-0,01
**ATG4A**	9,32	9,33	-0,01
**SMPD4**	13,43	13,43	-0,01
**ATP5PF**	12,64	12,65	-0,01
**ZIK1**	12,03	12,04	-0,01
**SEPHS1**	13,63	13,63	-0,01
**SSR4**	12,52	12,52	-0,01
**SRI**	12,74	12,75	-0,01
**RAPGEFL1**	9,57	9,57	-0,01
**PLXND1**	13,41	13,42	-0,01
**RNGTT**	11,08	11,09	-0,01
**SUB1**	13,81	13,81	-0,01
**ARFGAP3**	12,04	12,04	-0,01
**CSNK2B**	13,07	13,08	-0,01
**MAPK13**	11,89	11,90	-0,01
**PDIA4**	14,61	14,62	-0,01
**POLR2K**	12,52	12,53	-0,01
**CENPJ**	11,91	11,92	-0,01
**ARHGEF10**	11,53	11,54	-0,01
**ZNF75A**	10,72	10,72	-0,01
**SMURF1**	11,94	11,94	-0,01
**PCBD2**	10,20	10,21	-0,01
**ATXN7L1**	9,91	9,91	-0,01
**STMN1**	16,12	16,13	-0,01
**PDHB**	12,30	12,31	-0,01
**GPX1**	13,66	13,67	-0,01
**PIGM**	11,76	11,76	-0,01
**SLC25A17**	11,42	11,43	-0,01
**UQCRC1**	13,91	13,92	-0,01
**AMER1**	11,73	11,73	-0,01
**ZSWIM3**	9,11	9,11	-0,01
**SAMD10**	8,26	8,27	-0,01
**SERAC1**	8,61	8,61	-0,01
**PSMB2**	13,65	13,65	-0,01
**RPN1**	13,97	13,97	-0,01
**KDELR2**	13,22	13,22	-0,01
**PI4K2B**	12,17	12,18	-0,01
**AC087289.3**	3,48	3,49	-0,01
**LIN7C**	11,43	11,44	-0,01
**MRPL13**	12,07	12,08	-0,01
**RDH11**	12,15	12,15	-0,01
**UHRF1BP1L**	10,77	10,77	-0,01
**RPL10A**	15,92	15,92	-0,01
**HDAC10**	10,44	10,45	-0,01
**COA6**	11,36	11,36	-0,01
**GGNBP2**	12,80	12,80	-0,01

**TPM4**	14,94	14,94	-0,01
**TM2D1**	10,29	10,29	-0,01
**ZNF665**	9,35	9,36	-0,01
**PTRH2**	11,44	11,45	-0,01
**PHF20L1**	13,27	13,27	-0,01
**TSSK4**	7,25	7,25	-0,01
**CCNH**	11,00	11,01	-0,01
**UROD**	10,92	10,92	-0,00
**ALG6**	11,19	11,19	-0,00
**KARS1**	14,80	14,80	-0,00
**PC**	10,64	10,64	-0,00
**SH3PXD2B**	9,73	9,74	-0,00
**ATP8B2**	13,02	13,02	-0,00
**GPR173**	9,82	9,83	-0,00
**INO80D**	11,89	11,90	-0,00
**LRRFIP1**	14,98	14,98	-0,00
**RACGAP1**	13,25	13,25	-0,00
**DSTYK**	10,33	10,33	-0,00
**C20orf194**	10,97	10,97	-0,00
**TM9SF1**	10,92	10,93	-0,00
**SUDS3**	12,53	12,53	-0,00
**FAM200A**	9,86	9,87	-0,00
**PPIL1**	12,00	12,00	-0,00
**FAN1**	11,93	11,94	-0,00
**CD74**	16,32	16,32	-0,00
**RPS13**	15,53	15,53	-0,00
**CNIH1**	12,45	12,45	-0,00
**EAF2**	10,93	10,93	-0,00
**CLK3**	10,76	10,76	-0,00
**GCNT1**	8,85	8,86	-0,00
**LPIN2**	12,78	12,79	-0,00
**FMNL1**	11,89	11,89	-0,00
**CSNK2A2**	11,67	11,68	-0,00
**MMP25**	5,55	5,55	-0,00
**ALPK3**	5,55	5,55	-0,00
**CRKL**	13,49	13,49	-0,00
**MED16**	11,92	11,93	-0,00
**C22orf23**	8,19	8,20	-0,00
**ETS2**	13,42	13,43	-0,00
**ZNF57**	9,86	9,86	-0,00
**B4GALT4**	10,37	10,38	-0,00
**SLC6A13**	2,16	2,16	-0,00
**H2AZ1**	15,52	15,52	-0,00
**ATP6V1E2**	8,73	8,73	-0,00
**SATB2**	5,35	5,36	-0,00
**RCE1**	10,31	10,32	-0,00
**PWP1**	12,68	12,68	-0,00
**U2SURP**	14,54	14,54	-0,00
**GADD45A**	10,77	10,77	-0,00
**ANXA7**	12,66	12,66	-0,00
**MFAP3L**	8,24	8,24	-0,00
**WIPF1**	13,81	13,81	-0,00
**ANKRD40**	12,94	12,95	-0,00
**ZC3HAV1**	13,10	13,10	-0,00
**SEC61B**	12,24	12,24	-0,00
**YWHAG**	15,18	15,18	-0,00

**EIF1B**	11,41	11,42	-0,00
**HACD4**	10,72	10,72	-0,00
**PIK3R4**	12,07	12,07	-0,00
**SYNRG**	12,33	12,33	-0,00
**FNBP1**	6,39	6,40	-0,00
**MMP24OS**	10,33	10,34	-0,00
**KPNA6**	12,80	12,80	-0,00
**NPIPA5**	10,21	10,21	-0,00
**MFSD11**	9,86	9,87	-0,00
**NUP214**	13,07	13,07	-0,00
**TCP1**	15,55	15,55	-0,00
**PCDHGA12**	9,89	9,89	-0,00
**C5orf24**	12,64	12,64	-0,00
**GADD45G**	5,37	5,37	-0,00
**AC068547.1**	10,23	10,24	-0,00
**UBB**	15,74	15,74	-0,00
**TAF4B**	11,09	11,09	-0,00
**INTS6L**	11,42	11,42	-0,00
**GPHN**	10,60	10,60	-0,00
**BRSK1**	8,97	8,97	-0,00
**KANK2**	12,41	12,41	-0,00
**UCK2**	13,09	13,09	-0,00
**SPTAN1**	15,11	15,11	-0,00
**SLC9A3R2**	9,04	9,05	-0,00
**ZYX**	11,33	11,33	-0,00
**CERK**	13,97	13,97	-0,00
**ATIC**	13,97	13,98	-0,00
**SUMO2**	14,93	14,93	-0,00
**MOGS**	11,70	11,70	-0,00
**CACNA1A**	10,33	10,33	-0,00
**ST13**	14,88	14,88	-0,00
**SLC25A33**	11,55	11,55	-0,00
**USP53**	9,80	9,81	-0,00
**TFAP4**	11,85	11,85	-0,00
**GPN2**	11,41	11,41	-0,00
**SGPP2**	8,76	8,77	-0,00
**ZNF350**	9,95	9,95	-0,00
**ZHX2**	13,29	13,29	-0,00
**NDUFV2**	13,01	13,01	-0,00
**PHKA2**	11,99	11,99	-0,00
**RHOB**	10,69	10,69	-0,00
**PARP12**	11,28	11,28	-0,00
**NACA**	15,97	15,97	-0,00
**POLA2**	12,54	12,54	-0,00
**ZC3H8**	11,77	11,77	-0,00
**PSMA6**	15,35	15,35	-0,00
**MND1**	11,11	11,11	-0,00
**HSPA4**	14,85	14,85	-0,00
**LAMP1**	12,98	12,98	-0,00
**EIF2A**	13,29	13,29	-0,00
**MACROD2**	8,87	8,87	-0,00
**SYNGR1**	11,45	11,45	-0,00
**DDB1**	14,99	14,99	-0,00
**CDKN2AIPNL**	10,97	10,97	-0,00
**RTCB**	13,16	13,16	-0,00
**GCSAM**	11,75	11,75	-0,00

**BLK**	14,58	14,58	-0,00
**VEGFB**	11,35	11,35	-0,00
**SERP1**	14,32	14,32	-0,00
**KCNA6**	10,69	10,69	-0,00
**SLC19A1**	11,92	11,92	-0,00
**RPS2**	17,30	17,30	-0,00
**SRSF3**	15,97	15,97	-0,00
**SRGAP2C**	11,78	11,78	-0,00
**KLHL14**	8,36	8,36	-0,00
**ZBTB25**	11,43	11,43	-0,00
**SLF2**	12,64	12,64	-0,00
**SSC5D**	5,74	5,74	-0,00
**AC004593.2**	4,20	4,20	-0,00
**AC117378.1**	4,20	4,20	-0,00
**RIPOR2**	10,67	10,67	-0,00
**TFE3**	10,45	10,45	-0,00
**ABHD15**	11,95	11,95	-0,00
**DAG1**	10,86	10,86	-0,00
**PSMD7**	13,57	13,57	-0,00
**IKZF4**	10,41	10,41	-0,00
**ATG3**	12,52	12,52	-0,00
**TSPAN6**	0,00	0,00	0,00
**TNMD**	0,00	0,00	0,00
**ENPP4**	0,00	0,00	0,00
**CFTR**	0,00	0,00	0,00
**TMEM176A**	0,00	0,00	0,00
**TFPI**	0,00	0,00	0,00
**PDK4**	0,00	0,00	0,00
**ABCB5**	0,00	0,00	0,00
**SLC4A1**	0,00	0,00	0,00
**PRSS22**	0,00	0,00	0,00
**HOXA11**	0,00	0,00	0,00
**MEOX1**	0,00	0,00	0,00
**PON1**	0,00	0,00	0,00
**ASB4**	0,00	0,00	0,00
**KRT33A**	0,00	0,00	0,00
**CACNG3**	0,00	0,00	0,00
**TAC1**	0,00	0,00	0,00
**CX3CL1**	0,00	0,00	0,00
**DLX6**	0,00	0,00	0,00
**TTC22**	0,00	0,00	0,00
**CCL26**	0,00	0,00	0,00
**USH1C**	0,00	0,00	0,00
**LGALS14**	0,00	0,00	0,00
**PNPLA4**	0,00	0,00	0,00
**UPP2**	0,00	0,00	0,00
**SLC13A2**	0,00	0,00	0,00
**MATK**	0,00	0,00	0,00
**CEACAM7**	0,00	0,00	0,00
**PAX6**	0,00	0,00	0,00
**SELE**	0,00	0,00	0,00
**FMO3**	0,00	0,00	0,00
**CAMK1G**	0,00	0,00	0,00
**TFAP2B**	0,00	0,00	0,00
**TFAP2D**	0,00	0,00	0,00
**MGST1**	0,00	0,00	0,00

**PAX7**	0,00	0,00	0,00
**IYD**	0,00	0,00	0,00
**MLXIPL**	0,00	0,00	0,00
**SEMA3G**	0,00	0,00	0,00
**CD4**	0,00	0,00	0,00
**FMO1**	0,00	0,00	0,00
**SLC6A7**	0,00	0,00	0,00
**DCN**	0,00	0,00	0,00
**TYROBP**	0,00	0,00	0,00
**GABRA3**	0,00	0,00	0,00
**NR1H4**	0,00	0,00	0,00
**KDM5D**	0,00	0,00	0,00
**MAP4K5**	0,00	0,00	0,00
**SLC7A14**	0,00	0,00	0,00
**MTMR11**	0,00	0,00	0,00
**STMN4**	0,00	0,00	0,00
**ISL1**	0,00	0,00	0,00
**CLCA1**	0,00	0,00	0,00
**CLCA4**	0,00	0,00	0,00
**CNTN1**	0,00	0,00	0,00
**MARCO**	0,00	0,00	0,00
**CYP24A1**	0,00	0,00	0,00
**SYT13**	0,00	0,00	0,00
**GABRA1**	0,00	0,00	0,00
**IBSP**	0,00	0,00	0,00
**MUSK**	0,00	0,00	0,00
**EFCAB1**	0,00	0,00	0,00
**MYOC**	0,00	0,00	0,00
**CASR**	0,00	0,00	0,00
**HOXC8**	0,00	0,00	0,00
**C6**	0,00	0,00	0,00
**CDH10**	0,00	0,00	0,00
**ZPBP**	0,00	0,00	0,00
**ADAM28**	0,00	0,00	0,00
**BARX2**	0,00	0,00	0,00
**GUCA2B**	0,00	0,00	0,00
**MAGEC2**	0,00	0,00	0,00
**PREX2**	0,00	0,00	0,00
**ANO2**	0,00	0,00	0,00
**ROS1**	0,00	0,00	0,00
**LMO3**	0,00	0,00	0,00
**KITLG**	0,00	0,00	0,00
**UTS2**	0,00	0,00	0,00
**ELN**	0,00	0,00	0,00
**ENTPD2**	0,00	0,00	0,00
**PTPRN**	0,00	0,00	0,00
**FOXC1**	0,00	0,00	0,00
**SPO11**	0,00	0,00	0,00
**CBLN4**	0,00	0,00	0,00
**MCOLN3**	0,00	0,00	0,00
**SERPINB3**	0,00	0,00	0,00
**F7**	0,00	0,00	0,00
**RIMBP2**	0,00	0,00	0,00
**COL11A1**	0,00	0,00	0,00
**NCKAP1**	0,00	0,00	0,00
**ARSF**	0,00	0,00	0,00

**GSC2**	0,00	0,00	0,00
**DLX3**	0,00	0,00	0,00
**TSPAN32**	0,00	0,00	0,00
**DMRT3**	0,00	0,00	0,00
**POU1F1**	0,00	0,00	0,00
**GLP2R**	0,00	0,00	0,00
**ROPN1**	0,00	0,00	0,00
**ME1**	0,00	0,00	0,00
**NGEF**	0,00	0,00	0,00
**ACSM2B**	0,00	0,00	0,00
**DDX3Y**	0,00	0,00	0,00
**ZFY**	0,00	0,00	0,00
**PAGE1**	0,00	0,00	0,00
**PITX1**	0,00	0,00	0,00
**TRPC7**	0,00	0,00	0,00
**ADGRF5**	0,00	0,00	0,00
**ADAM7**	0,00	0,00	0,00
**MAOB**	0,00	0,00	0,00
**FGF10**	0,00	0,00	0,00
**ST6GALNAC1**	0,00	0,00	0,00
**CNGB1**	0,00	0,00	0,00
**ST6GALNAC2**	0,00	0,00	0,00
**TRPM5**	0,00	0,00	0,00
**MS4A12**	0,00	0,00	0,00
**LMCD1**	0,00	0,00	0,00
**PRLH**	0,00	0,00	0,00
**CDH19**	0,00	0,00	0,00
**SLC6A15**	0,00	0,00	0,00
**RPS6KA6**	0,00	0,00	0,00
**MOV10L1**	0,00	0,00	0,00
**CLCN4**	0,00	0,00	0,00
**CD5L**	0,00	0,00	0,00
**PTGS2**	0,00	0,00	0,00
**TEAD2**	0,00	0,00	0,00
**WSCD2**	0,00	0,00	0,00
**SEMA3C**	0,00	0,00	0,00
**ATP12A**	0,00	0,00	0,00
**PAX2**	0,00	0,00	0,00
**RGS11**	0,00	0,00	0,00
**NMRK2**	0,00	0,00	0,00
**CTTNBP2**	0,00	0,00	0,00
**ACTL6B**	0,00	0,00	0,00
**RARB**	0,00	0,00	0,00
**PAK3**	0,00	0,00	0,00
**CAPN6**	0,00	0,00	0,00
**DCX**	0,00	0,00	0,00
**TYR**	0,00	0,00	0,00
**ADCY2**	0,00	0,00	0,00
**ADCYAP1R1**	0,00	0,00	0,00
**FGF20**	0,00	0,00	0,00
**BRINP1**	0,00	0,00	0,00
**MYH7B**	0,00	0,00	0,00
**BPIFB2**	0,00	0,00	0,00
**RUNX1T1**	0,00	0,00	0,00
**CDH17**	0,00	0,00	0,00
**DUSP13**	0,00	0,00	0,00

**AFM**	0,00	0,00	0,00
**SCGN**	0,00	0,00	0,00
**RIMS1**	0,00	0,00	0,00
**MOXD1**	0,00	0,00	0,00
**SCTR**	0,00	0,00	0,00
**RDH8**	0,00	0,00	0,00
**DNAAF6**	0,00	0,00	0,00
**KCNN2**	0,00	0,00	0,00
**CFHR2**	0,00	0,00	0,00
**CXCL2**	0,00	0,00	0,00
**AFP**	0,00	0,00	0,00
**CDH7**	0,00	0,00	0,00
**SLC13A1**	0,00	0,00	0,00
**PCDHA6**	0,00	0,00	0,00
**PGR**	0,00	0,00	0,00
**COL19A1**	0,00	0,00	0,00
**EPB41L3**	0,00	0,00	0,00
**KCNK2**	0,00	0,00	0,00
**TRPM3**	0,00	0,00	0,00
**GRHL2**	0,00	0,00	0,00
**EPYC**	0,00	0,00	0,00
**SLCO1A2**	0,00	0,00	0,00
**GCKR**	0,00	0,00	0,00
**FCN1**	0,00	0,00	0,00
**ABCB1**	0,00	0,00	0,00
**WNT11**	0,00	0,00	0,00
**FOLH1**	0,00	0,00	0,00
**HBQ1**	0,00	0,00	0,00
**FAT2**	0,00	0,00	0,00
**PPEF1**	0,00	0,00	0,00
**NOX4**	0,00	0,00	0,00
**TMPRSS11E**	0,00	0,00	0,00
**MT3**	0,00	0,00	0,00
**PTHLH**	0,00	0,00	0,00
**PIR**	0,00	0,00	0,00
**REM1**	0,00	0,00	0,00
**SLC15A1**	0,00	0,00	0,00
**TMEM40**	0,00	0,00	0,00
**DEFB127**	0,00	0,00	0,00
**SIRPG**	0,00	0,00	0,00
**LHX5**	0,00	0,00	0,00
**RPH3A**	0,00	0,00	0,00
**TBX5**	0,00	0,00	0,00
**HEPH**	0,00	0,00	0,00
**LAG3**	0,00	0,00	0,00
**ANKRD24**	0,00	0,00	0,00
**SI**	0,00	0,00	0,00
**FETUB**	0,00	0,00	0,00
**P3H2**	0,00	0,00	0,00
**THPO**	0,00	0,00	0,00
**CHRD**	0,00	0,00	0,00
**CD209**	0,00	0,00	0,00
**LAMB4**	0,00	0,00	0,00
**SMPX**	0,00	0,00	0,00
**APOH**	0,00	0,00	0,00
**SLC17A6**	0,00	0,00	0,00

**CPA1**	0,00	0,00	0,00
**MYH7**	0,00	0,00	0,00
**SLC22A17**	0,00	0,00	0,00
**DAZL**	0,00	0,00	0,00
**TBL1Y**	0,00	0,00	0,00
**VNN3**	0,00	0,00	0,00
**OR1I1**	0,00	0,00	0,00
**GABRP**	0,00	0,00	0,00
**KRT31**	0,00	0,00	0,00
**FMO2**	0,00	0,00	0,00
**NXPE1**	0,00	0,00	0,00
**TDRD1**	0,00	0,00	0,00
**TPSD1**	0,00	0,00	0,00
**KCNK16**	0,00	0,00	0,00
**CRISP3**	0,00	0,00	0,00
**PGC**	0,00	0,00	0,00
**NCR2**	0,00	0,00	0,00
**MLN**	0,00	0,00	0,00
**ERMP1**	0,00	0,00	0,00
**PALMD**	0,00	0,00	0,00
**MAGEB2**	0,00	0,00	0,00
**AMELY**	0,00	0,00	0,00
**SLC7A4**	0,00	0,00	0,00
**GSTT2**	0,00	0,00	0,00
**CRYBB3**	0,00	0,00	0,00
**PLA2G3**	0,00	0,00	0,00
**LGALS2**	0,00	0,00	0,00
**Z83844.1**	0,00	0,00	0,00
**GGTLC2**	0,00	0,00	0,00
**SLC5A4**	0,00	0,00	0,00
**PDGFB**	0,00	0,00	0,00
**APOL4**	0,00	0,00	0,00
**PNPLA5**	0,00	0,00	0,00
**GZMH**	0,00	0,00	0,00
**GZMB**	0,00	0,00	0,00
**CCDC198**	0,00	0,00	0,00
**LRRC74A**	0,00	0,00	0,00
**SAMD15**	0,00	0,00	0,00
**SIX4**	0,00	0,00	0,00
**SERPINA4**	0,00	0,00	0,00
**BDKRB1**	0,00	0,00	0,00
**EFS**	0,00	0,00	0,00
**CPNE6**	0,00	0,00	0,00
**VSX1**	0,00	0,00	0,00
**R3HDML**	0,00	0,00	0,00
**HNF4A**	0,00	0,00	0,00
**RIMS4**	0,00	0,00	0,00
**DOK5**	0,00	0,00	0,00
**AVP**	0,00	0,00	0,00
**COL20A1**	0,00	0,00	0,00
**SEL1L2**	0,00	0,00	0,00
**SLC52A3**	0,00	0,00	0,00
**ANGPT4**	0,00	0,00	0,00
**SIRPB1**	0,00	0,00	0,00
**HAO1**	0,00	0,00	0,00
**PLCB4**	0,00	0,00	0,00

**TLDC2**	0,00	0,00	0,00
**OXT**	0,00	0,00	0,00
**BPI**	0,00	0,00	0,00
**CST9L**	0,00	0,00	0,00
**ASIP**	0,00	0,00	0,00
**CST4**	0,00	0,00	0,00
**SPINT3**	0,00	0,00	0,00
**EPPIN**	0,00	0,00	0,00
**SYNDIG1**	0,00	0,00	0,00
**CDH20**	0,00	0,00	0,00
**LAMA1**	0,00	0,00	0,00
**H2BW2**	0,00	0,00	0,00
**MXRA5**	0,00	0,00	0,00
**GPR143**	0,00	0,00	0,00
**GUCY2F**	0,00	0,00	0,00
**ATP1B4**	0,00	0,00	0,00
**PAGE4**	0,00	0,00	0,00
**F9**	0,00	0,00	0,00
**BMX**	0,00	0,00	0,00
**LUZP4**	0,00	0,00	0,00
**SMARCA1**	0,00	0,00	0,00
**PPP1R2C**	0,00	0,00	0,00
**OPN1LW**	0,00	0,00	0,00
**RS1**	0,00	0,00	0,00
**PCSK1N**	0,00	0,00	0,00
**GPR50**	0,00	0,00	0,00
**VGLL1**	0,00	0,00	0,00
**CD40LG**	0,00	0,00	0,00
**KLHL4**	0,00	0,00	0,00
**GABRE**	0,00	0,00	0,00
**PCDH11X**	0,00	0,00	0,00
**ITIH6**	0,00	0,00	0,00
**SRPX2**	0,00	0,00	0,00
**NALCN**	0,00	0,00	0,00
**FGF14**	0,00	0,00	0,00
**HTR2A**	0,00	0,00	0,00
**NDFIP2**	0,00	0,00	0,00
**UGGT2**	0,00	0,00	0,00
**RGCC**	0,00	0,00	0,00
**ACOD1**	0,00	0,00	0,00
**MEDAG**	0,00	0,00	0,00
**MT4**	0,00	0,00	0,00
**CBLN1**	0,00	0,00	0,00
**CCL22**	0,00	0,00	0,00
**PRSS54**	0,00	0,00	0,00
**NECAB2**	0,00	0,00	0,00
**SEC14L5**	0,00	0,00	0,00
**ZP2**	0,00	0,00	0,00
**SALL1**	0,00	0,00	0,00
**SLC6A2**	0,00	0,00	0,00
**AQP9**	0,00	0,00	0,00
**FAM189A1**	0,00	0,00	0,00
**PDGFRL**	0,00	0,00	0,00
**EYA1**	0,00	0,00	0,00
**TRPA1**	0,00	0,00	0,00
**CCN4**	0,00	0,00	0,00

**STMN2**	0,00	0,00	0,00
**GML**	0,00	0,00	0,00
**ANXA13**	0,00	0,00	0,00
**ADAM2**	0,00	0,00	0,00
**CGB2**	0,00	0,00	0,00
**CGB3**	0,00	0,00	0,00
**DKKL1**	0,00	0,00	0,00
**FCER2**	0,00	0,00	0,00
**CLEC4M**	0,00	0,00	0,00
**CASP14**	0,00	0,00	0,00
**LGALS13**	0,00	0,00	0,00
**CLC**	0,00	0,00	0,00
**EBI3**	0,00	0,00	0,00
**CEACAM4**	0,00	0,00	0,00
**MYH14**	0,00	0,00	0,00
**LIM2**	0,00	0,00	0,00
**ICAM4**	0,00	0,00	0,00
**CEACAM5**	0,00	0,00	0,00
**CRX**	0,00	0,00	0,00
**SULT2A1**	0,00	0,00	0,00
**ZNRF4**	0,00	0,00	0,00
**SIGLEC6**	0,00	0,00	0,00
**CABP5**	0,00	0,00	0,00
**FGF21**	0,00	0,00	0,00
**MIER2**	0,00	0,00	0,00
**CACNG7**	0,00	0,00	0,00
**KLF1**	0,00	0,00	0,00
**KCNN1**	0,00	0,00	0,00
**COMP**	0,00	0,00	0,00
**UPK1A**	0,00	0,00	0,00
**HAMP**	0,00	0,00	0,00
**RUNDC3B**	0,00	0,00	0,00
**CFAP69**	0,00	0,00	0,00
**PON3**	0,00	0,00	0,00
**DLX5**	0,00	0,00	0,00
**NPVF**	0,00	0,00	0,00
**CAV2**	0,00	0,00	0,00
**MET**	0,00	0,00	0,00
**WNT2**	0,00	0,00	0,00
**HOXA13**	0,00	0,00	0,00
**EVX1**	0,00	0,00	0,00
**GHRHR**	0,00	0,00	0,00
**HYAL4**	0,00	0,00	0,00
**SPAM1**	0,00	0,00	0,00
**PAX4**	0,00	0,00	0,00
**PPP1R17**	0,00	0,00	0,00
**MOGAT3**	0,00	0,00	0,00
**NOBOX**	0,00	0,00	0,00
**MYL10**	0,00	0,00	0,00
**MEOX2**	0,00	0,00	0,00
**RARRES2**	0,00	0,00	0,00
**AGR2**	0,00	0,00	0,00
**MYL7**	0,00	0,00	0,00
**GCK**	0,00	0,00	0,00
**GALNTL5**	0,00	0,00	0,00
**LHX2**	0,00	0,00	0,00

**TNFSF8**	0,00	0,00	0,00
**KCNT1**	0,00	0,00	0,00
**TYRP1**	0,00	0,00	0,00
**MPDZ**	0,00	0,00	0,00
**LHX3**	0,00	0,00	0,00
**PTGDS**	0,00	0,00	0,00
**TLX1**	0,00	0,00	0,00
**FGF8**	0,00	0,00	0,00
**PITX3**	0,00	0,00	0,00
**NEURL1**	0,00	0,00	0,00
**LGI1**	0,00	0,00	0,00
**KRT23**	0,00	0,00	0,00
**CRYBA1**	0,00	0,00	0,00
**CSF3**	0,00	0,00	0,00
**KRT37**	0,00	0,00	0,00
**HOXB6**	0,00	0,00	0,00
**CCL7**	0,00	0,00	0,00
**CCL8**	0,00	0,00	0,00
**CCL1**	0,00	0,00	0,00
**KRT32**	0,00	0,00	0,00
**ABCC3**	0,00	0,00	0,00
**PPY**	0,00	0,00	0,00
**PHOX2B**	0,00	0,00	0,00
**GABRA4**	0,00	0,00	0,00
**UGT2B10**	0,00	0,00	0,00
**CWH43**	0,00	0,00	0,00
**SULT1E1**	0,00	0,00	0,00
**ODAM**	0,00	0,00	0,00
**SMR3A**	0,00	0,00	0,00
**NMU**	0,00	0,00	0,00
**PF4V1**	0,00	0,00	0,00
**AREG**	0,00	0,00	0,00
**UCP1**	0,00	0,00	0,00
**IL2**	0,00	0,00	0,00
**CPE**	0,00	0,00	0,00
**ANXA10**	0,00	0,00	0,00
**GLRB**	0,00	0,00	0,00
**DBX1**	0,00	0,00	0,00
**P2RX3**	0,00	0,00	0,00
**MS4A6A**	0,00	0,00	0,00
**CCKBR**	0,00	0,00	0,00
**FOLR1**	0,00	0,00	0,00
**APOA5**	0,00	0,00	0,00
**APOA4**	0,00	0,00	0,00
**APOC3**	0,00	0,00	0,00
**UPK2**	0,00	0,00	0,00
**SLC1A2**	0,00	0,00	0,00
**SCGB2A2**	0,00	0,00	0,00
**C11orf21**	0,00	0,00	0,00
**CALCA**	0,00	0,00	0,00
**P3H3**	0,00	0,00	0,00
**DAO**	0,00	0,00	0,00
**SYT10**	0,00	0,00	0,00
**MYF6**	0,00	0,00	0,00
**MYF5**	0,00	0,00	0,00
**ACSS3**	0,00	0,00	0,00

**TRPV4**	0,00	0,00	0,00
**PRR4**	0,00	0,00	0,00
**PRMT8**	0,00	0,00	0,00
**MYL2**	0,00	0,00	0,00
**GPRC5D**	0,00	0,00	0,00
**MGP**	0,00	0,00	0,00
**FZD10**	0,00	0,00	0,00
**IL26**	0,00	0,00	0,00
**IFNG**	0,00	0,00	0,00
**SLCO1B3**	0,00	0,00	0,00
**APOBEC1**	0,00	0,00	0,00
**ST8SIA1**	0,00	0,00	0,00
**AICDA**	0,00	0,00	0,00
**COL12A1**	0,00	0,00	0,00
**OPRM1**	0,00	0,00	0,00
**TULP1**	0,00	0,00	0,00
**RHAG**	0,00	0,00	0,00
**IL17A**	0,00	0,00	0,00
**BMP5**	0,00	0,00	0,00
**KHDRBS2**	0,00	0,00	0,00
**PRDM13**	0,00	0,00	0,00
**SIM1**	0,00	0,00	0,00
**HDGFL1**	0,00	0,00	0,00
**COL9A1**	0,00	0,00	0,00
**VNN1**	0,00	0,00	0,00
**NR2E1**	0,00	0,00	0,00
**SLC17A2**	0,00	0,00	0,00
**PERP**	0,00	0,00	0,00
**ADGRG6**	0,00	0,00	0,00
**OR12D3**	0,00	0,00	0,00
**UNC93A**	0,00	0,00	0,00
**SLC22A2**	0,00	0,00	0,00
**C6orf118**	0,00	0,00	0,00
**IMPG1**	0,00	0,00	0,00
**TENT5A**	0,00	0,00	0,00
**CLIC5**	0,00	0,00	0,00
**MEP1A**	0,00	0,00	0,00
**TBX18**	0,00	0,00	0,00
**NUDT12**	0,00	0,00	0,00
**C7**	0,00	0,00	0,00
**CDH9**	0,00	0,00	0,00
**HAND1**	0,00	0,00	0,00
**PCDHB5**	0,00	0,00	0,00
**PCDHB6**	0,00	0,00	0,00
**HAVCR1**	0,00	0,00	0,00
**GABRG2**	0,00	0,00	0,00
**SLC27A6**	0,00	0,00	0,00
**AGXT2**	0,00	0,00	0,00
**IL5**	0,00	0,00	0,00
**CDX1**	0,00	0,00	0,00
**EHHADH**	0,00	0,00	0,00
**HRG**	0,00	0,00	0,00
**CLDN16**	0,00	0,00	0,00
**GRK7**	0,00	0,00	0,00
**FGF12**	0,00	0,00	0,00
**USP9Y**	0,00	0,00	0,00

**MORC1**	0,00	0,00	0,00
**UPK1B**	0,00	0,00	0,00
**EFCC1**	0,00	0,00	0,00
**PLSCR4**	0,00	0,00	0,00
**AADAC**	0,00	0,00	0,00
**POMC**	0,00	0,00	0,00
**ITGB6**	0,00	0,00	0,00
**FNDC4**	0,00	0,00	0,00
**GCG**	0,00	0,00	0,00
**CLIP4**	0,00	0,00	0,00
**TLX2**	0,00	0,00	0,00
**REG1A**	0,00	0,00	0,00
**EFHD1**	0,00	0,00	0,00
**NEU2**	0,00	0,00	0,00
**GNLY**	0,00	0,00	0,00
**IL1R2**	0,00	0,00	0,00
**PRKAG3**	0,00	0,00	0,00
**SMYD1**	0,00	0,00	0,00
**WNT6**	0,00	0,00	0,00
**IL1RL2**	0,00	0,00	0,00
**IL1RL1**	0,00	0,00	0,00
**IL18RAP**	0,00	0,00	0,00
**SLC5A7**	0,00	0,00	0,00
**DLX2**	0,00	0,00	0,00
**KYNU**	0,00	0,00	0,00
**CD207**	0,00	0,00	0,00
**VAX2**	0,00	0,00	0,00
**DHCR24**	0,00	0,00	0,00
**NPHS2**	0,00	0,00	0,00
**PRAMEF1**	0,00	0,00	0,00
**PRAMEF12**	0,00	0,00	0,00
**AMPD1**	0,00	0,00	0,00
**CFHR3**	0,00	0,00	0,00
**CD2**	0,00	0,00	0,00
**ZP4**	0,00	0,00	0,00
**ST6GALNAC5**	0,00	0,00	0,00
**ADGRL2**	0,00	0,00	0,00
**CR2**	0,00	0,00	0,00
**MROH9**	0,00	0,00	0,00
**FASLG**	0,00	0,00	0,00
**IRF6**	0,00	0,00	0,00
**PLPPR5**	0,00	0,00	0,00
**PLPPR4**	0,00	0,00	0,00
**MUC5B**	0,00	0,00	0,00
**A4GNT**	0,00	0,00	0,00
**ZNF541**	0,00	0,00	0,00
**CRYGD**	0,00	0,00	0,00
**TNP1**	0,00	0,00	0,00
**TTR**	0,00	0,00	0,00
**ATP10B**	0,00	0,00	0,00
**ELOVL4**	0,00	0,00	0,00
**SPACA1**	0,00	0,00	0,00
**SGIP1**	0,00	0,00	0,00
**ZC2HC1B**	0,00	0,00	0,00
**ADGB**	0,00	0,00	0,00
**RAB32**	0,00	0,00	0,00

**TCF21**	0,00	0,00	0,00
**GHRH**	0,00	0,00	0,00
**CASQ2**	0,00	0,00	0,00
**OLFM3**	0,00	0,00	0,00
**FGF23**	0,00	0,00	0,00
**GDA**	0,00	0,00	0,00
**ECRG4**	0,00	0,00	0,00
**VSX2**	0,00	0,00	0,00
**TECTB**	0,00	0,00	0,00
**NKX2-3**	0,00	0,00	0,00
**PPP1R3C**	0,00	0,00	0,00
**PRLHR**	0,00	0,00	0,00
**CPN1**	0,00	0,00	0,00
**HOXB8**	0,00	0,00	0,00
**HOXB5**	0,00	0,00	0,00
**CRHR1**	0,00	0,00	0,00
**HOXB3**	0,00	0,00	0,00
**HOXB1**	0,00	0,00	0,00
**MSX2**	0,00	0,00	0,00
**TEK**	0,00	0,00	0,00
**INSL4**	0,00	0,00	0,00
**IFNA6**	0,00	0,00	0,00
**IFNA8**	0,00	0,00	0,00
**GRIA2**	0,00	0,00	0,00
**MAGEB4**	0,00	0,00	0,00
**PCDHB8**	0,00	0,00	0,00
**SEC16B**	0,00	0,00	0,00
**GPR31**	0,00	0,00	0,00
**LYZL1**	0,00	0,00	0,00
**IQSEC3**	0,00	0,00	0,00
**ADRA1A**	0,00	0,00	0,00
**NPPB**	0,00	0,00	0,00
**PRAMEF2**	0,00	0,00	0,00
**TBX2**	0,00	0,00	0,00
**LRAT**	0,00	0,00	0,00
**TAS2R8**	0,00	0,00	0,00
**PLBD1**	0,00	0,00	0,00
**PRB2**	0,00	0,00	0,00
**IAPP**	0,00	0,00	0,00
**KCNJ8**	0,00	0,00	0,00
**TAS2R7**	0,00	0,00	0,00
**RGSL1**	0,00	0,00	0,00
**CSTA**	0,00	0,00	0,00
**DPPA4**	0,00	0,00	0,00
**GJA8**	0,00	0,00	0,00
**DEPDC7**	0,00	0,00	0,00
**GJB6**	0,00	0,00	0,00
**CCR2**	0,00	0,00	0,00
**GHSR**	0,00	0,00	0,00
**SLITRK3**	0,00	0,00	0,00
**CSMD2**	0,00	0,00	0,00
**SV2C**	0,00	0,00	0,00
**OBP2A**	0,00	0,00	0,00
**TBX22**	0,00	0,00	0,00
**MYOG**	0,00	0,00	0,00
**HS3ST2**	0,00	0,00	0,00

**PRM2**	0,00	0,00	0,00
**OPN4**	0,00	0,00	0,00
**PTGFR**	0,00	0,00	0,00
**WIPF3**	0,00	0,00	0,00
**NXPH1**	0,00	0,00	0,00
**HOXA7**	0,00	0,00	0,00
**RAMP3**	0,00	0,00	0,00
**TWIST1**	0,00	0,00	0,00
**CNTFR**	0,00	0,00	0,00
**SFTPA1**	0,00	0,00	0,00
**BICC1**	0,00	0,00	0,00
**CCDC70**	0,00	0,00	0,00
**ITIH5**	0,00	0,00	0,00
**NEUROD4**	0,00	0,00	0,00
**HOXC13**	0,00	0,00	0,00
**HOXC11**	0,00	0,00	0,00
**HOXC12**	0,00	0,00	0,00
**IL13RA2**	0,00	0,00	0,00
**SERPINA7**	0,00	0,00	0,00
**NRK**	0,00	0,00	0,00
**ESX1**	0,00	0,00	0,00
**MAGEA9**	0,00	0,00	0,00
**TNFAIP6**	0,00	0,00	0,00
**G0S2**	0,00	0,00	0,00
**PFKFB2**	0,00	0,00	0,00
**C4BPA**	0,00	0,00	0,00
**C4BPB**	0,00	0,00	0,00
**DAW1**	0,00	0,00	0,00
**FAM124B**	0,00	0,00	0,00
**MC3R**	0,00	0,00	0,00
**ARHGAP40**	0,00	0,00	0,00
**SEMG2**	0,00	0,00	0,00
**ZNF831**	0,00	0,00	0,00
**EDN3**	0,00	0,00	0,00
**ANKRD60**	0,00	0,00	0,00
**SEMG1**	0,00	0,00	0,00
**C20orf85**	0,00	0,00	0,00
**MAGEA10**	0,00	0,00	0,00
**HIF3A**	0,00	0,00	0,00
**PSG8**	0,00	0,00	0,00
**NDP**	0,00	0,00	0,00
**CRISP2**	0,00	0,00	0,00
**SLC17A3**	0,00	0,00	0,00
**SLC17A1**	0,00	0,00	0,00
**H1-1**	0,00	0,00	0,00
**MOCS1**	0,00	0,00	0,00
**KCNK17**	0,00	0,00	0,00
**CRISP1**	0,00	0,00	0,00
**OPN5**	0,00	0,00	0,00
**GCM2**	0,00	0,00	0,00
**CXCL6**	0,00	0,00	0,00
**EREG**	0,00	0,00	0,00
**TRIM51**	0,00	0,00	0,00
**SCGB1D2**	0,00	0,00	0,00
**SCGB2A1**	0,00	0,00	0,00
**WNT1**	0,00	0,00	0,00

**PIWIL1**	0,00	0,00	0,00
**SLC10A2**	0,00	0,00	0,00
**SOX21**	0,00	0,00	0,00
**AMELX**	0,00	0,00	0,00
**SOX9**	0,00	0,00	0,00
**TEKT3**	0,00	0,00	0,00
**MYH2**	0,00	0,00	0,00
**BARHL1**	0,00	0,00	0,00
**KIR2DL1**	0,00	0,00	0,00
**NPBWR2**	0,00	0,00	0,00
**AC046185.1**	0,00	0,00	0,00
**TNFSF14**	0,00	0,00	0,00
**TGM3**	0,00	0,00	0,00
**DEFB126**	0,00	0,00	0,00
**PAX1**	0,00	0,00	0,00
**CST8**	0,00	0,00	0,00
**NKX2-4**	0,00	0,00	0,00
**NKX2-2**	0,00	0,00	0,00
**CSTL1**	0,00	0,00	0,00
**CST11**	0,00	0,00	0,00
**PCSK2**	0,00	0,00	0,00
**GFRA4**	0,00	0,00	0,00
**BANF2**	0,00	0,00	0,00
**SIRPD**	0,00	0,00	0,00
**DEFB129**	0,00	0,00	0,00
**CITED1**	0,00	0,00	0,00
**GDF5**	0,00	0,00	0,00
**FAM83C**	0,00	0,00	0,00
**BPIFB1**	0,00	0,00	0,00
**SLURP1**	0,00	0,00	0,00
**FFAR2**	0,00	0,00	0,00
**KRT36**	0,00	0,00	0,00
**TSKS**	0,00	0,00	0,00
**CSN1S1**	0,00	0,00	0,00
**STATH**	0,00	0,00	0,00
**HTN1**	0,00	0,00	0,00
**DACH2**	0,00	0,00	0,00
**SIX1**	0,00	0,00	0,00
**RHOJ**	0,00	0,00	0,00
**PZP**	0,00	0,00	0,00
**PRDM7**	0,00	0,00	0,00
**CTAG2**	0,00	0,00	0,00
**TMEM35A**	0,00	0,00	0,00
**MASP1**	0,00	0,00	0,00
**PLAAT1**	0,00	0,00	0,00
**IL22**	0,00	0,00	0,00
**TSPAN8**	0,00	0,00	0,00
**PLA2G5**	0,00	0,00	0,00
**OR7A10**	0,00	0,00	0,00
**OR7C2**	0,00	0,00	0,00
**GNG13**	0,00	0,00	0,00
**OR1E2**	0,00	0,00	0,00
**GNGT1**	0,00	0,00	0,00
**A4GALT**	0,00	0,00	0,00
**GALR3**	0,00	0,00	0,00
**APOL5**	0,00	0,00	0,00

**APOBEC3A**	0,00	0,00	0,00
**KRT17**	0,00	0,00	0,00
**CPA4**	0,00	0,00	0,00
**TAS2R16**	0,00	0,00	0,00
**FEZF1**	0,00	0,00	0,00
**HOXD1**	0,00	0,00	0,00
**HOXD3**	0,00	0,00	0,00
**HOXD9**	0,00	0,00	0,00
**HOXD10**	0,00	0,00	0,00
**HOXD11**	0,00	0,00	0,00
**HOXD13**	0,00	0,00	0,00
**ISLR**	0,00	0,00	0,00
**THAP10**	0,00	0,00	0,00
**BBOX1**	0,00	0,00	0,00
**MYOD1**	0,00	0,00	0,00
**AIPL1**	0,00	0,00	0,00
**KLK10**	0,00	0,00	0,00
**KLK8**	0,00	0,00	0,00
**FOXA1**	0,00	0,00	0,00
**EGLN3**	0,00	0,00	0,00
**RNASE1**	0,00	0,00	0,00
**ART1**	0,00	0,00	0,00
**RPS4Y1**	0,00	0,00	0,00
**VCY1B**	0,00	0,00	0,00
**CDY2B**	0,00	0,00	0,00
**PLPPR3**	0,00	0,00	0,00
**INS-IGF2**	0,00	0,00	0,00
**LBP**	0,00	0,00	0,00
**TSPAN16**	0,00	0,00	0,00
**ANGPTL8**	0,00	0,00	0,00
**LRCH2**	0,00	0,00	0,00
**BMP15**	0,00	0,00	0,00
**UNC13A**	0,00	0,00	0,00
**HRC**	0,00	0,00	0,00
**OR11H1**	0,00	0,00	0,00
**OLFM1**	0,00	0,00	0,00
**TNNT3**	0,00	0,00	0,00
**CALY**	0,00	0,00	0,00
**HBZ**	0,00	0,00	0,00
**GATA5**	0,00	0,00	0,00
**RBBP8NL**	0,00	0,00	0,00
**HSD17B3**	0,00	0,00	0,00
**FBP2**	0,00	0,00	0,00
**PRRG1**	0,00	0,00	0,00
**BPIFA2**	0,00	0,00	0,00
**COX4I2**	0,00	0,00	0,00
**BPIFA3**	0,00	0,00	0,00
**DEFB118**	0,00	0,00	0,00
**PYY**	0,00	0,00	0,00
**TEX101**	0,00	0,00	0,00
**SLC34A1**	0,00	0,00	0,00
**ADGRE3**	0,00	0,00	0,00
**KCNC3**	0,00	0,00	0,00
**PDLIM4**	0,00	0,00	0,00
**G6PC**	0,00	0,00	0,00
**CA6**	0,00	0,00	0,00

**KRT33B**	0,00	0,00	0,00
**FSHB**	0,00	0,00	0,00
**RAI2**	0,00	0,00	0,00
**USP29**	0,00	0,00	0,00
**PPARG**	0,00	0,00	0,00
**HHLA1**	0,00	0,00	0,00
**IQCA1**	0,00	0,00	0,00
**RAMP1**	0,00	0,00	0,00
**FTHL17**	0,00	0,00	0,00
**ENAM**	0,00	0,00	0,00
**CLEC10A**	0,00	0,00	0,00
**GUCY2D**	0,00	0,00	0,00
**SCP2D1**	0,00	0,00	0,00
**SNAP25**	0,00	0,00	0,00
**SSTR4**	0,00	0,00	0,00
**RHBG**	0,00	0,00	0,00
**BCAN**	0,00	0,00	0,00
**CRP**	0,00	0,00	0,00
**APCS**	0,00	0,00	0,00
**NMUR2**	0,00	0,00	0,00
**RNF17**	0,00	0,00	0,00
**CHRM3**	0,00	0,00	0,00
**MYH8**	0,00	0,00	0,00
**DCLK1**	0,00	0,00	0,00
**RXFP2**	0,00	0,00	0,00
**STOML3**	0,00	0,00	0,00
**IRS4**	0,00	0,00	0,00
**BEX2**	0,00	0,00	0,00
**RNF128**	0,00	0,00	0,00
**LGALS12**	0,00	0,00	0,00
**PLAAT2**	0,00	0,00	0,00
**GSTT2B**	0,00	0,00	0,00
**GGT2**	0,00	0,00	0,00
**NTS**	0,00	0,00	0,00
**SPINK5**	0,00	0,00	0,00
**LYVE1**	0,00	0,00	0,00
**CATSPERB**	0,00	0,00	0,00
**VRTN**	0,00	0,00	0,00
**PEBP4**	0,00	0,00	0,00
**ADAMDEC1**	0,00	0,00	0,00
**MRO**	0,00	0,00	0,00
**CNTN6**	0,00	0,00	0,00
**REG4**	0,00	0,00	0,00
**TSHB**	0,00	0,00	0,00
**GSTM3**	0,00	0,00	0,00
**SYT6**	0,00	0,00	0,00
**CHIA**	0,00	0,00	0,00
**HMGCS2**	0,00	0,00	0,00
**ADAM30**	0,00	0,00	0,00
**VTCN1**	0,00	0,00	0,00
**NGF**	0,00	0,00	0,00
**ANO3**	0,00	0,00	0,00
**CFHR4**	0,00	0,00	0,00
**CFHR5**	0,00	0,00	0,00
**RAX**	0,00	0,00	0,00
**GRP**	0,00	0,00	0,00

**IL2RA**	0,00	0,00	0,00
**HRH4**	0,00	0,00	0,00
**RERG**	0,00	0,00	0,00
**SLCO1B1**	0,00	0,00	0,00
**KLRD1**	0,00	0,00	0,00
**USP26**	0,00	0,00	0,00
**SOX3**	0,00	0,00	0,00
**MTNR1B**	0,00	0,00	0,00
**SPOCD1**	0,00	0,00	0,00
**DSC2**	0,00	0,00	0,00
**DSG1**	0,00	0,00	0,00
**DSC3**	0,00	0,00	0,00
**DSC1**	0,00	0,00	0,00
**CBLIF**	0,00	0,00	0,00
**APLNR**	0,00	0,00	0,00
**TCN1**	0,00	0,00	0,00
**CLDN10**	0,00	0,00	0,00
**FAM189A2**	0,00	0,00	0,00
**OCM2**	0,00	0,00	0,00
**UGT2A3**	0,00	0,00	0,00
**UGT2B28**	0,00	0,00	0,00
**FAM71F1**	0,00	0,00	0,00
**TES**	0,00	0,00	0,00
**MDFIC**	0,00	0,00	0,00
**HTR1B**	0,00	0,00	0,00
**CGA**	0,00	0,00	0,00
**GJA10**	0,00	0,00	0,00
**ELF5**	0,00	0,00	0,00
**AMHR2**	0,00	0,00	0,00
**LACRT**	0,00	0,00	0,00
**KRT85**	0,00	0,00	0,00
**MAP7**	0,00	0,00	0,00
**EGR4**	0,00	0,00	0,00
**EMX1**	0,00	0,00	0,00
**KIAA0513**	0,00	0,00	0,00
**KCNK1**	0,00	0,00	0,00
**RGS8**	0,00	0,00	0,00
**PAX3**	0,00	0,00	0,00
**SLC19A3**	0,00	0,00	0,00
**WNT10A**	0,00	0,00	0,00
**SCEL**	0,00	0,00	0,00
**EDNRB**	0,00	0,00	0,00
**IL6**	0,00	0,00	0,00
**DGKB**	0,00	0,00	0,00
**MMD2**	0,00	0,00	0,00
**NKX2-8**	0,00	0,00	0,00
**GH2**	0,00	0,00	0,00
**CSH1**	0,00	0,00	0,00
**GALNT5**	0,00	0,00	0,00
**SCN7A**	0,00	0,00	0,00
**GATA4**	0,00	0,00	0,00
**IL36RN**	0,00	0,00	0,00
**IL36B**	0,00	0,00	0,00
**IL1F10**	0,00	0,00	0,00
**CFC1**	0,00	0,00	0,00
**OR13C9**	0,00	0,00	0,00

WDR38	0,00	0,00	0,00
HEMGN	0,00	0,00	0,00
NR5A1	0,00	0,00	0,00
OR1L4	0,00	0,00	0,00
LMX1B	0,00	0,00	0,00
IL33	0,00	0,00	0,00
CCL21	0,00	0,00	0,00
DMRT1	0,00	0,00	0,00
IGFBPL1	0,00	0,00	0,00
SLC22A7	0,00	0,00	0,00
TINAG	0,00	0,00	0,00
HCRTR2	0,00	0,00	0,00
CLPS	0,00	0,00	0,00
FGFBP1	0,00	0,00	0,00
TTC29	0,00	0,00	0,00
TMPRSS4	0,00	0,00	0,00
TRPC6	0,00	0,00	0,00
MMP7	0,00	0,00	0,00
MMP20	0,00	0,00	0,00
MMP27	0,00	0,00	0,00
TRIM29	0,00	0,00	0,00
BTG4	0,00	0,00	0,00
MMP13	0,00	0,00	0,00
CASP5	0,00	0,00	0,00
UNC13C	0,00	0,00	0,00
TTLL7	0,00	0,00	0,00
BRDT	0,00	0,00	0,00
CLCA2	0,00	0,00	0,00
LHCGR	0,00	0,00	0,00
SULT6B1	0,00	0,00	0,00
SIX3	0,00	0,00	0,00
CH25H	0,00	0,00	0,00
RBP4	0,00	0,00	0,00
GPR87	0,00	0,00	0,00
ZNF365	0,00	0,00	0,00
MYPN	0,00	0,00	0,00
GUCA1C	0,00	0,00	0,00
CCDC54	0,00	0,00	0,00
ARHGAP24	0,00	0,00	0,00
NDST4	0,00	0,00	0,00
PRKG2	0,00	0,00	0,00
IL21	0,00	0,00	0,00
PRDM5	0,00	0,00	0,00
CXCL9	0,00	0,00	0,00
C4orf17	0,00	0,00	0,00
TTLL8	0,00	0,00	0,00
SHISAL1	0,00	0,00	0,00
PIK3C2G	0,00	0,00	0,00
PLCZ1	0,00	0,00	0,00
SLCO1C1	0,00	0,00	0,00
SLC38A4	0,00	0,00	0,00
AMIGO2	0,00	0,00	0,00
ANP32D	0,00	0,00	0,00
LRIG3	0,00	0,00	0,00
LUM	0,00	0,00	0,00
KERA	0,00	0,00	0,00

**FOXN4**	0,00	0,00	0,00
**PDX1**	0,00	0,00	0,00
**SLC39A5**	0,00	0,00	0,00
**DHH**	0,00	0,00	0,00
**CELA1**	0,00	0,00	0,00
**KRT71**	0,00	0,00	0,00
**SRRM4**	0,00	0,00	0,00
**RNF113B**	0,00	0,00	0,00
**TTC6**	0,00	0,00	0,00
**SSTR1**	0,00	0,00	0,00
**NOVA1**	0,00	0,00	0,00
**KCNH5**	0,00	0,00	0,00
**STON2**	0,00	0,00	0,00
**FAM181A**	0,00	0,00	0,00
**SLC24A4**	0,00	0,00	0,00
**SERPINA10**	0,00	0,00	0,00
**SLC25A47**	0,00	0,00	0,00
**DUOXA2**	0,00	0,00	0,00
**DUOX2**	0,00	0,00	0,00
**BCL2A1**	0,00	0,00	0,00
**CYP11A1**	0,00	0,00	0,00
**GOLGA6D**	0,00	0,00	0,00
**CCDC33**	0,00	0,00	0,00
**CYP1A2**	0,00	0,00	0,00
**RHCG**	0,00	0,00	0,00
**WDR93**	0,00	0,00	0,00
**SEPTIN12**	0,00	0,00	0,00
**ABCC12**	0,00	0,00	0,00
**CLEC18B**	0,00	0,00	0,00
**MYOCD**	0,00	0,00	0,00
**DPEP3**	0,00	0,00	0,00
**UNC45B**	0,00	0,00	0,00
**OR4D1**	0,00	0,00	0,00
**KIF2B**	0,00	0,00	0,00
**SPATA22**	0,00	0,00	0,00
**RHBDL3**	0,00	0,00	0,00
**SPACA3**	0,00	0,00	0,00
**ABCA8**	0,00	0,00	0,00
**ADCYAP1**	0,00	0,00	0,00
**SLC13A5**	0,00	0,00	0,00
**CBLN2**	0,00	0,00	0,00
**CIB3**	0,00	0,00	0,00
**DMRTC2**	0,00	0,00	0,00
**DOP1B**	0,00	0,00	0,00
**IL19**	0,00	0,00	0,00
**TM4SF5**	0,00	0,00	0,00
**GPR32**	0,00	0,00	0,00
**ACP4**	0,00	0,00	0,00
**KLK3**	0,00	0,00	0,00
**PTH2**	0,00	0,00	0,00
**CELA2A**	0,00	0,00	0,00
**PADI3**	0,00	0,00	0,00
**MYOM3**	0,00	0,00	0,00
**IL22RA1**	0,00	0,00	0,00
**EVA1B**	0,00	0,00	0,00
**FCN3**	0,00	0,00	0,00

**CELA3A**	0,00	0,00	0,00
**CYP4B1**	0,00	0,00	0,00
**TMEM61**	0,00	0,00	0,00
**DMRTB1**	0,00	0,00	0,00
**BARHL2**	0,00	0,00	0,00
**KCNA10**	0,00	0,00	0,00
**GPA33**	0,00	0,00	0,00
**XCL1**	0,00	0,00	0,00
**XCL2**	0,00	0,00	0,00
**HMCN1**	0,00	0,00	0,00
**LHX9**	0,00	0,00	0,00
**SYT14**	0,00	0,00	0,00
**CRNN**	0,00	0,00	0,00
**S100A8**	0,00	0,00	0,00
**S100A7**	0,00	0,00	0,00
**AQP10**	0,00	0,00	0,00
**LEFTY2**	0,00	0,00	0,00
**REN**	0,00	0,00	0,00
**REG3G**	0,00	0,00	0,00
**MEIS1**	0,00	0,00	0,00
**ST6GAL2**	0,00	0,00	0,00
**C1QL2**	0,00	0,00	0,00
**CNGA3**	0,00	0,00	0,00
**NXPH2**	0,00	0,00	0,00
**SLC4A10**	0,00	0,00	0,00
**ZNF385B**	0,00	0,00	0,00
**TMEFF2**	0,00	0,00	0,00
**GULP1**	0,00	0,00	0,00
**PTH2R**	0,00	0,00	0,00
**NBEAL1**	0,00	0,00	0,00
**NYAP2**	0,00	0,00	0,00
**MARCHF4**	0,00	0,00	0,00
**LRTM1**	0,00	0,00	0,00
**ADGRG7**	0,00	0,00	0,00
**TAGLN3**	0,00	0,00	0,00
**NR1I2**	0,00	0,00	0,00
**AGTR1**	0,00	0,00	0,00
**ALDH1L1**	0,00	0,00	0,00
**SPATA16**	0,00	0,00	0,00
**AHSG**	0,00	0,00	0,00
**ECE2**	0,00	0,00	0,00
**EPHA5**	0,00	0,00	0,00
**CABS1**	0,00	0,00	0,00
**GC**	0,00	0,00	0,00
**PDGFC**	0,00	0,00	0,00
**SHISAL2B**	0,00	0,00	0,00
**HAPLN1**	0,00	0,00	0,00
**CRHBP**	0,00	0,00	0,00
**LIX1**	0,00	0,00	0,00
**ADAMTS19**	0,00	0,00	0,00
**CXCL14**	0,00	0,00	0,00
**LECT2**	0,00	0,00	0,00
**IL9**	0,00	0,00	0,00
**TIMD4**	0,00	0,00	0,00
**C1QTNF2**	0,00	0,00	0,00
**GABRA6**	0,00	0,00	0,00

**SPINK7**	0,00	0,00	0,00
**FAM50B**	0,00	0,00	0,00
**DCDC2**	0,00	0,00	0,00
**SLC17A4**	0,00	0,00	0,00
**H2BC1**	0,00	0,00	0,00
**KAAG1**	0,00	0,00	0,00
**PLA2G7**	0,00	0,00	0,00
**HMGCLL1**	0,00	0,00	0,00
**FAXC**	0,00	0,00	0,00
**CLVS2**	0,00	0,00	0,00
**GPR6**	0,00	0,00	0,00
**RSPO3**	0,00	0,00	0,00
**TAAR2**	0,00	0,00	0,00
**TAAR6**	0,00	0,00	0,00
**TAAR8**	0,00	0,00	0,00
**VIP**	0,00	0,00	0,00
**FERD3L**	0,00	0,00	0,00
**EGFR**	0,00	0,00	0,00
**IGFBP3**	0,00	0,00	0,00
**IGFBP1**	0,00	0,00	0,00
**ASB15**	0,00	0,00	0,00
**STRA8**	0,00	0,00	0,00
**ASB10**	0,00	0,00	0,00
**NLGN4X**	0,00	0,00	0,00
**TMEM47**	0,00	0,00	0,00
**SYTL5**	0,00	0,00	0,00
**AKAP4**	0,00	0,00	0,00
**DIPK2B**	0,00	0,00	0,00
**LPAR4**	0,00	0,00	0,00
**RIPPLY1**	0,00	0,00	0,00
**FRMPD3**	0,00	0,00	0,00
**HTR2C**	0,00	0,00	0,00
**IGSF1**	0,00	0,00	0,00
**ARHGAP36**	0,00	0,00	0,00
**GPR119**	0,00	0,00	0,00
**FATE1**	0,00	0,00	0,00
**MAGEA4**	0,00	0,00	0,00
**CHRNB3**	0,00	0,00	0,00
**CRH**	0,00	0,00	0,00
**PMP2**	0,00	0,00	0,00
**LACTB2**	0,00	0,00	0,00
**ATP6V0D2**	0,00	0,00	0,00
**DPYS**	0,00	0,00	0,00
**MAL2**	0,00	0,00	0,00
**FAM83A**	0,00	0,00	0,00
**GSDMC**	0,00	0,00	0,00
**ARHGAP39**	0,00	0,00	0,00
**CER1**	0,00	0,00	0,00
**IFNK**	0,00	0,00	0,00
**NTRK2**	0,00	0,00	0,00
**PLPPR1**	0,00	0,00	0,00
**OR13C4**	0,00	0,00	0,00
**ACTL7B**	0,00	0,00	0,00
**OR5C1**	0,00	0,00	0,00
**PTGES**	0,00	0,00	0,00
**LCN2**	0,00	0,00	0,00

**IDI2**	0,00	0,00	0,00
**LRIT1**	0,00	0,00	0,00
**RGR**	0,00	0,00	0,00
**LRMDA**	0,00	0,00	0,00
**VAX1**	0,00	0,00	0,00
**NPFFR1**	0,00	0,00	0,00
**NKX6-2**	0,00	0,00	0,00
**LRRC4C**	0,00	0,00	0,00
**SAA4**	0,00	0,00	0,00
**SCGB1A1**	0,00	0,00	0,00
**SYT8**	0,00	0,00	0,00
**ZNF214**	0,00	0,00	0,00
**ZNF215**	0,00	0,00	0,00
**OR5F1**	0,00	0,00	0,00
**TTC12**	0,00	0,00	0,00
**HTR3B**	0,00	0,00	0,00
**ST14**	0,00	0,00	0,00
**GGTLC1**	0,00	0,00	0,00
**SLC22A8**	0,00	0,00	0,00
**OOSP2**	0,00	0,00	0,00
**MS4A3**	0,00	0,00	0,00
**KIAA1755**	0,00	0,00	0,00
**SPINT4**	0,00	0,00	0,00
**CDH22**	0,00	0,00	0,00
**CABLES2**	0,00	0,00	0,00
**GPHA2**	0,00	0,00	0,00
**SLC22A9**	0,00	0,00	0,00
**MMP3**	0,00	0,00	0,00
**KLRF1**	0,00	0,00	0,00
**CLEC1A**	0,00	0,00	0,00
**FXYD4**	0,00	0,00	0,00
**TRIM48**	0,00	0,00	0,00
**OR5M9**	0,00	0,00	0,00
**PCDH15**	0,00	0,00	0,00
**KLHL1**	0,00	0,00	0,00
**CDH8**	0,00	0,00	0,00
**ADGRL3**	0,00	0,00	0,00
**LYPD6B**	0,00	0,00	0,00
**WDR17**	0,00	0,00	0,00
**CCDC83**	0,00	0,00	0,00
**RGS18**	0,00	0,00	0,00
**PPP1R1C**	0,00	0,00	0,00
**C11orf53**	0,00	0,00	0,00
**IL18**	0,00	0,00	0,00
**GPR158**	0,00	0,00	0,00
**LYZL2**	0,00	0,00	0,00
**THRB**	0,00	0,00	0,00
**ANK3**	0,00	0,00	0,00
**MIPOL1**	0,00	0,00	0,00
**ALLC**	0,00	0,00	0,00
**THRSP**	0,00	0,00	0,00
**SLC25A31**	0,00	0,00	0,00
**ANO4**	0,00	0,00	0,00
**DRD3**	0,00	0,00	0,00
**EDNRA**	0,00	0,00	0,00
**AKR1C2**	0,00	0,00	0,00

**VENTX**	0,00	0,00	0,00
**SERP2**	0,00	0,00	0,00
**TDO2**	0,00	0,00	0,00
**GABRA2**	0,00	0,00	0,00
**CCDC175**	0,00	0,00	0,00
**RBM46**	0,00	0,00	0,00
**SCHIP1**	0,00	0,00	0,00
**MCHR2**	0,00	0,00	0,00
**CFC1B**	0,00	0,00	0,00
**HSPB8**	0,00	0,00	0,00
**GRID2**	0,00	0,00	0,00
**RIT2**	0,00	0,00	0,00
**G6PC2**	0,00	0,00	0,00
**PTH**	0,00	0,00	0,00
**IGSF10**	0,00	0,00	0,00
**DMP1**	0,00	0,00	0,00
**MEPE**	0,00	0,00	0,00
**CLEC4F**	0,00	0,00	0,00
**ANKRD22**	0,00	0,00	0,00
**BMP3**	0,00	0,00	0,00
**CNTNAP4**	0,00	0,00	0,00
**LMNTD1**	0,00	0,00	0,00
**PLOD2**	0,00	0,00	0,00
**NRSN1**	0,00	0,00	0,00
**ZIC1**	0,00	0,00	0,00
**CPB1**	0,00	0,00	0,00
**OR14K1**	0,00	0,00	0,00
**PTPRR**	0,00	0,00	0,00
**PLA2R1**	0,00	0,00	0,00
**FEZF2**	0,00	0,00	0,00
**ADGRF4**	0,00	0,00	0,00
**FAM81B**	0,00	0,00	0,00
**SPACA7**	0,00	0,00	0,00
**LURAP1L**	0,00	0,00	0,00
**TGIF2LX**	0,00	0,00	0,00
**TMPRSS11D**	0,00	0,00	0,00
**SPHKAP**	0,00	0,00	0,00
**DDAH1**	0,00	0,00	0,00
**ANKFN1**	0,00	0,00	0,00
**SEMA3D**	0,00	0,00	0,00
**ASB17**	0,00	0,00	0,00
**CHST9**	0,00	0,00	0,00
**CDH12**	0,00	0,00	0,00
**ANGPT1**	0,00	0,00	0,00
**WNT3A**	0,00	0,00	0,00
**PPP1R3A**	0,00	0,00	0,00
**ASZ1**	0,00	0,00	0,00
**GBP5**	0,00	0,00	0,00
**C10orf90**	0,00	0,00	0,00
**PDLIM3**	0,00	0,00	0,00
**TMSB4Y**	0,00	0,00	0,00
**TMPRSS15**	0,00	0,00	0,00
**PDE1C**	0,00	0,00	0,00
**WNT7A**	0,00	0,00	0,00
**SEPTIN14**	0,00	0,00	0,00
**CNTNAP5**	0,00	0,00	0,00

ODF1	0,00	0,00	0,00
OR4K1	0,00	0,00	0,00
GPR78	0,00	0,00	0,00
SAMSN1	0,00	0,00	0,00
GRAMD2B	0,00	0,00	0,00
MAGEC1	0,00	0,00	0,00
GRIA1	0,00	0,00	0,00
XAGE2	0,00	0,00	0,00
VSIG4	0,00	0,00	0,00
CYLC2	0,00	0,00	0,00
TRIM42	0,00	0,00	0,00
RAET1L	0,00	0,00	0,00
MICU3	0,00	0,00	0,00
NAT2	0,00	0,00	0,00
GNA14	0,00	0,00	0,00
WIF1	0,00	0,00	0,00
UGT2B4	0,00	0,00	0,00
ADAMTSL3	0,00	0,00	0,00
SLC28A1	0,00	0,00	0,00
CXCL13	0,00	0,00	0,00
NAA11	0,00	0,00	0,00
CLDN17	0,00	0,00	0,00
CLDN8	0,00	0,00	0,00
CDK20	0,00	0,00	0,00
SORCS3	0,00	0,00	0,00
PCDH1	0,00	0,00	0,00
SH3RF2	0,00	0,00	0,00
PPP2R2B	0,00	0,00	0,00
HKDC1	0,00	0,00	0,00
PRG3	0,00	0,00	0,00
GLYATL2	0,00	0,00	0,00
COX6A2	0,00	0,00	0,00
ADGRG4	0,00	0,00	0,00
ZIC3	0,00	0,00	0,00
SST	0,00	0,00	0,00
LYZL4	0,00	0,00	0,00
TMEM171	0,00	0,00	0,00
C8A	0,00	0,00	0,00
TIMP4	0,00	0,00	0,00
HTR5A	0,00	0,00	0,00
CLEC18C	0,00	0,00	0,00
KIT	0,00	0,00	0,00
CACNA2D3	0,00	0,00	0,00
PWWP3B	0,00	0,00	0,00
SLC34A2	0,00	0,00	0,00
ACAN	0,00	0,00	0,00
CIB4	0,00	0,00	0,00
TRIM63	0,00	0,00	0,00
GRHL3	0,00	0,00	0,00
NLRP14	0,00	0,00	0,00
XDH	0,00	0,00	0,00
CD1B	0,00	0,00	0,00
CD1E	0,00	0,00	0,00
CPA2	0,00	0,00	0,00
CPA5	0,00	0,00	0,00
PPP1R9A	0,00	0,00	0,00

**POM121L2**	0,00	0,00	0,00
**ALAS2**	0,00	0,00	0,00
**SLAMF8**	0,00	0,00	0,00
**ITLN2**	0,00	0,00	0,00
**PLA2G2F**	0,00	0,00	0,00
**VWA5B1**	0,00	0,00	0,00
**CDA**	0,00	0,00	0,00
**PRAC1**	0,00	0,00	0,00
**HOXB13**	0,00	0,00	0,00
**C1QC**	0,00	0,00	0,00
**IGF2BP1**	0,00	0,00	0,00
**GIP**	0,00	0,00	0,00
**GJD2**	0,00	0,00	0,00
**ACTC1**	0,00	0,00	0,00
**PLA2G4D**	0,00	0,00	0,00
**IRX6**	0,00	0,00	0,00
**LCE2B**	0,00	0,00	0,00
**SPRR2G**	0,00	0,00	0,00
**ISL2**	0,00	0,00	0,00
**TEPP**	0,00	0,00	0,00
**LRRC36**	0,00	0,00	0,00
**TPPP3**	0,00	0,00	0,00
**ZFYVE28**	0,00	0,00	0,00
**PIP**	0,00	0,00	0,00
**OR3A3**	0,00	0,00	0,00
**TFF3**	0,00	0,00	0,00
**TFF2**	0,00	0,00	0,00
**TFF1**	0,00	0,00	0,00
**CRYAA**	0,00	0,00	0,00
**S100B**	0,00	0,00	0,00
**FCN2**	0,00	0,00	0,00
**NLRP4**	0,00	0,00	0,00
**CD3G**	0,00	0,00	0,00
**CXCR5**	0,00	0,00	0,00
**CCR5**	0,00	0,00	0,00
**LRRC71**	0,00	0,00	0,00
**AZGP1**	0,00	0,00	0,00
**CYP3A7**	0,00	0,00	0,00
**CCDC105**	0,00	0,00	0,00
**SH2B2**	0,00	0,00	0,00
**PGLYRP2**	0,00	0,00	0,00
**SCGB3A1**	0,00	0,00	0,00
**DMKN**	0,00	0,00	0,00
**COX7A1**	0,00	0,00	0,00
**KASH5**	0,00	0,00	0,00
**DCD**	0,00	0,00	0,00
**CD300LG**	0,00	0,00	0,00
**IZUMO2**	0,00	0,00	0,00
**OR7G1**	0,00	0,00	0,00
**KRT84**	0,00	0,00	0,00
**KRT82**	0,00	0,00	0,00
**TREML1**	0,00	0,00	0,00
**PRR35**	0,00	0,00	0,00
**HS3ST6**	0,00	0,00	0,00
**FGF19**	0,00	0,00	0,00
**CYP4A22**	0,00	0,00	0,00

**TAL1**	0,00	0,00	0,00
**SLC1A7**	0,00	0,00	0,00
**FAM151A**	0,00	0,00	0,00
**LEXM**	0,00	0,00	0,00
**BSND**	0,00	0,00	0,00
**RBP7**	0,00	0,00	0,00
**KNCN**	0,00	0,00	0,00
**TMEM82**	0,00	0,00	0,00
**ALPL**	0,00	0,00	0,00
**TTLL10**	0,00	0,00	0,00
**CCDC27**	0,00	0,00	0,00
**IL23R**	0,00	0,00	0,00
**C1orf87**	0,00	0,00	0,00
**ADGRL4**	0,00	0,00	0,00
**LRRC53**	0,00	0,00	0,00
**LHX8**	0,00	0,00	0,00
**KCNT2**	0,00	0,00	0,00
**VCAM1**	0,00	0,00	0,00
**SLAMF9**	0,00	0,00	0,00
**OR2M5**	0,00	0,00	0,00
**FCGR3B**	0,00	0,00	0,00
**LRRC52**	0,00	0,00	0,00
**TDRD5**	0,00	0,00	0,00
**WDR64**	0,00	0,00	0,00
**KLHDC8A**	0,00	0,00	0,00
**IL20**	0,00	0,00	0,00
**FCAMR**	0,00	0,00	0,00
**LRATD1**	0,00	0,00	0,00
**NEUROD1**	0,00	0,00	0,00
**ZSWIM2**	0,00	0,00	0,00
**ANKRD30BL**	0,00	0,00	0,00
**EN1**	0,00	0,00	0,00
**SPATA18**	0,00	0,00	0,00
**CFAP221**	0,00	0,00	0,00
**PDHA2**	0,00	0,00	0,00
**STPG2**	0,00	0,00	0,00
**LCE3D**	0,00	0,00	0,00
**SMCP**	0,00	0,00	0,00
**IVL**	0,00	0,00	0,00
**SPRR3**	0,00	0,00	0,00
**SPRR2D**	0,00	0,00	0,00
**BMP10**	0,00	0,00	0,00
**PGLYRP4**	0,00	0,00	0,00
**S100A9**	0,00	0,00	0,00
**S100A12**	0,00	0,00	0,00
**CRYGC**	0,00	0,00	0,00
**GABRB1**	0,00	0,00	0,00
**ALPI**	0,00	0,00	0,00
**CLDN1**	0,00	0,00	0,00
**LENEP**	0,00	0,00	0,00
**TAFA4**	0,00	0,00	0,00
**CCKAR**	0,00	0,00	0,00
**PROK2**	0,00	0,00	0,00
**TEX55**	0,00	0,00	0,00
**PDCL2**	0,00	0,00	0,00
**CCDC141**	0,00	0,00	0,00

**FEV**	0,00	0,00	0,00
**CRYBA2**	0,00	0,00	0,00
**FCRL4**	0,00	0,00	0,00
**TRAT1**	0,00	0,00	0,00
**DPPA2**	0,00	0,00	0,00
**MNDA**	0,00	0,00	0,00
**PYHIN1**	0,00	0,00	0,00
**SLC2A2**	0,00	0,00	0,00
**FABP1**	0,00	0,00	0,00
**CTLA4**	0,00	0,00	0,00
**ICOS**	0,00	0,00	0,00
**CADPS**	0,00	0,00	0,00
**NKX6-1**	0,00	0,00	0,00
**SYNPR**	0,00	0,00	0,00
**CLRN1**	0,00	0,00	0,00
**DCLK3**	0,00	0,00	0,00
**DNASE1L3**	0,00	0,00	0,00
**PCOLCE2**	0,00	0,00	0,00
**CXCL3**	0,00	0,00	0,00
**CXCL5**	0,00	0,00	0,00
**PPBP**	0,00	0,00	0,00
**PF4**	0,00	0,00	0,00
**CXCL1**	0,00	0,00	0,00
**PLSCR2**	0,00	0,00	0,00
**CCDC158**	0,00	0,00	0,00
**CPA3**	0,00	0,00	0,00
**TM4SF18**	0,00	0,00	0,00
**TCF23**	0,00	0,00	0,00
**CDCP1**	0,00	0,00	0,00
**CCR1**	0,00	0,00	0,00
**RTP3**	0,00	0,00	0,00
**CFAP100**	0,00	0,00	0,00
**OTOP1**	0,00	0,00	0,00
**CAMP**	0,00	0,00	0,00
**FBXW12**	0,00	0,00	0,00
**INTU**	0,00	0,00	0,00
**ETNPPL**	0,00	0,00	0,00
**PITX2**	0,00	0,00	0,00
**NDST3**	0,00	0,00	0,00
**HAND2**	0,00	0,00	0,00
**SMIM43**	0,00	0,00	0,00
**ADAD1**	0,00	0,00	0,00
**ASB5**	0,00	0,00	0,00
**TMEM144**	0,00	0,00	0,00
**NPY1R**	0,00	0,00	0,00
**NPY5R**	0,00	0,00	0,00
**EDIL3**	0,00	0,00	0,00
**RNF180**	0,00	0,00	0,00
**PRDM9**	0,00	0,00	0,00
**SCGB3A2**	0,00	0,00	0,00
**SPINK1**	0,00	0,00	0,00
**HTR4**	0,00	0,00	0,00
**GPX8**	0,00	0,00	0,00
**SPZ1**	0,00	0,00	0,00
**ENPP6**	0,00	0,00	0,00
**EGFLAM**	0,00	0,00	0,00

TMEM174	0,00	0,00	0,00
CARTPT	0,00	0,00	0,00
FAM170A	0,00	0,00	0,00
ACSL6	0,00	0,00	0,00
IL3	0,00	0,00	0,00
CSF2	0,00	0,00	0,00
GDF9	0,00	0,00	0,00
GRIK2	0,00	0,00	0,00
FABP7	0,00	0,00	0,00
TLX3	0,00	0,00	0,00
CALHM4	0,00	0,00	0,00
TBXT	0,00	0,00	0,00
IL22RA2	0,00	0,00	0,00
DACT2	0,00	0,00	0,00
H2AC1	0,00	0,00	0,00
ANKRD55	0,00	0,00	0,00
TBX20	0,00	0,00	0,00
HCN1	0,00	0,00	0,00
NEUROD6	0,00	0,00	0,00
GPR85	0,00	0,00	0,00
TEX47	0,00	0,00	0,00
SP8	0,00	0,00	0,00
SOX17	0,00	0,00	0,00
C8orf48	0,00	0,00	0,00
SLC30A8	0,00	0,00	0,00
TNFRSF11B	0,00	0,00	0,00
EN2	0,00	0,00	0,00
KCNV1	0,00	0,00	0,00
DEFA5	0,00	0,00	0,00
DEFA4	0,00	0,00	0,00
DEFA6	0,00	0,00	0,00
UNCX	0,00	0,00	0,00
SPAG11B	0,00	0,00	0,00
SLC7A13	0,00	0,00	0,00
DCSTAMP	0,00	0,00	0,00
FREM1	0,00	0,00	0,00
DIRAS2	0,00	0,00	0,00
PRKACG	0,00	0,00	0,00
ZMAT4	0,00	0,00	0,00
C8orf34	0,00	0,00	0,00
TMC1	0,00	0,00	0,00
SSMEM1	0,00	0,00	0,00
SVEP1	0,00	0,00	0,00
PGAP4	0,00	0,00	0,00
CFAP47	0,00	0,00	0,00
VEGFD	0,00	0,00	0,00
OR1K1	0,00	0,00	0,00
NLGN4Y	0,00	0,00	0,00
HDX	0,00	0,00	0,00
ARMC3	0,00	0,00	0,00
GPR101	0,00	0,00	0,00
LRFN5	0,00	0,00	0,00
PHYHIPL	0,00	0,00	0,00
PHOX2A	0,00	0,00	0,00
MBL2	0,00	0,00	0,00
HEPACAM	0,00	0,00	0,00

**PKNOX2**	0,00	0,00	0,00
**RPL10L**	0,00	0,00	0,00
**MAGEC3**	0,00	0,00	0,00
**NOXRED1**	0,00	0,00	0,00
**AKR1E2**	0,00	0,00	0,00
**OTX2**	0,00	0,00	0,00
**DRGX**	0,00	0,00	0,00
**CLEC1B**	0,00	0,00	0,00
**TMEM52B**	0,00	0,00	0,00
**OR4K2**	0,00	0,00	0,00
**ERICH6B**	0,00	0,00	0,00
**C10orf82**	0,00	0,00	0,00
**ANKRD2**	0,00	0,00	0,00
**SERPINA12**	0,00	0,00	0,00
**KCNC2**	0,00	0,00	0,00
**HTRA1**	0,00	0,00	0,00
**PASD1**	0,00	0,00	0,00
**TMCO5A**	0,00	0,00	0,00
**IL25**	0,00	0,00	0,00
**SVOP**	0,00	0,00	0,00
**SPATA19**	0,00	0,00	0,00
**AVPR1A**	0,00	0,00	0,00
**C16orf78**	0,00	0,00	0,00
**OPN1MW2**	0,00	0,00	0,00
**GABRB3**	0,00	0,00	0,00
**SPIC**	0,00	0,00	0,00
**TBATA**	0,00	0,00	0,00
**CYYR1**	0,00	0,00	0,00
**POTED**	0,00	0,00	0,00
**MOGAT2**	0,00	0,00	0,00
**SERPINB7**	0,00	0,00	0,00
**LMO1**	0,00	0,00	0,00
**CRABP1**	0,00	0,00	0,00
**PRTG**	0,00	0,00	0,00
**PKD1L2**	0,00	0,00	0,00
**CLEC3A**	0,00	0,00	0,00
**CLEC4E**	0,00	0,00	0,00
**CLEC4D**	0,00	0,00	0,00
**SLC38A8**	0,00	0,00	0,00
**CPLX4**	0,00	0,00	0,00
**GALR1**	0,00	0,00	0,00
**CDH16**	0,00	0,00	0,00
**MC4R**	0,00	0,00	0,00
**MMP10**	0,00	0,00	0,00
**HTR3A**	0,00	0,00	0,00
**PLIN1**	0,00	0,00	0,00
**CHP2**	0,00	0,00	0,00
**MS4A6E**	0,00	0,00	0,00
**MS4A5**	0,00	0,00	0,00
**EPB42**	0,00	0,00	0,00
**TGM6**	0,00	0,00	0,00
**CCDC178**	0,00	0,00	0,00
**MS4A15**	0,00	0,00	0,00
**TERB2**	0,00	0,00	0,00
**SUN5**	0,00	0,00	0,00
**BPIFB6**	0,00	0,00	0,00

**ANKRD40CL**	0,00	0,00	0,00
**TBC1D21**	0,00	0,00	0,00
**PRRX2**	0,00	0,00	0,00
**UGT1A6**	0,00	0,00	0,00
**C16orf92**	0,00	0,00	0,00
**CD3D**	0,00	0,00	0,00
**OR51E2**	0,00	0,00	0,00
**OR51I1**	0,00	0,00	0,00
**OR51Q1**	0,00	0,00	0,00
**CA4**	0,00	0,00	0,00
**LALBA**	0,00	0,00	0,00
**KMT2D**	0,00	0,00	0,00
**CYP2S1**	0,00	0,00	0,00
**ANKRD33**	0,00	0,00	0,00
**KIR3DL1**	0,00	0,00	0,00
**NLRP7**	0,00	0,00	0,00
**LY6D**	0,00	0,00	0,00
**GGT6**	0,00	0,00	0,00
**KLK4**	0,00	0,00	0,00
**KLK5**	0,00	0,00	0,00
**KLK6**	0,00	0,00	0,00
**KLK11**	0,00	0,00	0,00
**KRT1**	0,00	0,00	0,00
**SOAT2**	0,00	0,00	0,00
**CABP2**	0,00	0,00	0,00
**OR8J3**	0,00	0,00	0,00
**OR5I1**	0,00	0,00	0,00
**KRT24**	0,00	0,00	0,00
**SOST**	0,00	0,00	0,00
**PLAAT5**	0,00	0,00	0,00
**MAJIN**	0,00	0,00	0,00
**OR1F1**	0,00	0,00	0,00
**OR2B2**	0,00	0,00	0,00
**FAM83B**	0,00	0,00	0,00
**PTGDR**	0,00	0,00	0,00
**PTF1A**	0,00	0,00	0,00
**FOXI1**	0,00	0,00	0,00
**PPDPFL**	0,00	0,00	0,00
**FILIP1L**	0,00	0,00	0,00
**KCNG4**	0,00	0,00	0,00
**SCNN1B**	0,00	0,00	0,00
**TXNDC2**	0,00	0,00	0,00
**LGI3**	0,00	0,00	0,00
**SFTPC**	0,00	0,00	0,00
**HJV**	0,00	0,00	0,00
**SCGB1D1**	0,00	0,00	0,00
**MYL1**	0,00	0,00	0,00
**COL3A1**	0,00	0,00	0,00
**GFRA2**	0,00	0,00	0,00
**CRYGA**	0,00	0,00	0,00
**DYNLRB2**	0,00	0,00	0,00
**ADAM18**	0,00	0,00	0,00
**GDNF**	0,00	0,00	0,00
**MUCL3**	0,00	0,00	0,00
**VWA3B**	0,00	0,00	0,00
**WFDC12**	0,00	0,00	0,00

**TSPY2**	0,00	0,00	0,00
**CXXC4**	0,00	0,00	0,00
**SHOX2**	0,00	0,00	0,00
**HTR1E**	0,00	0,00	0,00
**FSTL5**	0,00	0,00	0,00
**SOX14**	0,00	0,00	0,00
**PLA2G4F**	0,00	0,00	0,00
**CTRB1**	0,00	0,00	0,00
**CTRB2**	0,00	0,00	0,00
**TRIM49**	0,00	0,00	0,00
**TM4SF20**	0,00	0,00	0,00
**GRM5**	0,00	0,00	0,00
**NTSR2**	0,00	0,00	0,00
**SLC49A3**	0,00	0,00	0,00
**KLK7**	0,00	0,00	0,00
**ZBBX**	0,00	0,00	0,00
**PARM1**	0,00	0,00	0,00
**FAM110B**	0,00	0,00	0,00
**GOT1L1**	0,00	0,00	0,00
**PCSK9**	0,00	0,00	0,00
**GSG1L**	0,00	0,00	0,00
**OR10G3**	0,00	0,00	0,00
**OR6F1**	0,00	0,00	0,00
**RSPO1**	0,00	0,00	0,00
**GCSAML**	0,00	0,00	0,00
**CXCL10**	0,00	0,00	0,00
**HSPB3**	0,00	0,00	0,00
**STK32A**	0,00	0,00	0,00
**IL1RAPL1**	0,00	0,00	0,00
**OR5AU1**	0,00	0,00	0,00
**PDILT**	0,00	0,00	0,00
**UMOD**	0,00	0,00	0,00
**GP2**	0,00	0,00	0,00
**RNASE2**	0,00	0,00	0,00
**ELSPBP1**	0,00	0,00	0,00
**RASSF6**	0,00	0,00	0,00
**SPRR1B**	0,00	0,00	0,00
**SPRR1A**	0,00	0,00	0,00
**OR4K14**	0,00	0,00	0,00
**CRCT1**	0,00	0,00	0,00
**CCDC8**	0,00	0,00	0,00
**ZNF280A**	0,00	0,00	0,00
**MUC15**	0,00	0,00	0,00
**CLIC3**	0,00	0,00	0,00
**GKN1**	0,00	0,00	0,00
**DRD5**	0,00	0,00	0,00
**MT1B**	0,00	0,00	0,00
**ACTRT2**	0,00	0,00	0,00
**PRYP3**	0,00	0,00	0,00
**PRY**	0,00	0,00	0,00
**RBMY1F**	0,00	0,00	0,00
**PRY2**	0,00	0,00	0,00
**TACR3**	0,00	0,00	0,00
**GSX1**	0,00	0,00	0,00
**MUC17**	0,00	0,00	0,00
**AHSP**	0,00	0,00	0,00

MUC3A	0,00	0,00	0,00
PYDC1	0,00	0,00	0,00
TM4SF4	0,00	0,00	0,00
S100G	0,00	0,00	0,00
TM4SF1	0,00	0,00	0,00
ZFPM2	0,00	0,00	0,00
HSFY2	0,00	0,00	0,00
SERPINA9	0,00	0,00	0,00
SERPINA6	0,00	0,00	0,00
VGLL2	0,00	0,00	0,00
HOXD4	0,00	0,00	0,00
GYPA	0,00	0,00	0,00
ANKK1	0,00	0,00	0,00
ADRA1B	0,00	0,00	0,00
MRGPRX1	0,00	0,00	0,00
C7orf33	0,00	0,00	0,00
FABP4	0,00	0,00	0,00
CST5	0,00	0,00	0,00
CST2	0,00	0,00	0,00
EMX2	0,00	0,00	0,00
CST1	0,00	0,00	0,00
ZNF804A	0,00	0,00	0,00
KRT8	0,00	0,00	0,00
KRT78	0,00	0,00	0,00
KRT86	0,00	0,00	0,00
KRT75	0,00	0,00	0,00
KRT6C	0,00	0,00	0,00
KRT4	0,00	0,00	0,00
KRT74	0,00	0,00	0,00
KRT72	0,00	0,00	0,00
COX7B2	0,00	0,00	0,00
KRT83	0,00	0,00	0,00
SIX2	0,00	0,00	0,00
DLGAP1	0,00	0,00	0,00
OR9K2	0,00	0,00	0,00
FOXA3	0,00	0,00	0,00
SGCD	0,00	0,00	0,00
HOXB9	0,00	0,00	0,00
RBMXL2	0,00	0,00	0,00
TPD52L3	0,00	0,00	0,00
OR10A4	0,00	0,00	0,00
SDR16C5	0,00	0,00	0,00
LMOD2	0,00	0,00	0,00
FSHR	0,00	0,00	0,00
PSG6	0,00	0,00	0,00
OR7G3	0,00	0,00	0,00
OR7G2	0,00	0,00	0,00
TEX13B	0,00	0,00	0,00
OR1M1	0,00	0,00	0,00
NCBP2L	0,00	0,00	0,00
MBD3L1	0,00	0,00	0,00
PGK2	0,00	0,00	0,00
CAVIN3	0,00	0,00	0,00
CEACAM3	0,00	0,00	0,00
DCDC1	0,00	0,00	0,00
HAS2	0,00	0,00	0,00

**PDGFD**	0,00	0,00	0,00
**PLAC1**	0,00	0,00	0,00
**DDI1**	0,00	0,00	0,00
**FPR2**	0,00	0,00	0,00
**PATE1**	0,00	0,00	0,00
**C8orf74**	0,00	0,00	0,00
**OBP2B**	0,00	0,00	0,00
**OR2K2**	0,00	0,00	0,00
**RLN3**	0,00	0,00	0,00
**MORN4**	0,00	0,00	0,00
**OR2M4**	0,00	0,00	0,00
**MUC7**	0,00	0,00	0,00
**OPRPN**	0,00	0,00	0,00
**SMR3B**	0,00	0,00	0,00
**FAM241B**	0,00	0,00	0,00
**UGT2B7**	0,00	0,00	0,00
**SOSTDC1**	0,00	0,00	0,00
**NPTX1**	0,00	0,00	0,00
**KRT19**	0,00	0,00	0,00
**KRT15**	0,00	0,00	0,00
**KRT38**	0,00	0,00	0,00
**KRTAP4-4**	0,00	0,00	0,00
**KRT13**	0,00	0,00	0,00
**XAGE3**	0,00	0,00	0,00
**KRT9**	0,00	0,00	0,00
**XAGE5**	0,00	0,00	0,00
**KRT20**	0,00	0,00	0,00
**KRT27**	0,00	0,00	0,00
**OR1L6**	0,00	0,00	0,00
**NLRP5**	0,00	0,00	0,00
**SPACA5**	0,00	0,00	0,00
**MROH2B**	0,00	0,00	0,00
**OR1N2**	0,00	0,00	0,00
**OR1N1**	0,00	0,00	0,00
**RXFP1**	0,00	0,00	0,00
**LPAR3**	0,00	0,00	0,00
**DSCAM**	0,00	0,00	0,00
**NMUR1**	0,00	0,00	0,00
**P2RY6**	0,00	0,00	0,00
**S100Z**	0,00	0,00	0,00
**DEFB4A**	0,00	0,00	0,00
**SPATA46**	0,00	0,00	0,00
**PAH**	0,00	0,00	0,00
**NXNL1**	0,00	0,00	0,00
**UTF1**	0,00	0,00	0,00
**KNDC1**	0,00	0,00	0,00
**WDR87**	0,00	0,00	0,00
**PCDHB1**	0,00	0,00	0,00
**PRND**	0,00	0,00	0,00
**KLF17**	0,00	0,00	0,00
**AQP4**	0,00	0,00	0,00
**CYP4F11**	0,00	0,00	0,00
**LGALS9C**	0,00	0,00	0,00
**OR10H3**	0,00	0,00	0,00
**OR10H2**	0,00	0,00	0,00
**OR52A5**	0,00	0,00	0,00

FOXB1	0,00	0,00	0,00
LDHAL6B	0,00	0,00	0,00
ANKRD20A4P	0,00	0,00	0,00
REG3A	0,00	0,00	0,00
GAP43	0,00	0,00	0,00
REG1B	0,00	0,00	0,00
LAMB2	0,00	0,00	0,00
TEX37	0,00	0,00	0,00
CD8B	0,00	0,00	0,00
CALB2	0,00	0,00	0,00
SLC9C1	0,00	0,00	0,00
OR1A1	0,00	0,00	0,00
OR1A2	0,00	0,00	0,00
LCE1D	0,00	0,00	0,00
CCL11	0,00	0,00	0,00
OR4C11	0,00	0,00	0,00
OR8U1	0,00	0,00	0,00
TPSAB1	0,00	0,00	0,00
ATOH1	0,00	0,00	0,00
CLEC7A	0,00	0,00	0,00
CDY1	0,00	0,00	0,00
OR10V1	0,00	0,00	0,00
OR5A1	0,00	0,00	0,00
CLEC12A	0,00	0,00	0,00
OR5A2	0,00	0,00	0,00
CDY1B	0,00	0,00	0,00
OR5B12	0,00	0,00	0,00
OR5B2	0,00	0,00	0,00
OR9I1	0,00	0,00	0,00
MYOZ2	0,00	0,00	0,00
OR9G4	0,00	0,00	0,00
FUT9	0,00	0,00	0,00
HSFY1	0,00	0,00	0,00
MAB21L4	0,00	0,00	0,00
OR8J1	0,00	0,00	0,00
OR5T3	0,00	0,00	0,00
FAM170B	0,00	0,00	0,00
MUCL1	0,00	0,00	0,00
PDE3A	0,00	0,00	0,00
SMPDL3A	0,00	0,00	0,00
THEMIS	0,00	0,00	0,00
MOS	0,00	0,00	0,00
MS4A10	0,00	0,00	0,00
CCL19	0,00	0,00	0,00
OR4D9	0,00	0,00	0,00
COL6A5	0,00	0,00	0,00
OR5B3	0,00	0,00	0,00
OR10W1	0,00	0,00	0,00
FADS6	0,00	0,00	0,00
HOXC5	0,00	0,00	0,00
CYP7B1	0,00	0,00	0,00
KRT2	0,00	0,00	0,00
NBEA	0,00	0,00	0,00
MYEOV	0,00	0,00	0,00
MRGPRF	0,00	0,00	0,00
MRGPRD	0,00	0,00	0,00

XKR3	0,00	0,00	0,00
CCDC63	0,00	0,00	0,00
ACSM6	0,00	0,00	0,00
RHOD	0,00	0,00	0,00
ADAMTS20	0,00	0,00	0,00
SYT12	0,00	0,00	0,00
C11orf86	0,00	0,00	0,00
LIPM	0,00	0,00	0,00
DMRT2	0,00	0,00	0,00
SNCG	0,00	0,00	0,00
OR10K1	0,00	0,00	0,00
GPR148	0,00	0,00	0,00
STOX2	0,00	0,00	0,00
CST9	0,00	0,00	0,00
SFT2D3	0,00	0,00	0,00
C1QB	0,00	0,00	0,00
C1QA	0,00	0,00	0,00
OLR1	0,00	0,00	0,00
GLIPR1L1	0,00	0,00	0,00
RNASE8	0,00	0,00	0,00
SAA1	0,00	0,00	0,00
TMEM196	0,00	0,00	0,00
RNASE11	0,00	0,00	0,00
AGR3	0,00	0,00	0,00
NLRP13	0,00	0,00	0,00
XCR1	0,00	0,00	0,00
UGT2A1	0,00	0,00	0,00
GPRC6A	0,00	0,00	0,00
TRAPPC3L	0,00	0,00	0,00
APOBEC4	0,00	0,00	0,00
SPDYE2B	0,00	0,00	0,00
OR1L1	0,00	0,00	0,00
SPATA3	0,00	0,00	0,00
MUC13	0,00	0,00	0,00
TOPAZ1	0,00	0,00	0,00
KRT28	0,00	0,00	0,00
HOXB2	0,00	0,00	0,00
SLCO4C1	0,00	0,00	0,00
RAX2	0,00	0,00	0,00
CBY2	0,00	0,00	0,00
TENT5D	0,00	0,00	0,00
GSTA3	0,00	0,00	0,00
SELP	0,00	0,00	0,00
SNX31	0,00	0,00	0,00
GLIS1	0,00	0,00	0,00
SLC6A19	0,00	0,00	0,00
C11orf45	0,00	0,00	0,00
STARD6	0,00	0,00	0,00
GOLGA6L2	0,00	0,00	0,00
IGDCC3	0,00	0,00	0,00
MYO1H	0,00	0,00	0,00
KLK15	0,00	0,00	0,00
TRAM1L1	0,00	0,00	0,00
CMKLR1	0,00	0,00	0,00
OR7D4	0,00	0,00	0,00
BRSK2	0,00	0,00	0,00

**LEP**	0,00	0,00	0,00
**PABPC5**	0,00	0,00	0,00
**BTC**	0,00	0,00	0,00
**AMY1B**	0,00	0,00	0,00
**OR9G1**	0,00	0,00	0,00
**OR5M3**	0,00	0,00	0,00
**AMZ1**	0,00	0,00	0,00
**GPR149**	0,00	0,00	0,00
**CD164L2**	0,00	0,00	0,00
**OR5J2**	0,00	0,00	0,00
**ZIC4**	0,00	0,00	0,00
**OR10AG1**	0,00	0,00	0,00
**OR4S2**	0,00	0,00	0,00
**ZG16**	0,00	0,00	0,00
**TEX36**	0,00	0,00	0,00
**DSG4**	0,00	0,00	0,00
**RTP1**	0,00	0,00	0,00
**DES**	0,00	0,00	0,00
**WFDC5**	0,00	0,00	0,00
**OR2T1**	0,00	0,00	0,00
**ABO**	0,00	0,00	0,00
**C1orf127**	0,00	0,00	0,00
**CHST1**	0,00	0,00	0,00
**CATSPER1**	0,00	0,00	0,00
**CST6**	0,00	0,00	0,00
**PROP1**	0,00	0,00	0,00
**ISX**	0,00	0,00	0,00
**PCSK1**	0,00	0,00	0,00
**LPL**	0,00	0,00	0,00
**LRRC25**	0,00	0,00	0,00
**DPP10**	0,00	0,00	0,00
**GPR152**	0,00	0,00	0,00
**UBQLN3**	0,00	0,00	0,00
**PNLIP**	0,00	0,00	0,00
**KCNE3**	0,00	0,00	0,00
**OR4B1**	0,00	0,00	0,00
**PRM1**	0,00	0,00	0,00
**TEX26**	0,00	0,00	0,00
**CCDC197**	0,00	0,00	0,00
**RBMXL3**	0,00	0,00	0,00
**NR2F1**	0,00	0,00	0,00
**EIF4E1B**	0,00	0,00	0,00
**CBLL2**	0,00	0,00	0,00
**CREG2**	0,00	0,00	0,00
**TMEM270**	0,00	0,00	0,00
**HOXD8**	0,00	0,00	0,00
**KLHL38**	0,00	0,00	0,00
**TMPRSS7**	0,00	0,00	0,00
**NUPR1**	0,00	0,00	0,00
**ZNF683**	0,00	0,00	0,00
**MC5R**	0,00	0,00	0,00
**FOXG1**	0,00	0,00	0,00
**CIDEA**	0,00	0,00	0,00
**OR11H4**	0,00	0,00	0,00
**OR4D11**	0,00	0,00	0,00
**OR11H6**	0,00	0,00	0,00

**OR4K17**	0,00	0,00	0,00
**OR10H4**	0,00	0,00	0,00
**OR51B6**	0,00	0,00	0,00
**OR4L1**	0,00	0,00	0,00
**OR4K13**	0,00	0,00	0,00
**HMGB4**	0,00	0,00	0,00
**OR4N2**	0,00	0,00	0,00
**OR4M1**	0,00	0,00	0,00
**DMRTA1**	0,00	0,00	0,00
**PLAAT3**	0,00	0,00	0,00
**OR4C5**	0,00	0,00	0,00
**OR4C3**	0,00	0,00	0,00
**OR4S1**	0,00	0,00	0,00
**DCAF4L2**	0,00	0,00	0,00
**OR4X1**	0,00	0,00	0,00
**CNBD1**	0,00	0,00	0,00
**TGIF2LY**	0,00	0,00	0,00
**FOXC2**	0,00	0,00	0,00
**OR4F17**	0,00	0,00	0,00
**OR51V1**	0,00	0,00	0,00
**MAGEB6**	0,00	0,00	0,00
**MAGEB18**	0,00	0,00	0,00
**DEFB104A**	0,00	0,00	0,00
**OR52E2**	0,00	0,00	0,00
**DEFB103A**	0,00	0,00	0,00
**OR51L1**	0,00	0,00	0,00
**OR51G2**	0,00	0,00	0,00
**OR51A7**	0,00	0,00	0,00
**TCIM**	0,00	0,00	0,00
**OR51F2**	0,00	0,00	0,00
**FIBIN**	0,00	0,00	0,00
**TRIM60**	0,00	0,00	0,00
**DEFB104B**	0,00	0,00	0,00
**SCN4B**	0,00	0,00	0,00
**ZDHHC22**	0,00	0,00	0,00
**FAM9B**	0,00	0,00	0,00
**CETN1**	0,00	0,00	0,00
**OR2T35**	0,00	0,00	0,00
**OR14C36**	0,00	0,00	0,00
**OR2M7**	0,00	0,00	0,00
**DEFB103B**	0,00	0,00	0,00
**DEFB4B**	0,00	0,00	0,00
**OR2AJ1**	0,00	0,00	0,00
**CLDN22**	0,00	0,00	0,00
**C10orf71**	0,00	0,00	0,00
**GPR4**	0,00	0,00	0,00
**ACOT4**	0,00	0,00	0,00
**OLIG3**	0,00	0,00	0,00
**OR2G3**	0,00	0,00	0,00
**OR2G2**	0,00	0,00	0,00
**VCX2**	0,00	0,00	0,00
**ST8SIA3**	0,00	0,00	0,00
**RPRM**	0,00	0,00	0,00
**CD163**	0,00	0,00	0,00
**DEFB114**	0,00	0,00	0,00
**MAGEB10**	0,00	0,00	0,00

OR4F4	0,00	0,00	0,00
YIPF7	0,00	0,00	0,00
MYOZ1	0,00	0,00	0,00
SPATA31C2	0,00	0,00	0,00
CAPZA3	0,00	0,00	0,00
ODF3B	0,00	0,00	0,00
SPATA31E1	0,00	0,00	0,00
TSPYL6	0,00	0,00	0,00
CALHM5	0,00	0,00	0,00
MLF1	0,00	0,00	0,00
HTR3C	0,00	0,00	0,00
PPP1R42	0,00	0,00	0,00
AMER3	0,00	0,00	0,00
SPINK6	0,00	0,00	0,00
ZNF366	0,00	0,00	0,00
SLITRK1	0,00	0,00	0,00
PRM3	0,00	0,00	0,00
TNP2	0,00	0,00	0,00
SPAG11A	0,00	0,00	0,00
CALML3	0,00	0,00	0,00
CALML5	0,00	0,00	0,00
HTR1A	0,00	0,00	0,00
CCDC185	0,00	0,00	0,00
NEUROG2	0,00	0,00	0,00
TUBAL3	0,00	0,00	0,00
UCN3	0,00	0,00	0,00
AMBN	0,00	0,00	0,00
CA8	0,00	0,00	0,00
ERBB4	0,00	0,00	0,00
OR6B3	0,00	0,00	0,00
DEFB125	0,00	0,00	0,00
OTOS	0,00	0,00	0,00
C10orf53	0,00	0,00	0,00
C5orf46	0,00	0,00	0,00
RIIAD1	0,00	0,00	0,00
H1-8	0,00	0,00	0,00
MSC	0,00	0,00	0,00
FOXE1	0,00	0,00	0,00
TPRX1	0,00	0,00	0,00
LGALS7B	0,00	0,00	0,00
ERICH3	0,00	0,00	0,00
TAS1R2	0,00	0,00	0,00
TRIML2	0,00	0,00	0,00
OR13D1	0,00	0,00	0,00
C9orf50	0,00	0,00	0,00
ZFP42	0,00	0,00	0,00
CCDC89	0,00	0,00	0,00
FAM133A	0,00	0,00	0,00
C12orf42	0,00	0,00	0,00
C10orf67	0,00	0,00	0,00
CYP11B2	0,00	0,00	0,00
GIMAP7	0,00	0,00	0,00
ALOXE3	0,00	0,00	0,00
HNRNPCL1	0,00	0,00	0,00
TMEM125	0,00	0,00	0,00
SIGLECL1	0,00	0,00	0,00

PCARE	0,00	0,00	0,00
TMEM151A	0,00	0,00	0,00
GATA2	0,00	0,00	0,00
DNAJB8	0,00	0,00	0,00
HNRNPCL4	0,00	0,00	0,00
SLC17A8	0,00	0,00	0,00
SLITRK4	0,00	0,00	0,00
ALOX15B	0,00	0,00	0,00
GPHB5	0,00	0,00	0,00
OR2AP1	0,00	0,00	0,00
OR6C4	0,00	0,00	0,00
TPPP2	0,00	0,00	0,00
FCER1A	0,00	0,00	0,00
ARL14	0,00	0,00	0,00
OR6C2	0,00	0,00	0,00
NLRP8	0,00	0,00	0,00
SYCN	0,00	0,00	0,00
FOXS1	0,00	0,00	0,00
FAM216B	0,00	0,00	0,00
MRGPRX4	0,00	0,00	0,00
MRGPRX3	0,00	0,00	0,00
NKPD1	0,00	0,00	0,00
C1orf194	0,00	0,00	0,00
B3GNT3	0,00	0,00	0,00
ITLN1	0,00	0,00	0,00
OR10A7	0,00	0,00	0,00
CCR8	0,00	0,00	0,00
OR1E1	0,00	0,00	0,00
OR1R1P	0,00	0,00	0,00
FAM71E2	0,00	0,00	0,00
NKX2-6	0,00	0,00	0,00
WFDC11	0,00	0,00	0,00
OR3A1	0,00	0,00	0,00
C12orf40	0,00	0,00	0,00
LYNX1	0,00	0,00	0,00
TH	0,00	0,00	0,00
WFDC9	0,00	0,00	0,00
ADGRD2	0,00	0,00	0,00
GPR139	0,00	0,00	0,00
WFDC10A	0,00	0,00	0,00
ALX1	0,00	0,00	0,00
ITPRID1	0,00	0,00	0,00
KRTAP9-7	0,00	0,00	0,00
DEFB123	0,00	0,00	0,00
CYP8B1	0,00	0,00	0,00
OR6K6	0,00	0,00	0,00
SERTM1	0,00	0,00	0,00
OR10Q1	0,00	0,00	0,00
GLIPR1L2	0,00	0,00	0,00
DEFB119	0,00	0,00	0,00
SKIDA1	0,00	0,00	0,00
MB21D2	0,00	0,00	0,00
GSX2	0,00	0,00	0,00
SLC47A2	0,00	0,00	0,00
OR2A4	0,00	0,00	0,00
YOD1	0,00	0,00	0,00

**C3orf22**	0,00	0,00	0,00
**OR10K2**	0,00	0,00	0,00
**CHRM4**	0,00	0,00	0,00
**CLRN3**	0,00	0,00	0,00
**CHST13**	0,00	0,00	0,00
**AGTR2**	0,00	0,00	0,00
**ANKRD30B**	0,00	0,00	0,00
**OR51E1**	0,00	0,00	0,00
**ARSJ**	0,00	0,00	0,00
**HOXC9**	0,00	0,00	0,00
**HOXC10**	0,00	0,00	0,00
**DEFB112**	0,00	0,00	0,00
**OR56B4**	0,00	0,00	0,00
**FAM83H**	0,00	0,00	0,00
**OR56A1**	0,00	0,00	0,00
**OR52E4**	0,00	0,00	0,00
**C1orf105**	0,00	0,00	0,00
**OR52N1**	0,00	0,00	0,00
**FCRL6**	0,00	0,00	0,00
**CHRM2**	0,00	0,00	0,00
**MAPK15**	0,00	0,00	0,00
**MUC16**	0,00	0,00	0,00
**PENK**	0,00	0,00	0,00
**H2AW**	0,00	0,00	0,00
**CCL13**	0,00	0,00	0,00
**CFAP65**	0,00	0,00	0,00
**SAGE1**	0,00	0,00	0,00
**SOX2**	0,00	0,00	0,00
**OR8D4**	0,00	0,00	0,00
**EDDM3B**	0,00	0,00	0,00
**EDDM3A**	0,00	0,00	0,00
**FDCSP**	0,00	0,00	0,00
**TNFSF15**	0,00	0,00	0,00
**OR8H1**	0,00	0,00	0,00
**OR5T1**	0,00	0,00	0,00
**OR5T2**	0,00	0,00	0,00
**OR2Z1**	0,00	0,00	0,00
**OR8K5**	0,00	0,00	0,00
**OR8H3**	0,00	0,00	0,00
**OR8H2**	0,00	0,00	0,00
**OR5AS1**	0,00	0,00	0,00
**ACTL9**	0,00	0,00	0,00
**OR6S1**	0,00	0,00	0,00
**FTMT**	0,00	0,00	0,00
**OR4C6**	0,00	0,00	0,00
**OR4P4**	0,00	0,00	0,00
**OR4C15**	0,00	0,00	0,00
**OR4A15**	0,00	0,00	0,00
**OR52K2**	0,00	0,00	0,00
**NEUROG1**	0,00	0,00	0,00
**ADIG**	0,00	0,00	0,00
**USH1G**	0,00	0,00	0,00
**MGAT4C**	0,00	0,00	0,00
**TRIM49B**	0,00	0,00	0,00
**OR52A1**	0,00	0,00	0,00
**TMEM30B**	0,00	0,00	0,00

**MRGPRG**	0,00	0,00	0,00
**ASB18**	0,00	0,00	0,00
**CRYGB**	0,00	0,00	0,00
**LDOC1**	0,00	0,00	0,00
**FAM153B**	0,00	0,00	0,00
**GABRG3**	0,00	0,00	0,00
**NLRP10**	0,00	0,00	0,00
**FIGN**	0,00	0,00	0,00
**TMIGD1**	0,00	0,00	0,00
**PRAMEF8**	0,00	0,00	0,00
**OR5P3**	0,00	0,00	0,00
**DAOA**	0,00	0,00	0,00
**ZNF804B**	0,00	0,00	0,00
**IFNL1**	0,00	0,00	0,00
**CDY2A**	0,00	0,00	0,00
**OTOL1**	0,00	0,00	0,00
**KCNK4**	0,00	0,00	0,00
**TBPL2**	0,00	0,00	0,00
**CLEC4G**	0,00	0,00	0,00
**VCX**	0,00	0,00	0,00
**KRTAP11-1**	0,00	0,00	0,00
**RXFP3**	0,00	0,00	0,00
**OR10G7**	0,00	0,00	0,00
**NDN**	0,00	0,00	0,00
**OR4Q3**	0,00	0,00	0,00
**KCNB2**	0,00	0,00	0,00
**RESP18**	0,00	0,00	0,00
**HOXB4**	0,00	0,00	0,00
**OR2T29**	0,00	0,00	0,00
**GSTA5**	0,00	0,00	0,00
**C1orf116**	0,00	0,00	0,00
**KRTAP13-2**	0,00	0,00	0,00
**PLCXD3**	0,00	0,00	0,00
**OR4F15**	0,00	0,00	0,00
**TMEM95**	0,00	0,00	0,00
**TCHHL1**	0,00	0,00	0,00
**RGS7**	0,00	0,00	0,00
**TCEAL7**	0,00	0,00	0,00
**WFDC10B**	0,00	0,00	0,00
**OTOP3**	0,00	0,00	0,00
**ODF3L1**	0,00	0,00	0,00
**SOX1**	0,00	0,00	0,00
**OR4M2B**	0,00	0,00	0,00
**ZNF662**	0,00	0,00	0,00
**OTOP2**	0,00	0,00	0,00
**CYLC1**	0,00	0,00	0,00
**PCP4**	0,00	0,00	0,00
**NKX2-5**	0,00	0,00	0,00
**FREM3**	0,00	0,00	0,00
**BEGAIN**	0,00	0,00	0,00
**GPC6**	0,00	0,00	0,00
**FAM43B**	0,00	0,00	0,00
**CALHM3**	0,00	0,00	0,00
**TMEM119**	0,00	0,00	0,00
**GABRR3**	0,00	0,00	0,00
**POTEC**	0,00	0,00	0,00

**CTNNA3**	0,00	0,00	0,00
**CCDC60**	0,00	0,00	0,00
**FAM9A**	0,00	0,00	0,00
**MAGEA2B**	0,00	0,00	0,00
**OR2T34**	0,00	0,00	0,00
**OR52L1**	0,00	0,00	0,00
**SPDYE4**	0,00	0,00	0,00
**REC114**	0,00	0,00	0,00
**CABCOCO1**	0,00	0,00	0,00
**OR56A4**	0,00	0,00	0,00
**TFDP3**	0,00	0,00	0,00
**TRIM61**	0,00	0,00	0,00
**TREX2**	0,00	0,00	0,00
**ACSM5**	0,00	0,00	0,00
**C10orf120**	0,00	0,00	0,00
**IZUMO1R**	0,00	0,00	0,00
**PGPEP1L**	0,00	0,00	0,00
**TNFAIP8L3**	0,00	0,00	0,00
**FBXL7**	0,00	0,00	0,00
**GKN2**	0,00	0,00	0,00
**CCR3**	0,00	0,00	0,00
**PRR32**	0,00	0,00	0,00
**TP53TG3**	0,00	0,00	0,00
**KRTAP8-1**	0,00	0,00	0,00
**HOATZ**	0,00	0,00	0,00
**MARCHF11**	0,00	0,00	0,00
**PSG9**	0,00	0,00	0,00
**OR4N4**	0,00	0,00	0,00
**IFNL2**	0,00	0,00	0,00
**FIGLA**	0,00	0,00	0,00
**ASCL2**	0,00	0,00	0,00
**ACSM2A**	0,00	0,00	0,00
**BPY2**	0,00	0,00	0,00
**ACP7**	0,00	0,00	0,00
**B3GALT5**	0,00	0,00	0,00
**EMILIN3**	0,00	0,00	0,00
**FAM162B**	0,00	0,00	0,00
**ANKRD45**	0,00	0,00	0,00
**CFAP91**	0,00	0,00	0,00
**ARSI**	0,00	0,00	0,00
**UTY**	0,00	0,00	0,00
**SRARP**	0,00	0,00	0,00
**LRRC55**	0,00	0,00	0,00
**DNAH2**	0,00	0,00	0,00
**OR2T10**	0,00	0,00	0,00
**KRTAP20-2**	0,00	0,00	0,00
**TRIML1**	0,00	0,00	0,00
**OR4F6**	0,00	0,00	0,00
**SPRR4**	0,00	0,00	0,00
**OR10J5**	0,00	0,00	0,00
**OR1D2**	0,00	0,00	0,00
**OLIG1**	0,00	0,00	0,00
**DEFB108B**	0,00	0,00	0,00
**SIX6**	0,00	0,00	0,00
**PRKD1**	0,00	0,00	0,00
**CCSER1**	0,00	0,00	0,00

**S100A7A**	0,00	0,00	0,00
**GDF3**	0,00	0,00	0,00
**IQCF2**	0,00	0,00	0,00
**MRGPRE**	0,00	0,00	0,00
**KRTAP19-1**	0,00	0,00	0,00
**COLEC10**	0,00	0,00	0,00
**PABPC1L2B**	0,00	0,00	0,00
**OR4N5**	0,00	0,00	0,00
**KCND2**	0,00	0,00	0,00
**NCMAP**	0,00	0,00	0,00
**BPIFC**	0,00	0,00	0,00
**C1QTNF8**	0,00	0,00	0,00
**OR56A3**	0,00	0,00	0,00
**BEX5**	0,00	0,00	0,00
**DHRS7C**	0,00	0,00	0,00
**PIWIL3**	0,00	0,00	0,00
**C14orf180**	0,00	0,00	0,00
**CLDN6**	0,00	0,00	0,00
**OR51M1**	0,00	0,00	0,00
**RNLS**	0,00	0,00	0,00
**KRTAP6-1**	0,00	0,00	0,00
**DDX53**	0,00	0,00	0,00
**TRARG1**	0,00	0,00	0,00
**PRR23B**	0,00	0,00	0,00
**ZBTB7C**	0,00	0,00	0,00
**PRR16**	0,00	0,00	0,00
**DRD1**	0,00	0,00	0,00
**SRY**	0,00	0,00	0,00
**TCEAL2**	0,00	0,00	0,00
**CLCNKB**	0,00	0,00	0,00
**OR6A2**	0,00	0,00	0,00
**AQP12A**	0,00	0,00	0,00
**OR6C70**	0,00	0,00	0,00
**SLC22A10**	0,00	0,00	0,00
**RFX6**	0,00	0,00	0,00
**SPDYE16**	0,00	0,00	0,00
**SLC24A3**	0,00	0,00	0,00
**C5orf47**	0,00	0,00	0,00
**KRT76**	0,00	0,00	0,00
**FLRT2**	0,00	0,00	0,00
**INPP5J**	0,00	0,00	0,00
**NPY2R**	0,00	0,00	0,00
**MFSD6L**	0,00	0,00	0,00
**AQP12B**	0,00	0,00	0,00
**ZNF479**	0,00	0,00	0,00
**MC2R**	0,00	0,00	0,00
**MAGEA11**	0,00	0,00	0,00
**TEX33**	0,00	0,00	0,00
**NUPR2**	0,00	0,00	0,00
**SPPL2C**	0,00	0,00	0,00
**SFTPA2**	0,00	0,00	0,00
**SCN10A**	0,00	0,00	0,00
**HS6ST3**	0,00	0,00	0,00
**OR7A17**	0,00	0,00	0,00
**KRT6B**	0,00	0,00	0,00
**NR2F2**	0,00	0,00	0,00

**DLK1**	0,00	0,00	0,00
**KRT79**	0,00	0,00	0,00
**NTF3**	0,00	0,00	0,00
**SMIM23**	0,00	0,00	0,00
**MORN5**	0,00	0,00	0,00
**SRL**	0,00	0,00	0,00
**KCNIP4**	0,00	0,00	0,00
**SPATA31A6**	0,00	0,00	0,00
**OR6C76**	0,00	0,00	0,00
**NPAP1**	0,00	0,00	0,00
**CCDC190**	0,00	0,00	0,00
**TMPRSS11B**	0,00	0,00	0,00
**PRSS38**	0,00	0,00	0,00
**TAS2R60**	0,00	0,00	0,00
**OR4C46**	0,00	0,00	0,00
**CALHM1**	0,00	0,00	0,00
**KRTAP5-5**	0,00	0,00	0,00
**LCE3A**	0,00	0,00	0,00
**LCE3E**	0,00	0,00	0,00
**CCIN**	0,00	0,00	0,00
**DEFB128**	0,00	0,00	0,00
**SLITRK2**	0,00	0,00	0,00
**LEMD1**	0,00	0,00	0,00
**HTR3E**	0,00	0,00	0,00
**KRT73**	0,00	0,00	0,00
**ZPBP2**	0,00	0,00	0,00
**KRT5**	0,00	0,00	0,00
**NBPF6**	0,00	0,00	0,00
**HTR3D**	0,00	0,00	0,00
**OR4F5**	0,00	0,00	0,00
**OR5D14**	0,00	0,00	0,00
**PRR30**	0,00	0,00	0,00
**UBL4B**	0,00	0,00	0,00
**FFAR4**	0,00	0,00	0,00
**BPIFB3**	0,00	0,00	0,00
**BPIFB4**	0,00	0,00	0,00
**SLC51B**	0,00	0,00	0,00
**LCE5A**	0,00	0,00	0,00
**LCE1E**	0,00	0,00	0,00
**BTLA**	0,00	0,00	0,00
**PABPC1L2A**	0,00	0,00	0,00
**OR10T2**	0,00	0,00	0,00
**NAP1L3**	0,00	0,00	0,00
**TMEM212**	0,00	0,00	0,00
**SLC36A3**	0,00	0,00	0,00
**SLC36A2**	0,00	0,00	0,00
**MINAR2**	0,00	0,00	0,00
**CYP4X1**	0,00	0,00	0,00
**KRT26**	0,00	0,00	0,00
**CD300E**	0,00	0,00	0,00
**OR6P1**	0,00	0,00	0,00
**KRT3**	0,00	0,00	0,00
**TMPRSS12**	0,00	0,00	0,00
**DEFB132**	0,00	0,00	0,00
**NAP1L2**	0,00	0,00	0,00
**KLK12**	0,00	0,00	0,00

RGS7BP	0,00	0,00	0,00
OR9Q1	0,00	0,00	0,00
OR9Q2	0,00	0,00	0,00
CYP4F8	0,00	0,00	0,00
DEFB107A	0,00	0,00	0,00
DEFB106A	0,00	0,00	0,00
DEFB105B	0,00	0,00	0,00
MAGEE2	0,00	0,00	0,00
FOXI2	0,00	0,00	0,00
KCNK18	0,00	0,00	0,00
IFNA10	0,00	0,00	0,00
CXCR3	0,00	0,00	0,00
TNFRSF4	0,00	0,00	0,00
KRT16	0,00	0,00	0,00
LCE1A	0,00	0,00	0,00
KRT14	0,00	0,00	0,00
KRTAP17-1	0,00	0,00	0,00
OR13F1	0,00	0,00	0,00
OR5D3P	0,00	0,00	0,00
FGF3	0,00	0,00	0,00
SERPINA11	0,00	0,00	0,00
KRTAP22-1	0,00	0,00	0,00
KRTAP19-6	0,00	0,00	0,00
KRTAP6-2	0,00	0,00	0,00
OR13C8	0,00	0,00	0,00
KRTAP19-2	0,00	0,00	0,00
KRTAP19-4	0,00	0,00	0,00
KRTAP13-4	0,00	0,00	0,00
FAM183A	0,00	0,00	0,00
KRTAP19-5	0,00	0,00	0,00
KRTAP23-1	0,00	0,00	0,00
KRTAP21-1	0,00	0,00	0,00
PNLIPRP1	0,00	0,00	0,00
KRTAP21-2	0,00	0,00	0,00
CYP4A11	0,00	0,00	0,00
OR2AK2	0,00	0,00	0,00
DEFB106B	0,00	0,00	0,00
CCK	0,00	0,00	0,00
LILRA5	0,00	0,00	0,00
LYPD6	0,00	0,00	0,00
AKR1C1	0,00	0,00	0,00
SPATA21	0,00	0,00	0,00
ANGPTL5	0,00	0,00	0,00
H1-7	0,00	0,00	0,00
LCE2A	0,00	0,00	0,00
KRTAP12-1	0,00	0,00	0,00
DAZ3	0,00	0,00	0,00
LCE2D	0,00	0,00	0,00
LCE3B	0,00	0,00	0,00
NPSR1	0,00	0,00	0,00
KRTAP9-8	0,00	0,00	0,00
LUZP2	0,00	0,00	0,00
H1-6	0,00	0,00	0,00
H2AP	0,00	0,00	0,00
ATP13A5	0,00	0,00	0,00
PRR27	0,00	0,00	0,00

**PRAMEF10**	0,00	0,00	0,00
**AGMO**	0,00	0,00	0,00
**SBK2**	0,00	0,00	0,00
**NHLRC1**	0,00	0,00	0,00
**DPPA3**	0,00	0,00	0,00
**COX8C**	0,00	0,00	0,00
**OR5W2**	0,00	0,00	0,00
**PERM1**	0,00	0,00	0,00
**C5orf52**	0,00	0,00	0,00
**ERC2**	0,00	0,00	0,00
**AMTN**	0,00	0,00	0,00
**OR2T27**	0,00	0,00	0,00
**SLC18A3**	0,00	0,00	0,00
**AMY1C**	0,00	0,00	0,00
**SSX7**	0,00	0,00	0,00
**KRTAP10-8**	0,00	0,00	0,00
**FAM205C**	0,00	0,00	0,00
**PEAR1**	0,00	0,00	0,00
**TMEM202**	0,00	0,00	0,00
**HELT**	0,00	0,00	0,00
**RTL4**	0,00	0,00	0,00
**C2orf78**	0,00	0,00	0,00
**OR6C75**	0,00	0,00	0,00
**PALM3**	0,00	0,00	0,00
**GFRAL**	0,00	0,00	0,00
**SHISA7**	0,00	0,00	0,00
**OR51I2**	0,00	0,00	0,00
**OVCH1**	0,00	0,00	0,00
**ZCCHC13**	0,00	0,00	0,00
**ZSCAN23**	0,00	0,00	0,00
**C17orf99**	0,00	0,00	0,00
**S100A3**	0,00	0,00	0,00
**CLCN1**	0,00	0,00	0,00
**RNF133**	0,00	0,00	0,00
**TREML4**	0,00	0,00	0,00
**WNT7B**	0,00	0,00	0,00
**SCGB1C1**	0,00	0,00	0,00
**PLA2G4E**	0,00	0,00	0,00
**MESP2**	0,00	0,00	0,00
**FAM25A**	0,00	0,00	0,00
**DAZ1**	0,00	0,00	0,00
**OR2AG2**	0,00	0,00	0,00
**KRTAP10-6**	0,00	0,00	0,00
**OTOG**	0,00	0,00	0,00
**FAM166A**	0,00	0,00	0,00
**AGAP4**	0,00	0,00	0,00
**PLA2G2A**	0,00	0,00	0,00
**IL17REL**	0,00	0,00	0,00
**IGFL1**	0,00	0,00	0,00
**OR6C6**	0,00	0,00	0,00
**BSPH1**	0,00	0,00	0,00
**OR6N2**	0,00	0,00	0,00
**C10orf99**	0,00	0,00	0,00
**H3-5**	0,00	0,00	0,00
**IFNA2**	0,00	0,00	0,00
**CLEC2A**	0,00	0,00	0,00

GPR21	0,00	0,00	0,00
IGHV1OR15-9	0,00	0,00	0,00
MAGEB5	0,00	0,00	0,00
NANOS2	0,00	0,00	0,00
SERPINA5	0,00	0,00	0,00
KRTDAP	0,00	0,00	0,00
OR2G6	0,00	0,00	0,00
KRTAP1-1	0,00	0,00	0,00
IGFL3	0,00	0,00	0,00
RNASE9	0,00	0,00	0,00
C2orf80	0,00	0,00	0,00
OR56A5	0,00	0,00	0,00
KRTAP24-1	0,00	0,00	0,00
OSTN	0,00	0,00	0,00
OPTC	0,00	0,00	0,00
PLA2G2E	0,00	0,00	0,00
TMCO2	0,00	0,00	0,00
SNTN	0,00	0,00	0,00
ENTPD8	0,00	0,00	0,00
BSX	0,00	0,00	0,00
GJB3	0,00	0,00	0,00
INSYN2A	0,00	0,00	0,00
UTS2B	0,00	0,00	0,00
AADACL3	0,00	0,00	0,00
SLC15A5	0,00	0,00	0,00
MAGEB16	0,00	0,00	0,00
DUSP21	0,00	0,00	0,00
CGB5	0,00	0,00	0,00
APOD	0,00	0,00	0,00
VSTM1	0,00	0,00	0,00
PRSS48	0,00	0,00	0,00
IL1RAPL2	0,00	0,00	0,00
ANKRD34B	0,00	0,00	0,00
FAM47B	0,00	0,00	0,00
FSCB	0,00	0,00	0,00
KRTAP10-12	0,00	0,00	0,00
OR14I1	0,00	0,00	0,00
KRT77	0,00	0,00	0,00
DCAF8L2	0,00	0,00	0,00
SPANXN3	0,00	0,00	0,00
TRIM64B	0,00	0,00	0,00
SPANXN4	0,00	0,00	0,00
CXCL17	0,00	0,00	0,00
OTUD6A	0,00	0,00	0,00
GJB4	0,00	0,00	0,00
OR2L13	0,00	0,00	0,00
PTPRT	0,00	0,00	0,00
MYBPC1	0,00	0,00	0,00
OR5K4	0,00	0,00	0,00
ZNF676	0,00	0,00	0,00
OR8A1	0,00	0,00	0,00
VN1R2	0,00	0,00	0,00
KRTAP4-3	0,00	0,00	0,00
OR6K2	0,00	0,00	0,00
OR10J1	0,00	0,00	0,00
HRCT1	0,00	0,00	0,00

SIRPB2	0,00	0,00	0,00
RYR1	0,00	0,00	0,00
KRTAP5-3	0,00	0,00	0,00
SULT1C3	0,00	0,00	0,00
OR2T2	0,00	0,00	0,00
OR2C3	0,00	0,00	0,00
OR10S1	0,00	0,00	0,00
OR10J3	0,00	0,00	0,00
GRM7	0,00	0,00	0,00
BECN2	0,00	0,00	0,00
CGB7	0,00	0,00	0,00
OR8D1	0,00	0,00	0,00
ADH7	0,00	0,00	0,00
ELAVL3	0,00	0,00	0,00
SPANXD	0,00	0,00	0,00
PRTN3	0,00	0,00	0,00
NBPF4	0,00	0,00	0,00
CRYBA4	0,00	0,00	0,00
ASMT	0,00	0,00	0,00
GK2	0,00	0,00	0,00
OR2T3	0,00	0,00	0,00
SPTSSB	0,00	0,00	0,00
CCDC196	0,00	0,00	0,00
HBG2	0,00	0,00	0,00
LAMA2	0,00	0,00	0,00
PFN3	0,00	0,00	0,00
OR5AC2	0,00	0,00	0,00
MBD3L2B	0,00	0,00	0,00
SLC22A25	0,00	0,00	0,00
POTEF	0,00	0,00	0,00
MMP1	0,00	0,00	0,00
UGT2B15	0,00	0,00	0,00
ZNF502	0,00	0,00	0,00
SLC30A10	0,00	0,00	0,00
ALKAL1	0,00	0,00	0,00
LCE1B	0,00	0,00	0,00
POU3F4	0,00	0,00	0,00
OR14A16	0,00	0,00	0,00
OR52K1	0,00	0,00	0,00
SPINK14	0,00	0,00	0,00
CHRNG	0,00	0,00	0,00
OR11G2	0,00	0,00	0,00
POTEI	0,00	0,00	0,00
KRT39	0,00	0,00	0,00
H2AC7	0,00	0,00	0,00
HCAR1	0,00	0,00	0,00
TMEM26	0,00	0,00	0,00
DTHD1	0,00	0,00	0,00
LCE1C	0,00	0,00	0,00
IFNL3	0,00	0,00	0,00
OR8B8	0,00	0,00	0,00
MAGEA6	0,00	0,00	0,00
ADGRA1	0,00	0,00	0,00
OR1J2	0,00	0,00	0,00
TPSB2	0,00	0,00	0,00
C6orf141	0,00	0,00	0,00

**IL27**	0,00	0,00	0,00
**GUCA2A**	0,00	0,00	0,00
**OR10D3**	0,00	0,00	0,00
**LYPD2**	0,00	0,00	0,00
**S100A7L2**	0,00	0,00	0,00
**OR6N1**	0,00	0,00	0,00
**CYP2B6**	0,00	0,00	0,00
**H3C4**	0,00	0,00	0,00
**FABP12**	0,00	0,00	0,00
**OR51D1**	0,00	0,00	0,00
**OR13G1**	0,00	0,00	0,00
**CYP2F1**	0,00	0,00	0,00
**OR2L5**	0,00	0,00	0,00
**GALP**	0,00	0,00	0,00
**SLC28A3**	0,00	0,00	0,00
**OR6Y1**	0,00	0,00	0,00
**ELANE**	0,00	0,00	0,00
**COL4A6**	0,00	0,00	0,00
**DMBX1**	0,00	0,00	0,00
**OR11L1**	0,00	0,00	0,00
**MFAP5**	0,00	0,00	0,00
**SERPINB2**	0,00	0,00	0,00
**SERPINB13**	0,00	0,00	0,00
**CCER1**	0,00	0,00	0,00
**SLC22A24**	0,00	0,00	0,00
**OR51C1P**	0,00	0,00	0,00
**KRTAP26-1**	0,00	0,00	0,00
**CR1L**	0,00	0,00	0,00
**SCGB1D4**	0,00	0,00	0,00
**HOXC6**	0,00	0,00	0,00
**MAP1LC3C**	0,00	0,00	0,00
**OR5B17**	0,00	0,00	0,00
**OR52M1**	0,00	0,00	0,00
**CCDC180**	0,00	0,00	0,00
**CYP2A13**	0,00	0,00	0,00
**OR8G1**	0,00	0,00	0,00
**PRB3**	0,00	0,00	0,00
**OR1S2**	0,00	0,00	0,00
**SLC22A12**	0,00	0,00	0,00
**SLC22A6**	0,00	0,00	0,00
**IFNA1**	0,00	0,00	0,00
**HES5**	0,00	0,00	0,00
**AADACL2**	0,00	0,00	0,00
**AL592490.1**	0,00	0,00	0,00
**CLEC9A**	0,00	0,00	0,00
**SPANXA1**	0,00	0,00	0,00
**TUBA3C**	0,00	0,00	0,00
**POTEH**	0,00	0,00	0,00
**AKR1B10**	0,00	0,00	0,00
**SULT1C4**	0,00	0,00	0,00
**KRTAP9-9**	0,00	0,00	0,00
**KRTAP4-6**	0,00	0,00	0,00
**TMPRSS11F**	0,00	0,00	0,00
**ADH4**	0,00	0,00	0,00
**OR2T6**	0,00	0,00	0,00
**OR2L3**	0,00	0,00	0,00

DEFB107B	0,00	0,00	0,00
TMEM229B	0,00	0,00	0,00
FAM47C	0,00	0,00	0,00
CLEC4C	0,00	0,00	0,00
BPIFA1	0,00	0,00	0,00
ZNF334	0,00	0,00	0,00
SULT1C2	0,00	0,00	0,00
AC092143.1	0,00	0,00	0,00
CSF2RA	0,00	0,00	0,00
KRTAP4-5	0,00	0,00	0,00
TMEM239	0,00	0,00	0,00
HOXC4	0,00	0,00	0,00
DCAF12L2	0,00	0,00	0,00
KRTAP13-1	0,00	0,00	0,00
TMEM207	0,00	0,00	0,00
OR14L1P	0,00	0,00	0,00
SH3BGRL2	0,00	0,00	0,00
B3GNT6	0,00	0,00	0,00
C2CD4A	0,00	0,00	0,00
ITGBL1	0,00	0,00	0,00
RD3	0,00	0,00	0,00
SPANXC	0,00	0,00	0,00
SH2D1B	0,00	0,00	0,00
ZNF536	0,00	0,00	0,00
OR2M2	0,00	0,00	0,00
AKR1C4	0,00	0,00	0,00
OR10G6	0,00	0,00	0,00
OR5BS1P	0,00	0,00	0,00
MAGEA1	0,00	0,00	0,00
LRRTM3	0,00	0,00	0,00
EPS8L3	0,00	0,00	0,00
EGFL6	0,00	0,00	0,00
SYCP1	0,00	0,00	0,00
APCDD1L	0,00	0,00	0,00
RASSF9	0,00	0,00	0,00
ALPK2	0,00	0,00	0,00
MAGEB3	0,00	0,00	0,00
LRRC10	0,00	0,00	0,00
GRM3	0,00	0,00	0,00
SUCNR1	0,00	0,00	0,00
STYXL2	0,00	0,00	0,00
CES1	0,00	0,00	0,00
CD3E	0,00	0,00	0,00
C1orf68	0,00	0,00	0,00
PNMA5	0,00	0,00	0,00
DCAF12L1	0,00	0,00	0,00
POU3F3	0,00	0,00	0,00
CSAG1	0,00	0,00	0,00
SOWAHA	0,00	0,00	0,00
TGM2	0,00	0,00	0,00
OR10R2	0,00	0,00	0,00
GP1BB	0,00	0,00	0,00
OR2T5	0,00	0,00	0,00
GJE1	0,00	0,00	0,00
METTL11B	0,00	0,00	0,00
FCGR3A	0,00	0,00	0,00

**TMEM244**	0,00	0,00	0,00
**LORICRIN**	0,00	0,00	0,00
**PRR9**	0,00	0,00	0,00
**LELP1**	0,00	0,00	0,00
**SPRR2E**	0,00	0,00	0,00
**KPRP**	0,00	0,00	0,00
**FAM24A**	0,00	0,00	0,00
**PLPP4**	0,00	0,00	0,00
**H2BC18**	0,00	0,00	0,00
**PNLIPRP3**	0,00	0,00	0,00
**HSD3B1**	0,00	0,00	0,00
**HSD3B2**	0,00	0,00	0,00
**RIPPLY2**	0,00	0,00	0,00
**DPPA5**	0,00	0,00	0,00
**SPANXN1**	0,00	0,00	0,00
**SPANXA2**	0,00	0,00	0,00
**CXorf66**	0,00	0,00	0,00
**C10orf62**	0,00	0,00	0,00
**CCDC160**	0,00	0,00	0,00
**C1orf141**	0,00	0,00	0,00
**DEFB110**	0,00	0,00	0,00
**GLYATL3**	0,00	0,00	0,00
**LDLRAD1**	0,00	0,00	0,00
**RHOXF2B**	0,00	0,00	0,00
**C1orf185**	0,00	0,00	0,00
**GLT6D1**	0,00	0,00	0,00
**IFIT1B**	0,00	0,00	0,00
**CT83**	0,00	0,00	0,00
**LIPN**	0,00	0,00	0,00
**LIPJ**	0,00	0,00	0,00
**LRIT2**	0,00	0,00	0,00
**TCEAL5**	0,00	0,00	0,00
**C2orf72**	0,00	0,00	0,00
**CLPSL1**	0,00	0,00	0,00
**NPY4R**	0,00	0,00	0,00
**TXNDC8**	0,00	0,00	0,00
**AWAT1**	0,00	0,00	0,00
**OR13C3**	0,00	0,00	0,00
**TMEM235**	0,00	0,00	0,00
**PAGE3**	0,00	0,00	0,00
**TSBP1**	0,00	0,00	0,00
**TMEM225**	0,00	0,00	0,00
**SP5**	0,00	0,00	0,00
**CD300LD**	0,00	0,00	0,00
**BTBD17**	0,00	0,00	0,00
**SPANXN5**	0,00	0,00	0,00
**XAGE1A**	0,00	0,00	0,00
**LAYN**	0,00	0,00	0,00
**XAGE1B**	0,00	0,00	0,00
**CASP12**	0,00	0,00	0,00
**CSHL1**	0,00	0,00	0,00
**LY6G6F**	0,00	0,00	0,00
**TRIM49C**	0,00	0,00	0,00
**TRIM64**	0,00	0,00	0,00
**PRAMEF20**	0,00	0,00	0,00
**PRAMEF17**	0,00	0,00	0,00

**PRAMEF19**	0,00	0,00	0,00
**PRAMEF14**	0,00	0,00	0,00
**PRAMEF15**	0,00	0,00	0,00
**PRAMEF9**	0,00	0,00	0,00
**PRAMEF7**	0,00	0,00	0,00
**AADACL4**	0,00	0,00	0,00
**ZSCAN5C**	0,00	0,00	0,00
**PSORS1C2**	0,00	0,00	0,00
**CDSN**	0,00	0,00	0,00
**C6orf15**	0,00	0,00	0,00
**MUC21**	0,00	0,00	0,00
**DEFB121**	0,00	0,00	0,00
**KRTAP5-11**	0,00	0,00	0,00
**DPRX**	0,00	0,00	0,00
**TRIM15**	0,00	0,00	0,00
**FOXB2**	0,00	0,00	0,00
**TRIM10**	0,00	0,00	0,00
**TRIM40**	0,00	0,00	0,00
**NMS**	0,00	0,00	0,00
**ZFP57**	0,00	0,00	0,00
**C9orf57**	0,00	0,00	0,00
**IL31**	0,00	0,00	0,00
**MAS1L**	0,00	0,00	0,00
**OR11A1**	0,00	0,00	0,00
**OR14J1**	0,00	0,00	0,00
**OR2J2**	0,00	0,00	0,00
**OR2J1**	0,00	0,00	0,00
**OR2B3**	0,00	0,00	0,00
**SPDYC**	0,00	0,00	0,00
**C9orf135**	0,00	0,00	0,00
**RANBP17**	0,00	0,00	0,00
**SPATA31A1**	0,00	0,00	0,00
**IGFL2**	0,00	0,00	0,00
**IGFL4**	0,00	0,00	0,00
**KRTAP9-3**	0,00	0,00	0,00
**KRTAP4-8**	0,00	0,00	0,00
**GPR20**	0,00	0,00	0,00
**KRT40**	0,00	0,00	0,00
**KRT25**	0,00	0,00	0,00
**PRR20A**	0,00	0,00	0,00
**GRXCR2**	0,00	0,00	0,00
**CD177**	0,00	0,00	0,00
**PSG5**	0,00	0,00	0,00
**FBXO47**	0,00	0,00	0,00
**PCDHA9**	0,00	0,00	0,00
**PCDHA8**	0,00	0,00	0,00
**PCDHA7**	0,00	0,00	0,00
**PCDHA5**	0,00	0,00	0,00
**PCDHA2**	0,00	0,00	0,00
**MS4A13**	0,00	0,00	0,00
**PRSS1**	0,00	0,00	0,00
**AARD**	0,00	0,00	0,00
**OR5D16**	0,00	0,00	0,00
**OR5L2**	0,00	0,00	0,00
**PKHD1L1**	0,00	0,00	0,00
**LGALS7**	0,00	0,00	0,00

**FRG2**	0,00	0,00	0,00
**FAM205A**	0,00	0,00	0,00
**ACCSL**	0,00	0,00	0,00
**C11orf91**	0,00	0,00	0,00
**FABP9**	0,00	0,00	0,00
**SCGB2B2**	0,00	0,00	0,00
**VIT**	0,00	0,00	0,00
**TRBV20OR9-2**	0,00	0,00	0,00
**CTXN3**	0,00	0,00	0,00
**MGAT4D**	0,00	0,00	0,00
**OR6C68**	0,00	0,00	0,00
**OR6C3**	0,00	0,00	0,00
**OR6C1**	0,00	0,00	0,00
**SLCO6A1**	0,00	0,00	0,00
**MT1A**	0,00	0,00	0,00
**MT1M**	0,00	0,00	0,00
**OR52E6**	0,00	0,00	0,00
**KRT6A**	0,00	0,00	0,00
**KRT81**	0,00	0,00	0,00
**EXOC3L4**	0,00	0,00	0,00
**KRTAP12-3**	0,00	0,00	0,00
**IZUMO3**	0,00	0,00	0,00
**TP53TG3D**	0,00	0,00	0,00
**TP53TG3C**	0,00	0,00	0,00
**OR52J3**	0,00	0,00	0,00
**OR51A2**	0,00	0,00	0,00
**OR51A4**	0,00	0,00	0,00
**C2CD4B**	0,00	0,00	0,00
**RGL3**	0,00	0,00	0,00
**VCX3B**	0,00	0,00	0,00
**HTN3**	0,00	0,00	0,00
**ARSH**	0,00	0,00	0,00
**TECRL**	0,00	0,00	0,00
**DPF3**	0,00	0,00	0,00
**GAGE1**	0,00	0,00	0,00
**KLRC2**	0,00	0,00	0,00
**KLRC3**	0,00	0,00	0,00
**GMNC**	0,00	0,00	0,00
**CLEC6A**	0,00	0,00	0,00
**NANOGNB**	0,00	0,00	0,00
**LRRC72**	0,00	0,00	0,00
**KRTAP5-6**	0,00	0,00	0,00
**KRTAP5-1**	0,00	0,00	0,00
**DEFB134**	0,00	0,00	0,00
**DEFB136**	0,00	0,00	0,00
**BHLHA9**	0,00	0,00	0,00
**DAZ4**	0,00	0,00	0,00
**CEMP1**	0,00	0,00	0,00
**OLIG2**	0,00	0,00	0,00
**DAZ2**	0,00	0,00	0,00
**SMIM21**	0,00	0,00	0,00
**DEFA1**	0,00	0,00	0,00
**TMEM211**	0,00	0,00	0,00
**SERPINB11**	0,00	0,00	0,00
**SERPINB4**	0,00	0,00	0,00
**SERPINB5**	0,00	0,00	0,00

KRTAP19-8	0,00	0,00	0,00
KRTAP20-3	0,00	0,00	0,00
KRTAP20-4	0,00	0,00	0,00
KRTAP22-2	0,00	0,00	0,00
KRTAP27-1	0,00	0,00	0,00
HBA1	0,00	0,00	0,00
HBM	0,00	0,00	0,00
ANKUB1	0,00	0,00	0,00
TSSK2	0,00	0,00	0,00
PRR23A	0,00	0,00	0,00
FOXL2NB	0,00	0,00	0,00
TMEM200C	0,00	0,00	0,00
OR10C1	0,00	0,00	0,00
CD200R1L	0,00	0,00	0,00
OR5K3	0,00	0,00	0,00
IGKJ5	0,00	0,00	0,00
IGKJ4	0,00	0,00	0,00
IGKJ3	0,00	0,00	0,00
IGKJ2	0,00	0,00	0,00
IGKJ1	0,00	0,00	0,00
IGKV4-1	0,00	0,00	0,00
IGKV5-2	0,00	0,00	0,00
IGKV2D-26	0,00	0,00	0,00
IGKV3D-20	0,00	0,00	0,00
IGKV6D-41	0,00	0,00	0,00
IGKV3D-11	0,00	0,00	0,00
IGKV1D-42	0,00	0,00	0,00
IGLV4-69	0,00	0,00	0,00
IGLV8-61	0,00	0,00	0,00
IGLV6-57	0,00	0,00	0,00
IGLV11-55	0,00	0,00	0,00
IGLV10-54	0,00	0,00	0,00
IGLV5-48	0,00	0,00	0,00
IGLV1-44	0,00	0,00	0,00
IGLV7-43	0,00	0,00	0,00
IGLV5-37	0,00	0,00	0,00
IGLV1-36	0,00	0,00	0,00
IGLV2-33	0,00	0,00	0,00
IGLV3-32	0,00	0,00	0,00
IGLV3-27	0,00	0,00	0,00
IGLV3-25	0,00	0,00	0,00
IGLV2-23	0,00	0,00	0,00
IGLV3-22	0,00	0,00	0,00
IGLV3-19	0,00	0,00	0,00
IGLV2-18	0,00	0,00	0,00
IGLV3-12	0,00	0,00	0,00
IGLV2-11	0,00	0,00	0,00
IGLV3-10	0,00	0,00	0,00
IGLJ1	0,00	0,00	0,00
IGLJ3	0,00	0,00	0,00
IGLJ4	0,00	0,00	0,00
IGLJ5	0,00	0,00	0,00
IGLJ6	0,00	0,00	0,00
IGLJ7	0,00	0,00	0,00
TRGJ2	0,00	0,00	0,00
TRGJP2	0,00	0,00	0,00

**TRGC1**	0,00	0,00	0,00
**TRGJP**	0,00	0,00	0,00
**TRGJP1**	0,00	0,00	0,00
**TRGV11**	0,00	0,00	0,00
**TRBV6-1**	0,00	0,00	0,00
**TRBV7-1**	0,00	0,00	0,00
**TRBV6-4**	0,00	0,00	0,00
**TRBV7-3**	0,00	0,00	0,00
**TRBV5-3**	0,00	0,00	0,00
**TRBV9**	0,00	0,00	0,00
**TRBV10-1**	0,00	0,00	0,00
**TRBV11-1**	0,00	0,00	0,00
**TRBV6-5**	0,00	0,00	0,00
**TRBV6-6**	0,00	0,00	0,00
**TRBV5-5**	0,00	0,00	0,00
**TRBV7-6**	0,00	0,00	0,00
**TRBV5-6**	0,00	0,00	0,00
**TRBV5-7**	0,00	0,00	0,00
**TRBV5-1**	0,00	0,00	0,00
**TRBV4-2**	0,00	0,00	0,00
**TRBV19**	0,00	0,00	0,00
**TRBV23-1**	0,00	0,00	0,00
**TRBV24-1**	0,00	0,00	0,00
**TRBC1**	0,00	0,00	0,00
**TRBV27**	0,00	0,00	0,00
**TRBV28**	0,00	0,00	0,00
**TRBJ2-1**	0,00	0,00	0,00
**TRBJ2-2**	0,00	0,00	0,00
**TRBJ2-2P**	0,00	0,00	0,00
**TRBJ2-3**	0,00	0,00	0,00
**TRBJ2-4**	0,00	0,00	0,00
**TRBJ2-5**	0,00	0,00	0,00
**TRBJ2-6**	0,00	0,00	0,00
**TRBJ2-7**	0,00	0,00	0,00
**TRBC2**	0,00	0,00	0,00
**TRAV2**	0,00	0,00	0,00
**TRAV3**	0,00	0,00	0,00
**TRAV4**	0,00	0,00	0,00
**TRAV5**	0,00	0,00	0,00
**TRAV6**	0,00	0,00	0,00
**TRAV7**	0,00	0,00	0,00
**TRAV8-1**	0,00	0,00	0,00
**TRAV9-1**	0,00	0,00	0,00
**TRAV10**	0,00	0,00	0,00
**TRAV12-1**	0,00	0,00	0,00
**TRAV8-2**	0,00	0,00	0,00
**TRAV8-3**	0,00	0,00	0,00
**TRAV13-1**	0,00	0,00	0,00
**TRAV12-2**	0,00	0,00	0,00
**TRAV8-4**	0,00	0,00	0,00
**TRAV13-2**	0,00	0,00	0,00
**TRAV9-2**	0,00	0,00	0,00
**TRAV12-3**	0,00	0,00	0,00
**TRAV8-6**	0,00	0,00	0,00
**TRAV16**	0,00	0,00	0,00
**TRAV17**	0,00	0,00	0,00

**TRAV18**	0,00	0,00	0,00
**TRAV19**	0,00	0,00	0,00
**TRAV20**	0,00	0,00	0,00
**TRAV21**	0,00	0,00	0,00
**TRAV22**	0,00	0,00	0,00
**TRDV1**	0,00	0,00	0,00
**TRAV24**	0,00	0,00	0,00
**TRAV25**	0,00	0,00	0,00
**TRAV8-7**	0,00	0,00	0,00
**TRAV26-2**	0,00	0,00	0,00
**TRAV34**	0,00	0,00	0,00
**TRAV36DV7**	0,00	0,00	0,00
**TRAV38-1**	0,00	0,00	0,00
**TRAV38-2DV8**	0,00	0,00	0,00
**TRAV39**	0,00	0,00	0,00
**TRAV40**	0,00	0,00	0,00
**TRAV41**	0,00	0,00	0,00
**TRDJ1**	0,00	0,00	0,00
**TRDJ4**	0,00	0,00	0,00
**TRDJ2**	0,00	0,00	0,00
**TRDJ3**	0,00	0,00	0,00
**TRAJ61**	0,00	0,00	0,00
**TRAJ59**	0,00	0,00	0,00
**TRAJ57**	0,00	0,00	0,00
**TRAJ56**	0,00	0,00	0,00
**TRAJ54**	0,00	0,00	0,00
**TRAJ53**	0,00	0,00	0,00
**TRAJ52**	0,00	0,00	0,00
**TRAJ50**	0,00	0,00	0,00
**TRAJ49**	0,00	0,00	0,00
**TRAJ48**	0,00	0,00	0,00
**TRAJ47**	0,00	0,00	0,00
**TRAJ46**	0,00	0,00	0,00
**TRAJ45**	0,00	0,00	0,00
**TRAJ44**	0,00	0,00	0,00
**TRAJ43**	0,00	0,00	0,00
**TRAJ42**	0,00	0,00	0,00
**TRAJ41**	0,00	0,00	0,00
**TRAJ40**	0,00	0,00	0,00
**TRAJ39**	0,00	0,00	0,00
**TRAJ38**	0,00	0,00	0,00
**TRAJ35**	0,00	0,00	0,00
**TRAJ34**	0,00	0,00	0,00
**TRAJ33**	0,00	0,00	0,00
**TRAJ32**	0,00	0,00	0,00
**TRAJ31**	0,00	0,00	0,00
**TRAJ30**	0,00	0,00	0,00
**TRAJ29**	0,00	0,00	0,00
**TRAJ28**	0,00	0,00	0,00
**TRAJ27**	0,00	0,00	0,00
**TRAJ26**	0,00	0,00	0,00
**TRAJ25**	0,00	0,00	0,00
**TRAJ24**	0,00	0,00	0,00
**TRAJ23**	0,00	0,00	0,00
**TRAJ22**	0,00	0,00	0,00
**TRAJ21**	0,00	0,00	0,00

**TRAJ20**	0,00	0,00	0,00
**TRAJ19**	0,00	0,00	0,00
**TRAJ18**	0,00	0,00	0,00
**TRAJ17**	0,00	0,00	0,00
**TRAJ16**	0,00	0,00	0,00
**TRAJ14**	0,00	0,00	0,00
**TRAJ13**	0,00	0,00	0,00
**TRAJ12**	0,00	0,00	0,00
**TRAJ11**	0,00	0,00	0,00
**TRAJ10**	0,00	0,00	0,00
**TRAJ9**	0,00	0,00	0,00
**TRAJ7**	0,00	0,00	0,00
**TRAJ6**	0,00	0,00	0,00
**TRAJ5**	0,00	0,00	0,00
**TRAJ4**	0,00	0,00	0,00
**TRAJ3**	0,00	0,00	0,00
**TRAJ2**	0,00	0,00	0,00
**TRAJ1**	0,00	0,00	0,00
**IGHE**	0,00	0,00	0,00
**IGHJ6**	0,00	0,00	0,00
**IGHJ2**	0,00	0,00	0,00
**IGHJ1**	0,00	0,00	0,00
**IGHD1-26**	0,00	0,00	0,00
**IGHD5-24**	0,00	0,00	0,00
**IGHD6-19**	0,00	0,00	0,00
**IGHD5-18**	0,00	0,00	0,00
**IGHD6-13**	0,00	0,00	0,00
**IGHD5-12**	0,00	0,00	0,00
**IGHD3-10**	0,00	0,00	0,00
**IGHD3-9**	0,00	0,00	0,00
**IGHD3-3**	0,00	0,00	0,00
**IGHV6-1**	0,00	0,00	0,00
**IGHV1-2**	0,00	0,00	0,00
**IGHV2-5**	0,00	0,00	0,00
**IGHV3-7**	0,00	0,00	0,00
**IGHV3-11**	0,00	0,00	0,00
**IGHV3-16**	0,00	0,00	0,00
**IGHV1-18**	0,00	0,00	0,00
**IGHV3-21**	0,00	0,00	0,00
**IGHV2-26**	0,00	0,00	0,00
**IGHV3-35**	0,00	0,00	0,00
**IGHV1-45**	0,00	0,00	0,00
**IGHV1-46**	0,00	0,00	0,00
**IGHV3-48**	0,00	0,00	0,00
**IGHV3-49**	0,00	0,00	0,00
**IGHV1-69**	0,00	0,00	0,00
**TSSK1B**	0,00	0,00	0,00
**KRTAP29-1**	0,00	0,00	0,00
**KRTAP9-6**	0,00	0,00	0,00
**CTAGE1**	0,00	0,00	0,00
**KRTAP4-11**	0,00	0,00	0,00
**KRTAP4-9**	0,00	0,00	0,00
**KRTAP2-3**	0,00	0,00	0,00
**KRTAP2-1**	0,00	0,00	0,00
**KRTAP3-3**	0,00	0,00	0,00
**KRTAP3-2**	0,00	0,00	0,00

KRTAP3-1	0,00	0,00	0,00
KRTAP12-4	0,00	0,00	0,00
KRTAP10-3	0,00	0,00	0,00
KRTAP6-3	0,00	0,00	0,00
KLK9	0,00	0,00	0,00
ACKR1	0,00	0,00	0,00
OR2F1	0,00	0,00	0,00
MAGEA12	0,00	0,00	0,00
KRTAP4-12	0,00	0,00	0,00
KRTAP2-4	0,00	0,00	0,00
UGT2B11	0,00	0,00	0,00
CEACAM18	0,00	0,00	0,00
UBD	0,00	0,00	0,00
CEACAM16	0,00	0,00	0,00
LEUTX	0,00	0,00	0,00
CCL27	0,00	0,00	0,00
HBG1	0,00	0,00	0,00
CLDN9	0,00	0,00	0,00
TM6SF2	0,00	0,00	0,00
IQCJ	0,00	0,00	0,00
NPS	0,00	0,00	0,00
LCNL1	0,00	0,00	0,00
TRIM77	0,00	0,00	0,00
GNAT3	0,00	0,00	0,00
KRTAP2-2	0,00	0,00	0,00
DEFB113	0,00	0,00	0,00
ZNF727	0,00	0,00	0,00
B3GNT10	0,00	0,00	0,00
IQCF5	0,00	0,00	0,00
IQCF6	0,00	0,00	0,00
C10orf105	0,00	0,00	0,00
MS4A18	0,00	0,00	0,00
CDRT15L2	0,00	0,00	0,00
EVPLL	0,00	0,00	0,00
TRIM64C	0,00	0,00	0,00
PNMA6E	0,00	0,00	0,00
SPATA31D1	0,00	0,00	0,00
GPR33	0,00	0,00	0,00
TBC1D26	0,00	0,00	0,00
TCP11X2	0,00	0,00	0,00
PRSS41	0,00	0,00	0,00
MUC5AC	0,00	0,00	0,00
GRXCR1	0,00	0,00	0,00
C5orf49	0,00	0,00	0,00
KCNU1	0,00	0,00	0,00
RNF212B	0,00	0,00	0,00
ZNF705B	0,00	0,00	0,00
KRTAP10-1	0,00	0,00	0,00
SKOR2	0,00	0,00	0,00
EFCAB8	0,00	0,00	0,00
DEFB116	0,00	0,00	0,00
DEFB115	0,00	0,00	0,00
GAB4	0,00	0,00	0,00
HMX1	0,00	0,00	0,00
CELA2B	0,00	0,00	0,00
LACTBL1	0,00	0,00	0,00

**C1orf167**	0,00	0,00	0,00
**IGSF23**	0,00	0,00	0,00
**SP9**	0,00	0,00	0,00
**SYCE3**	0,00	0,00	0,00
**TDRD15**	0,00	0,00	0,00
**TRIM51GP**	0,00	0,00	0,00
**OR6B1**	0,00	0,00	0,00
**OR2A5**	0,00	0,00	0,00
**KRTAP10-9**	0,00	0,00	0,00
**OR4A5**	0,00	0,00	0,00
**KRTAP1-5**	0,00	0,00	0,00
**TAS2R41**	0,00	0,00	0,00
**OR2A12**	0,00	0,00	0,00
**KRTAP10-10**	0,00	0,00	0,00
**KRTAP12-2**	0,00	0,00	0,00
**MAGEA3**	0,00	0,00	0,00
**PSG7**	0,00	0,00	0,00
**KRTAP1-3**	0,00	0,00	0,00
**OR3A2**	0,00	0,00	0,00
**HMSD**	0,00	0,00	0,00
**OR1C1**	0,00	0,00	0,00
**POM121L12**	0,00	0,00	0,00
**HEPN1**	0,00	0,00	0,00
**OR2A25**	0,00	0,00	0,00
**TAS2R40**	0,00	0,00	0,00
**OR2A14**	0,00	0,00	0,00
**OR4C12**	0,00	0,00	0,00
**OR4E2**	0,00	0,00	0,00
**OR52B4**	0,00	0,00	0,00
**PSMB11**	0,00	0,00	0,00
**POTEJ**	0,00	0,00	0,00
**IGLV9-49**	0,00	0,00	0,00
**TRIM49D1**	0,00	0,00	0,00
**USP17L15**	0,00	0,00	0,00
**CKMT1A**	0,00	0,00	0,00
**HBD**	0,00	0,00	0,00
**ZNF735**	0,00	0,00	0,00
**IGHV3-64**	0,00	0,00	0,00
**C1GALT1C1L**	0,00	0,00	0,00
**SUPT20HL1**	0,00	0,00	0,00
**TRDD1**	0,00	0,00	0,00
**IGKV3D-15**	0,00	0,00	0,00
**CT47A10**	0,00	0,00	0,00
**ETDB**	0,00	0,00	0,00
**CENPVL3**	0,00	0,00	0,00
**GPX5**	0,00	0,00	0,00
**PPP4R3C**	0,00	0,00	0,00
**TMEM233**	0,00	0,00	0,00
**PNMA6F**	0,00	0,00	0,00
**USP17L3**	0,00	0,00	0,00
**TDGF1P3**	0,00	0,00	0,00
**FAM236D**	0,00	0,00	0,00
**IGKV6D-21**	0,00	0,00	0,00
**Z82206.1**	0,00	0,00	0,00
**C2CD4D**	0,00	0,00	0,00
**DEFB131B**	0,00	0,00	0,00

IGHD6-25	0,00	0,00	0,00
NTF4	0,00	0,00	0,00
CT47A6	0,00	0,00	0,00
CROCC2	0,00	0,00	0,00
DCAF8L1	0,00	0,00	0,00
AC138647.1	0,00	0,00	0,00
TRBV2	0,00	0,00	0,00
CT47A12	0,00	0,00	0,00
AC013470.2	0,00	0,00	0,00
CT47A11	0,00	0,00	0,00
RBMY1J	0,00	0,00	0,00
ANHX	0,00	0,00	0,00
IGHD1-14	0,00	0,00	0,00
USP17L5	0,00	0,00	0,00
TRGC2	0,00	0,00	0,00
SPANXB1	0,00	0,00	0,00
AKR1B15	0,00	0,00	0,00
USP17L12	0,00	0,00	0,00
RD3L	0,00	0,00	0,00
TEX46	0,00	0,00	0,00
MRLN	0,00	0,00	0,00
BOD1L2	0,00	0,00	0,00
IGHD6-6	0,00	0,00	0,00
IGKV3D-7	0,00	0,00	0,00
CT47A7	0,00	0,00	0,00
VN1R4	0,00	0,00	0,00
CLDN25	0,00	0,00	0,00
CT45A5	0,00	0,00	0,00
TSPY3	0,00	0,00	0,00
TRDD3	0,00	0,00	0,00
PGA4	0,00	0,00	0,00
RFPL4AL1	0,00	0,00	0,00
NKX1-2	0,00	0,00	0,00
TSPY8	0,00	0,00	0,00
PRAMEF25	0,00	0,00	0,00
PRAC2	0,00	0,00	0,00
H2AL1RP	0,00	0,00	0,00
TRBV10-2	0,00	0,00	0,00
PRPS1L1	0,00	0,00	0,00
IQCF3	0,00	0,00	0,00
POTEB2	0,00	0,00	0,00
ANKRD66	0,00	0,00	0,00
TRBV5-4	0,00	0,00	0,00
OR4F3	0,00	0,00	0,00
SSU72P8	0,00	0,00	0,00
OR5H6	0,00	0,00	0,00
ANKRD18B	0,00	0,00	0,00
MBD3L2	0,00	0,00	0,00
PRB4	0,00	0,00	0,00
STMND1	0,00	0,00	0,00
KRTAP21-3	0,00	0,00	0,00
OR5H1	0,00	0,00	0,00
PLSCR5	0,00	0,00	0,00
USP17L10	0,00	0,00	0,00
AKAIN1	0,00	0,00	0,00
PSG1	0,00	0,00	0,00

**PPP1R2B**	0,00	0,00	0,00
**MAGEB6B**	0,00	0,00	0,00
**TEX50**	0,00	0,00	0,00
**DYTN**	0,00	0,00	0,00
**IGHV3-43**	0,00	0,00	0,00
**TMEM114**	0,00	0,00	0,00
**KRTAP25-1**	0,00	0,00	0,00
**USP17L24**	0,00	0,00	0,00
**OR52I1**	0,00	0,00	0,00
**OR5K1**	0,00	0,00	0,00
**USP17L13**	0,00	0,00	0,00
**PRAMEF6**	0,00	0,00	0,00
**TRBV29-1**	0,00	0,00	0,00
**DEFB130B**	0,00	0,00	0,00
**USP17L11**	0,00	0,00	0,00
**OR5H15**	0,00	0,00	0,00
**TWIST2**	0,00	0,00	0,00
**PIRT**	0,00	0,00	0,00
**PRR23C**	0,00	0,00	0,00
**IGHV3OR16-10**	0,00	0,00	0,00
**TRIM49D2**	0,00	0,00	0,00
**TSPY4**	0,00	0,00	0,00
**IFNA13**	0,00	0,00	0,00
**POTEB**	0,00	0,00	0,00
**IGKV3OR2-268**	0,00	0,00	0,00
**PAGE2**	0,00	0,00	0,00
**C16orf82**	0,00	0,00	0,00
**TMEM229A**	0,00	0,00	0,00
**H2BS1**	0,00	0,00	0,00
**RBMY1A1**	0,00	0,00	0,00
**CLDN34**	0,00	0,00	0,00
**OR10G8**	0,00	0,00	0,00
**C11orf94**	0,00	0,00	0,00
**IQCM**	0,00	0,00	0,00
**SHISA8**	0,00	0,00	0,00
**FAM237A**	0,00	0,00	0,00
**SMIM1**	0,00	0,00	0,00
**KDM4E**	0,00	0,00	0,00
**SPAAR**	0,00	0,00	0,00
**NKX1-1**	0,00	0,00	0,00
**RNF148**	0,00	0,00	0,00
**CXorf51B**	0,00	0,00	0,00
**LCE6A**	0,00	0,00	0,00
**OR5H14**	0,00	0,00	0,00
**USP17L4**	0,00	0,00	0,00
**IGHD1-1**	0,00	0,00	0,00
**CLEC2L**	0,00	0,00	0,00
**PPIAL4G**	0,00	0,00	0,00
**SLC35G4**	0,00	0,00	0,00
**TAS2R39**	0,00	0,00	0,00
**TSPY10**	0,00	0,00	0,00
**AL354761.1**	0,00	0,00	0,00
**IGHD7-27**	0,00	0,00	0,00
**GAGE12B**	0,00	0,00	0,00
**C3orf84**	0,00	0,00	0,00
**OR10G9**	0,00	0,00	0,00

IGHD1-20	0,00	0,00	0,00
USP17L8	0,00	0,00	0,00
TAAR9	0,00	0,00	0,00
C4orf51	0,00	0,00	0,00
IGHD1-7	0,00	0,00	0,00
TRDD2	0,00	0,00	0,00
MBD3L5	0,00	0,00	0,00
TRBV30	0,00	0,00	0,00
RNF223	0,00	0,00	0,00
PATE4	0,00	0,00	0,00
AL445989.1	0,00	0,00	0,00
PRSS56	0,00	0,00	0,00
OR7E24	0,00	0,00	0,00
TEX51	0,00	0,00	0,00
PRAMEF33	0,00	0,00	0,00
TRBV3-1	0,00	0,00	0,00
AMY1A	0,00	0,00	0,00
OR2I1P	0,00	0,00	0,00
TSPY9P	0,00	0,00	0,00
PAGE2B	0,00	0,00	0,00
PCDHA13	0,00	0,00	0,00
AC118470.1	0,00	0,00	0,00
MEIKIN	0,00	0,00	0,00
PRAMEF11	0,00	0,00	0,00
DEFA3	0,00	0,00	0,00
IGKV1-6	0,00	0,00	0,00
IGKV1-37	0,00	0,00	0,00
KRTAP9-2	0,00	0,00	0,00
IGKV1D-33	0,00	0,00	0,00
IGHJ4	0,00	0,00	0,00
STRIT1	0,00	0,00	0,00
IGKV1-17	0,00	0,00	0,00
KRTAP13-3	0,00	0,00	0,00
KRTAP9-1	0,00	0,00	0,00
L1TD1	0,00	0,00	0,00
C1QTNF9	0,00	0,00	0,00
KRBOX1	0,00	0,00	0,00
IGKV1-16	0,00	0,00	0,00
KRTAP4-7	0,00	0,00	0,00
UGT1A9	0,00	0,00	0,00
KRTAP10-5	0,00	0,00	0,00
OR14A2	0,00	0,00	0,00
ZNF722P	0,00	0,00	0,00
TDGF1	0,00	0,00	0,00
C3orf85	0,00	0,00	0,00
KRTAP4-16	0,00	0,00	0,00
IGKV1D-16	0,00	0,00	0,00
IGKV3-11	0,00	0,00	0,00
SSX2	0,00	0,00	0,00
IGKV2D-24	0,00	0,00	0,00
KRTAP9-4	0,00	0,00	0,00
KRTAP5-4	0,00	0,00	0,00
UGT1A1	0,00	0,00	0,00
AL035460.1	0,00	0,00	0,00
IGKV1-9	0,00	0,00	0,00
SPRR2A	0,00	0,00	0,00

**HOGA1**	0,00	0,00	0,00
**IGKV1-33**	0,00	0,00	0,00
**MDFIC2**	0,00	0,00	0,00
**PSG2**	0,00	0,00	0,00
**RBMY1E**	0,00	0,00	0,00
**IGHJ5**	0,00	0,00	0,00
**UGT1A10**	0,00	0,00	0,00
**IGKV2D-28**	0,00	0,00	0,00
**SERPINB10**	0,00	0,00	0,00
**CCDC169**	0,00	0,00	0,00
**IGKV1D-17**	0,00	0,00	0,00
**RBMY1B**	0,00	0,00	0,00
**IGHJ3**	0,00	0,00	0,00
**IGKV3-7**	0,00	0,00	0,00
**PRAMEF4**	0,00	0,00	0,00
**PSG11**	0,00	0,00	0,00
**PSG4**	0,00	0,00	0,00
**PCDHAC2**	0,00	0,00	0,00
**IGKV2D-29**	0,00	0,00	0,00
**VSIG8**	0,00	0,00	0,00
**IGKV1-12**	0,00	0,00	0,00
**IGKV1-5**	0,00	0,00	0,00
**KRTAP10-11**	0,00	0,00	0,00
**AL365232.1**	0,00	0,00	0,00
**WFDC6**	0,00	0,00	0,00
**SMIM34A**	0,00	0,00	0,00
**OR5V1**	0,00	0,00	0,00
**KIR2DL3**	0,00	0,00	0,00
**TUBA4B**	0,00	0,00	0,00
**GSTA1**	0,00	0,00	0,00
**RTL9**	0,00	0,00	0,00
**MT1HL1**	0,00	0,00	0,00
**KRTAP19-3**	0,00	0,00	0,00
**LCE3C**	0,00	0,00	0,00
**GSTA2**	0,00	0,00	0,00
**UGT1A7**	0,00	0,00	0,00
**GMCL2**	0,00	0,00	0,00
**AL645922.1**	0,00	0,00	0,00
**LY6G6D**	0,00	0,00	0,00
**KRTAP19-7**	0,00	0,00	0,00
**RBMY1D**	0,00	0,00	0,00
**CFHR1**	0,00	0,00	0,00
**IGKV3-15**	0,00	0,00	0,00
**UGT1A4**	0,00	0,00	0,00
**KRTAP4-2**	0,00	0,00	0,00
**RAD21L1**	0,00	0,00	0,00
**KRTAP20-1**	0,00	0,00	0,00
**HBB**	0,00	0,00	0,00
**USP51**	0,00	0,00	0,00
**ADH1C**	0,00	0,00	0,00
**AC037459.1**	0,00	0,00	0,00
**PCDHAC1**	0,00	0,00	0,00
**TARM1**	0,00	0,00	0,00
**FOXL3**	0,00	0,00	0,00
**SMIM31**	0,00	0,00	0,00
**TNFSF12-TNFSF13**	0,00	0,00	0,00

USP17L19	0,00	0,00	0,00
USP17L22	0,00	0,00	0,00
USP17L17	0,00	0,00	0,00
EPPIN-WFDC6	0,00	0,00	0,00
AL159163.1	0,00	0,00	0,00
TAF11L12	0,00	0,00	0,00
PCDHA11	0,00	0,00	0,00
CLRN2	0,00	0,00	0,00
LINC02218	0,00	0,00	0,00
USP17L21	0,00	0,00	0,00
MTRNR2L5	0,00	0,00	0,00
LGALS16	0,00	0,00	0,00
AL355315.1	0,00	0,00	0,00
TMEM158	0,00	0,00	0,00
IGKV1D-37	0,00	0,00	0,00
GIMD1	0,00	0,00	0,00
SMIM20	0,00	0,00	0,00
AF241726.1	0,00	0,00	0,00
KIAA1210	0,00	0,00	0,00
AC004691.2	0,00	0,00	0,00
LY6G6F-LY6G6D	0,00	0,00	0,00
CCDC169-SOHLH2	0,00	0,00	0,00
P3R3URF	0,00	0,00	0,00
SELENOP	0,00	0,00	0,00
NT5C1B-RDH14	0,00	0,00	0,00
USP17L20	0,00	0,00	0,00
TAF11L14	0,00	0,00	0,00
PRODH2	0,00	0,00	0,00
AC010255.2	0,00	0,00	0,00
EXOC1L	0,00	0,00	0,00
USP17L18	0,00	0,00	0,00
USP17L23	0,00	0,00	0,00
AC083800.1	0,00	0,00	0,00
IGKV2D-40	0,00	0,00	0,00
AC002456.2	0,00	0,00	0,00
AL672142.1	0,00	0,00	0,00
AL691442.1	0,00	0,00	0,00
DCANP1	0,00	0,00	0,00
FOXD1	0,00	0,00	0,00
PRB1	0,00	0,00	0,00
PCDHA12	0,00	0,00	0,00
OC90	0,00	0,00	0,00
RGS21	0,00	0,00	0,00
TRBV6-7	0,00	0,00	0,00
TRBV7-7	0,00	0,00	0,00
HOXA10	0,00	0,00	0,00
C1orf210	0,00	0,00	0,00
TRBV7-4	0,00	0,00	0,00
NACA2	0,00	0,00	0,00
TRBV6-8	0,00	0,00	0,00
PYDC2	0,00	0,00	0,00
UTP14C	0,00	0,00	0,00
ETV3L	0,00	0,00	0,00
PCDHGB2	0,00	0,00	0,00
CD8B2	0,00	0,00	0,00
PCDHGB1	0,00	0,00	0,00

**PBOV1**	0,00	0,00	0,00
**HSPB2-C11orf52**	0,00	0,00	0,00
**OR4D10**	0,00	0,00	0,00
**SIGLEC12**	0,00	0,00	0,00
**PABPC4L**	0,00	0,00	0,00
**OMP**	0,00	0,00	0,00
**ARMS2**	0,00	0,00	0,00
**INS**	0,00	0,00	0,00
**RTL1**	0,00	0,00	0,00
**AP001931.1**	0,00	0,00	0,00
**UBTFL1**	0,00	0,00	0,00
**OR5M1**	0,00	0,00	0,00
**AL020996.2**	0,00	0,00	0,00
**SAA2-SAA4**	0,00	0,00	0,00
**FXYD6-FXYD2**	0,00	0,00	0,00
**SMIM35**	0,00	0,00	0,00
**OR8G5**	0,00	0,00	0,00
**NOX5**	0,00	0,00	0,00
**CCDC179**	0,00	0,00	0,00
**OOSP4B**	0,00	0,00	0,00
**OR8G3P**	0,00	0,00	0,00
**AP001458.2**	0,00	0,00	0,00
**CARD18**	0,00	0,00	0,00
**LY6G6E**	0,00	0,00	0,00
**TRAV1-1**	0,00	0,00	0,00
**OR10G2**	0,00	0,00	0,00
**AC068775.1**	0,00	0,00	0,00
**OR4D2**	0,00	0,00	0,00
**OR6J1**	0,00	0,00	0,00
**KLRC4-KLRK1**	0,00	0,00	0,00
**AL117348.2**	0,00	0,00	0,00
**MTRNR2L10**	0,00	0,00	0,00
**ASIC5**	0,00	0,00	0,00
**SALL3**	0,00	0,00	0,00
**TRAV1-2**	0,00	0,00	0,00
**CLEC12B**	0,00	0,00	0,00
**LIMS4**	0,00	0,00	0,00
**STH**	0,00	0,00	0,00
**KLRF2**	0,00	0,00	0,00
**AC026786.1**	0,00	0,00	0,00
**AC048338.1**	0,00	0,00	0,00
**SLC5A8**	0,00	0,00	0,00
**MTRNR2L7**	0,00	0,00	0,00
**AC069503.2**	0,00	0,00	0,00
**GPR142**	0,00	0,00	0,00
**HP**	0,00	0,00	0,00
**SLCO1B3-SLCO1B7**	0,00	0,00	0,00
**AC022335.1**	0,00	0,00	0,00
**AL049844.1**	0,00	0,00	0,00
**TAS2R38**	0,00	0,00	0,00
**AC004080.3**	0,00	0,00	0,00
**AC034102.2**	0,00	0,00	0,00
**SFTA3**	0,00	0,00	0,00
**MGAM2**	0,00	0,00	0,00
**TEX49**	0,00	0,00	0,00
**AC073612.1**	0,00	0,00	0,00

**OR9A4**	0,00	0,00	0,00
**PRSS58**	0,00	0,00	0,00
**CLEC5A**	0,00	0,00	0,00
**AC100868.1**	0,00	0,00	0,00
**RNASE12**	0,00	0,00	0,00
**OR11H2**	0,00	0,00	0,00
**SPESP1**	0,00	0,00	0,00
**AP001781.2**	0,00	0,00	0,00
**TRIM6-TRIM34**	0,00	0,00	0,00
**AL355102.2**	0,00	0,00	0,00
**C20orf141**	0,00	0,00	0,00
**OR4C13**	0,00	0,00	0,00
**AC008575.1**	0,00	0,00	0,00
**DUXA**	0,00	0,00	0,00
**TMEM179**	0,00	0,00	0,00
**TSPY1**	0,00	0,00	0,00
**BLOC1S5-TXNDC5**	0,00	0,00	0,00
**AL163195.3**	0,00	0,00	0,00
**TRAV30**	0,00	0,00	0,00
**AC007375.2**	0,00	0,00	0,00
**IGHV4OR15-8**	0,00	0,00	0,00
**BUB1B-PAK6**	0,00	0,00	0,00
**IGHV2OR16-5**	0,00	0,00	0,00
**IGHV3OR15-7**	0,00	0,00	0,00
**BLID**	0,00	0,00	0,00
**IGHV3OR16-17**	0,00	0,00	0,00
**AC093525.1**	0,00	0,00	0,00
**AC026464.1**	0,00	0,00	0,00
**AL845331.1**	0,00	0,00	0,00
**HOXB7**	0,00	0,00	0,00
**AC090527.2**	0,00	0,00	0,00
**AC020636.2**	0,00	0,00	0,00
**AC093525.2**	0,00	0,00	0,00
**TBC1D3G**	0,00	0,00	0,00
**AC026464.3**	0,00	0,00	0,00
**AC012184.2**	0,00	0,00	0,00
**TLE7**	0,00	0,00	0,00
**AL049634.2**	0,00	0,00	0,00
**EPPK1**	0,00	0,00	0,00
**CLEC19A**	0,00	0,00	0,00
**MUC22**	0,00	0,00	0,00
**TP53TG3B**	0,00	0,00	0,00
**GOLGA8S**	0,00	0,00	0,00
**TCF24**	0,00	0,00	0,00
**SMIM36**	0,00	0,00	0,00
**PCDHGA9**	0,00	0,00	0,00
**GFY**	0,00	0,00	0,00
**MYMX**	0,00	0,00	0,00
**AC003688.1**	0,00	0,00	0,00
**MMP12**	0,00	0,00	0,00
**SMIM28**	0,00	0,00	0,00
**OR1D5**	0,00	0,00	0,00
**AC005670.2**	0,00	0,00	0,00
**AC104581.2**	0,00	0,00	0,00
**MYZAP**	0,00	0,00	0,00
**MSMB**	0,00	0,00	0,00

**GDF2**	0,00	0,00	0,00
**AKR1C8P**	0,00	0,00	0,00
**ANXA8L1**	0,00	0,00	0,00
**MYH4**	0,00	0,00	0,00
**NPY4R2**	0,00	0,00	0,00
**AC113554.1**	0,00	0,00	0,00
**ANXA8**	0,00	0,00	0,00
**AC004805.1**	0,00	0,00	0,00
**PNLIPRP2**	0,00	0,00	0,00
**AC005697.1**	0,00	0,00	0,00
**KLF14**	0,00	0,00	0,00
**GDF10**	0,00	0,00	0,00
**AC015688.4**	0,00	0,00	0,00
**IGBP1P2**	0,00	0,00	0,00
**AC090227.2**	0,00	0,00	0,00
**AC005837.2**	0,00	0,00	0,00
**AC245748.1**	0,00	0,00	0,00
**ATF7-NPFF**	0,00	0,00	0,00
**AC008687.1**	0,00	0,00	0,00
**AC011498.4**	0,00	0,00	0,00
**APOC4**	0,00	0,00	0,00
**AC008481.3**	0,00	0,00	0,00
**AC093227.2**	0,00	0,00	0,00
**CGB1**	0,00	0,00	0,00
**AC105052.3**	0,00	0,00	0,00
**AC020922.1**	0,00	0,00	0,00
**AC243967.1**	0,00	0,00	0,00
**AC008878.1**	0,00	0,00	0,00
**MAGEA9B**	0,00	0,00	0,00
**SSX4**	0,00	0,00	0,00
**GABRQ**	0,00	0,00	0,00
**AC003002.1**	0,00	0,00	0,00
**OPN1MW**	0,00	0,00	0,00
**ARL14EPL**	0,00	0,00	0,00
**TCP11X1**	0,00	0,00	0,00
**SCGB1C2**	0,00	0,00	0,00
**L34079.1**	0,00	0,00	0,00
**SSX2B**	0,00	0,00	0,00
**AC008403.1**	0,00	0,00	0,00
**AC018755.2**	0,00	0,00	0,00
**MAGEA2**	0,00	0,00	0,00
**AC008878.2**	0,00	0,00	0,00
**TEX13A**	0,00	0,00	0,00
**AC006486.1**	0,00	0,00	0,00
**CTAG1A**	0,00	0,00	0,00
**HSFX2**	0,00	0,00	0,00
**AC008537.1**	0,00	0,00	0,00
**H3Y2**	0,00	0,00	0,00
**CSAG2**	0,00	0,00	0,00
**CSAG3**	0,00	0,00	0,00
**CT45A1**	0,00	0,00	0,00
**ERVV-2**	0,00	0,00	0,00
**SPANXN2**	0,00	0,00	0,00
**FAM236B**	0,00	0,00	0,00
**AC003006.1**	0,00	0,00	0,00
**AC010319.2**	0,00	0,00	0,00

**CALR3**	0,00	0,00	0,00
**ZNF728**	0,00	0,00	0,00
**CT45A3**	0,00	0,00	0,00
**TRABD2B**	0,00	0,00	0,00
**AC011452.1**	0,00	0,00	0,00
**OPN1MW3**	0,00	0,00	0,00
**NXF2B**	0,00	0,00	0,00
**H3Y1**	0,00	0,00	0,00
**DMRTC1**	0,00	0,00	0,00
**AC011455.2**	0,00	0,00	0,00
**CT45A10**	0,00	0,00	0,00
**AC010422.5**	0,00	0,00	0,00
**AC010422.6**	0,00	0,00	0,00
**ZIM2**	0,00	0,00	0,00
**AC008763.3**	0,00	0,00	0,00
**AC011473.4**	0,00	0,00	0,00
**MEI4**	0,00	0,00	0,00
**AL365273.2**	0,00	0,00	0,00
**MTRNR2L11**	0,00	0,00	0,00
**AL121758.1**	0,00	0,00	0,00
**BORCS7-ASMT**	0,00	0,00	0,00
**MTRNR2L13**	0,00	0,00	0,00
**IGHV3OR16-12**	0,00	0,00	0,00
**IGHV3OR16-9**	0,00	0,00	0,00
**PRAMEF5**	0,00	0,00	0,00
**MTRNR2L6**	0,00	0,00	0,00
**CT45A9**	0,00	0,00	0,00
**IGHD5OR15-5A**	0,00	0,00	0,00
**CTAGE15**	0,00	0,00	0,00
**IGHV3OR16-8**	0,00	0,00	0,00
**IGHV3OR16-13**	0,00	0,00	0,00
**UGT2A2**	0,00	0,00	0,00
**IGHD1OR15-1A**	0,00	0,00	0,00
**CT45A2**	0,00	0,00	0,00
**AL589666.1**	0,00	0,00	0,00
**AL603832.3**	0,00	0,00	0,00
**AC093423.3**	0,00	0,00	0,00
**Z84492.1**	0,00	0,00	0,00
**AC018709.1**	0,00	0,00	0,00
**FAM47E-STBD1**	0,00	0,00	0,00
**DOC2B**	0,00	0,00	0,00
**AC005020.2**	0,00	0,00	0,00
**PCDHB16**	0,00	0,00	0,00
**KRTAP10-7**	0,00	0,00	0,00
**AC009690.3**	0,00	0,00	0,00
**AL121845.2**	0,00	0,00	0,00
**AC012531.3**	0,00	0,00	0,00
**GRIN2B**	0,00	0,00	0,00
**LYPD4**	0,00	0,00	0,00
**AC002094.3**	0,00	0,00	0,00
**AC008695.1**	0,00	0,00	0,00
**TMEM271**	0,00	0,00	0,00
**AL049839.2**	0,00	0,00	0,00
**AC092042.3**	0,00	0,00	0,00
**TM4SF19-DYNLT2B**	0,00	0,00	0,00
**AGBL1**	0,00	0,00	0,00

**CT45A7**	0,00	0,00	0,00
**LHX1**	0,00	0,00	0,00
**IGKV2-40**	0,00	0,00	0,00
**GOLGA6L1**	0,00	0,00	0,00
**GGTLC3**	0,00	0,00	0,00
**AL136531.2**	0,00	0,00	0,00
**TMEM269**	0,00	0,00	0,00
**TBC1D3D**	0,00	0,00	0,00
**DGKK**	0,00	0,00	0,00
**CCL23**	0,00	0,00	0,00
**KRTAP7-1**	0,00	0,00	0,00
**TRBV12-3**	0,00	0,00	0,00
**PRAMEF27**	0,00	0,00	0,00
**TP53TG3E**	0,00	0,00	0,00
**GAGE2E**	0,00	0,00	0,00
**CCL16**	0,00	0,00	0,00
**TRBV12-5**	0,00	0,00	0,00
**AC117457.1**	0,00	0,00	0,00
**TRBV16**	0,00	0,00	0,00
**CCL4**	0,00	0,00	0,00
**CCL18**	0,00	0,00	0,00
**HNF1B**	0,00	0,00	0,00
**C17orf98**	0,00	0,00	0,00
**FAM236A**	0,00	0,00	0,00
**H4C7**	0,00	0,00	0,00
**CCL15-CCL14**	0,00	0,00	0,00
**CCL15**	0,00	0,00	0,00
**LYZL6**	0,00	0,00	0,00
**TRBV14**	0,00	0,00	0,00
**TRBV10-3**	0,00	0,00	0,00
**PRSS2**	0,00	0,00	0,00
**TBC1D3F**	0,00	0,00	0,00
**SPATA31A3**	0,00	0,00	0,00
**SPATA31A7**	0,00	0,00	0,00
**CRYAA2**	0,00	0,00	0,00
**OR4E1**	0,00	0,00	0,00
**TRBV13**	0,00	0,00	0,00
**AL583836.1**	0,00	0,00	0,00
**PCDHGB5**	0,00	0,00	0,00
**TRBV18**	0,00	0,00	0,00
**IGKV1D-13**	0,00	0,00	0,00
**SPATA31A5**	0,00	0,00	0,00
**TRBV11-3**	0,00	0,00	0,00
**TRAJ36**	0,00	0,00	0,00
**PADI6**	0,00	0,00	0,00
**TRBV15**	0,00	0,00	0,00
**GSTT4**	0,00	0,00	0,00
**TRBV12-4**	0,00	0,00	0,00
**HNRNPCL3**	0,00	0,00	0,00
**IGHV1OR21-1**	0,00	0,00	0,00
**OR13C5**	0,00	0,00	0,00
**NEFL**	0,00	0,00	0,00
**Z98752.3**	0,00	0,00	0,00
**FO681492.1**	0,00	0,00	0,00
**GOLGA6L22**	0,00	0,00	0,00
**TRBV17**	0,00	0,00	0,00

**OR52E5**	0,00	0,00	0,00
**AC007731.4**	0,00	0,00	0,00
**TRBV7-9**	0,00	0,00	0,00
**TEX28**	0,00	0,00	0,00
**CT45A8**	0,00	0,00	0,00
**SSTR3**	0,00	0,00	0,00
**AC135068.1**	0,00	0,00	0,00
**CT45A6**	0,00	0,00	0,00
**ZNF229**	0,00	0,00	0,00
**AC008162.2**	0,00	0,00	0,00
**TRAJ37**	0,00	0,00	0,00
**IQCA1L**	0,00	0,00	0,00
**TP53TG3F**	0,00	0,00	0,00
**IGKV1D-12**	0,00	0,00	0,00
**OR51G1**	0,00	0,00	0,00
**OR2S2**	0,00	0,00	0,00
**OR10A6**	0,00	0,00	0,00
**AL807752.6**	0,00	0,00	0,00
**OR10X1**	0,00	0,00	0,00
**PRAMEF13**	0,00	0,00	0,00
**OR2L8**	0,00	0,00	0,00
**OR52R1**	0,00	0,00	0,00
**OR2T11**	0,00	0,00	0,00
**OR5L1**	0,00	0,00	0,00
**OR4N4C**	0,00	0,00	0,00
**OR2AG1**	0,00	0,00	0,00
**FP565260.4**	0,00	0,00	0,00
**OR4C16**	0,00	0,00	0,00
**AC142391.1**	0,00	0,00	0,00
**OR5D13**	0,00	0,00	0,00
**PRAMEF18**	0,00	0,00	0,00
**AC073082.1**	0,00	0,00	0,00
**OR8U3**	0,00	0,00	0,00
**OR1B1**	0,00	0,00	0,00
**AL049844.3**	0,00	0,00	0,00
**PCDH20**	0,00	0,00	0,00
**OR1S1**	0,00	0,00	0,00
**OR12D2**	0,00	0,00	0,00
**OR8K3**	0,00	0,00	0,00
**LINC02203**	0,00	0,00	0,00
**AC243547.3**	0,00	0,00	0,00
**RPS4Y2**	0,00	0,00	0,00
**AC135068.5**	0,00	0,00	0,00
**AL365214.2**	0,00	0,00	0,00
**AC034228.3**	0,00	0,00	0,00
**TRBJ1-4**	0,00	0,00	0,00
**IGHV1-69-2**	0,00	0,00	0,00
**TRBJ1-3**	0,00	0,00	0,00
**TRBJ1-5**	0,00	0,00	0,00
**AL132671.2**	0,00	0,00	0,00
**AC058822.1**	0,00	0,00	0,00
**TRBJ1-1**	0,00	0,00	0,00
**TEX13D**	0,00	0,00	0,00
**TRBJ1-2**	0,00	0,00	0,00
**TRBD1**	0,00	0,00	0,00
**TRBV25-1**	0,00	0,00	0,00

**IGHV3-64D**	0,00	0,00	0,00
**IGHV5-10-1**	0,00	0,00	0,00
**DUXB**	0,00	0,00	0,00
**TRBJ1-6**	0,00	0,00	0,00
**AL512428.1**	0,00	0,00	0,00
**TEX13C**	0,00	0,00	0,00
**C1orf232**	0,00	0,00	0,00
**TMEM275**	0,00	0,00	0,00
**KLF18**	0,00	0,00	0,00
**TRBV6-2**	0,00	0,00	0,00
**LBHD2**	0,00	0,00	0,00
**CENPVL2**	0,00	0,00	0,00
**AL353572.3**	0,00	0,00	0,00
**SPRR5**	0,00	0,00	0,00
**CCDC201**	0,00	0,00	0,00
**CTXND2**	0,00	0,00	0,00
**FAM240B**	0,00	0,00	0,00
**CNTNAP3C**	0,00	0,00	0,00
**CSNKA2IP**	0,00	0,00	0,00
**SPEM3**	0,00	0,00	0,00
**FAM240A**	0,00	0,00	0,00
**PRRT1B**	0,00	0,00	0,00
**AC021072.1**	0,00	0,00	0,00
**BX276092.9**	0,00	0,00	0,00
**ETDC**	0,00	0,00	0,00
**AC008687.8**	0,00	0,00	0,00
**MYOCOS**	0,00	0,00	0,00
**HSFX3**	0,00	0,00	0,00
**VSIG10L2**	0,00	0,00	0,00
**PRSS50**	0,00	0,00	0,00
**TAF11L11**	0,00	0,00	0,00
**CPHXL**	0,00	0,00	0,00
**PMIS2**	0,00	0,00	0,00
**TAF11L13**	0,00	0,00	0,00
**SSU72P4**	0,00	0,00	0,00
**AC104304.2**	0,00	0,00	0,00
**AC092017.2**	0,00	0,00	0,00
**TAF11L8**	0,00	0,00	0,00
**TAF11L9**	0,00	0,00	0,00
**SLURP2**	0,00	0,00	0,00
**SSU72P5**	0,00	0,00	0,00
**TAF11L6**	0,00	0,00	0,00
**TAF11L5**	0,00	0,00	0,00
**TAF11L4**	0,00	0,00	0,00
**SSU72P2**	0,00	0,00	0,00
**AL807752.7**	0,00	0,00	0,00
**TAF11L2**	0,00	0,00	0,00
**AC092111.3**	0,00	0,00	0,00
**SSU72P7**	0,00	0,00	0,00
**TAF11L3**	0,00	0,00	0,00
**TAF11L7**	0,00	0,00	0,00
**SMIM39**	0,00	0,00	0,00
**LYNX1-SLURP2**	0,00	0,00	0,00
**SSU72P3**	0,00	0,00	0,00
**OR8B3**	0,00	0,00	0,00
**SCYGR1**	0,00	0,00	0,00

**SCYGR4**	0,00	0,00	0,00
**SCYGR8**	0,00	0,00	0,00
**SCYGR2**	0,00	0,00	0,00
**SCYGR5**	0,00	0,00	0,00
**OR8B2**	0,00	0,00	0,00
**AC092442.1**	0,00	0,00	0,00
**CD300H**	0,00	0,00	0,00
**AC108941.2**	0,00	0,00	0,00
**TMEM247**	0,00	0,00	0,00
**SCYGR3**	0,00	0,00	0,00
**SMIM38**	0,00	0,00	0,00
**SCYGR7**	0,00	0,00	0,00
**OR8S1**	0,00	0,00	0,00
**SCYGR6**	0,00	0,00	0,00
**AP002512.3**	0,00	0,00	0,00
**PDE11A**	0,00	0,00	0,00
**AC132217.2**	0,00	0,00	0,00
**AC008397.1**	0,00	0,00	0,00
**AP000812.3**	0,00	0,00	0,00
**AC119676.1**	0,00	0,00	0,00
**EEF1AKMT4-ECE2**	0,00	0,00	0,00
**AC104389.6**	0,00	0,00	0,00
**AL049629.2**	0,00	0,00	0,00
**U52112.1**	0,00	0,00	0,00
**AL451062.2**	0,00	0,00	0,00
**OOSP4A**	0,00	0,00	0,00
**AL022312.1**	0,00	0,00	0,00
**AL662820.1**	0,00	0,00	0,00
**AC022506.1**	0,00	0,00	0,00
**AC008397.2**	0,00	0,00	0,00
**AC026316.4**	0,00	0,00	0,00
**AC025283.2**	0,00	0,00	0,00
**Z82190.2**	0,00	0,00	0,00
**AL162377.3**	0,00	0,00	0,00
**Z84488.1**	0,00	0,00	0,00
**AC007846.2**	0,00	0,00	0,00
**AC134980.2**	0,00	0,00	0,00
**AC010616.1**	0,00	0,00	0,00
**AC097104.1**	0,00	0,00	0,00
**AC013717.1**	0,00	0,00	0,00
**AC112504.2**	0,00	0,00	0,00
**AL445238.1**	0,00	0,00	0,00
**AC069444.2**	0,00	0,00	0,00
**FAM90A9P**	0,00	0,00	0,00
**FAM90A16P**	0,00	0,00	0,00
**AC021660.3**	0,00	0,00	0,00
**AC097636.1**	0,00	0,00	0,00
**AC253536.7**	0,00	0,00	0,00
**AC000120.2**	0,00	0,00	0,00
**AL358472.7**	0,00	0,00	0,00
**AL445685.3**	0,00	0,00	0,00
**AC144573.1**	0,00	0,00	0,00
**MSANTD5**	0,00	0,00	0,00
**FAM90A18P**	0,00	0,00	0,00
**AC087721.2**	0,00	0,00	0,00
**AL162596.1**	0,00	0,00	0,00

**FAM90A10P**	0,00	0,00	0,00
**AC113189.9**	0,00	0,00	0,00
**AC009412.1**	0,00	0,00	0,00
**FAM246A**	0,00	0,00	0,00
**AL353579.1**	0,00	0,00	0,00
**AC083977.1**	0,00	0,00	0,00
**AL603764.2**	0,00	0,00	0,00
**FAM246B**	0,00	0,00	0,00
**NOTCH2NLC**	0,00	0,00	0,00
**AP000471.1**	0,00	0,00	0,00
**SMIM40**	0,00	0,00	0,00
**GUCA1ANB**	0,00	0,00	0,00
**AC231656.1**	0,00	0,00	0,00
**AC106741.1**	0,00	0,00	0,00
**SMIM42**	0,00	0,00	0,00
**AL357075.4**	0,00	0,00	0,00
**AL035699.1**	0,00	0,00	0,00
**AL355385.2**	0,00	0,00	0,00
**AL359092.3**	0,00	0,00	0,00
**AC012254.6**	0,00	0,00	0,00
**AC010973.3**	0,00	0,00	0,00
**NPBWR1**	0,00	0,00	0,00
**AL031178.1**	0,00	0,00	0,00
**AC090373.2**	0,00	0,00	0,00
**AC063943.2**	0,00	0,00	0,00
**AC068870.4**	0,00	0,00	0,00
**AC242628.1**	0,00	0,00	0,00
**AC099569.1**	0,00	0,00	0,00
**AC012254.7**	0,00	0,00	0,00
**AL049569.3**	0,00	0,00	0,00
**AC114402.2**	0,00	0,00	0,00
**AC084756.2**	0,00	0,00	0,00
**AL031178.2**	0,00	0,00	0,00
**AC119733.1**	0,00	0,00	0,00
**AL451106.1**	0,00	0,00	0,00
**AC008763.4**	0,00	0,00	0,00
**AC006486.3**	0,00	0,00	0,00
**AC105206.4**	0,00	0,00	0,00
**AL136115.4**	0,00	0,00	0,00
**AC136475.9**	0,00	0,00	0,00
**SLC12A6**	11,43	11,43	0,00
**OXA1L**	12,65	12,65	0,00
**LHPP**	8,05	8,05	0,00
**SMO**	11,23	11,23	0,00
**C19orf54**	10,39	10,39	0,00
**PCBP2**	15,69	15,69	0,00
**MACROH2A1**	14,40	14,39	0,00
**CHRM5**	4,52	4,52	0,00
**PDS5B**	13,28	13,28	0,00
**ZNF286A**	12,09	12,09	0,00
**RWDD1**	12,30	12,30	0,00
**WDR20**	11,01	11,01	0,00
**ARMC8**	12,03	12,03	0,00
**TMEM159**	9,68	9,68	0,00
**ELL2**	8,64	8,64	0,00
**PCYOX1L**	10,93	10,93	0,00

**ZNF641**	10,35	10,35	0,00
**C19orf47**	9,90	9,90	0,00
**KCTD18**	10,78	10,78	0,00
**TVP23C**	5,65	5,65	0,00
**PSMC6**	13,02	13,02	0,00
**UBA7**	11,80	11,80	0,00
**NPM1**	17,43	17,43	0,00
**MED4**	12,59	12,59	0,00
**HNRNPU**	16,92	16,92	0,00
**EFEMP1**	10,06	10,06	0,00
**CKAP2**	13,58	13,58	0,00
**NAB1**	11,99	11,99	0,00
**ZNF589**	10,89	10,89	0,00
**SUPT16H**	15,20	15,20	0,00
**DAGLB**	12,53	12,53	0,00
**PLOD1**	11,01	11,01	0,00
**ARPP19**	13,96	13,96	0,00
**METTL5**	11,73	11,73	0,00
**PPA2**	12,42	12,42	0,00
**SLC39A9**	12,54	12,53	0,00
**ALG9**	11,17	11,17	0,00
**KHDC4**	13,20	13,20	0,00
**ACTR3B**	10,00	10,00	0,00
**ATP6AP1**	12,01	12,01	0,00
**THOC5**	11,64	11,64	0,00
**PRKCE**	12,82	12,82	0,00
**ATP6V0B**	12,67	12,67	0,00
**HLA-DQA1**	8,44	8,44	0,00
**ZNHIT1**	11,59	11,58	0,00
**PUM3**	13,15	13,15	0,00
**YY2**	8,26	8,26	0,00
**SLC35C1**	10,24	10,24	0,00
**COPS3**	13,26	13,26	0,00
**FAF2**	12,60	12,60	0,00
**DNAJB11**	12,92	12,92	0,00
**ZNF445**	12,45	12,45	0,00
**ATP6V1G1**	12,97	12,97	0,00
**MESD**	12,36	12,36	0,00
**ABI3**	6,93	6,93	0,00
**ERI2**	4,93	4,92	0,00
**SPATS1**	4,93	4,92	0,00
**AC098850.3**	6,48	6,48	0,00
**HTR7**	11,02	11,02	0,00
**RBX1**	12,35	12,35	0,00
**ZDHHC5**	12,82	12,82	0,00
**PDPR**	13,54	13,54	0,00
**CCT3**	15,68	15,68	0,00
**NUDT13**	8,14	8,14	0,00
**ABCC10**	9,46	9,46	0,00
**HSPA8**	17,71	17,71	0,00
**PLIN2**	13,18	13,18	0,00
**BMS1**	13,76	13,76	0,00
**CHD7**	14,16	14,16	0,00
**ABCF2-H2BE1**	11,93	11,93	0,00
**CDC6**	13,16	13,16	0,00
**YIPF6**	11,11	11,11	0,00

**ACBD4**	7,90	7,90	0,00
**TBL2**	11,18	11,18	0,00
**DENND6A**	11,85	11,85	0,00
**PPP1CB**	14,34	14,34	0,00
**LZTS1**	8,41	8,40	0,00
**TMCO1**	12,43	12,43	0,00
**PLD4**	11,23	11,23	0,00
**LLGL2**	10,44	10,44	0,00
**KIAA0319L**	11,05	11,05	0,00
**PTK6**	5,41	5,40	0,00
**PDSS1**	11,38	11,38	0,00
**WDFY2**	11,45	11,45	0,00
**PARVB**	8,98	8,98	0,00
**UBE2A**	12,66	12,66	0,00
**OSGEP**	11,91	11,90	0,00
**NUP58**	13,57	13,57	0,00
**MPND**	10,29	10,28	0,00
**EEF1E1**	11,83	11,83	0,00
**AGAP1**	11,75	11,74	0,00
**OCIAD1**	12,35	12,34	0,00
**PSMA1**	13,76	13,75	0,00
**ACTG1**	17,78	17,78	0,00
**SREK1IP1**	12,16	12,15	0,00
**N6AMT1**	9,69	9,69	0,00
**CHCHD10**	11,81	11,81	0,00
**HPS3**	11,28	11,28	0,00
**ZKSCAN3**	9,86	9,85	0,00
**HSP90AA1**	18,35	18,35	0,00
**CHST7**	9,06	9,06	0,00
**DTNBP1**	11,16	11,16	0,00
**ITSN1**	10,51	10,50	0,00
**CCDC39**	8,95	8,94	0,00
**HEMK1**	11,67	11,67	0,00
**MAT2A**	15,92	15,92	0,00
**CCL25**	5,01	5,01	0,00
**NFRKB**	9,60	9,59	0,00
**LDHA**	16,70	16,69	0,00
**GMCL1**	12,00	12,00	0,00
**MRPL47**	12,05	12,05	0,00
**C12orf66**	10,10	10,10	0,00
**PLK1**	13,38	13,38	0,00
**ALDH18A1**	13,92	13,92	0,00
**ZNF583**	10,02	10,02	0,00
**PSMA5**	13,64	13,64	0,00
**ACOT13**	11,36	11,36	0,00
**PCGF3**	13,13	13,12	0,01
**IMPA2**	11,27	11,26	0,01
**CEP128**	10,81	10,81	0,01
**AGPAT1**	11,56	11,55	0,01
**PGK1**	14,96	14,95	0,01
**DNAJB6**	13,24	13,24	0,01
**CANT1**	12,15	12,15	0,01
**ATP5F1B**	16,08	16,07	0,01
**GLB1L**	6,98	6,98	0,01
**GLB1**	11,27	11,26	0,01
**TBC1D13**	11,53	11,53	0,01

RPS27L	12,06	12,06	0,01
PARP16	11,37	11,36	0,01
ZRANB1	11,31	11,30	0,01
DUSP6	11,84	11,83	0,01
SELENOT	12,74	12,74	0,01
SETD7	13,04	13,03	0,01
RPL7A	16,94	16,94	0,01
PSMD6	12,97	12,97	0,01
TANK	10,33	10,32	0,01
RNF122	11,07	11,06	0,01
TMEM165	12,48	12,47	0,01
MAS1	5,57	5,57	0,01
DENND10	10,62	10,61	0,01
TMOD2	10,53	10,52	0,01
PHF20	12,18	12,17	0,01
TFAM	13,95	13,94	0,01
USP21	11,02	11,01	0,01
SLC25A30	11,01	11,00	0,01
LTV1	12,86	12,85	0,01
AOC3	7,20	7,19	0,01
HNRNPDL	15,83	15,83	0,01
C7orf26	12,21	12,21	0,01
GOLM1	12,46	12,45	0,01
PCDHB10	4,64	4,64	0,01
LRRC41	12,75	12,74	0,01
FXYD5	12,52	12,52	0,01
UBALD2	11,28	11,28	0,01
C20orf96	10,47	10,47	0,01
AC005192.1	6,19	6,18	0,01
RPL15	16,42	16,42	0,01
DHX33	13,48	13,47	0,01
MS4A14	2,91	2,90	0,01
NEIL2	11,03	11,02	0,01
TBX21	9,51	9,50	0,01
CPS1	7,34	7,34	0,01
CEP78	13,05	13,04	0,01
ACADS	8,85	8,85	0,01
DDX54	13,28	13,27	0,01
TGFBRAP1	12,20	12,19	0,01
ERCC5	12,37	12,36	0,01
DNAJC19	11,19	11,19	0,01
ZNF841	9,73	9,72	0,01
ITGB7	9,67	9,66	0,01
GMNN	12,51	12,50	0,01
MCFD2	12,83	12,82	0,01
LRIG1	13,06	13,05	0,01
CD52	7,80	7,80	0,01
TM6SF1	6,54	6,53	0,01
HERC2	13,71	13,70	0,01
DPM2	10,68	10,67	0,01
TIMM17A	13,10	13,10	0,01
ZNF789	10,99	10,98	0,01
VPS26B	12,83	12,82	0,01
KDM7A	10,04	10,03	0,01
GPR146	9,09	9,09	0,01
ARHGAP29	8,26	8,25	0,01

**SPATA2**	10,16	10,15	0,01
**FAM204A**	11,91	11,91	0,01
**ARL2BP**	11,52	11,51	0,01
**NSMF**	12,24	12,23	0,01
**GTF2IRD2**	7,76	7,75	0,01
**EIF2AK1**	13,94	13,93	0,01
**SZT2**	12,13	12,12	0,01
**RAB5B**	13,09	13,08	0,01
**ARHGAP10**	10,25	10,25	0,01
**KCNK6**	11,22	11,21	0,01
**ANKS6**	12,26	12,25	0,01
**FUT10**	9,78	9,78	0,01
**SLC25A11**	12,04	12,03	0,01
**TXNL4A**	12,43	12,42	0,01
**DOCK10**	14,09	14,08	0,01
**CAMSAP1**	12,72	12,71	0,01
**ST6GALNAC6**	9,72	9,72	0,01
**HIPK1**	13,03	13,03	0,01
**RABEPK**	12,02	12,02	0,01
**MICOS10**	12,39	12,38	0,01
**KIFAP3**	11,26	11,25	0,01
**PRSS21**	9,70	9,69	0,01
**LRP10**	11,75	11,74	0,01
**FLG**	7,99	7,99	0,01
**TACC1**	13,06	13,05	0,01
**CENPS-CORT**	8,38	8,37	0,01
**AP002990.1**	9,73	9,72	0,01
**METTL23**	10,24	10,23	0,01
**HLA-DMA**	11,01	11,01	0,01
**CSTF3**	12,53	12,52	0,01
**CSPP1**	12,45	12,44	0,01
**SLC9A7**	11,02	11,01	0,01
**UBE2D3**	14,97	14,96	0,01
**MTHFD2**	14,50	14,49	0,01
**SMPD1**	6,13	6,12	0,01
**PTTG1IP**	12,59	12,58	0,01
**SLC16A10**	9,45	9,44	0,01
**ZNF397**	12,09	12,08	0,01
**NCKAP1L**	13,35	13,34	0,01
**NUP42**	11,34	11,33	0,01
**RNF19B**	10,82	10,81	0,01
**RALA**	12,24	12,23	0,01
**B4GALNT4**	9,39	9,38	0,01
**KIF2A**	13,72	13,71	0,01
**INSR**	13,72	13,71	0,01
**SLC25A5**	15,34	15,33	0,01
**MAP4K2**	10,66	10,66	0,01
**HAT1**	12,96	12,95	0,01
**TK1**	12,75	12,74	0,01
**SLC39A14**	13,91	13,90	0,01
**MGAT5**	14,16	14,16	0,01
**NELL2**	11,03	11,02	0,01
**RBM34**	12,49	12,48	0,01
**DHX9**	15,73	15,72	0,01
**CORO2B**	11,33	11,32	0,01
**TRIM22**	13,12	13,11	0,01

**COMMD10**	10,42	10,41	0,01
**SYNJ1**	10,02	10,01	0,01
**ZNRF3**	10,42	10,41	0,01
**SNX29**	11,47	11,46	0,01
**ADAT3**	8,29	8,28	0,01
**SLC2A5**	12,05	12,04	0,01
**PRIM2**	11,33	11,32	0,01
**WDR82**	14,19	14,18	0,01
**SAV1**	11,37	11,36	0,01
**ZNF134**	11,72	11,71	0,01
**MIEN1**	10,82	10,81	0,01
**ZNF736**	12,78	12,77	0,01
**SCD**	15,36	15,35	0,01
**DNAAF5**	12,63	12,62	0,01
**POLR3H**	11,92	11,91	0,01
**NOL4L**	10,46	10,45	0,01
**SS18L2**	11,42	11,41	0,01
**EIF1**	14,89	14,88	0,01
**IFT81**	11,38	11,37	0,01
**TNKS**	12,95	12,94	0,01
**ANKRD10**	13,33	13,32	0,01
**SRGAP3**	9,80	9,79	0,01
**MRPL16**	12,07	12,06	0,01
**CD34**	6,78	6,77	0,01
**AC006254.1**	7,90	7,89	0,01
**ACAT2**	12,12	12,11	0,01
**NUDT15**	11,60	11,59	0,01
**TRIP4**	10,47	10,46	0,01
**UBE2L3**	13,83	13,82	0,01
**ACY3**	6,11	6,10	0,01
**UBA2**	14,27	14,26	0,01
**ECHDC1**	12,91	12,90	0,01
**FBXO21**	12,47	12,46	0,01
**EBLN2**	7,52	7,51	0,01
**ANXA2**	12,80	12,79	0,01
**VPS54**	11,61	11,60	0,01
**PBRM1**	14,09	14,08	0,01
**ACTB**	18,19	18,18	0,01
**CDCA7L**	13,86	13,85	0,01
**ZNF394**	10,57	10,56	0,01
**FUBP1**	15,19	15,18	0,01
**LRP2BP**	8,16	8,15	0,01
**TOP1**	15,01	15,00	0,01
**AS3MT**	11,37	11,36	0,01
**DBH**	7,15	7,13	0,01
**SYNPO2**	4,86	4,85	0,01
**CD81**	13,34	13,33	0,01
**PNPO**	11,31	11,30	0,01
**SHC1**	12,65	12,63	0,01
**ICAM2**	11,50	11,49	0,01
**AGRP**	6,33	6,32	0,01
**TMUB1**	10,46	10,45	0,01
**NXN**	13,28	13,26	0,01
**CCDC149**	11,37	11,36	0,01
**CDON**	8,81	8,80	0,01
**GOLPH3L**	11,62	11,61	0,01

**RPLP0**	17,51	17,50	0,01
**BMP2**	9,67	9,66	0,01
**PP2D1**	4,04	4,03	0,01
**SLC15A2**	10,65	10,64	0,01
**DAP3**	13,33	13,32	0,01
**FBH1**	12,13	12,12	0,01
**MT-ND1**	17,17	17,16	0,01
**TUBE1**	10,74	10,73	0,01
**VPS39**	12,62	12,61	0,01
**HASPIN**	11,05	11,03	0,01
**DNASE1**	10,97	10,96	0,01
**RNASEH1**	12,80	12,79	0,01
**CYP2U1**	9,29	9,28	0,01
**G3BP1**	15,21	15,20	0,01
**NAA20**	12,71	12,70	0,01
**NCAPH**	13,26	13,25	0,01
**LY86**	8,45	8,44	0,01
**PEX11B**	11,30	11,29	0,01
**SMARCE1**	14,36	14,35	0,01
**PRR14L**	12,91	12,89	0,01
**NASP**	15,43	15,41	0,01
**KRBA1**	9,72	9,70	0,01
**TPRKB**	11,45	11,44	0,01
**MAST2**	11,96	11,94	0,01
**COA5**	10,10	10,09	0,01
**LYSMD3**	11,53	11,52	0,01
**UCP2**	12,12	12,11	0,01
**MED21**	11,42	11,41	0,01
**RAB21**	12,40	12,38	0,01
**RPL13A**	17,32	17,30	0,01
**CCT5**	15,55	15,54	0,01
**INO80**	12,58	12,56	0,01
**FXYD7**	4,86	4,84	0,01
**H2AC15**	4,29	4,27	0,01
**SMIM13**	10,74	10,73	0,01
**BCL2L13**	11,87	11,86	0,01
**NELFCD**	13,30	13,29	0,01
**XAF1**	11,20	11,19	0,01
**ABCB4**	9,34	9,33	0,01
**ZNF658**	8,20	8,19	0,01
**TREX1**	9,94	9,92	0,01
**CUL7**	9,48	9,47	0,01
**TMEM132D**	5,25	5,24	0,01
**TLN1**	14,77	14,75	0,01
**TM9SF4**	12,20	12,19	0,01
**AC092718.2**	8,35	8,34	0,01
**ZNF697**	8,27	8,26	0,01
**ACO1**	11,65	11,63	0,01
**E2F3**	13,32	13,31	0,01
**XPA**	10,36	10,34	0,01
**NDUFA4L2**	6,94	6,92	0,01
**UNC5CL**	7,39	7,38	0,01
**LIX1L**	12,68	12,67	0,01
**SRSF2**	15,84	15,83	0,01
**TARS1**	14,24	14,23	0,01
**SGTA**	13,10	13,09	0,01

RCBTB1	13,46	13,45	0,01
TROAP	11,87	11,86	0,01
METTL21A	11,32	11,31	0,01
C1orf43	14,10	14,09	0,01
ZNF33A	12,68	12,66	0,01
LSG1	12,71	12,70	0,01
BACH1	11,97	11,95	0,01
ZNF273	10,28	10,27	0,01
INPP5B	11,72	11,71	0,01
PITPNM2	11,47	11,46	0,01
NBPF9	10,82	10,80	0,01
NFIX	12,57	12,55	0,01
COX7A2L	12,82	12,81	0,01
FKBP5	13,70	13,69	0,01
UPF2	13,40	13,39	0,01
SCML2	12,35	12,34	0,01
HEATR5B	11,70	11,68	0,01
BACE1	10,14	10,13	0,01
JAKMIP1	8,84	8,83	0,01
SLK	12,37	12,36	0,01
PSEN2	9,42	9,40	0,01
PSMB1	13,56	13,55	0,01
FXR1	13,84	13,83	0,01
HDDC2	12,61	12,60	0,01
NOL10	12,38	12,37	0,01
NTPCR	11,49	11,47	0,01
HIPK2	12,61	12,60	0,01
LRRC75A	12,87	12,86	0,01
TUB	11,75	11,73	0,01
SSH2	12,90	12,88	0,01
SETBP1	10,15	10,13	0,01
FEM1C	11,50	11,48	0,01
TMEM106A	9,21	9,19	0,01
PLSCR3	10,71	10,69	0,01
PSMD12	12,92	12,91	0,01
FP565260.6	10,07	10,06	0,01
ERG28	10,52	10,51	0,01
URB1	13,01	12,99	0,01
AP2A1	12,35	12,33	0,01
ISYNA1	11,34	11,32	0,01
MKKS	11,22	11,20	0,01
ARL4C	14,29	14,28	0,01
FABP3	5,48	5,46	0,01
DUSP8	5,48	5,46	0,01
PSMA4	14,23	14,21	0,01
TET3	13,18	13,16	0,01
PRR36	9,52	9,51	0,01
GALC	9,44	9,43	0,01
RBIS	12,05	12,04	0,01
VPS29	11,61	11,59	0,01
PPP1R3B	10,65	10,63	0,01
MFSD1	11,51	11,49	0,01
KDM3A	11,89	11,87	0,01
NUFIP1	11,21	11,20	0,01
LARP4B	13,11	13,09	0,01
GDE1	12,17	12,15	0,01

**ABRACL**	13,31	13,30	0,02
**COPS8**	12,48	12,47	0,02
**DPY30**	11,13	11,11	0,02
**TRAC**	12,78	12,76	0,02
**FXN**	11,19	11,18	0,02
**ZC3H3**	11,56	11,54	0,02
**IGF1R**	12,30	12,29	0,02
**FDX2**	8,43	8,41	0,02
**REXO2**	10,96	10,94	0,02
**GNAI3**	13,02	13,01	0,02
**IGHV1-3**	13,95	13,93	0,02
**CEP170B**	7,42	7,41	0,02
**ZNF141**	11,73	11,72	0,02
**SELL**	8,70	8,68	0,02
**ZNF471**	9,09	9,07	0,02
**PLAGL2**	12,59	12,58	0,02
**SDCCAG8**	9,23	9,22	0,02
**MGME1**	11,58	11,56	0,02
**ADIPOR2**	13,10	13,08	0,02
**TNFAIP2**	11,24	11,22	0,02
**RIDA**	10,37	10,35	0,02
**USP16**	12,34	12,32	0,02
**YBX3**	13,65	13,64	0,02
**NBPF20**	10,17	10,15	0,02
**RPS4X**	17,00	16,99	0,02
**VRK2**	11,41	11,40	0,02
**IRS2**	12,01	11,99	0,02
**AL133352.1**	7,42	7,40	0,02
**RBBP5**	12,42	12,40	0,02
**MT-ND4L**	15,05	15,03	0,02
**C17orf58**	6,36	6,35	0,02
**HVCN1**	9,88	9,86	0,02
**PPP4R3B**	13,59	13,57	0,02
**CD53**	13,53	13,52	0,02
**WDR48**	11,86	11,84	0,02
**RELCH**	11,55	11,53	0,02
**MYO1D**	9,90	9,89	0,02
**TMEM248**	12,94	12,92	0,02
**UACA**	8,52	8,51	0,02
**CWC27**	11,57	11,55	0,02
**TMEM127**	11,66	11,64	0,02
**MMAA**	9,86	9,84	0,02
**ITPKC**	9,07	9,06	0,02
**HNRNPH2**	13,43	13,41	0,02
**LIMK1**	11,44	11,43	0,02
**ALKBH3**	9,48	9,47	0,02
**KCNS3**	10,89	10,88	0,02
**MAP2K4**	11,86	11,84	0,02
**NOD1**	9,48	9,46	0,02
**DCAF4L1**	6,37	6,35	0,02
**IER5L**	6,37	6,35	0,02
**IMPDH2**	15,02	15,00	0,02
**DCAF13**	13,82	13,80	0,02
**NPRL2**	10,66	10,64	0,02
**APOBEC3G**	10,37	10,36	0,02
**MED13L**	13,41	13,39	0,02

PLEKHM1	11,35	11,33	0,02
EEF1G	17,17	17,16	0,02
NSUN2	14,12	14,11	0,02
ALG10	10,17	10,15	0,02
CXXC5	12,13	12,11	0,02
EARS2	12,15	12,14	0,02
FTH1	14,84	14,82	0,02
P4HA2	10,88	10,87	0,02
COMMD7	11,81	11,80	0,02
H3-3A	16,08	16,06	0,02
BCAT1	16,23	16,21	0,02
OSTF1	11,05	11,03	0,02
NPC1L1	3,47	3,45	0,02
CHD4	15,53	15,51	0,02
RAB28	10,72	10,70	0,02
ZW10	11,94	11,92	0,02
TTPAL	11,32	11,30	0,02
RIOK1	12,34	12,32	0,02
TRAK1	12,75	12,74	0,02
PRMT6	11,93	11,91	0,02
MYORG	9,37	9,36	0,02
MTMR12	12,56	12,55	0,02
ELP3	12,39	12,37	0,02
PSMB3	13,33	13,31	0,02
HLCS	10,40	10,38	0,02
PDCD6IP	13,81	13,79	0,02
APOL2	10,77	10,75	0,02
RTKN2	12,18	12,16	0,02
NFATC3	13,34	13,33	0,02
HERC4	11,86	11,85	0,02
ZSCAN32	10,85	10,84	0,02
METAP1	12,93	12,91	0,02
ZNF581	10,85	10,83	0,02
STIMATE	11,24	11,22	0,02
INTS4	11,81	11,79	0,02
PRKX	14,06	14,05	0,02
KCNK10	9,04	9,02	0,02
NPTXR	8,37	8,35	0,02
PDPK1	12,47	12,46	0,02
FBXO5	12,89	12,87	0,02
ATG14	10,60	10,58	0,02
NIT1	10,21	10,19	0,02
KIAA1614	8,49	8,47	0,02
SORCS1	3,59	3,57	0,02
PPCS	10,98	10,96	0,02
RFXAP	11,16	11,14	0,02
RFWD3	14,08	14,06	0,02
TSPAN33	10,35	10,33	0,02
AC093827.5	6,77	6,75	0,02
EDRF1	12,05	12,04	0,02
CNOT9	13,59	13,58	0,02
RAB7A	13,82	13,80	0,02
PTP4A2	13,95	13,93	0,02
BIRC5	13,53	13,51	0,02
ABHD2	12,25	12,23	0,02
PPP2R5D	12,61	12,59	0,02

**GLT8D1**	11,29	11,27	0,02
**CIB1**	10,99	10,97	0,02
**GON4L**	12,57	12,55	0,02
**TRIP11**	12,12	12,10	0,02
**ICMT**	13,26	13,24	0,02
**HMCES**	12,73	12,71	0,02
**DNAJC7**	13,34	13,33	0,02
**WDR77**	13,20	13,18	0,02
**SRD5A1**	9,51	9,49	0,02
**AP4B1**	11,26	11,24	0,02
**SLFN13**	11,14	11,12	0,02
**NR1D2**	11,17	11,15	0,02
**ICE2**	12,97	12,95	0,02
**SUGT1**	12,66	12,64	0,02
**RABGAP1**	11,91	11,89	0,02
**PDIA6**	16,32	16,30	0,02
**GTSE1**	12,72	12,70	0,02
**SLC6A9**	9,31	9,29	0,02
**CNOT4**	10,71	10,69	0,02
**ENOPH1**	12,81	12,79	0,02
**PRPF4**	12,94	12,92	0,02
**CDKN1B**	11,85	11,83	0,02
**MGST3**	11,22	11,20	0,02
**RNF2**	11,45	11,43	0,02
**ALDH6A1**	10,63	10,61	0,02
**SNX2**	13,42	13,40	0,02
**KMT5B**	12,20	12,18	0,02
**C11orf71**	9,34	9,32	0,02
**KHDRBS3**	12,49	12,47	0,02
**ENOSF1**	11,45	11,43	0,02
**GTF2IRD2B**	10,99	10,97	0,02
**C17orf80**	11,18	11,16	0,02
**VIRMA**	13,55	13,53	0,02
**MITD1**	10,86	10,84	0,02
**RGPD8**	11,12	11,10	0,02
**MT-ATP6**	18,01	17,99	0,02
**YIPF4**	12,13	12,11	0,02
**LRRFIP2**	11,25	11,23	0,02
**DDB2**	11,03	11,01	0,02
**CMTR2**	11,96	11,94	0,02
**GIGYF2**	12,93	12,91	0,02
**SFXN3**	10,50	10,48	0,02
**RAB4A**	9,56	9,54	0,02
**OGFOD3**	10,41	10,39	0,02
**TRNAU1AP**	10,39	10,37	0,02
**WDR92**	10,97	10,95	0,02
**RAP2C**	11,43	11,41	0,02
**TSPAN7**	6,78	6,76	0,02
**RAN**	16,05	16,03	0,02
**KBTBD6**	11,20	11,17	0,02
**TRAPPC10**	11,60	11,58	0,02
**TMEM208**	10,83	10,81	0,02
**SP1**	13,91	13,89	0,02
**MED6**	11,61	11,59	0,02
**SLC7A11**	11,27	11,25	0,02
**IGIP**	7,82	7,80	0,02

**ACOT2**	8,51	8,48	0,02
**ELOC**	12,63	12,61	0,02
**SUPV3L1**	12,02	12,00	0,02
**COQ5**	11,33	11,31	0,02
**RASA3**	11,38	11,36	0,02
**VAMP8**	11,06	11,04	0,02
**AGO1**	12,65	12,63	0,02
**RBM12**	14,54	14,52	0,02
**TDRD3**	10,84	10,82	0,02
**CTPS1**	13,99	13,97	0,02
**MPV17L2**	9,95	9,93	0,02
**TTYH1**	8,42	8,40	0,02
**FBXL17**	10,60	10,58	0,02
**DYNC2I2**	12,15	12,13	0,02
**PLP2**	10,90	10,88	0,02
**NAT10**	14,25	14,23	0,02
**PSMD1**	14,25	14,23	0,02
**TEKT4**	2,91	2,89	0,02
**RNF146**	11,24	11,22	0,02
**DAP**	13,20	13,18	0,02
**ACSL1**	10,62	10,60	0,02
**CASTOR2**	10,35	10,32	0,02
**DGUOK**	11,54	11,52	0,02
**WLS**	9,23	9,21	0,02
**MAP3K14**	10,79	10,77	0,02
**PSMD11**	13,82	13,80	0,02
**ZNF701**	10,38	10,35	0,02
**COLQ**	7,96	7,94	0,02
**SNX3**	11,90	11,88	0,02
**SLC44A1**	13,05	13,03	0,02
**IFT52**	11,27	11,24	0,02
**ARID4A**	11,54	11,51	0,02
**TMEM183A**	13,34	13,32	0,02
**HNRNPK**	16,65	16,63	0,02
**TSEN15**	12,55	12,53	0,02
**RAP2B**	12,25	12,23	0,02
**N4BP2L1**	10,26	10,24	0,02
**METTL3**	12,52	12,50	0,02
**STRADB**	11,55	11,53	0,02
**WWTR1**	7,42	7,39	0,02
**LYG2**	4,17	4,15	0,02
**SMDT1**	11,05	11,02	0,02
**P2RX4**	9,46	9,43	0,02
**ST3GAL1**	11,82	11,80	0,02
**PHKG2**	11,06	11,04	0,02
**FAM216A**	11,60	11,58	0,02
**FBXW8**	11,27	11,25	0,02
**HSF2BP**	6,22	6,20	0,02
**POC1A**	11,20	11,18	0,02
**FCHO1**	10,64	10,62	0,02
**FAM171A1**	12,40	12,37	0,02
**ZNF253**	12,29	12,27	0,02
**ACTR5**	10,85	10,83	0,02
**DTD2**	11,23	11,21	0,02
**STAG2**	14,00	13,98	0,02
**SAAL1**	11,65	11,63	0,02

FOXP3	3,82	3,79	0,02
ALDOB	3,82	3,79	0,02
SHE	3,82	3,79	0,02
MAPRE1	14,08	14,05	0,02
HSPA5	15,90	15,87	0,02
RC3H2	12,90	12,87	0,02
PRKAB2	11,00	10,98	0,02
AP1AR	11,89	11,87	0,02
ZBED4	13,38	13,35	0,02
EIF4EBP2	14,54	14,52	0,02
IVD	12,43	12,41	0,02
COMMD2	11,81	11,78	0,02
PTPRA	12,19	12,16	0,02
SLC37A3	10,79	10,77	0,02
MTHFD1	14,24	14,21	0,02
TMEM191C	5,03	5,01	0,02
NUP93	13,80	13,78	0,02
TAMALIN	6,91	6,89	0,02
RPS6KA2	8,54	8,51	0,02
CCDC18	9,48	9,45	0,02
CEP97	11,45	11,43	0,02
FAM104B	9,62	9,60	0,02
TDP1	12,19	12,16	0,02
CATSPERE	5,99	5,97	0,02
CD99L2	10,84	10,81	0,02
PRDX6	13,94	13,92	0,02
LRFN3	8,42	8,40	0,02
TBC1D15	11,71	11,69	0,02
HMX2	5,07	5,05	0,02
SLC1A1	5,33	5,31	0,02
ALDH8A1	5,33	5,31	0,02
FBXL16	5,33	5,31	0,02
THSD7B	5,33	5,31	0,02
TPR	14,67	14,64	0,02
CCNB1	13,37	13,34	0,02
ODC1	16,36	16,34	0,02
GCN1	14,23	14,21	0,02
TRAPPC4	11,28	11,25	0,02
C17orf113	5,90	5,88	0,02
ZNF90	11,40	11,37	0,02
SPC25	11,37	11,34	0,02
ITPR3	12,41	12,39	0,02
VIPR2	9,08	9,05	0,02
MARK3	12,58	12,56	0,02
INSIG1	12,92	12,90	0,02
RRH	6,65	6,62	0,02
CDC23	12,61	12,59	0,02
FNBP1L	11,91	11,89	0,03
FAM160A2	10,22	10,19	0,03
MRPL40	10,23	10,21	0,03
ZNF461	9,44	9,42	0,03
ITM2C	12,45	12,43	0,03
PRELID3B	13,29	13,27	0,03
AIMP2	11,97	11,94	0,03
SDHD	12,57	12,55	0,03
TMEM230	12,53	12,51	0,03

**TICRR**	12,76	12,73	0,03
**SLC26A8**	3,03	3,00	0,03
**HBEGF**	11,32	11,30	0,03
**PTPDC1**	10,17	10,14	0,03
**TP53BP2**	13,10	13,08	0,03
**PARP6**	11,62	11,60	0,03
**NEMF**	12,88	12,85	0,03
**ZBTB40**	12,53	12,50	0,03
**TCEA1**	14,73	14,70	0,03
**RPL4**	17,25	17,23	0,03
**INTS13**	12,71	12,68	0,03
**NIN**	14,54	14,52	0,03
**ERLIN2**	12,03	12,01	0,03
**RHOC**	10,61	10,59	0,03
**C2orf49**	11,37	11,34	0,03
**ABCE1**	14,28	14,26	0,03
**SRP72**	14,15	14,12	0,03
**CCDC30**	7,92	7,89	0,03
**JPH1**	10,15	10,12	0,03
**TCAIM**	11,31	11,28	0,03
**PRAF2**	9,54	9,52	0,03
**SLC4A8**	7,46	7,43	0,03
**STMP1**	11,97	11,95	0,03
**KNSTRN**	11,68	11,65	0,03
**CD55**	10,11	10,08	0,03
**SNX18**	11,02	10,99	0,03
**SLC7A6OS**	12,01	11,98	0,03
**POGLUT2**	8,53	8,50	0,03
**HSDL1**	11,85	11,83	0,03
**AGO3**	12,25	12,22	0,03
**FOXJ3**	12,82	12,80	0,03
**ARID5B**	13,26	13,23	0,03
**CDC14A**	10,13	10,10	0,03
**PLEKHG4B**	9,62	9,60	0,03
**APOO**	10,21	10,18	0,03
**CCDC154**	4,36	4,34	0,03
**GLE1**	12,31	12,28	0,03
**FOXO3**	11,78	11,75	0,03
**PYGO1**	10,50	10,48	0,03
**OSGIN1**	4,32	4,29	0,03
**NFX1**	12,97	12,94	0,03
**YME1L1**	14,20	14,17	0,03
**BCCIP**	13,35	13,32	0,03
**NUP107**	13,70	13,68	0,03
**TP53**	14,31	14,29	0,03
**DDX46**	14,03	14,01	0,03
**DDX24**	14,18	14,15	0,03
**GIT2**	12,37	12,34	0,03
**WDR27**	10,30	10,27	0,03
**DPM1**	11,84	11,81	0,03
**LMLN**	10,08	10,06	0,03
**DPYSL2**	14,05	14,02	0,03
**SOWAHD**	6,44	6,42	0,03
**EIF4ENIF1**	11,86	11,83	0,03
**ZBTB21**	10,62	10,60	0,03
**HMGXB3**	12,55	12,53	0,03

G6PC3	11,61	11,59	0,03
ZNF839	10,90	10,88	0,03
ACSS2	10,57	10,54	0,03
BNIP2	13,12	13,09	0,03
ATP5MF-PTCD1	9,38	9,35	0,03
SEC23B	12,38	12,35	0,03
FAM32A	12,21	12,19	0,03
POLR2C	12,76	12,73	0,03
RND2	6,08	6,05	0,03
TRIP12	14,18	14,15	0,03
SDHA	14,10	14,07	0,03
SNRNP40	13,17	13,14	0,03
TYMS	14,91	14,88	0,03
CRTC1	10,72	10,69	0,03
SSBP3	12,15	12,12	0,03
MDH1	13,43	13,41	0,03
C8orf76	11,14	11,11	0,03
CENPE	13,18	13,15	0,03
MAML1	12,65	12,62	0,03
RIT1	10,70	10,67	0,03
C21orf91	11,84	11,82	0,03
WAPL	14,14	14,11	0,03
NPIPB11	9,15	9,12	0,03
PPP4R3A	13,40	13,37	0,03
HLA-DPA1	11,80	11,78	0,03
EXOC8	11,38	11,35	0,03
ACVR2B	11,71	11,68	0,03
TOLLIP	10,99	10,96	0,03
ASTE1	9,70	9,67	0,03
TPX2	14,38	14,35	0,03
AC022414.1	5,77	5,74	0,03
GNG12	9,61	9,58	0,03
ACAD8	10,92	10,90	0,03
C16orf70	11,29	11,26	0,03
CUL3	13,44	13,41	0,03
RSRP1	12,88	12,85	0,03
MSI2	14,96	14,93	0,03
UBASH3B	14,78	14,75	0,03
CTCF	14,01	13,99	0,03
SLC66A3	9,32	9,29	0,03
RALBP1	13,35	13,32	0,03
BRAP	11,70	11,67	0,03
ABCC4	12,50	12,47	0,03
MPC1	11,12	11,09	0,03
GIMAP6	4,46	4,43	0,03
RITA1	10,88	10,85	0,03
HDAC1	13,54	13,51	0,03
DYRK1B	7,51	7,48	0,03
NMT1	13,52	13,49	0,03
COQ2	10,75	10,72	0,03
ANAPC16	12,29	12,26	0,03
PPFIA1	12,25	12,22	0,03
CCDC43	11,80	11,77	0,03
SUSD6	12,10	12,07	0,03
PPARA	5,64	5,61	0,03
UTP4	13,23	13,20	0,03

POFUT2	10,10	10,07	0,03
ARL11	10,64	10,61	0,03
CNBP	15,38	15,35	0,03
POU2F2	9,13	9,10	0,03
ADGRD1	9,88	9,85	0,03
GPN1	12,28	12,25	0,03
ARL3	10,88	10,85	0,03
OSGIN2	11,73	11,70	0,03
MAPK4	7,75	7,72	0,03
NRSN2	9,30	9,27	0,03
E2F8	12,04	12,01	0,03
TMEM141	10,37	10,34	0,03
ZNFX1	12,30	12,27	0,03
DNAJC2	12,94	12,91	0,03
SLC25A3	15,13	15,09	0,03
RAB22A	11,62	11,59	0,03
PARP2	11,98	11,95	0,03
SEPTIN8	12,06	12,03	0,03
RPE	12,16	12,13	0,03
STARD3	10,97	10,94	0,03
YAE1	9,56	9,53	0,03
NEIL3	10,65	10,62	0,03
ORC6	12,58	12,55	0,03
RFC3	12,92	12,89	0,03
APOBEC3F	9,79	9,76	0,03
LGR6	10,69	10,66	0,03
NMI	11,62	11,59	0,03
RFESD	8,23	8,20	0,03
CHD6	12,43	12,40	0,03
ZNF490	9,47	9,44	0,03
IDS	12,33	12,30	0,03
ZMYND11	12,01	11,98	0,03
SNX21	7,53	7,50	0,03
NDRG1	9,84	9,81	0,03
PHF10	13,64	13,61	0,03
SHLD1	9,31	9,28	0,03
IST1	13,58	13,55	0,03
C14orf119	11,30	11,27	0,03
NKAP	11,10	11,07	0,03
IKZF1	14,81	14,78	0,03
CIT	12,84	12,81	0,03
AP000356.5	11,33	11,30	0,03
BPTF	13,98	13,95	0,03
DAPL1	4,05	4,02	0,03
MBP	12,37	12,34	0,03
NDUFAF4	11,81	11,78	0,03
PPOX	9,83	9,80	0,03
SH3GLB2	11,34	11,30	0,03
DNA2	12,16	12,13	0,03
MRPL35	12,17	12,14	0,03
ADNP2	12,10	12,06	0,03
S1PR3	6,54	6,51	0,03
CSNK1G1	11,60	11,57	0,03
P4HTM	9,41	9,38	0,03
TXNDC11	11,61	11,58	0,03
DHPS	11,83	11,79	0,03

**MINDY1**	9,26	9,23	0,03
**DDR2**	7,28	7,25	0,03
**RBM15**	11,12	11,09	0,03
**ZNF512**	12,67	12,64	0,03
**HIVEP2**	13,07	13,04	0,03
**C14orf132**	12,77	12,74	0,03
**ZNF550**	11,82	11,79	0,03
**SNX27**	12,07	12,04	0,03
**EIF5**	14,71	14,68	0,03
**FPGT-TNNI3K**	7,20	7,17	0,03
**PTPN2**	12,71	12,68	0,03
**VEZF1**	13,23	13,20	0,03
**ARF4**	12,59	12,56	0,03
**CD248**	11,20	11,17	0,03
**EAF1**	12,11	12,08	0,03
**DSG2**	11,16	11,13	0,03
**NAXD**	10,72	10,68	0,03
**GNAI1**	11,59	11,56	0,03
**SPTBN1**	16,03	16,00	0,03
**MRPS14**	11,65	11,62	0,03
**C18orf25**	11,33	11,30	0,03
**LYSMD2**	10,39	10,36	0,03
**PAK1IP1**	11,97	11,94	0,03
**HSPD1**	16,88	16,84	0,03
**SSR3**	13,33	13,30	0,03
**GOT2**	13,90	13,87	0,03
**FAM122A**	11,39	11,36	0,03
**TGOLN2**	13,68	13,65	0,03
**LPCAT2**	10,14	10,11	0,03
**ZNF813**	11,61	11,58	0,03
**ATF7IP2**	11,24	11,20	0,03
**AURKA**	12,24	12,21	0,03
**ZNF621**	11,26	11,23	0,03
**GLCE**	10,10	10,06	0,03
**SDHAF2**	11,08	11,05	0,03
**RRM2**	14,76	14,72	0,03
**XYLT1**	12,80	12,77	0,03
**CYB561D2**	9,82	9,79	0,03
**XRCC6**	15,75	15,72	0,03
**GIMAP2**	10,88	10,85	0,03
**TFRC**	15,13	15,10	0,03
**MTFR1**	11,51	11,48	0,03
**STIM2**	12,91	12,88	0,03
**SOGA1**	13,46	13,43	0,03
**ARRB1**	12,90	12,87	0,03
**UBAC2**	11,84	11,81	0,03
**DNAJB7**	7,36	7,33	0,03
**MKS1**	10,17	10,14	0,03
**EXOSC8**	11,28	11,24	0,03
**EIF3F**	14,57	14,54	0,03
**NECAB3**	8,77	8,73	0,03
**PPFIA4**	9,86	9,82	0,03
**ZBTB1**	11,52	11,49	0,03
**NUP160**	13,90	13,87	0,03
**TNFRSF21**	11,95	11,92	0,03
**LHX4**	9,50	9,47	0,03

**DLK2**	6,52	6,49	0,03
**MRPS27**	13,30	13,26	0,03
**ADAT1**	11,91	11,88	0,03
**CDC25A**	12,96	12,93	0,03
**GNL1**	10,29	10,25	0,03
**CEP135**	11,75	11,71	0,03
**MTMR9**	11,94	11,91	0,03
**FTSJ1**	11,89	11,85	0,03
**DNAJA2**	13,06	13,03	0,03
**APBB2**	12,90	12,87	0,03
**LMO7**	9,64	9,61	0,03
**ZNF211**	10,14	10,10	0,03
**RBBP9**	10,62	10,58	0,03
**HTR6**	6,96	6,92	0,03
**BCL7B**	11,80	11,77	0,03
**ETS1**	12,43	12,40	0,03
**ZNF317**	12,20	12,17	0,03
**ZBTB5**	11,73	11,69	0,03
**CEP112**	8,83	8,79	0,03
**PTPN12**	11,35	11,32	0,03
**IRF2**	11,75	11,71	0,03
**MEF2A**	12,44	12,40	0,03
**SLC26A6**	10,53	10,49	0,03
**DUSP22**	9,30	9,26	0,03
**SKA2**	12,43	12,39	0,03
**TLCD5**	11,62	11,59	0,03
**EXO1**	12,91	12,87	0,03
**ZNF441**	8,73	8,70	0,03
**IGHV2-70**	12,54	12,50	0,03
**RPS10**	15,46	15,42	0,03
**RCHY1**	9,92	9,89	0,03
**NR3C1**	13,50	13,47	0,03
**GNA12**	13,24	13,21	0,03
**LAPTM5**	15,66	15,63	0,03
**SYNGAP1**	10,92	10,89	0,03
**RAP1A**	11,77	11,74	0,03
**GSTO1**	12,36	12,33	0,03
**PDCD10**	11,83	11,80	0,03
**ZSCAN29**	11,67	11,64	0,03
**RPS6**	17,22	17,18	0,03
**SLC25A15**	11,64	11,61	0,03
**ORAI3**	10,75	10,72	0,03
**SMIM10**	5,41	5,37	0,04
**ESF1**	12,79	12,75	0,04
**WNT16**	15,10	15,07	0,04
**RNF10**	13,10	13,06	0,04
**PAXBP1**	13,07	13,03	0,04
**TUBGCP3**	12,89	12,85	0,04
**SAP130**	11,61	11,57	0,04
**SLAIN1**	12,84	12,80	0,04
**MRPL39**	11,75	11,71	0,04
**DFFB**	10,71	10,67	0,04
**SNRPB2**	13,71	13,67	0,04
**LTA4H**	12,82	12,78	0,04
**GFM1**	12,99	12,96	0,04
**MYH10**	13,92	13,88	0,04

**SARNP**	12,68	12,65	0,04
**MYADM**	12,59	12,56	0,04
**UQCR10**	11,87	11,83	0,04
**SRSF7**	15,12	15,09	0,04
**C12orf65**	11,61	11,57	0,04
**KAT6A**	14,26	14,23	0,04
**ADSL**	13,34	13,30	0,04
**GINS2**	13,10	13,06	0,04
**ACBD3**	11,90	11,87	0,04
**GNE**	11,37	11,34	0,04
**THBS3**	10,88	10,84	0,04
**MED30**	11,35	11,32	0,04
**TMEM9**	11,36	11,33	0,04
**CDPF1**	8,94	8,91	0,04
**DCAF12**	13,67	13,64	0,04
**RFT1**	11,39	11,35	0,04
**LRRC37A2**	10,43	10,39	0,04
**C9orf85**	10,10	10,06	0,04
**ZSCAN21**	9,34	9,31	0,04
**SMAD1**	12,11	12,07	0,04
**COMMD3**	12,39	12,35	0,04
**NEK7**	11,95	11,91	0,04
**ETFA**	12,51	12,48	0,04
**ILK**	11,30	11,26	0,04
**NPIPB5**	14,28	14,24	0,04
**UNC119B**	12,66	12,62	0,04
**PCBD1**	10,13	10,09	0,04
**TMEM50A**	12,63	12,59	0,04
**MAPK3**	11,14	11,10	0,04
**ZNF559**	10,82	10,78	0,04
**VPS45**	10,52	10,48	0,04
**NIPA2**	13,38	13,34	0,04
**NUDT2**	9,35	9,31	0,04
**NKTR**	14,25	14,21	0,04
**CASS4**	2,75	2,72	0,04
**CYB561D1**	10,72	10,68	0,04
**CDK18**	10,68	10,64	0,04
**SSB**	14,74	14,70	0,04
**CHKA**	11,69	11,65	0,04
**CFAP298-TCP10L**	9,87	9,84	0,04
**MBD1**	12,30	12,27	0,04
**RGL1**	11,46	11,42	0,04
**PCNA**	15,18	15,14	0,04
**CLINT1**	13,48	13,44	0,04
**FUT8**	11,24	11,20	0,04
**MYNN**	11,06	11,03	0,04
**ZFR**	13,76	13,73	0,04
**CREBL2**	10,65	10,61	0,04
**MICU2**	12,01	11,97	0,04
**POLA1**	13,27	13,23	0,04
**ZNF106**	13,15	13,12	0,04
**PHACTR1**	9,86	9,82	0,04
**PPP2R5B**	8,18	8,14	0,04
**ZNRD2**	11,17	11,14	0,04
**ZNF738**	11,76	11,72	0,04
**IFNGR2**	11,15	11,11	0,04

**RNF145**	13,29	13,26	0,04
**NAA50**	14,55	14,51	0,04
**HILPDA**	11,34	11,31	0,04
**MX2**	8,79	8,75	0,04
**SKA3**	12,58	12,54	0,04
**BLMH**	13,61	13,58	0,04
**CHAC2**	10,98	10,94	0,04
**AMBP**	6,90	6,87	0,04
**APEH**	12,82	12,78	0,04
**PPP1R7**	11,95	11,91	0,04
**ARHGAP1**	11,46	11,42	0,04
**BACE2**	8,54	8,50	0,04
**SLC6A8**	10,02	9,98	0,04
**DGKE**	12,34	12,30	0,04
**HPCAL1**	11,27	11,23	0,04
**NLRC4**	3,92	3,88	0,04
**TCL1A**	15,62	15,58	0,04
**UTP20**	13,57	13,53	0,04
**DOCK3**	7,40	7,36	0,04
**TTLL11**	7,59	7,55	0,04
**POLD3**	12,70	12,66	0,04
**AC005833.1**	11,90	11,86	0,04
**LMTK2**	11,71	11,68	0,04
**ACTL6A**	13,67	13,64	0,04
**NDUFB9**	13,70	13,66	0,04
**ARHGEF25**	8,53	8,49	0,04
**WSB1**	13,06	13,02	0,04
**ATP5F1A**	15,53	15,49	0,04
**DDX21**	15,41	15,37	0,04
**SMARCC1**	15,03	14,99	0,04
**MME**	9,14	9,11	0,04
**GREB1**	8,04	8,00	0,04
**THOC1**	12,28	12,24	0,04
**CENPA**	11,23	11,19	0,04
**TOMM70**	13,43	13,39	0,04
**SMARCD1**	13,68	13,64	0,04
**STAU2**	12,49	12,45	0,04
**SAP30BP**	12,01	11,97	0,04
**ZNF557**	10,93	10,89	0,04
**RHEB**	12,89	12,85	0,04
**DSTN**	12,21	12,17	0,04
**CNEP1R1**	10,69	10,65	0,04
**G3BP2**	14,22	14,18	0,04
**ZNF708**	11,62	11,58	0,04
**LCP1**	15,88	15,84	0,04
**STRAP**	14,13	14,09	0,04
**PHF19**	12,01	11,97	0,04
**GNG11**	7,41	7,37	0,04
**DDX1**	14,24	14,20	0,04
**RAD18**	12,44	12,40	0,04
**TAS2R10**	4,87	4,83	0,04
**IFI44L**	4,87	4,83	0,04
**DCTN5**	12,97	12,93	0,04
**TRAPPC9**	11,69	11,65	0,04
**UBE3B**	11,46	11,42	0,04
**P3H4**	9,28	9,24	0,04

**ARF1**	14,88	14,84	0,04
**SORD**	13,64	13,60	0,04
**TRRAP**	14,00	13,96	0,04
**PGBD1**	7,73	7,69	0,04
**PSPC1**	12,91	12,87	0,04
**FCRLA**	9,10	9,06	0,04
**DOCK2**	13,61	13,57	0,04
**WHAMM**	10,71	10,67	0,04
**EXOSC10**	13,26	13,22	0,04
**ZBTB46**	7,08	7,04	0,04
**WDR83OS**	10,77	10,73	0,04
**ADI1**	12,94	12,90	0,04
**CALD1**	14,37	14,33	0,04
**RIMKLB**	11,90	11,86	0,04
**UBE2D2**	13,35	13,31	0,04
**SMIM10L1**	11,73	11,69	0,04
**CCNL1**	12,61	12,57	0,04
**FZD4**	6,17	6,13	0,04
**HLA-DMB**	9,44	9,40	0,04
**TBX19**	7,73	7,69	0,04
**WDR25**	7,96	7,92	0,04
**DDX47**	12,79	12,75	0,04
**PTPN4**	11,32	11,28	0,04
**FBXL5**	11,82	11,78	0,04
**ADAT2**	11,89	11,85	0,04
**CLIP3**	6,74	6,69	0,04
**ACVR1B**	10,93	10,89	0,04
**IDI1**	13,76	13,72	0,04
**ABCD4**	11,08	11,04	0,04
**SSR2**	13,09	13,05	0,04
**ZNF568**	9,52	9,48	0,04
**FHOD1**	11,93	11,89	0,04
**KLF5**	6,81	6,77	0,04
**ACSL3**	13,17	13,13	0,04
**OSBPL2**	10,16	10,12	0,04
**TARDBP**	14,90	14,86	0,04
**TAF5L**	12,11	12,07	0,04
**PDK1**	11,67	11,62	0,04
**FGD6**	10,29	10,25	0,04
**ASB3**	10,39	10,35	0,04
**EMSY**	11,51	11,47	0,04
**FLNB**	13,32	13,28	0,04
**BTBD3**	12,61	12,57	0,04
**DENND6B**	9,96	9,92	0,04
**CDC20**	13,55	13,51	0,04
**CLNS1A**	13,10	13,06	0,04
**MYO1B**	13,17	13,13	0,04
**SIKE1**	12,70	12,66	0,04
**PLEKHA7**	6,58	6,54	0,04
**PEF1**	12,09	12,04	0,04
**GTF3A**	13,65	13,61	0,04
**POLR2B**	14,38	14,34	0,04
**CEBPZOS**	12,61	12,56	0,04
**NIBAN1**	8,36	8,32	0,04
**CCT6A**	15,50	15,46	0,04
**FAM151B**	5,75	5,71	0,04

**OSBPL9**	12,79	12,75	0,04
**PPP6R2**	12,16	12,12	0,04
**CHKB-CPT1B**	8,85	8,81	0,04
**CEP20**	12,10	12,06	0,04
**UEVLD**	10,60	10,56	0,04
**PCNX1**	12,37	12,33	0,04
**RASSF2**	12,66	12,62	0,04
**POLR2D**	13,11	13,07	0,04
**ATP6V1B2**	13,60	13,56	0,04
**EXOSC5**	11,56	11,52	0,04
**COASY**	12,27	12,23	0,04
**POMP**	13,43	13,39	0,04
**PER2**	11,80	11,76	0,04
**SMAD2**	12,60	12,56	0,04
**C8orf33**	13,60	13,56	0,04
**UBA3**	12,58	12,54	0,04
**KIF3A**	11,01	10,97	0,04
**RAD50**	12,01	11,97	0,04
**PLEKHA1**	9,31	9,27	0,04
**TMEM14B**	12,51	12,47	0,04
**ZDHHC7**	12,17	12,12	0,04
**DHRS13**	10,16	10,12	0,04
**BRF2**	10,64	10,60	0,04
**YWHAQ**	16,12	16,07	0,04
**RANBP10**	11,81	11,77	0,04
**SECISBP2**	12,47	12,43	0,04
**AGO2**	14,50	14,45	0,04
**TMEM245**	12,81	12,77	0,04
**PLCD3**	7,37	7,33	0,04
**TXNIP**	12,19	12,15	0,04
**MRPS18A**	10,98	10,94	0,04
**GLTP**	11,46	11,42	0,04
**PMAIP1**	12,64	12,60	0,04
**ATP5F1C**	13,85	13,80	0,04
**CBR4**	10,62	10,57	0,04
**LBHD1**	8,92	8,87	0,04
**RNF214**	10,44	10,40	0,04
**MTERF1**	10,59	10,55	0,04
**CUL4A**	13,65	13,60	0,04
**NPHP4**	10,86	10,82	0,04
**CHRNA5**	10,73	10,69	0,04
**TIMM9**	11,13	11,09	0,04
**NSF**	12,11	12,07	0,04
**PLEKHA3**	10,48	10,44	0,04
**MRPL44**	11,93	11,88	0,04
**CCNYL1**	10,65	10,61	0,04
**DYNC1I2**	12,80	12,76	0,04
**GOT1**	11,57	11,52	0,04
**BZW2**	13,80	13,76	0,04
**SCRN3**	10,20	10,15	0,04
**SPG21**	12,64	12,60	0,04
**TCEAL8**	11,73	11,68	0,04
**WDR43**	13,91	13,87	0,04
**PRRC1**	13,26	13,21	0,04
**VPS52**	12,01	11,97	0,04
**CGAS**	11,30	11,26	0,04

**ZFP69**	9,84	9,79	0,04
**TRIM6**	10,20	10,16	0,04
**UBA5**	12,37	12,32	0,04
**TGM5**	4,51	4,47	0,04
**ANGPTL4**	4,51	4,47	0,04
**CEP104**	11,34	11,30	0,04
**TMEM260**	10,97	10,92	0,04
**ESAM**	9,54	9,50	0,04
**MPHOSPH9**	12,56	12,52	0,04
**KDM3B**	13,88	13,84	0,04
**PEX2**	11,33	11,29	0,04
**C11orf80**	9,70	9,65	0,04
**ACBD7**	10,20	10,15	0,04
**PPP2R2A**	13,48	13,43	0,04
**MT-CYB**	17,59	17,55	0,04
**TGIF1**	11,41	11,36	0,04
**NRDC**	14,06	14,01	0,04
**SERPINE2**	11,18	11,13	0,04
**RAB34**	12,79	12,74	0,04
**CDK2**	13,23	13,19	0,04
**PTTG1**	12,83	12,79	0,04
**SERINC1**	12,35	12,31	0,04
**HEATR6**	9,87	9,83	0,04
**RASSF3**	12,18	12,14	0,04
**RTN2**	5,91	5,87	0,04
**DDX52**	12,41	12,36	0,04
**B4GAT1**	10,65	10,60	0,04
**AP3S2**	10,30	10,26	0,04
**PARG**	12,14	12,09	0,04
**PPP3CB**	12,20	12,16	0,04
**GID4**	10,83	10,79	0,04
**C16orf71**	3,83	3,79	0,04
**TRERF1**	11,99	11,94	0,04
**GGA2**	14,01	13,97	0,04
**TYRO3**	9,47	9,42	0,04
**ZNF692**	11,53	11,49	0,04
**TTC30A**	8,50	8,46	0,04
**TOR1AIP1**	12,11	12,06	0,04
**TATDN2**	12,96	12,92	0,05
**C5orf63**	6,10	6,06	0,05
**RPL5**	16,58	16,53	0,05
**CCDC138**	10,74	10,70	0,05
**SIMC1**	11,60	11,55	0,05
**GCC1**	11,37	11,33	0,05
**MAT2B**	13,55	13,51	0,05
**MTPN**	14,11	14,06	0,05
**SNRK**	11,85	11,81	0,05
**NQO1**	10,21	10,16	0,05
**PUDP**	11,17	11,12	0,05
**ZNF85**	11,24	11,19	0,05
**YTHDC1**	13,39	13,34	0,05
**ATP6V1G2**	6,64	6,59	0,05
**TENT4A**	12,56	12,52	0,05
**NUP205**	13,95	13,90	0,05
**MRS2**	10,78	10,73	0,05
**ABHD12**	11,54	11,50	0,05

**SPAG16**	8,76	8,71	0,05
**WDR44**	10,07	10,02	0,05
**DLG5**	10,94	10,89	0,05
**QSER1**	13,39	13,34	0,05
**BBS4**	9,36	9,31	0,05
**ALG11**	12,20	12,15	0,05
**VANGL2**	11,99	11,95	0,05
**TRIM68**	10,25	10,20	0,05
**MAK16**	13,09	13,04	0,05
**LRATD2**	9,64	9,59	0,05
**NAPEPLD**	10,53	10,48	0,05
**BBIP1**	9,13	9,08	0,05
**ZNF37A**	12,13	12,08	0,05
**LSM3**	12,46	12,41	0,05
**MCL1**	14,36	14,32	0,05
**RNF4**	12,74	12,69	0,05
**YES1**	10,28	10,23	0,05
**NDC80**	12,43	12,38	0,05
**EMC6**	10,66	10,62	0,05
**TATDN1**	11,02	10,97	0,05
**RTCA**	11,39	11,35	0,05
**SGSM3**	12,25	12,21	0,05
**TYW1B**	7,46	7,42	0,05
**NCBP1**	13,41	13,36	0,05
**HECA**	11,77	11,72	0,05
**S100A1**	8,62	8,58	0,05
**LDHB**	16,61	16,56	0,05
**SACM1L**	13,05	13,01	0,05
**NECTIN2**	11,19	11,15	0,05
**RABL2A**	9,58	9,53	0,05
**GHITM**	14,04	13,99	0,05
**FEZ2**	10,70	10,65	0,05
**FAM72A**	11,08	11,04	0,05
**TSNAXIP1**	6,95	6,90	0,05
**ARIH1**	13,49	13,44	0,05
**DCST1**	3,11	3,06	0,05
**ZNF32**	11,28	11,23	0,05
**C1QBP**	14,72	14,67	0,05
**STX7**	11,72	11,67	0,05
**GSR**	13,91	13,86	0,05
**LEMD3**	12,43	12,38	0,05
**GALNT7**	13,28	13,23	0,05
**DYNLRB1**	11,87	11,82	0,05
**BPGM**	11,04	10,99	0,05
**ITSN2**	11,96	11,92	0,05
**DPY19L4**	12,20	12,15	0,05
**DCP1B**	10,39	10,34	0,05
**HIP1R**	12,84	12,79	0,05
**LRRC56**	8,13	8,08	0,05
**PARD6A**	9,13	9,08	0,05
**TMUB2**	10,48	10,43	0,05
**PEX14**	9,63	9,58	0,05
**MRPL10**	11,45	11,40	0,05
**WDR37**	11,63	11,59	0,05
**RPSA**	16,96	16,91	0,05
**CBWD1**	12,29	12,25	0,05

**UBR1**	12,04	11,99	0,05
**EXOC2**	11,51	11,46	0,05
**NRBP1**	12,26	12,22	0,05
**HROB**	10,41	10,36	0,05
**RNPEPL1**	12,08	12,03	0,05
**EFTUD2**	13,96	13,92	0,05
**PSIP1**	14,10	14,05	0,05
**LRRCC1**	12,73	12,69	0,05
**LRRK2**	6,99	6,94	0,05
**CD59**	12,28	12,23	0,05
**DET1**	9,06	9,01	0,05
**CRYL1**	9,40	9,35	0,05
**AC019117.1**	5,25	5,20	0,05
**DHX32**	10,66	10,61	0,05
**PARP4**	13,05	13,00	0,05
**NEMP2**	10,59	10,54	0,05
**ZFP91**	13,74	13,69	0,05
**RBM7**	11,58	11,53	0,05
**ARPIN-AP3S2**	8,44	8,39	0,05
**STX1B**	8,31	8,26	0,05
**FSCN1**	13,10	13,05	0,05
**ANO10**	9,43	9,39	0,05
**LYG1**	7,07	7,02	0,05
**BTBD11**	9,64	9,59	0,05
**DAB2IP**	10,47	10,42	0,05
**PTGFRN**	11,63	11,58	0,05
**LGALS4**	4,71	4,67	0,05
**SLC48A1**	9,32	9,28	0,05
**FBXO36**	7,06	7,01	0,05
**CERS4**	10,26	10,21	0,05
**CTDSPL**	11,29	11,24	0,05
**ERAS**	1,96	1,91	0,05
**SUPT3H**	9,73	9,68	0,05
**PANX1**	11,50	11,45	0,05
**GXYLT1**	12,33	12,28	0,05
**MTFMT**	10,37	10,32	0,05
**ZNF70**	11,57	11,52	0,05
**VWCE**	11,87	11,82	0,05
**ZNF84**	13,02	12,97	0,05
**HIPK3**	12,58	12,53	0,05
**ESYT2**	13,82	13,77	0,05
**NBN**	13,62	13,57	0,05
**REV1**	11,90	11,85	0,05
**ING3**	11,33	11,28	0,05
**RNASEH2B**	13,86	13,81	0,05
**GTF2F2**	12,06	12,01	0,05
**ACADM**	13,15	13,10	0,05
**TGFBR2**	13,75	13,71	0,05
**SEC22B**	13,48	13,43	0,05
**WDR89**	11,71	11,66	0,05
**BOD1**	11,31	11,26	0,05
**ASS1**	12,57	12,52	0,05
**RAP1B**	14,28	14,23	0,05
**GRPEL2**	11,73	11,68	0,05
**MFF**	12,11	12,06	0,05
**CHFR**	12,02	11,97	0,05

**DNMT3B**	9,95	9,90	0,05
**PPFIBP1**	12,60	12,55	0,05
**DLST**	13,02	12,97	0,05
**MTMR10**	11,92	11,87	0,05
**CRBN**	11,49	11,44	0,05
**SH3RF1**	11,02	10,97	0,05
**PRDX1**	16,83	16,78	0,05
**PGAM1**	14,95	14,90	0,05
**PELO**	10,73	10,68	0,05
**TPT1**	17,06	17,01	0,05
**TAF11**	11,65	11,60	0,05
**ITGA8**	11,50	11,45	0,05
**ZNF224**	9,57	9,51	0,05
**SMAP2**	11,89	11,84	0,05
**ZNF16**	9,81	9,75	0,05
**GNPTAB**	11,55	11,50	0,05
**IQGAP3**	12,14	12,09	0,05
**PCSK6**	10,19	10,14	0,05
**ILDR2**	10,28	10,23	0,05
**RCL1**	11,19	11,14	0,05
**IFT80**	11,72	11,67	0,05
**WFS1**	9,99	9,94	0,05
**CAMK2G**	11,59	11,54	0,05
**RHEX**	9,49	9,44	0,05
**SLC43A1**	11,89	11,84	0,05
**SPDL1**	12,42	12,37	0,05
**TDG**	13,59	13,54	0,05
**MBIP**	10,52	10,47	0,05
**SETD3**	12,35	12,30	0,05
**NDUFB4**	12,81	12,76	0,05
**EIF3H**	15,12	15,07	0,05
**FERMT1**	12,24	12,19	0,05
**PKIG**	9,11	9,06	0,05
**LIPE**	10,01	9,96	0,05
**IDH3G**	11,83	11,77	0,05
**PSMD10**	11,89	11,84	0,05
**C16orf54**	10,47	10,42	0,05
**RARS1**	13,10	13,05	0,05
**KIF4A**	12,49	12,43	0,05
**SH3BGRL3**	12,58	12,53	0,05
**ZC3H14**	12,95	12,90	0,05
**RMC1**	11,23	11,18	0,05
**PIK3CD**	14,20	14,15	0,05
**ATP1B1**	8,90	8,85	0,05
**TEX10**	12,62	12,57	0,05
**MT1E**	4,02	3,97	0,05
**ADAMTS17**	9,03	8,98	0,05
**WDR70**	11,91	11,85	0,05
**MKI67**	15,84	15,79	0,05
**BRIX1**	12,89	12,84	0,05
**ANP32E**	14,88	14,83	0,05
**MCTS1**	12,50	12,45	0,05
**NOXA1**	2,18	2,13	0,05
**TBCD**	13,03	12,98	0,05
**MGA**	8,44	8,38	0,05
**RAB10**	13,82	13,77	0,05

**HNRNPR**	15,33	15,28	0,05
**C12orf4**	11,23	11,18	0,05
**FAM86B1**	10,09	10,04	0,05
**TMEM25**	8,83	8,78	0,05
**DCP1A**	12,79	12,74	0,05
**ATP5PO**	13,56	13,51	0,05
**CAST**	12,45	12,40	0,05
**ZNF436**	10,33	10,28	0,05
**PEMT**	10,36	10,30	0,05
**XRN2**	13,74	13,69	0,05
**POFUT1**	12,43	12,37	0,05
**UHRF1BP1**	12,23	12,17	0,05
**SLX4IP**	10,44	10,39	0,05
**PPP2CA**	14,02	13,97	0,05
**SETX**	13,93	13,88	0,05
**NRF1**	11,61	11,56	0,05
**VPREB3**	11,80	11,75	0,05
**MTF2**	13,24	13,19	0,05
**RPS5**	16,04	15,98	0,05
**TMEM177**	9,96	9,91	0,05
**APBB1IP**	14,35	14,30	0,05
**GGCT**	12,54	12,48	0,05
**MRPL45**	11,68	11,63	0,05
**DPY19L1**	12,11	12,05	0,05
**AK3**	12,07	12,01	0,05
**TTC27**	11,96	11,90	0,05
**RNF216**	12,67	12,61	0,05
**C2CD3**	12,21	12,15	0,05
**DDX18**	14,26	14,21	0,05
**GOLM2**	12,40	12,35	0,05
**BCL11A**	13,43	13,38	0,05
**DCAF7**	14,31	14,26	0,05
**LRRC34**	9,49	9,43	0,05
**SSU72**	13,04	12,99	0,05
**DNTTIP2**	13,34	13,29	0,05
**PDHX**	11,48	11,42	0,05
**HADHA**	13,91	13,86	0,05
**THAP1**	10,33	10,28	0,05
**EPC1**	11,97	11,92	0,05
**RFNG**	9,47	9,41	0,05
**TUBGCP4**	12,67	12,62	0,05
**FNIP2**	11,61	11,56	0,05
**OTUD7A**	4,98	4,92	0,05
**RPS6KA1**	12,70	12,64	0,05
**ZSWIM7**	10,31	10,26	0,05
**SIRPA**	10,71	10,66	0,05
**POLR1D**	12,83	12,78	0,05
**CPZ**	4,05	4,00	0,05
**RABGAP1L**	13,00	12,95	0,05
**DCLRE1B**	11,69	11,64	0,05
**NUP188**	14,05	14,00	0,05
**HDAC3**	12,58	12,53	0,05
**CAAP1**	11,14	11,09	0,05
**RPA2**	12,67	12,61	0,05
**AC004687.2**	5,29	5,24	0,05
**CCNB2**	12,67	12,62	0,05

**ELOVL5**	15,02	14,97	0,05
**TRIAP1**	11,75	11,70	0,05
**N4BP1**	11,64	11,59	0,05
**POLR3A**	12,43	12,38	0,05
**STK39**	14,24	14,18	0,05
**SCAF11**	14,47	14,42	0,05
**TMEM126B**	11,35	11,30	0,05
**AC069544.1**	11,96	11,91	0,05
**BEND5**	9,19	9,14	0,05
**RPL26**	16,11	16,06	0,05
**FAR1**	13,59	13,54	0,05
**CCDC58**	11,32	11,26	0,05
**DISP1**	8,32	8,27	0,05
**CYTH4**	8,91	8,85	0,05
**ARHGAP19**	13,30	13,25	0,05
**SLC38A1**	15,32	15,26	0,05
**CPOX**	11,69	11,64	0,05
**KIF3B**	11,32	11,27	0,05
**ZNF280B**	9,27	9,22	0,05
**C18orf54**	11,18	11,13	0,05
**SLC25A12**	11,05	10,99	0,05
**CHD2**	13,35	13,30	0,05
**B3GALNT2**	12,38	12,33	0,05
**ARL1**	12,08	12,02	0,05
**BANP**	9,21	9,15	0,05
**HSD17B12**	12,91	12,86	0,05
**FAAP24**	9,07	9,02	0,05
**HDLBP**	14,39	14,34	0,05
**GBE1**	12,64	12,59	0,05
**ZNF780B**	11,40	11,34	0,05
**BRK1**	12,89	12,83	0,05
**NFYB**	12,53	12,47	0,05
**VAV3**	13,91	13,86	0,05
**SLC20A2**	11,60	11,55	0,05
**MPPE1**	9,88	9,82	0,05
**SMS**	14,04	13,98	0,05
**RTEL1**	11,16	11,10	0,05
**ZFP3**	9,49	9,43	0,05
**SKI**	10,15	10,10	0,05
**NDUFS1**	13,75	13,70	0,05
**CCNY**	13,15	13,10	0,05
**CMIP**	12,74	12,68	0,05
**CENPU**	12,97	12,91	0,05
**GABPB1**	12,63	12,58	0,06
**ZNF470**	10,84	10,78	0,06
**RFK**	11,98	11,93	0,06
**OBSL1**	10,70	10,65	0,06
**ORC1**	12,44	12,39	0,06
**ADAM15**	11,73	11,68	0,06
**DCPS**	11,78	11,72	0,06
**PDLIM1**	14,43	14,38	0,06
**HSPB11**	11,33	11,27	0,06
**LDLR**	12,26	12,21	0,06
**CUTC**	11,58	11,52	0,06
**CYTH3**	11,58	11,52	0,06
**EIF1AX**	14,85	14,80	0,06

**BCAM**	8,92	8,87	0,06
**DERL1**	12,59	12,54	0,06
**TCF12**	13,76	13,71	0,06
**PABPC1**	18,22	18,16	0,06
**GUCY1B1**	7,59	7,54	0,06
**SUCLG1**	12,41	12,35	0,06
**NREP**	14,30	14,24	0,06
**GRB10**	13,45	13,40	0,06
**ZNF644**	13,00	12,95	0,06
**FOXJ2**	11,43	11,37	0,06
**DTX2**	9,46	9,40	0,06
**MDFI**	11,91	11,85	0,06
**VAPB**	12,00	11,94	0,06
**LOXL1**	3,83	3,78	0,06
**SCP2**	12,53	12,47	0,06
**TEX2**	11,03	10,97	0,06
**MEAF6**	12,50	12,45	0,06
**SIRT5**	10,87	10,82	0,06
**HELQ**	10,16	10,11	0,06
**FAM189B**	11,49	11,44	0,06
**FBXO38**	11,81	11,75	0,06
**TXNDC5**	14,01	13,95	0,06
**HADH**	13,16	13,10	0,06
**C14orf93**	9,67	9,61	0,06
**IFT172**	11,53	11,47	0,06
**APOL6**	11,16	11,11	0,06
**VHL**	13,51	13,45	0,06
**EGR1**	6,66	6,61	0,06
**RNF207**	6,55	6,49	0,06
**NFKBIA**	11,98	11,92	0,06
**DENND11**	11,28	11,22	0,06
**PCBP3**	10,53	10,48	0,06
**XPO5**	13,76	13,71	0,06
**ESPL1**	12,46	12,40	0,06
**FAM210B**	10,73	10,68	0,06
**AGK**	11,76	11,70	0,06
**GRINA**	11,84	11,78	0,06
**FAM104A**	11,14	11,09	0,06
**KIAA0232**	12,79	12,74	0,06
**AP2B1**	14,23	14,17	0,06
**NOTCH1**	12,65	12,60	0,06
**LRRC8D**	12,20	12,14	0,06
**PAAF1**	10,49	10,43	0,06
**CUL4B**	12,34	12,29	0,06
**MARK1**	10,79	10,74	0,06
**CFAP70**	8,44	8,38	0,06
**SDAD1**	12,95	12,89	0,06
**ATP5PB**	14,14	14,08	0,06
**ZDHHC9**	10,64	10,58	0,06
**TASOR2**	13,15	13,09	0,06
**DIMT1**	11,99	11,93	0,06
**MIA3**	12,78	12,73	0,06
**MARS2**	11,85	11,79	0,06
**PIGP**	9,74	9,68	0,06
**FGFR1OP2**	11,89	11,83	0,06
**RALGPS1**	9,77	9,72	0,06

**WDCP**	11,35	11,29	0,06
**BBS12**	7,05	7,00	0,06
**HAUS1**	12,48	12,43	0,06
**ACBD5**	11,33	11,28	0,06
**FBXO34**	11,72	11,66	0,06
**RCN1**	13,47	13,41	0,06
**ZCCHC8**	12,06	12,00	0,06
**NUCKS1**	16,19	16,13	0,06
**FECH**	11,01	10,95	0,06
**GAPVD1**	12,98	12,92	0,06
**UBE2B**	11,77	11,71	0,06
**NEPRO**	12,09	12,03	0,06
**CCZ1**	11,48	11,43	0,06
**LZIC**	12,02	11,96	0,06
**DPYD**	9,52	9,46	0,06
**FANCA**	13,29	13,23	0,06
**PFKFB4**	11,14	11,08	0,06
**C1orf174**	11,44	11,39	0,06
**KCNJ16**	9,05	8,99	0,06
**PHF7**	8,77	8,71	0,06
**RUNX1**	15,20	15,14	0,06
**RAB14**	13,52	13,46	0,06
**KDM4C**	11,28	11,22	0,06
**BCL2**	13,51	13,45	0,06
**MAPK1**	13,64	13,58	0,06
**ZNF529**	11,57	11,51	0,06
**ZAP70**	11,78	11,72	0,06
**SHF**	9,78	9,73	0,06
**RAC1**	14,26	14,20	0,06
**VMO1**	1,61	1,56	0,06
**FAM111B**	13,61	13,56	0,06
**KPNA2**	14,76	14,70	0,06
**MRPL58**	10,66	10,60	0,06
**GLI1**	6,77	6,71	0,06
**ADAM17**	11,84	11,78	0,06
**VCL**	13,81	13,76	0,06
**NLRC3**	9,72	9,66	0,06
**MARCHF5**	12,15	12,10	0,06
**UNC13D**	11,24	11,18	0,06
**DCAF6**	11,54	11,49	0,06
**ASIC3**	7,67	7,62	0,06
**LYSMD1**	8,83	8,77	0,06
**TAOK3**	11,87	11,81	0,06
**CASP10**	9,48	9,42	0,06
**VKORC1L1**	13,09	13,03	0,06
**SEPTIN3**	11,16	11,10	0,06
**ATG12**	12,04	11,98	0,06
**MYL12A**	13,19	13,14	0,06
**MGAT5B**	11,20	11,14	0,06
**ATL3**	12,93	12,87	0,06
**FAM133B**	12,55	12,49	0,06
**MBTPS1**	13,18	13,12	0,06
**CRY1**	11,46	11,40	0,06
**GGT1**	6,07	6,01	0,06
**SMG7**	13,68	13,62	0,06
**GBP1**	9,84	9,78	0,06

**GATC**	12,30	12,25	0,06
**TSPO**	11,76	11,70	0,06
**ZBTB44**	13,29	13,23	0,06
**LRIT3**	6,77	6,72	0,06
**NEK1**	10,46	10,40	0,06
**LRP1**	10,03	9,97	0,06
**CNIH4**	11,07	11,02	0,06
**UBQLN1**	14,12	14,06	0,06
**INTS10**	13,10	13,05	0,06
**POLR2G**	12,10	12,04	0,06
**TRAF3IP1**	10,66	10,60	0,06
**TRAPPC3**	11,54	11,48	0,06
**KIF20A**	12,66	12,60	0,06
**RNF115**	12,24	12,18	0,06
**URB2**	12,93	12,88	0,06
**IFT140**	10,64	10,58	0,06
**FNDC3A**	12,06	12,00	0,06
**TRAPPC2B**	9,03	8,97	0,06
**PAQR7**	8,70	8,64	0,06
**LAMC3**	1,97	1,91	0,06
**PLPP6**	9,59	9,53	0,06
**RIPOR1**	12,02	11,96	0,06
**KDELR3**	5,40	5,35	0,06
**SPEN**	14,04	13,98	0,06
**GPN3**	11,59	11,53	0,06
**CEP57**	12,75	12,69	0,06
**BTNL9**	9,91	9,85	0,06
**SMC4**	15,44	15,38	0,06
**DYNC1H1**	15,15	15,09	0,06
**PARN**	12,60	12,54	0,06
**BAG5**	12,73	12,67	0,06
**RAB2A**	12,78	12,72	0,06
**PRDX3**	13,83	13,77	0,06
**HERC1**	13,05	12,99	0,06
**CSNK1G2**	12,66	12,60	0,06
**ALDH3B1**	10,02	9,96	0,06
**NINJ1**	11,40	11,34	0,06
**RPN2**	13,98	13,92	0,06
**ME2**	13,69	13,63	0,06
**CEP83**	10,84	10,78	0,06
**AC092835.1**	8,08	8,02	0,06
**ANKMY2**	10,85	10,79	0,06
**ZBTB45**	10,16	10,10	0,06
**AP3M2**	12,43	12,37	0,06
**MT-ND4**	18,50	18,44	0,06
**SRRM1**	14,00	13,94	0,06
**PCDHB12**	4,64	4,58	0,06
**LHFPL5**	9,74	9,68	0,06
**GSTA4**	9,43	9,37	0,06
**LTBP1**	10,98	10,92	0,06
**KMT2C**	13,20	13,14	0,06
**PRPS1**	13,04	12,98	0,06
**TRMT61B**	10,86	10,80	0,06
**CLPTM1L**	13,17	13,11	0,06
**COA1**	11,43	11,36	0,06
**PKN2**	12,41	12,35	0,06

**PLEK**	11,89	11,83	0,06
**CHMP2B**	11,80	11,74	0,06
**ENTPD1**	12,79	12,73	0,06
**NPC1**	12,20	12,14	0,06
**BACH2**	13,09	13,03	0,06
**KLHDC2**	11,47	11,41	0,06
**RABEP1**	13,33	13,26	0,06
**SPRYD7**	10,65	10,59	0,06
**IPP**	8,67	8,61	0,06
**PDP1**	11,09	11,03	0,06
**NDRG3**	11,55	11,48	0,06
**KAT7**	12,55	12,49	0,06
**EI24**	13,18	13,12	0,06
**QRICH1**	13,37	13,31	0,06
**ZNF330**	12,53	12,47	0,06
**EOLA1**	10,02	9,96	0,06
**CCNA2**	13,53	13,47	0,06
**SNX5**	14,16	14,09	0,06
**KCNJ12**	11,80	11,74	0,06
**NUSAP1**	14,49	14,42	0,06
**PELI2**	12,11	12,05	0,06
**GPR107**	11,85	11,79	0,06
**STAT5A**	10,62	10,55	0,06
**SLC50A1**	10,58	10,52	0,06
**NHLRC2**	12,30	12,24	0,06
**MFSD10**	10,54	10,48	0,06
**RHOBTB1**	11,40	11,34	0,06
**AHI1**	11,70	11,64	0,06
**CUL2**	10,08	10,02	0,06
**HPS4**	15,88	15,81	0,06
**TBC1D8**	8,59	8,53	0,06
**ADAR**	15,43	15,37	0,06
**SEC24A**	11,73	11,67	0,06
**EEF2K**	12,56	12,49	0,06
**FASTKD5**	11,57	11,51	0,06
**COPRS**	11,28	11,22	0,06
**MGAT2**	12,69	12,63	0,06
**ANKRD13C**	11,49	11,43	0,06
**CLPX**	12,85	12,78	0,06
**SPOP**	12,46	12,39	0,06
**MMUT**	11,37	11,31	0,06
**PDGFRB**	12,91	12,85	0,06
**TMEM41A**	11,26	11,20	0,06
**C1QTNF5**	4,97	4,91	0,06
**MSL3**	11,82	11,75	0,06
**C12orf45**	11,19	11,13	0,06
**WEE1**	13,07	13,00	0,06
**IPO9**	14,08	14,02	0,06
**AKT1S1**	10,74	10,68	0,06
**RAD54B**	10,93	10,87	0,06
**SH2D4A**	9,16	9,09	0,06
**POP5**	9,94	9,87	0,06
**RPF2**	12,66	12,60	0,06
**WDR59**	11,84	11,78	0,06
**CDK6**	15,63	15,56	0,06
**BCORL1**	11,46	11,40	0,06

**SAR1A**	13,53	13,46	0,06
**TEX30**	10,78	10,72	0,06
**MPP4**	1,62	1,55	0,06
**CAPRIN1**	15,55	15,48	0,06
**YAF2**	11,17	11,10	0,06
**OTULIN**	12,57	12,51	0,06
**SORT1**	8,82	8,75	0,06
**COPG2**	11,31	11,25	0,06
**TCERG1**	14,11	14,04	0,06
**FLRT1**	8,33	8,27	0,06
**AC005832.4**	11,10	11,04	0,06
**DPH3**	10,72	10,66	0,06
**MFSD4B**	11,40	11,33	0,06
**LBH**	11,59	11,53	0,06
**GART**	14,08	14,01	0,06
**JMY**	10,56	10,50	0,06
**CLTCL1**	10,95	10,89	0,06
**FGF9**	13,10	13,04	0,06
**IL21R**	8,62	8,56	0,06
**NUF2**	12,47	12,40	0,06
**NFXL1**	10,81	10,74	0,06
**PKIA**	11,64	11,58	0,06
**USP46**	11,73	11,66	0,06
**CDK2AP1**	14,23	14,16	0,06
**PPTC7**	12,66	12,60	0,06
**ALG8**	11,75	11,69	0,06
**TM9SF2**	13,40	13,34	0,06
**TTC1**	12,43	12,37	0,06
**TMED5**	12,71	12,65	0,06
**FNTA**	13,32	13,25	0,06
**CD93**	9,78	9,72	0,06
**HUWE1**	14,84	14,78	0,06
**MAPRE3**	5,07	5,01	0,06
**SLC7A3**	10,05	9,99	0,06
**BROX**	11,93	11,87	0,06
**ZNF611**	10,50	10,44	0,06
**NARS1**	14,23	14,17	0,07
**CLASP1**	13,02	12,96	0,07
**LGMN**	9,89	9,83	0,07
**CD9**	14,87	14,81	0,07
**SS18**	12,61	12,55	0,07
**ZCCHC7**	14,91	14,85	0,07
**UBE2V1**	13,22	13,16	0,07
**LNX1**	7,57	7,50	0,07
**ADAMTS6**	7,36	7,30	0,07
**SEMA4D**	12,41	12,34	0,07
**ARPC1A**	12,94	12,87	0,07
**ZNF419**	10,23	10,17	0,07
**NEMP1**	12,99	12,93	0,07
**RBSN**	10,94	10,88	0,07
**NOA1**	11,57	11,50	0,07
**INKA2**	8,93	8,87	0,07
**MAD2L1BP**	11,01	10,94	0,07
**SLC9A6**	10,73	10,66	0,07
**ANXA5**	13,71	13,64	0,07
**G2E3**	12,39	12,32	0,07

**CSNK1A1**	13,91	13,84	0,07
**DUS2**	10,46	10,39	0,07
**MTRES1**	10,46	10,40	0,07
**CTPS2**	11,31	11,24	0,07
**MAP3K7**	11,99	11,92	0,07
**HSD17B4**	13,09	13,02	0,07
**TMX2**	12,60	12,54	0,07
**CHST15**	13,83	13,77	0,07
**C6orf120**	11,28	11,22	0,07
**PPRC1**	13,42	13,35	0,07
**DAB2**	10,36	10,30	0,07
**GPD1L**	11,81	11,74	0,07
**TRA2A**	12,71	12,64	0,07
**ERAL1**	12,01	11,94	0,07
**VRK3**	11,19	11,12	0,07
**DDX17**	15,90	15,83	0,07
**SCNN1D**	8,58	8,51	0,07
**PRPF38A**	13,64	13,57	0,07
**TRAIP**	10,70	10,63	0,07
**S100PBP**	12,00	11,94	0,07
**CNOT8**	13,20	13,14	0,07
**SMAP1**	12,31	12,24	0,07
**ZNF337**	11,74	11,67	0,07
**ASNS**	12,48	12,41	0,07
**WRAP53**	11,12	11,05	0,07
**EDA2R**	9,26	9,19	0,07
**ATP2A2**	14,75	14,69	0,07
**HIVEP3**	10,58	10,52	0,07
**SRP19**	11,90	11,83	0,07
**MED17**	11,87	11,80	0,07
**GTF2E2**	12,15	12,08	0,07
**ANKRD33B**	12,43	12,37	0,07
**SLBP**	13,98	13,91	0,07
**SEC24B**	12,31	12,25	0,07
**COPG1**	13,23	13,17	0,07
**YEATS4**	12,29	12,22	0,07
**CDK1**	13,68	13,62	0,07
**PXK**	12,74	12,67	0,07
**GOSR2**	12,34	12,28	0,07
**PTPRG**	12,31	12,24	0,07
**ZFYVE26**	11,28	11,21	0,07
**BTK**	14,06	13,99	0,07
**CIP2A**	12,51	12,45	0,07
**FANCL**	11,07	11,00	0,07
**EIF4E**	13,65	13,58	0,07
**ROBO4**	5,41	5,34	0,07
**FUNDC1**	10,89	10,82	0,07
**RBMX**	15,71	15,65	0,07
**EMC2**	11,90	11,84	0,07
**GNL3**	14,48	14,41	0,07
**WWP1**	11,98	11,91	0,07
**OSTC**	12,61	12,54	0,07
**SLC9B2**	11,85	11,78	0,07
**FAM220A**	10,69	10,62	0,07
**ARHGEF11**	12,46	12,40	0,07
**SH3BP1**	11,40	11,33	0,07

**XPNPEP1**	12,34	12,27	0,07
**SERINC5**	13,71	13,64	0,07
**AP3B1**	12,70	12,63	0,07
**FHOD3**	10,38	10,31	0,07
**KLHDC10**	11,86	11,79	0,07
**PRKAG2**	10,73	10,67	0,07
**PCID2**	12,27	12,20	0,07
**KIZ**	10,08	10,01	0,07
**GINS1**	13,18	13,12	0,07
**LRP11**	10,83	10,76	0,07
**ADPRHL1**	6,36	6,30	0,07
**RDX**	12,31	12,24	0,07
**RASA1**	11,95	11,88	0,07
**GALNT12**	9,54	9,47	0,07
**HS3ST3B1**	12,06	12,00	0,07
**TNFAIP8L1**	10,62	10,55	0,07
**PTPN7**	7,86	7,79	0,07
**CNOT2**	13,02	12,95	0,07
**RAI14**	12,19	12,12	0,07
**POLR1E**	12,30	12,23	0,07
**MRPL48**	11,64	11,57	0,07
**KIF14**	12,74	12,67	0,07
**FOXK2**	13,24	13,17	0,07
**KCTD2**	10,93	10,86	0,07
**PDE12**	12,67	12,60	0,07
**ADO**	12,25	12,19	0,07
**SCFD2**	11,40	11,33	0,07
**MPI**	11,32	11,25	0,07
**ZNF319**	10,33	10,26	0,07
**ANXA11**	12,94	12,88	0,07
**KCTD13**	11,08	11,01	0,07
**TBC1D22B**	10,28	10,21	0,07
**NDFIP1**	11,08	11,01	0,07
**PARPBP**	11,58	11,51	0,07
**CENPN**	12,37	12,30	0,07
**USP12**	11,65	11,58	0,07
**TTC4**	11,92	11,85	0,07
**LARS2**	12,47	12,40	0,07
**CDC5L**	13,16	13,09	0,07
**TSEN2**	11,56	11,50	0,07
**AC124312.1**	7,20	7,13	0,07
**SEC23IP**	12,74	12,67	0,07
**TASP1**	9,68	9,61	0,07
**CLP1**	10,90	10,83	0,07
**FRG1**	12,48	12,41	0,07
**TSHZ3**	9,22	9,15	0,07
**BICDL1**	8,78	8,71	0,07
**H2BC14**	3,28	3,21	0,07
**TUBB2B**	3,28	3,21	0,07
**FRMD7**	3,28	3,21	0,07
**PSCA**	3,28	3,21	0,07
**FAM180B**	3,28	3,21	0,07
**AC007906.2**	3,28	3,21	0,07
**RNF227**	8,51	8,44	0,07
**FANCF**	11,59	11,52	0,07
**CTBP2**	13,46	13,39	0,07

**MSH2**	13,69	13,62	0,07
**TRIM72**	8,10	8,03	0,07
**SERF1B**	10,56	10,49	0,07
**PPID**	12,20	12,13	0,07
**NIP7**	13,08	13,01	0,07
**DCTN4**	12,18	12,11	0,07
**THG1L**	9,65	9,58	0,07
**LRRC1**	10,93	10,86	0,07
**GUCY1A1**	9,91	9,84	0,07
**XBP1**	14,24	14,17	0,07
**ACTR10**	11,76	11,69	0,07
**MRPS16**	13,02	12,95	0,07
**ZNF354A**	11,27	11,20	0,07
**CCNE2**	12,47	12,40	0,07
**FHL3**	10,22	10,15	0,07
**SNAPC4**	6,66	6,59	0,07
**SMN1**	12,88	12,81	0,07
**CISD1**	11,44	11,37	0,07
**AC010323.1**	8,55	8,48	0,07
**DPH6**	9,05	8,98	0,07
**ZBED5**	12,41	12,34	0,07
**CCDC74A**	9,11	9,04	0,07
**KANSL1**	12,59	12,52	0,07
**EPB41**	14,06	13,99	0,07
**RAB3A**	8,73	8,66	0,07
**BRI3BP**	13,21	13,14	0,07
**TUBA4A**	8,42	8,35	0,07
**SMURF2**	11,33	11,26	0,07
**C10orf88**	10,77	10,70	0,07
**API5**	14,39	14,32	0,07
**EPAS1**	11,48	11,40	0,07
**PTDSS1**	14,03	13,96	0,07
**OTUD5**	11,60	11,53	0,07
**AHCYL1**	13,30	13,23	0,07
**WDR62**	12,30	12,23	0,07
**GK**	11,48	11,41	0,07
**SLC35A5**	11,44	11,37	0,07
**C1QTNF1**	5,64	5,56	0,07
**DRG1**	13,01	12,94	0,07
**TLE4**	14,14	14,07	0,07
**ZNF638**	13,88	13,80	0,07
**TARS2**	11,63	11,56	0,07
**CORO1C**	12,82	12,75	0,07
**GATA6**	9,28	9,21	0,07
**CBX3**	15,39	15,32	0,07
**EIF4G2**	16,86	16,78	0,07
**MCM6**	14,69	14,62	0,07
**VPS4B**	12,87	12,80	0,07
**HADHB**	12,25	12,18	0,07
**TXNL4B**	11,24	11,17	0,07
**CCDC28A**	9,87	9,79	0,07
**ACOT11**	8,05	7,98	0,07
**ZNF649**	10,57	10,50	0,07
**ZNF124**	10,89	10,82	0,07
**NADK2**	12,43	12,36	0,07
**SEC31A**	13,57	13,50	0,07

**WAC**	13,65	13,58	0,07
**SHLD2**	12,51	12,44	0,07
**GPR19**	6,68	6,61	0,07
**FAM71C**	5,44	5,37	0,07
**PTPRD**	9,83	9,75	0,07
**LAMA5**	11,19	11,12	0,07
**COG7**	10,78	10,71	0,07
**TUG1**	14,48	14,41	0,07
**ZNF600**	8,97	8,90	0,07
**TRIM44**	14,06	13,99	0,07
**TRIM5**	10,91	10,84	0,07
**TMEM68**	11,63	11,56	0,07
**IRF1**	12,74	12,67	0,07
**PYURF**	12,70	12,63	0,07
**MBLAC2**	10,82	10,75	0,07
**RHOH**	10,50	10,43	0,07
**ASPSCR1**	10,08	10,01	0,07
**SPAST**	11,90	11,82	0,07
**GABRB2**	9,14	9,06	0,07
**PPP2R5C**	14,14	14,07	0,07
**ACTR6**	11,44	11,37	0,07
**CRISPLD1**	11,73	11,66	0,07
**MACF1**	13,29	13,22	0,07
**WTAP**	13,57	13,49	0,07
**PTER**	11,50	11,43	0,07
**PLCL2**	11,23	11,16	0,07
**ZNF318**	11,89	11,82	0,07
**DDX39B**	14,87	14,80	0,07
**SLC35B3**	10,07	10,00	0,07
**TBC1D14**	14,09	14,02	0,07
**PARP14**	13,54	13,46	0,07
**YY1**	14,03	13,95	0,07
**HERC5**	10,24	10,17	0,07
**MYO7A**	8,80	8,73	0,07
**OSTM1**	10,82	10,75	0,07
**ACTN1**	12,21	12,14	0,07
**TIA1**	14,09	14,02	0,07
**MAN1A1**	10,95	10,88	0,07
**ZNF852**	9,13	9,06	0,07
**PSMA2**	13,76	13,68	0,07
**LARP7**	12,44	12,37	0,07
**BAZ1A**	14,53	14,46	0,07
**ZNF225**	9,74	9,66	0,07
**TAF12**	9,34	9,27	0,07
**ZNF302**	12,04	11,97	0,07
**ZNF33B**	11,69	11,61	0,07
**LRRC3B**	12,50	12,42	0,07
**YTHDF2**	13,78	13,71	0,07
**RPRD2**	12,27	12,20	0,07
**C1orf226**	4,20	4,13	0,07
**SLC6A3**	4,20	4,13	0,07
**TGFA**	4,20	4,13	0,07
**LDHAL6A**	4,20	4,13	0,07
**NOTCH4**	6,69	6,62	0,07
**CLPB**	11,98	11,90	0,07
**KLHL9**	12,57	12,49	0,07

**GTF2I**	15,39	15,31	0,07
**CCDC191**	8,63	8,56	0,07
**RNF168**	12,58	12,51	0,07
**TMEM87A**	11,78	11,70	0,07
**PPWD1**	12,15	12,08	0,07
**NDUFV3**	11,18	11,11	0,07
**POLR3C**	12,60	12,53	0,07
**COMMD1**	6,74	6,66	0,07
**MRPS35**	13,00	12,93	0,07
**TTL**	12,85	12,78	0,07
**ARCN1**	13,65	13,58	0,07
**ACAT1**	12,78	12,70	0,07
**UBQLN2**	12,60	12,53	0,07
**CAT**	13,09	13,02	0,07
**AP4E1**	11,79	11,71	0,07
**TXN**	13,91	13,84	0,07
**NFATC1**	10,68	10,61	0,07
**GRID1**	5,66	5,59	0,07
**MBOAT2**	11,40	11,32	0,07
**DHFR**	14,78	14,71	0,07
**DDI2**	13,48	13,41	0,07
**C2orf42**	9,71	9,64	0,07
**SLC25A35**	9,29	9,22	0,07
**LRRC23**	8,79	8,72	0,07
**ETFRF1**	8,98	8,90	0,07
**LYPLA1**	14,01	13,94	0,07
**PKP4**	12,46	12,38	0,07
**RRAS2**	10,06	9,98	0,07
**BRD3OS**	10,57	10,50	0,07
**SEL1L**	13,33	13,26	0,07
**ALDH5A1**	13,45	13,38	0,07
**TMPO**	15,75	15,67	0,07
**VIPAS39**	10,40	10,32	0,07
**SMIM4**	9,17	9,09	0,07
**STYX**	11,15	11,07	0,07
**SLCO3A1**	8,60	8,53	0,07
**ELOVL3**	4,76	4,69	0,07
**SLC35F1**	4,76	4,69	0,07
**RASAL3**	10,32	10,24	0,07
**SALL2**	10,89	10,81	0,07
**AREL1**	12,29	12,21	0,07
**EXOSC6**	12,79	12,71	0,07
**PCMTD2**	12,05	11,97	0,08
**CDKN3**	11,50	11,43	0,08
**CAVIN4**	9,06	8,99	0,08
**SIRT1**	12,19	12,12	0,08
**STXBP1**	10,32	10,24	0,08
**TMEM131L**	14,90	14,82	0,08
**CRTC3**	11,18	11,11	0,08
**THAP6**	9,76	9,68	0,08
**NRG2**	5,16	5,09	0,08
**FRMD3**	5,16	5,09	0,08
**MAP1B**	13,48	13,41	0,08
**ZFP36**	10,14	10,07	0,08
**SAP18**	13,64	13,56	0,08
**MYCBP2**	13,96	13,88	0,08

**MED13**	13,11	13,03	0,08
**C4orf19**	2,83	2,75	0,08
**SEPTIN2**	14,56	14,49	0,08
**GSE1**	13,00	12,92	0,08
**GOPC**	12,91	12,83	0,08
**RBM48**	10,88	10,80	0,08
**TBC1D1**	13,36	13,28	0,08
**CAP2**	9,66	9,59	0,08
**EEF1A1**	20,25	20,17	0,08
**TCEAL9**	11,27	11,19	0,08
**CRYZL1**	10,83	10,76	0,08
**UROS**	12,04	11,97	0,08
**UFD1**	12,25	12,17	0,08
**CCDC120**	9,25	9,17	0,08
**APBA2**	10,63	10,56	0,08
**PTGES3**	15,70	15,62	0,08
**HAUS3**	11,83	11,75	0,08
**ADAM33**	7,79	7,71	0,08
**SNX19**	12,14	12,07	0,08
**PMM2**	12,05	11,98	0,08
**MYO1F**	7,74	7,67	0,08
**GOLGA4**	12,38	12,31	0,08
**ZBTB10**	12,55	12,48	0,08
**AC011511.4**	10,03	9,96	0,08
**USP48**	13,24	13,17	0,08
**ABHD5**	10,48	10,41	0,08
**TSG101**	11,19	11,11	0,08
**WDR11**	12,25	12,18	0,08
**DPCD**	7,85	7,77	0,08
**RFC1**	13,68	13,61	0,08
**SNX17**	12,27	12,20	0,08
**PMS2**	12,33	12,25	0,08
**RPS6KL1**	9,87	9,79	0,08
**ZNF764**	10,61	10,53	0,08
**PDZD7**	9,52	9,44	0,08
**NIPSNAP3A**	11,53	11,45	0,08
**ENO2**	11,12	11,04	0,08
**NEXN**	10,41	10,33	0,08
**RBM23**	13,02	12,94	0,08
**CELSR1**	11,18	11,10	0,08
**SLC35G1**	8,91	8,84	0,08
**RBM18**	11,32	11,24	0,08
**RSL24D1**	13,98	13,90	0,08
**BRAF**	11,89	11,82	0,08
**AJUBA**	11,26	11,18	0,08
**TMEM200A**	5,82	5,74	0,08
**TAF5**	11,05	10,98	0,08
**VWC2**	8,85	8,78	0,08
**CETN3**	11,04	10,97	0,08
**ZMAT1**	10,42	10,34	0,08
**NFKBIZ**	8,47	8,39	0,08
**GCFC2**	10,90	10,82	0,08
**TMEM203**	11,19	11,11	0,08
**LCP2**	11,56	11,49	0,08
**UBC**	15,74	15,67	0,08
**MTCH2**	13,38	13,31	0,08

**THSD1**	7,80	7,72	0,08
**SMAD3**	12,09	12,01	0,08
**TMEM243**	11,31	11,23	0,08
**UVRAG**	11,00	10,92	0,08
**NCAPG2**	13,28	13,20	0,08
**CGGBP1**	14,03	13,96	0,08
**RACK1**	16,64	16,57	0,08
**TMEM268**	12,07	11,99	0,08
**JAKMIP2**	10,18	10,10	0,08
**CALM2**	15,64	15,57	0,08
**NOTCH2**	13,20	13,12	0,08
**NUDT3**	13,66	13,58	0,08
**CCT2**	15,39	15,31	0,08
**METTL16**	12,74	12,66	0,08
**GPRC5B**	3,28	3,21	0,08
**MSRA**	11,38	11,30	0,08
**SAYSD1**	9,52	9,44	0,08
**ZNF572**	9,18	9,10	0,08
**GOLPH3**	12,88	12,80	0,08
**NAA60**	11,44	11,36	0,08
**CENPK**	11,96	11,89	0,08
**GJC1**	13,11	13,03	0,08
**HCCS**	10,78	10,71	0,08
**ZFAND5**	14,05	13,97	0,08
**ACOX1**	11,56	11,48	0,08
**RPAP3**	12,23	12,16	0,08
**NMB**	8,03	7,95	0,08
**HSD17B7**	5,80	5,72	0,08
**DHX15**	15,08	15,00	0,08
**RNMT**	12,71	12,64	0,08
**ARL5A**	12,87	12,79	0,08
**ANAPC5**	13,81	13,73	0,08
**TCFL5**	11,82	11,74	0,08
**LARS1**	14,77	14,69	0,08
**DDX59**	10,91	10,83	0,08
**CTNNBIP1**	10,95	10,87	0,08
**NOTUM**	4,74	4,66	0,08
**SDC1**	11,27	11,20	0,08
**SKAP2**	5,77	5,69	0,08
**PTPN3**	9,95	9,87	0,08
**EMC7**	11,17	11,09	0,08
**NARF**	12,62	12,54	0,08
**PACRGL**	10,99	10,91	0,08
**SUMF2**	12,29	12,21	0,08
**MCTS2P**	8,76	8,68	0,08
**ACTR8**	11,30	11,22	0,08
**DTL**	13,67	13,59	0,08
**TERF1**	12,30	12,22	0,08
**BTBD10**	11,50	11,42	0,08
**CLDND1**	11,77	11,69	0,08
**FARSB**	13,50	13,42	0,08
**GLYATL1**	2,10	2,02	0,08
**BLM**	12,95	12,87	0,08
**PRDM4**	11,66	11,58	0,08
**NID2**	14,47	14,39	0,08
**XRCC5**	16,11	16,03	0,08

**TBC1D17**	9,52	9,44	0,08
**PIGL**	9,22	9,14	0,08
**SLC25A43**	10,12	10,04	0,08
**COX14**	10,14	10,06	0,08
**GJA1**	12,83	12,75	0,08
**ASIC1**	8,96	8,88	0,08
**RC3H1**	12,34	12,26	0,08
**LOXL2**	8,27	8,19	0,08
**PIBF1**	11,09	11,00	0,08
**B2M**	15,88	15,80	0,08
**C16orf95**	7,18	7,10	0,08
**MRPS22**	11,67	11,59	0,08
**FAM83D**	11,44	11,36	0,08
**CTNNA1**	14,27	14,18	0,08
**PTPRU**	6,26	6,18	0,08
**FAM126B**	11,82	11,74	0,08
**GPAM**	11,44	11,36	0,08
**RBL2**	13,08	13,00	0,08
**MT-CO2**	17,70	17,62	0,08
**MCAM**	13,09	13,01	0,08
**TRAM2**	13,61	13,53	0,08
**TIMM8A**	11,35	11,27	0,08
**RNF41**	11,92	11,84	0,08
**CD38**	13,30	13,21	0,08
**ADPGK**	11,86	11,78	0,08
**EIF4A2**	14,96	14,88	0,08
**RTTN**	11,78	11,70	0,08
**ATP10D**	10,50	10,42	0,08
**RPP30**	11,93	11,85	0,08
**RWDD4**	12,08	12,00	0,08
**WDR33**	13,39	13,31	0,08
**ZNF44**	9,83	9,75	0,08
**FZD5**	9,38	9,29	0,08
**SCD5**	10,83	10,74	0,08
**TTC13**	11,20	11,12	0,08
**NFYC**	10,60	10,51	0,08
**CCDC150**	10,46	10,37	0,08
**STRN3**	11,75	11,67	0,08
**CRLF3**	11,63	11,55	0,08
**SRFBP1**	11,37	11,29	0,08
**PREX1**	9,66	9,58	0,08
**LY75**	12,23	12,14	0,08
**SSR1**	13,48	13,39	0,08
**ZNF655**	12,25	12,17	0,08
**ZKSCAN1**	13,42	13,34	0,08
**SH3GL3**	5,61	5,53	0,08
**CYRIA**	8,60	8,52	0,08
**PCNP**	14,03	13,95	0,08
**TMEM218**	9,93	9,84	0,08
**NOX3**	4,29	4,20	0,08
**RPL9**	15,75	15,66	0,08
**ZNF700**	11,69	11,61	0,08
**SGPP1**	10,37	10,29	0,08
**PMPCB**	12,86	12,78	0,08
**DYNC2H1**	9,95	9,87	0,08
**SLC37A1**	10,60	10,52	0,08

**IFIH1**	10,49	10,40	0,08
**MEX3C**	13,31	13,22	0,08
**CDK8**	11,34	11,25	0,08
**COPS5**	13,02	12,94	0,08
**KIAA1143**	13,02	12,94	0,08
**CCNL2**	12,94	12,86	0,08
**PACC1**	10,79	10,71	0,08
**MAPK14**	13,17	13,09	0,08
**NAF1**	11,41	11,33	0,08
**ASCC3**	13,43	13,35	0,08
**TMBIM4**	10,79	10,70	0,08
**MAP7D3**	12,22	12,13	0,08
**PPM1B**	12,00	11,92	0,08
**SLC35F6**	11,00	10,92	0,08
**USP10**	13,90	13,81	0,08
**SLC25A14**	9,40	9,32	0,08
**ARL17B**	10,70	10,61	0,08
**SEPTIN6**	14,79	14,71	0,08
**NKAPD1**	12,53	12,45	0,08
**SLAMF1**	11,88	11,79	0,08
**AAK1**	12,47	12,39	0,08
**IKZF5**	11,65	11,57	0,08
**CAMKK2**	12,97	12,89	0,08
**AC114490.2**	8,44	8,35	0,08
**ST6GALNAC3**	7,77	7,69	0,08
**TMEM237**	12,05	11,97	0,08
**BCKDK**	11,37	11,29	0,08
**AP1G2**	11,87	11,79	0,08
**FAM167A**	6,53	6,45	0,08
**JADE3**	12,30	12,21	0,08
**MED18**	10,84	10,76	0,08
**TTLL5**	11,70	11,61	0,08
**PCDH10**	6,91	6,82	0,08
**ZNF586**	10,84	10,76	0,08
**ACAD10**	10,67	10,59	0,08
**CCT8**	15,28	15,20	0,08
**COMTD1**	8,75	8,66	0,08
**CNKSR3**	9,94	9,85	0,08
**CISH**	8,92	8,84	0,08
**SOS1**	11,70	11,62	0,08
**FAM120B**	11,47	11,39	0,08
**SLC35B4**	12,47	12,39	0,08
**PPT1**	13,23	13,15	0,08
**SIDT2**	9,45	9,37	0,08
**SYAP1**	11,15	11,06	0,08
**FAT1**	16,06	15,98	0,08
**HDAC9**	12,71	12,62	0,08
**RFC5**	12,50	12,42	0,09
**STX2**	11,08	11,00	0,09
**MASTL**	12,12	12,03	0,09
**SEH1L**	13,63	13,54	0,09
**ZNF207**	14,79	14,71	0,09
**ADGRA3**	13,13	13,05	0,09
**ABCC1**	13,18	13,09	0,09
**NCKAP5L**	10,44	10,35	0,09
**LRRC63**	1,63	1,54	0,09

**MTMR3**	11,61	11,52	0,09
**DROSHA**	12,88	12,80	0,09
**LAMTOR3**	11,33	11,25	0,09
**MICU1**	11,59	11,51	0,09
**NR4A1**	10,15	10,06	0,09
**C11orf24**	10,29	10,20	0,09
**HARS2**	11,67	11,59	0,09
**ZNF623**	12,43	12,34	0,09
**ZNF485**	10,10	10,01	0,09
**GPBP1L1**	12,83	12,74	0,09
**LRBA**	12,56	12,48	0,09
**HYAL1**	5,92	5,83	0,09
**CACUL1**	12,52	12,43	0,09
**TIAL1**	13,67	13,58	0,09
**CLN5**	10,70	10,61	0,09
**DTWD2**	9,39	9,31	0,09
**FBXW7**	13,41	13,33	0,09
**TOPBP1**	14,08	13,99	0,09
**FASTKD2**	12,21	12,12	0,09
**KIAA0100**	14,54	14,45	0,09
**SPIN2B**	8,86	8,77	0,09
**ARL8B**	12,36	12,27	0,09
**STK24**	13,15	13,06	0,09
**RCOR1**	13,84	13,75	0,09
**NEK6**	11,51	11,42	0,09
**KCTD17**	7,73	7,64	0,09
**UQCC1**	11,69	11,60	0,09
**MED27**	11,26	11,18	0,09
**ZWILCH**	12,60	12,52	0,09
**STX3**	11,60	11,52	0,09
**ATP6V0E1**	11,59	11,50	0,09
**MORF4L2**	14,11	14,02	0,09
**DOCK7**	12,01	11,92	0,09
**PPM1L**	11,92	11,83	0,09
**ZSCAN9**	8,83	8,74	0,09
**UBE2Q2**	11,93	11,84	0,09
**VILL**	8,71	8,62	0,09
**ADK**	12,38	12,29	0,09
**FAF1**	12,35	12,26	0,09
**C8orf88**	8,57	8,49	0,09
**CA2**	8,66	8,57	0,09
**ZNF12**	12,04	11,95	0,09
**BTRC**	10,92	10,83	0,09
**ASIC2**	9,17	9,08	0,09
**WDR54**	10,18	10,10	0,09
**CLN3**	10,87	10,78	0,09
**CNTRL**	12,11	12,02	0,09
**CIR1**	11,12	11,04	0,09
**SLC44A2**	13,07	12,99	0,09
**PHF21A**	11,04	10,95	0,09
**UNC50**	10,05	9,96	0,09
**PPP3R1**	13,47	13,38	0,09
**HPF1**	11,38	11,29	0,09
**SSBP2**	12,61	12,52	0,09
**PAIP2**	14,04	13,95	0,09
**MCMBP**	14,00	13,91	0,09

**S100A4**	9,59	9,50	0,09
**ELOVL2**	12,24	12,16	0,09
**BLVRB**	9,55	9,47	0,09
**LUC7L2**	13,44	13,35	0,09
**RTRAF**	13,93	13,84	0,09
**ABHD17B**	13,09	13,00	0,09
**HMGXB4**	12,31	12,23	0,09
**ABHD16A**	10,87	10,79	0,09
**RBM26**	13,75	13,66	0,09
**TOMM34**	12,49	12,40	0,09
**BET1L**	9,82	9,73	0,09
**MBOAT1**	11,46	11,37	0,09
**RNF6**	12,50	12,42	0,09
**CIAO2A**	12,19	12,10	0,09
**GSPT1**	14,76	14,67	0,09
**CREBBP**	13,86	13,77	0,09
**ZNF805**	9,46	9,38	0,09
**BAG4**	11,99	11,90	0,09
**ZCCHC14**	12,32	12,23	0,09
**TPP1**	12,00	11,91	0,09
**TMEM35B**	9,80	9,71	0,09
**TCAF1**	12,50	12,41	0,09
**CXorf38**	10,99	10,90	0,09
**TPST2**	11,26	11,17	0,09
**USP8**	12,70	12,61	0,09
**BFAR**	12,36	12,28	0,09
**USP41**	6,43	6,34	0,09
**MYL12B**	13,86	13,77	0,09
**AC118553.2**	7,90	7,81	0,09
**SNX30**	11,01	10,92	0,09
**SFR1**	10,05	9,96	0,09
**CDH2**	11,81	11,72	0,09
**CEP95**	12,03	11,94	0,09
**EFNA1**	7,34	7,25	0,09
**BNIP1**	10,16	10,07	0,09
**ARHGEF12**	13,04	12,95	0,09
**ZNF592**	12,64	12,55	0,09
**ZMYND8**	12,84	12,75	0,09
**ZBTB24**	12,27	12,18	0,09
**LANCL1**	13,44	13,35	0,09
**CAPN2**	12,99	12,90	0,09
**EMC1**	12,64	12,55	0,09
**CSTB**	11,34	11,25	0,09
**ZDHHC17**	12,34	12,25	0,09
**MOB1B**	12,23	12,14	0,09
**SEC31B**	10,53	10,44	0,09
**COL6A1**	7,07	6,98	0,09
**SP4**	11,41	11,32	0,09
**MLH3**	11,51	11,42	0,09
**PBX1**	15,73	15,64	0,09
**SERTAD2**	11,47	11,38	0,09
**RNF43**	6,47	6,38	0,09
**TTC3**	14,27	14,18	0,09
**SPARC**	10,14	10,05	0,09
**STK38**	13,90	13,81	0,09
**MLLT11**	12,82	12,73	0,09

**KIAA1109**	11,76	11,66	0,09
**ANKRD53**	2,74	2,65	0,09
**XIAP**	12,45	12,36	0,09
**RECK**	10,79	10,69	0,09
**AKR7A3**	6,85	6,76	0,09
**KTN1**	14,96	14,87	0,09
**NUMB**	11,83	11,74	0,09
**MRPS10**	12,47	12,37	0,09
**IFTAP**	8,71	8,62	0,09
**SPRED2**	10,93	10,84	0,09
**UBE2K**	13,60	13,51	0,09
**SMARCA5**	14,76	14,67	0,09
**IFNGR1**	11,04	10,95	0,09
**CAMSAP3**	4,37	4,27	0,09
**XKR7**	4,37	4,27	0,09
**HDAC4**	11,45	11,36	0,09
**TMEM161A**	11,20	11,11	0,09
**CYP4V2**	10,47	10,38	0,09
**MTO1**	12,20	12,11	0,09
**TENT4B**	11,40	11,31	0,09
**GON7**	10,28	10,19	0,09
**ACSM3**	4,54	4,45	0,09
**RBBP6**	13,92	13,83	0,09
**INPP4A**	11,50	11,40	0,09
**HACE1**	10,65	10,56	0,09
**PMEPA1**	11,64	11,55	0,09
**CCDC47**	13,37	13,27	0,09
**BMPR2**	11,23	11,13	0,09
**SLC16A9**	9,45	9,36	0,09
**TBC1D8B**	9,48	9,39	0,09
**COPB2**	13,72	13,63	0,09
**PXDN**	15,25	15,16	0,09
**ZNF275**	11,27	11,18	0,09
**GARRE1**	9,49	9,40	0,09
**CELF4**	6,10	6,01	0,09
**CELF2**	14,82	14,73	0,09
**CHRNB4**	6,15	6,06	0,09
**ZNF142**	12,49	12,40	0,09
**NFS1**	10,75	10,66	0,09
**STARD4**	11,64	11,55	0,09
**IVNS1ABP**	14,07	13,98	0,09
**ZNF561**	10,89	10,80	0,09
**GEN1**	13,45	13,35	0,09
**CPSF3**	13,02	12,93	0,09
**MYOM2**	8,87	8,78	0,09
**GJA5**	10,07	9,98	0,09
**RSF1**	11,45	11,36	0,09
**TMEM175**	9,46	9,37	0,09
**EVC2**	9,61	9,52	0,09
**TUFT1**	7,49	7,40	0,09
**PCGF5**	12,30	12,21	0,09
**UBE3C**	13,23	13,14	0,09
**FAM122B**	12,57	12,47	0,09
**INCENP**	13,03	12,94	0,09
**TNFAIP8**	11,24	11,15	0,09
**TAGLN**	8,52	8,43	0,09

**LONP2**	12,93	12,84	0,09
**ELMOD2**	11,53	11,44	0,09
**ZNF597**	9,42	9,33	0,09
**AFDN**	11,56	11,47	0,09
**PACS1**	12,12	12,03	0,09
**SEPTIN11**	14,41	14,32	0,09
**MIA2**	11,12	11,03	0,09
**CDYL**	12,38	12,29	0,09
**EFHC2**	7,37	7,28	0,09
**USP35**	8,15	8,06	0,09
**RNPEP**	12,84	12,75	0,09
**FADS2**	14,20	14,11	0,09
**AZIN1**	14,32	14,23	0,09
**POLR1F**	13,18	13,09	0,09
**NBPF26**	12,21	12,11	0,09
**ABCD2**	7,61	7,51	0,09
**TASOR**	12,28	12,18	0,09
**JAM2**	9,56	9,47	0,09
**AVL9**	11,71	11,62	0,09
**RECQL**	12,97	12,88	0,09
**MYCBP**	11,91	11,82	0,09
**E2F6**	11,44	11,34	0,09
**SEC11A**	11,70	11,61	0,09
**ZNF75D**	10,50	10,41	0,09
**CNOT10**	9,31	9,21	0,09
**MARCHF6**	13,80	13,71	0,09
**GDI2**	15,43	15,34	0,09
**RBBP4**	15,44	15,34	0,09
**CFAP97**	13,31	13,22	0,09
**BEND3**	11,62	11,53	0,09
**ZBTB34**	10,39	10,30	0,09
**RBM22**	12,91	12,81	0,09
**CCT4**	15,06	14,97	0,09
**SLC40A1**	6,63	6,54	0,09
**PBK**	13,12	13,03	0,09
**MCM10**	13,38	13,29	0,09
**BOD1L1**	13,42	13,33	0,09
**UBE4B**	12,50	12,40	0,09
**SPTLC2**	12,28	12,18	0,09
**UGGT1**	13,97	13,88	0,09
**KDM5A**	13,36	13,26	0,09
**LIMS1**	13,04	12,95	0,09
**CYP46A1**	5,83	5,73	0,09
**PNRC2**	14,18	14,08	0,09
**ZNF624**	9,25	9,15	0,09
**MGAT4A**	10,08	9,98	0,09
**HACD1**	11,20	11,10	0,09
**IQCB1**	12,30	12,20	0,09
**GCLC**	12,15	12,05	0,09
**CTSC**	13,90	13,81	0,10
**NAV1**	13,56	13,47	0,10
**COQ10A**	10,38	10,28	0,10
**KBTBD11**	13,39	13,29	0,10
**KATNA1**	11,32	11,23	0,10
**PCGF6**	11,23	11,14	0,10
**RHOA**	15,62	15,53	0,10

**EPOR**	7,53	7,43	0,10
**NAA25**	12,90	12,80	0,10
**ZNHIT3**	11,01	10,91	0,10
**ZNF22**	13,98	13,89	0,10
**PLGRKT**	9,41	9,31	0,10
**EIF3I**	14,20	14,11	0,10
**CORO2A**	9,28	9,18	0,10
**ATP2B4**	13,52	13,42	0,10
**HMGN3**	11,48	11,38	0,10
**RRN3**	13,23	13,14	0,10
**TMEM161B**	10,95	10,85	0,10
**CWC22**	11,99	11,90	0,10
**ABL2**	12,41	12,32	0,10
**PM20D2**	12,66	12,56	0,10
**TRIM14**	12,45	12,35	0,10
**IMPA1**	12,37	12,27	0,10
**DRAM1**	9,15	9,05	0,10
**NELL1**	11,84	11,74	0,10
**GCH1**	9,72	9,62	0,10
**RBM27**	12,80	12,70	0,10
**UBR7**	12,88	12,78	0,10
**FAM199X**	12,08	11,99	0,10
**ZNF711**	12,10	12,00	0,10
**ARRDC4**	10,39	10,30	0,10
**CREB5**	10,77	10,67	0,10
**IBTK**	11,88	11,78	0,10
**ETV5**	13,16	13,07	0,10
**FAM20B**	12,87	12,78	0,10
**VWA8**	11,30	11,20	0,10
**RAD51AP1**	12,05	11,96	0,10
**EPRS1**	14,82	14,72	0,10
**VAMP7**	11,49	11,40	0,10
**UBE2V2**	13,77	13,67	0,10
**DAAM1**	10,88	10,78	0,10
**NAA15**	14,17	14,07	0,10
**CTSS**	10,90	10,81	0,10
**BMI1**	15,39	15,30	0,10
**CAPZA2**	12,94	12,84	0,10
**ITGA9**	10,65	10,55	0,10
**ESD**	13,13	13,03	0,10
**EIF2S3**	15,29	15,20	0,10
**PBX3**	10,38	10,28	0,10
**SPIDR**	12,73	12,63	0,10
**C16orf87**	11,54	11,44	0,10
**DENND4A**	11,98	11,89	0,10
**RBM41**	8,51	8,41	0,10
**YWHAB**	15,31	15,21	0,10
**RB1CC1**	12,96	12,86	0,10
**RNF144A**	12,46	12,36	0,10
**GALNT9**	2,61	2,51	0,10
**GPAT4**	13,29	13,19	0,10
**MRI1**	10,74	10,65	0,10
**PRRT3**	8,73	8,63	0,10
**EP400**	13,66	13,56	0,10
**MICAL3**	11,63	11,53	0,10
**UXS1**	12,56	12,46	0,10

**WASHC3**	9,68	9,58	0,10
**ERRFI1**	8,38	8,29	0,10
**CARD19**	9,89	9,79	0,10
**YWHAH**	13,57	13,47	0,10
**ANKHD1-EIF4EBP3**	12,46	12,36	0,10
**EFCAB12**	4,44	4,34	0,10
**XRCC4**	10,56	10,46	0,10
**SMC5**	13,61	13,51	0,10
**CARD8**	12,57	12,47	0,10
**RAP1GDS1**	12,19	12,09	0,10
**NSMAF**	13,28	13,18	0,10
**SH3GLB1**	12,96	12,86	0,10
**FANCD2OS**	3,57	3,47	0,10
**CHD5**	6,17	6,07	0,10
**ZNF587**	11,85	11,76	0,10
**TOMM20**	14,84	14,74	0,10
**IL4I1**	6,89	6,79	0,10
**PLGLB2**	9,43	9,33	0,10
**ECI2**	11,83	11,73	0,10
**SH3BGRL**	12,80	12,70	0,10
**ITGA4**	15,49	15,39	0,10
**FTO**	11,98	11,89	0,10
**JPH3**	5,95	5,86	0,10
**ZNF599**	8,67	8,57	0,10
**SDF2**	10,50	10,40	0,10
**TIMMDC1**	11,73	11,63	0,10
**IMMT**	13,67	13,57	0,10
**LILRB1**	5,86	5,76	0,10
**CHML**	13,81	13,71	0,10
**EP300**	13,46	13,36	0,10
**MPP1**	12,82	12,72	0,10
**STX16**	13,06	12,96	0,10
**BLNK**	13,72	13,62	0,10
**MED20**	11,65	11,55	0,10
**SCCPDH**	14,79	14,69	0,10
**RARG**	9,91	9,81	0,10
**PSME4**	13,78	13,68	0,10
**RRM1**	14,35	14,25	0,10
**SCAF8**	13,01	12,91	0,10
**ORC2**	12,39	12,29	0,10
**MFSD14A**	12,48	12,38	0,10
**EFCAB14**	12,79	12,69	0,10
**DSCC1**	11,99	11,89	0,10
**TPRG1L**	9,47	9,37	0,10
**THOC7**	12,87	12,77	0,10
**ALG14**	9,02	8,92	0,10
**CRLF2**	4,26	4,16	0,10
**RAB11FIP2**	11,38	11,28	0,10
**C12orf60**	6,37	6,27	0,10
**SENP1**	12,37	12,27	0,10
**PSMD14**	13,49	13,39	0,10
**GLS**	14,32	14,22	0,10
**AKIRIN1**	13,10	13,00	0,10
**NYNRIN**	12,46	12,36	0,10
**NCAPG**	13,74	13,64	0,10
**SNX4**	12,16	12,06	0,10

**CHMP1B**	11,85	11,75	0,10
**PMF1-BGLAP**	8,12	8,02	0,10
**RANBP3L**	5,55	5,45	0,10
**CISD2**	11,97	11,87	0,10
**HDC**	3,57	3,47	0,10
**EFNA5**	3,57	3,47	0,10
**GGACT**	3,57	3,47	0,10
**DCTN6**	11,62	11,52	0,10
**EBF1**	15,32	15,22	0,10
**TERB1**	3,14	3,03	0,10
**NACC1**	12,79	12,69	0,10
**TMOD3**	12,86	12,75	0,10
**NUDT21**	14,79	14,69	0,10
**CLK1**	12,58	12,48	0,10
**FAM3C**	14,63	14,53	0,10
**MPHOSPH10**	13,17	13,07	0,10
**HSPG2**	12,14	12,04	0,10
**KCTD3**	12,83	12,73	0,10
**GOLGA8N**	8,36	8,26	0,10
**CDC7**	13,10	13,00	0,10
**ZYG11B**	11,85	11,75	0,10
**MAPKAP1**	12,81	12,71	0,10
**SPTY2D1**	12,08	11,98	0,10
**FKBP1B**	4,48	4,38	0,10
**TTC19**	12,20	12,09	0,10
**FANCI**	14,06	13,96	0,10
**SLC22A23**	9,90	9,80	0,10
**NCOA2**	13,15	13,05	0,10
**AQP11**	7,37	7,27	0,10
**NT5E**	9,87	9,77	0,10
**RNF135**	10,87	10,76	0,10
**GTF3C4**	12,99	12,89	0,10
**REPS1**	12,35	12,25	0,10
**UBE2N**	13,82	13,72	0,10
**C1D**	11,58	11,48	0,10
**OXCT1**	13,60	13,49	0,10
**PITRM1**	12,42	12,32	0,10
**KLF11**	11,32	11,22	0,10
**ZFHX3**	10,85	10,75	0,10
**SLC25A36**	13,05	12,95	0,10
**MTIF2**	11,74	11,63	0,10
**ERCC6**	9,98	9,88	0,10
**CDCA7**	14,57	14,46	0,10
**OGA**	13,72	13,62	0,10
**RP1L1**	5,74	5,63	0,10
**COMMD8**	10,62	10,51	0,10
**CPSF6**	14,58	14,47	0,10
**CD164**	14,00	13,90	0,10
**TFG**	12,48	12,37	0,10
**GCHFR**	8,83	8,73	0,10
**TUBGCP5**	11,98	11,88	0,10
**KLHL42**	12,72	12,62	0,10
**ERGIC3**	12,46	12,35	0,10
**PRPF40A**	14,56	14,45	0,10
**SLC12A2**	12,32	12,22	0,10
**TNRC6A**	13,33	13,22	0,10

**DR1**	13,45	13,34	0,10
**SKP1**	14,27	14,16	0,10
**CD24**	16,48	16,37	0,10
**CDH23**	5,85	5,74	0,10
**PAPSS2**	6,00	5,90	0,10
**ZNF549**	11,15	11,05	0,10
**ODR4**	11,23	11,13	0,10
**PHACTR4**	12,33	12,23	0,10
**L2HGDH**	11,18	11,07	0,10
**RMI2**	11,04	10,93	0,10
**TVP23B**	10,46	10,35	0,10
**ZFAND3**	12,13	12,03	0,10
**CAPZA1**	14,34	14,24	0,10
**AC022415.2**	10,76	10,65	0,10
**SLA2**	5,44	5,34	0,10
**SARAF**	14,29	14,18	0,10
**DMAC2**	11,53	11,43	0,10
**MTX2**	10,73	10,63	0,10
**CPA6**	10,92	10,81	0,10
**CAPN13**	6,23	6,13	0,10
**C7orf61**	6,69	6,58	0,10
**VAMP3**	11,24	11,13	0,10
**SINHCAF**	14,31	14,21	0,10
**CEP295**	11,74	11,63	0,10
**SPECC1L**	12,90	12,80	0,10
**CIAO1**	13,30	13,20	0,10
**LIMS3**	7,65	7,54	0,10
**ELP1**	13,05	12,95	0,10
**TENT2**	11,60	11,50	0,10
**PPP2R5A**	11,72	11,61	0,10
**CKS1B**	13,28	13,17	0,10
**ELMO2**	11,73	11,63	0,10
**ACKR2**	4,33	4,22	0,10
**PRUNE1**	11,33	11,23	0,10
**ZNF114**	11,46	11,36	0,10
**VPS36**	13,13	13,03	0,10
**IFNAR1**	11,75	11,65	0,10
**ECM1**	12,37	12,26	0,10
**RPL22L1**	12,29	12,19	0,11
**ZNF8**	11,56	11,45	0,11
**PIGA**	10,24	10,14	0,11
**SNRNP27**	11,49	11,38	0,11
**HPRT1**	12,70	12,59	0,11
**BTG3**	11,32	11,22	0,11
**OXR1**	11,64	11,53	0,11
**KLHL12**	12,48	12,38	0,11
**UBTD2**	12,44	12,33	0,11
**ATRX**	13,89	13,79	0,11
**KDM5B**	13,25	13,15	0,11
**PIK3R2**	10,98	10,87	0,11
**GCLM**	11,41	11,30	0,11
**TLR1**	7,90	7,79	0,11
**LAMC1**	13,91	13,81	0,11
**NRM**	10,20	10,09	0,11
**TIAM2**	8,52	8,41	0,11
**MOB3B**	10,17	10,06	0,11

**RER1**	12,74	12,64	0,11
**DNTT**	17,31	17,20	0,11
**POGLUT1**	11,23	11,13	0,11
**CD2AP**	12,52	12,41	0,11
**ANKRD17**	14,03	13,92	0,11
**TAF9B**	12,13	12,02	0,11
**UBXN7**	12,68	12,58	0,11
**ELOVL1**	11,89	11,79	0,11
**TMEM123**	15,06	14,96	0,11
**RUFY3**	11,31	11,20	0,11
**BMP2K**	13,35	13,24	0,11
**LONRF1**	13,08	12,98	0,11
**DDX3X**	15,48	15,37	0,11
**MAP1LC3B**	12,90	12,79	0,11
**SCML1**	7,39	7,28	0,11
**CEP152**	12,56	12,45	0,11
**RBPJ**	13,52	13,41	0,11
**TPST1**	8,76	8,65	0,11
**KIF23**	12,50	12,40	0,11
**RSBN1**	12,37	12,26	0,11
**C2CD5**	12,52	12,42	0,11
**MAP3K13**	7,94	7,83	0,11
**SMIM3**	9,27	9,16	0,11
**YY1AP1**	12,61	12,50	0,11
**KIFBP**	10,87	10,76	0,11
**NAGA**	11,24	11,13	0,11
**UBE2T**	12,12	12,01	0,11
**SNTB1**	10,71	10,61	0,11
**NCOA4**	13,87	13,77	0,11
**ABI1**	12,97	12,86	0,11
**IPO7**	14,78	14,67	0,11
**TNKS2**	13,18	13,08	0,11
**DHX40**	12,35	12,24	0,11
**AC116366.1**	11,46	11,35	0,11
**MAD2L1**	13,68	13,57	0,11
**TAF1**	12,25	12,15	0,11
**CLGN**	10,21	10,10	0,11
**MDM4**	13,99	13,88	0,11
**RRP8**	11,71	11,60	0,11
**B4GALT7**	10,02	9,91	0,11
**SFI1**	10,76	10,66	0,11
**MARCHF7**	13,36	13,25	0,11
**CDKN2AIP**	12,16	12,06	0,11
**NET1**	12,59	12,48	0,11
**EXT2**	10,49	10,38	0,11
**ATP8A1**	12,65	12,54	0,11
**DHX29**	12,19	12,09	0,11
**COG2**	12,18	12,07	0,11
**FANCM**	11,02	10,91	0,11
**ZNF354C**	7,44	7,33	0,11
**FBXO30**	11,65	11,54	0,11
**WASF1**	13,23	13,13	0,11
**SDK1**	8,00	7,89	0,11
**TRIP13**	12,37	12,26	0,11
**CCP110**	12,93	12,82	0,11
**ANLN**	12,70	12,59	0,11

**SELENON**	12,88	12,77	0,11
**C1QTNF4**	7,15	7,04	0,11
**ZNF721**	11,38	11,27	0,11
**COQ9**	11,36	11,25	0,11
**FBXO11**	11,93	11,82	0,11
**APOBEC3C**	12,34	12,23	0,11
**HSPA13**	12,13	12,02	0,11
**NBPF3**	9,34	9,23	0,11
**TLR10**	8,07	7,96	0,11
**KIAA1191**	11,42	11,31	0,11
**PEG10**	12,32	12,21	0,11
**PPP1R15B**	13,17	13,06	0,11
**HSP90B1**	16,35	16,24	0,11
**GTF3C2**	13,17	13,06	0,11
**MED23**	12,08	11,97	0,11
**TRPT1**	10,02	9,91	0,11
**MICB**	11,66	11,56	0,11
**TAF8**	10,79	10,68	0,11
**DIS3L2**	11,41	11,30	0,11
**HACD3**	13,66	13,56	0,11
**GSKIP**	10,47	10,36	0,11
**AC012254.2**	7,84	7,73	0,11
**HMMR**	13,01	12,90	0,11
**TTI2**	11,59	11,48	0,11
**ZNF618**	12,29	12,18	0,11
**CLCN3**	11,97	11,86	0,11
**HDAC2**	14,74	14,63	0,11
**ZNF69**	10,89	10,78	0,11
**ATXN7**	12,08	11,97	0,11
**ZNF326**	13,25	13,14	0,11
**ZCCHC9**	10,98	10,87	0,11
**GK5**	10,86	10,75	0,11
**DTWD1**	10,99	10,88	0,11
**FSD2**	9,42	9,31	0,11
**THUMPD2**	11,24	11,13	0,11
**TAF7**	13,33	13,22	0,11
**HSPA9**	15,80	15,69	0,11
**PROSER1**	13,50	13,39	0,11
**AC002985.1**	2,75	2,64	0,11
**LARP4**	13,57	13,46	0,11
**MEST**	10,59	10,48	0,11
**COG8**	12,05	11,94	0,11
**ELF1**	14,63	14,52	0,11
**KBTBD7**	10,55	10,44	0,11
**NUP210**	14,82	14,71	0,11
**ST6GAL1**	13,06	12,95	0,11
**ABCD3**	12,39	12,28	0,11
**CASP8AP2**	11,75	11,64	0,11
**DMPK**	8,30	8,19	0,11
**HEATR1**	14,34	14,23	0,11
**MRPL3**	13,64	13,53	0,11
**RNF14**	11,60	11,49	0,11
**SLC16A1**	14,55	14,44	0,11
**KLF10**	11,23	11,12	0,11
**RAB29**	9,32	9,21	0,11
**DTX3L**	13,18	13,07	0,11

**VOPP1**	12,13	12,02	0,11
**GARS1**	14,47	14,36	0,11
**SURF1**	9,23	9,12	0,11
**NSMCE4A**	12,19	12,08	0,11
**OR2H2**	4,47	4,36	0,11
**TESMIN**	9,05	8,94	0,11
**MIOS**	11,92	11,81	0,11
**BRMS1L**	9,92	9,81	0,11
**PXDC1**	7,41	7,30	0,11
**MIPEP**	10,74	10,63	0,11
**AP1G1**	13,60	13,49	0,11
**JAGN1**	11,26	11,15	0,11
**PIK3C2B**	8,05	7,93	0,11
**METTL9**	13,33	13,22	0,11
**COG5**	11,96	11,85	0,11
**RANBP9**	12,90	12,79	0,11
**MRPS18B**	12,20	12,09	0,11
**SRD5A3**	10,49	10,37	0,11
**TSN**	13,93	13,82	0,11
**PSAT1**	13,95	13,84	0,11
**BHLHE40**	8,11	8,00	0,11
**ZKSCAN8**	12,45	12,34	0,11
**BTBD19**	4,58	4,47	0,11
**MDGA2**	10,25	10,14	0,11
**CEP85L**	9,57	9,46	0,11
**BRPF3**	12,35	12,23	0,11
**THOC3**	12,85	12,74	0,11
**C6orf62**	13,61	13,50	0,11
**SRSF11**	13,86	13,75	0,11
**SLC35E4**	7,32	7,21	0,11
**NLRC5**	12,01	11,90	0,11
**NCAM1**	8,82	8,70	0,11
**H6PD**	11,60	11,49	0,11
**TMF1**	12,17	12,06	0,11
**LPIN1**	12,49	12,38	0,11
**MAP3K20**	13,24	13,13	0,11
**TAF1A**	10,79	10,68	0,11
**IGKV2-30**	3,70	3,59	0,11
**SPON1**	3,70	3,59	0,11
**GRM6**	3,70	3,59	0,11
**ETF1**	14,24	14,13	0,11
**ZNF256**	10,45	10,34	0,11
**ZC3H11A**	14,35	14,24	0,11
**MORC4**	10,69	10,57	0,11
**USP37**	12,31	12,20	0,11
**COCH**	13,87	13,76	0,11
**ENTPD4**	13,21	13,10	0,11
**CLCN5**	10,23	10,12	0,11
**MSL2**	12,81	12,70	0,11
**FLOT2**	12,13	12,02	0,11
**FAM160B1**	11,46	11,35	0,11
**AGTPBP1**	11,97	11,85	0,11
**VPS37A**	11,68	11,57	0,11
**GRSF1**	13,66	13,55	0,11
**TMEM62**	10,13	10,02	0,11
**SRSF10**	14,94	14,83	0,11

**AACS**	10,35	10,24	0,11
**FAM241A**	12,33	12,22	0,11
**RINL**	8,84	8,73	0,11
**WASL**	10,02	9,91	0,11
**IARS2**	14,03	13,91	0,11
**SLC25A45**	8,18	8,07	0,11
**EEF1A2**	9,35	9,24	0,11
**YBEY**	8,35	8,23	0,11
**VPS35L**	11,01	10,90	0,11
**SDC4**	5,55	5,43	0,11
**RALGAPB**	13,02	12,91	0,11
**MSRB1**	9,37	9,25	0,11
**TYW5**	10,66	10,55	0,11
**SSH1**	11,28	11,16	0,11
**NDUFAF5**	10,47	10,36	0,11
**FAM234A**	11,78	11,67	0,11
**NIPAL2**	10,57	10,45	0,11
**MS4A4A**	8,11	8,00	0,11
**TRUB1**	11,98	11,87	0,11
**TXNDC12**	12,21	12,09	0,11
**KPNA4**	13,58	13,46	0,11
**SULT1A1**	9,93	9,82	0,11
**CPT1A**	12,96	12,85	0,11
**ARL17A**	11,06	10,94	0,11
**SRBD1**	10,54	10,43	0,11
**CCNT1**	12,98	12,86	0,11
**RGMB**	11,44	11,33	0,11
**PSMA8**	7,29	7,18	0,11
**XPR1**	12,14	12,02	0,11
**ASB13**	10,33	10,21	0,11
**COL1A1**	8,92	8,81	0,11
**ERBIN**	13,45	13,34	0,11
**BCAS4**	8,91	8,80	0,11
**CHUK**	11,70	11,59	0,11
**PSD4**	9,56	9,45	0,11
**LAPTM4B**	15,03	14,92	0,11
**VCPIP1**	12,39	12,28	0,12
**COLEC12**	8,42	8,30	0,12
**SMIM19**	10,39	10,28	0,12
**NFAT5**	11,91	11,80	0,12
**IRF2BP2**	14,45	14,33	0,12
**TRMT6**	11,68	11,56	0,12
**ATP8B3**	8,57	8,46	0,12
**SMC3**	14,17	14,05	0,12
**EZH2**	13,60	13,48	0,12
**PIGK**	10,86	10,74	0,12
**VTA1**	12,94	12,82	0,12
**TTF1**	13,08	12,97	0,12
**PRDM2**	12,70	12,59	0,12
**ADAM22**	11,10	10,99	0,12
**NUP153**	14,39	14,27	0,12
**ESPNL**	7,41	7,29	0,12
**TRIM34**	10,50	10,39	0,12
**PANK3**	14,20	14,09	0,12
**PRKCI**	12,43	12,31	0,12
**CHD8**	13,50	13,38	0,12

**RSPRY1**	12,25	12,13	0,12
**SPTSSA**	12,05	11,93	0,12
**TRIM38**	12,67	12,55	0,12
**SLC45A4**	9,22	9,10	0,12
**KCNMB1**	9,16	9,04	0,12
**ESRP1**	2,38	2,26	0,12
**PRKAA1**	8,51	8,39	0,12
**PI4K2A**	9,97	9,85	0,12
**AC015802.6**	12,23	12,11	0,12
**FBXW11**	12,50	12,38	0,12
**MTDH**	15,33	15,22	0,12
**BCAS2**	12,11	11,99	0,12
**FMNL2**	14,27	14,15	0,12
**SLC25A53**	9,31	9,19	0,12
**FGF17**	5,06	4,94	0,12
**DGAT2L6**	4,64	4,53	0,12
**CD300LF**	4,64	4,53	0,12
**FH**	13,98	13,86	0,12
**FAM162A**	11,53	11,41	0,12
**TREML2**	6,72	6,61	0,12
**AGFG1**	12,82	12,70	0,12
**STAG1**	13,52	13,40	0,12
**VPS11**	11,40	11,28	0,12
**BET1**	10,99	10,87	0,12
**MAPK6**	12,23	12,11	0,12
**HSPBAP1**	9,89	9,77	0,12
**TOP2A**	15,99	15,87	0,12
**ZBTB18**	12,58	12,46	0,12
**FCGRT**	8,94	8,82	0,12
**CALM1**	15,39	15,27	0,12
**OVGP1**	8,07	7,95	0,12
**UFL1**	11,89	11,77	0,12
**ERAP2**	13,21	13,09	0,12
**ZNF726**	10,00	9,89	0,12
**CTSB**	12,95	12,83	0,12
**CENPF**	14,93	14,82	0,12
**ARL5B**	13,27	13,15	0,12
**HMGCR**	13,26	13,14	0,12
**TMEM120B**	11,65	11,53	0,12
**PIEZO1**	13,23	13,11	0,12
**ALG1**	9,46	9,34	0,12
**FBXW2**	9,86	9,74	0,12
**ATXN10**	13,99	13,87	0,12
**KLHL20**	10,82	10,70	0,12
**QRSL1**	14,21	14,09	0,12
**RPS3A**	17,01	16,89	0,12
**ZNF410**	11,86	11,74	0,12
**KCMF1**	12,49	12,37	0,12
**LATS1**	12,33	12,21	0,12
**ERG**	13,52	13,40	0,12
**BZW1**	14,69	14,57	0,12
**ELK3**	12,34	12,23	0,12
**MGAT4B**	11,93	11,82	0,12
**TARBP1**	13,72	13,60	0,12
**MINPP1**	8,81	8,69	0,12
**MXI1**	10,40	10,28	0,12

ARL6IP6	7,88	7,76	0,12
BLCAP	10,80	10,68	0,12
CEP55	12,55	12,44	0,12
CTNNB1	14,17	14,05	0,12
TRAPPC8	13,01	12,89	0,12
ZUP1	10,83	10,72	0,12
MBD4	11,86	11,75	0,12
SAP30L	11,98	11,86	0,12
SYT11	12,29	12,17	0,12
DHX8	12,54	12,42	0,12
PPP6C	13,03	12,91	0,12
ZNF322	11,63	11,51	0,12
AVPI1	6,39	6,27	0,12
IPO5	15,31	15,20	0,12
REC8	10,37	10,25	0,12
SNRNP48	11,98	11,87	0,12
YTHDF1	12,96	12,84	0,12
FHL1	12,71	12,59	0,12
WRN	13,16	13,04	0,12
ARPP21	9,23	9,11	0,12
P2RX7	8,90	8,78	0,12
DZIP3	10,28	10,16	0,12
NIPSNAP3B	11,51	11,39	0,12
BORA	11,23	11,11	0,12
RNF130	12,79	12,67	0,12
SH3BP2	11,07	10,95	0,12
RNF103	10,60	10,48	0,12
SLC38A9	10,49	10,37	0,12
PRRT1	6,25	6,13	0,12
SHTN1	9,76	9,64	0,12
SH3BP5	7,65	7,54	0,12
BTF3L4	12,57	12,45	0,12
DDX6	14,44	14,32	0,12
ALDOC	10,27	10,15	0,12
FDXACB1	9,57	9,45	0,12
CST3	8,45	8,34	0,12
INVS	11,06	10,94	0,12
ARNT	12,00	11,88	0,12
CD58	11,04	10,92	0,12
LDLRAD3	12,28	12,17	0,12
IFNLR1	6,75	6,63	0,12
POLR2H	11,83	11,71	0,12
SLC25A24	12,13	12,01	0,12
ZNF792	9,47	9,35	0,12
ZKSCAN2	10,85	10,73	0,12
AC004922.1	7,48	7,36	0,12
B3GNT2	11,95	11,83	0,12
DNM1L	13,37	13,25	0,12
SPTLC1	12,07	11,95	0,12
SERPINB9	10,41	10,29	0,12
ZNF430	11,43	11,31	0,12
MOSPD3	9,19	9,07	0,12
EEA1	11,91	11,79	0,12
AMD1	14,28	14,16	0,12
PPM1A	12,47	12,35	0,12
BTBD1	13,31	13,19	0,12

**WASHC2C**	12,41	12,29	0,12
**PAICS**	15,70	15,58	0,12
**MAGI3**	10,60	10,48	0,12
**ZBTB26**	10,20	10,08	0,12
**FAM234B**	11,07	10,95	0,12
**ALG2**	10,98	10,86	0,12
**HOMER1**	11,63	11,51	0,12
**ITM2B**	13,23	13,11	0,12
**GPATCH2**	10,38	10,26	0,12
**SENP5**	12,36	12,24	0,12
**COIL**	12,04	11,91	0,12
**RIBC2**	7,90	7,78	0,12
**EIF3L**	14,93	14,81	0,12
**NIPBL**	13,65	13,53	0,12
**DNAJC13**	12,76	12,64	0,12
**SLC35B1**	12,11	11,99	0,12
**MED28**	13,46	13,34	0,12
**NCK1**	11,13	11,01	0,12
**LPAR5**	10,92	10,80	0,12
**TAF6**	11,69	11,57	0,12
**PAPOLG**	11,59	11,47	0,12
**RNF138**	13,43	13,31	0,12
**STRBP**	13,81	13,69	0,12
**RASSF4**	9,26	9,14	0,12
**ZDHHC20**	12,79	12,67	0,12
**ZC3H12C**	11,19	11,06	0,12
**PLAU**	8,94	8,82	0,12
**GTF2H1**	12,35	12,23	0,12
**ZNF782**	9,54	9,42	0,12
**GCAT**	7,90	7,78	0,12
**FBXO4**	9,31	9,19	0,12
**PGM3**	10,47	10,35	0,12
**AQR**	13,40	13,27	0,12
**WDTC1**	11,74	11,62	0,12
**H2AC20**	6,78	6,66	0,12
**SPIN1**	13,92	13,80	0,12
**HECTD4**	12,67	12,55	0,12
**SPACA4**	5,25	5,13	0,12
**STEAP1**	3,82	3,69	0,12
**FAM187A**	3,82	3,69	0,12
**H3-2**	3,82	3,69	0,12
**FGF13**	8,20	8,08	0,12
**UBE2J2**	12,19	12,06	0,12
**SLC30A9**	12,04	11,92	0,12
**UBE4A**	12,99	12,87	0,12
**GSDMB**	8,48	8,36	0,12
**AP2A2**	12,28	12,16	0,12
**LRIF1**	11,48	11,36	0,12
**RABGGTB**	13,02	12,90	0,12
**NCOA7**	11,16	11,04	0,12
**TMTC2**	7,61	7,48	0,12
**VTI1A**	11,31	11,18	0,12
**RNF114**	12,70	12,58	0,12
**TRIM59**	11,79	11,67	0,12
**CYP2R1**	10,29	10,16	0,12
**ARPC3**	13,86	13,74	0,12

**PIK3AP1**	15,40	15,28	0,12
**PPP2R2D**	11,98	11,86	0,12
**RBAK**	11,79	11,67	0,12
**IRAG2**	16,19	16,07	0,12
**SMU1**	13,33	13,21	0,12
**ZNF439**	10,33	10,20	0,12
**COPS7B**	12,16	12,04	0,12
**SEC63**	13,76	13,64	0,12
**TRIM24**	12,82	12,69	0,12
**CHST10**	10,74	10,61	0,12
**NPTN**	11,81	11,69	0,12
**FAHD2B**	9,34	9,22	0,12
**APIP**	11,67	11,54	0,12
**WDR24**	10,62	10,50	0,12
**KBTBD4**	10,99	10,87	0,12
**ETFDH**	10,25	10,13	0,12
**ZFPM1**	9,81	9,68	0,12
**GTDC1**	9,91	9,79	0,12
**ACTRT3**	6,23	6,11	0,12
**PRSS3**	1,06	0,94	0,12
**PLEK2**	1,06	0,94	0,12
**ATP6V1B1**	1,06	0,94	0,12
**KCNK15**	1,06	0,94	0,12
**CCR4**	1,06	0,94	0,12
**C1orf146**	1,06	0,94	0,12
**C16orf90**	1,06	0,94	0,12
**ZNF595**	1,06	0,94	0,12
**AL021997.2**	1,06	0,94	0,12
**TBC1D5**	13,30	13,18	0,12
**TTI1**	12,37	12,25	0,12
**M6PR**	13,41	13,29	0,12
**CDC14B**	9,00	8,87	0,12
**PTPRE**	12,16	12,03	0,12
**WDR5B**	10,17	10,04	0,12
**ZNF146**	13,83	13,71	0,12
**COP1**	12,47	12,34	0,12
**ZNF235**	9,21	9,09	0,12
**GPX7**	11,98	11,85	0,12
**SLC23A2**	12,37	12,24	0,12
**EEF2KMT**	10,50	10,37	0,12
**TMEM43**	12,26	12,13	0,12
**CDK5**	10,08	9,95	0,12
**ARHGAP25**	10,93	10,81	0,12
**IQGAP1**	14,82	14,69	0,12
**CCNT2**	12,80	12,67	0,12
**KIF11**	14,21	14,08	0,12
**AASDH**	10,54	10,41	0,12
**EMILIN2**	10,41	10,29	0,12
**WDHD1**	13,09	12,96	0,12
**SRP14**	14,41	14,29	0,12
**HIGD1A**	12,57	12,45	0,12
**SSBP4**	11,43	11,30	0,12
**ECHDC3**	8,72	8,60	0,12
**PLK4**	12,77	12,64	0,13
**RENBP**	8,36	8,24	0,13
**ZC3H12A**	9,07	8,94	0,13

**GPR180**	11,99	11,87	0,13
**DONSON**	11,48	11,35	0,13
**THTPA**	10,23	10,10	0,13
**TLK1**	12,84	12,71	0,13
**BCDIN3D**	9,18	9,06	0,13
**CCNJ**	12,64	12,51	0,13
**ANGEL2**	11,55	11,42	0,13
**DNAL1**	10,46	10,33	0,13
**PGAP6**	11,06	10,94	0,13
**ROCK1**	13,94	13,81	0,13
**ANKRD50**	12,37	12,25	0,13
**HOMER2**	10,60	10,48	0,13
**GAN**	11,50	11,37	0,13
**ERCC6L2**	11,93	11,80	0,13
**PREPL**	12,45	12,33	0,13
**NRAS**	13,67	13,54	0,13
**ZNF879**	10,09	9,96	0,13
**ADAP1**	10,48	10,35	0,13
**SKA1**	11,34	11,21	0,13
**TMEM69**	11,50	11,37	0,13
**SIPA1L3**	10,96	10,84	0,13
**GGH**	13,40	13,28	0,13
**ZNF131**	11,94	11,81	0,13
**RIC1**	13,03	12,90	0,13
**LRRC8B**	12,46	12,34	0,13
**ZNF398**	11,87	11,75	0,13
**SCFD1**	11,82	11,70	0,13
**ANO6**	12,52	12,39	0,13
**LSM8**	13,09	12,97	0,13
**CLK4**	10,91	10,78	0,13
**ADGRA2**	6,48	6,35	0,13
**KIF5B**	13,89	13,76	0,13
**CIITA**	13,64	13,51	0,13
**CASP2**	13,55	13,43	0,13
**ACER3**	11,31	11,18	0,13
**AKAP9**	14,18	14,05	0,13
**NAP1L1**	16,89	16,76	0,13
**THOC2**	13,79	13,66	0,13
**PAG1**	12,82	12,69	0,13
**ITIH4**	7,47	7,34	0,13
**FBXO43**	10,72	10,59	0,13
**MTRF1L**	11,78	11,66	0,13
**STT3B**	15,54	15,42	0,13
**ZFP36L2**	14,07	13,95	0,13
**CACNA1H**	11,52	11,39	0,13
**ARL15**	9,86	9,73	0,13
**TOX2**	4,76	4,64	0,13
**B4GALT1**	12,55	12,42	0,13
**DRAM2**	11,01	10,88	0,13
**ENDOD1**	13,54	13,41	0,13
**CDKL5**	6,27	6,14	0,13
**ZNF10**	10,53	10,41	0,13
**ARHGAP15**	10,34	10,21	0,13
**DUSP3**	9,99	9,86	0,13
**DIPK2A**	11,54	11,41	0,13
**ZNF607**	10,59	10,46	0,13

**F11R**	11,81	11,68	0,13
**RAD21**	15,29	15,16	0,13
**CAMKMT**	9,28	9,16	0,13
**KNTC1**	13,71	13,58	0,13
**TMX3**	12,96	12,84	0,13
**LRRC42**	11,18	11,05	0,13
**MITF**	7,89	7,76	0,13
**FBXL3**	11,87	11,74	0,13
**TPP2**	13,88	13,75	0,13
**RNF24**	11,86	11,73	0,13
**AP3M1**	13,28	13,15	0,13
**TXLNG**	12,69	12,56	0,13
**NOL11**	13,98	13,85	0,13
**SLC2A13**	8,77	8,64	0,13
**CEP350**	13,43	13,30	0,13
**CAMSAP2**	11,57	11,44	0,13
**GNAZ**	9,49	9,36	0,13
**HBS1L**	12,79	12,66	0,13
**FLVCR1**	11,76	11,64	0,13
**MAST3**	10,52	10,40	0,13
**CBWD5**	11,37	11,25	0,13
**PPP1R14D**	1,07	0,94	0,13
**SLC38A6**	7,20	7,07	0,13
**KLHL8**	12,33	12,20	0,13
**CLIC4**	13,71	13,58	0,13
**ETNK1**	13,64	13,51	0,13
**ANGPTL2**	9,91	9,78	0,13
**CUL1**	13,45	13,32	0,13
**LRRC28**	9,74	9,61	0,13
**ABCA2**	11,21	11,08	0,13
**SS18L1**	11,72	11,59	0,13
**RARA**	9,65	9,52	0,13
**TTLL4**	11,84	11,71	0,13
**PARD3**	12,07	11,94	0,13
**NDUFB5**	11,78	11,65	0,13
**GLT8D2**	5,66	5,53	0,13
**CALHM2**	9,17	9,04	0,13
**ASAP2**	12,58	12,45	0,13
**ABRAXAS2**	11,47	11,34	0,13
**SP100**	11,92	11,79	0,13
**SPAG6**	11,04	10,91	0,13
**CMC2**	11,31	11,18	0,13
**ZNF268**	12,02	11,89	0,13
**RTN4IP1**	10,22	10,09	0,13
**VWA7**	7,02	6,89	0,13
**UGP2**	13,37	13,24	0,13
**FAM91A1**	13,50	13,37	0,13
**ZBTB8OS**	7,06	6,93	0,13
**GLG1**	13,59	13,46	0,13
**SCPEP1**	12,04	11,91	0,13
**NIPA1**	11,85	11,72	0,13
**PGAP1**	9,96	9,83	0,13
**RHEBL1**	7,42	7,29	0,13
**RRP15**	12,42	12,29	0,13
**HSD11B1L**	6,70	6,57	0,13
**PATL1**	12,84	12,71	0,13

**ZNF92**	12,25	12,12	0,13
**SCARB2**	12,49	12,36	0,13
**TMEM126A**	11,08	10,95	0,13
**ZNF281**	12,56	12,43	0,13
**MNS1**	9,44	9,31	0,13
**SLC16A7**	11,21	11,08	0,13
**TSPAN13**	11,65	11,52	0,13
**STAM**	10,87	10,74	0,13
**NMNAT1**	8,76	8,63	0,13
**NUS1**	13,47	13,34	0,13
**ARL14EP**	12,21	12,08	0,13
**CEP192**	12,62	12,49	0,13
**ERC1**	11,99	11,85	0,13
**MIS18BP1**	13,38	13,25	0,13
**OLA1**	13,84	13,71	0,13
**NDC1**	13,65	13,52	0,13
**CDIN1**	10,21	10,08	0,13
**TGS1**	13,90	13,77	0,13
**OBI1**	12,63	12,50	0,13
**PROZ**	3,92	3,79	0,13
**ANXA3**	3,92	3,79	0,13
**LY96**	5,89	5,75	0,13
**TMEM135**	11,54	11,41	0,13
**ABHD13**	11,31	11,18	0,13
**PLA2G2C**	3,87	3,74	0,13
**SF3B1**	15,50	15,37	0,13
**DEPDC1B**	12,74	12,61	0,13
**ZYG11A**	3,77	3,63	0,13
**PPP2R3C**	11,93	11,79	0,13
**DMXL2**	11,23	11,10	0,13
**NUP98**	14,05	13,92	0,13
**GZF1**	11,34	11,21	0,13
**ABLIM1**	11,18	11,04	0,13
**CCNC**	12,49	12,36	0,13
**FCF1**	12,46	12,33	0,13
**FAM89A**	9,32	9,19	0,13
**TSNAX**	13,28	13,15	0,13
**MROH1**	9,81	9,68	0,13
**SORL1**	11,79	11,66	0,13
**PCLAF**	13,72	13,59	0,13
**AP3S1**	12,16	12,03	0,13
**GSTM2**	9,00	8,87	0,13
**IGHMBP2**	10,24	10,11	0,13
**PFN2**	10,59	10,46	0,13
**NEK2**	11,78	11,64	0,13
**CDK14**	12,78	12,65	0,13
**XPNPEP2**	7,63	7,50	0,13
**KLHL32**	6,01	5,88	0,13
**PREP**	12,56	12,43	0,13
**PPAT**	13,20	13,06	0,13
**TRIM23**	10,30	10,17	0,13
**NUP50**	14,37	14,23	0,13
**C2orf69**	11,39	11,26	0,13
**SEMA7A**	4,27	4,14	0,13
**CRNKL1**	12,20	12,07	0,13
**DGKH**	13,37	13,24	0,13

**SLC25A32**	13,09	12,96	0,13
**WDR76**	12,57	12,44	0,13
**FLYWCH1**	11,24	11,10	0,13
**CDCA2**	12,77	12,63	0,13
**PPM1E**	10,59	10,46	0,13
**SPIN4**	11,27	11,14	0,13
**KPNA5**	10,83	10,69	0,13
**PTPN11**	14,50	14,37	0,13
**RAPGEF6**	12,59	12,46	0,13
**TST**	10,87	10,73	0,13
**SPCS3**	13,83	13,70	0,13
**PRH1**	3,07	2,93	0,13
**HIBADH**	10,96	10,83	0,13
**PYROXD1**	10,60	10,47	0,13
**NUDCD1**	12,79	12,66	0,13
**GEMIN6**	10,89	10,76	0,13
**OSBPL11**	11,66	11,53	0,13
**TUBGCP2**	11,82	11,69	0,13
**ELL3**	8,12	7,99	0,13
**C1QTNF3**	9,03	8,90	0,13
**PPP1R3D**	9,54	9,41	0,13
**ZNF761**	11,96	11,83	0,13
**AMIGO1**	9,13	9,00	0,13
**UBP1**	13,71	13,58	0,13
**ACCS**	7,41	7,28	0,13
**SON**	15,47	15,34	0,13
**ICE1**	13,45	13,32	0,13
**DIPK1C**	10,54	10,40	0,13
**SLAIN2**	12,05	11,91	0,13
**SLC35E1**	11,51	11,38	0,13
**ZNF605**	11,38	11,25	0,13
**FKBP3**	13,11	12,97	0,13
**ARFIP1**	10,36	10,22	0,13
**ETHE1**	10,21	10,08	0,13
**ACOX3**	10,23	10,10	0,13
**HEXA**	10,87	10,74	0,13
**TPMT**	9,07	8,94	0,13
**UBR2**	12,09	11,96	0,13
**GNPDA2**	11,00	10,86	0,14
**NPRL3**	12,24	12,10	0,14
**MED1**	13,62	13,48	0,14
**UAP1**	12,28	12,15	0,14
**NBDY**	10,45	10,31	0,14
**DLGAP5**	13,33	13,20	0,14
**DEPDC5**	11,45	11,32	0,14
**TMEM14A**	10,31	10,17	0,14
**AIDA**	12,56	12,42	0,14
**ASF1A**	12,07	11,93	0,14
**METTL18**	10,16	10,03	0,14
**CYTH1**	11,90	11,77	0,14
**TMEM199**	11,40	11,27	0,14
**JAK1**	13,27	13,13	0,14
**HSPA14**	11,75	11,62	0,14
**LCORL**	12,00	11,86	0,14
**CD69**	4,87	4,74	0,14
**ARMC2**	7,77	7,63	0,14

**CD44**	13,41	13,28	0,14
**N4BP2L2**	12,62	12,48	0,14
**BCL2L15**	6,96	6,82	0,14
**C1orf109**	10,44	10,31	0,14
**NAMPT**	12,85	12,72	0,14
**SENP6**	13,49	13,35	0,14
**KIAA1217**	9,39	9,25	0,14
**LOX**	5,82	5,69	0,14
**SUV39H2**	12,61	12,47	0,14
**BEND4**	13,06	12,92	0,14
**NUP88**	13,27	13,14	0,14
**LETMD1**	11,18	11,04	0,14
**COL10A1**	3,39	3,25	0,14
**RBL1**	12,74	12,60	0,14
**CHAMP1**	12,89	12,76	0,14
**PHKB**	13,29	13,15	0,14
**VAT1L**	8,30	8,17	0,14
**TMEM167A**	13,13	13,00	0,14
**ZNF682**	10,81	10,67	0,14
**CSMD1**	13,00	12,87	0,14
**GEMIN5**	13,05	12,92	0,14
**MIER1**	12,17	12,03	0,14
**TANC1**	10,60	10,46	0,14
**CCDC186**	11,08	10,94	0,14
**NUP35**	11,08	10,94	0,14
**ADSS2**	13,40	13,26	0,14
**ABRAXAS1**	11,49	11,35	0,14
**DCAF16**	13,29	13,16	0,14
**PLXNB3**	5,44	5,31	0,14
**ZNF620**	7,51	7,37	0,14
**SETD4**	10,68	10,55	0,14
**RAB23**	8,94	8,80	0,14
**GPR35**	7,38	7,24	0,14
**CLASP2**	12,30	12,16	0,14
**GNPNAT1**	12,95	12,81	0,14
**RBMS2**	10,12	9,98	0,14
**MYO5B**	6,41	6,27	0,14
**PRDX4**	12,32	12,18	0,14
**PRDM10**	11,23	11,09	0,14
**PDE4DIP**	11,31	11,17	0,14
**DDX20**	12,49	12,35	0,14
**SPPL2A**	11,28	11,14	0,14
**MON2**	12,17	12,03	0,14
**CD48**	11,37	11,23	0,14
**EMB**	13,98	13,84	0,14
**METTL1**	10,65	10,51	0,14
**DTD1**	9,29	9,15	0,14
**SOCS5**	10,54	10,40	0,14
**FAM76B**	11,92	11,78	0,14
**ZDHHC13**	11,63	11,49	0,14
**PAFAH2**	9,36	9,23	0,14
**HS1BP3**	10,14	10,00	0,14
**IL17RA**	11,52	11,38	0,14
**RNF38**	11,98	11,84	0,14
**RAD17**	11,66	11,53	0,14
**SNRPE**	13,81	13,67	0,14

**WASHC5**	13,01	12,87	0,14
**GTSF1L**	4,02	3,88	0,14
**ERMARD**	10,30	10,16	0,14
**C7orf31**	9,38	9,25	0,14
**PTPN13**	11,33	11,19	0,14
**CCDC82**	11,86	11,72	0,14
**CENPQ**	10,52	10,38	0,14
**AGL**	11,42	11,28	0,14
**PIKFYVE**	13,13	12,99	0,14
**PEX1**	11,15	11,01	0,14
**RIOX2**	12,37	12,23	0,14
**RPL7L1**	14,71	14,57	0,14
**AC008770.4**	7,73	7,59	0,14
**TAOK1**	13,43	13,29	0,14
**GPR176**	13,12	12,98	0,14
**CNOT11**	12,64	12,50	0,14
**CARNMT1**	12,16	12,02	0,14
**EGFL8**	7,63	7,49	0,14
**OGT**	14,35	14,21	0,14
**KAT2B**	10,60	10,46	0,14
**PAQR3**	12,05	11,91	0,14
**MOCS2**	9,69	9,55	0,14
**ZMYND10**	5,77	5,63	0,14
**MELK**	11,95	11,81	0,14
**RNF7**	11,57	11,43	0,14
**TRNT1**	11,90	11,76	0,14
**LARGE1**	10,11	9,97	0,14
**TNFAIP1**	10,47	10,33	0,14
**PTAR1**	13,38	13,24	0,14
**EID2B**	8,96	8,82	0,14
**SCMH1**	12,48	12,34	0,14
**NRBF2**	10,81	10,67	0,14
**FAM171B**	9,23	9,09	0,14
**APAF1**	12,02	11,88	0,14
**NAA35**	11,21	11,06	0,14
**EFR3A**	12,01	11,87	0,14
**HOOK3**	13,94	13,80	0,14
**ANKRD36B**	7,24	7,10	0,14
**KCTD20**	14,13	13,99	0,14
**CERT1**	11,77	11,62	0,14
**ZNF417**	11,32	11,18	0,14
**NUCB2**	13,03	12,89	0,14
**ZXDB**	10,65	10,50	0,14
**NSUN6**	10,18	10,03	0,14
**SLC25A20**	10,45	10,31	0,14
**DLAT**	13,22	13,08	0,14
**ABHD18**	11,49	11,35	0,14
**TSC22D2**	10,15	10,01	0,14
**TOGARAM2**	3,20	3,06	0,14
**VDAC1**	15,19	15,05	0,14
**IL1RAP**	9,32	9,18	0,14
**ZSCAN12**	9,96	9,82	0,14
**ZNF385A**	9,72	9,58	0,14
**ZNF431**	12,72	12,57	0,14
**UBE2E3**	12,94	12,80	0,14
**PRKDC**	16,88	16,73	0,14

**BCL9**	11,51	11,37	0,14
**DIXDC1**	9,98	9,84	0,14
**GTF2A1**	12,75	12,61	0,14
**HMG20A**	12,42	12,28	0,14
**CMTM1**	8,14	8,00	0,14
**CHRNA10**	7,46	7,31	0,14
**ESR1**	6,17	6,02	0,14
**POLR3G**	11,64	11,50	0,14
**COPB1**	13,75	13,61	0,14
**FAM111A**	13,52	13,38	0,14
**AOX1**	7,68	7,54	0,14
**CNKSR2**	10,27	10,13	0,14
**UBE2J1**	13,36	13,22	0,14
**RAD51C**	12,01	11,87	0,14
**MCM8**	13,16	13,02	0,14
**ISOC1**	12,13	11,99	0,14
**ZBTB9**	10,59	10,45	0,14
**SFT2D1**	11,01	10,87	0,14
**NPIPB4**	11,24	11,10	0,14
**ARMC10**	11,95	11,81	0,14
**EEFSEC**	10,84	10,70	0,14
**POGLUT3**	12,32	12,18	0,14
**NGLY1**	12,87	12,73	0,14
**TATDN3**	10,66	10,52	0,14
**CEBPZ**	13,48	13,34	0,14
**AK6**	11,71	11,57	0,14
**NFYA**	10,71	10,57	0,14
**ATAD2**	14,72	14,58	0,14
**PCDHGA2**	6,71	6,56	0,14
**AIMP1**	12,44	12,30	0,14
**TM9SF3**	14,24	14,09	0,14
**LRRC58**	13,95	13,81	0,14
**ATP6V1D**	11,16	11,02	0,14
**TTK**	11,37	11,22	0,14
**NT5DC3**	11,07	10,92	0,14
**INIP**	12,25	12,10	0,14
**AGO4**	11,93	11,79	0,14
**TIPARP**	10,83	10,68	0,14
**CCDC112**	10,74	10,60	0,14
**VRK1**	13,22	13,07	0,14
**CNPY2**	11,51	11,36	0,14
**FASTKD1**	11,53	11,39	0,14
**SYF2**	11,98	11,83	0,14
**COPS4**	12,16	12,01	0,14
**DUSP12**	11,16	11,02	0,14
**C15orf62**	7,08	6,93	0,14
**ACAD11**	10,36	10,22	0,14
**AAGAB**	12,27	12,12	0,14
**CNST**	12,11	11,97	0,14
**CSDE1**	15,88	15,73	0,14
**AL157392.5**	7,98	7,83	0,14
**CLIP1**	12,35	12,20	0,14
**ATMIN**	12,73	12,58	0,14
**TDRD7**	9,50	9,36	0,14
**PDS5A**	14,86	14,71	0,14
**NUDT17**	8,15	8,00	0,14

**USPL1**	11,79	11,65	0,14
**RNF141**	11,17	11,02	0,14
**CIAPIN1**	12,72	12,57	0,14
**COMT**	12,33	12,18	0,14
**CHP1**	12,46	12,32	0,14
**TNS3**	12,76	12,62	0,14
**LRP2**	4,12	3,97	0,14
**MTMR1**	11,47	11,32	0,15
**PSTPIP2**	7,90	7,76	0,15
**CDK12**	13,25	13,10	0,15
**PPM1K**	11,60	11,46	0,15
**OAT**	12,44	12,30	0,15
**PUS7**	12,63	12,48	0,15
**ZMYND12**	5,48	5,34	0,15
**THUMPD1**	12,69	12,54	0,15
**PXYLP1**	10,82	10,67	0,15
**PIK3CG**	13,69	13,54	0,15
**S100A10**	9,83	9,69	0,15
**PHF3**	13,41	13,26	0,15
**SUMO1**	13,63	13,48	0,15
**BUB1B**	13,64	13,49	0,15
**WBP11**	13,61	13,46	0,15
**USP1**	14,16	14,01	0,15
**AP000944.5**	7,62	7,48	0,15
**SBSPON**	8,01	7,86	0,15
**TMEM131**	12,64	12,49	0,15
**TCTN3**	11,58	11,44	0,15
**GATAD2B**	12,80	12,65	0,15
**CUEDC1**	9,53	9,38	0,15
**GABARAPL2**	11,84	11,69	0,15
**ATP1B3**	13,36	13,22	0,15
**MRPS30**	11,69	11,54	0,15
**GSPT2**	10,79	10,65	0,15
**CSE1L**	14,72	14,57	0,15
**SERPINB1**	10,91	10,77	0,15
**EXOC1**	11,54	11,40	0,15
**FAM72B**	11,02	10,87	0,15
**NAPG**	11,12	10,97	0,15
**ZNF367**	12,51	12,36	0,15
**CXCR4**	13,99	13,84	0,15
**GYG1**	12,16	12,01	0,15
**EXOSC9**	13,04	12,89	0,15
**GEM**	5,58	5,43	0,15
**PGRMC1**	12,39	12,24	0,15
**CACNG6**	7,32	7,18	0,15
**ERAP1**	13,37	13,22	0,15
**STX6**	11,80	11,65	0,15
**NME6**	11,76	11,61	0,15
**SUCLG2**	11,53	11,38	0,15
**LSM11**	10,81	10,66	0,15
**OIP5**	10,20	10,06	0,15
**FAM118B**	10,63	10,48	0,15
**NRIP1**	14,99	14,84	0,15
**GPR153**	9,29	9,14	0,15
**NABP1**	10,22	10,07	0,15
**SEC24D**	11,94	11,79	0,15

**TNFRSF10B**	12,10	11,95	0,15
**TAMM41**	7,93	7,79	0,15
**CKAP2L**	12,49	12,34	0,15
**GNB4**	13,60	13,45	0,15
**ZC3H7A**	12,72	12,57	0,15
**COX10**	10,97	10,83	0,15
**NIFK**	13,15	13,00	0,15
**MZT1**	12,29	12,14	0,15
**ZMYM6**	11,17	11,03	0,15
**ADRB2**	8,53	8,38	0,15
**NIT2**	12,16	12,01	0,15
**PPP1R21**	10,93	10,78	0,15
**CCDC122**	8,25	8,10	0,15
**DENND1B**	10,88	10,73	0,15
**RHOT1**	11,72	11,57	0,15
**DNAJC24**	10,52	10,37	0,15
**ADCY9**	12,14	11,99	0,15
**ZNF793**	10,24	10,09	0,15
**ALG13**	11,30	11,15	0,15
**NHLRC3**	10,97	10,82	0,15
**CCNI**	14,59	14,44	0,15
**ATAD5**	12,69	12,54	0,15
**INTS9**	11,97	11,82	0,15
**ABCB10**	12,33	12,18	0,15
**GNA11**	11,52	11,38	0,15
**VMA21**	12,82	12,67	0,15
**WDSUB1**	10,71	10,56	0,15
**NXPE3**	11,40	11,25	0,15
**IFT22**	10,76	10,62	0,15
**TRAM1**	14,43	14,28	0,15
**R3HDM2**	9,71	9,57	0,15
**SH3KBP1**	11,44	11,29	0,15
**MKRN3**	6,50	6,35	0,15
**TSPAN31**	9,86	9,71	0,15
**KCTD9**	12,05	11,90	0,15
**NPIPB2**	9,57	9,42	0,15
**KLHL22**	10,45	10,30	0,15
**AASS**	12,65	12,50	0,15
**ERO1B**	11,26	11,12	0,15
**RB1**	14,39	14,24	0,15
**SCYL2**	12,44	12,29	0,15
**H4C3**	5,07	4,92	0,15
**RIMBP3**	8,72	8,57	0,15
**ASPH**	12,34	12,19	0,15
**UFM1**	13,06	12,91	0,15
**DLG1**	12,84	12,69	0,15
**TTLL9**	3,70	3,55	0,15
**TBC1D23**	11,56	11,41	0,15
**RAMAC**	10,87	10,72	0,15
**RMI1**	12,86	12,71	0,15
**MTIF3**	11,02	10,87	0,15
**EIF2AK4**	12,84	12,69	0,15
**STT3A**	13,62	13,47	0,15
**HMGCL**	9,94	9,79	0,15
**ZXDC**	11,02	10,87	0,15
**ZNF706**	12,43	12,28	0,15

**C5orf22**	11,56	11,41	0,15
**KATNBL1**	11,58	11,43	0,15
**LPGAT1**	13,33	13,18	0,15
**PPME1**	11,88	11,73	0,15
**YWHAZ**	16,32	16,17	0,15
**LILRA2**	11,05	10,90	0,15
**DISC1**	9,54	9,39	0,15
**ZNF626**	10,22	10,07	0,15
**LIN54**	12,12	11,97	0,15
**SH3YL1**	11,91	11,76	0,15
**NCOA6**	12,82	12,66	0,15
**RMND5A**	13,90	13,75	0,15
**PAQR8**	10,89	10,74	0,15
**RORB**	13,75	13,60	0,15
**MAGI2**	7,91	7,76	0,15
**SH3BP4**	7,47	7,32	0,15
**SLC27A1**	9,97	9,82	0,15
**GPLD1**	8,70	8,55	0,15
**CCDC117**	12,86	12,71	0,15
**TGFBR3**	9,80	9,65	0,15
**ZNF639**	12,33	12,18	0,15
**SYT3**	6,76	6,61	0,15
**SGO2**	12,52	12,37	0,15
**PLAA**	12,25	12,10	0,15
**MFSD9**	10,17	10,02	0,15
**SLC30A6**	11,46	11,30	0,15
**DUSP11**	10,95	10,80	0,15
**ATP2B2**	11,46	11,31	0,15
**POLB**	11,65	11,50	0,15
**ZNF718**	10,26	10,11	0,15
**POLK**	11,20	11,05	0,15
**MAPK1IP1L**	13,65	13,50	0,15
**SOCS6**	11,28	11,13	0,15
**TLE1**	12,54	12,39	0,15
**SRP54**	12,62	12,46	0,15
**CBX5**	15,50	15,35	0,15
**AURKC**	4,17	4,02	0,15
**TOX3**	8,19	8,04	0,15
**TRPM7**	13,80	13,65	0,15
**DLD**	13,04	12,88	0,15
**TTPA**	11,74	11,59	0,15
**GPBP1**	13,53	13,37	0,15
**PIAS1**	12,76	12,61	0,15
**KSR1**	9,20	9,05	0,15
**NSD1**	11,58	11,43	0,15
**SPPL3**	11,56	11,41	0,15
**CYRIB**	14,15	13,99	0,15
**SASS6**	11,67	11,52	0,15
**EIF2D**	12,12	11,97	0,15
**CENPH**	12,59	12,44	0,15
**OCRL**	10,63	10,48	0,15
**PROX2**	6,39	6,24	0,15
**HTATIP2**	11,17	11,02	0,15
**INTS2**	12,38	12,23	0,15
**RALGAPA2**	11,41	11,26	0,15
**SLC39A6**	13,11	12,95	0,15

**JAM3**	10,72	10,56	0,15
**GPR63**	10,69	10,53	0,15
**VPS53**	12,16	12,00	0,15
**SPATS2**	11,85	11,70	0,15
**SBNO1**	13,85	13,69	0,15
**GPRIN3**	9,99	9,83	0,15
**TIAM1**	11,98	11,82	0,15
**TAPT1**	10,88	10,73	0,15
**ATPSCKMT**	10,32	10,16	0,15
**GOSR1**	12,52	12,37	0,15
**MMADHC**	12,95	12,79	0,15
**FPGT**	6,87	6,72	0,15
**PRKRA**	12,44	12,28	0,15
**RECQL5**	10,42	10,26	0,15
**STX12**	10,88	10,73	0,15
**FAM107B**	14,34	14,19	0,15
**OAS2**	9,80	9,65	0,16
**BUB1**	13,98	13,82	0,16
**UHRF2**	13,00	12,84	0,16
**ESCO1**	11,40	11,24	0,16
**SMARCAL1**	11,40	11,24	0,16
**FAM153A**	5,16	5,01	0,16
**CSGALNACT2**	11,71	11,55	0,16
**CEP43**	11,65	11,50	0,16
**CHPT1**	10,62	10,47	0,16
**RHBDD1**	10,47	10,31	0,16
**TRIO**	13,38	13,23	0,16
**BCAR3**	10,98	10,82	0,16
**NR2C1**	11,20	11,05	0,16
**GLRX3**	13,32	13,17	0,16
**RHCE**	3,93	3,77	0,16
**WDFY1**	11,74	11,58	0,16
**STXBP3**	11,94	11,79	0,16
**RNFT1**	10,61	10,45	0,16
**PRIM1**	11,95	11,80	0,16
**RNF111**	11,74	11,58	0,16
**AC093155.3**	4,29	4,13	0,16
**NAALAD2**	7,54	7,38	0,16
**ZFX**	13,27	13,11	0,16
**RCN2**	12,61	12,45	0,16
**PCNX4**	12,87	12,72	0,16
**ASAP1**	14,21	14,06	0,16
**XRCC2**	13,08	12,93	0,16
**VEZT**	12,27	12,11	0,16
**MSANTD3**	11,34	11,18	0,16
**TAF1D**	13,39	13,23	0,16
**GFPT1**	13,03	12,88	0,16
**ZNF24**	13,78	13,62	0,16
**ARMT1**	11,29	11,13	0,16
**BEST1**	7,23	7,07	0,16
**ARHGAP21**	12,55	12,40	0,16
**CAND1**	14,40	14,24	0,16
**RASAL2**	10,16	10,01	0,16
**SWT1**	8,41	8,25	0,16
**RFX3**	8,53	8,37	0,16
**RIF1**	14,15	13,99	0,16

PPA1	14,71	14,55	0,16
PIGF	10,38	10,22	0,16
RNF13	11,08	10,92	0,16
ST3GAL4	10,80	10,65	0,16
ADH1B	1,66	1,51	0,16
ERGIC2	11,79	11,63	0,16
MRPL42	13,13	12,97	0,16
NRP1	10,53	10,37	0,16
PIAS2	12,40	12,24	0,16
LRRC57	11,21	11,06	0,16
AASDHPPT	12,82	12,66	0,16
PIGT	11,75	11,60	0,16
HTD2	9,87	9,71	0,16
SLC30A5	12,33	12,18	0,16
ITPRID2	12,79	12,64	0,16
NLK	11,80	11,64	0,16
DENND5B	10,94	10,78	0,16
ZFP1	11,47	11,31	0,16
RBMXL1	11,47	11,31	0,16
TTC37	13,41	13,25	0,16
TEC	12,37	12,21	0,16
ITCH	12,86	12,70	0,16
SETD5	13,77	13,61	0,16
ATAD2B	12,15	11,99	0,16
CITED2	11,54	11,38	0,16
CCNB1IP1	12,51	12,35	0,16
AC068831.6	11,90	11,74	0,16
CNTLN	11,25	11,09	0,16
ANAPC1	14,11	13,95	0,16
ZZZ3	12,39	12,23	0,16
KIAA0586	12,20	12,04	0,16
GTPBP8	10,17	10,01	0,16
PDE7A	13,66	13,50	0,16
PAPSS1	12,46	12,30	0,16
ABHD12B	4,57	4,41	0,16
MCEE	8,55	8,39	0,16
GATM	12,51	12,35	0,16
ARMC1	13,14	12,98	0,16
DSN1	12,11	11,95	0,16
UGCG	11,07	10,92	0,16
TNFRSF10D	10,72	10,57	0,16
ZNF304	10,98	10,82	0,16
ZNF608	12,99	12,83	0,16
USO1	13,11	12,95	0,16
RINT1	11,50	11,34	0,16
ANKIB1	12,07	11,91	0,16
METTL6	10,22	10,06	0,16
ZNF510	11,55	11,39	0,16
CAMKV	6,93	6,77	0,16
NOL8	13,19	13,03	0,16
ZNF484	9,46	9,30	0,16
PHF11	12,45	12,29	0,16
STK4	12,76	12,60	0,16
C16orf72	13,71	13,55	0,16
CTDSP2	13,15	12,99	0,16
PPP2R1B	13,21	13,05	0,16

**DICER1**	13,63	13,47	0,16
**PDE3B**	11,32	11,16	0,16
**AC003002.2**	7,47	7,31	0,16
**SMC2**	14,62	14,46	0,16
**DYNLT1**	10,04	9,88	0,16
**GNMT**	6,19	6,03	0,16
**CLTC**	15,30	15,14	0,16
**MTERF3**	12,36	12,20	0,16
**PPM1D**	11,38	11,22	0,16
**OR10Z1**	5,25	5,09	0,16
**NFE2L2**	12,46	12,29	0,16
**DDHD1**	13,02	12,85	0,16
**CUX1**	13,59	13,43	0,16
**MARCHF3**	10,94	10,78	0,16
**BBOF1**	8,55	8,39	0,16
**PIK3CA**	12,40	12,24	0,16
**AQP6**	4,37	4,20	0,16
**TMEM14C**	11,62	11,46	0,16
**WDR53**	9,91	9,75	0,16
**RSL1D1**	15,09	14,93	0,16
**PCDHGA1**	6,51	6,35	0,16
**ESCO2**	12,95	12,79	0,16
**FBXO9**	11,95	11,79	0,16
**ZNF45**	10,80	10,63	0,16
**NUP133**	13,51	13,35	0,16
**ZFP14**	10,87	10,71	0,16
**XPO6**	13,64	13,48	0,16
**AMMECR1**	12,54	12,38	0,16
**DPH5**	11,45	11,28	0,16
**JAZF1**	10,28	10,12	0,16
**ARHGAP26**	11,59	11,43	0,16
**SHOC2**	13,07	12,91	0,16
**USP49**	8,90	8,74	0,16
**CLOCK**	11,56	11,40	0,16
**CFAP58**	7,88	7,72	0,16
**FANCD2**	13,13	12,97	0,16
**INAFM2**	11,32	11,16	0,16
**RAE1**	12,59	12,43	0,16
**IFIT5**	11,57	11,40	0,16
**CFAP251**	8,68	8,52	0,16
**C5orf15**	12,11	11,95	0,16
**DYRK1A**	12,64	12,47	0,16
**UTP6**	12,91	12,75	0,16
**DOP1A**	6,74	6,58	0,16
**ZNF814**	11,75	11,59	0,16
**EIF2B3**	12,20	12,04	0,16
**SLC5A3**	12,85	12,69	0,16
**KMO**	11,27	11,11	0,16
**FAM210A**	11,30	11,13	0,16
**TOM1L2**	9,72	9,56	0,16
**ZMYM2**	13,32	13,16	0,16
**REST**	12,85	12,69	0,16
**WDR61**	11,98	11,81	0,16
**ARAP2**	11,30	11,14	0,16
**ZDHHC4**	9,88	9,72	0,16
**TSHZ1**	11,44	11,28	0,16

**TMED10**	13,91	13,75	0,16
**NEU3**	12,98	12,82	0,16
**CETN2**	10,93	10,77	0,16
**FLT3LG**	6,53	6,37	0,16
**THADA**	12,08	11,92	0,16
**ZNF737**	12,27	12,10	0,16
**SPAG9**	12,72	12,55	0,16
**RAB2B**	10,94	10,78	0,16
**ADAD2**	4,99	4,82	0,16
**EOGT**	11,12	10,96	0,16
**KDM1B**	12,34	12,18	0,16
**CHRNB1**	8,70	8,54	0,16
**INSC**	7,58	7,42	0,16
**CILK1**	12,14	11,98	0,16
**KPNA3**	13,58	13,42	0,16
**ZNF182**	10,49	10,33	0,16
**TMEM167B**	11,53	11,37	0,16
**LMAN1**	13,74	13,58	0,16
**SLC26A2**	11,88	11,72	0,16
**CCNG1**	13,33	13,17	0,16
**PBLD**	9,31	9,15	0,16
**HSD3B7**	6,81	6,64	0,16
**RAD9B**	7,11	6,94	0,16
**NDUFAF7**	10,69	10,53	0,16
**SLC5A1**	1,67	1,50	0,16
**CDX2**	1,67	1,50	0,16
**SDR9C7**	1,67	1,50	0,16
**C20orf204**	1,67	1,50	0,16
**AC078815.1**	1,67	1,50	0,16
**TKTL1**	1,67	1,50	0,16
**GUCA1A**	1,67	1,50	0,16
**SYDE2**	1,67	1,50	0,16
**KLC3**	1,67	1,50	0,16
**OLFML3**	1,67	1,50	0,16
**IL17C**	1,67	1,50	0,16
**TREM1**	1,67	1,50	0,16
**NXNL2**	1,67	1,50	0,16
**NCAN**	1,67	1,50	0,16
**GFPT2**	1,67	1,50	0,16
**CHI3L1**	1,67	1,50	0,16
**CA1**	1,67	1,50	0,16
**DSG3**	1,67	1,50	0,16
**CD36**	1,67	1,50	0,16
**SULF1**	1,67	1,50	0,16
**ABCG5**	1,67	1,50	0,16
**ADAMTS14**	1,67	1,50	0,16
**PLA1A**	1,67	1,50	0,16
**UCN2**	1,67	1,50	0,16
**IFNA16**	1,67	1,50	0,16
**FCER1G**	1,67	1,50	0,16
**TMEM190**	1,67	1,50	0,16
**FRZB**	1,67	1,50	0,16
**TDRD10**	1,67	1,50	0,16
**TMEM169**	1,67	1,50	0,16
**F2RL1**	1,67	1,50	0,16
**SHH**	1,67	1,50	0,16

**FNDC1**	1,67	1,50	0,16
**CXorf58**	1,67	1,50	0,16
**C12orf50**	1,67	1,50	0,16
**TRPV3**	1,67	1,50	0,16
**PTCRA**	1,67	1,50	0,16
**GJD4**	1,67	1,50	0,16
**TEX44**	1,67	1,50	0,16
**MAF**	1,67	1,50	0,16
**CD300LB**	1,67	1,50	0,16
**CDH5**	1,67	1,50	0,16
**ANKRD62**	1,67	1,50	0,16
**HS3ST4**	1,67	1,50	0,16
**SLC25A21**	1,67	1,50	0,16
**RIPPLY3**	1,67	1,50	0,16
**CCDC73**	1,67	1,50	0,16
**FYB2**	1,67	1,50	0,16
**DPP4**	1,67	1,50	0,16
**GPR52**	1,67	1,50	0,16
**FAM221B**	1,67	1,50	0,16
**IGLJ2**	1,67	1,50	0,16
**KLRK1**	1,67	1,50	0,16
**EBF2**	1,67	1,50	0,16
**ZNF578**	1,67	1,50	0,16
**AC090004.1**	1,67	1,50	0,16
**AC011530.1**	1,67	1,50	0,16
**AC069257.3**	1,67	1,50	0,16
**ELOA3CP**	1,67	1,50	0,16
**H3C1**	1,67	1,50	0,16
**IGLV2-8**	1,67	1,50	0,16
**H4C1**	1,67	1,50	0,16
**AL353795.4**	1,67	1,50	0,16
**LRRC15**	12,69	12,53	0,16
**PKP1**	6,58	6,42	0,16
**ARF6**	13,49	13,32	0,17
**RANBP2**	14,62	14,45	0,17
**MED31**	9,03	8,86	0,17
**IFT57**	10,44	10,27	0,17
**BTBD7**	11,89	11,72	0,17
**CMTM6**	13,39	13,22	0,17
**NSD2**	14,90	14,73	0,17
**FYCO1**	11,04	10,87	0,17
**ARHGEF39**	9,56	9,40	0,17
**NDUFA5**	12,51	12,35	0,17
**CPNE2**	12,36	12,20	0,17
**MT-CO1**	19,18	19,01	0,17
**OSBPL8**	12,44	12,27	0,17
**C16orf74**	9,77	9,60	0,17
**ATG2B**	12,23	12,06	0,17
**TMCO3**	10,90	10,74	0,17
**COG6**	10,81	10,65	0,17
**TMX1**	13,24	13,07	0,17
**C9orf72**	10,29	10,12	0,17
**KRAS**	13,40	13,24	0,17
**WDR36**	13,10	12,93	0,17
**ATP11C**	12,78	12,62	0,17
**PCGF2**	8,16	7,99	0,17

**XYLB**	10,15	9,98	0,17
**SCOC**	11,93	11,76	0,17
**MBNL3**	13,64	13,47	0,17
**SNX6**	13,15	12,98	0,17
**EVI2A**	10,08	9,91	0,17
**CDC42SE2**	12,92	12,75	0,17
**FYTTD1**	13,70	13,54	0,17
**LRP4**	5,90	5,74	0,17
**SMIM17**	6,86	6,70	0,17
**GNPAT**	12,83	12,66	0,17
**ZNF274**	11,62	11,46	0,17
**ZNF507**	12,46	12,29	0,17
**MOB1A**	14,75	14,59	0,17
**NOC3L**	12,31	12,15	0,17
**SUZ12**	13,90	13,73	0,17
**AP1S3**	10,57	10,40	0,17
**PWWP2A**	11,96	11,79	0,17
**NOMO3**	11,84	11,67	0,17
**RWDD3**	10,08	9,92	0,17
**MRTFB**	12,27	12,10	0,17
**MMP14**	11,61	11,44	0,17
**IL7R**	16,78	16,61	0,17
**MFSD4A**	7,44	7,28	0,17
**RSBN1L**	12,68	12,51	0,17
**APPBP2**	11,93	11,76	0,17
**TAB2**	12,40	12,23	0,17
**SRXN1**	9,22	9,05	0,17
**ZNF827**	11,61	11,44	0,17
**SERPINB8**	10,48	10,31	0,17
**WASHC2A**	12,40	12,23	0,17
**TUBA8**	5,60	5,44	0,17
**ETV6**	11,85	11,68	0,17
**ERCC3**	12,23	12,06	0,17
**SPATA6**	9,03	8,86	0,17
**LYRM2**	10,57	10,40	0,17
**WSB2**	11,84	11,67	0,17
**HEYL**	10,59	10,43	0,17
**CRADD**	9,23	9,06	0,17
**SLC31A1**	12,20	12,03	0,17
**SLC7A2**	13,25	13,08	0,17
**C5orf51**	12,05	11,88	0,17
**GSTZ1**	8,93	8,76	0,17
**SQLE**	13,24	13,07	0,17
**DBF4**	12,59	12,42	0,17
**EPDR1**	11,49	11,32	0,17
**AKAP11**	12,87	12,70	0,17
**CCNDBP1**	10,82	10,66	0,17
**QKI**	13,56	13,39	0,17
**PURB**	13,05	12,88	0,17
**RP2**	11,89	11,72	0,17
**C5**	9,25	9,08	0,17
**ZHX1**	12,43	12,26	0,17
**SIK3**	9,64	9,47	0,17
**MARCHF8**	11,03	10,86	0,17
**LAPTM4A**	12,55	12,38	0,17
**FBXL15**	4,76	4,59	0,17

**JAK2**	10,49	10,32	0,17
**ZNF382**	10,48	10,31	0,17
**CSTF1**	12,11	11,94	0,17
**RNF149**	11,37	11,20	0,17
**NEDD1**	12,17	12,00	0,17
**CCDC6**	12,62	12,45	0,17
**PELI1**	10,90	10,73	0,17
**SYNJ2BP**	11,66	11,49	0,17
**ATP7A**	10,26	10,09	0,17
**CCDC14**	14,00	13,83	0,17
**E2F7**	12,47	12,30	0,17
**FLT3**	10,54	10,37	0,17
**ARHGEF19**	9,51	9,34	0,17
**WNK1**	14,09	13,92	0,17
**VPS35**	14,18	14,01	0,17
**PPP1CC**	14,65	14,48	0,17
**ZNF493**	11,76	11,59	0,17
**HYAL3**	8,11	7,94	0,17
**PAFAH1B2**	12,82	12,65	0,17
**MRPS31**	11,44	11,27	0,17
**CRIM1**	11,68	11,51	0,17
**NUP43**	13,25	13,08	0,17
**RTN1**	7,34	7,17	0,17
**GLO1**	13,87	13,70	0,17
**LRIG2**	11,38	11,21	0,17
**PAFAH1B1**	14,08	13,91	0,17
**LMAN2L**	10,55	10,38	0,17
**CAB39**	13,27	13,10	0,17
**PLD2**	9,28	9,11	0,17
**ROBO1**	14,89	14,72	0,17
**USP14**	13,63	13,46	0,17
**NSD3**	14,10	13,93	0,17
**MMP17**	7,80	7,63	0,17
**JMJD1C**	12,91	12,74	0,17
**LYPLAL1**	9,90	9,73	0,17
**CTTNBP2NL**	10,13	9,96	0,17
**CREB1**	13,34	13,17	0,17
**ZNF569**	11,16	10,99	0,17
**MTREX**	13,97	13,80	0,17
**SMTNL2**	5,44	5,27	0,17
**NAT1**	9,98	9,81	0,17
**VAPA**	13,37	13,19	0,17
**AC090517.4**	8,86	8,69	0,17
**DLGAP2**	10,29	10,12	0,17
**WDR12**	12,30	12,13	0,17
**ATPAF1**	12,52	12,34	0,17
**FBXO6**	7,68	7,51	0,17
**SLC30A4**	8,80	8,63	0,17
**SCLT1**	10,55	10,37	0,17
**ZNF486**	11,61	11,44	0,17
**ANXA6**	13,04	12,87	0,17
**SP3**	13,64	13,47	0,17
**CYP51A1**	12,64	12,47	0,17
**OCEL1**	7,90	7,73	0,17
**DDIT3**	8,81	8,64	0,17
**NCOA3**	12,79	12,62	0,17

**ZNF165**	4,58	4,41	0,17
**PHOSPHO2**	7,40	7,23	0,17
**SELENOM**	5,43	5,25	0,17
**VBP1**	13,16	12,99	0,17
**MIS12**	12,06	11,88	0,17
**AC129492.3**	7,39	7,22	0,17
**F13A1**	11,16	10,99	0,17
**SIN3A**	13,49	13,31	0,17
**LBR**	15,03	14,86	0,17
**NMD3**	12,57	12,39	0,17
**FNDC3B**	12,15	11,97	0,17
**PGM2L1**	9,73	9,56	0,17
**MID1**	8,61	8,43	0,17
**SUGCT**	3,67	3,50	0,17
**NXT2**	10,49	10,31	0,17
**SEPTIN7**	13,47	13,30	0,17
**LRRC2**	8,39	8,22	0,17
**MARF1**	12,94	12,77	0,17
**SCAMP1**	11,96	11,79	0,17
**DCK**	13,38	13,20	0,17
**CRLS1**	12,02	11,85	0,17
**FIG4**	9,69	9,52	0,17
**AC138894.1**	8,01	7,83	0,17
**HTT**	8,13	7,96	0,17
**SRGAP2**	12,24	12,07	0,17
**NARS2**	12,03	11,86	0,17
**MKLN1**	12,97	12,80	0,17
**ZNF469**	9,17	8,99	0,17
**IRF8**	10,43	10,26	0,17
**PPHLN1**	13,09	12,92	0,17
**LGALS8**	12,03	11,86	0,17
**SLC4A7**	13,34	13,17	0,17
**NAE1**	13,65	13,47	0,17
**FAR2**	11,73	11,56	0,17
**EIF3M**	14,22	14,04	0,17
**TDP2**	12,48	12,30	0,17
**STRN**	12,49	12,32	0,17
**GLMN**	10,95	10,78	0,17
**MLKL**	10,12	9,95	0,17
**MFSD14B**	12,92	12,75	0,17
**ZNF93**	11,55	11,37	0,17
**CCDC159**	6,06	5,89	0,17
**CCDC66**	11,34	11,17	0,18
**ACP2**	9,45	9,28	0,18
**CHSY1**	12,80	12,63	0,18
**ANKRD26**	9,50	9,32	0,18
**BIVM**	10,24	10,07	0,18
**ZFC3H1**	12,77	12,60	0,18
**ARSK**	9,28	9,10	0,18
**CIPC**	11,62	11,44	0,18
**ARFGEF2**	12,80	12,62	0,18
**CADM2**	7,09	6,91	0,18
**RAB11FIP1**	10,65	10,48	0,18
**SLC30A7**	12,10	11,93	0,18
**PRPF4B**	14,29	14,12	0,18
**TRIP10**	10,01	9,83	0,18

**TMED8**	11,78	11,60	0,18
**PTBP2**	11,86	11,68	0,18
**SMAD5**	13,02	12,85	0,18
**MAP2K6**	10,44	10,26	0,18
**MAGOHB**	11,61	11,43	0,18
**RANBP6**	12,48	12,31	0,18
**LIN7B**	6,97	6,79	0,18
**ARHGEF6**	12,97	12,79	0,18
**PIK3R1**	13,34	13,17	0,18
**HPS5**	11,78	11,61	0,18
**PDE8A**	10,64	10,47	0,18
**AGPS**	12,71	12,53	0,18
**SNAP29**	11,70	11,53	0,18
**WNK2**	10,31	10,14	0,18
**GPATCH11**	11,94	11,77	0,18
**C18orf32**	8,88	8,71	0,18
**HACD2**	12,06	11,88	0,18
**ADD3**	12,95	12,78	0,18
**WDR86**	5,09	4,91	0,18
**IRF4**	13,94	13,76	0,18
**MYT1L**	10,68	10,51	0,18
**ATP13A3**	14,00	13,83	0,18
**SAMD8**	11,25	11,07	0,18
**DDIT4L**	9,05	8,87	0,18
**GAB1**	11,41	11,23	0,18
**ZFP28**	10,15	9,98	0,18
**NUP54**	12,41	12,23	0,18
**ARID2**	13,02	12,85	0,18
**UHMK1**	13,75	13,57	0,18
**AFTPH**	11,88	11,70	0,18
**IRAK4**	11,70	11,52	0,18
**TRPM2**	9,78	9,60	0,18
**EIF3E**	15,78	15,61	0,18
**HPSE**	10,18	10,01	0,18
**DCAF10**	12,08	11,90	0,18
**PACS2**	10,33	10,16	0,18
**RNF217**	4,73	4,55	0,18
**OTUD4**	13,28	13,11	0,18
**SYK**	13,47	13,29	0,18
**ESPN**	6,60	6,42	0,18
**PTK2**	13,61	13,44	0,18
**DNM3**	6,21	6,03	0,18
**RYR3**	6,82	6,64	0,18
**ZDHHC21**	11,19	11,01	0,18
**XPO1**	15,72	15,54	0,18
**TUT4**	13,30	13,13	0,18
**SUCO**	12,07	11,89	0,18
**TRIM33**	13,56	13,38	0,18
**RASL11A**	6,14	5,97	0,18
**MTRNR2L12**	6,85	6,67	0,18
**PRKCB**	10,83	10,65	0,18
**LAMP2**	12,61	12,43	0,18
**LRRN2**	10,55	10,37	0,18
**PARVG**	10,97	10,79	0,18
**DIAPH3**	11,77	11,59	0,18
**VPS26A**	12,59	12,41	0,18

**TOR1A**	11,65	11,47	0,18
**GOLGA5**	10,99	10,81	0,18
**SEC14L1**	12,05	11,87	0,18
**POLE2**	11,07	10,89	0,18
**NES**	5,61	5,43	0,18
**CDYL2**	11,81	11,63	0,18
**FAM117B**	11,27	11,09	0,18
**ZNF654**	12,01	11,83	0,18
**ZNF200**	11,25	11,07	0,18
**FNIP1**	11,36	11,18	0,18
**ZNF138**	10,71	10,54	0,18
**SMARCAD1**	13,30	13,12	0,18
**OPA1**	13,67	13,49	0,18
**GNS**	12,38	12,20	0,18
**PLXNA2**	7,54	7,36	0,18
**ZNF627**	11,13	10,95	0,18
**ZNF473**	10,98	10,80	0,18
**C1orf112**	11,95	11,77	0,18
**LINC02210-CRHR1**	7,36	7,18	0,18
**DOCK11**	12,72	12,54	0,18
**PRKAR2B**	10,02	9,84	0,18
**TMEM117**	9,71	9,53	0,18
**ATRN**	12,11	11,93	0,18
**CPPED1**	10,44	10,26	0,18
**OAS3**	12,05	11,87	0,18
**AC112128.1**	9,39	9,21	0,18
**SNAI3**	7,37	7,19	0,18
**INTS7**	12,93	12,75	0,18
**TMEM53**	7,80	7,62	0,18
**ERCC4**	10,80	10,62	0,18
**ZNF845**	11,30	11,12	0,18
**MILR1**	10,19	10,01	0,18
**DMTF1**	12,48	12,30	0,18
**C1orf115**	8,14	7,96	0,18
**BLOC1S6**	13,09	12,91	0,18
**TRAF5**	11,08	10,90	0,18
**ZNF426**	11,88	11,70	0,18
**NUP155**	13,46	13,27	0,18
**SPATA7**	9,14	8,96	0,18
**ERN1**	9,90	9,72	0,18
**MICOS10-NBL1**	9,74	9,56	0,18
**ZCRB1**	11,56	11,38	0,18
**PAPOLA**	11,61	11,43	0,18
**C17orf75**	11,46	11,28	0,18
**FAM102B**	12,48	12,30	0,18
**TFAP2A**	5,56	5,38	0,18
**SLC35A3**	11,88	11,70	0,18
**SLC38A2**	14,65	14,47	0,18
**GKAP1**	9,13	8,95	0,18
**CENPC**	12,07	11,89	0,18
**NAA16**	11,41	11,23	0,18
**DUSP16**	10,70	10,52	0,18
**CERS5**	11,24	11,05	0,18
**KMT2E**	13,53	13,35	0,18
**DUS4L**	9,70	9,52	0,18
**CD40**	8,41	8,23	0,18

**UBLCP1**	9,00	8,81	0,18
**ERCC6L**	11,38	11,20	0,18
**CSNK1G3**	11,40	11,22	0,18
**KIAA1958**	11,81	11,63	0,18
**GNA13**	13,65	13,47	0,18
**ZNF670**	11,40	11,21	0,18
**ADAMTS1**	9,69	9,50	0,18
**PLD1**	11,60	11,42	0,18
**KCNK9**	9,31	9,12	0,18
**MUC4**	6,35	6,16	0,18
**PERCC1**	6,27	6,09	0,18
**SDCBP**	13,11	12,92	0,18
**ADNP**	14,47	14,28	0,18
**AP1S2**	12,33	12,15	0,18
**AC093668.1**	6,98	6,80	0,18
**TWSG1**	10,91	10,73	0,18
**FKBP10**	7,65	7,47	0,18
**SDHAF4**	9,02	8,83	0,18
**METTL15**	10,66	10,48	0,18
**HCFC2**	10,28	10,09	0,18
**FAM135A**	11,40	11,22	0,18
**USP34**	14,70	14,51	0,18
**AKTIP**	9,09	8,90	0,18
**ENG**	11,77	11,59	0,19
**CBL**	13,05	12,86	0,19
**MAP3K8**	8,32	8,14	0,19
**TET2**	11,22	11,04	0,19
**PTGS1**	7,62	7,44	0,19
**DNAJC15**	10,36	10,18	0,19
**POU2F1**	13,05	12,87	0,19
**SAMD9**	12,01	11,82	0,19
**PICALM**	12,89	12,71	0,19
**GOLGA6A**	2,09	1,91	0,19
**IQCD**	2,09	1,91	0,19
**HIGD1C**	2,09	1,91	0,19
**IGHV4-59**	2,09	1,91	0,19
**MAP1LC3B2**	2,09	1,91	0,19
**RIN3**	7,89	7,70	0,19
**ACSL4**	12,87	12,68	0,19
**SOGA3**	5,25	5,06	0,19
**SDCBP2**	6,20	6,01	0,19
**KCNQ3**	11,26	11,07	0,19
**HOXA4**	5,60	5,41	0,19
**DHX36**	13,31	13,13	0,19
**IFIT3**	10,08	9,89	0,19
**PMS1**	12,00	11,81	0,19
**ITGB1**	14,00	13,82	0,19
**ZNF675**	11,42	11,23	0,19
**ATPAF2**	10,16	9,97	0,19
**SUPT20H**	12,26	12,07	0,19
**EIF2S1**	14,10	13,92	0,19
**S100A11**	7,88	7,69	0,19
**TMEM30A**	12,91	12,73	0,19
**SUCLA2**	12,05	11,86	0,19
**PRPF18**	11,05	10,87	0,19
**RMDN1**	11,63	11,44	0,19

**CLIC6**	5,42	5,23	0,19
**SYT1**	11,78	11,59	0,19
**OAS1**	8,13	7,94	0,19
**GMFB**	12,57	12,38	0,19
**ERV3-1**	11,24	11,05	0,19
**ZNF551**	11,55	11,36	0,19
**LURAP1**	5,82	5,63	0,19
**GNL3L**	13,49	13,30	0,19
**SEC23A**	12,46	12,27	0,19
**ABHD11**	5,18	4,99	0,19
**ACVR2A**	8,55	8,37	0,19
**BBS10**	10,08	9,89	0,19
**IPMK**	10,97	10,78	0,19
**DCUN1D3**	9,74	9,55	0,19
**NKRF**	11,76	11,57	0,19
**FOXN3**	12,90	12,71	0,19
**RPP40**	10,58	10,40	0,19
**TRANK1**	11,61	11,42	0,19
**AL591806.2**	9,29	9,10	0,19
**CTNND1**	10,94	10,75	0,19
**ZNF514**	11,18	10,99	0,19
**PTBP3**	13,61	13,42	0,19
**HSDL2**	11,90	11,71	0,19
**ZNF25**	9,22	9,03	0,19
**SLC35D1**	12,01	11,82	0,19
**APBB1**	9,98	9,79	0,19
**SELENOF**	13,82	13,63	0,19
**RAB6A**	13,06	12,87	0,19
**IL15**	5,73	5,54	0,19
**MAP3K4**	12,64	12,45	0,19
**STAM2**	11,19	11,00	0,19
**HELZ**	12,64	12,45	0,19
**GVQW3**	11,17	10,98	0,19
**RPAP2**	12,40	12,21	0,19
**CEP19**	9,10	8,91	0,19
**SRRD**	12,46	12,27	0,19
**IQGAP2**	13,59	13,40	0,19
**TMEM59**	11,84	11,65	0,19
**PSD3**	13,86	13,67	0,19
**FRMPD1**	8,50	8,31	0,19
**BCL2L2-PABPN1**	8,17	7,97	0,19
**ASAH1**	12,38	12,19	0,19
**EDEM1**	16,87	16,68	0,19
**RHOBTB3**	11,76	11,56	0,19
**USP18**	6,46	6,27	0,19
**COX15**	12,14	11,95	0,19
**APOBEC2**	5,03	4,83	0,19
**UBXN2A**	12,29	12,09	0,19
**SLC16A5**	6,93	6,74	0,19
**ZBTB33**	12,94	12,74	0,19
**CDK13**	12,80	12,61	0,19
**TTC14**	12,63	12,44	0,19
**SLC16A2**	11,14	10,94	0,19
**RPS6KA5**	9,65	9,46	0,19
**ZNF468**	11,41	11,22	0,19
**LIMCH1**	9,02	8,83	0,19

**SAMD3**	5,71	5,52	0,19
**UCHL3**	11,25	11,06	0,19
**TTC9**	6,48	6,29	0,19
**TCF7L2**	10,94	10,75	0,19
**GCC2**	11,94	11,75	0,19
**DHX35**	11,07	10,88	0,19
**DARS2**	13,01	12,82	0,19
**VNN2**	4,60	4,40	0,19
**PRKCQ**	9,89	9,70	0,19
**USP24**	13,68	13,49	0,19
**FCHO2**	8,66	8,47	0,19
**PER3**	9,11	8,92	0,19
**KCNAB1**	5,95	5,76	0,19
**ZNF251**	11,35	11,15	0,19
**ELP2**	12,83	12,64	0,19
**ST7L**	9,01	8,81	0,19
**RHPN2**	8,19	8,00	0,19
**NEDD4**	11,54	11,35	0,19
**LNPK**	12,16	11,97	0,19
**FAM160A1**	5,07	4,88	0,19
**GYG2**	8,40	8,20	0,19
**ZNF766**	11,96	11,76	0,19
**NR2C2**	12,76	12,57	0,19
**TTC33**	11,16	10,96	0,19
**BTG1**	12,65	12,46	0,19
**HEBP2**	10,96	10,76	0,19
**DPY19L2**	8,25	8,05	0,19
**SLC27A2**	11,58	11,38	0,19
**RNPC3**	11,85	11,66	0,19
**RHBDL2**	3,74	3,55	0,19
**CFAP92**	8,58	8,39	0,20
**MIB1**	13,31	13,11	0,20
**FCRLB**	6,94	6,75	0,20
**CD46**	12,83	12,63	0,20
**GFM2**	11,98	11,78	0,20
**STK26**	13,41	13,21	0,20
**CHEK1**	13,11	12,92	0,20
**GLP1R**	10,54	10,35	0,20
**UBE3D**	8,46	8,27	0,20
**UCHL5**	13,14	12,95	0,20
**ABCC2**	8,41	8,22	0,20
**SPICE1**	10,02	9,82	0,20
**ATP11B**	12,81	12,61	0,20
**IFI16**	14,12	13,93	0,20
**LNPEP**	13,30	13,10	0,20
**LIPA**	12,22	12,02	0,20
**PPP1R16B**	10,44	10,24	0,20
**HIF1A**	13,72	13,52	0,20
**HERPUD1**	12,52	12,32	0,20
**OPN3**	12,63	12,43	0,20
**APLF**	8,32	8,13	0,20
**MIER3**	12,71	12,51	0,20
**EIF2AK2**	13,52	13,32	0,20
**ZSCAN26**	10,16	9,97	0,20
**SCN1B**	5,68	5,49	0,20
**KCTD6**	9,98	9,78	0,20

TEAD3	6,60	6,40	0,20
CCRL2	6,05	5,86	0,20
CILP	6,05	5,86	0,20
EHBP1	11,48	11,28	0,20
LIMS2	10,62	10,42	0,20
CEPT1	11,27	11,07	0,20
ELK4	13,69	13,49	0,20
XPOT	14,85	14,65	0,20
SETD2	12,31	12,11	0,20
TMEM19	11,37	11,17	0,20
ZNF770	13,37	13,18	0,20
SPDYE6	8,56	8,36	0,20
SGPL1	11,79	11,60	0,20
TASL	15,40	15,21	0,20
STOX1	9,74	9,54	0,20
FSD1L	11,29	11,09	0,20
USP47	13,24	13,05	0,20
EPHA8	2,42	2,22	0,20
SPP2	2,42	2,22	0,20
MECOM	2,42	2,22	0,20
CPVL	2,42	2,22	0,20
ID1	2,42	2,22	0,20
EDN2	2,42	2,22	0,20
HCN4	2,42	2,22	0,20
PTPRQ	2,42	2,22	0,20
LRRC18	2,42	2,22	0,20
AK8	2,42	2,22	0,20
MSRB3	2,42	2,22	0,20
STX19	2,42	2,22	0,20
SNORC	2,42	2,22	0,20
GALR2	2,42	2,22	0,20
KPNA7	2,42	2,22	0,20
NPIPA8	2,42	2,22	0,20
KCNQ1	2,42	2,22	0,20
TMEM156	2,42	2,22	0,20
ASCL1	2,42	2,22	0,20
SFTPB	2,42	2,22	0,20
ADRA1D	2,42	2,22	0,20
RTN3	13,67	13,47	0,20
ACADSB	11,68	11,48	0,20
STX17	12,01	11,81	0,20
FBXO3	11,68	11,48	0,20
ARHGAP11A	13,71	13,51	0,20
STK11IP	11,13	10,94	0,20
ATXN2	11,87	11,68	0,20
AFF3	11,15	10,95	0,20
NPEPPS	13,36	13,16	0,20
HSF2	11,61	11,41	0,20
SNAPC3	11,45	11,25	0,20
MAP3K5	11,34	11,14	0,20
SMC6	13,30	13,10	0,20
NIPSNAP2	12,80	12,60	0,20
PIGN	11,96	11,76	0,20
ANKRD36	11,30	11,10	0,20
RRM2B	11,92	11,73	0,20
MSH6	13,73	13,53	0,20

**MAN1A2**	12,64	12,44	0,20
**LRP6**	12,71	12,52	0,20
**ZNF185**	7,08	6,88	0,20
**C8orf37**	9,34	9,14	0,20
**CEP126**	7,84	7,65	0,20
**KCNC4**	9,77	9,57	0,20
**ETAA1**	11,28	11,08	0,20
**CFL2**	9,98	9,78	0,20
**LRRC39**	7,17	6,97	0,20
**TMEM184C**	11,88	11,68	0,20
**HAUS6**	13,69	13,49	0,20
**CTH**	7,44	7,24	0,20
**TRIM37**	11,85	11,65	0,20
**SKIL**	12,22	12,02	0,20
**BRCA2**	12,66	12,46	0,20
**IPPK**	10,33	10,13	0,20
**RILPL1**	7,66	7,46	0,20
**DCP2**	13,64	13,44	0,20
**GNGT2**	7,47	7,27	0,20
**CASD1**	12,11	11,91	0,20
**NUP85**	12,62	12,42	0,20
**LRRC37A**	9,48	9,28	0,20
**PARP15**	12,31	12,11	0,20
**PHIP**	13,46	13,26	0,20
**SMYD3**	11,19	10,98	0,20
**GABPA**	13,06	12,86	0,20
**TRMT10C**	12,63	12,43	0,20
**ASNSD1**	12,17	11,97	0,20
**RNF169**	12,43	12,23	0,20
**WDR26**	13,17	12,97	0,20
**ARHGAP18**	9,90	9,70	0,20
**TMEM41B**	11,49	11,29	0,20
**PIK3CB**	11,24	11,04	0,20
**CHSY3**	6,21	6,01	0,20
**STARD3NL**	11,54	11,34	0,20
**TMED1**	10,22	10,02	0,20
**CEP76**	11,47	11,27	0,20
**MBNL1**	12,76	12,56	0,20
**PDZD8**	12,42	12,22	0,20
**ZNF616**	11,36	11,16	0,20
**PLXNC1**	9,66	9,46	0,20
**CCDC88A**	14,34	14,14	0,20
**EPHA3**	15,39	15,19	0,20
**AC120114.4**	9,37	9,17	0,20
**RIOK3**	12,08	11,88	0,20
**TEX15**	11,78	11,58	0,20
**SFXN1**	13,51	13,31	0,20
**PAK2**	14,45	14,25	0,20
**ZNF594**	10,93	10,73	0,20
**AFMID**	10,40	10,19	0,20
**MAPRE2**	12,85	12,65	0,20
**MYB**	15,65	15,45	0,20
**PTGR2**	8,36	8,16	0,20
**ROCK2**	13,13	12,93	0,20
**IGHV4-31**	5,37	5,16	0,20
**FZD3**	10,64	10,44	0,20

**MARCHF1**	10,20	10,00	0,20
**CEMIP2**	12,09	11,88	0,20
**METTL25**	8,71	8,50	0,20
**TMED7**	12,12	11,92	0,20
**CCDC90B**	10,97	10,76	0,20
**PISD**	9,58	9,37	0,20
**DBR1**	11,74	11,54	0,20
**CCSAP**	12,08	11,87	0,20
**RNF11**	11,53	11,33	0,20
**METTL2A**	11,49	11,29	0,20
**USP6NL**	8,79	8,58	0,20
**ZNF329**	10,65	10,45	0,20
**ATG5**	11,61	11,40	0,21
**LIPT1**	9,84	9,64	0,21
**MROH7**	5,90	5,69	0,21
**MBTD1**	12,41	12,20	0,21
**RAG2**	13,25	13,04	0,21
**S100A13**	9,36	9,15	0,21
**YTHDC2**	12,55	12,34	0,21
**PHTF2**	11,69	11,48	0,21
**EVI2B**	11,16	10,96	0,21
**CERS6**	13,28	13,08	0,21
**ETNK2**	8,83	8,62	0,21
**PDIA5**	9,68	9,47	0,21
**PDE6G**	7,08	6,88	0,21
**RAPH1**	2,69	2,48	0,21
**MMRN2**	2,69	2,48	0,21
**IGHV4-34**	2,69	2,48	0,21
**C12orf71**	2,69	2,48	0,21
**SLC35E3**	12,32	12,12	0,21
**ANKRD46**	11,20	11,00	0,21
**PKP2**	8,21	8,00	0,21
**RARS2**	8,44	8,23	0,21
**SNX14**	10,16	9,95	0,21
**MTR**	13,69	13,48	0,21
**DPY19L3**	12,31	12,10	0,21
**APPL1**	13,19	12,98	0,21
**NAIP**	11,65	11,44	0,21
**CCDC113**	8,28	8,07	0,21
**ASPM**	14,30	14,09	0,21
**CTRC**	3,75	3,54	0,21
**NCR1**	2,66	2,46	0,21
**ECT2**	12,36	12,15	0,21
**KANSL1L**	11,00	10,79	0,21
**NDUFAF1**	11,21	11,01	0,21
**LAMA3**	9,37	9,17	0,21
**ADAL**	9,58	9,38	0,21
**MARCHF2**	8,46	8,26	0,21
**ATF2**	12,43	12,22	0,21
**BX664615.2**	6,39	6,18	0,21
**LRRC8C**	13,25	13,05	0,21
**PPP1R12A**	13,41	13,20	0,21
**LRRC37B**	9,99	9,78	0,21
**TSPAN2**	9,35	9,15	0,21
**KNL1**	13,68	13,48	0,21
**PIGX**	11,54	11,33	0,21

**ZNF791**	11,16	10,95	0,21
**IREB2**	13,85	13,64	0,21
**ZNF254**	12,08	11,88	0,21
**CHRNB2**	5,51	5,31	0,21
**SACS**	13,99	13,78	0,21
**DIP2B**	13,00	12,79	0,21
**TRMT9B**	10,20	9,99	0,21
**BAG2**	12,55	12,34	0,21
**CD276**	9,18	8,97	0,21
**MDN1**	13,29	13,08	0,21
**CRYBG1**	11,61	11,40	0,21
**TCF4**	15,13	14,93	0,21
**IARS1**	15,18	14,97	0,21
**DCBLD2**	11,65	11,44	0,21
**ZFAND2A**	8,94	8,73	0,21
**ADCK1**	8,68	8,47	0,21
**ZNF678**	11,50	11,30	0,21
**RGS17**	7,71	7,50	0,21
**TMED7-TICAM2**	8,54	8,33	0,21
**TSC1**	12,22	12,01	0,21
**PTPRK**	12,19	11,98	0,21
**KPTN**	9,05	8,84	0,21
**KYAT3**	11,15	10,94	0,21
**ZSCAN30**	10,66	10,45	0,21
**UBQLNL**	5,58	5,37	0,21
**CNN3**	12,79	12,58	0,21
**NBPF11**	11,69	11,48	0,21
**ITGA11**	6,50	6,29	0,21
**DCUN1D1**	12,22	12,01	0,21
**EVC**	9,91	9,70	0,21
**PHC1**	12,02	11,81	0,21
**SOCS4**	13,27	13,06	0,21
**C11orf65**	5,68	5,47	0,21
**KRCC1**	11,36	11,15	0,21
**IL10RB**	10,45	10,24	0,21
**MAPK7**	9,57	9,36	0,21
**DNAJC3**	12,56	12,35	0,21
**DEGS1**	11,73	11,52	0,21
**TMEM87B**	11,27	11,06	0,21
**C1orf94**	3,54	3,33	0,21
**GALNT3**	9,03	8,82	0,21
**CCDC163**	7,27	7,06	0,21
**RNF213**	12,90	12,68	0,21
**ZFAND6**	11,19	10,98	0,21
**DENND5A**	12,21	11,99	0,21
**TCEA3**	6,00	5,79	0,21
**SLC23A1**	8,14	7,93	0,21
**TMEM100**	8,88	8,67	0,21
**CDH1**	2,91	2,70	0,21
**C20orf203**	2,91	2,70	0,21
**ITM2A**	2,91	2,70	0,21
**PLA2G4A**	2,91	2,70	0,21
**FBN2**	2,91	2,70	0,21
**KCNJ9**	2,91	2,70	0,21
**GPR22**	2,91	2,70	0,21
**DNAH14**	2,91	2,70	0,21

**AC012213.5**	2,91	2,70	0,21
**SOX8**	2,91	2,70	0,21
**WNT8B**	2,91	2,70	0,21
**OLFM4**	2,91	2,70	0,21
**CYP4F2**	2,91	2,70	0,21
**SORBS1**	14,12	13,91	0,21
**PUS3**	10,24	10,02	0,21
**GSTM4**	10,48	10,27	0,21
**NFIL3**	10,61	10,40	0,21
**KIF20B**	13,70	13,48	0,21
**PFKFB1**	4,81	4,60	0,21
**PUM2**	14,45	14,24	0,21
**GPD2**	12,65	12,43	0,21
**DYNLT3**	9,81	9,59	0,21
**EXOC5**	12,71	12,50	0,21
**RLIM**	13,05	12,84	0,21
**SUSD3**	10,14	9,93	0,21
**FRS2**	11,10	10,89	0,21
**TM2D3**	11,09	10,88	0,21
**PLEKHG4**	8,74	8,53	0,21
**C4orf46**	12,71	12,49	0,21
**TRMT11**	9,75	9,54	0,21
**MAN2A1**	12,59	12,38	0,21
**CBFA2T3**	10,56	10,34	0,21
**FAM227A**	8,65	8,44	0,21
**STK31**	6,06	5,85	0,21
**MRE11**	13,19	12,98	0,21
**USP3**	12,88	12,66	0,21
**FUT11**	9,26	9,05	0,21
**BRWD3**	11,13	10,92	0,21
**PDE5A**	10,26	10,04	0,21
**TCTN2**	9,27	9,06	0,21
**CRYZ**	11,45	11,24	0,21
**NAPB**	10,55	10,34	0,21
**DNAJC10**	13,75	13,54	0,21
**EGLN1**	13,00	12,79	0,21
**GPM6B**	11,44	11,23	0,21
**CREB3L2**	11,59	11,37	0,22
**SPX**	10,08	9,86	0,22
**ACSBG1**	5,82	5,61	0,22
**TFAP2E**	5,82	5,61	0,22
**WDR47**	11,09	10,88	0,22
**TMEM263**	12,68	12,46	0,22
**FP565260.3**	8,83	8,62	0,22
**IGF2BP3**	6,54	6,33	0,22
**ZNF100**	12,47	12,26	0,22
**ZMPSTE24**	12,88	12,66	0,22
**SLC16A6**	8,17	7,96	0,22
**PCDHGA8**	4,22	4,01	0,22
**PDIK1L**	11,32	11,11	0,22
**DCAF17**	11,84	11,63	0,22
**SESTD1**	12,08	11,87	0,22
**FSBP**	10,16	9,94	0,22
**TOB1**	11,97	11,76	0,22
**ANAPC4**	11,66	11,45	0,22
**MAPK8**	11,68	11,47	0,22

**ANKRD28**	12,64	12,43	0,22
**FAM124A**	8,63	8,41	0,22
**DHDDS**	11,26	11,04	0,22
**AFF2**	14,60	14,38	0,22
**RAVER2**	12,34	12,12	0,22
**TMEM116**	8,67	8,45	0,22
**MORC3**	10,79	10,57	0,22
**IL15RA**	8,37	8,16	0,22
**SLC18A2**	3,11	2,89	0,22
**KRTAP5-10**	3,11	2,89	0,22
**ITGAV**	11,39	11,18	0,22
**FER**	10,26	10,05	0,22
**IL17RB**	6,79	6,58	0,22
**TLCD1**	7,49	7,27	0,22
**TGFBR1**	10,86	10,65	0,22
**POMT1**	9,49	9,27	0,22
**EBAG9**	10,93	10,72	0,22
**SLC8A1**	11,81	11,59	0,22
**PRKAR2A**	11,94	11,72	0,22
**SGO1**	11,76	11,54	0,22
**LIN9**	11,62	11,40	0,22
**TRIB1**	10,92	10,70	0,22
**NDEL1**	11,25	11,03	0,22
**THAP2**	9,76	9,54	0,22
**TWF1**	11,65	11,43	0,22
**RNF34**	10,72	10,50	0,22
**ALG5**	11,34	11,12	0,22
**CD33**	2,67	2,45	0,22
**TMEM138**	8,79	8,57	0,22
**MMP2**	7,86	7,64	0,22
**ANKRD36C**	10,75	10,53	0,22
**STK3**	11,61	11,39	0,22
**AK4**	12,96	12,74	0,22
**CCDC85A**	3,28	3,06	0,22
**SEMA3A**	3,28	3,06	0,22
**RGS13**	3,28	3,06	0,22
**RGS20**	3,28	3,06	0,22
**TRABD2A**	3,28	3,06	0,22
**AC109583.1**	3,28	3,06	0,22
**IGHV3-74**	3,28	3,06	0,22
**GALNT15**	3,28	3,06	0,22
**FRAS1**	3,28	3,06	0,22
**ISCA1**	11,91	11,69	0,22
**RAPGEF5**	11,94	11,72	0,22
**SRPK1**	13,88	13,66	0,22
**PHC3**	12,49	12,27	0,22
**SLMAP**	12,32	12,10	0,22
**PRKACB**	9,79	9,57	0,22
**ZFAND1**	11,57	11,35	0,22
**PTP4A1**	12,96	12,74	0,22
**PCM1**	14,26	14,04	0,22
**TRAF6**	11,58	11,36	0,22
**NPIPB7**	6,18	5,96	0,22
**OGN**	4,15	3,93	0,22
**CCDC74B**	7,47	7,25	0,22
**DBT**	11,18	10,95	0,22

**SLC46A3**	8,00	7,78	0,22
**ITPR2**	11,07	10,85	0,22
**LIG4**	11,22	10,99	0,22
**CTDSPL2**	13,32	13,10	0,22
**GRIA4**	3,43	3,21	0,22
**ZNF217**	13,08	12,86	0,22
**ZNF43**	12,70	12,48	0,22
**AC131160.1**	7,13	6,91	0,22
**RFC4**	12,59	12,37	0,22
**ZNF765**	11,42	11,20	0,22
**CMAS**	11,79	11,57	0,22
**RWDD2B**	8,67	8,45	0,22
**ADAM9**	11,71	11,49	0,22
**LIG3**	12,20	11,97	0,22
**BRWD1**	13,17	12,94	0,22
**MERTK**	10,30	10,08	0,22
**MYBPH**	10,84	10,62	0,22
**RESF1**	12,47	12,24	0,22
**REL**	11,01	10,79	0,22
**FGFR4**	9,11	8,89	0,22
**ALS2**	11,58	11,35	0,22
**SLC35F3**	6,33	6,11	0,22
**ZNF438**	8,30	8,08	0,22
**CDK17**	11,58	11,36	0,22
**HSD11B1**	3,57	3,35	0,22
**GLRA3**	3,57	3,35	0,22
**ZNF556**	3,57	3,35	0,22
**OLFML2A**	3,57	3,35	0,22
**NRN1**	3,57	3,35	0,22
**MGARP**	3,57	3,35	0,22
**MAEL**	3,57	3,35	0,22
**VWA2**	3,57	3,35	0,22
**C17orf50**	3,57	3,35	0,22
**NPAT**	12,22	12,00	0,22
**MINDY3**	10,51	10,28	0,23
**RNF139**	11,91	11,69	0,23
**THAP12**	12,15	11,93	0,23
**ZNF266**	11,58	11,36	0,23
**MIGA2**	9,64	9,41	0,23
**POP4**	11,54	11,31	0,23
**MPP5**	11,33	11,11	0,23
**AL034430.1**	7,82	7,60	0,23
**CD109**	6,97	6,75	0,23
**DCLRE1A**	9,06	8,84	0,23
**SLC35A1**	10,29	10,06	0,23
**GTPBP10**	12,08	11,86	0,23
**UBN2**	11,84	11,62	0,23
**POLQ**	12,90	12,67	0,23
**MTMR6**	11,85	11,63	0,23
**ZNF30**	8,71	8,49	0,23
**ZNF730**	10,50	10,27	0,23
**CCDC50**	12,29	12,06	0,23
**NKAPL**	3,70	3,47	0,23
**CNOT6**	13,28	13,05	0,23
**DQX1**	3,60	3,37	0,23
**ABCA1**	12,69	12,47	0,23

**BDP1**	13,42	13,19	0,23
**HPGD**	1,70	1,47	0,23
**GUF1**	12,61	12,39	0,23
**WNT3**	7,97	7,74	0,23
**LRRC6**	5,47	5,24	0,23
**ATP8B4**	7,52	7,29	0,23
**POMK**	11,98	11,76	0,23
**CPNE3**	13,61	13,38	0,23
**SLC28A2**	3,82	3,59	0,23
**RIPOR3**	3,82	3,59	0,23
**COL4A4**	3,82	3,59	0,23
**OR2B6**	3,82	3,59	0,23
**ADAM21**	3,82	3,59	0,23
**COL1A2**	3,82	3,59	0,23
**ROR2**	3,82	3,59	0,23
**CST7**	9,97	9,74	0,23
**DESI2**	13,57	13,34	0,23
**VPS13C**	13,74	13,51	0,23
**CEP41**	11,78	11,56	0,23
**WASHC4**	13,03	12,80	0,23
**DCLK2**	9,89	9,66	0,23
**RSAD2**	8,26	8,04	0,23
**EXOC6B**	9,39	9,17	0,23
**GDAP2**	10,00	9,77	0,23
**HSD17B11**	10,53	10,30	0,23
**ZBTB41**	11,39	11,16	0,23
**ZNF829**	10,28	10,05	0,23
**PCSK5**	8,06	7,83	0,23
**GSDME**	8,95	8,72	0,23
**CD83**	10,92	10,69	0,23
**FGGY**	8,51	8,28	0,23
**PHF6**	13,24	13,01	0,23
**DENND2A**	7,00	6,77	0,23
**PCCA**	8,90	8,67	0,23
**SMAD9**	10,81	10,58	0,23
**USP30**	10,39	10,16	0,23
**TMEM184A**	4,02	3,79	0,23
**VSIR**	4,02	3,79	0,23
**EPHX4**	4,02	3,79	0,23
**SAMD7**	4,02	3,79	0,23
**HLA-DOB**	8,06	7,83	0,23
**LHFPL2**	9,64	9,41	0,23
**LINS1**	9,93	9,70	0,23
**GREM1**	9,98	9,74	0,23
**NECAB1**	8,27	8,04	0,23
**ZNF117**	13,00	12,77	0,23
**SRPK2**	12,27	12,03	0,23
**CAMK1D**	12,04	11,81	0,23
**ZNF652**	11,04	10,81	0,23
**ZBTB6**	10,12	9,89	0,23
**CDC16**	12,02	11,79	0,23
**DNAJC1**	8,97	8,74	0,23
**DDR1**	10,82	10,59	0,23
**NQO2**	8,72	8,48	0,23
**HID1**	7,62	7,39	0,23
**ARG2**	9,06	8,83	0,23

**PML**	10,95	10,72	0,23
**SERHL2**	9,61	9,38	0,23
**SNX20**	8,79	8,56	0,23
**APH1B**	10,21	9,98	0,23
**CASTOR1**	7,17	6,94	0,23
**STX18**	11,37	11,14	0,23
**JRKL**	10,66	10,43	0,23
**AGGF1**	12,44	12,21	0,23
**NEK3**	6,94	6,70	0,23
**SLC30A1**	10,93	10,70	0,23
**C1orf100**	4,20	3,97	0,23
**OLFML1**	4,20	3,97	0,23
**CHM**	10,53	10,30	0,23
**IFNAR2**	11,10	10,87	0,23
**EYA4**	10,07	9,83	0,23
**USP33**	12,51	12,28	0,23
**RPS6KA3**	12,59	12,36	0,23
**C2CD4C**	4,37	4,13	0,23
**CEND1**	4,37	4,13	0,23
**NIM1K**	4,37	4,13	0,23
**EFCAB6**	5,83	5,60	0,23
**TADA1**	11,69	11,46	0,23
**OAZ3**	5,57	5,34	0,23
**MEGF11**	3,52	3,29	0,24
**UBXN2B**	12,41	12,17	0,24
**TTC8**	9,10	8,86	0,24
**MPZL1**	11,74	11,50	0,24
**PJA2**	12,95	12,71	0,24
**E2F5**	11,49	11,25	0,24
**POLR2J2**	9,42	9,18	0,24
**RASAL1**	10,53	10,29	0,24
**ZNF414**	9,79	9,55	0,24
**LNP1**	7,30	7,07	0,24
**MDM2**	11,17	10,93	0,24
**RBMS1**	11,29	11,06	0,24
**KLHL34**	4,58	4,34	0,24
**TRAPPC2**	11,26	11,02	0,24
**MINAR1**	9,12	8,88	0,24
**MAP2**	10,53	10,30	0,24
**TBC1D31**	12,03	11,79	0,24
**TUBA3E**	4,64	4,41	0,24
**RNASE6**	4,64	4,41	0,24
**CSNK1D**	10,42	10,19	0,24
**MAP3K1**	15,36	15,12	0,24
**COL5A1**	11,42	11,19	0,24
**SECISBP2L**	11,69	11,45	0,24
**ZNF433**	4,70	4,47	0,24
**IL1B**	9,76	9,52	0,24
**LMBRD2**	11,19	10,95	0,24
**PAN3**	13,69	13,45	0,24
**SIGLEC10**	9,98	9,74	0,24
**TPCN1**	6,83	6,59	0,24
**RAD1**	12,67	12,43	0,24
**C2orf50**	4,76	4,53	0,24
**IGLV1-51**	4,76	4,53	0,24
**CTNND2**	3,02	2,79	0,24

**KCNRG**	7,40	7,16	0,24
**MTPAP**	12,14	11,90	0,24
**PCNX2**	10,44	10,20	0,24
**USP45**	11,05	10,81	0,24
**BAG3**	10,40	10,16	0,24
**C1orf21**	11,39	11,15	0,24
**ABHD17C**	11,12	10,89	0,24
**SLC39A10**	11,72	11,48	0,24
**UQCRHL**	4,87	4,64	0,24
**C3orf38**	12,09	11,85	0,24
**LCOR**	12,72	12,48	0,24
**TNFSF13B**	5,86	5,62	0,24
**FAM184A**	6,68	6,44	0,24
**ETV7**	6,87	6,63	0,24
**ASTN1**	4,98	4,74	0,24
**PLRG1**	12,92	12,68	0,24
**TNF**	7,80	7,56	0,24
**HHIPL2**	5,03	4,79	0,24
**CYTL1**	5,03	4,79	0,24
**CHD1**	14,08	13,84	0,24
**OCIAD2**	6,84	6,60	0,24
**ZNF260**	12,69	12,45	0,24
**N4BP2**	13,13	12,89	0,24
**CPLANE1**	11,19	10,95	0,24
**UCP3**	8,40	8,16	0,24
**ZNF772**	10,37	10,13	0,24
**EPS15**	12,70	12,46	0,24
**KIF26A**	10,14	9,90	0,24
**ZNF776**	11,42	11,18	0,24
**TEKT5**	3,25	3,01	0,24
**ADGRB1**	5,25	5,01	0,24
**ECPAS**	13,67	13,43	0,24
**HAUS7**	7,92	7,68	0,24
**CCDC65**	6,20	5,96	0,24
**RHOBTB2**	8,52	8,28	0,24
**TTBK2**	11,81	11,57	0,24
**ARL6IP5**	11,82	11,58	0,24
**PDK3**	11,64	11,39	0,24
**EFCAB7**	10,09	9,85	0,24
**ZNF248**	10,99	10,75	0,24
**ABCG1**	10,01	9,77	0,24
**ATR**	12,56	12,32	0,24
**SORCS2**	8,90	8,65	0,24
**ANTXR1**	10,20	9,96	0,24
**RAB3GAP2**	13,11	12,87	0,24
**CHIC2**	9,83	9,59	0,24
**STUM**	5,74	5,49	0,24
**KLF4**	5,82	5,58	0,24
**GNG10**	11,22	10,98	0,24
**BCAP29**	10,40	10,16	0,24
**NFATC2**	10,85	10,61	0,24
**SLIT1**	5,98	5,74	0,24
**NFE2**	6,03	5,79	0,24
**ATG4B**	12,40	12,16	0,24
**RCAN1**	12,64	12,39	0,24
**MTMR2**	11,84	11,60	0,24

**HSPA1L**	6,17	5,92	0,24
**SFT2D2**	13,56	13,31	0,24
**TTC7B**	10,30	10,06	0,24
**TEX19**	6,23	5,99	0,24
**ACAP2**	13,34	13,09	0,24
**PBX4**	7,17	6,93	0,24
**LTA**	8,08	7,83	0,24
**ANKRA2**	9,49	9,24	0,24
**USP32**	11,71	11,46	0,24
**KLF2**	6,51	6,27	0,24
**KCNAB2**	12,19	11,95	0,24
**DPAGT1**	11,18	10,93	0,24
**DUSP10**	7,93	7,68	0,25
**FOXO1**	12,75	12,50	0,25
**AGBL2**	6,60	6,36	0,25
**ASPHD2**	9,90	9,65	0,25
**CDC42**	14,20	13,95	0,25
**STK17B**	11,85	11,60	0,25
**SLC19A2**	10,58	10,33	0,25
**TOM1L1**	5,61	5,37	0,25
**STARD10**	8,77	8,53	0,25
**TNFAIP8L2**	7,98	7,73	0,25
**ATM**	13,07	12,83	0,25
**FANCC**	10,92	10,68	0,25
**PIGC**	10,95	10,71	0,25
**PYGM**	6,59	6,34	0,25
**DNAJC27**	9,34	9,10	0,25
**CEP290**	11,62	11,37	0,25
**SNTB2**	4,16	3,91	0,25
**PEX13**	10,83	10,58	0,25
**PRICKLE1**	9,02	8,77	0,25
**ZNF506**	11,68	11,44	0,25
**FHIT**	7,41	7,16	0,25
**OGFRL1**	13,88	13,63	0,25
**PLEKHB2**	10,43	10,18	0,25
**PLCH1**	8,40	8,15	0,25
**RGS10**	10,00	9,75	0,25
**INTS12**	10,80	10,55	0,25
**MTHFR**	10,51	10,27	0,25
**NSUN3**	10,84	10,59	0,25
**PPP1R13B**	8,88	8,63	0,25
**ATP2C1**	12,88	12,63	0,25
**AVPR2**	5,75	5,50	0,25
**DCLRE1C**	12,28	12,03	0,25
**PPP2CB**	12,18	11,93	0,25
**DYNC2LI1**	9,51	9,26	0,25
**ATP10A**	9,42	9,17	0,25
**MIF4GD**	10,33	10,08	0,25
**LEPROT**	10,82	10,57	0,25
**ITGA5**	12,93	12,68	0,25
**OPALIN**	9,36	9,11	0,25
**FEM1B**	13,31	13,06	0,25
**HDAC11**	6,79	6,54	0,25
**GPR155**	9,95	9,70	0,25
**SCAPER**	10,19	9,94	0,25
**RNF8**	11,25	11,00	0,25

**TBC1D12**	8,68	8,43	0,25
**MEI1**	6,36	6,11	0,25
**MINDY2**	10,80	10,55	0,25
**DCHS2**	12,12	11,87	0,25
**SHC4**	2,06	1,81	0,25
**AMZ2**	10,97	10,72	0,25
**SIT1**	8,05	7,80	0,25
**ZNF669**	10,90	10,65	0,25
**SLC18B1**	10,15	9,90	0,25
**DIS3**	13,06	12,80	0,25
**KIAA0040**	11,99	11,73	0,25
**AGPAT5**	13,75	13,50	0,25
**BARD1**	12,57	12,32	0,25
**BORCS5**	9,34	9,09	0,25
**TMEM102**	9,38	9,13	0,25
**ATF3**	9,49	9,24	0,25
**DEPDC1**	12,45	12,19	0,25
**ZNF234**	10,79	10,53	0,25
**GPR65**	8,64	8,39	0,25
**TGFB3**	7,33	7,07	0,25
**DHTKD1**	11,97	11,72	0,26
**IL16**	10,60	10,35	0,26
**HCK**	7,12	6,87	0,26
**ZNF844**	9,92	9,66	0,26
**TMEM65**	11,67	11,41	0,26
**TSSK3**	7,30	7,05	0,26
**AZI2**	11,39	11,13	0,26
**MFN1**	11,68	11,42	0,26
**ITFG1**	11,78	11,53	0,26
**ZBTB17**	11,15	10,90	0,26
**IQCE**	11,22	10,97	0,26
**USP9X**	13,81	13,56	0,26
**ACP6**	9,93	9,68	0,26
**ATP6V1C1**	12,68	12,42	0,26
**CCDC42**	5,50	5,25	0,26
**GGPS1**	11,56	11,30	0,26
**FAM13B**	12,03	11,78	0,26
**LRRIQ3**	6,39	6,13	0,26
**VPS13B**	13,67	13,41	0,26
**NLRP3**	11,00	10,74	0,26
**CMTM8**	9,86	9,60	0,26
**AGFG2**	9,31	9,05	0,26
**IFT46**	10,48	10,22	0,26
**ETV4**	8,50	8,24	0,26
**AKR1C3**	6,84	6,58	0,26
**TBCCD1**	11,01	10,75	0,26
**TP53INP1**	12,82	12,56	0,26
**HNRNPLL**	9,57	9,31	0,26
**RXYLT1**	9,69	9,43	0,26
**CAMK2D**	13,95	13,69	0,26
**FXYD1**	3,38	3,12	0,26
**TCTN1**	8,01	7,75	0,26
**NF1**	12,09	11,83	0,26
**CD200**	7,68	7,42	0,26
**ALCAM**	11,05	10,79	0,26
**VPS50**	11,15	10,89	0,26

**LPXN**	10,31	10,05	0,26
**FKTN**	11,69	11,43	0,26
**COPS2**	13,09	12,83	0,26
**LGALSL**	7,77	7,51	0,26
**PCMTD1**	12,00	11,74	0,26
**PRSS36**	6,58	6,32	0,26
**GET1**	11,14	10,88	0,26
**ANKRD49**	11,28	11,02	0,26
**CUBN**	8,71	8,45	0,26
**ST8SIA4**	12,38	12,12	0,26
**ICAM5**	2,07	1,81	0,26
**PLAC8**	6,59	6,33	0,26
**ATRAID**	10,60	10,34	0,26
**CLMN**	10,58	10,32	0,26
**TCEANC2**	11,23	10,97	0,26
**CCL28**	9,97	9,71	0,26
**MLLT3**	6,40	6,14	0,26
**IL12RB2**	12,37	12,10	0,26
**SERF1A**	6,43	6,16	0,26
**SEC22C**	12,33	12,06	0,26
**POLR3B**	11,25	10,99	0,26
**COL28A1**	1,92	1,66	0,26
**PHKG1**	7,00	6,74	0,26
**PLEKHB1**	8,00	7,73	0,26
**MS4A1**	8,37	8,11	0,26
**SSX2IP**	11,14	10,88	0,26
**TMPPE**	7,64	7,38	0,26
**UAP1L1**	7,51	7,25	0,26
**ZCWPW1**	6,94	6,68	0,26
**CREG1**	10,89	10,62	0,26
**AC008581.2**	7,42	7,15	0,26
**ZNF192P1**	4,09	3,82	0,26
**RAD54L2**	12,22	11,95	0,26
**PLEKHF2**	7,38	7,12	0,26
**DNASE1L1**	9,40	9,13	0,27
**TMEM266**	5,42	5,15	0,27
**POT1**	11,62	11,36	0,27
**SPATA13**	11,83	11,56	0,27
**PCDH18**	10,55	10,29	0,27
**IDE**	13,05	12,78	0,27
**PAX8**	6,53	6,27	0,27
**BIRC3**	4,17	3,90	0,27
**DPP8**	12,75	12,49	0,27
**KIAA1586**	11,11	10,84	0,27
**ZNF155**	8,93	8,66	0,27
**USP13**	13,12	12,85	0,27
**AC022150.4**	11,03	10,76	0,27
**SPEF2**	7,42	7,16	0,27
**SRCIN1**	5,58	5,31	0,27
**MPRIP**	14,78	14,51	0,27
**KLRG2**	5,98	5,71	0,27
**NOTCH2NLA**	8,50	8,23	0,27
**GMPS**	14,35	14,08	0,27
**NPDC1**	8,34	8,07	0,27
**STOML1**	7,76	7,50	0,27
**PRICKLE2**	9,33	9,06	0,27

**TMEM38A**	8,16	7,89	0,27
**ZNF177**	9,59	9,32	0,27
**AC104452.1**	8,13	7,87	0,27
**ZNF501**	9,14	8,87	0,27
**RNF144B**	9,13	8,86	0,27
**CPQ**	9,04	8,77	0,27
**ASXL2**	10,95	10,68	0,27
**RAB39B**	9,28	9,01	0,27
**SLC25A38**	11,39	11,12	0,27
**CCN5**	2,46	2,19	0,27
**GPER1**	7,16	6,89	0,27
**NPPC**	6,97	6,70	0,27
**RMDN2**	7,41	7,14	0,27
**RAB5A**	11,43	11,16	0,27
**DNAJB4**	9,52	9,25	0,27
**SMCHD1**	13,86	13,59	0,27
**HMGN4**	12,85	12,58	0,27
**SMIM27**	7,92	7,65	0,27
**MTAP**	13,40	13,13	0,27
**RICTOR**	12,35	12,08	0,27
**CEP120**	11,76	11,49	0,27
**EXD3**	8,23	7,95	0,27
**ZNF614**	11,72	11,45	0,27
**PARD6B**	9,09	8,81	0,27
**TAS2R4**	7,49	7,22	0,27
**ABCA3**	9,64	9,36	0,27
**PLCB1**	11,05	10,77	0,27
**RLF**	11,91	11,64	0,27
**MTHFSD**	10,96	10,69	0,27
**C1GALT1**	11,75	11,48	0,27
**EDEM3**	10,88	10,60	0,27
**APOOL**	10,73	10,46	0,27
**ZNF562**	12,00	11,72	0,27
**WARS2**	10,47	10,19	0,27
**LGI2**	10,71	10,44	0,27
**TRIM2**	11,63	11,36	0,27
**ZADH2**	12,03	11,75	0,27
**TMEM192**	11,88	11,61	0,27
**ST8SIA2**	10,74	10,47	0,27
**PVR**	9,44	9,17	0,27
**ZNF440**	10,26	9,99	0,27
**FRYL**	13,09	12,81	0,27
**PDE4D**	10,41	10,14	0,27
**TYW3**	12,37	12,09	0,28
**REEP3**	11,64	11,37	0,28
**CAMLG**	11,38	11,11	0,28
**IGHV2-70D**	8,97	8,70	0,28
**SLC16A3**	9,04	8,76	0,28
**NPIPA7**	9,76	9,49	0,28
**ZNF91**	12,32	12,04	0,28
**CHN2**	9,40	9,13	0,28
**LRRC40**	12,16	11,89	0,28
**MYSM1**	12,88	12,60	0,28
**TXNDC9**	11,61	11,34	0,28
**CPT1C**	5,56	5,29	0,28
**DDIAS**	11,43	11,15	0,28

**AFF1**	11,44	11,16	0,28
**GNPDA1**	10,32	10,05	0,28
**ADAMTSL2**	7,41	7,14	0,28
**FOXO4**	9,01	8,73	0,28
**MOSMO**	10,41	10,14	0,28
**TMPRSS5**	4,66	4,38	0,28
**CDK19**	12,08	11,80	0,28
**CSTF2**	11,82	11,54	0,28
**METTL14**	11,71	11,43	0,28
**TMEM273**	7,40	7,12	0,28
**GZMK**	5,37	5,09	0,28
**SPATA33**	10,50	10,23	0,28
**WDFY3**	11,82	11,54	0,28
**CCL5**	7,64	7,36	0,28
**DMXL1**	11,29	11,01	0,28
**TLL2**	7,89	7,61	0,28
**BANK1**	8,14	7,86	0,28
**ARTN**	6,14	5,86	0,28
**APBB3**	7,44	7,16	0,28
**SOS2**	11,79	11,51	0,28
**CYP20A1**	11,22	10,94	0,28
**RBM14-RBM4**	9,02	8,74	0,28
**ZBTB2**	11,58	11,30	0,28
**KIAA1841**	10,69	10,41	0,28
**BBS2**	10,03	9,75	0,28
**FAM114A1**	2,59	2,31	0,28
**RAD54L**	12,34	12,06	0,28
**SMIM30**	10,53	10,25	0,28
**TEKT2**	7,21	6,93	0,28
**ZDHHC6**	11,24	10,96	0,28
**EPHX1**	9,71	9,43	0,28
**CYP3A5**	8,08	7,80	0,28
**SLC31A2**	5,25	4,97	0,28
**SERPING1**	7,36	7,08	0,28
**FBXO8**	9,57	9,29	0,28
**POC1B**	10,94	10,65	0,28
**FBXO45**	11,96	11,67	0,28
**RADX**	11,41	11,12	0,28
**ZNF680**	11,43	11,15	0,28
**RNFT2**	9,04	8,75	0,28
**CCNJL**	7,69	7,41	0,28
**TGFB1I1**	7,43	7,14	0,28
**PI16**	5,41	5,12	0,28
**PLEKHA6**	6,80	6,52	0,28
**SPRTN**	11,16	10,88	0,28
**ARHGEF9**	11,58	11,30	0,28
**PAIP1**	12,95	12,67	0,28
**MTTP**	3,13	2,85	0,28
**DOK2**	7,59	7,30	0,28
**TRMT1L**	11,91	11,62	0,28
**GAPDHS**	5,16	4,88	0,28
**DNAJC16**	11,21	10,92	0,28
**SMG1**	15,31	15,03	0,28
**TMEM204**	5,10	4,81	0,28
**GINM1**	10,67	10,39	0,28
**EPHA2**	6,27	5,99	0,29

**TOPORS**	11,93	11,64	0,29
**UNC5D**	9,83	9,54	0,29
**ZC2HC1A**	10,00	9,71	0,29
**ZNF823**	8,36	8,08	0,29
**RIN1**	7,33	7,05	0,29
**TLK2**	12,52	12,24	0,29
**PLAAT4**	8,05	7,77	0,29
**INPP5F**	11,42	11,14	0,29
**GPR182**	1,93	1,65	0,29
**IFI30**	9,82	9,53	0,29
**CUL5**	12,57	12,29	0,29
**ZNF227**	11,13	10,85	0,29
**SCYL3**	10,65	10,36	0,29
**HOPX**	6,16	5,87	0,29
**MYO7B**	7,83	7,54	0,29
**GPR89A**	11,26	10,97	0,29
**ZNF554**	8,85	8,56	0,29
**PTCH1**	12,45	12,16	0,29
**NEU4**	6,10	5,81	0,29
**CNTD1**	8,01	7,72	0,29
**NLN**	12,87	12,58	0,29
**SGK3**	11,08	10,79	0,29
**ADGRG1**	9,39	9,10	0,29
**NT5C3A**	11,24	10,95	0,29
**MSMO1**	11,73	11,44	0,29
**CMC4**	9,31	9,02	0,29
**ARMH4**	9,43	9,13	0,29
**NME7**	10,83	10,54	0,29
**ISM1**	7,13	6,84	0,29
**ARFGEF1**	14,32	14,03	0,29
**SHISA2**	9,35	9,05	0,29
**PLS3**	17,23	16,94	0,29
**DHRS1**	9,60	9,31	0,29
**CHEK2**	12,08	11,79	0,29
**IKBIP**	10,75	10,45	0,29
**SNAP23**	12,23	11,94	0,29
**PUS7L**	12,65	12,36	0,29
**ZNF547**	8,88	8,59	0,29
**TNS1**	10,84	10,54	0,29
**ATF6**	12,38	12,09	0,29
**L3MBTL4**	9,45	9,16	0,29
**AC233992.2**	6,00	5,71	0,29
**BORCS8-MEF2B**	8,52	8,23	0,29
**OTOA**	8,19	7,90	0,29
**MX1**	9,99	9,70	0,29
**BAIAP2L1**	9,25	8,96	0,29
**MATR3**	15,74	15,45	0,29
**SMIM10L2B**	4,82	4,53	0,29
**GPCPD1**	11,91	11,62	0,29
**NME5**	5,95	5,66	0,29
**CASP4**	9,76	9,47	0,29
**BBX**	12,96	12,66	0,29
**NT5C2**	13,39	13,09	0,29
**NTM**	2,96	2,66	0,30
**NANP**	11,47	11,17	0,30
**LGR4**	9,27	8,97	0,30

**TTC21B**	11,94	11,64	0,30
**ANKRD44**	8,23	7,93	0,30
**CCDC32**	10,37	10,08	0,30
**CALHM6**	7,17	6,87	0,30
**CEMIP**	5,05	4,76	0,30
**AD000671.2**	5,90	5,61	0,30
**OSR1**	5,90	5,61	0,30
**ASH2L**	13,18	12,88	0,30
**ZNF236**	11,42	11,13	0,30
**AFF4**	12,97	12,67	0,30
**KIF18A**	11,46	11,16	0,30
**CDC40**	12,36	12,06	0,30
**CAPSL**	8,54	8,25	0,30
**ARHGAP33**	10,30	10,00	0,30
**BCO2**	6,70	6,41	0,30
**CHRNA3**	7,26	6,96	0,30
**MYO5A**	11,61	11,32	0,30
**MCOLN1**	9,28	8,98	0,30
**ZNF610**	10,09	9,79	0,30
**C11orf54**	11,12	10,82	0,30
**ZNF385C**	4,70	4,41	0,30
**TIFA**	12,83	12,53	0,30
**UBR3**	12,11	11,81	0,30
**AGAP5**	8,70	8,40	0,30
**ROR1**	12,26	11,96	0,30
**LTB4R**	10,83	10,53	0,30
**P4HA1**	11,07	10,77	0,30
**XRN1**	12,45	12,14	0,30
**PRKD3**	12,10	11,80	0,30
**NSG1**	7,46	7,16	0,30
**CNNM2**	8,60	8,30	0,30
**MAP2K3**	12,80	12,50	0,30
**ARHGEF3**	6,84	6,54	0,30
**LDAH**	12,17	11,87	0,30
**ATRNL1**	10,89	10,59	0,30
**PHYHD1**	10,30	10,00	0,30
**ZC3H6**	9,91	9,61	0,30
**EPM2A**	10,32	10,02	0,30
**ATG9A**	10,88	10,58	0,30
**LAMB1**	7,37	7,06	0,30
**CCDC13**	4,58	4,27	0,30
**MOB4**	11,58	11,28	0,30
**PLXDC2**	10,31	10,01	0,30
**MRPL50**	11,77	11,47	0,30
**MNAT1**	10,76	10,45	0,30
**ATP23**	9,80	9,49	0,30
**APOBEC3D**	9,48	9,18	0,30
**MMD**	11,92	11,61	0,30
**DZANK1**	8,51	8,21	0,30
**ARHGAP5**	12,94	12,64	0,30
**ATP8B1**	7,46	7,15	0,30
**ZNF391**	8,86	8,55	0,30
**AC018630.2**	7,46	7,16	0,30
**ATXN3**	11,45	11,14	0,30
**LONRF3**	10,32	10,01	0,30
**ZFYVE9**	11,12	10,81	0,30

**TMTC3**	10,31	10,01	0,30
**ZNF267**	11,01	10,70	0,31
**EMP2**	11,39	11,09	0,31
**SCIMP**	5,56	5,26	0,31
**ASGR1**	7,49	7,19	0,31
**FSIP1**	5,64	5,34	0,31
**MFAP2**	7,31	7,01	0,31
**ZNF180**	9,90	9,60	0,31
**ACRV1**	6,21	5,90	0,31
**HS3ST3A1**	6,49	6,19	0,31
**PRTFDC1**	9,57	9,27	0,31
**FAT4**	6,94	6,64	0,31
**SGCE**	9,58	9,27	0,31
**IPO8**	12,58	12,27	0,31
**GPR137C**	9,19	8,88	0,31
**ZNF709**	9,79	9,48	0,31
**CYSTM1**	9,05	8,74	0,31
**CAPN7**	11,07	10,76	0,31
**ARL6**	8,32	8,02	0,31
**GBF1**	12,91	12,60	0,31
**SRPX**	5,55	5,24	0,31
**ANXA9**	6,66	6,35	0,31
**RAB31**	8,88	8,57	0,31
**TMSB15B**	9,99	9,68	0,31
**RERE**	13,41	13,10	0,31
**AL133353.2**	4,44	4,13	0,31
**SPATA5L1**	10,76	10,45	0,31
**OLAH**	5,44	5,13	0,31
**NDUFC2-KCTD14**	7,14	6,83	0,31
**SHLD3**	8,43	8,12	0,31
**TBC1D9**	8,59	8,28	0,31
**TLR2**	10,40	10,09	0,31
**SPIRE1**	7,23	6,92	0,31
**ASB11**	1,16	0,85	0,31
**RHBDL1**	5,55	5,24	0,31
**ZNF460**	8,08	7,77	0,31
**EMP3**	8,99	8,68	0,31
**S100A6**	6,92	6,61	0,31
**P2RY14**	8,87	8,55	0,31
**GRIP2**	4,37	4,05	0,31
**PCDHGB3**	6,06	5,74	0,31
**KIF1B**	11,49	11,18	0,31
**C4A**	8,05	7,74	0,31
**CHRDL1**	9,45	9,14	0,31
**FLACC1**	4,61	4,30	0,31
**BLOC1S2**	11,24	10,93	0,31
**RFTN2**	6,10	5,79	0,31
**SYPL1**	11,44	11,13	0,31
**CDRT4**	5,05	4,73	0,31
**UTRN**	8,92	8,61	0,31
**AP5M1**	12,42	12,10	0,31
**KCND1**	7,65	7,34	0,31
**FBXL13**	5,69	5,38	0,31
**BABAM2**	11,33	11,02	0,31
**BCKDHB**	10,41	10,09	0,32
**STXBP5**	10,39	10,08	0,32

**EPM2AIP1**	12,39	12,07	0,32
**POLR3F**	10,75	10,43	0,32
**SLC43A2**	8,90	8,58	0,32
**TRPM8**	4,29	3,97	0,32
**FBN1**	7,68	7,37	0,32
**BMT2**	9,80	9,48	0,32
**SLC49A4**	8,25	7,93	0,32
**IL32**	2,72	2,40	0,32
**PLEKHM3**	10,14	9,82	0,32
**MAGEL2**	8,23	7,92	0,32
**FBXL22**	7,27	6,96	0,32
**GLCCI1**	11,14	10,82	0,32
**NR4A3**	5,41	5,09	0,32
**AC016027.6**	7,30	6,99	0,32
**FKBP15**	10,60	10,29	0,32
**ZNF660**	8,14	7,82	0,32
**PEX5L**	6,79	6,48	0,32
**AL590764.2**	4,89	4,57	0,32
**VPS13A**	13,84	13,52	0,32
**XG**	1,95	1,63	0,32
**ZBTB48**	10,50	10,18	0,32
**NTAQ1**	10,40	10,08	0,32
**CAB39L**	9,86	9,54	0,32
**MBTPS2**	11,06	10,74	0,32
**TFDP2**	12,36	12,04	0,32
**GGT7**	4,20	3,88	0,32
**SCRT2**	4,20	3,88	0,32
**KNG1**	4,20	3,88	0,32
**ONECUT2**	7,91	7,59	0,32
**SPTA1**	8,85	8,53	0,32
**AC092718.7**	6,74	6,42	0,32
**DYDC1**	4,06	3,74	0,32
**NLRP6**	5,33	5,01	0,32
**AGTRAP**	8,94	8,62	0,32
**BMPER**	4,13	3,81	0,32
**SGMS1**	9,77	9,44	0,32
**NMT2**	8,61	8,28	0,32
**CDC42BPA**	12,22	11,89	0,32
**UPRT**	10,63	10,31	0,32
**PFDN1**	11,99	11,67	0,32
**GADL1**	5,29	4,97	0,32
**KMT2A**	13,05	12,73	0,32
**FAXDC2**	6,04	5,72	0,32
**ZNF320**	12,16	11,83	0,32
**PROSER3**	10,29	9,96	0,32
**PIK3IP1**	7,49	7,16	0,32
**SRSF8**	12,67	12,34	0,32
**ZNF724**	10,68	10,36	0,32
**ADGRF3**	7,21	6,88	0,32
**EVI5**	9,51	9,18	0,32
**ATP6V1A**	11,94	11,61	0,32
**SLC35G5**	4,12	3,79	0,32
**H2BC17**	4,12	3,79	0,32
**RASD2**	5,25	4,92	0,33
**RRAD**	5,25	4,92	0,33
**USP43**	9,42	9,10	0,33

**NOS1AP**	3,04	2,71	0,33
**ORMDL1**	12,01	11,68	0,33
**ZNF532**	8,31	7,98	0,33
**PIP4K2C**	9,11	8,78	0,33
**RHOQ**	11,00	10,67	0,33
**LYST**	10,43	10,10	0,33
**RNF150**	8,73	8,40	0,33
**CNTNAP3**	5,15	4,82	0,33
**SCUBE3**	6,58	6,25	0,33
**TRAPPC11**	12,29	11,96	0,33
**SHPRH**	12,38	12,05	0,33
**S100A16**	5,50	5,18	0,33
**RETSAT**	10,70	10,37	0,33
**COL22A1**	4,02	3,69	0,33
**CPT2**	10,87	10,54	0,33
**LTN1**	12,38	12,05	0,33
**EXTL3**	13,09	12,76	0,33
**PIGH**	8,83	8,50	0,33
**PTCD2**	11,18	10,85	0,33
**TMSB15B**	10,19	9,86	0,33
**ZNF695**	10,53	10,19	0,33
**RASGRP1**	10,23	9,90	0,33
**SERTAD3**	8,75	8,42	0,33
**YIPF5**	11,42	11,09	0,33
**UBAP1L**	6,13	5,80	0,33
**RASL11B**	6,53	6,20	0,33
**COL6A2**	7,14	6,81	0,33
**AC092329.3**	8,49	8,16	0,33
**DYM**	12,50	12,16	0,33
**ZNF571**	8,88	8,55	0,33
**ZNF570**	10,36	10,03	0,33
**ZNF184**	11,23	10,90	0,33
**ZNF699**	9,48	9,15	0,33
**USP28**	11,76	11,43	0,33
**PABPN1**	12,97	12,63	0,33
**F2R**	10,41	10,08	0,33
**ARHGAP12**	10,71	10,37	0,33
**CA13**	7,52	7,18	0,33
**KIF13A**	11,47	11,14	0,33
**MUC1**	6,74	6,40	0,33
**TRMT5**	9,28	8,95	0,33
**CYSLTR1**	6,14	5,81	0,34
**SPEG**	8,74	8,41	0,34
**MTMR4**	13,46	13,12	0,34
**ZNF454**	5,07	4,74	0,34
**LSMEM2**	5,07	4,74	0,34
**KRBA2**	10,69	10,36	0,34
**FBXO39**	6,51	6,17	0,34
**KATNAL2**	4,63	4,29	0,34
**PECAM1**	9,95	9,62	0,34
**ARHGEF28**	6,99	6,66	0,34
**SLC16A11**	4,63	4,29	0,34
**FCGR2A**	7,54	7,21	0,34
**AC139491.7**	3,92	3,59	0,34
**SERPINE1**	3,92	3,59	0,34
**LHFPL1**	3,92	3,59	0,34

**U2AF1L4**	7,41	7,08	0,34
**RPGRIP1L**	11,12	10,79	0,34
**MYRF**	7,43	7,09	0,34
**CEP44**	11,19	10,86	0,34
**SERPINB6**	7,67	7,33	0,34
**PLD5P1**	7,15	6,81	0,34
**P2RY1**	10,73	10,39	0,34
**PDZD4**	2,56	2,22	0,34
**PRCP**	10,69	10,35	0,34
**MCMDC2**	7,86	7,52	0,34
**TECPR2**	9,76	9,43	0,34
**SLF1**	11,57	11,23	0,34
**MAGEE1**	9,15	8,82	0,34
**CTSO**	10,32	9,98	0,34
**LIPI**	5,03	4,69	0,34
**KEL**	5,03	4,69	0,34
**KLHL23**	12,76	12,42	0,34
**RTEL1-TNFRSF6B**	7,12	6,78	0,34
**DST**	11,83	11,49	0,34
**MOSPD2**	10,20	9,86	0,34
**BMERB1**	5,64	5,31	0,34
**TTC17**	12,82	12,48	0,34
**METTL8**	9,11	8,77	0,34
**GPC3**	3,30	2,96	0,34
**OXTR**	7,80	7,46	0,34
**NPIPB6**	7,73	7,39	0,34
**FBXL4**	11,45	11,11	0,34
**TCP11L1**	10,05	9,71	0,34
**TAS2R19**	5,61	5,27	0,34
**ETV1**	8,14	7,80	0,34
**SRGN**	10,75	10,41	0,34
**OSGEPL1**	9,78	9,44	0,34
**METTL2B**	12,48	12,14	0,34
**UBE2W**	11,09	10,75	0,34
**RAG1**	15,15	14,81	0,34
**ELF3**	5,30	4,96	0,34
**LILRA6**	5,21	4,87	0,34
**OCLN**	8,91	8,57	0,34
**CDS1**	3,82	3,47	0,34
**CYB5B**	10,73	10,39	0,34
**FAM161A**	10,17	9,83	0,34
**HERC6**	9,42	9,08	0,34
**RNF175**	10,91	10,57	0,34
**ANK2**	9,70	9,36	0,34
**ZNF107**	11,98	11,64	0,34
**SERPINF1**	9,78	9,44	0,34
**AC087632.2**	8,17	7,82	0,34
**AK7**	5,59	5,25	0,34
**SYNJ2**	10,34	10,00	0,34
**TUNAR**	8,73	8,38	0,35
**KIF27**	10,76	10,41	0,35
**PDIA3**	10,90	10,55	0,35
**GRID2IP**	10,81	10,46	0,35
**ADA2**	6,28	5,94	0,35
**AC001226.2**	6,01	5,67	0,35
**FZD6**	7,49	7,14	0,35

**FASTKD3**	11,06	10,71	0,35
**SLIT2**	8,14	7,79	0,35
**TFEC**	4,98	4,63	0,35
**BBS9**	8,89	8,54	0,35
**C19orf38**	4,18	3,83	0,35
**FAM166B**	2,34	1,99	0,35
**GTF2H2C**	12,75	12,40	0,35
**AC009779.3**	8,40	8,05	0,35
**FSIP2**	6,30	5,95	0,35
**PEX3**	9,99	9,64	0,35
**CRPPA**	7,66	7,31	0,35
**SESN1**	9,52	9,17	0,35
**SRD5A2**	3,70	3,35	0,35
**CYB5D2**	8,14	7,79	0,35
**TIGD3**	7,75	7,40	0,35
**FAM13A**	8,29	7,94	0,35
**ITGB1BP2**	6,80	6,45	0,35
**FAM185A**	7,15	6,80	0,35
**TMEM198**	5,73	5,38	0,35
**STPG1**	6,31	5,95	0,35
**TPH1**	6,72	6,37	0,35
**ORC4**	11,45	11,10	0,35
**KRTCAP3**	5,34	4,99	0,35
**NBPF15**	8,69	8,33	0,35
**SLC10A7**	10,24	9,88	0,35
**STEAP1B**	7,67	7,32	0,35
**ABHD3**	11,72	11,36	0,35
**NDST2**	10,49	10,14	0,36
**ZSCAN10**	2,12	1,76	0,36
**GSAP**	8,16	7,80	0,36
**CDRT15**	5,50	5,14	0,36
**TMEM220**	9,99	9,64	0,36
**MTFR2**	10,85	10,49	0,36
**PRR11**	13,47	13,12	0,36
**TTC23**	5,63	5,27	0,36
**IL4R**	9,75	9,39	0,36
**TRIB2**	12,57	12,21	0,36
**ITGA2**	7,55	7,20	0,36
**PYGL**	5,37	5,01	0,36
**HLTF**	13,45	13,09	0,36
**ZNF396**	3,69	3,33	0,36
**GEMIN7**	10,70	10,34	0,36
**PROCR**	7,29	6,93	0,36
**R3HDM1**	13,42	13,06	0,36
**AGAP3**	8,66	8,30	0,36
**EPB41L5**	10,72	10,36	0,36
**RNF19A**	11,96	11,60	0,36
**CSF1**	6,97	6,61	0,36
**SNX33**	9,15	8,79	0,36
**EXTL2**	10,73	10,37	0,36
**PEX7**	8,11	7,75	0,36
**ANKRD18A**	6,12	5,76	0,36
**HSF4**	7,48	7,12	0,36
**ZHX1-C8orf76**	8,57	8,21	0,36
**TAS2R13**	3,57	3,21	0,36
**MTA3**	11,29	10,93	0,36

**SF3B3**	15,47	15,11	0,36
**FKBP1C**	4,70	4,34	0,36
**GIN1**	8,89	8,52	0,36
**ZNF181**	10,62	10,26	0,36
**PCDH9**	13,06	12,70	0,36
**IL1R1**	3,17	2,81	0,36
**VPS33A**	11,81	11,45	0,36
**NCEH1**	8,80	8,44	0,36
**ZNF653**	8,63	8,27	0,36
**MYL4**	8,48	8,11	0,36
**GABBR2**	7,24	6,88	0,36
**ZNF132**	7,10	6,74	0,36
**DYRK4**	7,50	7,14	0,36
**DNAJC28**	4,90	4,54	0,36
**BAALC**	6,14	5,78	0,37
**FAM13C**	2,99	2,63	0,37
**AC022384.1**	9,78	9,41	0,37
**SYT4**	7,94	7,57	0,37
**XK**	8,23	7,86	0,37
**CEP162**	10,22	9,85	0,37
**HES7**	4,80	4,43	0,37
**RTL8C**	7,75	7,38	0,37
**AATK**	3,99	3,62	0,37
**AGT**	7,20	6,83	0,37
**CRACR2B**	4,23	3,87	0,37
**CYB561**	8,44	8,07	0,37
**SCN3A**	6,78	6,41	0,37
**TNFRSF19**	7,31	6,94	0,37
**ARHGAP11B**	9,42	9,05	0,37
**CCDC200**	5,54	5,17	0,37
**BIVM-ERCC5**	5,75	5,38	0,37
**ZC3H12B**	9,64	9,27	0,37
**STARD13**	8,12	7,75	0,37
**ZBED8**	8,81	8,44	0,37
**TOP2B**	17,99	17,62	0,37
**MYLK3**	6,00	5,63	0,37
**ZNF850**	11,07	10,70	0,37
**TTC39B**	6,50	6,13	0,37
**SPDYE3**	7,52	7,14	0,37
**KLHDC7A**	6,69	6,32	0,37
**CXCR6**	4,43	4,06	0,37
**GLIS3**	5,98	5,61	0,37
**EPHA6**	2,35	1,98	0,37
**TBCE**	11,14	10,77	0,37
**MATN1**	3,43	3,06	0,37
**MANEA**	11,86	11,49	0,37
**EPHB2**	5,42	5,04	0,37
**SYTL4**	4,14	3,77	0,37
**ANAPC10**	9,64	9,26	0,37
**RNF182**	6,48	6,11	0,37
**IL13RA1**	7,88	7,50	0,37
**MEF2B**	6,59	6,21	0,37
**SAMD9L**	9,79	9,41	0,38
**IGKC**	8,25	7,88	0,38
**MYO9A**	12,09	11,72	0,38
**ARHGAP44**	9,32	8,95	0,38

**TNFSF4**	9,25	8,87	0,38
**DRP2**	8,38	8,01	0,38
**CPEB3**	8,38	8,00	0,38
**SCAI**	11,65	11,27	0,38
**TXNDC16**	11,16	10,78	0,38
**PRKCH**	11,65	11,27	0,38
**SLC25A40**	12,00	11,61	0,38
**CPNE5**	6,27	5,88	0,38
**AL357673.1**	4,51	4,13	0,38
**QRFP**	4,06	3,68	0,38
**IGHG3**	6,56	6,18	0,38
**FUT2**	7,17	6,78	0,38
**NEDD8-MDP1**	2,75	2,37	0,38
**RAPGEF2**	11,72	11,34	0,38
**C1orf54**	8,45	8,07	0,38
**ZMYM1**	10,36	9,98	0,38
**PRIMPOL**	10,10	9,72	0,38
**PGA5**	5,60	5,21	0,38
**TNS2**	6,17	5,78	0,39
**PTAFR**	5,51	5,13	0,39
**ERP44**	11,18	10,80	0,39
**AHCYL2**	10,39	10,00	0,39
**COQ6**	8,65	8,26	0,39
**NLGN1**	10,69	10,31	0,39
**PAN2**	10,15	9,76	0,39
**KLF3**	9,27	8,88	0,39
**HDHD3**	9,62	9,23	0,39
**FANCB**	10,03	9,64	0,39
**TBC1D10C**	8,42	8,04	0,39
**HEG1**	11,00	10,61	0,39
**PRSS51**	7,51	7,12	0,39
**LEPR**	8,30	7,91	0,39
**PLEKHH1**	7,69	7,30	0,39
**TMEM120A**	8,38	7,99	0,39
**TNRC18**	9,87	9,48	0,39
**DNAAF4**	4,97	4,58	0,39
**SUSD2**	5,03	4,64	0,39
**LILRB2**	8,52	8,13	0,39
**PHYH**	11,09	10,70	0,39
**LIMA1**	10,51	10,12	0,39
**PIK3C3**	12,08	11,69	0,39
**SLC9A3R1**	9,34	8,95	0,39
**PPP3CA**	12,34	11,94	0,39
**FAM149B1**	10,36	9,97	0,39
**PSTPIP1**	8,10	7,70	0,39
**EVA1A**	5,44	5,05	0,39
**RYK**	7,88	7,49	0,39
**HSH2D**	9,25	8,86	0,39
**EFHC1**	10,73	10,33	0,39
**TMEM132B**	4,37	3,97	0,39
**SNX10**	8,74	8,35	0,39
**RMND1**	10,35	9,96	0,39
**NUBPL**	9,72	9,32	0,39
**ASTN2**	7,26	6,87	0,39
**EEPD1**	8,53	8,14	0,39
**CFAP52**	4,33	3,93	0,39

**DIAPH2**	11,41	11,02	0,39
**TAF1B**	10,80	10,41	0,40
**CCDC3**	4,95	4,55	0,40
**CDC37L1**	10,11	9,72	0,40
**SLC1A3**	4,56	4,16	0,40
**TPTE2**	2,76	2,37	0,40
**ADAM32**	4,98	4,58	0,40
**TRDV2**	4,98	4,58	0,40
**SIAE**	9,12	8,72	0,40
**DNMT3L**	1,48	1,08	0,40
**CLYBL**	7,50	7,11	0,40
**AC079594.1**	5,41	5,01	0,40
**DNAH6**	7,62	7,23	0,40
**ZDHHC11B**	6,96	6,56	0,40
**SPG11**	11,69	11,29	0,40
**NAA30**	11,94	11,55	0,40
**RBMS3**	7,28	6,88	0,40
**CLEC2D**	13,45	13,05	0,40
**CORO6**	9,08	8,68	0,40
**B3GALNT1**	9,16	8,76	0,40
**PTPRB**	7,02	6,61	0,40
**POC5**	10,81	10,41	0,40
**MYO6**	6,88	6,48	0,40
**TMEM81**	7,80	7,40	0,40
**CNTN2**	8,40	8,00	0,40
**ZNF891**	11,41	11,00	0,40
**PATL2**	3,55	3,15	0,40
**KIF9**	9,44	9,03	0,40
**SCN11A**	5,97	5,57	0,40
**SENP7**	10,86	10,46	0,40
**C3orf70**	7,84	7,44	0,41
**MDH1B**	6,84	6,44	0,41
**SPSB4**	6,53	6,13	0,41
**TBC1D2**	7,20	6,79	0,41
**NAT8**	3,11	2,70	0,41
**AL139142.2**	3,11	2,70	0,41
**DRC7**	4,74	4,34	0,41
**UFSP2**	10,07	9,66	0,41
**VPS8**	11,41	11,00	0,41
**THBS1**	4,87	4,47	0,41
**ZNF763**	8,70	8,29	0,41
**STAC**	6,37	5,96	0,41
**MAG**	5,37	4,96	0,41
**PPAN-P2RY11**	9,84	9,43	0,41
**SORBS2**	7,92	7,51	0,41
**ZNF154**	6,12	5,71	0,41
**NFASC**	4,37	3,96	0,41
**CACNB2**	8,25	7,84	0,41
**B3GNTL1**	7,87	7,46	0,41
**FZD7**	7,50	7,09	0,41
**NKG7**	8,71	8,30	0,41
**PPP1R13L**	9,09	8,68	0,41
**AC010547.4**	6,17	5,76	0,41
**AMACR**	9,67	9,26	0,41
**CACNG5**	4,20	3,79	0,41
**GRIN2C**	4,20	3,79	0,41

**RGS9BP**	4,20	3,79	0,41
**IPCEF1**	7,89	7,48	0,41
**PRKAA2**	9,27	8,86	0,41
**SHBG**	1,80	1,39	0,41
**ZRANB3**	10,48	10,07	0,41
**USP31**	7,36	6,94	0,41
**MPDU1**	11,94	11,52	0,42
**LRRC38**	6,99	6,58	0,42
**NCKAP5**	9,88	9,46	0,42
**ZNF713**	9,49	9,07	0,42
**FSCN2**	4,94	4,52	0,42
**ZNF559-ZNF177**	7,99	7,57	0,42
**ULBP3**	7,52	7,10	0,42
**ATP11A**	12,69	12,27	0,42
**ZNF292**	12,49	12,07	0,42
**ACVR1**	8,89	8,47	0,42
**LPP**	12,09	11,67	0,42
**COL4A2**	5,67	5,25	0,42
**SENP8**	7,63	7,21	0,42
**AC011499.1**	4,76	4,34	0,42
**MAP9**	8,94	8,51	0,42
**ALB**	5,84	5,42	0,42
**SCN5A**	9,37	8,95	0,42
**AC010132.3**	10,71	10,29	0,42
**ZNF519**	11,41	10,98	0,42
**SLC39A2**	3,37	2,95	0,42
**ITPRIPL2**	9,07	8,64	0,42
**ARHGAP32**	11,56	11,14	0,42
**C5orf34**	10,28	9,86	0,42
**ICA1**	6,66	6,24	0,43
**PLA2G12A**	10,82	10,39	0,43
**RBAK-RBAKDN**	6,08	5,65	0,43
**ZNF555**	10,15	9,72	0,43
**MORN1**	6,85	6,42	0,43
**PTGES3L**	6,75	6,33	0,43
**ST3GAL5**	7,66	7,23	0,43
**MAP3K15**	6,89	6,46	0,43
**PHLDA1**	6,65	6,22	0,43
**AOC2**	7,90	7,47	0,43
**WIPI1**	7,12	6,69	0,43
**ZNF596**	7,76	7,33	0,43
**ADPRM**	9,59	9,16	0,43
**PPP1R2**	11,32	10,88	0,43
**MYL5**	8,13	7,70	0,43
**PDE6C**	2,91	2,48	0,43
**CCN6**	2,91	2,48	0,43
**FUT5**	2,91	2,48	0,43
**CSPG5**	2,91	2,48	0,43
**FNDC7**	2,91	2,48	0,43
**CCDC126**	8,98	8,55	0,43
**HGF**	10,06	9,63	0,43
**IQCG**	8,26	7,82	0,43
**ZNF28**	11,76	11,32	0,43
**DIP2C**	11,69	11,26	0,43
**MROH8**	6,89	6,46	0,43
**DDX19B**	11,40	10,97	0,43

**DPEP2**	4,64	4,20	0,43
**IKZF2**	10,61	10,17	0,44
**C3orf18**	7,20	6,76	0,44
**POU6F2**	4,02	3,59	0,44
**MAFA**	4,02	3,59	0,44
**CFAP36**	10,31	9,88	0,44
**ESRRB**	2,78	2,35	0,44
**BHLHB9**	6,58	6,14	0,44
**SMUG1**	9,00	8,56	0,44
**GALNS**	10,18	9,74	0,44
**CCN2**	7,03	6,59	0,44
**CCDC125**	10,71	10,27	0,44
**CDS2**	12,05	11,61	0,44
**CACNA2D1**	4,46	4,02	0,44
**FN3K**	5,41	4,97	0,44
**ME3**	11,10	10,66	0,44
**CROT**	9,55	9,11	0,44
**RO60**	12,70	12,26	0,44
**MYO1E**	7,36	6,92	0,44
**SPRED1**	12,05	11,61	0,44
**PALLD**	7,87	7,43	0,44
**CCDC168**	7,19	6,75	0,44
**RNF170**	11,04	10,60	0,44
**MBD5**	10,10	9,66	0,44
**FGD5**	4,83	4,39	0,44
**LY6H**	6,01	5,57	0,44
**FAM114A2**	10,75	10,31	0,44
**RAB40B**	8,05	7,61	0,44
**ZNF615**	10,33	9,89	0,44
**STBD1**	6,66	6,22	0,44
**DLL4**	5,37	4,92	0,44
**POLI**	10,08	9,63	0,44
**GRK3**	11,46	11,02	0,44
**OPRL1**	7,03	6,59	0,44
**NPHP1**	7,85	7,41	0,44
**RASA2**	10,53	10,09	0,45
**HSD17B14**	7,10	6,65	0,45
**SNED1**	8,48	8,04	0,45
**DNAAF3**	7,25	6,80	0,45
**RSPH3**	9,26	8,81	0,45
**CCDC146**	8,09	7,65	0,45
**SLC6A6**	11,11	10,66	0,45
**ENDOV**	7,88	7,43	0,45
**VDAC2**	10,96	10,51	0,45
**HAL**	4,53	4,08	0,45
**SLC44A5**	6,94	6,49	0,45
**AC231657.3**	3,92	3,47	0,45
**MMP28**	3,93	3,48	0,45
**CDNF**	5,66	5,21	0,45
**SEMA5A**	7,50	7,05	0,45
**THNSL2**	5,52	5,07	0,45
**WDR31**	9,10	8,65	0,45
**SPEM2**	1,82	1,37	0,45
**TMEM150C**	6,61	6,15	0,45
**MCF2L2**	6,08	5,63	0,45
**EPHA7**	6,42	5,97	0,45

**CCDC114**	6,27	5,81	0,45
**USF3**	11,52	11,06	0,46
**MIOX**	4,59	4,13	0,46
**RBPMS**	6,95	6,49	0,46
**C1orf56**	7,46	7,01	0,46
**IFFO2**	10,28	9,82	0,46
**DENND2C**	5,11	4,65	0,46
**NOTCH3**	13,03	12,57	0,46
**KCNG3**	4,35	3,89	0,46
**PLD5**	3,55	3,09	0,46
**GAL3ST2**	4,93	4,47	0,46
**PITPNM3**	6,52	6,06	0,46
**ADAM8**	5,67	5,21	0,46
**TMCC3**	7,88	7,42	0,46
**PLVAP**	6,66	6,20	0,46
**SLC35D2**	5,53	5,07	0,46
**CFP**	4,55	4,08	0,46
**SNX25**	11,49	11,02	0,46
**LCLAT1**	11,51	11,05	0,46
**AP003419.1**	8,85	8,39	0,46
**CRABP2**	6,75	6,28	0,46
**CTSF**	1,83	1,36	0,46
**SEC22A**	10,41	9,94	0,46
**AC008770.2**	5,52	5,05	0,46
**PTPRM**	7,50	7,04	0,46
**ANKS1B**	12,43	11,96	0,46
**ITGB5**	2,69	2,22	0,46
**TMEM74B**	2,69	2,22	0,46
**DZIP1**	2,69	2,22	0,46
**NAT16**	2,69	2,22	0,46
**TEDDM1**	2,69	2,22	0,46
**ONECUT3**	2,69	2,22	0,46
**IGLV1-50**	2,69	2,22	0,46
**GNG14**	2,69	2,22	0,46
**AC020907.6**	2,69	2,22	0,46
**FBXO25**	11,19	10,72	0,47
**MAP4K3**	10,95	10,48	0,47
**ZNF17**	9,66	9,19	0,47
**AFAP1L2**	8,59	8,12	0,47
**ITGA6**	8,94	8,48	0,47
**DBNDD1**	6,31	5,85	0,47
**TMEM150B**	3,17	2,70	0,47
**ERCC8**	10,60	10,13	0,47
**IGLV5-52**	7,84	7,38	0,47
**CHAT**	3,82	3,35	0,47
**NANOGP8**	3,82	3,35	0,47
**H4C12**	3,82	3,35	0,47
**AC025287.4**	3,82	3,35	0,47
**PGBD5**	6,50	6,03	0,47
**CALB1**	3,27	2,81	0,47
**PROS1**	8,00	7,53	0,47
**CD86**	4,87	4,41	0,47
**BEST4**	4,87	4,41	0,47
**PPIC**	4,87	4,41	0,47
**LAMP5**	5,48	5,01	0,47
**TTC34**	6,08	5,61	0,47

**NAALADL1**	3,48	3,01	0,47
**GPR160**	11,24	10,76	0,47
**JAKMIP3**	4,79	4,32	0,47
**LYN**	12,15	11,67	0,47
**RIBC1**	6,35	5,87	0,47
**AL929554.1**	7,07	6,59	0,47
**ANKRD20A3P**	3,76	3,29	0,47
**PRAME**	3,92	3,45	0,47
**ARMCX1**	5,68	5,20	0,47
**TAGAP**	8,38	7,90	0,47
**TBXA2R**	3,58	3,11	0,47
**LIFR**	8,28	7,81	0,47
**PRORP**	12,07	11,60	0,47
**AGA**	9,82	9,34	0,48
**KIF26B**	5,08	4,61	0,48
**PALM2AKAP2**	11,41	10,93	0,48
**ARHGEF5**	7,40	6,92	0,48
**CR589904.2**	5,16	4,69	0,48
**HBP1**	10,24	9,76	0,48
**CFLAR**	11,67	11,20	0,48
**KANTR**	5,85	5,37	0,48
**RASGEF1A**	5,52	5,04	0,48
**VAMP4**	11,45	10,97	0,48
**FBXL20**	10,45	9,97	0,48
**HAPLN3**	6,07	5,59	0,48
**ADTRP**	6,40	5,92	0,48
**NHSL1**	10,07	9,59	0,48
**PARP9**	9,92	9,44	0,48
**CAPRIN2**	10,83	10,35	0,48
**RBFOX1**	2,64	2,15	0,48
**RBFOX2**	10,64	10,16	0,48
**RIMKLA**	8,11	7,63	0,48
**FRMD4A**	9,25	8,77	0,48
**IFT43**	8,11	7,62	0,48
**COG1**	10,81	10,33	0,48
**MSANTD3-TMEFF1**	10,70	10,22	0,48
**ATP6V0A4**	1,84	1,35	0,48
**METTL4**	10,61	10,12	0,48
**BTBD8**	7,67	7,19	0,48
**ZNF230**	9,73	9,24	0,48
**F8**	8,32	7,84	0,48
**BIN2**	5,61	5,13	0,49
**TBC1D16**	10,01	9,53	0,49
**TCP10L**	4,53	4,05	0,49
**LY6G5C**	5,20	4,71	0,49
**BCAR1**	6,73	6,25	0,49
**RFX7**	10,83	10,34	0,49
**TMEM71**	6,38	5,89	0,49
**AL513165.2**	3,70	3,21	0,49
**NEB**	6,67	6,18	0,49
**ALDH3A1**	4,76	4,27	0,49
**INPP5A**	9,43	8,94	0,49
**CNRIP1**	4,06	3,56	0,49
**ULBP1**	6,10	5,61	0,49
**RAB3B**	8,32	7,83	0,49
**TLN2**	7,73	7,24	0,49

**ZNF418**	9,58	9,09	0,49
**TIGAR**	11,27	10,77	0,49
**PRSS16**	5,57	5,07	0,49
**FOXP1**	9,94	9,45	0,50
**TPO**	6,60	6,10	0,50
**DHRS11**	9,68	9,18	0,50
**TRPC1**	8,78	8,29	0,50
**H2AJ**	7,76	7,26	0,50
**GABARAPL1**	7,30	6,81	0,50
**GOLGA8O**	4,99	4,49	0,50
**AHNAK2**	9,92	9,42	0,50
**TJP3**	3,81	3,32	0,50
**SPAG1**	7,21	6,72	0,50
**CYP2W1**	5,87	5,37	0,50
**KCNN3**	5,74	5,24	0,50
**BLACAT1**	5,74	5,24	0,50
**RCOR3**	12,20	11,70	0,50
**SLC14A1**	4,06	3,56	0,50
**PIP5KL1**	5,03	4,53	0,50
**TCF7L1**	5,03	4,53	0,50
**SERPINI1**	7,68	7,18	0,50
**ZNF285**	9,52	9,01	0,50
**AL451136.1**	6,03	5,52	0,51
**ZNF23**	9,93	9,42	0,51
**GOLT1B**	11,65	11,14	0,51
**B3GNT4**	6,17	5,66	0,51
**BCAS3**	8,69	8,18	0,51
**SYNJ2BP-COX16**	9,98	9,47	0,51
**STON1**	9,74	9,23	0,51
**ASAP3**	8,34	7,83	0,51
**ZNF853**	6,00	5,49	0,51
**PLEKHF1**	5,68	5,16	0,51
**HSPB7**	4,64	4,13	0,51
**RUFY4**	4,20	3,69	0,51
**TNFRSF11A**	8,00	7,49	0,51
**CEACAM6**	2,91	2,40	0,51
**DDX25**	3,57	3,06	0,51
**BHLHE23**	3,57	3,06	0,51
**LINGO4**	3,57	3,06	0,51
**CD244**	3,57	3,06	0,51
**HRK**	3,57	3,06	0,51
**SMIM5**	7,51	7,00	0,51
**LMBRD1**	10,25	9,74	0,51
**CACNA1B**	4,11	3,59	0,51
**ADAMTS16**	2,42	1,91	0,51
**PGLYRP1**	2,42	1,91	0,51
**AKNAD1**	2,42	1,91	0,51
**HCAR3**	2,42	1,91	0,51
**HSPB9**	2,42	1,91	0,51
**CCER2**	2,42	1,91	0,51
**IGKV1D-8**	2,42	1,91	0,51
**RND3**	8,54	8,03	0,51
**CCDC157**	5,98	5,46	0,52
**CTBS**	9,35	8,83	0,52
**EFNA2**	5,64	5,13	0,52
**CPED1**	7,75	7,23	0,52

**KRTAP5-9**	5,44	4,92	0,52
**C1orf162**	5,39	4,87	0,52
**TCN2**	7,45	6,93	0,52
**XKR5**	4,93	4,41	0,52
**IL10RA**	2,59	2,07	0,52
**AL713999.1**	6,72	6,20	0,52
**CYLD**	10,59	10,07	0,52
**IGFALS**	3,51	2,99	0,52
**AP006621.6**	6,21	5,69	0,52
**ELOVL7**	7,70	7,18	0,52
**ABAT**	9,99	9,46	0,52
**TIE1**	6,43	5,90	0,53
**APOE**	6,26	5,74	0,53
**CYP1A1**	4,22	3,69	0,53
**SOX18**	7,52	7,00	0,53
**IQCJ-SCHIP1**	9,15	8,62	0,53
**MTRNR2L8**	5,16	4,64	0,53
**MSR1**	3,40	2,87	0,53
**AQP1**	6,79	6,26	0,53
**PNMA8B**	8,45	7,92	0,53
**SRGAP1**	6,49	5,96	0,53
**ZNF492**	9,35	8,82	0,53
**AC008982.1**	5,35	4,82	0,53
**ABCC11**	3,40	2,87	0,53
**ARHGAP31**	5,90	5,37	0,53
**CEL**	7,05	6,52	0,53
**SAMD4B**	10,40	9,87	0,53
**HELB**	8,18	7,65	0,53
**SUMO4**	4,87	4,34	0,53
**CCDC153**	4,87	4,34	0,53
**GNRH2**	4,31	3,78	0,53
**MAP3K21**	10,78	10,25	0,53
**RRAS**	7,98	7,45	0,53
**RBM44**	6,74	6,20	0,54
**PRELID2**	8,08	7,54	0,54
**MS4A7**	5,83	5,29	0,54
**CLEC20A**	8,18	7,64	0,54
**MAP3K9**	7,95	7,41	0,54
**AK5**	4,51	3,97	0,54
**FAM8A1**	10,06	9,52	0,54
**SLC37A2**	5,71	5,16	0,54
**CYP27A1**	3,43	2,89	0,54
**ENPP1**	8,68	8,13	0,54
**MTSS1**	8,52	7,98	0,54
**AQP3**	6,53	5,99	0,55
**CXCL12**	5,42	4,88	0,55
**ST8SIA5**	9,77	9,22	0,55
**ZNF585B**	10,43	9,89	0,55
**PTGR1**	2,22	1,67	0,55
**RFFL**	10,69	10,15	0,55
**KLHDC8B**	6,10	5,55	0,55
**NUDT6**	7,88	7,33	0,55
**TRIM17**	7,03	6,48	0,55
**METTL7A**	10,28	9,73	0,55
**PROKR1**	4,02	3,47	0,55
**TSPAN10**	4,02	3,47	0,55

**AGAP2**	10,99	10,44	0,55
**PANK1**	8,91	8,36	0,55
**CIDEB**	8,67	8,11	0,55
**CABLES1**	7,86	7,30	0,55
**FAM229B**	8,16	7,61	0,55
**SCNN1A**	2,87	2,31	0,56
**FAS**	7,70	7,14	0,56
**NAGS**	4,44	3,88	0,56
**RUNX2**	4,08	3,53	0,56
**AL445423.3**	6,48	5,92	0,56
**EFCAB5**	8,30	7,75	0,56
**RUNDC3A**	2,85	2,29	0,56
**PTPN14**	8,21	7,65	0,56
**ZSCAN1**	7,18	6,62	0,56
**SIRT4**	6,99	6,43	0,56
**TBC1D32**	10,29	9,73	0,56
**DOCK4**	8,82	8,26	0,56
**CENPL**	9,90	9,34	0,56
**SOCS2**	8,03	7,47	0,56
**SQOR**	7,49	6,93	0,56
**DNASE1L2**	7,75	7,19	0,57
**NUTM1**	4,92	4,36	0,57
**ZNF449**	8,81	8,24	0,57
**OMG**	5,41	4,83	0,57
**PDE9A**	4,70	4,13	0,57
**TAB3**	11,32	10,75	0,57
**SYN1**	5,98	5,40	0,57
**RPGRIP1**	4,37	3,79	0,57
**OSBPL10**	7,89	7,32	0,57
**PABPC3**	3,92	3,35	0,58
**IGHV4-28**	3,92	3,35	0,58
**GDPD3**	7,41	6,83	0,58
**NCOA1**	11,12	10,54	0,58
**LRRC61**	5,85	5,27	0,58
**DNM1**	10,19	9,61	0,58
**CA9**	3,28	2,70	0,58
**SOD3**	3,28	2,70	0,58
**HPCA**	3,28	2,70	0,58
**CAPNS2**	3,28	2,70	0,58
**TBR1**	3,28	2,70	0,58
**AC005702.1**	3,28	2,70	0,58
**SLC2A9**	6,57	5,99	0,58
**USP36**	9,46	8,88	0,58
**RASGRF2**	7,30	6,72	0,58
**UBA6**	13,04	12,46	0,58
**CKB**	7,40	6,82	0,58
**RBP5**	4,24	3,66	0,58
**TIGD7**	8,51	7,92	0,58
**ARAP3**	8,30	7,71	0,58
**TNPO3**	9,85	9,26	0,59
**TIMM23B-AGAP6**	11,03	10,45	0,59
**TMEM240**	4,34	3,75	0,59
**ODF2L**	9,95	9,36	0,59
**MIGA1**	8,69	8,10	0,59
**GAS2L3**	9,49	8,90	0,59
**KLHL13**	3,11	2,52	0,59

**TMCC2**	4,38	3,80	0,59
**IQSEC2**	7,67	7,08	0,59
**MSX1**	5,51	4,92	0,59
**LDB2**	2,86	2,27	0,59
**OR10H5**	2,09	1,50	0,59
**CCDC177**	2,09	1,50	0,59
**VSIG1**	2,09	1,50	0,59
**IL17B**	2,09	1,50	0,59
**CD8A**	2,09	1,50	0,59
**AIM2**	2,09	1,50	0,59
**GPR82**	2,09	1,50	0,59
**AP002884.2**	2,09	1,50	0,59
**AC097625.1**	2,09	1,50	0,59
**PCF11**	11,40	10,81	0,59
**SETD1A**	11,94	11,35	0,59
**FAM90A26**	5,33	4,74	0,59
**OPHN1**	9,88	9,29	0,59
**ZNF530**	8,85	8,26	0,59
**CLDN2**	3,00	2,40	0,59
**CIBAR1**	11,11	10,51	0,60
**GRIK5**	10,11	9,52	0,60
**DDX58**	10,41	9,82	0,60
**SUN2**	10,08	9,48	0,60
**AL391628.1**	8,81	8,21	0,60
**AC079447.1**	6,41	5,81	0,60
**BPHL**	9,97	9,37	0,60
**RPRML**	4,87	4,27	0,60
**ASB9**	4,87	4,27	0,60
**OTOR**	6,84	6,24	0,60
**LSMEM1**	3,73	3,13	0,60
**CYSLTR2**	3,43	2,83	0,60
**TLCD4**	5,61	5,01	0,60
**RAB42**	6,26	5,66	0,60
**PCDHGC5**	6,70	6,10	0,60
**CD300C**	4,58	3,97	0,61
**CENPI**	7,62	7,01	0,61
**C19orf18**	6,68	6,07	0,61
**POLR2J3**	12,24	11,63	0,61
**SLC9B1**	6,34	5,73	0,61
**ZNF674**	7,82	7,21	0,61
**IRAK1BP1**	9,64	9,03	0,61
**MRPL30**	12,32	11,71	0,61
**ZBTB49**	9,31	8,70	0,61
**CPEB4**	8,99	8,38	0,61
**PPFIA2**	6,44	5,82	0,61
**MDM1**	10,66	10,05	0,61
**BCL6B**	7,36	6,74	0,61
**SRSF12**	6,34	5,73	0,61
**CD1C**	5,25	4,64	0,61
**C1R**	7,43	6,81	0,61
**HEATR4**	4,70	4,09	0,61
**EID3**	6,17	5,55	0,62
**RBM20**	6,58	5,97	0,62
**IGLC3**	5,71	5,09	0,62
**SLC26A4**	4,20	3,59	0,62
**PDZD9**	4,20	3,59	0,62

**ANKRD20A1**	7,30	6,68	0,62
**ZNF564**	10,41	9,79	0,62
**DGCR6**	5,83	5,21	0,62
**AL035078.4**	8,87	8,25	0,62
**SH3GL2**	5,03	4,41	0,62
**TENM3**	12,06	11,44	0,62
**C3orf62**	10,61	9,98	0,62
**FAM186A**	4,48	3,86	0,62
**MAIP1**	7,52	6,90	0,62
**ANG**	5,08	4,46	0,62
**COL8A1**	3,30	2,68	0,62
**BX255925.3**	8,33	7,71	0,62
**MCC**	5,93	5,31	0,62
**CA11**	8,62	7,99	0,62
**REV3L**	12,86	12,24	0,62
**SLC13A3**	4,51	3,88	0,63
**ALDH1A3**	4,51	3,88	0,63
**MOB3C**	8,01	7,38	0,63
**ADGRE2**	5,17	4,54	0,63
**PRR15L**	4,76	4,13	0,63
**SEMA4A**	6,66	6,03	0,63
**TTC30B**	8,25	7,62	0,63
**TEAD1**	8,16	7,52	0,63
**ZNF26**	11,77	11,13	0,64
**ASB8**	11,46	10,82	0,64
**ORAI2**	11,55	10,91	0,64
**INSL3**	4,21	3,58	0,64
**KLKB1**	6,51	5,87	0,64
**PROC**	2,83	2,20	0,64
**NDOR1**	8,73	8,09	0,64
**FAAH2**	3,70	3,06	0,64
**H2AC16**	3,70	3,06	0,64
**SFN**	3,70	3,06	0,64
**RAB33A**	5,16	4,53	0,64
**HSD17B13**	5,16	4,53	0,64
**C1QL3**	6,71	6,07	0,64
**CASP9**	9,44	8,80	0,64
**LRRD1**	4,60	3,95	0,64
**HSF5**	6,57	5,92	0,64
**CARD14**	1,62	0,98	0,64
**GALNT6**	5,09	4,44	0,64
**MOBP**	3,18	2,54	0,64
**ADAMTS3**	6,69	6,05	0,64
**SHISA4**	6,25	5,61	0,64
**TMEM178B**	5,74	5,09	0,65
**DNAH7**	2,12	1,48	0,65
**STAB1**	4,18	3,54	0,65
**PIH1D2**	6,21	5,56	0,65
**KCNJ1**	4,44	3,79	0,65
**C2orf88**	7,61	6,96	0,65
**AC067968.1**	6,74	6,09	0,65
**H3C7**	4,70	4,05	0,65
**TINAGL1**	6,14	5,49	0,65
**PPP1R3F**	9,23	8,58	0,65
**TRAF3IP3**	8,99	8,34	0,65
**SCN8A**	7,48	6,82	0,65

**UBXN10**	7,51	6,85	0,65
**TMEM236**	5,12	4,47	0,65
**RET**	4,07	3,42	0,65
**CCR10**	6,15	5,50	0,66
**PNMA8A**	4,72	4,06	0,66
**TCTE1**	6,29	5,63	0,66
**C2**	3,84	3,18	0,66
**SLC25A27**	6,75	6,09	0,66
**PRDM1**	6,84	6,17	0,66
**ERVW-1**	4,61	3,94	0,66
**NMNAT3**	6,05	5,38	0,66
**SLC35F5**	12,27	11,61	0,67
**FAM174B**	7,13	6,46	0,67
**EXPH5**	7,69	7,03	0,67
**PCDHGA6**	6,78	6,12	0,67
**GDF7**	5,79	5,13	0,67
**FOCAD**	7,77	7,11	0,67
**ARHGEF33**	4,67	4,00	0,67
**FRK**	5,07	4,41	0,67
**HEY2**	3,33	2,66	0,67
**CASP1**	7,55	6,88	0,67
**ZNF226**	9,55	8,88	0,67
**SH2D3A**	6,50	5,83	0,67
**KCNQ5**	11,66	10,99	0,67
**TMC7**	4,37	3,69	0,67
**RELN**	4,31	3,64	0,67
**ZDHHC23**	7,91	7,24	0,67
**ZNF625**	7,86	7,18	0,67
**GIPC3**	5,14	4,46	0,67
**WNT10B**	4,02	3,35	0,68
**CUZD1**	6,76	6,09	0,68
**EXD2**	7,36	6,68	0,68
**LRRC8E**	6,34	5,66	0,68
**INPP1**	8,31	7,63	0,68
**ZNF277**	10,56	9,88	0,68
**ZNF283**	9,47	8,79	0,68
**ZNF774**	7,19	6,51	0,68
**GTF2H5**	9,49	8,81	0,68
**VCAN**	2,67	1,99	0,68
**PPP1R16A**	9,88	9,19	0,68
**GATD3A**	6,85	6,17	0,69
**FLRT3**	5,42	4,73	0,69
**DUX4**	3,48	2,79	0,69
**STAMBP**	5,61	4,92	0,69
**CDRT1**	5,26	4,57	0,69
**PDC**	2,91	2,22	0,69
**KCNE2**	2,91	2,22	0,69
**PIGR**	2,91	2,22	0,69
**SH2D7**	2,91	2,22	0,69
**HSFX4**	2,91	2,22	0,69
**GPC4**	2,91	2,22	0,69
**SULT4A1**	2,91	2,22	0,69
**TGM4**	2,91	2,22	0,69
**KIR3DL2**	2,91	2,22	0,69
**ZFHX4**	3,48	2,79	0,69
**FAM3D**	5,70	5,01	0,69

**IFT74**	7,26	6,57	0,69
**ERVMER34-1**	3,09	2,40	0,69
**GNG4**	4,58	3,88	0,69
**ARHGEF35**	5,33	4,64	0,69
**FAM3B**	1,95	1,26	0,69
**KCNH3**	6,19	5,50	0,70
**PLA2G1B**	1,95	1,26	0,70
**SYCP2**	7,15	6,45	0,70
**CD300A**	5,56	4,86	0,70
**ZNF81**	10,79	10,10	0,70
**AKAP5**	8,02	7,32	0,70
**RHOU**	6,71	6,01	0,70
**WDR88**	5,58	4,88	0,70
**CDHR4**	4,10	3,40	0,70
**PDZK1**	6,28	5,57	0,70
**GRAMD1C**	7,77	7,07	0,70
**PPP1R12B**	11,15	10,44	0,70
**ZNF625-ZNF20**	7,82	7,12	0,71
**NEDD9**	7,17	6,46	0,71
**CARMIL1**	7,82	7,12	0,71
**ACSM1**	3,17	2,46	0,71
**SLC46A2**	4,76	4,05	0,71
**HTR2B**	4,76	4,05	0,71
**GJA9**	5,02	4,31	0,71
**PEAK1**	9,57	8,86	0,71
**CNN2**	5,88	5,16	0,71
**ARL5C**	4,82	4,10	0,71
**ALOX12**	5,55	4,83	0,71
**MAGI1**	9,74	9,02	0,72
**GPR174**	4,51	3,79	0,72
**RGPD5**	10,53	9,81	0,72
**FPR3**	2,53	1,81	0,72
**PAK6**	4,49	3,77	0,72
**CPN2**	8,18	7,46	0,72
**STAP1**	5,25	4,53	0,72
**MFSD8**	11,12	10,39	0,73
**PRR15**	6,92	6,19	0,73
**PPP1R27**	3,41	2,68	0,73
**MAML2**	5,74	5,01	0,73
**HESX1**	6,41	5,68	0,73
**PCDHGB7**	7,29	6,56	0,73
**TNN**	1,67	0,94	0,73
**BCL2L14**	1,67	0,94	0,73
**TSPAN1**	1,67	0,94	0,73
**MROH2A**	1,67	0,94	0,73
**ZNF98**	1,67	0,94	0,73
**MCCD1**	1,67	0,94	0,73
**GK3P**	1,67	0,94	0,73
**ETDA**	1,67	0,94	0,73
**AC010619.1**	1,67	0,94	0,73
**IGHV7-4-1**	1,67	0,94	0,73
**HCRT**	1,67	0,94	0,73
**SLC8A3**	4,52	3,79	0,73
**ABCC6**	3,43	2,70	0,73
**IGHV5-51**	3,43	2,70	0,73
**DLX1**	2,44	1,71	0,73

**SGSH**	5,38	4,64	0,73
**RUNX3**	5,47	4,73	0,73
**TMEM31**	4,70	3,97	0,73
**AL049650.1**	5,07	4,34	0,73
**CD27**	6,00	5,27	0,73
**B4GALNT1**	6,42	5,69	0,73
**NEFM**	3,62	2,88	0,73
**POF1B**	3,36	2,62	0,74
**ACOXL**	5,97	5,23	0,74
**C2orf15**	5,21	4,47	0,74
**TOGARAM1**	10,00	9,26	0,74
**CATSPER2**	8,82	8,08	0,74
**WDR35**	7,53	6,78	0,74
**PADI4**	5,58	4,83	0,75
**HSBP1L1**	6,65	5,90	0,75
**PTPN20**	2,33	1,58	0,75
**VASH2**	10,46	9,71	0,75
**LRRIQ1**	6,70	5,95	0,75
**WSCD1**	6,69	5,94	0,75
**SLX1A**	10,28	9,53	0,75
**RASGEF1B**	8,19	7,43	0,75
**BRPF1**	9,75	9,00	0,75
**MAPT**	6,31	5,56	0,75
**MSH4**	5,44	4,69	0,75
**GDF6**	3,82	3,06	0,75
**OR13A1**	4,83	4,08	0,76
**APOA1**	5,16	4,41	0,76
**GAS6**	5,55	4,79	0,76
**SLC7A7**	6,37	5,61	0,77
**NAPSA**	4,41	3,65	0,77
**DNAJC18**	9,61	8,85	0,77
**PCDHGB6**	5,74	4,97	0,77
**ZBED2**	4,12	3,35	0,77
**PSMD5**	11,57	10,80	0,77
**GORAB**	9,02	8,25	0,77
**RAB7B**	2,56	1,79	0,77
**LMBR1L**	10,68	9,90	0,77
**NRN1L**	4,75	3,98	0,77
**LSP1**	4,98	4,20	0,77
**HAPLN4**	4,98	4,20	0,77
**CELF5**	6,49	5,72	0,77
**ERN2**	1,70	0,92	0,78
**AC105052.1**	5,61	4,83	0,78
**UNC79**	5,12	4,34	0,78
**FOXD4L3**	5,12	4,34	0,78
**PAMR1**	5,06	4,29	0,78
**GPR25**	4,37	3,59	0,78
**AOPEP**	8,28	7,50	0,78
**TRIM39**	9,84	9,06	0,78
**DNAH3**	2,69	1,91	0,78
**AC008758.6**	2,69	1,91	0,78
**ERICH6**	2,69	1,91	0,78
**SPRED3**	2,69	1,91	0,78
**AC137834.1**	7,36	6,58	0,78
**KIAA0319**	5,58	4,79	0,78
**SAMD11**	6,40	5,62	0,78

**PLXDC1**	4,58	3,79	0,79
**VIPR1**	6,09	5,31	0,79
**CEACAM19**	4,85	4,06	0,79
**ENPP3**	4,67	3,88	0,79
**LANCL3**	4,76	3,97	0,79
**CTNNA2**	3,84	3,04	0,79
**RASD1**	6,64	5,84	0,79
**INSIG2**	10,82	10,02	0,80
**HCAR2**	3,28	2,48	0,80
**SV2B**	5,06	4,26	0,80
**CCDC144A**	6,43	5,63	0,80
**NECAP1**	11,46	10,66	0,80
**NCALD**	7,66	6,86	0,80
**H2BC6**	6,81	6,01	0,80
**ZNF300**	5,21	4,41	0,80
**IGSF22**	4,24	3,44	0,80
**NECTIN3**	8,34	7,53	0,81
**LIPH**	3,70	2,89	0,81
**AC005324.2**	3,70	2,89	0,81
**SLC6A5**	5,44	4,64	0,81
**SAT1**	8,19	7,38	0,81
**BST1**	7,34	6,53	0,81
**WTIP**	7,56	6,75	0,81
**MR1**	10,60	9,79	0,82
**ELAPOR1**	4,26	3,45	0,82
**LY9**	7,99	7,17	0,82
**FAM90A14P**	4,70	3,88	0,82
**FAM90A1**	7,10	6,28	0,82
**SUSD5**	6,48	5,66	0,82
**GCGR**	2,37	1,55	0,82
**GSTM5**	4,33	3,51	0,82
**F3**	4,87	4,05	0,82
**AL139300.1**	9,30	8,48	0,82
**MYLPF**	2,71	1,89	0,82
**DEPTOR**	5,16	4,34	0,82
**TRPS1**	4,41	3,59	0,82
**GCA**	9,15	8,32	0,83
**RIMS2**	4,41	3,59	0,83
**TAF2**	12,12	11,29	0,83
**COBLL1**	7,81	6,99	0,83
**LOXHD1**	5,39	4,56	0,83
**ZNF383**	8,91	8,08	0,83
**PLAT**	6,39	5,55	0,84
**ANKEF1**	7,09	6,25	0,84
**AC005154.5**	6,00	5,16	0,84
**CRHR2**	3,13	2,29	0,84
**MAMSTR**	6,93	6,09	0,84
**GABRA5**	3,71	2,86	0,85
**TMEM191B**	4,98	4,13	0,85
**CLIC2**	6,31	5,46	0,85
**PTN**	4,64	3,79	0,85
**CABYR**	6,33	5,48	0,85
**DPYSL5**	4,44	3,59	0,85
**MEIG1**	4,44	3,59	0,85
**SMOC2**	5,13	4,28	0,85
**ZSCAN31**	7,98	7,13	0,85

SIDT1	5,74	4,88	0,86
GPR171	4,20	3,35	0,86
CCDC152	4,20	3,35	0,86
DACH1	4,20	3,35	0,86
STAR	4,99	4,13	0,86
CCSER2	11,92	11,05	0,86
POU4F3	3,92	3,06	0,86
KLHL41	3,92	3,06	0,86
HLA-DQB2	6,81	5,95	0,86
BNIPL	6,63	5,76	0,86
ZKSCAN7	5,20	4,34	0,86
TMEM210	4,17	3,30	0,87
RAPGEF4	5,08	4,21	0,87
TSPAN18	3,57	2,70	0,87
KCND3	3,57	2,70	0,87
ARHGEF26	3,57	2,70	0,87
CNDP1	3,57	2,70	0,87
SLC35G2	3,57	2,70	0,87
MYO18B	3,61	2,74	0,87
CX3CR1	4,35	3,48	0,87
ABCA13	2,78	1,90	0,87
SLC26A7	5,35	4,47	0,87
CCNB3	4,54	3,66	0,87
ESR2	6,79	5,91	0,88
ATP1A2	8,48	7,60	0,88
TVP23C-CDRT4	3,62	2,74	0,88
PSORS1C1	3,01	2,13	0,88
PKHD1	5,41	4,53	0,88
AL139353.1	5,79	4,90	0,88
NTNG1	3,74	2,85	0,88
AK1	9,78	8,90	0,88
ATP5MGL	3,11	2,22	0,89
C19orf84	3,11	2,22	0,89
OOSP1	3,11	2,22	0,89
AC027644.4	6,05	5,16	0,89
CLUL1	5,13	4,24	0,89
PSD	6,29	5,40	0,89
PTPN22	8,27	7,38	0,89
IKZF3	5,90	5,01	0,89
NKIRAS1	10,40	9,50	0,90
GNRHR	6,10	5,20	0,90
KATNIP	6,43	5,53	0,90
CFAP43	4,07	3,17	0,90
AL645941.2	5,37	4,47	0,90
CCM2L	4,87	3,97	0,90
TLE6	6,56	5,65	0,90
HCST	4,53	3,63	0,90
RGN	1,77	0,87	0,90
LMX1A	3,97	3,06	0,91
PLPPR2	7,43	6,52	0,91
CTF1	5,64	4,74	0,91
NT5C1B	3,41	2,50	0,91
GYS2	5,25	4,34	0,91
EVPL	4,23	3,32	0,91
KLF8	5,55	4,64	0,91
MAGEA8	3,69	2,78	0,91

RNF183	2,80	1,88	0,91
CSF3R	4,87	3,95	0,92
H2BC15	6,52	5,60	0,92
ARHGAP28	6,41	5,49	0,92
TRPM6	5,24	4,32	0,92
PWWP2B	5,74	4,83	0,92
RHOXF1	2,42	1,50	0,92
SCG3	2,42	1,50	0,92
KLRB1	2,42	1,50	0,92
LRRN4	2,42	1,50	0,92
STARD8	2,42	1,50	0,92
PGM5	2,42	1,50	0,92
OXGR1	2,42	1,50	0,92
CAVIN2	2,42	1,50	0,92
SOWAHB	2,42	1,50	0,92
AL158151.3	2,42	1,50	0,92
GALNT16	2,42	1,50	0,92
CDO1	2,42	1,50	0,92
WNK4	2,42	1,50	0,92
FAIM2	2,42	1,50	0,92
UGT3A1	2,42	1,50	0,92
ALPP	2,42	1,50	0,92
IGF2	2,42	1,50	0,92
H3-4	2,42	1,50	0,92
TMEM37	2,42	1,50	0,92
LRG1	2,42	1,50	0,92
PHOSPHO1	2,42	1,50	0,92
PSAPL1	2,42	1,50	0,92
C2orf66	2,42	1,50	0,92
GGTA1	2,42	1,50	0,92
PLIN5	2,42	1,50	0,92
TMEM238L	2,42	1,50	0,92
AC107959.5	2,42	1,50	0,92
FAM90A7P	2,42	1,50	0,92
SSUH2	5,33	4,41	0,92
DOK7	4,15	3,23	0,92
NEBL	3,28	2,36	0,92
APLN	4,51	3,59	0,92
TIGD4	5,61	4,69	0,92
FOXP2	5,50	4,57	0,93
COL24A1	4,79	3,86	0,93
BOLL	2,09	1,16	0,93
AZU1	2,09	1,16	0,93
PLLP	7,60	6,67	0,93
SDC2	8,82	7,89	0,93
CDHR2	6,19	5,25	0,93
SOHLH2	4,98	4,05	0,93
GPM6A	5,15	4,22	0,93
WBP2NL	4,46	3,53	0,93
ADCY4	4,73	3,80	0,94
ZNF415	8,75	7,81	0,94
EPHX3	4,29	3,35	0,94
LMOD1	5,82	4,88	0,94
ITGAL	6,19	5,25	0,94
KRIT1	10,55	9,61	0,95
SPTBN4	4,47	3,53	0,95

**PDE1A**	2,94	2,00	0,95
**AL096711.2**	5,74	4,79	0,95
**PINX1**	5,29	4,34	0,95
**EPX**	3,43	2,48	0,95
**ADCY10**	3,43	2,48	0,95
**SMCO3**	3,43	2,48	0,95
**SMIM34B**	3,43	2,48	0,95
**CNIH3**	5,48	4,53	0,95
**MROH7-TTC4**	5,62	4,66	0,95
**AANAT**	3,65	2,69	0,95
**FCGBP**	6,00	5,05	0,95
**MARCHF10**	4,75	3,80	0,95
**MCPH1**	10,36	9,40	0,96
**PTEN**	9,60	8,64	0,96
**PAPOLB**	4,02	3,06	0,96
**SCG5**	4,00	3,04	0,96
**SH3D19**	4,44	3,47	0,97
**NIPAL1**	5,61	4,64	0,97
**PCDHB3**	4,76	3,79	0,97
**FGF16**	4,76	3,79	0,97
**ZNF35**	7,29	6,32	0,97
**DNAH10**	6,16	5,18	0,97
**VIL1**	5,25	4,27	0,97
**AMOTL2**	6,54	5,56	0,98
**RSPH4A**	6,84	5,86	0,98
**CACNA1D**	5,44	4,46	0,98
**C4orf50**	4,35	3,36	0,99
**COQ10B**	6,17	5,18	0,99
**BGN**	7,83	6,84	0,99
**CEACAM1**	6,95	5,97	0,99
**AKAP10**	11,77	10,78	0,99
**LOXL4**	5,12	4,13	0,99
**AXL**	4,58	3,59	0,99
**AVPR1B**	4,20	3,21	0,99
**COL4A3**	3,96	2,96	0,99
**GRIK1**	3,70	2,70	1,00
**PXT1**	3,70	2,70	1,00
**PATE2**	3,70	2,70	1,00
**SP140**	2,91	1,91	1,00
**CSGALNACT1**	5,77	4,77	1,00
**ZNF205**	2,47	1,47	1,00
**DAGLA**	5,41	4,41	1,00
**GABRD**	5,34	4,34	1,00
**SYT15**	7,56	6,56	1,00
**HFM1**	8,17	7,17	1,00
**GFRA3**	4,76	3,75	1,00
**SIAH3**	2,91	1,91	1,01
**MTARC2**	2,91	1,91	1,01
**KCNS1**	2,91	1,91	1,01
**KCNJ18**	2,91	1,91	1,01
**SGK1**	10,98	9,98	1,01
**PRUNE2**	7,40	6,40	1,01
**CHI3L2**	5,30	4,29	1,01
**ZNF221**	6,94	5,93	1,01
**MGAM**	4,70	3,69	1,01
**SH3BGR**	6,66	5,65	1,01

**ZNF732**	5,48	4,47	1,01
**AC098588.2**	6,25	5,24	1,01
**GIMAP8**	4,37	3,35	1,02
**TAT**	4,37	3,35	1,02
**NKAIN2**	7,18	6,17	1,02
**MAP7D2**	5,55	4,53	1,02
**BEST3**	2,86	1,83	1,02
**PKP3**	3,09	2,07	1,03
**FANK1**	5,75	4,73	1,03
**RGPD4**	6,99	5,97	1,03
**PKD2L2**	4,47	3,44	1,03
**TAS2R3**	5,16	4,13	1,03
**TPD52L1**	4,80	3,76	1,04
**CCDC181**	3,54	2,51	1,04
**RTF1**	7,65	6,61	1,04
**CDHR3**	4,91	3,87	1,04
**SLC25A52**	4,44	3,40	1,05
**QPCT**	6,35	5,31	1,05
**SULT1A2**	5,50	4,45	1,05
**SLC35F4**	3,55	2,50	1,05
**APOM**	6,03	4,98	1,05
**DDX60L**	10,74	9,69	1,05
**TM4SF19**	4,68	3,63	1,05
**IGHG1**	2,50	1,44	1,05
**EFCAB10**	5,98	4,93	1,05
**PECR**	6,14	5,09	1,06
**RALGPS2**	4,64	3,59	1,06
**MED12L**	9,34	8,28	1,06
**ACTN2**	3,28	2,22	1,06
**ACR**	3,28	2,22	1,06
**SERPINC1**	3,28	2,22	1,06
**STX11**	3,28	2,22	1,06
**SH2D6**	3,28	2,22	1,06
**EPO**	3,28	2,22	1,06
**SLC10A4**	3,28	2,22	1,06
**TRIM55**	3,28	2,22	1,06
**SYNDIG1L**	3,28	2,22	1,06
**SOCS3**	3,28	2,22	1,06
**LEFTY1**	3,28	2,22	1,06
**PTCH2**	7,43	6,37	1,06
**TMEM67**	8,62	7,56	1,06
**TLR6**	6,31	5,25	1,06
**TULP2**	4,05	2,99	1,06
**COPZ2**	1,06	0,00	1,06
**CHRDL2**	1,06	0,00	1,06
**ERBB3**	1,06	0,00	1,06
**SCT**	1,06	0,00	1,06
**BRS3**	1,06	0,00	1,06
**PRSS33**	1,06	0,00	1,06
**KISS1R**	1,06	0,00	1,06
**TREH**	1,06	0,00	1,06
**HCRTR1**	1,06	0,00	1,06
**DNAI1**	1,06	0,00	1,06
**ZBP1**	1,06	0,00	1,06
**GPR42**	1,06	0,00	1,06
**SPINK2**	1,06	0,00	1,06

**CNN1**	1,06	0,00	1,06
**C17orf64**	1,06	0,00	1,06
**ZIM3**	1,06	0,00	1,06
**SELENBP1**	1,06	0,00	1,06
**HORMAD1**	1,06	0,00	1,06
**CNPY1**	1,06	0,00	1,06
**CYP17A1**	1,06	0,00	1,06
**ADCY8**	1,06	0,00	1,06
**SNX7**	1,06	0,00	1,06
**RAET1E**	1,06	0,00	1,06
**DEFB1**	1,06	0,00	1,06
**B4GALNT2**	1,06	0,00	1,06
**TMEM145**	1,06	0,00	1,06
**ATCAY**	1,06	0,00	1,06
**NSG2**	1,06	0,00	1,06
**KISS1**	1,06	0,00	1,06
**GTSF1**	1,06	0,00	1,06
**NEUROD2**	1,06	0,00	1,06
**CFAP46**	1,06	0,00	1,06
**KCNH6**	1,06	0,00	1,06
**OR4F21**	1,06	0,00	1,06
**FOXR1**	1,06	0,00	1,06
**FMR1NB**	1,06	0,00	1,06
**OR52D1**	1,06	0,00	1,06
**FFAR3**	1,06	0,00	1,06
**CYP4F12**	1,06	0,00	1,06
**QRFPR**	1,06	0,00	1,06
**ACTL7A**	1,06	0,00	1,06
**ADH1A**	1,06	0,00	1,06
**CPSF4L**	1,06	0,00	1,06
**NCCRP1**	1,06	0,00	1,06
**TEX45**	1,06	0,00	1,06
**IGKV6-21**	1,06	0,00	1,06
**TRGV10**	1,06	0,00	1,06
**TRGV5**	1,06	0,00	1,06
**TAS2R50**	1,06	0,00	1,06
**GBP7**	1,06	0,00	1,06
**MROH5**	1,06	0,00	1,06
**TEX48**	1,06	0,00	1,06
**CFAP97D1**	1,06	0,00	1,06
**OR1J4**	1,06	0,00	1,06
**IGKV2-28**	1,06	0,00	1,06
**IGKV1-27**	1,06	0,00	1,06
**AL049779.1**	1,06	0,00	1,06
**SLC6A14**	1,06	0,00	1,06
**AC008763.2**	1,06	0,00	1,06
**CTAGE6**	1,06	0,00	1,06
**HNRNPCL2**	1,06	0,00	1,06
**OR4F29**	1,06	0,00	1,06
**DHRS9**	1,06	0,00	1,06
**ZFR2**	1,06	0,00	1,06
**FGFBP2**	1,06	0,00	1,06
**MMEL1**	1,06	0,00	1,06
**RXRG**	1,06	0,00	1,06
**OR10A3**	1,06	0,00	1,06
**OR2T12**	1,06	0,00	1,06

**ADIPOQ**	1,06	0,00	1,06
**XKRX**	1,06	0,00	1,06
**SERINC4**	1,06	0,00	1,06
**SHOX**	1,06	0,00	1,06
**CPNE4**	1,06	0,00	1,06
**OOEP**	1,06	0,00	1,06
**IGLV3-16**	1,06	0,00	1,06
**APOC2**	1,06	0,00	1,06
**STPG4**	1,06	0,00	1,06
**C11orf97**	1,06	0,00	1,06
**AC244517.10**	1,06	0,00	1,06
**C5AR2**	1,06	0,00	1,06
**ZNF716**	1,06	0,00	1,06
**KCNH7**	1,06	0,00	1,06
**PDE4B**	6,78	5,71	1,07
**SEL1L3**	10,02	8,95	1,07
**NCR3LG1**	6,30	5,23	1,07
**MED12**	10,58	9,51	1,08
**CARNS1**	4,29	3,21	1,08
**CHAD**	4,29	3,21	1,08
**SMPDL3B**	4,25	3,17	1,08
**AC064824.1**	7,06	5,98	1,08
**SPATA32**	4,87	3,79	1,08
**ZNF534**	4,77	3,69	1,08
**DCBLD1**	6,10	5,01	1,09
**MRGPRX2**	3,57	2,48	1,09
**CD1D**	2,64	1,55	1,09
**ASPDH**	1,89	0,80	1,09
**FAM227B**	7,39	6,29	1,10
**LINC00672**	6,19	5,09	1,10
**RUBCNL**	4,78	3,68	1,10
**FAM71F2**	6,71	5,61	1,11
**ECM2**	4,49	3,39	1,11
**IL11**	5,06	3,95	1,11
**TNFSF10**	3,82	2,70	1,11
**ITIH2**	3,82	2,70	1,11
**ATOH8**	3,82	2,70	1,11
**JCHAIN**	8,43	7,32	1,12
**PURG**	4,70	3,59	1,12
**CORO7-PAM16**	6,16	5,04	1,12
**PPP1R14C**	5,25	4,13	1,12
**STX16-NPEPL1**	6,21	5,09	1,12
**TRO**	6,25	5,12	1,12
**EXTL1**	4,02	2,89	1,13
**SYNGR4**	4,02	2,89	1,13
**AP003071.5**	4,02	2,89	1,13
**SGCA**	2,21	1,07	1,14
**THEG**	3,15	2,02	1,14
**NPL**	8,68	7,54	1,14
**GAL3ST1**	4,47	3,33	1,14
**PDE6A**	4,20	3,06	1,14
**NACAD**	4,20	3,06	1,14
**POU5F2**	4,20	3,06	1,14
**DND1**	5,61	4,47	1,15
**IGKV3-20**	5,12	3,97	1,15
**TENM4**	12,85	11,70	1,15

**PTH1R**	4,37	3,21	1,15
**CTHRC1**	4,37	3,21	1,15
**AC006978.2**	4,37	3,21	1,15
**ALOX5AP**	6,12	4,97	1,15
**RHD**	4,21	3,05	1,16
**TGIF2-RAB5IF**	7,02	5,86	1,16
**FCGR1A**	4,51	3,35	1,16
**PIWIL2**	5,51	4,35	1,16
**CTSE**	3,73	2,56	1,17
**MEFV**	3,68	2,52	1,17
**CCDC110**	5,28	4,11	1,17
**ABCA9**	6,81	5,64	1,17
**TBC1D19**	9,47	8,29	1,17
**ASPG**	5,04	3,86	1,18
**TSTD3**	5,64	4,47	1,18
**GRIN2D**	5,64	4,47	1,18
**IFI44**	7,32	6,14	1,18
**KBTBD3**	8,66	7,48	1,18
**AGBL3**	7,30	6,12	1,18
**REP15**	6,99	5,81	1,18
**SLC9C2**	2,69	1,50	1,19
**RARRES1**	2,69	1,50	1,19
**NUTM2F**	2,69	1,50	1,19
**PDCD1LG2**	2,69	1,50	1,19
**IFNA17**	2,69	1,50	1,19
**THEGL**	2,69	1,50	1,19
**CCDC195**	2,69	1,50	1,19
**AC012488.2**	2,69	1,50	1,19
**CYGB**	5,43	4,24	1,19
**PLEKHG5**	4,23	3,04	1,19
**FMN1**	7,90	6,70	1,19
**PTCHD4**	3,92	2,73	1,19
**SMARCD3**	7,30	6,10	1,20
**DAPP1**	5,33	4,13	1,20
**ARID3C**	3,11	1,91	1,20
**H2AC14**	3,11	1,91	1,20
**CCL3**	3,11	1,91	1,20
**SERPINB12**	5,41	4,20	1,20
**MOCOS**	5,55	4,34	1,21
**TNFRSF12A**	5,63	4,42	1,21
**CLTRN**	5,58	4,37	1,21
**CD226**	6,73	5,52	1,21
**EMP1**	5,74	4,53	1,21
**HIGD1B**	3,71	2,50	1,21
**SLC4A11**	6,60	5,38	1,21
**LMOD3**	5,95	4,74	1,22
**CHURC1**	3,70	2,48	1,22
**ERICH2**	3,70	2,48	1,22
**TEX12**	3,61	2,40	1,22
**ARHGEF37**	4,78	3,56	1,22
**PADI2**	3,29	2,07	1,22
**NEURL2**	6,29	5,07	1,22
**PIK3R6**	5,91	4,69	1,22
**ABHD4**	4,53	3,31	1,22
**TNC**	3,50	2,28	1,22
**CNIH2**	6,42	5,20	1,22

**HPX**	3,92	2,70	1,22
**PTX3**	3,92	2,70	1,22
**ESM1**	3,92	2,70	1,22
**NRCAM**	4,47	3,25	1,22
**CCNP**	3,76	2,53	1,22
**FBXO16**	7,34	6,11	1,22
**RIC3**	4,02	2,79	1,22
**FOLR2**	4,12	2,89	1,22
**DLG2**	5,71	4,49	1,23
**FAM98B**	13,91	12,68	1,23
**AC072022.2**	4,44	3,21	1,23
**CYBRD1**	4,58	3,35	1,23
**SYT2**	4,58	3,35	1,23
**CXCL16**	3,71	2,48	1,23
**PAPLN**	5,75	4,51	1,23
**PPP1R3G**	4,70	3,47	1,23
**PRSS12**	5,12	3,88	1,24
**SAMD5**	5,58	4,34	1,24
**NUP210L**	2,90	1,66	1,24
**MYL3**	4,77	3,53	1,24
**SLC11A1**	4,21	2,97	1,24
**GAD1**	4,40	3,16	1,24
**CDKL3**	7,56	6,31	1,25
**NPIPA3**	8,25	7,00	1,26
**C3orf52**	7,17	5,91	1,26
**FEZ1**	4,82	3,56	1,26
**ATAD3C**	6,72	5,46	1,27
**JHY**	6,19	4,92	1,27
**UPK3B**	3,45	2,18	1,27
**CRELD1**	7,30	6,03	1,27
**SATL1**	2,40	1,12	1,28
**CARD6**	5,07	3,79	1,28
**C4B**	6,26	4,97	1,28
**ST3GAL6**	2,84	1,56	1,29
**VWF**	4,87	3,59	1,29
**AC073585.1**	4,87	3,59	1,29
**LRRC7**	6,51	5,22	1,29
**CADM3**	4,32	3,03	1,29
**CORIN**	4,24	2,94	1,30
**USP44**	4,51	3,21	1,30
**DDO**	2,85	1,55	1,30
**RAB40AL**	4,37	3,06	1,30
**CES3**	5,66	4,35	1,31
**SLC22A1**	4,02	2,70	1,32
**IGHV1-69D**	4,02	2,70	1,32
**MAPK10**	6,19	4,87	1,32
**PATJ**	9,35	8,02	1,33
**SOX5**	7,44	6,12	1,33
**AC093899.2**	5,03	3,69	1,33
**TPRG1**	2,66	1,32	1,33
**CCDC91**	10,62	9,28	1,34
**TLR3**	7,68	6,34	1,34
**CYP2C18**	3,10	1,76	1,34
**OR2T8**	4,93	3,59	1,34
**TLR5**	4,66	3,31	1,34
**PRPH2**	3,57	2,22	1,35

**TMC3**	3,57	2,22	1,35
**PTPRO**	4,48	3,13	1,35
**ZNF843**	5,29	3,94	1,35
**FCRL1**	3,77	2,42	1,35
**FAM83G**	5,31	3,95	1,35
**USP17L7**	4,70	3,35	1,36
**NTRK3**	4,27	2,92	1,36
**KCNAB3**	6,73	5,37	1,36
**PCDHGA5**	4,58	3,21	1,37
**CSNK2A3**	4,58	3,21	1,37
**H4C15**	7,03	5,66	1,37
**CELA3B**	3,28	1,91	1,37
**NPAS1**	3,28	1,91	1,37
**AC055811.2**	3,28	1,91	1,37
**H2BC3**	3,28	1,91	1,37
**LY75-CD302**	7,97	6,59	1,37
**RORA**	5,82	4,44	1,38
**USH2A**	4,44	3,06	1,38
**SPOCK2**	4,44	3,06	1,38
**AL121753.1**	4,44	3,06	1,38
**ZNF705E**	4,44	3,06	1,38
**MUC6**	5,83	4,44	1,38
**ADAM23**	8,26	6,88	1,38
**DUOX1**	5,00	3,62	1,39
**CLVS1**	3,76	2,37	1,39
**KCNV2**	5,61	4,20	1,41
**ASB12**	5,61	4,20	1,41
**CACNA1G**	2,91	1,50	1,41
**RCVRN**	2,91	1,50	1,41
**IGFBP5**	2,91	1,50	1,41
**LPA**	2,91	1,50	1,41
**SMLR1**	2,91	1,50	1,41
**AC006030.1**	2,91	1,50	1,41
**PAK5**	2,91	1,50	1,41
**SIGLEC5**	2,91	1,50	1,41
**LIN28A**	2,91	1,50	1,41
**EHF**	2,91	1,50	1,41
**DBX2**	2,91	1,50	1,41
**CFAP73**	2,91	1,50	1,41
**FER1L5**	2,91	1,50	1,41
**WNT8A**	2,38	0,97	1,41
**SIGLEC11**	2,56	1,15	1,41
**VSTM5**	4,12	2,70	1,41
**KCNA1**	4,76	3,35	1,42
**CCDC148**	4,64	3,22	1,42
**BICRAL**	9,14	7,72	1,42
**TMEM232**	4,34	2,91	1,43
**REELD1**	5,95	4,53	1,43
**CYP2E1**	6,63	5,19	1,43
**GCNT7**	4,64	3,21	1,43
**SLC17A7**	3,08	1,65	1,43
**FAM95C**	5,32	3,89	1,44
**PTGER1**	3,92	2,48	1,44
**PDZD2**	6,08	4,64	1,44
**ZNF862**	10,34	8,90	1,44
**HSD11B2**	4,44	2,99	1,45

RAB44	4,51	3,06	1,45
TMEM130	4,00	2,54	1,46
TAFA1	5,51	4,05	1,46
WDFY4	4,37	2,89	1,47
AC004997.1	4,37	2,89	1,47
IL18R1	3,70	2,22	1,47
GRM1	3,70	2,22	1,47
TAC4	3,70	2,22	1,47
C6orf58	2,42	0,94	1,48
TRIM75P	2,42	0,94	1,48
CCDC166	2,42	0,94	1,48
IGLV3-9	2,42	0,94	1,48
MYBPHL	2,42	0,94	1,48
MYOT	2,61	1,12	1,48
HORMAD2	2,43	0,94	1,49
MYCN	3,90	2,41	1,49
CCL20	2,75	1,26	1,49
IMPG2	5,90	4,41	1,50
AC036214.3	5,29	3,79	1,50
SNTG2	4,29	2,77	1,51
RBPMS2	4,76	3,24	1,52
ITGA2B	5,41	3,89	1,52
AC010327.2	3,43	1,91	1,53
DPEP1	6,05	4,51	1,54
RFX2	8,32	6,77	1,55
ZNF582	5,51	3,95	1,55
PRSS55	3,67	2,11	1,56
HGD	3,94	2,38	1,56
TCP11	3,89	2,33	1,56
PCDHGA10	6,35	4,79	1,56
EME2	9,23	7,66	1,57
IL2RB	6,10	4,53	1,57
AL162417.1	8,66	7,09	1,58
SBK3	4,21	2,63	1,58
SPATA45	4,93	3,35	1,58
KCNE1	4,64	3,06	1,58
H1-5	4,64	3,06	1,58
FAM156A	7,69	6,10	1,58
NPPA	4,90	3,32	1,58
MEIOC	7,71	6,12	1,59
LRRC43	3,82	2,22	1,59
PM20D1	3,82	2,22	1,59
METTL7B	3,82	2,22	1,59
SDR42E1	3,82	2,22	1,59
CRYGN	3,30	1,70	1,60
GALNT4	9,51	7,91	1,60
LVRN	4,71	3,11	1,60
BIRC6	10,15	8,55	1,60
FGL2	3,11	1,50	1,61
ANO1	3,11	1,50	1,61
ERICH5	3,11	1,50	1,61
FOXI3	3,11	1,50	1,61
PRSS37	3,03	1,42	1,61
HMOX1	5,41	3,79	1,61
SMIM11A	6,29	4,67	1,61
NPIPA9	6,55	4,93	1,62

**H4C8**	7,41	5,79	1,62
**SBF2**	8,11	6,48	1,63
**RDH16**	5,33	3,69	1,64
**HHIPL1**	5,59	3,94	1,65
**RAB4B-EGLN2**	4,37	2,70	1,66
**MSMP**	4,37	2,70	1,66
**FGF2**	3,57	1,91	1,66
**RXFP4**	3,57	1,91	1,66
**COL4A5**	1,67	0,00	1,67
**PCDHA3**	1,67	0,00	1,67
**TLL1**	1,67	0,00	1,67
**CLDN18**	1,67	0,00	1,67
**CHRNA4**	1,67	0,00	1,67
**RSPH6A**	1,67	0,00	1,67
**RLN2**	1,67	0,00	1,67
**WNT5B**	1,67	0,00	1,67
**ENPP5**	1,67	0,00	1,67
**CCDC170**	1,67	0,00	1,67
**GJA3**	1,67	0,00	1,67
**PPP1R1B**	1,67	0,00	1,67
**PCDH8**	1,67	0,00	1,67
**LRRC32**	1,67	0,00	1,67
**DNASE2B**	1,67	0,00	1,67
**MMRN1**	1,67	0,00	1,67
**PROK1**	1,67	0,00	1,67
**SLC16A12**	1,67	0,00	1,67
**FRMD1**	1,67	0,00	1,67
**ADAMTS5**	1,67	0,00	1,67
**DKK2**	1,67	0,00	1,67
**HPD**	1,67	0,00	1,67
**SCUBE1**	1,67	0,00	1,67
**JAML**	1,67	0,00	1,67
**ACOX2**	1,67	0,00	1,67
**FBXW10**	1,67	0,00	1,67
**HPSE2**	1,67	0,00	1,67
**KY**	1,67	0,00	1,67
**SLCO2A1**	1,67	0,00	1,67
**IRX3**	1,67	0,00	1,67
**CAV3**	1,67	0,00	1,67
**SH2D1A**	1,67	0,00	1,67
**IFNE**	1,67	0,00	1,67
**CRACDL**	1,67	0,00	1,67
**PCDHA1**	1,67	0,00	1,67
**IGHV3-20**	1,67	0,00	1,67
**FER1L6**	1,67	0,00	1,67
**SLC22A31**	1,67	0,00	1,67
**LINC00514**	1,67	0,00	1,67
**AL024498.2**	1,67	0,00	1,67
**GOLGA6L6**	1,67	0,00	1,67
**AC009879.2**	1,67	0,00	1,67
**AC053503.6**	1,67	0,00	1,67
**HHATL**	1,67	0,00	1,67
**LTF**	1,67	0,00	1,67
**GPRC5A**	1,67	0,00	1,67
**C8B**	1,67	0,00	1,67
**SLAMF7**	1,67	0,00	1,67

**SLC18A1**	1,67	0,00	1,67
**P2RY10**	1,67	0,00	1,67
**APOB**	1,67	0,00	1,67
**TREM2**	1,67	0,00	1,67
**SEC14L3**	1,67	0,00	1,67
**CARD10**	1,67	0,00	1,67
**GLRA2**	1,67	0,00	1,67
**FGL1**	1,67	0,00	1,67
**RETN**	1,67	0,00	1,67
**SHD**	1,67	0,00	1,67
**PKD2L1**	1,67	0,00	1,67
**MYH1**	1,67	0,00	1,67
**CRYAB**	1,67	0,00	1,67
**FOLR3**	1,67	0,00	1,67
**CLEC2B**	1,67	0,00	1,67
**KRT18**	1,67	0,00	1,67
**GSG1**	1,67	0,00	1,67
**FHL5**	1,67	0,00	1,67
**PDE10A**	1,67	0,00	1,67
**C9**	1,67	0,00	1,67
**GNAT1**	1,67	0,00	1,67
**OTX1**	1,67	0,00	1,67
**SLC25A2**	1,67	0,00	1,67
**TAS2R9**	1,67	0,00	1,67
**NEUROG3**	1,67	0,00	1,67
**PI3**	1,67	0,00	1,67
**WFDC3**	1,67	0,00	1,67
**PDE11A**	1,67	0,00	1,67
**MNX1**	1,67	0,00	1,67
**BARX1**	1,67	0,00	1,67
**FCRL2**	1,67	0,00	1,67
**CHIT1**	1,67	0,00	1,67
**KL**	1,67	0,00	1,67
**SEC14L4**	1,67	0,00	1,67
**GSC**	1,67	0,00	1,67
**IL1RN**	1,67	0,00	1,67
**STRA6**	1,67	0,00	1,67
**FBLN5**	1,67	0,00	1,67
**LMAN1L**	1,67	0,00	1,67
**ITGAX**	1,67	0,00	1,67
**PLPP2**	1,67	0,00	1,67
**FBN3**	1,67	0,00	1,67
**MALL**	1,67	0,00	1,67
**SLC25A48**	1,67	0,00	1,67
**GLRA1**	1,67	0,00	1,67
**FAM217A**	1,67	0,00	1,67
**TPBG**	1,67	0,00	1,67
**SHROOM2**	1,67	0,00	1,67
**CPXCR1**	1,67	0,00	1,67
**PRDM14**	1,67	0,00	1,67
**ADAM12**	1,67	0,00	1,67
**ADRA2A**	1,67	0,00	1,67
**FREM2**	1,67	0,00	1,67
**PANX3**	1,67	0,00	1,67
**GPR15**	1,67	0,00	1,67
**SAXO1**	1,67	0,00	1,67

**CFAP161**	1,67	0,00	1,67
**KLHL40**	1,67	0,00	1,67
**C1orf158**	1,67	0,00	1,67
**IL34**	1,67	0,00	1,67
**SVOPL**	1,67	0,00	1,67
**SLC30A2**	1,67	0,00	1,67
**APOA2**	1,67	0,00	1,67
**TNNI1**	1,67	0,00	1,67
**ZDHHC1**	1,67	0,00	1,67
**GJB7**	1,67	0,00	1,67
**SYTL3**	1,67	0,00	1,67
**MAMDC2**	1,67	0,00	1,67
**OR1Q1**	1,67	0,00	1,67
**CLDN3**	1,67	0,00	1,67
**GJB2**	1,67	0,00	1,67
**RNASE7**	1,67	0,00	1,67
**CYP2C19**	1,67	0,00	1,67
**NETO1**	1,67	0,00	1,67
**A2ML1**	1,67	0,00	1,67
**NNMT**	1,67	0,00	1,67
**CACNG2**	1,67	0,00	1,67
**KLK2**	1,67	0,00	1,67
**VXN**	1,67	0,00	1,67
**IL13**	1,67	0,00	1,67
**LINGO1**	1,67	0,00	1,67
**ZPLD1**	1,67	0,00	1,67
**HOXD12**	1,67	0,00	1,67
**CNGB3**	1,67	0,00	1,67
**CDK5R2**	1,67	0,00	1,67
**FGG**	1,67	0,00	1,67
**FGB**	1,67	0,00	1,67
**DNAI2**	1,67	0,00	1,67
**MAL**	1,67	0,00	1,67
**AGXT**	1,67	0,00	1,67
**HES3**	1,67	0,00	1,67
**CD7**	1,67	0,00	1,67
**ANKS4B**	1,67	0,00	1,67
**OR51T1**	1,67	0,00	1,67
**UBE2U**	1,67	0,00	1,67
**ODF3**	1,67	0,00	1,67
**CSRNP3**	1,67	0,00	1,67
**EXD1**	1,67	0,00	1,67
**NRXN1**	1,67	0,00	1,67
**KCNA4**	1,67	0,00	1,67
**NXPH4**	1,67	0,00	1,67
**MAGEB17**	1,67	0,00	1,67
**MCEMP1**	1,67	0,00	1,67
**TACSTD2**	1,67	0,00	1,67
**LRRC19**	1,67	0,00	1,67
**CYP4Z1**	1,67	0,00	1,67
**CYP27C1**	1,67	0,00	1,67
**EMID1**	1,67	0,00	1,67
**FAM9C**	1,67	0,00	1,67
**ZAR1L**	1,67	0,00	1,67
**FAM180A**	1,67	0,00	1,67
**TEX43**	1,67	0,00	1,67

**COL13A1**	1,67	0,00	1,67
**MUC2**	1,67	0,00	1,67
**KHDC3L**	1,67	0,00	1,67
**PCDHA4**	1,67	0,00	1,67
**TTC23L**	1,67	0,00	1,67
**IGLV3-21**	1,67	0,00	1,67
**IGHV3-38**	1,67	0,00	1,67
**IGHV1-58**	1,67	0,00	1,67
**KRT222**	1,67	0,00	1,67
**ZNF705D**	1,67	0,00	1,67
**OR2M3**	1,67	0,00	1,67
**ARHGDIG**	1,67	0,00	1,67
**AC069368.1**	1,67	0,00	1,67
**IGKV1D-39**	1,67	0,00	1,67
**AL110118.1**	1,67	0,00	1,67
**TPBGL**	1,67	0,00	1,67
**CEACAM20**	1,67	0,00	1,67
**KCNE1B**	1,67	0,00	1,67
**IL20RA**	1,67	0,00	1,67
**CHGA**	1,67	0,00	1,67
**CACNG1**	1,67	0,00	1,67
**FGF6**	1,67	0,00	1,67
**IL12B**	1,67	0,00	1,67
**CDH6**	1,67	0,00	1,67
**FGF1**	1,67	0,00	1,67
**SPP1**	1,67	0,00	1,67
**SLPI**	1,67	0,00	1,67
**PCK1**	1,67	0,00	1,67
**SHC2**	1,67	0,00	1,67
**LPIN3**	1,67	0,00	1,67
**CD101**	1,67	0,00	1,67
**NKX2-1**	1,67	0,00	1,67
**ZFP37**	1,67	0,00	1,67
**TRPC3**	1,67	0,00	1,67
**TPH2**	1,67	0,00	1,67
**GABRR1**	1,67	0,00	1,67
**MS4A2**	1,67	0,00	1,67
**HNMT**	1,67	0,00	1,67
**VEGFC**	1,67	0,00	1,67
**TCTEX1D1**	1,67	0,00	1,67
**PID1**	1,67	0,00	1,67
**GPR26**	1,67	0,00	1,67
**SPAG17**	1,67	0,00	1,67
**PCDH19**	1,67	0,00	1,67
**EVA1C**	1,67	0,00	1,67
**FAM71B**	1,67	0,00	1,67
**NDNF**	1,67	0,00	1,67
**VWC2L**	1,67	0,00	1,67
**TSHZ2**	1,67	0,00	1,67
**FOXL2**	1,67	0,00	1,67
**CTAG1B**	1,67	0,00	1,67
**ROBO2**	1,67	0,00	1,67
**ARGFX**	1,67	0,00	1,67
**TMEM262**	1,67	0,00	1,67
**FOXD3**	1,67	0,00	1,67
**COL14A1**	1,67	0,00	1,67

**PPP3R2**	1,67	0,00	1,67
**HRH1**	1,67	0,00	1,67
**TRPC5OS**	1,67	0,00	1,67
**FAM24B**	1,67	0,00	1,67
**FRG2B**	1,67	0,00	1,67
**CTAGE9**	1,67	0,00	1,67
**INMT**	1,67	0,00	1,67
**ACTN3**	1,67	0,00	1,67
**SMIM18**	1,67	0,00	1,67
**CDR1**	1,67	0,00	1,67
**PRRX1**	1,67	0,00	1,67
**PPP4R4**	1,67	0,00	1,67
**FAM83F**	1,67	0,00	1,67
**TDRD9**	1,67	0,00	1,67
**GJB1**	1,67	0,00	1,67
**RGS6**	1,67	0,00	1,67
**TMEM215**	1,67	0,00	1,67
**BEND7**	5,03	3,35	1,68
**TNFRSF13B**	3,29	1,61	1,68
**OPRK1**	2,55	0,87	1,69
**TMEM253**	3,46	1,77	1,69
**PCDHGC4**	7,74	6,05	1,69
**KALRN**	8,08	6,38	1,69
**SELPLG**	3,63	1,93	1,69
**PIK3C2A**	11,18	9,49	1,70
**ZBTB38**	4,76	3,06	1,70
**RNF151**	3,93	2,22	1,70
**ADGRB3**	5,85	4,13	1,72
**POLN**	6,52	4,79	1,72
**PRSS8**	2,58	0,85	1,74
**CBARP**	4,44	2,70	1,74
**IL11RA**	4,89	3,15	1,74
**PTCD3**	14,23	12,48	1,75
**TP63**	6,69	4,94	1,75
**PRRG3**	4,82	3,06	1,76
**AC002996.1**	4,82	3,06	1,76
**CPM**	11,71	9,95	1,76
**NACC2**	3,28	1,50	1,78
**ZCCHC12**	3,28	1,50	1,78
**CALCB**	3,28	1,50	1,78
**IGHV3-66**	3,28	1,50	1,78
**PRCD**	3,28	1,50	1,78
**HIPK4**	3,28	1,50	1,78
**ZDHHC19**	3,28	1,50	1,78
**GZMM**	3,28	1,50	1,78
**AC233723.1**	3,28	1,50	1,78
**H2AC4**	3,28	1,50	1,78
**FBXO27**	5,73	3,94	1,79
**CYP3A4**	2,80	1,01	1,79
**C9orf24**	4,02	2,22	1,80
**C11orf16**	4,36	2,55	1,80
**MAP3K2**	11,72	9,91	1,81
**PLA2G10**	4,51	2,70	1,81
**PRLR**	4,51	2,70	1,81
**TRHR**	4,70	2,89	1,81
**SH2D5**	4,08	2,25	1,82

**AOAH**	4,04	2,21	1,83
**AKAP7**	9,30	7,47	1,83
**ADARB2**	7,82	5,99	1,83
**LRCOL1**	3,44	1,60	1,84
**RBPJL**	3,59	1,74	1,85
**TBC1D2B**	5,90	4,05	1,85
**MAP1LC3A**	2,99	1,11	1,88
**C17orf78**	4,37	2,48	1,88
**TEX54**	4,37	2,48	1,88
**CFAP299**	3,87	1,98	1,88
**RSPH14**	2,69	0,80	1,89
**DCT**	4,12	2,22	1,89
**FGF18**	4,12	2,22	1,89
**PPL**	5,37	3,47	1,90
**ANKRD65**	4,13	2,22	1,91
**KCNH1**	4,76	2,83	1,92
**PRR5-ARHGAP8**	6,29	4,35	1,94
**MANSC1**	5,31	3,36	1,95
**B3GNT8**	5,16	3,21	1,95
**SYNPO2L**	5,21	3,24	1,96
**BCLAF3**	6,11	4,14	1,97
**CHL1**	5,01	3,03	1,97
**F2**	3,52	1,54	1,97
**ARL13A**	2,91	0,94	1,97
**ZNF860**	2,91	0,94	1,97
**ARG1**	2,91	0,94	1,97
**C4orf47**	2,91	0,94	1,97
**SPRR2F**	2,91	0,94	1,97
**AC008073.3**	2,91	0,94	1,97
**OR2L2**	4,20	2,22	1,98
**KCNJ13**	5,05	3,04	2,02
**TRPV1**	8,75	6,72	2,02
**TP53TG5**	4,51	2,48	2,03
**OTUB2**	5,72	3,69	2,03
**AC245033.1**	5,74	3,69	2,04
**RNASE4**	6,22	4,17	2,05
**EML6**	5,57	3,52	2,05
**ROPN1L**	3,44	1,39	2,05
**PCP4L1**	5,12	3,06	2,06
**ZNF560**	4,76	2,70	2,06
**IGHV3-15**	4,29	2,22	2,06
**RP1**	3,57	1,50	2,07
**ASPN**	3,57	1,50	2,07
**ACVR1C**	3,57	1,50	2,07
**CD1A**	3,57	1,50	2,07
**CNMD**	3,57	1,50	2,07
**CLSTN2**	3,57	1,50	2,07
**ANKRD31**	5,06	2,98	2,08
**ANKRD6**	6,71	4,63	2,08
**OASL**	4,34	2,25	2,09
**FCAR**	2,82	0,73	2,09
**ADAMTSL5**	2,82	0,73	2,09
**CRLF1**	2,09	0,00	2,09
**CP**	2,09	0,00	2,09
**RFX4**	2,09	0,00	2,09
**IDO1**	2,09	0,00	2,09

**SYCP3**	2,09	0,00	2,09
**DPT**	2,09	0,00	2,09
**UNC80**	2,09	0,00	2,09
**PSKH2**	2,09	0,00	2,09
**TMEM45B**	2,09	0,00	2,09
**ADGRF1**	2,09	0,00	2,09
**PPM1J**	2,09	0,00	2,09
**KCNJ6**	2,09	0,00	2,09
**CYP11B1**	2,09	0,00	2,09
**ACTG2**	2,09	0,00	2,09
**PPP1R36**	2,09	0,00	2,09
**HTRA4**	2,09	0,00	2,09
**MRAP**	2,09	0,00	2,09
**DSCAML1**	2,09	0,00	2,09
**LCN15**	2,09	0,00	2,09
**ODF3L2**	2,09	0,00	2,09
**OR51B4**	2,09	0,00	2,09
**ACSL5**	2,09	0,00	2,09
**AL662899.1**	2,09	0,00	2,09
**MOG**	2,09	0,00	2,09
**ZNF705G**	2,09	0,00	2,09
**TEX53**	2,09	0,00	2,09
**IGKV2D-30**	2,09	0,00	2,09
**SMIM22**	2,09	0,00	2,09
**CU639417.1**	2,09	0,00	2,09
**FAP**	2,09	0,00	2,09
**GGT5**	2,09	0,00	2,09
**OR7C1**	2,09	0,00	2,09
**SLC14A2**	2,09	0,00	2,09
**CFAP300**	2,09	0,00	2,09
**DUOXA1**	2,09	0,00	2,09
**RAB3C**	2,09	0,00	2,09
**AC005041.1**	2,09	0,00	2,09
**KCNJ3**	2,09	0,00	2,09
**TUBB8**	2,09	0,00	2,09
**SIK1B**	2,09	0,00	2,09
**TCAF2C**	2,09	0,00	2,09
**AC119674.2**	4,58	2,48	2,10
**GDF15**	4,02	1,91	2,11
**SPTY2D1OS**	6,53	4,41	2,12
**ACKR4**	5,33	3,21	2,12
**CLDN7**	3,49	1,36	2,13
**SLC10A1**	4,37	2,22	2,14
**B3GALT2**	4,37	2,22	2,14
**C9orf152**	4,37	2,22	2,14
**TUSC3**	4,69	2,54	2,15
**CIBAR2**	4,64	2,48	2,16
**ADM**	3,36	1,20	2,16
**GJC3**	3,11	0,94	2,17
**AC008676.3**	5,64	3,47	2,17
**ROPN1B**	3,17	1,00	2,17
**FXYD6**	3,18	1,00	2,18
**SLC9A2**	3,70	1,50	2,20
**MMP8**	3,70	1,50	2,20
**TCF7**	5,02	2,82	2,20
**CARD16**	5,41	3,21	2,20

**SLC4A9**	4,12	1,91	2,21
**AC006064.6**	10,75	8,54	2,21
**SAMD13**	4,59	2,37	2,22
**PTGIS**	6,19	3,97	2,22
**ALDH3B2**	3,21	0,98	2,23
**JMJD7-PLA2G4B**	7,88	5,64	2,23
**PLEKHS1**	4,63	2,38	2,24
**PIK3R5**	8,62	6,34	2,28
**ZNF566**	8,91	6,62	2,29
**CFH**	4,51	2,22	2,29
**TAS2R42**	4,51	2,22	2,29
**TRBV20-1**	4,51	2,22	2,29
**KLF7**	4,23	1,93	2,29
**INHA**	3,82	1,50	2,31
**SLC5A2**	3,82	1,50	2,31
**SP7**	3,82	1,50	2,31
**PHLDA2**	3,82	1,50	2,31
**IGLV2-14**	3,82	1,50	2,31
**PDZD3**	3,14	0,82	2,32
**POTEM**	5,46	3,13	2,33
**VWA3A**	5,10	2,75	2,35
**HNF4G**	4,86	2,51	2,35
**SULT1A3**	10,30	7,95	2,35
**RNASEK-C17orf49**	4,58	2,22	2,35
**NR2E3**	3,30	0,93	2,36
**METTL27**	4,36	1,95	2,41
**FABP2**	3,92	1,50	2,42
**TAS2R30**	3,92	1,50	2,42
**AC112229.3**	3,92	1,50	2,42
**THSD8**	3,92	1,50	2,42
**FYB1**	2,42	0,00	2,42
**TRPM1**	2,42	0,00	2,42
**CALCR**	2,42	0,00	2,42
**ITIH1**	2,42	0,00	2,42
**NPFFR2**	2,42	0,00	2,42
**SULT2B1**	2,42	0,00	2,42
**DSP**	2,42	0,00	2,42
**PVALB**	2,42	0,00	2,42
**FOXN1**	2,42	0,00	2,42
**PACRG**	2,42	0,00	2,42
**TAF1L**	2,42	0,00	2,42
**ANGPTL3**	2,42	0,00	2,42
**HNF1A**	2,42	0,00	2,42
**GMPR**	2,42	0,00	2,42
**POU2F3**	2,42	0,00	2,42
**RLBP1**	2,42	0,00	2,42
**SLC22A3**	2,42	0,00	2,42
**KCNK13**	2,42	0,00	2,42
**CLDN14**	2,42	0,00	2,42
**SLC13A4**	2,42	0,00	2,42
**LDHC**	2,42	0,00	2,42
**SAMD14**	2,42	0,00	2,42
**ENTPD3**	2,42	0,00	2,42
**NIPAL4**	2,42	0,00	2,42
**URAD**	2,42	0,00	2,42
**C12orf56**	2,42	0,00	2,42

**PLEKHG7**	2,42	0,00	2,42
**MYMK**	2,42	0,00	2,42
**KBTBD12**	2,42	0,00	2,42
**CCDC151**	2,42	0,00	2,42
**MB**	2,42	0,00	2,42
**PLN**	2,42	0,00	2,42
**CR1**	2,42	0,00	2,42
**TRGV8**	2,42	0,00	2,42
**SMCO1**	2,42	0,00	2,42
**CKMT1B**	2,42	0,00	2,42
**PTTG2**	2,42	0,00	2,42
**HPR**	2,42	0,00	2,42
**DPEP2NB**	2,42	0,00	2,42
**H2AB1**	2,42	0,00	2,42
**GPIHBP1**	2,42	0,00	2,42
**FGR**	2,42	0,00	2,42
**ACP3**	2,42	0,00	2,42
**TNIP3**	2,42	0,00	2,42
**ASIC4**	2,42	0,00	2,42
**PRODH**	2,42	0,00	2,42
**WFDC2**	2,42	0,00	2,42
**TLR8**	2,42	0,00	2,42
**IL17F**	2,42	0,00	2,42
**WNT5A**	2,42	0,00	2,42
**PAPPA2**	2,42	0,00	2,42
**FBXO2**	2,42	0,00	2,42
**OCM**	2,42	0,00	2,42
**MOGAT1**	2,42	0,00	2,42
**IRGC**	2,42	0,00	2,42
**GRM4**	2,42	0,00	2,42
**DPP6**	2,42	0,00	2,42
**TRIM54**	2,42	0,00	2,42
**MSLNL**	2,42	0,00	2,42
**FGA**	2,42	0,00	2,42
**LINGO2**	2,42	0,00	2,42
**THBD**	2,42	0,00	2,42
**GBP6**	2,42	0,00	2,42
**TAFA3**	2,42	0,00	2,42
**TMPRSS11A**	2,42	0,00	2,42
**AL162231.1**	2,42	0,00	2,42
**PRELP**	2,42	0,00	2,42
**S100A5**	2,42	0,00	2,42
**DIO3**	2,42	0,00	2,42
**PEG3**	2,42	0,00	2,42
**TMEM213**	2,42	0,00	2,42
**TSPAN19**	2,42	0,00	2,42
**GPR162**	2,42	0,00	2,42
**TMEM200B**	2,42	0,00	2,42
**AC025263.2**	2,42	0,00	2,42
**GH1**	2,42	0,00	2,42
**ANTXRL**	2,42	0,00	2,42
**DNAH5**	2,42	0,00	2,42
**ITK**	2,42	0,00	2,42
**SH3TC2**	2,42	0,00	2,42
**ESRRG**	2,42	0,00	2,42
**BCAS1**	4,06	1,63	2,42

**MFSD2B**	3,21	0,79	2,43
**RGS22**	5,82	3,36	2,46
**CACNA1E**	5,15	2,69	2,46
**BMPR1B**	3,38	0,89	2,49
**ATP1B2**	4,39	1,89	2,49
**SPOCK1**	4,02	1,50	2,52
**CALN1**	4,02	1,50	2,52
**CD14**	5,01	2,49	2,53
**AP000295.1**	6,31	3,77	2,53
**H3C2**	4,76	2,22	2,54
**ZNF521**	5,12	2,58	2,55
**CA14**	4,09	1,52	2,57
**PRG4**	5,29	2,70	2,59
**LZTS3**	5,33	2,70	2,63
**AC098650.1**	3,57	0,94	2,63
**PRL**	4,76	2,12	2,64
**TCAF2**	6,89	4,25	2,64
**FAM178B**	5,26	2,61	2,65
**CCND1**	4,58	1,91	2,67
**ARSL**	8,65	5,97	2,68
**BICDL2**	2,69	0,00	2,69
**KIAA2012**	2,69	0,00	2,69
**AIFM3**	2,69	0,00	2,69
**AC106873.8**	2,69	0,00	2,69
**DKK3**	2,69	0,00	2,69
**OVOL3**	2,69	0,00	2,69
**RBP2**	2,69	0,00	2,69
**CKMT2**	2,69	0,00	2,69
**ZNF483**	2,69	0,00	2,69
**AC005324.3**	2,69	0,00	2,69
**AC235565.2**	2,69	0,00	2,69
**ATP2B3**	2,69	0,00	2,69
**SLITRK5**	2,69	0,00	2,69
**ZACN**	2,69	0,00	2,69
**ZNF729**	2,69	0,00	2,69
**MIA**	2,69	0,00	2,69
**SGCZ**	3,51	0,82	2,69
**XIRP1**	4,20	1,50	2,70
**AC010325.1**	3,60	0,85	2,75
**KIF6**	6,55	3,79	2,76
**EIF3CL**	10,89	8,13	2,76
**ABI3BP**	5,33	2,56	2,76
**GNG2**	11,10	8,28	2,82
**HOXA3**	5,09	2,26	2,83
**ZSWIM4**	6,07	3,24	2,84
**REXO5**	4,52	1,68	2,84
**SLC6A11**	4,19	1,33	2,86
**EXOC6**	11,52	8,65	2,86
**WDR49**	4,83	1,97	2,87
**CACNA1F**	3,82	0,94	2,88
**ZBTB8B**	3,82	0,94	2,88
**KCNJ15**	5,73	2,84	2,89
**MYO16**	2,91	0,00	2,91
**MYH13**	2,91	0,00	2,91
**ACHE**	2,91	0,00	2,91
**HPN**	2,91	0,00	2,91

**CD5**	2,91	0,00	2,91
**GCNT2**	2,91	0,00	2,91
**SPART**	2,91	0,00	2,91
**DNAJC5B**	2,91	0,00	2,91
**CXCR1**	2,91	0,00	2,91
**GPX6**	2,91	0,00	2,91
**LRRC10B**	2,91	0,00	2,91
**C18orf63**	2,91	0,00	2,91
**IGLV4-3**	2,91	0,00	2,91
**TRGV1**	2,91	0,00	2,91
**MFRP**	2,91	0,00	2,91
**CADPS2**	2,91	0,00	2,91
**SEMA5B**	2,91	0,00	2,91
**GRAP2**	2,91	0,00	2,91
**PLCL1**	2,91	0,00	2,91
**TNR**	2,91	0,00	2,91
**EPCAM**	2,91	0,00	2,91
**C3**	2,91	0,00	2,91
**DOCK6**	2,91	0,00	2,91
**CYP2J2**	2,91	0,00	2,91
**LCA5**	2,91	0,00	2,91
**LYPD1**	2,91	0,00	2,91
**ASGR2**	2,91	0,00	2,91
**DIRAS3**	2,91	0,00	2,91
**CLDN19**	2,91	0,00	2,91
**BEAN1**	2,91	0,00	2,91
**SCUBE2**	2,91	0,00	2,91
**LRRN1**	2,91	0,00	2,91
**NHLH2**	2,91	0,00	2,91
**OR52N5**	2,91	0,00	2,91
**CCDC172**	2,91	0,00	2,91
**KCTD8**	2,91	0,00	2,91
**SPATA12**	2,91	0,00	2,91
**ADRB3**	2,91	0,00	2,91
**PABPN1L**	2,91	0,00	2,91
**CYP19A1**	2,91	0,00	2,91
**ABCA6**	4,44	1,50	2,94
**ZNF257**	4,21	1,25	2,96
**MARVELD2**	5,33	2,34	2,99
**RAB18**	10,23	7,23	3,01
**AVIL**	4,51	1,50	3,01
**SCN1A**	4,51	1,50	3,01
**CGNL1**	5,25	2,22	3,03
**NANOG**	3,11	0,00	3,11
**WFDC8**	3,11	0,00	3,11
**SCRG1**	3,11	0,00	3,11
**P2RY12**	3,11	0,00	3,11
**AL109827.1**	3,11	0,00	3,11
**H4C4**	3,11	0,00	3,11
**AC010531.1**	3,11	0,00	3,11
**ATXN7L2**	3,11	0,00	3,11
**P2RY13**	3,11	0,00	3,11
**CLDN24**	3,11	0,00	3,11
**SMAD7**	6,65	3,46	3,19
**MCU**	4,99	1,78	3,20
**AC004832.3**	4,76	1,50	3,26

**AC011330.3**	4,76	1,50	3,26
**MAMLD1**	3,28	0,00	3,28
**CHRND**	3,28	0,00	3,28
**SCN2B**	3,28	0,00	3,28
**BEX1**	3,28	0,00	3,28
**IFNB1**	3,28	0,00	3,28
**HIGD2B**	3,28	0,00	3,28
**MUC12**	3,28	0,00	3,28
**IGLV4-60**	3,28	0,00	3,28
**PCDHB7**	3,28	0,00	3,28
**FBXO40**	3,28	0,00	3,28
**NLRP11**	3,28	0,00	3,28
**OPCML**	3,28	0,00	3,28
**OR7A5**	3,28	0,00	3,28
**ARPIN**	3,28	0,00	3,28
**INMT-MINDY4**	3,28	0,00	3,28
**AL359736.1**	3,28	0,00	3,28
**AL031681.2**	3,28	0,00	3,28
**PTPN21**	4,48	1,09	3,39
**GPR161**	7,86	4,46	3,40
**AJAP1**	4,50	1,08	3,43
**ACE2**	3,43	0,00	3,43
**FOXF2**	3,43	0,00	3,43
**MALRD1**	3,43	0,00	3,43
**CHDH**	3,43	0,00	3,43
**MISP3**	3,43	0,00	3,43
**H4C13**	3,43	0,00	3,43
**DTNA**	6,02	2,57	3,45
**RASSF8**	6,23	2,78	3,45
**AP001267.5**	7,53	3,99	3,55
**HECW2**	4,54	0,99	3,55
**HYDIN**	6,97	3,42	3,56
**EHD2**	3,57	0,00	3,57
**WNT4**	3,57	0,00	3,57
**AD000671.1**	3,57	0,00	3,57
**ARHGAP19-SLIT1**	3,57	0,00	3,57
**GAS2L2**	3,57	0,00	3,57
**AC114982.2**	3,57	0,00	3,57
**STON1-GTF2A1L**	3,57	0,00	3,57
**LAMA4**	3,57	0,00	3,57
**SLC8A2**	3,57	0,00	3,57
**OVOL2**	3,57	0,00	3,57
**SCN2A**	3,57	0,00	3,57
**ERMN**	3,57	0,00	3,57
**BCL2L10**	3,57	0,00	3,57
**CD200R1**	3,57	0,00	3,57
**GASK1B**	3,57	0,00	3,57
**CXCL8**	3,57	0,00	3,57
**GPR34**	3,57	0,00	3,57
**PAX9**	3,57	0,00	3,57
**OR13C2**	3,57	0,00	3,57
**CCDC68**	4,37	0,77	3,60
**OR2B11**	5,55	1,91	3,64
**NTN4**	4,46	0,82	3,64
**TRIB3**	5,83	2,06	3,77
**TEKT1**	5,04	1,25	3,79

**PDGFRA**	3,82	0,00	3,82
**NPTX2**	3,82	0,00	3,82
**KLK13**	3,82	0,00	3,82
**GIMAP5**	3,82	0,00	3,82
**SPATA6L**	3,82	0,00	3,82
**CCL14**	3,82	0,00	3,82
**ADORA3**	3,82	0,00	3,82
**AC099811.2**	5,33	1,50	3,83
**KIF5A**	4,79	0,93	3,86
**MYO10**	5,17	1,31	3,86
**ZNF511-PRAP1**	3,92	0,00	3,92
**SNAP91**	4,02	0,00	4,02
**IL36G**	4,02	0,00	4,02
**SLC6A1**	4,02	0,00	4,02
**GLDN**	4,02	0,00	4,02
**NLRP2B**	4,02	0,00	4,02
**OR2A1**	4,02	0,00	4,02
**PRR20G**	4,02	0,00	4,02
**SYS1-DBNDD2**	4,02	0,00	4,02
**CFAP206**	4,02	0,00	4,02
**PLXNA4**	4,02	0,00	4,02
**TTLL6**	4,12	0,00	4,12
**AL021546.1**	4,12	0,00	4,12
**UBE2F-SCLY**	4,12	0,00	4,12
**AC004223.3**	4,12	0,00	4,12
**SULF2**	5,64	1,50	4,14
**DLL1**	4,20	0,00	4,20
**MUSTN1**	4,20	0,00	4,20
**MYO3B**	4,20	0,00	4,20
**AC008758.5**	4,20	0,00	4,20
**OSR2**	4,37	0,00	4,37
**MAGED4**	4,37	0,00	4,37
**UMODL1**	4,37	0,00	4,37
**TEN1-CDK3**	4,37	0,00	4,37
**AC007687.1**	4,44	0,00	4,44
**NRIP3**	4,44	0,00	4,44
**ANO7**	4,51	0,00	4,51
**AC110275.1**	4,51	0,00	4,51
**AHRR**	5,78	1,26	4,52
**NOTCH2NLR**	4,58	0,00	4,58
**ANGPTL6**	4,64	0,00	4,64
**FGF5**	4,64	0,00	4,64
**PXDNL**	4,64	0,00	4,64
**RGPD1**	4,64	0,00	4,64
**AC120057.2**	4,70	0,00	4,70
**SPECC1L-ADORA2A**	7,71	2,89	4,82
**ZFP91-CNTF**	6,60	1,75	4,85
**KIAA1671**	4,93	0,00	4,93
**AC009163.3**	5,03	0,00	5,03
**SPATA1**	5,21	0,00	5,21
**AC139530.2**	5,25	0,00	5,25
**PRSS53**	5,44	0,00	5,44
**AC097637.1**	5,48	0,00	5,48
**AC068234.1**	6,00	0,00	6,00
**AC099850.2**	6,10	0,00	6,10
**AC104472.3**	6,29	0,00	6,29

**P3R3URF-PIK3R3**	6,39	0,00	6,39
**IGLC1**	6,53	0,00	6,53
**ARHGAP20**	7,75	0,00	7,75
**CHIC1**	7,80	0,00	7,80
**CECR2**	8,60	0,00	8,60
