# Supplementary material for: NKX6-3 in B-Cell Progenitor Differentiation and Leukemia
Source: Genes (Basel). 2025 Oct 14;16(10):1199. doi: 10.3390/genes16101199 (PMC12563489; doi:10.3390/genes16101199)
Supplement: Supplementary file 1 [file genes-16-01199-s001.zip › Supplemental Figures 1 and 2.pdf]

fastp report <https://github.com/OpenGene/fastp>
Summary
General
fastp version: 0.23.4 (https://github.com/OpenGene/fastp <https://github.com/OpenGene/fastp>)
sequencing: paired end (151 cycles + 151 cycles)
mean length before filtering: 148bp, 148bp
mean length after filtering: 148bp, 147bp
duplication rate: 21.080915%
Insert size peak: 205

Before filtering
total reads: 105.224312 M
total bases: 15.589329 G
Q20 bases: 15.393529 G (98.744011%)
Q30 bases: 14.948972 G (95.892333%)
GC content: 47.931888%

After filtering
total reads: 104.135950 M
total bases: 15.409465 G
Q20 bases: 15.277961 G (99.146601%)
Q30 bases: 14.875898 G (96.537410%)
GC content: 47.888298%

Filtering result
reads passed filters: 104.135950 M (98.965674%)
reads corrected: 5.721254 M (5.437198%)
bases corrected: 10.115539 M (0.064888%)
reads with low quality: 1.004808 M (0.954920%)
reads with too many N: 83.554000 K (0.079406%)
reads too short: 0 (0.000000%)

Adapters
Adapter or bad ligation of read1
The input has little adapter percentage (~0.046026%), probably it's trimmed before.
Sequence Occurrences
all adapter sequences 77210

Adapter or bad ligation of read2
The input has little adapter percentage (~0.180668%), probably it's trimmed before.
Sequence Occurrences
AGATCGGAAGAGCGTCGTGGGGGGG 10253
AGATCGGAAGAGCGTCGTGGGGGGGA 8151
other adapter sequences 371732

Insert size estimation
05010015020025000.10.20.30.4

Insert size distribution (38.209788% reads are with unknown length)Insert sizeRead percent (%)

<https://plot.ly/>
This estimation is based on paired-end overlap analysis, and there are 38.209788% reads found not overlapped.
The nonoverlapped read pairs may have insert size <30 or >272, or

contain too much sequencing errors to be detected as overlapped.
Before filtering
Before filtering: read1: quality
Value of each position will be shown on mouse over.
20406080100120140 37.538 38.539 39.5

positionqualityATCGmean

<https://plot.ly/>
Before filtering: read1: base contents
Value of each position will be shown on mouse over.
204060801001201400 0.10.20.30.40.50.60.7

positionbase content ratiosA(25.24%)T(27.15%)C(23.94%)G(23.56%)N(0.087%)GC(47.51%)

<https://plot.ly/>
Before filtering: read1: KMER counting
Darker background means larger counts. The count will be shown on mouse over.

AA AT AC AG TA TT TC TG CA CT CC CG GA GT GC GG
AAA AAAAA AAAAT AAAAC AAAAG AAATA AAATT AAATC AAATG AAACA AAACT AAACC
AAACG AAAGA AAAGT AAAGC AAAGG
AAT AATAA AATAT AATAC AATAG AATTA AATTT AATTC AATTG AATCA AATCT AATCC
AATCG AATGA AATGT AATGC AATGG
AAC AACAA AACAT AACAC AACAG AACTA AACTT AACTC AACTG AACCA AACCT AACCC
AACCG AACGA AACGT AACGC AACGG
AAG AAGAA AAGAT AAGAC AAGAG AAGTA AAGTT AAGTC AAGTG AAGCA AAGCT AAGCC
AAGCG AAGGA AAGGT AAGGC AAGGG
ATA ATAAA ATAAT ATAAC ATAAG ATATA ATATT ATATC ATATG ATACA ATACT ATACC
ATACG ATAGA ATAGT ATAGC ATAGG
ATT ATTAA ATTAT ATTAC ATTAG ATTTA ATTTT ATTTC ATTTG ATTCA ATTCT ATTCC
ATTCG ATTGA ATTGT ATTGC ATTGG
ATC ATCAA ATCAT ATCAC ATCAG ATCTA ATCTT ATCTC ATCTG ATCCA ATCCT ATCCC
ATCCG ATCGA ATCGT ATCGC ATCGG
ATG ATGAA ATGAT ATGAC ATGAG ATGTA ATGTT ATGTC ATGTG ATGCA ATGCT ATGCC
ATGCG ATGGA ATGGT ATGGC ATGGG
ACA ACAAA ACAAT ACAAC ACAAG ACATA ACATT ACATC ACATG ACACA ACACT ACACC
ACACG ACAGA ACAGT ACAGC ACAGG
ACT ACTAA ACTAT ACTAC ACTAG ACTTA ACTTT ACTTC ACTTG ACTCA ACTCT ACTCC
ACTCG ACTGA ACTGT ACTGC ACTGG
ACC ACCAA ACCAT ACCAC ACCAG ACCTA ACCTT ACCTC ACCTG ACCCA ACCCT ACCCC
ACCCG ACCGA ACCGT ACCGC ACCGG
ACG ACGAA ACGAT ACGAC ACGAG ACGTA ACGTT ACGTC ACGTG ACGCA ACGCT ACGCC
ACGCG ACGGA ACGGT ACGGC ACGGG
AGA AGAAA AGAAT AGAAC AGAAG AGATA AGATT AGATC AGATG AGACA AGACT AGACC
AGACG AGAGA AGAGT AGAGC AGAGG

AGT AGTAA AGTAT AGTAC AGTAG AGTTA AGTTT AGTTC AGTTG AGTCA AGTCT AGTCC
AGTCG AGTGA AGTGT AGTGC AGTGG
AGC AGCAA AGCAT AGCAC AGCAG AGCTA AGCTT AGCTC AGCTG AGCCA AGCCT AGCCC
AGCCG AGCGA AGCGT AGCGC AGCGG
AGG AGGAA AGGAT AGGAC AGGAG AGGTA AGGTT AGGTC AGGTG AGGCA AGGCT AGGCC
AGGCG AGGGA AGGGT AGGGC AGGGG
TAA TAAAA TAAAT TAAAC TAAAG TAATA TAATT TAATC TAATG TAACA TAACT TAACC
TAACG TAAGA TAAGT TAAGC TAAGG
TAT TATAA TATAT TATAC TATAG TATTA TATTT TATTC TATTG TATCA TATCT TATCC
TATCG TATGA TATGT TATGC TATGG
TAC TACAA TACAT TACAC TACAG TACTA TACTT TACTC TACTG TACCA TACCT TACCC
TACCG TACGA TACGT TACGC TACGG
TAG TAGAA TAGAT TAGAC TAGAG TAGTA TAGTT TAGTC TAGTG TAGCA TAGCT TAGCC
TAGCG TAGGA TAGGT TAGGC TAGGG
TTA TTAAA TTAAT TTAAC TTAAG TTATA TTATT TTATC TTATG TTACA TTACT TTACC
TTACG TTAGA TTAGT TTAGC TTAGG
TTT TTTAA TTTAT TTTAC TTTAG TTTTA TTTTT TTTTC TTTTG TTTCA TTTCT TTTCC
TTTCG TTTGA TTTGT TTTGC TTTGG
TTC TTCAA TTCAT TTCAC TTCAG TTCTA TTCTT TTCTC TTCTG TTCCA TTCCT TTCCC
TTCCG TTCGA TTCGT TTCGC TTCGG
TTG TTGAA TTGAT TTGAC TTGAG TTGTA TTGTT TTGTC TTGTG TTGCA TTGCT TTGCC
TTGCG TTGGA TTGGT TTGGC TTGGG
TCA TCAAA TCAAT TCAAC TCAAG TCATA TCATT TCATC TCATG TCACA TCACT TCACC
TCACG TCAGA TCAGT TCAGC TCAGG
TCT TCTAA TCTAT TCTAC TCTAG TCTTA TCTTT TCTTC TCTTG TCTCA TCTCT TCTCC
TCTCG TCTGA TCTGT TCTGC TCTGG
TCC TCCAA TCCAT TCCAC TCCAG TCCTA TCCTT TCCTC TCCTG TCCCA TCCCT TCCCC
TCCCG TCCGA TCCGT TCCGC TCCGG
TCG TCGAA TCGAT TCGAC TCGAG TCGTA TCGTT TCGTC TCGTG TCGCA TCGCT TCGCC
TCGCG TCGGA TCGGT TCGGC TCGGG
TGA TGAAA TGAAT TGAAC TGAAG TGATA TGATT TGATC TGATG TGACA TGACT TGACC
TGACG TGAGA TGAGT TGAGC TGAGG
TGT TGTAA TGTAT TGTAC TGTAG TGTTA TGTTT TGTTC TGTTG TGTCA TGTCT TGTCC
TGTCG TGTGA TGTGT TGTGC TGTGG
TGC TGCAA TGCAT TGCAC TGCAG TGCTA TGCTT TGCTC TGCTG TGCCA TGCCT TGCCC
TGCCG TGCGA TGCGT TGCGC TGCGG
TGG TGGAA TGGAT TGGAC TGGAG TGGTA TGGTT TGGTC TGGTG TGGCA TGGCT TGGCC
TGGCG TGGGA TGGGT TGGGC TGGGG
CAA CAAAA CAAAT CAAAC CAAAG CAATA CAATT CAATC CAATG CAACA CAACT CAACC
CAACG CAAGA CAAGT CAAGC CAAGG
CAT CATAA CATAT CATAC CATAG CATTA CATTT CATTC CATTG CATCA CATCT CATCC
CATCG CATGA CATGT CATGC CATGG
CAC CACAA CACAT CACAC CACAG CACTA CACTT CACTC CACTG CACCA CACCT CACCC
CACCG CACGA CACGT CACGC CACGG

CAG CAGAA CAGAT CAGAC CAGAG CAGTA CAGTT CAGTC CAGTG CAGCA CAGCT
CAGCC
CAGCG CAGGA CAGGT CAGGC CAGGG
CTA CTAAA CTAAT CTAAC CTAAG CTATA CTATT CTATC CTATG CTACA CTACT
CTACC
CTACG CTAGA CTAGT CTAGC CTAGG
CTT CTTAA CTTAT CTTAC CTTAG CTTTA CTTTT CTTTC CTTTG CTTCA CTTCT CTTCC
CTTCG CTTGA CTTGT CTTGC CTTGG
CTC CTCAA CTCAT CTCAC CTCAG CTCTA CTCTT CTCTC CTCTG CTCCA CTCCT CTCCC
CTCCG CTCGA CTCGT CTCGC CTCGG
CTG CTGAA CTGAT CTGAC CTGAG CTGTA CTGTT CTGTC CTGTG CTGCA CTGCT
CTGCC
CTGCG CTGGA CTGGT CTGGC CTGGG
CCA CCAAA CCAAT CCAAC CCAAG CCATA CCATT CCATC CCATG CCACA CCACT
CCACC
CCACG CCAGA CCAGT CCAGC CCAGG
CCT CCTAA CCTAT CCTAC CCTAG CCTTA CCTTT CCTTC CCTTG CCTCA CCTCT CCTCC
CCTCG CCTGA CCTGT CCTGC CCTGG
CCC CCCAA CCCAT CCCAC CCCAG CCCTA CCCTT CCCTC CCCTG CCCCA CCCCT
CCCCC
CCCCG CCCGA CCCGT CCCGC CCCGG
CCG CCGAA CCGAT CCGAC CCGAG CCGTA CCGTT CCGTC CCGTG CCGCA CCGCT
CCGCC
CCGCG CCGGA CCGGT CCGGC CCGGG
CGA CGAAA CGAAT CGAAC CGAAG CGATA CGATT CGATC CGATG CGACA CGACT
CGACC
CGACG CGAGA CGAGT CGAGC CGAGG
CGT CGTAA CGTAT CGTAC CGTAG CGTTA CGTTT CGTTC CGTTG CGTCA CGTCT
CGTCC
CGTCG CGTGA CGTGT CGTGC CGTGG
CGC CGCAA CGCAT CGCAC CGCAG CGCTA CGCTT CGCTC CGCTG CGCCA CGCCT
CGCCC
CGCCG CGCGA CGCGT CGCGC CGCGG
CGG CGGAA CGGAT CGGAC CGGAG CGGTA CGGTT CGGTC CGGTG CGGCA CGGCT
CGGCC
CGGCG CGGGA CGGGT CGGGC CGGGG
GAA GAAAA GAAAT GAAAC GAAAG GAATA GAATT GAATC GAATG GAACA GAACT
GAACC
GAACG GAAGA GAAGT GAAGC GAAGG
GAT GATAA GATAT GATAC GATAG GATTA GATTT GATTC GATTG GATCA GATCT
GATCC
GATCG GATGA GATGT GATGC GATGG
GAC GACAA GACAT GACAC GACAG GACTA GACTT GACTC GACTG GACCA GACCT
GACCC
GACCG GACGA GACGT GACGC GACGG
GAG GAGAA GAGAT GAGAC GAGAG GAGTA GAGTT GAGTC GAGTG GAGCA GAGCT
GAGCC
GAGCG GAGGA GAGGT GAGGC GAGGG
GTA GTAAA GTAAT GTAAC GTAAG GTATA GTATT GTATC GTATG GTACA GTACT
GTACC
GTACG GTAGA GTAGT GTAGC GTAGG
GTT GTTAA GTTAT GTTAC GTTAG GTTTA GTTTT GTTTC GTTTG GTTCA GTTCT GTTCC
GTTCG GTTGA GTTGT GTTGC GTTGG
GTC GTCAA GTCAT GTCAC GTCAG GTCTA GTCTT GTCTC GTCTG GTCCA GTCCT
GTCCC
GTCCG GTCGA GTCGT GTCGC GTCGG
GTG GTGAA GTGAT GTGAC GTGAG GTGTA GTGTT GTGTC GTGTG GTGCA GTGCT
GTGCC

GTGCG GTGGA GTGGT GTGGC GTGGG
GCA GCAAA GCAAT GCAAC GCAAG GCATA GCATT GCATC GCATG GCACA GCACT
GCACC
GCACG GCAGA GCAGT GCAGC GCAGG
GCT GCTAA GCTAT GCTAC GCTAG GCTTA GCTTT GCTTC GCTTG GCTCA GCTCT
GCTCC
GCTCG GCTGA GCTGT GCTGC GCTGG
GCC GCCAA GCCAT GCCAC GCCAG GCCTA GCCTT GCCTC GCCTG GCCCA GCCCT
GCCCC
GCCCG GCCGA GCCGT GCCGC GCCGG
GCG GCGAA GCGAT GCGAC GCGAG GCGTA GCGTT GCGTC GCGTG GCGCA GCGCT
GCGCC
GCGCG GCGGA GCGGT GCGGC GCGGG
GGA GGAAA GGAAT GGAAC GGAAG GGATA GGATT GGATC GGATG GGACA GGACT
GGACC
GGACG GGAGA GGAGT GGAGC GGAGG
GGT GGTAA GGTAT GGTAC GGTAG GGTTA GGTTT GGTTC GGTTG GGTCA GGTCT
GGTCC
GGTCG GGTGA GGTGT GGTGC GGTGG
GGC GGCAA GGCAT GGCAC GGCAG GGCTA GGCTT GGCTC GGCTG GGCCA GGCCT
GGCCC
GGCCG GGCGA GGCGT GGCGC GGCGG
GGG GGGAA GGGAT GGGAC GGGAG GGGTA GGGTT GGGTC GGGTG GGGCA GGGCT
GGGCC
GGGCG GGGGA GGGGT GGGGC GGGGG

Before filtering: read1: overrepresented sequences
Sampling rate: 1 / 20
overrepresented sequence count (% of bases) distribution: cycle 1 ~
cycle 151
AAAAAAAAAAAAAAAAAAAAAAAAAAAAAAAAAAAAAAAA 3397 (0.034880%)

AAAATTCTGTGACAAATTTTTGGTCAAGTTGTTTCCATTAAAAGTACTGATTTTAAAAACTAATAACTT
AAAACTGCCACACGCAAAAAAGAAAACCAA 43 (0.001104%)

AAACTTGCATGCAATGTGAGCCGTGTGGCAATCCAATACAGGGGCATAGCCGGCGCTTATTTGGCCTGG
ATGGTTCAGGATAATCACCTGAGCAGTGAAG 4 (0.000103%)

AAAGTGAACAGATATTCAGCATCTAACAGTTCAAAAGAAGCCACTACATACTCTTTTCACAAATATGTT
TTCACAGAGCCAATACAGTACTAGCCATTAA 3 (0.000077%)

AAATATGTAGAGGGAGTATAGGGCTGTGACTAGTATGTTGAGTCCTGTAAGTAGGAGAGTGATATTTGA
TCAGGAGAACGTGGTTACTAGCACAGAGAGT 6 (0.000154%)

AAATTCTGTGACAAATTTTTGGTCAAGTTGTTTCCATTAAAAGTACTGATTTTAAAAACTAATAACTTA
AAACTGCCACACGCAAAAAAGAAAACCAAA 14 (0.000359%)

AAATTTCCACAAGGAGATATCAATGGTGATACCACGTTCACGCTCAGCTTTCAGTTTATCCAAGACCCA
GGCATACTTGAAGGAGCCCTTTCCCATCTCA 4 (0.000103%)

AAATTTTTCAATGGTTCTTTTGTCGATGCCACCGCATTTATAGATCAGATGGCCAGTAGTGGTGGACTTG
CCCGAATCTACGTGTCCAATGACGACAATG 17 (0.000436%)

AAATTTTTGGTCAAGTTGTTTCCATTAAAAGTACTGATTTTAAAAACTAATAACTTAAAACTGCCACAC
GCAAAAAAGAAAACCAAAGTGGTCCACAAA 14 (0.000359%)

AACAATGGCAGCATCACCAGACTTCAAGAATTTAGGGCCATCTTCCAGCTTTTTACCAGAACGGCGATC
AATCTTTTCCTTCAGCTCAGCAAACTTGCAT 5 (0.000128%)

AACAATTTCCTCATATCTCTTCTGGCTGTAGGGTGGCTCAGTGGAATCCATTTTGTTAACACCGACAATT
AGTTGTTTCACACCCAGTGTGTAAGCCAGA 10 (0.000257%)

AACAGATATTCAGCATCTAACAGTTCAAAAGAAGCCACTACATACTCTTTTCACAAATATGTTTTCACA
GAGCCAATACAGTACTAGCCATTAACCCAGT 4 (0.000103%)

AACCAGTCTTTTACTACTAAACTTAAATGGCCAATTGAAACAAACAGTTCTGAGACCGTTCTTCCACCA
CTGATTAAGAGTGGGGTGGCAGGTATTAGGG 6 (0.000154%)

AACCATATCAACAATGGCAGCATCACCAGACTTCAAGAATTTAGGGCCATCTTCCAGCTTTTTACCAGA
ACGGCGATCAATCTTTTCCTTCAGCTCAGCA 29 (0.000744%)

AAGAATTTAGGGCCATCTTCCAGCTTTTTACCAGAACGGCGATCAATCTTTTCCTTCAGCTCAGCAAACT
TGCATGCAATGTGAGCCGTGTGGCAATCCA 6 (0.000154%)

AAGTGCTGACTTCCTTAACAATTTCCTCATATCTCTTCTGGCTGTAGGGTGGCTCAGTGGAATCCATTTT
GTTAACACCGACAATTAGTTGTTTCACACC 3 (0.000077%)

AAGTTGTTTCCATTAAAAAGTACTGATTTTAAAAACTAATAACTTAAAACTGCCACACGCAAAAAAGAA
AACCAAAGTGGTCCACAAAACATTCTCCTTT 7 (0.000180%)

AATATGTAGAGGGAGTATAGGGCTGTGACTAGTATGTTGAGTCCTGTAAGTAGGAGAGTGATATTTGAT
CAGGAGAACGTGGTTACTAGCACAGAGAGTT 11 (0.000282%)

AATATTCATTTAGCCTTCTGAGCTTTCTGGGCAGACTTGGTGACCTTGCCAGCTCCAGCAGCCTTCTTGT
CCACTGCTTTGATGACACCCACCGCAACTG 9 (0.000231%)

AATCACCTGAGCAGTGAAGCCAGCTGCTTCCATTGGTGGGTCATTTTTGCTGTCACCAGCAACGTTGCC
ACGACGAACATCCTTGACAGACACATTCTTG 9 (0.000231%)

AATCTTTTCCTTCAGCTCAGCAAACTTGCATGCAATGTGAGCCGTGTGGCAATCCAATACAGGGGCATA
GCCGGCGCTTATTTGGCCTGGATGGTTCAGG 14 (0.000359%)

AATGGGTCTCAAAATTCTGTGACAAATTTTTGGTCAAGTTGTTTCCATTAAAAAGTACTGATTTTAAAAA
CTAATAACTTAAAACTGCCACACGCAAAAA 6 (0.000154%)

AATGGTTCTTTTGTCGATGCCACCGCATTTATAGATCAGATGGCCAGTAGTGGTGGACTTGCCCGAATCT
ACGTGTCCAATGACGACAATGTTGATATGA 3 (0.000077%)

AATGTCACAGGTCCAGGGCAGAGGACCAACATGGGCATTTTGTTTATGAGCAAGGTGGGTCTCAGAGG
TGATCGGCGATCAGAGGGCGATGAAGTTCTAG 4 (0.000103%)

AATTCTGTGACAAATTTTTGGTCAAGTTGTTTCCATTAAAAAGTACTGATTTTAAAAACTAATAACTTAA
AACTGCCACACGCAAAAAAGAAAACCAAAG 9 (0.000231%)

AATTTCCACAAGGAGATATCAATGGTGATACCACGTTCACGCTCAGCTTTCAGTTTATCCAAGACCCAG
GCATACTTGAAGGAGCCCTTTCCCATCTCAG 11 (0.000282%)

AATTTCCTCATATCTCTTCTGGCTGTAGGGTGGCTCAGTGGAATCCATTTTGTTAACACCGACAATTAGT
TGTTTCACACCCAGTGTGTAAGCCAGAAGG 12 (0.000308%)

AATTTTCTTAATGTAAGTGCTGACTTCCTTAACAATTTCCTCATATCTCTTCTGGCTGTAGGGTGGCTCAG
TGGAATCCATTTTGTTAACACCGACAATT 34 (0.000873%)

AATTTTTCAATGGTTCTTTTGTCGATGCCACCGCATTTATAGATCAGATGGCCAGTAGTGGTGGACTTGC
CCGAATCTACGTGTCCAATGACGACAATGT 3 (0.000077%)

AATTTTTGGTCAAGTTGTTTCCATTAAAAAGTACTGATTTTAAAAACTAATAACTTAAAACTGCCACACG
CAAAAAAGAAAACCAAAGTGGTCCACAAAA 3 (0.000077%)

ACAAATTTTTGGTCAAGTTGTTTCCATTAAAAAGTACTGATTTTAAAAACTAATAACTTAAAACTGCCAC
ACGCAAAAAAGAAAACCAAAGTGGTCCACA 4 (0.000103%)

ACAATTTCCTCATATCTCTTCTGGCTGTAGGGTGGCTCAGTGGAATCCATTTTGTTAACACCGACAATTA
GTTGTTTCACACCCAGTGTGTAAGCCAGAA 4 (0.000103%)

ACACACTTCCTAATGGCAGAATTTGGCTGTTTGGCTTCAACTCCTACTTTTTCCAGCACGATTCCTTTTGC
ATGAGAAGCACCTCCAAAAGGGTTGGCCT 3 (0.000077%)

ACACGAAGGCCCCAGAAGTGACGCAGCCCTCTATGGGCCCGAATCTTCTTCAGTCGCTCCAGGTCTTCA
CGGAGCTTGTTGTCCAGACCATTGGCTAGGA 3 (0.000077%)

ACACTTCCTAATGGCAGAATTTGGCTGTTTGGCTTCAACTCCTACTTTTTCCAGCACGATTCCTTTTGCAT
GAGAAGCACCTCCAAAAGGGTTGGCCTTT 4 (0.000103%)

ACAGATATTCAGCATCTAACAGTTCAAAAGAAGCCACTACATACTCTTTTCACAAATATGTTTTCACAG
AGCCAATACAGTACTAGCCATTAACCCAGTA 5 (0.000128%)

ACAGGTCCAGGGCAGAGGACCAACATGGGCATTTTGTTTATGAGCAAGGTGGGTCTCAGAGGTGATCG
GCGATCAGAGGGCGATGAAGTTCTAGATCCAT 4 (0.000103%)

ACCAACATGGGCATTTTGTTTATGAGCAAGGTGGGTCTCAGAGGTGATCGGCGATCAGAGGGCGATGA
AGTTCTAGATCCATTGAGACAAGCTCTAGACA 3 (0.000077%)

ACCAGACTTCAAGAATTTAGGGCCATCTTCCAGCTTTTTACCAGAACGGCGATCAATCTTTTCCTTCAGC
TCAGCAAACTTGCATGCAATGTGAGCCGTG 12 (0.000308%)

ACCAGTCTTTTACTACTAAACTTAAATGGCCAATTGAAACAAACAGTTCTGAGACCGTTCTTCCACCACT
GATTAAGAGTGGGGTGGCAGGTATTAGGGA 6 (0.000154%)

ACCATATCAACAATGGCAGCATCACCAGACTTCAAGAATTTAGGGCCATCTTCCAGCTTTTTACCAGAA
CGGCGATCAATCTTTTCCTTCAGCTCAGCAA 24 (0.000616%)

ACCTGAGCAGTGAAGCCAGCTGCTTCCATTGGTGGGTCATTTTTGCTGTCACCAGCAACGTTGCCACGA
CGAACATCCTTGACAGACACATTCTTGACAT 3 (0.000077%)

ACCTTCTCTGGCATTCGGGCATTGGCTGTACCCTTCCGCTTACCTATGCCCATGTGCCTGCCCTTCCGGC
GGGCCAAGGTGTTTTTCCGGCATCGAGCCC 3 (0.000077%)

ACTAAACTTAAATGGCCAATTGAAACAAACAGTTCTGAGACCGTTCTTCCACCACTGATTAAGAGTGGG
GTGGCAGGTATTAGGGATAATATTCATTTAG 3 (0.000077%)

ACTTCAAGAATTTAGGGCCATCTTCCAGCTTTTTACCAGAACGGCGATCAATCTTTTCCTTCAGCTCAGC
AAACTTGCATGCAATGTGAGCCGTGTGGCA 3 (0.000077%)

ACTTCCTAATGGCAGAATTTGGCTGTTTGGCTTCAACTCCTACTTTTTCCAGCACGATTCCTTTTGCATGA
GAAGCACCTCCAAAAGGGTTGGCCTTTAG 3 (0.000077%)

ACTTCCTTAACAATTTCCTCATATCTCTTCTGGCTGTAGGGTGGCTCAGTGGAATCCATTTTGTTAACAC
CGACAATTAGTTGTTTCACACCCAGTGTGT 4 (0.000103%)

ACTTGCATGCAATGTGAGCCGTGTGGCAATCCAATACAGGGGCATAGCCGGCGCTTATTTGGCCTGGAT
GGTTCAGGATAATCACCTGAGCAGTGAAGCC 5 (0.000128%)

ACTTGCTGGTCTCAAATTTCCACAAGGAGATATCAATGGTGATACCACGTTCACGCTCAGCTTTCAGTTT

ATCCAAGACCCAGGCATACTTGAAGGAGCC 1073 (0.027544%)

ACTTGGTGACCTTGCCAGCTCCAGCAGCCTTCTTGTCCACTGCTTTGATGACACCCACCGCAACTGTCTG
TCTCATATCACGAACAGCAAAGCGACCCAA 12 (0.000308%)

AGAATGTCACAGGTCCAGGGCAGAGGACCAACATGGGCATTTTGTTTATGAGCAAGGTGGGTCTCAGA
GGTGATCGGCGATCAGAGGGCGATGAAGTTCT 17 (0.000436%)

AGAATTTAGGGCCATCTTCCAGCTTTTTACCAGAACGGCGATCAATCTTTTCCTTCAGCTCAGCAAACTT
GCATGCAATGTGAGCCGTGTGGCAATCCAA 6 (0.000154%)

AGAATTTGGCTGTTTGGCTTCAACTCCTACTTTTTCCAGCACGATTCCTTTTGCATGAGAAGCACCTCCA
AAAGGGTTGGCCTTTAGGGCTGTGCCCAAA 5 (0.000128%)

AGACGATGCCAGTGCCCCTGGGTGCAGGGATGAGGCGTACCAGCACAGAGCCGCAGCGGCCTGTCACC
TTGCAAGGGACAGTGTGGGGCTTGCCGATCTT 6 (0.000154%)

AGACTTCAAGAATTTAGGGCCATCTTCCAGCTTTTTACCAGAACGGCGATCAATCTTTTCCTTCAGCTCA
GCAAACTTGCATGCAATGTGAGCCGTGTGG 5 (0.000128%)

AGACTTGGTGACCTTGCCAGCTCCAGCAGCCTTCTTGTCCACTGCTTTGATGACACCCACCGCAACTGTC
TGTCTCATATCACGAACAGCAAAGCGACCC 8 (0.000205%)

AGAGGACCAACATGGGCATTTTGTTTATGAGCAAGGTGGGTCTCAGAGGTGATCGGCGATCAGAGGGC
GATGAAGTTCTAGATCCATTGAGACAAGCTCT 9 (0.000231%)

AGAGGGAGTATAGGGCTGTGACTAGTATGTTGAGTCCTGTAAGTAGGAGAGTGATATTTGATCAGGAG
AACGTGGTTACTAGCACAGAGAGTTCTCCCAG 7 (0.000180%)

AGCAAACTTGCATGCAATGTGAGCCGTGTGGCAATCCAATACAGGGGCATAGCCGGCGCTTATTTGGCC
TGGATGGTTCAGGATAATCACCTGAGCAGTG   19 (0.000488%)

AGCAGCAACAATCAGGACAGCACAGTCAGCCTGAGATGTCCCTGTAATCATGTTTTTGATAAAGTCTCT
GTGTCCTGGGGCATCAATGATAGTCACATAG   10 (0.000257%)

AGCAGCCTTCTTGTCCACTGCTTTGATGACACCCACCGCAACTGTCTGTCTCATATCACGAACAGCAAA
GCGACCCAAAGGTGGATAGTCTGAGAAGCTC 3 (0.000077%)

AGCAGTGAAGCCAGCTGCTTCCATTGGTGGGTCATTTTTGCTGTCACCAGCAACGTTGCCACGACGAAC
ATCCTTGACAGACACATTCTTGACATTGAAG   5 (0.000128%)

AGCATCACCAGACTTCAAGAATTTAGGGCCATCTTCCAGCTTTTTACCAGAACGGCGATCAATCTTTTCC
TTCAGCTCAGCAAACTTGCATGCAATGTGA   11 (0.000282%)

AGCCAATTTTCTTAATGTAAGTGCTGACTTCCTTAACAATTTCCTCATATCTCTTCTGGCTGTAGGGTGG
CTCAGTGGAATCCATTTTGTTAACACCGAC   5 (0.000128%)

AGCCTGAGAATAGGGGAAATCAGTGAATGAAGCCTCCTATGATGGCAAATACAGCTCCTATTGATAGG
ACATAGTGGAAGTGAGCTACAACGTAGTACGT   3 (0.000077%)

AGCCTTCTGAGCTTTCTGGGCAGACTTGGTGACCTTGCCAGCTCCAGCAGCCTTCTTGTCCACTGCTTTG
ATGACACCCACCGCAACTGTCTGTCTCATA   13 (0.000334%)

AGCCTTCTTGTCCACTGCTTTGATGACACCCACCGCAACTGTCTGTCTCATATCACGAACAGCAAAGCG
ACCCAAAGGTGGATAGTCTGAGAAGCTCTCA 3 (0.000077%)

AGCGATTTCTAGGATAGTCAGTAGAATTAGAATTGTGAAGATGATAAGTGTAGAGGGAAGGTTAATGG
TTGATATTGCTAGGGTGGCGCTTCCAATTAGG 27 (0.000693%)

AGCTCAGCAAACTTGCATGCAATGTGAGCCGTGTGGCAATCCAATACAGGGGCATAGCCGGCGCTTATT
TGGCCTGGATGGTTCAGGATAATCACCTGAG 4 (0.000103%)

AGCTCCAGCAGCCTTCTTGTCCACTGCTTTGATGACACCCACCGCAACTGTCTGTCTCATATCACGAACA
GCAAAGCGACCCAAAGGTGGATAGTCTGAG 6 (0.000154%)

AGCTTCTTAGGCACAGGTGCGGAGACGATGCCAGTGCCCCTGGGTGCAGGGATGAGGCGTACCAGCAC
AGAGCCGCAGCGGCCTGTCACCTTGCAAGGGA 562 (0.014426%)

AGCTTTCTGGGCAGACTTGGTGACCTTGCCAGCTCCAGCAGCCTTCTTGTCCACTGCTTTGATGACACCC
ACCGCAACTGTCTGTCTCATATCACGAACA 11 (0.000282%)

AGGAACCATATCAACAATGGCAGCATCACCAGACTTCAAGAATTTAGGGCCATCTTCCAGCTTTTTACC
AGAACGGCGATCAATCTTTTCCTTCAGCTCA 49 (0.001258%)

AGGACCAACATGGGCATTTTGTTTATGAGCAAGGTGGGTCTCAGAGGTGATCGGCGATCAGAGGGCGA
TGAAGTTCTAGATCCATTGAGACAAGCTCTAG 5 (0.000128%)

AGGAGATATCAATGGTGATACCACGTTCACGCTCAGCTTTCAGTTTATCCAAGACCCAGGCATACTTGA
AGGAGCCCTTTCCCATCTCAGCAGCCTCCTT 3 (0.000077%)

AGGATAATCACCTGAGCAGTGAAGCCAGCTGCTTCCATTGGTGGGTCATTTTTGCTGTCACCAGCAACG
TTGCCACGACGAACATCCTTGACAGACACAT 18 (0.000462%)

AGGCCACCTACGGTGAAAAGAAAGATGAATCCTAGGGCTCAGAGCACTGCAGCAGATCATTTCATATT
GCTTCCGTGGAGTGTGGCGAGTCAGCTAAATA 16 (0.000411%)

AGGGAATCATAAATCATGCCAAAGCCAGTTGTCTTGCCACCACCAAAATGAGTTCTGAATCCAAATACA
AAGATGACATCCGGTGTGGTCTTGTACATTT 4 (0.000103%)

AGGGAGTATAGGGCTGTGACTAGTATGTTGAGTCCTGTAAGTAGGAGAGTGATATTTGATCAGGAGAA
CGTGGTTACTAGCACAGAGAGTTCTCCCAGTA 16 (0.000411%)

AGGGATAATATTCATTTAGCCTTCTGAGCTTTCTGGGCAGACTTGGTGACCTTGCCAGCTCCAGCAGCCT
TCTTGTCCACTGCTTTGATGACACCCACCG 6 (0.000154%)

AGGGCCATCTTCCAGCTTTTTACCAGAACGGCGATCAATCTTTTCCTTCAGCTCAGCAAACTTGCATGCA
ATGTGAGCCGTGTGGCAATCCAATACAGGG 7 (0.000180%)

AGGTCCAGGGCAGAGGACCAACATGGGCATTTTGTTTATGAGCAAGGTGGGTCTCAGAGGTGATCGGC
GATCAGAGGGCGATGAAGTTCTAGATCCATTG3 (0.000077%)

AGTAGAATTAGAATTGTGAAGATGATAAGTGTAGAGGGAAGGTTAATGGTTGATATTGCTAGGGTGGC
GCTTCCAATTAGGTGCATGAGTAGGTGGCCTG 3 (0.000077%)

AGTAGTTCCCTGCTAAGGGAGGGTAGACTGTTCAACCTGTTCCTGCTCCGGCCTCCACTATAGCAGATG
CGAGCAGGAGTAGGAGAGAGGGAGGTAAGAG 13 (0.000334%)

AGTCAGGCCACCTACGGTGAAAAGAAAGATGAATCCTAGGGCTCAGAGCACTGCAGCAGATCATTTCA
TATTGCTTCCGTGGAGTGTGGCGAGTCAGCTA 22 (0.000565%)

AGTCTTTTACTACTAAACTTAAATGGCCAATTGAAACAAACAGTTCTGAGACCGTTCTTCCACCACTGAT
TAAGAGTGGGGTGGCAGGTATTAGGGATAA 3 (0.000077%)

AGTGCCCCTGGGTGCAGGGATGAGGCGTACCAGCACAGAGCCGCAGCGGCCTGTCACCTTGCAAGGGA
CAGTGTGGGGCTTGCCGATCTTGTTCCCCCAG 4 (0.000103%)

AGTGCCCTCCTAATTGGGGGGTAGGGGCTAGGCTGGAGTGGTAAAAGGCTCAGAAAAATCCTGCGAAG
AAAAAAACTTCTGAGGTAATAAATAGGATTAT3 (0.000077%)

AGTTGTTTCCATTAAAAAGTACTGATTTTAAAAACTAATAACTTAAAACTGCCACACGCAAAAAAGAAA
ACCAAAGTGGTCCACAAAACATTCTCCTTTC 3 (0.000077%)

ATAATATTCATTTAGCCTTCTGAGCTTTCTGGGCAGACTTGGTGACCTTGCCAGCTCCAGCAGCCTTCTT
GTCCACTGCTTTGATGACACCCACCGCAAC 89 (0.002285%)

ATAATCACCTGAGCAGTGAAGCCAGCTGCTTCCATTGGTGGGTCATTTTTGCTGTCACCAGCAACGTTGC
CACGACGAACATCCTTGACAGACACATTCT 11 (0.000282%)

ATACAATGCCAGTCAGGCCACCTACGGTGAAAAGAAAGATGAATCCTAGGGCTCAGAGCACTGCAGCA
GATCATTTCATATTGCTTCCGTGGAGTGTGGC 13 (0.000334%)

ATAGTCAGTAGAATTAGAATTGTGAAGATGATAAGTGTAGAGGGAAGGTTAATGGTTGATATTGCTAG
GGTGGCGCTTCCAATTAGGTGCATGAGTAGGT 4 (0.000103%)

ATATCAACAATGGCAGCATCACCAGACTTCAAGAATTTAGGGCCATCTTCCAGCTTTTTACCAGAACGG
CGATCAATCTTTTCCTTCAGCTCAGCAAACT 19 (0.000488%)

ATATGTAGAGGGAGTATAGGGCTGTGACTAGTATGTTGAGTCCTGTAAGTAGGAGAGTGATATTTGATC
AGGAGAACGTGGTTACTAGCACAGAGAGTTC 3 (0.000077%)

ATATTCATTTAGCCTTCTGAGCTTTCTGGGCAGACTTGGTGACCTTGCCAGCTCCAGCAGCCTTCTTGTC
CACTGCTTTGATGACACCCACCGCAACTGT 10 (0.000257%)

ATCAACAATGGCAGCATCACCAGACTTCAAGAATTTAGGGCCATCTTCCAGCTTTTTACCAGAACGGCG
ATCAATCTTTTCCTTCAGCTCAGCAAACTTG 27 (0.000693%)

ATCACCAGACTTCAAGAATTTAGGGCCATCTTCCAGCTTTTTACCAGAACGGCGATCAATCTTTTCCTTC
AGCTCAGCAAACTTGCATGCAATGTGAGCC 6 (0.000154%)

ATCGGAAGAGCACACGTCTGAACTCCAGTCACATCGCAATTGCGATCTGGGGGGGGGGGGGGGGGGG
GGGGGGGGGGGGGGGGGGGGGGGGGGGGGGGGGGGGGGGGGGGGGGGGGGGGGGGGGGGGGGGGGGG
GGGGGGGGGGGGGGGG 4 (0.000153%)

ATCTGCGGGTGGCACTGCCCACGGTGGGCGGCGGGCCTCTCTACTCGAAGGTGACCACGTTTAGATTC
TGAGACGGGAAGTGGAGGGTGAATAGGTCAC 5 (0.000128%)

ATCTTTTCCTTCAGCTCAGCAAACTTGCATGCAATGTGAGCCGTGTGGCAATCCAATACAGGGGCATAG
CCGGCGCTTATTTGGCCTGGATGGTTCAGGA 24 (0.000616%)

ATGAGCAAGGTGGGTCTCAGAGGTGATCGGCGATCAGAGGGCGATGAAGTTCTAGATCCATTGAGACA
AGCTCTAGACAGTAGCATGCAGTCCCACAACT3 (0.000077%)

ATGCAATGTGAGCCGTGTGGCAATCCAATACAGGGGCATAGCCGGCGCTTATTTGGCCTGGATGGTTCA
GGATAATCACCTGAGCAGTGAAGCCAGCTGC 13 (0.000334%)

ATGCCAGTCAGGCCACCTACGGTGAAAAGAAAGATGAATCCTAGGGCTCAGAGCACTGCAGCAGATCA
TTTCATATTGCTTCCGTGGAGTGTGGCGAGTC 4 (0.000103%)

ATGCCAGTGCCCCTGGGTGCAGGGATGAGGCGTACCAGCACAGAGCCGCAGCGGCCTGTCACCTTGCA
AGGGACAGTGTGGGGCTTGCCGATCTTGTTCC 4 (0.000103%)

ATGGCAGCATCACCAGACTTCAAGAATTTAGGGCCATCTTCCAGCTTTTTACCAGAACGGCGATCAATC
TTTTCCTTCAGCTCAGCAAACTTGCATGCAA 4 (0.000103%)

ATGGGTCTCAAAATTCTGTGACAAATTTTTGGTCAAGTTGTTTCCATTAAAAAGTACTGATTTTAAAAAC TAATAACTTAAAACTGCCACACGCAAAAAA 12 (0.000308%)

ATGTAGAGGGAGTATAGGGCTGTGACTAGTATGTTGAGTCCTGTAAGTAGGAGAGTGATATTTGATCAG GAGAACGTGGTTACTAGCACAGAGAGTTCTC 6 (0.000154%)

ATGTCACAGGTCCAGGGCAGAGGACCAACATGGGCATTTTGTTTATGAGCAAGGTGGGTCTCAGAGGT GATCGGCGATCAGAGGGCGATGAAGTTCTAGA 8 (0.000205%)

ATGTGCTTTCTCGTGTTACATCGCGCCATCATTGGTATATGGTTAGTGTGTTGGTTAGTAGGCCTAGTAT GAGGAGCGTTATGGAGTGGAAGTGAAATCA 3 (0.000077%)

ATGTGGGGACAGCTCATGAGTGCAAGACGTCTTGTGATGTAATTATTATACGAATGGGGGCTTCAATCG GGAGTACTACTCGATTGTCAACGTCAAGGAG 3 (0.000077%)

ATTAACCAGTCTTTTACTACTAAACTTAAATGGCCAATTGAAACAAACAGTTCTGAGACCGTTCTTCCAC CACTGATTAAGAGTGGGGTGGCAGGTATTA 870 (0.022333%)

ATTAGGGATAATATTCATTTAGCCTTCTGAGCTTTCTGGGCAGACTTGGTGACCTTGCCAGCTCCAGCAG CCTTCTTGTCCACTGCTTTGATGACACCCA923 (0.023693%)

ATTCATTTAGCCTTCTGAGCTTTCTGGGCAGACTTGGTGACCTTGCCAGCTCCAGCAGCCTTCTTGTCCA CTGCTTTGATGACACCCACCGCAACTGTCT 217 (0.005570%)

ATTCTGTGACAAATTTTTGGTCAAGTTGTTTCCATTAAAAAGTACTGATTTTAAAAACTAATAACTTAAA ACTGCCACACGCAAAAAAGAAAACCAAAGT 15 (0.000385%)

ATTGAGAGAGTGAGGAGAAGGCTTACGTTTAGTGAGGGAGAGATTTGGTATATGATTGAGATGGGGGC TAGTTTTTGTCATGTGAGAAGAAGCAGGCCGG3 (0.000077%)

ATTGTGTTGTGGTAAATATGTAGAGGGAGTATAGGGCTGTGACTAGTATGTTGAGTCCTGTAAGTAGGA GAGTGATATTTGATCAGGAGAACGTGGTTAC 10 (0.000257%)

ATTTAGCCTTCTGAGCTTTCTGGGCAGACTTGGTGACCTTGCCAGCTCCAGCAGCCTTCTTGTCCACTGC TTTGATGACACCCACCGCAACTGTCTGTCT 92 (0.002362%)

ATTTAGGGCCATCTTCCAGCTTTTTACCAGAACGGCGATCAATCTTTTCCTTCAGCTCAGCAAACTTGCA TGCAATGTGAGCCGTGTGGCAATCCAATAC 27 (0.000693%)

ATTTCAAAGATTTTTAGGGGAATTAATTCTAGGACGATGGGCATGAAACTGTGGTTTGCTCCACAGATT TCAGAGCATTGACCGTAGTATACCCCCGGTC 51 (0.001309%)

ATTTCCACAAGGAGATATCAATGGTGATACCACGTTCACGCTCAGCTTTCAGTTTATCCAAGACCCAGG CATACTTGAAGGAGCCCTTTCCCATCTCAGC 12 (0.000308%)

ATTTCCTCATATCTCTTCTGGCTGTAGGGTGGCTCAGTGGAATCCATTTTGTTAACACCGACAATTAGTT GTTTCACACCCAGTGTGTAAGCCAGAAGGG 16 (0.000411%)

ATTTTCTTAATGTAAGTGCTGACTTCCTTAACAATTTCCTCATATCTCTTCTGGCTGTAGGGTGGCTCAGT GGAATCCATTTTGTTAACACCGACAATTA 16 (0.000411%)

ATTTTGGCGTAGGTTTGGTCTAGGGTGTAGCCTGAGAATAGGGGAAATCAGTGAATGAAGCCTCCTATG ATGGCAAATACAGCTCCTATTGATAGGACAT 32 (0.000821%)

ATTTTGTTTATGAGCAAGGTGGGTCTCAGAGGTGATCGGCGATCAGAGGGCGATGAAGTTCTAGATCCA TTGAGACAAGCTCTAGACAGTAGCATGCAGT 52 (0.001335%)

ATTTTTAATCTTAGAGCGAAAGCCTATAATCACTGCGCCTGTTCATAAGGGGATGGCCATGGCTAGGTT

TATAGATAGTTGGGTGGTTGGTGTAAATGAG 4 (0.000103%)

ATTTTTCAATGGTTCTTTTGTCGATGCCACCGCATTTATAGATCAGATGGCCAGTAGTGGTGGACTTGCC
CGAATCTACGTGTCCAATGACGACAATGTT 6 (0.000154%)

ATTTTTCTTTGCATAATCCAGGGAATCATAAATCATGCCAAAGCCAGTTGTCTTGCCACCACCAAAATG
AGTTCTGAATCCAAATACAAAGATGACATCC 269 (0.006905%)

ATTTTTGGTCAAGTTGTTTCCATTAAAAAGTACTGATTTTAAAAACTAATAACTTAAAACTGCCACACGC
AAAAAAGAAAACCAAAGTGGTCCACAAAAC 11 (0.000282%)

CAAAATTCTGTGACAAATTTTTGGTCAAGTTGTTTCCATTAAAAAGTACTGATTTTAAAAACTAATAACT
TAAAACTGCCACACGCAAAAAAGAAAACCA 214 (0.005493%)

CAAACTTGCATGCAATGTGAGCCGTGTGGCAATCCAATACAGGGGCATAGCCGGCGCTTATTTGGCCTG
GATGGTTCAGGATAATCACCTGAGCAGTGAA 8 (0.000205%)

CAAATTTCCACAAGGAGATATCAATGGTGATACCACGTTCACGCTCAGCTTTCAGTTTATCCAAGACCC
AGGCATACTTGAAGGAGCCCTTTCCCATCTC 90 (0.002310%)

CAAATTTTTCAATGGTTCTTTTGTCGATGCCACCGCATTTATAGATCAGATGGCCAGTAGTGGTGGACTT
GCCCGAATCTACGTGTCCAATGACGACAAT 21 (0.000539%)

CAAATTTTTGGTCAAGTTGTTTCCATTAAAAAGTACTGATTTTAAAAACTAATAACTTAAAACTGCCACA
CGCAAAAAAGAAAACCAAAGTGGTCCACAA 18 (0.000462%)

CAACAATGGCAGCATCACCAGACTTCAAGAATTTAGGGCCATCTTCCAGCTTTTTACCAGAACGGCGAT
CAATCTTTTCCTTCAGCTCAGCAAACTTGCA 38 (0.000975%)

CAACATGGGCATTTTGTTTATGAGCAAGGTGGGTCTCAGAGGTGATCGGCGATCAGAGGGCGATGAAG
TTCTAGATCCATTGAGACAAGCTCTAGACAGT 5 (0.000128%)

CAAGAATTTAGGGCCATCTTCCAGCTTTTTACCAGAACGGCGATCAATCTTTTCCTTCAGCTCAGCAAAC
TTGCATGCAATGTGAGCCGTGTGGCAATCC 34 (0.000873%)

CAAGGAGATATCAATGGTGATACCACGTTCACGCTCAGCTTTCAGTTTATCCAAGACCCAGGCATACTT
GAAGGAGCCCTTTCCCATCTCAGCAGCCTCC 7 (0.000180%)

CAAGTTGTTTCCATTAAAAAGTACTGATTTTAAAAACTAATAACTTAAAACTGCCACACGCAAAAAAGA
AAACCAAAGTGGTCCACAAAACATTCTCCTT 19 (0.000488%)

CAATCCAATACAGGGGCATAGCCGGCGCTTATTTGGCCTGGATGGTTCAGGATAATCACCTGAGCAGTG
AAGCCAGCTGCTTCCATTGGTGGGTCATTTT 19 (0.000488%)

CAATCTTTTCCTTCAGCTCAGCAAACTTGCATGCAATGTGAGCCGTGTGGCAATCCAATACAGGGGCAT
AGCCGGCGCTTATTTGGCCTGGATGGTTCAG 103 (0.002644%)

CAATGTGAGCCGTGTGGCAATCCAATACAGGGGCATAGCCGGCGCTTATTTGGCCTGGATGGTTCAGGA
TAATCACCTGAGCAGTGAAGCCAGCTGCTTC 4 (0.000103%)

CAATTTCCTCATATCTCTTCTGGCTGTAGGGTGGCTCAGTGGAATCCATTTTGTTAACACCGACAATTAG
TTGTTTCACACCCAGTGTGTAAGCCAGAAG 18 (0.000462%)

CAATTTTCTTAATGTAAGTGCTGACTTCCTTAACAATTTCCTCATATCTCTTCTGGCTGTAGGGTGGCTCA
GTGGAATCCATTTTGTTAACACCGACAAT 36 (0.000924%)

CACAAGGAGATATCAATGGTGATACCACGTTCACGCTCAGCTTTCAGTTTATCCAAGACCCAGGCATAC
TTGAAGGAGCCCTTTCCCATCTCAGCAGCCT 4 (0.000103%)

CACACTTCCTAATGGCAGAATTTGGCTGTTTGGCTTCAACTCCTACTTTTTCCAGCACGATTCCTTTTGCATGAGAAGCACCTCCAAAAGGGTTGGCCTT 14 (0.000359%)

CACAGGTCCAGGGCAGAGGACCAACATGGGCATTTTGTTTATGAGCAAGGTGGGTCTCAGAGGTGATCGGCGATCAGAGGGCGATGAAGTTCTAGATCCA 53 (0.001360%)

CACAGTCTTGGTTCCCCGGAGACGTCCAGTCCGGCGGGCAGCAATGAGACCCACTTTGCGGCCAGCAGGGGCATCTCTGCGGATGGTGGAGGGCTTGCCG 8 (0.000205%)

CACCAGACTTCAAGAATTTAGGGCCATCTTCCAGCTTTTTACCAGAACGGCGATCAATCTTTTCCTTCAGCTCAGCAAACTTGCATGCAATGTGAGCCGT 25 (0.000642%)

CACCGCATTTATAGATCAGATGGCCAGTAGTGGTGGACTTGCCCGAATCTACGTGTCCAATGACGACAATGTTGATATGAGTCTTTTCCTTTCCCATTTT 3 (0.000077%)

CACCTACGGTGAAAAGAAAGATGAATCCTAGGGCTCAGAGCACTGCAGCAGATCATTTCATATTGCTTCCGTGGAGTGTGGCGAGTCAGCTAAATACTTT 6 (0.000154%)

CACCTGAGCAGTGAAGCCAGCTGCTTCCATTGGTGGGTCATTTTTGCTGTCACCAGCAACGTTGCCACGACGAACATCCTTGACAGACACATTCTTGACA 7 (0.000180%)

CACTTCCTAATGGCAGAATTTGGCTGTTTGGCTTCAACTCCTACTTTTTCCAGCACGATTCCTTTTGCATGAGAAGCACCTCCAAAAGGGTTGGCCTTTA 3 (0.000077%)

CAGAATGTCACAGGTCCAGGGCAGAGGACCAACATGGGCATTTTGTTTATGAGCAAGGTGGGTCTCAGAGGTGATCGGCGATCAGAGGGCGATGAAGTTC 678 (0.017404%)

CAGAATTTGGCTGTTTGGCTTCAACTCCTACTTTTTCCAGCACGATTCCTTTTGCATGAGAAGCACCTCCAAAAGGGTTGGCCTTTAGGGCTGTGCCCAA 33 (0.000847%)

CAGACTTCAAGAATTTAGGGCCATCTTCCAGCTTTTTACCAGAACGGCGATCAATCTTTTCCTTCAGCTCAGCAAACTTGCATGCAATGTGAGCCGTGTG 43 (0.001104%)

CAGACTTGGTGACCTTGCCAGCTCCAGCAGCCTTCTTGTCCACTGCTTTGATGACACCCACCGCAACTGTCTGTCTCATATCACGAACAGCAAAGCGACC 40 (0.001027%)

CAGAGGACCAACATGGGCATTTTGTTTATGAGCAAGGTGGGTCTCAGAGGTGATCGGCGATCAGAGGGCGATGAAGTTCTAGATCCATTGAGACAAGCTC 14 (0.000359%)

CAGAGGGCGATGAAGTTCTAGATCCATTGAGACAAGCTCTAGACAGTAGCATGCAGTCCCACAACTTGTACCAGCATCCCCAGCGTCTGGCATTCCATGT 10 (0.000257%)

CAGATATTCAGCATCTAACAGTTCAAAAGAAGCCACTACATACTCTTTTCACAAATATGTTTTCACAGAGCCAATACAGTACTAGCCATTAACCCAGTAC 12 (0.000308%)

CAGCAAACTTGCATGCAATGTGAGCCGTGTGGCAATCCAATACAGGGGCATAGCCGGCGCTTATTTGGCCTGGATGGTTCAGGATAATCACCTGAGCAGT 28 (0.000719%)

CAGCAGCAACAATCAGGACAGCACAGTCAGCCTGAGATGTCCCTGTAATCATGTTTTTGATAAAGTCTCTGTGTCCTGGGGCATCAATGATAGTCACATA 1443 (0.037041%)

CAGCAGCCTTCTTGTCCACTGCTTTGATGACACCCACCGCAACTGTCTGTCTCATATCACGAACAGCAAAGCGACCCAAAGGTGGATAGTCTGAGAAGCT 7 (0.000180%)

CAGCATCACCAGACTTCAAGAATTTAGGGCCATCTTCCAGCTTTTTACCAGAACGGCGATCAATCTTTTCCTTCAGCTCAGCAAACTTGCATGCAATGTG 14 (0.000359%)

CAGCATCTAACAGTTCAAAAGAAGCCACTACATACTCTTTTCACAAATATGTTTTCACAGAGCCAATAC
AGTACTAGCCATTAACCCAGTACACCAAGTG 5 (0.000128%)

CAGCCTTCTTGTCCACTGCTTTGATGACACCCACCGCAACTGTCTGTCTCATATCACGAACAGCAAAGCG
ACCCAAAGGTGGATAGTCTGAGAAGCTCTC 10 (0.000257%)

CAGCGATTTCTAGGATAGTCAGTAGAATTAGAATTGTGAAGATGATAAGTGTAGAGGGAAGGTTAATG
GTTGATATTGCTAGGGTGGCGCTTCCAATTAG 10 (0.000257%)

CAGCTCAGCAAACTTGCATGCAATGTGAGCCGTGTGGCAATCCAATACAGGGGCATAGCCGGCGCTTAT
TTGGCCTGGATGGTTCAGGATAATCACCTGA 12 (0.000308%)

CAGCTCCAGCAGCCTTCTTGTCCACTGCTTTGATGACACCCACCGCAACTGTCTGTCTCATATCACGAAC
AGCAAAGCGACCCAAAGGTGGATAGTCTGA 10 (0.000257%)

CAGCTTTTTACCAGAACGGCGATCAATCTTTTCCTTCAGCTCAGCAAACTTGCATGCAATGTGAGCCGTG
TGGCAATCCAATACAGGGGCATAGCCGGCG 356 (0.009138%)

CAGGAACCATATCAACAATGGCAGCATCACCAGACTTCAAGAATTTAGGGCCATCTTCCAGCTTTTTAC
CAGAACGGCGATCAATCTTTTCCTTCAGCTC 59 (0.001515%)

CAGGATAATCACCTGAGCAGTGAAGCCAGCTGCTTCCATTGGTGGGTCATTTTTGCTGTCACCAGCAAC
GTTGCCACGACGAACATCCTTGACAGACACA 19 (0.000488%)

CAGGCCACCTACGGTGAAAAGAAAGATGAATCCTAGGGCTCAGAGCACTGCAGCAGATCATTTCATAT
TGCTTCCGTGGAGTGTGGCGAGTCAGCTAAAT 16 (0.000411%)

CAGGGAATCATAAATCATGCCAAAGCCAGTTGTCTTGCCACCACCAAAATGAGTTCTGAATCCAAATAC
AAAGATGACATCCGGTGTGGTCTTGTACATT 4 (0.000103%)

CAGGGATGAGGCGTACCAGCACAGAGCCGCAGCGGCCTGTCACCTTGCAAGGGACAGTGTGGGGCTTG
CCGATCTTGTTCCCCCAGTAGCCTCTGCGCAC 20 (0.000513%)

CAGGTCCAGGGCAGAGGACCAACATGGGCATTTTGTTTATGAGCAAGGTGGGTCTCAGAGGTGATCGG
CGATCAGAGGGCGATGAAGTTCTAGATCCATT 5 (0.000128%)

CAGTCAGGCCACCTACGGTGAAAAGAAAGATGAATCCTAGGGCTCAGAGCACTGCAGCAGATCATTTC
ATATTGCTTCCGTGGAGTGTGGCGAGTCAGCT 3 (0.000077%)

CAGTCTTTTACTACTAAACTTAAATGGCCAATTGAAACAAACAGTTCTGAGACCGTTCTTCCACCACTGA
TTAAGAGTGGGGTGGCAGGTATTAGGGATA 45 (0.001155%)

CAGTGCCCCTGGGTGCAGGGATGAGGCGTACCAGCACAGAGCCGCAGCGGCCTGTCACCTTGCAAGGG
ACAGTGTGGGGCTTGCCGATCTTGTTCCCCCA 8 (0.000205%)

CAGTGCCCTCCTAATTGGGGGGTAGGGGCTAGGCTGGAGTGGTAAAAGGCTCAGAAAAATCCTGCGAA
GAAAAAAACTTCTGAGGTAATAAATAGGATTA 10 (0.000257%)

CATATCAACAATGGCAGCATCACCAGACTTCAAGAATTTAGGGCCATCTTCCAGCTTTTTACCAGAACG
GCGATCAATCTTTTCCTTCAGCTCAGCAAAC 6 (0.000154%)

CATCACCAGACTTCAAGAATTTAGGGCCATCTTCCAGCTTTTTACCAGAACGGCGATCAATCTTTTCCTT
CAGCTCAGCAAACTTGCATGCAATGTGAGC 16 (0.000411%)

CATCCATGTGACCTTCTCTGGCATTCGGGCATTGGCTGTACCCTTCCGCTTACCTATGCCCATGTGCCTG
CCCTTCCGGCGGGCCAAGGTGTTTTTCCGG 6 (0.000154%)

CATTGTGTTGTGGTAAATATGTAGAGGGAGTATAGGGCTGTGACTAGTATGTTGAGTCCTGTAAGTAGG

AGAGTGATATTTGATCAGGAGAACGTGGTTA 10 (0.000257%)

CATTTAGCCTTCTGAGCTTTCTGGGCAGACTTGGTGACCTTGCCAGCTCCAGCAGCCTTCTTGTCCACTG
CTTTGATGACACCCACCGCAACTGTCTGTC 21 (0.000539%)

CATTTTGTTTATGAGCAAGGTGGGTCTCAGAGGTGATCGGCGATCAGAGGGCGATGAAGTTCTAGATCC
ATTGAGACAAGCTCTAGACAGTAGCATGCAG 3 (0.000077%)

CCAACATGGGCATTTTGTTTATGAGCAAGGTGGGTCTCAGAGGTGATCGGCGATCAGAGGGCGATGAA
GTTCTAGATCCATTGAGACAAGCTCTAGACAG 26 (0.000667%)

CCAATTTTCTTAATGTAAGTGCTGACTTCCTTAACAATTTCCTCATATCTCTTCTGGCTGTAGGGTGGCTC
AGTGGAATCCATTTTGTTAACACCGACAA 9 (0.000231%)

CCACAAGGAGATATCAATGGTGATACCACGTTCACGCTCAGCTTTCAGTTTATCCAAGACCCAGGCATA
CTTGAAGGAGCCCTTTCCCATCTCAGCAGCC 8 (0.000205%)

CCAGACTTCAAGAATTTAGGGCCATCTTCCAGCTTTTTACCAGAACGGCGATCAATCTTTTCCTTCAGCT
CAGCAAACTTGCATGCAATGTGAGCCGTGT 88 (0.002259%)

CCAGCTCCAGCAGCCTTCTTGTCCACTGCTTTGATGACACCCACCGCAACTGTCTGTCTCATATCACGAA
CAGCAAAGCGACCCAAAGGTGGATAGTCTG 7 (0.000180%)

CCAGGAACCATATCAACAATGGCAGCATCACCAGACTTCAAGAATTTAGGGCCATCTTCCAGCTTTTTA
CCAGAACGGCGATCAATCTTTTCCTTCAGCT 57 (0.001463%)

CCAGGGAATCATAAATCATGCCAAAGCCAGTTGTCTTGCCACCACCAAAATGAGTTCTGAATCCAAATA
CAAAGATGACATCCGGTGTGGTCTTGTACAT 10 (0.000257%)

CCAGGGCAGAGGACCAACATGGGCATTTTGTTTATGAGCAAGGTGGGTCTCAGAGGTGATCGGCGATC
AGAGGGCGATGAAGTTCTAGATCCATTGAGAC 7 (0.000180%)

CCAGTCAGGCCACCTACGGTGAAAAGAAAGATGAATCCTAGGGCTCAGAGCACTGCAGCAGATCATTT
CATATTGCTTCCGTGGAGTGTGGCGAGTCAGC 4 (0.000103%)

CCATATCAACAATGGCAGCATCACCAGACTTCAAGAATTTAGGGCCATCTTCCAGCTTTTTACCAGAAC
GGCGATCAATCTTTTCCTTCAGCTCAGCAAA 6 (0.000154%)

CCATGTGACCTTCTCTGGCATTCGGGCATTGGCTGTACCCTTCCGCTTACCTATGCCCATGTGCCTGCCC
TTCCGGCGGGCCAAGGTGTTTTTCCGGCAT 5 (0.000128%)

CCATTGTGTTGTGGTAAATATGTAGAGGGAGTATAGGGCTGTGACTAGTATGTTGAGTCCTGTAAGTAG
GAGAGTGATATTTGATCAGGAGAACGTGGTT 6 (0.000154%)

CCCACTCCTGATGCTGAACCAATGCACCATCTGTAAAGTTGCAGACAGTCTGAGTTTTTCTGCCATCAGC
TGTGGTTTCTTCAAACTTCTCTCCCAGGGT 3 (0.000077%)

CCCATTGTGTTGTGGTAAATATGTAGAGGGAGTATAGGGCTGTGACTAGTATGTTGAGTCCTGTAAGTA
GGAGAGTGATATTTGATCAGGAGAACGTGGT 8 (0.000205%)

CCCCATTGTGTTGTGGTAAATATGTAGAGGGAGTATAGGGCTGTGACTAGTATGTTGAGTCCTGTAAGT
AGGAGAGTGATATTTGATCAGGAGAACGTGG 20 (0.000513%)

CCCCTGGGTGCAGGGATGAGGCGTACCAGCACAGAGCCGCAGCGGCCTGTCACCTTGCAAGGGACAGT
GTGGGGCTTGCCGATCTTGTTCCCCCAGTAGC 3 (0.000077%)

CCCGGAGACGTCCAGTCCGGCGGGCAGCAATGAGACCCACTTTGCGGCCAGCAGGGGCATCTCTGCGG
ATGGTGGAGGGCTTGCCGATGTGCTGGTGGTT 9 (0.000231%)

CCCTGCTAAGGGAGGGTAGACTGTTCAACCTGTTCCTGCTCCGGCCTCCACTATAGCAGATGCGAGCAG
GAGTAGGAGAGAGGGAGGTAAGAGTCAGAAG3 (0.000077%)

CCCTGGGTGCAGGGATGAGGCGTACCAGCACAGAGCCGCAGCGGCCTGTCACCTTGCAAGGGACAGTG
TGGGGCTTGCCGATCTTGTTCCCCCAGTAGCC 4 (0.000103%)

CCGCATTTATAGATCAGATGGCCAGTAGTGGTGGACTTGCCCGAATCTACGTGTCCAATGACGACAATG
TTGATATGAGTCTTTTCCTTTCCCATTTTGG 12 (0.000308%)

CCGGAGACGTCCAGTCCGGCGGGCAGCAATGAGACCCACTTTGCGGCCAGCAGGGGCATCTCTGCGGA
TGGTGGAGGGCTTGCCGATGTGCTGGTGGTTG 5 (0.000128%)

CCGTGTGGCAATCCAATACAGGGGCATAGCCGGCGCTTATTTGGCCTGGATGGTTCAGGATAATCACCT
GAGCAGTGAAGCCAGCTGCTTCCATTGGTGG 11 (0.000282%)

CCTAATGGCAGAATTTGGCTGTTTGGCTTCAACTCCTACTTTTTCCAGCACGATTCCTTTTGCATGAGAA
GCACCTCCAAAAGGGTTGGCCTTTAGGGCT 6 (0.000154%)

CCTAATTGGGGGGTAGGGGCTAGGCTGGAGTGGTAAAAGGCTCAGAAAAATCCTGCGAAGAAAAAAAC
TTCTGAGGTAATAAATAGGATTATCCCGTATC 9 (0.000231%)

CCTACGGTGAAAAGAAAGATGAATCCTAGGGCTCAGAGCACTGCAGCAGATCATTTCATATTGCTTCCG
TGGAGTGTGGCGAGTCAGCTAAATACTTTGA 4 (0.000103%)

CCTATTTCAAAGATTTTTAGGGGAATTAATTCTAGGACGATGGGCATGAAACTGTGGTTTGCTCCACAG
ATTTCAGAGCATTGACCGTAGTATACCCCCG 521 (0.013374%)

CCTCATCCATGTGACCTTCTCTGGCATTCGGGCATTGGCTGTACCCTTCCGCTTACCTATGCCCATGTGC
CTGCCCTTCCGGCGGGCCAAGGTGTTTTTC 4 (0.000103%)

CCTCGGACACGAAGGCCCCAGAAGTGACGCAGCCCTCTATGGGCCCGAATCTTCTTCAGTCGCTCCAGG
TCTTCACGGAGCTTGTTGTCCAGACCATTGG 3 (0.000077%)

CCTGAGAATAGGGGAAATCAGTGAATGAAGCCTCCTATGATGGCAAATACAGCTCCTATTGATAGGAC
ATAGTGGAAGTGAGCTACAACGTAGTACGTGT12 (0.000308%)

CCTGAGCAGTGAAGCCAGCTGCTTCCATTGGTGGGTCATTTTTGCTGTCACCAGCAACGTTGCCACGAC
GAACATCCTTGACAGACACATTCTTGACATT 5 (0.000128%)

CCTGCTAAGGGAGGGTAGACTGTTCAACCTGTTCCTGCTCCGGCCTCCACTATAGCAGATGCGAGCAGG
AGTAGGAGAGAGGGAGGTAAGAGTCAGAAGC3 (0.000077%)

CCTGGGTGCAGGGATGAGGCGTACCAGCACAGAGCCGCAGCGGCCTGTCACCTTGCAAGGGACAGTGT
GGGGCTTGCCGATCTTGTTCCCCCAGTAGCCT 14 (0.000359%)

CCTGTTCATAAGGGGATGGCCATGGCTAGGTTTATAGATAGTTGGGTGGTTGGTGTAAATGAGTGAGGC
AGGAGTCCGAGGAGGTTAGTTGTGGCAATAA 3 (0.000077%)

CCTTAACAATTTCCTCATATCTCTTCTGGCTGTAGGGTGGCTCAGTGGAATCCATTTTGTTAACACCGAC
AATTAGTTGTTTCACACCCAGTGTGTAAGC 14 (0.000359%)

CCTTCAGCTCAGCAAACTTGCATGCAATGTGAGCCGTGTGGCAATCCAATACAGGGGCATAGCCGGCGC
TTATTTGGCCTGGATGGTTCAGGATAATCAC 14 (0.000359%)

CCTTCTCTGGCATTCGGGCATTGGCTGTACCCTTCCGCTTACCTATGCCCATGTGCCTGCCCTTCCGGCG
GGCCAAGGTGTTTTTCCGGCATCGAGCCCG 11 (0.000282%)

CCTTCTGAGCTTTCTGGGCAGACTTGGTGACCTTGCCAGCTCCAGCAGCCTTCTTGTCCACTGCTTTGAT
GACACCCACCGCAACTGTCTGTCTCATATC 3 (0.000077%)

CCTTCTTGTCCACTGCTTTGATGACACCCACCGCAACTGTCTGTCTCATATCACGAACAGCAAAGCGACC
CAAAGGTGGATAGTCTGAGAAGCTCTCAAC 7 (0.000180%)

CCTTGCCAGCTCCAGCAGCCTTCTTGTCCACTGCTTTGATGACACCCACCGCAACTGTCTGTCTCATATC
ACGAACAGCAAAGCGACCCAAAGGTGGATA 7 (0.000180%)

CCTTGGTATGTGCTTTCTCGTGTTACATCGCGCCATCATTGGTATATGGTTAGTGTGTTGGTTAGTAGGC
CTAGTATGAGGAGCGTTATGGAGTGGAAGT 55 (0.001412%)

CGAAAGCCTATAATCACTGCGCCTGTTCATAAGGGGATGGCCATGGCTAGGTTTATAGATAGTTGGGTG
GTTGGTGTAAATGAGTGAGGCAGGAGTCCGA 4 (0.000103%)

CGAAGGCCCCAGAAGTGACGCAGCCCTCTATGGGCCCGAATCTTCTTCAGTCGCTCCAGGTCTTCACGG
AGCTTGTTGTCCAGACCATTGGCTAGGACCT 7 (0.000180%)

CGACAGCGATTTCTAGGATAGTCAGTAGAATTAGAATTGTGAAGATGATAAGTGTAGAGGGAAGGTTA
ATGGTTGATATTGCTAGGGTGGCGCTTCCAAT 4 (0.000103%)

CGATCAATCTTTTCCTTCAGCTCAGCAAACTTGCATGCAATGTGAGCCGTGTGGCAATCCAATACAGGG
GCATAGCCGGCGCTTATTTGGCCTGGATGGT 12 (0.000308%)

CGATCAGAGGGCGATGAAGTTCTAGATCCATTGAGACAAGCTCTAGACAGTAGCATGCAGTCCCACAA
CTTGTACCAGCATCCCCAGCGTCTGGCATTCC 3 (0.000077%)

CGATGCCACCGCATTTATAGATCAGATGGCCAGTAGTGGTGGACTTGCCCGAATCTACGTGTCCAATGA
CGACAATGTTGATATGAGTCTTTTCCTTTCC 13 (0.000334%)

CGATGCCAGTGCCCCTGGGTGCAGGGATGAGGCGTACCAGCACAGAGCCGCAGCGGCCTGTCACCTTG
CAAGGGACAGTGTGGGGCTTGCCGATCTTGTT 20 (0.000513%)

CGATTTCTAGGATAGTCAGTAGAATTAGAATTGTGAAGATGATAAGTGTAGAGGGAAGGTTAATGGTTG
ATATTGCTAGGGTGGCGCTTCCAATTAGGTG 6 (0.000154%)

CGCATTTATAGATCAGATGGCCAGTAGTGGTGGACTTGCCCGAATCTACGTGTCCAATGACGACAATGT
TGATATGAGTCTTTTCCTTTCCCATTTTGGC 22 (0.000565%)

CGGACACGAAGGCCCCAGAAGTGACGCAGCCCTCTATGGGCCCGAATCTTCTTCAGTCGCTCCAGGTCT
TCACGGAGCTTGTTGTCCAGACCATTGGCTA 40 (0.001027%)

CGGAGACGATGCCAGTGCCCCTGGGTGCAGGGATGAGGCGTACCAGCACAGAGCCGCAGCGGCCTGTC
ACCTTGCAAGGGACAGTGTGGGGCTTGCCGAT20 (0.000513%)

CGGAGACGTCCAGTCCGGCGGGCAGCAATGAGACCCACTTTGCGGCCAGCAGGGGCATCTCTGCGGAT
GGTGGAGGGCTTGCCGATGTGCTGGTGGTTGC 26 (0.000667%)

CGGCGATCAGAGGGCGATGAAGTTCTAGATCCATTGAGACAAGCTCTAGACAGTAGCATGCAGTCCCA
CAACTTGTACCAGCATCCCCAGCGTCTGGCAT 340 (0.008728%)

CGGGTGGCACTGCCCACGGTGGGCGGGCGGGCCTCTCTACTCGAAGGTGACCACGTTTAGATTCTGAGA
CGGGAAGTGGAGGGTGAATAGGTCACGGCGG 3 (0.000077%)

CGGTTGTTGATGAGATATTTGGAGGTGGGGATCAATAGAGGGGGAAATAGAATGATCAGTACTGCGGC
GGGTAGGCCTAGGATTGTGGGGGCAATGAATG 24 (0.000616%)

CGTAGGTTTGGTCTAGGGTGTAGCCTGAGAATAGGGGAAATCAGTGAATGAAGCCTCCTATGATGGCA

AATACAGCTCCTATTGATAGGACATAGTGGAA5 (0.000128%)

CGTGTGGCAATCCAATACAGGGGCATAGCCGGCGCTTATTTGGCCTGGATGGTTCAGGATAATCACCTG
AGCAGTGAAGCCAGCTGCTTCCATTGGTGGG 6 (0.000154%)

CGTGTTACATCGCGCCATCATTGGTATATGGTTAGTGTGTTGGTTAGTAGGCCTAGTATGAGGAGCGTTA
TGGAGTGGAAGTGAAATCACATGGCTAGGC 12 (0.000308%)

CTAATACAATGCCAGTCAGGCCACCTACGGTGAAAAGAAAGATGAATCCTAGGGCTCAGAGCACTGCA
GCAGATCATTTCATATTGCTTCCGTGGAGTGT 12 (0.000308%)

CTAATGGCAGAATTTGGCTGTTTGGCTTCAACTCCTACTTTTTCCAGCACGATTCCTTTTGCATGAGAAG
CACCTCCAAAAGGGTTGGCCTTTAGGGCTG 8 (0.000205%)

CTAATTGGGGGGTAGGGGCTAGGCTGGAGTGGTAAAAGGCTCAGAAAAATCCTGCGAAGAAAAAAACT
TCTGAGGTAATAAATAGGATTATCCCGTATCG 7 (0.000180%)

CTACGGTGAAAAGAAAGATGAATCCTAGGGCTCAGAGCACTGCAGCAGATCATTTCATATTGCTTCCGT
GGAGTGTGGCGAGTCAGCTAAATACTTTGAC 10 (0.000257%)

CTACTAAACTTAAATGGCCAATTGAAACAAACAGTTCTGAGACCGTTCTTCCACCACTGATTAAGAGTG
GGGTGGCAGGTATTAGGGATAATATTCATTT 7 (0.000180%)

CTAGGATAGTCAGTAGAATTAGAATTGTGAAGATGATAAGTGTAGAGGGAAGGTTAATGGTTGATATT
GCTAGGGTGGCGCTTCCAATTAGGTGCATGAG10 (0.000257%)

CTAGGGTGTAGCCTGAGAATAGGGGAAATCAGTGAATGAAGCCTCCTATGATGGCAAATACAGCTCCT
ATTGATAGGACATAGTGGAAGTGAGCTACAAC 58 (0.001489%)

CTATAATCACTGCGCCTGTTCATAAGGGGATGGCCATGGCTAGGTTTATAGATAGTTGGGTGGTTGGTG
TAAATGAGTGAGGCAGGAGTCCGAGGAGGTT 6 (0.000154%)

CTCAAAATTCTGTGACAAATTTTTGGTCAAGTTGTTTCCATTAAAAAGTACTGATTTTAAAAACTAATAA
CTTAAAACTGCCACACGCAAAAAAGAAAAC 41 (0.001052%)

CTCAAATTTCCACAAGGAGATATCAATGGTGATACCACGTTCACGCTCAGCTTTCAGTTTATCCAAGAC
CCAGGCATACTTGAAGGAGCCCTTTCCCATC 120 (0.003080%)

CTCAAATTTTTCAATGGTTCTTTTGTCGATGCCACCGCATTTATAGATCAGATGGCCAGTAGTGGTGGAC
TTGCCCGAATCTACGTGTCCAATGACGACA 29 (0.000744%)

CTCAGCAAACTTGCATGCAATGTGAGCCGTGTGGCAATCCAATACAGGGGCATAGCCGGCGCTTATTTG
GCCTGGATGGTTCAGGATAATCACCTGAGCA 10 (0.000257%)

CTCATCCATGTGACCTTCTCTGGCATTCGGGCATTGGCTGTACCCTTCCGCTTACCTATGCCCATGTGCCT
GCCCTTCCGGCGGGCCAAGGTGTTTTTCC 5 (0.000128%)

CTCCAGCAGCCTTCTTGTCCACTGCTTTGATGACACCCACCGCAACTGTCTGTCTCATATCACGAACAGC
AAAGCGACCCAAAGGTGGATAGTCTGAGAA 11 (0.000282%)

CTCCTAATTGGGGGGTAGGGGCTAGGCTGGAGTGGTAAAAGGCTCAGAAAAATCCTGCGAAGAAAAAA
ACTTCTGAGGTAATAAATAGGATTATCCCGTA 9 (0.000231%)

CTCGGACACGAAGGCCCCAGAAGTGACGCAGCCCTCTATGGGCCCGAATCTTCTTCAGTCGCTCCAGGT
CTTCACGGAGCTTGTTGTCCAGACCATTGGC 16 (0.000411%)

CTCGTGTTACATCGCGCCATCATTGGTATATGGTTAGTGTGTTGGTTAGTAGGCCTAGTATGAGGAGCGT
TATGGAGTGGAAGTGAAATCACATGGCTAG 31 (0.000796%)

CTCTGGCATTCGGGCATTGGCTGTACCCTTCCGCTTACCTATGCCCATGTGCCTGCCCTTCCGGCGGGCC
AAGGTGTTTTTCCGGCATCGAGCCCGGGAA 12 (0.000308%)

CTCTTATCAAGTCAGCACACACCTTTTCCAAGGATTTACGTTGCGGCTTGTTAGGGTGATTCGAATTCG
GTGAATTGCCACCTCCGGCTCCACGGGTGT 399 (0.010242%)

CTGAACCAATGCACCATCTGTAAAGTTGCAGACAGTCTGAGTTTTTCTGCCATCAGCTGTGGTTTCTTCA
AACTTCTCTCCCAGGGTACAAGAAAACTGT 3 (0.000077%)

CTGACTTCCTTAACAATTTCCTCATATCTCTTCTGGCTGTAGGGTGGCTCAGTGGAATCCATTTTGTTAAC
ACCGACAATTAGTTGTTTCACACCCAGTG 61 (0.001566%)

CTGAGCAGTGAAGCCAGCTGCTTCCATTGGTGGGTCATTTTTGCTGTCACCAGCAACGTTGCCACGACG
AACATCCTTGACAGACACATTCTTGACATTG 5 (0.000128%)

CTGAGCTTTCTGGGCAGACTTGGTGACCTTGCCAGCTCCAGCAGCCTTCTTGTCCACTGCTTTGATGACA
CCCACCGCAACTGTCTGTCTCATATCACGA 41 (0.001052%)

CTGCACAGTCTTGGTTCCCCGGAGACGTCCAGTCCGGCGGGCAGCAATGAGACCCACTTTGCGGCCAGC
AGGGGCATCTCTGCGGATGGTGGAGGGCTTG 311 (0.007983%)

CTGCCCACGGTGGGCGGGCGGGCCTCTCTACTCGAAGGTGACCACGTTTAGATTCTGAGACGGGAAGTG
GAGGGTGAATAGGTCACGGCGGCCTTTTTTT 3 (0.000077%)

CTGCGCCTGTTCATAAGGGGATGGCCATGGCTAGGTTTATAGATAGTTGGGTGGTTGGTGTAAATGAGT
GAGGCAGGAGTCCGAGGAGGTTAGTTGTGGC 6 (0.000154%)

CTGCGGGTGGCACTGCCCACGGTGGGCGGGCGGGCCTCTCTACTCGAAGGTGACCACGTTTAGATTCTG
AGACGGGAAGTGGAGGGTGAATAGGTCACGG 22 (0.000565%)

CTGGCATTCGGGCATTGGCTGTACCCTTCCGCTTACCTATGCCCATGTGCCTGCCCTTCCGGCGGGCCAA
GGTGTTTTTCCGGCATCGAGCCCGGGAATG 21 (0.000539%)

CTGGGCAGACTTGGTGACCTTGCCAGCTCCAGCAGCCTTCTTGTCCACTGCTTTGATGACACCCACCGCA
ACTGTCTGTCTCATATCACGAACAGCAAAG 25 (0.000642%)

CTGGGTGCAGGGATGAGGCGTACCAGCACAGAGCCGCAGCGGCCTGTCACCTTGCAAGGGACAGTGTG
GGGCTTGCCGATCTTGTTCCCCCAGTAGCCTC 16 (0.000411%)

CTGGTCTCAAATTTCCACAAGGAGATATCAATGGTGATACCACGTTCACGCTCAGCTTTCAGTTTATCCA
AGACCCAGGCATACTTGAAGGAGCCCTTTC 55 (0.001412%)

CTGTGACAAATTTTTGGTCAAGTTGTTTCCATTAAAAAGTACTGATTTTAAAAACTAATAACTTAAAACT
GCCACACGCAAAAAAGAAAACCAAAGTGGT 32 (0.000821%)

CTGTTGGGGGCCAGTGCCCTCCTAATTGGGGGGTAGGGGCTAGGCTGGAGTGGTAAAAGGCTCAGAAA
AATCCTGCGAAGAAAAAAACTTCTGAGGTAAT 803 (0.020613%)

CTGTTTTTAAGCCTAATGTGGGGACAGCTCATGAGTGCAAGACGTCTTGTGATGTAATTATTATACGAAT
GGGGGCTTCAATCGGGAGTACTACTCGATT 824 (0.021152%)

CTTAACAATTTCCTCATATCTCTTCTGGCTGTAGGGTGGCTCAGTGGAATCCATTTTGTTAACACCGACA
ATTAGTTGTTTCACACCCAGTGTGTAAGCC 21 (0.000539%)

CTTAATGTAAGTGCTGACTTCCTTAACAATTTCCTCATATCTCTTCTGGCTGTAGGGTGGCTCAGTGGAA
TCCATTTTGTTAACACCGACAATTAGTTGT9 (0.000231%)

CTTACACACTTCCTAATGGCAGAATTTGGCTGTTTGGCTTCAACTCCTACTTTTTCCAGCACGATTCCTTT
TGCATGAGAAGCACCTCCAAAAGGGTTGG 563 (0.014452%)

CTTAGGCACAGGTGCGGAGACGATGCCAGTGCCCCTGGGTGCAGGGATGAGGCGTACCAGCACAGAGC
CGCAGCGGCCTGTCACCTTGCAAGGGACAGTG 18 (0.000462%)

CTTATCAAGTCAGCACACACCTTTTCCAAGGATTTTACGTTGCGGCTTGTTAGGGTGATTCGAATTCGGT
GAATTGCCACCTCCGGCTCCACGGGTGTTT 16 (0.000411%)

CTTCAAGAATTTAGGGCCATCTTCCAGCTTTTTACCAGAACGGCGATCAATCTTTTCCTTCAGCTCAGCA
AACTTGCATGCAATGTGAGCCGTGTGGCAA 6 (0.000154%)

CTTCAGCTCAGCAAACTTGCATGCAATGTGAGCCGTGTGGCAATCCAATACAGGGGCATAGCCGGCGCT
TATTTGGCCTGGATGGTTCAGGATAATCACC 33 (0.000847%)

CTTCCTAATGGCAGAATTTGGCTGTTTGGCTTCAACTCCTACTTTTTCCAGCACGATTCCTTTTGCATGAG
AAGCACCTCCAAAAGGGTTGGCCTTTAGG 8 (0.000205%)

CTTCCTTAACAATTTCCTCATATCTCTTCTGGCTGTAGGGTGGCTCAGTGGAATCCATTTTGTTAACACC
GACAATTAGTTGTTTCACACCCAGTGTGTA 50 (0.001283%)

CTTCTCAAATTTTTCAATGGTTCTTTTGTCGATGCCACCGCATTTATAGATCAGATGGCCAGTAGTGGTG
GACTTGCCCGAATCTACGTGTCCAATGACG 910 (0.023359%)

CTTCTCTGGCATTCGGGCATTGGCTGTACCCTTCCGCTTACCTATGCCCATGTGCCTGCCCTTCCGGCGG
GCCAAGGTGTTTTTCCGGCATCGAGCCCGG 11 (0.000282%)

CTTCTGAGCTTTCTGGGCAGACTTGGTGACCTTGCCAGCTCCAGCAGCCTTCTTGTCCACTGCTTTGATG
ACACCCACCGCAACTGTCTGTCTCATATCA 14 (0.000359%)

CTTCTTAGGCACAGGTGCGGAGACGATGCCAGTGCCCCTGGGTGCAGGGATGAGGCGTACCAGCACAG
AGCCGCAGCGGCCTGTCACCTTGCAAGGGACA 5 (0.000128%)

CTTCTTGTCCACTGCTTTGATGACACCCACCGCAACTGTCTGTCTCATATCACGAACAGCAAAGCGACCC
AAAGGTGGATAGTCTGAGAAGCTCTCAACA 9 (0.000231%)

CTTGCATGCAATGTGAGCCGTGTGGCAATCCAATACAGGGGCATAGCCGGCGCTTATTTGGCCTGGATG
GTTCAGGATAATCACCTGAGCAGTGAAGCCA 15 (0.000385%)

CTTGCCAGCTCCAGCAGCCTTCTTGTCCACTGCTTTGATGACACCCACCGCAACTGTCTGTCTCATATCA
CGAACAGCAAAGCGACCCAAAGGTGGATAG 27 (0.000693%)

CTTGCTGGTCTCAAATTTCCACAAGGAGATATCAATGGTGATACCACGTTCACGCTCAGCTTTCAGTTTA
TCCAAGACCCAGGCATACTTGAAGGAGCCC 22 (0.000565%)

CTTGGTATGTGCTTTCTCGTGTTACATCGCGCCATCATTGGTATATGGTTAGTGTGTTGGTTAGTAGGCC
TAGTATGAGGAGCGTTATGGAGTGGAAGTG 26 (0.000667%)

CTTGGTGACCTTGCCAGCTCCAGCAGCCTTCTTGTCCACTGCTTTGATGACACCCACCGCAACTGTCTGT
CTCATATCACGAACAGCAAAGCGACCCAAA 22 (0.000565%)

CTTGGTTCCCCGGAGACGTCCAGTCCGGCGGGCAGCAATGAGACCCACTTTGCGGCCAGCAGGGGCATC
TCTGCGGATGGTGGAGGGCTTGCCGATGTGC 26 (0.000667%)

CTTGTCCACTGCTTTGATGACACCCACCGCAACTGTCTGTCTCATATCACGAACAGCAAAGCGACCCAA
AGGTGGATAGTCTGAGAAGCTCTCAACACAC 31 (0.000796%)

CTTTCTCGTGTTACATCGCGCCATCATTGGTATATGGTTAGTGTGTTGGTTAGTAGGCCTAGTATGAGGA

GCGTTATGGAGTGGAAGTGAAATCACATGG 10 (0.000257%)

CTTTCTGGGCAGACTTGGTGACCTTGCCAGCTCCAGCAGCCTTCTTGTCCACTGCTTTGATGACACCCAC
CGCAACTGTCTGTCTCATATCACGAACAGC 6 (0.000154%)

CTTTGCATAATCCAGGGAATCATAAATCATGCCAAAGCCAGTTGTCTTGCCACCACCAAAATGAGTTCT
GAATCCAAATACAAAGATGACATCCGGTGTG 9 (0.000231%)

CTTTTACTACTAAACTTAAATGGCCAATTGAAACAAACAGTTCTGAGACCGTTCTTCCACCACTGATTAA
GAGTGGGGTGGCAGGTATTAGGGATAATAT 34 (0.000873%)

CTTTTCCTTCAGCTCAGCAAACTTGCATGCAATGTGAGCCGTGTGGCAATCCAATACAGGGGCATAGCC
GGCGCTTATTTGGCCTGGATGGTTCAGGATA 104 (0.002670%)

CTTTTGTCGATGCCACCGCATTTATAGATCAGATGGCCAGTAGTGGTGGACTTGCCCGAATCTACGTGTC
CAATGACGACAATGTTGATATGAGTCTTTT 66 (0.001694%)

CTTTTTACCAGAACGGCGATCAATCTTTTCCTTCAGCTCAGCAAACTTGCATGCAATGTGAGCCGTGTGG
CAATCCAATACAGGGGCATAGCCGGCGCTT 5 (0.000128%)

CTTTTTTAATGGGTCTCAAAATTCTGTGACAAATTTTTGGTCAAGTTGTTTCCATTAAAAAGTACTGATTT
TAAAAACTAATAACTTAAAACTGCCACAC 430 (0.011038%)

GAACCATATCAACAATGGCAGCATCACCAGACTTCAAGAATTTAGGGCCATCTTCCAGCTTTTTACCAG
AACGGCGATCAATCTTTTCCTTCAGCTCAGC 23 (0.000590%)

GAATGTCACAGGTCCAGGGCAGAGGACCAACATGGGCATTTTGTTTATGAGCAAGGTGGGTCTCAGAG
GTGATCGGCGATCAGAGGGCGATGAAGTTCTA 14 (0.000359%)

GAATTTAGGGCCATCTTCCAGCTTTTTACCAGAACGGCGATCAATCTTTTCCTTCAGCTCAGCAAACTTG
CATGCAATGTGAGCCGTGTGGCAATCCAAT 8 (0.000205%)

GAATTTGGCTGTTTGGCTTCAACTCCTACTTTTTCCAGCACGATTCCTTTTGCATGAGAAGCACCTCCAA
AAGGGTTGGCCTTTAGGGCTGTGCCCAAAT 5 (0.000128%)

GACAAATTTTTGGTCAAGTTGTTTCCATTAAAAAGTACTGATTTTAAAAACTAATAACTTAAAACTGCCA
CACGCAAAAAAGAAAACCAAAGTGGTCCAC 10 (0.000257%)

GACACGAAGGCCCCAGAAGTGACGCAGCCCTCTATGGGCCCGAATCTTCTTCAGTCGCTCCAGGTCTTC
ACGGAGCTTGTTGTCCAGACCATTGGCTAGG 5 (0.000128%)

GACAGCGATTTCTAGGATAGTCAGTAGAATTAGAATTGTGAAGATGATAAGTGTAGAGGGAAGGTTAA
TGGTTGATATTGCTAGGGTGGCGCTTCCAATT 6 (0.000154%)

GACCAACATGGGCATTTTGTTTATGAGCAAGGTGGGTCTCAGAGGTGATCGGCGATCAGAGGGCGATG
AAGTTCTAGATCCATTGAGACAAGCTCTAGAC 9 (0.000231%)

GACCTTCTCTGGCATTCGGGCATTGGCTGTACCCTTCCGCTTACCTATGCCCATGTGCCTGCCCTTCCGG
CGGGCCAAGGTGTTTTTCCGGCATCGAGCC 5 (0.000128%)

GACCTTGCCAGCTCCAGCAGCCTTCTTGTCCACTGCTTTGATGACACCCACCGCAACTGTCTGTCTCATA
TCACGAACAGCAAAGCGACCCAAAGGTGGA 5 (0.000128%)

GACGATGCCAGTGCCCCTGGGTGCAGGGATGAGGCGTACCAGCACAGAGCCGCAGCGGCCTGTCACCT
TGCAAGGGACAGTGTGGGGCTTGCCGATCTTG 17 (0.000436%)

GACGTCCAGTCCGGCGGGCAGCAATGAGACCCACTTTGCGGCCAGCAGGGGCATCTCTGCGGATGGTG
GAGGGCTTGCCGATGTGCTGGTGGTTGCCACC 6 (0.000154%)

GACTTCAAGAATTTAGGGCCATCTTCCAGCTTTTACCAGAACGGCGATCAATCTTTTCCTTCAGCTCAG CAAACTTGCATGCAATGTGAGCCGTGTGGC 14 (0.000359%)

GACTTCCTTAACAATTTCCTCATATCTCTTCTGGCTGTAGGGTGGCTCAGTGGAATCCATTTTGTTAACA CCGACAATTAGTTGTTTCACACCCAGTGTG 7 (0.000180%)

GACTTGGTGACCTTGCCAGCTCCAGCAGCCTTCTTGTCCACTGCTTTGATGACACCCACCGCAACTGTCT GTCTCATATCACGAACAGCAAAGCGACCCA 28 (0.000719%)

GAGACGATGCCAGTGCCCCTGGGTGCAGGGATGAGGCGTACCAGCACAGAGCCGCAGCGGCCTGTCAC CTTGCAAGGGACAGTGTGGGGCTTGCCGATCT 3 (0.000077%)

GAGAGAGTGAGGAGAAGGCTTACGTTTAGTGAGGGAGAGATTTGGTATATGATTGAGATGGGGGCTAG TTTTTGTCATGTGAGAAGAAGCAGGCCGGATG 9 (0.000231%)

GAGCAAGGTGGGTCTCAGAGGTGATCGGCGATCAGAGGGCGATGAAGTTCTAGATCCATTGAGACAAG CTCTAGACAGTAGCATGCAGTCCCACAACTTG 6 (0.000154%)

GAGCAGTGAAGCCAGCTGCTTCCATTGGTGGGTCATTTTTGCTGTCACCAGCAACGTTGCCACGACGAA CATCCTTGACAGACACATTCTTGACATTGAA 6 (0.000154%)

GAGCCCCATTGTGTTGTGGTAAATATGTAGAGGGAGTATAGGGCTGTGACTAGTATGTTGAGTCCTGTA AGTAGGAGAGTGATATTTGATCAGGAGAACG 5 (0.000128%)

GAGCCGTGTGGCAATCCAATACAGGGGCATAGCCGGCGCTTATTTGGCCTGGATGGTTCAGGATAATCA CCTGAGCAGTGAAGCCAGCTGCTTCCATTGG 10 (0.000257%)

GAGCTTTCTGGGCAGACTTGGTGACCTTGCCAGCTCCAGCAGCCTTCTTGTCCACTGCTTTGATGACACC CACCGCAACTGTCTGTCTCATATCACGAAC 22 (0.000565%)

GAGGGAGTATAGGGCTGTGACTAGTATGTTGAGTCCTGTAAGTAGGAGAGTGATATTTGATCAGGAGA ACGTGGTTACTAGCACAGAGAGTTCTCCCAGT 12 (0.000308%)

GAGTAGTTCCCTGCTAAGGGAGGGTAGACTGTTCAACCTGTTCCTGCTCCGGCCTCCACTATAGCAGAT GCGAGCAGGAGTAGGAGAGAGGGAGGTAAGA 11 (0.000282%)

GAGTGAGCCCCATTGTGTTGTGGTAAATATGTAGAGGGAGTATAGGGCTGTGACTAGTATGTTGAGTCC TGTAAGTAGGAGAGTGATATTTGATCAGGAG 9 (0.000231%)

GATAATATTCATTTAGCCTTCTGAGCTTTCTGGGCAGACTTGGTGACCTTGCCAGCTCCAGCAGCCTTCT TGTCCACTGCTTTGATGACACCCACCGCAA 22 (0.000565%)

GATAGTCAGTAGAATTAGAATTGTGAAGATGATAAGTGTAGAGGGAAGGTTAATGGTTGATATTGCTA GGGTGGCGCTTCCAATTAGGTGCATGAGTAGG3 (0.000077%)

GATCAATCTTTTCCTTCAGCTCAGCAAACTTGCATGCAATGTGAGCCGTGTGGCAATCCAATACAGGGG CATAGCCGGCGCTTATTTGGCCTGGATGGTT 31 (0.000796%)

GATCGGAAGAGCACACGTCTGAACTCCAGTCACATCGCAATTGCGATCTGGGGGGGGGGGGGGGGGG GGGGGGGGGGGGGGGGGGGGGGGGGGGGGGGGGGGGGGGGGGGGGGGGGGGGGGGGGGGGGGGGGG GGGGGGGGGGGGGGGG 939 (0.035915%)

GATGCCAGTGCCCCTGGGTGCAGGGATGAGGCGTACCAGCACAGAGCCGCAGCGGCCTGTCACCTTGC AAGGGACAGTGTGGGGCTTGCCGATCTTGTTC 7 (0.000180%)

GATGGTTCAGGATAATCACCTGAGCAGTGAAGCCAGCTGCTTCCATTGGTGGGTCATTTTTGCTGTCACC AGCAACGTTGCCACGACGAACATCCTTGAC 15 (0.000385%)

GATTGAGAGAGTGAGGAGAAGGCTTACGTTTAGTGAGGGAGAGATTTGGTATATGATTGAGATGGGGG
CTAGTTTTTGTCATGTGAGAAGAAGCAGGCCG 326 (0.008368%)

GCAAACTTGCATGCAATGTGAGCCGTGTGGCAATCCAATACAGGGGCATAGCCGGCGCTTATTTGGCCT
GGATGGTTCAGGATAATCACCTGAGCAGTGA 6 (0.000154%)

GCAATCCAATACAGGGGCATAGCCGGCGCTTATTTGGCCTGGATGGTTCAGGATAATCACCTGAGCAGT
GAAGCCAGCTGCTTCCATTGGTGGGTCATTT 25 (0.000642%)

GCAATGTGAGCCGTGTGGCAATCCAATACAGGGGCATAGCCGGCGCTTATTTGGCCTGGATGGTTCAGG
ATAATCACCTGAGCAGTGAAGCCAGCTGCTT 28 (0.000719%)

GCACAGTCTTGGTTCCCCGGAGACGTCCAGTCCGGCGGGCAGCAATGAGACCCACTTTGCGGCCAGCAG
GGGCATCTCTGCGGATGGTGGAGGGCTTGCC 12 (0.000308%)

GCACTGCCCACGGTGGGCGGGCGGGCCTCTCTACTCGAAGGTGACCACGTTTAGATTCTGAGACGGGAA
GTGGAGGGTGAATAGGTCACGGCGGCCTTTT 14 (0.000359%)

GCAGAATTTGGCTGTTTGGCTTCAACTCCTACTTTTTCCAGCACGATTCCTTTTGCATGAGAAGCACCTC
CAAAAGGGTTGGCCTTTAGGGCTGTGCCCA 28 (0.000719%)

GCAGACTTGGTGACCTTGCCAGCTCCAGCAGCCTTCTTGTCCACTGCTTTGATGACACCCACCGCAACTG
TCTGTCTCATATCACGAACAGCAAAGCGAC 78 (0.002002%)

GCAGCATCACCAGACTTCAAGAATTTAGGGCCATCTTCCAGCTTTTTACCAGAACGGCGATCAATCTTTT
CCTTCAGCTCAGCAAACTTGCATGCAATGT 27 (0.000693%)

GCAGCCTTCTTGTCCACTGCTTTGATGACACCCACCGCAACTGTCTGTCTCATATCACGAACAGCAAAGC
GACCCAAAGGTGGATAGTCTGAGAAGCTCT 8 (0.000205%)

GCAGGGATGAGGCGTACCAGCACAGAGCCGCAGCGGCCTGTCACCTTGCAAGGGACAGTGTGGGGCTT
GCCGATCTTGTTCCCCCAGTAGCCTCTGCGCA 13 (0.000334%)

GCATAATCCAGGGAATCATAAATCATGCCAAAGCCAGTTGTCTTGCCACCACCAAAATGAGTTCTGAAT
CCAAATACAAAGATGACATCCGGTGTGGTCT 5 (0.000128%)

GCATCACCAGACTTCAAGAATTTAGGGCCATCTTCCAGCTTTTTACCAGAACGGCGATCAATCTTTTCCT
TCAGCTCAGCAAACTTGCATGCAATGTGAG 25 (0.000642%)

GCATGCAATGTGAGCCGTGTGGCAATCCAATACAGGGGCATAGCCGGCGCTTATTTGGCCTGGATGGTT
CAGGATAATCACCTGAGCAGTGAAGCCAGCT 7 (0.000180%)

GCATTTTGTTTATGAGCAAGGTGGGTCTCAGAGGTGATCGGCGATCAGAGGGCGATGAAGTTCTAGATC
CATTGAGACAAGCTCTAGACAGTAGCATGCA 19 (0.000488%)

GCATTTTTAATCTTAGAGCGAAAGCCTATAATCACTGCGCCTGTTCATAAGGGGATGGCCATGGCTAGG
TTTATAGATAGTTGGGTGGTTGGTGTAAATG 16 (0.000411%)

GCCAATTTTCTTAATGTAAGTGCTGACTTCCTTAACAATTTCCTCATATCTCTTCTGGCTGTAGGGTGGCT
CAGTGGAATCCATTTTGTTAACACCGACA 45 (0.001155%)

GCCACCGCATTTATAGATCAGATGGCCAGTAGTGGTGGACTTGCCCGAATCTACGTGTCCAATGACGAC
AATGTTGATATGAGTCTTTTCCTTTCCCATT 6 (0.000154%)

GCCACCTACGGTGAAAAGAAAGATGAATCCTAGGGCTCAGAGCACTGCAGCAGATCATTTCATATTGCT
TCCGTGGAGTGTGGCGAGTCAGCTAAATACT 34 (0.000873%)

GCCAGCTCCAGCAGCCTTCTTGTCCACTGCTTTGATGACACCCACCGCAACTGTCTGTCTCATATCACGA
ACAGCAAAGCGACCCAAAGGTGGATAGTCT 49 (0.001258%)

GCCAGGAACCATATCAACAATGGCAGCATCACCAGACTTCAAGAATTTAGGGCCATCTTCCAGCTTTTT
ACCAGAACGGCGATCAATCTTTTCCTTCAGC 680 (0.017455%)

GCCAGTCAGGCCACCTACGGTGAAAAGAAAGATGAATCCTAGGGCTCAGAGCACTGCAGCAGATCATT
TCATATTGCTTCCGTGGAGTGTGGCGAGTCAG 23 (0.000590%)

GCCAGTGCCCCTGGGTGCAGGGATGAGGCGTACCAGCACAGAGCCGCAGCGGCCTGTCACCTTGCAAG
GGACAGTGTGGGGCTTGCCGATCTTGTTCCCC 14 (0.000359%)

GCCAGTGCCCTCCTAATTGGGGGGTAGGGGCTAGGCTGGAGTGGTAAAAGGCTCAGAAAAATCCTGCG
AAGAAAAAAACTTCTGAGGTAATAAATAGGAT 7 (0.000180%)

GCCCCATTGTGTTGTGGTAAATATGTAGAGGGAGTATAGGGCTGTGACTAGTATGTTGAGTCCTGTAAG
TAGGAGAGTGATATTTGATCAGGAGAACGTG 20 (0.000513%)

GCCCCTGGGTGCAGGGATGAGGCGTACCAGCACAGAGCCGCAGCGGCCTGTCACCTTGCAAGGGACAG
TGTGGGGCTTGCCGATCTTGTTCCCCCAGTAG 7 (0.000180%)

GCCCTCCTAATTGGGGGGTAGGGGCTAGGCTGGAGTGGTAAAAGGCTCAGAAAAATCCTGCGAAGAAA
AAAACTTCTGAGGTAATAAATAGGATTATCCC 7 (0.000180%)

GCCGTGTGGCAATCCAATACAGGGGCATAGCCGGCGCTTATTTGGCCTGGATGGTTCAGGATAATCACC
TGAGCAGTGAAGCCAGCTGCTTCCATTGGTG 29 (0.000744%)

GCCTAATGTGGGGACAGCTCATGAGTGCAAGACGTCTTGTGATGTAATTATTATACGAATGGGGGCTTC
AATCGGGAGTACTACTCGATTGTCAACGTCA 6 (0.000154%)

GCCTCGGACACGAAGGCCCCAGAAGTGACGCAGCCCTCTATGGGCCCGAATCTTCTTCAGTCGCTCCAG
GTCTTCACGGAGCTTGTTGTCCAGACCATTG 8 (0.000205%)

GCCTGAGAATAGGGGAAATCAGTGAATGAAGCCTCCTATGATGGCAAATACAGCTCCTATTGATAGGA
CATAGTGGAAGTGAGCTACAACGTAGTACGTG 7 (0.000180%)

GCCTGTTCATAAGGGGATGGCCATGGCTAGGTTTATAGATAGTTGGGTGGTTGGTGTAAATGAGTGAGG
CAGGAGTCCGAGGAGGTTAGTTGTGGCAATA 4 (0.000103%)

GCCTTCTGAGCTTTCTGGGCAGACTTGGTGACCTTGCCAGCTCCAGCAGCCTTCTTGTCCACTGCTTTGA
TGACACCCACCGCAACTGTCTGTCTCATAT 38 (0.000975%)

GCCTTCTTGTCCACTGCTTTGATGACACCCACCGCAACTGTCTGTCTCATATCACGAACAGCAAAGCGAC
CCAAAGGTGGATAGTCTGAGAAGCTCTCAA 18 (0.000462%)

GCCTTGGTATGTGCTTTCTCGTGTTACATCGCGCCATCATTGGTATATGGTTAGTGTGTTGGTTAGTAGG
CCTAGTATGAGGAGCGTTATGGAGTGGAAG 19 (0.000488%)

GCGACAGCGATTTCTAGGATAGTCAGTAGAATTAGAATTGTGAAGATGATAAGTGTAGAGGGAAGGTT
AATGGTTGATATTGCTAGGGTGGCGCTTCCAA 714 (0.018328%)

GCGATCAATCTTTTCCTTCAGCTCAGCAAACTTGCATGCAATGTGAGCCGTGTGGCAATCCAATACAGG
GGCATAGCCGGCGCTTATTTGGCCTGGATGG 22 (0.000565%)

GCGATCAGAGGGCGATGAAGTTCTAGATCCATTGAGACAAGCTCTAGACAGTAGCATGCAGTCCCACA
ACTTGTACCAGCATCCCCAGCGTCTGGCATTC 4 (0.000103%)

GCGATTTCTAGGATAGTCAGTAGAATTAGAATTGTGAAGATGATAAGTGTAGAGGGAAGGTTAATGGTT

GATATTGCTAGGGTGGCGCTTCCAATTAGGT 60 (0.001540%)

GCGCCTGTTCATAAGGGGATGGCCATGGCTAGGTTTATAGATAGTTGGGTGGTTGGTGTAAATGAGTGA
GGCAGGAGTCCGAGGAGGTTAGTTGTGGCAA 4 (0.000103%)

GCGGAGACGATGCCAGTGCCCCTGGGTGCAGGGATGAGGCGTACCAGCACAGAGCCGCAGCGGCCTGT
CACCTTGCAAGGGACAGTGTGGGGCTTGCCGA 28 (0.000719%)

GCGGGTGGCACTGCCCACGGTGGGCGGGCGGGCCTCTCTACTCGAAGGTGACCACGTTTAGATTCTGAG
ACGGGAAGTGGAGGGTGAATAGGTCACGGCG 22 (0.000565%)

GCGTAGGTTTGGTCTAGGGTGTAGCCTGAGAATAGGGGAAATCAGTGAATGAAGCCTCCTATGATGGC
AAATACAGCTCCTATTGATAGGACATAGTGGA 18 (0.000462%)

GCTAATACAATGCCAGTCAGGCCACCTACGGTGAAAAGAAAGATGAATCCTAGGGCTCAGAGCACTGC
AGCAGATCATTTCATATTGCTTCCGTGGAGTG 14 (0.000359%)

GCTCAGCAAACTTGCATGCAATGTGAGCCGTGTGGCAATCCAATACAGGGGCATAGCCGGCGCTTATTT
GGCCTGGATGGTTCAGGATAATCACCTGAGC 8 (0.000205%)

GCTCCAGCAGCCTTCTTGTCCACTGCTTTGATGACACCCACCGCAACTGTCTGTCTCATATCACGAACAG
CAAAGCGACCCAAAGGTGGATAGTCTGAGA 14 (0.000359%)

GCTGAACCAATGCACCATCTGTAAAGTTGCAGACAGTCTGAGTTTTTCTGCCATCAGCTGTGGTTTCTTC
AAACTTCTCTCCCAGGGTACAAGAAAACTG 5 (0.000128%)

GCTGACTTCCTTAACAATTTCCTCATATCTCTTCTGGCTGTAGGGTGGCTCAGTGGAATCCATTTTGTTA
ACACCGACAATTAGTTGTTTCACACCCAGT 77 (0.001977%)

GCTGGTCTCAAATTTCCACAAGGAGATATCAATGGTGATACCACGTTCACGCTCAGCTTTCAGTTTATCC
AAGACCCAGGCATACTTGAAGGAGCCCTTT 38 (0.000975%)

GCTGTGACTAGTATGTTGAGTCCTGTAAGTAGGAGAGTGATATTTGATCAGGAGAACGTGGTTACTAGC
ACAGAGAGTTCTCCCAGTAGGTTAATAGTGG 11 (0.000282%)

GCTTCTTAGGCACAGGTGCGGAGACGATGCCAGTGCCCCTGGGTGCAGGGATGAGGCGTACCAGCACA
GAGCCGCAGCGGCCTGTCACCTTGCAAGGGAC 10 (0.000257%)

GCTTTCTCGTGTTACATCGCGCCATCATTGGTATATGGTTAGTGTGTTGGTTAGTAGGCCTAGTATGAGG
AGCGTTATGGAGTGGAAGTGAAATCACATG 13 (0.000334%)

GCTTTCTGGGCAGACTTGGTGACCTTGCCAGCTCCAGCAGCCTTCTTGTCCACTGCTTTGATGACACCCA
CCGCAACTGTCTGTCTCATATCACGAACAG 16 (0.000411%)

GCTTTTTACCAGAACGGCGATCAATCTTTTCCTTCAGCTCAGCAAACTTGCATGCAATGTGAGCCGTGTG
GCAATCCAATACAGGGGCATAGCCGGCGCT 10 (0.000257%)

GGAACCATATCAACAATGGCAGCATCACCAGACTTCAAGAATTTAGGGCCATCTTCCAGCTTTTTACCA
GAACGGCGATCAATCTTTTCCTTCAGCTCAG 32 (0.000821%)

GGACACGAAGGCCCCAGAAGTGACGCAGCCCTCTATGGGCCCGAATCTTCTTCAGTCGCTCCAGGTCTT
CACGGAGCTTGTTGTCCAGACCATTGGCTAG 12 (0.000308%)

GGACCAACATGGGCATTTTGTTTATGAGCAAGGTGGGTCTCAGAGGTGATCGGCGATCAGAGGGCGAT
GAAGTTCTAGATCCATTGAGACAAGCTCTAGA 10 (0.000257%)

GGAGACGATGCCAGTGCCCCTGGGTGCAGGGATGAGGCGTACCAGCACAGAGCCGCAGCGGCCTGTCA
CCTTGCAAGGGACAGTGTGGGGCTTGCCGATC 52 (0.001335%)

GGAGACGTCCAGTCCGGCGGGCAGCAATGAGACCCACTTTGCGGCCAGCAGGGGCATCTCTGCGGATG
GTGGAGGGCTTGCCGATGTGCTGGTGGTTGCC 10 (0.000257%)

GGAGATATCAATGGTGATACCACGTTCACGCTCAGCTTTCAGTTTATCCAAGACCCAGGCATACTTGAA
GGAGCCCTTTCCCATCTCAGCAGCCTCCTTC 21 (0.000539%)

GGAGTAGTTCCCTGCTAAGGGAGGGTAGACTGTTCAACCTGTTCCTGCTCCGGCCTCCACTATAGCAGA
TGCGAGCAGGAGTAGGAGAGAGGGAGGTAAG30 (0.000770%)

GGAGTATAGGGCTGTGACTAGTATGTTGAGTCCTGTAAGTAGGAGAGTGATATTTGATCAGGAGAACGT
GGTTACTAGCACAGAGAGTTCTCCCAGTAGG 17 (0.000436%)

GGATAATATTCATTTAGCCTTCTGAGCTTTCTGGGCAGACTTGGTGACCTTGCCAGCTCCAGCAGCCTTC
TTGTCCACTGCTTTGATGACACCCACCGCA 167 (0.004287%)

GGATAATCACCTGAGCAGTGAAGCCAGCTGCTTCCATTGGTGGGTCATTTTTGCTGTCACCAGCAACGT
TGCCACGACGAACATCCTTGACAGACACATT 35 (0.000898%)

GGATAGTCAGTAGAATTAGAATTGTGAAGATGATAAGTGTAGAGGGAAGGTTAATGGTTGATATTGCT
AGGGTGGCGCTTCCAATTAGGTGCATGAGTAG6 (0.000154%)

GGATGGTTCAGGATAATCACCTGAGCAGTGAAGCCAGCTGCTTCCATTGGTGGGTCATTTTTGCTGTCA
CCAGCAACGTTGCCACGACGAACATCCTTGA 856 (0.021973%)

GGATTTTGGCGTAGGTTTGGTCTAGGGTGTAGCCTGAGAATAGGGGAAATCAGTGAATGAAGCCTCCTA
TGATGGCAAATACAGCTCCTATTGATAGGAC 13 (0.000334%)

GGCAATCCAATACAGGGGCATAGCCGGCGCTTATTTGGCCTGGATGGTTCAGGATAATCACCTGAGCAG
TGAAGCCAGCTGCTTCCATTGGTGGGTCATT 55 (0.001412%)

GGCACTGCCCACGGTGGGCGGGCGGGCCTCTCTACTCGAAGGTGACCACGTTTAGATTCTGAGACGGGA
AGTGGAGGGTGAATAGGTCACGGCGGCCTTT 10 (0.000257%)

GGCAGAATTTGGCTGTTTGGCTTCAACTCCTACTTTTTCCAGCACGATTCCTTTTGCATGAGAAGCACCT
CCAAAAGGGTTGGCCTTTAGGGCTGTGCCC 12 (0.000308%)

GGCAGACTTGGTGACCTTGCCAGCTCCAGCAGCCTTCTTGTCCACTGCTTTGATGACACCCACCGCAACT
GTCTGTCTCATATCACGAACAGCAAAGCGA 129 (0.003311%)

GGCAGAGGACCAACATGGGCATTTTGTTTATGAGCAAGGTGGGTCTCAGAGGTGATCGGCGATCAGAG
GGCGATGAAGTTCTAGATCCATTGAGACAAGC6 (0.000154%)

GGCAGCATCACCAGACTTCAAGAATTTAGGGCCATCTTCCAGCTTTTTACCAGAACGGCGATCAATCTT
TTCCTTCAGCTCAGCAAACTTGCATGCAATG 58 (0.001489%)

GGCATTCGGGCATTGGCTGTACCCTTCCGCTTACCTATGCCCATGTGCCTGCCCTTCCGGCGGGCCAAGG
TGTTTTTCCGGCATCGAGCCCGGGAATGGA 4 (0.000103%)

GGCATTTTGTTTATGAGCAAGGTGGGTCTCAGAGGTGATCGGCGATCAGAGGGCGATGAAGTTCTAGAT
CCATTGAGACAAGCTCTAGACAGTAGCATGC 42 (0.001078%)

GGCATTTTTAATCTTAGAGCGAAAGCCTATAATCACTGCGCCTGTTCATAAGGGGATGGCCATGGCTAG
GTTTATAGATAGTTGGGTGGTTGGTGTAAAT 450 (0.011551%)

GGCCACCTACGGTGAAAAGAAAGATGAATCCTAGGGCTCAGAGCACTGCAGCAGATCATTTCATATTG
CTTCCGTGGAGTGTGGCGAGTCAGCTAAATAC 18 (0.000462%)

GGCCAGTGCCCTCCTAATTGGGGGGTAGGGGCTAGGCTGGAGTGGTAAAAGGCTCAGAAAAATCCTGC
GAAGAAAAAAACTTCTGAGGTAATAAATAGGA 24 (0.000616%)

GGCCTCGGACACGAAGGCCCCAGAAGTGACGCAGCCCTCTATGGGCCCGAATCTTCTTCAGTCGCTCCA
GGTCTTCACGGAGCTTGTTGTCCAGACCATT 5 (0.000128%)

GGCCTTGGTATGTGCTTTCTCGTGTTACATCGCGCCATCATTGGTATATGGTTAGTGTGTTGGTTAGTAG
GCCTAGTATGAGGAGCGTTATGGAGTGGAA 16 (0.000411%)

GGCGATCAATCTTTTCCTTCAGCTCAGCAAACTTGCATGCAATGTGAGCCGTGTGGCAATCCAATACAG
GGGCATAGCCGGCGCTTATTTGGCCTGGATG 153 (0.003927%)

GGCGATCAGAGGGCGATGAAGTTCTAGATCCATTGAGACAAGCTCTAGACAGTAGCATGCAGTCCCAC
AACTTGTACCAGCATCCCCAGCGTCTGGCATT 17 (0.000436%)

GGCGTAGGTTTGGTCTAGGGTGTAGCCTGAGAATAGGGGAAATCAGTGAATGAAGCCTCCTATGATGG
CAAATACAGCTCCTATTGATAGGACATAGTGG 9 (0.000231%)

GGCTGTGACTAGTATGTTGAGTCCTGTAAGTAGGAGAGTGATATTTGATCAGGAGAACGTGGTTACTAG
CACAGAGAGTTCTCCCAGTAGGTTAATAGTG 18 (0.000462%)

GGGAATCATAAATCATGCCAAAGCCAGTTGTCTTGCCACCACCAAAATGAGTTCTGAATCCAAATACAA
AGATGACATCCGGTGTGGTCTTGTACATTTT 19 (0.000488%)

GGGAGTAGTTCCCTGCTAAGGGAGGGTAGACTGTTCAACCTGTTCCTGCTCCGGCCTCCACTATAGCAG
ATGCGAGCAGGAGTAGGAGAGAGGGAGGTAA 36 (0.000924%)

GGGAGTATAGGGCTGTGACTAGTATGTTGAGTCCTGTAAGTAGGAGAGTGATATTTGATCAGGAGAAC
GTGGTTACTAGCACAGAGAGTTCTCCCAGTAG 20 (0.000513%)

GGGATAATATTCATTTAGCCTTCTGAGCTTTCTGGGCAGACTTGGTGACCTTGCCAGCTCCAGCAGCCTT
CTTGTCCACTGCTTTGATGACACCCACCGC 26 (0.000667%)

GGGATGAGGCGTACCAGCACAGAGCCGCAGCGGCCTGTCACCTTGCAAGGGACAGTGTGGGGCTTGCC
GATCTTGTTCCCCCAGTAGCCTCTGCGCACGG 8 (0.000205%)

GGGCAGACTTGGTGACCTTGCCAGCTCCAGCAGCCTTCTTGTCCACTGCTTTGATGACACCCACCGCAA
CTGTCTGTCTCATATCACGAACAGCAAAGCG 34 (0.000873%)

GGGCAGAGGACCAACATGGGCATTTTGTTTATGAGCAAGGTGGGTCTCAGAGGTGATCGGCGATCAGA
GGGCGATGAAGTTCTAGATCCATTGAGACAAG 11 (0.000282%)

GGGCATTTTGTTTATGAGCAAGGTGGGTCTCAGAGGTGATCGGCGATCAGAGGGCGATGAAGTTCTAGA
TCCATTGAGACAAGCTCTAGACAGTAGCATG 62 (0.001592%)

GGGCCAGTGCCCTCCTAATTGGGGGGTAGGGGCTAGGCTGGAGTGGTAAAAGGCTCAGAAAAATCCTG
CGAAGAAAAAAACTTCTGAGGTAATAAATAGG 13 (0.000334%)

GGGCCATCTTCCAGCTTTTTACCAGAACGGCGATCAATCTTTTCCTTCAGCTCAGCAAACTTGCATGCAA
TGTGAGCCGTGTGGCAATCCAATACAGGGG 122 (0.003132%)

GGGGCCAGTGCCCTCCTAATTGGGGGGTAGGGGCTAGGCTGGAGTGGTAAAAGGCTCAGAAAAATCCT
GCGAAGAAAAAAACTTCTGAGGTAATAAATAG 28 (0.000719%)

GGGGGCCAGTGCCCTCCTAATTGGGGGGTAGGGGCTAGGCTGGAGTGGTAAAAGGCTCAGAAAAATCC
TGCGAAGAAAAAAACTTCTGAGGTAATAAATA 14 (0.000359%)
GGGGGGGGGGGGGGGGGGGGGGGGGGGGGGGGGGGGGGGG 6051 (0.062131%)

GGGTCTCAAAATTCTGTGACAAATTTTTGGTCAAGTTGTTTCCATTAAAAAGTACTGATTTTAAAAACTA ATAACTTAAAACTGCCACACGCAAAAAAGA 43 (0.001104%)

GGGTGAAAGAGTATGATGGGGTGGTGGTTGTGGTAAACTTTAATAGTGTAGGAAGCTGAATAATTTATG AAGGAGAGGGGTCAGGGTTGATTCGGGAGGA 3 (0.000077%)

GGGTGAGTGAGCCCCATTGTGTTGTGGTAAATATGTAGAGGGAGTATAGGGCTGTGACTAGTATGTTGA GTCCTGTAAGTAGGAGAGTGATATTTGATCA 60 (0.001540%)

GGGTGGCACTGCCCACGGTGGGCGGGCGGGCCTCTCTACTCGAAGGTGACCACGTTTAGATTCTGAGAC GGGAAGTGGAGGGTGAATAGGTCACGGCGGC 9 (0.000231%)

GGGTGTAGCCTGAGAATAGGGGAAATCAGTGAATGAAGCCTCCTATGATGGCAAATACAGCTCCTATT GATAGGACATAGTGGAAGTGAGCTACAACGTA 11 (0.000282%)

GGGTTAGCGATGGAGGTAGGATTGGTGCTGTGGGTGAAAGAGTATGATGGGGTGGTGGTTGTGGTAAA CTTTAATAGTGTAGGAAGCTGAATAATTTATG 120 (0.003080%)

GGGTTGTAGCCAATTTTCTTAATGTAAGTGCTGACTTCCTTAACAATTTCCTCATATCTCTTCTGGCTGTA GGGTGGCTCAGTGGAATCCATTTTGTTAA 1009 (0.025901%)

GGTAAATATGTAGAGGGAGTATAGGGCTGTGACTAGTATGTTGAGTCCTGTAAGTAGGAGAGTGATATT TGATCAGGAGAACGTGGTTACTAGCACAGAG 21 (0.000539%)

GGTAGGATTGGTGCTGTGGGTGAAAGAGTATGATGGGGTGGTGGTTGTGGTAAACTTTAATAGTGTAGG AAGCTGAATAATTTATGAAGGAGAGGGGTCA 5 (0.000128%)

GGTATGTGCTTTCTCGTGTTACATCGCGCCATCATTGGTATATGGTTAGTGTGTTGGTTAGTAGGCCTAG TATGAGGAGCGTTATGGAGTGGAAGTGAAA 4 (0.000103%)

GGTCAAGTTGTTTCCATTAAAAAGTACTGATTTTAAAAACTAATAACTTAAAACTGCCACACGCAAAAA AGAAAACCAAAGTGGTCCACAAAACATTCTC 9 (0.000231%)

GGTCTAGGGTGTAGCCTGAGAATAGGGGAAATCAGTGAATGAAGCCTCCTATGATGGCAAATACAGCT CCTATTGATAGGACATAGTGGAAGTGAGCTAC4 (0.000103%)

GGTCTCAAAATTCTGTGACAAATTTTTGGTCAAGTTGTTTCCATTAAAAAGTACTGATTTTAAAAACTAA TAACTTAAAACTGCCACACGCAAAAAAGAA 5 (0.000128%)

GGTGACCTTGCCAGCTCCAGCAGCCTTCTTGTCCACTGCTTTGATGACACCCACCGCAACTGTCTGTCTC ATATCACGAACAGCAAAGCGACCCAAAGGT 16 (0.000411%)

GGTGAGTGAGCCCCATTGTGTTGTGGTAAATATGTAGAGGGAGTATAGGGCTGTGACTAGTATGTTGAG TCCTGTAAGTAGGAGAGTGATATTTGATCAG 19 (0.000488%)

GGTGGCACTGCCCACGGTGGGCGGGCGGGCCTCTCTACTCGAAGGTGACCACGTTTAGATTCTGAGACG GGAAGTGGAGGGTGAATAGGTCACGGCGGCC 13 (0.000334%)

GGTGGCCTTGGTATGTGCTTTCTCGTGTTACATCGCGCCATCATTGGTATATGGTTAGTGTGTTGGTTAG TAGGCCTAGTATGAGGAGCGTTATGGAGTG 14 (0.000359%)

GGTGGGTGAGTGAGCCCCATTGTGTTGTGGTAAATATGTAGAGGGAGTATAGGGCTGTGACTAGTATGT TGAGTCCTGTAAGTAGGAGAGTGATATTTGA 4 (0.000103%)

GGTGTAGCCTGAGAATAGGGGAAATCAGTGAATGAAGCCTCCTATGATGGCAAATACAGCTCCTATTG ATAGGACATAGTGGAAGTGAGCTACAACGTAG 4 (0.000103%)

GGTTCAGGATAATCACCTGAGCAGTGAAGCCAGCTGCTTCCATTGGTGGGTCATTTTTGCTGTCACCAG

CAACGTTGCCACGACGAACATCCTTGACAGA 4 (0.000103%)

GGTTCCCCGGAGACGTCCAGTCCGGCGGGCAGCAATGAGACCCACTTTGCGGCCAGCAGGGGCATCTCT
GCGGATGGTGGAGGGCTTGCCGATGTGCTGG 5 (0.000128%)

GGTTGTAGCCAATTTTCTTAATGTAAGTGCTGACTTCCTTAACAATTTCCTCATATCTCTTCTGGCTGTAG
GGTGGCTCAGTGGAATCCATTTTGTTAAC 6 (0.000154%)

GGTTTGGTCTAGGGTGTAGCCTGAGAATAGGGGAAATCAGTGAATGAAGCCTCCTATGATGGCAAATA
CAGCTCCTATTGATAGGACATAGTGGAAGTGA 17 (0.000436%)

GTAAATATGTAGAGGGAGTATAGGGCTGTGACTAGTATGTTGAGTCCTGTAAGTAGGAGAGTGATATTT
GATCAGGAGAACGTGGTTACTAGCACAGAGA 42 (0.001078%)

GTAAGTGCTGACTTCCTTAACAATTTCCTCATATCTCTTCTGGCTGTAGGGTGGCTCAGTGGAATCCATT
TTGTTAACACCGACAATTAGTTGTTTCACA 7 (0.000180%)

GTAGAATTAGAATTGTGAAGATGATAAGTGTAGAGGGAAGGTTAATGGTTGATATTGCTAGGGTGGCG
CTTCCAATTAGGTGCATGAGTAGGTGGCCTGC 49 (0.001258%)

GTAGAGGGAGTATAGGGCTGTGACTAGTATGTTGAGTCCTGTAAGTAGGAGAGTGATATTTGATCAGGA
GAACGTGGTTACTAGCACAGAGAGTTCTCCC 5 (0.000128%)

GTAGCCAATTTTCTTAATGTAAGTGCTGACTTCCTTAACAATTTCCTCATATCTCTTCTGGCTGTAGGGTG
GCTCAGTGGAATCCATTTTGTTAACACCG 23 (0.000590%)

GTAGCCTGAGAATAGGGGAAATCAGTGAATGAAGCCTCCTATGATGGCAAATACAGCTCCTATTGATA
GGACATAGTGGAAGTGAGCTACAACGTAGTAC 10 (0.000257%)

GTAGGATTGGTGCTGTGGGTGAAAGAGTATGATGGGGTGGTGGTTGTGGTAAACTTTAATAGTGTAGGA
AGCTGAATAATTTATGAAGGAGAGGGGTCAG 15 (0.000385%)

GTAGGTTTGGTCTAGGGTGTAGCCTGAGAATAGGGGAAATCAGTGAATGAAGCCTCCTATGATGGCAA
ATACAGCTCCTATTGATAGGACATAGTGGAAG 30 (0.000770%)

GTAGTTCCCTGCTAAGGGAGGGTAGACTGTTCAACCTGTTCCTGCTCCGGCCTCCACTATAGCAGATGC
GAGCAGGAGTAGGAGAGAGGGAGGTAAGAGT 18 (0.000462%)

GTATAGGGCTGTGACTAGTATGTTGAGTCCTGTAAGTAGGAGAGTGATATTTGATCAGGAGAACGTGGT
TACTAGCACAGAGAGTTCTCCCAGTAGGTTA 7 (0.000180%)

GTCAAGTTGTTTCCATTAAAAAGTACTGATTTTAAAAACTAATAACTTAAAACTGCCACACGCAAAAAA
GAAAACCAAAGTGGTCCACAAAACATTCTCC 57 (0.001463%)

GTCACAGGTCCAGGGCAGAGGACCAACATGGGCATTTTGTTTATGAGCAAGGTGGGTCTCAGAGGTGA
TCGGCGATCAGAGGGCGATGAAGTTCTAGATC18 (0.000462%)

GTCAGGCCACCTACGGTGAAAAGAAAGATGAATCCTAGGGCTCAGAGCACTGCAGCAGATCATTTCAT
ATTGCTTCCGTGGAGTGTGGCGAGTCAGCTAA 15 (0.000385%)

GTCAGTAGAATTAGAATTGTGAAGATGATAAGTGTAGAGGGAAGGTTAATGGTTGATATTGCTAGGGT
GGCGCTTCCAATTAGGTGCATGAGTAGGTGGC 7 (0.000180%)

GTCATCTGCGGGTGGCACTGCCCACGGTGGGCGGGCGGGCCTCTCTACTCGAAGGTGACCACGTTTAGA
TTCTGAGACGGGAAGTGGAGGGTGAATAGGT 780 (0.020022%)

GTCCAGGGCAGAGGACCAACATGGGCATTTTGTTTATGAGCAAGGTGGGTCTCAGAGGTGATCGGCGA
TCAGAGGGCGATGAAGTTCTAGATCCATTGAG13 (0.000334%)

GTCCAGTCCGGCGGGCAGCAATGAGACCCACTTTGCGGCCAGCAGGGGCATCTCTGCGGATGGTGGAG
GGCTTGCCGATGTGCTGGTGGTTGCCACCTCC 7 (0.000180%)

GTCGATGCCACCGCATTTATAGATCAGATGGCCAGTAGTGGTGGACTTGCCCGAATCTACGTGTCCAAT
GACGACAATGTTGATATGAGTCTTTTCCTTT 35 (0.000898%)

GTCGGTTGTTGATGAGATATTTGGAGGTGGGGATCAATAGAGGGGGAAATAGAATGATCAGTACTGCG
GCGGGTAGGCCTAGGATTGTGGGGGCAATGAA 875 (0.022461%)

GTCTAGGGTGTAGCCTGAGAATAGGGGAAATCAGTGAATGAAGCCTCCTATGATGGCAAATACAGCTC
CTATTGATAGGACATAGTGGAAGTGAGCTACA7 (0.000180%)

GTCTCAAAATTCTGTGACAAATTTTTGGTCAAGTTGTTTCCATTAAAAAGTACTGATTTTAAAAACTAAT
AACTTAAAACTGCCACACGCAAAAAAGAAA 11 (0.000282%)

GTCTCAAATTTCCACAAGGAGATATCAATGGTGATACCACGTTCACGCTCAGCTTTCAGTTTATCCAAG
ACCCAGGCATACTTGAAGGAGCCCTTTCCCA 15 (0.000385%)

GTCTTGGTTCCCCGGAGACGTCCAGTCCGGCGGGCAGCAATGAGACCCACTTTGCGGCCAGCAGGGGC
ATCTCTGCGGATGGTGGAGGGCTTGCCGATGT 7 (0.000180%)

GTCTTTTACTACTAAACTTAAATGGCCAATTGAAACAAACAGTTCTGAGACCGTTCTTCCACCACTGATT
AAGAGTGGGGTGGCAGGTATTAGGGATAAT 35 (0.000898%)

GTGACAAATTTTTGGTCAAGTTGTTTCCATTAAAAAGTACTGATTTTAAAAACTAATAACTTAAAACTGC
CACACGCAAAAAAGAAAACCAAAGTGGTCC 3 (0.000077%)

GTGACCTTGCCAGCTCCAGCAGCCTTCTTGTCCACTGCTTTGATGACACCCACCGCAACTGTCTGTCTCA
TATCACGAACAGCAAAGCGACCCAAAGGTG 15 (0.000385%)

GTGAGCCCCATTGTGTTGTGGTAAATATGTAGAGGGAGTATAGGGCTGTGACTAGTATGTTGAGTCCTG
TAAGTAGGAGAGTGATATTTGATCAGGAGAA 17 (0.000436%)

GTGAGCCGTGTGGCAATCCAATACAGGGGCATAGCCGGCGCTTATTTGGCCTGGATGGTTCAGGATAAT
CACCTGAGCAGTGAAGCCAGCTGCTTCCATT 13 (0.000334%)

GTGAGGAGAAGGCTTACGTTTAGTGAGGGAGAGATTTGGTATATGATTGAGATGGGGGCTAGTTTTTGT
CATGTGAGAAGAAGCAGGCCGGATGTCAGAG 10 (0.000257%)

GTGAGTGAGCCCCATTGTGTTGTGGTAAATATGTAGAGGGAGTATAGGGCTGTGACTAGTATGTTGAGT
CCTGTAAGTAGGAGAGTGATATTTGATCAGG 21 (0.000539%)

GTGCCCCTGGGTGCAGGGATGAGGCGTACCAGCACAGAGCCGCAGCGGCCTGTCACCTTGCAAGGGAC
AGTGTGGGGCTTGCCGATCTTGTTCCCCCAGT 17 (0.000436%)

GTGCCCTCCTAATTGGGGGGTAGGGGCTAGGCTGGAGTGGTAAAAGGCTCAGAAAAATCCTGCGAAGA
AAAAAACTTCTGAGGTAATAAATAGGATTATC7 (0.000180%)

GTGCTGACTTCCTTAACAATTTCCTCATATCTCTTCTGGCTGTAGGGTGGCTCAGTGGAATCCATTTTGTT
AACACCGACAATTAGTTGTTTCACACCCA 20 (0.000513%)

GTGCTTTCTCGTGTTACATCGCGCCATCATTGGTATATGGTTAGTGTGTTGGTTAGTAGGCCTAGTATGA
GGAGCGTTATGGAGTGGAAGTGAAATCACA 21 (0.000539%)

GTGGCAATCCAATACAGGGGCATAGCCGGCGCTTATTTGGCCTGGATGGTTCAGGATAATCACCTGAGC
AGTGAAGCCAGCTGCTTCCATTGGTGGGTCA 37 (0.000950%)

GTGGCACTGCCCACGGTGGGCGGGCGGGCCTCTCTACTCGAAGGTGACCACGTTTAGATTCTGAGACGG
GAAGTGGAGGGTGAATAGGTCACGGCGGCCT 8 (0.000205%)

GTGGCCTTGGTATGTGCTTTCTCGTGTTACATCGCGCCATCATTGGTATATGGTTAGTGTGTTGGTTAGT
AGGCCTAGTATGAGGAGCGTTATGGAGTGG 27 (0.000693%)

GTGGGAGTAGTTCCCTGCTAAGGGAGGGTAGACTGTTCAACCTGTTCCTGCTCCGGCCTCCACTATAGC
AGATGCGAGCAGGAGTAGGAGAGAGGGAGGT541 (0.013887%)

GTGGGGACAGCTCATGAGTGCAAGACGTCTTGTGATGTAATTATTATACGAATGGGGGCTTCAATCGGG
AGTACTACTCGATTGTCAACGTCAAGGAGTC 8 (0.000205%)

GTGGGTGAAAGAGTATGATGGGGTGGTGGTTGTGGTAAACTTTAATAGTGTAGGAAGCTGAATAATTTA
TGAAGGAGAGGGGTCAGGGTTGATTCGGGAG 3 (0.000077%)

GTGGGTGAGTGAGCCCCATTGTGTTGTGGTAAATATGTAGAGGGAGTATAGGGCTGTGACTAGTATGTT
GAGTCCTGTAAGTAGGAGAGTGATATTTGAT 5 (0.000128%)

GTGGTAAATATGTAGAGGGAGTATAGGGCTGTGACTAGTATGTTGAGTCCTGTAAGTAGGAGAGTGAT
ATTTGATCAGGAGAACGTGGTTACTAGCACAG6 (0.000154%)

GTGGTGGCCTTGGTATGTGCTTTCTCGTGTTACATCGCGCCATCATTGGTATATGGTTAGTGTGTTGGTT
AGTAGGCCTAGTATGAGGAGCGTTATGGAG 558 (0.014324%)

GTGTAGCCTGAGAATAGGGGAAATCAGTGAATGAAGCCTCCTATGATGGCAAATACAGCTCCTATTGAT
AGGACATAGTGGAAGTGAGCTACAACGTAGT 8 (0.000205%)

GTGTGGCAATCCAATACAGGGGCATAGCCGGCGCTTATTTGGCCTGGATGGTTCAGGATAATCACCTGA
GCAGTGAAGCCAGCTGCTTCCATTGGTGGGT 15 (0.000385%)

GTGTTACATCGCGCCATCATTGGTATATGGTTAGTGTGTTGGTTAGTAGGCCTAGTATGAGGAGCGTTAT
GGAGTGGAAGTGAAATCACATGGCTAGGCC 13 (0.000334%)

GTGTTGTGGTAAATATGTAGAGGGAGTATAGGGCTGTGACTAGTATGTTGAGTCCTGTAAGTAGGAGAG
TGATATTTGATCAGGAGAACGTGGTTACTAG 6 (0.000154%)

GTTAGCGATGGAGGTAGGATTGGTGCTGTGGGTGAAAGAGTATGATGGGGTGGTGGTTGTGGTAAACTT
TAATAGTGTAGGAAGCTGAATAATTTATGAA 6 (0.000154%)

GTTCAGGATAATCACCTGAGCAGTGAAGCCAGCTGCTTCCATTGGTGGGTCATTTTTGCTGTCACCAGC
AACGTTGCCACGACGAACATCCTTGACAGAC 38 (0.000975%)

GTTCATAAGGGGATGGCCATGGCTAGGTTTATAGATAGTTGGGTGGTTGGTGTAAATGAGTGAGGCAGG
AGTCCGAGGAGGTTAGTTGTGGCAATAAAAA 133 (0.003414%)

GTTCCCCGGAGACGTCCAGTCCGGCGGGCAGCAATGAGACCCACTTTGCGGCCAGCAGGGGCATCTCTG
CGGATGGTGGAGGGCTTGCCGATGTGCTGGT 5 (0.000128%)

GTTCCCTGCTAAGGGAGGGTAGACTGTTCAACCTGTTCCTGCTCCGGCCTCCACTATAGCAGATGCGAG
CAGGAGTAGGAGAGAGGGAGGTAAGAGTCAG12 (0.000308%)

GTTCTTTTGTCGATGCCACCGCATTTATAGATCAGATGGCCAGTAGTGGTGGACTTGCCCGAATCTACGT
GTCCAATGACGACAATGTTGATATGAGTCT 26 (0.000667%)

GTTGGGGGCCAGTGCCCTCCTAATTGGGGGGTAGGGGCTAGGCTGGAGTGGTAAAAGGCTCAGAAAAA
TCCTGCGAAGAAAAAAACTTCTGAGGTAATAA 28 (0.000719%)

GTTGTAGCCAATTTTCTTAATGTAAGTGCTGACTTCCTTAACAATTTCCTCATATCTCTTCTGGCTGTAGG

GTGGCTCAGTGGAATCCATTTTGTTAACA 85 (0.002182%)

GTTGTGGTAAATATGTAGAGGGAGTATAGGGCTGTGACTAGTATGTTGAGTCCTGTAAGTAGGAGAGTG
ATATTTGATCAGGAGAACGTGGTTACTAGCA 3 (0.000077%)

GTTGTTTCCATTAAAAAGTACTGATTTTAAAAACTAATAACTTAAAACTGCCACACGCAAAAAAGAAAA
CCAAAGTGGTCCACAAAACATTCTCCTTTCC 24 (0.000616%)

GTTTATGAGCAAGGTGGGTCTCAGAGGTGATCGGCGATCAGAGGGCGATGAAGTTCTAGATCCATTGA
GACAAGCTCTAGACAGTAGCATGCAGTCCCAC 17 (0.000436%)

GTTTCCATTAAAAAGTACTGATTTTAAAAACTAATAACTTAAAACTGCCACACGCAAAAAAGAAAACCA
AAGTGGTCCACAAAACATTCTCCTTTCCTTC 68 (0.001746%)

GTTTGGTCTAGGGTGTAGCCTGAGAATAGGGGAAATCAGTGAATGAAGCCTCCTATGATGGCAAATAC
AGCTCCTATTGATAGGACATAGTGGAAGTGAG131 (0.003363%)

GTTTTTAAGCCTAATGTGGGGACAGCTCATGAGTGCAAGACGTCTTGTGATGTAATTATTATACGAATG
GGGGCTTCAATCGGGAGTACTACTCGATTGT 24 (0.000616%)

TAAATATGTAGAGGGAGTATAGGGCTGTGACTAGTATGTTGAGTCCTGTAAGTAGGAGAGTGATATTTG
ATCAGGAGAACGTGGTTACTAGCACAGAGAG 16 (0.000411%)

TAACAATTTCCTCATATCTCTTCTGGCTGTAGGGTGGCTCAGTGGAATCCATTTTGTTAACACCGACAAT
TAGTTGTTTCACACCCAGTGTGTAAGCCAG 20 (0.000513%)

TAACAGTTCAAAAGAAGCCACTACATACTCTTTTCACAAATATGTTTTCACAGAGCCAATACAGTACTA
GCCATTAACCCAGTACACCAAGTGTACTGAA 9 (0.000231%)

TAACCAGTCTTTTACTACTAAACTTAAATGGCCAATTGAAACAAACAGTTCTGAGACCGTTCTTCCACCA
CTGATTAAGAGTGGGGTGGCAGGTATTAGG 9 (0.000231%)

TAAGGGAGGGTAGACTGTTCAACCTGTTCCTGCTCCGGCCTCCACTATAGCAGATGCGAGCAGGAGTAG
GAGAGAGGGAGGTAAGAGTCAGAAGCTTATG 3 (0.000077%)

TAAGTGCTGACTTCCTTAACAATTTCCTCATATCTCTTCTGGCTGTAGGGTGGCTCAGTGGAATCCATTT
TGTTAACACCGACAATTAGTTGTTTCACAC 7 (0.000180%)

TAATACAATGCCAGTCAGGCCACCTACGGTGAAAAGAAAGATGAATCCTAGGGCTCAGAGCACTGCAG
CAGATCATTTCATATTGCTTCCGTGGAGTGTG 10 (0.000257%)

TAATATTCATTTAGCCTTCTGAGCTTTCTGGGCAGACTTGGTGACCTTGCCAGCTCCAGCAGCCTTCTTG
TCCACTGCTTTGATGACACCCACCGCAACT 13 (0.000334%)

TAATCACCTGAGCAGTGAAGCCAGCTGCTTCCATTGGTGGGTCATTTTTGCTGTCACCAGCAACGTTGCC
ACGACGAACATCCTTGACAGACACATTCTT 8 (0.000205%)

TAATGGCAGAATTTGGCTGTTTGGCTTCAACTCCTACTTTTTCCAGCACGATTCCTTTTGCATGAGAAGC
ACCTCCAAAAGGGTTGGCCTTTAGGGCTGT 6 (0.000154%)

TAATGGGTCTCAAAATTCTGTGACAAATTTTTGGTCAAGTTGTTTCCATTAAAAAGTACTGATTTTAAAA
ACTAATAACTTAAAACTGCCACACGCAAAA 6 (0.000154%)

TAATTGGGGGGTAGGGGCTAGGCTGGAGTGGTAAAAGGCTCAGAAAAATCCTGCGAAGAAAAAAACTT
CTGAGGTAATAAATAGGATTATCCCGTATCGA 10 (0.000257%)

TACAATGCCAGTCAGGCCACCTACGGTGAAAAGAAAGATGAATCCTAGGGCTCAGAGCACTGCAGCAG
ATCATTTCATATTGCTTCCGTGGAGTGTGGCG 4 (0.000103%)

TACACACTTCCTAATGGCAGAATTTGGCTGTTTGGCTTCAACTCCTACTTTTTCCAGCACGATTCCTTTTG
CATGAGAAGCACCTCCAAAAGGGTTGGCC 11 (0.000282%)

TAGAATTAGAATTGTGAAGATGATAAGTGTAGAGGGAAGGTTAATGGTTGATATTGCTAGGGTGGCGCT
TCCAATTAGGTGCATGAGTAGGTGGCCTGCA 10 (0.000257%)

TAGAGGGAGTATAGGGCTGTGACTAGTATGTTGAGTCCTGTAAGTAGGAGAGTGATATTTGATCAGGAG
AACGTGGTTACTAGCACAGAGAGTTCTCCCA 3 (0.000077%)

TAGCCAATTTTCTTAATGTAAGTGCTGACTTCCTTAACAATTTCCTCATATCTCTTCTGGCTGTAGGGTGG
CTCAGTGGAATCCATTTTGTTAACACCGA 3 (0.000077%)

TAGCCTGAGAATAGGGGAAATCAGTGAATGAAGCCTCCTATGATGGCAAATACAGCTCCTATTGATAG
GACATAGTGGAAGTGAGCTACAACGTAGTACG 3 (0.000077%)

TAGCCTTCTGAGCTTTCTGGGCAGACTTGGTGACCTTGCCAGCTCCAGCAGCCTTCTTGTCCACTGCTTT
GATGACACCCACCGCAACTGTCTGTCTCAT 31 (0.000796%)

TAGCGATGGAGGTAGGATTGGTGCTGTGGGTGAAAGAGTATGATGGGGTGGTGGTTGTGGTAAACTTTA
ATAGTGTAGGAAGCTGAATAATTTATGAAGG 4 (0.000103%)

TAGGATAGTCAGTAGAATTAGAATTGTGAAGATGATAAGTGTAGAGGGAAGGTTAATGGTTGATATTG
CTAGGGTGGCGCTTCCAATTAGGTGCATGAGT 4 (0.000103%)

TAGGGATAATATTCATTTAGCCTTCTGAGCTTTCTGGGCAGACTTGGTGACCTTGCCAGCTCCAGCAGCC
TTCTTGTCCACTGCTTTGATGACACCCACC30 (0.000770%)

TAGGGCCATCTTCCAGCTTTTTACCAGAACGGCGATCAATCTTTTCCTTCAGCTCAGCAAACTTGCATGC
AATGTGAGCCGTGTGGCAATCCAATACAGG 4 (0.000103%)

TAGGGTGTAGCCTGAGAATAGGGGAAATCAGTGAATGAAGCCTCCTATGATGGCAAATACAGCTCCTA
TTGATAGGACATAGTGGAAGTGAGCTACAACG 5 (0.000128%)

TAGTCAGTAGAATTAGAATTGTGAAGATGATAAGTGTAGAGGGAAGGTTAATGGTTGATATTGCTAGG
GTGGCGCTTCCAATTAGGTGCATGAGTAGGTG 3 (0.000077%)

TATCAACAATGGCAGCATCACCAGACTTCAAGAATTTAGGGCCATCTTCCAGCTTTTTACCAGAACGGC
GATCAATCTTTTCCTTCAGCTCAGCAAACTT 19 (0.000488%)

TATGTGCTTTCTCGTGTTACATCGCGCCATCATTGGTATATGGTTAGTGTGTTGGTTAGTAGGCCTAGTA
TGAGGAGCGTTATGGAGTGGAAGTGAAATC 4 (0.000103%)

TATTCATTTAGCCTTCTGAGCTTTCTGGGCAGACTTGGTGACCTTGCCAGCTCCAGCAGCCTTCTTGTCC
ACTGCTTTGATGACACCCACCGCAACTGTC 43 (0.001104%)

TCAAAATTCTGTGACAAATTTTTGGTCAAGTTGTTTCCATTAAAAAGTACTGATTTTAAAAACTAATAAC
TTAAAACTGCCACACGCAAAAAAGAAAACC 28 (0.000719%)

TCAAATTTCCACAAGGAGATATCAATGGTGATACCACGTTCACGCTCAGCTTTCAGTTTATCCAAGACC
CAGGCATACTTGAAGGAGCCCTTTCCCATCT 8 (0.000205%)

TCAAATTTTTCAATGGTTCTTTTGTCGATGCCACCGCATTTATAGATCAGATGGCCAGTAGTGGTGGACT
TGCCCGAATCTACGTGTCCAATGACGACAA 5 (0.000128%)

TCAACAATGGCAGCATCACCAGACTTCAAGAATTTAGGGCCATCTTCCAGCTTTTTACCAGAACGGCGA
TCAATCTTTTCCTTCAGCTCAGCAAACTTGC 6 (0.000154%)

TCAAGAATTTAGGGCCATCTTCCAGCTTTTACCAGAACGGCGATCAATCTTTTCCTTCAGCTCAGCAAA
CTTGCATGCAATGTGAGCCGTGTGGCAATC 4 (0.000103%)

TCAAGTTGTTTCCATTAAAAAGTACTGATTTAAAAACTAATAACTTAAAACTGCCACACGCAAAAAAG
AAAACCAAAGTGGTCCACAAAACATTCTCCT 6 (0.000154%)

TCACCAGACTTCAAGAATTTAGGGCCATCTTCCAGCTTTTACCAGAACGGCGATCAATCTTTTCCTTCA
GCTCAGCAAACTTGCATGCAATGTGAGCCG 3 (0.000077%)

TCAGAGGGCGATGAAGTTCTAGATCCATTGAGACAAGCTCTAGACAGTAGCATGCAGTCCCACAACTTG
TACCAGCATCCCCAGCGTCTGGCATTCCATG 3 (0.000077%)

TCAGCAAACTTGCATGCAATGTGAGCCGTGTGGCAATCCAATACAGGGGCATAGCCGGCGCTTATTTGG
CCTGGATGGTTCAGGATAATCACCTGAGCAG 12 (0.000308%)

TCAGCATCTAACAGTTCAAAAGAAGCCACTACATACTCTTTTCACAAATATGTTTTCACAGAGCCAATA
CAGTACTAGCCATTAACCCAGTACACCAAGT 4 (0.000103%)

TCAGCTCAGCAAACTTGCATGCAATGTGAGCCGTGTGGCAATCCAATACAGGGGCATAGCCGGCGCTTA
TTTGGCCTGGATGGTTCAGGATAATCACCTG 5 (0.000128%)

TCAGGATAATCACCTGAGCAGTGAAGCCAGCTGCTTCCATTGGTGGGTCATTTTTGCTGTCACCAGCAA
CGTTGCCACGACGAACATCCTTGACAGACAC 12 (0.000308%)

TCAGGCCACCTACGGTGAAAAGAAAGATGAATCCTAGGGCTCAGAGCACTGCAGCAGATCATTTCATA
TTGCTTCCGTGGAGTGTGGCGAGTCAGCTAAA 5 (0.000128%)

TCATTTAGCCTTCTGAGCTTTCTGGGCAGACTTGGTGACCTTGCCAGCTCCAGCAGCCTTCTTGTCCACT
GCTTTGATGACACCCACCGCAACTGTCTGT 6 (0.000154%)

TCCACAAGGAGATATCAATGGTGATACCACGTTCACGCTCAGCTTTCAGTTTATCCAAGACCCAGGCAT
ACTTGAAGGAGCCCTTTCCCATCTCAGCAGC 6 (0.000154%)

TCCAGCAGCCTTCTTGTCCACTGCTTTGATGACACCCACCGCAACTGTCTGTCTCATATCACGAACAGCA
AAGCGACCCAAAGGTGGATAGTCTGAGAAG 8 (0.000205%)

TCCAGGGCAGAGGACCAACATGGGCATTTTGTTTATGAGCAAGGTGGGTCTCAGAGGTGATCGGCGATC
AGAGGGCGATGAAGTTCTAGATCCATTGAGA 3 (0.000077%)

TCCAGTCCGGCGGGCAGCAATGAGACCCACTTTGCGGCCAGCAGGGGCATCTCTGCGGATGGTGGAGG
GCTTGCCGATGTGCTGGTGGTTGCCACCTCCA 5 (0.000128%)

TCCCACTCCTGATGCTGAACCAATGCACCATCTGTAAAGTTGCAGACAGTCTGAGTTTTTCTGCCATCAG
CTGTGGTTTCTTCAAACTTCTCTCCCAGGG155 (0.003979%)

TCCTAATTGGGGGGTAGGGGCTAGGCTGGAGTGGTAAAAGGCTCAGAAAAATCCTGCGAAGAAAAAAA
CTTCTGAGGTAATAAATAGGATTATCCCGTAT 7 (0.000180%)

TCCTCATATCTCTTCTGGCTGTAGGGTGGCTCAGTGGAATCCATTTTGTTAACACCGACAATTAGTTGTT
TCACACCCAGTGTGTAAGCCAGAAGGGCAT 22 (0.000565%)

TCCTCATCCATGTGACCTTCTCTGGCATTCGGGCATTGGCTGTACCCTTCCGCTTACCTATGCCCATGTGC
CTGCCCTTCCGGCGGGCCAAGGTGTTTTT 184 (0.004723%)

TCCTTAACAATTTCCTCATATCTCTTCTGGCTGTAGGGTGGCTCAGTGGAATCCATTTTGTTAACACCGA
CAATTAGTTGTTTCACACCCAGTGTGTAAG 7 (0.000180%)

TCCTTCAGCTCAGCAAACTTGCATGCAATGTGAGCCGTGTGGCAATCCAATACAGGGGCATAGCCGGCG

CTTATTTGGCCTGGATGGTTCAGGATAATCA 3 (0.000077%)

TCGGACACGAAGGCCCCAGAAGTGACGCAGCCCTCTATGGGCCCGAATCTTCTTCAGTCGCTCCAGGTC
TTCACGGAGCTTGTTGTCCAGACCATTGGCT 8 (0.000205%)

TCGGTTGTTGATGAGATATTTGGAGGTGGGGATCAATAGAGGGGGAAATAGAATGATCAGTACTGCGG
CGGGTAGGCCTAGGATTGTGGGGGCAATGAAT 5 (0.000128%)

TCGTGTTACATCGCGCCATCATTGGTATATGGTTAGTGTGTTGGTTAGTAGGCCTAGTATGAGGAGCGTT
ATGGAGTGGAAGTGAAATCACATGGCTAGG 5 (0.000128%)

TCTAGGATAGTCAGTAGAATTAGAATTGTGAAGATGATAAGTGTAGAGGGAAGGTTAATGGTTGATATT
GCTAGGGTGGCGCTTCCAATTAGGTGCATGA 5 (0.000128%)

TCTAGGGTGTAGCCTGAGAATAGGGGAAATCAGTGAATGAAGCCTCCTATGATGGCAAATACAGCTCCT
ATTGATAGGACATAGTGGAAGTGAGCTACAA 8 (0.000205%)

TCTCAAAATTCTGTGACAAATTTTTGGTCAAGTTGTTTCCATTAAAAAGTACTGATTTTAAAAACTAATA
ACTTAAAACTGCCACACGCAAAAAAGAAAA 4 (0.000103%)

TCTCAAATTTTTCAATGGTTCTTTTGTCGATGCCACCGCATTTATAGATCAGATGGCCAGTAGTGGTGGA
CTTGCCCGAATCTACGTGTCCAATGACGAC 3 (0.000077%)

TCTCGTGTTACATCGCGCCATCATTGGTATATGGTTAGTGTGTTGGTTAGTAGGCCTAGTATGAGGAGCG
TTATGGAGTGGAAGTGAAATCACATGGCTA 8 (0.000205%)

TCTGAGCTTTCTGGGCAGACTTGGTGACCTTGCCAGCTCCAGCAGCCTTCTTGTCCACTGCTTTGATGAC
ACCCACCGCAACTGTCTGTCTCATATCACG 7 (0.000180%)

TCTGCGGGTGGCACTGCCCACGGTGGGCGGGCGGGCCTCTCTACTCGAAGGTGACCACGTTTAGATTCT
GAGACGGGAAGTGGAGGGTGAATAGGTCACG 3 (0.000077%)

TCTGGCATTCGGGCATTGGCTGTACCCTTCCGCTTACCTATGCCCATGTGCCTGCCCTTCCGGCGGGCCA
AGGTGTTTTTCCGGCATCGAGCCCGGGAAT 3 (0.000077%)

TCTGGGCAGACTTGGTGACCTTGCCAGCTCCAGCAGCCTTCTTGTCCACTGCTTTGATGACACCCACCGC
AACTGTCTGTCTCATATCACGAACAGCAAA 11 (0.000282%)

TCTGTGACAAATTTTTGGTCAAGTTGTTTCCATTAAAAAGTACTGATTTTAAAAACTAATAACTTAAAAC
TGCCACACGCAAAAAAGAAAACCAAAGTGG 19 (0.000488%)

TCTTAATGTAAGTGCTGACTTCCTTAACAATTTCCTCATATCTCTTCTGGCTGTAGGGTGGCTCAGTGGA
ATCCATTTTGTTAACACCGACAATTAGTTG 30 (0.000770%)

TCTTATCAAGTCAGCACACACCTTTTCCAAGGATTTTACGTTGCGGCTTGTTAGGGTGATTCGAATTCGG
TGAATTGCCACCTCCGGCTCCACGGGTGTT 5 (0.000128%)

TCTTGTCCACTGCTTTGATGACACCCACCGCAACTGTCTGTCTCATATCACGAACAGCAAAGCGACCCA
AAGGTGGATAGTCTGAGAAGCTCTCAACACA 5 (0.000128%)

TCTTTTACTACTAAACTTAAATGGCCAATTGAAACAAACAGTTCTGAGACCGTTCTTCCACCACTGATTA
AGAGTGGGGTGGCAGGTATTAGGGATAATA 7 (0.000180%)

TCTTTTCCTTCAGCTCAGCAAACTTGCATGCAATGTGAGCCGTGTGGCAATCCAATACAGGGGCATAGC
CGGCGCTTATTTGGCCTGGATGGTTCAGGAT 8 (0.000205%)

TCTTTTGTCGATGCCACCGCATTTATAGATCAGATGGCCAGTAGTGGTGGACTTGCCCGAATCTACGTGT
CCAATGACGACAATGTTGATATGAGTCTTT 6 (0.000154%)

TGAAAAGTGAACAGATATTCAGCATCTAACAGTTCAAAAGAAGCCACTACATACTCTTTTCACAAATAT
GTTTTCACAGAGCCAATACAGTACTAGCCAT 7 (0.000180%)

TGAAAGAGTATGATGGGGTGGTGGTTGTGGTAAACTTTAATAGTGTAGGAAGCTGAATAATTTATGAAG
GAGAGGGGTCAGGGTTGATTCGGGAGGATCC 7 (0.000180%)

TGACAAATTTTTGGTCAAGTTGTTTCCATTAAAAAGTACTGATTTTAAAAACTAATAACTTAAAACTGCC
ACACGCAAAAAAGAAAACCAAAGTGGTCCA 5 (0.000128%)

TGACCTTGCCAGCTCCAGCAGCCTTCTTGTCCACTGCTTTGATGACACCCACCGCAACTGTCTGTCTCAT
ATCACGAACAGCAAAGCGACCCAAAGGTGG 9 (0.000231%)

TGACTTCCTTAACAATTTCCTCATATCTCTTCTGGCTGTAGGGTGGCTCAGTGGAATCCATTTTGTTAAC
ACCGACAATTAGTTGTTTCACACCCAGTGT 14 (0.000359%)

TGAGAGAGTGAGGAGAAGGCTTACGTTTAGTGAGGGAGAGATTTGGTATATGATTGAGATGGGGGCTA
GTTTTTGTCATGTGAGAAGAAGCAGGCCGGAT 14 (0.000359%)

TGAGCAAGGTGGGTCTCAGAGGTGATCGGCGATCAGAGGGCGATGAAGTTCTAGATCCATTGAGACAA
GCTCTAGACAGTAGCATGCAGTCCCACAACTT 8 (0.000205%)

TGAGCAGTGAAGCCAGCTGCTTCCATTGGTGGGTCATTTTTGCTGTCACCAGCAACGTTGCCACGACGA
ACATCCTTGACAGACACATTCTTGACATTGA 15 (0.000385%)

TGAGCCGTGTGGCAATCCAATACAGGGGCATAGCCGGCGCTTATTTGGCCTGGATGGTTCAGGATAATC
ACCTGAGCAGTGAAGCCAGCTGCTTCCATTG 3 (0.000077%)

TGAGCTTTCTGGGCAGACTTGGTGACCTTGCCAGCTCCAGCAGCCTTCTTGTCCACTGCTTTGATGACAC
CCACCGCAACTGTCTGTCTCATATCACGAA 32 (0.000821%)

TGAGTGAGCCCCATTGTGTTGTGGTAAATATGTAGAGGGAGTATAGGGCTGTGACTAGTATGTTGAGTC
CTGTAAGTAGGAGAGTGATATTTGATCAGGA 13 (0.000334%)

TGCAATGTGAGCCGTGTGGCAATCCAATACAGGGGCATAGCCGGCGCTTATTTGGCCTGGATGGTTCAG
GATAATCACCTGAGCAGTGAAGCCAGCTGCT 10 (0.000257%)

TGCACAGTCTTGGTTCCCCGGAGACGTCCAGTCCGGCGGGCAGCAATGAGACCCACTTTGCGGCCAGCA
GGGGCATCTCTGCGGATGGTGGAGGGCTTGC 3 (0.000077%)

TGCATAATCCAGGGAATCATAAATCATGCCAAAGCCAGTTGTCTTGCCACCACCAAAATGAGTTCTGAA
TCCAAATACAAAGATGACATCCGGTGTGGTC 3 (0.000077%)

TGCATGCAATGTGAGCCGTGTGGCAATCCAATACAGGGGCATAGCCGGCGCTTATTTGGCCTGGATGGT
TCAGGATAATCACCTGAGCAGTGAAGCCAGC 5 (0.000128%)

TGCCACCGCATTTATAGATCAGATGGCCAGTAGTGGTGGACTTGCCCGAATCTACGTGTCCAATGACGA
CAATGTTGATATGAGTCTTTTCCTTTCCCAT 3 (0.000077%)

TGCCAGCTCCAGCAGCCTTCTTGTCCACTGCTTTGATGACACCCACCGCAACTGTCTGTCTCATATCACG
AACAGCAAAGCGACCCAAAGGTGGATAGTC 9 (0.000231%)

TGCCAGTCAGGCCACCTACGGTGAAAAGAAAGATGAATCCTAGGGCTCAGAGCACTGCAGCAGATCAT
TTCATATTGCTTCCGTGGAGTGTGGCGAGTCA 10 (0.000257%)

TGCCAGTGCCCCTGGGTGCAGGGATGAGGCGTACCAGCACAGAGCCGCAGCGGCCTGTCACCTTGCAA
GGGACAGTGTGGGGCTTGCCGATCTTGTTCCC 12 (0.000308%)

TGCCCACGGTGGGCGGGCGGGCCTCTCTACTCGAAGGTGACCACGTTTAGATTCTGAGACGGGAAGTGG AGGGTGAATAGGTCACGGCGGCCTTTTTTTT 3 (0.000077%)

TGCCCCTGGGTGCAGGGATGAGGCGTACCAGCACAGAGCCGCAGCGGCCTGTCACCTTGCAAGGGACA GTGTGGGGCTTGCCGATCTTGTTCCCCCAGTA 4 (0.000103%)

TGCCCTCCTAATTGGGGGGTAGGGGCTAGGCTGGAGTGGTAAAAGGCTCAGAAAAATCCTGCGAAGAA AAAAACTTCTGAGGTAATAAATAGGATTATCC 6 (0.000154%)

TGCGCCTGTTCATAAGGGGATGGCCATGGCTAGGTTTATAGATAGTTGGGTGGTTGGTGTAAATGAGTG AGGCAGGAGTCCGAGGAGGTTAGTTGTGGCA 3 (0.000077%)

TGCGGAGACGATGCCAGTGCCCCTGGGTGCAGGGATGAGGCGTACCAGCACAGAGCCGCAGCGGCCTG TCACCTTGCAAGGGACAGTGTGGGGCTTGCCG 31 (0.000796%)

TGCGGGTGGCACTGCCCACGGTGGGCGGGCGGGCCTCTCTACTCGAAGGTGACCACGTTTAGATTCTGA GACGGGAAGTGGAGGGTGAATAGGTCACGGC 11 (0.000282%)

TGCTAATACAATGCCAGTCAGGCCACCTACGGTGAAAAGAAAGATGAATCCTAGGGCTCAGAGCACTG CAGCAGATCATTTCATATTGCTTCCGTGGAGT 541 (0.013887%)

TGCTGAACCAATGCACCATCTGTAAAGTTGCAGACAGTCTGAGTTTTTCTGCCATCAGCTGTGGTTTCTT CAAACTTCTCTCCCAGGGTACAAGAAAACT 3 (0.000077%)

TGCTGACTTCCTTAACAATTTCCTCATATCTCTTCTGGCTGTAGGGTGGCTCAGTGGAATCCATTTTGTTA ACACCGACAATTAGTTGTTTCACACCCAG 34 (0.000873%)

TGCTGGTCTCAAATTTCCACAAGGAGATATCAATGGTGATACCACGTTCACGCTCAGCTTTCAGTTTATC CAAGACCCAGGCATACTTGAAGGAGCCCTT 17 (0.000436%)

TGCTTTCTCGTGTTACATCGCGCCATCATTGGTATATGGTTAGTGTGTTGGTTAGTAGGCCTAGTATGAG GAGCGTTATGGAGTGGAAGTGAAATCACAT 7 (0.000180%)

TGGATTTTGGCGTAGGTTTGGTCTAGGGTGTAGCCTGAGAATAGGGGAAATCAGTGAATGAAGCCTCCT ATGATGGCAAATACAGCTCCTATTGATAGGA 338 (0.008676%)

TGGCAATCCAATACAGGGGCATAGCCGGCGCTTATTTGGCCTGGATGGTTCAGGATAATCACCTGAGCA GTGAAGCCAGCTGCTTCCATTGGTGGGTCAT 217 (0.005570%)

TGGCACTGCCCACGGTGGGCGGGCGGGCCTCTCTACTCGAAGGTGACCACGTTTAGATTCTGAGACGGG AAGTGGAGGGTGAATAGGTCACGGCGGCCTT 10 (0.000257%)

TGGCAGCATCACCAGACTTCAAGAATTTAGGGCCATCTTCCAGCTTTTTACCAGAACGGCGATCAATCT TTTCCTTCAGCTCAGCAAACTTGCATGCAAT 10 (0.000257%)

TGGCATTCGGGCATTGGCTGTACCCTTCCGCTTACCTATGCCCATGTGCCTGCCCTTCCGGCGGGCCAAG GTGTTTTTCCGGCATCGAGCCCGGGAATGG 10 (0.000257%)

TGGCCTCGGACACGAAGGCCCCAGAAGTGACGCAGCCCTCTATGGGCCCGAATCTTCTTCAGTCGCTCC AGGTCTTCACGGAGCTTGTTGTCCAGACCAT 295 (0.007573%)

TGGCCTTGGTATGTGCTTTCTCGTGTTACATCGCGCCATCATTGGTATATGGTTAGTGTGTTGGTTAGTA GGCCTAGTATGAGGAGCGTTATGGAGTGGA 4 (0.000103%)

TGGCGTAGGTTTGGTCTAGGGTGTAGCCTGAGAATAGGGGAAATCAGTGAATGAAGCCTCCTATGATGG CAAATACAGCTCCTATTGATAGGACATAGTG 3 (0.000077%)

TGGGAGTAGTTCCCTGCTAAGGGAGGGTAGACTGTTCAACCTGTTCCTGCTCCGGCCTCCACTATAGCA

GATGCGAGCAGGAGTAGGAGAGAGGGAGGTA3 (0.000077%)

TGGGCAGACTTGGTGACCTTGCCAGCTCCAGCAGCCTTCTTGTCCACTGCTTTGATGACACCCACCGCAA
CTGTCTGTCTCATATCACGAACAGCAAAGC 8 (0.000205%)

TGGGGACAGCTCATGAGTGCAAGACGTCTTGTGATGTAATTATTATACGAATGGGGGCTTCAATCGGGA
GTACTACTCGATTGTCAACGTCAAGGAGTCG 9 (0.000231%)

TGGGGGCCAGTGCCCTCCTAATTGGGGGGTAGGGGCTAGGCTGGAGTGGTAAAAGGCTCAGAAAAATC
CTGCGAAGAAAAAAACTTCTGAGGTAATAAAT 7 (0.000180%)

TGGGTCTCAAAATTCTGTGACAAATTTTTGGTCAAGTTGTTTCCATTAAAAAGTACTGATTTTAAAAACT
AATAACTTAAAACTGCCACACGCAAAAAAG 96 (0.002464%)

TGGGTGAGTGAGCCCCATTGTGTTGTGGTAAATATGTAGAGGGAGTATAGGGCTGTGACTAGTATGTTG
AGTCCTGTAAGTAGGAGAGTGATATTTGATC 6 (0.000154%)

TGGGTGCAGGGATGAGGCGTACCAGCACAGAGCCGCAGCGGCCTGTCACCTTGCAAGGGACAGTGTGG
GGCTTGCCGATCTTGTTCCCCCAGTAGCCTCT 4 (0.000103%)

TGGTAAATATGTAGAGGGAGTATAGGGCTGTGACTAGTATGTTGAGTCCTGTAAGTAGGAGAGTGATAT
TTGATCAGGAGAACGTGGTTACTAGCACAGA 4 (0.000103%)

TGGTCAAGTTGTTTCCATTAAAAAGTACTGATTTTAAAAACTAATAACTTAAAACTGCCACACGCAAAA
AAGAAAACCAAAGTGGTCCACAAAACATTCT 12 (0.000308%)

TGGTCTCAAATTTCCACAAGGAGATATCAATGGTGATACCACGTTCACGCTCAGCTTTCAGTTTATCCAA
GACCCAGGCATACTTGAAGGAGCCCTTTCC 4 (0.000103%)

TGGTGACCTTGCCAGCTCCAGCAGCCTTCTTGTCCACTGCTTTGATGACACCCACCGCAACTGTCTGTCT
CATATCACGAACAGCAAAGCGACCCAAAGG 11 (0.000282%)

TGGTGGCCTTGGTATGTGCTTTCTCGTGTTACATCGCGCCATCATTGGTATATGGTTAGTGTGTTGGTTA
GTAGGCCTAGTATGAGGAGCGTTATGGAGT 7 (0.000180%)

TGGTGGGTGAGTGAGCCCCATTGTGTTGTGGTAAATATGTAGAGGGAGTATAGGGCTGTGACTAGTATG
TTGAGTCCTGTAAGTAGGAGAGTGATATTTG 308 (0.007906%)

TGGTTCAGGATAATCACCTGAGCAGTGAAGCCAGCTGCTTCCATTGGTGGGTCATTTTTGCTGTCACCAG
CAACGTTGCCACGACGAACATCCTTGACAG 5 (0.000128%)

TGGTTCTTTTGTCGATGCCACCGCATTTATAGATCAGATGGCCAGTAGTGGTGGACTTGCCCGAATCTAC
GTGTCCAATGACGACAATGTTGATATGAGT 5 (0.000128%)

TGTAAGTGCTGACTTCCTTAACAATTTCCTCATATCTCTTCTGGCTGTAGGGTGGCTCAGTGGAATCCAT
TTTGTTAACACCGACAATTAGTTGTTTCAC7 (0.000180%)

TGTAGAGGGAGTATAGGGCTGTGACTAGTATGTTGAGTCCTGTAAGTAGGAGAGTGATATTTGATCAGG
AGAACGTGGTTACTAGCACAGAGAGTTCTCC 13 (0.000334%)

TGTAGCCAATTTTCTTAATGTAAGTGCTGACTTCCTTAACAATTTCCTCATATCTCTTCTGGCTGTAGGGT
GGCTCAGTGGAATCCATTTTGTTAACACC 19 (0.000488%)

TGTAGCCTGAGAATAGGGGAAATCAGTGAATGAAGCCTCCTATGATGGCAAATACAGCTCCTATTGATA
GGACATAGTGGAAGTGAGCTACAACGTAGTA 21 (0.000539%)

TGTCGATGCCACCGCATTTATAGATCAGATGGCCAGTAGTGGTGGACTTGCCCGAATCTACGTGTCCAA
TGACGACAATGTTGATATGAGTCTTTTCCTT 4 (0.000103%)

TGTGACAAATTTTTGGTCAAGTTGTTTCCATTAAAAAGTACTGATTTTAAAAACTAATAACTTAAAACTG
CCACACGCAAAAAAGAAAACCAAAGTGGTC 7 (0.000180%)

TGTGACCTTCTCTGGCATTCGGGCATTGGCTGTACCCTTCCGCTTACCTATGCCCATGTGCCTGCCCTTCC
GGCGGGCCAAGGTGTTTTTCCGGCATCGA 4 (0.000103%)

TGTGCTTTCTCGTGTTACATCGCGCCATCATTGGTATATGGTTAGTGTGTTGGTTAGTAGGCCTAGTATG
AGGAGCGTTATGGAGTGGAAGTGAAATCAC 5 (0.000128%)

TGTGGCAATCCAATACAGGGGCATAGCCGGCGCTTATTTGGCCTGGATGGTTCAGGATAATCACCTGAG
CAGTGAAGCCAGCTGCTTCCATTGGTGGGTC 13 (0.000334%)

TGTGGGGACAGCTCATGAGTGCAAGACGTCTTGTGATGTAATTATTATACGAATGGGGGCTTCAATCGG
GAGTACTACTCGATTGTCAACGTCAAGGAGT 4 (0.000103%)

TGTGGGTGAAAGAGTATGATGGGGTGGTGGTTGTGGTAAACTTTAATAGTGTAGGAAGCTGAATAATTT
ATGAAGGAGAGGGGTCAGGGTTGATTCGGGA 3 (0.000077%)

TGTTCATAAGGGGATGGCCATGGCTAGGTTTATAGATAGTTGGGTGGTTGGTGTAAATGAGTGAGGCAG
GAGTCCGAGGAGGTTAGTTGTGGCAATAAAA 7 (0.000180%)

TGTTTATGAGCAAGGTGGGTCTCAGAGGTGATCGGCGATCAGAGGGCGATGAAGTTCTAGATCCATTGA
GACAAGCTCTAGACAGTAGCATGCAGTCCCA 7 (0.000180%)

TGTTTCCATTAAAAAGTACTGATTTTAAAAACTAATAACTTAAAACTGCCACACGCAAAAAAGAAAACC
AAAGTGGTCCACAAAACATTCTCCTTTCCTT 12 (0.000308%)

TGTTTTTAAGCCTAATGTGGGGACAGCTCATGAGTGCAAGACGTCTTGTGATGTAATTATTATACGAAT
GGGGGCTTCAATCGGGAGTACTACTCGATTG 7 (0.000180%)

TTAACAATTTCCTCATATCTCTTCTGGCTGTAGGGTGGCTCAGTGGAATCCATTTGTTAACACCGACAA
TTAGTTGTTTCACACCCAGTGTGTAAGCCA 18 (0.000462%)

TTAACCAGTCTTTTACTACTAAACTTAAATGGCCAATTGAAACAAACAGTTCTGAGACCGTTCTTCCACC
ACTGATTAAGAGTGGGGTGGCAGGTATTAG 5 (0.000128%)

TTAATGGGTCTCAAAATTCTGTGACAAATTTTTGGTCAAGTTGTTTCCATTAAAAAGTACTGATTTTAAA
AACTAATAACTTAAAACTGCCACACGCAAA 9 (0.000231%)

TTAATGTAAGTGCTGACTTCCTTAACAATTTCCTCATATCTCTTCTGGCTGTAGGGTGGCTCAGTGGAAT
CCATTTTGTTAACACCGACAATTAGTTGTT5 (0.000128%)

TTACACACTTCCTAATGGCAGAATTTGGCTGTTTGGCTTCAACTCCTACTTTTTCCAGCACGATTCCTTTT
GCATGAGAAGCACCTCCAAAAGGGTTGGC 26 (0.000667%)

TTACTACTAAACTTAAATGGCCAATTGAAACAAACAGTTCTGAGACCGTTCTTCCACCACTGATTAAGA
GTGGGGTGGCAGGTATTAGGGATAATATTCA 3 (0.000077%)

TTAGCCTTCTGAGCTTTCTGGGCAGACTTGGTGACCTTGCCAGCTCCAGCAGCCTTCTTGTCCACTGCTT
TGATGACACCCACCGCAACTGTCTGTCTCA 63 (0.001617%)

TTAGGCACAGGTGCGGAGACGATGCCAGTGCCCCTGGGTGCAGGGATGAGGCGTACCAGCACAGAGCC
GCAGCGGCCTGTCACCTTGCAAGGGACAGTGT3 (0.000077%)

TTAGGGATAATATTCATTTAGCCTTCTGAGCTTTCTGGGCAGACTTGGTGACCTTGCCAGCTCCAGCAGC
CTTCTTGTCCACTGCTTTGATGACACCCAC3 (0.000077%)

TTATCAAGTCAGCACACACCTTTTCCAAGGATTTACGTTGCGGCTTGTTAGGGTGATTCGAATTCGGTG AATTGCCACCTCCGGCTCCACGGGTGTTTT 7 (0.000180%)

TTATGAGCAAGGTGGGTCTCAGAGGTGATCGGCGATCAGAGGGCGATGAAGTTCTAGATCCATTGAGA CAAGCTCTAGACAGTAGCATGCAGTCCCACAA 8 (0.000205%)

TTCAAAGATTTTTAGGGGAATTAATTCTAGGACGATGGGCATGAAACTGTGGTTTGCTCCACAGATTTC AGAGCATTGACCGTAGTATACCCCCGGTCGT 7 (0.000180%)

TTCAAGAATTTAGGGCCATCTTCCAGCTTTTTACCAGAACGGCGATCAATCTTTTCCTTCAGCTCAGCAA ACTTGCATGCAATGTGAGCCGTGTGGCAAT 4 (0.000103%)

TTCAATGGTTCTTTTGTCGATGCCACCGCATTTATAGATCAGATGGCCAGTAGTGGTGGACTTGCCCGAA TCTACGTGTCCAATGACGACAATGTTGATA 3 (0.000077%)

TTCAGCATCTAACAGTTCAAAAGAAGCCACTACATACTCTTTTCACAAATATGTTTTCACAGAGCCAAT ACAGTACTAGCCATTAACCCAGTACACCAAG 3 (0.000077%)

TTCAGCTCAGCAAACTTGCATGCAATGTGAGCCGTGTGGCAATCCAATACAGGGGCATAGCCGGCGCTT ATTTGGCCTGGATGGTTCAGGATAATCACCT 13 (0.000334%)

TTCAGGATAATCACCTGAGCAGTGAAGCCAGCTGCTTCCATTGGTGGGTCATTTTTGCTGTCACCAGCA ACGTTGCCACGACGAACATCCTTGACAGACA 5 (0.000128%)

TTCATAAGGGGATGGCCATGGCTAGGTTTATAGATAGTTGGGTGGTTGGTGTAAATGAGTGAGGCAGGA GTCCGAGGAGGTTAGTTGTGGCAATAAAAAT 3 (0.000077%)

TTCATTTAGCCTTCTGAGCTTTCTGGGCAGACTTGGTGACCTTGCCAGCTCCAGCAGCCTTCTTGTCCAC TGCTTTGATGACACCCACCGCAACTGTCTG 28 (0.000719%)

TTCCACAAGGAGATATCAATGGTGATACCACGTTCACGCTCAGCTTTCAGTTTATCCAAGACCCAGGCA TACTTGAAGGAGCCCTTTCCCATCTCAGCAG 7 (0.000180%)

TTCCATTAAAAAGTACTGATTTTAAAAACTAATAACTTAAAACTGCCACACGCAAAAAAGAAAACCAA AGTGGTCCACAAAACATTCTCCTTTCCTTCTG 75 (0.001925%)

TTCCTAATGGCAGAATTTGGCTGTTTGGCTTCAACTCCTACTTTTTCCAGCACGATTCCTTTTGCATGAGA AGCACCTCCAAAAGGGTTGGCCTTTAGGG 10 (0.000257%)

TTCCTCATATCTCTTCTGGCTGTAGGGTGGCTCAGTGGAATCCATTTTGTTAACACCGACAATTAGTTGT TTCACACCCAGTGTGTAAGCCAGAAGGGCA 23 (0.000590%)

TTCCTTAACAATTTCCTCATATCTCTTCTGGCTGTAGGGTGGCTCAGTGGAATCCATTTTGTTAACACCG ACAATTAGTTGTTTCACACCCAGTGTGTAA 24 (0.000616%)

TTCCTTCAGCTCAGCAAACTTGCATGCAATGTGAGCCGTGTGGCAATCCAATACAGGGGCATAGCCGGC GCTTATTTGGCCTGGATGGTTCAGGATAATC 6 (0.000154%)

TTCTAGGATAGTCAGTAGAATTAGAATTGTGAAGATGATAAGTGTAGAGGGAAGGTTAATGGTTGATAT TGCTAGGGTGGCGCTTCCAATTAGGTGCATG 3 (0.000077%)

TTCTCAAATTTTTCAATGGTTCTTTTGTCGATGCCACCGCATTTATAGATCAGATGGCCAGTAGTGGTGG ACTTGCCCGAATCTACGTGTCCAATGACGA 7 (0.000180%)

TTCTCGTGTTACATCGCGCCATCATTGGTATATGGTTAGTGTGTTGGTTAGTAGGCCTAGTATGAGGAGC GTTATGGAGTGGAAGTGAAATCACATGGCT 7 (0.000180%)

TTCTGAGCTTTCTGGGCAGACTTGGTGACCTTGCCAGCTCCAGCAGCCTTCTTGTCCACTGCTTTGATGA

CACCCACCGCAACTGTCTGTCTCATATCAC 5 (0.000128%)

TTCTGGGCAGACTTGGTGACCTTGCCAGCTCCAGCAGCCTTCTTGTCCACTGCTTTGATGACACCCACCG
CAACTGTCTGTCTCATATCACGAACAGCAA 10 (0.000257%)

TTCTGTGACAAATTTTTGGTCAAGTTGTTTCCATTAAAAAGTACTGATTTTAAAAACTAATAACTTAAAA
CTGCCACACGCAAAAAAGAAAACCAAAGTG 7 (0.000180%)

TTCTTAATGTAAGTGCTGACTTCCTTAACAATTTCCTCATATCTCTTCTGGCTGTAGGGTGGCTCAGTGG
AATCCATTTTGTTAACACCGACAATTAGTT 5 (0.000128%)

TTCTTGTCCACTGCTTTGATGACACCCACCGCAACTGTCTGTCTCATATCACGAACAGCAAAGCGACCCA
AAGGTGGATAGTCTGAGAAGCTCTCAACAC 12 (0.000308%)

TTGAGAGAGTGAGGAGAAGGCTTACGTTTAGTGAGGGAGAGATTTGGTATATGATTGAGATGGGGGCT
AGTTTTTGTCATGTGAGAAGAAGCAGGCCGGA8 (0.000205%)

TTGCATGCAATGTGAGCCGTGTGGCAATCCAATACAGGGGCATAGCCGGCGCTTATTTGGCCTGGATGG
TTCAGGATAATCACCTGAGCAGTGAAGCCAG 4 (0.000103%)

TTGCCAGCTCCAGCAGCCTTCTTGTCCACTGCTTTGATGACACCCACCGCAACTGTCTGTCTCATATCAC
GAACAGCAAAGCGACCCAAAGGTGGATAGT 4 (0.000103%)

TTGCTGGTCTCAAATTTCCACAAGGAGATATCAATGGTGATACCACGTTCACGCTCAGCTTTCAGTTTAT
CCAAGACCCAGGCATACTTGAAGGAGCCCT 5 (0.000128%)

TTGGCGTAGGTTTGGTCTAGGGTGTAGCCTGAGAATAGGGGAAATCAGTGAATGAAGCCTCCTATGATG
GCAAATACAGCTCCTATTGATAGGACATAGT 3 (0.000077%)

TTGGGATTGAAAAGTGAACAGATATTCAGCATCTAACAGTTCAAAAGAAGCCACTACATACTCTTTTCA
CAAATATGTTTTCACAGAGCCAATACAGTAC 99 (0.002541%)

TTGGGGGCCAGTGCCCTCCTAATTGGGGGGTAGGGGCTAGGCTGGAGTGGTAAAAGGCTCAGAAAAAT
CCTGCGAAGAAAAAAACTTCTGAGGTAATAAA 10 (0.000257%)

TTGGTATGTGCTTTCTCGTGTTACATCGCGCCATCATTGGTATATGGTTAGTGTGTTGGTTAGTAGGCCT
AGTATGAGGAGCGTTATGGAGTGGAAGTGA 19 (0.000488%)

TTGGTCAAGTTGTTTCCATTAAAAAGTACTGATTTTAAAAACTAATAACTTAAAACTGCCACACGCAAA
AAAGAAAACCAAAGTGGTCCACAAAACATTC 3 (0.000077%)

TTGGTCTAGGGTGTAGCCTGAGAATAGGGGAAATCAGTGAATGAAGCCTCCTATGATGGCAAATACAG
CTCCTATTGATAGGACATAGTGGAAGTGAGCT 16 (0.000411%)

TTGGTGACCTTGCCAGCTCCAGCAGCCTTCTTGTCCACTGCTTTGATGACACCCACCGCAACTGTCTGTC
TCATATCACGAACAGCAAAGCGACCCAAAG 6 (0.000154%)

TTGGTTCCCCGGAGACGTCCAGTCCGGCGGGCAGCAATGAGACCCACTTTGCGGCCAGCAGGGGCATCT
CTGCGGATGGTGGAGGGCTTGCCGATGTGCT 3 (0.000077%)

TTGTAGCCAATTTTCTTAATGTAAGTGCTGACTTCCTTAACAATTTCCTCATATCTCTTCTGGCTGTAGGG
TGGCTCAGTGGAATCCATTTTGTTAACAC 7 (0.000180%)

TTGTCCACTGCTTTGATGACACCCACCGCAACTGTCTGTCTCATATCACGAACAGCAAAGCGACCCAAA
GGTGGATAGTCTGAGAAGCTCTCAACACACA 6 (0.000154%)

TTGTCGATGCCACCGCATTTATAGATCAGATGGCCAGTAGTGGTGGACTTGCCCGAATCTACGTGTCCA
ATGACGACAATGTTGATATGAGTCTTTTCCT 3 (0.000077%)

TTGTGTTGTGGTAAATATGTAGAGGGAGTATAGGGCTGTGACTAGTATGTTGAGTCCTGTAAGTAGGAG
AGTGATATTTGATCAGGAGAACGTGGTTACT 7 (0.000180%)

TTGTTTATGAGCAAGGTGGGTCTCAGAGGTGATCGGCGATCAGAGGGCGATGAAGTTCTAGATCCATTG
AGACAAGCTCTAGACAGTAGCATGCAGTCCC 6 (0.000154%)

TTTAAGCCTAATGTGGGGACAGCTCATGAGTGCAAGACGTCTTGTGATGTAATTATTATACGAATGGGG
GCTTCAATCGGGAGTACTACTCGATTGTCAA 8 (0.000205%)

TTTAATCTTAGAGCGAAAGCCTATAATCACTGCGCCTGTTCATAAGGGGATGGCCATGGCTAGGTTTAT
AGATAGTTGGGTGGTTGGTGTAAATGAGTGA 10 (0.000257%)

TTTAATGGGTCTCAAAATTCTGTGACAAATTTTGGTCAAGTTGTTTCCATTAAAAAGTACTGATTTTAA
AAACTAATAACTTAAAACTGCCACACGCAA 5 (0.000128%)

TTTACTACTAAACTTAAATGGCCAATTGAAACAAACAGTTCTGAGACCGTTCTTCCACCACTGATTAAG
AGTGGGGTGGCAGGTATTAGGGATAATATTC 19 (0.000488%)

TTTAGCCTTCTGAGCTTTCTGGGCAGACTTGGTGACCTTGCCAGCTCCAGCAGCCTTCTTGTCCACTGCT
TTGATGACACCCACCGCAACTGTCTGTCTC 21 (0.000539%)

TTTAGGGCCATCTTCCAGCTTTTACCAGAACGGCGATCAATCTTTTCCTTCAGCTCAGCAAACTTGCAT
GCAATGTGAGCCGTGTGGCAATCCAATACA 14 (0.000359%)

TTTATGAGCAAGGTGGGTCTCAGAGGTGATCGGCGATCAGAGGGCGATGAAGTTCTAGATCCATTGAG
ACAAGCTCTAGACAGTAGCATGCAGTCCCACA 15 (0.000385%)

TTTCAAAGATTTTTAGGGGAATTAATTCTAGGACGATGGGCATGAAACTGTGGTTTGCTCCACAGATTTC
AGAGCATTGACCGTAGTATACCCCCGGTCG 13 (0.000334%)

TTTCAATGGTTCTTTTGTCGATGCCACCGCATTTATAGATCAGATGGCCAGTAGTGGTGGACTTGCCCGA
ATCTACGTGTCCAATGACGACAATGTTGAT 3 (0.000077%)

TTTCCATTAAAAAGTACTGATTTTAAAAACTAATAACTTAAAACTGCCACACGCAAAAAAGAAAACCAA
AGTGGTCCACAAAACATTCTCCTTTCCTTCT 65 (0.001669%)

TTTCCTCATATCTCTTCTGGCTGTAGGGTGGCTCAGTGGAATCCATTTTGTTAACACCGACAATTAGTTG
TTTCACACCCAGTGTGTAAGCCAGAAGGGC 9 (0.000231%)

TTTCCTTCAGCTCAGCAAACTTGCATGCAATGTGAGCCGTGTGGCAATCCAATACAGGGGCATAGCCGG
CGCTTATTTGGCCTGGATGGTTCAGGATAAT 9 (0.000231%)

TTTCTAGGATAGTCAGTAGAATTAGAATTGTGAAGATGATAAGTGTAGAGGGAAGGTTAATGGTTGATA
TTGCTAGGGTGGCGCTTCCAATTAGGTGCAT 3 (0.000077%)

TTTCTCGTGTTACATCGCGCCATCATTGGTATATGGTTAGTGTGTTGGTTAGTAGGCCTAGTATGAGGAG
CGTTATGGAGTGGAAGTGAAATCACATGGC 4 (0.000103%)

TTTCTGGGCAGACTTGGTGACCTTGCCAGCTCCAGCAGCCTTCTTGTCCACTGCTTTGATGACACCCACC
GCAACTGTCTGTCTCATATCACGAACAGCA 9 (0.000231%)

TTTCTTAATGTAAGTGCTGACTTCCTTAACAATTTCCTCATATCTCTTCTGGCTGTAGGGTGGCTCAGTGG
AATCCATTTTGTTAACACCGACAATTAGT 7 (0.000180%)

TTTCTTTGCATAATCCAGGGAATCATAAATCATGCCAAAGCCAGTTGTCTTGCCACCACCAAAATGAGT
TCTGAATCCAAATACAAAGATGACATCCGGT 4 (0.000103%)

TTTGCATAATCCAGGGAATCATAAATCATGCCAAAGCCAGTTGTCTTGCCACCACCAAAATGAGTTCTG
AATCCAAATACAAAGATGACATCCGGTGTGG 6 (0.000154%)

TTTGGCGTAGGTTTGGTCTAGGGTGTAGCCTGAGAATAGGGGAAATCAGTGAATGAAGCCTCCTATGAT
GGCAAATACAGCTCCTATTGATAGGACATAG 13 (0.000334%)

TTTGGCTGTTTGGCTTCAACTCCTACTTTTCCAGCACGATTCCTTTTGCATGAGAAGCACCTCCAAAAG
GGTTGGCCTTTAGGGCTGTGCCCAAATGAG 5 (0.000128%)

TTTGGTCAAGTTGTTTCCATTAAAAAGTACTGATTTTAAAAACTAATAACTTAAAACTGCCACACGCAA
AAAAGAAAACCAAAGTGGTCCACAAAACATT 4 (0.000103%)

TTTGGTCTAGGGTGTAGCCTGAGAATAGGGGAAATCAGTGAATGAAGCCTCCTATGATGGCAAATACA
GCTCCTATTGATAGGACATAGTGGAAGTGAGC3 (0.000077%)

TTTGTCGATGCCACCGCATTTATAGATCAGATGGCCAGTAGTGGTGGACTTGCCCGAATCTACGTGTCC
AATGACGACAATGTTGATATGAGTCTTTTCC 19 (0.000488%)

TTTGTTTATGAGCAAGGTGGGTCTCAGAGGTGATCGGCGATCAGAGGGCGATGAAGTTCTAGATCCATT
GAGACAAGCTCTAGACAGTAGCATGCAGTCC 108 (0.002772%)

TTTTAAGCCTAATGTGGGGACAGCTCATGAGTGCAAGACGTCTTGTGATGTAATTATTATACGAATGGG
GGCTTCAATCGGGAGTACTACTCGATTGTCA 16 (0.000411%)

TTTTAATCTTAGAGCGAAAGCCTATAATCACTGCGCCTGTTCATAAGGGGATGGCCATGGCTAGGTTTA
TAGATAGTTGGGTGGTTGGTGTAAATGAGTG 47 (0.001206%)

TTTTAATGGGTCTCAAAATTCTGTGACAAATTTTTGGTCAAGTTGTTTCCATTAAAAAGTACTGATTTTA
AAAACTAATAACTTAAAACTGCCACACGCA 33 (0.000847%)

TTTTACCAGAACGGCGATCAATCTTTTCCTTCAGCTCAGCAAACTTGCATGCAATGTGAGCCGTGTGGCA
ATCCAATACAGGGGCATAGCCGGCGCTTAT 4 (0.000103%)

TTTTACTACTAAACTTAAATGGCCAATTGAAACAAACAGTTCTGAGACCGTTCTTCCACCACTGATTAAG
AGTGGGGTGGCAGGTATTAGGGATAATATT 58 (0.001489%)

TTTTCAATGGTTCTTTTGTCGATGCCACCGCATTTATAGATCAGATGGCCAGTAGTGGTGGACTTGCCCG
AATCTACGTGTCCAATGACGACAATGTTGA 8 (0.000205%)

TTTTCCTCCTGTAGGCTGGCAGAGGACAGTGGAGCAGCCAACACACAAAACTACCGTTTGTGCATGGCT
AAAGACCGTGGTGATTTTATAGCATCCTGGG 448 (0.011500%)

TTTTCCTTCAGCTCAGCAAACTTGCATGCAATGTGAGCCGTGTGGCAATCCAATACAGGGGCATAGCCG
GCGCTTATTTGGCCTGGATGGTTCAGGATAA 36 (0.000924%)

TTTTCTTAATGTAAGTGCTGACTTCCTTAACAATTTCCTCATATCTCTTCTGGCTGTAGGGTGGCTCAGTG
GAATCCATTTTGTTAACACCGACAATTAG 18 (0.000462%)

TTTTCTTTGCATAATCCAGGGAATCATAAATCATGCCAAAGCCAGTTGTCTTGCCACCACCAAAATGAG
TTCTGAATCCAAATACAAAGATGACATCCGG 10 (0.000257%)

TTTTGGCGTAGGTTTGGTCTAGGGTGTAGCCTGAGAATAGGGGAAATCAGTGAATGAAGCCTCCTATGA
TGGCAAATACAGCTCCTATTGATAGGACATA 24 (0.000616%)

TTTTGGTCAAGTTGTTTCCATTAAAAAGTACTGATTTTAAAAACTAATAACTTAAAACTGCCACACGCAA
AAAAGAAAACCAAAGTGGTCCACAAAACAT 10 (0.000257%)

TTTTGTCGATGCCACCGCATTTATAGATCAGATGGCCAGTAGTGGTGGACTTGCCCGAATCTACGTGTCC

AATGACGACAATGTTGATATGAGTCTTTTC 23 (0.000590%)

TTTTGTTTATGAGCAAGGTGGGTCTCAGAGGTGATCGGCGATCAGAGGGCGATGAAGTTCTAGATCCAT
TGAGACAAGCTCTAGACAGTAGCATGCAGTC 31 (0.000796%)

TTTTTAAGCCTAATGTGGGGACAGCTCATGAGTGCAAGACGTCTTGTGATGTAATTATTATACGAATGG
GGGCTTCAATCGGGAGTACTACTCGATTGTC 4 (0.000103%)

TTTTTAATGGGTCTCAAAATTCTGTGACAAATTTTTGGTCAAGTTGTTTCCATTAAAAAGTACTGATTTT
AAAAACTAATAACTTAAAACTGCCACACGC 9 (0.000231%)

TTTTTCTTTGCATAATCCAGGGAATCATAAATCATGCCAAAGCCAGTTGTCTTGCCACCACCAAAATGA
GTTCTGAATCCAAATACAAAGATGACATCCG 10 (0.000257%)

TTTTTGGTCAAGTTGTTTCCATTAAAAAGTACTGATTTTAAAAACTAATAACTTAAAACTGCCACACGCA
AAAAAGAAAACCAAAGTGGTCCACAAAACA 14 (0.000359%)

TTTTTTAATGGGTCTCAAAATTCTGTGACAAATTTTTGGTCAAGTTGTTTCCATTAAAAAGTACTGATTTT
AAAAACTAATAACTTAAAACTGCCACACG 14 (0.000359%)

TTTTTTTTTTTTTTTTTTTTTTTTTTTTTTTTTTTTTTTTTTTTTTTTTTTTTTTTTTTTTTTTTTTTTTTTTT
TTTTTTTTTTTTTTTTTTTTTTT 902 (0.023154%)

TTTTTTTTTTTTTTTTTTTTTTTTTTTTTTTTTTTTTTTTTTTTTTTTTTTTTTTTTTTTTTTTTTTTTTTTTT
TTTTTTTTTTTTTTTTTTTTTTTTTTTTTTTTTTTTTTTTTTTTTTTTTTTTTTTTTTTTTTTTTTTTTT378
(0.014458%)

Before filtering: read2: quality
Value of each position will be shown on mouse over.
2040608010012014037.53838.53939.5

positionqualityATCGmean

<https://plot.ly/>
Before filtering: read2: base contents
Value of each position will be shown on mouse over.
2040608010012014000.10.20.30.40.50.6

positionbase content ratiosA(26.51%)T(25.05%)C(23.81%)G(24.53%)N(0.077%)GC(48.35%)

<https://plot.ly/>
Before filtering: read2: KMER counting
Darker background means larger counts. The count will be shown on mouse over.

AA AT AC AG TA TT TC TG CA CT CC CG GA GT GC GG
AAA AAAAA AAAAT AAAAC AAAAG AAATA AAATT AAATC AAATG AAACA AAACT
AAACC
AAACG AAAGA AAAGT AAAGC AAAGG
AAT AATAA AATAT AATAC AATAG AATTA AATTT AATTC AATTG AATCA AATCT
AATCC
AATCGAATGA AATGT AATGC AATGG
AAC AACAA AACAT AACAC AACAG AACTA AACTT AACTC AACTG AACCA AACCT
AACCC
AACCG AACGA AACGT AACGC AACGG
AAG AAGAA AAGAT AAGAC AAGAG AAGTA AAGTT AAGTC AAGTG AAGCA AAGCT
AAGCC
AAGCG AAGGA AAGGT AAGGC AAGGG
ATA ATAAA ATAAT ATAAC ATAAG ATATA ATATT ATATC ATATG ATACA ATACT

ATACC
ATACG ATAGA ATAGT ATAGC ATAGG
ATT ATTAA ATTAT ATTAC ATTAG ATTTA ATTTT ATTTC ATTTG ATTCA ATTCT ATTCC
ATTCG ATTGA ATTGT ATTGC ATTGG
ATC ATCAA ATCAT ATCAC ATCAG ATCTA ATCTT ATCTC ATCTG ATCCA ATCCT ATCCC
ATCCG ATCGA ATCGT ATCGC ATCGG
ATG ATGAA ATGAT ATGAC ATGAG ATGTA ATGTT ATGTC ATGTG ATGCA ATGCT
ATGCC
ATGCG ATGGA ATGGT ATGGC ATGGG
ACA ACAAA ACAAT ACAAC ACAAG ACATA ACATT ACATC ACATG ACACA ACACT
ACACC
ACACG ACAGA ACAGT ACAGC ACAGG
ACT ACTAA ACTAT ACTAC ACTAG ACTTA ACTTT ACTTC ACTTG ACTCA ACTCT ACTCC
ACTCG ACTGA ACTGT ACTGC ACTGG
ACC ACCAA ACCAT ACCAC ACCAG ACCTA ACCTT ACCTC ACCTG ACCCA ACCCT
ACCCC
ACCCG ACCGA ACCGT ACCGC ACCGG
ACG ACGAA ACGAT ACGAC ACGAG ACGTA ACGTT ACGTC ACGTG ACGCA ACGCT
ACGCC
ACGCG ACGGA ACGGT ACGGC ACGGG
AGA AGAAA AGAAT AGAAC AGAAG AGATA AGATT AGATC AGATG AGACA AGACT
AGACC
AGACG AGAGA AGAGT AGAGC AGAGG
AGT AGTAA AGTAT AGTAC AGTAG AGTTA AGTTT AGTTC AGTTG AGTCA AGTCT
AGTCC
AGTCG AGTGA AGTGT AGTGC AGTGG
AGC AGCAA AGCAT AGCAC AGCAG AGCTA AGCTT AGCTC AGCTG AGCCA AGCCT
AGCCC
AGCCG AGCGA AGCGT AGCGC AGCGG
AGG AGGAA AGGAT AGGAC AGGAG AGGTA AGGTT AGGTC AGGTG AGGCA AGGCT
AGGCC
AGGCG AGGGA AGGGT AGGGC AGGGG
TAA TAAAA TAAAT TAAAC TAAAG TAATA TAATT TAATC TAATG TAACA TAACT
TAACC
TAACG TAAGA TAAGT TAAGC TAAGG
TAT TATAA TATAT TATAC TATAG TATTA TATTT TATTC TATTG TATCA TATCT TATCC
TATCG TATGA TATGT TATGC TATGG
TAC TACAA TACAT TACAC TACAG TACTA TACTT TACTC TACTG TACCA TACCT TACCC
TACCG TACGA TACGT TACGC TACGG
TAG TAGAA TAGAT TAGAC TAGAG TAGTA TAGTT TAGTC TAGTG TAGCA TAGCT
TAGCC
TAGCG TAGGA TAGGT TAGGC TAGGG
TTA TTAAA TTAAT TTAAC TTAAG TTATA TTATT TTATC TTATG TTACA TTACT TTACC
TTACG TTAGA TTAGT TTAGC TTAGG
TTT TTTAA TTTAT TTTAC TTTAG TTTTA TTTTT TTTTC TTTTG TTTCA TTTCT TTTCC
TTTCG TTTGA TTTGT TTTGC TTTGG
TTC TTCAA TTCAT TTCAC TTCAG TTCTA TTCTT TTCTC TTCTG TTCCA TTCCT TTCCC
TTCCG TTCGA TTCGT TTCGC TTCGG
TTG TTGAA TTGAT TTGAC TTGAG TTGTA TTGTT TTGTC TTGTG TTGCA TTGCT TTGCC
TTGCG TTGGA TTGGT TTGGC TTGGG
TCA TCAAA TCAAT TCAAC TCAAG TCATA TCATT TCATC TCATG TCACA TCACT TCACC
TCACG TCAGA TCAGT TCAGC TCAGG
TCT TCTAA TCTAT TCTAC TCTAG TCTTA TCTTT TCTTC TCTTG TCTCA TCTCT TCTCC
TCTCG TCTGA TCTGT TCTGC TCTGG
TCC TCCAA TCCAT TCCAC TCCAG TCCTA TCCTT TCCTC TCCTG TCCCA TCCCT TCCCC
TCCCG TCCGA TCCGT TCCGC TCCGG
TCG TCGAA TCGAT TCGAC TCGAG TCGTA TCGTT TCGTC TCGTG TCGCA TCGCT TCGCC
TCGCG TCGGA TCGGT TCGGC TCGGG

TGA TGAAA TGAAT TGAAC TGAAG TGATA TGATT TGATC TGATG TGACA TGACT
TGACC
TGACGTGAGA TGAGT TGAGC TGAGG
TGT TGTAA TGTAT TGTAC TGTAG TGTTA TGTTT TGTTC TGTTG TGTCA TGTCT TGTCC
TGTCG TGTGA TGTGT TGTGC TGTGG
TGC TGCAA TGCAT TGCAC TGCAG TGCTA TGCTT TGCTC TGCTG TGCCA TGCCT TGCCC
TGCCGTGCGA TGCGT TGCGC TGCGG
TGG TGGAA TGGAT TGGAC TGGAG TGGTA TGGTT TGGTC TGGTG TGGCA TGGCT
TGGCC
TGGCGTGGGA TGGGT TGGGC TGGGG
CAA CAAAA CAAAT CAAAC CAAAG CAATA CAATT CAATC CAATG CAACA CAACT
CAACC
CAACG CAAGA CAAGT CAAGC CAAGG
CAT CATAA CATAT CATAC CATAG CATTA CATTT CATTC CATTG CATCA CATCT CATCC
CATCG CATGA CATGT CATGC CATGG
CAC CACAA CACAT CACAC CACAG CACTA CACTT CACTC CACTG CACCA CACCT
CACCC
CACCGCACGA CACGT CACGC CACGG
CAG CAGAA CAGAT CAGAC CAGAG CAGTA CAGTT CAGTC CAGTG CAGCA CAGCT
CAGCC
CAGCG CAGGA CAGGT CAGGC CAGGG
CTA CTAAA CTAAT CTAAC CTAAG CTATA CTATT CTATC CTATG CTACA CTACT CTACC
CTACGCTAGA CTAGT CTAGC CTAGG
CTT CTTAA CTTAT CTTAC CTTAG CTTTA CTTTT CTTTC CTTTG CTTCA CTTCT CTTCC
CTTCG CTTGA CTTGT CTTGC CTTGG
CTC CTCAA CTCAT CTCAC CTCAG CTCTA CTCTT CTCTC CTCTG CTCCA CTCCT CTCCC
CTCCG CTCGA CTCGT CTCGC CTCGG
CTG CTGAA CTGAT CTGAC CTGAG CTGTA CTGTT CTGTC CTGTG CTGCA CTGCT CTGCC
CTGCG CTGGA CTGGT CTGGC CTGGG
CCA CCAAA CCAAT CCAAC CCAAG CCATA CCATT CCATC CCATG CCACA CCACT
CCACC
CCACGCCAGA CCAGT CCAGC CCAGG
CCT CCTAA CCTAT CCTAC CCTAG CCTTA CCTTT CCTTC CCTTG CCTCA CCTCT CCTCC
CCTCG CCTGA CCTGT CCTGC CCTGG
CCC CCCAA CCCAT CCCAC CCCAG CCCTA CCCTT CCCTC CCCTG CCCCA CCCCT CCCCC
CCCCG CCCGA CCCGT CCCGC CCCGG
CCG CCGAA CCGAT CCGAC CCGAG CCGTA CCGTT CCGTC CCGTG CCGCA CCGCT
CCGCC
CCGCGCCGGA CCGGT CCGGC CCGGG
CGA CGAAA CGAAT CGAAC CGAAG CGATA CGATT CGATC CGATG CGACA CGACT
CGACC
CGACG CGAGA CGAGT CGAGC CGAGG
CGT CGTAA CGTAT CGTAC CGTAG CGTTA CGTTT CGTTC CGTTG CGTCA CGTCT CGTCC
CGTCG CGTGA CGTGT CGTGC CGTGG
CGC CGCAA CGCAT CGCAC CGCAG CGCTA CGCTT CGCTC CGCTG CGCCA CGCCT
CGCCC
CGCCGCGCGA CGCGT CGCGC CGCGG
CGG CGGAA CGGAT CGGAC CGGAG CGGTA CGGTT CGGTC CGGTG CGGCA CGGCT
CGGCC
CGGCG CGGGA CGGGT CGGGC CGGGG
GAA GAAAA GAAAT GAAAC GAAAG GAATA GAATT GAATC GAATG GAACA GAACT
GAACC
GAACG GAAGA GAAGT GAAGC GAAGG
GAT GATAA GATAT GATAC GATAG GATTA GATTT GATTC GATTG GATCA GATCT
GATCC
GATCGGATGA GATGT GATGC GATGG
GAC GACAA GACAT GACAC GACAG GACTA GACTT GACTC GACTG GACCA GACCT
GACCC

GACCG GACGA GACGT GACGC GACGG
GAG GAGAA GAGAT GAGAC GAGAG GAGTA GAGTT GAGTC GAGTG GAGCA GAGCT
GAGCC
GAGCG GAGGA GAGGT GAGGC GAGGG
GTA GTAAA GTAAT GTAAC GTAAG GTATA GTATT GTATC GTATG GTACA GTACT
GTACC
GTACGGTAGA GTAGT GTAGC GTAGG
GTT GTTAA GTTAT GTTAC GTTAG GTTTA GTTTT GTTTC GTTTG GTTCA GTTCT GTTCC
GTTCGGTTGA GTTGT GTTGC GTTGG
GTC GTCAA GTCAT GTCAC GTCAG GTCTA GTCTT GTCTC GTCTG GTCCA GTCCT GTCCC
GTCCGGTCGA GTCGT GTCGC GTCGG
GTG GTGAA GTGAT GTGAC GTGAG GTGTA GTGTT GTGTC GTGTG GTGCA GTGCT
GTGCC
GTGCGGTGGA GTGGT GTGGC GTGGG
GCA GCAAA GCAAT GCAAC GCAAG GCATA GCATT GCATC GCATG GCACA GCACT
GCACC
GCACG GCAGA GCAGT GCAGC GCAGG
GCT GCTAA GCTAT GCTAC GCTAG GCTTA GCTTT GCTTC GCTTG GCTCA GCTCT GCTCC
GCTCGGCTGA GCTGT GCTGC GCTGG
GCC GCCAA GCCAT GCCAC GCCAG GCCTA GCCTT GCCTC GCCTG GCCCA GCCCT
GCCCC
GCCCGGCCGA GCCGT GCCGC GCCGG
GCG GCGAA GCGAT GCGAC GCGAG GCGTA GCGTT GCGTC GCGTG GCGCA GCGCT
GCGCC
GCGCG GCGGA GCGGT GCGGC GCGGG
GGA GGAAA GGAAT GGAAC GGAAG GGATA GGATT GGATC GGATG GGACA GGACT
GGACC
GGACG GGAGA GGAGT GGAGC GGAGG
GGT GGTAA GGTAT GGTAC GGTAG GGTTA GGTTT GGTTC GGTTG GGTCA GGTCT
GGTCC
GGTCGGGTGA GGTGT GGTGC GGTGG
GGC GGCAA GGCAT GGCAC GGCAG GGCTA GGCTT GGCTC GGCTG GGCCA GGCCT
GGCCC
GGCCG GGCGA GGCGT GGCGC GGCGG
GGG GGGAA GGGAT GGGAC GGGAG GGGTA GGGTT GGGTC GGGTG GGGCA GGGCT
GGGCC
GGGCG GGGGA GGGGT GGGGC GGGGG

Before filtering: read2: overrepresented sequences
Sampling rate: 1 / 20
overrepresented sequence count (% of bases) distribution: cycle 1 ~
cycle 151

AAAAAAAAAAAAAAAAAAAAAAAAAAAAAAAAAAAAAAAAAAAAAAAAAAAAAAAAAAAAAAAAAAA
AAAAAAAAAAAAAAAAAAAAAAAAAAAAAAAAAAAA 231 (0.005925%)

AAAAAAAAGGCCGCCGTGACCTATTCACCCTCCACTTCCCGTCTCAGAATCTAAACGTGGTCACCTTCG
AGTAGAGAGGCCCGCCCGCCCACCGTGGGCA 486 (0.012465%)

AAAAAAAGGCCGCCGTGACCTATTCACCCTCCACTTCCCGTCTCAGAATCTAAACGTGGTCACCTTCGA
GTAGAGAGGCCCGCCCGCCCACCGTGGGCAG 19 (0.000487%)

AAAAAAGGCCGCCGTGACCTATTCACCCTCCACTTCCCGTCTCAGAATCTAAACGTGGTCACCTTCGAG
TAGAGAGGCCCGCCCGCCCACCGTGGGCAGT 21 (0.000539%)

AAAAAATTATAACAAACCCTGAGAACCAAAATGAACGAAAATCTGTTCGCTTCATTCATTGCCCCCACA
ATCCTAGGCCTACCCGCCGCAGTACTGATCA 54 (0.001385%)

AAAAAGCTGGAAGATGGCCCTAAATTCTTGAAGTCTGGTGATGCTGCCATTGTTGATATGGTTCCTGGC
AAGCCCATGTGTGTTGAGAGCTTCTCAGACT 25 (0.000641%)

AAAAAGGCCGCCGTGACCTATTCACCCTCCACTTCCCGTCTCAGAATCTAAACGTGGTCACCTTCGAGT
AGAGAGGCCCGCCCGCCCACCGTGGGCAGTG 14 (0.000359%)

AAAAAGGGCCGTTCTGCCATCAACGAAGTGGTAACCCGAGAATACACCATCAACATTCACAAGCGCAT
CCATGGAGTGGGCTTCAAGAAGCGTGCACCTC28 (0.000718%)

AAAAATAAAAAATTATAACAAACCCTGAGAACCAAAATGAACGAAAATCTGTTCGCTTCATTCATTGCC
CCCACAATCCTAGGCCTACCCGCCGCAGTAC 27 (0.000692%)

AAAAATGACCCACCAATGGAAGCAGCTGGCTTCACTGCTCAGGTGATTATCCTGAACCATCCAGGCCAA
ATAAGCGCCGGCTATGCCCCTGTATTGGATT 16 (0.000410%)

AAAAATTATAACAAACCCTGAGAACCAAAATGAACGAAAATCTGTTCGCTTCATTCATTGCCCCCACAA
TCCTAGGCCTACCCGCCGCAGTACTGATCAT 87 (0.002231%)

AAAACAAATGATAACCATACACAACACTAAAGGACGAACCTGATCTCTTATACTAGTATCCTTAATCAT
TTTTATTGCCACAACTAACCTCCTCGGACTC 9 (0.000231%)

AAAACAAGAAGAGGGTCCTGCTGGGAGAAACTGGCAAGGAGAAGCTCCCGCGGTACTACAAGAACAT
CGGTCTGGGCTTCAAGACACCCAAGGAGGCTAT 3 (0.000077%)

AAAACCCAGCCCATGACCCCTAACAGGGGCCCTCTCAGCCCTCCTAATGACCTCCGGCCTAGCCATGTG
ATTTCACTTCCACTCCATAACGCTCCTCATA 36 (0.000923%)

AAAACCCGCCGGACTTTCTGTAAGAAGTGTGGCAAGCACCAACCCCATAAAGTGACACAGTACAAGAA
GGGCAAGGATTCTCTGTACGCCCAGGGAAAGC 3 (0.000077%)

AAAACTACCCCTAAAAGCCAAAATGGGAAAGGAAAAGACTCATATCAACATTGTCGTCATTGGACACG
TAGATTCGGGCAAGTCCACCACTACTGGCCAT 23 (0.000590%)

AAAAGAAGAAAGATGAGGCAGAGGTCCAAGTAAACCGCTAGCTTGTTGCACCGTGGAGGCCACAGGA
GCAGAAACATGGAATGCCAGACGCTGGGGATGC 10 (0.000256%)

AAAAGACTCATATCAACATTGTCGTCATTGGACACGTAGATTCGGGCAAGTCCACCACTACTGGCCATC
TGATCTATAAATGCGGTGGCATCGACAAAAG 131 (0.003360%)

AAAAGCAGCCGACCATCTTTCAAAACAAGAAGAGGGTCCTGCTGGGAGAAACTGGCAAGGAGAAGCTC
CCGCGGTACTACAAGAACATCGGTCTGGGCTT 5 (0.000128%)

AAAAGCCAAAATGGGAAAGGAAAAGACTCATATCAACATTGTCGTCATTGGACACGTAGATTCGGGCA
AGTCCACCACTACTGGCCATCTGATCTATAAA 24 (0.000616%)

AAAAGCTGGAAGATGGCCCTAAATTCTTGAAGTCTGGTGATGCTGCCATTGTTGATATGGTTCCTGGCA
AGCCCATGTGTGTTGAGAGCTTCTCAGACTA 35 (0.000898%)

AAAAGGCCGCCGTGACCTATTCACCCTCCACTTCCCGTCTCAGAATCTAAACGTGGTCACCTTCGAGTA
GAGAGGCCCGCCCGCCCACCGTGGGCAGTGC 4 (0.000103%)

AAAAGGGCCGTTCTGCCATCAACGAAGTGGTAACCCGAGAATACACCATCAACATTCACAAGCGCATC
CATGGAGTGGGCTTCAAGAAGCGTGCACCTCG 21 (0.000539%)

AAAATAAAAAATTATAACAAACCCTGAGAACCAAAATGAACGAAAATCTGTTCGCTTCATTCATTGCCC
CCACAATCCTAGGCCTACCCGCCGCAGTACT 8 (0.000205%)

AAAATCTGTCGAAATGCACCATGAAGCTTTGAGTGAAGCTCTTCCTGGGGACAATGTGGGCTTCAATGT

CAAGAATGTGTCTGTCAAGGATGTTCGTCGT 13 (0.000333%)

AAAATGACCCACCAATGGAAGCAGCTGGCTTCACTGCTCAGGTGATTATCCTGAACCATCCAGGCCAAA
TAAGCGCCGGCTATGCCCCTGTATTGGATTG 10 (0.000256%)

AAAATGGGAAAGGAAAAGACTCATATCAACATTGTCGTCATTGGACACGTAGATTCGGGCAAGTCCAC
CACTACTGGCCATCTGATCTATAAATGCGGTG 30 (0.000769%)

AAAATTATAACAAACCCTGAGAACCAAAATGAACGAAAATCTGTTCGCTTCATTCATTGCCCCCACAAT
CCTAGGCCTACCCGCCGCAGTACTGATCATT 11 (0.000282%)

AAACAAATGATAACCATACACAACACTAAAGGACGAACCTGATCTCTTATACTAGTATCCTTAATCATT
TTTATTGCCACAACTAACCTCCTCGGACTCC 10 (0.000256%)

AAACAAATGGTCATTGATGTCCTTCACCCCGGGAAGGCGACAGTGCCTAAGACAGAAATTCGGGAAAA
ACTAGCCAAAATGTACAAGACCACACCGGATG 12 (0.000308%)

AAACAAGAAGAGGGTCCTGCTGGGAGAAACTGGCAAGGAGAAGCTCCCGCGGTACTACAAGAACATC
GGTCTGGGCTTCAAGACACCCAAGGAGGCTATT 3 (0.000077%)

AAACCCAGCCCATGACCCCTAACAGGGGCCCTCTCAGCCCTCCTAATGACCTCCGGCCTAGCCATGTGA
TTTCACTTCCACTCCATAACGCTCCTCATAC 5 (0.000128%)

AAACCCTGAGAACCAAAATGAACGAAAATCTGTTCGCTTCATTCATTGCCCCCACAATCCTAGGCCTAC
CCGCCGCAGTACTGATCATTCTATTTCCCCC 7 (0.000180%)

AAACCGCTAGCTTGTTGCACCGTGGAGGCCACAGGAGCAGAAACATGGAATGCCAGACGCTGGGGATG
CTGGTACAAGTTGTGGGACTGCATGCTACTGT 6 (0.000154%)

AAACTACCCCTAAAAGCCAAAATGGGAAAGGAAAAGACTCATATCAACATTGTCGTCATTGGACACGT
AGATTCGGGCAAGTCCACCACTACTGGCCATC 34 (0.000872%)

AAACTCTACTCCCACTAATAGCTTTTTGATGACTTCTAGCAAGCCTCGCTAACCTCGCCTTACCCCCCAC
TATTAACCTACTGGGAGAACTCTCTGTGCT 5 (0.000128%)

AAAGAAGAAAGATGAGGCAGAGGTCCAAGTAAACCGCTAGCTTGTTGCACCGTGGAGGCCACAGGAGC
AGAAACATGGAATGCCAGACGCTGGGGATGCT 4 (0.000103%)

AAAGAAGGGTGGCGAGAAGAAAAAGGGCCGTTCTGCCATCAACGAAGTGGTAACCCGAGAATACACC
ATCAACATTCACAAGCGCATCCATGGAGTGGGC 5 (0.000128%)

AAAGACTCATATCAACATTGTCGTCATTGGACACGTAGATTCGGGCAAGTCCACCACTACTGGCCATCT
GATCTATAAATGCGGTGGCATCGACAAAAGA 29 (0.000744%)

AAAGCAGCCGACCATCTTTCAAAACAAGAAGAGGGTCCTGCTGGGAGAAACTGGCAAGGAGAAGCTCC
CGCGGTACTACAAGAACATCGGTCTGGGCTTC 5 (0.000128%)

AAAGCCAAAATGGGAAAGGAAAAGACTCATATCAACATTGTCGTCATTGGACACGTAGATTCGGGCAA
GTCCACCACTACTGGCCATCTGATCTATAAAT 9 (0.000231%)

AAAGCCCATAAAAATAAAAAATTATAACAAACCCTGAGAACCAAAATGAACGAAAATCTGTTCGCTTC
ATTCATTGCCCCCACAATCCTAGGCCTACCCG 17 (0.000436%)

AAAGCTCAGAAGGCTAAATGAATATTATCCCTAATACCTGCCACCCCACTCTTAATCAGTGGTGGAAGA
ACGGTCTCAGAACTGTTTGTTTCAATTGGCC 14 (0.000359%)

AAAGCTGGAAGATGGCCCTAAATTCTTGAAGTCTGGTGATGCTGCCATTGTTGATATGGTTCCTGGCAA
GCCCATGTGTGTTGAGAGCTTCTCAGACTAT 16 (0.000410%)

AAAGGAAAAGACTCATATCAACATTGTCGTCATTGGACACGTAGATTCGGGCAAGTCCACCACTACTGGCCATCTGATCTATAAATGCGGTGGCATCGAC 16 (0.000410%)

AAAGGACGAACCTGATCTCTTATACTAGTATCCTTAATCATTTTTATTGCCACAACTAACCTCCTCGGACTCCTGCCTCACTCATTTACACCAACCACCC 12 (0.000308%)

AAAGGATCTCCTTCATCCCTCTCCAGAAGAGGAGAAGAGGAAACACAAGAAGAAACGCCTGGTGCAGAGCCCCAATTCCTACTTCATGGATGTGAAATGC 5 (0.000128%)

AAAGGGCCGTTCTGCCATCAACGAAGTGGTAACCCGAGAATACACCATCAACATTCACAAGCGCATCCATGGAGTGGGCTTCAAGAAGCGTGCACCTCGG 5 (0.000128%)

AAAGTTCTCCGCTCCCAGACATGGGTCCCTCGGCTTCCTGCCTCGGAAGCGCAGCAGCAGGCATCGTGGGAAGGTGAAGAGCTTCCCTAAGGATGACCCG 5 (0.000128%)

AAATAAAAAATTATAACAAACCCTGAGAACCAAAATGAACGAAAATCTGTTCGCTTCATTCATTGCCCCCACAATCCTAGGCCTACCCGCCGCAGTACTG 3 (0.000077%)

AAATCTGTCGAAATGCACCATGAAGCTTTGAGTGAAGCTCTTCCTGGGGACAATGTGGGCTTCAATGTCAAGAATGTGTCTGTCAAGGATGTTCGTCGTG 4 (0.000103%)

AAATGAATATTATCCCTAATACCTGCCACCCCACTCTTAATCAGTGGTGGAAGAACGGTCTCAGAACTGTTTGTTTCAATTGGCCATTTAAGTTTAGTAG 5 (0.000128%)

AAATGACCCACCAATGGAAGCAGCTGGCTTCACTGCTCAGGTGATTATCCTGAACCATCCAGGCCAAATAAGCGCCGGCTATGCCCCTGTATTGGATTGC 28 (0.000718%)

AAATGATAACCATACACAACACTAAAGGACGAACCTGATCTCTTATACTAGTATCCTTAATCATTTTTATTGCCACAACTAACCTCCTCGGACTCCTGCC 10 (0.000256%)

AAATGGGAAAGGAAAAGACTCATATCAACATTGTCGTCATTGGACACGTAGATTCGGGCAAGTCCACCACTACTGGCCATCTGATCTATAAATGCGGTGG 6 (0.000154%)

AAATTATAACAAACCCTGAGAACCAAAATGAACGAAAATCTGTTCGCTTCATTCATTGCCCCCACAATCCTAGGCCTACCCGCCGCAGTACTGATCATTC 13 (0.000333%)

AAATTCTTGAAGTCTGGTGATGCTGCCATTGTTGATATGGTTCCTGGCAAGCCCATGTGTGTTGAGAGCTTCTCAGACTATCCACCTTTGGGTCGCTTTG84 (0.002154%)

AACAAAACTAACTAATACTAACATCTCAGACGCTCAGGAAATAGAAACCGTCTGAACTATCCTGCCCGCCATCATCCTAGTCCTCATCGCCCTCCCATCC 37 (0.000949%)

AACAAACCCTGAGAACCAAAATGAACGAAAATCTGTTCGCTTCATTCATTGCCCCCACAATCCTAGGCCTACCCGCCGCAGTACTGATCATTCTATTTCC 12 (0.000308%)

AACAAATGATAACCATACACAACACTAAAGGACGAACCTGATCTCTTATACTAGTATCCTTAATCATTTTTATTGCCACAACTAACCTCCTCGGACTCCT 8 (0.000205%)

AACACAGGTGTCGTGAAAACTACCCCTAAAAGCCAAAATGGGAAAGGAAAAGACTCATATCAACATTGTCGTCATTGGACACGTAGATTCGGGCAAGTCC 41 (0.001052%)

AACACTCACAACAAAACTAACTAATACTAACATCTCAGACGCTCAGGAAATAGAAACCGTCTGAACTATCCTGCCCGCCATCATCCTAGTCCTCATCGCC 7 (0.000180%)

AACAGGGGCCCTCTCAGCCCTCCTAATGACCTCCGGCCTAGCCATGTGATTTCACTTCCACTCCATAACGCTCCTCATACTAGGCCTACTAACCAACACA 11 (0.000282%)

AACATCTCCGCATGATGAAACTTCGGCTCACTCCTTGGCGCCTGCCTGATCCTCCAAATCACCACAGGA
CTATTCCTAGCCATGCACTACTCACCAGACG 11 (0.000282%)

AACATGCCTCTCGCAAAGGATCTCCTTCATCCCTCTCCAGAAGAGGAGAAGAGGAAACACAAGAAGAA
ACGCCTGGTGCAGAGCCCCAATTCCTACTTCA 4 (0.000103%)

AACCCAGCCCATGACCCCTAACAGGGGCCCTCTCAGCCCTCCTAATGACCTCCGGCCTAGCCATGTGAT
TTCACTTCCACTCCATAACGCTCCTCATACT 8 (0.000205%)

AACCCTGAGAACCAAAATGAACGAAAATCTGTTCGCTTCATTCATTGCCCCCACAATCCTAGGCCTACC
CGCCGCAGTACTGATCATTCTATTTCCCCCT 5 (0.000128%)

AACCGCTAGCTTGTTGCACCGTGGAGGCCACAGGAGCAGAAACATGGAATGCCAGACGCTGGGGATGC
TGGTACAAGTTGTGGGACTGCATGCTACTGTC 4 (0.000103%)

AACGCAGGCACATACTTCCTATTCTACACCCTAGTAGGCTCCCTTCCCCTACTCATCGCACTGATTTACA
CTCACAACACCCTAGGCTCACTAAACATTC 3 (0.000077%)

AACGGAAGTAAAATCTGTCGAAATGCACCATGAAGCTTTGAGTGAAGCTCTTCCTGGGGACAATGTGG
GCTTCAATGTCAAGAATGTGTCTGTCAAGGAT 15 (0.000385%)

AACGTTACAACGGAAGTAAAATCTGTCGAAATGCACCATGAAGCTTTGAGTGAAGCTCTTCCTGGGGAC
AATGTGGGCTTCAATGTCAAGAATGTGTCTG 5 (0.000128%)

AACGTTATCGTCACAGCCCATGCATTTGTAATAATCTTCTTCATAGTAATACCCATCATAATCGGAGGCT
TTGGCAACTGACTAGTTCCCCTAATAATCG 4 (0.000103%)

AACGTTGCTGGTGACAGCAAAAATGACCCACCAATGGAAGCAGCTGGCTTCACTGCTCAGGTGATTATC
CTGAACCATCCAGGCCAAATAAGCGCCGGCT 4 (0.000103%)

AACTACCCCTAAAAGCCAAAATGGGAAAGGAAAAGACTCATATCAACATTGTCGTCATTGGACACGTA
GATTCGGGCAAGTCCACCACTACTGGCCATCT 14 (0.000359%)

AACTTCCTTCGGTCGTCCCGAATCCGGGTTCATCCGACACCAGCCGCCTCCACCATGCCGCCGAAGTTC
GACCCCAACGAGATCAAAGTCGTATACCTGA 6 (0.000154%)

AAGAAAGATGAGGCAGAGGTCCAAGTAAACCGCTAGCTTGTTGCACCGTGGAGGCCACAGGAGCAGAA
ACATGGAATGCCAGACGCTGGGGATGCTGGTA 6 (0.000154%)

AAGAAGAAAGATGAGGCAGAGGTCCAAGTAAACCGCTAGCTTGTTGCACCGTGGAGGCCACAGGAGCA
GAAACATGGAATGCCAGACGCTGGGGATGCTG 3 (0.000077%)

AAGAAGGTGATGGTGAGGAAGAGGATGGAGATGAAGATGAGGAAGCTGAGTCAGCTACGGGCAAGCG
GGCAGCTGAAGATGATGAGGATGACGATGTCGA 433 (0.011105%)

AAGAATGTGTCTGTCAAGGATGTTCGTCGTGGCAACGTTGCTGGTGACAGCAAAAATGACCCACCAATG
GAAGCAGCTGGCTTCACTGCTCAGGTGATTA 18 (0.000462%)

AAGACTCATATCAACATTGTCGTCATTGGACACGTAGATTCGGGCAAGTCCACCACTACTGGCCATCTG
ATCTATAAATGCGGTGGCATCGACAAAAGAA 17 (0.000436%)

AAGAGGATGGAGATGAAGATGAGGAAGCTGAGTCAGCTACGGGCAAGCGGGCAGCTGAAGATGATGA
GGATGACGATGTCGATACCAAGAAGCAGAAGAC 4 (0.000103%)

AAGATATGCTCATGTGGTGTTGAGGAAAGCAGACATTGACCTCACCAAGAGGGCGGGAGAACTCACTG
AGGATGAGGTGGAACGTGTGATCACCATTATG6 (0.000154%)

AAGATGAGGAAGCTGAGTCAGCTACGGGCAAGCGGGCAGCTGAAGATGATGAGGATGACGATGTCGAT

ACCAAGAAGCAGAAGACCGACGAGGATGACTA 36 (0.000923%)

AAGATGAGGCAGAGGTCCAAGTAAACCGCTAGCTTGTTGCACCGTGGAGGCCACAGGAGCAGAAACAT
GGAATGCCAGACGCTGGGGATGCTGGTACAAG 5 (0.000128%)

AAGATGGCCCTAAATTCTTGAAGTCTGGTGATGCTGCCATTGTTGATATGGTTCCTGGCAAGCCCATGTG
TGTTGAGAGCTTCTCAGACTATCCACCTTT8 (0.000205%)

AAGCAGCCGACCATCTTTCAAAACAAGAAGAGGGTCCTGCTGGGAGAAACTGGCAAGGAGAAGCTCCC
GCGGTACTACAAGAACATCGGTCTGGGCTTCA 17 (0.000436%)

AAGCAGCTGGCTTCACTGCTCAGGTGATTATCCTGAACCATCCAGGCCAAATAAGCGCCGGCTATGCCC
CTGTATTGGATTGCCACACGGCTCACATTGC 14 (0.000359%)

AAGCCCATAAAAATAAAAAATTATAACAAACCCTGAGAACCAAAATGAACGAAAATCTGTTCGCTTCA
TTCATTGCCCCCACAATCCTAGGCCTACCCGC 7 (0.000180%)

AAGCTCAGAAGGCTAAATGAATATTATCCCTAATACCTGCCACCCCACTCTTAATCAGTGGTGGAAGAA
CGGTCTCAGAACTGTTTGTTTCAATTGGCCA 7 (0.000180%)

AAGCTGAGTCAGCTACGGGCAAGCGGGCAGCTGAAGATGATGAGGATGACGATGTCGATACCAAGAAG
CAGAAGACCGACGAGGATGACTAGACAGCAAA 34 (0.000872%)

AAGGAAAAGACTCATATCAACATTGTCGTCATTGGACACGTAGATTCGGGCAAGTCCACCACTACTGGC
CATCTGATCTATAAATGCGGTGGCATCGACA 9 (0.000231%)

AAGGACGAACCTGATCTCTTATACTAGTATCCTTAATCATTTTTATTGCCACAACTAACCTCCTCGGACT
CCTGCCTCACTCATTTACACCAACCACCCA 3 (0.000077%)

AAGGACTTCAAACTCTACTCCCACTAATAGCTTTTTGATGACTTCTAGCAAGCCTCGCTAACCTCGCCTT
ACCCCCCACTATTAACCTACTGGGAGAACT 9 (0.000231%)

AAGGAGCTAGGAGTGGGAATAGCTTTGCGAAAAATGGGCGCAATGGCCAAGCCAGATTGTATCATCAC
TTGTGATGGTAAAAACCTCACCATAAAAACTG 97 (0.002488%)

AAGGATGTTCGTCGTGGCAACGTTGCTGGTGACAGCAAAAATGACCCACCAATGGAAGCAGCTGGCTT
CACTGCTCAGGTGATTATCCTGAACCATCCAG 6 (0.000154%)

AAGGCCTCGGGCACGCTACGAGAGTACAAGGTAGTGGGTCGCTGCCTGCCCACCCCCAAATGCCACAC
GCCGCCCCTCTACCGCATGCGAATCTTTGCGC 5 (0.000128%)

AAGGCTGCTGGAGCTGGCAAGGTCACCAAGTCTGCCCAGAAAGCTCAGAAGGCTAAATGAATATTATC
CCTAATACCTGCCACCCCACTCTTAATCAGTG 1182 (0.030315%)

AAGGGCCGTTCTGCCATCAACGAAGTGGTAACCCGAGAATACACCATCAACATTCACAAGCGCATCCAT
GGAGTGGGCTTCAAGAAGCGTGCACCTCGGG 3 (0.000077%)

AAGGTCACCAAGTCTGCCCAGAAAGCTCAGAAGGCTAAATGAATATTATCCCTAATACCTGCCACCCCA
CTCTTAATCAGTGGTGGAAGAACGGTCTCAG 8 (0.000205%)

AAGGTGATGGTGAGGAAGAGGATGGAGATGAAGATGAGGAAGCTGAGTCAGCTACGGGCAAGCGGGC
AGCTGAAGATGATGAGGATGACGATGTCGATAC 10 (0.000256%)

AAGTAAAATCTGTCGAAATGCACCATGAAGCTTTGAGTGAAGCTCTTCCTGGGGACAATGTGGGCTTCA
ATGTCAAGAATGTGTCTGTCAAGGATGTTCG 4 (0.000103%)

AAGTAAACCGCTAGCTTGTTGCACCGTGGAGGCCACAGGAGCAGAAACATGGAATGCCAGACGCTGGG
GATGCTGGTACAAGTTGTGGGACTGCATGCTA 16 (0.000410%)

AAGTTCTCCGCTCCCAGACATGGGTCCCTCGGCTTCCTGCCTCGGAAGCGCAGCAGCAGGCATCGTGGG
AAGGTGAAGAGCTTCCCTAAGGATGACCCGT 3 (0.000077%)

AAGTTGGGCCGCTTGGTCAAGGACATGAAGATCAAGTCCCTGGAGGAGATCTATCTCTTCTCCCTGCCC
ATTAAGGAATCAGAGATCATTGATTTCTTCC 3 (0.000077%)

AATAATCTTCTTCATAGTAATACCCATCATAATCGGAGGCTTTGGCAACTGACTAGTTCCCCTAATAATC
GGTGCCCCCGATATGGCGTTTCCCCGCATA 3 (0.000077%)

AATACCTGCCACCCCACTCTTAATCAGTGGTGGAAGAACGGTCTCAGAACTGTTTGTTTCAATTGGCCAT
TTAAGTTTAGTAGTAAAAGACTGGTTAATG 7 (0.000180%)

AATCACAGCAGTCCTACTTCTCCTATCTCTCCCAGTCCTAGCTGCTGGCATCACTATACTACTAACAGAC
CGCAACCTCAACACCACCTTCTTCGACCCC 4 (0.000103%)

AATCCCTTTGTGACTTCCGACCGAAGCAAGAATCGCAAAAGGCATTTCAATGCACCTTCCCACATTCGA
AGGAAGATTATGTCTTCCCCTCTTTCCAAAG 7 (0.000180%)

AATCTGTCGAAATGCACCATGAAGCTTTGAGTGAAGCTCTTCCTGGGGACAATGTGGGCTTCAATGTCA
AGAATGTGTCTGTCAAGGATGTTCGTCGTGG 24 (0.000616%)

AATGAATATTATCCCTAATACCTGCCACCCCACTCTTAATCAGTGGTGGAAGAACGGTCTCAGAACTGT
TTGTTTCAATTGGCCATTTAAGTTTAGTAGT 10 (0.000256%)

AATGACCCACCAATGGAAGCAGCTGGCTTCACTGCTCAGGTGATTATCCTGAACCATCCAGGCCAAATA
AGCGCCGGCTATGCCCCTGTATTGGATTGCC 5 (0.000128%)

AATGCGCAGGCTGAAGCGCAAAAGAAGAAAGATGAGGCAGAGGTCCAAGTAAACCGCTAGCTTGTTGC
ACCGTGGAGGCCACAGGAGCAGAAACATGGAA 6 (0.000154%)

AATGGAAGCAGCTGGCTTCACTGCTCAGGTGATTATCCTGAACCATCCAGGCCAAATAAGCGCCGGCTA
TGCCCCTGTATTGGATTGCCACACGGCTCAC 26 (0.000667%)

AATGGGAAAGGAAAAGACTCATATCAACATTGTCGTCATTGGACACGTAGATTCGGGCAAGTCCACCA
CTACTGGCCATCTGATCTATAAATGCGGTGGC 6 (0.000154%)

AATGTCAAGAATGTGTCTGTCAAGGATGTTCGTCGTGGCAACGTTGCTGGTGACAGCAAAAATGACCCA
CCAATGGAAGCAGCTGGCTTCACTGCTCAGG 3 (0.000077%)

AATGTGTCTGTCAAGGATGTTCGTCGTGGCAACGTTGCTGGTGACAGCAAAAATGACCCACCAATGGAA
GCAGCTGGCTTCACTGCTCAGGTGATTATCC 9 (0.000231%)

AATTATAACAAACCCTGAGAACCAAAATGAACGAAAATCTGTTCGCTTCATTCATTGCCCCCACAATCC
TAGGCCTACCCGCCGCAGTACTGATCATTCT 5 (0.000128%)

AATTCTTGAAGTCTGGTGATGCTGCCATTGTTGATATGGTTCCTGGCAAGCCCATGTGTGTTGAGAGCTT
CTCAGACTATCCACCTTTGGGTCGCTTTGC 34 (0.000872%)

ACAAATGATAACCATACACAACACTAAAGGACGAACCTGATCTCTTATACTAGTATCCTTAATCATTTTT
ATTGCCACAACTAACCTCCTCGGACTCCTG 4 (0.000103%)

ACAACACTAAAGGACGAACCTGATCTCTTATACTAGTATCCTTAATCATTTTTATTGCCACAACTAACCT
CCTCGGACTCCTGCCTCACTCATTTACACC 17 (0.000436%)

ACAACGGAAGTAAAATCTGTCGAAATGCACCATGAAGCTTTGAGTGAAGCTCTTCCTGGGGACAATGTG
GGCTTCAATGTCAAGAATGTGTCTGTCAAGG 3 (0.000077%)

ACAACGTTATCGTCACAGCCCATGCATTTGTAATAATCTTCTTCATAGTAATACCCATCATAATCGGAGG
CTTTGGCAACTGACTAGTTCCCCTAATAAT 3 (0.000077%)

ACACAACACTAAAGGACGAACCTGATCTCTTATACTAGTATCCTTAATCATTTTTATTGCCACAACTAAC
CTCCTCGGACTCCTGCCTCACTCATTTACA 5 (0.000128%)

ACACAGGTGTCGTGAAAACTACCCCTAAAAGCCAAAATGGGAAAGGAAAAGACTCATATCAACATTGT
CGTCATTGGACACGTAGATTCGGGCAAGTCCA 17 (0.000436%)

ACACCTCATATCCTCCCTACTATGCCTAGAAGGAATAATACTATCGCTGTTCATTATAGCTACTCTCATA
ACCCTCAACACCCACTCCCTCTTAGCCAAT 466 (0.011952%)

ACACGAGAACATGCCTCTCGCAAAGGATCTCCTTCATCCCTCTCCAGAAGAGGAGAAGAGGAAACACA
AGAAGAAACGCCTGGTGCAGAGCCCCAATTCC 4 (0.000103%)

ACAGAAAGTTCTCCGCTCCCAGACATGGGTCCCTCGGCTTCCTGCCTCGGAAGCGCAGCAGCAGGCATC
GTGGGAAGGTGAAGAGCTTCCCTAAGGATGA 3 (0.000077%)

ACAGGGGCCCTCTCAGCCCTCCTAATGACCTCCGGCCTAGCCATGTGATTTCACTTCCACTCCATAACGC
TCCTCATACTAGGCCTACTAACCAACACAC 3 (0.000077%)

ACAGGTGTCGTGAAAACTACCCCTAAAAGCCAAAATGGGAAAGGAAAAGACTCATATCAACATTGTCG
TCATTGGACACGTAGATTCGGGCAAGTCCACC 17 (0.000436%)

ACATAGGTATGGTCTGAGCTATGATATCAATTGGCTTCCTAGGGTTTATCGTGTGAGCACACCATATATT
TACAGTAGGAATAGACGTAGACACACGAGC 3 (0.000077%)

ACATCTCCGCATGATGAAACTTCGGCTCACTCCTTGGCGCCTGCCTGATCCTCCAAATCACCACAGGACT
ATTCCTAGCCATGCACTACTCACCAGACGC 4 (0.000103%)

ACATGCCTCTCGCAAAGGATCTCCTTCATCCCTCTCCAGAAGAGGAGAAGAGGAAACACAAGAAGAAA
CGCCTGGTGCAGAGCCCCAATTCCTACTTCAT 10 (0.000256%)

ACCAAAAGCAGCCGACCATCTTTCAAAACAAGAAGAGGGTCCTGCTGGGAGAAACTGGCAAGGAGAA
GCTCCCGCGGTACTACAAGAACATCGGTCTGGG 5 (0.000128%)

ACCAAAGCCCATAAAAATAAAAAATTATAACAAACCCTGAGAACCAAAATGAACGAAAATCTGTTCGC
TTCATTCATTGCCCCCACAATCCTAGGCCTAC 10 (0.000256%)

ACCAAGTCTGCCCAGAAAGCTCAGAAGGCTAAATGAATATTATCCCTAATACCTGCCACCCCACTCTTA
ATCAGTGGTGGAAGAACGGTCTCAGAACTGT 32 (0.000821%)

ACCAAGTTGGGCCGCTTGGTCAAGGACATGAAGATCAAGTCCCTGGAGGAGATCTATCTCTTCTCCCTG
CCCATTAAGGAATCAGAGATCATTGATTTCT 7 (0.000180%)

ACCAATGGAAGCAGCTGGCTTCACTGCTCAGGTGATTATCCTGAACCATCCAGGCCAAATAAGCGCCGG
CTATGCCCCTGTATTGGATTGCCACACGGCT 14 (0.000359%)

ACCACATCTACAACGTTATCGTCACAGCCCATGCATTTGTAATAATCTTCTTCATAGTAATACCCATCAT
AATCGGAGGCTTTGGCAACTGACTAGTTCC 16 (0.000410%)

ACCATACACAACACTAAAGGACGAACCTGATCTCTTATACTAGTATCCTTAATCATTTTTATTGCCACAA
CTAACCTCCTCGGACTCCTGCCTCACTCAT 16 (0.000410%)

ACCATTTGGATACATAGGTATGGTCTGAGCTATGATATCAATTGGCTTCCTAGGGTTTATCGTGTGAGCA
CACCATATATTTACAGTAGGAATAGACGTA 546 (0.014004%)

ACCCACCAATGGAAGCAGCTGGCTTCACTGCTCAGGTGATTATCCTGAACCATCCAGGCCAAATAAGCG

CCGGCTATGCCCCTGTATTGGATTGCCACAC 6 (0.000154%)

ACCCAGCCCATGACCCCTAACAGGGGCCCTCTCAGCCCTCCTAATGACCTCCGGCCTAGCCATGTGATT
TCACTTCCACTCCATAACGCTCCTCATACTA 11 (0.000282%)

ACCCCTAAAAGCCAAAATGGGAAAGGAAAAGACTCATATCAACATTGTCGTCATTGGACACGTAGATT
CGGGCAAGTCCACCACTACTGGCCATCTGATC 3 (0.000077%)

ACCCGCTATGGGGCCTCCCTCCGGAAAATGGTGAAGAAAATTGAAATCAGCCAGCACGCCAAGTACAC
TTGCTCTTTCTGTGGCAAAACCAAGATGAAGA 4 (0.000103%)

ACCGAAGCAAGAATCGCAAAAGGCATTTCAATGCACCTTCCCACATTCGAAGGAAGATTATGTCTTCCC
CTCTTTCCAAAGAGCTGAGACAGAAGTACAA 4 (0.000103%)

ACCGCTAGCTTGTTGCACCGTGGAGGCCACAGGAGCAGAAACATGGAATGCCAGACGCTGGGGATGCT
GGTACAAGTTGTGGGACTGCATGCTACTGTCT 3 (0.000077%)

ACCTATTCACCCTCCACTTCCCGTCTCAGAATCTAAACGTGGTCACCTTCGAGTAGAGAGGCCCGCCCG
CCCACCGTGGGCAGTGCCACCCGCAGATGAC 6 (0.000154%)

ACCTCCCTCACCAAAGCCCATAAAAATAAAAAATTATAACAAACCCTGAGAACCAAAATGAACGAAAA
TCTGTTCGCTTCATTCATTGCCCCCACAATCC 618 (0.015850%)

ACGAGAACATGCCTCTCGCAAAGGATCTCCTTCATCCCTCTCCAGAAGAGGAGAAGAGGAAACACAAG
AAGAAACGCCTGGTGCAGAGCCCCAATTCCTA 16 (0.000410%)

ACGCAAGCATGGTTAACGTCCCTAAAACCCGCCGGACTTTCTGTAAGAAGTGTGGCAAGCACCAACCCC
ATAAAGTGACACAGTACAAGAAGGGCAAGGA 4 (0.000103%)

ACGCACACGAGAACATGCCTCTCGCAAAGGATCTCCTTCATCCCTCTCCAGAAGAGGAGAAGAGGAAA
CACAAGAAGAAACGCCTGGTGCAGAGCCCCAA 18 (0.000462%)

ACGCCTGAACGCAGGCACATACTTCCTATTCTACACCCTAGTAGGCTCCCTTCCCCTACTCATCGCACTG
ATTTACACTCACAACACCCTAGGCTCACTA 471 (0.012080%)

ACGGAAGTAAAATCTGTCGAAATGCACCATGAAGCTTTGAGTGAAGCTCTTCCTGGGGACAATGTGGGC
TTCAATGTCAAGAATGTGTCTGTCAAGGATG 22 (0.000564%)

ACGGGACCCGCTATGGGGCCTCCCTCCGGAAAATGGTGAAGAAAATTGAAATCAGCCAGCACGCCAAG
TACACTTGCTCTTTCTGTGGCAAAACCAAGAT 548 (0.014055%)

ACGGGATAATCCTATTTATTACCTCAGAAGTTTTTTCTTCGCAGGATTTTTCTGAGCCTTTTACCACTCC
AGCCTAGCCCCTACCCCCCAATTAGGAGG 6 (0.000154%)

ACTAAAGGACGAACCTGATCTCTTATACTAGTATCCTTAATCATTTTTATTGCCACAACTAACCTCCTCG
GACTCCTGCCTCACTCATTTACACCAACCA 6 (0.000154%)

ACTACCCCTAAAAGCCAAAATGGGAAAGGAAAAGACTCATATCAACATTGTCGTCATTGGACACGTAG
ATTCGGGCAAGTCCACCACTACTGGCCATCTG 25 (0.000641%)

ACTCACAACAAAACTAACTAATACTAACATCTCAGACGCTCAGGAAATAGAAACCGTCTGAACTATCCT
GCCCGCCATCATCCTAGTCCTCATCGCCCTC 4 (0.000103%)

ACTGAGCGTGCCTACCAAAAGCAGCCGACCATCTTTCAAAACAAGAAGAGGGTCCTGCTGGGAGAAAC
TGGCAAGGAGAAGCTCCCGCGGTACTACAAGA 9 (0.000231%)

ACTGCTAGGAAGCTCCGTAGTCACCGACGAGACCAGAAGTGGCATGATAAACAGTATAAGAAAGCTCA
TTTGGGCACAGCCCTAAAGGCCAACCCTTTTG 5 (0.000128%)

AGAAAAAGGGCCGTTCTGCCATCAACGAAGTGGTAACCCGAGAATACACCATCAACATTCACAAGCGC
ATCCATGGAGTGGGCTTCAAGAAGCGTGCACC 15 (0.000385%)

AGAAAGATGAGGCAGAGGTCCAAGTAAACCGCTAGCTTGTTGCACCGTGGAGGCCACAGGAGCAGAAA
CATGGAATGCCAGACGCTGGGGATGCTGGTAC 8 (0.000205%)

AGAAAGCTCAGAAGGCTAAATGAATATTATCCCTAATACCTGCCACCCCACTCTTAATCAGTGGTGGAA
GAACGGTCTCAGAACTGTTTGTTTCAATTGG 8 (0.000205%)

AGAAAGTTCTCCGCTCCCAGACATGGGTCCCTCGGCTTCCTGCCTCGGAAGCGCAGCAGCAGGCATCGT
GGGAAGGTGAAGAGCTTCCCTAAGGATGACC 21 (0.000539%)

AGAACACAGGTGTCGTGAAAACTACCCCTAAAAGCCAAAATGGGAAAGGAAAAGACTCATATCAACAT
TGTCGTCATTGGACACGTAGATTCGGGCAAGT 190 (0.004873%)

AGAACATGCCTCTCGCAAAGGATCTCCTTCATCCCTCTCCAGAAGAGGAGAAGAGGAAACACAAGAAG
AAACGCCTGGTGCAGAGCCCCAATTCCTACTT 19 (0.000487%)

AGAAGAAAAAGGGCCGTTCTGCCATCAACGAAGTGGTAACCCGAGAATACACCATCAACATTCACAAG
CGCATCCATGGAGTGGGCTTCAAGAAGCGTGC 3 (0.000077%)

AGAAGAAAGATGAGGCAGAGGTCCAAGTAAACCGCTAGCTTGTTGCACCGTGGAGGCCACAGGAGCAG
AAACATGGAATGCCAGACGCTGGGGATGCTGG 6 (0.000154%)

AGAAGGCTAAATGAATATTATCCCTAATACCTGCCACCCCACTCTTAATCAGTGGTGGAAGAACGGTCT
CAGAACTGTTTGTTTCAATTGGCCATTTAAG 9 (0.000231%)

AGAAGGGTGGCGAGAAGAAAAAGGGCCGTTCTGCCATCAACGAAGTGGTAACCCGAGAATACACCATC
AACATTCACAAGCGCATCCATGGAGTGGGCTT 4 (0.000103%)

AGAAGGTGATGGTGAGGAAGAGGATGGAGATGAAGATGAGGAAGCTGAGTCAGCTACGGGCAAGCGG
GCAGCTGAAGATGATGAGGATGACGATGTCGAT 37 (0.000949%)

AGAATGTGTCTGTCAAGGATGTTCGTCGTGGCAACGTTGCTGGTGACAGCAAAAATGACCCACCAATGG
AAGCAGCTGGCTTCACTGCTCAGGTGATTAT 9 (0.000231%)

AGACCCGAGAGCATGCCCTTCTGGCTTACACACTGGGTGTGAAACAACTAATTGTCGGTGTTAACAAAA
TGGATTCCACTGAGCCACCCTACAGCCAGAA 7 (0.000180%)

AGACTGAGCGTGCCTACCAAAAGCAGCCGACCATCTTTCAAAACAAGAAGAGGGTCCTGCTGGGAGAA
ACTGGCAAGGAGAAGCTCCCGCGGTACTACAA 4 (0.000103%)

AGAGCACGCCATGAAGGCCTCGGGCACGCTACGAGAGTACAAGGTAGTGGGTCGCTGCCTGCCCACCC
CCAAATGCCACACGCCGCCCCTCTACCGCATG 4 (0.000103%)

AGAGCATGCCCTTCTGGCTTACACACTGGGTGTGAAACAACTAATTGTCGGTGTTAACAAAATGGATTC
CACTGAGCCACCCTACAGCCAGAAGAGATAT 29 (0.000744%)

AGAGGATGGAGATGAAGATGAGGAAGCTGAGTCAGCTACGGGCAAGCGGGCAGCTGAAGATGATGAG
GATGACGATGTCGATACCAAGAAGCAGAAGACC 15 (0.000385%)

AGAGGTCCAAGTAAACCGCTAGCTTGTTGCACCGTGGAGGCCACAGGAGCAGAAACATGGAATGCCAG
ACGCTGGGGATGCTGGTACAAGTTGTGGGACT 4 (0.000103%)

AGAGTACAAGGTAGTGGGTCGCTGCCTGCCCACCCCCAAATGCCACACGCCGCCCCTCTACCGCATGCG
AATCTTTGCGCCTAATCATGTCGTCGCCAAG 5 (0.000128%)

AGATATGCTCATGTGGTGTTGAGGAAAGCAGACATTGACCTCACCAAGAGGGCGGGAGAACTCACTGAGGATGAGGTGGAACGTGTGATCACCATTATGC3 (0.000077%)

AGATGAAGATGAGGAAGCTGAGTCAGCTACGGGCAAGCGGGCAGCTGAAGATGATGAGGATGACGATGTCGATACCAAGAAGCAGAAGACCGACGAGGAT 3 (0.000077%)

AGATGAGGAAGCTGAGTCAGCTACGGGCAAGCGGGCAGCTGAAGATGATGAGGATGACGATGTCGATACCAAGAAGCAGAAGACCGACGAGGATGACTAG 23 (0.000590%)

AGATGAGGCAGAGGTCCAAGTAAACCGCTAGCTTGTTGCACCGTGGAGGCCACAGGAGCAGAAACATGGAATGCCAGACGCTGGGGATGCTGGTACAAGT 14 (0.000359%)

AGATGGCCCTAAATTCTTGAAGTCTGGTGATGCTGCCATTGTTGATATGGTTCCTGGCAAGCCCATGTGTGTTGAGAGCTTCTCAGACTATCCACCTTTG 10 (0.000256%)

AGATGGGAAAGGGCTCCTTCAAGTATGCCTGGGTCTTGGATAAACTGAAAGCTGAGCGTGAACGTGGTATCACCATTGATATCTCCTTGTGGAAATTTGA 18 (0.000462%)

AGCACGCCATGAAGGCCTCGGGCACGCTACGAGAGTACAAGGTAGTGGGTCGCTGCCTGCCCACCCCCAAATGCCACACGCCGCCCCTCTACCGCATGCG 6 (0.000154%)

AGCAGCCGACCATCTTTCAAAACAAGAAGAGGGTCCTGCTGGGAGAAACTGGCAAGGAGAAGCTCCCGCGGTACTACAAGAACATCGGTCTGGGCTTCAA 7 (0.000180%)

AGCAGCTGGCTTCACTGCTCAGGTGATTATCCTGAACCATCCAGGCCAAATAAGCGCCGGCTATGCCCCTGTATTGGATTGCCACACGGCTCACATTGCA 13 (0.000333%)

AGCATGCCCTTCTGGCTTACACACTGGGTGTGAAACAACTAATTGTCGGTGTTAACAAAATGGATTCCACTGAGCCACCCTACAGCCAGAAGAGATATGA 10 (0.000256%)

AGCCAAAATGGGAAAGGAAAAGACTCATATCAACATTGTCGTCATTGGACACGTAGATTCGGGCAAGTCCACCACTACTGGCCATCTGATCTATAAATGC 4 (0.000103%)

AGCCCATAAAAATAAAAAATTATAACAAACCCTGAGAACCAAAATGAACGAAAATCTGTTCGCTTCATTCATTGCCCCCACAATCCTAGGCCTACCCGCC 10 (0.000256%)

AGCCCATGACCCCTAACAGGGGCCCTCTCAGCCCTCCTAATGACCTCCGGCCTAGCCATGTGATTTCACTTCCACTCCATAACGCTCCTCATACTAGGCC 12 (0.000308%)

AGCCCATGCATTTGTAATAATCTTCTTCATAGTAATACCCATCATAATCGGAGGCTTTGGCAACTGACTAGTTCCCCTAATAATCGGTGCCCCCGATATG 118 (0.003026%)

AGCCCTCCTAATGACCTCCGGCCTAGCCATGTGATTTCACTTCCACTCCATAACGCTCCTCATACTAGGCCTACTAACCAACACACTAACCATATACCAA 4 (0.000103%)

AGCCCTTGCGCCTGCCTCTCCAGGATGTCTACAAAATTGGTGGTATTGGTACTGTTCCTGTTGGCCGAGTGGAGACTGGTGTTCTCAAACCCGGTATGGT 520 (0.013337%)

AGCCGACCATCTTTCAAAACAAGAAGAGGGTCCTGCTGGGAGAAACTGGCAAGGAGAAGCTCCCGCGGTACTACAAGAACATCGGTCTGGGCTTCAAGAC 8 (0.000205%)

AGCGTGCCTACCAAAAGCAGCCGACCATCTTTCAAAACAAGAAGAGGGTCCTGCTGGGAGAAACTGGCAAGGAGAAGCTCCCGCGGTACTACAAGAACAT 13 (0.000333%)

AGCTAGGAGTGGGAATAGCTTTGCGAAAAATGGGCGCAATGGCCAAGCCAGATTGTATCATCACTTGTGATGGTAAAAACCTCACCATAAAAACTGAGAG 3 (0.000077%)

AGCTCAGAAGGCTAAATGAATATTATCCCTAATACCTGCCACCCCACTCTTAATCAGTGGTGGAAGAAC

GGTCTCAGAACTGTTTGTTTCAATTGGCCAT 10 (0.000256%)

AGCTCCGTAGTCACCGACGAGACCAGAAGTGGCATGATAAACAGTATAAGAAAGCTCATTTGGGCACA
GCCCTAAAGGCCAACCCTTTTGGAGGTGCTTC 3 (0.000077%)

AGCTGAGTCAGCTACGGGCAAGCGGGCAGCTGAAGATGATGAGGATGACGATGTCGATACCAAGAAGC
AGAAGACCGACGAGGATGACTAGACAGCAAAA 44 (0.001128%)

AGCTGGAAGATGGCCCTAAATTCTTGAAGTCTGGTGATGCTGCCATTGTTGATATGGTTCCTGGCAAGC
CCATGTGTGTTGAGAGCTTCTCAGACTATCC 11 (0.000282%)

AGCTGGCAAGGTCACCAAGTCTGCCCAGAAAGCTCAGAAGGCTAAATGAATATTATCCCTAATACCTGC
CACCCCACTCTTAATCAGTGGTGGAAGAACG 10 (0.000256%)

AGCTGGCTTCACTGCTCAGGTGATTATCCTGAACCATCCAGGCCAAATAAGCGCCGGCTATGCCCCTGT
ATTGGATTGCCACACGGCTCACATTGCATGC 12 (0.000308%)

AGCTTTTTGATGACTTCTAGCAAGCCTCGCTAACCTCGCCTTACCCCCCACTATTAACCTACTGGGAGAA
CTCTCTGTGCTAGTAACCACGTTCTCCTGA 3 (0.000077%)

AGGAAACAAATGGTCATTGATGTCCTTCACCCCGGGAAGGCGACAGTGCCTAAGACAGAAATTCGGGA
AAAACTAGCCAAAATGTACAAGACCACACCGG 3 (0.000077%)

AGGAAGAGGATGGAGATGAAGATGAGGAAGCTGAGTCAGCTACGGGCAAGCGGGCAGCTGAAGATGA
TGAGGATGACGATGTCGATACCAAGAAGCAGAA 9 (0.000231%)

AGGAAGCTCCGTAGTCACCGACGAGACCAGAAGTGGCATGATAAACAGTATAAGAAAGCTCATTTGGG
CACAGCCCTAAAGGCCAACCCTTTTGGAGGTG 4 (0.000103%)

AGGAAGCTGAGTCAGCTACGGGCAAGCGGGCAGCTGAAGATGATGAGGATGACGATGTCGATACCAAG
AAGCAGAAGACCGACGAGGATGACTAGACAGC 11 (0.000282%)

AGGACTTCAAACTCTACTCCCACTAATAGCTTTTTGATGACTTCTAGCAAGCCTCGCTAACCTCGCCTTA
CCCCCCACTATTAACCTACTGGGAGAACTC 7 (0.000180%)

AGGATCTCCTTCATCCCTCTCCAGAAGAGGAGAAGAGGAAACACAAGAAGAAACGCCTGGTGCAGAGC
CCCAATTCCTACTTCATGGATGTGAAATGCCC 3 (0.000077%)

AGGATGGAGATGAAGATGAGGAAGCTGAGTCAGCTACGGGCAAGCGGGCAGCTGAAGATGATGAGGA
TGACGATGTCGATACCAAGAAGCAGAAGACCGA 8 (0.000205%)

AGGATGTTCGTCGTGGCAACGTTGCTGGTGACAGCAAAAATGACCCACCAATGGAAGCAGCTGGCTTC
ACTGCTCAGGTGATTATCCTGAACCATCCAGG 7 (0.000180%)

AGGCAGAGGTCCAAGTAAACCGCTAGCTTGTTGCACCGTGGAGGCCACAGGAGCAGAAACATGGAATG
CCAGACGCTGGGGATGCTGGTACAAGTTGTGG4 (0.000103%)

AGGCCGCCGTGACCTATTCACCCTCCACTTCCCGTCTCAGAATCTAAACGTGGTCACCTTCGAGTAGAG
AGGCCCGCCCGCCCACCGTGGGCAGTGCCAC 3 (0.000077%)

AGGCTGCTGAGATGGGAAAGGGCTCCTTCAAGTATGCCTGGGTCTTGGATAAACTGAAAGCTGAGCGT
GAACGTGGTATCACCATTGATATCTCCTTGTG 3 (0.000077%)

AGGCTGCTGGAGCTGGCAAGGTCACCAAGTCTGCCCAGAAAGCTCAGAAGGCTAAATGAATATTATCC
CTAATACCTGCCACCCCACTCTTAATCAGTGG 29 (0.000744%)

AGGGCCGTTCTGCCATCAACGAAGTGGTAACCCGAGAATACACCATCAACATTCACAAGCGCATCCATG
GAGTGGGCTTCAAGAAGCGTGCACCTCGGGC 4 (0.000103%)

AGGGGCCCTCTCAGCCCTCCTAATGACCTCCGGCCTAGCCATGTGATTTCACTTCCACTCCATAACGCTC
CTCATACTAGGCCTACTAACCAACACACTA 9 (0.000231%)

AGGTCACCAAGTCTGCCCAGAAAGCTCAGAAGGCTAAATGAATATTATCCCTAATACCTGCCACCCCAC
TCTTAATCAGTGGTGGAAGAACGGTCTCAGA 7 (0.000180%)

AGGTGATGGTGAGGAAGAGGATGGAGATGAAGATGAGGAAGCTGAGTCAGCTACGGGCAAGCGGGCA
GCTGAAGATGATGAGGATGACGATGTCGATACC 21 (0.000539%)

AGGTGATTATCCTGAACCATCCAGGCCAAATAAGCGCCGGCTATGCCCCTGTATTGGATTGCCACACGG
CTCACATTGCATGCAAGTTTGCTGAGCTGAA 3 (0.000077%)

AGGTGTCGTGAAAACTACCCCTAAAAGCCAAAATGGGAAAGGAAAAGACTCATATCAACATTGTCGTC
ATTGGACACGTAGATTCGGGCAAGTCCACCAC10 (0.000256%)

AGTAAAACCCAGCCCATGACCCCTAACAGGGGCCCTCTCAGCCCTCCTAATGACCTCCGGCCTAGCCAT
GTGATTTCACTTCCACTCCATAACGCTCCTC 18 (0.000462%)

AGTAAAATCTGTCGAAATGCACCATGAAGCTTTGAGTGAAGCTCTTCCTGGGGACAATGTGGGCTTCAA
TGTCAAGAATGTGTCTGTCAAGGATGTTCGT 5 (0.000128%)

AGTAAACCGCTAGCTTGTTGCACCGTGGAGGCCACAGGAGCAGAAACATGGAATGCCAGACGCTGGGG
ATGCTGGTACAAGTTGTGGGACTGCATGCTAC 5 (0.000128%)

AGTCTGGTGATGCTGCCATTGTTGATATGGTTCCTGGCAAGCCCATGTGTGTTGAGAGCTTCTCAGACTA
TCCACCTTTGGGTCGCTTTGCTGTTCGTGA 19 (0.000487%)

AGTTGGGCCGCTTGGTCAAGGACATGAAGATCAAGTCCCTGGAGGAGATCTATCTCTTCTCCCTGCCCA
TTAAGGAATCAGAGATCATTGATTTCTTCCT 14 (0.000359%)

AGTTTAATCCCTTTGTGACTTCCGACCGAAGCAAGAATCGCAAAAGGCATTTCAATGCACCTTCCCACA
TTCGAAGGAAGATTATGTCTTCCCCTCTTTC 3 (0.000077%)

ATAAAAATAAAAAATTATAACAAACCCTGAGAACCAAAATGAACGAAAATCTGTTCGCTTCATTCATTG
CCCCCACAATCCTAGGCCTACCCGCCGCAGT 20 (0.000513%)

ATAACAAACCCTGAGAACCAAAATGAACGAAAATCTGTTCGCTTCATTCATTGCCCCCACAATCCTAGG
CCTACCCGCCGCAGTACTGATCATTCTATTT 15 (0.000385%)

ATAACCATACACAACACTAAAGGACGAACCTGATCTCTTATACTAGTATCCTTAATCATTTTTATTGCCA
CAACTAACCTCCTCGGACTCCTGCCTCACT 9 (0.000231%)

ATAATCTTCTTCATAGTAATACCCATCATAATCGGAGGCTTTGGCAACTGACTAGTTCCCCTAATAATCG
GTGCCCCCGATATGGCGTTTCCCCGCATAA 6 (0.000154%)

ATACACAACACTAAAGGACGAACCTGATCTCTTATACTAGTATCCTTAATCATTTTTATTGCCACAACTA
ACCTCCTCGGACTCCTGCCTCACTCATTTA26 (0.000667%)

ATACATAGGTATGGTCTGAGCTATGATATCAATTGGCTTCCTAGGGTTTATCGTGTGAGCACACCATATA
TTTACAGTAGGAATAGACGTAGACACACGA 3 (0.000077%)

ATACCTGCCACCCCACTCTTAATCAGTGGTGGAAGAACGGTCTCAGAACTGTTTGTTTCAATTGGCCATT
TAAGTTTAGTAGTAAAAGACTGGTTAATGA 6 (0.000154%)

ATACGGGATAATCCTATTTATTACCTCAGAAGTTTTTTTCTTCGCAGGATTTTTCTGAGCCTTTTACCACT
CCAGCCTAGCCCCTACCCCCCAATTAGGA 7 (0.000180%)

ATAGCTTTTTGATGACTTCTAGCAAGCCTCGCTAACCTCGCCTTACCCCCCACTATTAACCTACTGGGAG
AACTCTCTGTGCTAGTAACCACGTTCTCCT 3 (0.000077%)

ATAGGTATGGTCTGAGCTATGATATCAATTGGCTTCCTAGGGTTTATCGTGTGAGCACACCATATATTTA
CAGTAGGAATAGACGTAGACACACGAGCAT 3 (0.000077%)

ATAGTAAAACCCAGCCCATGACCCCTAACAGGGGCCCTCTCAGCCCTCCTAATGACCTCCGGCCTAGCC
ATGTGATTTCACTTCCACTCCATAACGCTCC 11 (0.000282%)

ATATGCTCATGTGGTGTTGAGGAAAGCAGACATTGACCTCACCAAGAGGGCGGGAGAACTCACTGAGG
ATGAGGTGGAACGTGTGATCACCATTATGCAG4 (0.000103%)

ATCAACGAAGTGGTAACCCGAGAATACACCATCAACATTCACAAGCGCATCCATGGAGTGGGCTTCAA
GAAGCGTGCACCTCGGGCACTCAAAGAGATTC 5 (0.000128%)

ATCACCCTATAGAAGAACTAATGTTAGTATAAGTAACATGAAAACATTCTCCTCCGCATAAGCCTGCGT
CAGATTAAAACACTGAACTGACAATTAACAG 9 (0.000231%)

ATCACCTTCCACCCTTACTACACAATCAAAGACGCCCTCGGCTTACTTCTCTTCATTCTCTCCTTAATGAC
ATTAACACTATTCTCACCAGACCTCCTAG 255 (0.006540%)

ATCCAACATCTCCGCATGATGAAACTTCGGCTCACTCCTTGGCGCCTGCCTGATCCTCCAAATCACCACA
GGACTATTCCTAGCCATGCACTACTCACCA 468 (0.012003%)

ATCCCTAATACCTGCCACCCCACTCTTAATCAGTGGTGGAAGAACGGTCTCAGAACTGTTTGTTTCAATT
GGCCATTTAAGTTTAGTAGTAAAAGACTGG 18 (0.000462%)

ATCCCTCTCCAGAAGAGGAGAAGAGGAAACACAAGAAGAAACGCCTGGTGCAGAGCCCCAATTCCTAC
TTCATGGATGTGAAATGCCCAGGATGCTATAA 4 (0.000103%)

ATCCGTCCTAATCACAGCAGTCCTACTTCTCCTATCTCTCCCAGTCCTAGCTGCTGGCATCACTATACTA
CTAACAGACCGCAACCTCAACACCACCTTC 3 (0.000077%)

ATCGCCGTTCTGGTAAAAAGCTGGAAGATGGCCCTAAATTCTTGAAGTCTGGTGATGCTGCCATTGTTG
ATATGGTTCCTGGCAAGCCCATGTGTGTTGA 35 (0.000898%)

ATCTACAACGTTATCGTCACAGCCCATGCATTTGTAATAATCTTCTTCATAGTAATACCCATCATAATCG
GAGGCTTTGGCAACTGACTAGTTCCCCTAA 3 (0.000077%)

ATCTCCGCATGATGAAACTTCGGCTCACTCCTTGGCGCCTGCCTGATCCTCCAAATCACCACAGGACTAT
TCCTAGCCATGCACTACTCACCAGACGCCT 3 (0.000077%)

ATCTCCTTCATCCCTCTCCAGAAGAGGAGAAGAGGAAACACAAGAAGAAACGCCTGGTGCAGAGCCCC
AATTCCTACTTCATGGATGTGAAATGCCCAGG 4 (0.000103%)

ATCTGTCGAAATGCACCATGAAGCTTTGAGTGAAGCTCTTCCTGGGGACAATGTGGGCTTCAATGTCAA
GAATGTGTCTGTCAAGGATGTTCGTCGTGGC 26 (0.000667%)

ATGAAACTTCGGCTCACTCCTTGGCGCCTGCCTGATCCTCCAAATCACCACAGGACTATTCCTAGCCATG
CACTACTCACCAGACGCCTCAACCGCCTTT 12 (0.000308%)

ATGAAGATGAGGAAGCTGAGTCAGCTACGGGCAAGCGGGCAGCTGAAGATGATGAGGATGACGATGTC
GATACCAAGAAGCAGAAGACCGACGAGGATGA 32 (0.000821%)

ATGAAGGCCTCGGGCACGCTACGAGAGTACAAGGTAGTGGGTCGCTGCCTGCCCACCCCCAAATGCCA
CACGCCGCCCCTCTACCGCATGCGAATCTTTG 13 (0.000333%)

ATGAAGTTTAATCCCTTTGTGACTTCCGACCGAAGCAAGAATCGCAAAAGGCATTTCAATGCACCTTCC

CACATTCGAAGGAAGATTATGTCTTCCCCTC 628 (0.016107%)

ATGAATCCTGTGGAGCATCCTTTGGAGGTGGCAACCACCAGCACATCGGCAAGCCCTCCACCATCCGC
AGAGATGCCCCTGCTGGCCGCAAAGTGGGTC 158 (0.004052%)

ATGACCCCTAACAGGGGCCCTCTCAGCCCTCCTAATGACCTCCGGCCTAGCCATGTGATTTCACTTCCAC
TCCATAACGCTCCTCATACTAGGCCTACTA 23 (0.000590%)

ATGACTTCTAGCAAGCCTCGCTAACCTCGCCTTACCCCCCACTATTAACCTACTGGGAGAACTCTCTGTG
CTAGTAACCACGTTCTCCTGATCAAATATC 4 (0.000103%)

ATGAGGAAGCTGAGTCAGCTACGGGCAAGCGGGCAGCTGAAGATGATGAGGATGACGATGTCGATACC
AAGAAGCAGAAGACCGACGAGGATGACTAGAC 4 (0.000103%)

ATGAGGCAGAGGTCCAAGTAAACCGCTAGCTTGTTGCACCGTGGAGGCCACAGGAGCAGAAACATGGA
ATGCCAGACGCTGGGGATGCTGGTACAAGTTG4 (0.000103%)

ATGATAACCATACACAACACTAAAGGACGAACCTGATCTCTTATACTAGTATCCTTAATCATTTTTATTG
CCACAACTAACCTCCTCGGACTCCTGCCTC 7 (0.000180%)

ATGCATTTGTAATAATCTTCTTCATAGTAATACCCATCATAATCGGAGGCTTTGGCAACTGACTAGTTCC
CCTAATAATCGGTGCCCCCGATATGGCGTT 4 (0.000103%)

ATGCCCGTCACCAAGTTGGGCCGCTTGGTCAAGGACATGAAGATCAAGTCCCTGGAGGAGATCTATCTC
TTCTCCCTGCCCATTAAGGAATCAGAGATCA 26 (0.000667%)

ATGCCCTTCTGGCTTACACACTGGGTGTGAAACAACTAATTGTCGGTGTTAACAAAATGGATTCCACTG
AGCCACCCTACAGCCAGAAGAGATATGAGGA 7 (0.000180%)

ATGCGCAGGCTGAAGCGCAAAAGAAGAAAGATGAGGCAGAGGTCCAAGTAAACCGCTAGCTTGTTGCA
CCGTGGAGGCCACAGGAGCAGAAACATGGAAT 4 (0.000103%)

ATGCTCATGTGGTGTTGAGGAAAGCAGACATTGACCTCACCAAGAGGGCGGGAGAACTCACTGAGGAT
GAGGTGGAACGTGTGATCACCATTATGCAGAA 6 (0.000154%)

ATGGAAGCAGCTGGCTTCACTGCTCAGGTGATTATCCTGAACCATCCAGGCCAAATAAGCGCCGGCTAT
GCCCCTGTATTGGATTGCCACACGGCTCACA 8 (0.000205%)

ATGGAGATGAAGATGAGGAAGCTGAGTCAGCTACGGGCAAGCGGGCAGCTGAAGATGATGAGGATGA
CGATGTCGATACCAAGAAGCAGAAGACCGACGA 10 (0.000256%)

ATGGCCCTAAATTCTTGAAGTCTGGTGATGCTGCCATTGTTGATATGGTTCCTGGCAAGCCCATGTGTGT
TGAGAGCTTCTCAGACTATCCACCTTTGGG 6 (0.000154%)

ATGGGAAAGGAAAAGACTCATATCAACATTGTCGTCATTGGACACGTAGATTCGGGCAAGTCCACCACT
ACTGGCCATCTGATCTATAAATGCGGTGGCA 5 (0.000128%)

ATGGGAAAGGGCTCCTTCAAGTATGCCTGGGTCTTGGATAAACTGAAAGCTGAGCGTGAACGTGGTATC
ACCATTGATATCTCCTTGTGGAAATTTGAGA 19 (0.000487%)

ATGGGTCCCTCGGCTTCCTGCCTCGGAAGCGCAGCAGCAGGCATCGTGGGAAGGTGAAGAGCTTCCCTA
AGGATGACCCGTCCAAGCCGGTCCACCTCAC 4 (0.000103%)

ATGGTGAGGAAGAGGATGGAGATGAAGATGAGGAAGCTGAGTCAGCTACGGGCAAGCGGGCAGCTGA
AGATGATGAGGATGACGATGTCGATACCAAGAA 10 (0.000256%)

ATGTCAAGAATGTGTCTGTCAAGGATGTTCGTCGTGGCAACGTTGCTGGTGACAGCAAAAATGACCCAC
CAATGGAAGCAGCTGGCTTCACTGCTCAGGT 4 (0.000103%)

ATTATAACAAACCCTGAGAACCAAAATGAACGAAAATCTGTTCGCTTCATTCATTGCCCCCACAATCCT AGGCCTACCCGCCGCAGTACTGATCATTCTA 86 (0.002206%)

ATTATCCCTAATACCTGCCACCCCACTCTTAATCAGTGGTGGAAGAACGGTCTCAGAACTGTTTGTTTCA ATTGGCCATTTAAGTTTAGTAGTAAAAGAC 33 (0.000846%)

ATTATCCTGAACCATCCAGGCCAAATAAGCGCCGGCTATGCCCCTGTATTGGATTGCCACACGGCTCAC ATTGCATGCAAGTTTGCTGAGCTGAAGGAAA 13 (0.000333%)

ATTCAGACTGAGCGTGCCTACCAAAAGCAGCCGACCATCTTTCAAAACAAGAAGAGGGTCCTGCTGGG AGAAACTGGCAAGGAGAAGCTCCCGCGGTACT 50 (0.001282%)

ATTCTTGAAGTCTGGTGATGCTGCCATTGTTGATATGGTTCCTGGCAAGCCCATGTGTGTTGAGAGCTTC TCAGACTATCCACCTTTGGGTCGCTTTGCT18 (0.000462%)

ATTGATCGCCGTTCTGGTAAAAAGCTGGAAGATGGCCCTAAATTCTTGAAGTCTGGTGATGCTGCCATT GTTGATATGGTTCCTGGCAAGCCCATGTGTG 992 (0.025442%)

ATTTGGATACATAGGTATGGTCTGAGCTATGATATCAATTGGCTTCCTAGGGTTTATCGTGTGAGCACAC CATATATTTACAGTAGGAATAGACGTAGAC 43 (0.001103%)

ATTTGTAATAATCTTCTTCATAGTAATACCCATCATAATCGGAGGCTTTGGCAACTGACTAGTTCCCCTA ATAATCGGTGCCCCCGATATGGCGTTTCCC 5 (0.000128%)

CAAAAATGACCCACCAATGGAAGCAGCTGGCTTCACTGCTCAGGTGATTATCCTGAACCATCCAGGCCA AATAAGCGCCGGCTATGCCCCTGTATTGGAT 98 (0.002513%)

CAAAACAAATGATAACCATACACAACACTAAAGGACGAACCTGATCTCTTATACTAGTATCCTTAATCA TTTTTATTGCCACAACTAACCTCCTCGGACT 9 (0.000231%)

CAAAACAAGAAGAGGGTCCTGCTGGGAGAAACTGGCAAGGAGAAGCTCCCGCGGTACTACAAGAACA TCGGTCTGGGCTTCAAGACACCCAAGGAGGCTA 3 (0.000077%)

CAAAAGAAGAAAGATGAGGCAGAGGTCCAAGTAAACCGCTAGCTTGTTGCACCGTGGAGGCCACAGGA GCAGAAACATGGAATGCCAGACGCTGGGGATG 6 (0.000154%)

CAAAAGCAGCCGACCATCTTTCAAAACAAGAAGAGGGTCCTGCTGGGAGAAACTGGCAAGGAGAAGCT CCCGCGGTACTACAAGAACATCGGTCTGGGCT10 (0.000256%)

CAAAATGGGAAAGGAAAAGACTCATATCAACATTGTCGTCATTGGACACGTAGATTCGGGCAAGTCCA CCACTACTGGCCATCTGATCTATAAATGCGGT 15 (0.000385%)

CAAACCCTGAGAACCAAAATGAACGAAAATCTGTTCGCTTCATTCATTGCCCCCACAATCCTAGGCCTA CCCGCCGCAGTACTGATCATTCTATTTCCCC 41 (0.001052%)

CAAACTCTACTCCCACTAATAGCTTTTTGATGACTTCTAGCAAGCCTCGCTAACCTCGCCTTACCCCCCA CTATTAACCTACTGGGAGAACTCTCTGTGC 5 (0.000128%)

CAAAGCCCATAAAAATAAAAAATTATAACAAACCCTGAGAACCAAAATGAACGAAAATCTGTTCGCTT CATTCATTGCCCCCACAATCCTAGGCCTACCC 32 (0.000821%)

CAAAGGATCTCCTTCATCCCTCTCCAGAAGAGGAGAAGAGGAAACACAAGAAGAAACGCCTGGTGCAG AGCCCCAATTCCTACTTCATGGATGTGAAATG 3 (0.000077%)

CAAATGATAACCATACACAACACTAAAGGACGAACCTGATCTCTTATACTAGTATCCTTAATCATTTTTA TTGCCACAACTAACCTCCTCGGACTCCTGC 15 (0.000385%)

CAAATGGTCATTGATGTCCTTCACCCCGGGAAGGCGACAGTGCCTAAGACAGAAATTCGGGAAAAACT
AGCCAAAATGTACAAGACCACACCGGATGTCA 10 (0.000256%)

CAACAAAACTAACTAATACTAACATCTCAGACGCTCAGGAAATAGAAACCGTCTGAACTATCCTGCCCG
CCATCATCCTAGTCCTCATCGCCCTCCCATC 4 (0.000103%)

CAACACTAAAGGACGAACCTGATCTCTTATACTAGTATCCTTAATCATTTTTATTGCCACAACTAACCTC
CTCGGACTCCTGCCTCACTCATTTACACCA 71 (0.001821%)

CAACATCTCCGCATGATGAAACTTCGGCTCACTCCTTGGCGCCTGCCTGATCCTCCAAATCACCACAGG
ACTATTCCTAGCCATGCACTACTCACCAGAC 29 (0.000744%)

CAACCGACTACTTCAGAGGAAACAAATGGTCATTGATGTCCTTCACCCCGGGAAGGCGACAGTGCCTAA
GACAGAAATTCGGGAAAAACTAGCCAAAATG 421 (0.010798%)

CAACGGAAGTAAAATCTGTCGAAATGCACCATGAAGCTTTGAGTGAAGCTCTTCCTGGGGACAATGTGG
GCTTCAATGTCAAGAATGTGTCTGTCAAGGA 6 (0.000154%)

CAACGTTACAACGGAAGTAAAATCTGTCGAAATGCACCATGAAGCTTTGAGTGAAGCTCTTCCTGGGGA
CAATGTGGGCTTCAATGTCAAGAATGTGTCT 27 (0.000692%)

CAACGTTATCGTCACAGCCCATGCATTTGTAATAATCTTCTTCATAGTAATACCCATCATAATCGGAGGC
TTTGGCAACTGACTAGTTCCCCTAATAATC 29 (0.000744%)

CAACGTTGCTGGTGACAGCAAAAATGACCCACCAATGGAAGCAGCTGGCTTCACTGCTCAGGTGATTAT
CCTGAACCATCCAGGCCAAATAAGCGCCGGC 32 (0.000821%)

CAACTTCCTTCGGTCGTCCCGAATCCGGGTTCATCCGACACCAGCCGCCTCCACCATGCCGCCGAAGTTC
GACCCCAACGAGATCAAAGTCGTATACCTG 22 (0.000564%)

CAAGAATGTGTCTGTCAAGGATGTTCGTCGTGGCAACGTTGCTGGTGACAGCAAAAATGACCCACCAAT
GGAAGCAGCTGGCTTCACTGCTCAGGTGATT 80 (0.002052%)

CAAGCATGGTTAACGTCCCTAAAACCCGCCGGACTTTCTGTAAGAAGTGTGGCAAGCACCAACCCCATA
AAGTGACACAGTACAAGAAGGGCAAGGATTC 7 (0.000180%)

CAAGGACTTCAAACTCTACTCCCACTAATAGCTTTTTGATGACTTCTAGCAAGCCTCGCTAACCTCGCCT
TACCCCCCACTATTAACCTACTGGGAGAAC 7 (0.000180%)

CAAGGATGAGGTTTTGAAGATTATGCCAGTGCAGAAGCAGACCCGTGCCGGCCAGCGCACCAGGTTCA
AGGCATTTGTTGCTATCGGGGACTACAATGGC 16 (0.000410%)

CAAGGATGTTCGTCGTGGCAACGTTGCTGGTGACAGCAAAAATGACCCACCAATGGAAGCAGCTGGCT
TCACTGCTCAGGTGATTATCCTGAACCATCCA 70 (0.001795%)

CAAGGTCACCAAGTCTGCCCAGAAAGCTCAGAAGGCTAAATGAATATTATCCCTAATACCTGCCACCCC
ACTCTTAATCAGTGGTGGAAGAACGGTCTCA 5 (0.000128%)

CAAGGTGCAACTTCCTTCGGTCGTCCCGAATCCGGGTTCATCCGACACCAGCCGCCTCCACCATGCCGC
CGAAGTTCGACCCCAACGAGATCAAAGTCGT 25 (0.000641%)

CAAGTAAACCGCTAGCTTGTTGCACCGTGGAGGCCACAGGAGCAGAAACATGGAATGCCAGACGCTGG
GGATGCTGGTACAAGTTGTGGGACTGCATGCT 4 (0.000103%)

CAAGTCCACCACTACTGGCCATCTGATCTATAAATGCGGTGGCATCGACAAAAGAACCATTGAAAAATT
TGAGAAGGAGGCTGCTGAGATGGGAAAGGGC 6 (0.000154%)

CAAGTCTGCCCAGAAAGCTCAGAAGGCTAAATGAATATTATCCCTAATACCTGCCACCCCACTCTTAAT

CAGTGGTGGAAGAACGGTCTCAGAACTGTTT 23 (0.000590%)

CAAGTTGGGCCGCTTGGTCAAGGACATGAAGATCAAGTCCCTGGAGGAGATCTATCTCTTCTCCCTGCC
CATTAAGGAATCAGAGATCATTGATTTCTTC 21 (0.000539%)

CAATGGAAGCAGCTGGCTTCACTGCTCAGGTGATTATCCTGAACCATCCAGGCCAAATAAGCGCCGGCT
ATGCCCCTGTATTGGATTGCCACACGGCTCA 23 (0.000590%)

CAATGTCAAGAATGTGTCTGTCAAGGATGTTCGTCGTGGCAACGTTGCTGGTGACAGCAAAAATGACCC
ACCAATGGAAGCAGCTGGCTTCACTGCTCAG 9 (0.000231%)

CACAACAAAACTAACTAATACTAACATCTCAGACGCTCAGGAAATAGAAACCGTCTGAACTATCCTGCC
CGCCATCATCCTAGTCCTCATCGCCCTCCCA 5 (0.000128%)

CACAACACTAAAGGACGAACCTGATCTCTTATACTAGTATCCTTAATCATTTTTATTGCCACAACTAACC
TCCTCGGACTCCTGCCTCACTCATTTACAC28 (0.000718%)

CACACGAGAACATGCCTCTCGCAAAGGATCTCCTTCATCCCTCTCCAGAAGAGGAGAAGAGGAAACAC
AAGAAGAAACGCCTGGTGCAGAGCCCCAATTC 18 (0.000462%)

CACAGAAAGTTCTCCGCTCCCAGACATGGGTCCCTCGGCTTCCTGCCTCGGAAGCGCAGCAGCAGGCAT
CGTGGGAAGGTGAAGAGCTTCCCTAAGGATG 6 (0.000154%)

CACAGGTGTCGTGAAAACTACCCCTAAAAGCCAAAATGGGAAAGGAAAAGACTCATATCAACATTGTC
GTCATTGGACACGTAGATTCGGGCAAGTCCAC 189 (0.004847%)

CACATCTACAACGTTATCGTCACAGCCCATGCATTTGTAATAATCTTCTTCATAGTAATACCCATCATAA
TCGGAGGCTTTGGCAACTGACTAGTTCCCC 14 (0.000359%)

CACCAAAGCCCATAAAAATAAAAAATTATAACAAACCCTGAGAACCAAAATGAACGAAAATCTGTTCG
CTTCATTCATTGCCCCCACAATCCTAGGCCTA 13 (0.000333%)

CACCAAGTCTGCCCAGAAAGCTCAGAAGGCTAAATGAATATTATCCCTAATACCTGCCACCCCACTCTT
AATCAGTGGTGGAAGAACGGTCTCAGAACTG 21 (0.000539%)

CACCAAGTTGGGCCGCTTGGTCAAGGACATGAAGATCAAGTCCCTGGAGGAGATCTATCTCTTCTCCCT
GCCCATTAAGGAATCAGAGATCATTGATTTC 20 (0.000513%)

CACCAATGGAAGCAGCTGGCTTCACTGCTCAGGTGATTATCCTGAACCATCCAGGCCAAATAAGCGCCG
GCTATGCCCCTGTATTGGATTGCCACACGGC 123 (0.003155%)

CACCTCATATCCTCCCTACTATGCCTAGAAGGAATAATACTATCGCTGTTCATTATAGCTACTCTCATAA
CCCTCAACACCCACTCCCTCTTAGCCAATA 4 (0.000103%)

CACCTTCCACCCTTACTACACAATCAAAGACGCCCTCGGCTTACTTCTCTTCATTCTCTCCTTAATGACAT
TAACACTATTCTCACCAGACCTCCTAGGC 7 (0.000180%)

CACGAGAACATGCCTCTCGCAAAGGATCTCCTTCATCCCTCTCCAGAAGAGGAGAAGAGGAAACACAA
GAAGAAACGCCTGGTGCAGAGCCCCAATTCCT 16 (0.000410%)

CACGCAAGCATGGTTAACGTCCCTAAAACCCGCCGGACTTTCTGTAAGAAGTGTGGCAAGCACCAACCC
CATAAAGTGACACAGTACAAGAAGGGCAAGG 10 (0.000256%)

CACGCCATGAAGGCCTCGGGCACGCTACGAGAGTACAAGGTAGTGGGTCGCTGCCTGCCCACCCCCAA
ATGCCACACGCCGCCCCTCTACCGCATGCGAA 6 (0.000154%)

CACGCTACGAGAGTACAAGGTAGTGGGTCGCTGCCTGCCCACCCCCAAATGCCACACGCCGCCCCTCTA
CCGCATGCGAATCTTTGCGCCTAATCATGTC 6 (0.000154%)

CACTAAAGGACGAACCTGATCTCTTATACTAGTATCCTTAATCATTTTTATTGCCACAACTAACCTCCTC
GGACTCCTGCCTCACTCATTTACACCAACC 13 (0.000333%)

CACTAATAGCTTTTTGATGACTTCTAGCAAGCCTCGCTAACCTCGCCTTACCCCCCACTATTAACCTACT
GGGAGAACTCTCTGTGCTAGTAACCACGTT 6 (0.000154%)

CACTCACAACAAAACTAACTAATACTAACATCTCAGACGCTCAGGAAATAGAAACCGTCTGAACTATCC
TGCCCGCCATCATCCTAGTCCTCATCGCCCT 10 (0.000256%)

CACTGCTCAGGTGATTATCCTGAACCATCCAGGCCAAATAAGCGCCGGCTATGCCCCTGTATTGGATTG
CCACACGGCTCACATTGCATGCAAGTTTGCT 11 (0.000282%)

CAGAAAGCTCAGAAGGCTAAATGAATATTATCCCTAATACCTGCCACCCCACTCTTAATCAGTGGTGGA
AGAACGGTCTCAGAACTGTTTGTTTCAATTG 27 (0.000692%)

CAGAAAGTTCTCCGCTCCCAGACATGGGTCCCTCGGCTTCCTGCCTCGGAAGCGCAGCAGCAGGCATCG
TGGGAAGGTGAAGAGCTTCCCTAAGGATGAC 17 (0.000436%)

CAGAACACAGGTGTCGTGAAAACTACCCCTAAAAGCCAAAATGGGAAAGGAAAAGACTCATATCAACA
TTGTCGTCATTGGACACGTAGATTCGGGCAAG 203 (0.005206%)

CAGAAGGCTAAATGAATATTATCCCTAATACCTGCCACCCCACTCTTAATCAGTGGTGGAAGAACGGTC
TCAGAACTGTTTGTTTCAATTGGCCATTTAA 25 (0.000641%)

CAGACATGGGTCCCTCGGCTTCCTGCCTCGGAAGCGCAGCAGCAGGCATCGTGGGAAGGTGAAGAGCT
TCCCTAAGGATGACCCGTCCAAGCCGGTCCAC 7 (0.000180%)

CAGACCCGAGAGCATGCCCTTCTGGCTTACACACTGGGTGTGAAACAACTAATTGTCGGTGTTAACAAA
ATGGATTCCACTGAGCCACCCTACAGCCAGA 443 (0.011362%)

CAGACTGAGCGTGCCTACCAAAAGCAGCCGACCATCTTTCAAAACAAGAAGAGGGTCCTGCTGGGAGA
AACTGGCAAGGAGAAGCTCCCGCGGTACTACA 9 (0.000231%)

CAGAGGTCCAAGTAAACCGCTAGCTTGTTGCACCGTGGAGGCCACAGGAGCAGAAACATGGAATGCCA
GACGCTGGGGATGCTGGTACAAGTTGTGGGAC 67 (0.001718%)

CAGCCCATGACCCCTAACAGGGGCCCTCTCAGCCCTCCTAATGACCTCCGGCCTAGCCATGTGATTTCA
CTTCCACTCCATAACGCTCCTCATACTAGGC 35 (0.000898%)

CAGCCCTCCTAATGACCTCCGGCCTAGCCATGTGATTTCACTTCCACTCCATAACGCTCCTCATACTAGG
CCTACTAACCAACACACTAACCATATACCA 15 (0.000385%)

CAGCCGACCATCTTTCAAAACAAGAAGAGGGTCCTGCTGGGAGAAACTGGCAAGGAGAAGCTCCCGCG
GTACTACAAGAACATCGGTCTGGGCTTCAAGA6 (0.000154%)

CAGCCGCCTCCGCCGCGCGCCTCCTCCGCCGCCGCGGACTCCGGCAGCTTTATCGCCAGAGTCCCTGAA
CTCTCGCTTTCTTTTTAATCCCCTGCATCGG 3 (0.000077%)

CAGCTGGCTTCACTGCTCAGGTGATTATCCTGAACCATCCAGGCCAAATAAGCGCCGGCTATGCCCCTG
TATTGGATTGCCACACGGCTCACATTGCATG 15 (0.000385%)

CAGGCACATACTTCCTATTCTACACCCTAGTAGGCTCCCTTCCCCTACTCATCGCACTGATTTACACTCA
CAACACCCTAGGCTCACTAAACATTCTACT 27 (0.000692%)

CAGGGGCCCTCTCAGCCCTCCTAATGACCTCCGGCCTAGCCATGTGATTTCACTTCCACTCCATAACGCT
CCTCATACTAGGCCTACTAACCAACACACT 23 (0.000590%)

CAGGTGTCGTGAAAACTACCCCTAAAAGCCAAAATGGGAAAGGAAAAGACTCATATCAACATTGTCGT
CATTGGACACGTAGATTCGGGCAAGTCCACCA89 (0.002283%)

CATAAAAATAAAAAATTATAACAAACCCTGAGAACCAAAATGAACGAAAATCTGTTCGCTTCATTCATT
GCCCCCACAATCCTAGGCCTACCCGCCGCAG 4 (0.000103%)

CATACACAACACTAAAGGACGAACCTGATCTCTTATACTAGTATCCTTAATCATTTTTATTGCCACAACT
AACCTCCTCGGACTCCTGCCTCACTCATTT27 (0.000692%)

CATAGGTATGGTCTGAGCTATGATATCAATTGGCTTCCTAGGGTTTATCGTGTGAGCACACCATATATTT
ACAGTAGGAATAGACGTAGACACACGAGCA 5 (0.000128%)

CATATCAACATTGTCGTCATTGGACACGTAGATTCGGGCAAGTCCACCACTACTGGCCATCTGATCTAT
AAATGCGGTGGCATCGACAAAAGAACCATTG 14 (0.000359%)

CATCAACGAAGTGGTAACCCGAGAATACACCATCAACATTCACAAGCGCATCCATGGAGTGGGCTTCA
AGAAGCGTGCACCTCGGGCACTCAAAGAGATT 14 (0.000359%)

CATCCCTCTCCAGAAGAGGAGAAGAGGAAACACAAGAAGAAACGCCTGGTGCAGAGCCCCAATTCCTA
CTTCATGGATGTGAAATGCCCAGGATGCTATA 5 (0.000128%)

CATCTACAACGTTATCGTCACAGCCCATGCATTTGTAATAATCTTCTTCATAGTAATACCCATCATAATC
GGAGGCTTTGGCAACTGACTAGTTCCCCTA 9 (0.000231%)

CATCTCCGCATGATGAAACTTCGGCTCACTCCTTGGCGCCTGCCTGATCCTCCAAATCACCACAGGACTA
TTCCTAGCCATGCACTACTCACCAGACGCC 8 (0.000205%)

CATCTTTCAAAACAAGAAGAGGGTCCTGCTGGGAGAAACTGGCAAGGAGAAGCTCCCGCGGTACTACA
AGAACATCGGTCTGGGCTTCAAGACACCCAAG 5 (0.000128%)

CATGAAGGCCTCGGGCACGCTACGAGAGTACAAGGTAGTGGGTCGCTGCCTGCCCACCCCCAAATGCC
ACACGCCGCCCCTCTACCGCATGCGAATCTTT 7 (0.000180%)

CATGACCCCTAACAGGGGCCCTCTCAGCCCTCCTAATGACCTCCGGCCTAGCCATGTGATTTCACTTCCA
CTCCATAACGCTCCTCATACTAGGCCTACT 24 (0.000616%)

CATGATGAAACTTCGGCTCACTCCTTGGCGCCTGCCTGATCCTCCAAATCACCACAGGACTATTCCTAGC
CATGCACTACTCACCAGACGCCTCAACCGC 3 (0.000077%)

CATGCATTTGTAATAATCTTCTTCATAGTAATACCCATCATAATCGGAGGCTTTGGCAACTGACTAGTTC
CCCTAATAATCGGTGCCCCCGATATGGCGT 3 (0.000077%)

CATGCCCTTCTGGCTTACACACTGGGTGTGAAACAACTAATTGTCGGTGTTAACAAAATGGATTCCACT
GAGCCACCCTACAGCCAGAAGAGATATGAGG 3 (0.000077%)

CATGCCTCCATCTAGAAGAGATTATGATGATATGAGCCCTCGTCGAGGACCACCTCCCCCTCCTCCCGG
ACGAGGCGGCCGGGGTGGTAGCAGAGCTCGG 6 (0.000154%)

CATGCCTCTCGCAAAGGATCTCCTTCATCCCTCTCCAGAAGAGGAGAAGAGGAAACACAAGAAGAAAC
GCCTGGTGCAGAGCCCCAATTCCTACTTCATG 31 (0.000795%)

CATTCAGACTGAGCGTGCCTACCAAAAGCAGCCGACCATCTTTCAAAACAAGAAGAGGGTCCTGCTGG
GAGAAACTGGCAAGGAGAAGCTCCCGCGGTAC 9 (0.000231%)

CATTTGGATACATAGGTATGGTCTGAGCTATGATATCAATTGGCTTCCTAGGGTTTATCGTGTGAGCACA
CCATATATTTACAGTAGGAATAGACGTAGA 11 (0.000282%)

CCAAAAGCAGCCGACCATCTTTCAAAACAAGAAGAGGGTCCTGCTGGGAGAAACTGGCAAGGAGAAGC

TCCCGCGGTACTACAAGAACATCGGTCTGGGC 5 (0.000128%)

CCAAAATGGGAAAGGAAAAGACTCATATCAACATTGTCGTCATTGGACACGTAGATTCGGGCAAGTCC
ACCACTACTGGCCATCTGATCTATAAATGCGG 18 (0.000462%)

CCAAAGCCCATAAAAATAAAAAATTATAACAAACCCTGAGAACCAAAATGAACGAAAATCTGTTCGCT
TCATTCATTGCCCCCACAATCCTAGGCCTACC 14 (0.000359%)

CCAACATCTCCGCATGATGAAACTTCGGCTCACTCCTTGGCGCCTGCCTGATCCTCCAAATCACCACAG
GACTATTCCTAGCCATGCACTACTCACCAGA 77 (0.001975%)

CCAAGGTGCAACTTCCTTCGGTCGTCCCGAATCCGGGTTCATCCGACACCAGCCGCCTCCACCATGCCG
CCGAAGTTCGACCCCAACGAGATCAAAGTCG 177 (0.004540%)

CCAAGTAAACCGCTAGCTTGTTGCACCGTGGAGGCCACAGGAGCAGAAACATGGAATGCCAGACGCTG
GGGATGCTGGTACAAGTTGTGGGACTGCATGC 12 (0.000308%)

CCAAGTCTGCCCAGAAAGCTCAGAAGGCTAAATGAATATTATCCCTAATACCTGCCACCCCACTCTTAA
TCAGTGGTGGAAGAACGGTCTCAGAACTGTT 11 (0.000282%)

CCAAGTTGGGCCGCTTGGTCAAGGACATGAAGATCAAGTCCCTGGAGGAGATCTATCTCTTCTCCCTGC
CCATTAAGGAATCAGAGATCATTGATTTCTT 36 (0.000923%)

CCAATGGAAGCAGCTGGCTTCACTGCTCAGGTGATTATCCTGAACCATCCAGGCCAAATAAGCGCCGGC
TATGCCCCTGTATTGGATTGCCACACGGCTC 14 (0.000359%)

CCACATCTACAACGTTATCGTCACAGCCCATGCATTTGTAATAATCTTCTTCATAGTAATACCCATCATA
ATCGGAGGCTTTGGCAACTGACTAGTTCCC 3 (0.000077%)

CCACCAATGGAAGCAGCTGGCTTCACTGCTCAGGTGATTATCCTGAACCATCCAGGCCAAATAAGCGCC
GGCTATGCCCCTGTATTGGATTGCCACACGG 4 (0.000103%)

CCACTAATAGCTTTTTGATGACTTCTAGCAAGCCTCGCTAACCTCGCCTTACCCCCCACTATTAACCTAC
TGGGAGAACTCTCTGTGCTAGTAACCACGT 3 (0.000077%)

CCAGAAAGCTCAGAAGGCTAAATGAATATTATCCCTAATACCTGCCACCCCACTCTTAATCAGTGGTGG
AAGAACGGTCTCAGAACTGTTTGTTTCAATT 6 (0.000154%)

CCAGAACACAGGTGTCGTGAAAACTACCCCTAAAAGCCAAAATGGGAAAGGAAAAGACTCATATCAAC
ATTGTCGTCATTGGACACGTAGATTCGGGCAA 845 (0.021672%)

CCAGCCCATGACCCCTAACAGGGGCCCTCTCAGCCCTCCTAATGACCTCCGGCCTAGCCATGTGATTTC
ACTTCCACTCCATAACGCTCCTCATACTAGG 19 (0.000487%)

CCAGTCAACGTTACAACGGAAGTAAAATCTGTCGAAATGCACCATGAAGCTTTGAGTGAAGCTCTTCCT
GGGGACAATGTGGGCTTCAATGTCAAGAATG 7 (0.000180%)

CCATAAAAATAAAAAATTATAACAAACCCTGAGAACCAAAATGAACGAAAATCTGTTCGCTTCATTCAT
TGCCCCCACAATCCTAGGCCTACCCGCCGCA 4 (0.000103%)

CCATACACAACACTAAAGGACGAACCTGATCTCTTATACTAGTATCCTTAATCATTTTTATTGCCACAAC
TAACCTCCTCGGACTCCTGCCTCACTCATT3 (0.000077%)

CCATGAAGGCCTCGGGCACGCTACGAGAGTACAAGGTAGTGGGTCGCTGCCTGCCCACCCCCAAATGC
CACACGCCGCCCCTCTACCGCATGCGAATCTT 3 (0.000077%)

CCATGACCCCTAACAGGGGCCCTCTCAGCCCTCCTAATGACCTCCGGCCTAGCCATGTGATTTCACTTCC
ACTCCATAACGCTCCTCATACTAGGCCTAC 21 (0.000539%)

CCATGCCTCCATCTAGAAGAGATTATGATGATATGAGCCCTCGTCGAGGACCACCTCCCCCTCCTCCCG
GACGAGGCGGCCGGGGTGGTAGCAGAGCTCG 3 (0.000077%)

CCCACCAATGGAAGCAGCTGGCTTCACTGCTCAGGTGATTATCCTGAACCATCCAGGCCAAATAAGCGC
CGGCTATGCCCCTGTATTGGATTGCCACACG 9 (0.000231%)

CCCACTAATAGCTTTTTGATGACTTCTAGCAAGCCTCGCTAACCTCGCCTTACCCCCCACTATTAACCTA
CTGGGAGAACTCTCTGTGCTAGTAACCACG 3 (0.000077%)

CCCAGAAAGCTCAGAAGGCTAAATGAATATTATCCCTAATACCTGCCACCCCACTCTTAATCAGTGGTG
GAAGAACGGTCTCAGAACTGTTTGTTTCAAT 6 (0.000154%)

CCCAGACATGGGTCCCTCGGCTTCCTGCCTCGGAAGCGCAGCAGCAGGCATCGTGGGAAGGTGAAGAG
CTTCCCTAAGGATGACCCGTCCAAGCCGGTCC 9 (0.000231%)

CCCAGCCCATGACCCCTAACAGGGGCCCTCTCAGCCCTCCTAATGACCTCCGGCCTAGCCATGTGATTTC
ACTTCCACTCCATAACGCTCCTCATACTAG 41 (0.001052%)

CCCATGACCCCTAACAGGGGCCCTCTCAGCCCTCCTAATGACCTCCGGCCTAGCCATGTGATTTCACTTC
CACTCCATAACGCTCCTCATACTAGGCCTA 23 (0.000590%)

CCCATGCCTCCATCTAGAAGAGATTATGATGATATGAGCCCTCGTCGAGGACCACCTCCCCCTCCTCCC
GGACGAGGCGGCCGGGGTGGTAGCAGAGCTC 6 (0.000154%)

CCCCTAAAAGCCAAAATGGGAAAGGAAAAGACTCATATCAACATTGTCGTCATTGGACACGTAGATTC
GGGCAAGTCCACCACTACTGGCCATCTGATCT 6 (0.000154%)

CCCCTAACAGGGGCCCTCTCAGCCCTCCTAATGACCTCCGGCCTAGCCATGTGATTTCACTTCCACTCCA
TAACGCTCCTCATACTAGGCCTACTAACCA 18 (0.000462%)

CCCGAATCCGGGTTCATCCGACACCAGCCGCCTCCACCATGCCGCCGAAGTTCGACCCCAACGAGATCA
AAGTCGTATACCTGAGGTGCACCGGAGGTGA 14 (0.000359%)

CCCGAGAGCATGCCCTTCTGGCTTACACACTGGGTGTGAAACAACTAATTGTCGGTGTTAACAAAATGG
ATTCCACTGAGCCACCCTACAGCCAGAAGAG 15 (0.000385%)

CCCGCTATGGGGCCTCCCTCCGGAAAATGGTGAAGAAAATTGAAATCAGCCAGCACGCCAAGTACACT
TGCTCTTTCTGTGGCAAAACCAAGATGAAGAG 6 (0.000154%)

CCCGTCACCAAGTTGGGCCGCTTGGTCAAGGACATGAAGATCAAGTCCCTGGAGGAGATCTATCTCTTC
TCCCTGCCCATTAAGGAATCAGAGATCATTG 60 (0.001539%)

CCCTAAAAGCCAAAATGGGAAAGGAAAAGACTCATATCAACATTGTCGTCATTGGACACGTAGATTCG
GGCAAGTCCACCACTACTGGCCATCTGATCTA 10 (0.000256%)

CCCTAACAGGGGCCCTCTCAGCCCTCCTAATGACCTCCGGCCTAGCCATGTGATTTCACTTCCACTCCAT
AACGCTCCTCATACTAGGCCTACTAACCAA 49 (0.001257%)

CCCTAATACCTGCCACCCCACTCTTAATCAGTGGTGGAAGAACGGTCTCAGAACTGTTTGTTTCAATTGG
CCATTTAAGTTTAGTAGTAAAAGACTGGTT 39 (0.001000%)

CCCTCACCAAAGCCCATAAAAATAAAAAATTATAACAAACCCTGAGAACCAAAATGAACGAAAATCTG
TTCGCTTCATTCATTGCCCCCACAATCCTAGG 16 (0.000410%)

CCCTCGGCTTCCTGCCTCGGAAGCGCAGCAGCAGGCATCGTGGGAAGGTGAAGAGCTTCCCTAAGGAT
GACCCGTCCAAGCCGGTCCACCTCACAGCCTT 4 (0.000103%)

CCCTCTCAGCCCTCCTAATGACCTCCGGCCTAGCCATGTGATTTCACTTCCACTCCATAACGCTCCTCAT
ACTAGGCCTACTAACCAACACACTAACCAT 8 (0.000205%)

CCCTGATCGGCGCACTGCGAGCAGTAGCCCAAACAATCTCATATGAAGTCACCCTAGCCATCATTCTAC
TATCAACATTACTAATAAGTGGCTCCTTTAA 30 (0.000769%)

CCCTTCTGGCTTACACACTGGGTGTGAAACAACTAATTGTCGGTGTTAACAAAATGGATTCCACTGAGC
CACCCTACAGCCAGAAGAGATATGAGGAAAT 5 (0.000128%)

CCCTTGCGCCTGCCTCTCCAGGATGTCTACAAAATTGGTGGTATTGGTACTGTTCCTGTTGGCCGAGTGG
AGACTGGTGTTCTCAAACCCGGTATGGTGG 3 (0.000077%)

CCGAAGCAAGAATCGCAAAAGGCATTTCAATGCACCTTCCCACATTCGAAGGAAGATTATGTCTTCCCC
TCTTTCCAAAGAGCTGAGACAGAAGTACAAC 4 (0.000103%)

CCGAATCCGGGTTCATCCGACACCAGCCGCCTCCACCATGCCGCCGAAGTTCGACCCCAACGAGATCAA
AGTCGTATACCTGAGGTGCACCGGAGGTGAA 4 (0.000103%)

CCGACACCAGCCGCCTCCACCATGCCGCCGAAGTTCGACCCCAACGAGATCAAAGTCGTATACCTGAGG
TGCACCGGAGGTGAAGTCGGTGCCACTTCTG 21 (0.000539%)

CCGACCGAAGCAAGAATCGCAAAAGGCATTTCAATGCACCTTCCCACATTCGAAGGAAGATTATGTCTT
CCCCTCTTTCCAAAGAGCTGAGACAGAAGTA 7 (0.000180%)

CCGAGAGCATGCCCTTCTGGCTTACACACTGGGTGTGAAACAACTAATTGTCGGTGTTAACAAAATGGA
TTCCACTGAGCCACCCTACAGCCAGAAGAGA 35 (0.000898%)

CCGAGGAGCACCCCGTGCTGCTGACCGAGGCCCCCCTGAACCCCAAGGCCAACCGCGAGAAGATGACC
CAGATCATGTTTGAGACCTTCAACACCCCAGC 11 (0.000282%)

CCGCAGCCGCCTCCGCCGCGCGCCTCCTCCGCCGCCGCGGACTCCGGCAGCTTTATCGCCAGAGTCCCT
GAACTCTCGCTTTCTTTTTAATCCCCTGCAT 10 (0.000256%)

CCGCATGATGAAACTTCGGCTCACTCCTTGGCGCCTGCCTGATCCTCCAAATCACCACAGGACTATTCCT
AGCCATGCACTACTCACCAGACGCCTCAAC 5 (0.000128%)

CCGCCGCAGACGCCGCCGCGATGCGCTACGTCGCCTCCTACCTGCTGGCTGCCCTAGGGGGCAACTCCT
CCCCCAGCGCCAAGGACATCAAGAAGATCTT 20 (0.000513%)

CCGCCGTGACCTATTCACCCTCCACTTCCCGTCTCAGAATCTAAACGTGGTCACCTTCGAGTAGAGAGG
CCCGCCCGCCCACCGTGGGCAGTGCCACCCG 7 (0.000180%)

CCGCCTCCTTGCTCGCCGCAGCCGCCTCCGCCGCGCGCCTCCTCCGCCGCCGCGGACTCCGGCAGCTTTA
TCGCCAGAGTCCCTGAACTCTCGCTTTCTT28 (0.000718%)

CCGCGATGCGCTACGTCGCCTCCTACCTGCTGGCTGCCCTAGGGGGCAACTCCTCCCCCAGCGCCAAGG
ACATCAAGAAGATCTTGGACAGCGTGGGTAT 11 (0.000282%)

CCGCTAGCTTGTTGCACCGTGGAGGCCACAGGAGCAGAAACATGGAATGCCAGACGCTGGGGATGCTG
GTACAAGTTGTGGGACTGCATGCTACTGTCTA 9 (0.000231%)

CCGCTATGGGGCCTCCCTCCGGAAAATGGTGAAGAAAATTGAAATCAGCCAGCACGCCAAGTACACTT
GCTCTTTCTGTGGCAAAACCAAGATGAAGAGA14 (0.000359%)

CCGCTCCCAGACATGGGTCCCTCGGCTTCCTGCCTCGGAAGCGCAGCAGCAGGCATCGTGGGAAGGTGA
AGAGCTTCCCTAAGGATGACCCGTCCAAGCC 5 (0.000128%)

CCGGGTTCATCCGACACCAGCCGCCTCCACCATGCCGCCGAAGTTCGACCCCAACGAGATCAAAGTCGT

ATACCTGAGGTGCACCGGAGGTGAAGTCGGT 8 (0.000205%)

CCGTCACCAAGTTGGGCCGCTTGGTCAAGGACATGAAGATCAAGTCCCTGGAGGAGATCTATCTCTTCT
CCCTGCCCATTAAGGAATCAGAGATCATTGA 75 (0.001924%)

CCGTCCTAATCACAGCAGTCCTACTTCTCCTATCTCTCCCAGTCCTAGCTGCTGGCATCACTATACTACT
AACAGACCGCAACCTCAACACCACCTTCTT 5 (0.000128%)

CCGTGACCTATTCACCCTCCACTTCCCGTCTCAGAATCTAAACGTGGTCACCTTCGAGTAGAGAGGCCC
GCCCGCCCACCGTGGGCAGTGCCACCCGCAG 14 (0.000359%)

CCGTTCTGCCATCAACGAAGTGGTAACCCGAGAATACACCATCAACATTCACAAGCGCATCCATGGAGT
GGGCTTCAAGAAGCGTGCACCTCGGGCACTC 5 (0.000128%)

CCGTTCTGGTAAAAAGCTGGAAGATGGCCCTAAATTCTTGAAGTCTGGTGATGCTGCCATTGTTGATAT
GGTTCCTGGCAAGCCCATGTGTGTTGAGAGC 17 (0.000436%)

CCTAAAACCCGCCGGACTTTCTGTAAGAAGTGTGGCAAGCACCAACCCCATAAAGTGACACAGTACAA
GAAGGGCAAGGATTCTCTGTACGCCCAGGGAA 9 (0.000231%)

CCTAAAAGCCAAAATGGGAAAGGAAAAGACTCATATCAACATTGTCGTCATTGGACACGTAGATTCGG
GCAAGTCCACCACTACTGGCCATCTGATCTAT 20 (0.000513%)

CCTAAATTCTTGAAGTCTGGTGATGCTGCCATTGTTGATATGGTTCCTGGCAAGCCCATGTGTGTTGAGA
GCTTCTCAGACTATCCACCTTTGGGTCGCT 31 (0.000795%)

CCTAACAGGGGCCCTCTCAGCCCTCCTAATGACCTCCGGCCTAGCCATGTGATTTCACTTCCACTCCATA
ACGCTCCTCATACTAGGCCTACTAACCAAC 14 (0.000359%)

CCTAATACCTGCCACCCCACTCTTAATCAGTGGTGGAAGAACGGTCTCAGAACTGTTTGTTTCAATTGGC
CATTTAAGTTTAGTAGTAAAAGACTGGTTA 13 (0.000333%)

CCTAATCACAGCAGTCCTACTTCTCCTATCTCTCCCAGTCCTAGCTGCTGGCATCACTATACTACTAACA
GACCGCAACCTCAACACCACCTTCTTCGAC 25 (0.000641%)

CCTAATGACCTCCGGCCTAGCCATGTGATTTCACTTCCACTCCATAACGCTCCTCATACTAGGCCTACTA
ACCAACACACTAACCATATACCAATGATGG 6 (0.000154%)

CCTACCAAAAGCAGCCGACCATCTTTCAAAACAAGAAGAGGGTCCTGCTGGGAGAAACTGGCAAGGAG
AAGCTCCCGCGGTACTACAAGAACATCGGTCT 5 (0.000128%)

CCTACGCACACGAGAACATGCCTCTCGCAAAGGATCTCCTTCATCCCTCTCCAGAAGAGGAGAAGAGG
AAACACAAGAAGAAACGCCTGGTGCAGAGCCC 440 (0.011285%)

CCTCAAAACAAATGATAACCATACACAACACTAAAGGACGAACCTGATCTCTTATACTAGTATCCTTAA
TCATTTTTATTGCCACAACTAACCTCCTCGG 11 (0.000282%)

CCTCACCAAAGCCCATAAAAATAAAAAATTATAACAAACCCTGAGAACCAAAATGAACGAAAATCTGT
TCGCTTCATTCATTGCCCCCACAATCCTAGGC 62 (0.001590%)

CCTCCCTCACCAAAGCCCATAAAAATAAAAAATTATAACAAACCCTGAGAACCAAAATGAACGAAAAT
CTGTTCGCTTCATTCATTGCCCCCACAATCCT 16 (0.000410%)

CCTCGGCTTCCTGCCTCGGAAGCGCAGCAGCAGGCATCGTGGGAAGGTGAAGAGCTTCCCTAAGGATG
ACCCGTCCAAGCCGGTCCACCTCACAGCCTTC 9 (0.000231%)

CCTCGGGCACGCTACGAGAGTACAAGGTAGTGGGTCGCTGCCTGCCCACCCCCAAATGCCACACGCCGC
CCCTCTACCGCATGCGAATCTTTGCGCCTAA 6 (0.000154%)

CCTCTCAGCCCTCCTAATGACCTCCGGCCTAGCCATGTGATTTCACTTCCACTCCATAACGCTCCTCATA
CTAGGCCTACTAACCAACACACTAACCATA 6 (0.000154%)

CCTCTCGCAAAGGATCTCCTTCATCCCTCTCCAGAAGAGGAGAAGAGGAAACACAAGAAGAAACGCCT
GGTGCAGAGCCCCAATTCCTACTTCATGGATG 9 (0.000231%)

CCTCTCTCAAGGATGAGGTTTTGAAGATTATGCCAGTGCAGAAGCAGACCCGTGCCGGCCAGCGCACCA
GGTTCAAGGCATTTGTTGCTATCGGGGACTA 9 (0.000231%)

CCTGAACGCAGGCACATACTTCCTATTCTACACCCTAGTAGGCTCCCTTCCCCTACTCATCGCACTGATT
TACACTCACAACACCCTAGGCTCACTAAAC 43 (0.001103%)

CCTGATCGGCGCACTGCGAGCAGTAGCCCAAACAATCTCATATGAAGTCACCCTAGCCATCATTCTACT
ATCAACATTACTAATAAGTGGCTCCTTTAAC 5 (0.000128%)

CCTGGGGGCCTCTCTCAAGGATGAGGTTTTGAAGATTATGCCAGTGCAGAAGCAGACCCGTGCCGGCCA
GCGCACCAGGTTCAAGGCATTTGTTGCTATC 5 (0.000128%)

CCTTACTACACAATCAAAGACGCCCTCGGCTTACTTCTCTTCATTCTCTCCTTAATGACATTAACACTATT
CTCACCAGACCTCCTAGGCGACCCAGACA 16 (0.000410%)

CCTTCATCCCTCTCCAGAAGAGGAGAAGAGGAAACACAAGAAGAAACGCCTGGTGCAGAGCCCCAATT
CCTACTTCATGGATGTGAAATGCCCAGGATGC 9 (0.000231%)

CCTTCGGTCGTCCCGAATCCGGGTTCATCCGACACCAGCCGCCTCCACCATGCCGCCGAAGTTCGACCC
CAACGAGATCAAAGTCGTATACCTGAGGTGC 8 (0.000205%)

CCTTCTGGCTTACACACTGGGTGTGAAACAACTAATTGTCGGTGTTAACAAAATGGATTCCACTGAGCC
ACCCTACAGCCAGAAGAGATATGAGGAAATT 3 (0.000077%)

CCTTGCGCCTGCCTCTCCAGGATGTCTACAAAATTGGTGGTATTGGTACTGTTCCTGTTGGCCGAGTGGA
GACTGGTGTTCTCAAACCCGGTATGGTGGT 18 (0.000462%)

CCTTGCTCGCCGCAGCCGCCTCCGCCGCGCGCCTCCTCCGCCGCCGCGGACTCCGGCAGCTTTATCGCCA
GAGTCCCTGAACTCTCGCTTTCTTTTTAAT 37 (0.000949%)

CCTTTGTGACTTCCGACCGAAGCAAGAATCGCAAAAGGCATTTCAATGCACCTTCCCACATTCGAAGGA
AGATTATGTCTTCCCCTCTTTCCAAAGAGCT 3 (0.000077%)

CGAAATGCACCATGAAGCTTTGAGTGAAGCTCTTCCTGGGGACAATGTGGGCTTCAATGTCAAGAATGT
GTCTGTCAAGGATGTTCGTCGTGGCAACGTT 14 (0.000359%)

CGAACCTGATCTCTTATACTAGTATCCTTAATCATTTTTATTGCCACAACTAACCTCCTCGGACTCCTGCC
TCACTCATTTACACCAACCACCCAACTAT 117 (0.003001%)

CGAAGATATGCTCATGTGGTGTTGAGGAAAGCAGACATTGACCTCACCAAGAGGGCGGGAGAACTCAC
TGAGGATGAGGTGGAACGTGTGATCACCATTA425 (0.010900%)

CGAAGCAAGAATCGCAAAAGGCATTTCAATGCACCTTCCCACATTCGAAGGAAGATTATGTCTTCCCCT
CTTTCCAAAGAGCTGAGACAGAAGTACAACG 4 (0.000103%)

CGAATCCGGGTTCATCCGACACCAGCCGCCTCCACCATGCCGCCGAAGTTCGACCCCAACGAGATCAAA
GTCGTATACCTGAGGTGCACCGGAGGTGAAG 5 (0.000128%)

CGAATGCGCAGGCTGAAGCGCAAAAGAAGAAAGATGAGGCAGAGGTCCAAGTAAACCGCTAGCTTGTT
GCACCGTGGAGGCCACAGGAGCAGAAACATGG 79 (0.002026%)

CGACCACATCTACAACGTTATCGTCACAGCCCATGCATTTGTAATAATCTTCTTCATAGTAATACCCATC
ATAATCGGAGGCTTTGGCAACTGACTAGTT 277 (0.007104%)

CGACCATCTTTCAAAACAAGAAGAGGGTCCTGCTGGGAGAAACTGGCAAGGAGAAGCTCCCGCGGTAC
TACAAGAACATCGGTCTGGGCTTCAAGACACC 13 (0.000333%)

CGAGAACATGCCTCTCGCAAAGGATCTCCTTCATCCCTCTCCAGAAGAGGAGAAGAGGAAACACAAGA
AGAAACGCCTGGTGCAGAGCCCCAATTCCTAC 135 (0.003462%)

CGAGAAGAAAAAGGGCCGTTCTGCCATCAACGAAGTGGTAACCCGAGAATACACCATCAACATTCACA
AGCGCATCCATGGAGTGGGCTTCAAGAAGCGT 13 (0.000333%)

CGAGAGCATGCCCTTCTGGCTTACACACTGGGTGTGAAACAACTAATTGTCGGTGTTAACAAAATGGAT
TCCACTGAGCCACCCTACAGCCAGAAGAGAT 75 (0.001924%)

CGAGAGTACAAGGTAGTGGGTCGCTGCCTGCCCACCCCCAAATGCCACACGCCGCCCCTCTACCGCATG
CGAATCTTTGCGCCTAATCATGTCGTCGCCA 24 (0.000616%)

CGAGGAGCACCCCGTGCTGCTGACCGAGGCCCCCCTGAACCCCAAGGCCAACCGCGAGAAGATGACCC
AGATCATGTTTGAGACCTTCAACACCCCAGCC 3 (0.000077%)

CGATACGGGATAATCCTATTTATTACCTCAGAAGTTTTTTCTTCGCAGGATTTTTCTGAGCCTTTTACCA
CTCCAGCCTAGCCCCTACCCCCCAATTAG 5 (0.000128%)

CGCAAAAGAAGAAAGATGAGGCAGAGGTCCAAGTAAACCGCTAGCTTGTTGCACCGTGGAGGCCACAG
GAGCAGAAACATGGAATGCCAGACGCTGGGGA 138 (0.003539%)

CGCAAAGAAGGGTGGCGAGAAGAAAAAGGGCCGTTCTGCCATCAACGAAGTGGTAACCCGAGAATAC
ACCATCAACATTCACAAGCGCATCCATGGAGTG 431 (0.011054%)

CGCAAAGGATCTCCTTCATCCCTCTCCAGAAGAGGAGAAGAGGAAACACAAGAAGAAACGCCTGGTGC
AGAGCCCCAATTCCTACTTCATGGATGTGAAA 13 (0.000333%)

CGCAAGCATGGTTAACGTCCCTAAAACCCGCCGGACTTTCTGTAAGAAGTGTGGCAAGCACCAACCCCA
TAAAGTGACACAGTACAAGAAGGGCAAGGAT 35 (0.000898%)

CGCACACGAGAACATGCCTCTCGCAAAGGATCTCCTTCATCCCTCTCCAGAAGAGGAGAAGAGGAAAC
ACAAGAAGAAACGCCTGGTGCAGAGCCCCAAT 119 (0.003052%)

CGCACTGCGAGCAGTAGCCCAAACAATCTCATATGAAGTCACCCTAGCCATCATTCTACTATCAACATT
ACTAATAAGTGGCTCCTTTAACCTCTCCACC 5 (0.000128%)

CGCAGACGCCGCCGCGATGCGCTACGTCGCCTCCTACCTGCTGGCTGCCCTAGGGGGCAACTCCTCCCC
CAGCGCCAAGGACATCAAGAAGATCTTGGAC 12 (0.000308%)

CGCAGCCGCCTCCGCCGCGCGCCTCCTCCGCCGCCGCGGACTCCGGCAGCTTTATCGCCAGAGTCCCTG
AACTCTCGCTTTCTTTTTAATCCCCTGCATC 8 (0.000205%)

CGCAGGCACATACTTCCTATTCTACACCCTAGTAGGCTCCCTTCCCCTACTCATCGCACTGATTTACACT
CACAACACCCTAGGCTCACTAAACATTCTA 18 (0.000462%)

CGCATGATGAAACTTCGGCTCACTCCTTGGCGCCTGCCTGATCCTCCAAATCACCACAGGACTATTCCTA
GCCATGCACTACTCACCAGACGCCTCAACC 31 (0.000795%)

CGCCATGAAGGCCTCGGGCACGCTACGAGAGTACAAGGTAGTGGGTCGCTGCCTGCCCACCCCCAAAT
GCCACACGCCGCCCCTCTACCGCATGCGAATC 9 (0.000231%)

CGCCGCAGACGCCGCCGCGATGCGCTACGTCGCCTCCTACCTGCTGGCTGCCCTAGGGGGCAACTCCTC

CCCCAGCGCCAAGGACATCAAGAAGATCTTG 5 (0.000128%)

CGCCGCAGCCGCCTCCGCCGCGCGCCTCCTCCGCCGCCGCGGACTCCGGCAGCTTTATCGCCAGAGTCC
CTGAACTCTCGCTTTCTTTTAATCCCCTGC 4 (0.000103%)

CGCCGCGATGCGCTACGTCGCCTCCTACCTGCTGGCTGCCCTAGGGGGCAACTCCTCCCCCAGCGCCAA
GGACATCAAGAAGATCTTGGACAGCGTGGGT 17 (0.000436%)

CGCCGTGACCTATTCACCCTCCACTTCCCGTCTCAGAATCTAAACGTGGTCACCTTCGAGTAGAGAGGC
CCGCCCGCCCACCGTGGGCAGTGCCACCCGC 3 (0.000077%)

CGCCGTTCTGGTAAAAAGCTGGAAGATGGCCCTAAATTCTTGAAGTCTGGTGATGCTGCCATTGTTGAT
ATGGTTCCTGGCAAGCCCATGTGTGTTGAGA 213 (0.005463%)

CGCCTCCTTGCTCGCCGCAGCCGCCTCCGCCGCGCGCCTCCTCCGCCGCCGCGGACTCCGGCAGCTTTAT
CGCCAGAGTCCCTGAACTCTCGCTTTCTTT11 (0.000282%)

CGCCTGAACGCAGGCACATACTTCCTATTCTACACCCTAGTAGGCTCCCTTCCCCTACTCATCGCACTGA
TTTACACTCACAACACCCTAGGCTCACTAA 5 (0.000128%)

CGCTACGAGAGTACAAGGTAGTGGGTCGCTGCCTGCCCACCCCCAAATGCCACACGCCGCCCCTCTACC
GCATGCGAATCTTTGCGCCTAATCATGTCGT 10 (0.000256%)

CGCTAGCTTGTTGCACCGTGGAGGCCACAGGAGCAGAAACATGGAATGCCAGACGCTGGGGATGCTGG
TACAAGTTGTGGGACTGCATGCTACTGTCTAG 18 (0.000462%)

CGCTATGGGGCCTCCCTCCGGAAAATGGTGAAGAAAATTGAAATCAGCCAGCACGCCAAGTACACTTG
CTCTTTCTGTGGCAAAACCAAGATGAAGAGAC15 (0.000385%)

CGCTCCCAGACATGGGTCCCTCGGCTTCCTGCCTCGGAAGCGCAGCAGCAGGCATCGTGGGAAGGTGA
AGAGCTTCCCTAAGGATGACCCGTCCAAGCCG9 (0.000231%)

CGCTGCCGTCGCCGCCGCCACCATGCCCAAGAGAAAGGCTGAAGGGGATGCTAAGGGAGATAAAGCAA
AGGTGAAGGACGAACCACAGAGAAGATCCGCG 179 (0.004591%)

CGGAAGTAAAATCTGTCGAAATGCACCATGAAGCTTTGAGTGAAGCTCTTCCTGGGGACAATGTGGGCT
TCAATGTCAAGAATGTGTCTGTCAAGGATGT 90 (0.002308%)

CGGAGAGCACGCCATGAAGGCCTCGGGCACGCTACGAGAGTACAAGGTAGTGGGTCGCTGCCTGCCCA
CCCCCAAATGCCACACGCCGCCCCTCTACCGC 14 (0.000359%)

CGGCGCACTGCGAGCAGTAGCCCAAACAATCTCATATGAAGTCACCCTAGCCATCATTCTACTATCAAC
ATTACTAATAAGTGGCTCCTTTAACCTCTCC 22 (0.000564%)

CGGCTCACTCCTTGGCGCCTGCCTGATCCTCCAAATCACCACAGGACTATTCCTAGCCATGCACTACTCA
CCAGACGCCTCAACCGCCTTTTCATCAATC 12 (0.000308%)

CGGCTTCCTGCCTCGGAAGCGCAGCAGCAGGCATCGTGGGAAGGTGAAGAGCTTCCCTAAGGATGACC
CGTCCAAGCCGGTCCACCTCACAGCCTTCCTG 21 (0.000539%)

CGGGACCCGCTATGGGGCCTCCCTCCGGAAAATGGTGAAGAAAATTGAAATCAGCCAGCACGCCAAGT
ACACTTGCTCTTTCTGTGGCAAAACCAAGATG 12 (0.000308%)

CGGGATAATCCTATTTATTACCTCAGAAGTTTTTTTCTTCGCAGGATTTTTCTGAGCCTTTTACCACTCCA
GCCTAGCCCCTACCCCCCAATTAGGAGGG18 (0.000462%)

CGGGCACGCTACGAGAGTACAAGGTAGTGGGTCGCTGCCTGCCCACCCCCAAATGCCACACGCCGCCC
CTCTACCGCATGCGAATCTTTGCGCCTAATCA 8 (0.000205%)

CGGGTTCATCCGACACCAGCCGCCTCCACCATGCCGCCGAAGTTCGACCCCAACGAGATCAAAGTCGTA
TACCTGAGGTGCACCGGAGGTGAAGTCGGTG 9 (0.000231%)

CGTACTGCTAGGAAGCTCCGTAGTCACCGACGAGACCAGAAGTGGCATGATAAACAGTATAAGAAAGC
TCATTTGGGCACAGCCCTAAAGGCCAACCCTT 11 (0.000282%)

CGTCACCAAGTTGGGCCGCTTGGTCAAGGACATGAAGATCAAGTCCCTGGAGGAGATCTATCTCTTCTC
CCTGCCCATTAAGGAATCAGAGATCATTGAT 75 (0.001924%)

CGTCCCTAAAACCCGCCGGACTTTCTGTAAGAAGTGTGGCAAGCACCAACCCCATAAAGTGACACAGTA
CAAGAAGGGCAAGGATTCTCTGTACGCCCAG 11 (0.000282%)

CGTCCTAATCACAGCAGTCCTACTTCTCCTATCTCTCCCAGTCCTAGCTGCTGGCATCACTATACTACTA
ACAGACCGCAACCTCAACACCACCTTCTTC 6 (0.000154%)

CGTCGTGGCAACGTTGCTGGTGACAGCAAAAATGACCCACCAATGGAAGCAGCTGGCTTCACTGCTCAG
GTGATTATCCTGAACCATCCAGGCCAAATAA 20 (0.000513%)

CGTCTGATCCGTCCTAATCACAGCAGTCCTACTTCTCCTATCTCTCCCAGTCCTAGCTGCTGGCATCACT
ATACTACTAACAGACCGCAACCTCAACACC 3 (0.000077%)

CGTGAAAACTACCCCTAAAAGCCAAAATGGGAAAGGAAAAGACTCATATCAACATTGTCGTCATTGGA
CACGTAGATTCGGGCAAGTCCACCACTACTGG12 (0.000308%)

CGTGACCTATTCACCCTCCACTTCCCGTCTCAGAATCTAAACGTGGTCACCTTCGAGTAGAGAGGCCCG
CCCGCCCACCGTGGGCAGTGCCACCCGCAGA 3 (0.000077%)

CGTGCCTACCAAAAGCAGCCGACCATCTTTCAAAACAAGAAGAGGGTCCTGCTGGGAGAAACTGGCAA
GGAGAAGCTCCCGCGGTACTACAAGAACATCG 7 (0.000180%)

CGTGGCAACGTTGCTGGTGACAGCAAAAATGACCCACCAATGGAAGCAGCTGGCTTCACTGCTCAGGT
GATTATCCTGAACCATCCAGGCCAAATAAGCG10 (0.000256%)

CGTGGTTGCATTGTGGATGCAAATCTGAGCGTTCTCAACTTGGTTATTGTAAAAAAAGGAGAGAAGGAT
ATTCCTGGACTGACTGATACTACAGTGCCTC 11 (0.000282%)

CGTTATCGTCACAGCCCATGCATTTGTAATAATCTTCTTCATAGTAATACCCATCATAATCGGAGGCTTT
GGCAACTGACTAGTTCCCCTAATAATCGGT 11 (0.000282%)

CGTTCTGCCATCAACGAAGTGGTAACCCGAGAATACACCATCAACATTCACAAGCGCATCCATGGAGTG
GGCTTCAAGAAGCGTGCACCTCGGGCACTCA 9 (0.000231%)

CGTTCTGGTAAAAAGCTGGAAGATGGCCCTAAATTCTTGAAGTCTGGTGATGCTGCCATTGTTGATATG
GTTCCTGGCAAGCCCATGTGTGTTGAGAGCT 22 (0.000564%)

CGTTGCTGGTGACAGCAAAAATGACCCACCAATGGAAGCAGCTGGCTTCACTGCTCAGGTGATTATCCT
GAACCATCCAGGCCAAATAAGCGCCGGCTAT 16 (0.000410%)

CTAAAACCCGCCGGACTTTCTGTAAGAAGTGTGGCAAGCACCAACCCCATAAAGTGACACAGTACAAG
AAGGGCAAGGATTCTCTGTACGCCCAGGGAAA 49 (0.001257%)

CTAAAAGCCAAAATGGGAAAGGAAAAGACTCATATCAACATTGTCGTCATTGGACACGTAGATTCGGG
CAAGTCCACCACTACTGGCCATCTGATCTATA 115 (0.002949%)

CTAAAGGACGAACCTGATCTCTTATACTAGTATCCTTAATCATTTTATTGCCACAACTAACCTCCTCGG
ACTCCTGCCTCACTCATTTACACCAACCAC 4 (0.000103%)

CTAAATGAATATTATCCCTAATACCTGCCACCCCACTCTTAATCAGTGGTGGAAGAACGGTCTCAGAAC
TGTTTGTTTCAATTGGCCATTTAAGTTTAGT 13 (0.000333%)

CTAAATTCTTGAAGTCTGGTGATGCTGCCATTGTTGATATGGTTCCTGGCAAGCCCATGTGTGTTGAGAG
CTTCTCAGACTATCCACCTTTGGGTCGCTT 104 (0.002667%)

CTAACACTCACAACAAAACTAACTAATACTAACATCTCAGACGCTCAGGAAATAGAAACCGTCTGAACT
ATCCTGCCCGCCATCATCCTAGTCCTCATCG 436 (0.011182%)

CTAACAGGGGCCCTCTCAGCCCTCCTAATGACCTCCGGCCTAGCCATGTGATTTCACTTCCACTCCATAA
CGCTCCTCATACTAGGCCTACTAACCAACA 45 (0.001154%)

CTAACCGCTAACATTACTGCAGGCCACCTACTCATGCACCTAATTGGAAGCGCCACCCTAGCAATATCA
ACCATTAACCTTCCCTCTACACTTATCATCT 385 (0.009874%)

CTAACCTCAAAACAAATGATAACCATACACAACACTAAAGGACGAACCTGATCTCTTATACTAGTATCC
TTAATCATTTTTATTGCCACAACTAACCTCC 812 (0.020826%)

CTAATACCTGCCACCCCACTCTTAATCAGTGGTGGAAGAACGGTCTCAGAACTGTTTGTTTCAATTGGCC
ATTTAAGTTTAGTAGTAAAAGACTGGTTAA 65 (0.001667%)

CTACAACGTTATCGTCACAGCCCATGCATTTGTAATAATCTTCTTCATAGTAATACCCATCATAATCGGA
GGCTTTGGCAACTGACTAGTTCCCCTAATA 38 (0.000975%)

CTACCCCTAAAAGCCAAAATGGGAAAGGAAAAGACTCATATCAACATTGTCGTCATTGGACACGTAGA
TTCGGGCAAGTCCACCACTACTGGCCATCTGA 23 (0.000590%)

CTACGAGAGTACAAGGTAGTGGGTCGCTGCCTGCCCACCCCCAAATGCCACACGCCGCCCCTCTACCGC
ATGCGAATCTTTGCGCCTAATCATGTCGTCG 3 (0.000077%)

CTACGCACACGAGAACATGCCTCTCGCAAAGGATCTCCTTCATCCCTCTCCAGAAGAGGAGAAGAGGA
AACACAAGAAGAAACGCCTGGTGCAGAGCCCC 29 (0.000744%)

CTACTCCCACTAATAGCTTTTTGATGACTTCTAGCAAGCCTCGCTAACCTCGCCTTACCCCCCACTATTA
ACCTACTGGGAGAACTCTCTGTGCTAGTAA 6 (0.000154%)

CTAGCTTGTTGCACCGTGGAGGCCACAGGAGCAGAAACATGGAATGCCAGACGCTGGGGATGCTGGTA
CAAGTTGTGGGACTGCATGCTACTGTCTAGAG 20 (0.000513%)

CTAGGAAGCTCCGTAGTCACCGACGAGACCAGAAGTGGCATGATAAACAGTATAAGAAAGCTCATTTG
GGCACAGCCCTAAAGGCCAACCCTTTTGGAGG 6 (0.000154%)

CTATCACCCTATAGAAGAACTAATGTTAGTATAAGTAACATGAAAACATTCTCCTCCGCATAAGCCTGC
GTCAGATTAAAACACTGAACTGACAATTAAC 531 (0.013619%)

CTATGGGGCCTCCCTCCGGAAAATGGTGAAGAAAATTGAAATCAGCCAGCACGCCAAGTACACTTGCTC
TTTCTGTGGCAAAACCAAGATGAAGAGACGA 5 (0.000128%)

CTCAAAACAAATGATAACCATACACAACACTAAAGGACGAACCTGATCTCTTATACTAGTATCCTTAAT
CATTTTTATTGCCACAACTAACCTCCTCGGA 28 (0.000718%)

CTCAAGGACTTCAAACTCTACTCCCACTAATAGCTTTTTGATGACTTCTAGCAAGCCTCGCTAACCTCGC
CTTACCCCCCACTATTAACCTACTGGGAGA 15 (0.000385%)

CTCAAGGATGAGGTTTTGAAGATTATGCCAGTGCAGAAGCAGACCCGTGCCGGCCAGCGCACCAGGTT
CAAGGCATTTGTTGCTATCGGGGACTACAATG 12 (0.000308%)

CTCACAACAAAACTAACTAATACTAACATCTCAGACGCTCAGGAAATAGAAACCGTCTGAACTATCCTG

CCCGCCATCATCCTAGTCCTCATCGCCCTCC 32 (0.000821%)

CTCACAGAAAGTTCTCCGCTCCCAGACATGGGTCCCTCGGCTTCCTGCCTCGGAAGCGCAGCAGCAGGC
ATCGTGGGAAGGTGAAGAGCTTCCCTAAGGA 345 (0.008848%)

CTCACCAAAGCCCATAAAAATAAAAAATTATAACAAACCCTGAGAACCAAAATGAACGAAAATCTGTT
CGCTTCATTCATTGCCCCCACAATCCTAGGCC 23 (0.000590%)

CTCACGCAAGCATGGTTAACGTCCCTAAAACCCGCCGGACTTTCTGTAAGAAGTGTGGCAAGCACCAAC
CCCATAAAGTGACACAGTACAAGAAGGGCAA 18 (0.000462%)

CTCAGAAGGCTAAATGAATATTATCCCTAATACCTGCCACCCCACTCTTAATCAGTGGTGGAAGAACGG
TCTCAGAACTGTTTGTTTCAATTGGCCATTT 14 (0.000359%)

CTCAGCCCTCCTAATGACCTCCGGCCTAGCCATGTGATTTCACTTCCACTCCATAACGCTCCTCATACTA
GGCCTACTAACCAACACACTAACCATATAC 21 (0.000539%)

CTCAGGTGATTATCCTGAACCATCCAGGCCAAATAAGCGCCGGCTATGCCCCTGTATTGGATTGCCACA
CGGCTCACATTGCATGCAAGTTTGCTGAGCT 13 (0.000333%)

CTCATATCAACATTGTCGTCATTGGACACGTAGATTCGGGCAAGTCCACCACTACTGGCCATCTGATCTA
TAAATGCGGTGGCATCGACAAAAGAACCAT 279 (0.007156%)

CTCCAGAAGAGGAGAAGAGGAAACACAAGAAGAAACGCCTGGTGCAGAGCCCCAATTCCTACTTCATG
GATGTGAAATGCCCAGGATGCTATAAAATCAC5 (0.000128%)

CTCCAGTCAACGTTACAACGGAAGTAAAATCTGTCGAAATGCACCATGAAGCTTTGAGTGAAGCTCTTC
CTGGGGACAATGTGGGCTTCAATGTCAAGAA 32 (0.000821%)

CTCCCACTAATAGCTTTTTGATGACTTCTAGCAAGCCTCGCTAACCTCGCCTTACCCCCCACTATTAACC
TACTGGGAGAACTCTCTGTGCTAGTAACCA 4 (0.000103%)

CTCCCGAGGAGCACCCCGTGCTGCTGACCGAGGCCCCCCTGAACCCCAAGGCCAACCGCGAGAAGATG
ACCCAGATCATGTTTGAGACCTTCAACACCCC 4 (0.000103%)

CTCCCTCACCAAAGCCCATAAAAATAAAAAATTATAACAAACCCTGAGAACCAAAATGAACGAAAATC
TGTTCGCTTCATTCATTGCCCCCACAATCCTA 231 (0.005925%)

CTCCGCATGATGAAACTTCGGCTCACTCCTTGGCGCCTGCCTGATCCTCCAAATCACCACAGGACTATTC
CTAGCCATGCACTACTCACCAGACGCCTCA 16 (0.000410%)

CTCCGCCGCAGACGCCGCCGCGATGCGCTACGTCGCCTCCTACCTGCTGGCTGCCCTAGGGGGCAACTC
CTCCCCCAGCGCCAAGGACATCAAGAAGATC 526 (0.013491%)

CTCCGCTCCCAGACATGGGTCCCTCGGCTTCCTGCCTCGGAAGCGCAGCAGCAGGCATCGTGGGAAGGT
GAAGAGCTTCCCTAAGGATGACCCGTCCAAG 8 (0.000205%)

CTCCTAATGACCTCCGGCCTAGCCATGTGATTTCACTTCCACTCCATAACGCTCCTCATACTAGGCCTAC
TAACCAACACACTAACCATATACCAATGAT 18 (0.000462%)

CTCCTTCATCCCTCTCCAGAAGAGGAGAAGAGGAAACACAAGAAGAAACGCCTGGTGCAGAGCCCCAA
TTCCTACTTCATGGATGTGAAATGCCCAGGAT 19 (0.000487%)

CTCCTTGCTCGCCGCAGCCGCCTCCGCCGCGCGCCTCCTCCGCCGCCGCGGACTCCGGCAGCTTTATCGC
CAGAGTCCCTGAACTCTCGCTTTCTTTTTA 3 (0.000077%)

CTCCTTGGCGCCTGCCTGATCCTCCAAATCACCACAGGACTATTCCTAGCCATGCACTACTCACCAGACG
CCTCAACCGCCTTTTCATCAATCGCCCACA 8 (0.000205%)

CTCGCAAAGGATCTCCTTCATCCCTCTCCAGAAGAGGAGAAGAGGAAACACAAGAAGAAACGCCTGGT
GCAGAGCCCCAATTCCTACTTCATGGATGTGA 4 (0.000103%)

CTCGCCGCAGCCGCCTCCGCCGCGCGCCTCCTCCGCCGCCGCGGACTCCGGCAGCTTTATCGCCAGAGT
CCCTGAACTCTCGCTTTCTTTTTAATCCCCT 12 (0.000308%)

CTCGGCTTCCTGCCTCGGAAGCGCAGCAGCAGGCATCGTGGGAAGGTGAAGAGCTTCCCTAAGGATGA
CCCGTCCAAGCCGGTCCACCTCACAGCCTTCC 15 (0.000385%)

CTCGGGCACGCTACGAGAGTACAAGGTAGTGGGTCGCTGCCTGCCCACCCCCAAATGCCACACGCCGCC
CCTCTACCGCATGCGAATCTTTGCGCCTAAT 13 (0.000333%)

CTCTACTCCCACTAATAGCTTTTGATGACTTCTAGCAAGCCTCGCTAACCTCGCCTTACCCCCCACTATT
AACCTACTGGGAGAACTCTCTGTGCTAGT 18 (0.000462%)

CTCTCAAGGACTTCAAACTCTACTCCCACTAATAGCTTTTGATGACTTCTAGCAAGCCTCGCTAACCTC
GCCTTACCCCCCACTATTAACCTACTGGGA 480 (0.012311%)

CTCTCAAGGATGAGGTTTTGAAGATTATGCCAGTGCAGAAGCAGACCCGTGCCGGCCAGCGCACCAGG
TTCAAGGCATTTGTTGCTATCGGGGACTACAA 4 (0.000103%)

CTCTCAGCCCTCCTAATGACCTCCGGCCTAGCCATGTGATTTCACTTCCACTCCATAACGCTCCTCATAC
TAGGCCTACTAACCAACACACTAACCATAT 12 (0.000308%)

CTCTCGCAAAGGATCTCCTTCATCCCTCTCCAGAAGAGGAGAAGAGGAAACACAAGAAGAAACGCCTG
GTGCAGAGCCCCAATTCCTACTTCATGGATGT 29 (0.000744%)

CTCTCTCAAGGATGAGGTTTTGAAGATTATGCCAGTGCAGAAGCAGACCCGTGCCGGCCAGCGCACCAG
GTTCAAGGCATTTGTTGCTATCGGGGACTAC 7 (0.000180%)

CTGAACGCAGGCACATACTTCCTATTCTACACCCTAGTAGGCTCCCTTCCCCTACTCATCGCACTGATTT
ACACTCACAACACCCTAGGCTCACTAAACA 20 (0.000513%)

CTGAGATGGGAAAGGGCTCCTTCAAGTATGCCTGGGTCTTGGATAAACTGAAAGCTGAGCGTGAACGT
GGTATCACCATTGATATCTCCTTGTGGAAATT 47 (0.001205%)

CTGAGCGTGCCTACCAAAAGCAGCCGACCATCTTTCAAAACAAGAAGAGGGTCCTGCTGGGAGAAACT
GGCAAGGAGAAGCTCCCGCGGTACTACAAGAA 17 (0.000436%)

CTGATCCGTCCTAATCACAGCAGTCCTACTTCTCCTATCTCTCCCAGTCCTAGCTGCTGGCATCACTATA
CTACTAACAGACCGCAACCTCAACACCACC 15 (0.000385%)

CTGCCATCAACGAAGTGGTAACCCGAGAATACACCATCAACATTCACAAGCGCATCCATGGAGTGGGC
TTCAAGAAGCGTGCACCTCGGGCACTCAAAGA 24 (0.000616%)

CTGCCCAGAAAGCTCAGAAGGCTAAATGAATATTATCCCTAATACCTGCCACCCCACTCTTAATCAGTG
GTGGAAGAACGGTCTCAGAACTGTTTGTTTC 32 (0.000821%)

CTGCTAGGAAGCTCCGTAGTCACCGACGAGACCAGAAGTGGCATGATAAACAGTATAAGAAAGCTCAT
TTGGGCACAGCCCTAAAGGCCAACCCTTTTGG 9 (0.000231%)

CTGCTCAGGTGATTATCCTGAACCATCCAGGCCAAATAAGCGCCGGCTATGCCCCTGTATTGGATTGCC
ACACGGCTCACATTGCATGCAAGTTTGCTGA 9 (0.000231%)

CTGCTGAGATGGGAAAGGGCTCCTTCAAGTATGCCTGGGTCTTGGATAAACTGAAAGCTGAGCGTGAAC
GTGGTATCACCATTGATATCTCCTTGTGGAA 14 (0.000359%)

CTGCTGGAGCTGGCAAGGTCACCAAGTCTGCCCAGAAAGCTCAGAAGGCTAAATGAATATTATCCCTAA
TACCTGCCACCCCACTCTTAATCAGTGGTGG 7 (0.000180%)

CTGGAAGATGGCCCTAAATTCTTGAAGTCTGGTGATGCTGCCATTGTTGATATGGTTCCTGGCAAGCCC
ATGTGTGTTGAGAGCTTCTCAGACTATCCAC 88 (0.002257%)

CTGGAGCTGGCAAGGTCACCAAGTCTGCCCAGAAAGCTCAGAAGGCTAAATGAATATTATCCCTAATAC
CTGCCACCCCACTCTTAATCAGTGGTGGAAG 12 (0.000308%)

CTGGCAAGGTCACCAAGTCTGCCCAGAAAGCTCAGAAGGCTAAATGAATATTATCCCTAATACCTGCCA
CCCCACTCTTAATCAGTGGTGGAAGAACGGT 7 (0.000180%)

CTGGCTTCACTGCTCAGGTGATTATCCTGAACCATCCAGGCCAAATAAGCGCCGGCTATGCCCCTGTATT
GGATTGCCACACGGCTCACATTGCATGCAA 22 (0.000564%)

CTGGGGGCCTCTCTCAAGGATGAGGTTTTGAAGATTATGCCAGTGCAGAAGCAGACCCGTGCCGGCCAG
CGCACCAGGTTCAAGGCATTTGTTGCTATCG 8 (0.000205%)

CTGGTAAAAAGCTGGAAGATGGCCCTAAATTCTTGAAGTCTGGTGATGCTGCCATTGTTGATATGGTTC
CTGGCAAGCCCATGTGTGTTGAGAGCTTCTC 23 (0.000590%)

CTGTCAAGGATGTTCGTCGTGGCAACGTTGCTGGTGACAGCAAAAATGACCCACCAATGGAAGCAGCT
GGCTTCACTGCTCAGGTGATTATCCTGAACCA 6 (0.000154%)

CTGTCGAAATGCACCATGAAGCTTTGAGTGAAGCTCTTCCTGGGGACAATGTGGGCTTCAATGTCAAGA
ATGTGTCTGTCAAGGATGTTCGTCGTGGCAA 33 (0.000846%)

CTTACTACACAATCAAAGACGCCCTCGGCTTACTTCTCTTCATTCTCTCCTTAATGACATTAACACTATTC
TCACCAGACCTCCTAGGCGACCCAGACAA7 (0.000180%)

CTTCAAACTCTACTCCCACTAATAGCTTTTTGATGACTTCTAGCAAGCCTCGCTAACCTCGCCTTACCCC
CCACTATTAACCTACTGGGAGAACTCTCTG 12 (0.000308%)

CTTCAATGTCAAGAATGTGTCTGTCAAGGATGTTCGTCGTGGCAACGTTGCTGGTGACAGCAAAAATGA
CCCACCAATGGAAGCAGCTGGCTTCACTGCT 162 (0.004155%)

CTTCACTGCTCAGGTGATTATCCTGAACCATCCAGGCCAAATAAGCGCCGGCTATGCCCCTGTATTGGA
TTGCCACACGGCTCACATTGCATGCAAGTTT 24 (0.000616%)

CTTCAGAGGAAACAAATGGTCATTGATGTCCTTCACCCCGGGAAGGCGACAGTGCCTAAGACAGAAAT
TCGGGAAAAACTAGCCAAAATGTACAAGACCA 38 (0.000975%)

CTTCATCCCTCTCCAGAAGAGGAGAAGAGGAAACACAAGAAGAAACGCCTGGTGCAGAGCCCCAATTC
CTACTTCATGGATGTGAAATGCCCAGGATGCT 24 (0.000616%)

CTTCCACCCTTACTACACAATCAAAGACGCCCTCGGCTTACTTCTCTTCATTCTCTCCTTAATGACATTAA
CACTATTCTCACCAGACCTCCTAGGCGAC 10 (0.000256%)

CTTCCTGGGGGCCTCTCTCAAGGATGAGGTTTTGAAGATTATGCCAGTGCAGAAGCAGACCCGTGCCGG
CCAGCGCACCAGGTTCAAGGCATTTGTTGCT 7 (0.000180%)

CTTCCTTCGGTCGTCCCGAATCCGGGTTCATCCGACACCAGCCGCCTCCACCATGCCGCCGAAGTTCGAC
CCCAACGAGATCAAAGTCGTATACCTGAGG 42 (0.001077%)

CTTCGATACGGGATAATCCTATTTATTACCTCAGAAGTTTTTTTCTTCGCAGGATTTTTCTGAGCCTTTTA
CCACTCCAGCCTAGCCCCTACCCCCCAAT 964 (0.024724%)

CTTCGGCTCACTCCTTGGCGCCTGCCTGATCCTCCAAATCACCACAGGACTATTCCTAGCCATGCACTAC

TCACCAGACGCCTCAACCGCCTTTTCATCA 38 (0.000975%)

CTTCGGTCGTCCCGAATCCGGGTTCATCCGACACCAGCCGCCTCCACCATGCCGCCGAAGTTCGACCCC
AACGAGATCAAAGTCGTATACCTGAGGTGCA 28 (0.000718%)

CTTCGTACTGCTAGGAAGCTCCGTAGTCACCGACGAGACCAGAAGTGGCATGATAAACAGTATAAGAA
AGCTCATTTGGGCACAGCCCTAAAGGCCAACC907 (0.023262%)

CTTCGTCTGATCCGTCCTAATCACAGCAGTCCTACTTCTCCTATCTCTCCCAGTCCTAGCTGCTGGCATCA
CTATACTACTAACAGACCGCAACCTCAAC 897 (0.023006%)

CTTCTAGCAAGCCTCGCTAACCTCGCCTTACCCCCCACTATTAACCTACTGGGAGAACTCTCTGTGCTAG
TAACCACGTTCTCCTGATCAAATATCACTC 27 (0.000692%)

CTTCTGGCTTACACACTGGGTGTGAAACAACTAATTGTCGGTGTTAACAAAATGGATTCCACTGAGCCA
CCCTACAGCCAGAAGAGATATGAGGAAATTG 26 (0.000667%)

CTTCTTCATAGTAATACCCATCATAATCGGAGGCTTTGGCAACTGACTAGTTCCCCTAATAATCGGTGCC
CCCGATATGGCGTTTCCCCGCATAAACAAC 37 (0.000949%)

CTTGAAGTCTGGTGATGCTGCCATTGTTGATATGGTTCCTGGCAAGCCCATGTGTGTTGAGAGCTTCTCA
GACTATCCACCTTTGGGTCGCTTTGCTGTT271 (0.006950%)

CTTGCTCGCCGCAGCCGCCTCCGCCGCGCGCCTCCTCCGCCGCCGCGGACTCCGGCAGCTTTATCGCCA
GAGTCCCTGAACTCTCGCTTTCTTTTTAATC 16 (0.000410%)

CTTGGCGCCTGCCTGATCCTCCAAATCACCACAGGACTATTCCTAGCCATGCACTACTCACCAGACGCCT
CAACCGCCTTTTCATCAATCGCCCACATCA 11 (0.000282%)

CTTGTTGCACCGTGGAGGCCACAGGAGCAGAAACATGGAATGCCAGACGCTGGGGATGCTGGTACAAG
TTGTGGGACTGCATGCTACTGTCTAGAGCTTG 4 (0.000103%)

CTTTCAAAACAAGAAGAGGGTCCTGCTGGGAGAAACTGGCAAGGAGAAGCTCCCGCGGTACTACAAGA
ACATCGGTCTGGGCTTCAAGACACCCAAGGAG 4 (0.000103%)

CTTTGCTCCAGTCAACGTTACAACGGAAGTAAAATCTGTCGAAATGCACCATGAAGCTTTGAGTGAAGC
TCTTCCTGGGGACAATGTGGGCTTCAATGTC 1107 (0.028392%)

CTTTGTGACTTCCGACCGAAGCAAGAATCGCAAAAGGCATTTCAATGCACCTTCCCACATTCGAAGGAA
GATTATGTCTTCCCCTCTTTCCAAAGAGCTG 10 (0.000256%)

CTTTTTGATGACTTCTAGCAAGCCTCGCTAACCTCGCCTTACCCCCCACTATTAACCTACTGGGAGAACT
CTCTGTGCTAGTAACCACGTTCTCCTGATC 12 (0.000308%)

GAAAAAGGGCCGTTCTGCCATCAACGAAGTGGTAACCCGAGAATACACCATCAACATTCACAAGCGCA
TCCATGGAGTGGGCTTCAAGAAGCGTGCACCT26 (0.000667%)

GAAAACTACCCCTAAAAGCCAAAATGGGAAAGGAAAAGACTCATATCAACATTGTCGTCATTGGACAC
GTAGATTCGGGCAAGTCCACCACTACTGGCCA138 (0.003539%)

GAAAAGACTCATATCAACATTGTCGTCATTGGACACGTAGATTCGGGCAAGTCCACCACTACTGGCCAT
CTGATCTATAAATGCGGTGGCATCGACAAAA 39 (0.001000%)

GAAACAAATGGTCATTGATGTCCTTCACCCCGGGAAGGCGACAGTGCCTAAGACAGAAATTCGGGAAA
AACTAGCCAAAATGTACAAGACCACACCGGAT 3 (0.000077%)

GAAACTTCGGCTCACTCCTTGGCGCCTGCCTGATCCTCCAAATCACCACAGGACTATTCCTAGCCATGCA
CTACTCACCAGACGCCTCAACCGCCTTTTC 13 (0.000333%)

GAAAGATGAGGCAGAGGTCCAAGTAAACCGCTAGCTTGTTGCACCGTGGAGGCCACAGGAGCAGAAAC ATGGAATGCCAGACGCTGGGGATGCTGGTACA 22 (0.000564%)

GAAAGCTCAGAAGGCTAAATGAATATTATCCCTAATACCTGCCACCCCACTCTTAATCAGTGGTGGAAG AACGGTCTCAGAACTGTTTGTTTCAATTGGC 13 (0.000333%)

GAAAGGAAAAGACTCATATCAACATTGTCGTCATTGGACACGTAGATTCGGGCAAGTCCACCACTACTG GCCATCTGATCTATAAATGCGGTGGCATCGA 7 (0.000180%)

GAAAGTTCTCCGCTCCCAGACATGGGTCCCTCGGCTTCCTGCCTCGGAAGCGCAGCAGCAGGCATCGTG GGAAGGTGAAGAGCTTCCCTAAGGATGACCC 45 (0.001154%)

GAAATTTTCACAATGTCCGGAGCCCTTGATGTCCTGCAAATGAAGGAGGAGGATGTCCTTAAGTTCCTT GCAGCAGGAACCCACTTAGGTGGCACCAATC 14 (0.000359%)

GAACACAGGTGTCGTGAAAACTACCCCTAAAAGCCAAAATGGGAAAGGAAAAGACTCATATCAACATT GTCGTCATTGGACACGTAGATTCGGGCAAGTC 60 (0.001539%)

GAACATGCCTCTCGCAAAGGATCTCCTTCATCCCTCTCCAGAAGAGGAGAAGAGGAAACACAAGAAGA AACGCCTGGTGCAGAGCCCCAATTCCTACTTC 9 (0.000231%)

GAACCTGATCTCTTATACTAGTATCCTTAATCATTTTTATTGCCACAACTAACCTCCTCGGACTCCTGCCT CACTCATTTACACCAACCACCCAACTATC 10 (0.000256%)

GAAGAAAAAGGGCCGTTCTGCCATCAACGAAGTGGTAACCCGAGAATACACCATCAACATTCACAAGC GCATCCATGGAGTGGGCTTCAAGAAGCGTGCA 4 (0.000103%)

GAAGAAAGATGAGGCAGAGGTCCAAGTAAACCGCTAGCTTGTTGCACCGTGGAGGCCACAGGAGCAGA AACATGGAATGCCAGACGCTGGGGATGCTGGT 4 (0.000103%)

GAAGAGGATGGAGATGAAGATGAGGAAGCTGAGTCAGCTACGGGCAAGCGGGCAGCTGAAGATGATG AGGATGACGATGTCGATACCAAGAAGCAGAAGA 5 (0.000128%)

GAAGATATGCTCATGTGGTGTTGAGGAAAGCAGACATTGACCTCACCAAGAGGGCGGGAGAACTCACT GAGGATGAGGTGGAACGTGTGATCACCATTAT11 (0.000282%)

GAAGATGAGGAAGCTGAGTCAGCTACGGGCAAGCGGGCAGCTGAAGATGATGAGGATGACGATGTCG ATACCAAGAAGCAGAAGACCGACGAGGATGACT 9 (0.000231%)

GAAGATGGCCCTAAATTCTTGAAGTCTGGTGATGCTGCCATTGTTGATATGGTTCCTGGCAAGCCCATGT GTGTTGAGAGCTTCTCAGACTATCCACCTT 11 (0.000282%)

GAAGCAGCTGGCTTCACTGCTCAGGTGATTATCCTGAACCATCCAGGCCAAATAAGCGCCGGCTATGCC CCTGTATTGGATTGCCACACGGCTCACATTG 22 (0.000564%)

GAAGCTGAGTCAGCTACGGGCAAGCGGGCAGCTGAAGATGATGAGGATGACGATGTCGATACCAAGAA GCAGAAGACCGACGAGGATGACTAGACAGCAA 18 (0.000462%)

GAAGGCCTCGGGCACGCTACGAGAGTACAAGGTAGTGGGTCGCTGCCTGCCCACCCCCAAATGCCACA CGCCGCCCCTCTACCGCATGCGAATCTTTGCG 13 (0.000333%)

GAAGGCTAAATGAATATTATCCCTAATACCTGCCACCCCACTCTTAATCAGTGGTGGAAGAACGGTCTC AGAACTGTTTGTTTCAATTGGCCATTTAAGT 8 (0.000205%)

GAAGGTGATGGTGAGGAAGAGGATGGAGATGAAGATGAGGAAGCTGAGTCAGCTACGGGCAAGCGGG CAGCTGAAGATGATGAGGATGACGATGTCGATA 12 (0.000308%)

GAAGTAAAATCTGTCGAAATGCACCATGAAGCTTTGAGTGAAGCTCTTCCTGGGGACAATGTGGGCTTC
AATGTCAAGAATGTGTCTGTCAAGGATGTTC 5 (0.000128%)

GAAGTCTGGTGATGCTGCCATTGTTGATATGGTTCCTGGCAAGCCCATGTGTGTTGAGAGCTTCTCAGAC
TATCCACCTTTGGGTCGCTTTGCTGTTCGT 15 (0.000385%)

GAAGTTTAATCCCTTTGTGACTTCCGACCGAAGCAAGAATCGCAAAAGGCATTTCAATGCACCTTCCCA
CATTCGAAGGAAGATTATGTCTTCCCCTCTT 10 (0.000256%)

GAATGCGCAGGCTGAAGCGCAAAAGAAGAAAGATGAGGCAGAGGTCCAAGTAAACCGCTAGCTTGTTG
CACCGTGGAGGCCACAGGAGCAGAAACATGGA 27 (0.000692%)

GAATGTGTCTGTCAAGGATGTTCGTCGTGGCAACGTTGCTGGTGACAGCAAAAATGACCCACCAATGGA
AGCAGCTGGCTTCACTGCTCAGGTGATTATC 13 (0.000333%)

GACATGGGTCCCTCGGCTTCCTGCCTCGGAAGCGCAGCAGCAGGCATCGTGGGAAGGTGAAGAGCTTC
CCTAAGGATGACCCGTCCAAGCCGGTCCACCT 3 (0.000077%)

GACATTCAGACTGAGCGTGCCTACCAAAAGCAGCCGACCATCTTTCAAAACAAGAAGAGGGTCCTGCT
GGGAGAAACTGGCAAGGAGAAGCTCCCGCGGT 6 (0.000154%)

GACCCACCAATGGAAGCAGCTGGCTTCACTGCTCAGGTGATTATCCTGAACCATCCAGGCCAAATAAGC
GCCGGCTATGCCCCTGTATTGGATTGCCACA 8 (0.000205%)

GACCCCTAACAGGGGCCCTCTCAGCCCTCCTAATGACCTCCGGCCTAGCCATGTGATTTCACTTCCACTC
CATAACGCTCCTCATACTAGGCCTACTAAC 9 (0.000231%)

GACCCGAGAGCATGCCCTTCTGGCTTACACACTGGGTGTGAAACAACTAATTGTCGGTGTTAACAAAAT
GGATTCCACTGAGCCACCCTACAGCCAGAAG 3 (0.000077%)

GACCCGCTATGGGGCCTCCCTCCGGAAAATGGTGAAGAAAATTGAAATCAGCCAGCACGCCAAGTACA
CTTGCTCTTTCTGTGGCAAAACCAAGATGAAG 3 (0.000077%)

GACCGAAGCAAGAATCGCAAAAGGCATTTCAATGCACCTTCCCACATTCGAAGGAAGATTATGTCTTCC
CCTCTTTCCAAAGAGCTGAGACAGAAGTACA 3 (0.000077%)

GACCTATTCACCCTCCACTTCCCGTCTCAGAATCTAAACGTGGTCACCTTCGAGTAGAGAGGCCCGCCC
GCCCACCGTGGGCAGTGCCACCCGCAGATGA 26 (0.000667%)

GACTGAGCGTGCCTACCAAAAGCAGCCGACCATCTTTCAAAACAAGAAGAGGGTCCTGCTGGGAGAAA
CTGGCAAGGAGAAGCTCCCGCGGTACTACAAG 14 (0.000359%)

GACTTCCGACCGAAGCAAGAATCGCAAAAGGCATTTCAATGCACCTTCCCACATTCGAAGGAAGATTAT
GTCTTCCCCTCTTTCCAAAGAGCTGAGACAG 3 (0.000077%)

GACTTCTAGCAAGCCTCGCTAACCTCGCCTTACCCCCCACTATTAACCTACTGGGAGAACTCTCTGTGCT
AGTAACCACGTTCTCCTGATCAAATATCAC 3 (0.000077%)

GAGAACATGCCTCTCGCAAAGGATCTCCTTCATCCCTCTCCAGAAGAGGAGAAGAGGAAACACAAGAA
GAAACGCCTGGTGCAGAGCCCCAATTCCTACT 16 (0.000410%)

GAGAAGAAAAAGGGCCGTTCTGCCATCAACGAAGTGGTAACCCGAGAATACACCATCAACATTCACAA
GCGCATCCATGGAGTGGGCTTCAAGAAGCGTG 6 (0.000154%)

GAGAGCATGCCCTTCTGGCTTACACACTGGGTGTGAAACAACTAATTGTCGGTGTTAACAAAATGGATT
CCACTGAGCCACCCTACAGCCAGAAGAGATA 17 (0.000436%)

GAGAGTACAAGGTAGTGGGTCGCTGCCTGCCCACCCCCAAATGCCACACGCCGCCCCTCTACCGCATGC

GAATCTTTGCGCCTAATCATGTCGTCGCCAA 13 (0.000333%)

GAGATGGGAAAGGGCTCCTTCAAGTATGCCTGGGTCTTGGATAAACTGAAAGCTGAGCGTGAACGTGG
TATCACCATTGATATCTCCTTGTGGAAATTTG 15 (0.000385%)

GAGCACGCCATGAAGGCCTCGGGCACGCTACGAGAGTACAAGGTAGTGGGTCGCTGCCTGCCCACCCC
CAAATGCCACACGCCGCCCCTCTACCGCATGC 12 (0.000308%)

GAGCATGCCCTTCTGGCTTACACACTGGGTGTGAAACAACTAATTGTCGGTGTTAACAAAATGGATTCC
ACTGAGCCACCCTACAGCCAGAAGAGATATG 9 (0.000231%)

GAGCGTGCCTACCAAAAGCAGCCGACCATCTTTCAAAACAAGAAGAGGGTCCTGCTGGGAGAAACTGG
CAAGGAGAAGCTCCCGCGGTACTACAAGAACA 5 (0.000128%)

GAGCTGGCAAGGTCACCAAGTCTGCCCAGAAAGCTCAGAAGGCTAAATGAATATTATCCCTAATACCTG
CCACCCCACTCTTAATCAGTGGTGGAAGAAC 12 (0.000308%)

GAGGAAACAAATGGTCATTGATGTCCTTCACCCCGGGAAGGCGACAGTGCCTAAGACAGAAATTCGGG
AAAAACTAGCCAAAATGTACAAGACCACACCG 4 (0.000103%)

GAGGAAGAGGATGGAGATGAAGATGAGGAAGCTGAGTCAGCTACGGGCAAGCGGGCAGCTGAAGATG
ATGAGGATGACGATGTCGATACCAAGAAGCAGA 14 (0.000359%)

GAGGAAGCTGAGTCAGCTACGGGCAAGCGGGCAGCTGAAGATGATGAGGATGACGATGTCGATACCAA
GAAGCAGAAGACCGACGAGGATGACTAGACAG 12 (0.000308%)

GAGGCAGAGGTCCAAGTAAACCGCTAGCTTGTTGCACCGTGGAGGCCACAGGAGCAGAAACATGGAAT
GCCAGACGCTGGGGATGCTGGTACAAGTTGTG7 (0.000180%)

GAGGCTGCTGAGATGGGAAAGGGCTCCTTCAAGTATGCCTGGGTCTTGGATAAACTGAAAGCTGAGCG
TGAACGTGGTATCACCATTGATATCTCCTTGT 6 (0.000154%)

GAGGTTTTGAAGATTATGCCAGTGCAGAAGCAGACCCGTGCCGGCCAGCGCACCAGGTTCAAGGCATTT
GTTGCTATCGGGGACTACAATGGCCACGTCG 4 (0.000103%)

GATAACCATACACAACACTAAAGGACGAACCTGATCTCTTATACTAGTATCCTTAATCATTTTTATTGCC
ACAACTAACCTCCTCGGACTCCTGCCTCAC 20 (0.000513%)

GATACATAGGTATGGTCTGAGCTATGATATCAATTGGCTTCCTAGGGTTTATCGTGTGAGCACACCATAT
ATTTACAGTAGGAATAGACGTAGACACACG 6 (0.000154%)

GATACGGGATAATCCTATTTATTACCTCAGAAGTTTTTTTCTTCGCAGGATTTTTCTGAGCCTTTTACCAC
TCCAGCCTAGCCCCTACCCCCCAATTAGG 7 (0.000180%)

GATCCGTCCTAATCACAGCAGTCCTACTTCTCCTATCTCTCCCAGTCCTAGCTGCTGGCATCACTATACT
ACTAACAGACCGCAACCTCAACACCACCTT 5 (0.000128%)

GATCGCCGTTCTGGTAAAAAGCTGGAAGATGGCCCTAAATTCTTGAAGTCTGGTGATGCTGCCATTGTT
GATATGGTTCCTGGCAAGCCCATGTGTGTTG 47 (0.001205%)

GATCGGCGCACTGCGAGCAGTAGCCCAAACAATCTCATATGAAGTCACCCTAGCCATCATTCTACTATC
AACATTACTAATAAGTGGCTCCTTTAACCTC 3 (0.000077%)

GATCTCCTTCATCCCTCTCCAGAAGAGGAGAAGAGGAAACACAAGAAGAAACGCCTGGTGCAGAGCCC
CAATTCCTACTTCATGGATGTGAAATGCCCAG 5 (0.000128%)

GATGAAACTTCGGCTCACTCCTTGGCGCCTGCCTGATCCTCCAAATCACCACAGGACTATTCCTAGCCAT
GCACTACTCACCAGACGCCTCAACCGCCTT 12 (0.000308%)

GATGACTTCTAGCAAGCCTCGCTAACCTCGCCTTACCCCCCACTATTAACCTACTGGGAGAACTCTCTGT
GCTAGTAACCACGTTCTCCTGATCAAATAT 13 (0.000333%)

GATGAGGAAGCTGAGTCAGCTACGGGCAAGCGGCAGCTGAAGATGATGAGGATGACGATGTCGATAC
CAAGAAGCAGAAGACCGACGAGGATGACTAGA 16 (0.000410%)

GATGAGGCAGAGGTCCAAGTAAACCGCTAGCTTGTTGCACCGTGGAGGCCACAGGAGCAGAAACATGG
AATGCCAGACGCTGGGGATGCTGGTACAAGTT7 (0.000180%)

GATGCCCGTCACCAAGTTGGGCCGCTTGGTCAAGGACATGAAGATCAAGTCCCTGGAGGAGATCTATCT
CTTCTCCCTGCCCATTAAGGAATCAGAGATC 24 (0.000616%)

GATGGAGATGAAGATGAGGAAGCTGAGTCAGCTACGGGCAAGCGGCAGCTGAAGATGATGAGGATG
ACGATGTCGATACCAAGAAGCAGAAGACCGACG 3 (0.000077%)

GATGGCCCTAAATTCTTGAAGTCTGGTGATGCTGCCATTGTTGATATGGTTCCTGGCAAGCCCATGTGTG
TTGAGAGCTTCTCAGACTATCCACCTTTGG 24 (0.000616%)

GATGGGAAAGGGCTCCTTCAAGTATGCCTGGGTCTTGGATAAACTGAAAGCTGAGCGTGAACGTGGTAT
CACCATTGATATCTCCTTGTGGAAATTTGAG 11 (0.000282%)

GATTATCCTGAACCATCCAGGCCAAATAAGCGCCGGCTATGCCCCTGTATTGGATTGCCACACGGCTCA
CATTGCATGCAAGTTTGCTGAGCTGAAGGAA 5 (0.000128%)

GCAAAAATGACCCACCAATGGAAGCAGCTGGCTTCACTGCTCAGGTGATTATCCTGAACCATCCAGGCC
AAATAAGCGCCGGCTATGCCCCTGTATTGGA 162 (0.004155%)

GCAAAAGAAGAAAGATGAGGCAGAGGTCCAAGTAAACCGCTAGCTTGTTGCACCGTGGAGGCCACAGG
AGCAGAAACATGGAATGCCAGACGCTGGGGAT 4 (0.000103%)

GCAAAGAAGGGTGGCGAGAAGAAAAAGGGCCGTTCTGCCATCAACGAAGTGGTAACCCGAGAATACA
CCATCAACATTCACAAGCGCATCCATGGAGTGG 4 (0.000103%)

GCAAAGGATCTCCTTCATCCCTCTCCAGAAGAGGAGAAGAGGAAACACAAGAAGAAACGCCTGGTGCA
GAGCCCCAATTCCTACTTCATGGATGTGAAAT 6 (0.000154%)

GCAACGTTGCTGGTGACAGCAAAAATGACCCACCAATGGAAGCAGCTGGCTTCACTGCTCAGGTGATTA
TCCTGAACCATCCAGGCCAAATAAGCGCCGG 8 (0.000205%)

GCAACTTCCTTCGGTCGTCCCGAATCCGGGTTCATCCGACACCAGCCGCCTCCACCATGCCGCCGAAGT
TCGACCCCAACGAGATCAAAGTCGTATACCT 20 (0.000513%)

GCAAGCATGGTTAACGTCCCTAAAACCCGCCGGACTTTCTGTAAGAAGTGTGGCAAGCACCAACCCCAT
AAAGTGACACAGTACAAGAAGGGCAAGGATT 8 (0.000205%)

GCAAGGTCACCAAGTCTGCCCAGAAAGCTCAGAAGGCTAAATGAATATTATCCCTAATACCTGCCACCC
CACTCTTAATCAGTGGTGGAAGAACGGTCTC 12 (0.000308%)

GCAAGTCCACCACTACTGGCCATCTGATCTATAAATGCGGTGGCATCGACAAAAGAACCATTGAAAAAT
TTGAGAAGGAGGCTGCTGAGATGGGAAAGGG 6 (0.000154%)

GCACACGAGAACATGCCTCTCGCAAAGGATCTCCTTCATCCCTCTCCAGAAGAGGAGAAGAGGAAACA
CAAGAAGAAACGCCTGGTGCAGAGCCCCAATT 12 (0.000308%)

GCACATACTTCCTATTCTACACCCTAGTAGGCTCCCTTCCCCTACTCATCGCACTGATTTACACTCACAA
CACCCTAGGCTCACTAAACATTCTACTACT 3 (0.000077%)

GCACCGTGGAGGCCACAGGAGCAGAAACATGGAATGCCAGACGCTGGGGATGCTGGTACAAGTTGTGG
GACTGCATGCTACTGTCTAGAGCTTGTCTCAA 4 (0.000103%)

GCAGACGCCGCCGCGATGCGCTACGTCGCCTCCTACCTGCTGGCTGCCCTAGGGGGCAACTCCTCCCCC
AGCGCCAAGGACATCAAGAAGATCTTGGACA 8 (0.000205%)

GCAGAGGTCCAAGTAAACCGCTAGCTTGTTGCACCGTGGAGGCCACAGGAGCAGAAACATGGAATGCC
AGACGCTGGGGATGCTGGTACAAGTTGTGGGA 15 (0.000385%)

GCAGCCGACCATCTTTCAAAACAAGAAGAGGGTCCTGCTGGGAGAAACTGGCAAGGAGAAGCTCCCGC
GGTACTACAAGAACATCGGTCTGGGCTTCAAG3 (0.000077%)

GCAGCCGCCTCCGCCGCGCGCCTCCTCCGCCGCCGCGGACTCCGGCAGCTTTATCGCCAGAGTCCCTGA
ACTCTCGCTTTCTTTTTAATCCCCTGCATCG 3 (0.000077%)

GCAGCTGGCTTCACTGCTCAGGTGATTATCCTGAACCATCCAGGCCAAATAAGCGCCGGCTATGCCCCT
GTATTGGATTGCCACACGGCTCACATTGCAT 7 (0.000180%)

GCATGATGAAACTTCGGCTCACTCCTTGGCGCCTGCCTGATCCTCCAAATCACCACAGGACTATTCCTAG
CCATGCACTACTCACCAGACGCCTCAACCG 30 (0.000769%)

GCATGCCCTTCTGGCTTACACACTGGGTGTGAAACAACTAATTGTCGGTGTTAACAAAATGGATTCCAC
TGAGCCACCCTACAGCCAGAAGAGATATGAG 13 (0.000333%)

GCATGGTTAACGTCCCTAAAACCCGCCGGACTTTCTGTAAGAAGTGTGGCAAGCACCAACCCCATAAAG
TGACACAGTACAAGAAGGGCAAGGATTCTCT 6 (0.000154%)

GCATTGTGGATGCAAATCTGAGCGTTCTCAACTTGGTTATTGTAAAAAAAGGAGAGAAGGATATTCCTG
GACTGACTGATACTACAGTGCCTCGCCGCCT 4 (0.000103%)

GCCAAAATGGGAAAGGAAAAGACTCATATCAACATTGTCGTCATTGGACACGTAGATTCGGGCAAGTC
CACCACTACTGGCCATCTGATCTATAAATGCG 32 (0.000821%)

GCCATGAAGGCCTCGGGCACGCTACGAGAGTACAAGGTAGTGGGTCGCTGCCTGCCCACCCCCAAATG
CCACACGCCGCCCCTCTACCGCATGCGAATCT 15 (0.000385%)

GCCCAGAAAGCTCAGAAGGCTAAATGAATATTATCCCTAATACCTGCCACCCCACTCTTAATCAGTGGT
GGAAGAACGGTCTCAGAACTGTTTGTTTCAA 10 (0.000256%)

GCCCATAAAAATAAAAAATTATAACAAACCCTGAGAACCAAAATGAACGAAAATCTGTTCGCTTCATTC
ATTGCCCCCACAATCCTAGGCCTACCCGCCG 10 (0.000256%)

GCCCATGACCCCTAACAGGGGCCCTCTCAGCCCTCCTAATGACCTCCGGCCTAGCCATGTGATTTCACTT
CCACTCCATAACGCTCCTCATACTAGGCCT 29 (0.000744%)

GCCCGTCACCAAGTTGGGCCGCTTGGTCAAGGACATGAAGATCAAGTCCCTGGAGGAGATCTATCTCTT
CTCCCTGCCCATTAAGGAATCAGAGATCATT 19 (0.000487%)

GCCCTAAATTCTTGAAGTCTGGTGATGCTGCCATTGTTGATATGGTTCCTGGCAAGCCCATGTGTGTTGA
GAGCTTCTCAGACTATCCACCTTTGGGTCG 7 (0.000180%)

GCCCTCCTAATGACCTCCGGCCTAGCCATGTGATTTCACTTCCACTCCATAACGCTCCTCATACTAGGCC
TACTAACCAACACACTAACCATATACCAAT 9 (0.000231%)

GCCCTCTCAGCCCTCCTAATGACCTCCGGCCTAGCCATGTGATTTCACTTCCACTCCATAACGCTCCTCA
TACTAGGCCTACTAACCAACACACTAACCA 24 (0.000616%)

GCCCTTCTGGCTTACACACTGGGTGTGAAACAACTAATTGTCGGTGTTAACAAAATGGATTCCACTGAG

CCACCCTACAGCCAGAAGAGATATGAGGAAA 19 (0.000487%)

GCCCTTGCGCCTGCCTCTCCAGGATGTCTACAAAATTGGTGGTATTGGTACTGTTCCTGTTGGCCGAGTG
GAGACTGGTGTTCTCAAACCCGGTATGGTG 5 (0.000128%)

GCCGACCATCTTTCAAAACAAGAAGAGGGTCCTGCTGGGAGAAACTGGCAAGGAGAAGCTCCCGCGGT
ACTACAAGAACATCGGTCTGGGCTTCAAGACA 8 (0.000205%)

GCCGCAGACGCCGCCGCGATGCGCTACGTCGCCTCCTACCTGCTGGCTGCCCTAGGGGGCAACTCCTCC
CCCAGCGCCAAGGACATCAAGAAGATCTTGG 9 (0.000231%)

GCCGCAGCCGCCTCCGCCGCGCGCCTCCTCCGCCGCCGCGGACTCCGGCAGCTTTATCGCCAGAGTCCC
TGAACTCTCGCTTTCTTTTTAATCCCCTGCA 19 (0.000487%)

GCCGCCGCGATGCGCTACGTCGCCTCCTACCTGCTGGCTGCCCTAGGGGGCAACTCCTCCCCCAGCGCC
AAGGACATCAAGAAGATCTTGGACAGCGTGG 12 (0.000308%)

GCCGCCGTGACCTATTCACCCTCCACTTCCCGTCTCAGAATCTAAACGTGGTCACCTTCGAGTAGAGAG
GCCCGCCCGCCCACCGTGGGCAGTGCCACCC 11 (0.000282%)

GCCGCCTCCGCCGCGCGCCTCCTCCGCCGCCGCGGACTCCGGCAGCTTTATCGCCAGAGTCCCTGAACT
CTCGCTTTCTTTTTAATCCCCTGCATCGGAT 13 (0.000333%)

GCCGCGATGCGCTACGTCGCCTCCTACCTGCTGGCTGCCCTAGGGGGCAACTCCTCCCCCAGCGCCAAG
GACATCAAGAAGATCTTGGACAGCGTGGGTA 3 (0.000077%)

GCCGCTTGGTCAAGGACATGAAGATCAAGTCCCTGGAGGAGATCTATCTCTTCTCCCTGCCCATTAAGG
AATCAGAGATCATTGATTTCTTCCTGGGGGC 16 (0.000410%)

GCCGTGACCTATTCACCCTCCACTTCCCGTCTCAGAATCTAAACGTGGTCACCTTCGAGTAGAGAGGCC
CGCCCGCCCACCGTGGGCAGTGCCACCCGCA 7 (0.000180%)

GCCGTTCTGCCATCAACGAAGTGGTAACCCGAGAATACACCATCAACATTCACAAGCGCATCCATGGAG
TGGGCTTCAAGAAGCGTGCACCTCGGGCACT 9 (0.000231%)

GCCGTTCTGGTAAAAAGCTGGAAGATGGCCCTAAATTCTTGAAGTCTGGTGATGCTGCCATTGTTGATA
TGGTTCCTGGCAAGCCCATGTGTGTTGAGAG 48 (0.001231%)

GCCTACCAAAAGCAGCCGACCATCTTTCAAAACAAGAAGAGGGTCCTGCTGGGAGAAACTGGCAAGGA
GAAGCTCCCGCGGTACTACAAGAACATCGGTC 16 (0.000410%)

GCCTCCTTGCTCGCCGCAGCCGCCTCCGCCGCGCGCCTCCTCCGCCGCCGCGGACTCCGGCAGCTTTATC
GCCAGAGTCCCTGAACTCTCGCTTTCTTTT 8 (0.000205%)

GCCTCGGGCACGCTACGAGAGTACAAGGTAGTGGGTCGCTGCCTGCCCACCCCCAAATGCCACACGCC
GCCCCTCTACCGCATGCGAATCTTTGCGCCTA 31 (0.000795%)

GCCTCTCGCAAAGGATCTCCTTCATCCCTCTCCAGAAGAGGAGAAGAGGAAACACAAGAAGAAACGCC
TGGTGCAGAGCCCCAATTCCTACTTCATGGAT 9 (0.000231%)

GCCTGAACGCAGGCACATACTTCCTATTCTACACCCTAGTAGGCTCCCTTCCCCTACTCATCGCACTGAT
TTACACTCACAACACCCTAGGCTCACTAAA 13 (0.000333%)

GCGAATGCGCAGGCTGAAGCGCAAAAGAAGAAAGATGAGGCAGAGGTCCAAGTAAACCGCTAGCTTG
TTGCACCGTGGAGGCCACAGGAGCAGAAACATG 1128 (0.028930%)

GCGAGAAGAAAAAGGGCCGTTCTGCCATCAACGAAGTGGTAACCCGAGAATACACCATCAACATTCAC
AAGCGCATCCATGGAGTGGGCTTCAAGAAGCG 7 (0.000180%)

GCGCACTGCGAGCAGTAGCCCAAACAATCTCATATGAAGTCACCCTAGCCATCATTCTACTATCAACAT
TACTAATAAGTGGCTCCTTTAACCTCTCCAC 3 (0.000077%)

GCGCAGGCTGAAGCGCAAAAGAAGAAAGATGAGGCAGAGGTCCAAGTAAACCGCTAGCTTGTTGCACC
GTGGAGGCCACAGGAGCAGAAACATGGAATGC 18 (0.000462%)

GCGGAGAGCACGCCATGAAGGCCTCGGGCACGCTACGAGAGTACAAGGTAGTGGGTCGCTGCCTGCCC
ACCCCCAAATGCCACACGCCGCCCCTCTACCG 473 (0.012131%)

GCGTCCCATGCCTCCATCTAGAAGAGATTATGATGATATGAGCCCTCGTCGAGGACCACCTCCCCCTCC
TCCCGGACGAGGCGGCCGGGGTGGTAGCAGA 4 (0.000103%)

GCTAAATGAATATTATCCCTAATACCTGCCACCCCACTCTTAATCAGTGGTGGAAGAACGGTCTCAGAA
CTGTTTGTTTCAATTGGCCATTTAAGTTTAG 39 (0.001000%)

GCTAGCTTGTTGCACCGTGGAGGCCACAGGAGCAGAAACATGGAATGCCAGACGCTGGGGATGCTGGT
ACAAGTTGTGGGACTGCATGCTACTGTCTAGA 22 (0.000564%)

GCTAGGAAGCTCCGTAGTCACCGACGAGACCAGAAGTGGCATGATAAACAGTATAAGAAAGCTCATTT
GGGCACAGCCCTAAAGGCCAACCCTTTTGGAG 6 (0.000154%)

GCTATGGGGCCTCCCTCCGGAAAATGGTGAAGAAAATTGAAATCAGCCAGCACGCCAAGTACACTTGC
TCTTTCTGTGGCAAAACCAAGATGAAGAGACG4 (0.000103%)

GCTCACGCAAGCATGGTTAACGTCCCTAAAACCCGCCGGACTTTCTGTAAGAAGTGTGGCAAGCACCAA
CCCCATAAAGTGACACAGTACAAGAAGGGCA 296 (0.007592%)

GCTCACTCCTTGGCGCCTGCCTGATCCTCCAAATCACCACAGGACTATTCCTAGCCATGCACTACTCACC
AGACGCCTCAACCGCCTTTTCATCAATCGC 3 (0.000077%)

GCTCAGAAGGCTAAATGAATATTATCCCTAATACCTGCCACCCCACTCTTAATCAGTGGTGGAAGAACG
GTCTCAGAACTGTTTGTTTCAATTGGCCATT 19 (0.000487%)

GCTCAGGTGATTATCCTGAACCATCCAGGCCAAATAAGCGCCGGCTATGCCCCTGTATTGGATTGCCAC
ACGGCTCACATTGCATGCAAGTTTGCTGAGC 6 (0.000154%)

GCTCCAGTCAACGTTACAACGGAAGTAAAATCTGTCGAAATGCACCATGAAGCTTTGAGTGAAGCTCTT
CCTGGGGACAATGTGGGCTTCAATGTCAAGA 7 (0.000180%)

GCTCGCCGCAGCCGCCTCCGCCGCGCGCCTCCTCCGCCGCCGCGGACTCCGGCAGCTTTATCGCCAGAG
TCCCTGAACTCTCGCTTTCTTTTTAATCCCC 6 (0.000154%)

GCTGAGATGGGAAAGGGCTCCTTCAAGTATGCCTGGGTCTTGGATAAACTGAAAGCTGAGCGTGAACG
TGGTATCACCATTGATATCTCCTTGTGGAAAT 22 (0.000564%)

GCTGCCGTCGCCGCCGCCACCATGCCCAAGAGAAAGGCTGAAGGGGATGCTAAGGGAGATAAAGCAAA
GGTGAAGGACGAACCACAGAGAAGATCCGCGA 4 (0.000103%)

GCTGCGTGTGGCTCCCGAGGAGCACCCCGTGCTGCTGACCGAGGCCCCCCTGAACCCCAAGGCCAACCG
CGAGAAGATGACCCAGATCATGTTTGAGACC 207 (0.005309%)

GCTGCTGAGATGGGAAAGGGCTCCTTCAAGTATGCCTGGGTCTTGGATAAACTGAAAGCTGAGCGTGA
ACGTGGTATCACCATTGATATCTCCTTGTGGA 19 (0.000487%)

GCTGCTGGAGCTGGCAAGGTCACCAAGTCTGCCCAGAAAGCTCAGAAGGCTAAATGAATATTATCCCTA
ATACCTGCCACCCCACTCTTAATCAGTGGTG 20 (0.000513%)

GCTGGAAGATGGCCCTAAATTCTTGAAGTCTGGTGATGCTGCCATTGTTGATATGGTTCCTGGCAAGCC
CATGTGTGTTGAGAGCTTCTCAGACTATCCA 12 (0.000308%)

GCTGGAGCTGGCAAGGTCACCAAGTCTGCCCAGAAAGCTCAGAAGGCTAAATGAATATTATCCCTAAT
ACCTGCCACCCCACTCTTAATCAGTGGTGGAA 25 (0.000641%)

GCTGGCAAGGTCACCAAGTCTGCCCAGAAAGCTCAGAAGGCTAAATGAATATTATCCCTAATACCTGCC
ACCCCACTCTTAATCAGTGGTGGAAGAACGG 4 (0.000103%)

GCTGGCTTCACTGCTCAGGTGATTATCCTGAACCATCCAGGCCAAATAAGCGCCGGCTATGCCCCTGTA
TTGGATTGCCACACGGCTCACATTGCATGCA 70 (0.001795%)

GCTGGTGACAGCAAAAATGACCCACCAATGGAAGCAGCTGGCTTCACTGCTCAGGTGATTATCCTGAAC
CATCCAGGCCAAATAAGCGCCGGCTATGCCC 17 (0.000436%)

GCTTCAATGTCAAGAATGTGTCTGTCAAGGATGTTCGTCGTGGCAACGTTGCTGGTGACAGCAAAAATG
ACCCACCAATGGAAGCAGCTGGCTTCACTGC 773 (0.019825%)

GCTTCACTGCTCAGGTGATTATCCTGAACCATCCAGGCCAAATAAGCGCCGGCTATGCCCCTGTATTGG
ATTGCCACACGGCTCACATTGCATGCAAGTT 7 (0.000180%)

GCTTCCTGCCTCGGAAGCGCAGCAGCAGGCATCGTGGGAAGGTGAAGAGCTTCCCTAAGGATGACCCG
TCCAAGCCGGTCCACCTCACAGCCTTCCTGGG 4 (0.000103%)

GCTTGTTGCACCGTGGAGGCCACAGGAGCAGAAACATGGAATGCCAGACGCTGGGGATGCTGGTACAA
GTTGTGGGACTGCATGCTACTGTCTAGAGCTT 10 (0.000256%)

GGAAAAGACTCATATCAACATTGTCGTCATTGGACACGTAGATTCGGGCAAGTCCACCACTACTGGCCA
TCTGATCTATAAATGCGGTGGCATCGACAAA 84 (0.002154%)

GGAAACAAATGGTCATTGATGTCCTTCACCCCGGGAAGGCGACAGTGCCTAAGACAGAAATTCGGGAA
AAACTAGCCAAAATGTACAAGACCACACCGGA 3 (0.000077%)

GGAAAGGAAAAGACTCATATCAACATTGTCGTCATTGGACACGTAGATTCGGGCAAGTCCACCACTACT
GGCCATCTGATCTATAAATGCGGTGGCATCG 25 (0.000641%)

GGAAAGGGCTCCTTCAAGTATGCCTGGGTCTTGGATAAACTGAAAGCTGAGCGTGAACGTGGTATCACC
ATTGATATCTCCTTGTGGAAATTTGAGACCA 176 (0.004514%)

GGAAATTTTCACAATGTCCGGAGCCCTTGATGTCCTGCAAATGAAGGAGGAGGATGTCCTTAAGTTCCT
TGCAGCAGGAACCCACTTAGGTGGCACCAAT 14 (0.000359%)

GGAAGAGGATGGAGATGAAGATGAGGAAGCTGAGTCAGCTACGGGCAAGCGGGCAGCTGAAGATGAT
GAGGATGACGATGTCGATACCAAGAAGCAGAAG 35 (0.000898%)

GGAAGATGGCCCTAAATTCTTGAAGTCTGGTGATGCTGCCATTGTTGATATGGTTCCTGGCAAGCCCAT
GTGTGTTGAGAGCTTCTCAGACTATCCACCT 166 (0.004257%)

GGAAGCAGCTGGCTTCACTGCTCAGGTGATTATCCTGAACCATCCAGGCCAAATAAGCGCCGGCTATGC
CCCTGTATTGGATTGCCACACGGCTCACATT 23 (0.000590%)

GGAAGCTCCGTAGTCACCGACGAGACCAGAAGTGGCATGATAAACAGTATAAGAAAGCTCATTTGGGC
ACAGCCCTAAAGGCCAACCCTTTTGGAGGTGC 14 (0.000359%)

GGAAGCTGAGTCAGCTACGGGCAAGCGGGCAGCTGAAGATGATGAGGATGACGATGTCGATACCAAGA
AGCAGAAGACCGACGAGGATGACTAGACAGCA 40 (0.001026%)

GGAAGTAAAATCTGTCGAAATGCACCATGAAGCTTTGAGTGAAGCTCTTCCTGGGGACAATGTGGGCTT

CAATGTCAAGAATGTGTCTGTCAAGGATGTT 18 (0.000462%)

GGACATTCAGACTGAGCGTGCCTACCAAAAGCAGCCGACCATCTTTCAAAACAAGAAGAGGGTCCTGC
TGGGAGAAACTGGCAAGGAGAAGCTCCCGCGG 1025 (0.026289%)

GGACCCGCTATGGGGCCTCCCTCCGGAAAATGGTGAAGAAAATTGAAATCAGCCAGCACGCCAAGTAC
ACTTGCTCTTTCTGTGGCAAAACCAAGATGAA 14 (0.000359%)

GGACTTCAAACTCTACTCCCACTAATAGCTTTTTGATGACTTCTAGCAAGCCTCGCTAACCTCGCCTTAC
CCCCCACTATTAACCTACTGGGAGAACTCT 9 (0.000231%)

GGAGAGCACGCCATGAAGGCCTCGGGCACGCTACGAGAGTACAAGGTAGTGGGTCGCTGCCTGCCCAC
CCCCAAATGCCACACGCCGCCCCTCTACCGCA 18 (0.000462%)

GGAGATGAAGATGAGGAAGCTGAGTCAGCTACGGGCAAGCGGGCAGCTGAAGATGATGAGGATGACG
ATGTCGATACCAAGAAGCAGAAGACCGACGAGG 8 (0.000205%)

GGAGCACCCCGTGCTGCTGACCGAGGCCCCCCTGAACCCCAAGGCCAACCGCGAGAAGATGACCCAGA
TCATGTTTGAGACCTTCAACACCCCAGCCATG 7 (0.000180%)

GGAGCTGGCAAGGTCACCAAGTCTGCCCAGAAAGCTCAGAAGGCTAAATGAATATTATCCCTAATACCT
GCCACCCCACTCTTAATCAGTGGTGGAAGAA 7 (0.000180%)

GGAGGCTGCTGAGATGGGAAAGGGCTCCTTCAAGTATGCCTGGGTCTTGGATAAACTGAAAGCTGAGC
GTGAACGTGGTATCACCATTGATATCTCCTTG 850 (0.021800%)

GGATACATAGGTATGGTCTGAGCTATGATATCAATTGGCTTCCTAGGGTTTATCGTGTGAGCACACCAT
ATATTTACAGTAGGAATAGACGTAGACACAC 8 (0.000205%)

GGATCTCCTTCATCCCTCTCCAGAAGAGGAGAAGAGGAAACACAAGAAGAAACGCCTGGTGCAGAGCC
CCAATTCCTACTTCATGGATGTGAAATGCCCA 5 (0.000128%)

GGATGCCCGTCACCAAGTTGGGCCGCTTGGTCAAGGACATGAAGATCAAGTCCCTGGAGGAGATCTATC
TCTTCTCCCTGCCCATTAAGGAATCAGAGAT 55 (0.001411%)

GGATGGAGATGAAGATGAGGAAGCTGAGTCAGCTACGGGCAAGCGGGCAGCTGAAGATGATGAGGAT
GACGATGTCGATACCAAGAAGCAGAAGACCGAC 6 (0.000154%)

GGATGTTCGTCGTGGCAACGTTGCTGGTGACAGCAAAAATGACCCACCAATGGAAGCAGCTGGCTTCAC
TGCTCAGGTGATTATCCTGAACCATCCAGGC 27 (0.000692%)

GGCAACGTTGCTGGTGACAGCAAAAATGACCCACCAATGGAAGCAGCTGGCTTCACTGCTCAGGTGATT
ATCCTGAACCATCCAGGCCAAATAAGCGCCG 31 (0.000795%)

GGCAAGGTCACCAAGTCTGCCCAGAAAGCTCAGAAGGCTAAATGAATATTATCCCTAATACCTGCCACC
CCACTCTTAATCAGTGGTGGAAGAACGGTCT 42 (0.001077%)

GGCAAGTCCACCACTACTGGCCATCTGATCTATAAATGCGGTGGCATCGACAAAAGAACCATTGAAAA
ATTTGAGAAGGAGGCTGCTGAGATGGGAAAGG 547 (0.014029%)

GGCACATACTTCCTATTCTACACCCTAGTAGGCTCCCTTCCCCTACTCATCGCACTGATTTACACTCACA
ACACCCTAGGCTCACTAAACATTCTACTAC 5 (0.000128%)

GGCAGAGGTCCAAGTAAACCGCTAGCTTGTTGCACCGTGGAGGCCACAGGAGCAGAAACATGGAATGC
CAGACGCTGGGGATGCTGGTACAAGTTGTGGG 37 (0.000949%)

GGCCCTAAATTCTTGAAGTCTGGTGATGCTGCCATTGTTGATATGGTTCCTGGCAAGCCCATGTGTGTTG
AGAGCTTCTCAGACTATCCACCTTTGGGTC 5 (0.000128%)

GGCCCTCTCAGCCCTCCTAATGACCTCCGGCCTAGCCATGTGATTTCACTTCCACTCCATAACGCTCCTC
ATACTAGGCCTACTAACCAACACACTAACC 9 (0.000231%)

GGCCGCCGTGACCTATTCACCCTCCACTTCCCGTCTCAGAATCTAAACGTGGTCACCTTCGAGTAGAGA
GGCCCGCCCGCCCACCGTGGGCAGTGCCACC 4 (0.000103%)

GGCCGCTTGGTCAAGGACATGAAGATCAAGTCCCTGGAGGAGATCTATCTCTTCTCCCTGCCCATTAAG
GAATCAGAGATCATTGATTTCTTCCTGGGGG 19 (0.000487%)

GGCCGTTCTGCCATCAACGAAGTGGTAACCCGAGAATACACCATCAACATTCACAAGCGCATCCATGGA
GTGGGCTTCAAGAAGCGTGCACCTCGGGCAC 34 (0.000872%)

GGCCTCGGGCACGCTACGAGAGTACAAGGTAGTGGGTCGCTGCCTGCCCACCCCCAAATGCCACACGC
CGCCCCTCTACCGCATGCGAATCTTTGCGCCT 3 (0.000077%)

GGCCTCTCTCAAGGATGAGGTTTTGAAGATTATGCCAGTGCAGAAGCAGACCCGTGCCGGCCAGCGCAC
CAGGTTCAAGGCATTTGTTGCTATCGGGGAC 6 (0.000154%)

GGCGAGAAGAAAAAGGGCCGTTCTGCCATCAACGAAGTGGTAACCCGAGAATACACCATCAACATTCA
CAAGCGCATCCATGGAGTGGGCTTCAAGAAGC 3 (0.000077%)

GGCGCACTGCGAGCAGTAGCCCAAACAATCTCATATGAAGTCACCCTAGCCATCATTCTACTATCAACA
TTACTAATAAGTGGCTCCTTTAACCTCTCCA 6 (0.000154%)

GGCTAAATGAATATTATCCCTAATACCTGCCACCCCACTCTTAATCAGTGGTGGAAGAACGGTCTCAGA
ACTGTTTGTTTCAATTGGCCATTTAAGTTTA 22 (0.000564%)

GGCTCACTCCTTGGCGCCTGCCTGATCCTCCAAATCACCACAGGACTATTCCTAGCCATGCACTACTCAC
CAGACGCCTCAACCGCCTTTTCATCAATCG 7 (0.000180%)

GGCTGCTGAGATGGGAAAGGGCTCCTTCAAGTATGCCTGGGTCTTGGATAAACTGAAAGCTGAGCGTG
AACGTGGTATCACCATTGATATCTCCTTGTGG 28 (0.000718%)

GGCTGCTGGAGCTGGCAAGGTCACCAAGTCTGCCCAGAAAGCTCAGAAGGCTAAATGAATATTATCCCT
AATACCTGCCACCCCACTCTTAATCAGTGGT 93 (0.002385%)

GGCTTCACTGCTCAGGTGATTATCCTGAACCATCCAGGCCAAATAAGCGCCGGCTATGCCCCTGTATTG
GATTGCCACACGGCTCACATTGCATGCAAGT 11 (0.000282%)

GGCTTCCTGCCTCGGAAGCGCAGCAGCAGGCATCGTGGGAAGGTGAAGAGCTTCCCTAAGGATGACCC
GTCCAAGCCGGTCCACCTCACAGCCTTCCTGG 4 (0.000103%)

GGGAAAGGAAAAGACTCATATCAACATTGTCGTCATTGGACACGTAGATTCGGGCAAGTCCACCACTA
CTGGCCATCTGATCTATAAATGCGGTGGCATC 39 (0.001000%)

GGGAAAGGGCTCCTTCAAGTATGCCTGGGTCTTGGATAAACTGAAAGCTGAGCGTGAACGTGGTATCAC
CATTGATATCTCCTTGTGGAAATTTGAGACC 90 (0.002308%)

GGGAAATTTTCACAATGTCCGGAGCCCTTGATGTCCTGCAAATGAAGGAGGAGGATGTCCTTAAGTTCC
TTGCAGCAGGAACCCACTTAGGTGGCACCAA 13 (0.000333%)

GGGACCCGCTATGGGGCCTCCCTCCGGAAAATGGTGAAGAAAATTGAAATCAGCCAGCACGCCAAGTA
CACTTGCTCTTTCTGTGGCAAAACCAAGATGA 20 (0.000513%)

GGGCACGCTACGAGAGTACAAGGTAGTGGGTCGCTGCCTGCCCACCCCCAAATGCCACACGCCGCCCCT
CTACCGCATGCGAATCTTTGCGCCTAATCAT 22 (0.000564%)

GGGCCCTCTCAGCCCTCCTAATGACCTCCGGCCTAGCCATGTGATTTCACTTCCACTCCATAACGCTCCT
CATACTAGGCCTACTAACCAACACACTAAC 19 (0.000487%)

GGGCCGTTCTGCCATCAACGAAGTGGTAACCCGAGAATACACCATCAACATTCACAAGCGCATCCATGG
AGTGGGCTTCAAGAAGCGTGCACCTCGGGCA 8 (0.000205%)

GGGCGTCCCATGCCTCCATCTAGAAGAGATTATGATGATATGAGCCCTCGTCGAGGACCACCTCCCCCT
CCTCCCGGACGAGGCGGCCGGGGTGGTAGCA 6 (0.000154%)

GGGGCCCTCTCAGCCCTCCTAATGACCTCCGGCCTAGCCATGTGATTTCACTTCCACTCCATAACGCTCC
TCATACTAGGCCTACTAACCAACACACTAA 27 (0.000692%)

GGGGCCTCTCTCAAGGATGAGGTTTTGAAGATTATGCCAGTGCAGAAGCAGACCCGTGCCGGCCAGCG
CACCAGGTTCAAGGCATTTGTTGCTATCGGGG 7 (0.000180%)

GGGGGCCTCTCTCAAGGATGAGGTTTTGAAGATTATGCCAGTGCAGAAGCAGACCCGTGCCGGCCAGC
GCACCAGGTTCAAGGCATTTGTTGCTATCGGG 9 (0.000231%)

GGGGGGGGGGGGGGGGGGGGGGGGGGGGGGGGGGGGGGGGGGGGGGGGGGGGGGGGGGGGGGGGGG
GGGGGGGGGGGGGGGGGGGGGGGGGGGGGGGGGG 6947 (0.178173%)

GGGGGGGGGGGGGGGGGGGGGGGGGGGGGGGGGGGGGGGGGGGGGGGGGGGGGGGGGGGGGGGGGG
GGGGGGGGGGGGGGGGGGGGGGGGGGGGGGGGGGGGGGGGGGGGGGGGGGGGGGGGGGGGGGGGGG
GGGGGGGGGGGGGGGGGG 5572 (0.212932%)

GGGTCCCTCGGCTTCCTGCCTCGGAAGCGCAGCAGCAGGCATCGTGGGAAGGTGAAGAGCTTCCCTAA
GGATGACCCGTCCAAGCCGGTCCACCTCACAG3 (0.000077%)

GGGTGGGCGTCCCATGCCTCCATCTAGAAGAGATTATGATGATATGAGCCCTCGTCGAGGACCACCTCC
CCCTCCTCCCGGACGAGGCGGCCGGGGTGGT 63 (0.001616%)

GGTAAAAAGCTGGAAGATGGCCCTAAATTCTTGAAGTCTGGTGATGCTGCCATTGTTGATATGGTTCCT
GGCAAGCCCATGTGTGTTGAGAGCTTCTCAG 15 (0.000385%)

GGTATGGTCTGAGCTATGATATCAATTGGCTTCCTAGGGTTTATCGTGTGAGCACACCATATATTTACAG
TAGGAATAGACGTAGACACACGAGCATATT 3 (0.000077%)

GGTCACCAAGTCTGCCCAGAAAGCTCAGAAGGCTAAATGAATATTATCCCTAATACCTGCCACCCCACT
CTTAATCAGTGGTGGAAGAACGGTCTCAGAA 8 (0.000205%)

GGTCCAAGTAAACCGCTAGCTTGTTGCACCGTGGAGGCCACAGGAGCAGAAACATGGAATGCCAGACG
CTGGGGATGCTGGTACAAGTTGTGGGACTGCA5 (0.000128%)

GGTCCCTCGGCTTCCTGCCTCGGAAGCGCAGCAGCAGGCATCGTGGGAAGGTGAAGAGCTTCCCTAAG
GATGACCCGTCCAAGCCGGTCCACCTCACAGC16 (0.000410%)

GGTGAGGAAGAGGATGGAGATGAAGATGAGGAAGCTGAGTCAGCTACGGGCAAGCGGGCAGCTGAAG
ATGATGAGGATGACGATGTCGATACCAAGAAGC 21 (0.000539%)

GGTGATGGTGAGGAAGAGGATGGAGATGAAGATGAGGAAGCTGAGTCAGCTACGGGCAAGCGGGCAG
CTGAAGATGATGAGGATGACGATGTCGATACCA 10 (0.000256%)

GGTGATTATCCTGAACCATCCAGGCCAAATAAGCGCCGGCTATGCCCCTGTATTGGATTGCCACACGGC
TCACATTGCATGCAAGTTTGCTGAGCTGAAG 13 (0.000333%)

GGTGCAACTTCCTTCGGTCGTCCCGAATCCGGGTTCATCCGACACCAGCCGCCTCCACCATGCCGCCGA
AGTTCGACCCCAACGAGATCAAAGTCGTATA 7 (0.000180%)

GGTGTCGTGAAAACTACCCCTAAAAGCCAAAATGGGAAAGGAAAAGACTCATATCAACATTGTCGTCA
TTGGACACGTAGATTCGGGCAAGTCCACCACT 56 (0.001436%)

GGTTAACGTCCCTAAAACCCGCCGGACTTTCTGTAAGAAGTGTGGCAAGCACCAACCCCATAAAGTGAC
ACAGTACAAGAAGGGCAAGGATTCTCTGTAC 19 (0.000487%)

GTAAAAAGCTGGAAGATGGCCCTAAATTCTTGAAGTCTGGTGATGCTGCCATTGTTGATATGGTTCCTG
GCAAGCCCATGTGTGTTGAGAGCTTCTCAGA 52 (0.001334%)

GTAAAACCCAGCCCATGACCCCTAACAGGGGCCCTCTCAGCCCTCCTAATGACCTCCGGCCTAGCCATG
TGATTTCACTTCCACTCCATAACGCTCCTCA 232 (0.005950%)

GTAAAATCTGTCGAAATGCACCATGAAGCTTTGAGTGAAGCTCTTCCTGGGGACAATGTGGGCTTCAAT
GTCAAGAATGTGTCTGTCAAGGATGTTCGTC 85 (0.002180%)

GTAATAATCTTCTTCATAGTAATACCCATCATAATCGGAGGCTTTGGCAACTGACTAGTTCCCCTAATAA
TCGGTGCCCCCGATATGGCGTTTCCCCGCA 9 (0.000231%)

GTACAAGGTAGTGGGTCGCTGCCTGCCCACCCCCAAATGCCACACGCCGCCCCTCTACCGCATGCGAAT
CTTTGCGCCTAATCATGTCGTCGCCAAGTCC 22 (0.000564%)

GTACTGCTAGGAAGCTCCGTAGTCACCGACGAGACCAGAAGTGGCATGATAAACAGTATAAGAAAGCT
CATTTGGGCACAGCCCTAAAGGCCAACCCTTT 7 (0.000180%)

GTATGGTCTGAGCTATGATATCAATTGGCTTCCTAGGGTTTATCGTGTGAGCACACCATATATTTACAGT
AGGAATAGACGTAGACACACGAGCATATTT 28 (0.000718%)

GTCAACGTTACAACGGAAGTAAAATCTGTCGAAATGCACCATGAAGCTTTGAGTGAAGCTCTTCCTGGG
GACAATGTGGGCTTCAATGTCAAGAATGTGT 6 (0.000154%)

GTCAAGAATGTGTCTGTCAAGGATGTTCGTCGTGGCAACGTTGCTGGTGACAGCAAAAATGACCCACCA
ATGGAAGCAGCTGGCTTCACTGCTCAGGTGA 20 (0.000513%)

GTCAAGGATGTTCGTCGTGGCAACGTTGCTGGTGACAGCAAAAATGACCCACCAATGGAAGCAGCTGG
CTTCACTGCTCAGGTGATTATCCTGAACCATC 11 (0.000282%)

GTCACCAAGTCTGCCCAGAAAGCTCAGAAGGCTAAATGAATATTATCCCTAATACCTGCCACCCCACTC
TTAATCAGTGGTGGAAGAACGGTCTCAGAAC 7 (0.000180%)

GTCACCAAGTTGGGCCGCTTGGTCAAGGACATGAAGATCAAGTCCCTGGAGGAGATCTATCTCTTCTCC
CTGCCCATTAAGGAATCAGAGATCATTGATT 22 (0.000564%)

GTCCAAGTAAACCGCTAGCTTGTTGCACCGTGGAGGCCACAGGAGCAGAAACATGGAATGCCAGACGC
TGGGGATGCTGGTACAAGTTGTGGGACTGCAT 26 (0.000667%)

GTCCACCACTACTGGCCATCTGATCTATAAATGCGGTGGCATCGACAAAAGAACCATTGAAAAATTTGA
GAAGGAGGCTGCTGAGATGGGAAAGGGCTCC 31 (0.000795%)

GTCCCATGCCTCCATCTAGAAGAGATTATGATGATATGAGCCCTCGTCGAGGACCACCTCCCCCTCCTCC
CGGACGAGGCGGCCGGGGTGGTAGCAGAGC 3 (0.000077%)

GTCCCTCGGCTTCCTGCCTCGGAAGCGCAGCAGCAGGCATCGTGGGAAGGTGAAGAGCTTCCCTAAGG
ATGACCCGTCCAAGCCGGTCCACCTCACAGCC 3 (0.000077%)

GTCCTAATCACAGCAGTCCTACTTCTCCTATCTCTCCCAGTCCTAGCTGCTGGCATCACTATACTACTAA
CAGACCGCAACCTCAACACCACCTTCTTCG 19 (0.000487%)

GTCGAAATGCACCATGAAGCTTTGAGTGAAGCTCTTCCTGGGGACAATGTGGGCTTCAATGTCAAGAAT

GTGTCTGTCAAGGATGTTCGTCGTGGCAACG 74 (0.001898%)

GTCGTCCCGAATCCGGGTTCATCCGACACCAGCCGCCTCCACCATGCCGCCGAAGTTCGACCCCAACGA
GATCAAAGTCGTATACCTGAGGTGCACCGGA 6 (0.000154%)

GTCGTGAAAACTACCCCTAAAAGCCAAAATGGGAAAGGAAAAGACTCATATCAACATTGTCGTCATTG
GACACGTAGATTCGGGCAAGTCCACCACTACT 23 (0.000590%)

GTCGTGGCAACGTTGCTGGTGACAGCAAAAATGACCCACCAATGGAAGCAGCTGGCTTCACTGCTCAG
GTGATTATCCTGAACCATCCAGGCCAAATAAG 33 (0.000846%)

GTCTGAGCTATGATATCAATTGGCTTCCTAGGGTTTATCGTGTGAGCACACCATATATTTACAGTAGGAA
TAGACGTAGACACACGAGCATATTTCACCT 23 (0.000590%)

GTCTGATCCGTCCTAATCACAGCAGTCCTACTTCTCCTATCTCTCCCAGTCCTAGCTGCTGGCATCACTA
TACTACTAACAGACCGCAACCTCAACACCA 32 (0.000821%)

GTCTGCCCAGAAAGCTCAGAAGGCTAAATGAATATTATCCCTAATACCTGCCACCCCACTCTTAATCAG
TGGTGGAAGAACGGTCTCAGAACTGTTTGTT 22 (0.000564%)

GTCTGTCAAGGATGTTCGTCGTGGCAACGTTGCTGGTGACAGCAAAAATGACCCACCAATGGAAGCAG
CTGGCTTCACTGCTCAGGTGATTATCCTGAAC 10 (0.000256%)

GTGAAAACTACCCCTAAAAGCCAAAATGGGAAAGGAAAAGACTCATATCAACATTGTCGTCATTGGAC
ACGTAGATTCGGGCAAGTCCACCACTACTGGC 326 (0.008361%)

GTGAATTTGAAGCTGGTATCTCCAAGAATGGGCAGACCCGAGAGCATGCCCTTCTGGCTTACACACTGG
GTGTGAAACAACTAATTGTCGGTGTTAACAA 709 (0.018184%)

GTGACCTATTCACCCTCCACTTCCCGTCTCAGAATCTAAACGTGGTCACCTTCGAGTAGAGAGGCCCGC
CCGCCCACCGTGGGCAGTGCCACCCGCAGAT 6 (0.000154%)

GTGACTTCCGACCGAAGCAAGAATCGCAAAAGGCATTTCAATGCACCTTCCCACATTCGAAGGAAGATT
ATGTCTTCCCCTCTTTCCAAAGAGCTGAGAC 4 (0.000103%)

GTGAGGAAGAGGATGGAGATGAAGATGAGGAAGCTGAGTCAGCTACGGGCAAGCGGGCAGCTGAAGA
TGATGAGGATGACGATGTCGATACCAAGAAGCA 24 (0.000616%)

GTGATGGTGAGGAAGAGGATGGAGATGAAGATGAGGAAGCTGAGTCAGCTACGGGCAAGCGGGCAGC
TGAAGATGATGAGGATGACGATGTCGATACCAA 42 (0.001077%)

GTGATTATCCTGAACCATCCAGGCCAAATAAGCGCCGGCTATGCCCCTGTATTGGATTGCCACACGGCT
CACATTGCATGCAAGTTTGCTGAGCTGAAGG 7 (0.000180%)

GTGCAACTTCCTTCGGTCGTCCCGAATCCGGGTTCATCCGACACCAGCCGCCTCCACCATGCCGCCGAA
GTTCGACCCCAACGAGATCAAAGTCGTATAC 40 (0.001026%)

GTGCCTACCAAAAGCAGCCGACCATCTTTCAAAACAAGAAGAGGGTCCTGCTGGGAGAAACTGGCAAG
GAGAAGCTCCCGCGGTACTACAAGAACATCGG 7 (0.000180%)

GTGGATGCCCGTCACCAAGTTGGGCCGCTTGGTCAAGGACATGAAGATCAAGTCCCTGGAGGAGATCT
ATCTCTTCTCCCTGCCCATTAAGGAATCAGAG 590 (0.015132%)

GTGGCAACGTTGCTGGTGACAGCAAAAATGACCCACCAATGGAAGCAGCTGGCTTCACTGCTCAGGTG
ATTATCCTGAACCATCCAGGCCAAATAAGCGC 56 (0.001436%)

GTGTCGTGAAAACTACCCCTAAAAGCCAAAATGGGAAAGGAAAAGACTCATATCAACATTGTCGTCAT
TGGACACGTAGATTCGGGCAAGTCCACCACTA 46 (0.001180%)

GTGTCTGTCAAGGATGTTCGTCGTGGCAACGTTGCTGGTGACAGCAAAAATGACCCACCAATGGAAGCA
GCTGGCTTCACTGCTCAGGTGATTATCCTGA 21 (0.000539%)

GTGTGGCTCCCGAGGAGCACCCCGTGCTGCTGACCGAGGCCCCCCTGAACCCCAAGGCCAACCGCGAG
AAGATGACCCAGATCATGTTTGAGACCTTCAA 3 (0.000077%)

GTTAACGTCCCTAAAACCCGCCGGACTTTCTGTAAGAAGTGTGGCAAGCACCAACCCCATAAAGTGACA
CAGTACAAGAAGGGCAAGGATTCTCTGTACG 10 (0.000256%)

GTTACAACGGAAGTAAAATCTGTCGAAATGCACCATGAAGCTTTGAGTGAAGCTCTTCCTGGGGACAAT
GTGGGCTTCAATGTCAAGAATGTGTCTGTCA 29 (0.000744%)

GTTCATCCGACACCAGCCGCCTCCACCATGCCGCCGAAGTTCGACCCCAACGAGATCAAAGTCGTATAC
CTGAGGTGCACCGGAGGTGAAGTCGGTGCCA 6 (0.000154%)

GTTCGTCGTGGCAACGTTGCTGGTGACAGCAAAAATGACCCACCAATGGAAGCAGCTGGCTTCACTGCT
CAGGTGATTATCCTGAACCATCCAGGCCAAA 54 (0.001385%)

GTTCGTGGTTGCATTGTGGATGCAAATCTGAGCGTTCTCAACTTGGTTATTGTAAAAAAAGGAGAGAAG
GATATTCCTGGACTGACTGATACTACAGTGC 382 (0.009797%)

GTTCTCCGCTCCCAGACATGGGTCCCTCGGCTTCCTGCCTCGGAAGCGCAGCAGCAGGCATCGTGGGAA
GGTGAAGAGCTTCCCTAAGGATGACCCGTCC 8 (0.000205%)

GTTCTGCCATCAACGAAGTGGTAACCCGAGAATACACCATCAACATTCACAAGCGCATCCATGGAGTGG
GCTTCAAGAAGCGTGCACCTCGGGCACTCAA 10 (0.000256%)

GTTCTGGTAAAAAGCTGGAAGATGGCCCTAAATTCTTGAAGTCTGGTGATGCTGCCATTGTTGATATGG
TTCCTGGCAAGCCCATGTGTGTTGAGAGCTT 60 (0.001539%)

GTTGCACCGTGGAGGCCACAGGAGCAGAAACATGGAATGCCAGACGCTGGGGATGCTGGTACAAGTTG
TGGGACTGCATGCTACTGTCTAGAGCTTGTCT 3 (0.000077%)

GTTGCATTGTGGATGCAAATCTGAGCGTTCTCAACTTGGTTATTGTAAAAAAAGGAGAGAAGGATATTC
CTGGACTGACTGATACTACAGTGCCTCGCCG 10 (0.000256%)

GTTGCTGGTGACAGCAAAAATGACCCACCAATGGAAGCAGCTGGCTTCACTGCTCAGGTGATTATCCTG
AACCATCCAGGCCAAATAAGCGCCGGCTATG 14 (0.000359%)

GTTGGGCCGCTTGGTCAAGGACATGAAGATCAAGTCCCTGGAGGAGATCTATCTCTTCTCCCTGCCCAT
TAAGGAATCAGAGATCATTGATTTCTTCCTG 11 (0.000282%)

GTTTAATCCCTTTGTGACTTCCGACCGAAGCAAGAATCGCAAAAGGCATTTCAATGCACCTTCCCACATT
CGAAGGAAGATTATGTCTTCCCCTCTTTCC 83 (0.002129%)

GTTTTGAAGATTATGCCAGTGCAGAAGCAGACCCGTGCCGGCCAGCGCACCAGGTTCAAGGCATTTGTT
GCTATCGGGGACTACAATGGCCACGTCGGTC 5 (0.000128%)

TAAAAAATTATAACAAACCCTGAGAACCAAAATGAACGAAAATCTGTTCGCTTCATTCATTGCCCCCAC
AATCCTAGGCCTACCCGCCGCAGTACTGATC 5 (0.000128%)

TAAAAAGCTGGAAGATGGCCCTAAATTCTTGAAGTCTGGTGATGCTGCCATTGTTGATATGGTTCCTGG
CAAGCCCATGTGTGTTGAGAGCTTCTCAGAC 30 (0.000769%)

TAAAAATAAAAAATTATAACAAACCCTGAGAACCAAAATGAACGAAAATCTGTTCGCTTCATTCATTGC
CCCCACAATCCTAGGCCTACCCGCCGCAGTA 62 (0.001590%)

TAAAACCCAGCCCATGACCCCTAACAGGGGCCCTCTCAGCCCTCCTAATGACCTCCGGCCTAGCCATGT
GATTTCACTTCCACTCCATAACGCTCCTCAT 22 (0.000564%)

TAAAACCCGCCGGACTTTCTGTAAGAAGTGTGGCAAGCACCAACCCCATAAAGTGACACAGTACAAGA
AGGGCAAGGATTCTCTGTACGCCCAGGGAAAG 7 (0.000180%)

TAAAAGCCAAAATGGGAAAGGAAAAGACTCATATCAACATTGTCGTCATTGGACACGTAGATTCGGGC
AAGTCCACCACTACTGGCCATCTGATCTATAA 44 (0.001128%)

TAAAATCTGTCGAAATGCACCATGAAGCTTTGAGTGAAGCTCTTCCTGGGGACAATGTGGGCTTCAATG
TCAAGAATGTGTCTGTCAAGGATGTTCGTCG 7 (0.000180%)

TAAACCGCTAGCTTGTTGCACCGTGGAGGCCACAGGAGCAGAAACATGGAATGCCAGACGCTGGGGAT
GCTGGTACAAGTTGTGGGACTGCATGCTACTG 41 (0.001052%)

TAAAGGACGAACCTGATCTCTTATACTAGTATCCTTAATCATTTTTATTGCCACAACTAACCTCCTCGGA
CTCCTGCCTCACTCATTTACACCAACCACC 27 (0.000692%)

TAAAGGGAAATTTTCACAATGTCCGGAGCCCTTGATGTCCTGCAAATGAAGGAGGAGGATGTCCTTAAG
TTCCTTGCAGCAGGAACCCACTTAGGTGGCA 549 (0.014080%)

TAAATTCTTGAAGTCTGGTGATGCTGCCATTGTTGATATGGTTCCTGGCAAGCCCATGTGTGTTGAGAGC
TTCTCAGACTATCCACCTTTGGGTCGCTTT 15 (0.000385%)

TAACAAACCCTGAGAACCAAAATGAACGAAAATCTGTTCGCTTCATTCATTGCCCCCACAATCCTAGGC
CTACCCGCCGCAGTACTGATCATTCTATTTC 11 (0.000282%)

TAACACTCACAACAAAACTAACTAATACTAACATCTCAGACGCTCAGGAAATAGAAACCGTCTGAACT
ATCCTGCCCGCCATCATCCTAGTCCTCATCGC 12 (0.000308%)

TAACAGGGGCCCTCTCAGCCCTCCTAATGACCTCCGGCCTAGCCATGTGATTTCACTTCCACTCCATAAC
GCTCCTCATACTAGGCCTACTAACCAACAC 25 (0.000641%)

TAACCATACACAACACTAAAGGACGAACCTGATCTCTTATACTAGTATCCTTAATCATTTTTATTGCCAC
AACTAACCTCCTCGGACTCCTGCCTCACTC 18 (0.000462%)

TAACCGCTAACATTACTGCAGGCCACCTACTCATGCACCTAATTGGAAGCGCCACCCTAGCAATATCAA
CCATTAACCTTCCCTCTACACTTATCATCTT 3 (0.000077%)

TAACCTCAAAACAAATGATAACCATACACAACACTAAAGGACGAACCTGATCTCTTATACTAGTATCCT
TAATCATTTTTATTGCCACAACTAACCTCCT 8 (0.000205%)

TAATAATCTTCTTCATAGTAATACCCATCATAATCGGAGGCTTTGGCAACTGACTAGTTCCCCTAATAAT
CGGTGCCCCCGATATGGCGTTTCCCCGCAT 6 (0.000154%)

TAATACCTGCCACCCCACTCTTAATCAGTGGTGGAAGAACGGTCTCAGAACTGTTTGTTTCAATTGGCCA
TTTAAGTTTAGTAGTAAAAGACTGGTTAAT 4 (0.000103%)

TAATCACAGCAGTCCTACTTCTCCTATCTCTCCCAGTCCTAGCTGCTGGCATCACTATACTACTAACAGA
CCGCAACCTCAACACCACCTTCTTCGACCC 7 (0.000180%)

TAATCCCTTTGTGACTTCCGACCGAAGCAAGAATCGCAAAAGGCATTTCAATGCACCTTCCCACATTCG
AAGGAAGATTATGTCTTCCCCTCTTTCCAAA 3 (0.000077%)

TAATCTTCTTCATAGTAATACCCATCATAATCGGAGGCTTTGGCAACTGACTAGTTCCCCTAATAATCGG
TGCCCCCGATATGGCGTTTCCCCGCATAAA 7 (0.000180%)

TAATGACCTCCGGCCTAGCCATGTGATTTCACTTCCACTCCATAACGCTCCTCATACTAGGCCTACTAAC

CAACACACTAACCATATACCAATGATGGCG 12 (0.000308%)

TACAACGGAAGTAAAATCTGTCGAAATGCACCATGAAGCTTTGAGTGAAGCTCTTCCTGGGACAATGT
GGGCTTCAATGTCAAGAATGTGTCTGTCAAG 8 (0.000205%)

TACAACGTTATCGTCACAGCCCATGCATTTGTAATAATCTTCTTCATAGTAATACCCATCATAATCGGAG
GCTTTGGCAACTGACTAGTTCCCCTAATAA 17 (0.000436%)

TACACAACACTAAAGGACGAACCTGATCTCTTATACTAGTATCCTTAATCATTTTTATTGCCACAACTAA
CCTCCTCGGACTCCTGCCTCACTCATTTAC 3 (0.000077%)

TACATAGGTATGGTCTGAGCTATGATATCAATTGGCTTCCTAGGGTTTATCGTGTGAGCACACCATATAT
TTACAGTAGGAATAGACGTAGACACACGAG 8 (0.000205%)

TACCCCTAAAAGCCAAAATGGGAAAGGAAAAGACTCATATCAACATTGTCGTCATTGGACACGTAGAT
TCGGGCAAGTCCACCACTACTGGCCATCTGAT 11 (0.000282%)

TACCTGCCACCCCACTCTTAATCAGTGGTGGAAGAACGGTCTCAGAACTGTTTGTTTCAATTGGCCATTT
AAGTTTAGTAGTAAAAGACTGGTTAATGAT 4 (0.000103%)

TACGCACACGAGAACATGCCTCTCGCAAAGGATCTCCTTCATCCCTCTCCAGAAGAGGAGAAGAGGAA
ACACAAGAAGAAACGCCTGGTGCAGAGCCCCA 19 (0.000487%)

TACGCCCTGATCGGCGCACTGCGAGCAGTAGCCCAAACAATCTCATATGAAGTCACCCTAGCCATCATT
CTACTATCAACATTACTAATAAGTGGCTCCT 211 (0.005412%)

TAGCTTGTTGCACCGTGGAGGCCACAGGAGCAGAAACATGGAATGCCAGACGCTGGGGATGCTGGTAC
AAGTTGTGGGACTGCATGCTACTGTCTAGAGC 8 (0.000205%)

TAGGAAGCTCCGTAGTCACCGACGAGACCAGAAGTGGCATGATAAACAGTATAAGAAAGCTCATTTGG
GCACAGCCCTAAAGGCCAACCCTTTTGGAGGT 10 (0.000256%)

TAGGTATGGTCTGAGCTATGATATCAATTGGCTTCCTAGGGTTTATCGTGTGAGCACACCATATATTTAC
AGTAGGAATAGACGTAGACACACGAGCATA 3 (0.000077%)

TAGTAAAACCCAGCCCATGACCCCTAACAGGGGCCCTCTCAGCCCTCCTAATGACCTCCGGCCTAGCCA
TGTGATTTCACTTCCACTCCATAACGCTCCT 6 (0.000154%)

TATAACAAACCCTGAGAACCAAAATGAACGAAAATCTGTTCGCTTCATTCATTGCCCCCACAATCCTAG
GCCTACCCGCCGCAGTACTGATCATTCTATT 13 (0.000333%)

TATAGTAAAACCCAGCCCATGACCCCTAACAGGGGCCCTCTCAGCCCTCCTAATGACCTCCGGCCTAGC
CATGTGATTTCACTTCCACTCCATAACGCTC 747 (0.019159%)

TATCACCCTATAGAAGAACTAATGTTAGTATAAGTAACATGAAAACATTCTCCTCCGCATAAGCCTGCG
TCAGATTAAAACACTGAACTGACAATTAACA 26 (0.000667%)

TATCCCTAATACCTGCCACCCCACTCTTAATCAGTGGTGGAAGAACGGTCTCAGAACTGTTTGTTTCAAT
TGGCCATTTAAGTTTAGTAGTAAAAGACTG 16 (0.000410%)

TATGCTCATGTGGTGTTGAGGAAAGCAGACATTGACCTCACCAAGAGGGCGGGAGAACTCACTGAGGA
TGAGGTGGAACGTGTGATCACCATTATGCAGA 9 (0.000231%)

TATGGGGCCTCCCTCCGGAAAATGGTGAAGAAAATTGAAATCAGCCAGCACGCCAAGTACACTTGCTCT
TTCTGTGGCAAAACCAAGATGAAGAGACGAG 5 (0.000128%)

TATGGTCTGAGCTATGATATCAATTGGCTTCCTAGGGTTTATCGTGTGAGCACACCATATATTTACAGTA
GGAATAGACGTAGACACACGAGCATATTTC 12 (0.000308%)

TATTATCCCTAATACCTGCCACCCCACTCTTAATCAGTGGTGGAAGAACGGTCTCAGAACTGTTTGTTTC
AATTGGCCATTTAAGTTTAGTAGTAAAAGA 64 (0.001641%)

TCAAAACAAATGATAACCATACACAACACTAAAGGACGAACCTGATCTCTTATACTAGTATCCTTAATC
ATTTTTATTGCCACAACTAACCTCCTCGGAC 12 (0.000308%)

TCAAACTCTACTCCCACTAATAGCTTTTTGATGACTTCTAGCAAGCCTCGCTAACCTCGCCTTACCCCCC
ACTATTAACCTACTGGGAGAACTCTCTGTG 6 (0.000154%)

TCAAGAATGTGTCTGTCAAGGATGTTCGTCGTGGCAACGTTGCTGGTGACAGCAAAAATGACCCACCAA
TGGAAGCAGCTGGCTTCACTGCTCAGGTGAT 3 (0.000077%)

TCAAGGATGAGGTTTTGAAGATTATGCCAGTGCAGAAGCAGACCCGTGCCGGCCAGCGCACCAGGTTC
AAGGCATTTGTTGCTATCGGGGACTACAATGG 3 (0.000077%)

TCAATGTCAAGAATGTGTCTGTCAAGGATGTTCGTCGTGGCAACGTTGCTGGTGACAGCAAAAATGACC
CACCAATGGAAGCAGCTGGCTTCACTGCTCA 9 (0.000231%)

TCACAACAAAACTAACTAATACTAACATCTCAGACGCTCAGGAAATAGAAACCGTCTGAACTATCCTGC
CCGCCATCATCCTAGTCCTCATCGCCCTCCC 32 (0.000821%)

TCACCAAAGCCCATAAAAATAAAAAATTATAACAAACCCTGAGAACCAAAATGAACGAAAATCTGTTC
GCTTCATTCATTGCCCCCACAATCCTAGGCCT 4 (0.000103%)

TCACCAAGTTGGGCCGCTTGGTCAAGGACATGAAGATCAAGTCCCTGGAGGAGATCTATCTCTTCTCCC
TGCCCATTAAGGAATCAGAGATCATTGATTT 5 (0.000128%)

TCACCCTATAGAAGAACTAATGTTAGTATAAGTAACATGAAAACATTCTCCTCCGCATAAGCCTGCGTC
AGATTAAAACACTGAACTGACAATTAACAGC 3 (0.000077%)

TCACCTTCCACCCTTACTACACAATCAAAGACGCCCTCGGCTTACTTCTCTTCATTCTCTCCTTAATGACA
TTAACACTATTCTCACCAGACCTCCTAGG 7 (0.000180%)

TCAGAAGGCTAAATGAATATTATCCCTAATACCTGCCACCCCACTCTTAATCAGTGGTGGAAGAACGGT
CTCAGAACTGTTTGTTTCAATTGGCCATTTA 4 (0.000103%)

TCAGACTGAGCGTGCCTACCAAAAGCAGCCGACCATCTTTCAAAACAAGAAGAGGGTCCTGCTGGGAG
AAACTGGCAAGGAGAAGCTCCCGCGGTACTAC 5 (0.000128%)

TCAGCCCTCCTAATGACCTCCGGCCTAGCCATGTGATTTCACTTCCACTCCATAACGCTCCTCATACTAG
GCCTACTAACCAACACACTAACCATATACC 5 (0.000128%)

TCAGGTGATTATCCTGAACCATCCAGGCCAAATAAGCGCCGGCTATGCCCCTGTATTGGATTGCCACAC
GGCTCACATTGCATGCAAGTTTGCTGAGCTG 4 (0.000103%)

TCATATCAACATTGTCGTCATTGGACACGTAGATTCGGGCAAGTCCACCACTACTGGCCATCTGATCTAT
AAATGCGGTGGCATCGACAAAAGAACCATT 8 (0.000205%)

TCCAAGTAAACCGCTAGCTTGTTGCACCGTGGAGGCCACAGGAGCAGAAACATGGAATGCCAGACGCT
GGGGATGCTGGTACAAGTTGTGGGACTGCATG14 (0.000359%)

TCCACCACTACTGGCCATCTGATCTATAAATGCGGTGGCATCGACAAAAGAACCATTGAAAAATTTGAG
AAGGAGGCTGCTGAGATGGGAAAGGGCTCCT 11 (0.000282%)

TCCACCCTTACTACACAATCAAAGACGCCCTCGGCTTACTTCTCTTCATTCTCTCCTTAATGACATTAAC
ACTATTCTCACCAGACCTCCTAGGCGACCC 4 (0.000103%)

TCCAGTCAACGTTACAACGGAAGTAAAATCTGTCGAAATGCACCATGAAGCTTTGAGTGAAGCTCTTCC
TGGGGACAATGTGGGCTTCAATGTCAAGAAT 6 (0.000154%)

TCCCACTAATAGCTTTTGATGACTTCTAGCAAGCCTCGCTAACCTCGCCTTACCCCCCACTATTAACCT
ACTGGGAGAACTCTCTGTGCTAGTAACCAC 5 (0.000128%)

TCCCTAATACCTGCCACCCCACTCTTAATCAGTGGTGGAAGAACGGTCTCAGAACTGTTTGTTTCAATTG
GCCATTTAAGTTTAGTAGTAAAAGACTGGT 3 (0.000077%)

TCCCTCACCAAAGCCCATAAAAATAAAAAATTATAACAAACCCTGAGAACCAAAATGAACGAAAATCT
GTTCGCTTCATTCATTGCCCCCACAATCCTAG 55 (0.001411%)

TCCGACCGAAGCAAGAATCGCAAAAGGCATTTCAATGCACCTTCCCACATTCGAAGGAAGATTATGTCT
TCCCCTCTTTCCAAAGAGCTGAGACAGAAGT 6 (0.000154%)

TCCGCATGATGAAACTTCGGCTCACTCCTTGGCGCCTGCCTGATCCTCCAAATCACCACAGGACTATTCC
TAGCCATGCACTACTCACCAGACGCCTCAA 5 (0.000128%)

TCCGCCTCCTTGCTCGCCGCAGCCGCCTCCGCCGCGCGCCTCCTCCGCCGCCGCGGACTCCGGCAGCTTT
ATCGCCAGAGTCCCTGAACTCTCGCTTTCT 614 (0.015748%)

TCCTAATCACAGCAGTCCTACTTCTCCTATCTCTCCCAGTCCTAGCTGCTGGCATCACTATACTACTAAC
AGACCGCAACCTCAACACCACCTTCTTCGA 14 (0.000359%)

TCCTAATGACCTCCGGCCTAGCCATGTGATTTCACTTCCACTCCATAACGCTCCTCATACTAGGCCTACT
AACCAACACACTAACCATATACCAATGATG 5 (0.000128%)

TCCTTCGGTCGTCCCGAATCCGGGTTCATCCGACACCAGCCGCCTCCACCATGCCGCCGAAGTTCGACC
CCAACGAGATCAAAGTCGTATACCTGAGGTG 3 (0.000077%)

TCCTTGCTCGCCGCAGCCGCCTCCGCCGCGCGCCTCCTCCGCCGCCGCGGACTCCGGCAGCTTTATCGCC
AGAGTCCCTGAACTCTCGCTTTCTTTTTAA 4 (0.000103%)

TCGAAATGCACCATGAAGCTTTGAGTGAAGCTCTTCCTGGGGACAATGTGGGCTTCAATGTCAAGAATG
TGTCTGTCAAGGATGTTCGTCGTGGCAACGT 26 (0.000667%)

TCGCCGTTCTGGTAAAAAGCTGGAAGATGGCCCTAAATTCTTGAAGTCTGGTGATGCTGCCATTGTTGA
TATGGTTCCTGGCAAGCCCATGTGTGTTGAG 31 (0.000795%)

TCGGCTTCCTGCCTCGGAAGCGCAGCAGCAGGCATCGTGGGAAGGTGAAGAGCTTCCCTAAGGATGAC
CCGTCCAAGCCGGTCCACCTCACAGCCTTCCT 6 (0.000154%)

TCGGGCACGCTACGAGAGTACAAGGTAGTGGGTCGCTGCCTGCCCACCCCCAAATGCCACACGCCGCCC
CTCTACCGCATGCGAATCTTTGCGCCTAATC 3 (0.000077%)

TCGGTCGTCCCGAATCCGGGTTCATCCGACACCAGCCGCCTCCACCATGCCGCCGAAGTTCGACCCCAA
CGAGATCAAAGTCGTATACCTGAGGTGCACC 5 (0.000128%)

TCGTACTGCTAGGAAGCTCCGTAGTCACCGACGAGACCAGAAGTGGCATGATAAACAGTATAAGAAAG
CTCATTTGGGCACAGCCCTAAAGGCCAACCCT 7 (0.000180%)

TCGTGAAAACTACCCCTAAAAGCCAAAATGGGAAAGGAAAAGACTCATATCAACATTGTCGTCATTGG
ACACGTAGATTCGGGCAAGTCCACCACTACTG 3 (0.000077%)

TCGTGGCAACGTTGCTGGTGACAGCAAAAATGACCCACCAATGGAAGCAGCTGGCTTCACTGCTCAGGT
GATTATCCTGAACCATCCAGGCCAAATAAGC 3 (0.000077%)

TCTACTCCCACTAATAGCTTTTGATGACTTCTAGCAAGCCTCGCTAACCTCGCCTTACCCCCCACTATT

AACCTACTGGGAGAACTCTCTGTGCTAGTA 15 (0.000385%)

TCTCAAGGATGAGGTTTTGAAGATTATGCCAGTGCAGAAGCAGACCCGTGCCGGCCAGCGCACCAGGTT
CAAGGCATTTGTTGCTATCGGGGACTACAAT 3 (0.000077%)

TCTCAGCCCTCCTAATGACCTCCGGCCTAGCCATGTGATTTCACTTCCACTCCATAACGCTCCTCATACT
AGGCCTACTAACCAACACACTAACCATATA 14 (0.000359%)

TCTCCTTCATCCCTCTCCAGAAGAGGAGAAGAGGAAACACAAGAAGAAACGCCTGGTGCAGAGCCCCA
ATTCCTACTTCATGGATGTGAAATGCCCAGGA 3 (0.000077%)

TCTCGCAAAGGATCTCCTTCATCCCTCTCCAGAAGAGGAGAAGAGGAAACACAAGAAGAAACGCCTGG
TGCAGAGCCCCAATTCCTACTTCATGGATGTG 11 (0.000282%)

TCTGATCCGTCCTAATCACAGCAGTCCTACTTCTCCTATCTCTCCCAGTCCTAGCTGCTGGCATCACTAT
ACTACTAACAGACCGCAACCTCAACACCAC 7 (0.000180%)

TCTGCCCAGAAAGCTCAGAAGGCTAAATGAATATTATCCCTAATACCTGCCACCCCACTCTTAATCAGT
GGTGGAAGAACGGTCTCAGAACTGTTTGTTT 13 (0.000333%)

TCTGGTAAAAAGCTGGAAGATGGCCCTAAATTCTTGAAGTCTGGTGATGCTGCCATTGTTGATATGGTT
CCTGGCAAGCCCATGTGTGTTGAGAGCTTCT 27 (0.000692%)

TCTGTCGAAATGCACCATGAAGCTTTGAGTGAAGCTCTTCCTGGGGACAATGTGGGCTTCAATGTCAAG
AATGTGTCTGTCAAGGATGTTCGTCGTGGCA 51 (0.001308%)

TCTTCCTGGGGGCCTCTCTCAAGGATGAGGTTTTGAAGATTATGCCAGTGCAGAAGCAGACCCGTGCCG
GCCAGCGCACCAGGTTCAAGGCATTTGTTGC 4 (0.000103%)

TCTTGAAGTCTGGTGATGCTGCCATTGTTGATATGGTTCCTGGCAAGCCCATGTGTGTTGAGAGCTTCTC
AGACTATCCACCTTTGGGTCGCTTTGCTGT41 (0.001052%)

TGAAAACTACCCCTAAAAGCCAAAATGGGAAAGGAAAAGACTCATATCAACATTGTCGTCATTGGACA
CGTAGATTCGGGCAAGTCCACCACTACTGGCC 100 (0.002565%)

TGAAACTTCGGCTCACTCCTTGGCGCCTGCCTGATCCTCCAAATCACCACAGGACTATTCCTAGCCATGC
ACTACTCACCAGACGCCTCAACCGCCTTTT 5 (0.000128%)

TGAACGCAGGCACATACTTCCTATTCTACACCCTAGTAGGCTCCCTTCCCCTACTCATCGCACTGATTTA
CACTCACAACACCCTAGGCTCACTAAACAT 8 (0.000205%)

TGAAGATGAGGAAGCTGAGTCAGCTACGGGCAAGCGGGCAGCTGAAGATGATGAGGATGACGATGTCG
ATACCAAGAAGCAGAAGACCGACGAGGATGAC 29 (0.000744%)

TGAAGATTATGCCAGTGCAGAAGCAGACCCGTGCCGGCCAGCGCACCAGGTTCAAGGCATTTGTTGCTA
TCGGGGACTACAATGGCCACGTCGGTCTGGG 12 (0.000308%)

TGAAGGCCTCGGGCACGCTACGAGAGTACAAGGTAGTGGGTCGCTGCCTGCCCACCCCCAAATGCCAC
ACGCCGCCCCTCTACCGCATGCGAATCTTTGC 15 (0.000385%)

TGAAGTCTGGTGATGCTGCCATTGTTGATATGGTTCCTGGCAAGCCCATGTGTGTTGAGAGCTTCTCAGA
CTATCCACCTTTGGGTCGCTTTGCTGTTCG 12 (0.000308%)

TGAAGTTTAATCCCTTTGTGACTTCCGACCGAAGCAAGAATCGCAAAAGGCATTTCAATGCACCTTCCC
ACATTCGAAGGAAGATTATGTCTTCCCCTCT 6 (0.000154%)

TGACCCACCAATGGAAGCAGCTGGCTTCACTGCTCAGGTGATTATCCTGAACCATCCAGGCCAAATAAG
CGCCGGCTATGCCCCTGTATTGGATTGCCAC 5 (0.000128%)

TGACCCCTAACAGGGGCCCTCTCAGCCCTCCTAATGACCTCCGGCCTAGCCATGTGATTTCACTTCCACT
CCATAACGCTCCTCATACTAGGCCTACTAA 10 (0.000256%)

TGACTTCTAGCAAGCCTCGCTAACCTCGCCTTACCCCCCACTATTAACCTACTGGGAGAACTCTCTGTGC
TAGTAACCACGTTCTCCTGATCAAATATCA 5 (0.000128%)

TGAGATGGGAAAGGGCTCCTTCAAGTATGCCTGGGTCTTGGATAAACTGAAAGCTGAGCGTGAACGTG
GTATCACCATTGATATCTCCTTGTGGAAATTT 16 (0.000410%)

TGAGCGTGCCTACCAAAAGCAGCCGACCATCTTTCAAAACAAGAAGAGGGTCCTGCTGGGAGAAACTG
GCAAGGAGAAGCTCCCGCGGTACTACAAGAAC 8 (0.000205%)

TGAGGAAGAGGATGGAGATGAAGATGAGGAAGCTGAGTCAGCTACGGGCAAGCGGGCAGCTGAAGAT
GATGAGGATGACGATGTCGATACCAAGAAGCAG 3 (0.000077%)

TGAGGAAGCTGAGTCAGCTACGGGCAAGCGGGCAGCTGAAGATGATGAGGATGACGATGTCGATACCA
AGAAGCAGAAGACCGACGAGGATGACTAGACA 6 (0.000154%)

TGAGGCAGAGGTCCAAGTAAACCGCTAGCTTGTTGCACCGTGGAGGCCACAGGAGCAGAAACATGGAA
TGCCAGACGCTGGGGATGCTGGTACAAGTTGT 4 (0.000103%)

TGATAACCATACACAACACTAAAGGACGAACCTGATCTCTTATACTAGTATCCTTAATCATTTTTATTGC
CACAACTAACCTCCTCGGACTCCTGCCTCA 22 (0.000564%)

TGATCCGTCCTAATCACAGCAGTCCTACTTCTCCTATCTCTCCCAGTCCTAGCTGCTGGCATCACTATAC
TACTAACAGACCGCAACCTCAACACCACCT 3 (0.000077%)

TGATCGCCGTTCTGGTAAAAAGCTGGAAGATGGCCCTAAATTCTTGAAGTCTGGTGATGCTGCCATTGT
TGATATGGTTCCTGGCAAGCCCATGTGTGTT 11 (0.000282%)

TGATGACTTCTAGCAAGCCTCGCTAACCTCGCCTTACCCCCCACTATTAACCTACTGGGAGAACTCTCTG
TGCTAGTAACCACGTTCTCCTGATCAAATA 5 (0.000128%)

TGATGGTGAGGAAGAGGATGGAGATGAAGATGAGGAAGCTGAGTCAGCTACGGGCAAGCGGGCAGCT
GAAGATGATGAGGATGACGATGTCGATACCAAG 8 (0.000205%)

TGATTATCCTGAACCATCCAGGCCAAATAAGCGCCGGCTATGCCCCTGTATTGGATTGCCACACGGCTC
ACATTGCATGCAAGTTTGCTGAGCTGAAGGA 11 (0.000282%)

TGCAACTTCCTTCGGTCGTCCCGAATCCGGGTTCATCCGACACCAGCCGCCTCCACCATGCCGCCGAAG
TTCGACCCCAACGAGATCAAAGTCGTATACC 18 (0.000462%)

TGCATTTGTAATAATCTTCTTCATAGTAATACCCATCATAATCGGAGGCTTTGGCAACTGACTAGTTCCC
CTAATAATCGGTGCCCCCGATATGGCGTTT 19 (0.000487%)

TGCCCAGAAAGCTCAGAAGGCTAAATGAATATTATCCCTAATACCTGCCACCCCACTCTTAATCAGTGG
TGGAAGAACGGTCTCAGAACTGTTTGTTTCA 11 (0.000282%)

TGCCCGTCACCAAGTTGGGCCGCTTGGTCAAGGACATGAAGATCAAGTCCCTGGAGGAGATCTATCTCT
TCTCCCTGCCCATTAAGGAATCAGAGATCAT 13 (0.000333%)

TGCCCTTCTGGCTTACACACTGGGTGTGAAACAACTAATTGTCGGTGTTAACAAAATGGATTCCACTGA
GCCACCCTACAGCCAGAAGAGATATGAGGAA 8 (0.000205%)

TGCCTACCAAAAGCAGCCGACCATCTTTCAAAACAAGAAGAGGGTCCTGCTGGGAGAAACTGGCAAGG
AGAAGCTCCCGCGGTACTACAAGAACATCGGT 4 (0.000103%)

TGCCTCTCGCAAAGGATCTCCTTCATCCCTCTCCAGAAGAGGAGAAGAGGAAACACAAGAAGAAACGC
CTGGTGCAGAGCCCCAATTCCTACTTCATGGA 5 (0.000128%)

TGCGCAGGCTGAAGCGCAAAAGAAGAAAGATGAGGCAGAGGTCCAAGTAAACCGCTAGCTTGTTGCAC
CGTGGAGGCCACAGGAGCAGAAACATGGAATG 12 (0.000308%)

TGCTAGGAAGCTCCGTAGTCACCGACGAGACCAGAAGTGGCATGATAAACAGTATAAGAAAGCTCATT
TGGGCACAGCCCTAAAGGCCAACCCTTTTGGA 4 (0.000103%)

TGCTCAGGTGATTATCCTGAACCATCCAGGCCAAATAAGCGCCGGCTATGCCCCTGTATTGGATTGCCA
CACGGCTCACATTGCATGCAAGTTTGCTGAG 8 (0.000205%)

TGCTCATGTGGTGTTGAGGAAAGCAGACATTGACCTCACCAAGAGGGCGGGAGAACTCACTGAGGATG
AGGTGGAACGTGTGATCACCATTATGCAGAAT 23 (0.000590%)

TGCTGAGATGGGAAAGGGCTCCTTCAAGTATGCCTGGGTCTTGGATAAACTGAAAGCTGAGCGTGAAC
GTGGTATCACCATTGATATCTCCTTGTGGAAA 47 (0.001205%)

TGCTGGAGCTGGCAAGGTCACCAAGTCTGCCCAGAAAGCTCAGAAGGCTAAATGAATATTATCCCTAAT
ACCTGCCACCCCACTCTTAATCAGTGGTGGA 12 (0.000308%)

TGCTGGTGACAGCAAAAATGACCCACCAATGGAAGCAGCTGGCTTCACTGCTCAGGTGATTATCCTGAA
CCATCCAGGCCAAATAAGCGCCGGCTATGCC 28 (0.000718%)

TGGAAGATGGCCCTAAATTCTTGAAGTCTGGTGATGCTGCCATTGTTGATATGGTTCCTGGCAAGCCCAT
GTGTGTTGAGAGCTTCTCAGACTATCCACC 69 (0.001770%)

TGGAAGCAGCTGGCTTCACTGCTCAGGTGATTATCCTGAACCATCCAGGCCAAATAAGCGCCGGCTATG
CCCCTGTATTGGATTGCCACACGGCTCACAT 6 (0.000154%)

TGGAGATGAAGATGAGGAAGCTGAGTCAGCTACGGGCAAGCGGGCAGCTGAAGATGATGAGGATGAC
GATGTCGATACCAAGAAGCAGAAGACCGACGAG 51 (0.001308%)

TGGAGCTGGCAAGGTCACCAAGTCTGCCCAGAAAGCTCAGAAGGCTAAATGAATATTATCCCTAATACC
TGCCACCCCACTCTTAATCAGTGGTGGAAGA 5 (0.000128%)

TGGATACATAGGTATGGTCTGAGCTATGATATCAATTGGCTTCCTAGGGTTTATCGTGTGAGCACACCAT
ATATTTACAGTAGGAATAGACGTAGACACA 19 (0.000487%)

TGGATGCCCGTCACCAAGTTGGGCCGCTTGGTCAAGGACATGAAGATCAAGTCCCTGGAGGAGATCTAT
CTCTTCTCCCTGCCCATTAAGGAATCAGAGA 47 (0.001205%)

TGGCAACGTTGCTGGTGACAGCAAAAATGACCCACCAATGGAAGCAGCTGGCTTCACTGCTCAGGTGAT
TATCCTGAACCATCCAGGCCAAATAAGCGCC 37 (0.000949%)

TGGCAAGGTCACCAAGTCTGCCCAGAAAGCTCAGAAGGCTAAATGAATATTATCCCTAATACCTGCCAC
CCCACTCTTAATCAGTGGTGGAAGAACGGTC 16 (0.000410%)

TGGCCCTAAATTCTTGAAGTCTGGTGATGCTGCCATTGTTGATATGGTTCCTGGCAAGCCCATGTGTGTT
GAGAGCTTCTCAGACTATCCACCTTTGGGT 15 (0.000385%)

TGGCTTCACTGCTCAGGTGATTATCCTGAACCATCCAGGCCAAATAAGCGCCGGCTATGCCCCTGTATT
GGATTGCCACACGGCTCACATTGCATGCAAG 14 (0.000359%)

TGGGAAAGGAAAAGACTCATATCAACATTGTCGTCATTGGACACGTAGATTCGGGCAAGTCCACCACTA
CTGGCCATCTGATCTATAAATGCGGTGGCAT 25 (0.000641%)

TGGGAAAGGGCTCCTTCAAGTATGCCTGGGTCTTGGATAAACTGAAAGCTGAGCGTGAACGTGGTATCA

CCATTGATATCTCCTTGTGGAAATTTGAGAC 46 (0.001180%)

TGGGCCGCTTGGTCAAGGACATGAAGATCAAGTCCCTGGAGGAGATCTATCTCTTCTCCCTGCCCATTA
AGGAATCAGAGATCATTGATTTCTTCCTGGG 10 (0.000256%)

TGGGGGCCTCTCTCAAGGATGAGGTTTTGAAGATTATGCCAGTGCAGAAGCAGACCCGTGCCGGCCAGC
GCACCAGGTTCAAGGCATTTGTTGCTATCGG 3 (0.000077%)

TGGGTCCCTCGGCTTCCTGCCTCGGAAGCGCAGCAGCAGGCATCGTGGGAAGGTGAAGAGCTTCCCTAA
GGATGACCCGTCCAAGCCGGTCCACCTCACA 7 (0.000180%)

TGGTAAAAAGCTGGAAGATGGCCCTAAATTCTTGAAGTCTGGTGATGCTGCCATTGTTGATATGGTTCC
TGGCAAGCCCATGTGTGTTGAGAGCTTCTCA 6 (0.000154%)

TGGTGAGGAAGAGGATGGAGATGAAGATGAGGAAGCTGAGTCAGCTACGGGCAAGCGGGCAGCTGAA
GATGATGAGGATGACGATGTCGATACCAAGAAG 13 (0.000333%)

TGTCAAGAATGTGTCTGTCAAGGATGTTCGTCGTGGCAACGTTGCTGGTGACAGCAAAAATGACCCACC
AATGGAAGCAGCTGGCTTCACTGCTCAGGTG 19 (0.000487%)

TGTCAAGGATGTTCGTCGTGGCAACGTTGCTGGTGACAGCAAAAATGACCCACCAATGGAAGCAGCTG
GCTTCACTGCTCAGGTGATTATCCTGAACCAT 13 (0.000333%)

TGTCGAAATGCACCATGAAGCTTTGAGTGAAGCTCTTCCTGGGGACAATGTGGGCTTCAATGTCAAGAA
TGTGTCTGTCAAGGATGTTCGTCGTGGCAAC 32 (0.000821%)

TGTCGTGAAAACTACCCCTAAAAGCCAAAATGGGAAAGGAAAAGACTCATATCAACATTGTCGTCATT
GGACACGTAGATTCGGGCAAGTCCACCACTAC 13 (0.000333%)

TGTCTGTCAAGGATGTTCGTCGTGGCAACGTTGCTGGTGACAGCAAAAATGACCCACCAATGGAAGCAG
CTGGCTTCACTGCTCAGGTGATTATCCTGAA 23 (0.000590%)

TGTGGCTCCCGAGGAGCACCCCGTGCTGCTGACCGAGGCCCCCCTGAACCCCAAGGCCAACCGCGAGA
AGATGACCCAGATCATGTTTGAGACCTTCAAC 4 (0.000103%)

TGTGTCTGTCAAGGATGTTCGTCGTGGCAACGTTGCTGGTGACAGCAAAAATGACCCACCAATGGAAGC
AGCTGGCTTCACTGCTCAGGTGATTATCCTG 11 (0.000282%)

TGTTCGTCGTGGCAACGTTGCTGGTGACAGCAAAAATGACCCACCAATGGAAGCAGCTGGCTTCACTGC
TCAGGTGATTATCCTGAACCATCCAGGCCAA 13 (0.000333%)

TTAATCCCTTTGTGACTTCCGACCGAAGCAAGAATCGCAAAAGGCATTTCAATGCACCTTCCCACATTC
GAAGGAAGATTATGTCTTCCCCTCTTTCCAA 5 (0.000128%)

TTACAACGGAAGTAAAATCTGTCGAAATGCACCATGAAGCTTTGAGTGAAGCTCTTCCTGGGGACAATG
TGGGCTTCAATGTCAAGAATGTGTCTGTCAA 19 (0.000487%)

TTATAACAAACCCTGAGAACCAAAATGAACGAAAATCTGTTCGCTTCATTCATTGCCCCCACAATCCTA
GGCCTACCCGCCGCAGTACTGATCATTCTAT 42 (0.001077%)

TTATCCCTAATACCTGCCACCCCACTCTTAATCAGTGGTGGAAGAACGGTCTCAGAACTGTTTGTTTCAA
TTGGCCATTTAAGTTTAGTAGTAAAAGACT 6 (0.000154%)

TTCAAAACAAGAAGAGGGTCCTGCTGGGAGAAACTGGCAAGGAGAAGCTCCCGCGGTACTACAAGAAC
ATCGGTCTGGGCTTCAAGACACCCAAGGAGGC 18 (0.000462%)

TTCAAACTCTACTCCCACTAATAGCTTTTTGATGACTTCTAGCAAGCCTCGCTAACCTCGCCTTACCCCC
CACTATTAACCTACTGGGAGAACTCTCTGT 23 (0.000590%)

TTCAATGTCAAGAATGTGTCTGTCAAGGATGTTCGTCGTGGCAACGTTGCTGGTGACAGCAAAAATGAC
CCACCAATGGAAGCAGCTGGCTTCACTGCTC 40 (0.001026%)

TTCACTGCTCAGGTGATTATCCTGAACCATCCAGGCCAAATAAGCGCCGGCTATGCCCCTGTATTGGATT
GCCACACGGCTCACATTGCATGCAAGTTTG 8 (0.000205%)

TTCAGACTGAGCGTGCCTACCAAAAGCAGCCGACCATCTTTCAAAACAAGAAGAGGGTCCTGCTGGGA
GAAACTGGCAAGGAGAAGCTCCCGCGGTACTA 62 (0.001590%)

TTCAGAGGAAACAAATGGTCATTGATGTCCTTCACCCCGGGAAGGCGACAGTGCCTAAGACAGAAATT
CGGGAAAAACTAGCCAAAATGTACAAGACCAC 6 (0.000154%)

TTCATCCCTCTCCAGAAGAGGAGAAGAGGAAACACAAGAAGAAACGCCTGGTGCAGAGCCCCAATTCC
TACTTCATGGATGTGAAATGCCCAGGATGCTA 7 (0.000180%)

TTCCACCCTTACTACACAATCAAAGACGCCCTCGGCTTACTTCTCTTCATTCTCTCCTTAATGACATTAAC
ACTATTCTCACCAGACCTCCTAGGCGACC 7 (0.000180%)

TTCCGACCGAAGCAAGAATCGCAAAAGGCATTTCAATGCACCTTCCCACATTCGAAGGAAGATTATGTC
TTCCCCTCTTTCCAAAGAGCTGAGACAGAAG 8 (0.000205%)

TTCCTGGGGGCCTCTCTCAAGGATGAGGTTTTGAAGATTATGCCAGTGCAGAAGCAGACCCGTGCCGGC
CAGCGCACCAGGTTCAAGGCATTTGTTGCTA 9 (0.000231%)

TTCGATACGGGATAATCCTATTTATTACCTCAGAAGTTTTTTTCTTCGCAGGATTTTTCTGAGCCTTTTAC
CACTCCAGCCTAGCCCCTACCCCCCAATT 9 (0.000231%)

TTCGGCTCACTCCTTGGCGCCTGCCTGATCCTCCAAATCACCACAGGACTATTCCTAGCCATGCACTACT
CACCAGACGCCTCAACCGCCTTTTCATCAA 8 (0.000205%)

TTCGGTCGTCCCGAATCCGGGTTCATCCGACACCAGCCGCCTCCACCATGCCGCCGAAGTTCGACCCCA
ACGAGATCAAAGTCGTATACCTGAGGTGCAC 3 (0.000077%)

TTCGTACTGCTAGGAAGCTCCGTAGTCACCGACGAGACCAGAAGTGGCATGATAAACAGTATAAGAAA
GCTCATTTGGGCACAGCCCTAAAGGCCAACCC 6 (0.000154%)

TTCGTCGTGGCAACGTTGCTGGTGACAGCAAAAATGACCCACCAATGGAAGCAGCTGGCTTCACTGCTC
AGGTGATTATCCTGAACCATCCAGGCCAAAT 12 (0.000308%)

TTCGTCTGATCCGTCCTAATCACAGCAGTCCTACTTCTCCTATCTCTCCCAGTCCTAGCTGCTGGCATCAC
TATACTACTAACAGACCGCAACCTCAACA 11 (0.000282%)

TTCGTGGTTGCATTGTGGATGCAAATCTGAGCGTTCTCAACTTGGTTATTGTAAAAAAAGGAGAGAAGG
ATATTCCTGGACTGACTGATACTACAGTGCC 8 (0.000205%)

TTCTAGCAAGCCTCGCTAACCTCGCCTTACCCCCCACTATTAACCTACTGGGAGAACTCTCTGTGCTAGT
AACCACGTTCTCCTGATCAAATATCACTCT 5 (0.000128%)

TTCTGCCATCAACGAAGTGGTAACCCGAGAATACACCATCAACATTCACAAGCGCATCCATGGAGTGGG
CTTCAAGAAGCGTGCACCTCGGGCACTCAAA 7 (0.000180%)

TTCTGGTAAAAAGCTGGAAGATGGCCCTAAATTCTTGAAGTCTGGTGATGCTGCCATTGTTGATATGGTT
CCTGGCAAGCCCATGTGTGTTGAGAGCTTC 21 (0.000539%)

TTCTTCCTGGGGGCCTCTCTCAAGGATGAGGTTTTGAAGATTATGCCAGTGCAGAAGCAGACCCGTGCC
GGCCAGCGCACCAGGTTCAAGGCATTTGTTG 247 (0.006335%)

TTCTTGAAGTCTGGTGATGCTGCCATTGTTGATATGGTTCCTGGCAAGCCCATGTGTGTTGAGAGCTTCT
CAGACTATCCACCTTTGGGTCGCTTTGCTG 13 (0.000333%)

TTGAAGATTATGCCAGTGCAGAAGCAGACCCGTGCCGGCCAGCGCACCAGGTTCAAGGCATTTGTTGCT
ATCGGGGACTACAATGGCCACGTCGGTCTGG 8 (0.000205%)

TTGAAGTCTGGTGATGCTGCCATTGTTGATATGGTTCCTGGCAAGCCCATGTGTGTTGAGAGCTTCTCAG
ACTATCCACCTTTGGGTCGCTTTGCTGTTC 55 (0.001411%)

TTGATCGCCGTTCTGGTAAAAAGCTGGAAGATGGCCCTAAATTCTTGAAGTCTGGTGATGCTGCCATTG
TTGATATGGTTCCTGGCAAGCCCATGTGTGT 18 (0.000462%)

TTGCTCGCCGCAGCCGCCTCCGCCGCGCGCCTCCTCCGCCGCCGCGGACTCCGGCAGCTTTATCGCCAG
AGTCCCTGAACTCTCGCTTTCTTTTTAATCC 3 (0.000077%)

TTGCTGGTGACAGCAAAAATGACCCACCAATGGAAGCAGCTGGCTTCACTGCTCAGGTGATTATCCTGA
ACCATCCAGGCCAAATAAGCGCCGGCTATGC 6 (0.000154%)

TTGGATACATAGGTATGGTCTGAGCTATGATATCAATTGGCTTCCTAGGGTTTATCGTGTGAGCACACCA
TATATTTACAGTAGGAATAGACGTAGACAC 8 (0.000205%)

TTGGGCCGCTTGGTCAAGGACATGAAGATCAAGTCCCTGGAGGAGATCTATCTCTTCTCCCTGCCCATT
AAGGAATCAGAGATCATTGATTTCTTCCTGG 13 (0.000333%)

TTGTAATAATCTTCTTCATAGTAATACCCATCATAATCGGAGGCTTTGGCAACTGACTAGTTCCCCTAAT
AATCGGTGCCCCCGATATGGCGTTTCCCCG 8 (0.000205%)

TTGTGACTTCCGACCGAAGCAAGAATCGCAAAAGGCATTTCAATGCACCTTCCCACATTCGAAGGAAGA
TTATGTCTTCCCCTCTTTCCAAAGAGCTGAG 5 (0.000128%)

TTTAATCCCTTTGTGACTTCCGACCGAAGCAAGAATCGCAAAAGGCATTTCAATGCACCTTCCCACATTC
GAAGGAAGATTATGTCTTCCCCTCTTTCCA 9 (0.000231%)

TTTCAAAACAAGAAGAGGGTCCTGCTGGGAGAAACTGGCAAGGAGAAGCTCCCGCGGTACTACAAGAA
CATCGGTCTGGGCTTCAAGACACCCAAGGAGG 22 (0.000564%)

TTTGAAGATTATGCCAGTGCAGAAGCAGACCCGTGCCGGCCAGCGCACCAGGTTCAAGGCATTTGTTGC
TATCGGGGACTACAATGGCCACGTCGGTCTG 29 (0.000744%)

TTTGATGACTTCTAGCAAGCCTCGCTAACCTCGCCTTACCCCCCACTATTAACCTACTGGGAGAACTCTC
TGTGCTAGTAACCACGTTCTCCTGATCAAA 8 (0.000205%)

TTTGCTCCAGTCAACGTTACAACGGAAGTAAAATCTGTCGAAATGCACCATGAAGCTTTGAGTGAAGCT
CTTCCTGGGGACAATGTGGGCTTCAATGTCA 4 (0.000103%)

TTTGGATACATAGGTATGGTCTGAGCTATGATATCAATTGGCTTCCTAGGGTTTATCGTGTGAGCACACC
ATATATTTACAGTAGGAATAGACGTAGACA 52 (0.001334%)

TTTGTAATAATCTTCTTCATAGTAATACCCATCATAATCGGAGGCTTTGGCAACTGACTAGTTCCCCTAA
TAATCGGTGCCCCCGATATGGCGTTTCCCC 7 (0.000180%)

TTTGTGACTTCCGACCGAAGCAAGAATCGCAAAAGGCATTTCAATGCACCTTCCCACATTCGAAGGAAG
ATTATGTCTTCCCCTCTTTCCAAAGAGCTGA 8 (0.000205%)

TTTTGATGACTTCTAGCAAGCCTCGCTAACCTCGCCTTACCCCCCACTATTAACCTACTGGGAGAACTCT
CTGTGCTAGTAACCACGTTCTCCTGATCAA 12 (0.000308%)

TTTTTGATGACTTCTAGCAAGCCTCGCTAACCTCGCCTTACCCCCCACTATTAACCTACTGGGAGAACTC

TCTGTGCTAGTAACCACGTTCTCCTGATCA 16 (0.000410%)

TTTTTTTTTTTTTTTTTTTTTTTTTTTTTTTTTTTTTTTTTTTTTTTTTTTTTTTTTTTTTTTTTTTTTTTTTTTTTTTT
TTTTTTTTTTTTTTTTTTTTTT 1660 (0.042575%)

After filtering
After filtering: read1: quality
Value of each position will be shown on mouse over.
2040608010012014037.53838.53939.540

positionqualityATCGmean

<https://plot.ly/>
After filtering: read1: base contents
Value of each position will be shown on mouse over.
2040608010012014000.10.20.30.40.50.60.7

positionbase content ratiosA(25.27%)T(27.17%)C(23.92%)G(23.56%)N(0.052%)GC(47.49%)

<https://plot.ly/>
After filtering: read1: KMER counting
Darker background means larger counts. The count will be shown on
mouse over.
AA AT AC AG TA TT TC TG CA CT CC CG GA GT GC GG
AAA AAAAA AAAAT AAAAC AAAAG AAATA AAATT AAATC AAATG AAACA AAACT
AAACC
AAACG AAAGA AAAGT AAAGC AAAGG
AAT AATAA AATAT AATAC AATAG AATTA AATTT AATTC AATTG AATCA AATCT
AATCC
AATCGAATGA AATGT AATGC AATGG
AAC AACAA AACAT AACAC AACAG AACTA AACTT AACTC AACTG AACCA AACCT
AACCC
AACCG AACGA AACGT AACGC AACGG
AAG AAGAA AAGAT AAGAC AAGAG AAGTA AAGTT AAGTC AAGTG AAGCA AAGCT
AAGCC
AAGCG AAGGA AAGGT AAGGC AAGGG
ATA ATAAA ATAAT ATAAC ATAAG ATATA ATATT ATATC ATATG ATACA ATACT
ATACC
ATACGATAGA ATAGT ATAGC ATAGG
ATT ATTAA ATTAT ATTAC ATTAG ATTTA ATTTT ATTTC ATTTG ATTCA ATTCT ATTCC
ATTCG ATTGA ATTGT ATTGC ATTGG
ATC ATCAA ATCAT ATCAC ATCAG ATCTA ATCTT ATCTC ATCTG ATCCA ATCCT ATCCC
ATCCGATCGA ATCGT ATCGC ATCGG
ATG ATGAA ATGAT ATGAC ATGAG ATGTA ATGTT ATGTC ATGTG ATGCA ATGCT
ATGCC
ATGCGATGGA ATGGT ATGGC ATGGG
ACA ACAAA ACAAT ACAAC ACAAG ACATA ACATT ACATC ACATG ACACA ACACT
ACACC
ACACG ACAGA ACAGT ACAGC ACAGG
ACT ACTAA ACTAT ACTAC ACTAG ACTTA ACTTT ACTTC ACTTG ACTCA ACTCT ACTCC
ACTCGACTGA ACTGT ACTGC ACTGG
ACC ACCAA ACCAT ACCAC ACCAG ACCTA ACCTT ACCTC ACCTG ACCCA ACCCT
ACCCC
ACCCGACCGA ACCGT ACCGC ACCGG
ACG ACGAA ACGAT ACGAC ACGAG ACGTA ACGTT ACGTC ACGTG ACGCA ACGCT
ACGCC
ACGCG ACGGA ACGGT ACGGC ACGGG
AGA AGAAA AGAAT AGAAC AGAAG AGATA AGATT AGATC AGATG AGACA AGACT

AGACC
AGACG AGAGA AGAGT AGAGC AGAGG
AGT AGTAA AGTAT AGTAC AGTAG AGTTA AGTTT AGTTC AGTTG AGTCA AGTCT
AGTCC
AGTCGAGTGA AGTGT AGTGC AGTGG
AGC AGCAA AGCAT AGCAC AGCAG AGCTA AGCTT AGCTC AGCTG AGCCA AGCCT
AGCCC
AGCCG AGCGA AGCGT AGCGC AGCGG
AGG AGGAA AGGAT AGGAC AGGAG AGGTA AGGTT AGGTC AGGTG AGGCA AGGCT
AGGCC
AGGCG AGGGA AGGGT AGGGC AGGGG
TAA TAAAA TAAAT TAAAC TAAAG TAATA TAATT TAATC TAATG TAACA TAACT
TAACC
TAACGTAAGA TAAGT TAAGC TAAGG
TAT TATAA TATAT TATAC TATAG TATTA TATTT TATTC TATTG TATCA TATCT TATCC
TATCG TATGA TATGT TATGC TATGG
TAC TACAA TACAT TACAC TACAG TACTA TACTT TACTC TACTG TACCA TACCT TACCC
TACCG TACGA TACGT TACGC TACGG
TAG TAGAA TAGAT TAGAC TAGAG TAGTA TAGTT TAGTC TAGTG TAGCA TAGCT
TAGCC
TAGCGTAGGA TAGGT TAGGC TAGGG
TTA TTAAA TTAAT TTAAC TTAAG TTATA TTATT TTATC TTATG TTACA TTACT TTACC
TTACG TTAGA TTAGT TTAGC TTAGG
TTT TTTAA TTTAT TTTAC TTTAG TTTTA TTTTT TTTTC TTTTG TTTCA TTTCT TTTCC
TTTCG TTTGA TTTGT TTTGC TTTGG
TTC TTCAA TTCAT TTCAC TTCAG TTCTA TTCTT TTCTC TTCTG TTCCA TTCCT TTCCC
TTCCG TTCGA TTCGT TTCGC TTCGG
TTG TTGAA TTGAT TTGAC TTGAG TTGTA TTGTT TTGTC TTGTG TTGCA TTGCT TTGCC
TTGCG TTGGA TTGGT TTGGC TTGGG
TCA TCAAA TCAAT TCAAC TCAAG TCATA TCATT TCATC TCATG TCACA TCACT TCACC
TCACG TCAGA TCAGT TCAGC TCAGG
TCT TCTAA TCTAT TCTAC TCTAG TCTTA TCTTT TCTTC TCTTG TCTCA TCTCT TCTCC
TCTCG TCTGA TCTGT TCTGC TCTGG
TCC TCCAA TCCAT TCCAC TCCAG TCCTA TCCTT TCCTC TCCTG TCCCA TCCCT TCCCC
TCCCG TCCGA TCCGT TCCGC TCCGG
TCG TCGAA TCGAT TCGAC TCGAG TCGTA TCGTT TCGTC TCGTG TCGCA TCGCT TCGCC
TCGCG TCGGA TCGGT TCGGC TCGGG
TGA TGAAA TGAAT TGAAC TGAAG TGATA TGATT TGATC TGATG TGACA TGACT
TGACC
TGACGTGAGA TGAGT TGAGC TGAGG
TGT TGTAA TGTAT TGTAC TGTAG TGTTA TGTTT TGTTC TGTTG TGTCA TGTCT TGTCC
TGTCG TGTGA TGTGT TGTGC TGTGG
TGC TGCAA TGCAT TGCAC TGCAG TGCTA TGCTT TGCTC TGCTG TGCCA TGCCT TGCCC
TGCCG TGCGA TGCGT TGCGC TGCGG
TGG TGGAA TGGAT TGGAC TGGAG TGGTA TGGTT TGGTC TGGTG TGGCA TGGCT
TGGCC
TGGCGTGGGA TGGGT TGGGC TGGGG
CAA CAAAA CAAAT CAAAC CAAAG CAATA CAATT CAATC CAATG CAACA CAACT
CAACC
CAACG CAAGA CAAGT CAAGC CAAGG
CAT CATAA CATAT CATAC CATAG CATTA CATTT CATTC CATTG CATCA CATCT CATCC
CATCGCATGA CATGT CATGC CATGG
CAC CACAA CACAT CACAC CACAG CACTA CACTT CACTC CACTG CACCA CACCT
CACCC
CACCGCACGA CACGT CACGC CACGG
CAG CAGAA CAGAT CAGAC CAGAG CAGTA CAGTT CAGTC CAGTG CAGCA CAGCT
CAGCC
CAGCG CAGGA CAGGT CAGGC CAGGG

CTA CTAAA CTAAT CTAAC CTAAG CTATA CTATT CTATC CTATG CTACA CTACT CTACC
CTACG CTAGA CTAGT CTAGC CTAGG
CTT CTTAA CTTAT CTTAC CTTAG CTTTA CTTTT CTTTC CTTTG CTTCA CTTCT CTTCC
CTTCG CTTGA CTTGT CTTGC CTTGG
CTC CTCAA CTCAT CTCAC CTCAG CTCTA CTCTT CTCTC CTCTG CTCCA CTCCT CTCCC
CTCCG CTCGA CTCGT CTCGC CTCGG
CTG CTGAA CTGAT CTGAC CTGAG CTGTA CTGTT CTGTC CTGTG CTGCA CTGCT CTGCC
CTGCG CTGGA CTGGT CTGGC CTGGG
CCA CCAAA CCAAT CCAAC CCAAG CCATA CCATT CCATC CCATG CCACA CCACT
CCACC
CCACGCCAGA CCAGT CCAGC CCAGG
CCT CCTAA CCTAT CCTAC CCTAG CCTTA CCTTT CCTTC CCTTG CCTCA CCTCT CCTCC
CCTCG CCTGA CCTGT CCTGC CCTGG
CCC CCCAA CCCAT CCCAC CCCAG CCCTA CCCTT CCCTC CCCTG CCCCA CCCCT CCCCC
CCCCG CCCGA CCCGT CCCGC CCCGG
CCG CCGAA CCGAT CCGAC CCGAG CCGTA CCGTT CCGTC CCGTG CCGCA CCGCT
CCGCC
CCGCGCCGGA CCGGT CCGGC CCGGG
CGA CGAAA CGAAT CGAAC CGAAG CGATA CGATT CGATC CGATG CGACA CGACT
CGACC
CGACG CGAGA CGAGT CGAGC CGAGG
CGT CGTAA CGTAT CGTAC CGTAG CGTTA CGTTT CGTTC CGTTG CGTCA CGTCT CGTCC
CGTCG CGTGA CGTGT CGTGC CGTGG
CGC CGCAA CGCAT CGCAC CGCAG CGCTA CGCTT CGCTC CGCTG CGCCA CGCCT
CGCCC
CGCCGCGCGA CGCGT CGCGC CGCGG
CGG CGGAA CGGAT CGGAC CGGAG CGGTA CGGTT CGGTC CGGTG CGGCA CGGCT
CGGCC
CGGCG CGGGA CGGGT CGGGC CGGGG
GAA GAAAA GAAAT GAAAC GAAAG GAATA GAATT GAATC GAATG GAACA GAACT
GAACC
GAACG GAAGA GAAGT GAAGC GAAGG
GAT GATAA GATAT GATAC GATAG GATTA GATTT GATTC GATTG GATCA GATCT
GATCC
GATCGGATGA GATGT GATGC GATGG
GAC GACAA GACAT GACAC GACAG GACTA GACTT GACTC GACTG GACCA GACCT
GACCC
GACCG GACGA GACGT GACGC GACGG
GAG GAGAA GAGAT GAGAC GAGAG GAGTA GAGTT GAGTC GAGTG GAGCA GAGCT
GAGCC
GAGCG GAGGA GAGGT GAGGC GAGGG
GTA GTAAA GTAAT GTAAC GTAAG GTATA GTATT GTATC GTATG GTACA GTACT
GTACC
GTACGGTAGA GTAGT GTAGC GTAGG
GTT GTTAA GTTAT GTTAC GTTAG GTTTA GTTTT GTTTC GTTTG GTTCA GTTCT GTTCC
GTTCG GTTGA GTTGT GTTGC GTTGG
GTC GTCAA GTCAT GTCAC GTCAG GTCTA GTCTT GTCTC GTCTG GTCCA GTCCT GTCCC
GTCCG GTCGA GTCGT GTCGC GTCGG
GTG GTGAA GTGAT GTGAC GTGAG GTGTA GTGTT GTGTC GTGTG GTGCA GTGCT
GTGCC
GTGCGGTGGA GTGGT GTGGC GTGGG
GCA GCAAA GCAAT GCAAC GCAAG GCATA GCATT GCATC GCATG GCACA GCACT
GCACC
GCACG GCAGA GCAGT GCAGC GCAGG
GCT GCTAA GCTAT GCTAC GCTAG GCTTA GCTTT GCTTC GCTTG GCTCA GCTCT GCTCC
GCTCG GCTGA GCTGT GCTGC GCTGG
GCC GCCAA GCCAT GCCAC GCCAG GCCTA GCCTT GCCTC GCCTG GCCCA GCCCT
GCCCC

GCCCGGCCGA GCCGT GCCGC GCCGG
GCG GCGAA GCGAT GCGAC GCGAG GCGTA GCGTT GCGTC GCGTG GCGCA GCGCT GCGCC
GCGCG GCGGA GCGGT GCGGC GCGGG
GGA GGAAA GGAAT GGAAC GGAAG GGATA GGATT GGATC GGATG GGACA GGACT GGACC
GGACG GGAGA GGAGT GGAGC GGAGG
GGT GGTAA GGTAT GGTAC GGTAG GGTTA GGTTT GGTTC GGTTG GGTCA GGTCT GGTCC
GGTCGGGTGA GGTGT GGTGC GGTGG
GGC GGCAA GGCAT GGCAC GGCAG GGCTA GGCTT GGCTC GGCTG GGCCA GGCCT GGCCC
GGCCG GGCGA GGCGT GGCGC GGCGG
GGG GGGAA GGGAT GGGAC GGGAG GGGTA GGGTT GGGTC GGGTG GGGCA GGGCT GGGCC
GGGCG GGGGA GGGGT GGGGC GGGGG

After filtering: read1: overrepresented sequences
Sampling rate: 1 / 20
overrepresented sequence count (% of bases) distribution: cycle 1 ~ cycle 151
AAAAAAAAAAAAAAAAAAAAAAAAAAAAAAAAAAAAAAAA 3616 (0.037536%)

AAAATTCTGTGACAAATTTTTGGTCAAGTTGTTTCCATTAAAAAGTACTGATTTTAAAAACTAATAACTTAAAACTGCCACACGCAAAAAAGAAAACCAA 42 (0.001090%)

AAACTTGCATGCAATGTGAGCCGTGTGGCAATCCAATACAGGGGCATAGCCGGCGCTTATTTGGCCTGGATGGTTCAGGATAATCACCTGAGCAGTGAAG 3 (0.000078%)

AAATATGTAGAGGGAGTATAGGGCTGTGACTAGTATGTTGAGTCCTGTAAGTAGGAGAGTGATATTTGATCAGGAGAACGTGGTTACTAGCACAGAGAGT 5 (0.000130%)

AAATTCTGTGACAAATTTTTGGTCAAGTTGTTTCCATTAAAAAGTACTGATTTTAAAAACTAATAACTTAAAACTGCCACACGCAAAAAAGAAAACCAAA 11 (0.000285%)

AAATTTCCACAAGGAGATATCAATGGTGATACCACGTTCACGCTCAGCTTTCAGTTTATCCAAGACCCAGGCATACTTGAAGGAGCCCTTTCCCATCTCA 10 (0.000260%)

AAATTTTTCAATGGTTCTTTTGTCGATGCCACCGCATTTATAGATCAGATGGCCAGTAGTGGTGGACTTGCCCGAATCTACGTGTCCAATGACGACAATG 10 (0.000260%)

AAATTTTTGGTCAAGTTGTTTCCATTAAAAAGTACTGATTTTAAAAACTAATAACTTAAAACTGCCACACGCAAAAAAGAAAACCAAAGTGGTCCACAAA 8 (0.000208%)

AACAATGGCAGCATCACCAGACTTCAAGAATTTAGGGCCATCTTCCAGCTTTTTACCAGAACGGCGATCAATCTTTTCCTTCAGCTCAGCAAACTTGCAT 6 (0.000156%)

AACAATTTCCTCATATCTCTTCTGGCTGTAGGGTGGCTCAGTGGAATCCATTTTGTTAACACCGACAATTAGTTGTTTCACACCCAGTGTGTAAGCCAGA 10 (0.000260%)

AACCAGTCTTTTACTACTAAACTTAAATGGCCAATTGAAACAAACAGTTCTGAGACCGTTCTTCCACCACTGATTAAGAGTGGGGTGGCAGGTATTAGGG 9 (0.000234%)

AACCATATCAACAATGGCAGCATCACCAGACTTCAAGAATTTAGGGCCATCTTCCAGCTTTTTACCAGAACGGCGATCAATCTTTTCCTTCAGCTCAGCA 17 (0.000441%)

AAGAATTTAGGGCCATCTTCCAGCTTTTTACCAGAACGGCGATCAATCTTTTCCTTCAGCTCAGCAAACTTGCATGCAATGTGAGCCGTGTGGCAATCCA 6 (0.000156%)

AAGTTGTTTCCATTAAAAAGTACTGATTTTAAAAACTAATAACTTAAAACTGCCACACGCAAAAAAGAA
AACCAAAGTGGTCCACAAAACATTCTCCTTT 4 (0.000104%)

AATACAATGCCAGTCAGGCCACCTACGGTGAAAAGAAAGATGAATCCTAGGGCTCAGAGCACTGCAGC
AGATCATTTCATATTGCTTCCGTGGAGTGTGG 5 (0.000130%)

AATATGTAGAGGGAGTATAGGGCTGTGACTAGTATGTTGAGTCCTGTAAGTAGGAGAGTGATATTTGAT
CAGGAGAACGTGGTTACTAGCACAGAGAGTT 8 (0.000208%)

AATATTCATTTAGCCTTCTGAGCTTTCTGGGCAGACTTGGTGACCTTGCCAGCTCCAGCAGCCTTCTTGT
CCACTGCTTTGATGACACCCACCGCAACTG 7 (0.000182%)

AATCACCTGAGCAGTGAAGCCAGCTGCTTCCATTGGTGGGTCATTTTTGCTGTCACCAGCAACGTTGCC
ACGACGAACATCCTTGACAGACACATTCTTG 6 (0.000156%)

AATCTTTTCCTTCAGCTCAGCAAACTTGCATGCAATGTGAGCCGTGTGGCAATCCAATACAGGGGCATA
GCCGGCGCTTATTTGGCCTGGATGGTTCAGG 12 (0.000311%)

AATGGCAGAATTTGGCTGTTTGGCTTCAACTCCTACTTTTTCCAGCACGATTCCTTTTGCATGAGAAGCA
CCTCCAAAAGGGTTGGCCTTTAGGGCTGTG 3 (0.000078%)

AATGGGTCTCAAAATTCTGTGACAAATTTTTGGTCAAGTTGTTTCCATTAAAAAGTACTGATTTTAAAAA
CTAATAACTTAAAACTGCCACACGCAAAAA 7 (0.000182%)

AATGTCACAGGTCCAGGGCAGAGGACCAACATGGGCATTTTGTTTATGAGCAAGGTGGGTCTCAGAGG
TGATCGGCGATCAGAGGGCGATGAAGTTCTAG 3 (0.000078%)

AATGTGAGCCGTGTGGCAATCCAATACAGGGGCATAGCCGGCGCTTATTTGGCCTGGATGGTTCAGGAT
AATCACCTGAGCAGTGAAGCCAGCTGCTTCC 5 (0.000130%)

AATTCTGTGACAAATTTTTGGTCAAGTTGTTTCCATTAAAAAGTACTGATTTTAAAAACTAATAACTTAA
AACTGCCACACGCAAAAAAGAAAACCAAAG 10 (0.000260%)

AATTTCCACAAGGAGATATCAATGGTGATACCACGTTCACGCTCAGCTTTCAGTTTATCCAAGACCCAG
GCATACTTGAAGGAGCCCTTTCCCATCTCAG 8 (0.000208%)

AATTTCCTCATATCTCTTCTGGCTGTAGGGTGGCTCAGTGGAATCCATTTTGTTAACACCGACAATTAGT
TGTTTCACACCCAGTGTGTAAGCCAGAAGG 9 (0.000234%)

AATTTTCTTAATGTAAGTGCTGACTTCCTTAACAATTTCCTCATATCTCTTCTGGCTGTAGGGTGGCTCAG
TGGAATCCATTTTGTTAACACCGACAATT 20 (0.000519%)

AATTTTTCAATGGTTCTTTTGTCGATGCCACCGCATTTATAGATCAGATGGCCAGTAGTGGTGGACTTGC
CCGAATCTACGTGTCCAATGACGACAATGT 4 (0.000104%)

AATTTTTGGTCAAGTTGTTTCCATTAAAAAGTACTGATTTTAAAAACTAATAACTTAAAACTGCCACACG
CAAAAAAGAAAACCAAAGTGGTCCACAAAA 3 (0.000078%)

ACAATGCCAGTCAGGCCACCTACGGTGAAAAGAAAGATGAATCCTAGGGCTCAGAGCACTGCAGCAGA
TCATTTCATATTGCTTCCGTGGAGTGTGGCGA 5 (0.000130%)

ACAATTTCCTCATATCTCTTCTGGCTGTAGGGTGGCTCAGTGGAATCCATTTTGTTAACACCGACAATTA
GTTGTTTCACACCCAGTGTGTAAGCCAGAA 8 (0.000208%)

ACACACTTCCTAATGGCAGAATTTGGCTGTTTGGCTTCAACTCCTACTTTTTCCAGCACGATTCCTTTTGC
ATGAGAAGCACCTCCAAAAGGGTTGGCCT 4 (0.000104%)

ACACGAAGGCCCCAGAAGTGACGCAGCCCTCTATGGGCCCGAATCTTCTTCAGTCGCTCCAGGTCTTCA
CGGAGCTTGTTGTCCAGACCATTGGCTAGGA 3 (0.000078%)

ACACTTCCTAATGGCAGAATTTGGCTGTTTGGCTTCAACTCCTACTTTTTCCAGCACGATTCCTTTTGCAT
GAGAAGCACCTCCAAAAGGGTTGGCCTTT 5 (0.000130%)

ACAGATATTCAGCATCTAACAGTTCAAAAGAAGCCACTACATACTCTTTTCACAAATATGTTTTCACAG
AGCCAATACAGTACTAGCCATTAACCCAGTA 4 (0.000104%)

ACAGCGATTTCTAGGATAGTCAGTAGAATTAGAATTGTGAAGATGATAAGTGTAGAGGGAAGGTTAAT
GGTTGATATTGCTAGGGTGGCGCTTCCAATTA 3 (0.000078%)

ACAGGTCCAGGGCAGAGGACCAACATGGGCATTTTGTTTATGAGCAAGGTGGGTCTCAGAGGTGATCG
GCGATCAGAGGGCGATGAAGTTCTAGATCCAT4 (0.000104%)

ACCAGACTTCAAGAATTTAGGGCCATCTTCCAGCTTTTTACCAGAACGGCGATCAATCTTTTCCTTCAGC
TCAGCAAACTTGCATGCAATGTGAGCCGTG 9 (0.000234%)

ACCAGTCTTTTACTACTAAACTTAAATGGCCAATTGAAACAAACAGTTCTGAGACCGTTCTTCCACCACT
GATTAAGAGTGGGGTGGCAGGTATTAGGGA 9 (0.000234%)

ACCATATCAACAATGGCAGCATCACCAGACTTCAAGAATTTAGGGCCATCTTCCAGCTTTTTACCAGAA
CGGCGATCAATCTTTTCCTTCAGCTCAGCAA 18 (0.000467%)

ACCTACGGTGAAAAGAAAGATGAATCCTAGGGCTCAGAGCACTGCAGCAGATCATTTCATATTGCTTCC
GTGGAGTGTGGCGAGTCAGCTAAATACTTTG 4 (0.000104%)

ACCTGAGCAGTGAAGCCAGCTGCTTCCATTGGTGGGTCATTTTTGCTGTCACCAGCAACGTTGCCACGA
CGAACATCCTTGACAGACACATTCTTGACAT 3 (0.000078%)

ACCTTGCCAGCTCCAGCAGCCTTCTTGTCCACTGCTTTGATGACACCCACCGCAACTGTCTGTCTCATAT
CACGAACAGCAAAGCGACCCAAAGGTGGAT 6 (0.000156%)

ACTAAACTTAAATGGCCAATTGAAACAAACAGTTCTGAGACCGTTCTTCCACCACTGATTAAGAGTGGG
GTGGCAGGTATTAGGGATAATATTCATTTAG 6 (0.000156%)

ACTGCCCACGGTGGGCGGGCGGGCCTCTCTACTCGAAGGTGACCACGTTTAGATTCTGAGACGGGAAGT
GGAGGGTGAATAGGTCACGGCGGCCTTTTTT 4 (0.000104%)

ACTTGCATGCAATGTGAGCCGTGTGGCAATCCAATACAGGGGCATAGCCGGCGCTTATTTGGCCTGGAT
GGTTCAGGATAATCACCTGAGCAGTGAAGCC 3 (0.000078%)

ACTTGCTGGTCTCAAATTTCCACAAGGAGATATCAATGGTGATACCACGTTCACGCTCAGCTTTCAGTTT
ATCCAAGACCCAGGCATACTTGAAGGAGCC 1172 (0.030415%)

ACTTGGTGACCTTGCCAGCTCCAGCAGCCTTCTTGTCCACTGCTTTGATGACACCCACCGCAACTGTCTG
TCTCATATCACGAACAGCAAAGCGACCCAA 9 (0.000234%)

AGAATGTCACAGGTCCAGGGCAGAGGACCAACATGGGCATTTTGTTTATGAGCAAGGTGGGTCTCAGA
GGTGATCGGCGATCAGAGGGCGATGAAGTTCT 15 (0.000389%)

AGAATTAGAATTGTGAAGATGATAAGTGTAGAGGGAAGGTTAATGGTTGATATTGCTAGGGTGGCGCTT
CCAATTAGGTGCATGAGTAGGTGGCCTGCAG 9 (0.000234%)

AGAATTTGGCTGTTTGGCTTCAACTCCTACTTTTTCCAGCACGATTCCTTTTGCATGAGAAGCACCTCCA
AAAGGGTTGGCCTTTAGGGCTGTGCCCAAA 6 (0.000156%)

AGACGATGCCAGTGCCCCTGGGTGCAGGGATGAGGCGTACCAGCACAGAGCCGCAGCGGCCTGTCACC

TTGCAAGGGACAGTGTGGGGCTTGCCGATCTT 15 (0.000389%)

AGACTTCAAGAATTTAGGGCCATCTTCCAGCTTTTACCAGAACGGCGATCAATCTTTTCCTTCAGCTCA
GCAAACTTGCATGCAATGTGAGCCGTGTGG 9 (0.000234%)

AGACTTGGTGACCTTGCCAGCTCCAGCAGCCTTCTTGTCCACTGCTTTGATGACACCCACCGCAACTGTC
TGTCTCATATCACGAACAGCAAAGCGACCC 9 (0.000234%)

AGAGAGTGAGGAGAAGGCTTACGTTTAGTGAGGGAGAGATTTGGTATATGATTGAGATGGGGGCTAGT
TTTTGTCATGTGAGAAGAAGCAGGCCGGATGT 4 (0.000104%)

AGAGGACCAACATGGGCATTTTGTTTATGAGCAAGGTGGGTCTCAGAGGTGATCGGCGATCAGAGGGC
GATGAAGTTCTAGATCCATTGAGACAAGCTCT 9 (0.000234%)

AGAGGGAGTATAGGGCTGTGACTAGTATGTTGAGTCCTGTAAGTAGGAGAGTGATATTTGATCAGGAG
AACGTGGTTACTAGCACAGAGAGTTCTCCCAG 5 (0.000130%)

AGAGTGAGGAGAAGGCTTACGTTTAGTGAGGGAGAGATTTGGTATATGATTGAGATGGGGGCTAGTTTT
TGTCATGTGAGAAGAAGCAGGCCGGATGTCA 4 (0.000104%)

AGCAAACTTGCATGCAATGTGAGCCGTGTGGCAATCCAATACAGGGGCATAGCCGGCGCTTATTTGGCC
TGGATGGTTCAGGATAATCACCTGAGCAGTG 21 (0.000545%)

AGCAGCAACAATCAGGACAGCACAGTCAGCCTGAGATGTCCCTGTAATCATGTTTTTGATAAAGTCTCT
GTGTCCTGGGGCATCAATGATAGTCACATAG 25 (0.000649%)

AGCAGCCTTCTTGTCCACTGCTTTGATGACACCCACCGCAACTGTCTGTCTCATATCACGAACAGCAAA
GCGACCCAAAGGTGGATAGTCTGAGAAGCTC 6 (0.000156%)

AGCAGTGAAGCCAGCTGCTTCCATTGGTGGGTCATTTTTGCTGTCACCAGCAACGTTGCCACGACGAAC
ATCCTTGACAGACACATTCTTGACATTGAAG 4 (0.000104%)

AGCATCACCAGACTTCAAGAATTTAGGGCCATCTTCCAGCTTTTACCAGAACGGCGATCAATCTTTTCC
TTCAGCTCAGCAAACTTGCATGCAATGTGA 13 (0.000337%)

AGCCAATTTTCTTAATGTAAGTGCTGACTTCCTTAACAATTTCCTCATATCTCTTCTGGCTGTAGGGTGG
CTCAGTGGAATCCATTTTGTTAACACCGAC 10 (0.000260%)

AGCCGTGTGGCAATCCAATACAGGGGCATAGCCGGCGCTTATTTGGCCTGGATGGTTCAGGATAATCAC
CTGAGCAGTGAAGCCAGCTGCTTCCATTGGT 4 (0.000104%)

AGCCTTCTGAGCTTTCTGGGCAGACTTGGTGACCTTGCCAGCTCCAGCAGCCTTCTTGTCCACTGCTTTG
ATGACACCCACCGCAACTGTCTGTCTCATA 19 (0.000493%)

AGCGATTTCTAGGATAGTCAGTAGAATTAGAATTGTGAAGATGATAAGTGTAGAGGGAAGGTTAATGG
TTGATATTGCTAGGGTGGCGCTTCCAATTAGG 32 (0.000830%)

AGCTCAGCAAACTTGCATGCAATGTGAGCCGTGTGGCAATCCAATACAGGGGCATAGCCGGCGCTTATT
TGGCCTGGATGGTTCAGGATAATCACCTGAG 5 (0.000130%)

AGCTCCAGCAGCCTTCTTGTCCACTGCTTTGATGACACCCACCGCAACTGTCTGTCTCATATCACGAACA
GCAAAGCGACCCAAAGGTGGATAGTCTGAG 6 (0.000156%)

AGCTTCTTAGGCACAGGTGCGGAGACGATGCCAGTGCCCCTGGGTGCAGGGATGAGGCGTACCAGCAC
AGAGCCGCAGCGGCCTGTCACCTTGCAAGGGA 522 (0.013547%)

AGCTTTCTGGGCAGACTTGGTGACCTTGCCAGCTCCAGCAGCCTTCTTGTCCACTGCTTTGATGACACCC
ACCGCAACTGTCTGTCTCATATCACGAACA 8 (0.000208%)

AGCTTTTTACCAGAACGGCGATCAATCTTTTCCTTCAGCTCAGCAAACTTGCATGCAATGTGAGCCGTGT GGCAATCCAATACAGGGGCATAGCCGGCGC 5 (0.000130%)

AGGAACCATATCAACAATGGCAGCATCACCAGACTTCAAGAATTTAGGGCCATCTTCCAGCTTTTTACC AGAACGGCGATCAATCTTTTCCTTCAGCTCA 25 (0.000649%)

AGGACCAACATGGGCATTTTGTTTATGAGCAAGGTGGGTCTCAGAGGTGATCGGCGATCAGAGGGCGA TGAAGTTCTAGATCCATTGAGACAAGCTCTAG 3 (0.000078%)

AGGAGATATCAATGGTGATACCACGTTCACGCTCAGCTTTCAGTTTATCCAAGACCCAGGCATACTTGA AGGAGCCCTTTCCCATCTCAGCAGCCTCCTT 4 (0.000104%)

AGGATAATCACCTGAGCAGTGAAGCCAGCTGCTTCCATTGGTGGGTCATTTTTGCTGTCACCAGCAACG TTGCCACGACGAACATCCTTGACAGACACAT 22 (0.000571%)

AGGCCACCTACGGTGAAAAGAAAGATGAATCCTAGGGCTCAGAGCACTGCAGCAGATCATTTCATATT GCTTCCGTGGAGTGTGGCGAGTCAGCTAAATA 23 (0.000597%)

AGGGAGTATAGGGCTGTGACTAGTATGTTGAGTCCTGTAAGTAGGAGAGTGATATTTGATCAGGAGAA CGTGGTTACTAGCACAGAGAGTTCTCCCAGTA 11 (0.000285%)

AGGGATAATATTCATTTAGCCTTCTGAGCTTTCTGGGCAGACTTGGTGACCTTGCCAGCTCCAGCAGCCT TCTTGTCCACTGCTTTGATGACACCCACCG 11 (0.000285%)

AGGGCAGAGGACCAACATGGGCATTTTGTTTATGAGCAAGGTGGGTCTCAGAGGTGATCGGCGATCAG AGGGCGATGAAGTTCTAGATCCATTGAGACAA 3 (0.000078%)

AGGGTGTAGCCTGAGAATAGGGGAAATCAGTGAATGAAGCCTCCTATGATGGCAAATACAGCTCCTAT TGATAGGACATAGTGGAAGTGAGCTACAACGT 5 (0.000130%)

AGGTCCAGGGCAGAGGACCAACATGGGCATTTTGTTTATGAGCAAGGTGGGTCTCAGAGGTGATCGGC GATCAGAGGGCGATGAAGTTCTAGATCCATTG3 (0.000078%)

AGGTTTGGTCTAGGGTGTAGCCTGAGAATAGGGGAAATCAGTGAATGAAGCCTCCTATGATGGCAAAT ACAGCTCCTATTGATAGGACATAGTGGAAGTG4 (0.000104%)

AGTAGAATTAGAATTGTGAAGATGATAAGTGTAGAGGGAAGGTTAATGGTTGATATTGCTAGGGTGGC GCTTCCAATTAGGTGCATGAGTAGGTGGCCTG 5 (0.000130%)

AGTAGTTCCCTGCTAAGGGAGGGTAGACTGTTCAACCTGTTCCTGCTCCGGCCTCCACTATAGCAGATG CGAGCAGGAGTAGGAGAGAGGGAGGTAAGAG 9 (0.000234%)

AGTCAGGCCACCTACGGTGAAAAGAAAGATGAATCCTAGGGCTCAGAGCACTGCAGCAGATCATTTCA TATTGCTTCCGTGGAGTGTGGCGAGTCAGCTA 22 (0.000571%)

AGTCAGTAGAATTAGAATTGTGAAGATGATAAGTGTAGAGGGAAGGTTAATGGTTGATATTGCTAGGG TGGCGCTTCCAATTAGGTGCATGAGTAGGTGG 4 (0.000104%)

AGTTGTTTCCATTAAAAAGTACTGATTTTAAAAACTAATAACTTAAAACTGCCACACGCAAAAAAGAAA ACCAAAGTGGTCCACAAAACATTCTCCTTTC 4 (0.000104%)

ATAATATTCATTTAGCCTTCTGAGCTTTCTGGGCAGACTTGGTGACCTTGCCAGCTCCAGCAGCCTTCTT GTCCACTGCTTTGATGACACCCACCGCAAC 94 (0.002439%)

ATAATCACCTGAGCAGTGAAGCCAGCTGCTTCCATTGGTGGGTCATTTTTGCTGTCACCAGCAACGTTGC CACGACGAACATCCTTGACAGACACATTCT 8 (0.000208%)

ATACAATGCCAGTCAGGCCACCTACGGTGAAAAGAAAGATGAATCCTAGGGCTCAGAGCACTGCAGCA GATCATTTCATATTGCTTCCGTGGAGTGTGGC 10 (0.000260%)

ATAGTCAGTAGAATTAGAATTGTGAAGATGATAAGTGTAGAGGGAAGGTTAATGGTTGATATTGCTAG GGTGGCGCTTCCAATTAGGTGCATGAGTAGGT 8 (0.000208%)

ATATCAACAATGGCAGCATCACCAGACTTCAAGAATTTAGGGCCATCTTCCAGCTTTTACCAGAACGG CGATCAATCTTTTCCTTCAGCTCAGCAAACT 9 (0.000234%)

ATATTCATTTAGCCTTCTGAGCTTTCTGGGCAGACTTGGTGACCTTGCCAGCTCCAGCAGCCTTCTTGTC CACTGCTTTGATGACACCCACCGCAACTGT 5 (0.000130%)

ATCAACAATGGCAGCATCACCAGACTTCAAGAATTTAGGGCCATCTTCCAGCTTTTACCAGAACGGCG ATCAATCTTTTCCTTCAGCTCAGCAAACTTG 22 (0.000571%)

ATCACCAGACTTCAAGAATTTAGGGCCATCTTCCAGCTTTTACCAGAACGGCGATCAATCTTTTCCTTC AGCTCAGCAAACTTGCATGCAATGTGAGCC 4 (0.000104%)

ATCAGAGGGCGATGAAGTTCTAGATCCATTGAGACAAGCTCTAGACAGTAGCATGCAGTCCCACAACTT GTACCAGCATCCCCAGCGTCTGGCATTCCAT 4 (0.000104%)

ATCCATGTGACCTTCTCTGGCATTCGGGCATTGGCTGTACCCTTCCGCTTACCTATGCCCATGTGCCTGC CCTTCCGGCGGGCCAAGGTGTTTTTCCGGC 7 (0.000182%)

ATCGGAAGAGCACACGTCTGAACTCCAGTCACATCGCAATTGCGATCTGGGGGGGGGGGGGGGGGG GGGGGGGGGGGGGGGGGGGGGGGGGGGGGGGGGGGGGGGGGGGGGGGGGGGGGGGGGGGGGG GGGGGGGGGGGGGGGG 11 (0.000425%)

ATCTGCGGGTGGCACTGCCCACGGTGGGCGGGCGGGCCTCTCTACTCGAAGGTGACCACGTTTAGATTC TGAGACGGGAAGTGGAGGGTGAATAGGTCAC 3 (0.000078%)

ATCTTTTCCTTCAGCTCAGCAAACTTGCATGCAATGTGAGCCGTGTGGCAATCCAATACAGGGGCATAG CCGGCGCTTATTTGGCCTGGATGGTTCAGGA 8 (0.000208%)

ATGCAATGTGAGCCGTGTGGCAATCCAATACAGGGGCATAGCCGGCGCTTATTTGGCCTGGATGGTTCA GGATAATCACCTGAGCAGTGAAGCCAGCTGC 9 (0.000234%)

ATGCCAGTCAGGCCACCTACGGTGAAAAGAAAGATGAATCCTAGGGCTCAGAGCACTGCAGCAGATCA TTTCATATTGCTTCCGTGGAGTGTGGCGAGTC 4 (0.000104%)

ATGCCAGTGCCCCTGGGTGCAGGGATGAGGCGTACCAGCACAGAGCCGCAGCGGCCTGTCACCTTGCA AGGGACAGTGTGGGGCTTGCCGATCTTGTTCC 3 (0.000078%)

ATGGCAGAATTTGGCTGTTTGGCTTCAACTCCTACTTTTTCCAGCACGATTCCTTTTGCATGAGAAGCAC CTCCAAAAGGGTTGGCCTTTAGGGCTGTGC 3 (0.000078%)

ATGGCAGCATCACCAGACTTCAAGAATTTAGGGCCATCTTCCAGCTTTTACCAGAACGGCGATCAATC TTTTCCTTCAGCTCAGCAAACTTGCATGCAA 4 (0.000104%)

ATGGGTCTCAAAATTCTGTGACAAATTTTTGGTCAAGTTGTTTCCATTAAAAAGTACTGATTTTAAAAAC TAATAACTTAAAACTGCCACACGCAAAAAA 29 (0.000753%)

ATGTAGAGGGAGTATAGGGCTGTGACTAGTATGTTGAGTCCTGTAAGTAGGAGAGTGATATTTGATCAG GAGAACGTGGTTACTAGCACAGAGAGTTCTC 6 (0.000156%)

ATGTGGGGACAGCTCATGAGTGCAAGACGTCTTGTGATGTAATTATTATACGAATGGGGGCTTCAATCG GGAGTACTACTCGATTGTCAACGTCAAGGAG 5 (0.000130%)

ATTAACCAGTCTTTTACTACTAAACTTAAATGGCCAATTGAAACAAACAGTTCTGAGACCGTTCTTCCAC
CACTGATTAAGAGTGGGGTGGCAGGTATTA 963 (0.024991%)

ATTAGGGATAATATTCATTTAGCCTTCTGAGCTTTCTGGGCAGACTTGGTGACCTTGCCAGCTCCAGCAG
CCTTCTTGTCCACTGCTTTGATGACACCCA 964 (0.025017%)

ATTCATTTAGCCTTCTGAGCTTTCTGGGCAGACTTGGTGACCTTGCCAGCTCCAGCAGCCTTCTTGTCCA
CTGCTTTGATGACACCCACCGCAACTGTCT 245 (0.006358%)

ATTCTGTGACAAATTTTTGGTCAAGTTGTTTCCATTAAAAAGTACTGATTTTAAAAACTAATAACTTAAA
ACTGCCACACGCAAAAAAGAAAACCAAAGT 8 (0.000208%)

ATTGAGAGAGTGAGGAGAAGGCTTACGTTTAGTGAGGGAGAGATTTGGTATATGATTGAGATGGGGGC
TAGTTTTTGTCATGTGAGAAGAAGCAGGCCGG 6 (0.000156%)

ATTGTGTTGTGGTAAATATGTAGAGGGAGTATAGGGCTGTGACTAGTATGTTGAGTCCTGTAAGTAGGA
GAGTGATATTTGATCAGGAGAACGTGGTTAC 16 (0.000415%)

ATTTAGCCTTCTGAGCTTTCTGGGCAGACTTGGTGACCTTGCCAGCTCCAGCAGCCTTCTTGTCCACTGC
TTTGATGACACCCACCGCAACTGTCTGTCT 88 (0.002284%)

ATTTAGGGCCATCTTCCAGCTTTTACCAGAACGGCGATCAATCTTTTCCTTCAGCTCAGCAAACTTGCA
TGCAATGTGAGCCGTGTGGCAATCCAATAC 15 (0.000389%)

ATTTCAAAGATTTTTAGGGGAATTAATTCTAGGACGATGGGCATGAAACTGTGGTTTGCTCCACAGATT
TCAGAGCATTGACCGTAGTATACCCCCGGTC 41 (0.001064%)

ATTTCCACAAGGAGATATCAATGGTGATACCACGTTCACGCTCAGCTTTCAGTTTATCCAAGACCCAGG
CATACTTGAAGGAGCCCTTTCCCATCTCAGC 17 (0.000441%)

ATTTCCTCATATCTCTTCTGGCTGTAGGGTGGCTCAGTGGAATCCATTTTGTTAACACCGACAATTAGTT
GTTTCACACCCAGTGTGTAAGCCAGAAGGG 20 (0.000519%)

ATTTCTAGGATAGTCAGTAGAATTAGAATTGTGAAGATGATAAGTGTAGAGGGAAGGTTAATGGTTGAT
ATTGCTAGGGTGGCGCTTCCAATTAGGTGCA 3 (0.000078%)

ATTTGGCTGTTTGGCTTCAACTCCTACTTTTTCCAGCACGATTCCTTTTGCATGAGAAGCACCTCCAAAA
GGGTTGGCCTTTAGGGCTGTGCCCAAATGA 4 (0.000104%)

ATTTTCTTAATGTAAGTGCTGACTTCCTTAACAATTTCCTCATATCTCTTCTGGCTGTAGGGTGGCTCAGT
GGAATCCATTTTGTTAACACCGACAATTA 20 (0.000519%)

ATTTTGGCGTAGGTTTGGTCTAGGGTGTAGCCTGAGAATAGGGGAAATCAGTGAATGAAGCCTCCTATG
ATGGCAAATACAGCTCCTATTGATAGGACAT 27 (0.000701%)

ATTTTGTTTATGAGCAAGGTGGGTCTCAGAGGTGATCGGCGATCAGAGGGCGATGAAGTTCTAGATCCA
TTGAGACAAGCTCTAGACAGTAGCATGCAGT 45 (0.001168%)

ATTTTTAATCTTAGAGCGAAAGCCTATAATCACTGCGCCTGTTCATAAGGGGATGGCCATGGCTAGGTT
TATAGATAGTTGGGTGGTTGGTGTAAATGAG 5 (0.000130%)

ATTTTTCAATGGTTCTTTTGTCGATGCCACCGCATTTATAGATCAGATGGCCAGTAGTGGTGGACTTGCC
CGAATCTACGTGTCCAATGACGACAATGTT 6 (0.000156%)

ATTTTTCTTTGCATAATCCAGGGAATCATAAATCATGCCAAAGCCAGTTGTCTTGCCACCACCAAAATG
AGTTCTGAATCCAAATACAAAGATGACATCC 292 (0.007578%)

ATTTTTGGTCAAGTTGTTTCCATTAAAAAGTACTGATTTTAAAAACTAATAACTTAAAACTGCCACACGC

AAAAAAGAAAACCAAAGTGGTCCACAAAAC 14 (0.000363%)

CAAAATTCTGTGACAAATTTTTGGTCAAGTTGTTTCCATTAAAAAGTACTGATTTTAAAAACTAATAACT
TAAAACTGCCACACGCAAAAAAGAAAACCA 220 (0.005709%)

CAAACTTGCATGCAATGTGAGCCGTGTGGCAATCCAATACAGGGGCATAGCCGGCGCTTATTTGGCCTG
GATGGTTCAGGATAATCACCTGAGCAGTGAA 6 (0.000156%)

CAAATTTCCACAAGGAGATATCAATGGTGATACCACGTTCACGCTCAGCTTTCAGTTTATCCAAGACCC
AGGCATACTTGAAGGAGCCCTTTCCCATCTC 81 (0.002102%)

CAAATTTTTCAATGGTTCTTTTGTCGATGCCACCGCATTTATAGATCAGATGGCCAGTAGTGGTGGACTT
GCCCGAATCTACGTGTCCAATGACGACAAT 8 (0.000208%)

CAAATTTTTGGTCAAGTTGTTTCCATTAAAAAGTACTGATTTTAAAAACTAATAACTTAAAACTGCCACA
CGCAAAAAAGAAAACCAAAGTGGTCCACAA 29 (0.000753%)

CAACAATGGCAGCATCACCAGACTTCAAGAATTTAGGGCCATCTTCCAGCTTTTTACCAGAACGGCGAT
CAATCTTTTCCTTCAGCTCAGCAAACTTGCA 50 (0.001298%)

CAACATGGGCATTTTGTTTATGAGCAAGGTGGGTCTCAGAGGTGATCGGCGATCAGAGGGCGATGAAG
TTCTAGATCCATTGAGACAAGCTCTAGACAGT 3 (0.000078%)

CAAGAATTTAGGGCCATCTTCCAGCTTTTTACCAGAACGGCGATCAATCTTTTCCTTCAGCTCAGCAAAC
TTGCATGCAATGTGAGCCGTGTGGCAATCC 37 (0.000960%)

CAAGGAGATATCAATGGTGATACCACGTTCACGCTCAGCTTTCAGTTTATCCAAGACCCAGGCATACTT
GAAGGAGCCCTTTCCCATCTCAGCAGCCTCC 13 (0.000337%)

CAAGTTGTTTCCATTAAAAAGTACTGATTTTAAAAACTAATAACTTAAAACTGCCACACGCAAAAAAGA
AAACCAAAGTGGTCCACAAAACATTCTCCTT 26 (0.000675%)

CAATCCAATACAGGGGCATAGCCGGCGCTTATTTGGCCTGGATGGTTCAGGATAATCACCTGAGCAGTG
AAGCCAGCTGCTTCCATTGGTGGGTCATTTT 18 (0.000467%)

CAATCTTTTCCTTCAGCTCAGCAAACTTGCATGCAATGTGAGCCGTGTGGCAATCCAATACAGGGGCAT
AGCCGGCGCTTATTTGGCCTGGATGGTTCAG 104 (0.002699%)

CAATGGTTCTTTTGTCGATGCCACCGCATTTATAGATCAGATGGCCAGTAGTGGTGGACTTGCCCGAATC
TACGTGTCCAATGACGACAATGTTGATATG 3 (0.000078%)

CAATGTGAGCCGTGTGGCAATCCAATACAGGGGCATAGCCGGCGCTTATTTGGCCTGGATGGTTCAGGA
TAATCACCTGAGCAGTGAAGCCAGCTGCTTC 4 (0.000104%)

CAATTTCCTCATATCTCTTCTGGCTGTAGGGTGGCTCAGTGGAATCCATTTTGTTAACACCGACAATTAG
TTGTTTCACACCCAGTGTGTAAGCCAGAAG 18 (0.000467%)

CAATTTTCTTAATGTAAGTGCTGACTTCCTTAACAATTTCCTCATATCTCTTCTGGCTGTAGGGTGGCTCA
GTGGAATCCATTTTGTTAACACCGACAAT 38 (0.000986%)

CACAAGGAGATATCAATGGTGATACCACGTTCACGCTCAGCTTTCAGTTTATCCAAGACCCAGGCATAC
TTGAAGGAGCCCTTTCCCATCTCAGCAGCCT 6 (0.000156%)

CACACTTCCTAATGGCAGAATTTGGCTGTTTGGCTTCAACTCCTACTTTTTCCAGCACGATTCCTTTTGCA
TGAGAAGCACCTCCAAAAGGGTTGGCCTT 15 (0.000389%)

CACAGGTCCAGGGCAGAGGACCAACATGGGCATTTTGTTTATGAGCAAGGTGGGTCTCAGAGGTGATC
GGCGATCAGAGGGCGATGAAGTTCTAGATCCA 72 (0.001868%)

CACAGTCTTGGTTCCCCGGAGACGTCCAGTCCGGCGGGCAGCAATGAGACCCACTTTGCGGCCAGCAGG
GGCATCTCTGCGGATGGTGGAGGGCTTGCCG 5 (0.000130%)

CACCAGACTTCAAGAATTTAGGGCCATCTTCCAGCTTTTACCAGAACGGCGATCAATCTTTTCCTTCAG
CTCAGCAAACTTGCATGCAATGTGAGCCGT 24 (0.000623%)

CACCTACGGTGAAAAGAAAGATGAATCCTAGGGCTCAGAGCACTGCAGCAGATCATTTCATATTGCTTC
CGTGGAGTGTGGCGAGTCAGCTAAATACTTT 3 (0.000078%)

CACCTGAGCAGTGAAGCCAGCTGCTTCCATTGGTGGGTCATTTTTGCTGTCACCAGCAACGTTGCCACG
ACGAACATCCTTGACAGACACATTCTTGACA 8 (0.000208%)

CACGAAGGCCCCAGAAGTGACGCAGCCCTCTATGGGCCCGAATCTTCTTCAGTCGCTCCAGGTCTTCAC
GGAGCTTGTTGTCCAGACCATTGGCTAGGAC 4 (0.000104%)

CACTTCCTAATGGCAGAATTTGGCTGTTTGGCTTCAACTCCTACTTTTTCCAGCACGATTCCTTTTGCATG
AGAAGCACCTCCAAAAGGGTTGGCCTTTA 8 (0.000208%)

CAGAATGTCACAGGTCCAGGGCAGAGGACCAACATGGGCATTTTGTTTATGAGCAAGGTGGGTCTCAG
AGGTGATCGGCGATCAGAGGGCGATGAAGTTC 656 (0.017024%)

CAGAATTTGGCTGTTTGGCTTCAACTCCTACTTTTTCCAGCACGATTCCTTTTGCATGAGAAGCACCTCC
AAAAGGGTTGGCCTTTAGGGCTGTGCCCAA 43 (0.001116%)

CAGACTTCAAGAATTTAGGGCCATCTTCCAGCTTTTACCAGAACGGCGATCAATCTTTTCCTTCAGCTC
AGCAAACTTGCATGCAATGTGAGCCGTGTG 54 (0.001401%)

CAGACTTGGTGACCTTGCCAGCTCCAGCAGCCTTCTTGTCCACTGCTTTGATGACACCCACCGCAACTGT
CTGTCTCATATCACGAACAGCAAAGCGACC 46 (0.001194%)

CAGAGGACCAACATGGGCATTTTGTTTATGAGCAAGGTGGGTCTCAGAGGTGATCGGCGATCAGAGGG
CGATGAAGTTCTAGATCCATTGAGACAAGCTC 15 (0.000389%)

CAGAGGGCGATGAAGTTCTAGATCCATTGAGACAAGCTCTAGACAGTAGCATGCAGTCCCACAACTTGT
ACCAGCATCCCCAGCGTCTGGCATTCCATGT 8 (0.000208%)

CAGATATTCAGCATCTAACAGTTCAAAAGAAGCCACTACATACTCTTTTCACAAATATGTTTTCACAGA
GCCAATACAGTACTAGCCATTAACCCAGTAC 6 (0.000156%)

CAGCAAACTTGCATGCAATGTGAGCCGTGTGGCAATCCAATACAGGGGCATAGCCGGCGCTTATTTGGC
CTGGATGGTTCAGGATAATCACCTGAGCAGT 31 (0.000804%)

CAGCAGCAACAATCAGGACAGCACAGTCAGCCTGAGATGTCCCTGTAATCATGTTTTTGATAAAGTCTC
TGTGTCCTGGGGCATCAATGATAGTCACATA 1426 (0.037007%)

CAGCAGCCTTCTTGTCCACTGCTTTGATGACACCCACCGCAACTGTCTGTCTCATATCACGAACAGCAAA
GCGACCCAAAGGTGGATAGTCTGAGAAGCT 10 (0.000260%)

CAGCATCACCAGACTTCAAGAATTTAGGGCCATCTTCCAGCTTTTACCAGAACGGCGATCAATCTTTTC
CTTCAGCTCAGCAAACTTGCATGCAATGTG 13 (0.000337%)

CAGCATCTAACAGTTCAAAAGAAGCCACTACATACTCTTTTCACAAATATGTTTTCACAGAGCCAATAC
AGTACTAGCCATTAACCCAGTACACCAAGTG 5 (0.000130%)

CAGCCTTCTTGTCCACTGCTTTGATGACACCCACCGCAACTGTCTGTCTCATATCACGAACAGCAAAGCG
ACCCAAAGGTGGATAGTCTGAGAAGCTCTC 15 (0.000389%)

CAGCGATTTCTAGGATAGTCAGTAGAATTAGAATTGTGAAGATGATAAGTGTAGAGGGAAGGTTAATG
GTTGATATTGCTAGGGTGGCGCTTCCAATTAG 12 (0.000311%)

CAGCTCAGCAAACTTGCATGCAATGTGAGCCGTGTGGCAATCCAATACAGGGGCATAGCCGGCGCTTAT
TTGGCCTGGATGGTTCAGGATAATCACCTGA 14 (0.000363%)

CAGCTCCAGCAGCCTTCTTGTCCACTGCTTTGATGACACCCACCGCAACTGTCTGTCTCATATCACGAAC
AGCAAAGCGACCCAAAGGTGGATAGTCTGA 5 (0.000130%)

CAGCTTTTTACCAGAACGGCGATCAATCTTTTCCTTCAGCTCAGCAAACTTGCATGCAATGTGAGCCGTG
TGGCAATCCAATACAGGGGCATAGCCGGCG 383 (0.009939%)

CAGGAACCATATCAACAATGGCAGCATCACCAGACTTCAAGAATTTAGGGCCATCTTCCAGCTTTTTAC
CAGAACGGCGATCAATCTTTTCCTTCAGCTC 71 (0.001843%)

CAGGATAATCACCTGAGCAGTGAAGCCAGCTGCTTCCATTGGTGGGTCATTTTTGCTGTCACCAGCAAC
GTTGCCACGACGAACATCCTTGACAGACACA 24 (0.000623%)

CAGGCCACCTACGGTGAAAAGAAAGATGAATCCTAGGGCTCAGAGCACTGCAGCAGATCATTTCATAT
TGCTTCCGTGGAGTGTGGCGAGTCAGCTAAAT 16 (0.000415%)

CAGGGAATCATAAATCATGCCAAAGCCAGTTGTCTTGCCACCACCAAAATGAGTTCTGAATCCAAATAC
AAAGATGACATCCGGTGTGGTCTTGTACATT 5 (0.000130%)

CAGGGATGAGGCGTACCAGCACAGAGCCGCAGCGGCCTGTCACCTTGCAAGGGACAGTGTGGGGCTTG
CCGATCTTGTTCCCCCAGTAGCCTCTGCGCAC 15 (0.000389%)

CAGGGCAGAGGACCAACATGGGCATTTTGTTTATGAGCAAGGTGGGTCTCAGAGGTGATCGGCGATCA
GAGGGCGATGAAGTTCTAGATCCATTGAGACA 4 (0.000104%)

CAGGTCCAGGGCAGAGGACCAACATGGGCATTTTGTTTATGAGCAAGGTGGGTCTCAGAGGTGATCGG
CGATCAGAGGGCGATGAAGTTCTAGATCCATT 11 (0.000285%)

CAGTAGAATTAGAATTGTGAAGATGATAAGTGTAGAGGGAAGGTTAATGGTTGATATTGCTAGGGTGG
CGCTTCCAATTAGGTGCATGAGTAGGTGGCCT 8 (0.000208%)

CAGTCTTTTACTACTAAACTTAAATGGCCAATTGAAACAAACAGTTCTGAGACCGTTCTTCCACCACTGA
TTAAGAGTGGGGTGGCAGGTATTAGGGATA 57 (0.001479%)

CAGTGCCCCTGGGTGCAGGGATGAGGCGTACCAGCACAGAGCCGCAGCGGCCTGTCACCTTGCAAGGG
ACAGTGTGGGGCTTGCCGATCTTGTTCCCCCA 4 (0.000104%)

CAGTGCCCTCCTAATTGGGGGGTAGGGGCTAGGCTGGAGTGGTAAAAGGCTCAGAAAAATCCTGCGAA
GAAAAAAACTTCTGAGGTAATAAATAGGATTA 11 (0.000285%)

CATATCAACAATGGCAGCATCACCAGACTTCAAGAATTTAGGGCCATCTTCCAGCTTTTTACCAGAACG
GCGATCAATCTTTTCCTTCAGCTCAGCAAAC 16 (0.000415%)

CATCACCAGACTTCAAGAATTTAGGGCCATCTTCCAGCTTTTTACCAGAACGGCGATCAATCTTTTCCTT
CAGCTCAGCAAACTTGCATGCAATGTGAGC 13 (0.000337%)

CATGCAATGTGAGCCGTGTGGCAATCCAATACAGGGGCATAGCCGGCGCTTATTTGGCCTGGATGGTTC
AGGATAATCACCTGAGCAGTGAAGCCAGCTG 4 (0.000104%)

CATTGTGTTGTGGTAAATATGTAGAGGGAGTATAGGGCTGTGACTAGTATGTTGAGTCCTGTAAGTAGG
AGAGTGATATTTGATCAGGAGAACGTGGTTA 7 (0.000182%)

CATTTAGCCTTCTGAGCTTTCTGGGCAGACTTGGTGACCTTGCCAGCTCCAGCAGCCTTCTTGTCCACTG

CTTTGATGACACCCACCGCAACTGTCTGTC 10 (0.000260%)

CCAACATGGGCATTTGTTTATGAGCAAGGTGGGTCTCAGAGGTGATCGGCGATCAGAGGGCGATGAA
GTTCTAGATCCATTGAGACAAGCTCTAGACAG 14 (0.000363%)

CCAATTTTCTTAATGTAAGTGCTGACTTCCTTAACAATTTCCTCATATCTCTTCTGGCTGTAGGGTGGCTC
AGTGGAATCCATTTTGTTAACACCGACAA 3 (0.000078%)

CCACAAGGAGATATCAATGGTGATACCACGTTCACGCTCAGCTTTCAGTTTATCCAAGACCCAGGCATA
CTTGAAGGAGCCCTTTCCCATCTCAGCAGCC 8 (0.000208%)

CCACCTACGGTGAAAAGAAAGATGAATCCTAGGGCTCAGAGCACTGCAGCAGATCATTTCATATTGCTT
CCGTGGAGTGTGGCGAGTCAGCTAAATACTT 4 (0.000104%)

CCACGGTGGGCGGGCGGGCCTCTCTACTCGAAGGTGACCACGTTTAGATTCTGAGACGGGAAGTGGAG
GGTGAATAGGTCACGGCGGCCTTTTTTTTTT 7 (0.000182%)

CCACTCCTGATGCTGAACCAATGCACCATCTGTAAAGTTGCAGACAGTCTGAGTTTTTCTGCCATCAGCT
GTGGTTTCTTCAAACTTCTCTCCCAGGGTA 5 (0.000130%)

CCAGACTTCAAGAATTTAGGGCCATCTTCCAGCTTTTTACCAGAACGGCGATCAATCTTTTCCTTCAGCT
CAGCAAACTTGCATGCAATGTGAGCCGTGT 72 (0.001868%)

CCAGCAGCCTTCTTGTCCACTGCTTTGATGACACCCACCGCAACTGTCTGTCTCATATCACGAACAGCAA
AGCGACCCAAAGGTGGATAGTCTGAGAAGC 5 (0.000130%)

CCAGCTCCAGCAGCCTTCTTGTCCACTGCTTTGATGACACCCACCGCAACTGTCTGTCTCATATCACGAA
CAGCAAAGCGACCCAAAGGTGGATAGTCTG 5 (0.000130%)

CCAGGAACCATATCAACAATGGCAGCATCACCAGACTTCAAGAATTTAGGGCCATCTTCCAGCTTTTTA
CCAGAACGGCGATCAATCTTTTCCTTCAGCT 55 (0.001427%)

CCAGGGAATCATAAATCATGCCAAAGCCAGTTGTCTTGCCACCACCAAAATGAGTTCTGAATCCAAATA
CAAAGATGACATCCGGTGTGGTCTTGTACAT 9 (0.000234%)

CCAGGGCAGAGGACCAACATGGGCATTTGTTTATGAGCAAGGTGGGTCTCAGAGGTGATCGGCGATC
AGAGGGCGATGAAGTTCTAGATCCATTGAGAC 9 (0.000234%)

CCAGTCAGGCCACCTACGGTGAAAAGAAAGATGAATCCTAGGGCTCAGAGCACTGCAGCAGATCATTT
CATATTGCTTCCGTGGAGTGTGGCGAGTCAGC 8 (0.000208%)

CCAGTGCCCCTGGGTGCAGGGATGAGGCGTACCAGCACAGAGCCGCAGCGGCCTGTCACCTTGCAAGG
GACAGTGTGGGGCTTGCCGATCTTGTTCCCCC 3 (0.000078%)

CCAGTGCCCTCCTAATTGGGGGGTAGGGGCTAGGCTGGAGTGGTAAAAGGCTCAGAAAAATCCTGCGA
AGAAAAAAACTTCTGAGGTAATAAATAGGATT 6 (0.000156%)

CCATATCAACAATGGCAGCATCACCAGACTTCAAGAATTTAGGGCCATCTTCCAGCTTTTTACCAGAAC
GGCGATCAATCTTTTCCTTCAGCTCAGCAAA 8 (0.000208%)

CCATGTGACCTTCTCTGGCATTCGGGCATTGGCTGTACCCTTCCGCTTACCTATGCCCATGTGCCTGCCC
TTCCGGCGGGCCAAGGTGTTTTTCCGGCAT 7 (0.000182%)

CCATTGTGTTGTGGTAAATATGTAGAGGGAGTATAGGGCTGTGACTAGTATGTTGAGTCCTGTAAGTAG
GAGAGTGATATTTGATCAGGAGAACGTGGTT 6 (0.000156%)

CCCACGGTGGGCGGGCGGGCCTCTCTACTCGAAGGTGACCACGTTTAGATTCTGAGACGGGAAGTGGA
GGGTGAATAGGTCACGGCGGCCTTTTTTTTTT 3 (0.000078%)

CCCACTCCTGATGCTGAACCAATGCACCATCTGTAAAGTTGCAGACAGTCTGAGTTTTTCTGCCATCAGC
TGTGGTTTCTTCAAACTTCTCTCCCAGGGT 3 (0.000078%)

CCCATTGTGTTGTGGTAAATATGTAGAGGGAGTATAGGGCTGTGACTAGTATGTTGAGTCCTGTAAGTA
GGAGAGTGATATTTGATCAGGAGAACGTGGT 3 (0.000078%)

CCCCATTGTGTTGTGGTAAATATGTAGAGGGAGTATAGGGCTGTGACTAGTATGTTGAGTCCTGTAAGT
AGGAGAGTGATATTTGATCAGGAGAACGTGG 29 (0.000753%)

CCCCTGGGTGCAGGGATGAGGCGTACCAGCACAGAGCCGCAGCGGCCTGTCACCTTGCAAGGGACAGT
GTGGGGCTTGCCGATCTTGTTCCCCCAGTAGC 6 (0.000156%)

CCCGGAGACGTCCAGTCCGGCGGGCAGCAATGAGACCCACTTTGCGGCCAGCAGGGGCATCTCTGCGG
ATGGTGGAGGGCTTGCCGATGTGCTGGTGGTT 9 (0.000234%)

CCCTGCTAAGGGAGGGTAGACTGTTCAACCTGTTCCTGCTCCGGCCTCCACTATAGCAGATGCGAGCAG
GAGTAGGAGAGAGGGAGGTAAGAGTCAGAAG 6 (0.000156%)

CCCTGGGTGCAGGGATGAGGCGTACCAGCACAGAGCCGCAGCGGCCTGTCACCTTGCAAGGGACAGTG
TGGGGCTTGCCGATCTTGTTCCCCCAGTAGCC 3 (0.000078%)

CCGCATTTATAGATCAGATGGCCAGTAGTGGTGGACTTGCCCGAATCTACGTGTCCAATGACGACAATG
TTGATATGAGTCTTTTCCTTTCCCATTTTGG 5 (0.000130%)

CCGGAGACGTCCAGTCCGGCGGGCAGCAATGAGACCCACTTTGCGGCCAGCAGGGGCATCTCTGCGGA
TGGTGGAGGGCTTGCCGATGTGCTGGTGGTTG 8 (0.000208%)

CCGTGTGGCAATCCAATACAGGGGCATAGCCGGCGCTTATTTGGCCTGGATGGTTCAGGATAATCACCT
GAGCAGTGAAGCCAGCTGCTTCCATTGGTGG 3 (0.000078%)

CCTAATGGCAGAATTTGGCTGTTTGGCTTCAACTCCTACTTTTTCCAGCACGATTCCTTTTGCATGAGAA
GCACCTCCAAAAGGGTTGGCCTTTAGGGCT 6 (0.000156%)

CCTAATGTGGGGACAGCTCATGAGTGCAAGACGTCTTGTGATGTAATTATTATACGAATGGGGGCTTCA
ATCGGGAGTACTACTCGATTGTCAACGTCAA 3 (0.000078%)

CCTAATTGGGGGGTAGGGGCTAGGCTGGAGTGGTAAAAGGCTCAGAAAAATCCTGCGAAGAAAAAAAC
TTCTGAGGTAATAAATAGGATTATCCCGTATC 8 (0.000208%)

CCTACGGTGAAAAGAAAGATGAATCCTAGGGCTCAGAGCACTGCAGCAGATCATTTCATATTGCTTCCG
TGGAGTGTGGCGAGTCAGCTAAATACTTTGA 4 (0.000104%)

CCTATAATCACTGCGCCTGTTCATAAGGGGATGGCCATGGCTAGGTTTATAGATAGTTGGGTGGTTGGT
GTAAATGAGTGAGGCAGGAGTCCGAGGAGGT 3 (0.000078%)

CCTATTTCAAAGATTTTTAGGGGAATTAATTCTAGGACGATGGGCATGAAACTGTGGTTTGCTCCACAG
ATTTCAGAGCATTGACCGTAGTATACCCCCG 566 (0.014688%)

CCTCATCCATGTGACCTTCTCTGGCATTCGGGCATTGGCTGTACCCTTCCGCTTACCTATGCCCATGTGC
CTGCCCTTCCGGCGGGCCAAGGTGTTTTTC 7 (0.000182%)

CCTCCTAATTGGGGGGTAGGGGCTAGGCTGGAGTGGTAAAAGGCTCAGAAAAATCCTGCGAAGAAAAA
AACTTCTGAGGTAATAAATAGGATTATCCCGT 4 (0.000104%)

CCTCGGACACGAAGGCCCCAGAAGTGACGCAGCCCTCTATGGGCCCGAATCTTCTTCAGTCGCTCCAGG
TCTTCACGGAGCTTGTTGTCCAGACCATTGG 8 (0.000208%)

CCTGAGAATAGGGGAAATCAGTGAATGAAGCCTCCTATGATGGCAAATACAGCTCCTATTGATAGGAC
ATAGTGGAAGTGAGCTACAACGTAGTACGTGT 14 (0.000363%)

CCTGAGCAGTGAAGCCAGCTGCTTCCATTGGTGGGTCATTTTTGCTGTCACCAGCAACGTTGCCACGAC
GAACATCCTTGACAGACACATTCTTGACATT 5 (0.000130%)

CCTGCTAAGGGAGGGTAGACTGTTCAACCTGTTCCTGCTCCGGCCTCCACTATAGCAGATGCGAGCAGG
AGTAGGAGAGAGGGAGGTAAGAGTCAGAAGC 3 (0.000078%)

CCTGGGTGCAGGGATGAGGCGTACCAGCACAGAGCCGCAGCGGCCTGTCACCTTGCAAGGGACAGTGT
GGGGCTTGCCGATCTTGTTCCCCCAGTAGCCT 13 (0.000337%)

CCTTAACAATTTCCTCATATCTCTTCTGGCTGTAGGGTGGCTCAGTGGAATCCATTTTGTTAACACCGAC
AATTAGTTGTTTCACACCCAGTGTGTAAGC 13 (0.000337%)

CCTTCAGCTCAGCAAACTTGCATGCAATGTGAGCCGTGTGGCAATCCAATACAGGGGCATAGCCGGCGC
TTATTTGGCCTGGATGGTTCAGGATAATCAC 14 (0.000363%)

CCTTCTCTGGCATTCGGGCATTGGCTGTACCCTTCCGCTTACCTATGCCCATGTGCCTGCCCTTCCGGCG
GGCCAAGGTGTTTTTCCGGCATCGAGCCCG 7 (0.000182%)

CCTTCTGAGCTTTCTGGGCAGACTTGGTGACCTTGCCAGCTCCAGCAGCCTTCTTGTCCACTGCTTTGAT
GACACCCACCGCAACTGTCTGTCTCATATC 6 (0.000156%)

CCTTCTTGTCCACTGCTTTGATGACACCCACCGCAACTGTCTGTCTCATATCACGAACAGCAAAGCGACC
CAAAGGTGGATAGTCTGAGAAGCTCTCAAC 13 (0.000337%)

CCTTGCCAGCTCCAGCAGCCTTCTTGTCCACTGCTTTGATGACACCCACCGCAACTGTCTGTCTCATATC
ACGAACAGCAAAGCGACCCAAAGGTGGATA 14 (0.000363%)

CCTTGGTATGTGCTTTCTCGTGTTACATCGCGCCATCATTGGTATATGGTTAGTGTGTTGGTTAGTAGGC
CTAGTATGAGGAGCGTTATGGAGTGGAAGT 56 (0.001453%)

CGACAGCGATTTCTAGGATAGTCAGTAGAATTAGAATTGTGAAGATGATAAGTGTAGAGGGAAGGTTA
ATGGTTGATATTGCTAGGGTGGCGCTTCCAAT 5 (0.000130%)

CGATCAATCTTTTCCTTCAGCTCAGCAAACTTGCATGCAATGTGAGCCGTGTGGCAATCCAATACAGGG
GCATAGCCGGCGCTTATTTGGCCTGGATGGT 10 (0.000260%)

CGATCAGAGGGCGATGAAGTTCTAGATCCATTGAGACAAGCTCTAGACAGTAGCATGCAGTCCCACAA
CTTGTACCAGCATCCCCAGCGTCTGGCATTCC 5 (0.000130%)

CGATGCCACCGCATTTATAGATCAGATGGCCAGTAGTGGTGGACTTGCCCGAATCTACGTGTCCAATGA
CGACAATGTTGATATGAGTCTTTTCCTTTCC 14 (0.000363%)

CGATGCCAGTGCCCCTGGGTGCAGGGATGAGGCGTACCAGCACAGAGCCGCAGCGGCCTGTCACCTTG
CAAGGGACAGTGTGGGGCTTGCCGATCTTGTT 13 (0.000337%)

CGATGGAGGTAGGATTGGTGCTGTGGGTGAAAGAGTATGATGGGGTGGTGGTTGTGGTAAACTTTAATA
GTGTAGGAAGCTGAATAATTTATGAAGGAGA 3 (0.000078%)

CGATTTCTAGGATAGTCAGTAGAATTAGAATTGTGAAGATGATAAGTGTAGAGGGAAGGTTAATGGTTG
ATATTGCTAGGGTGGCGCTTCCAATTAGGTG 8 (0.000208%)

CGCATTTATAGATCAGATGGCCAGTAGTGGTGGACTTGCCCGAATCTACGTGTCCAATGACGACAATGT
TGATATGAGTCTTTTCCTTTCCCATTTTGGC 22 (0.000571%)

CGGACACGAAGGCCCCAGAAGTGACGCAGCCCTCTATGGGCCCGAATCTTCTTCAGTCGCTCCAGGTCT

TCACGGAGCTTGTTGTCCAGACCATTGGCTA 45 (0.001168%)

CGGAGACGATGCCAGTGCCCCTGGGTGCAGGGATGAGGCGTACCAGCACAGAGCCGCAGCGGCCTGTC
ACCTTGCAAGGGACAGTGTGGGGCTTGCCGAT 25 (0.000649%)

CGGAGACGTCCAGTCCGGCGGGCAGCAATGAGACCCACTTTGCGGCCAGCAGGGGCATCTCTGCGGAT
GGTGGAGGGCTTGCCGATGTGCTGGTGGTTGC 30 (0.000779%)

CGGCGATCAGAGGGCGATGAAGTTCTAGATCCATTGAGACAAGCTCTAGACAGTAGCATGCAGTCCCA
CAACTTGTACCAGCATCCCCAGCGTCTGGCAT 334 (0.008668%)

CGGGTGGCACTGCCCACGGTGGGCGGGCGGGCCTCTCTACTCGAAGGTGACCACGTTTAGATTCTGAGA
CGGGAAGTGGAGGGTGAATAGGTCACGGCGG 6 (0.000156%)

CGGTTGTTGATGAGATATTTGGAGGTGGGGATCAATAGAGGGGGAAATAGAATGATCAGTACTGCGGC
GGGTAGGCCTAGGATTGTGGGGGCAATGAATG 22 (0.000571%)

CGTAGGTTTGGTCTAGGGTGTAGCCTGAGAATAGGGGAAATCAGTGAATGAAGCCTCCTATGATGGCA
AATACAGCTCCTATTGATAGGACATAGTGGAA 6 (0.000156%)

CGTGTGGCAATCCAATACAGGGGCATAGCCGGCGCTTATTTGGCCTGGATGGTTCAGGATAATCACCTG
AGCAGTGAAGCCAGCTGCTTCCATTGGTGGG 13 (0.000337%)

CGTGTTACATCGCGCCATCATTGGTATATGGTTAGTGTGTTGGTTAGTAGGCCTAGTATGAGGAGCGTTA
TGGAGTGGAAGTGAAATCACATGGCTAGGC 6 (0.000156%)

CTAAGGGAGGGTAGACTGTTCAACCTGTTCCTGCTCCGGCCTCCACTATAGCAGATGCGAGCAGGAGTA
GGAGAGAGGGAGGTAAGAGTCAGAAGCTTAT 4 (0.000104%)

CTAATACAATGCCAGTCAGGCCACCTACGGTGAAAAGAAAGATGAATCCTAGGGCTCAGAGCACTGCA
GCAGATCATTTCATATTGCTTCCGTGGAGTGT 7 (0.000182%)

CTAATGGCAGAATTTGGCTGTTTGGCTTCAACTCCTACTTTTTCCAGCACGATTCCTTTTGCATGAGAAG
CACCTCCAAAAGGGTTGGCCTTTAGGGCTG 8 (0.000208%)

CTAATTGGGGGGTAGGGGCTAGGCTGGAGTGGTAAAAGGCTCAGAAAAATCCTGCGAAGAAAAAAACT
TCTGAGGTAATAAATAGGATTATCCCGTATCG 7 (0.000182%)

CTACGGTGAAAAGAAAGATGAATCCTAGGGCTCAGAGCACTGCAGCAGATCATTTCATATTGCTTCCGT
GGAGTGTGGCGAGTCAGCTAAATACTTTGAC 10 (0.000260%)

CTACTAAACTTAAATGGCCAATTGAAACAAACAGTTCTGAGACCGTTCTTCCACCACTGATTAAGAGTG
GGGTGGCAGGTATTAGGGATAATATTCATTT 4 (0.000104%)

CTAGGATAGTCAGTAGAATTAGAATTGTGAAGATGATAAGTGTAGAGGGAAGGTTAATGGTTGATATT
GCTAGGGTGGCGCTTCCAATTAGGTGCATGAG 15 (0.000389%)

CTAGGGTGTAGCCTGAGAATAGGGGAAATCAGTGAATGAAGCCTCCTATGATGGCAAATACAGCTCCT
ATTGATAGGACATAGTGGAAGTGAGCTACAAC 47 (0.001220%)

CTATAATCACTGCGCCTGTTCATAAGGGGATGGCCATGGCTAGGTTTATAGATAGTTGGGTGGTTGGTG
TAAATGAGTGAGGCAGGAGTCCGAGGAGGTT 5 (0.000130%)

CTCAAAATTCTGTGACAAATTTTTGGTCAAGTTGTTTCCATTAAAAAGTACTGATTTTAAAAACTAATAA
CTTAAAACTGCCACACGCAAAAAAGAAAAC 27 (0.000701%)

CTCAAATTTCCACAAGGAGATATCAATGGTGATACCACGTTCACGCTCAGCTTTCAGTTTATCCAAGAC
CCAGGCATACTTGAAGGAGCCCTTTCCCATC 143 (0.003711%)

CTCAAATTTTTCAATGGTTCTTTTGTCGATGCCACCGCATTTATAGATCAGATGGCCAGTAGTGGTGGAC
TTGCCCGAATCTACGTGTCCAATGACGACA 21 (0.000545%)

CTCAGCAAACTTGCATGCAATGTGAGCCGTGTGGCAATCCAATACAGGGGCATAGCCGGCGCTTATTTG
GCCTGGATGGTTCAGGATAATCACCTGAGCA 11 (0.000285%)

CTCATCCATGTGACCTTCTCTGGCATTCGGGCATTGGCTGTACCCTTCCGCTTACCTATGCCCATGTGCCT
GCCCTTCCGGCGGGCCAAGGTGTTTTTCC 8 (0.000208%)

CTCCAGCAGCCTTCTTGTCCACTGCTTTGATGACACCCACCGCAACTGTCTGTCTCATATCACGAACAGC
AAAGCGACCCAAAGGTGGATAGTCTGAGAA 10 (0.000260%)

CTCGGACACGAAGGCCCCAGAAGTGACGCAGCCCTCTATGGGCCCGAATCTTCTTCAGTCGCTCCAGGT
CTTCACGGAGCTTGTTGTCCAGACCATTGGC 20 (0.000519%)

CTCGTGTTACATCGCGCCATCATTGGTATATGGTTAGTGTGTTGGTTAGTAGGCCTAGTATGAGGAGCGT
TATGGAGTGGAAGTGAAATCACATGGCTAG 43 (0.001116%)

CTCTGGCATTCGGGCATTGGCTGTACCCTTCCGCTTACCTATGCCCATGTGCCTGCCCTTCCGGCGGGCC
AAGGTGTTTTTCCGGCATCGAGCCCGGGAA 6 (0.000156%)

CTCTTATCAAGTCAGCACACACCTTTTCCAAGGATTTTACGTTGCGGCTTGTTAGGGTGATTCGAATTCG
GTGAATTGCCACCTCCGGCTCCACGGGTGT 432 (0.011211%)

CTGACTTCCTTAACAATTTCCTCATATCTCTTCTGGCTGTAGGGTGGCTCAGTGGAATCCATTTTGTTAAC
ACCGACAATTAGTTGTTTCACACCCAGTG 43 (0.001116%)

CTGAGCAGTGAAGCCAGCTGCTTCCATTGGTGGGTCATTTTTGCTGTCACCAGCAACGTTGCCACGACG
AACATCCTTGACAGACACATTCTTGACATTG 4 (0.000104%)

CTGAGCTTTCTGGGCAGACTTGGTGACCTTGCCAGCTCCAGCAGCCTTCTTGTCCACTGCTTTGATGACA
CCCACCGCAACTGTCTGTCTCATATCACGA 31 (0.000804%)

CTGCACAGTCTTGGTTCCCCGGAGACGTCCAGTCCGGCGGGCAGCAATGAGACCCACTTTGCGGCCAGC
AGGGGCATCTCTGCGGATGGTGGAGGGCTTG 346 (0.008979%)

CTGCGGGTGGCACTGCCCACGGTGGGCGGGCGGGCCTCTCTACTCGAAGGTGACCACGTTTAGATTCTG
AGACGGGAAGTGGAGGGTGAATAGGTCACGG 16 (0.000415%)

CTGGCATTCGGGCATTGGCTGTACCCTTCCGCTTACCTATGCCCATGTGCCTGCCCTTCCGGCGGGCCAA
GGTGTTTTTCCGGCATCGAGCCCGGGAATG 17 (0.000441%)

CTGGGCAGACTTGGTGACCTTGCCAGCTCCAGCAGCCTTCTTGTCCACTGCTTTGATGACACCCACCGCA
ACTGTCTGTCTCATATCACGAACAGCAAAG   21 (0.000545%)

CTGGGTGCAGGGATGAGGCGTACCAGCACAGAGCCGCAGCGGCCTGTCACCTTGCAAGGGACAGTGTG
GGGCTTGCCGATCTTGTTCCCCCAGTAGCCTC 8 (0.000208%)

CTGGTCTCAAATTTCCACAAGGAGATATCAATGGTGATACCACGTTCACGCTCAGCTTTCAGTTTATCCA
AGACCCAGGCATACTTGAAGGAGCCCTTTC   55 (0.001427%)

CTGTGACAAATTTTTGGTCAAGTTGTTTCCATTAAAAAGTACTGATTTTAAAAACTAATAACTTAAAACT
GCCACACGCAAAAAAGAAAACCAAAGTGGT   27 (0.000701%)

CTGTTGGGGGCCAGTGCCCTCCTAATTGGGGGGTAGGGGCTAGGCTGGAGTGGTAAAAGGCTCAGAAA
AATCCTGCGAAGAAAAAAACTTCTGAGGTAAT   714 (0.018529%)

CTGTTTTTAAGCCTAATGTGGGGACAGCTCATGAGTGCAAGACGTCTTGTGATGTAATTATTATACGAAT
GGGGGCTTCAATCGGGAGTACTACTCGATT 812 (0.021072%)

CTTAACAATTTCCTCATATCTCTTCTGGCTGTAGGGTGGCTCAGTGGAATCCATTTTGTTAACACCGACA
ATTAGTTGTTTCACACCCAGTGTGTAAGCC 29 (0.000753%)

CTTAATGTAAGTGCTGACTTCCTTAACAATTTCCTCATATCTCTTCTGGCTGTAGGGTGGCTCAGTGGAA
TCCATTTTGTTAACACCGACAATTAGTTGT23 (0.000597%)

CTTACACACTTCCTAATGGCAGAATTTGGCTGTTTGGCTTCAACTCCTACTTTTTCCAGCACGATTCCTTT
TGCATGAGAAGCACCTCCAAAAGGGTTGG 577 (0.014974%)

CTTAGGCACAGGTGCGGAGACGATGCCAGTGCCCCTGGGTGCAGGGATGAGGCGTACCAGCACAGAGC
CGCAGCGGCCTGTCACCTTGCAAGGGACAGTG 20 (0.000519%)

CTTATCAAGTCAGCACACACCTTTTCCAAGGATTTTACGTTGCGGCTTGTTAGGGTGATTCGAATTCGGT
GAATTGCCACCTCCGGCTCCACGGGTGTTT 17 (0.000441%)

CTTCAAGAATTTAGGGCCATCTTCCAGCTTTTTACCAGAACGGCGATCAATCTTTTCCTTCAGCTCAGCA
AACTTGCATGCAATGTGAGCCGTGTGGCAA 7 (0.000182%)

CTTCAGCTCAGCAAACTTGCATGCAATGTGAGCCGTGTGGCAATCCAATACAGGGGCATAGCCGGCGCT
TATTTGGCCTGGATGGTTCAGGATAATCACC 35 (0.000908%)

CTTCCTAATGGCAGAATTTGGCTGTTTGGCTTCAACTCCTACTTTTTCCAGCACGATTCCTTTTGCATGAG
AAGCACCTCCAAAAGGGTTGGCCTTTAGG6 (0.000156%)

CTTCCTTAACAATTTCCTCATATCTCTTCTGGCTGTAGGGTGGCTCAGTGGAATCCATTTTGTTAACACC
GACAATTAGTTGTTTCACACCCAGTGTGTA 50 (0.001298%)

CTTCTCAAATTTTTCAATGGTTCTTTTGTCGATGCCACCGCATTTATAGATCAGATGGCCAGTAGTGGTG
GACTTGCCCGAATCTACGTGTCCAATGACG 860 (0.022318%)

CTTCTCTGGCATTCGGGCATTGGCTGTACCCTTCCGCTTACCTATGCCCATGTGCCTGCCCTTCCGGCGG
GCCAAGGTGTTTTTCCGGCATCGAGCCCGG 10 (0.000260%)

CTTCTGAGCTTTCTGGGCAGACTTGGTGACCTTGCCAGCTCCAGCAGCCTTCTTGTCCACTGCTTTGATG
ACACCCACCGCAACTGTCTGTCTCATATCA 15 (0.000389%)

CTTCTTAGGCACAGGTGCGGAGACGATGCCAGTGCCCCTGGGTGCAGGGATGAGGCGTACCAGCACAG
AGCCGCAGCGGCCTGTCACCTTGCAAGGGACA 6 (0.000156%)

CTTCTTGTCCACTGCTTTGATGACACCCACCGCAACTGTCTGTCTCATATCACGAACAGCAAAGCGACCC
AAAGGTGGATAGTCTGAGAAGCTCTCAACA 10 (0.000260%)

CTTGCATGCAATGTGAGCCGTGTGGCAATCCAATACAGGGGCATAGCCGGCGCTTATTTGGCCTGGATG
GTTCAGGATAATCACCTGAGCAGTGAAGCCA 16 (0.000415%)

CTTGCCAGCTCCAGCAGCCTTCTTGTCCACTGCTTTGATGACACCCACCGCAACTGTCTGTCTCATATCA
CGAACAGCAAAGCGACCCAAAGGTGGATAG 16 (0.000415%)

CTTGCTGGTCTCAAATTTCCACAAGGAGATATCAATGGTGATACCACGTTCACGCTCAGCTTTCAGTTTA
TCCAAGACCCAGGCATACTTGAAGGAGCCC 26 (0.000675%)

CTTGGTATGTGCTTTCTCGTGTTACATCGCGCCATCATTGGTATATGGTTAGTGTGTTGGTTAGTAGGCC
TAGTATGAGGAGCGTTATGGAGTGGAAGTG 36 (0.000934%)

CTTGGTGACCTTGCCAGCTCCAGCAGCCTTCTTGTCCACTGCTTTGATGACACCCACCGCAACTGTCTGT

CTCATATCACGAACAGCAAAGCGACCCAAA 24 (0.000623%)

CTTGGTTCCCCGGAGACGTCCAGTCCGGCGGGCAGCAATGAGACCCACTTTGCGGCCAGCAGGGGCATC
TCTGCGGATGGTGGAGGGCTTGCCGATGTGC 38 (0.000986%)

CTTGTCCACTGCTTTGATGACACCCACCGCAACTGTCTGTCTCATATCACGAACAGCAAAGCGACCCAA
AGGTGGATAGTCTGAGAAGCTCTCAACACAC 32 (0.000830%)

CTTTCTCGTGTTACATCGCGCCATCATTGGTATATGGTTAGTGTGTTGGTTAGTAGGCCTAGTATGAGGA
GCGTTATGGAGTGGAAGTGAAATCACATGG 16 (0.000415%)

CTTTCTGGGCAGACTTGGTGACCTTGCCAGCTCCAGCAGCCTTCTTGTCCACTGCTTTGATGACACCCAC
CGCAACTGTCTGTCTCATATCACGAACAGC 8 (0.000208%)

CTTTTACTACTAAACTTAAATGGCCAATTGAAACAAACAGTTCTGAGACCGTTCTTCCACCACTGATTAA
GAGTGGGGTGGCAGGTATTAGGGATAATAT 30 (0.000779%)

CTTTTCCTTCAGCTCAGCAAACTTGCATGCAATGTGAGCCGTGTGGCAATCCAATACAGGGGCATAGCC
GGCGCTTATTTGGCCTGGATGGTTCAGGATA 117 (0.003036%)

CTTTTGTCGATGCCACCGCATTTATAGATCAGATGGCCAGTAGTGGTGGACTTGCCCGAATCTACGTGTC
CAATGACGACAATGTTGATATGAGTCTTTT 76 (0.001972%)

CTTTTTACCAGAACGGCGATCAATCTTTTCCTTCAGCTCAGCAAACTTGCATGCAATGTGAGCCGTGTGG
CAATCCAATACAGGGGCATAGCCGGCGCTT 7 (0.000182%)

CTTTTTTAATGGGTCTCAAAATTCTGTGACAAATTTTTGGTCAAGTTGTTTCCATTAAAAAGTACTGATTT
TAAAAACTAATAACTTAAAACTGCCACAC 461 (0.011964%)

GAAAAGTGAACAGATATTCAGCATCTAACAGTTCAAAAGAAGCCACTACATACTCTTTTCACAAATATG
TTTTCACAGAGCCAATACAGTACTAGCCATT 4 (0.000104%)

GAACCATATCAACAATGGCAGCATCACCAGACTTCAAGAATTTAGGGCCATCTTCCAGCTTTTTACCAG
AACGGCGATCAATCTTTTCCTTCAGCTCAGC 17 (0.000441%)

GAATGTCACAGGTCCAGGGCAGAGGACCAACATGGGCATTTTGTTTATGAGCAAGGTGGGTCTCAGAG
GTGATCGGCGATCAGAGGGCGATGAAGTTCTA 13 (0.000337%)

GAATTTAGGGCCATCTTCCAGCTTTTTACCAGAACGGCGATCAATCTTTTCCTTCAGCTCAGCAAACTTG
CATGCAATGTGAGCCGTGTGGCAATCCAAT 3 (0.000078%)

GAATTTGGCTGTTTGGCTTCAACTCCTACTTTTTCCAGCACGATTCCTTTTGCATGAGAAGCACCTCCAA
AAGGGTTGGCCTTTAGGGCTGTGCCCAAAT 5 (0.000130%)

GACAAATTTTTGGTCAAGTTGTTTCCATTAAAAAGTACTGATTTTAAAAACTAATAACTTAAAACTGCCA
CACGCAAAAAAGAAAACCAAAGTGGTCCAC 13 (0.000337%)

GACACGAAGGCCCCAGAAGTGACGCAGCCCTCTATGGGCCCGAATCTTCTTCAGTCGCTCCAGGTCTTC
ACGGAGCTTGTTGTCCAGACCATTGGCTAGG 3 (0.000078%)

GACAGCGATTTCTAGGATAGTCAGTAGAATTAGAATTGTGAAGATGATAAGTGTAGAGGGAAGGTTAA
TGGTTGATATTGCTAGGGTGGCGCTTCCAATT 6 (0.000156%)

GACCAACATGGGCATTTTGTTTATGAGCAAGGTGGGTCTCAGAGGTGATCGGCGATCAGAGGGCGATG
AAGTTCTAGATCCATTGAGACAAGCTCTAGAC 6 (0.000156%)

GACCTTCTCTGGCATTCGGGCATTGGCTGTACCCTTCCGCTTACCTATGCCCATGTGCCTGCCCTTCCGG
CGGGCCAAGGTGTTTTCCGGCATCGAGCC 4 (0.000104%)

GACCTTGCCAGCTCCAGCAGCCTTCTTGTCCACTGCTTTGATGACACCCACCGCAACTGTCTGTCTCATATCACGAACAGCAAAGCGACCCAAAGGTGGA 8 (0.000208%)

GACGATGCCAGTGCCCCTGGGTGCAGGGATGAGGCGTACCAGCACAGAGCCGCAGCGGCCTGTCACCTTGCAAGGGACAGTGTGGGGCTTGCCGATCTTG 18 (0.000467%)

GACGTCCAGTCCGGCGGGCAGCAATGAGACCCACTTTGCGGCCAGCAGGGGCATCTCTGCGGATGGTGGAGGGCTTGCCGATGTGCTGGTGGTTGCCACC 4 (0.000104%)

GACTTCAAGAATTTAGGGCCATCTTCCAGCTTTTTACCAGAACGGCGATCAATCTTTTCCTTCAGCTCAGCAAACTTGCATGCAATGTGAGCCGTGTGGC 15 (0.000389%)

GACTTCCTTAACAATTTCCTCATATCTCTTCTGGCTGTAGGGTGGCTCAGTGGAATCCATTTTGTTAACACCGACAATTAGTTGTTTCACACCCAGTGTG 10 (0.000260%)

GACTTGGTGACCTTGCCAGCTCCAGCAGCCTTCTTGTCCACTGCTTTGATGACACCCACCGCAACTGTCTGTCTCATATCACGAACAGCAAAGCGACCCA 13 (0.000337%)

GAGACGATGCCAGTGCCCCTGGGTGCAGGGATGAGGCGTACCAGCACAGAGCCGCAGCGGCCTGTCACCTTGCAAGGGACAGTGTGGGGCTTGCCGATCT 3 (0.000078%)

GAGACGTCCAGTCCGGCGGGCAGCAATGAGACCCACTTTGCGGCCAGCAGGGGCATCTCTGCGGATGGTGGAGGGCTTGCCGATGTGCTGGTGGTTGCCA 6 (0.000156%)

GAGAGAGTGAGGAGAAGGCTTACGTTTAGTGAGGGAGAGATTTGGTATATGATTGAGATGGGGGCTAGTTTTTGTCATGTGAGAAGAAGCAGGCCGGATG 6 (0.000156%)

GAGCAAGGTGGGTCTCAGAGGTGATCGGCGATCAGAGGGCGATGAAGTTCTAGATCCATTGAGACAAGCTCTAGACAGTAGCATGCAGTCCCACAACTTG 5 (0.000130%)

GAGCAGTGAAGCCAGCTGCTTCCATTGGTGGGTCATTTTTGCTGTCACCAGCAACGTTGCCACGACGAACATCCTTGACAGACACATTCTTGACATTGAA 8 (0.000208%)

GAGCCCCATTGTGTTGTGGTAAATATGTAGAGGGAGTATAGGGCTGTGACTAGTATGTTGAGTCCTGTAAGTAGGAGAGTGATATTTGATCAGGAGAACG 4 (0.000104%)

GAGCCGTGTGGCAATCCAATACAGGGGCATAGCCGGCGCTTATTTGGCCTGGATGGTTCAGGATAATCACCTGAGCAGTGAAGCCAGCTGCTTCCATTGG 9 (0.000234%)

GAGCTTTCTGGGCAGACTTGGTGACCTTGCCAGCTCCAGCAGCCTTCTTGTCCACTGCTTTGATGACACCCACCGCAACTGTCTGTCTCATATCACGAAC 21 (0.000545%)

GAGGACCAACATGGGCATTTTGTTTATGAGCAAGGTGGGTCTCAGAGGTGATCGGCGATCAGAGGGCGATGAAGTTCTAGATCCATTGAGACAAGCTCTA 4 (0.000104%)

GAGGGAGTATAGGGCTGTGACTAGTATGTTGAGTCCTGTAAGTAGGAGAGTGATATTTGATCAGGAGAACGTGGTTACTAGCACAGAGAGTTCTCCCAGT 14 (0.000363%)

GAGTAGTTCCCTGCTAAGGGAGGGTAGACTGTTCAACCTGTTCCTGCTCCGGCCTCCACTATAGCAGATGCGAGCAGGAGTAGGAGAGAGGGAGGTAAGA 11 (0.000285%)

GAGTGAGCCCCATTGTGTTGTGGTAAATATGTAGAGGGAGTATAGGGCTGTGACTAGTATGTTGAGTCCTGTAAGTAGGAGAGTGATATTTGATCAGGAG 7 (0.000182%)

GATAATATTCATTTAGCCTTCTGAGCTTTCTGGGCAGACTTGGTGACCTTGCCAGCTCCAGCAGCCTTCTTGTCCACTGCTTTGATGACACCCACCGCAA 28 (0.000727%)

GATAATCACCTGAGCAGTGAAGCCAGCTGCTTCCATTGGTGGGTCATTTTGCTGTCACCAGCAACGTT
GCCACGACGAACATCCTTGACAGACACATTC 3 (0.000078%)

GATCAATCTTTTCCTTCAGCTCAGCAAACTTGCATGCAATGTGAGCCGTGTGGCAATCCAATACAGGGG
CATAGCCGGCGCTTATTTGGCCTGGATGGTT 28 (0.000727%)

GATCAGAGGGCGATGAAGTTCTAGATCCATTGAGACAAGCTCTAGACAGTAGCATGCAGTCCCACAAC
TTGTACCAGCATCCCCAGCGTCTGGCATTCCA 3 (0.000078%)

GATCGGAAGAGCACACGTCTGAACTCCAGTCACATCGCAATTGCGATCTGGGGGGGGGGGGGGGGGG
GGGGGGGGGGGGGGGGGGGGGGGGGGGGGGGGGGGGGGGGGGGGGGGGGGGGGGGGGGGGGGGGGG
GGGGGGGGGGGGGGG 951 (0.036773%)

GATGCCAGTGCCCCTGGGTGCAGGGATGAGGCGTACCAGCACAGAGCCGCAGCGGCCTGTCACCTTGC
AAGGGACAGTGTGGGGCTTGCCGATCTTGTTC 7 (0.000182%)

GATGGAGGTAGGATTGGTGCTGTGGGTGAAAGAGTATGATGGGGTGGTGGTTGTGGTAAACTTTAATA
GTGTAGGAAGCTGAATAATTTATGAAGGAGAG 3 (0.000078%)

GATGGTTCAGGATAATCACCTGAGCAGTGAAGCCAGCTGCTTCCATTGGTGGGTCATTTTGCTGTCACC
AGCAACGTTGCCACGACGAACATCCTTGAC 18 (0.000467%)

GATTGAGAGAGTGAGGAGAAGGCTTACGTTTAGTGAGGGAGAGATTTGGTATATGATTGAGATGGGGG
CTAGTTTTTGTCATGTGAGAAGAAGCAGGCCG 357 (0.009265%)

GCAAACTTGCATGCAATGTGAGCCGTGTGGCAATCCAATACAGGGGCATAGCCGGCGCTTATTTGGCCT
GGATGGTTCAGGATAATCACCTGAGCAGTGA 8 (0.000208%)

GCAATCCAATACAGGGGCATAGCCGGCGCTTATTTGGCCTGGATGGTTCAGGATAATCACCTGAGCAGT
GAAGCCAGCTGCTTCCATTGGTGGGTCATTT 25 (0.000649%)

GCAATGTGAGCCGTGTGGCAATCCAATACAGGGGCATAGCCGGCGCTTATTTGGCCTGGATGGTTCAGG
ATAATCACCTGAGCAGTGAAGCCAGCTGCTT 15 (0.000389%)

GCACAGTCTTGGTTCCCCGGAGACGTCCAGTCCGGCGGGCAGCAATGAGACCCACTTTGCGGCCAGCAG
GGGCATCTCTGCGGATGGTGGAGGGCTTGCC 14 (0.000363%)

GCACTGCCCACGGTGGGCGGGCGGGCCTCTCTACTCGAAGGTGACCACGTTTAGATTCTGAGACGGGAA
GTGGAGGGTGAATAGGTCACGGCGGCCTTTT 8 (0.000208%)

GCAGAATTTGGCTGTTTGGCTTCAACTCCTACTTTTTCCAGCACGATTCCTTTTGCATGAGAAGCACCTC
CAAAAGGGTTGGCCTTTAGGGCTGTGCCCA 32 (0.000830%)

GCAGACTTGGTGACCTTGCCAGCTCCAGCAGCCTTCTTGTCCACTGCTTTGATGACACCCACCGCAACTG
TCTGTCTCATATCACGAACAGCAAAGCGAC 71 (0.001843%)

GCAGCATCACCAGACTTCAAGAATTTAGGGCCATCTTCCAGCTTTTTACCAGAACGGCGATCAATCTTTT
CCTTCAGCTCAGCAAACTTGCATGCAATGT 30 (0.000779%)

GCAGCCTTCTTGTCCACTGCTTTGATGACACCCACCGCAACTGTCTGTCTCATATCACGAACAGCAAAGC
GACCCAAAGGTGGATAGTCTGAGAAGCTCT 10 (0.000260%)

GCAGGGATGAGGCGTACCAGCACAGAGCCGCAGCGGCCTGTCACCTTGCAAGGGACAGTGTGGGGCTT
GCCGATCTTGTTCCCCCAGTAGCCTCTGCGCA 9 (0.000234%)

GCATAATCCAGGGAATCATAAATCATGCCAAAGCCAGTTGTCTTGCCACCACCAAAATGAGTTCTGAAT
CCAAATACAAAGATGACATCCGGTGTGGTCT 15 (0.000389%)

GCATCACCAGACTTCAAGAATTTAGGGCCATCTTCCAGCTTTTTACCAGAACGGCGATCAATCTTTTCCT
TCAGCTCAGCAAACTTGCATGCAATGTGAG 21 (0.000545%)

GCATGCAATGTGAGCCGTGTGGCAATCCAATACAGGGGCATAGCCGGCGCTTATTTGGCCTGGATGGTT
CAGGATAATCACCTGAGCAGTGAAGCCAGCT 13 (0.000337%)

GCATTTTGTTTATGAGCAAGGTGGGTCTCAGAGGTGATCGGCGATCAGAGGGCGATGAAGTTCTAGATC
CATTGAGACAAGCTCTAGACAGTAGCATGCA 19 (0.000493%)

GCATTTTTAATCTTAGAGCGAAAGCCTATAATCACTGCGCCTGTTCATAAGGGGATGGCCATGGCTAGG
TTTATAGATAGTTGGGTGGTTGGTGTAAATG 10 (0.000260%)

GCCAATTTTCTTAATGTAAGTGCTGACTTCCTTAACAATTTCCTCATATCTCTTCTGGCTGTAGGGTGGCT
CAGTGGAATCCATTTTGTTAACACCGACA 36 (0.000934%)

GCCACCTACGGTGAAAAGAAAGATGAATCCTAGGGCTCAGAGCACTGCAGCAGATCATTTCATATTGCT
TCCGTGGAGTGTGGCGAGTCAGCTAAATACT 23 (0.000597%)

GCCAGCTCCAGCAGCCTTCTTGTCCACTGCTTTGATGACACCCACCGCAACTGTCTGTCTCATATCACGA
ACAGCAAAGCGACCCAAAGGTGGATAGTCT 54 (0.001401%)

GCCAGGAACCATATCAACAATGGCAGCATCACCAGACTTCAAGAATTTAGGGCCATCTTCCAGCTTTTT
ACCAGAACGGCGATCAATCTTTTCCTTCAGC 751 (0.019489%)

GCCAGTCAGGCCACCTACGGTGAAAAGAAAGATGAATCCTAGGGCTCAGAGCACTGCAGCAGATCATT
TCATATTGCTTCCGTGGAGTGTGGCGAGTCAG 20 (0.000519%)

GCCAGTGCCCCTGGGTGCAGGGATGAGGCGTACCAGCACAGAGCCGCAGCGGCCTGTCACCTTGCAAG
GGACAGTGTGGGGCTTGCCGATCTTGTTCCCC 18 (0.000467%)

GCCAGTGCCCTCCTAATTGGGGGGTAGGGGCTAGGCTGGAGTGGTAAAAGGCTCAGAAAAATCCTGCG
AAGAAAAAAACTTCTGAGGTAATAAATAGGAT 8 (0.000208%)

GCCCCATTGTGTTGTGGTAAATATGTAGAGGGAGTATAGGGCTGTGACTAGTATGTTGAGTCCTGTAAG
TAGGAGAGTGATATTTGATCAGGAGAACGTG 19 (0.000493%)

GCCCCTGGGTGCAGGGATGAGGCGTACCAGCACAGAGCCGCAGCGGCCTGTCACCTTGCAAGGGACAG
TGTGGGGCTTGCCGATCTTGTTCCCCCAGTAG 9 (0.000234%)

GCCCTCCTAATTGGGGGGTAGGGGCTAGGCTGGAGTGGTAAAAGGCTCAGAAAAATCCTGCGAAGAAA
AAAACTTCTGAGGTAATAAATAGGATTATCCC 5 (0.000130%)

GCCGTGTGGCAATCCAATACAGGGGCATAGCCGGCGCTTATTTGGCCTGGATGGTTCAGGATAATCACC
TGAGCAGTGAAGCCAGCTGCTTCCATTGGTG 17 (0.000441%)

GCCTAATGTGGGGACAGCTCATGAGTGCAAGACGTCTTGTGATGTAATTATTATACGAATGGGGGCTTC
AATCGGGAGTACTACTCGATTGTCAACGTCA 8 (0.000208%)

GCCTCGGACACGAAGGCCCCAGAAGTGACGCAGCCCTCTATGGGCCCGAATCTTCTTCAGTCGCTCCAG
GTCTTCACGGAGCTTGTTGTCCAGACCATTG 16 (0.000415%)

GCCTGAGAATAGGGGAAATCAGTGAATGAAGCCTCCTATGATGGCAAATACAGCTCCTATTGATAGGA
CATAGTGGAAGTGAGCTACAACGTAGTACGTG 4 (0.000104%)

GCCTGTTCATAAGGGGATGGCCATGGCTAGGTTTATAGATAGTTGGGTGGTTGGTGTAAATGAGTGAGG
CAGGAGTCCGAGGAGGTTAGTTGTGGCAATA 5 (0.000130%)

GCCTTCTGAGCTTTCTGGGCAGACTTGGTGACCTTGCCAGCTCCAGCAGCCTTCTTGTCCACTGCTTTGA

TGACACCCACCGCAACTGTCTGTCTCATAT 37 (0.000960%)

GCCTTCTTGTCCACTGCTTTGATGACACCCACCGCAACTGTCTGTCTCATATCACGAACAGCAAAGCGAC
CCAAAGGTGGATAGTCTGAGAAGCTCTCAA 20 (0.000519%)

GCCTTGGTATGTGCTTTCTCGTGTTACATCGCGCCATCATTGGTATATGGTTAGTGTGTTGGTTAGTAGG
CCTAGTATGAGGAGCGTTATGGAGTGGAAG 25 (0.000649%)

GCGACAGCGATTTCTAGGATAGTCAGTAGAATTAGAATTGTGAAGATGATAAGTGTAGAGGGAAGGTT
AATGGTTGATATTGCTAGGGTGGCGCTTCCAA 737 (0.019126%)

GCGATCAATCTTTTCCTTCAGCTCAGCAAACTTGCATGCAATGTGAGCCGTGTGGCAATCCAATACAGG
GGCATAGCCGGCGCTTATTTGGCCTGGATGG 42 (0.001090%)

GCGATCAGAGGGCGATGAAGTTCTAGATCCATTGAGACAAGCTCTAGACAGTAGCATGCAGTCCCACA
ACTTGTACCAGCATCCCCAGCGTCTGGCATTC 8 (0.000208%)

GCGATTTCTAGGATAGTCAGTAGAATTAGAATTGTGAAGATGATAAGTGTAGAGGGAAGGTTAATGGTT
GATATTGCTAGGGTGGCGCTTCCAATTAGGT 60 (0.001557%)

GCGGAGACGATGCCAGTGCCCCTGGGTGCAGGGATGAGGCGTACCAGCACAGAGCCGCAGCGGCCTGT
CACCTTGCAAGGGACAGTGTGGGGCTTGCCGA 27 (0.000701%)

GCGGGTGGCACTGCCCACGGTGGGCGGGCGGGCCTCTCTACTCGAAGGTGACCACGTTTAGATTCTGAG
ACGGGAAGTGGAGGGTGAATAGGTCACGGCG 15 (0.000389%)

GCGTAGGTTTGGTCTAGGGTGTAGCCTGAGAATAGGGGAAATCAGTGAATGAAGCCTCCTATGATGGC
AAATACAGCTCCTATTGATAGGACATAGTGGA16 (0.000415%)

GCTAAGGGAGGGTAGACTGTTCAACCTGTTCCTGCTCCGGCCTCCACTATAGCAGATGCGAGCAGGAGT
AGGAGAGAGGGAGGTAAGAGTCAGAAGCTTA 3 (0.000078%)

GCTAATACAATGCCAGTCAGGCCACCTACGGTGAAAAGAAAGATGAATCCTAGGGCTCAGAGCACTGC
AGCAGATCATTTCATATTGCTTCCGTGGAGTG 17 (0.000441%)

GCTCAGCAAACTTGCATGCAATGTGAGCCGTGTGGCAATCCAATACAGGGGCATAGCCGGCGCTTATTT
GGCCTGGATGGTTCAGGATAATCACCTGAGC 13 (0.000337%)

GCTCCAGCAGCCTTCTTGTCCACTGCTTTGATGACACCCACCGCAACTGTCTGTCTCATATCACGAACAG
CAAAGCGACCCAAAGGTGGATAGTCTGAGA 7 (0.000182%)

GCTGACTTCCTTAACAATTTCCTCATATCTCTTCTGGCTGTAGGGTGGCTCAGTGGAATCCATTTTGTTA
ACACCGACAATTAGTTGTTTCACACCCAGT 82 (0.002128%)

GCTGGTCTCAAATTTCCACAAGGAGATATCAATGGTGATACCACGTTCACGCTCAGCTTTCAGTTTATCC
AAGACCCAGGCATACTTGAAGGAGCCCTTT 48 (0.001246%)

GCTGTGACTAGTATGTTGAGTCCTGTAAGTAGGAGAGTGATATTTGATCAGGAGAACGTGGTTACTAGC
ACAGAGAGTTCTCCCAGTAGGTTAATAGTGG 10 (0.000260%)

GCTTCTTAGGCACAGGTGCGGAGACGATGCCAGTGCCCCTGGGTGCAGGGATGAGGCGTACCAGCACA
GAGCCGCAGCGGCCTGTCACCTTGCAAGGGAC 4 (0.000104%)

GCTTTCTCGTGTTACATCGCGCCATCATTGGTATATGGTTAGTGTGTTGGTTAGTAGGCCTAGTATGAGG
AGCGTTATGGAGTGGAAGTGAAATCACATG 20 (0.000519%)

GCTTTCTGGGCAGACTTGGTGACCTTGCCAGCTCCAGCAGCCTTCTTGTCCACTGCTTTGATGACACCCA
CCGCAACTGTCTGTCTCATATCACGAACAG 15 (0.000389%)

GCTTTTTACCAGAACGGCGATCAATCTTTTCCTTCAGCTCAGCAAACTTGCATGCAATGTGAGCCGTGTG
GCAATCCAATACAGGGGCATAGCCGGCGCT 5 (0.000130%)

GGAACCATATCAACAATGGCAGCATCACCAGACTTCAAGAATTTAGGGCCATCTTCCAGCTTTTTACCA
GAACGGCGATCAATCTTTTCCTTCAGCTCAG 36 (0.000934%)

GGACACGAAGGCCCCAGAAGTGACGCAGCCCTCTATGGGCCCGAATCTTCTTCAGTCGCTCCAGGTCTT
CACGGAGCTTGTTGTCCAGACCATTGGCTAG 10 (0.000260%)

GGACCAACATGGGCATTTTGTTTATGAGCAAGGTGGGTCTCAGAGGTGATCGGCGATCAGAGGGCGAT
GAAGTTCTAGATCCATTGAGACAAGCTCTAGA 13 (0.000337%)

GGAGACGATGCCAGTGCCCCTGGGTGCAGGGATGAGGCGTACCAGCACAGAGCCGCAGCGGCCTGTCA
CCTTGCAAGGGACAGTGTGGGGCTTGCCGATC 48 (0.001246%)

GGAGACGTCCAGTCCGGCGGGCAGCAATGAGACCCACTTTGCGGCCAGCAGGGGCATCTCTGCGGATG
GTGGAGGGCTTGCCGATGTGCTGGTGGTTGCC 9 (0.000234%)

GGAGATATCAATGGTGATACCACGTTCACGCTCAGCTTTCAGTTTATCCAAGACCCAGGCATACTTGAA
GGAGCCCTTTCCCATCTCAGCAGCCTCCTTC 20 (0.000519%)

GGAGTAGTTCCCTGCTAAGGGAGGGTAGACTGTTCAACCTGTTCCTGCTCCGGCCTCCACTATAGCAGA
TGCGAGCAGGAGTAGGAGAGAGGGAGGTAAG 24 (0.000623%)

GGAGTATAGGGCTGTGACTAGTATGTTGAGTCCTGTAAGTAGGAGAGTGATATTTGATCAGGAGAACGT
GGTTACTAGCACAGAGAGTTCTCCCAGTAGG 30 (0.000779%)

GGATAATATTCATTTAGCCTTCTGAGCTTTCTGGGCAGACTTGGTGACCTTGCCAGCTCCAGCAGCCTTC
TTGTCCACTGCTTTGATGACACCCACCGCA 171 (0.004438%)

GGATAATCACCTGAGCAGTGAAGCCAGCTGCTTCCATTGGTGGGTCATTTTTGCTGTCACCAGCAACGT
TGCCACGACGAACATCCTTGACAGACACATT 51 (0.001324%)

GGATAGTCAGTAGAATTAGAATTGTGAAGATGATAAGTGTAGAGGGAAGGTTAATGGTTGATATTGCT
AGGGTGGCGCTTCCAATTAGGTGCATGAGTAG 7 (0.000182%)

GGATGAGGCGTACCAGCACAGAGCCGCAGCGGCCTGTCACCTTGCAAGGGACAGTGTGGGGCTTGCCG
ATCTTGTTCCCCCAGTAGCCTCTGCGCACGGG 4 (0.000104%)

GGATGGTTCAGGATAATCACCTGAGCAGTGAAGCCAGCTGCTTCCATTGGTGGGTCATTTTTGCTGTCA
CCAGCAACGTTGCCACGACGAACATCCTTGA 918 (0.023823%)

GGATTGAAAAGTGAACAGATATTCAGCATCTAACAGTTCAAAAGAAGCCACTACATACTCTTTTCACAA
ATATGTTTTCACAGAGCCAATACAGTACTAG 5 (0.000130%)

GGATTTTGGCGTAGGTTTGGTCTAGGGTGTAGCCTGAGAATAGGGGAAATCAGTGAATGAAGCCTCCTA
TGATGGCAAATACAGCTCCTATTGATAGGAC 12 (0.000311%)

GGCAATCCAATACAGGGGCATAGCCGGCGCTTATTTGGCCTGGATGGTTCAGGATAATCACCTGAGCAG
TGAAGCCAGCTGCTTCCATTGGTGGGTCATT 58 (0.001505%)

GGCACTGCCCACGGTGGGCGGGCGGGCCTCTCTACTCGAAGGTGACCACGTTTAGATTCTGAGACGGGA
AGTGGAGGGTGAATAGGTCACGGCGGCCTTT 6 (0.000156%)

GGCAGAATTTGGCTGTTTGGCTTCAACTCCTACTTTTTCCAGCACGATTCCTTTTGCATGAGAAGCACCT
CCAAAAGGGTTGGCCTTTAGGGCTGTGCCC 10 (0.000260%)

GGCAGACTTGGTGACCTTGCCAGCTCCAGCAGCCTTCTTGTCCACTGCTTTGATGACACCCACCGCAACT
GTCTGTCTCATATCACGAACAGCAAAGCGA 107 (0.002777%)

GGCAGAGGACCAACATGGGCATTTTGTTTATGAGCAAGGTGGGTCTCAGAGGTGATCGGCGATCAGAG
GGCGATGAAGTTCTAGATCCATTGAGACAAGC7 (0.000182%)

GGCAGCATCACCAGACTTCAAGAATTTAGGGCCATCTTCCAGCTTTTTACCAGAACGGCGATCAATCTT
TTCCTTCAGCTCAGCAAACTTGCATGCAATG 57 (0.001479%)

GGCATTCGGGCATTGGCTGTACCCTTCCGCTTACCTATGCCCATGTGCCTGCCCTTCCGGCGGGCCAAGG
TGTTTTTCCGGCATCGAGCCCGGGAATGGA 3 (0.000078%)

GGCATTTTGTTTATGAGCAAGGTGGGTCTCAGAGGTGATCGGCGATCAGAGGGCGATGAAGTTCTAGAT
CCATTGAGACAAGCTCTAGACAGTAGCATGC 36 (0.000934%)

GGCATTTTTAATCTTAGAGCGAAAGCCTATAATCACTGCGCCTGTTCATAAGGGGATGGCCATGGCTAG
GTTTATAGATAGTTGGGTGGTTGGTGTAAAT 270 (0.007007%)

GGCCACCTACGGTGAAAAGAAAGATGAATCCTAGGGCTCAGAGCACTGCAGCAGATCATTTCATATTG
CTTCCGTGGAGTGTGGCGAGTCAGCTAAATAC 8 (0.000208%)

GGCCAGTGCCCTCCTAATTGGGGGGTAGGGGCTAGGCTGGAGTGGTAAAAGGCTCAGAAAAATCCTGC
GAAGAAAAAAACTTCTGAGGTAATAAATAGGA 20 (0.000519%)

GGCCCCAGAAGTGACGCAGCCCTCTATGGGCCCGAATCTTCTTCAGTCGCTCCAGGTCTTCACGGAGCT
TGTTGTCCAGACCATTGGCTAGGACCTGGCT 3 (0.000078%)

GGCCTTGGTATGTGCTTTCTCGTGTTACATCGCGCCATCATTGGTATATGGTTAGTGTGTTGGTTAGTAG
GCCTAGTATGAGGAGCGTTATGGAGTGGAA 22 (0.000571%)

GGCGATCAATCTTTTCCTTCAGCTCAGCAAACTTGCATGCAATGTGAGCCGTGTGGCAATCCAATACAG
GGGCATAGCCGGCGCTTATTTGGCCTGGATG 155 (0.004022%)

GGCGATCAGAGGGCGATGAAGTTCTAGATCCATTGAGACAAGCTCTAGACAGTAGCATGCAGTCCCAC
AACTTGTACCAGCATCCCCAGCGTCTGGCATT 14 (0.000363%)

GGCGTAGGTTTGGTCTAGGGTGTAGCCTGAGAATAGGGGAAATCAGTGAATGAAGCCTCCTATGATGG
CAAATACAGCTCCTATTGATAGGACATAGTGG 9 (0.000234%)

GGCTGTGACTAGTATGTTGAGTCCTGTAAGTAGGAGAGTGATATTTGATCAGGAGAACGTGGTTACTAG
CACAGAGAGTTCTCCCAGTAGGTTAATAGTG 16 (0.000415%)

GGGAATCATAAATCATGCCAAAGCCAGTTGTCTTGCCACCACCAAAATGAGTTCTGAATCCAAATACAA
AGATGACATCCGGTGTGGTCTTGTACATTTT 25 (0.000649%)

GGGAGTAGTTCCCTGCTAAGGGAGGGTAGACTGTTCAACCTGTTCCTGCTCCGGCCTCCACTATAGCAG
ATGCGAGCAGGAGTAGGAGAGAGGGAGGTAA32 (0.000830%)

GGGAGTATAGGGCTGTGACTAGTATGTTGAGTCCTGTAAGTAGGAGAGTGATATTTGATCAGGAGAAC
GTGGTTACTAGCACAGAGAGTTCTCCCAGTAG 15 (0.000389%)

GGGATAATATTCATTTAGCCTTCTGAGCTTTCTGGGCAGACTTGGTGACCTTGCCAGCTCCAGCAGCCTT
CTTGTCCACTGCTTTGATGACACCCACCGC 34 (0.000882%)

GGGATGAGGCGTACCAGCACAGAGCCGCAGCGGCCTGTCACCTTGCAAGGGACAGTGTGGGGCTTGCC
GATCTTGTTCCCCCAGTAGCCTCTGCGCACGG 10 (0.000260%)

GGGATTGAAAAGTGAACAGATATTCAGCATCTAACAGTTCAAAAGAAGCCACTACATACTCTTTTCACA

AATATGTTTTCACAGAGCCAATACAGTACTA 3 (0.000078%)

GGGCAGACTTGGTGACCTTGCCAGCTCCAGCAGCCTTCTTGTCCACTGCTTTGATGACACCCACCGCAA
CTGTCTGTCTCATATCACGAACAGCAAAGCG 31 (0.000804%)

GGGCAGAGGACCAACATGGGCATTTTGTTTATGAGCAAGGTGGGTCTCAGAGGTGATCGGCGATCAGA
GGGCGATGAAGTTCTAGATCCATTGAGACAAG 20 (0.000519%)

GGGCATTTTGTTTATGAGCAAGGTGGGTCTCAGAGGTGATCGGCGATCAGAGGGCGATGAAGTTCTAGA
TCCATTGAGACAAGCTCTAGACAGTAGCATG 59 (0.001531%)

GGGCCATCTTCCAGCTTTTTACCAGAACGGCGATCAATCTTTTCCTTCAGCTCAGCAAACTTGCATGCAA
TGTGAGCCGTGTGGCAATCCAATACAGGGG 139 (0.003607%)

GGGGCCAGTGCCCTCCTAATTGGGGGGTAGGGGCTAGGCTGGAGTGGTAAAAGGCTCAGAAAAATCCT
GCGAAGAAAAAAACTTCTGAGGTAATAAATAG 28 (0.000727%)

GGGGGCCAGTGCCCTCCTAATTGGGGGGTAGGGGCTAGGCTGGAGTGGTAAAAGGCTCAGAAAAATCC
TGCGAAGAAAAAAACTTCTGAGGTAATAAATA 21 (0.000545%)
GGGGGGGGGGGGGGGGGGGGGGGGGGGGGGGGGGGGG 5741 (0.059595%)

GGGTCTCAAAATTCTGTGACAAATTTTTGGTCAAGTTGTTTCCATTAAAAAGTACTGATTTTAAAAACTA
ATAACTTAAAACTGCCACACGCAAAAAAGA 56 (0.001453%)

GGGTGAAAGAGTATGATGGGGTGGTGGTTGTGGTAAACTTTAATAGTGTAGGAAGCTGAATAATTTATG
AAGGAGAGGGGTCAGGGTTGATTCGGGAGGA 5 (0.000130%)

GGGTGAGTGAGCCCCATTGTGTTGTGGTAAATATGTAGAGGGAGTATAGGGCTGTGACTAGTATGTTGA
GTCCTGTAAGTAGGAGAGTGATATTTGATCA 52 (0.001349%)

GGGTGGCACTGCCCACGGTGGGCGGGCGGGCCTCTCTACTCGAAGGTGACCACGTTTAGATTCTGAGAC
GGGAAGTGGAGGGTGAATAGGTCACGGCGGC 8 (0.000208%)

GGGTGTAGCCTGAGAATAGGGGAAATCAGTGAATGAAGCCTCCTATGATGGCAAATACAGCTCCTATT
GATAGGACATAGTGGAAGTGAGCTACAACGTA 20 (0.000519%)

GGGTTAGCGATGGAGGTAGGATTGGTGCTGTGGGTGAAAGAGTATGATGGGGTGGTGGTTGTGGTAAA
CTTTAATAGTGTAGGAAGCTGAATAATTTATG 110 (0.002855%)

GGGTTGTAGCCAATTTTCTTAATGTAAGTGCTGACTTCCTTAACAATTTCCTCATATCTCTTCTGGCTGTA
GGGTGGCTCAGTGGAATCCATTTTGTTAA 1063 (0.027586%)

GGTAAATATGTAGAGGGAGTATAGGGCTGTGACTAGTATGTTGAGTCCTGTAAGTAGGAGAGTGATATT
TGATCAGGAGAACGTGGTTACTAGCACAGAG 11 (0.000285%)

GGTAGGATTGGTGCTGTGGGTGAAAGAGTATGATGGGGTGGTGGTTGTGGTAAACTTTAATAGTGTAGG
AAGCTGAATAATTTATGAAGGAGAGGGGTCA 3 (0.000078%)

GGTATGTGCTTTCTCGTGTTACATCGCGCCATCATTGGTATATGGTTAGTGTGTTGGTTAGTAGGCCTAG
TATGAGGAGCGTTATGGAGTGGAAGTGAAA 3 (0.000078%)

GGTCAAGTTGTTTCCATTAAAAAGTACTGATTTTAAAAACTAATAACTTAAAACTGCCACACGCAAAAA
AGAAAACCAAAGTGGTCCACAAAACATTCTC 9 (0.000234%)

GGTCCAGGGCAGAGGACCAACATGGGCATTTTGTTTATGAGCAAGGTGGGTCTCAGAGGTGATCGGCG
ATCAGAGGGCGATGAAGTTCTAGATCCATTGA4 (0.000104%)

GGTCTAGGGTGTAGCCTGAGAATAGGGGAAATCAGTGAATGAAGCCTCCTATGATGGCAAATACAGCT

CCTATTGATAGGACATAGTGGAAGTGAGCTAC3 (0.000078%)

GGTCTCAAAATTCTGTGACAAATTTTGGTCAAGTTGTTTCCATTAAAAAGTACTGATTTTAAAAACTAA
TAACTTAAAACTGCCACACGCAAAAAAGAA 5 (0.000130%)

GGTCTCAAATTTCCACAAGGAGATATCAATGGTGATACCACGTTCACGCTCAGCTTTCAGTTTATCCAA
GACCCAGGCATACTTGAAGGAGCCCTTTCCC 3 (0.000078%)

GGTGACCTTGCCAGCTCCAGCAGCCTTCTTGTCCACTGCTTTGATGACACCCACCGCAACTGTCTGTCTC
ATATCACGAACAGCAAAGCGACCCAAAGGT 17 (0.000441%)

GGTGAGTGAGCCCCATTGTGTTGTGGTAAATATGTAGAGGGAGTATAGGGCTGTGACTAGTATGTTGAG
TCCTGTAAGTAGGAGAGTGATATTTGATCAG 22 (0.000571%)

GGTGGCACTGCCCACGGTGGGCGGGCGGGCCTCTCTACTCGAAGGTGACCACGTTTAGATTCTGAGACG
GGAAGTGGAGGGTGAATAGGTCACGGCGGCC 5 (0.000130%)

GGTGGCCTTGGTATGTGCTTTCTCGTGTTACATCGCGCCATCATTGGTATATGGTTAGTGTGTTGGTTAG
TAGGCCTAGTATGAGGAGCGTTATGGAGTG 18 (0.000467%)

GGTGGGTGAGTGAGCCCCATTGTGTTGTGGTAAATATGTAGAGGGAGTATAGGGCTGTGACTAGTATGT
TGAGTCCTGTAAGTAGGAGAGTGATATTTGA 3 (0.000078%)

GGTGTAGCCTGAGAATAGGGGAAATCAGTGAATGAAGCCTCCTATGATGGCAAATACAGCTCCTATTG
ATAGGACATAGTGGAAGTGAGCTACAACGTAG 12 (0.000311%)

GGTTAGCGATGGAGGTAGGATTGGTGCTGTGGGTGAAAGAGTATGATGGGGTGGTGGTTGTGGTAAAC
TTTAATAGTGTAGGAAGCTGAATAATTTATGA 3 (0.000078%)

GGTTGTAGCCAATTTTCTTAATGTAAGTGCTGACTTCCTTAACAATTTCCTCATATCTCTTCTGGCTGTAG
GGTGGCTCAGTGGAATCCATTTTGTTAAC 7 (0.000182%)

GGTTTGGTCTAGGGTGTAGCCTGAGAATAGGGGAAATCAGTGAATGAAGCCTCCTATGATGGCAAATA
CAGCTCCTATTGATAGGACATAGTGGAAGTGA17 (0.000441%)

GTAAATATGTAGAGGGAGTATAGGGCTGTGACTAGTATGTTGAGTCCTGTAAGTAGGAGAGTGATATTT
GATCAGGAGAACGTGGTTACTAGCACAGAGA 43 (0.001116%)

GTAAGTGCTGACTTCCTTAACAATTTCCTCATATCTCTTCTGGCTGTAGGGTGGCTCAGTGGAATCCATT
TTGTTAACACCGACAATTAGTTGTTTCACA 9 (0.000234%)

GTAGAATTAGAATTGTGAAGATGATAAGTGTAGAGGGAAGGTTAATGGTTGATATTGCTAGGGTGGCG
CTTCCAATTAGGTGCATGAGTAGGTGGCCTGC 70 (0.001817%)

GTAGAGGGAGTATAGGGCTGTGACTAGTATGTTGAGTCCTGTAAGTAGGAGAGTGATATTTGATCAGGA
GAACGTGGTTACTAGCACAGAGAGTTCTCCC 8 (0.000208%)

GTAGCCAATTTTCTTAATGTAAGTGCTGACTTCCTTAACAATTTCCTCATATCTCTTCTGGCTGTAGGGTG
GCTCAGTGGAATCCATTTTGTTAACACCG 22 (0.000571%)

GTAGCCTGAGAATAGGGGAAATCAGTGAATGAAGCCTCCTATGATGGCAAATACAGCTCCTATTGATA
GGACATAGTGGAAGTGAGCTACAACGTAGTAC 6 (0.000156%)

GTAGGATTGGTGCTGTGGGTGAAAGAGTATGATGGGGTGGTGGTTGTGGTAAACTTTAATAGTGTAGGA
AGCTGAATAATTTATGAAGGAGAGGGGTCAG 13 (0.000337%)

GTAGGTTTGGTCTAGGGTGTAGCCTGAGAATAGGGGAAATCAGTGAATGAAGCCTCCTATGATGGCAA
ATACAGCTCCTATTGATAGGACATAGTGGAAG44 (0.001142%)

GTAGTTCCCTGCTAAGGGAGGGTAGACTGTTCAACCTGTTCCTGCTCCGGCCTCCACTATAGCAGATGC
GAGCAGGAGTAGGAGAGAGGGAGGTAAGAGT26 (0.000675%)

GTATAGGGCTGTGACTAGTATGTTGAGTCCTGTAAGTAGGAGAGTGATATTTGATCAGGAGAACGTGGT
TACTAGCACAGAGAGTTCTCCCAGTAGGTTA 8 (0.000208%)

GTATGTGCTTTCTCGTGTTACATCGCGCCATCATTGGTATATGGTTAGTGTGTTGGTTAGTAGGCCTAGT
ATGAGGAGCGTTATGGAGTGGAAGTGAAAT 3 (0.000078%)

GTCAAGTTGTTTCCATTAAAAAGTACTGATTTTAAAAACTAATAACTTAAAACTGCCACACGCAAAAAA
GAAAACCAAAGTGGTCCACAAAACATTCTCC 51 (0.001324%)

GTCACAGGTCCAGGGCAGAGGACCAACATGGGCATTTTGTTTATGAGCAAGGTGGGTCTCAGAGGTGA
TCGGCGATCAGAGGGCGATGAAGTTCTAGATC18 (0.000467%)

GTCAGGCCACCTACGGTGAAAAGAAAGATGAATCCTAGGGCTCAGAGCACTGCAGCAGATCATTTCAT
ATTGCTTCCGTGGAGTGTGGCGAGTCAGCTAA 19 (0.000493%)

GTCAGTAGAATTAGAATTGTGAAGATGATAAGTGTAGAGGGAAGGTTAATGGTTGATATTGCTAGGGT
GGCGCTTCCAATTAGGTGCATGAGTAGGTGGC 7 (0.000182%)

GTCATCTGCGGGTGGCACTGCCCACGGTGGGCGGGCGGGCCTCTCTACTCGAAGGTGACCACGTTTAGA
TTCTGAGACGGGAAGTGGAGGGTGAATAGGT 794 (0.020605%)

GTCCAGGGCAGAGGACCAACATGGGCATTTTGTTTATGAGCAAGGTGGGTCTCAGAGGTGATCGGCGA
TCAGAGGGCGATGAAGTTCTAGATCCATTGAG18 (0.000467%)

GTCCAGTCCGGCGGGCAGCAATGAGACCCACTTTGCGGCCAGCAGGGGCATCTCTGCGGATGGTGGAG
GGCTTGCCGATGTGCTGGTGGTTGCCACCTCC 13 (0.000337%)

GTCCGGCGGGCAGCAATGAGACCCACTTTGCGGCCAGCAGGGGCATCTCTGCGGATGGTGGAGGGCTT
GCCGATGTGCTGGTGGTTGCCACCTCCAAAAG 3 (0.000078%)

GTCGATGCCACCGCATTTATAGATCAGATGGCCAGTAGTGGTGGACTTGCCCGAATCTACGTGTCCAAT
GACGACAATGTTGATATGAGTCTTTTCCTTT 43 (0.001116%)

GTCGGTTGTTGATGAGATATTTGGAGGTGGGGATCAATAGAGGGGGAAATAGAATGATCAGTACTGCG
GCGGGTAGGCCTAGGATTGTGGGGGCAATGAA 843 (0.021877%)

GTCTAGGGTGTAGCCTGAGAATAGGGGAAATCAGTGAATGAAGCCTCCTATGATGGCAAATACAGCTC
CTATTGATAGGACATAGTGGAAGTGAGCTACA6 (0.000156%)

GTCTCAAAATTCTGTGACAAATTTTTGGTCAAGTTGTTTCCATTAAAAAGTACTGATTTTAAAAACTAAT
AACTTAAAACTGCCACACGCAAAAAAGAAA 7 (0.000182%)

GTCTCAAATTTCCACAAGGAGATATCAATGGTGATACCACGTTCACGCTCAGCTTTCAGTTTATCCAAG
ACCCAGGCATACTTGAAGGAGCCCTTTCCCA 8 (0.000208%)

GTCTTGGTTCCCCGGAGACGTCCAGTCCGGCGGGCAGCAATGAGACCCACTTTGCGGCCAGCAGGGGC
ATCTCTGCGGATGGTGGAGGGCTTGCCGATGT 4 (0.000104%)

GTCTTTTACTACTAAACTTAAATGGCCAATTGAAACAAACAGTTCTGAGACCGTTCTTCCACCACTGATT
AAGAGTGGGGTGGCAGGTATTAGGGATAAT 36 (0.000934%)

GTGAAAGAGTATGATGGGGTGGTGGTTGTGGTAAACTTTAATAGTGTAGGAAGCTGAATAATTTATGAA
GGAGAGGGGTCAGGGTTGATTCGGGAGGATC 4 (0.000104%)

GTGACAAATTTTTGGTCAAGTTGTTTCCATTAAAAAGTACTGATTTTAAAAACTAATAACTTAAAACTGC
CACACGCAAAAAAGAAAACCAAAGTGGTCC 12 (0.000311%)

GTGACCTTCTCTGGCATTCGGGCATTGGCTGTACCCTTCCGCTTACCTATGCCCATGTGCCTGCCCTTCC
GGCGGGCCAAGGTGTTTTCCGGCATCGAG 5 (0.000130%)

GTGACCTTGCCAGCTCCAGCAGCCTTCTTGTCCACTGCTTTGATGACACCCACCGCAACTGTCTGTCTCA
TATCACGAACAGCAAAGCGACCCAAAGGTG 17 (0.000441%)

GTGAGCCCCATTGTGTTGTGGTAAATATGTAGAGGGAGTATAGGGCTGTGACTAGTATGTTGAGTCCTG
TAAGTAGGAGAGTGATATTTGATCAGGAGAA 17 (0.000441%)

GTGAGCCGTGTGGCAATCCAATACAGGGGCATAGCCGGCGCTTATTTGGCCTGGATGGTTCAGGATAAT
CACCTGAGCAGTGAAGCCAGCTGCTTCCATT 9 (0.000234%)

GTGAGGAGAAGGCTTACGTTTAGTGAGGGAGAGATTTGGTATATGATTGAGATGGGGGCTAGTTTTTGT
CATGTGAGAAGAAGCAGGCCGGATGTCAGAG 10 (0.000260%)

GTGAGTGAGCCCCATTGTGTTGTGGTAAATATGTAGAGGGAGTATAGGGCTGTGACTAGTATGTTGAGT
CCTGTAAGTAGGAGAGTGATATTTGATCAGG 21 (0.000545%)

GTGCCCCTGGGTGCAGGGATGAGGCGTACCAGCACAGAGCCGCAGCGGCCTGTCACCTTGCAAGGGAC
AGTGTGGGGCTTGCCGATCTTGTTCCCCCAGT 7 (0.000182%)

GTGCCCTCCTAATTGGGGGGTAGGGGCTAGGCTGGAGTGGTAAAAGGCTCAGAAAAATCCTGCGAAGA
AAAAAACTTCTGAGGTAATAAATAGGATTATC13 (0.000337%)

GTGCTGACTTCCTTAACAATTTCCTCATATCTCTTCTGGCTGTAGGGTGGCTCAGTGGAATCCATTTTGTT
AACACCGACAATTAGTTGTTTCACACCCA 24 (0.000623%)

GTGCTTTCTCGTGTTACATCGCGCCATCATTGGTATATGGTTAGTGTGTTGGTTAGTAGGCCTAGTATGA
GGAGCGTTATGGAGTGGAAGTGAAATCACA 20 (0.000519%)

GTGGCAATCCAATACAGGGGCATAGCCGGCGCTTATTTGGCCTGGATGGTTCAGGATAATCACCTGAGC
AGTGAAGCCAGCTGCTTCCATTGGTGGGTCA 31 (0.000804%)

GTGGCACTGCCCACGGTGGGCGGGCGGGCCTCTCTACTCGAAGGTGACCACGTTTAGATTCTGAGACGG
GAAGTGGAGGGTGAATAGGTCACGGCGGCCT 10 (0.000260%)

GTGGCCTTGGTATGTGCTTTCTCGTGTTACATCGCGCCATCATTGGTATATGGTTAGTGTGTTGGTTAGT
AGGCCTAGTATGAGGAGCGTTATGGAGTGG 30 (0.000779%)

GTGGGAGTAGTTCCCTGCTAAGGGAGGGTAGACTGTTCAACCTGTTCCTGCTCCGGCCTCCACTATAGC
AGATGCGAGCAGGAGTAGGAGAGAGGGAGGT532 (0.013806%)

GTGGGGACAGCTCATGAGTGCAAGACGTCTTGTGATGTAATTATTATACGAATGGGGGCTTCAATCGGG
AGTACTACTCGATTGTCAACGTCAAGGAGTC 16 (0.000415%)

GTGGGTGAGTGAGCCCCATTGTGTTGTGGTAAATATGTAGAGGGAGTATAGGGCTGTGACTAGTATGTT
GAGTCCTGTAAGTAGGAGAGTGATATTTGAT 4 (0.000104%)

GTGGTAAATATGTAGAGGGAGTATAGGGCTGTGACTAGTATGTTGAGTCCTGTAAGTAGGAGAGTGAT
ATTTGATCAGGAGAACGTGGTTACTAGCACAG7 (0.000182%)

GTGGTGGCCTTGGTATGTGCTTTCTCGTGTTACATCGCGCCATCATTGGTATATGGTTAGTGTGTTGGTT
AGTAGGCCTAGTATGAGGAGCGTTATGGAG 666 (0.017284%)

GTGTAGCCTGAGAATAGGGGAAATCAGTGAATGAAGCCTCCTATGATGGCAAATACAGCTCCTATTGAT

AGGACATAGTGGAAGTGAGCTACAACGTAGT 11 (0.000285%)

GTGTGGCAATCCAATACAGGGGCATAGCCGGCGCTTATTTGGCCTGGATGGTTCAGGATAATCACCTGA
GCAGTGAAGCCAGCTGCTTCCATTGGTGGGT 10 (0.000260%)

GTGTTACATCGCGCCATCATTGGTATATGGTTAGTGTGTTGGTTAGTAGGCCTAGTATGAGGAGCGTTAT
GGAGTGGAAGTGAAATCACATGGCTAGGCC 4 (0.000104%)

GTTCAGGATAATCACCTGAGCAGTGAAGCCAGCTGCTTCCATTGGTGGGTCATTTTTGCTGTCACCAGC
AACGTTGCCACGACGAACATCCTTGACAGAC 34 (0.000882%)

GTTCATAAGGGGATGGCCATGGCTAGGTTTATAGATAGTTGGGTGGTTGGTGTAAATGAGTGAGGCAGG
AGTCCGAGGAGGTTAGTTGTGGCAATAAAAA 124 (0.003218%)

GTTCCCCGGAGACGTCCAGTCCGGCGGGCAGCAATGAGACCCACTTTGCGGCCAGCAGGGGCATCTCTG
CGGATGGTGGAGGGCTTGCCGATGTGCTGGT 4 (0.000104%)

GTTCCCTGCTAAGGGAGGGTAGACTGTTCAACCTGTTCCTGCTCCGGCCTCCACTATAGCAGATGCGAG
CAGGAGTAGGAGAGAGGGAGGTAAGAGTCAG16 (0.000415%)

GTTCTTTTGTCGATGCCACCGCATTTATAGATCAGATGGCCAGTAGTGGTGGACTTGCCCGAATCTACGT
GTCCAATGACGACAATGTTGATATGAGTCT 27 (0.000701%)

GTTGGGGGCCAGTGCCCTCCTAATTGGGGGGTAGGGGCTAGGCTGGAGTGGTAAAAGGCTCAGAAAAA
TCCTGCGAAGAAAAAAACTTCTGAGGTAATAA 23 (0.000597%)

GTTGTAGCCAATTTTCTTAATGTAAGTGCTGACTTCCTTAACAATTTCCTCATATCTCTTCTGGCTGTAGG
GTGGCTCAGTGGAATCCATTTTGTTAACA 99 (0.002569%)

GTTGTGGTAAATATGTAGAGGGAGTATAGGGCTGTGACTAGTATGTTGAGTCCTGTAAGTAGGAGAGTG
ATATTTGATCAGGAGAACGTGGTTACTAGCA 8 (0.000208%)

GTTGTTTCCATTAAAAAGTACTGATTTTAAAAACTAATAACTTAAAACTGCCACACGCAAAAAAGAAAA
CCAAAGTGGTCCACAAAACATTCTCCTTTCC 22 (0.000571%)

GTTTATGAGCAAGGTGGGTCTCAGAGGTGATCGGCGATCAGAGGGCGATGAAGTTCTAGATCCATTGA
GACAAGCTCTAGACAGTAGCATGCAGTCCCAC 12 (0.000311%)

GTTTCCATTAAAAAGTACTGATTTTAAAAACTAATAACTTAAAACTGCCACACGCAAAAAAGAAAACCA
AAGTGGTCCACAAAACATTCTCCTTTCCTTC 66 (0.001713%)

GTTTGGTCTAGGGTGTAGCCTGAGAATAGGGGAAATCAGTGAATGAAGCCTCCTATGATGGCAAATAC
AGCTCCTATTGATAGGACATAGTGGAAGTGAG132 (0.003426%)

GTTTTTAAGCCTAATGTGGGGACAGCTCATGAGTGCAAGACGTCTTGTGATGTAATTATTATACGAATG
GGGGCTTCAATCGGGAGTACTACTCGATTGT 19 (0.000493%)

TAAATATGTAGAGGGAGTATAGGGCTGTGACTAGTATGTTGAGTCCTGTAAGTAGGAGAGTGATATTTG
ATCAGGAGAACGTGGTTACTAGCACAGAGAG 20 (0.000519%)

TAACAATTTCCTCATATCTCTTCTGGCTGTAGGGTGGCTCAGTGGAATCCATTTTGTTAACACCGACAAT
TAGTTGTTTCACACCCAGTGTGTAAGCCAG 17 (0.000441%)

TAACAGTTCAAAAGAAGCCACTACATACTCTTTTCACAAATATGTTTTCACAGAGCCAATACAGTACTA
GCCATTAACCCAGTACACCAAGTGTACTGAA 4 (0.000104%)

TAACCAGTCTTTTACTACTAAACTTAAATGGCCAATTGAAACAAACAGTTCTGAGACCGTTCTTCCACCA
CTGATTAAGAGTGGGGTGGCAGGTATTAGG 16 (0.000415%)

TAAGTGCTGACTTCCTTAACAATTTCCTCATATCTCTTCTGGCTGTAGGGTGGCTCAGTGGAATCCATTT
TGTTAACACCGACAATTAGTTGTTTCACAC 3 (0.000078%)

TAATACAATGCCAGTCAGGCCACCTACGGTGAAAAGAAAGATGAATCCTAGGGCTCAGAGCACTGCAG
CAGATCATTTCATATTGCTTCCGTGGAGTGTG 7 (0.000182%)

TAATATTCATTTAGCCTTCTGAGCTTTCTGGGCAGACTTGGTGACCTTGCCAGCTCCAGCAGCCTTCTTG
TCCACTGCTTTGATGACACCCACCGCAACT 18 (0.000467%)

TAATCACCTGAGCAGTGAAGCCAGCTGCTTCCATTGGTGGGTCATTTTTGCTGTCACCAGCAACGTTGCC
ACGACGAACATCCTTGACAGACACATTCTT 7 (0.000182%)

TAATGGGTCTCAAAATTCTGTGACAAATTTTTGGTCAAGTTGTTTCCATTAAAAAGTACTGATTTTAAAA
ACTAATAACTTAAAACTGCCACACGCAAAA 5 (0.000130%)

TAATTGGGGGGTAGGGGCTAGGCTGGAGTGGTAAAAGGCTCAGAAAAATCCTGCGAAGAAAAAAACTT
CTGAGGTAATAAATAGGATTATCCCGTATCGA 3 (0.000078%)

TACAATGCCAGTCAGGCCACCTACGGTGAAAAGAAAGATGAATCCTAGGGCTCAGAGCACTGCAGCAG
ATCATTTCATATTGCTTCCGTGGAGTGTGGCG 4 (0.000104%)

TACACACTTCCTAATGGCAGAATTTGGCTGTTTGGCTTCAACTCCTACTTTTTCCAGCACGATTCCTTTTG
CATGAGAAGCACCTCCAAAAGGGTTGGCC 9 (0.000234%)

TACTACTAAACTTAAATGGCCAATTGAAACAAACAGTTCTGAGACCGTTCTTCCACCACTGATTAAGAG
TGGGGTGGCAGGTATTAGGGATAATATTCAT 5 (0.000130%)

TAGAATTAGAATTGTGAAGATGATAAGTGTAGAGGGAAGGTTAATGGTTGATATTGCTAGGGTGGCGCT
TCCAATTAGGTGCATGAGTAGGTGGCCTGCA 10 (0.000260%)

TAGCCAATTTTCTTAATGTAAGTGCTGACTTCCTTAACAATTTCCTCATATCTCTTCTGGCTGTAGGGTGG
CTCAGTGGAATCCATTTTGTTAACACCGA 3 (0.000078%)

TAGCCTTCTGAGCTTTCTGGGCAGACTTGGTGACCTTGCCAGCTCCAGCAGCCTTCTTGTCCACTGCTTT
GATGACACCCACCGCAACTGTCTGTCTCAT 34 (0.000882%)

TAGCGATGGAGGTAGGATTGGTGCTGTGGGTGAAAGAGTATGATGGGGTGGTGGTTGTGGTAAACTTTA
ATAGTGTAGGAAGCTGAATAATTTATGAAGG 6 (0.000156%)

TAGGATAGTCAGTAGAATTAGAATTGTGAAGATGATAAGTGTAGAGGGAAGGTTAATGGTTGATATTG
CTAGGGTGGCGCTTCCAATTAGGTGCATGAGT 5 (0.000130%)

TAGGGATAATATTCATTTAGCCTTCTGAGCTTTCTGGGCAGACTTGGTGACCTTGCCAGCTCCAGCAGCC
TTCTTGTCCACTGCTTTGATGACACCCACC26 (0.000675%)

TAGGGTGTAGCCTGAGAATAGGGGAAATCAGTGAATGAAGCCTCCTATGATGGCAAATACAGCTCCTA
TTGATAGGACATAGTGGAAGTGAGCTACAACG 6 (0.000156%)

TATAATCACTGCGCCTGTTCATAAGGGGATGGCCATGGCTAGGTTTATAGATAGTTGGGTGGTTGGTGT
AAATGAGTGAGGCAGGAGTCCGAGGAGGTTA 3 (0.000078%)

TATAGGGCTGTGACTAGTATGTTGAGTCCTGTAAGTAGGAGAGTGATATTTGATCAGGAGAACGTGGTT
ACTAGCACAGAGAGTTCTCCCAGTAGGTTAA 6 (0.000156%)

TATCAACAATGGCAGCATCACCAGACTTCAAGAATTTAGGGCCATCTTCCAGCTTTTACCAGAACGGC
GATCAATCTTTTCCTTCAGCTCAGCAAACTT 15 (0.000389%)

TATTCATTTAGCCTTCTGAGCTTTCTGGGCAGACTTGGTGACCTTGCCAGCTCCAGCAGCCTTCTTGTCC ACTGCTTTGATGACACCCACCGCAACTGTC 57 (0.001479%)

TCAAAATTCTGTGACAAATTTTTGGTCAAGTTGTTTCCATTAAAAAGTACTGATTTTAAAAACTAATAAC TTAAAACTGCCACACGCAAAAAAGAAAACC 18 (0.000467%)

TCAAATTTCCACAAGGAGATATCAATGGTGATACCACGTTCACGCTCAGCTTTCAGTTTATCCAAGACC CAGGCATACTTGAAGGAGCCCTTTCCCATCT 7 (0.000182%)

TCAAATTTTTCAATGGTTCTTTTGTCGATGCCACCGCATTTATAGATCAGATGGCCAGTAGTGGTGGACT TGCCCGAATCTACGTGTCCAATGACGACAA 3 (0.000078%)

TCAACAATGGCAGCATCACCAGACTTCAAGAATTTAGGGCCATCTTCCAGCTTTTTACCAGAACGGCGA TCAATCTTTTCCTTCAGCTCAGCAAACTTGC 8 (0.000208%)

TCAAGAATTTAGGGCCATCTTCCAGCTTTTTACCAGAACGGCGATCAATCTTTTCCTTCAGCTCAGCAAA CTTGCATGCAATGTGAGCCGTGTGGCAATC 4 (0.000104%)

TCAAGTTGTTTCCATTAAAAAGTACTGATTTTAAAAACTAATAACTTAAAACTGCCACACGCAAAAAAG AAAACCAAAGTGGTCCACAAAACATTCTCCT 10 (0.000260%)

TCACCTGAGCAGTGAAGCCAGCTGCTTCCATTGGTGGGTCATTTTTGCTGTCACCAGCAACGTTGCCACG ACGAACATCCTTGACAGACACATTCTTGAC 3 (0.000078%)

TCAGAGGGCGATGAAGTTCTAGATCCATTGAGACAAGCTCTAGACAGTAGCATGCAGTCCCACAACTTG TACCAGCATCCCCAGCGTCTGGCATTCCATG 3 (0.000078%)

TCAGCAAACTTGCATGCAATGTGAGCCGTGTGGCAATCCAATACAGGGGCATAGCCGGCGCTTATTTGG CCTGGATGGTTCAGGATAATCACCTGAGCAG 11 (0.000285%)

TCAGCTCAGCAAACTTGCATGCAATGTGAGCCGTGTGGCAATCCAATACAGGGGCATAGCCGGCGCTTA TTTGGCCTGGATGGTTCAGGATAATCACCTG 6 (0.000156%)

TCAGGATAATCACCTGAGCAGTGAAGCCAGCTGCTTCCATTGGTGGGTCATTTTTGCTGTCACCAGCAA CGTTGCCACGACGAACATCCTTGACAGACAC 10 (0.000260%)

TCATTTAGCCTTCTGAGCTTTCTGGGCAGACTTGGTGACCTTGCCAGCTCCAGCAGCCTTCTTGTCCACT GCTTTGATGACACCCACCGCAACTGTCTGT 8 (0.000208%)

TCCACAAGGAGATATCAATGGTGATACCACGTTCACGCTCAGCTTTCAGTTTATCCAAGACCCAGGCAT ACTTGAAGGAGCCCTTTCCCATCTCAGCAGC 9 (0.000234%)

TCCCACTCCTGATGCTGAACCAATGCACCATCTGTAAAGTTGCAGACAGTCTGAGTTTTTCTGCCATCAG CTGTGGTTTCTTCAAACTTCTCTCCCAGGG171 (0.004438%)

TCCTCATATCTCTTCTGGCTGTAGGGTGGCTCAGTGGAATCCATTTTGTTAACACCGACAATTAGTTGTT TCACACCCAGTGTGTAAGCCAGAAGGGCAT 16 (0.000415%)

TCCTCATCCATGTGACCTTCTCTGGCATTCGGGCATTGGCTGTACCCTTCCGCTTACCTATGCCCATGTGC CTGCCCTTCCGGCGGGCCAAGGTGTTTTT 195 (0.005061%)

TCCTGATGCTGAACCAATGCACCATCTGTAAAGTTGCAGACAGTCTGAGTTTTTCTGCCATCAGCTGTGG TTTCTTCAAACTTCTCTCCCAGGGTACAAG 3 (0.000078%)

TCCTTAACAATTTCCTCATATCTCTTCTGGCTGTAGGGTGGCTCAGTGGAATCCATTTTGTTAACACCGA CAATTAGTTGTTTCACACCCAGTGTGTAAG 9 (0.000234%)

TCCTTCAGCTCAGCAAACTTGCATGCAATGTGAGCCGTGTGGCAATCCAATACAGGGGCATAGCCGGCG

CTTATTTGGCCTGGATGGTTCAGGATAATCA 7 (0.000182%)

TCGATGCCACCGCATTTATAGATCAGATGGCCAGTAGTGGTGGACTTGCCCGAATCTACGTGTCCAATG
ACGACAATGTTGATATGAGTCTTTTCCTTTC 3 (0.000078%)

TCGGACACGAAGGCCCCAGAAGTGACGCAGCCCTCTATGGGCCCGAATCTTCTTCAGTCGCTCCAGGTC
TTCACGGAGCTTGTTGTCCAGACCATTGGCT 9 (0.000234%)

TCTAACAGTTCAAAAGAAGCCACTACATACTCTTTTCACAAATATGTTTTCACAGAGCCAATACAGTAC
TAGCCATTAACCCAGTACACCAAGTGTACTG 3 (0.000078%)

TCTAGGATAGTCAGTAGAATTAGAATTGTGAAGATGATAAGTGTAGAGGGAAGGTTAATGGTTGATATT
GCTAGGGTGGCGCTTCCAATTAGGTGCATGA 4 (0.000104%)

TCTAGGGTGTAGCCTGAGAATAGGGGAAATCAGTGAATGAAGCCTCCTATGATGGCAAATACAGCTCCT
ATTGATAGGACATAGTGGAAGTGAGCTACAA 4 (0.000104%)

TCTCAAATTTCCACAAGGAGATATCAATGGTGATACCACGTTCACGCTCAGCTTTCAGTTTATCCAAGAC
CCAGGCATACTTGAAGGAGCCCTTTCCCAT 4 (0.000104%)

TCTCGTGTTACATCGCGCCATCATTGGTATATGGTTAGTGTGTTGGTTAGTAGGCCTAGTATGAGGAGCG
TTATGGAGTGGAAGTGAAATCACATGGCTA 7 (0.000182%)

TCTGAGCTTTCTGGGCAGACTTGGTGACCTTGCCAGCTCCAGCAGCCTTCTTGTCCACTGCTTTGATGAC
ACCCACCGCAACTGTCTGTCTCATATCACG 14 (0.000363%)

TCTGGCATTCGGGCATTGGCTGTACCCTTCCGCTTACCTATGCCCATGTGCCTGCCCTTCCGGCGGGCCA
AGGTGTTTTTCCGGCATCGAGCCCGGGAAT 8 (0.000208%)

TCTGGGCAGACTTGGTGACCTTGCCAGCTCCAGCAGCCTTCTTGTCCACTGCTTTGATGACACCCACCGC
AACTGTCTGTCTCATATCACGAACAGCAAA 9 (0.000234%)

TCTGTGACAAATTTTTGGTCAAGTTGTTTCCATTAAAAAGTACTGATTTTAAAAACTAATAACTTAAAAC
TGCCACACGCAAAAAAGAAAACCAAAGTGG 14 (0.000363%)

TCTTAATGTAAGTGCTGACTTCCTTAACAATTTCCTCATATCTCTTCTGGCTGTAGGGTGGCTCAGTGGA
ATCCATTTTGTTAACACCGACAATTAGTTG 28 (0.000727%)

TCTTAGGCACAGGTGCGGAGACGATGCCAGTGCCCCTGGGTGCAGGGATGAGGCGTACCAGCACAGAG
CCGCAGCGGCCTGTCACCTTGCAAGGGACAGT5 (0.000130%)

TCTTATCAAGTCAGCACACACCTTTTCCAAGGATTTTACGTTGCGGCTTGTTAGGGTGATTCGAATTCGG
TGAATTGCCACCTCCGGCTCCACGGGTGTT 5 (0.000130%)

TCTTGGTTCCCCGGAGACGTCCAGTCCGGCGGGCAGCAATGAGACCCACTTTGCGGCCAGCAGGGGCAT
CTCTGCGGATGGTGGAGGGCTTGCCGATGTG 3 (0.000078%)

TCTTGTCCACTGCTTTGATGACACCCACCGCAACTGTCTGTCTCATATCACGAACAGCAAAGCGACCCA
AAGGTGGATAGTCTGAGAAGCTCTCAACACA 4 (0.000104%)

TCTTTTACTACTAAACTTAAATGGCCAATTGAAACAAACAGTTCTGAGACCGTTCTTCCACCACTGATTA
AGAGTGGGGTGGCAGGTATTAGGGATAATA 6 (0.000156%)

TCTTTTCCTTCAGCTCAGCAAACTTGCATGCAATGTGAGCCGTGTGGCAATCCAATACAGGGGCATAGC
CGGCGCTTATTTGGCCTGGATGGTTCAGGAT 6 (0.000156%)

TCTTTTGTCGATGCCACCGCATTTATAGATCAGATGGCCAGTAGTGGTGGACTTGCCCGAATCTACGTGT
CCAATGACGACAATGTTGATATGAGTCTTT 3 (0.000078%)

TGAAAAGTGAACAGATATTCAGCATCTAACAGTTCAAAAGAAGCCACTACATACTCTTTTCACAAATAT
GTTTTCACAGAGCCAATACAGTACTAGCCAT 6 (0.000156%)

TGAAAGAGTATGATGGGGTGGTGGTTGTGGTAAACTTTAATAGTGTAGGAAGCTGAATAATTTATGAAG
GAGAGGGGTCAGGGTTGATTCGGGAGGATCC 4 (0.000104%)

TGACAAATTTTTGGTCAAGTTGTTTCCATTAAAAAGTACTGATTTTAAAAACTAATAACTTAAAACTGCC
ACACGCAAAAAAGAAAACCAAAGTGGTCCA 3 (0.000078%)

TGACCTTGCCAGCTCCAGCAGCCTTCTTGTCCACTGCTTTGATGACACCCACCGCAACTGTCTGTCTCAT
ATCACGAACAGCAAAGCGACCCAAAGGTGG 7 (0.000182%)

TGACTTCCTTAACAATTTCCTCATATCTCTTCTGGCTGTAGGGTGGCTCAGTGGAATCCATTTTGTTAAC
ACCGACAATTAGTTGTTTCACACCCAGTGT 22 (0.000571%)

TGAGAGAGTGAGGAGAAGGCTTACGTTTAGTGAGGGAGAGATTTGGTATATGATTGAGATGGGGGCTA
GTTTTTGTCATGTGAGAAGAAGCAGGCCGGAT 19 (0.000493%)

TGAGCAAGGTGGGTCTCAGAGGTGATCGGCGATCAGAGGGCGATGAAGTTCTAGATCCATTGAGACAA
GCTCTAGACAGTAGCATGCAGTCCCACAACTT 6 (0.000156%)

TGAGCAGTGAAGCCAGCTGCTTCCATTGGTGGGTCATTTTTGCTGTCACCAGCAACGTTGCCACGACGA
ACATCCTTGACAGACACATTCTTGACATTGA 7 (0.000182%)

TGAGCCGTGTGGCAATCCAATACAGGGGCATAGCCGGCGCTTATTTGGCCTGGATGGTTCAGGATAATC
ACCTGAGCAGTGAAGCCAGCTGCTTCCATTG 6 (0.000156%)

TGAGCTTTCTGGGCAGACTTGGTGACCTTGCCAGCTCCAGCAGCCTTCTTGTCCACTGCTTTGATGACAC
CCACCGCAACTGTCTGTCTCATATCACGAA 27 (0.000701%)

TGAGTGAGCCCCATTGTGTTGTGGTAAATATGTAGAGGGAGTATAGGGCTGTGACTAGTATGTTGAGTC
CTGTAAGTAGGAGAGTGATATTTGATCAGGA 15 (0.000389%)

TGCAATGTGAGCCGTGTGGCAATCCAATACAGGGGCATAGCCGGCGCTTATTTGGCCTGGATGGTTCAG
GATAATCACCTGAGCAGTGAAGCCAGCTGCT 18 (0.000467%)

TGCACAGTCTTGGTTCCCCGGAGACGTCCAGTCCGGCGGGCAGCAATGAGACCCACTTTGCGGCCAGCA
GGGGCATCTCTGCGGATGGTGGAGGGCTTGC 4 (0.000104%)

TGCAGGGATGAGGCGTACCAGCACAGAGCCGCAGCGGCCTGTCACCTTGCAAGGGACAGTGTGGGGCT
TGCCGATCTTGTTCCCCCAGTAGCCTCTGCGC 6 (0.000156%)

TGCATGCAATGTGAGCCGTGTGGCAATCCAATACAGGGGCATAGCCGGCGCTTATTTGGCCTGGATGGT
TCAGGATAATCACCTGAGCAGTGAAGCCAGC 7 (0.000182%)

TGCCAGCTCCAGCAGCCTTCTTGTCCACTGCTTTGATGACACCCACCGCAACTGTCTGTCTCATATCACG
AACAGCAAAGCGACCCAAAGGTGGATAGTC 19 (0.000493%)

TGCCAGTCAGGCCACCTACGGTGAAAAGAAAGATGAATCCTAGGGCTCAGAGCACTGCAGCAGATCAT
TTCATATTGCTTCCGTGGAGTGTGGCGAGTCA 9 (0.000234%)

TGCCAGTGCCCCTGGGTGCAGGGATGAGGCGTACCAGCACAGAGCCGCAGCGGCCTGTCACCTTGCAA
GGGACAGTGTGGGGCTTGCCGATCTTGTTCCC 13 (0.000337%)

TGCCCCTGGGTGCAGGGATGAGGCGTACCAGCACAGAGCCGCAGCGGCCTGTCACCTTGCAAGGGACA
GTGTGGGGCTTGCCGATCTTGTTCCCCCAGTA 6 (0.000156%)

TGCCCTCCTAATTGGGGGGTAGGGGCTAGGCTGGAGTGGTAAAAGGCTCAGAAAAATCCTGCGAAGAA
AAAAACTTCTGAGGTAATAAATAGGATTATCC 6 (0.000156%)

TGCGGAGACGATGCCAGTGCCCCTGGGTGCAGGGATGAGGCGTACCAGCACAGAGCCGCAGCGGCCTG
TCACCTTGCAAGGGACAGTGTGGGGCTTGCCG 41 (0.001064%)

TGCGGGTGGCACTGCCCACGGTGGGCGGGCGGGCCTCTCTACTCGAAGGTGACCACGTTTAGATTCTGA
GACGGGAAGTGGAGGGTGAATAGGTCACGGC 6 (0.000156%)

TGCTAAGGGAGGGTAGACTGTTCAACCTGTTCCTGCTCCGGCCTCCACTATAGCAGATGCGAGCAGGAG
TAGGAGAGAGGGAGGTAAGAGTCAGAAGCTT 8 (0.000208%)

TGCTAATACAATGCCAGTCAGGCCACCTACGGTGAAAAGAAAGATGAATCCTAGGGCTCAGAGCACTG
CAGCAGATCATTTCATATTGCTTCCGTGGAGT 578 (0.015000%)

TGCTGACTTCCTTAACAATTTCCTCATATCTCTTCTGGCTGTAGGGTGGCTCAGTGGAATCCATTTTGTTA
ACACCGACAATTAGTTGTTTCACACCCAG 24 (0.000623%)

TGCTGGTCTCAAATTTCCACAAGGAGATATCAATGGTGATACCACGTTCACGCTCAGCTTTCAGTTTATC
CAAGACCCAGGCATACTTGAAGGAGCCCTT 16 (0.000415%)

TGGAGGTAGGATTGGTGCTGTGGGTGAAAGAGTATGATGGGGTGGTGGTTGTGGTAAACTTTAATAGTG
TAGGAAGCTGAATAATTTATGAAGGAGAGGG 3 (0.000078%)

TGGATTTTGGCGTAGGTTTGGTCTAGGGTGTAGCCTGAGAATAGGGGAAATCAGTGAATGAAGCCTCCT
ATGATGGCAAATACAGCTCCTATTGATAGGA 369 (0.009576%)

TGGCAATCCAATACAGGGGCATAGCCGGCGCTTATTTGGCCTGGATGGTTCAGGATAATCACCTGAGCA
GTGAAGCCAGCTGCTTCCATTGGTGGGTCAT 211 (0.005476%)

TGGCACTGCCCACGGTGGGCGGGCGGGCCTCTCTACTCGAAGGTGACCACGTTTAGATTCTGAGACGGG
AAGTGGAGGGTGAATAGGTCACGGCGGCCTT 12 (0.000311%)

TGGCAGAATTTGGCTGTTTGGCTTCAACTCCTACTTTTTCCAGCACGATTCCTTTTGCATGAGAAGCACC
TCCAAAAGGGTTGGCCTTTAGGGCTGTGCC 4 (0.000104%)

TGGCAGCATCACCAGACTTCAAGAATTTAGGGCCATCTTCCAGCTTTTTACCAGAACGGCGATCAATCT
TTTCCTTCAGCTCAGCAAACTTGCATGCAAT 8 (0.000208%)

TGGCATTCGGGCATTGGCTGTACCCTTCCGCTTACCTATGCCCATGTGCCTGCCCTTCCGGCGGGCCAAG
GTGTTTTTCCGGCATCGAGCCCGGGAATGG 13 (0.000337%)

TGGCCTCGGACACGAAGGCCCCAGAAGTGACGCAGCCCTCTATGGGCCCGAATCTTCTTCAGTCGCTCC
AGGTCTTCACGGAGCTTGTTGTCCAGACCAT 331 (0.008590%)

TGGCCTTGGTATGTGCTTTCTCGTGTTACATCGCGCCATCATTGGTATATGGTTAGTGTGTTGGTTAGTA
GGCCTAGTATGAGGAGCGTTATGGAGTGGA 4 (0.000104%)

TGGCGTAGGTTTGGTCTAGGGTGTAGCCTGAGAATAGGGGAAATCAGTGAATGAAGCCTCCTATGATGG
CAAATACAGCTCCTATTGATAGGACATAGTG 10 (0.000260%)

TGGGAGTAGTTCCCTGCTAAGGGAGGGTAGACTGTTCAACCTGTTCCTGCTCCGGCCTCCACTATAGCA
GATGCGAGCAGGAGTAGGAGAGAGGGAGGTA 7 (0.000182%)

TGGGATTGAAAAGTGAACAGATATTCAGCATCTAACAGTTCAAAAGAAGCCACTACATACTCTTTTCAC
AAATATGTTTTCACAGAGCCAATACAGTACT 4 (0.000104%)

TGGGCAGACTTGGTGACCTTGCCAGCTCCAGCAGCCTTCTTGTCCACTGCTTTGATGACACCCACCGCAA

CTGTCTGTCTCATATCACGAACAGCAAAGC 6 (0.000156%)

TGGGGACAGCTCATGAGTGCAAGACGTCTTGTGATGTAATTATTATACGAATGGGGGCTTCAATCGGGA
GTACTACTCGATTGTCAACGTCAAGGAGTCG 5 (0.000130%)

TGGGGGCCAGTGCCCTCCTAATTGGGGGGTAGGGGCTAGGCTGGAGTGGTAAAAGGCTCAGAAAAATC
CTGCGAAGAAAAAAACTTCTGAGGTAATAAAT 6 (0.000156%)

TGGGTCTCAAAATTCTGTGACAAATTTTTGGTCAAGTTGTTTCCATTAAAAAGTACTGATTTTAAAAACT
AATAACTTAAAACTGCCACACGCAAAAAAG 113 (0.002932%)

TGGGTGAGTGAGCCCCATTGTGTTGTGGTAAATATGTAGAGGGAGTATAGGGCTGTGACTAGTATGTTG
AGTCCTGTAAGTAGGAGAGTGATATTTGATC 9 (0.000234%)

TGGTAAATATGTAGAGGGAGTATAGGGCTGTGACTAGTATGTTGAGTCCTGTAAGTAGGAGAGTGATAT
TTGATCAGGAGAACGTGGTTACTAGCACAGA 3 (0.000078%)

TGGTCAAGTTGTTTCCATTAAAAAGTACTGATTTTAAAAACTAATAACTTAAAACTGCCACACGCAAAA
AAGAAAACCAAAGTGGTCCACAAAACATTCT 6 (0.000156%)

TGGTGACCTTGCCAGCTCCAGCAGCCTTCTTGTCCACTGCTTTGATGACACCCACCGCAACTGTCTGTCT
CATATCACGAACAGCAAAGCGACCCAAAGG 16 (0.000415%)

TGGTGGCCTTGGTATGTGCTTTCTCGTGTTACATCGCGCCATCATTGGTATATGGTTAGTGTGTTGGTTA
GTAGGCCTAGTATGAGGAGCGTTATGGAGT 13 (0.000337%)

TGGTGGGTGAGTGAGCCCCATTGTGTTGTGGTAAATATGTAGAGGGAGTATAGGGCTGTGACTAGTATG
TTGAGTCCTGTAAGTAGGAGAGTGATATTTG 371 (0.009628%)

TGTAAGTGCTGACTTCCTTAACAATTTCCTCATATCTCTTCTGGCTGTAGGGTGGCTCAGTGGAATCCAT
TTTGTTAACACCGACAATTAGTTGTTTCAC6 (0.000156%)

TGTAGAGGGAGTATAGGGCTGTGACTAGTATGTTGAGTCCTGTAAGTAGGAGAGTGATATTTGATCAGG
AGAACGTGGTTACTAGCACAGAGAGTTCTCC 18 (0.000467%)

TGTAGCCAATTTTCTTAATGTAAGTGCTGACTTCCTTAACAATTTCCTCATATCTCTTCTGGCTGTAGGGT
GGCTCAGTGGAATCCATTTTGTTAACACC 21 (0.000545%)

TGTAGCCTGAGAATAGGGGAAATCAGTGAATGAAGCCTCCTATGATGGCAAATACAGCTCCTATTGATA
GGACATAGTGGAAGTGAGCTACAACGTAGTA 17 (0.000441%)

TGTCACAGGTCCAGGGCAGAGGACCAACATGGGCATTTTGTTTATGAGCAAGGTGGGTCTCAGAGGTG
ATCGGCGATCAGAGGGCGATGAAGTTCTAGAT 4 (0.000104%)

TGTCGATGCCACCGCATTTATAGATCAGATGGCCAGTAGTGGTGGACTTGCCCGAATCTACGTGTCCAA
TGACGACAATGTTGATATGAGTCTTTTCCTT 3 (0.000078%)

TGTGAGCCGTGTGGCAATCCAATACAGGGGCATAGCCGGCGCTTATTTGGCCTGGATGGTTCAGGATAA
TCACCTGAGCAGTGAAGCCAGCTGCTTCCAT 9 (0.000234%)

TGTGCTTTCTCGTGTTACATCGCGCCATCATTGGTATATGGTTAGTGTGTTGGTTAGTAGGCCTAGTATG
AGGAGCGTTATGGAGTGGAAGTGAAATCAC 5 (0.000130%)

TGTGGCAATCCAATACAGGGGCATAGCCGGCGCTTATTTGGCCTGGATGGTTCAGGATAATCACCTGAG
CAGTGAAGCCAGCTGCTTCCATTGGTGGGTC 23 (0.000597%)

TGTGGGGACAGCTCATGAGTGCAAGACGTCTTGTGATGTAATTATTATACGAATGGGGGCTTCAATCGG
GAGTACTACTCGATTGTCAACGTCAAGGAGT 8 (0.000208%)

TGTGTTGTGGTAAATATGTAGAGGGAGTATAGGGCTGTGACTAGTATGTTGAGTCCTGTAAGTAGGAGA
GTGATATTTGATCAGGAGAACGTGGTTACTA 4 (0.000104%)

TGTTCATAAGGGGATGGCCATGGCTAGGTTTATAGATAGTTGGGTGGTTGGTGTAAATGAGTGAGGCAG
GAGTCCGAGGAGGTTAGTTGTGGCAATAAAA 3 (0.000078%)

TGTTTATGAGCAAGGTGGGTCTCAGAGGTGATCGGCGATCAGAGGGCGATGAAGTTCTAGATCCATTGA
GACAAGCTCTAGACAGTAGCATGCAGTCCCA 7 (0.000182%)

TGTTTCCATTAAAAAGTACTGATTTTAAAAACTAATAACTTAAAACTGCCACACGCAAAAAAGAAAACC
AAAGTGGTCCACAAAACATTCTCCTTTCCTT 13 (0.000337%)

TGTTTTTAAGCCTAATGTGGGGACAGCTCATGAGTGCAAGACGTCTTGTGATGTAATTATTATACGAAT
GGGGGCTTCAATCGGGAGTACTACTCGATTG 15 (0.000389%)

TTAACAATTTCCTCATATCTCTTCTGGCTGTAGGGTGGCTCAGTGGAATCCATTTTGTTAACACCGACAA
TTAGTTGTTTCACACCCAGTGTGTAAGCCA 11 (0.000285%)

TTAATGGGTCTCAAAATTCTGTGACAAATTTTTGGTCAAGTTGTTTCCATTAAAAAGTACTGATTTTAAA
AACTAATAACTTAAAACTGCCACACGCAAA 9 (0.000234%)

TTAATGTAAGTGCTGACTTCCTTAACAATTTCCTCATATCTCTTCTGGCTGTAGGGTGGCTCAGTGGAAT
CCATTTTGTTAACACCGACAATTAGTTGTT6 (0.000156%)

TTACACACTTCCTAATGGCAGAATTTGGCTGTTTGGCTTCAACTCCTACTTTTTCCAGCACGATTCCTTTT
GCATGAGAAGCACCTCCAAAAGGGTTGGC 30 (0.000779%)

TTACTACTAAACTTAAATGGCCAATTGAAACAAACAGTTCTGAGACCGTTCTTCCACCACTGATTAAGA
GTGGGGTGGCAGGTATTAGGGATAATATTCA 6 (0.000156%)

TTAGCCTTCTGAGCTTTCTGGGCAGACTTGGTGACCTTGCCAGCTCCAGCAGCCTTCTTGTCCACTGCTT
TGATGACACCCACCGCAACTGTCTGTCTCA 73 (0.001894%)

TTAGGCACAGGTGCGGAGACGATGCCAGTGCCCCTGGGTGCAGGGATGAGGCGTACCAGCACAGAGCC
GCAGCGGCCTGTCACCTTGCAAGGGACAGTGT4 (0.000104%)

TTAGGGATAATATTCATTTAGCCTTCTGAGCTTTCTGGGCAGACTTGGTGACCTTGCCAGCTCCAGCAGC
CTTCTTGTCCACTGCTTTGATGACACCCAC9 (0.000234%)

TTAGGGCCATCTTCCAGCTTTTTACCAGAACGGCGATCAATCTTTTCCTTCAGCTCAGCAAACTTGCATG
CAATGTGAGCCGTGTGGCAATCCAATACAG 5 (0.000130%)

TTATCAAGTCAGCACACACCTTTTCCAAGGATTTTACGTTGCGGCTTGTTAGGGTGATTCGAATTCGGTG
AATTGCCACCTCCGGCTCCACGGGTGTTTT 16 (0.000415%)

TTATGAGCAAGGTGGGTCTCAGAGGTGATCGGCGATCAGAGGGCGATGAAGTTCTAGATCCATTGAGA
CAAGCTCTAGACAGTAGCATGCAGTCCCACAA 13 (0.000337%)

TTCAAAGATTTTTAGGGGAATTAATTCTAGGACGATGGGCATGAAACTGTGGTTTGCTCCACAGATTTC
AGAGCATTGACCGTAGTATACCCCCGGTCGT 14 (0.000363%)

TTCAAGAATTTAGGGCCATCTTCCAGCTTTTTACCAGAACGGCGATCAATCTTTTCCTTCAGCTCAGCAA
ACTTGCATGCAATGTGAGCCGTGTGGCAAT 3 (0.000078%)

TTCAGCTCAGCAAACTTGCATGCAATGTGAGCCGTGTGGCAATCCAATACAGGGGCATAGCCGGCGCTT
ATTTGGCCTGGATGGTTCAGGATAATCACCT 18 (0.000467%)

TTCAGGATAATCACCTGAGCAGTGAAGCCAGCTGCTTCCATTGGTGGGTCATTTTGCTGTCACCAGCA
ACGTTGCCACGACGAACATCCTTGACAGACA 5 (0.000130%)

TTCATTTAGCCTTCTGAGCTTTCTGGGCAGACTTGGTGACCTTGCCAGCTCCAGCAGCCTTCTTGTCCAC
TGCTTTGATGACACCCACCGCAACTGTCTG 26 (0.000675%)

TTCCACAAGGAGATATCAATGGTGATACCACGTTCACGCTCAGCTTTCAGTTTATCCAAGACCCAGGCA
TACTTGAAGGAGCCCTTTCCCATCTCAGCAG 8 (0.000208%)

TTCCATTAAAAAGTACTGATTTTAAAAACTAATAACTTAAAACTGCCACACGCAAAAAAGAAAACCAA
AGTGGTCCACAAAACATTCTCCTTTCCTTCTG 64 (0.001661%)

TTCCTAATGGCAGAATTTGGCTGTTTGGCTTCAACTCCTACTTTTTCCAGCACGATTCCTTTTGCATGAGA
AGCACCTCCAAAAGGGTTGGCCTTTAGGG 6 (0.000156%)

TTCCTCATATCTCTTCTGGCTGTAGGGTGGCTCAGTGGAATCCATTTTGTTAACACCGACAATTAGTTGT
TTCACACCCAGTGTGTAAGCCAGAAGGGCA 16 (0.000415%)

TTCCTTAACAATTTCCTCATATCTCTTCTGGCTGTAGGGTGGCTCAGTGGAATCCATTTTGTTAACACCG
ACAATTAGTTGTTTCACACCCAGTGTGTAA 28 (0.000727%)

TTCCTTCAGCTCAGCAAACTTGCATGCAATGTGAGCCGTGTGGCAATCCAATACAGGGGCATAGCCGGC
GCTTATTTGGCCTGGATGGTTCAGGATAATC 7 (0.000182%)

TTCTAGGATAGTCAGTAGAATTAGAATTGTGAAGATGATAAGTGTAGAGGGAAGGTTAATGGTTGATAT
TGCTAGGGTGGCGCTTCCAATTAGGTGCATG 5 (0.000130%)

TTCTCAAATTTTTCAATGGTTCTTTTGTCGATGCCACCGCATTTATAGATCAGATGGCCAGTAGTGGTGG
ACTTGCCCGAATCTACGTGTCCAATGACGA 6 (0.000156%)

TTCTCGTGTTACATCGCGCCATCATTGGTATATGGTTAGTGTGTTGGTTAGTAGGCCTAGTATGAGGAGC
GTTATGGAGTGGAAGTGAAATCACATGGCT 7 (0.000182%)

TTCTGAGCTTTCTGGGCAGACTTGGTGACCTTGCCAGCTCCAGCAGCCTTCTTGTCCACTGCTTTGATGA
CACCCACCGCAACTGTCTGTCTCATATCAC 14 (0.000363%)

TTCTGGGCAGACTTGGTGACCTTGCCAGCTCCAGCAGCCTTCTTGTCCACTGCTTTGATGACACCCACCG
CAACTGTCTGTCTCATATCACGAACAGCAA 6 (0.000156%)

TTCTGTGACAAATTTTTGGTCAAGTTGTTTCCATTAAAAAGTACTGATTTTAAAAACTAATAACTTAAAA
CTGCCACACGCAAAAAAGAAAACCAAAGTG 17 (0.000441%)

TTCTTAATGTAAGTGCTGACTTCCTTAACAATTTCCTCATATCTCTTCTGGCTGTAGGGTGGCTCAGTGG
AATCCATTTTGTTAACACCGACAATTAGTT 5 (0.000130%)

TTCTTGTCCACTGCTTTGATGACACCCACCGCAACTGTCTGTCTCATATCACGAACAGCAAAGCGACCCA
AAGGTGGATAGTCTGAGAAGCTCTCAACAC 13 (0.000337%)

TTGAAAAGTGAACAGATATTCAGCATCTAACAGTTCAAAAGAAGCCACTACATACTCTTTTCACAAATA
TGTTTTCACAGAGCCAATACAGTACTAGCCA 4 (0.000104%)

TTGAGAGAGTGAGGAGAAGGCTTACGTTTAGTGAGGGAGAGATTTGGTATATGATTGAGATGGGGGCT
AGTTTTTGTCATGTGAGAAGAAGCAGGCCGGA6 (0.000156%)

TTGCATAATCCAGGGAATCATAAATCATGCCAAAGCCAGTTGTCTTGCCACCACCAAAATGAGTTCTGA
ATCCAAATACAAAGATGACATCCGGTGTGGT 4 (0.000104%)

TTGCTGGTCTCAAATTTCCACAAGGAGATATCAATGGTGATACCACGTTCACGCTCAGCTTTCAGTTTAT

CCAAGACCCAGGCATACTTGAAGGAGCCCT 6 (0.000156%)

TTGGCGTAGGTTTGGTCTAGGGTGTAGCCTGAGAATAGGGGAAATCAGTGAATGAAGCCTCCTATGATG
GCAAATACAGCTCCTATTGATAGGACATAGT 9 (0.000234%)

TTGGGATTGAAAAGTGAACAGATATTCAGCATCTAACAGTTCAAAAGAAGCCACTACATACTCTTTTCA
CAAATATGTTTTCACAGAGCCAATACAGTAC 106 (0.002751%)

TTGGGGGCCAGTGCCCTCCTAATTGGGGGGTAGGGGCTAGGCTGGAGTGGTAAAAGGCTCAGAAAAAT
CCTGCGAAGAAAAAAACTTCTGAGGTAATAAA 13 (0.000337%)

TTGGTATGTGCTTTCTCGTGTTACATCGCGCCATCATTGGTATATGGTTAGTGTGTTGGTTAGTAGGCCT
AGTATGAGGAGCGTTATGGAGTGGAAGTGA 6 (0.000156%)

TTGGTCAAGTTGTTTCCATTAAAAAGTACTGATTTTAAAAACTAATAACTTAAAACTGCCACACGCAAA
AAAGAAAACCAAAGTGGTCCACAAAACATTC 4 (0.000104%)

TTGGTCTAGGGTGTAGCCTGAGAATAGGGGAAATCAGTGAATGAAGCCTCCTATGATGGCAAATACAG
CTCCTATTGATAGGACATAGTGGAAGTGAGCT 10 (0.000260%)

TTGGTGACCTTGCCAGCTCCAGCAGCCTTCTTGTCCACTGCTTTGATGACACCCACCGCAACTGTCTGTC
TCATATCACGAACAGCAAAGCGACCCAAAG 7 (0.000182%)

TTGGTTCCCCGGAGACGTCCAGTCCGGCGGGCAGCAATGAGACCCACTTTGCGGCCAGCAGGGGCATCT
CTGCGGATGGTGGAGGGCTTGCCGATGTGCT 3 (0.000078%)

TTGTAGCCAATTTTCTTAATGTAAGTGCTGACTTCCTTAACAATTTCCTCATATCTCTTCTGGCTGTAGGG
TGGCTCAGTGGAATCCATTTTGTTAACAC 10 (0.000260%)

TTGTTTATGAGCAAGGTGGGTCTCAGAGGTGATCGGCGATCAGAGGGCGATGAAGTTCTAGATCCATTG
AGACAAGCTCTAGACAGTAGCATGCAGTCCC 10 (0.000260%)

TTGTTTCCATTAAAAAGTACTGATTTTAAAAACTAATAACTTAAAACTGCCACACGCAAAAAAGAAAAC
CAAAGTGGTCCACAAAACATTCTCCTTTCCT 3 (0.000078%)

TTTAAGCCTAATGTGGGGACAGCTCATGAGTGCAAGACGTCTTGTGATGTAATTATTATACGAATGGGG
GCTTCAATCGGGAGTACTACTCGATTGTCAA 6 (0.000156%)

TTTAATCTTAGAGCGAAAGCCTATAATCACTGCGCCTGTTCATAAGGGGATGGCCATGGCTAGGTTTAT
AGATAGTTGGGTGGTTGGTGTAAATGAGTGA 8 (0.000208%)

TTTACTACTAAACTTAAATGGCCAATTGAAACAAACAGTTCTGAGACCGTTCTTCCACCACTGATTAAG
AGTGGGGTGGCAGGTATTAGGGATAATATTC 18 (0.000467%)

TTTAGCCTTCTGAGCTTTCTGGGCAGACTTGGTGACCTTGCCAGCTCCAGCAGCCTTCTTGTCCACTGCT
TTGATGACACCCACCGCAACTGTCTGTCTC 29 (0.000753%)

TTTAGGGCCATCTTCCAGCTTTTTACCAGAACGGCGATCAATCTTTTCCTTCAGCTCAGCAAACTTGCAT
GCAATGTGAGCCGTGTGGCAATCCAATACA 21 (0.000545%)

TTTATGAGCAAGGTGGGTCTCAGAGGTGATCGGCGATCAGAGGGCGATGAAGTTCTAGATCCATTGAG
ACAAGCTCTAGACAGTAGCATGCAGTCCCACA 12 (0.000311%)

TTTCAAAGATTTTTAGGGGAATTAATTCTAGGACGATGGGCATGAAACTGTGGTTTGCTCCACAGATTTC
AGAGCATTGACCGTAGTATACCCCCGGTCG 12 (0.000311%)

TTTCAATGGTTCTTTTGTCGATGCCACCGCATTTATAGATCAGATGGCCAGTAGTGGTGGACTTGCCCGA
ATCTACGTGTCCAATGACGACAATGTTGAT 3 (0.000078%)

TTTCCATTAAAAAGTACTGATTTTAAAAACTAATAACTTAAAACTGCCACACGCAAAAAAGAAAACCAA
AGTGGTCCACAAAACATTCTCCTTTCCTTCT 53 (0.001375%)

TTTCCTCATATCTCTTCTGGCTGTAGGGTGGCTCAGTGGAATCCATTTTGTTAACACCGACAATTAGTTG
TTTCACACCCAGTGTGTAAGCCAGAAGGGC 17 (0.000441%)

TTTCCTTCAGCTCAGCAAACTTGCATGCAATGTGAGCCGTGTGGCAATCCAATACAGGGGCATAGCCGG
CGCTTATTTGGCCTGGATGGTTCAGGATAAT 4 (0.000104%)

TTTCTCGTGTTACATCGCGCCATCATTGGTATATGGTTAGTGTGTTGGTTAGTAGGCCTAGTATGAGGAG
CGTTATGGAGTGGAAGTGAAATCACATGGC 5 (0.000130%)

TTTCTGGGCAGACTTGGTGACCTTGCCAGCTCCAGCAGCCTTCTTGTCCACTGCTTTGATGACACCCACC
GCAACTGTCTGTCTCATATCACGAACAGCA 7 (0.000182%)

TTTCTTAATGTAAGTGCTGACTTCCTTAACAATTTCCTCATATCTCTTCTGGCTGTAGGGTGGCTCAGTGG
AATCCATTTTGTTAACACCGACAATTAGT 7 (0.000182%)

TTTCTTTGCATAATCCAGGGAATCATAAATCATGCCAAAGCCAGTTGTCTTGCCACCACCAAAATGAGT
TCTGAATCCAAATACAAAGATGACATCCGGT 6 (0.000156%)

TTTGGCGTAGGTTTGGTCTAGGGTGTAGCCTGAGAATAGGGGAAATCAGTGAATGAAGCCTCCTATGAT
GGCAAATACAGCTCCTATTGATAGGACATAG 8 (0.000208%)

TTTGGCTGTTTGGCTTCAACTCCTACTTTTTCCAGCACGATTCCTTTTGCATGAGAAGCACCTCCAAAAG
GGTTGGCCTTTAGGGCTGTGCCCAAATGAG 6 (0.000156%)

TTTGGTCTAGGGTGTAGCCTGAGAATAGGGGAAATCAGTGAATGAAGCCTCCTATGATGGCAAATACA
GCTCCTATTGATAGGACATAGTGGAAGTGAGC 10 (0.000260%)

TTTGTCGATGCCACCGCATTTATAGATCAGATGGCCAGTAGTGGTGGACTTGCCCGAATCTACGTGTCC
AATGACGACAATGTTGATATGAGTCTTTTCC 16 (0.000415%)

TTTGTTTATGAGCAAGGTGGGTCTCAGAGGTGATCGGCGATCAGAGGGCGATGAAGTTCTAGATCCATT
GAGACAAGCTCTAGACAGTAGCATGCAGTCC 95 (0.002465%)

TTTTAAGCCTAATGTGGGGACAGCTCATGAGTGCAAGACGTCTTGTGATGTAATTATTATACGAATGGG
GGCTTCAATCGGGAGTACTACTCGATTGTCA 25 (0.000649%)

TTTTAATCTTAGAGCGAAAGCCTATAATCACTGCGCCTGTTCATAAGGGGATGGCCATGGCTAGGTTTA
TAGATAGTTGGGTGGTTGGTGTAAATGAGTG 28 (0.000727%)

TTTTAATGGGTCTCAAAATTCTGTGACAAATTTTTGGTCAAGTTGTTTCCATTAAAAAGTACTGATTTTA
AAAACTAATAACTTAAAACTGCCACACGCA 27 (0.000701%)

TTTTACCAGAACGGCGATCAATCTTTTCCTTCAGCTCAGCAAACTTGCATGCAATGTGAGCCGTGTGGCA
ATCCAATACAGGGGCATAGCCGGCGCTTAT 3 (0.000078%)

TTTTACTACTAAACTTAAATGGCCAATTGAAACAAACAGTTCTGAGACCGTTCTTCCACCACTGATTAAG
AGTGGGGTGGCAGGTATTAGGGATAATATT 44 (0.001142%)

TTTTCAATGGTTCTTTTGTCGATGCCACCGCATTTATAGATCAGATGGCCAGTAGTGGTGGACTTGCCCG
AATCTACGTGTCCAATGACGACAATGTTGA 8 (0.000208%)

TTTTCCTCCTGTAGGCTGGCAGAGGACAGTGGAGCAGCCAACACACAAAACTACCGTTTGTGCATGGCT
AAAGACCGTGGTGATTTTATAGCATCCTGGG 468 (0.012145%)

TTTTCCTTCAGCTCAGCAAACTTGCATGCAATGTGAGCCGTGTGGCAATCCAATACAGGGGCATAGCCG
GCGCTTATTTGGCCTGGATGGTTCAGGATAA 45 (0.001168%)

TTTTCTTAATGTAAGTGCTGACTTCCTTAACAATTTCCTCATATCTCTTCTGGCTGTAGGGTGGCTCAGTG
GAATCCATTTTGTTAACACCGACAATTAG 27 (0.000701%)

TTTTCTTTGCATAATCCAGGGAATCATAAATCATGCCAAAGCCAGTTGTCTTGCCACCACCAAAATGAG
TTCTGAATCCAAATACAAAGATGACATCCGG 11 (0.000285%)

TTTTGGCGTAGGTTTGGTCTAGGGTGTAGCCTGAGAATAGGGGAAATCAGTGAATGAAGCCTCCTATGA
TGGCAAATACAGCTCCTATTGATAGGACATA 14 (0.000363%)

TTTTGGTCAAGTTGTTTCCATTAAAAAGTACTGATTTTAAAAACTAATAACTTAAAACTGCCACACGCAA
AAAAGAAAACCAAAGTGGTCCACAAAACAT 11 (0.000285%)

TTTTGTCGATGCCACCGCATTTATAGATCAGATGGCCAGTAGTGGTGGACTTGCCCGAATCTACGTGTCC
AATGACGACAATGTTGATATGAGTCTTTTC 10 (0.000260%)

TTTTGTTTATGAGCAAGGTGGGTCTCAGAGGTGATCGGCGATCAGAGGGCGATGAAGTTCTAGATCCAT
TGAGACAAGCTCTAGACAGTAGCATGCAGTC 32 (0.000830%)

TTTTTAAGCCTAATGTGGGGACAGCTCATGAGTGCAAGACGTCTTGTGATGTAATTATTATACGAATGG
GGGCTTCAATCGGGAGTACTACTCGATTGTC 7 (0.000182%)

TTTTTAATGGGTCTCAAAATTCTGTGACAAATTTTTGGTCAAGTTGTTTCCATTAAAAAGTACTGATTTT
AAAAACTAATAACTTAAAACTGCCACACGC 7 (0.000182%)

TTTTTACCAGAACGGCGATCAATCTTTTCCTTCAGCTCAGCAAACTTGCATGCAATGTGAGCCGTGTGGC
AATCCAATACAGGGGCATAGCCGGCGCTTA 6 (0.000156%)

TTTTTCTTTGCATAATCCAGGGAATCATAAATCATGCCAAAGCCAGTTGTCTTGCCACCACCAAAATGA
GTTCTGAATCCAAATACAAAGATGACATCCG 17 (0.000441%)

TTTTTGGTCAAGTTGTTTCCATTAAAAAGTACTGATTTTAAAAACTAATAACTTAAAACTGCCACACGCA
AAAAAGAAAACCAAAGTGGTCCACAAAACA 9 (0.000234%)

TTTTTTAATGGGTCTCAAAATTCTGTGACAAATTTTTGGTCAAGTTGTTTCCATTAAAAAGTACTGATTTT
AAAAACTAATAACTTAAAACTGCCACACG 25 (0.000649%)

TTTTTTTTTTTTTTTTTTTTTTTTTTTTTTTTTTTTTTTTTTTTTTTTTTTTTTTTTTTTTTTTTTTTTTTTT
TTTTTTTTTTTTTTTTTTTTTTT 991 (0.025718%)

TTTTTTTTTTTTTTTTTTTTTTTTTTTTTTTTTTTTTTTTTTTTTTTTTTTTTTTTTTTTTTTTTTTTTTTTT
TTTTTTTTTTTTTTTTTTTTTTTTTTTTTTTTTTTTTTTTTTTTTTTTTTTTTTTTTTTTTTTTTTTT 427
(0.016511%)

After filtering: read2: quality
Value of each position will be shown on mouse over.
204060801001201403838.53939.540

positionqualityATCGmean

<https://plot.ly/>
After filtering: read2: base contents
Value of each position will be shown on mouse over.
2040608010012014000.10.20.30.40.50.6

positionbase content ratiosA(26.57%)T(25.09%)C(23.77%)G(24.50%)N(0.047%)GC(48.28%)

<https://plot.ly/>
After filtering: read2: KMER counting
Darker background means larger counts. The count will be shown on mouse over.

AA AT AC AG TA TT TC TG CA CT CC CG GA GT GC GG
AAA AAAAA AAAAT AAAAC AAAAG AAATA AAATT AAATC AAATG AAACA AAACT AAACC
AAACG AAAGA AAAGT AAAGC AAAGG
AAT AATAA AATAT AATAC AATAG AATTA AATTT AATTC AATTG AATCA AATCT AATCC
AATCG AATGA AATGT AATGC AATGG
AAC AACAA AACAT AACAC AACAG AACTA AACTT AACTC AACTG AACCA AACCT AACCC
AACCG AACGA AACGT AACGC AACGG
AAG AAGAA AAGAT AAGAC AAGAG AAGTA AAGTT AAGTC AAGTG AAGCA AAGCT AAGCC
AAGCG AAGGA AAGGT AAGGC AAGGG
ATA ATAAA ATAAT ATAAC ATAAG ATATA ATATT ATATC ATATG ATACA ATACT ATACC
ATACG ATAGA ATAGT ATAGC ATAGG
ATT ATTAA ATTAT ATTAC ATTAG ATTTA ATTTT ATTTC ATTTG ATTCA ATTCT ATTCC
ATTCG ATTGA ATTGT ATTGC ATTGG
ATC ATCAA ATCAT ATCAC ATCAG ATCTA ATCTT ATCTC ATCTG ATCCA ATCCT ATCCC
ATCCG ATCGA ATCGT ATCGC ATCGG
ATG ATGAA ATGAT ATGAC ATGAG ATGTA ATGTT ATGTC ATGTG ATGCA ATGCT ATGCC
ATGCG ATGGA ATGGT ATGGC ATGGG
ACA ACAAA ACAAT ACAAC ACAAG ACATA ACATT ACATC ACATG ACACA ACACT ACACC
ACACG ACAGA ACAGT ACAGC ACAGG
ACT ACTAA ACTAT ACTAC ACTAG ACTTA ACTTT ACTTC ACTTG ACTCA ACTCT ACTCC
ACTCG ACTGA ACTGT ACTGC ACTGG
ACC ACCAA ACCAT ACCAC ACCAG ACCTA ACCTT ACCTC ACCTG ACCCA ACCCT ACCCC
ACCCG ACCGA ACCGT ACCGC ACCGG
ACG ACGAA ACGAT ACGAC ACGAG ACGTA ACGTT ACGTC ACGTG ACGCA ACGCT ACGCC
ACGCG ACGGA ACGGT ACGGC ACGGG
AGA AGAAA AGAAT AGAAC AGAAG AGATA AGATT AGATC AGATG AGACA AGACT AGACC
AGACG AGAGA AGAGT AGAGC AGAGG
AGT AGTAA AGTAT AGTAC AGTAG AGTTA AGTTT AGTTC AGTTG AGTCA AGTCT AGTCC
AGTCG AGTGA AGTGT AGTGC AGTGG
AGC AGCAA AGCAT AGCAC AGCAG AGCTA AGCTT AGCTC AGCTG AGCCA AGCCT AGCCC
AGCCG AGCGA AGCGT AGCGC AGCGG
AGG AGGAA AGGAT AGGAC AGGAG AGGTA AGGTT AGGTC AGGTG AGGCA AGGCT AGGCC
AGGCG AGGGA AGGGT AGGGC AGGGG
TAA TAAAA TAAAT TAAAC TAAAG TAATA TAATT TAATC TAATG TAACA TAACT TAACC
TAACG TAAGA TAAGT TAAGC TAAGG
TAT TATAA TATAT TATAC TATAG TATTA TATTT TATTC TATTG TATCA TATCT TATCC
TATCG TATGA TATGT TATGC TATGG
TAC TACAA TACAT TACAC TACAG TACTA TACTT TACTC TACTG TACCA TACCT

TACCC
TACCG TACGA TACGT TACGC TACGG
TAG TAGAA TAGAT TAGAC TAGAG TAGTA TAGTT TAGTC TAGTG TAGCA TAGCT
TAGCC
TAGCG TAGGA TAGGT TAGGC TAGGG
TTA TTAAA TTAAT TTAAC TTAAG TTATA TTATT TTATC TTATG TTACA TTACT TTACC
TTACG TTAGA TTAGT TTAGC TTAGG
TTT TTTAA TTTAT TTTAC TTTAG TTTTA TTTTT TTTTC TTTTG TTTCA TTTCT TTTCC
TTTCG TTTGA TTTGT TTTGC TTTGG
TTC TTCAA TTCAT TTCAC TTCAG TTCTA TTCTT TTCTC TTCTG TTCCA TTCCT TTCCC
TTCCG TTCGA TTCGT TTCGC TTCGG
TTG TTGAA TTGAT TTGAC TTGAG TTGTA TTGTT TTGTC TTGTG TTGCA TTGCT TTGCC
TTGCG TTGGA TTGGT TTGGC TTGGG
TCA TCAAA TCAAT TCAAC TCAAG TCATA TCATT TCATC TCATG TCACA TCACT
TCACC
TCACG TCAGA TCAGT TCAGC TCAGG
TCT TCTAA TCTAT TCTAC TCTAG TCTTA TCTTT TCTTC TCTTG TCTCA TCTCT TCTCC
TCTCG TCTGA TCTGT TCTGC TCTGG
TCC TCCAA TCCAT TCCAC TCCAG TCCTA TCCTT TCCTC TCCTG TCCCA TCCCT TCCCC
TCCCG TCCGA TCCGT TCCGC TCCGG
TCG TCGAA TCGAT TCGAC TCGAG TCGTA TCGTT TCGTC TCGTG TCGCA TCGCT
TCGCC
TCGCG TCGGA TCGGT TCGGC TCGGG
TGA TGAAA TGAAT TGAAC TGAAG TGATA TGATT TGATC TGATG TGACA TGACT
TGACC
TGACG TGAGA TGAGT TGAGC TGAGG
TGT TGTAA TGTAT TGTAC TGTAG TGTTA TGTTT TGTTC TGTTG TGTCA TGTCT TGTCC
TGTCG TGTGA TGTGT TGTGC TGTGG
TGC TGCAA TGCAT TGCAC TGCAG TGCTA TGCTT TGCTC TGCTG TGCCA TGCCT
TGCCC
TGCCG TGCGA TGCGT TGCGC TGCGG
TGG TGGAA TGGAT TGGAC TGGAG TGGTA TGGTT TGGTC TGGTG TGGCA TGGCT
TGGCC
TGGCG TGGGA TGGGT TGGGC TGGGG
CAA CAAAA CAAAT CAAAC CAAAG CAATA CAATT CAATC CAATG CAACA CAACT
CAACC
CAACG CAAGA CAAGT CAAGC CAAGG
CAT CATAA CATAT CATAC CATAG CATTA CATTT CATTC CATTG CATCA CATCT
CATCC
CATCG CATGA CATGT CATGC CATGG
CAC CACAA CACAT CACAC CACAG CACTA CACTT CACTC CACTG CACCA CACCT
CACCC
CACCG CACGA CACGT CACGC CACGG
CAG CAGAA CAGAT CAGAC CAGAG CAGTA CAGTT CAGTC CAGTG CAGCA CAGCT
CAGCC
CAGCG CAGGA CAGGT CAGGC CAGGG
CTA CTAAA CTAAT CTAAC CTAAG CTATA CTATT CTATC CTATG CTACA CTACT
CTACC
CTACG CTAGA CTAGT CTAGC CTAGG
CTT CTTAA CTTAT CTTAC CTTAG CTTTA CTTTT CTTTC CTTTG CTTCA CTTCT CTTCC
CTTCG CTTGA CTTGT CTTGC CTTGG
CTC CTCAA CTCAT CTCAC CTCAG CTCTA CTCTT CTCTC CTCTG CTCCA CTCCT CTCCC
CTCCG CTCGA CTCGT CTCGC CTCGG
CTG CTGAA CTGAT CTGAC CTGAG CTGTA CTGTT CTGTC CTGTG CTGCA CTGCT
CTGCC
CTGCG CTGGA CTGGT CTGGC CTGGG
CCA CCAAA CCAAT CCAAC CCAAG CCATA CCATT CCATC CCATG CCACA CCACT
CCACC

CCACG CCAGA CCAGT CCAGC CCAGG
CCT CCTAA CCTAT CCTAC CCTAG CCTTA CCTTT CCTTC CCTTG CCTCA CCTCT CCTCC
CCTCG CCTGA CCTGT CCTGC CCTGG
CCC CCCAA CCCAT CCCAC CCCAG CCCTA CCCTT CCCTC CCCTG CCCCA CCCCT
CCCCC
CCCCG CCCGA CCCGT CCCGC CCCGG
CCG CCGAA CCGAT CCGAC CCGAG CCGTA CCGTT CCGTC CCGTG CCGCA CCGCT
CCGCC
CCGCG CCGGA CCGGT CCGGC CCGGG
CGA CGAAA CGAAT CGAAC CGAAG CGATA CGATT CGATC CGATG CGACA CGACT
CGACC
CGACG CGAGA CGAGT CGAGC CGAGG
CGT CGTAA CGTAT CGTAC CGTAG CGTTA CGTTT CGTTC CGTTG CGTCA CGTCT
CGTCC
CGTCG CGTGA CGTGT CGTGC CGTGG
CGC CGCAA CGCAT CGCAC CGCAG CGCTA CGCTT CGCTC CGCTG CGCCA CGCCT
CGCCC
CGCCG CGCGA CGCGT CGCGC CGCGG
CGG CGGAA CGGAT CGGAC CGGAG CGGTA CGGTT CGGTC CGGTG CGGCA CGGCT
CGGCC
CGGCG CGGGA CGGGT CGGGC CGGGG
GAA GAAAA GAAAT GAAAC GAAAG GAATA GAATT GAATC GAATG GAACA GAACT
GAACC
GAACG GAAGA GAAGT GAAGC GAAGG
GAT GATAA GATAT GATAC GATAG GATTA GATTT GATTC GATTG GATCA GATCT
GATCC
GATCG GATGA GATGT GATGC GATGG
GAC GACAA GACAT GACAC GACAG GACTA GACTT GACTC GACTG GACCA GACCT
GACCC
GACCG GACGA GACGT GACGC GACGG
GAG GAGAA GAGAT GAGAC GAGAG GAGTA GAGTT GAGTC GAGTG GAGCA GAGCT
GAGCC
GAGCG GAGGA GAGGT GAGGC GAGGG
GTA GTAAA GTAAT GTAAC GTAAG GTATA GTATT GTATC GTATG GTACA GTACT
GTACC
GTACG GTAGA GTAGT GTAGC GTAGG
GTT GTTAA GTTAT GTTAC GTTAG GTTTA GTTTT GTTTC GTTTG GTTCA GTTCT GTTCC
GTTCG GTTGA GTTGT GTTGC GTTGG
GTC GTCAA GTCAT GTCAC GTCAG GTCTA GTCTT GTCTC GTCTG GTCCA GTCCT
GTCCC
GTCCG GTCGA GTCGT GTCGC GTCGG
GTG GTGAA GTGAT GTGAC GTGAG GTGTA GTGTT GTGTC GTGTG GTGCA GTGCT
GTGCC
GTGCG GTGGA GTGGT GTGGC GTGGG
GCA GCAAA GCAAT GCAAC GCAAG GCATA GCATT GCATC GCATG GCACA GCACT
GCACC
GCACG GCAGA GCAGT GCAGC GCAGG
GCT GCTAA GCTAT GCTAC GCTAG GCTTA GCTTT GCTTC GCTTG GCTCA GCTCT
GCTCC
GCTCG GCTGA GCTGT GCTGC GCTGG
GCC GCCAA GCCAT GCCAC GCCAG GCCTA GCCTT GCCTC GCCTG GCCCA GCCCT
GCCCC
GCCCG GCCGA GCCGT GCCGC GCCGG
GCG GCGAA GCGAT GCGAC GCGAG GCGTA GCGTT GCGTC GCGTG GCGCA GCGCT
GCGCC
GCGCG GCGGA GCGGT GCGGC GCGGG
GGA GGAAA GGAAT GGAAC GGAAG GGATA GGATT GGATC GGATG GGACA GGACT
GGACC

GGACG GGAGA GGAGT GGAGC GGAGG
GGT GGTAA GGTAT GGTAC GGTAG GGTTA GGTTT GGTTC GGTTG GGTCA GGTCT GGTCC
GGTCG GGTGA GGTGT GGTGC GGTGG
GGC GGCAA GGCAT GGCAC GGCAG GGCTA GGCTT GGCTC GGCTG GGCCA GGCCT GGCCC
GGCCG GGCGA GGCGT GGCGC GGCGG
GGG GGGAA GGGAT GGGAC GGGAG GGGTA GGGTT GGGTC GGGTG GGGCA GGGCT GGGCC
GGGCG GGGGA GGGGT GGGGC GGGGG

After filtering: read2: overrepresented sequences
Sampling rate: 1 / 20
overrepresented sequence count (% of bases) distribution: cycle 1 ~ cycle 151

AAAAAAAAAAAAAAAAAAAAAAAAAAAAAAAAAAAAAAAAAAAAAAAAAAAAAAAAAAAAAAAAAAAAAAAAAAAAAAAAAAAAAAAAAAAAAAAAAAAAAAAAAAA 272 (0.007062%)

AAAAAAAAGGCCGCCGTGACCTATTCACCCTCCACTTCCCGTCTCAGAATCTAAACGTGGTCACCTTCGAGTAGAGAGGCCCGCCCGCCCACCGTGGGCA 487 (0.012645%)

AAAAAAAGGCCGCCGTGACCTATTCACCCTCCACTTCCCGTCTCAGAATCTAAACGTGGTCACCTTCGAGTAGAGAGGCCCGCCCGCCCACCGTGGGCAG 26 (0.000675%)

AAAAAAGGCCGCCGTGACCTATTCACCCTCCACTTCCCGTCTCAGAATCTAAACGTGGTCACCTTCGAGTAGAGAGGCCCGCCCGCCCACCGTGGGCAGT 18 (0.000467%)

AAAAAATTATAACAAACCCTGAGAACCAAAATGAACGAAAATCTGTTCGCTTCATTCATTGCCCCCACAATCCTAGGCCTACCCGCCGCAGTACTGATCA 49 (0.001272%)

AAAAAGCTGGAAGATGGCCCTAAATTCTTGAAGTCTGGTGATGCTGCCATTGTTGATATGGTTCCTGGCAAGCCCATGTGTGTTGAGAGCTTCTCAGACT 26 (0.000675%)

AAAAAGGCCGCCGTGACCTATTCACCCTCCACTTCCCGTCTCAGAATCTAAACGTGGTCACCTTCGAGTAGAGAGGCCCGCCCGCCCACCGTGGGCAGTG 13 (0.000338%)

AAAAAGGGCCGTTCTGCCATCAACGAAGTGGTAACCCGAGAATACACCATCAACATTCACAAGCGCATCCATGGAGTGGGCTTCAAGAAGCGTGCACCTC 34 (0.000883%)

AAAAATAAAAAATTATAACAAACCCTGAGAACCAAAATGAACGAAAATCTGTTCGCTTCATTCATTGCCCCCACAATCCTAGGCCTACCCGCCGCAGTAC 16 (0.000415%)

AAAAATGACCCACCAATGGAAGCAGCTGGCTTCACTGCTCAGGTGATTATCCTGAACCATCCAGGCCAAATAAGCGCCGGCTATGCCCCTGTATTGGATT 24 (0.000623%)

AAAAATTATAACAAACCCTGAGAACCAAAATGAACGAAAATCTGTTCGCTTCATTCATTGCCCCCACAATCCTAGGCCTACCCGCCGCAGTACTGATCAT 68 (0.001766%)

AAAACAAATGATAACCATACACAACACTAAAGGACGAACCTGATCTCTTATACTAGTATCCTTAATCATTTTTATTGCCACAACTAACCTCCTCGGACTC 17 (0.000441%)

AAAACAAGAAGAGGGTCCTGCTGGGAGAAACTGGCAAGGAGAAGCTCCCGCGGTACTACAAGAACATCGGTCTGGGCTTCAAGACACCCAAGGAGGCTAT 5 (0.000130%)

AAAACCCAGCCCATGACCCCTAACAGGGGCCCTCTCAGCCCTCCTAATGACCTCCGGCCTAGCCATGTGATTTCACTTCCACTCCATAACGCTCCTCATA 42 (0.001091%)

AAAACCCGCCGGACTTTCTGTAAGAAGTGTGGCAAGCACCAACCCCATAAAGTGACACAGTACAAGAA
GGGCAAGGATTCTCTGTACGCCCAGGGAAAGC 6 (0.000156%)

AAAACTACCCCTAAAAGCCAAAATGGGAAAGGAAAAGACTCATATCAACATTGTCGTCATTGGACACG
TAGATTCGGGCAAGTCCACCACTACTGGCCAT 41 (0.001065%)

AAAAGAAGAAAGATGAGGCAGAGGTCCAAGTAAACCGCTAGCTTGTTGCACCGTGGAGGCCACAGGA
GCAGAAACATGGAATGCCAGACGCTGGGGATGC 9 (0.000234%)

AAAAGACTCATATCAACATTGTCGTCATTGGACACGTAGATTCGGGCAAGTCCACCACTACTGGCCATC
TGATCTATAAATGCGGTGGCATCGACAAAAG 140 (0.003635%)

AAAAGCAGCCGACCATCTTTCAAAACAAGAAGAGGGTCCTGCTGGGAGAAACTGGCAAGGAGAAGCTC
CCGCGGTACTACAAGAACATCGGTCTGGGCTT 4 (0.000104%)

AAAAGCCAAAATGGGAAAGGAAAAGACTCATATCAACATTGTCGTCATTGGACACGTAGATTCGGGCA
AGTCCACCACTACTGGCCATCTGATCTATAAA 25 (0.000649%)

AAAAGCTGGAAGATGGCCCTAAATTCTTGAAGTCTGGTGATGCTGCCATTGTTGATATGGTTCCTGGCA
AGCCCATGTGTGTTGAGAGCTTCTCAGACTA 30 (0.000779%)

AAAAGGCCGCCGTGACCTATTCACCCTCCACTTCCCGTCTCAGAATCTAAACGTGGTCACCTTCGAGTA
GAGAGGCCCGCCCGCCCACCGTGGGCAGTGC 7 (0.000182%)

AAAAGGGCCGTTCTGCCATCAACGAAGTGGTAACCCGAGAATACACCATCAACATTCACAAGCGCATC
CATGGAGTGGGCTTCAAGAAGCGTGCACCTCG 18 (0.000467%)

AAAATAAAAAATTATAACAAACCCTGAGAACCAAAATGAACGAAAATCTGTTCGCTTCATTCATTGCCC
CCACAATCCTAGGCCTACCCGCCGCAGTACT 6 (0.000156%)

AAAATCTGTCGAAATGCACCATGAAGCTTTGAGTGAAGCTCTTCCTGGGGACAATGTGGGCTTCAATGT
CAAGAATGTGTCTGTCAAGGATGTTCGTCGT 13 (0.000338%)

AAAATGACCCACCAATGGAAGCAGCTGGCTTCACTGCTCAGGTGATTATCCTGAACCATCCAGGCCAAA
TAAGCGCCGGCTATGCCCCTGTATTGGATTG 3 (0.000078%)

AAAATGGGAAAGGAAAAGACTCATATCAACATTGTCGTCATTGGACACGTAGATTCGGGCAAGTCCAC
CACTACTGGCCATCTGATCTATAAATGCGGTG 33 (0.000857%)

AAAATTATAACAAACCCTGAGAACCAAAATGAACGAAAATCTGTTCGCTTCATTCATTGCCCCCACAAT
CCTAGGCCTACCCGCCGCAGTACTGATCATT 18 (0.000467%)

AAACAAATGATAACCATACACAACACTAAAGGACGAACCTGATCTCTTATACTAGTATCCTTAATCATT
TTTATTGCCACAACTAACCTCCTCGGACTCC 8 (0.000208%)

AAACAAATGGTCATTGATGTCCTTCACCCCGGGAAGGCGACAGTGCCTAAGACAGAAATTCGGGAAAA
ACTAGCCAAAATGTACAAGACCACACCGGATG 7 (0.000182%)

AAACAAGAAGAGGGTCCTGCTGGGAGAAACTGGCAAGGAGAAGCTCCCGCGGTACTACAAGAACATC
GGTCTGGGCTTCAAGACACCCAAGGAGGCTATT 7 (0.000182%)

AAACCCTGAGAACCAAAATGAACGAAAATCTGTTCGCTTCATTCATTGCCCCCACAATCCTAGGCCTAC
CCGCCGCAGTACTGATCATTCTATTTCCCCC 5 (0.000130%)

AAACCGCTAGCTTGTTGCACCGTGGAGGCCACAGGAGCAGAAACATGGAATGCCAGACGCTGGGGATG
CTGGTACAAGTTGTGGGACTGCATGCTACTGT 6 (0.000156%)

AAACTACCCCTAAAAGCCAAAATGGGAAAGGAAAAGACTCATATCAACATTGTCGTCATTGGACACGT

AGATTCGGGCAAGTCCACCACTACTGGCCATC 19 (0.000493%)

AAACTCTACTCCCACTAATAGCTTTTGATGACTTCTAGCAAGCCTCGCTAACCTCGCCTTACCCCCCAC
TATTAACCTACTGGGAGAACTCTCTGTGCT 3 (0.000078%)

AAACTTCGGCTCACTCCTTGGCGCCTGCCTGATCCTCCAAATCACCACAGGACTATTCCTAGCCATGCAC
TACTCACCAGACGCCTCAACCGCCTTTTCA 3 (0.000078%)

AAAGAAGAAAGATGAGGCAGAGGTCCAAGTAAACCGCTAGCTTGTTGCACCGTGGAGGCCACAGGAGC
AGAAACATGGAATGCCAGACGCTGGGGATGCT 4 (0.000104%)

AAAGAAGGGTGGCGAGAAGAAAAAGGGCCGTTCTGCCATCAACGAAGTGGTAACCCGAGAATACACC
ATCAACATTCACAAGCGCATCCATGGAGTGGGC 7 (0.000182%)

AAAGACTCATATCAACATTGTCGTCATTGGACACGTAGATTCGGGCAAGTCCACCACTACTGGCCATCT
GATCTATAAATGCGGTGGCATCGACAAAAGA 33 (0.000857%)

AAAGATGAGGCAGAGGTCCAAGTAAACCGCTAGCTTGTTGCACCGTGGAGGCCACAGGAGCAGAAACA
TGGAATGCCAGACGCTGGGGATGCTGGTACAA 5 (0.000130%)

AAAGCAGCCGACCATCTTTCAAAACAAGAAGAGGGTCCTGCTGGGAGAAACTGGCAAGGAGAAGCTCC
CGCGGTACTACAAGAACATCGGTCTGGGCTTC 10 (0.000260%)

AAAGCCAAAATGGGAAAGGAAAAGACTCATATCAACATTGTCGTCATTGGACACGTAGATTCGGGCAA
GTCCACCACTACTGGCCATCTGATCTATAAAT 8 (0.000208%)

AAAGCCCATAAAAATAAAAAATTATAACAAACCCTGAGAACCAAAATGAACGAAAATCTGTTCGCTTC
ATTCATTGCCCCCACAATCCTAGGCCTACCCG 19 (0.000493%)

AAAGCTCAGAAGGCTAAATGAATATTATCCCTAATACCTGCCACCCCACTCTTAATCAGTGGTGGAAGA
ACGGTCTCAGAACTGTTTGTTTCAATTGGCC 26 (0.000675%)

AAAGCTGGAAGATGGCCCTAAATTCTTGAAGTCTGGTGATGCTGCCATTGTTGATATGGTTCCTGGCAA
GCCCATGTGTGTTGAGAGCTTCTCAGACTAT 9 (0.000234%)

AAAGGAAAAGACTCATATCAACATTGTCGTCATTGGACACGTAGATTCGGGCAAGTCCACCACTACTGG
CCATCTGATCTATAAATGCGGTGGCATCGAC 19 (0.000493%)

AAAGGACGAACCTGATCTCTTATACTAGTATCCTTAATCATTTTTATTGCCACAACTAACCTCCTCGGAC
TCCTGCCTCACTCATTTACACCAACCACCC 12 (0.000312%)

AAAGGATCTCCTTCATCCCTCTCCAGAAGAGGAGAAGAGGAAACACAAGAAGAAACGCCTGGTGCAGA
GCCCCAATTCCTACTTCATGGATGTGAAATGC 9 (0.000234%)

AAAGGGCCGTTCTGCCATCAACGAAGTGGTAACCCGAGAATACACCATCAACATTCACAAGCGCATCC
ATGGAGTGGGCTTCAAGAAGCGTGCACCTCGG 4 (0.000104%)

AAAGTTCTCCGCTCCCAGACATGGGTCCCTCGGCTTCCTGCCTCGGAAGCGCAGCAGCAGGCATCGTGG
GAAGGTGAAGAGCTTCCCTAAGGATGACCCG 6 (0.000156%)

AAATAAAAAATTATAACAAACCCTGAGAACCAAAATGAACGAAAATCTGTTCGCTTCATTCATTGCCCC
CACAATCCTAGGCCTACCCGCCGCAGTACTG 3 (0.000078%)

AAATGAATATTATCCCTAATACCTGCCACCCCACTCTTAATCAGTGGTGGAAGAACGGTCTCAGAACTG
TTTGTTTCAATTGGCCATTTAAGTTTAGTAG 4 (0.000104%)

AAATGACCCACCAATGGAAGCAGCTGGCTTCACTGCTCAGGTGATTATCCTGAACCATCCAGGCCAAAT
AAGCGCCGGCTATGCCCCTGTATTGGATTGC 32 (0.000831%)

AAATGATAACCATACACAACACTAAAGGACGAACCTGATCTCTTATACTAGTATCCTTAATCATTTTTAT
TGCCACAACTAACCTCCTCGGACTCCTGCC 17 (0.000441%)

AAATGGGAAAGGAAAAGACTCATATCAACATTGTCGTCATTGGACACGTAGATTCGGGCAAGTCCACC
ACTACTGGCCATCTGATCTATAAATGCGGTGG 10 (0.000260%)

AAATTATAACAAACCCTGAGAACCAAAATGAACGAAAATCTGTTCGCTTCATTCATTGCCCCCACAATC
CTAGGCCTACCCGCCGCAGTACTGATCATTC 12 (0.000312%)

AAATTCTTGAAGTCTGGTGATGCTGCCATTGTTGATATGGTTCCTGGCAAGCCCATGTGTGTTGAGAGCT
TCTCAGACTATCCACCTTTGGGTCGCTTTG95 (0.002467%)

AACAAAACTAACTAATACTAACATCTCAGACGCTCAGGAAATAGAAACCGTCTGAACTATCCTGCCCGC
CATCATCCTAGTCCTCATCGCCCTCCCATCC 38 (0.000987%)

AACAAACCCTGAGAACCAAAATGAACGAAAATCTGTTCGCTTCATTCATTGCCCCCACAATCCTAGGCC
TACCCGCCGCAGTACTGATCATTCTATTTCC 11 (0.000286%)

AACAAATGATAACCATACACAACACTAAAGGACGAACCTGATCTCTTATACTAGTATCCTTAATCATTT
TTATTGCCACAACTAACCTCCTCGGACTCCT 11 (0.000286%)

AACAAATGGTCATTGATGTCCTTCACCCCGGGAAGGCGACAGTGCCTAAGACAGAAATTCGGGAAAAA
CTAGCCAAAATGTACAAGACCACACCGGATGT 3 (0.000078%)

AACACAGGTGTCGTGAAAACTACCCCTAAAAGCCAAAATGGGAAAGGAAAAGACTCATATCAACATTG
TCGTCATTGGACACGTAGATTCGGGCAAGTCC 24 (0.000623%)

AACACTAAAGGACGAACCTGATCTCTTATACTAGTATCCTTAATCATTTTTATTGCCACAACTAACCTCC
TCGGACTCCTGCCTCACTCATTTACACCAA 5 (0.000130%)

AACACTCACAACAAAACTAACTAATACTAACATCTCAGACGCTCAGGAAATAGAAACCGTCTGAACTA
TCCTGCCCGCCATCATCCTAGTCCTCATCGCC 9 (0.000234%)

AACAGGGGCCCTCTCAGCCCTCCTAATGACCTCCGGCCTAGCCATGTGATTTCACTTCCACTCCATAACG
CTCCTCATACTAGGCCTACTAACCAACACA 7 (0.000182%)

AACATCTCCGCATGATGAAACTTCGGCTCACTCCTTGGCGCCTGCCTGATCCTCCAAATCACCACAGGA
CTATTCCTAGCCATGCACTACTCACCAGACG 6 (0.000156%)

AACATGCCTCTCGCAAAGGATCTCCTTCATCCCTCTCCAGAAGAGGAGAAGAGGAAACACAAGAAGAA
ACGCCTGGTGCAGAGCCCCAATTCCTACTTCA 5 (0.000130%)

AACCATACACAACACTAAAGGACGAACCTGATCTCTTATACTAGTATCCTTAATCATTTTTATTGCCACA
ACTAACCTCCTCGGACTCCTGCCTCACTCA 3 (0.000078%)

AACCCAGCCCATGACCCCTAACAGGGGCCCTCTCAGCCCTCCTAATGACCTCCGGCCTAGCCATGTGAT
TTCACTTCCACTCCATAACGCTCCTCATACT 10 (0.000260%)

AACCGCTAGCTTGTTGCACCGTGGAGGCCACAGGAGCAGAAACATGGAATGCCAGACGCTGGGGATGC
TGGTACAAGTTGTGGGACTGCATGCTACTGTC 4 (0.000104%)

AACGCAGGCACATACTTCCTATTCTACACCCTAGTAGGCTCCCTTCCCCTACTCATCGCACTGATTTACA
CTCACAACACCCTAGGCTCACTAAACATTC 3 (0.000078%)

AACGGAAGTAAAATCTGTCGAAATGCACCATGAAGCTTTGAGTGAAGCTCTTCCTGGGGACAATGTGG
GCTTCAATGTCAAGAATGTGTCTGTCAAGGAT 10 (0.000260%)

AACGTTACAACGGAAGTAAAATCTGTCGAAATGCACCATGAAGCTTTGAGTGAAGCTCTTCCTGGGGAC
AATGTGGGCTTCAATGTCAAGAATGTGTCTG 4 (0.000104%)

AACGTTGCTGGTGACAGCAAAAATGACCCACCAATGGAAGCAGCTGGCTTCACTGCTCAGGTGATTATC
CTGAACCATCCAGGCCAAATAAGCGCCGGCT 6 (0.000156%)

AACTACCCCTAAAAGCCAAAATGGGAAAGGAAAAGACTCATATCAACATTGTCGTCATTGGACACGTA
GATTCGGGCAAGTCCACCACTACTGGCCATCT 14 (0.000364%)

AACTTCCTTCGGTCGTCCCGAATCCGGGTTCATCCGACACCAGCCGCCTCCACCATGCCGCCGAAGTTC
GACCCCAACGAGATCAAAGTCGTATACCTGA 5 (0.000130%)

AAGAAAAAGGGCCGTTCTGCCATCAACGAAGTGGTAACCCGAGAATACACCATCAACATTCACAAGCG
CATCCATGGAGTGGGCTTCAAGAAGCGTGCAC 4 (0.000104%)

AAGAAAGATGAGGCAGAGGTCCAAGTAAACCGCTAGCTTGTTGCACCGTGGAGGCCACAGGAGCAGAA
ACATGGAATGCCAGACGCTGGGGATGCTGGTA 4 (0.000104%)

AAGAAGGGTGGCGAGAAGAAAAAGGGCCGTTCTGCCATCAACGAAGTGGTAACCCGAGAATACACCAT
CAACATTCACAAGCGCATCCATGGAGTGGGCT3 (0.000078%)

AAGAAGGTGATGGTGAGGAAGAGGATGGAGATGAAGATGAGGAAGCTGAGTCAGCTACGGGCAAGCG
GGCAGCTGAAGATGATGAGGATGACGATGTCGA 380 (0.009867%)

AAGAATGTGTCTGTCAAGGATGTTCGTCGTGGCAACGTTGCTGGTGACAGCAAAAATGACCCACCAATG
GAAGCAGCTGGCTTCACTGCTCAGGTGATTA 9 (0.000234%)

AAGACTCATATCAACATTGTCGTCATTGGACACGTAGATTCGGGCAAGTCCACCACTACTGGCCATCTG
ATCTATAAATGCGGTGGCATCGACAAAAGAA 16 (0.000415%)

AAGAGGATGGAGATGAAGATGAGGAAGCTGAGTCAGCTACGGGCAAGCGGGCAGCTGAAGATGATGA
GGATGACGATGTCGATACCAAGAAGCAGAAGAC 8 (0.000208%)

AAGATATGCTCATGTGGTGTTGAGGAAAGCAGACATTGACCTCACCAAGAGGGCGGGAGAACTCACTG
AGGATGAGGTGGAACGTGTGATCACCATTATG5 (0.000130%)

AAGATGAGGAAGCTGAGTCAGCTACGGGCAAGCGGGCAGCTGAAGATGATGAGGATGACGATGTCGAT
ACCAAGAAGCAGAAGACCGACGAGGATGACTA 28 (0.000727%)

AAGATGGCCCTAAATTCTTGAAGTCTGGTGATGCTGCCATTGTTGATATGGTTCCTGGCAAGCCCATGTG
TGTTGAGAGCTTCTCAGACTATCCACCTTT9 (0.000234%)

AAGCAGCCGACCATCTTTCAAAACAAGAAGAGGGTCCTGCTGGGAGAAACTGGCAAGGAGAAGCTCCC
GCGGTACTACAAGAACATCGGTCTGGGCTTCA16 (0.000415%)

AAGCAGCTGGCTTCACTGCTCAGGTGATTATCCTGAACCATCCAGGCCAAATAAGCGCCGGCTATGCCC
CTGTATTGGATTGCCACACGGCTCACATTGC 15 (0.000389%)

AAGCCCATAAAAATAAAAAATTATAACAAACCCTGAGAACCAAAATGAACGAAAATCTGTTCGCTTCA
TTCATTGCCCCCACAATCCTAGGCCTACCCGC 6 (0.000156%)

AAGCTCAGAAGGCTAAATGAATATTATCCCTAATACCTGCCACCCCACTCTTAATCAGTGGTGGAAGAA
CGGTCTCAGAACTGTTTGTTTCAATTGGCCA 12 (0.000312%)

AAGCTGAGTCAGCTACGGGCAAGCGGGCAGCTGAAGATGATGAGGATGACGATGTCGATACCAAGAAG
CAGAAGACCGACGAGGATGACTAGACAGCAAA 31 (0.000805%)

AAGCTGGAAGATGGCCCTAAATTCTTGAAGTCTGGTGATGCTGCCATTGTTGATATGGTTCCTGGCAAG

CCCATGTGTGTTGAGAGCTTCTCAGACTATC 5 (0.000130%)

AAGGAAAAGACTCATATCAACATTGTCGTCATTGGACACGTAGATTCGGGCAAGTCCACCACTACTGGC
CATCTGATCTATAAATGCGGTGGCATCGACA 10 (0.000260%)

AAGGACTTCAAACTCTACTCCCACTAATAGCTTTTGATGACTTCTAGCAAGCCTCGCTAACCTCGCCTT
ACCCCCCACTATTAACCTACTGGGAGAACT 6 (0.000156%)

AAGGAGCTAGGAGTGGGAATAGCTTTGCGAAAAATGGGCGCAATGGCCAAGCCAGATTGTATCATCAC
TTGTGATGGTAAAAACCTCACCATAAAAACTG 133 (0.003453%)

AAGGATGTTCGTCGTGGCAACGTTGCTGGTGACAGCAAAAATGACCCACCAATGGAAGCAGCTGGCTT
CACTGCTCAGGTGATTATCCTGAACCATCCAG 10 (0.000260%)

AAGGCCTCGGGCACGCTACGAGAGTACAAGGTAGTGGGTCGCTGCCTGCCCACCCCCAAATGCCACAC
GCCGCCCCTCTACCGCATGCGAATCTTTGCGC 8 (0.000208%)

AAGGCTGCTGGAGCTGGCAAGGTCACCAAGTCTGCCCAGAAAGCTCAGAAGGCTAAATGAATATTATC
CCTAATACCTGCCACCCCACTCTTAATCAGTG 1220 (0.031677%)

AAGGGCCGTTCTGCCATCAACGAAGTGGTAACCCGAGAATACACCATCAACATTCACAAGCGCATCCAT
GGAGTGGGCTTCAAGAAGCGTGCACCTCGGG 3 (0.000078%)

AAGGTCACCAAGTCTGCCCAGAAAGCTCAGAAGGCTAAATGAATATTATCCCTAATACCTGCCACCCCA
CTCTTAATCAGTGGTGGAAGAACGGTCTCAG 6 (0.000156%)

AAGGTGATGGTGAGGAAGAGGATGGAGATGAAGATGAGGAAGCTGAGTCAGCTACGGGCAAGCGGGC
AGCTGAAGATGATGAGGATGACGATGTCGATAC 9 (0.000234%)

AAGTAAAATCTGTCGAAATGCACCATGAAGCTTTGAGTGAAGCTCTTCCTGGGGACAATGTGGGCTTCA
ATGTCAAGAATGTGTCTGTCAAGGATGTTCG 6 (0.000156%)

AAGTAAACCGCTAGCTTGTTGCACCGTGGAGGCCACAGGAGCAGAAACATGGAATGCCAGACGCTGGG
GATGCTGGTACAAGTTGTGGGACTGCATGCTA 13 (0.000338%)

AAGTCCACCACTACTGGCCATCTGATCTATAAATGCGGTGGCATCGACAAAAGAACCATTGAAAAATTT
GAGAAGGAGGCTGCTGAGATGGGAAAGGGCT 6 (0.000156%)

AAGTTGGGCCGCTTGGTCAAGGACATGAAGATCAAGTCCCTGGAGGAGATCTATCTCTTCTCCCTGCCC
ATTAAGGAATCAGAGATCATTGATTTCTTCC 6 (0.000156%)

AATACCTGCCACCCCACTCTTAATCAGTGGTGGAAGAACGGTCTCAGAACTGTTTGTTTCAATTGGCCAT
TTAAGTTTAGTAGTAAAAGACTGGTTAATG 7 (0.000182%)

AATCACAGCAGTCCTACTTCTCCTATCTCTCCCAGTCCTAGCTGCTGGCATCACTATACTACTAACAGAC
CGCAACCTCAACACCACCTTCTTCGACCCC 10 (0.000260%)

AATCTGTCGAAATGCACCATGAAGCTTTGAGTGAAGCTCTTCCTGGGGACAATGTGGGCTTCAATGTCA
AGAATGTGTCTGTCAAGGATGTTCGTCGTGG 18 (0.000467%)

AATGAATATTATCCCTAATACCTGCCACCCCACTCTTAATCAGTGGTGGAAGAACGGTCTCAGAACTGT
TTGTTTCAATTGGCCATTTAAGTTTAGTAGT 7 (0.000182%)

AATGACCCACCAATGGAAGCAGCTGGCTTCACTGCTCAGGTGATTATCCTGAACCATCCAGGCCAAATA
AGCGCCGGCTATGCCCCTGTATTGGATTGCC 4 (0.000104%)

AATGCGCAGGCTGAAGCGCAAAAGAAGAAAGATGAGGCAGAGGTCCAAGTAAACCGCTAGCTTGTTGC
ACCGTGGAGGCCACAGGAGCAGAAACATGGAA 3 (0.000078%)

AATGGAAGCAGCTGGCTTCACTGCTCAGGTGATTATCCTGAACCATCCAGGCCAAATAAGCGCCGGCTA
TGCCCCTGTATTGGATTGCCACACGGCTCAC 18 (0.000467%)

AATGGGAAAGGAAAAGACTCATATCAACATTGTCGTCATTGGACACGTAGATTCGGGCAAGTCCACCA
CTACTGGCCATCTGATCTATAAATGCGGTGGC 5 (0.000130%)

AATGTCAAGAATGTGTCTGTCAAGGATGTTCGTCGTGGCAACGTTGCTGGTGACAGCAAAAATGACCCA
CCAATGGAAGCAGCTGGCTTCACTGCTCAGG 5 (0.000130%)

AATGTGTCTGTCAAGGATGTTCGTCGTGGCAACGTTGCTGGTGACAGCAAAAATGACCCACCAATGGAA
GCAGCTGGCTTCACTGCTCAGGTGATTATCC 3 (0.000078%)

AATTATAACAAACCCTGAGAACCAAAATGAACGAAAATCTGTTCGCTTCATTCATTGCCCCCACAATCC
TAGGCCTACCCGCCGCAGTACTGATCATTCT 6 (0.000156%)

AATTCTTGAAGTCTGGTGATGCTGCCATTGTTGATATGGTTCCTGGCAAGCCCATGTGTGTTGAGAGCTT
CTCAGACTATCCACCTTTGGGTCGCTTTGC 16 (0.000415%)

ACAAATGATAACCATACACAACACTAAAGGACGAACCTGATCTCTTATACTAGTATCCTTAATCATTTTT
ATTGCCACAACTAACCTCCTCGGACTCCTG 6 (0.000156%)

ACAACACTAAAGGACGAACCTGATCTCTTATACTAGTATCCTTAATCATTTTTATTGCCACAACTAACCT
CCTCGGACTCCTGCCTCACTCATTTACACC 24 (0.000623%)

ACACAGGTGTCGTGAAAACTACCCCTAAAAGCCAAAATGGGAAAGGAAAAGACTCATATCAACATTGT
CGTCATTGGACACGTAGATTCGGGCAAGTCCA 22 (0.000571%)

ACACCTCATATCCTCCCTACTATGCCTAGAAGGAATAATACTATCGCTGTTCATTATAGCTACTCTCATA
ACCCTCAACACCCACTCCCTCTTAGCCAAT 469 (0.012178%)

ACAGGGGCCCTCTCAGCCCTCCTAATGACCTCCGGCCTAGCCATGTGATTTCACTTCCACTCCATAACGC
TCCTCATACTAGGCCTACTAACCAACACAC 3 (0.000078%)

ACAGGTGTCGTGAAAACTACCCCTAAAAGCCAAAATGGGAAAGGAAAAGACTCATATCAACATTGTCG
TCATTGGACACGTAGATTCGGGCAAGTCCACC 20 (0.000519%)

ACATGCCTCTCGCAAAGGATCTCCTTCATCCCTCTCCAGAAGAGGAGAAGAGGAAACACAAGAAGAAA
CGCCTGGTGCAGAGCCCCAATTCCTACTTCAT 7 (0.000182%)

ACCAAAAGCAGCCGACCATCTTTCAAAACAAGAAGAGGGTCCTGCTGGGAGAAACTGGCAAGGAGAA
GCTCCCGCGGTACTACAAGAACATCGGTCTGGG 4 (0.000104%)

ACCAAAGCCCATAAAAATAAAAAATTATAACAAACCCTGAGAACCAAAATGAACGAAAATCTGTTCGC
TTCATTCATTGCCCCCACAATCCTAGGCCTAC 11 (0.000286%)

ACCAAGTCTGCCCAGAAAGCTCAGAAGGCTAAATGAATATTATCCCTAATACCTGCCACCCCACTCTTA
ATCAGTGGTGGAAGAACGGTCTCAGAACTGT 33 (0.000857%)

ACCAAGTTGGGCCGCTTGGTCAAGGACATGAAGATCAAGTCCCTGGAGGAGATCTATCTCTTCTCCCTG
CCCATTAAGGAATCAGAGATCATTGATTTCT 8 (0.000208%)

ACCAATGGAAGCAGCTGGCTTCACTGCTCAGGTGATTATCCTGAACCATCCAGGCCAAATAAGCGCCGG
CTATGCCCCTGTATTGGATTGCCACACGGCT 13 (0.000338%)

ACCACATCTACAACGTTATCGTCACAGCCCATGCATTTGTAATAATCTTCTTCATAGTAATACCCATCAT
AATCGGAGGCTTTGGCAACTGACTAGTTCC 13 (0.000338%)

ACCATACACAACACTAAAGGACGAACCTGATCTCTTATACTAGTATCCTTAATCATTTTTATTGCCACAA
CTAACCTCCTCGGACTCCTGCCTCACTCAT 11 (0.000286%)

ACCATCTTTCAAAACAAGAAGAGGGTCCTGCTGGGAGAAACTGGCAAGGAGAAGCTCCCGCGGTACTA
CAAGAACATCGGTCTGGGCTTCAAGACACCCA 3 (0.000078%)

ACCATTTGGATACATAGGTATGGTCTGAGCTATGATATCAATTGGCTTCCTAGGGTTTATCGTGTGAGCA
CACCATATATTTACAGTAGGAATAGACGTA 533 (0.013839%)

ACCCACCAATGGAAGCAGCTGGCTTCACTGCTCAGGTGATTATCCTGAACCATCCAGGCCAAATAAGCG
CCGGCTATGCCCCTGTATTGGATTGCCACAC 3 (0.000078%)

ACCCAGCCCATGACCCCTAACAGGGGCCCTCTCAGCCCTCCTAATGACCTCCGGCCTAGCCATGTGATT
TCACTTCCACTCCATAACGCTCCTCATACTA 8 (0.000208%)

ACCCCTAAAAGCCAAAATGGGAAAGGAAAAGACTCATATCAACATTGTCGTCATTGGACACGTAGATT
CGGGCAAGTCCACCACTACTGGCCATCTGATC 3 (0.000078%)

ACCCCTAACAGGGGCCCTCTCAGCCCTCCTAATGACCTCCGGCCTAGCCATGTGATTTCACTTCCACTCC
ATAACGCTCCTCATACTAGGCCTACTAACC 3 (0.000078%)

ACCCGAGAGCATGCCCTTCTGGCTTACACACTGGGTGTGAAACAACTAATTGTCGGTGTTAACAAAATG
GATTCCACTGAGCCACCCTACAGCCAGAAGA 4 (0.000104%)

ACCCGCTATGGGGCCTCCCTCCGGAAAATGGTGAAGAAAATTGAAATCAGCCAGCACGCCAAGTACAC
TTGCTCTTTCTGTGGCAAAACCAAGATGAAGA 3 (0.000078%)

ACCGAAGCAAGAATCGCAAAAGGCATTTCAATGCACCTTCCCACATTCGAAGGAAGATTATGTCTTCCC
CTCTTTCCAAAGAGCTGAGACAGAAGTACAA 3 (0.000078%)

ACCTATTCACCCTCCACTTCCCGTCTCAGAATCTAAACGTGGTCACCTTCGAGTAGAGAGGCCCGCCCG
CCCACCGTGGGCAGTGCCACCCGCAGATGAC 4 (0.000104%)

ACCTCAAAACAAATGATAACCATACACAACACTAAAGGACGAACCTGATCTCTTATACTAGTATCCTTA
ATCATTTTTATTGCCACAACTAACCTCCTCG 3 (0.000078%)

ACCTCCCTCACCAAAGCCCATAAAAATAAAAAATTATAACAAACCCTGAGAACCAAAATGAACGAAAA
TCTGTTCGCTTCATTCATTGCCCCCACAATCC 683 (0.017734%)

ACGAGAACATGCCTCTCGCAAAGGATCTCCTTCATCCCTCTCCAGAAGAGGAGAAGAGGAAACACAAG
AAGAAACGCCTGGTGCAGAGCCCCAATTCCTA 18 (0.000467%)

ACGCAAGCATGGTTAACGTCCCTAAAACCCGCCGGACTTTCTGTAAGAAGTGTGGCAAGCACCAACCCC
ATAAAGTGACACAGTACAAGAAGGGCAAGGA 9 (0.000234%)

ACGCACACGAGAACATGCCTCTCGCAAAGGATCTCCTTCATCCCTCTCCAGAAGAGGAGAAGAGGAAA
CACAAGAAGAAACGCCTGGTGCAGAGCCCCAA 21 (0.000545%)

ACGCCATGAAGGCCTCGGGCACGCTACGAGAGTACAAGGTAGTGGGTCGCTGCCTGCCCACCCCCAAA
TGCCACACGCCGCCCCTCTACCGCATGCGAAT 3 (0.000078%)

ACGCCTGAACGCAGGCACATACTTCCTATTCTACACCCTAGTAGGCTCCCTTCCCCTACTCATCGCACTG
ATTTACACTCACAACACCCTAGGCTCACTA 515 (0.013372%)

ACGGAAGTAAAATCTGTCGAAATGCACCATGAAGCTTTGAGTGAAGCTCTTCCTGGGGACAATGTGGGC
TTCAATGTCAAGAATGTGTCTGTCAAGGATG 7 (0.000182%)

ACGGGACCCGCTATGGGGCCTCCCTCCGGAAAATGGTGAAGAAAATTGAAATCAGCCAGCACGCCAAG

TACACTTGCTCTTTCTGTGGCAAAACCAAGAT 598 (0.015527%)

ACGGGATAATCCTATTTATTACCTCAGAAGTTTTTTCTTCGCAGGATTTTTCTGAGCCTTTTACCACTCC
AGCCTAGCCCCTACCCCCCAATTAGGAGG 3 (0.000078%)

ACGTTATCGTCACAGCCCATGCATTTGTAATAATCTTCTTCATAGTAATACCCATCATAATCGGAGGCTT
TGGCAACTGACTAGTTCCCCTAATAATCGG 3 (0.000078%)

ACTAAAGGACGAACCTGATCTCTTATACTAGTATCCTTAATCATTTTTATTGCCACAACTAACCTCCTCG
GACTCCTGCCTCACTCATTTACACCAACCA 7 (0.000182%)

ACTACCCCTAAAAGCCAAAATGGGAAAGGAAAAGACTCATATCAACATTGTCGTCATTGGACACGTAG
ATTCGGGCAAGTCCACCACTACTGGCCATCTG 26 (0.000675%)

ACTGAGCGTGCCTACCAAAAGCAGCCGACCATCTTTCAAAACAAGAAGAGGGTCCTGCTGGGAGAAAC
TGGCAAGGAGAAGCTCCCGCGGTACTACAAGA 11 (0.000286%)

ACTGCTAGGAAGCTCCGTAGTCACCGACGAGACCAGAAGTGGCATGATAAACAGTATAAGAAAGCTCA
TTTGGGCACAGCCCTAAAGGCCAACCCTTTTG 5 (0.000130%)

AGAAAAAGGGCCGTTCTGCCATCAACGAAGTGGTAACCCGAGAATACACCATCAACATTCACAAGCGC
ATCCATGGAGTGGGCTTCAAGAAGCGTGCACC 10 (0.000260%)

AGAAAGATGAGGCAGAGGTCCAAGTAAACCGCTAGCTTGTTGCACCGTGGAGGCCACAGGAGCAGAAA
CATGGAATGCCAGACGCTGGGGATGCTGGTAC 6 (0.000156%)

AGAAAGCTCAGAAGGCTAAATGAATATTATCCCTAATACCTGCCACCCCACTCTTAATCAGTGGTGGAA
GAACGGTCTCAGAACTGTTTGTTTCAATTGG 9 (0.000234%)

AGAAAGTTCTCCGCTCCCAGACATGGGTCCCTCGGCTTCCTGCCTCGGAAGCGCAGCAGCAGGCATCGT
GGGAAGGTGAAGAGCTTCCCTAAGGATGACC 21 (0.000545%)

AGAACACAGGTGTCGTGAAAACTACCCCTAAAAGCCAAAATGGGAAAGGAAAAGACTCATATCAACAT
TGTCGTCATTGGACACGTAGATTCGGGCAAGT 183 (0.004752%)

AGAACATGCCTCTCGCAAAGGATCTCCTTCATCCCTCTCCAGAAGAGGAGAAGAGGAAACACAAGAAG
AAACGCCTGGTGCAGAGCCCCAATTCCTACTT 18 (0.000467%)

AGAAGAAAAAGGGCCGTTCTGCCATCAACGAAGTGGTAACCCGAGAATACACCATCAACATTCACAAG
CGCATCCATGGAGTGGGCTTCAAGAAGCGTGC 4 (0.000104%)

AGAAGAAAGATGAGGCAGAGGTCCAAGTAAACCGCTAGCTTGTTGCACCGTGGAGGCCACAGGAGCAG
AAACATGGAATGCCAGACGCTGGGGATGCTGG 3 (0.000078%)

AGAAGGCTAAATGAATATTATCCCTAATACCTGCCACCCCACTCTTAATCAGTGGTGGAAGAACGGTCT
CAGAACTGTTTGTTTCAATTGGCCATTTAAG 10 (0.000260%)

AGAAGGGTGGCGAGAAGAAAAAGGGCCGTTCTGCCATCAACGAAGTGGTAACCCGAGAATACACCATC
AACATTCACAAGCGCATCCATGGAGTGGGCTT 4 (0.000104%)

AGAAGGTGATGGTGAGGAAGAGGATGGAGATGAAGATGAGGAAGCTGAGTCAGCTACGGGCAAGCGG
GCAGCTGAAGATGATGAGGATGACGATGTCGAT 23 (0.000597%)

AGAATGTGTCTGTCAAGGATGTTCGTCGTGGCAACGTTGCTGGTGACAGCAAAAATGACCCACCAATGG
AAGCAGCTGGCTTCACTGCTCAGGTGATTAT 16 (0.000415%)

AGACCCGAGAGCATGCCCTTCTGGCTTACACACTGGGTGTGAAACAACTAATTGTCGGTGTTAACAAAA
TGGATTCCACTGAGCCACCCTACAGCCAGAA 4 (0.000104%)

AGACGCCGCCGCGATGCGCTACGTCGCCTCCTACCTGCTGGCTGCCCTAGGGGGCAACTCCTCCCCCAG
CGCCAAGGACATCAAGAAGATCTTGGACAGC 4 (0.000104%)

AGAGCACGCCATGAAGGCCTCGGGCACGCTACGAGAGTACAAGGTAGTGGGTCGCTGCCTGCCCACCC
CCAAATGCCACACGCCGCCCCTCTACCGCATG 4 (0.000104%)

AGAGCATGCCCTTCTGGCTTACACACTGGGTGTGAAACAACTAATTGTCGGTGTTAACAAAATGGATTC
CACTGAGCCACCCTACAGCCAGAAGAGATAT 24 (0.000623%)

AGAGGATGGAGATGAAGATGAGGAAGCTGAGTCAGCTACGGGCAAGCGGGCAGCTGAAGATGATGAG
GATGACGATGTCGATACCAAGAAGCAGAAGACC 23 (0.000597%)

AGAGGTCCAAGTAAACCGCTAGCTTGTTGCACCGTGGAGGCCACAGGAGCAGAAACATGGAATGCCAG
ACGCTGGGGATGCTGGTACAAGTTGTGGGACT9 (0.000234%)

AGAGTACAAGGTAGTGGGTCGCTGCCTGCCCACCCCCAAATGCCACACGCCGCCCCTCTACCGCATGCG
AATCTTTGCGCCTAATCATGTCGTCGCCAAG 4 (0.000104%)

AGATATGCTCATGTGGTGTTGAGGAAAGCAGACATTGACCTCACCAAGAGGGCGGGAGAACTCACTGA
GGATGAGGTGGAACGTGTGATCACCATTATGC5 (0.000130%)

AGATGAGGAAGCTGAGTCAGCTACGGGCAAGCGGGCAGCTGAAGATGATGAGGATGACGATGTCGATA
CCAAGAAGCAGAAGACCGACGAGGATGACTAG 32 (0.000831%)

AGATGAGGCAGAGGTCCAAGTAAACCGCTAGCTTGTTGCACCGTGGAGGCCACAGGAGCAGAAACATG
GAATGCCAGACGCTGGGGATGCTGGTACAAGT 7 (0.000182%)

AGATGGCCCTAAATTCTTGAAGTCTGGTGATGCTGCCATTGTTGATATGGTTCCTGGCAAGCCCATGTGT
GTTGAGAGCTTCTCAGACTATCCACCTTTG 11 (0.000286%)

AGATGGGAAAGGGCTCCTTCAAGTATGCCTGGGTCTTGGATAAACTGAAAGCTGAGCGTGAACGTGGT
ATCACCATTGATATCTCCTTGTGGAAATTTGA 11 (0.000286%)

AGCACGCCATGAAGGCCTCGGGCACGCTACGAGAGTACAAGGTAGTGGGTCGCTGCCTGCCCACCCCC
AAATGCCACACGCCGCCCCTCTACCGCATGCG 7 (0.000182%)

AGCAGCCGACCATCTTTCAAAACAAGAAGAGGGTCCTGCTGGGAGAAACTGGCAAGGAGAAGCTCCCG
CGGTACTACAAGAACATCGGTCTGGGCTTCAA 4 (0.000104%)

AGCAGCTGGCTTCACTGCTCAGGTGATTATCCTGAACCATCCAGGCCAAATAAGCGCCGGCTATGCCCC
TGTATTGGATTGCCACACGGCTCACATTGCA 21 (0.000545%)

AGCATGCCCTTCTGGCTTACACACTGGGTGTGAAACAACTAATTGTCGGTGTTAACAAAATGGATTCCA
CTGAGCCACCCTACAGCCAGAAGAGATATGA 9 (0.000234%)

AGCATGGTTAACGTCCCTAAAACCCGCCGGACTTTCTGTAAGAAGTGTGGCAAGCACCAACCCCATAAA
GTGACACAGTACAAGAAGGGCAAGGATTCTC 3 (0.000078%)

AGCCAAAATGGGAAAGGAAAAGACTCATATCAACATTGTCGTCATTGGACACGTAGATTCGGGCAAGT
CCACCACTACTGGCCATCTGATCTATAAATGC 4 (0.000104%)

AGCCCATAAAAATAAAAAATTATAACAAACCCTGAGAACCAAAATGAACGAAAATCTGTTCGCTTCAT
TCATTGCCCCCACAATCCTAGGCCTACCCGCC 9 (0.000234%)

AGCCCATGACCCCTAACAGGGGCCCTCTCAGCCCTCCTAATGACCTCCGGCCTAGCCATGTGATTTCACT
TCCACTCCATAACGCTCCTCATACTAGGCC 29 (0.000753%)

AGCCCATGCATTTGTAATAATCTTCTTCATAGTAATACCCATCATAATCGGAGGCTTTGGCAACTGACTA
GTTCCCCTAATAATCGGTGCCCCCGATATG 121 (0.003142%)

AGCCCTCCTAATGACCTCCGGCCTAGCCATGTGATTTCACTTCCACTCCATAACGCTCCTCATACTAGGC
CTACTAACCAACACACTAACCATATACCAA 3 (0.000078%)

AGCCCTTGCGCCTGCCTCTCCAGGATGTCTACAAAATTGGTGGTATTGGTACTGTTCCTGTTGGCCGAGT
GGAGACTGGTGTTCTCAAACCCGGTATGGT 579 (0.015034%)

AGCCGACCATCTTTCAAAACAAGAAGAGGGTCCTGCTGGGAGAAACTGGCAAGGAGAAGCTCCCGCGG
TACTACAAGAACATCGGTCTGGGCTTCAAGAC 4 (0.000104%)

AGCCGCCTCCGCCGCGCGCCTCCTCCGCCGCCGCGGACTCCGGCAGCTTTATCGCCAGAGTCCCTGAAC
TCTCGCTTTCTTTTTAATCCCCTGCATCGGA 3 (0.000078%)

AGCGTGCCTACCAAAAGCAGCCGACCATCTTTCAAAACAAGAAGAGGGTCCTGCTGGGAGAAACTGGC
AAGGAGAAGCTCCCGCGGTACTACAAGAACAT 15 (0.000389%)

AGCTAGGAGTGGGAATAGCTTTGCGAAAAATGGGCGCAATGGCCAAGCCAGATTGTATCATCACTTGT
GATGGTAAAAACCTCACCATAAAAACTGAGAG 5 (0.000130%)

AGCTCAGAAGGCTAAATGAATATTATCCCTAATACCTGCCACCCCACTCTTAATCAGTGGTGGAAGAAC
GGTCTCAGAACTGTTTGTTTCAATTGGCCAT 14 (0.000364%)

AGCTGAGTCAGCTACGGGCAAGCGGGCAGCTGAAGATGATGAGGATGACGATGTCGATACCAAGAAGC
AGAAGACCGACGAGGATGACTAGACAGCAAAA 48 (0.001246%)

AGCTGGAAGATGGCCCTAAATTCTTGAAGTCTGGTGATGCTGCCATTGTTGATATGGTTCCTGGCAAGC
CCATGTGTGTTGAGAGCTTCTCAGACTATCC 9 (0.000234%)

AGCTGGCAAGGTCACCAAGTCTGCCCAGAAAGCTCAGAAGGCTAAATGAATATTATCCCTAATACCTGC
CACCCCACTCTTAATCAGTGGTGGAAGAACG 7 (0.000182%)

AGCTGGCTTCACTGCTCAGGTGATTATCCTGAACCATCCAGGCCAAATAAGCGCCGGCTATGCCCCTGT
ATTGGATTGCCACACGGCTCACATTGCATGC 7 (0.000182%)

AGCTTGTTGCACCGTGGAGGCCACAGGAGCAGAAACATGGAATGCCAGACGCTGGGGATGCTGGTACA
AGTTGTGGGACTGCATGCTACTGTCTAGAGCT 4 (0.000104%)

AGCTTTTTGATGACTTCTAGCAAGCCTCGCTAACCTCGCCTTACCCCCCACTATTAACCTACTGGGAGAA
CTCTCTGTGCTAGTAACCACGTTCTCCTGA 5 (0.000130%)

AGGAAACAAATGGTCATTGATGTCCTTCACCCCGGGAAGGCGACAGTGCCTAAGACAGAAATTCGGGA
AAAACTAGCCAAAATGTACAAGACCACACCGG 5 (0.000130%)

AGGAAGAGGATGGAGATGAAGATGAGGAAGCTGAGTCAGCTACGGGCAAGCGGGCAGCTGAAGATGA
TGAGGATGACGATGTCGATACCAAGAAGCAGAA 6 (0.000156%)

AGGAAGCTCCGTAGTCACCGACGAGACCAGAAGTGGCATGATAAACAGTATAAGAAAGCTCATTTGGG
CACAGCCCTAAAGGCCAACCCTTTTGGAGGTG 4 (0.000104%)

AGGAAGCTGAGTCAGCTACGGGCAAGCGGGCAGCTGAAGATGATGAGGATGACGATGTCGATACCAAG
AAGCAGAAGACCGACGAGGATGACTAGACAGC 5 (0.000130%)

AGGACTTCAAACTCTACTCCCACTAATAGCTTTTTGATGACTTCTAGCAAGCCTCGCTAACCTCGCCTTA
CCCCCCACTATTAACCTACTGGGAGAACTC 7 (0.000182%)

AGGATCTCCTTCATCCCTCTCCAGAAGAGGAGAAGAGGAAACACAAGAAGAAACGCCTGGTGCAGAGC

CCCAATTCCTACTTCATGGATGTGAAATGCCC 4 (0.000104%)

AGGATGGAGATGAAGATGAGGAAGCTGAGTCAGCTACGGGCAAGCGGGCAGCTGAAGATGATGAGGA
TGACGATGTCGATACCAAGAAGCAGAAGACCGA 3 (0.000078%)

AGGATGTTCGTCGTGGCAACGTTGCTGGTGACAGCAAAAATGACCCACCAATGGAAGCAGCTGGCTTC
ACTGCTCAGGTGATTATCCTGAACCATCCAGG 9 (0.000234%)

AGGCAGAGGTCCAAGTAAACCGCTAGCTTGTTGCACCGTGGAGGCCACAGGAGCAGAAACATGGAATG
CCAGACGCTGGGGATGCTGGTACAAGTTGTGG5 (0.000130%)

AGGCCTCGGGCACGCTACGAGAGTACAAGGTAGTGGGTCGCTGCCTGCCCACCCCCAAATGCCACACG
CCGCCCCTCTACCGCATGCGAATCTTTGCGCC 3 (0.000078%)

AGGCTGCTGAGATGGGAAAGGGCTCCTTCAAGTATGCCTGGGTCTTGGATAAACTGAAAGCTGAGCGT
GAACGTGGTATCACCATTGATATCTCCTTGTG 3 (0.000078%)

AGGCTGCTGGAGCTGGCAAGGTCACCAAGTCTGCCCAGAAAGCTCAGAAGGCTAAATGAATATTATCC
CTAATACCTGCCACCCCACTCTTAATCAGTGG 29 (0.000753%)

AGGGCCGTTCTGCCATCAACGAAGTGGTAACCCGAGAATACACCATCAACATTCACAAGCGCATCCATG
GAGTGGGCTTCAAGAAGCGTGCACCTCGGGC 4 (0.000104%)

AGGGGCCCTCTCAGCCCTCCTAATGACCTCCGGCCTAGCCATGTGATTTCACTTCCACTCCATAACGCTC
CTCATACTAGGCCTACTAACCAACACACTA 7 (0.000182%)

AGGTATGGTCTGAGCTATGATATCAATTGGCTTCCTAGGGTTTATCGTGTGAGCACACCATATATTTACA
GTAGGAATAGACGTAGACACACGAGCATAT 3 (0.000078%)

AGGTCACCAAGTCTGCCCAGAAAGCTCAGAAGGCTAAATGAATATTATCCCTAATACCTGCCACCCCAC
TCTTAATCAGTGGTGGAAGAACGGTCTCAGA 4 (0.000104%)

AGGTGATGGTGAGGAAGAGGATGGAGATGAAGATGAGGAAGCTGAGTCAGCTACGGGCAAGCGGGCA
GCTGAAGATGATGAGGATGACGATGTCGATACC 18 (0.000467%)

AGGTGATTATCCTGAACCATCCAGGCCAAATAAGCGCCGGCTATGCCCCTGTATTGGATTGCCACACGG
CTCACATTGCATGCAAGTTTGCTGAGCTGAA 3 (0.000078%)

AGGTGTCGTGAAAACTACCCCTAAAAGCCAAAATGGGAAAGGAAAAGACTCATATCAACATTGTCGTC
ATTGGACACGTAGATTCGGGCAAGTCCACCAC7 (0.000182%)

AGTAAAACCCAGCCCATGACCCCTAACAGGGGCCCTCTCAGCCCTCCTAATGACCTCCGGCCTAGCCAT
GTGATTTCACTTCCACTCCATAACGCTCCTC 23 (0.000597%)

AGTAAACCGCTAGCTTGTTGCACCGTGGAGGCCACAGGAGCAGAAACATGGAATGCCAGACGCTGGGG
ATGCTGGTACAAGTTGTGGGACTGCATGCTAC 10 (0.000260%)

AGTCAACGTTACAACGGAAGTAAAATCTGTCGAAATGCACCATGAAGCTTTGAGTGAAGCTCTTCCTGG
GGACAATGTGGGCTTCAATGTCAAGAATGTG 8 (0.000208%)

AGTCTGCCCAGAAAGCTCAGAAGGCTAAATGAATATTATCCCTAATACCTGCCACCCCACTCTTAATCA
GTGGTGGAAGAACGGTCTCAGAACTGTTTGT 4 (0.000104%)

AGTCTGGTGATGCTGCCATTGTTGATATGGTTCCTGGCAAGCCCATGTGTGTTGAGAGCTTCTCAGACTA
TCCACCTTTGGGTCGCTTTGCTGTTCGTGA18 (0.000467%)

AGTTGGGCCGCTTGGTCAAGGACATGAAGATCAAGTCCCTGGAGGAGATCTATCTCTTCTCCCTGCCCA
TTAAGGAATCAGAGATCATTGATTTCTTCCT 15 (0.000389%)

AGTTTAATCCCTTTGTGACTTCCGACCGAAGCAAGAATCGCAAAAGGCATTTCAATGCACCTTCCCACA
TTCGAAGGAAGATTATGTCTTCCCCTCTTTC 5 (0.000130%)

ATAAAAAATTATAACAAACCCTGAGAACCAAAATGAACGAAAATCTGTTCGCTTCATTCATTGCCCCCA
CAATCCTAGGCCTACCCGCCGCAGTACTGAT 8 (0.000208%)

ATAAAAATAAAAAATTATAACAAACCCTGAGAACCAAAATGAACGAAAATCTGTTCGCTTCATTCATTG
CCCCCACAATCCTAGGCCTACCCGCCGCAGT 11 (0.000286%)

ATAACAAACCCTGAGAACCAAAATGAACGAAAATCTGTTCGCTTCATTCATTGCCCCCACAATCCTAGG
CCTACCCGCCGCAGTACTGATCATTCTATTT 18 (0.000467%)

ATACACAACACTAAAGGACGAACCTGATCTCTTATACTAGTATCCTTAATCATTTTTATTGCCACAACTA
ACCTCCTCGGACTCCTGCCTCACTCATTTA17 (0.000441%)

ATACATAGGTATGGTCTGAGCTATGATATCAATTGGCTTCCTAGGGTTTATCGTGTGAGCACACCATATA
TTTACAGTAGGAATAGACGTAGACACACGA 3 (0.000078%)

ATACCTGCCACCCCACTCTTAATCAGTGGTGGAAGAACGGTCTCAGAACTGTTTGTTTCAATTGGCCATT
TAAGTTTAGTAGTAAAAGACTGGTTAATGA 3 (0.000078%)

ATACGGGATAATCCTATTTATTACCTCAGAAGTTTTTTTCTTCGCAGGATTTTTCTGAGCCTTTTACCACT
CCAGCCTAGCCCCTACCCCCCAATTAGGA 4 (0.000104%)

ATAGCTTTTTGATGACTTCTAGCAAGCCTCGCTAACCTCGCCTTACCCCCCACTATTAACCTACTGGGAG
AACTCTCTGTGCTAGTAACCACGTTCTCCT 3 (0.000078%)

ATAGTAAAACCCAGCCCATGACCCCTAACAGGGGCCCTCTCAGCCCTCCTAATGACCTCCGGCCTAGCC
ATGTGATTTCACTTCCACTCCATAACGCTCC 14 (0.000364%)

ATATGCTCATGTGGTGTTGAGGAAAGCAGACATTGACCTCACCAAGAGGGCGGGAGAACTCACTGAGG
ATGAGGTGGAACGTGTGATCACCATTATGCAG4 (0.000104%)

ATCAACGAAGTGGTAACCCGAGAATACACCATCAACATTCACAAGCGCATCCATGGAGTGGGCTTCAA
GAAGCGTGCACCTCGGGCACTCAAAGAGATTC 5 (0.000130%)

ATCACCCTATAGAAGAACTAATGTTAGTATAAGTAACATGAAAACATTCTCCTCCGCATAAGCCTGCGT
CAGATTAAAACACTGAACTGACAATTAACAG 5 (0.000130%)

ATCACCTTCCACCCTTACTACACAATCAAAGACGCCCTCGGCTTACTTCTCTTCATTCTCTCCTTAATGAC
ATTAACACTATTCTCACCAGACCTCCTAG 278 (0.007218%)

ATCCAACATCTCCGCATGATGAAACTTCGGCTCACTCCTTGGCGCCTGCCTGATCCTCCAAATCACCACA
GGACTATTCCTAGCCATGCACTACTCACCA 518 (0.013450%)

ATCCCTAATACCTGCCACCCCACTCTTAATCAGTGGTGGAAGAACGGTCTCAGAACTGTTTGTTTCAATT
GGCCATTTAAGTTTAGTAGTAAAAGACTGG 11 (0.000286%)

ATCCCTTTGTGACTTCCGACCGAAGCAAGAATCGCAAAAGGCATTTCAATGCACCTTCCCACATTCGAA
GGAAGATTATGTCTTCCCCTCTTTCCAAAGA 3 (0.000078%)

ATCCGGGTTCATCCGACACCAGCCGCCTCCACCATGCCGCCGAAGTTCGACCCCAACGAGATCAAAGTC
GTATACCTGAGGTGCACCGGAGGTGAAGTCG 3 (0.000078%)

ATCCGTCCTAATCACAGCAGTCCTACTTCTCCTATCTCTCCCAGTCCTAGCTGCTGGCATCACTATACTA
CTAACAGACCGCAACCTCAACACCACCTTC 7 (0.000182%)

ATCGCCGTTCTGGTAAAAAGCTGGAAGATGGCCCTAAATTCTTGAAGTCTGGTGATGCTGCCATTGTTG
ATATGGTTCCTGGCAAGCCCATGTGTGTTGA 42 (0.001091%)

ATCGGCGCACTGCGAGCAGTAGCCCAAACAATCTCATATGAAGTCACCCTAGCCATCATTCTACTATCA
ACATTACTAATAAGTGGCTCCTTTAACCTCT 6 (0.000156%)

ATCTACAACGTTATCGTCACAGCCCATGCATTTGTAATAATCTTCTTCATAGTAATACCCATCATAATCG
GAGGCTTTGGCAACTGACTAGTTCCCCTAA 3 (0.000078%)

ATCTCCGCATGATGAAACTTCGGCTCACTCCTTGGCGCCTGCCTGATCCTCCAAATCACCACAGGACTAT
TCCTAGCCATGCACTACTCACCAGACGCCT 4 (0.000104%)

ATCTCCTTCATCCCTCTCCAGAAGAGGAGAAGAGGAAACACAAGAAGAAACGCCTGGTGCAGAGCCCC
AATTCCTACTTCATGGATGTGAAATGCCCAGG 5 (0.000130%)

ATCTGTCGAAATGCACCATGAAGCTTTGAGTGAAGCTCTTCCTGGGGACAATGTGGGCTTCAATGTCAA
GAATGTGTCTGTCAAGGATGTTCGTCGTGGC 22 (0.000571%)

ATGAAACTTCGGCTCACTCCTTGGCGCCTGCCTGATCCTCCAAATCACCACAGGACTATTCCTAGCCATG
CACTACTCACCAGACGCCTCAACCGCCTTT 3 (0.000078%)

ATGAAGATGAGGAAGCTGAGTCAGCTACGGGCAAGCGGGCAGCTGAAGATGATGAGGATGACGATGTC
GATACCAAGAAGCAGAAGACCGACGAGGATGA 24 (0.000623%)

ATGAAGGCCTCGGGCACGCTACGAGAGTACAAGGTAGTGGGTCGCTGCCTGCCCACCCCCAAATGCCA
CACGCCGCCCCTCTACCGCATGCGAATCTTTG 15 (0.000389%)

ATGAAGTTTAATCCCTTTGTGACTTCCGACCGAAGCAAGAATCGCAAAAGGCATTTCAATGCACCTTCC
CACATTCGAAGGAAGATTATGTCTTCCCCTC 602 (0.015631%)

ATGAATCCTGTGGAGCATCCTTTTGGAGGTGGCAACCACCAGCACATCGGCAAGCCCTCCACCATCCGC
AGAGATGCCCCTGCTGGCCGCAAAGTGGGTC 166 (0.004310%)

ATGACCCCTAACAGGGGCCCTCTCAGCCCTCCTAATGACCTCCGGCCTAGCCATGTGATTTCACTTCCAC
TCCATAACGCTCCTCATACTAGGCCTACTA 39 (0.001013%)

ATGACTTCTAGCAAGCCTCGCTAACCTCGCCTTACCCCCCACTATTAACCTACTGGGAGAACTCTCTGTG
CTAGTAACCACGTTCTCCTGATCAAATATC 3 (0.000078%)

ATGAGGCAGAGGTCCAAGTAAACCGCTAGCTTGTTGCACCGTGGAGGCCACAGGAGCAGAAACATGGA
ATGCCAGACGCTGGGGATGCTGGTACAAGTTG4 (0.000104%)

ATGATAACCATACACAACACTAAAGGACGAACCTGATCTCTTATACTAGTATCCTTAATCATTTTTATTG
CCACAACTAACCTCCTCGGACTCCTGCCTC 3 (0.000078%)

ATGCATTTGTAATAATCTTCTTCATAGTAATACCCATCATAATCGGAGGCTTTGGCAACTGACTAGTTCC
CCTAATAATCGGTGCCCCCGATATGGCGTT 3 (0.000078%)

ATGCCCGTCACCAAGTTGGGCCGCTTGGTCAAGGACATGAAGATCAAGTCCCTGGAGGAGATCTATCTC
TTCTCCCTGCCCATTAAGGAATCAGAGATCA 19 (0.000493%)

ATGCCCTTCTGGCTTACACACTGGGTGTGAAACAACTAATTGTCGGTGTTAACAAAATGGATTCCACTG
AGCCACCCTACAGCCAGAAGAGATATGAGGA 4 (0.000104%)

ATGCCTCTCGCAAAGGATCTCCTTCATCCCTCTCCAGAAGAGGAGAAGAGGAAACACAAGAAGAAACG
CCTGGTGCAGAGCCCCAATTCCTACTTCATGG 6 (0.000156%)

ATGCTCATGTGGTGTTGAGGAAAGCAGACATTGACCTCACCAAGAGGGCGGGAGAACTCACTGAGGAT

GAGGTGGAACGTGTGATCACCATTATGCAGAA 7 (0.000182%)

ATGGAAGCAGCTGGCTTCACTGCTCAGGTGATTATCCTGAACCATCCAGGCCAAATAAGCGCCGGCTAT
GCCCCTGTATTGGATTGCCACACGGCTCACA 14 (0.000364%)

ATGGAGATGAAGATGAGGAAGCTGAGTCAGCTACGGGCAAGCGGGCAGCTGAAGATGATGAGGATGA
CGATGTCGATACCAAGAAGCAGAAGACCGACGA 9 (0.000234%)

ATGGCCCTAAATTCTTGAAGTCTGGTGATGCTGCCATTGTTGATATGGTTCCTGGCAAGCCCATGTGTGT
TGAGAGCTTCTCAGACTATCCACCTTTGGG 6 (0.000156%)

ATGGGAAAGGAAAAGACTCATATCAACATTGTCGTCATTGGACACGTAGATTCGGGCAAGTCCACCACT
ACTGGCCATCTGATCTATAAATGCGGTGGCA 7 (0.000182%)

ATGGGAAAGGGCTCCTTCAAGTATGCCTGGGTCTTGGATAAACTGAAAGCTGAGCGTGAACGTGGTATC
ACCATTGATATCTCCTTGTGGAAATTTGAGA 12 (0.000312%)

ATGGTCTGAGCTATGATATCAATTGGCTTCCTAGGGTTTATCGTGTGAGCACACCATATATTTACAGTAG
GAATAGACGTAGACACACGAGCATATTTCA 3 (0.000078%)

ATGGTGAGGAAGAGGATGGAGATGAAGATGAGGAAGCTGAGTCAGCTACGGGCAAGCGGGCAGCTGA
AGATGATGAGGATGACGATGTCGATACCAAGAA 5 (0.000130%)

ATGTCAAGAATGTGTCTGTCAAGGATGTTCGTCGTGGCAACGTTGCTGGTGACAGCAAAAATGACCCAC
CAATGGAAGCAGCTGGCTTCACTGCTCAGGT 4 (0.000104%)

ATGTTCGTCGTGGCAACGTTGCTGGTGACAGCAAAAATGACCCACCAATGGAAGCAGCTGGCTTCACTG
CTCAGGTGATTATCCTGAACCATCCAGGCCA 4 (0.000104%)

ATTATAACAAACCCTGAGAACCAAAATGAACGAAAATCTGTTCGCTTCATTCATTGCCCCCACAATCCT
AGGCCTACCCGCCGCAGTACTGATCATTCTA 59 (0.001532%)

ATTATCCCTAATACCTGCCACCCCACTCTTAATCAGTGGTGGAAGAACGGTCTCAGAACTGTTTGTTTCA
ATTGGCCATTTAAGTTTAGTAGTAAAAGAC 32 (0.000831%)

ATTATCCTGAACCATCCAGGCCAAATAAGCGCCGGCTATGCCCCTGTATTGGATTGCCACACGGCTCAC
ATTGCATGCAAGTTTGCTGAGCTGAAGGAAA 23 (0.000597%)

ATTCAGACTGAGCGTGCCTACCAAAAGCAGCCGACCATCTTTCAAAACAAGAAGAGGGTCCTGCTGGG
AGAAACTGGCAAGGAGAAGCTCCCGCGGTACT 55 (0.001428%)

ATTCTTGAAGTCTGGTGATGCTGCCATTGTTGATATGGTTCCTGGCAAGCCCATGTGTGTTGAGAGCTTC
TCAGACTATCCACCTTTGGGTCGCTTTGCT17 (0.000441%)

ATTGATCGCCGTTCTGGTAAAAAGCTGGAAGATGGCCCTAAATTCTTGAAGTCTGGTGATGCTGCCATT
GTTGATATGGTTCCTGGCAAGCCCATGTGTG 1011 (0.026250%)

ATTTGGATACATAGGTATGGTCTGAGCTATGATATCAATTGGCTTCCTAGGGTTTATCGTGTGAGCACAC
CATATATTTACAGTAGGAATAGACGTAGAC 31 (0.000805%)

ATTTGTAATAATCTTCTTCATAGTAATACCCATCATAATCGGAGGCTTTGGCAACTGACTAGTTCCCCTA
ATAATCGGTGCCCCCGATATGGCGTTTCCC 6 (0.000156%)

CAAAAATGACCCACCAATGGAAGCAGCTGGCTTCACTGCTCAGGTGATTATCCTGAACCATCCAGGCCA
AATAAGCGCCGGCTATGCCCCTGTATTGGAT 114 (0.002960%)

CAAAACAAATGATAACCATACACAACACTAAAGGACGAACCTGATCTCTTATACTAGTATCCTTAATCA
TTTTTATTGCCACAACTAACCTCCTCGGACT 8 (0.000208%)

CAAAACAAGAAGAGGGTCCTGCTGGGAGAAACTGGCAAGGAGAAGCTCCCGCGGTACTACAAGAACA TCGGTCTGGGCTTCAAGACACCCAAGGAGGCTA 4 (0.000104%)

CAAAAGAAGAAAGATGAGGCAGAGGTCCAAGTAAACCGCTAGCTTGTTGCACCGTGGAGGCCACAGGA GCAGAAACATGGAATGCCAGACGCTGGGGATG 5 (0.000130%)

CAAAAGCAGCCGACCATCTTTCAAAACAAGAAGAGGGTCCTGCTGGGAGAAACTGGCAAGGAGAAGCT CCCGCGGTACTACAAGAACATCGGTCTGGGCT 10 (0.000260%)

CAAAATGGGAAAGGAAAAGACTCATATCAACATTGTCGTCATTGGACACGTAGATTCGGGCAAGTCCA CCACTACTGGCCATCTGATCTATAAATGCGGT 21 (0.000545%)

CAAACCCTGAGAACCAAAATGAACGAAAATCTGTTCGCTTCATTCATTGCCCCCACAATCCTAGGCCTA CCCGCCGCAGTACTGATCATTCTATTTCCCC 34 (0.000883%)

CAAACTCTACTCCCACTAATAGCTTTTTGATGACTTCTAGCAAGCCTCGCTAACCTCGCCTTACCCCCCA CTATTAACCTACTGGGAGAACTCTCTGTGC 5 (0.000130%)

CAAAGAAGGGTGGCGAGAAGAAAAAGGGCCGTTCTGCCATCAACGAAGTGGTAACCCGAGAATACAC CATCAACATTCACAAGCGCATCCATGGAGTGGG 4 (0.000104%)

CAAAGCCCATAAAAATAAAAAATTATAACAAACCCTGAGAACCAAAATGAACGAAAATCTGTTCGCTT CATTCATTGCCCCCACAATCCTAGGCCTACCC 25 (0.000649%)

CAAAGGATCTCCTTCATCCCTCTCCAGAAGAGGAGAAGAGGAAACACAAGAAGAAACGCCTGGTGCAG AGCCCCAATTCCTACTTCATGGATGTGAAATG 8 (0.000208%)

CAAATGATAACCATACACAACACTAAAGGACGAACCTGATCTCTTATACTAGTATCCTTAATCATTTTTA TTGCCACAACTAACCTCCTCGGACTCCTGC 23 (0.000597%)

CAAATGGTCATTGATGTCCTTCACCCCGGGAAGGCGACAGTGCCTAAGACAGAAATTCGGGAAAAACT AGCCAAAATGTACAAGACCACACCGGATGTCA 10 (0.000260%)

CAACAAAACTAACTAATACTAACATCTCAGACGCTCAGGAAATAGAAACCGTCTGAACTATCCTGCCCG CCATCATCCTAGTCCTCATCGCCCTCCCATC 3 (0.000078%)

CAACACTAAAGGACGAACCTGATCTCTTATACTAGTATCCTTAATCATTTTTATTGCCACAACTAACCTC CTCGGACTCCTGCCTCACTCATTTACACCA 73 (0.001895%)

CAACATCTCCGCATGATGAAACTTCGGCTCACTCCTTGGCGCCTGCCTGATCCTCCAAATCACCACAGG ACTATTCCTAGCCATGCACTACTCACCAGAC 35 (0.000909%)

CAACCGACTACTTCAGAGGAAACAAATGGTCATTGATGTCCTTCACCCCGGGAAGGCGACAGTGCCTAA GACAGAAATTCGGGAAAAACTAGCCAAAATG 449 (0.011658%)

CAACGTTACAACGGAAGTAAAATCTGTCGAAATGCACCATGAAGCTTTGAGTGAAGCTCTTCCTGGGGA CAATGTGGGCTTCAATGTCAAGAATGTGTCT 19 (0.000493%)

CAACGTTATCGTCACAGCCCATGCATTTGTAATAATCTTCTTCATAGTAATACCCATCATAATCGGAGGC TTTGGCAACTGACTAGTTCCCCTAATAATC 28 (0.000727%)

CAACGTTGCTGGTGACAGCAAAAATGACCCACCAATGGAAGCAGCTGGCTTCACTGCTCAGGTGATTAT CCTGAACCATCCAGGCCAAATAAGCGCCGGC 21 (0.000545%)

CAACTTCCTTCGGTCGTCCCGAATCCGGGTTCATCCGACACCAGCCGCCTCCACCATGCCGCCGAAGTTC GACCCCAACGAGATCAAAGTCGTATACCTG 15 (0.000389%)

CAAGAATGTGTCTGTCAAGGATGTTCGTCGTGGCAACGTTGCTGGTGACAGCAAAAATGACCCACCAAT
GGAAGCAGCTGGCTTCACTGCTCAGGTGATT 83 (0.002155%)

CAAGCATGGTTAACGTCCCTAAAACCCGCCGGACTTTCTGTAAGAAGTGTGGCAAGCACCAACCCCATA
AAGTGACACAGTACAAGAAGGGCAAGGATTC 7 (0.000182%)

CAAGGACTTCAAACTCTACTCCCACTAATAGCTTTTTGATGACTTCTAGCAAGCCTCGCTAACCTCGCCT
TACCCCCCACTATTAACCTACTGGGAGAAC 7 (0.000182%)

CAAGGATGAGGTTTTGAAGATTATGCCAGTGCAGAAGCAGACCCGTGCCGGCCAGCGCACCAGGTTCA
AGGCATTTGTTGCTATCGGGGACTACAATGGC 18 (0.000467%)

CAAGGATGTTCGTCGTGGCAACGTTGCTGGTGACAGCAAAAATGACCCACCAATGGAAGCAGCTGGCT
TCACTGCTCAGGTGATTATCCTGAACCATCCA 83 (0.002155%)

CAAGGTGCAACTTCCTTCGGTCGTCCCGAATCCGGGTTCATCCGACACCAGCCGCCTCCACCATGCCGC
CGAAGTTCGACCCCAACGAGATCAAAGTCGT 28 (0.000727%)

CAAGTAAACCGCTAGCTTGTTGCACCGTGGAGGCCACAGGAGCAGAAACATGGAATGCCAGACGCTGG
GGATGCTGGTACAAGTTGTGGGACTGCATGCT 11 (0.000286%)

CAAGTCCACCACTACTGGCCATCTGATCTATAAATGCGGTGGCATCGACAAAAGAACCATTGAAAAATT
TGAGAAGGAGGCTGCTGAGATGGGAAAGGGC 7 (0.000182%)

CAAGTCTGCCCAGAAAGCTCAGAAGGCTAAATGAATATTATCCCTAATACCTGCCACCCCACTCTTAAT
CAGTGGTGGAAGAACGGTCTCAGAACTGTTT 27 (0.000701%)

CAAGTTGGGCCGCTTGGTCAAGGACATGAAGATCAAGTCCCTGGAGGAGATCTATCTCTTCTCCCTGCC
CATTAAGGAATCAGAGATCATTGATTTCTTC 21 (0.000545%)

CAATGGAAGCAGCTGGCTTCACTGCTCAGGTGATTATCCTGAACCATCCAGGCCAAATAAGCGCCGGCT
ATGCCCCTGTATTGGATTGCCACACGGCTCA 27 (0.000701%)

CAATGTCAAGAATGTGTCTGTCAAGGATGTTCGTCGTGGCAACGTTGCTGGTGACAGCAAAAATGACCC
ACCAATGGAAGCAGCTGGCTTCACTGCTCAG 9 (0.000234%)

CACAACAAAACTAACTAATACTAACATCTCAGACGCTCAGGAAATAGAAACCGTCTGAACTATCCTGCC
CGCCATCATCCTAGTCCTCATCGCCCTCCCA 13 (0.000338%)

CACAACACTAAAGGACGAACCTGATCTCTTATACTAGTATCCTTAATCATTTTTATTGCCACAACTAACC
TCCTCGGACTCCTGCCTCACTCATTTACAC26 (0.000675%)

CACACGAGAACATGCCTCTCGCAAAGGATCTCCTTCATCCCTCTCCAGAAGAGGAGAAGAGGAAACAC
AAGAAGAAACGCCTGGTGCAGAGCCCCAATTC 12 (0.000312%)

CACAGAAAGTTCTCCGCTCCCAGACATGGGTCCCTCGGCTTCCTGCCTCGGAAGCGCAGCAGCAGGCAT
CGTGGGAAGGTGAAGAGCTTCCCTAAGGATG 7 (0.000182%)

CACAGGTGTCGTGAAAACTACCCCTAAAAGCCAAAATGGGAAAGGAAAAGACTCATATCAACATTGTC
GTCATTGGACACGTAGATTCGGGCAAGTCCAC 182 (0.004726%)

CACATCTACAACGTTATCGTCACAGCCCATGCATTTGTAATAATCTTCTTCATAGTAATACCCATCATAA
TCGGAGGCTTTGGCAACTGACTAGTTCCCC 16 (0.000415%)

CACCAAAGCCCATAAAAATAAAAAATTATAACAAACCCTGAGAACCAAAATGAACGAAAATCTGTTCG
CTTCATTCATTGCCCCCACAATCCTAGGCCTA 10 (0.000260%)

CACCAAGTCTGCCCAGAAAGCTCAGAAGGCTAAATGAATATTATCCCTAATACCTGCCACCCCACTCTT

AATCAGTGGTGGAAGAACGGTCTCAGAACTG 20 (0.000519%)

CACCAAGTTGGGCCGCTTGGTCAAGGACATGAAGATCAAGTCCCTGGAGGAGATCTATCTCTTCTCCCT
GCCCATTAAGGAATCAGAGATCATTGATTTC 18 (0.000467%)

CACCAATGGAAGCAGCTGGCTTCACTGCTCAGGTGATTATCCTGAACCATCCAGGCCAAATAAGCGCCG
GCTATGCCCCTGTATTGGATTGCCACACGGC 109 (0.002830%)

CACCTCATATCCTCCCTACTATGCCTAGAAGGAATAATACTATCGCTGTTCATTATAGCTACTCTCATAA
CCCTCAACACCCACTCCCTCTTAGCCAATA 4 (0.000104%)

CACCTTCCACCCTTACTACACAATCAAAGACGCCCTCGGCTTACTTCTCTTCATTCTCTCCTTAATGACAT
TAACACTATTCTCACCAGACCTCCTAGGC 7 (0.000182%)

CACGAGAACATGCCTCTCGCAAAGGATCTCCTTCATCCCTCTCCAGAAGAGGAGAAGAGGAAACACAA
GAAGAAACGCCTGGTGCAGAGCCCCAATTCCT 18 (0.000467%)

CACGCAAGCATGGTTAACGTCCCTAAAACCCGCCGGACTTTCTGTAAGAAGTGTGGCAAGCACCAACCC
CATAAAGTGACACAGTACAAGAAGGGCAAGG 13 (0.000338%)

CACGCCATGAAGGCCTCGGGCACGCTACGAGAGTACAAGGTAGTGGGTCGCTGCCTGCCCACCCCCAA
ATGCCACACGCCGCCCCTCTACCGCATGCGAA 6 (0.000156%)

CACTAAAGGACGAACCTGATCTCTTATACTAGTATCCTTAATCATTTTTATTGCCACAACTAACCTCCTC
GGACTCCTGCCTCACTCATTTACACCAACC 7 (0.000182%)

CACTAATAGCTTTTTGATGACTTCTAGCAAGCCTCGCTAACCTCGCCTTACCCCCCACTATTAACCTACT
GGGAGAACTCTCTGTGCTAGTAACCACGTT 5 (0.000130%)

CACTCACAACAAAACTAACTAATACTAACATCTCAGACGCTCAGGAAATAGAAACCGTCTGAACTATCC
TGCCCGCCATCATCCTAGTCCTCATCGCCCT 5 (0.000130%)

CACTCCTTGGCGCCTGCCTGATCCTCCAAATCACCACAGGACTATTCCTAGCCATGCACTACTCACCAGA
CGCCTCAACCGCCTTTTCATCAATCGCCCA 3 (0.000078%)

CACTGCTCAGGTGATTATCCTGAACCATCCAGGCCAAATAAGCGCCGGCTATGCCCCTGTATTGGATTG
CCACACGGCTCACATTGCATGCAAGTTTGCT 8 (0.000208%)

CAGAAAGCTCAGAAGGCTAAATGAATATTATCCCTAATACCTGCCACCCCACTCTTAATCAGTGGTGGA
AGAACGGTCTCAGAACTGTTTGTTTCAATTG 19 (0.000493%)

CAGAAAGTTCTCCGCTCCCAGACATGGGTCCCTCGGCTTCCTGCCTCGGAAGCGCAGCAGCAGGCATCG
TGGGAAGGTGAAGAGCTTCCCTAAGGATGAC 19 (0.000493%)

CAGAACACAGGTGTCGTGAAAACTACCCCTAAAAGCCAAAATGGGAAAGGAAAAGACTCATATCAACA
TTGTCGTCATTGGACACGTAGATTCGGGCAAG 199 (0.005167%)

CAGAAGGCTAAATGAATATTATCCCTAATACCTGCCACCCCACTCTTAATCAGTGGTGGAAGAACGGTC
TCAGAACTGTTTGTTTCAATTGGCCATTTAA 36 (0.000935%)

CAGACATGGGTCCCTCGGCTTCCTGCCTCGGAAGCGCAGCAGCAGGCATCGTGGGAAGGTGAAGAGCT
TCCCTAAGGATGACCCGTCCAAGCCGGTCCAC 7 (0.000182%)

CAGACCCGAGAGCATGCCCTTCTGGCTTACACACTGGGTGTGAAACAACTAATTGTCGGTGTTAACAAA
ATGGATTCCACTGAGCCACCCTACAGCCAGA 433 (0.011243%)

CAGACTGAGCGTGCCTACCAAAAGCAGCCGACCATCTTTCAAAACAAGAAGAGGGTCCTGCTGGGAGA
AACTGGCAAGGAGAAGCTCCCGCGGTACTACA 8 (0.000208%)

CAGAGGTCCAAGTAAACCGCTAGCTTGTTGCACCGTGGAGGCCACAGGAGCAGAAACATGGAATGCCA GACGCTGGGGATGCTGGTACAAGTTGTGGGAC 66 (0.001714%)

CAGCCCATGACCCCTAACAGGGGCCCTCTCAGCCCTCCTAATGACCTCCGGCCTAGCCATGTGATTTCA CTTCCACTCCATAACGCTCCTCATACTAGGC 22 (0.000571%)

CAGCCCTCCTAATGACCTCCGGCCTAGCCATGTGATTTCACTTCCACTCCATAACGCTCCTCATACTAGG CCTACTAACCAACACACTAACCATATACCA 9 (0.000234%)

CAGCCGACCATCTTTCAAAACAAGAAGAGGGTCCTGCTGGGAGAAACTGGCAAGGAGAAGCTCCCGCG GTACTACAAGAACATCGGTCTGGGCTTCAAGA3 (0.000078%)

CAGCTGGCTTCACTGCTCAGGTGATTATCCTGAACCATCCAGGCCAAATAAGCGCCGGCTATGCCCCTG TATTGGATTGCCACACGGCTCACATTGCATG 16 (0.000415%)

CAGGCACATACTTCCTATTCTACACCCTAGTAGGCTCCCTTCCCCTACTCATCGCACTGATTTACACTCA CAACACCCTAGGCTCACTAAACATTCTACT 24 (0.000623%)

CAGGGGCCCTCTCAGCCCTCCTAATGACCTCCGGCCTAGCCATGTGATTTCACTTCCACTCCATAACGCT CCTCATACTAGGCCTACTAACCAACACACT 21 (0.000545%)

CAGGTGATTATCCTGAACCATCCAGGCCAAATAAGCGCCGGCTATGCCCCTGTATTGGATTGCCACACG GCTCACATTGCATGCAAGTTTGCTGAGCTGA 3 (0.000078%)

CAGGTGTCGTGAAAACTACCCCTAAAAGCCAAAATGGGAAAGGAAAAGACTCATATCAACATTGTCGT CATTGGACACGTAGATTCGGGCAAGTCCACCA84 (0.002181%)

CATAAAAATAAAAAATTATAACAAACCCTGAGAACCAAAATGAACGAAAATCTGTTCGCTTCATTCATT GCCCCCACAATCCTAGGCCTACCCGCCGCAG 9 (0.000234%)

CATACACAACACTAAAGGACGAACCTGATCTCTTATACTAGTATCCTTAATCATTTTTATTGCCACAACT AACCTCCTCGGACTCCTGCCTCACTCATTT20 (0.000519%)

CATAGGTATGGTCTGAGCTATGATATCAATTGGCTTCCTAGGGTTTATCGTGTGAGCACACCATATATTT ACAGTAGGAATAGACGTAGACACACGAGCA 3 (0.000078%)

CATATCAACATTGTCGTCATTGGACACGTAGATTCGGGCAAGTCCACCACTACTGGCCATCTGATCTAT AAATGCGGTGGCATCGACAAAAGAACCATTG 5 (0.000130%)

CATCAACGAAGTGGTAACCCGAGAATACACCATCAACATTCACAAGCGCATCCATGGAGTGGGCTTCA AGAAGCGTGCACCTCGGGCACTCAAAGAGATT 17 (0.000441%)

CATCCCTCTCCAGAAGAGGAGAAGAGGAAACACAAGAAGAAACGCCTGGTGCAGAGCCCCAATTCCTA CTTCATGGATGTGAAATGCCCAGGATGCTATA 4 (0.000104%)

CATCTACAACGTTATCGTCACAGCCCATGCATTTGTAATAATCTTCTTCATAGTAATACCCATCATAATC GGAGGCTTTGGCAACTGACTAGTTCCCCTA 11 (0.000286%)

CATCTCCGCATGATGAAACTTCGGCTCACTCCTTGGCGCCTGCCTGATCCTCCAAATCACCACAGGACTA TTCCTAGCCATGCACTACTCACCAGACGCC 8 (0.000208%)

CATGAAGGCCTCGGGCACGCTACGAGAGTACAAGGTAGTGGGTCGCTGCCTGCCCACCCCCAAATGCC ACACGCCGCCCCTCTACCGCATGCGAATCTTT 10 (0.000260%)

CATGACCCCTAACAGGGGCCCTCTCAGCCCTCCTAATGACCTCCGGCCTAGCCATGTGATTTCACTTCCA CTCCATAACGCTCCTCATACTAGGCCTACT 21 (0.000545%)

CATGATGAAACTTCGGCTCACTCCTTGGCGCCTGCCTGATCCTCCAAATCACCACAGGACTATTCCTAGC
CATGCACTACTCACCAGACGCCTCAACCGC 4 (0.000104%)

CATGCCCTTCTGGCTTACACACTGGGTGTGAAACAACTAATTGTCGGTGTTAACAAAATGGATTCCACT
GAGCCACCCTACAGCCAGAAGAGATATGAGG 6 (0.000156%)

CATGCCTCCATCTAGAAGAGATTATGATGATATGAGCCCTCGTCGAGGACCACCTCCCCCTCCTCCCGG
ACGAGGCGGCCGGGGTGGTAGCAGAGCTCGG 7 (0.000182%)

CATGCCTCTCGCAAAGGATCTCCTTCATCCCTCTCCAGAAGAGGAGAAGAGGAAACACAAGAAGAAAC
GCCTGGTGCAGAGCCCCAATTCCTACTTCATG 28 (0.000727%)

CATTCAGACTGAGCGTGCCTACCAAAAGCAGCCGACCATCTTTCAAAACAAGAAGAGGGTCCTGCTGG
GAGAAACTGGCAAGGAGAAGCTCCCGCGGTAC 4 (0.000104%)

CATTTGGATACATAGGTATGGTCTGAGCTATGATATCAATTGGCTTCCTAGGGTTTATCGTGTGAGCACA
CCATATATTTACAGTAGGAATAGACGTAGA 18 (0.000467%)

CCAAAAGCAGCCGACCATCTTTCAAAACAAGAAGAGGGTCCTGCTGGGAGAAACTGGCAAGGAGAAGC
TCCCGCGGTACTACAAGAACATCGGTCTGGGC 5 (0.000130%)

CCAAAATGGGAAAGGAAAAGACTCATATCAACATTGTCGTCATTGGACACGTAGATTCGGGCAAGTCC
ACCACTACTGGCCATCTGATCTATAAATGCGG 20 (0.000519%)

CCAAAGCCCATAAAAATAAAAAATTATAACAAACCCTGAGAACCAAAATGAACGAAAATCTGTTCGCT
TCATTCATTGCCCCCACAATCCTAGGCCTACC 18 (0.000467%)

CCAACATCTCCGCATGATGAAACTTCGGCTCACTCCTTGGCGCCTGCCTGATCCTCCAAATCACCACAG
GACTATTCCTAGCCATGCACTACTCACCAGA 91 (0.002363%)

CCAAGGTGCAACTTCCTTCGGTCGTCCCGAATCCGGGTTCATCCGACACCAGCCGCCTCCACCATGCCG
CCGAAGTTCGACCCCAACGAGATCAAAGTCG 193 (0.005011%)

CCAAGTAAACCGCTAGCTTGTTGCACCGTGGAGGCCACAGGAGCAGAAACATGGAATGCCAGACGCTG
GGGATGCTGGTACAAGTTGTGGGACTGCATGC 11 (0.000286%)

CCAAGTCTGCCCAGAAAGCTCAGAAGGCTAAATGAATATTATCCCTAATACCTGCCACCCCACTCTTAA
TCAGTGGTGGAAGAACGGTCTCAGAACTGTT 12 (0.000312%)

CCAAGTTGGGCCGCTTGGTCAAGGACATGAAGATCAAGTCCCTGGAGGAGATCTATCTCTTCTCCCTGC
CCATTAAGGAATCAGAGATCATTGATTTCTT 23 (0.000597%)

CCAATGGAAGCAGCTGGCTTCACTGCTCAGGTGATTATCCTGAACCATCCAGGCCAAATAAGCGCCGGC
TATGCCCCTGTATTGGATTGCCACACGGCTC 10 (0.000260%)

CCACCAATGGAAGCAGCTGGCTTCACTGCTCAGGTGATTATCCTGAACCATCCAGGCCAAATAAGCGCC
GGCTATGCCCCTGTATTGGATTGCCACACGG 3 (0.000078%)

CCAGAAAGCTCAGAAGGCTAAATGAATATTATCCCTAATACCTGCCACCCCACTCTTAATCAGTGGTGG
AAGAACGGTCTCAGAACTGTTTGTTTCAATT 7 (0.000182%)

CCAGAACACAGGTGTCGTGAAAACTACCCCTAAAAGCCAAAATGGGAAAGGAAAAGACTCATATCAAC
ATTGTCGTCATTGGACACGTAGATTCGGGCAA 842 (0.021862%)

CCAGCCCATGACCCCTAACAGGGGCCCTCTCAGCCCTCCTAATGACCTCCGGCCTAGCCATGTGATTTC
ACTTCCACTCCATAACGCTCCTCATACTAGG 20 (0.000519%)

CCAGTCAACGTTACAACGGAAGTAAAATCTGTCGAAATGCACCATGAAGCTTTGAGTGAAGCTCTTCCT

GGGGACAATGTGGGCTTCAATGTCAAGAATG 5 (0.000130%)

CCATACACAACACTAAAGGACGAACCTGATCTCTTATACTAGTATCCTTAATCATTTTTATTGCCACAAC
TAACCTCCTCGGACTCCTGCCTCACTCATT3 (0.000078%)

CCATCAACGAAGTGGTAACCCGAGAATACACCATCAACATTCACAAGCGCATCCATGGAGTGGGCTTC
AAGAAGCGTGCACCTCGGGCACTCAAAGAGAT 3 (0.000078%)

CCATCTTTCAAAACAAGAAGAGGGTCCTGCTGGGAGAAACTGGCAAGGAGAAGCTCCCGCGGTACTAC
AAGAACATCGGTCTGGGCTTCAAGACACCCAA 3 (0.000078%)

CCATGACCCCTAACAGGGGCCCTCTCAGCCCTCCTAATGACCTCCGGCCTAGCCATGTGATTTCACTTCC
ACTCCATAACGCTCCTCATACTAGGCCTAC 19 (0.000493%)

CCCACTAATAGCTTTTTGATGACTTCTAGCAAGCCTCGCTAACCTCGCCTTACCCCCCACTATTAACCTA
CTGGGAGAACTCTCTGTGCTAGTAACCACG 9 (0.000234%)

CCCAGAAAGCTCAGAAGGCTAAATGAATATTATCCCTAATACCTGCCACCCCACTCTTAATCAGTGGTG
GAAGAACGGTCTCAGAACTGTTTGTTTCAAT 4 (0.000104%)

CCCAGACATGGGTCCCTCGGCTTCCTGCCTCGGAAGCGCAGCAGCAGGCATCGTGGGAAGGTGAAGAG
CTTCCCTAAGGATGACCCGTCCAAGCCGGTCC 4 (0.000104%)

CCCAGCCCATGACCCCTAACAGGGGCCCTCTCAGCCCTCCTAATGACCTCCGGCCTAGCCATGTGATTTC
ACTTCCACTCCATAACGCTCCTCATACTAG 48 (0.001246%)

CCCATAAAAATAAAAAATTATAACAAACCCTGAGAACCAAAATGAACGAAAATCTGTTCGCTTCATTCA
TTGCCCCCACAATCCTAGGCCTACCCGCCGC 4 (0.000104%)

CCCATGACCCCTAACAGGGGCCCTCTCAGCCCTCCTAATGACCTCCGGCCTAGCCATGTGATTTCACTTC
CACTCCATAACGCTCCTCATACTAGGCCTA 26 (0.000675%)

CCCATGCATTTGTAATAATCTTCTTCATAGTAATACCCATCATAATCGGAGGCTTTGGCAACTGACTAGT
TCCCCTAATAATCGGTGCCCCCGATATGGC 3 (0.000078%)

CCCATGCCTCCATCTAGAAGAGATTATGATGATATGAGCCCTCGTCGAGGACCACCTCCCCCTCCTCCC
GGACGAGGCGGCCGGGGTGGTAGCAGAGCTC 8 (0.000208%)

CCCCTAAAAGCCAAAATGGGAAAGGAAAAGACTCATATCAACATTGTCGTCATTGGACACGTAGATTC
GGGCAAGTCCACCACTACTGGCCATCTGATCT 3 (0.000078%)

CCCCTAACAGGGGCCCTCTCAGCCCTCCTAATGACCTCCGGCCTAGCCATGTGATTTCACTTCCACTCCA
TAACGCTCCTCATACTAGGCCTACTAACCA 15 (0.000389%)

CCCGAATCCGGGTTCATCCGACACCAGCCGCCTCCACCATGCCGCCGAAGTTCGACCCCAACGAGATCA
AAGTCGTATACCTGAGGTGCACCGGAGGTGA 17 (0.000441%)

CCCGAGAGCATGCCCTTCTGGCTTACACACTGGGTGTGAAACAACTAATTGTCGGTGTTAACAAAATGG
ATTCCACTGAGCCACCCTACAGCCAGAAGAG 14 (0.000364%)

CCCGCTATGGGGCCTCCCTCCGGAAAATGGTGAAGAAAATTGAAATCAGCCAGCACGCCAAGTACACT
TGCTCTTTCTGTGGCAAAACCAAGATGAAGAG4 (0.000104%)

CCCGTCACCAAGTTGGGCCGCTTGGTCAAGGACATGAAGATCAAGTCCCTGGAGGAGATCTATCTCTTC
TCCCTGCCCATTAAGGAATCAGAGATCATTG 71 (0.001844%)

CCCTAAAAGCCAAAATGGGAAAGGAAAAGACTCATATCAACATTGTCGTCATTGGACACGTAGATTCG
GGCAAGTCCACCACTACTGGCCATCTGATCTA 8 (0.000208%)

CCCTAAATTCTTGAAGTCTGGTGATGCTGCCATTGTTGATATGGTTCCTGGCAAGCCCATGTGTGTTGAG
AGCTTCTCAGACTATCCACCTTTGGGTCGC 10 (0.000260%)

CCCTAACAGGGGCCCTCTCAGCCCTCCTAATGACCTCCGGCCTAGCCATGTGATTTCACTTCCACTCCAT
AACGCTCCTCATACTAGGCCTACTAACCAA 57 (0.001480%)

CCCTAATACCTGCCACCCCACTCTTAATCAGTGGTGGAAGAACGGTCTCAGAACTGTTTGTTTCAATTGG
CCATTTAAGTTTAGTAGTAAAAGACTGGTT 45 (0.001168%)

CCCTCACCAAAGCCCATAAAAATAAAAAATTATAACAAACCCTGAGAACCAAAATGAACGAAAATCTG
TTCGCTTCATTCATTGCCCCCACAATCCTAGG 12 (0.000312%)

CCCTCCTAATGACCTCCGGCCTAGCCATGTGATTTCACTTCCACTCCATAACGCTCCTCATACTAGGCCT
ACTAACCAACACACTAACCATATACCAATG 6 (0.000156%)

CCCTCTCAGCCCTCCTAATGACCTCCGGCCTAGCCATGTGATTTCACTTCCACTCCATAACGCTCCTCAT
ACTAGGCCTACTAACCAACACACTAACCAT 11 (0.000286%)

CCCTCTCCAGAAGAGGAGAAGAGGAAACACAAGAAGAAACGCCTGGTGCAGAGCCCCAATTCCTACTT
CATGGATGTGAAATGCCCAGGATGCTATAAAA 4 (0.000104%)

CCCTGATCGGCGCACTGCGAGCAGTAGCCCAAACAATCTCATATGAAGTCACCCTAGCCATCATTCTAC
TATCAACATTACTAATAAGTGGCTCCTTTAA 15 (0.000389%)

CCCTTGCGCCTGCCTCTCCAGGATGTCTACAAAATTGGTGGTATTGGTACTGTTCCTGTTGGCCGAGTGG
AGACTGGTGTTCTCAAACCCGGTATGGTGG 6 (0.000156%)

CCCTTTGTGACTTCCGACCGAAGCAAGAATCGCAAAAGGCATTTCAATGCACCTTCCCACATTCGAAGG
AAGATTATGTCTTCCCCTCTTTCCAAAGAGC 7 (0.000182%)

CCGAAGCAAGAATCGCAAAAGGCATTTCAATGCACCTTCCCACATTCGAAGGAAGATTATGTCTTCCCC
TCTTTCCAAAGAGCTGAGACAGAAGTACAAC 3 (0.000078%)

CCGAATCCGGGTTCATCCGACACCAGCCGCCTCCACCATGCCGCCGAAGTTCGACCCCAACGAGATCAA
AGTCGTATACCTGAGGTGCACCGGAGGTGAA 9 (0.000234%)

CCGACACCAGCCGCCTCCACCATGCCGCCGAAGTTCGACCCCAACGAGATCAAAGTCGTATACCTGAGG
TGCACCGGAGGTGAAGTCGGTGCCACTTCTG 25 (0.000649%)

CCGACCGAAGCAAGAATCGCAAAAGGCATTTCAATGCACCTTCCCACATTCGAAGGAAGATTATGTCTT
CCCCTCTTTCCAAAGAGCTGAGACAGAAGTA 11 (0.000286%)

CCGAGAGCATGCCCTTCTGGCTTACACACTGGGTGTGAAACAACTAATTGTCGGTGTTAACAAAATGGA
TTCCACTGAGCCACCCTACAGCCAGAAGAGA 47 (0.001220%)

CCGAGGAGCACCCCGTGCTGCTGACCGAGGCCCCCCTGAACCCCAAGGCCAACCGCGAGAAGATGACC
CAGATCATGTTTGAGACCTTCAACACCCCAGC 6 (0.000156%)

CCGCAGCCGCCTCCGCCGCGCGCCTCCTCCGCCGCCGCGGACTCCGGCAGCTTTATCGCCAGAGTCCCT
GAACTCTCGCTTTCTTTTTAATCCCCTGCAT 3 (0.000078%)

CCGCATGATGAAACTTCGGCTCACTCCTTGGCGCCTGCCTGATCCTCCAAATCACCACAGGACTATTCCT
AGCCATGCACTACTCACCAGACGCCTCAAC 3 (0.000078%)

CCGCCGCAGACGCCGCCGCGATGCGCTACGTCGCCTCCTACCTGCTGGCTGCCCTAGGGGGCAACTCCT
CCCCCAGCGCCAAGGACATCAAGAAGATCTT 14 (0.000364%)

CCGCCGCGATGCGCTACGTCGCCTCCTACCTGCTGGCTGCCCTAGGGGGCAACTCCTCCCCCAGCGCCA
AGGACATCAAGAAGATCTTGGACAGCGTGGG 5 (0.000130%)

CCGCCGTGACCTATTCACCCTCCACTTCCCGTCTCAGAATCTAAACGTGGTCACCTTCGAGTAGAGAGG
CCCGCCCGCCCACCGTGGGCAGTGCCACCCG 8 (0.000208%)

CCGCCTCCTTGCTCGCCGCAGCCGCCTCCGCCGCGCCTCCTCCGCCGCCGCGGACTCCGGCAGCTTTA
TCGCCAGAGTCCCTGAACTCTCGCTTTCTT 27 (0.000701%)

CCGCGATGCGCTACGTCGCCTCCTACCTGCTGGCTGCCCTAGGGGGCAACTCCTCCCCCAGCGCCAAGG
ACATCAAGAAGATCTTGGACAGCGTGGGTAT 5 (0.000130%)

CCGCTAGCTTGTTGCACCGTGGAGGCCACAGGAGCAGAAACATGGAATGCCAGACGCTGGGGATGCTG
GTACAAGTTGTGGGACTGCATGCTACTGTCTA 9 (0.000234%)

CCGCTATGGGGCCTCCCTCCGGAAAATGGTGAAGAAAATTGAAATCAGCCAGCACGCCAAGTACACTT
GCTCTTTCTGTGGCAAAACCAAGATGAAGAGA 14 (0.000364%)

CCGCTCCCAGACATGGGTCCCTCGGCTTCCTGCCTCGGAAGCGCAGCAGCAGGCATCGTGGGAAGGTGA
AGAGCTTCCCTAAGGATGACCCGTCCAAGCC 3 (0.000078%)

CCGCTTGGTCAAGGACATGAAGATCAAGTCCCTGGAGGAGATCTATCTCTTCTCCCTGCCCATTAAGGA
ATCAGAGATCATTGATTTCTTCCTGGGGGCC 5 (0.000130%)

CCGGGTTCATCCGACACCAGCCGCCTCCACCATGCCGCCGAAGTTCGACCCCAACGAGATCAAAGTCGT
ATACCTGAGGTGCACCGGAGGTGAAGTCGGT 4 (0.000104%)

CCGTCACCAAGTTGGGCCGCTTGGTCAAGGACATGAAGATCAAGTCCCTGGAGGAGATCTATCTCTTCT
CCCTGCCCATTAAGGAATCAGAGATCATTGA 99 (0.002571%)

CCGTCCTAATCACAGCAGTCCTACTTCTCCTATCTCTCCCAGTCCTAGCTGCTGGCATCACTATACTACT
AACAGACCGCAACCTCAACACCACCTTCTT 7 (0.000182%)

CCGTGACCTATTCACCCTCCACTTCCCGTCTCAGAATCTAAACGTGGTCACCTTCGAGTAGAGAGGCCC
GCCCGCCCACCGTGGGCAGTGCCACCCGCAG 15 (0.000389%)

CCGTTCTGGTAAAAAGCTGGAAGATGGCCCTAAATTCTTGAAGTCTGGTGATGCTGCCATTGTTGATAT
GGTTCCTGGCAAGCCCATGTGTGTTGAGAGC 18 (0.000467%)

CCTAAAACCCGCCGGACTTTCTGTAAGAAGTGTGGCAAGCACCAACCCCATAAAGTGACACAGTACAA
GAAGGGCAAGGATTCTCTGTACGCCCAGGGAA 4 (0.000104%)

CCTAAAAGCCAAAATGGGAAAGGAAAAGACTCATATCAACATTGTCGTCATTGGACACGTAGATTCGG
GCAAGTCCACCACTACTGGCCATCTGATCTAT 18 (0.000467%)

CCTAAATTCTTGAAGTCTGGTGATGCTGCCATTGTTGATATGGTTCCTGGCAAGCCCATGTGTGTTGAGA
GCTTCTCAGACTATCCACCTTTGGGTCGCT 34 (0.000883%)

CCTAACAGGGGCCCTCTCAGCCCTCCTAATGACCTCCGGCCTAGCCATGTGATTTCACTTCCACTCCATA
ACGCTCCTCATACTAGGCCTACTAACCAAC 11 (0.000286%)

CCTAATACCTGCCACCCCACTCTTAATCAGTGGTGGAAGAACGGTCTCAGAACTGTTTGTTTCAATTGGC
CATTTAAGTTTAGTAGTAAAAGACTGGTTA 17 (0.000441%)

CCTAATCACAGCAGTCCTACTTCTCCTATCTCTCCCAGTCCTAGCTGCTGGCATCACTATACTACTAACA
GACCGCAACCTCAACACCACCTTCTTCGAC 22 (0.000571%)

CCTAATGACCTCCGGCCTAGCCATGTGATTTCACTTCCACTCCATAACGCTCCTCATACTAGGCCTACTA

ACCAACACACTAACCATATACCAATGATGG 5 (0.000130%)

CCTACCAAAAGCAGCCGACCATCTTTCAAAACAAGAAGAGGGTCCTGCTGGGAGAAACTGGCAAGGAG
AAGCTCCCGCGGTACTACAAGAACATCGGTCT 4 (0.000104%)

CCTACGCACACGAGAACATGCCTCTCGCAAAGGATCTCCTTCATCCCTCTCCAGAAGAGGAGAAGAGG
AAACACAAGAAGAAACGCCTGGTGCAGAGCCC 420 (0.010905%)

CCTCAAAACAAATGATAACCATACACAACACTAAAGGACGAACCTGATCTCTTATACTAGTATCCTTAA
TCATTTTTATTGCCACAACTAACCTCCTCGG 15 (0.000389%)

CCTCACCAAAGCCCATAAAAATAAAAAATTATAACAAACCCTGAGAACCAAAATGAACGAAAATCTGT
TCGCTTCATTCATTGCCCCCACAATCCTAGGC 52 (0.001350%)

CCTCCATCTAGAAGAGATTATGATGATATGAGCCCTCGTCGAGGACCACCTCCCCCTCCTCCCGGACGA
GGCGGCCGGGGTGGTAGCAGAGCTCGGAATC 6 (0.000156%)

CCTCCCTCACCAAAGCCCATAAAAATAAAAAATTATAACAAACCCTGAGAACCAAAATGAACGAAAAT
CTGTTCGCTTCATTCATTGCCCCCACAATCCT 17 (0.000441%)

CCTCCTAATGACCTCCGGCCTAGCCATGTGATTTCACTTCCACTCCATAACGCTCCTCATACTAGGCCTA
CTAACCAACACACTAACCATATACCAATGA 5 (0.000130%)

CCTCGGCTTCCTGCCTCGGAAGCGCAGCAGCAGGCATCGTGGGAAGGTGAAGAGCTTCCCTAAGGATG
ACCCGTCCAAGCCGGTCCACCTCACAGCCTTC 11 (0.000286%)

CCTCGGGCACGCTACGAGAGTACAAGGTAGTGGGTCGCTGCCTGCCCACCCCCAAATGCCACACGCCGC
CCCTCTACCGCATGCGAATCTTTGCGCCTAA 11 (0.000286%)

CCTCTCAGCCCTCCTAATGACCTCCGGCCTAGCCATGTGATTTCACTTCCACTCCATAACGCTCCTCATA
CTAGGCCTACTAACCAACACACTAACCATA 3 (0.000078%)

CCTCTCGCAAAGGATCTCCTTCATCCCTCTCCAGAAGAGGAGAAGAGGAAACACAAGAAGAAACGCCT
GGTGCAGAGCCCCAATTCCTACTTCATGGATG 10 (0.000260%)

CCTCTCTCAAGGATGAGGTTTTGAAGATTATGCCAGTGCAGAAGCAGACCCGTGCCGGCCAGCGCACCA
GGTTCAAGGCATTTGTTGCTATCGGGGACTA 8 (0.000208%)

CCTGAACGCAGGCACATACTTCCTATTCTACACCCTAGTAGGCTCCCTTCCCCTACTCATCGCACTGATT
TACACTCACAACACCCTAGGCTCACTAAAC 37 (0.000961%)

CCTGATCGGCGCACTGCGAGCAGTAGCCCAAACAATCTCATATGAAGTCACCCTAGCCATCATTCTACT
ATCAACATTACTAATAAGTGGCTCCTTTAAC 7 (0.000182%)

CCTGGGGGCCTCTCTCAAGGATGAGGTTTTGAAGATTATGCCAGTGCAGAAGCAGACCCGTGCCGGCCA
GCGCACCAGGTTCAAGGCATTTGTTGCTATC 7 (0.000182%)

CCTTACTACACAATCAAAGACGCCCTCGGCTTACTTCTCTTCATTCTCTCCTTAATGACATTAACACTATT
CTCACCAGACCTCCTAGGCGACCCAGACA 10 (0.000260%)

CCTTCATCCCTCTCCAGAAGAGGAGAAGAGGAAACACAAGAAGAAACGCCTGGTGCAGAGCCCCAATT
CCTACTTCATGGATGTGAAATGCCCAGGATGC 15 (0.000389%)

CCTTGCGCCTGCCTCTCCAGGATGTCTACAAAATTGGTGGTATTGGTACTGTTCCTGTTGGCCGAGTGGA
GACTGGTGTTCTCAAACCCGGTATGGTGGT 18 (0.000467%)

CCTTGCTCGCCGCAGCCGCCTCCGCCGCGCGCCTCCTCCGCCGCCGCGGACTCCGGCAGCTTTATCGCCA
GAGTCCCTGAACTCTCGCTTTCTTTTTAAT 39 (0.001013%)

CGAAATGCACCATGAAGCTTTGAGTGAAGCTCTTCCTGGGGACAATGTGGGCTTCAATGTCAAGAATGT
GTCTGTCAAGGATGTTCGTCGTGGCAACGTT 10 (0.000260%)

CGAACCTGATCTCTTATACTAGTATCCTTAATCATTTTTATTGCCACAACTAACCTCCTCGGACTCCTGCC
TCACTCATTTACACCAACCACCCAACTAT 116 (0.003012%)

CGAAGATATGCTCATGTGGTGTTGAGGAAAGCAGACATTGACCTCACCAAGAGGGCGGGAGAACTCAC
TGAGGATGAGGTGGAACGTGTGATCACCATTA401 (0.010412%)

CGAATGCGCAGGCTGAAGCGCAAAAGAAGAAAGATGAGGCAGAGGTCCAAGTAAACCGCTAGCTTGTT
GCACCGTGGAGGCCACAGGAGCAGAAACATGG 77 (0.001999%)

CGACCACATCTACAACGTTATCGTCACAGCCCATGCATTTGTAATAATCTTCTTCATAGTAATACCCATC
ATAATCGGAGGCTTTGGCAACTGACTAGTT 247 (0.006413%)

CGACCATCTTTCAAAACAAGAAGAGGGTCCTGCTGGGAGAAACTGGCAAGGAGAAGCTCCCGCGGTAC
TACAAGAACATCGGTCTGGGCTTCAAGACACC6 (0.000156%)

CGAGAACATGCCTCTCGCAAAGGATCTCCTTCATCCCTCTCCAGAAGAGGAGAAGAGGAAACACAAGA
AGAAACGCCTGGTGCAGAGCCCCAATTCCTAC147 (0.003817%)

CGAGAAGAAAAAGGGCCGTTCTGCCATCAACGAAGTGGTAACCCGAGAATACACCATCAACATTCACA
AGCGCATCCATGGAGTGGGCTTCAAGAAGCGT 19 (0.000493%)

CGAGAGCATGCCCTTCTGGCTTACACACTGGGTGTGAAACAACTAATTGTCGGTGTTAACAAAATGGAT
TCCACTGAGCCACCCTACAGCCAGAAGAGAT 83 (0.002155%)

CGAGAGTACAAGGTAGTGGGTCGCTGCCTGCCCACCCCCAAATGCCACACGCCGCCCCTCTACCGCATG
CGAATCTTTGCGCCTAATCATGTCGTCGCCA 14 (0.000364%)

CGAGGAGCACCCCGTGCTGCTGACCGAGGCCCCCCTGAACCCCAAGGCCAACCGCGAGAAGATGACCC
AGATCATGTTTGAGACCTTCAACACCCCAGCC 6 (0.000156%)

CGATACGGGATAATCCTATTTATTACCTCAGAAGTTTTTTTCTTCGCAGGATTTTTCTGAGCCTTTTACCA
CTCCAGCCTAGCCCCTACCCCCCAATTAG 6 (0.000156%)

CGCAAAAGAAGAAAGATGAGGCAGAGGTCCAAGTAAACCGCTAGCTTGTTGCACCGTGGAGGCCACAG
GAGCAGAAACATGGAATGCCAGACGCTGGGGA 129 (0.003349%)

CGCAAAGAAGGGTGGCGAGAAGAAAAAGGGCCGTTCTGCCATCAACGAAGTGGTAACCCGAGAATAC
ACCATCAACATTCACAAGCGCATCCATGGAGTG 413 (0.010723%)

CGCAAAGGATCTCCTTCATCCCTCTCCAGAAGAGGAGAAGAGGAAACACAAGAAGAAACGCCTGGTGC
AGAGCCCCAATTCCTACTTCATGGATGTGAAA 12 (0.000312%)

CGCAAGCATGGTTAACGTCCCTAAAACCCGCCGGACTTTCTGTAAGAAGTGTGGCAAGCACCAACCCCA
TAAAGTGACACAGTACAAGAAGGGCAAGGAT 38 (0.000987%)

CGCACACGAGAACATGCCTCTCGCAAAGGATCTCCTTCATCCCTCTCCAGAAGAGGAGAAGAGGAAAC
ACAAGAAGAAACGCCTGGTGCAGAGCCCCAAT 103 (0.002674%)

CGCAGACGCCGCCGCGATGCGCTACGTCGCCTCCTACCTGCTGGCTGCCCTAGGGGGCAACTCCTCCCC
CAGCGCCAAGGACATCAAGAAGATCTTGGAC 5 (0.000130%)

CGCAGCCGCCTCCGCCGCGCGCCTCCTCCGCCGCCGCGGACTCCGGCAGCTTTATCGCCAGAGTCCCTG
AACTCTCGCTTTCTTTTTAATCCCCTGCATC 13 (0.000338%)

CGCAGGCACATACTTCCTATTCTACACCCTAGTAGGCTCCCTTCCCCTACTCATCGCACTGATTTACACT
CACAACACCCTAGGCTCACTAAACATTCTA 24 (0.000623%)

CGCATGATGAAACTTCGGCTCACTCCTTGGCGCCTGCCTGATCCTCCAAATCACCACAGGACTATTCCTA
GCCATGCACTACTCACCAGACGCCTCAACC 19 (0.000493%)

CGCCATGAAGGCCTCGGGCACGCTACGAGAGTACAAGGTAGTGGGTCGCTGCCTGCCCACCCCCAAAT
GCCACACGCCGCCCCTCTACCGCATGCGAATC 12 (0.000312%)

CGCCGCAGACGCCGCCGCGATGCGCTACGTCGCCTCCTACCTGCTGGCTGCCCTAGGGGGCAACTCCTC
CCCCAGCGCCAAGGACATCAAGAAGATCTTG 6 (0.000156%)

CGCCGCAGCCGCCTCCGCCGCGCGCCTCCTCCGCCGCCGCGGACTCCGGCAGCTTTATCGCCAGAGTCC
CTGAACTCTCGCTTTCTTTTTAATCCCCTGC 7 (0.000182%)

CGCCGCCGCGATGCGCTACGTCGCCTCCTACCTGCTGGCTGCCCTAGGGGGCAACTCCTCCCCCAGCGC
CAAGGACATCAAGAAGATCTTGGACAGCGTG 3 (0.000078%)

CGCCGCGATGCGCTACGTCGCCTCCTACCTGCTGGCTGCCCTAGGGGGCAACTCCTCCCCCAGCGCCAA
GGACATCAAGAAGATCTTGGACAGCGTGGGT 26 (0.000675%)

CGCCGTGACCTATTCACCCTCCACTTCCCGTCTCAGAATCTAAACGTGGTCACCTTCGAGTAGAGAGGC
CCGCCCGCCCACCGTGGGCAGTGCCACCCGC 4 (0.000104%)

CGCCGTTCTGGTAAAAAGCTGGAAGATGGCCCTAAATTCTTGAAGTCTGGTGATGCTGCCATTGTTGAT
ATGGTTCCTGGCAAGCCCATGTGTGTTGAGA 219 (0.005686%)

CGCCTCCTTGCTCGCCGCAGCCGCCTCCGCCGCGCGCCTCCTCCGCCGCCGCGGACTCCGGCAGCTTTAT
CGCCAGAGTCCCTGAACTCTCGCTTTCTTT22 (0.000571%)

CGCCTGAACGCAGGCACATACTTCCTATTCTACACCCTAGTAGGCTCCCTTCCCCTACTCATCGCACTGA
TTTACACTCACAACACCCTAGGCTCACTAA 12 (0.000312%)

CGCTACGAGAGTACAAGGTAGTGGGTCGCTGCCTGCCCACCCCCAAATGCCACACGCCGCCCCTCTACC
GCATGCGAATCTTTGCGCCTAATCATGTCGT 10 (0.000260%)

CGCTAGCTTGTTGCACCGTGGAGGCCACAGGAGCAGAAACATGGAATGCCAGACGCTGGGGATGCTGG
TACAAGTTGTGGGACTGCATGCTACTGTCTAG 19 (0.000493%)

CGCTATGGGGCCTCCCTCCGGAAAATGGTGAAGAAAATTGAAATCAGCCAGCACGCCAAGTACACTTG
CTCTTTCTGTGGCAAAACCAAGATGAAGAGAC12 (0.000312%)

CGCTCCCAGACATGGGTCCCTCGGCTTCCTGCCTCGGAAGCGCAGCAGCAGGCATCGTGGGAAGGTGA
AGAGCTTCCCTAAGGATGACCCGTCCAAGCCG9 (0.000234%)

CGCTGCCGTCGCCGCCGCCACCATGCCCAAGAGAAAGGCTGAAGGGGATGCTAAGGGAGATAAAGCAA
AGGTGAAGGACGAACCACAGAGAAGATCCGCG 185 (0.004803%)

CGGAAGTAAAATCTGTCGAAATGCACCATGAAGCTTTGAGTGAAGCTCTTCCTGGGGACAATGTGGGCT
TCAATGTCAAGAATGTGTCTGTCAAGGATGT 93 (0.002415%)

CGGAGAGCACGCCATGAAGGCCTCGGGCACGCTACGAGAGTACAAGGTAGTGGGTCGCTGCCTGCCCA
CCCCCAAATGCCACACGCCGCCCCTCTACCGC 19 (0.000493%)

CGGCGCACTGCGAGCAGTAGCCCAAACAATCTCATATGAAGTCACCCTAGCCATCATTCTACTATCAAC
ATTACTAATAAGTGGCTCCTTTAACCTCTCC 20 (0.000519%)

CGGCTCACTCCTTGGCGCCTGCCTGATCCTCCAAATCACCACAGGACTATTCCTAGCCATGCACTACTCA

CCAGACGCCTCAACCGCCTTTTCATCAATC 13 (0.000338%)

CGGCTTCCTGCCTCGGAAGCGCAGCAGCAGGCATCGTGGGAAGGTGAAGAGCTTCCCTAAGGATGACC
CGTCCAAGCCGGTCCACCTCACAGCCTTCCTG 17 (0.000441%)

CGGGACCCGCTATGGGGCCTCCCTCCGGAAAATGGTGAAGAAAATTGAAATCAGCCAGCACGCCAAGT
ACACTTGCTCTTTCTGTGGCAAAACCAAGATG 7 (0.000182%)

CGGGATAATCCTATTTATTACCTCAGAAGTTTTTTCTTCGCAGGATTTTCTGAGCCTTTTACCACTCCA
GCCTAGCCCCTACCCCCCAATTAGGAGGG 21 (0.000545%)

CGGGCACGCTACGAGAGTACAAGGTAGTGGGTCGCTGCCTGCCCACCCCCAAATGCCACACGCCGCCC
CTCTACCGCATGCGAATCTTTGCGCCTAATCA 16 (0.000415%)

CGGGTTCATCCGACACCAGCCGCCTCCACCATGCCGCCGAAGTTCGACCCCAACGAGATCAAAGTCGTA
TACCTGAGGTGCACCGGAGGTGAAGTCGGTG 8 (0.000208%)

CGGTCGTCCCGAATCCGGGTTCATCCGACACCAGCCGCCTCCACCATGCCGCCGAAGTTCGACCCCAAC
GAGATCAAAGTCGTATACCTGAGGTGCACCG 4 (0.000104%)

CGTACTGCTAGGAAGCTCCGTAGTCACCGACGAGACCAGAAGTGGCATGATAAACAGTATAAGAAAGC
TCATTTGGGCACAGCCCTAAAGGCCAACCCTT 8 (0.000208%)

CGTCACCAAGTTGGGCCGCTTGGTCAAGGACATGAAGATCAAGTCCCTGGAGGAGATCTATCTCTTCTC
CCTGCCCATTAAGGAATCAGAGATCATTGAT 86 (0.002233%)

CGTCCCTAAAACCCGCCGGACTTTCTGTAAGAAGTGTGGCAAGCACCAACCCCATAAAGTGACACAGTA
CAAGAAGGGCAAGGATTCTCTGTACGCCCAG 12 (0.000312%)

CGTCCTAATCACAGCAGTCCTACTTCTCCTATCTCTCCCAGTCCTAGCTGCTGGCATCACTATACTACTA
ACAGACCGCAACCTCAACACCACCTTCTTC 3 (0.000078%)

CGTCGTGGCAACGTTGCTGGTGACAGCAAAAATGACCCACCAATGGAAGCAGCTGGCTTCACTGCTCAG
GTGATTATCCTGAACCATCCAGGCCAAATAA 26 (0.000675%)

CGTGAAAACTACCCCTAAAAGCCAAAATGGGAAAGGAAAAGACTCATATCAACATTGTCGTCATTGGA
CACGTAGATTCGGGCAAGTCCACCACTACTGG 20 (0.000519%)

CGTGCCTACCAAAAGCAGCCGACCATCTTTCAAAACAAGAAGAGGGTCCTGCTGGGAGAAACTGGCAA
GGAGAAGCTCCCGCGGTACTACAAGAACATCG 4 (0.000104%)

CGTGGCAACGTTGCTGGTGACAGCAAAAATGACCCACCAATGGAAGCAGCTGGCTTCACTGCTCAGGT
GATTATCCTGAACCATCCAGGCCAAATAAGCG 7 (0.000182%)

CGTGGTTGCATTGTGGATGCAAATCTGAGCGTTCTCAACTTGGTTATTGTAAAAAAAGGAGAGAAGGAT
ATTCCTGGACTGACTGATACTACAGTGCCTC 5 (0.000130%)

CGTTACAACGGAAGTAAAATCTGTCGAAATGCACCATGAAGCTTTGAGTGAAGCTCTTCCTGGGGACAA
TGTGGGCTTCAATGTCAAGAATGTGTCTGTC 5 (0.000130%)

CGTTATCGTCACAGCCCATGCATTTGTAATAATCTTCTTCATAGTAATACCCATCATAATCGGAGGCTTT
GGCAACTGACTAGTTCCCCTAATAATCGGT 14 (0.000364%)

CGTTCTGCCATCAACGAAGTGGTAACCCGAGAATACACCATCAACATTCACAAGCGCATCCATGGAGTG
GGCTTCAAGAAGCGTGCACCTCGGGCACTCA 10 (0.000260%)

CGTTCTGGTAAAAAGCTGGAAGATGGCCCTAAATTCTTGAAGTCTGGTGATGCTGCCATTGTTGATATG
GTTCCTGGCAAGCCCATGTGTGTTGAGAGCT 19 (0.000493%)

CGTTGCTGGTGACAGCAAAAATGACCCACCAATGGAAGCAGCTGGCTTCACTGCTCAGGTGATTATCCT
GAACCATCCAGGCCAAATAAGCGCCGGCTAT 10 (0.000260%)

CTAAAACCCGCCGGACTTTCTGTAAGAAGTGTGGCAAGCACCAACCCCATAAAGTGACACAGTACAAG
AAGGGCAAGGATTCTCTGTACGCCCAGGGAAA 46 (0.001194%)

CTAAAAGCCAAAATGGGAAAGGAAAAGACTCATATCAACATTGTCGTCATTGGACACGTAGATTCGGG
CAAGTCCACCACTACTGGCCATCTGATCTATA 110 (0.002856%)

CTAAAGGACGAACCTGATCTCTTATACTAGTATCCTTAATCATTTTTATTGCCACAACTAACCTCCTCGG
ACTCCTGCCTCACTCATTTACACCAACCAC 3 (0.000078%)

CTAAATGAATATTATCCCTAATACCTGCCACCCCACTCTTAATCAGTGGTGGAAGAACGGTCTCAGAAC
TGTTTGTTTCAATTGGCCATTTAAGTTTAGT 7 (0.000182%)

CTAAATTCTTGAAGTCTGGTGATGCTGCCATTGTTGATATGGTTCCTGGCAAGCCCATGTGTGTTGAGAG
CTTCTCAGACTATCCACCTTTGGGTCGCTT 116 (0.003012%)

CTAACACTCACAACAAAACTAACTAATACTAACATCTCAGACGCTCAGGAAATAGAAACCGTCTGAACT
ATCCTGCCCGCCATCATCCTAGTCCTCATCG 440 (0.011425%)

CTAACAGGGGCCCTCTCAGCCCTCCTAATGACCTCCGGCCTAGCCATGTGATTTCACTTCCACTCCATAA
CGCTCCTCATACTAGGCCTACTAACCAACA 38 (0.000987%)

CTAACCGCTAACATTACTGCAGGCCACCTACTCATGCACCTAATTGGAAGCGCCACCCTAGCAATATCA
ACCATTAACCTTCCCTCTACACTTATCATCT 453 (0.011762%)

CTAACCTCAAAACAAATGATAACCATACACAACACTAAAGGACGAACCTGATCTCTTATACTAGTATCC
TTAATCATTTTTATTGCCACAACTAACCTCC 825 (0.021421%)

CTAATACCTGCCACCCCACTCTTAATCAGTGGTGGAAGAACGGTCTCAGAACTGTTTGTTTCAATTGGCC
ATTTAAGTTTAGTAGTAAAAGACTGGTTAA 57 (0.001480%)

CTAATCACAGCAGTCCTACTTCTCCTATCTCTCCCAGTCCTAGCTGCTGGCATCACTATACTACTAACAG
ACCGCAACCTCAACACCACCTTCTTCGACC 4 (0.000104%)

CTAATGACCTCCGGCCTAGCCATGTGATTTCACTTCCACTCCATAACGCTCCTCATACTAGGCCTACTAA
CCAACACACTAACCATATACCAATGATGGC 6 (0.000156%)

CTACAACGTTATCGTCACAGCCCATGCATTTGTAATAATCTTCTTCATAGTAATACCCATCATAATCGGA
GGCTTTGGCAACTGACTAGTTCCCCTAATA 25 (0.000649%)

CTACCAAAAGCAGCCGACCATCTTTCAAAACAAGAAGAGGGTCCTGCTGGGAGAAACTGGCAAGGAGA
AGCTCCCGCGGTACTACAAGAACATCGGTCTG 6 (0.000156%)

CTACCCCTAAAAGCCAAAATGGGAAAGGAAAAGACTCATATCAACATTGTCGTCATTGGACACGTAGA
TTCGGGCAAGTCCACCACTACTGGCCATCTGA 21 (0.000545%)

CTACGCACACGAGAACATGCCTCTCGCAAAGGATCTCCTTCATCCCTCTCCAGAAGAGGAGAAGAGGA
AACACAAGAAGAAACGCCTGGTGCAGAGCCCC 29 (0.000753%)

CTAGCTTGTTGCACCGTGGAGGCCACAGGAGCAGAAACATGGAATGCCAGACGCTGGGGATGCTGGTA
CAAGTTGTGGGACTGCATGCTACTGTCTAGAG 13 (0.000338%)

CTAGGAAGCTCCGTAGTCACCGACGAGACCAGAAGTGGCATGATAAACAGTATAAGAAAGCTCATTTG
GGCACAGCCCTAAAGGCCAACCCTTTTGGAGG 6 (0.000156%)

CTATCACCCTATAGAAGAACTAATGTTAGTATAAGTAACATGAAAACATTCTCCTCCGCATAAGCCTGC
GTCAGATTAAAACACTGAACTGACAATTAAC 552 (0.014333%)

CTCAAAACAAATGATAACCATACACAACACTAAAGGACGAACCTGATCTCTTATACTAGTATCCTTAAT
CATTTTTATTGCCACAACTAACCTCCTCGGA 51 (0.001324%)

CTCAAGGACTTCAAACTCTACTCCCACTAATAGCTTTTTGATGACTTCTAGCAAGCCTCGCTAACCTCGC
CTTACCCCCCACTATTAACCTACTGGGAGA 13 (0.000338%)

CTCAAGGATGAGGTTTTGAAGATTATGCCAGTGCAGAAGCAGACCCGTGCCGGCCAGCGCACCAGGTT
CAAGGCATTTGTTGCTATCGGGGACTACAATG 11 (0.000286%)

CTCACAACAAAACTAACTAATACTAACATCTCAGACGCTCAGGAAATAGAAACCGTCTGAACTATCCTG
CCCGCCATCATCCTAGTCCTCATCGCCCTCC 27 (0.000701%)

CTCACAGAAAGTTCTCCGCTCCCAGACATGGGTCCCTCGGCTTCCTGCCTCGGAAGCGCAGCAGCAGGC
ATCGTGGGAAGGTGAAGAGCTTCCCTAAGGA 344 (0.008932%)

CTCACCAAAGCCCATAAAAATAAAAAATTATAACAAACCCTGAGAACCAAAATGAACGAAAATCTGTT
CGCTTCATTCATTGCCCCCACAATCCTAGGCC 32 (0.000831%)

CTCACGCAAGCATGGTTAACGTCCCTAAAACCCGCCGGACTTTCTGTAAGAAGTGTGGCAAGCACCAAC
CCCATAAAGTGACACAGTACAAGAAGGGCAA 16 (0.000415%)

CTCACTCCTTGGCGCCTGCCTGATCCTCCAAATCACCACAGGACTATTCCTAGCCATGCACTACTCACCA
GACGCCTCAACCGCCTTTTCATCAATCGCC 6 (0.000156%)

CTCAGAAGGCTAAATGAATATTATCCCTAATACCTGCCACCCCACTCTTAATCAGTGGTGGAAGAACGG
TCTCAGAACTGTTTGTTTCAATTGGCCATTT 11 (0.000286%)

CTCAGCCCTCCTAATGACCTCCGGCCTAGCCATGTGATTTCACTTCCACTCCATAACGCTCCTCATACTA
GGCCTACTAACCAACACACTAACCATATAC 27 (0.000701%)

CTCAGGTGATTATCCTGAACCATCCAGGCCAAATAAGCGCCGGCTATGCCCCTGTATTGGATTGCCACA
CGGCTCACATTGCATGCAAGTTTGCTGAGCT 6 (0.000156%)

CTCATATCAACATTGTCGTCATTGGACACGTAGATTCGGGCAAGTCCACCACTACTGGCCATCTGATCTA
TAAATGCGGTGGCATCGACAAAAGAACCAT 265 (0.006881%)

CTCCAGAAGAGGAGAAGAGGAAACACAAGAAGAAACGCCTGGTGCAGAGCCCCAATTCCTACTTCATG
GATGTGAAATGCCCAGGATGCTATAAAATCAC5 (0.000130%)

CTCCAGTCAACGTTACAACGGAAGTAAAATCTGTCGAAATGCACCATGAAGCTTTGAGTGAAGCTCTTC
CTGGGGACAATGTGGGCTTCAATGTCAAGAA 30 (0.000779%)

CTCCCACTAATAGCTTTTTGATGACTTCTAGCAAGCCTCGCTAACCTCGCCTTACCCCCCACTATTAACC
TACTGGGAGAACTCTCTGTGCTAGTAACCA 3 (0.000078%)

CTCCCAGACATGGGTCCCTCGGCTTCCTGCCTCGGAAGCGCAGCAGCAGGCATCGTGGGAAGGTGAAG
AGCTTCCCTAAGGATGACCCGTCCAAGCCGGT 3 (0.000078%)

CTCCCGAGGAGCACCCCGTGCTGCTGACCGAGGCCCCCCTGAACCCCAAGGCCAACCGCGAGAAGATG
ACCCAGATCATGTTTGAGACCTTCAACACCCC 6 (0.000156%)

CTCCCTCACCAAAGCCCATAAAAATAAAAAATTATAACAAACCCTGAGAACCAAAATGAACGAAAATC
TGTTCGCTTCATTCATTGCCCCCACAATCCTA 215 (0.005582%)

CTCCGCATGATGAAACTTCGGCTCACTCCTTGGCGCCTGCCTGATCCTCCAAATCACCACAGGACTATTC

CTAGCCATGCACTACTCACCAGACGCCTCA 23 (0.000597%)

CTCCGCCGCAGACGCCGCCGCGATGCGCTACGTCGCCTCCTACCTGCTGGCTGCCCTAGGGGGCAACTC
CTCCCCCAGCGCCAAGGACATCAAGAAGATC 515 (0.013372%)

CTCCGCTCCCAGACATGGGTCCCTCGGCTTCCTGCCTCGGAAGCGCAGCAGCAGGCATCGTGGGAAGGT
GAAGAGCTTCCCTAAGGATGACCCGTCCAAG 10 (0.000260%)

CTCCTAATGACCTCCGGCCTAGCCATGTGATTTCACTTCCACTCCATAACGCTCCTCATACTAGGCCTAC
TAACCAACACACTAACCATATACCAATGAT 14 (0.000364%)

CTCCTTCATCCCTCTCCAGAAGAGGAGAAGAGGAAACACAAGAAGAAACGCCTGGTGCAGAGCCCCAA
TTCCTACTTCATGGATGTGAAATGCCCAGGAT 18 (0.000467%)

CTCCTTGCTCGCCGCAGCCGCCTCCGCCGCGCGCCTCCTCCGCCGCCGCGGACTCCGGCAGCTTTATCGC
CAGAGTCCCTGAACTCTCGCTTTCTTTTTA 3 (0.000078%)

CTCCTTGGCGCCTGCCTGATCCTCCAAATCACCACAGGACTATTCCTAGCCATGCACTACTCACCAGACG
CCTCAACCGCCTTTTCATCAATCGCCCACA 3 (0.000078%)

CTCGCAAAGGATCTCCTTCATCCCTCTCCAGAAGAGGAGAAGAGGAAACACAAGAAGAAACGCCTGGT
GCAGAGCCCCAATTCCTACTTCATGGATGTGA 7 (0.000182%)

CTCGCCGCAGCCGCCTCCGCCGCGCGCCTCCTCCGCCGCCGCGGACTCCGGCAGCTTTATCGCCAGAGT
CCCTGAACTCTCGCTTTCTTTTTAATCCCCT 11 (0.000286%)

CTCGGCTTCCTGCCTCGGAAGCGCAGCAGCAGGCATCGTGGGAAGGTGAAGAGCTTCCCTAAGGATGA
CCCGTCCAAGCCGGTCCACCTCACAGCCTTCC 21 (0.000545%)

CTCGGGCACGCTACGAGAGTACAAGGTAGTGGGTCGCTGCCTGCCCACCCCCAAATGCCACACGCCGCC
CCTCTACCGCATGCGAATCTTTGCGCCTAAT 22 (0.000571%)

CTCTACTCCCACTAATAGCTTTTTGATGACTTCTAGCAAGCCTCGCTAACCTCGCCTTACCCCCCACTATT
AACCTACTGGGAGAACTCTCTGTGCTAGT 7 (0.000182%)

CTCTCAAGGACTTCAAACTCTACTCCCACTAATAGCTTTTTGATGACTTCTAGCAAGCCTCGCTAACCTC
GCCTTACCCCCCACTATTAACCTACTGGGA 481 (0.012489%)

CTCTCAGCCCTCCTAATGACCTCCGGCCTAGCCATGTGATTTCACTTCCACTCCATAACGCTCCTCATAC
TAGGCCTACTAACCAACACACTAACCATAT 9 (0.000234%)

CTCTCGCAAAGGATCTCCTTCATCCCTCTCCAGAAGAGGAGAAGAGGAAACACAAGAAGAAACGCCTG
GTGCAGAGCCCCAATTCCTACTTCATGGATGT 22 (0.000571%)

CTGAACGCAGGCACATACTTCCTATTCTACACCCTAGTAGGCTCCCTTCCCCTACTCATCGCACTGATTT
ACACTCACAACACCCTAGGCTCACTAAACA 21 (0.000545%)

CTGAGATGGGAAAGGGCTCCTTCAAGTATGCCTGGGTCTTGGATAAACTGAAAGCTGAGCGTGAACGT
GGTATCACCATTGATATCTCCTTGTGGAAATT 44 (0.001142%)

CTGAGCGTGCCTACCAAAAGCAGCCGACCATCTTTCAAAACAAGAAGAGGGTCCTGCTGGGAGAAACT
GGCAAGGAGAAGCTCCCGCGGTACTACAAGAA 11 (0.000286%)

CTGATCCGTCCTAATCACAGCAGTCCTACTTCTCCTATCTCTCCCAGTCCTAGCTGCTGGCATCACTATA
CTACTAACAGACCGCAACCTCAACACCACC 17 (0.000441%)

CTGATCGGCGCACTGCGAGCAGTAGCCCAAACAATCTCATATGAAGTCACCCTAGCCATCATTCTACTA
TCAACATTACTAATAAGTGGCTCCTTTAACC 6 (0.000156%)

CTGCCATCAACGAAGTGGTAACCCGAGAATACACCATCAACATTCACAAGCGCATCCATGGAGTGGGC
TTCAAGAAGCGTGCACCTCGGGCACTCAAAGA 21 (0.000545%)

CTGCCCAGAAAGCTCAGAAGGCTAAATGAATATTATCCCTAATACCTGCCACCCCACTCTTAATCAGTG
GTGGAAGAACGGTCTCAGAACTGTTTGTTTC 31 (0.000805%)

CTGCCGTCGCCGCCGCCACCATGCCCAAGAGAAAGGCTGAAGGGGATGCTAAGGGAGATAAAGCAAAG
GTGAAGGACGAACCACAGAGAAGATCCGCGAG 5 (0.000130%)

CTGCTAGGAAGCTCCGTAGTCACCGACGAGACCAGAAGTGGCATGATAAACAGTATAAGAAAGCTCAT
TTGGGCACAGCCCTAAAGGCCAACCCTTTTGG 13 (0.000338%)

CTGCTGAGATGGGAAAGGGCTCCTTCAAGTATGCCTGGGTCTTGGATAAACTGAAAGCTGAGCGTGAAC
GTGGTATCACCATTGATATCTCCTTGTGGAA 16 (0.000415%)

CTGCTGGAGCTGGCAAGGTCACCAAGTCTGCCCAGAAAGCTCAGAAGGCTAAATGAATATTATCCCTAA
TACCTGCCACCCCACTCTTAATCAGTGGTGG 8 (0.000208%)

CTGGAAGATGGCCCTAAATTCTTGAAGTCTGGTGATGCTGCCATTGTTGATATGGTTCCTGGCAAGCCC
ATGTGTGTTGAGAGCTTCTCAGACTATCCAC 100 (0.002596%)

CTGGAGCTGGCAAGGTCACCAAGTCTGCCCAGAAAGCTCAGAAGGCTAAATGAATATTATCCCTAATAC
CTGCCACCCCACTCTTAATCAGTGGTGGAAG 15 (0.000389%)

CTGGCAAGGTCACCAAGTCTGCCCAGAAAGCTCAGAAGGCTAAATGAATATTATCCCTAATACCTGCCA
CCCCACTCTTAATCAGTGGTGGAAGAACGGT 7 (0.000182%)

CTGGCTTCACTGCTCAGGTGATTATCCTGAACCATCCAGGCCAAATAAGCGCCGGCTATGCCCCTGTATT
GGATTGCCACACGGCTCACATTGCATGCAA 25 (0.000649%)

CTGGGGGCCTCTCTCAAGGATGAGGTTTTGAAGATTATGCCAGTGCAGAAGCAGACCCGTGCCGGCCAG
CGCACCAGGTTCAAGGCATTTGTTGCTATCG 16 (0.000415%)

CTGGTAAAAAGCTGGAAGATGGCCCTAAATTCTTGAAGTCTGGTGATGCTGCCATTGTTGATATGGTTC
CTGGCAAGCCCATGTGTGTTGAGAGCTTCTC 32 (0.000831%)

CTGTCAAGGATGTTCGTCGTGGCAACGTTGCTGGTGACAGCAAAAATGACCCACCAATGGAAGCAGCT
GGCTTCACTGCTCAGGTGATTATCCTGAACCA 11 (0.000286%)

CTGTCGAAATGCACCATGAAGCTTTGAGTGAAGCTCTTCCTGGGGACAATGTGGGCTTCAATGTCAAGA
ATGTGTCTGTCAAGGATGTTCGTCGTGGCAA 36 (0.000935%)

CTTCAAACTCTACTCCCACTAATAGCTTTTTGATGACTTCTAGCAAGCCTCGCTAACCTCGCCTTACCCC
CCACTATTAACCTACTGGGAGAACTCTCTG 13 (0.000338%)

CTTCAATGTCAAGAATGTGTCTGTCAAGGATGTTCGTCGTGGCAACGTTGCTGGTGACAGCAAAAATGA
CCCACCAATGGAAGCAGCTGGCTTCACTGCT 162 (0.004206%)

CTTCACTGCTCAGGTGATTATCCTGAACCATCCAGGCCAAATAAGCGCCGGCTATGCCCCTGTATTGGA
TTGCCACACGGCTCACATTGCATGCAAGTTT 19 (0.000493%)

CTTCAGAGGAAACAAATGGTCATTGATGTCCTTCACCCCGGGAAGGCGACAGTGCCTAAGACAGAAAT
TCGGGAAAAACTAGCCAAAATGTACAAGACCA 59 (0.001532%)

CTTCATCCCTCTCCAGAAGAGGAGAAGAGGAAACACAAGAAGAAACGCCTGGTGCAGAGCCCCAATTC
CTACTTCATGGATGTGAAATGCCCAGGATGCT 37 (0.000961%)

CTTCCACCCTTACTACACAATCAAAGACGCCCTCGGCTTACTTCTCTTCATTCTCTCCTTAATGACATTAA
CACTATTCTCACCAGACCTCCTAGGCGAC 7 (0.000182%)

CTTCCGACCGAAGCAAGAATCGCAAAAGGCATTTCAATGCACCTTCCCACATTCGAAGGAAGATTATGT
CTTCCCCTCTTTCCAAAGAGCTGAGACAGAA 3 (0.000078%)

CTTCCTGGGGGCCTCTCTCAAGGATGAGGTTTTGAAGATTATGCCAGTGCAGAAGCAGACCCGTGCCGG
CCAGCGCACCAGGTTCAAGGCATTTGTTGCT 4 (0.000104%)

CTTCCTTCGGTCGTCCCGAATCCGGGTTCATCCGACACCAGCCGCCTCCACCATGCCGCCGAAGTTCGAC
CCCAACGAGATCAAAGTCGTATACCTGAGG 46 (0.001194%)

CTTCGATACGGGATAATCCTATTTATTACCTCAGAAGTTTTTTTCTTCGCAGGATTTTTCTGAGCCTTTTA
CCACTCCAGCCTAGCCCCTACCCCCCAAT 973 (0.025264%)

CTTCGGCTCACTCCTTGGCGCCTGCCTGATCCTCCAAATCACCACAGGACTATTCCTAGCCATGCACTAC
TCACCAGACGCCTCAACCGCCTTTTCATCA 32 (0.000831%)

CTTCGGTCGTCCCGAATCCGGGTTCATCCGACACCAGCCGCCTCCACCATGCCGCCGAAGTTCGACCCC
AACGAGATCAAAGTCGTATACCTGAGGTGCA 28 (0.000727%)

CTTCGTACTGCTAGGAAGCTCCGTAGTCACCGACGAGACCAGAAGTGGCATGATAAACAGTATAAGAA
AGCTCATTTGGGCACAGCCCTAAAGGCCAACC959 (0.024900%)

CTTCGTCTGATCCGTCCTAATCACAGCAGTCCTACTTCTCCTATCTCTCCCAGTCCTAGCTGCTGGCATCA
CTATACTACTAACAGACCGCAACCTCAAC 948 (0.024615%)

CTTCTAGCAAGCCTCGCTAACCTCGCCTTACCCCCCACTATTAACCTACTGGGAGAACTCTCTGTGCTAG
TAACCACGTTCTCCTGATCAAATATCACTC 15 (0.000389%)

CTTCTGGCTTACACACTGGGTGTGAAACAACTAATTGTCGGTGTTAACAAAATGGATTCCACTGAGCCA
CCCTACAGCCAGAAGAGATATGAGGAAATTG 23 (0.000597%)

CTTCTTCATAGTAATACCCATCATAATCGGAGGCTTTGGCAACTGACTAGTTCCCCTAATAATCGGTGCC
CCCGATATGGCGTTTCCCCGCATAAACAAC 49 (0.001272%)

CTTGAAGTCTGGTGATGCTGCCATTGTTGATATGGTTCCTGGCAAGCCCATGTGTGTTGAGAGCTTCTCA
GACTATCCACCTTTGGGTCGCTTTGCTGTT245 (0.006361%)

CTTGCTCGCCGCAGCCGCCTCCGCCGCGCGCCTCCTCCGCCGCCGCGGACTCCGGCAGCTTTATCGCCA
GAGTCCCTGAACTCTCGCTTTCTTTTTAATC 15 (0.000389%)

CTTGGCGCCTGCCTGATCCTCCAAATCACCACAGGACTATTCCTAGCCATGCACTACTCACCAGACGCCT
CAACCGCCTTTTCATCAATCGCCCACATCA 7 (0.000182%)

CTTGTTGCACCGTGGAGGCCACAGGAGCAGAAACATGGAATGCCAGACGCTGGGGATGCTGGTACAAG
TTGTGGGACTGCATGCTACTGTCTAGAGCTTG 5 (0.000130%)

CTTTCAAAACAAGAAGAGGGTCCTGCTGGGAGAAACTGGCAAGGAGAAGCTCCCGCGGTACTACAAGA
ACATCGGTCTGGGCTTCAAGACACCCAAGGAG 4 (0.000104%)

CTTTGCTCCAGTCAACGTTACAACGGAAGTAAAATCTGTCGAAATGCACCATGAAGCTTTGAGTGAAGC
TCTTCCTGGGGACAATGTGGGCTTCAATGTC 1121 (0.029107%)

CTTTGTGACTTCCGACCGAAGCAAGAATCGCAAAAGGCATTTCAATGCACCTTCCCACATTCGAAGGAA
GATTATGTCTTCCCCTCTTTCCAAAGAGCTG 6 (0.000156%)

CTTTTTGATGACTTCTAGCAAGCCTCGCTAACCTCGCCTTACCCCCCACTATTAACCTACTGGGAGAACT

CTCTGTGCTAGTAACCACGTTCTCCTGATC 9 (0.000234%)

GAAAAAGGGCCGTTCTGCCATCAACGAAGTGGTAACCCGAGAATACACCATCAACATTCACAAGCGCA
TCCATGGAGTGGGCTTCAAGAAGCGTGCACCT 31 (0.000805%)

GAAAACTACCCCTAAAAGCCAAAATGGGAAAGGAAAAGACTCATATCAACATTGTCGTCATTGGACAC
GTAGATTCGGGCAAGTCCACCACTACTGGCCA 130 (0.003375%)

GAAAAGACTCATATCAACATTGTCGTCATTGGACACGTAGATTCGGGCAAGTCCACCACTACTGGCCAT
CTGATCTATAAATGCGGTGGCATCGACAAAA 42 (0.001091%)

GAAACAAATGGTCATTGATGTCCTTCACCCCGGGAAGGCGACAGTGCCTAAGACAGAAATTCGGGAAA
AACTAGCCAAAATGTACAAGACCACACCGGAT 4 (0.000104%)

GAAACTTCGGCTCACTCCTTGGCGCCTGCCTGATCCTCCAAATCACCACAGGACTATTCCTAGCCATGCA
CTACTCACCAGACGCCTCAACCGCCTTTTC 17 (0.000441%)

GAAAGATGAGGCAGAGGTCCAAGTAAACCGCTAGCTTGTTGCACCGTGGAGGCCACAGGAGCAGAAAC
ATGGAATGCCAGACGCTGGGGATGCTGGTACA 28 (0.000727%)

GAAAGCTCAGAAGGCTAAATGAATATTATCCCTAATACCTGCCACCCCACTCTTAATCAGTGGTGGAAG
AACGGTCTCAGAACTGTTTGTTTCAATTGGC 13 (0.000338%)

GAAAGGAAAAGACTCATATCAACATTGTCGTCATTGGACACGTAGATTCGGGCAAGTCCACCACTACTG
GCCATCTGATCTATAAATGCGGTGGCATCGA 10 (0.000260%)

GAAAGTTCTCCGCTCCCAGACATGGGTCCCTCGGCTTCCTGCCTCGGAAGCGCAGCAGCAGGCATCGTG
GGAAGGTGAAGAGCTTCCCTAAGGATGACCC 48 (0.001246%)

GAAATTTTCACAATGTCCGGAGCCCTTGATGTCCTGCAAATGAAGGAGGAGGATGTCCTTAAGTTCCTT
GCAGCAGGAACCCACTTAGGTGGCACCAATC 11 (0.000286%)

GAACACAGGTGTCGTGAAAACTACCCCTAAAAGCCAAAATGGGAAAGGAAAAGACTCATATCAACATT
GTCGTCATTGGACACGTAGATTCGGGCAAGTC 45 (0.001168%)

GAACATGCCTCTCGCAAAGGATCTCCTTCATCCCTCTCCAGAAGAGGAGAAGAGGAAACACAAGAAGA
AACGCCTGGTGCAGAGCCCCAATTCCTACTTC 11 (0.000286%)

GAACCTGATCTCTTATACTAGTATCCTTAATCATTTTTATTGCCACAACTAACCTCCTCGGACTCCTGCCT
CACTCATTTACACCAACCACCCAACTATC 6 (0.000156%)

GAACGCAGGCACATACTTCCTATTCTACACCCTAGTAGGCTCCCTTCCCCTACTCATCGCACTGATTTAC
ACTCACAACACCCTAGGCTCACTAAACATT 3 (0.000078%)

GAAGAAAAAGGGCCGTTCTGCCATCAACGAAGTGGTAACCCGAGAATACACCATCAACATTCACAAGC
GCATCCATGGAGTGGGCTTCAAGAAGCGTGCA 4 (0.000104%)

GAAGAAAGATGAGGCAGAGGTCCAAGTAAACCGCTAGCTTGTTGCACCGTGGAGGCCACAGGAGCAGA
AACATGGAATGCCAGACGCTGGGGATGCTGGT 4 (0.000104%)

GAAGAGGATGGAGATGAAGATGAGGAAGCTGAGTCAGCTACGGGCAAGCGGGCAGCTGAAGATGATG
AGGATGACGATGTCGATACCAAGAAGCAGAAGA 6 (0.000156%)

GAAGATATGCTCATGTGGTGTTGAGGAAAGCAGACATTGACCTCACCAAGAGGGCGGGAGAACTCACT
GAGGATGAGGTGGAACGTGTGATCACCATTAT10 (0.000260%)

GAAGATGAGGAAGCTGAGTCAGCTACGGGCAAGCGGGCAGCTGAAGATGATGAGGATGACGATGTCG
ATACCAAGAAGCAGAAGACCGACGAGGATGACT 3 (0.000078%)

GAAGATGGCCCTAAATTCTTGAAGTCTGGTGATGCTGCCATTGTTGATATGGTTCCTGGCAAGCCCATGT
GTGTTGAGAGCTTCTCAGACTATCCACCTT 10 (0.000260%)

GAAGCAGCTGGCTTCACTGCTCAGGTGATTATCCTGAACCATCCAGGCCAAATAAGCGCCGGCTATGCC
CCTGTATTGGATTGCCACACGGCTCACATTG 17 (0.000441%)

GAAGCTGAGTCAGCTACGGGCAAGCGGGCAGCTGAAGATGATGAGGATGACGATGTCGATACCAAGAA
GCAGAAGACCGACGAGGATGACTAGACAGCAA 14 (0.000364%)

GAAGGCCTCGGGCACGCTACGAGAGTACAAGGTAGTGGGTCGCTGCCTGCCCACCCCCAAATGCCACA
CGCCGCCCCTCTACCGCATGCGAATCTTTGCG 12 (0.000312%)

GAAGGCTAAATGAATATTATCCCTAATACCTGCCACCCCACTCTTAATCAGTGGTGGAAGAACGGTCTC
AGAACTGTTTGTTTCAATTGGCCATTTAAGT 4 (0.000104%)

GAAGGTGATGGTGAGGAAGAGGATGGAGATGAAGATGAGGAAGCTGAGTCAGCTACGGGCAAGCGGG
CAGCTGAAGATGATGAGGATGACGATGTCGATA 16 (0.000415%)

GAAGTAAAATCTGTCGAAATGCACCATGAAGCTTTGAGTGAAGCTCTTCCTGGGGACAATGTGGGCTTC
AATGTCAAGAATGTGTCTGTCAAGGATGTTC 10 (0.000260%)

GAAGTCTGGTGATGCTGCCATTGTTGATATGGTTCCTGGCAAGCCCATGTGTGTTGAGAGCTTCTCAGAC
TATCCACCTTTGGGTCGCTTTGCTGTTCGT 15 (0.000389%)

GAAGTTTAATCCCTTTGTGACTTCCGACCGAAGCAAGAATCGCAAAAGGCATTTCAATGCACCTTCCCA
CATTCGAAGGAAGATTATGTCTTCCCCTCTT 8 (0.000208%)

GAATGCGCAGGCTGAAGCGCAAAAGAAGAAAGATGAGGCAGAGGTCCAAGTAAACCGCTAGCTTGTTG
CACCGTGGAGGCCACAGGAGCAGAAACATGGA 27 (0.000701%)

GAATGTGTCTGTCAAGGATGTTCGTCGTGGCAACGTTGCTGGTGACAGCAAAAATGACCCACCAATGGA
AGCAGCTGGCTTCACTGCTCAGGTGATTATC 15 (0.000389%)

GACATGGGTCCCTCGGCTTCCTGCCTCGGAAGCGCAGCAGCAGGCATCGTGGGAAGGTGAAGAGCTTC
CCTAAGGATGACCCGTCCAAGCCGGTCCACCT 7 (0.000182%)

GACATTCAGACTGAGCGTGCCTACCAAAAGCAGCCGACCATCTTTCAAAACAAGAAGAGGGTCCTGCT
GGGAGAAACTGGCAAGGAGAAGCTCCCGCGGT 5 (0.000130%)

GACCCACCAATGGAAGCAGCTGGCTTCACTGCTCAGGTGATTATCCTGAACCATCCAGGCCAAATAAGC
GCCGGCTATGCCCCTGTATTGGATTGCCACA 6 (0.000156%)

GACCCCTAACAGGGGCCCTCTCAGCCCTCCTAATGACCTCCGGCCTAGCCATGTGATTTCACTTCCACTC
CATAACGCTCCTCATACTAGGCCTACTAAC 9 (0.000234%)

GACCCGCTATGGGGCCTCCCTCCGGAAAATGGTGAAGAAAATTGAAATCAGCCAGCACGCCAAGTACA
CTTGCTCTTTCTGTGGCAAAACCAAGATGAAG 4 (0.000104%)

GACCTATTCACCCTCCACTTCCCGTCTCAGAATCTAAACGTGGTCACCTTCGAGTAGAGAGGCCCGCCC
GCCCACCGTGGGCAGTGCCACCCGCAGATGA 25 (0.000649%)

GACTGAGCGTGCCTACCAAAAGCAGCCGACCATCTTTCAAAACAAGAAGAGGGTCCTGCTGGGAGAAA
CTGGCAAGGAGAAGCTCCCGCGGTACTACAAG 10 (0.000260%)

GAGAACATGCCTCTCGCAAAGGATCTCCTTCATCCCTCTCCAGAAGAGGAGAAGAGGAAACACAAGAA
GAAACGCCTGGTGCAGAGCCCCAATTCCTACT 17 (0.000441%)

GAGAAGAAAAAGGGCCGTTCTGCCATCAACGAAGTGGTAACCCGAGAATACACCATCAACATTCACAA
GCGCATCCATGGAGTGGGCTTCAAGAAGCGTG 6 (0.000156%)

GAGAGCACGCCATGAAGGCCTCGGGCACGCTACGAGAGTACAAGGTAGTGGGTCGCTGCCTGCCCACC
CCCAAATGCCACACGCCGCCCCTCTACCGCAT 3 (0.000078%)

GAGAGCATGCCCTTCTGGCTTACACACTGGGTGTGAAACAACTAATTGTCGGTGTTAACAAAATGGATT
CCACTGAGCCACCCTACAGCCAGAAGAGATA 16 (0.000415%)

GAGAGTACAAGGTAGTGGGTCGCTGCCTGCCCACCCCCAAATGCCACACGCCGCCCCTCTACCGCATGC
GAATCTTTGCGCCTAATCATGTCGTCGCCAA 4 (0.000104%)

GAGATGAAGATGAGGAAGCTGAGTCAGCTACGGGCAAGCGGGCAGCTGAAGATGATGAGGATGACGA
TGTCGATACCAAGAAGCAGAAGACCGACGAGGA 7 (0.000182%)

GAGATGGGAAAGGGCTCCTTCAAGTATGCCTGGGTCTTGGATAAACTGAAAGCTGAGCGTGAACGTGG
TATCACCATTGATATCTCCTTGTGGAAATTTG 16 (0.000415%)

GAGCACGCCATGAAGGCCTCGGGCACGCTACGAGAGTACAAGGTAGTGGGTCGCTGCCTGCCCACCCC
CAAATGCCACACGCCGCCCCTCTACCGCATGC 17 (0.000441%)

GAGCATGCCCTTCTGGCTTACACACTGGGTGTGAAACAACTAATTGTCGGTGTTAACAAAATGGATTCC
ACTGAGCCACCCTACAGCCAGAAGAGATATG 13 (0.000338%)

GAGCGTGCCTACCAAAAGCAGCCGACCATCTTTCAAAACAAGAAGAGGGTCCTGCTGGGAGAAACTGG
CAAGGAGAAGCTCCCGCGGTACTACAAGAACA 5 (0.000130%)

GAGCTGGCAAGGTCACCAAGTCTGCCCAGAAAGCTCAGAAGGCTAAATGAATATTATCCCTAATACCTG
CCACCCCACTCTTAATCAGTGGTGGAAGAAC 8 (0.000208%)

GAGGAAACAAATGGTCATTGATGTCCTTCACCCCGGGAAGGCGACAGTGCCTAAGACAGAAATTCGGG
AAAAACTAGCCAAAATGTACAAGACCACACCG 6 (0.000156%)

GAGGAAGAGGATGGAGATGAAGATGAGGAAGCTGAGTCAGCTACGGGCAAGCGGGCAGCTGAAGATG
ATGAGGATGACGATGTCGATACCAAGAAGCAGA 15 (0.000389%)

GAGGAAGCTGAGTCAGCTACGGGCAAGCGGGCAGCTGAAGATGATGAGGATGACGATGTCGATACCAA
GAAGCAGAAGACCGACGAGGATGACTAGACAG 15 (0.000389%)

GAGGCAGAGGTCCAAGTAAACCGCTAGCTTGTTGCACCGTGGAGGCCACAGGAGCAGAAACATGGAAT
GCCAGACGCTGGGGATGCTGGTACAAGTTGTG4 (0.000104%)

GAGGTTTTGAAGATTATGCCAGTGCAGAAGCAGACCCGTGCCGGCCAGCGCACCAGGTTCAAGGCATTT
GTTGCTATCGGGGACTACAATGGCCACGTCG 3 (0.000078%)

GATAACCATACACAACACTAAAGGACGAACCTGATCTCTTATACTAGTATCCTTAATCATTTTTATTGCC
ACAACTAACCTCCTCGGACTCCTGCCTCAC 20 (0.000519%)

GATACATAGGTATGGTCTGAGCTATGATATCAATTGGCTTCCTAGGGTTTATCGTGTGAGCACACCATAT
ATTTACAGTAGGAATAGACGTAGACACACG 5 (0.000130%)

GATACGGGATAATCCTATTTATTACCTCAGAAGTTTTTTCTTCGCAGGATTTTTCTGAGCCTTTTACCAC
TCCAGCCTAGCCCCTACCCCCCAATTAGG 4 (0.000104%)

GATATGCTCATGTGGTGTTGAGGAAAGCAGACATTGACCTCACCAAGAGGGCGGGAGAACTCACTGAG
GATGAGGTGGAACGTGTGATCACCATTATGCA4 (0.000104%)

GATCCGTCCTAATCACAGCAGTCCTACTTCTCCTATCTCTCCCAGTCCTAGCTGCTGGCATCACTATACT

ACTAACAGACCGCAACCTCAACACCACCTT 3 (0.000078%)

GATCGCCGTTCTGGTAAAAAGCTGGAAGATGGCCCTAAATTCTTGAAGTCTGGTGATGCTGCCATTGTT
GATATGGTTCCTGGCAAGCCCATGTGTGTTG 33 (0.000857%)

GATGAAACTTCGGCTCACTCCTTGGCGCCTGCCTGATCCTCCAAATCACCACAGGACTATTCCTAGCCAT
GCACTACTCACCAGACGCCTCAACCGCCTT 6 (0.000156%)

GATGACTTCTAGCAAGCCTCGCTAACCTCGCCTTACCCCCCACTATTAACCTACTGGGAGAACTCTCTGT
GCTAGTAACCACGTTCTCCTGATCAAATAT 20 (0.000519%)

GATGAGGAAGCTGAGTCAGCTACGGGCAAGCGGGCAGCTGAAGATGATGAGGATGACGATGTCGATAC
CAAGAAGCAGAAGACCGACGAGGATGACTAGA 7 (0.000182%)

GATGAGGCAGAGGTCCAAGTAAACCGCTAGCTTGTTGCACCGTGGAGGCCACAGGAGCAGAAACATGG
AATGCCAGACGCTGGGGATGCTGGTACAAGTT10 (0.000260%)

GATGCCCGTCACCAAGTTGGGCCGCTTGGTCAAGGACATGAAGATCAAGTCCCTGGAGGAGATCTATCT
CTTCTCCCTGCCCATTAAGGAATCAGAGATC 24 (0.000623%)

GATGGAGATGAAGATGAGGAAGCTGAGTCAGCTACGGGCAAGCGGGCAGCTGAAGATGATGAGGATG
ACGATGTCGATACCAAGAAGCAGAAGACCGACG 5 (0.000130%)

GATGGCCCTAAATTCTTGAAGTCTGGTGATGCTGCCATTGTTGATATGGTTCCTGGCAAGCCCATGTGTG
TTGAGAGCTTCTCAGACTATCCACCTTTGG 32 (0.000831%)

GATGGGAAAGGGCTCCTTCAAGTATGCCTGGGTCTTGGATAAACTGAAAGCTGAGCGTGAACGTGGTAT
CACCATTGATATCTCCTTGTGGAAATTTGAG 23 (0.000597%)

GATGTTCGTCGTGGCAACGTTGCTGGTGACAGCAAAAATGACCCACCAATGGAAGCAGCTGGCTTCACT
GCTCAGGTGATTATCCTGAACCATCCAGGCC 6 (0.000156%)

GATTATCCTGAACCATCCAGGCCAAATAAGCGCCGGCTATGCCCCTGTATTGGATTGCCACACGGCTCA
CATTGCATGCAAGTTTGCTGAGCTGAAGGAA 5 (0.000130%)

GCAAAAATGACCCACCAATGGAAGCAGCTGGCTTCACTGCTCAGGTGATTATCCTGAACCATCCAGGCC
AAATAAGCGCCGGCTATGCCCCTGTATTGGA 166 (0.004310%)

GCAAAAGAAGAAAGATGAGGCAGAGGTCCAAGTAAACCGCTAGCTTGTTGCACCGTGGAGGCCACAGG
AGCAGAAACATGGAATGCCAGACGCTGGGGAT 12 (0.000312%)

GCAAAGAAGGGTGGCGAGAAGAAAAAGGGCCGTTCTGCCATCAACGAAGTGGTAACCCGAGAATACA
CCATCAACATTCACAAGCGCATCCATGGAGTGG 8 (0.000208%)

GCAAAGGATCTCCTTCATCCCTCTCCAGAAGAGGAGAAGAGGAAACACAAGAAGAAACGCCTGGTGCA
GAGCCCCAATTCCTACTTCATGGATGTGAAAT 6 (0.000156%)

GCAACGTTGCTGGTGACAGCAAAAATGACCCACCAATGGAAGCAGCTGGCTTCACTGCTCAGGTGATTA
TCCTGAACCATCCAGGCCAAATAAGCGCCGG 14 (0.000364%)

GCAACTTCCTTCGGTCGTCCCGAATCCGGGTTCATCCGACACCAGCCGCCTCCACCATGCCGCCGAAGT
TCGACCCCAACGAGATCAAAGTCGTATACCT 19 (0.000493%)

GCAAGCATGGTTAACGTCCCTAAAACCCGCCGGACTTTCTGTAAGAAGTGTGGCAAGCACCAACCCCAT
AAAGTGACACAGTACAAGAAGGGCAAGGATT 6 (0.000156%)

GCAAGGTCACCAAGTCTGCCCAGAAAGCTCAGAAGGCTAAATGAATATTATCCCTAATACCTGCCACCC
CACTCTTAATCAGTGGTGGAAGAACGGTCTC 8 (0.000208%)

GCAAGTCCACCACTACTGGCCATCTGATCTATAAATGCGGTGGCATCGACAAAAGAACCATTGAAAAAT TTGAGAAGGAGGCTGCTGAGATGGGAAAGGG 6 (0.000156%)

GCACACGAGAACATGCCTCTCGCAAAGGATCTCCTTCATCCCTCTCCAGAAGAGGAGAAGAGGAAACA CAAGAAGAAACGCCTGGTGCAGAGCCCCAATT 26 (0.000675%)

GCACATACTTCCTATTCTACACCCTAGTAGGCTCCCTTCCCCTACTCATCGCACTGATTTACACTCACAA CACCCTAGGCTCACTAAACATTCTACTACT 5 (0.000130%)

GCACGCCATGAAGGCCTCGGGCACGCTACGAGAGTACAAGGTAGTGGGTCGCTGCCTGCCCACCCCCA AATGCCACACGCCGCCCCTCTACCGCATGCGA 5 (0.000130%)

GCACTGCGAGCAGTAGCCCAAACAATCTCATATGAAGTCACCCTAGCCATCATTCTACTATCAACATTA CTAATAAGTGGCTCCTTTAACCTCTCCACCC 5 (0.000130%)

GCAGACGCCGCCGCGATGCGCTACGTCGCCTCCTACCTGCTGGCTGCCCTAGGGGGCAACTCCTCCCCC AGCGCCAAGGACATCAAGAAGATCTTGGACA 10 (0.000260%)

GCAGAGGTCCAAGTAAACCGCTAGCTTGTTGCACCGTGGAGGCCACAGGAGCAGAAACATGGAATGCC AGACGCTGGGGATGCTGGTACAAGTTGTGGGA 13 (0.000338%)

GCAGCTGGCTTCACTGCTCAGGTGATTATCCTGAACCATCCAGGCCAAATAAGCGCCGGCTATGCCCCT GTATTGGATTGCCACACGGCTCACATTGCAT 7 (0.000182%)

GCATGATGAAACTTCGGCTCACTCCTTGGCGCCTGCCTGATCCTCCAAATCACCACAGGACTATTCCTAG CCATGCACTACTCACCAGACGCCTCAACCG 10 (0.000260%)

GCATGCCCTTCTGGCTTACACACTGGGTGTGAAACAACTAATTGTCGGTGTTAACAAAATGGATTCCAC TGAGCCACCCTACAGCCAGAAGAGATATGAG 10 (0.000260%)

GCATGGTTAACGTCCCTAAAACCCGCCGGACTTTCTGTAAGAAGTGTGGCAAGCACCAACCCCATAAAG TGACACAGTACAAGAAGGGCAAGGATTCTCT 4 (0.000104%)

GCATTGTGGATGCAAATCTGAGCGTTCTCAACTTGGTTATTGTAAAAAAAGGAGAGAAGGATATTCCTG GACTGACTGATACTACAGTGCCTCGCCGCCT 6 (0.000156%)

GCCAAAATGGGAAAGGAAAAGACTCATATCAACATTGTCGTCATTGGACACGTAGATTCGGGCAAGTC CACCACTACTGGCCATCTGATCTATAAATGCG 25 (0.000649%)

GCCATGAAGGCCTCGGGCACGCTACGAGAGTACAAGGTAGTGGGTCGCTGCCTGCCCACCCCCAAATG CCACACGCCGCCCCTCTACCGCATGCGAATCT 12 (0.000312%)

GCCCAGAAAGCTCAGAAGGCTAAATGAATATTATCCCTAATACCTGCCACCCCACTCTTAATCAGTGGT GGAAGAACGGTCTCAGAACTGTTTGTTTCAA 9 (0.000234%)

GCCCATAAAAATAAAAAATTATAACAAACCCTGAGAACCAAAATGAACGAAAATCTGTTCGCTTCATTC ATTGCCCCCACAATCCTAGGCCTACCCGCCG 5 (0.000130%)

GCCCATGACCCCTAACAGGGGCCCTCTCAGCCCTCCTAATGACCTCCGGCCTAGCCATGTGATTTCACTT CCACTCCATAACGCTCCTCATACTAGGCCT 20 (0.000519%)

GCCCGTCACCAAGTTGGGCCGCTTGGTCAAGGACATGAAGATCAAGTCCCTGGAGGAGATCTATCTCTT CTCCCTGCCCATTAAGGAATCAGAGATCATT 16 (0.000415%)

GCCCTAAATTCTTGAAGTCTGGTGATGCTGCCATTGTTGATATGGTTCCTGGCAAGCCCATGTGTGTTGA GAGCTTCTCAGACTATCCACCTTTGGGTCG 9 (0.000234%)

GCCCTCCTAATGACCTCCGGCCTAGCCATGTGATTTCACTTCCACTCCATAACGCTCCTCATACTAGGCC
TACTAACCAACACACTAACCATATACCAAT 5 (0.000130%)

GCCCTCTCAGCCCTCCTAATGACCTCCGGCCTAGCCATGTGATTTCACTTCCACTCCATAACGCTCCTCA
TACTAGGCCTACTAACCAACACACTAACCA 27 (0.000701%)

GCCCTTCTGGCTTACACACTGGGTGTGAAACAACTAATTGTCGGTGTTAACAAAATGGATTCCACTGAG
CCACCCTACAGCCAGAAGAGATATGAGGAAA 16 (0.000415%)

GCCCTTGCGCCTGCCTCTCCAGGATGTCTACAAAATTGGTGGTATTGGTACTGTTCCTGTTGGCCGAGTG
GAGACTGGTGTTCTCAAACCCGGTATGGTG 3 (0.000078%)

GCCGACCATCTTTCAAAACAAGAAGAGGGTCCTGCTGGGAGAAACTGGCAAGGAGAAGCTCCCGCGGT
ACTACAAGAACATCGGTCTGGGCTTCAAGACA 10 (0.000260%)

GCCGCAGACGCCGCCGCGATGCGCTACGTCGCCTCCTACCTGCTGGCTGCCCTAGGGGGCAACTCCTCC
CCCAGCGCCAAGGACATCAAGAAGATCTTGG 4 (0.000104%)

GCCGCAGCCGCCTCCGCCGCGCGCCTCCTCCGCCGCCGCGGACTCCGGCAGCTTTATCGCCAGAGTCCC
TGAACTCTCGCTTTCTTTTTAATCCCCTGCA 21 (0.000545%)

GCCGCCGCGATGCGCTACGTCGCCTCCTACCTGCTGGCTGCCCTAGGGGGCAACTCCTCCCCCAGCGCC
AAGGACATCAAGAAGATCTTGGACAGCGTGG 16 (0.000415%)

GCCGCCGTGACCTATTCACCCTCCACTTCCCGTCTCAGAATCTAAACGTGGTCACCTTCGAGTAGAGAG
GCCCGCCCGCCCACCGTGGGCAGTGCCACCC 23 (0.000597%)

GCCGCCTCCGCCGCGCGCCTCCTCCGCCGCCGCGGACTCCGGCAGCTTTATCGCCAGAGTCCCTGAACT
CTCGCTTTCTTTTTAATCCCCTGCATCGGAT 19 (0.000493%)

GCCGCGATGCGCTACGTCGCCTCCTACCTGCTGGCTGCCCTAGGGGGCAACTCCTCCCCCAGCGCCAAG
GACATCAAGAAGATCTTGGACAGCGTGGGTA 4 (0.000104%)

GCCGCTTGGTCAAGGACATGAAGATCAAGTCCCTGGAGGAGATCTATCTCTTCTCCCTGCCCATTAAGG
AATCAGAGATCATTGATTTCTTCCTGGGGGC 19 (0.000493%)

GCCGTGACCTATTCACCCTCCACTTCCCGTCTCAGAATCTAAACGTGGTCACCTTCGAGTAGAGAGGCC
CGCCCGCCCACCGTGGGCAGTGCCACCCGCA 3 (0.000078%)

GCCGTTCTGCCATCAACGAAGTGGTAACCCGAGAATACACCATCAACATTCACAAGCGCATCCATGGAG
TGGGCTTCAAGAAGCGTGCACCTCGGGCACT 13 (0.000338%)

GCCGTTCTGGTAAAAAGCTGGAAGATGGCCCTAAATTCTTGAAGTCTGGTGATGCTGCCATTGTTGATA
TGGTTCCTGGCAAGCCCATGTGTGTTGAGAG 35 (0.000909%)

GCCTACCAAAAGCAGCCGACCATCTTTCAAAACAAGAAGAGGGTCCTGCTGGGAGAAACTGGCAAGGA
GAAGCTCCCGCGGTACTACAAGAACATCGGTC 18 (0.000467%)

GCCTCCTTGCTCGCCGCAGCCGCCTCCGCCGCGCGCCTCCTCCGCCGCCGCGGACTCCGGCAGCTTTATC
GCCAGAGTCCCTGAACTCTCGCTTTCTTTT 9 (0.000234%)

GCCTCGGGCACGCTACGAGAGTACAAGGTAGTGGGTCGCTGCCTGCCCACCCCCAAATGCCACACGCC
GCCCCTCTACCGCATGCGAATCTTTGCGCCTA 39 (0.001013%)

GCCTCTCGCAAAGGATCTCCTTCATCCCTCTCCAGAAGAGGAGAAGAGGAAACACAAGAAGAAACGCC
TGGTGCAGAGCCCCAATTCCTACTTCATGGAT 13 (0.000338%)

GCCTCTCTCAAGGATGAGGTTTTGAAGATTATGCCAGTGCAGAAGCAGACCCGTGCCGGCCAGCGCACC

AGGTTCAAGGCATTTGTTGCTATCGGGGACT 4 (0.000104%)

GCCTGAACGCAGGCACATACTTCCTATTCTACACCCTAGTAGGCTCCCTTCCCCTACTCATCGCACTGAT
TTACACTCACAACACCCTAGGCTCACTAAA 13 (0.000338%)

GCGAATGCGCAGGCTGAAGCGCAAAAGAAGAAAGATGAGGCAGAGGTCCAAGTAAACCGCTAGCTTG
TTGCACCGTGGAGGCCACAGGAGCAGAAACATG 1092 (0.028354%)

GCGAGAAGAAAAAGGGCCGTTCTGCCATCAACGAAGTGGTAACCCGAGAATACACCATCAACATTCAC
AAGCGCATCCATGGAGTGGGCTTCAAGAAGCG 4 (0.000104%)

GCGCAGGCTGAAGCGCAAAAGAAGAAAGATGAGGCAGAGGTCCAAGTAAACCGCTAGCTTGTTGCACC
GTGGAGGCCACAGGAGCAGAAACATGGAATGC 20 (0.000519%)

GCGGAGAGCACGCCATGAAGGCCTCGGGCACGCTACGAGAGTACAAGGTAGTGGGTCGCTGCCTGCCC
ACCCCCAAATGCCACACGCCGCCCCTCTACCG 429 (0.011139%)

GCGTCCCATGCCTCCATCTAGAAGAGATTATGATGATATGAGCCCTCGTCGAGGACCACCTCCCCCTCC
TCCCGGACGAGGCGGCCGGGGTGGTAGCAGA 5 (0.000130%)

GCTAAATGAATATTATCCCTAATACCTGCCACCCCACTCTTAATCAGTGGTGGAAGAACGGTCTCAGAA
CTGTTTGTTTCAATTGGCCATTTAAGTTTAG 36 (0.000935%)

GCTAGCTTGTTGCACCGTGGAGGCCACAGGAGCAGAAACATGGAATGCCAGACGCTGGGGATGCTGGT
ACAAGTTGTGGGACTGCATGCTACTGTCTAGA 30 (0.000779%)

GCTAGGAAGCTCCGTAGTCACCGACGAGACCAGAAGTGGCATGATAAACAGTATAAGAAAGCTCATTT
GGGCACAGCCCTAAAGGCCAACCCTTTTGGAG 4 (0.000104%)

GCTATGGGGCCTCCCTCCGGAAAATGGTGAAGAAAATTGAAATCAGCCAGCACGCCAAGTACACTTGC
TCTTTCTGTGGCAAAACCAAGATGAAGAGACG8 (0.000208%)

GCTCACGCAAGCATGGTTAACGTCCCTAAAACCCGCCGGACTTTCTGTAAGAAGTGTGGCAAGCACCAA
CCCCATAAAGTGACACAGTACAAGAAGGGCA 276 (0.007166%)

GCTCACTCCTTGGCGCCTGCCTGATCCTCCAAATCACCACAGGACTATTCCTAGCCATGCACTACTCACC
AGACGCCTCAACCGCCTTTTCATCAATCGC 4 (0.000104%)

GCTCAGAAGGCTAAATGAATATTATCCCTAATACCTGCCACCCCACTCTTAATCAGTGGTGGAAGAACG
GTCTCAGAACTGTTTGTTTCAATTGGCCATT 17 (0.000441%)

GCTCAGGTGATTATCCTGAACCATCCAGGCCAAATAAGCGCCGGCTATGCCCCTGTATTGGATTGCCAC
ACGGCTCACATTGCATGCAAGTTTGCTGAGC 9 (0.000234%)

GCTCCAGTCAACGTTACAACGGAAGTAAAATCTGTCGAAATGCACCATGAAGCTTTGAGTGAAGCTCTT
CCTGGGGACAATGTGGGCTTCAATGTCAAGA 5 (0.000130%)

GCTCGCCGCAGCCGCCTCCGCCGCGCGCCTCCTCCGCCGCCGCGGACTCCGGCAGCTTTATCGCCAGAG
TCCCTGAACTCTCGCTTTCTTTTTAATCCCC 6 (0.000156%)

GCTGAGATGGGAAAGGGCTCCTTCAAGTATGCCTGGGTCTTGGATAAACTGAAAGCTGAGCGTGAACG
TGGTATCACCATTGATATCTCCTTGTGGAAAT 26 (0.000675%)

GCTGCGTGTGGCTCCCGAGGAGCACCCCGTGCTGCTGACCGAGGCCCCCCTGAACCCCAAGGCCAACCG
CGAGAAGATGACCCAGATCATGTTTGAGACC 203 (0.005271%)

GCTGCTGAGATGGGAAAGGGCTCCTTCAAGTATGCCTGGGTCTTGGATAAACTGAAAGCTGAGCGTGA
ACGTGGTATCACCATTGATATCTCCTTGTGGA 16 (0.000415%)

GCTGCTGGAGCTGGCAAGGTCACCAAGTCTGCCCAGAAAGCTCAGAAGGCTAAATGAATATTATCCCTA
ATACCTGCCACCCCACTCTTAATCAGTGGTG 15 (0.000389%)

GCTGGAAGATGGCCCTAAATTCTTGAAGTCTGGTGATGCTGCCATTGTTGATATGGTTCCTGGCAAGCC
CATGTGTGTTGAGAGCTTCTCAGACTATCCA 21 (0.000545%)

GCTGGAGCTGGCAAGGTCACCAAGTCTGCCCAGAAAGCTCAGAAGGCTAAATGAATATTATCCCTAAT
ACCTGCCACCCCACTCTTAATCAGTGGTGGAA 23 (0.000597%)

GCTGGCAAGGTCACCAAGTCTGCCCAGAAAGCTCAGAAGGCTAAATGAATATTATCCCTAATACCTGCC
ACCCCACTCTTAATCAGTGGTGGAAGAACGG 5 (0.000130%)

GCTGGCTTCACTGCTCAGGTGATTATCCTGAACCATCCAGGCCAAATAAGCGCCGGCTATGCCCCTGTA
TTGGATTGCCACACGGCTCACATTGCATGCA 60 (0.001558%)

GCTGGTGACAGCAAAAATGACCCACCAATGGAAGCAGCTGGCTTCACTGCTCAGGTGATTATCCTGAAC
CATCCAGGCCAAATAAGCGCCGGCTATGCCC 17 (0.000441%)

GCTTCAATGTCAAGAATGTGTCTGTCAAGGATGTTCGTCGTGGCAACGTTGCTGGTGACAGCAAAAATG
ACCCACCAATGGAAGCAGCTGGCTTCACTGC 865 (0.022460%)

GCTTCACTGCTCAGGTGATTATCCTGAACCATCCAGGCCAAATAAGCGCCGGCTATGCCCCTGTATTGG
ATTGCCACACGGCTCACATTGCATGCAAGTT 4 (0.000104%)

GCTTGTTGCACCGTGGAGGCCACAGGAGCAGAAACATGGAATGCCAGACGCTGGGGATGCTGGTACAA
GTTGTGGGACTGCATGCTACTGTCTAGAGCTT 15 (0.000389%)

GCTTTTTGATGACTTCTAGCAAGCCTCGCTAACCTCGCCTTACCCCCCACTATTAACCTACTGGGAGAAC
TCTCTGTGCTAGTAACCACGTTCTCCTGAT7 (0.000182%)

GGAAAAGACTCATATCAACATTGTCGTCATTGGACACGTAGATTCGGGCAAGTCCACCACTACTGGCCA
TCTGATCTATAAATGCGGTGGCATCGACAAA 90 (0.002337%)

GGAAACAAATGGTCATTGATGTCCTTCACCCCGGGAAGGCGACAGTGCCTAAGACAGAAATTCGGGAA
AAACTAGCCAAAATGTACAAGACCACACCGGA 5 (0.000130%)

GGAAAGGAAAAGACTCATATCAACATTGTCGTCATTGGACACGTAGATTCGGGCAAGTCCACCACTACT
GGCCATCTGATCTATAAATGCGGTGGCATCG 28 (0.000727%)

GGAAAGGGCTCCTTCAAGTATGCCTGGGTCTTGGATAAACTGAAAGCTGAGCGTGAACGTGGTATCACC
ATTGATATCTCCTTGTGGAAATTTGAGACCA 202 (0.005245%)

GGAAATTTTCACAATGTCCGGAGCCCTTGATGTCCTGCAAATGAAGGAGGAGGATGTCCTTAAGTTCCT
TGCAGCAGGAACCCACTTAGGTGGCACCAAT 11 (0.000286%)

GGAAGAGGATGGAGATGAAGATGAGGAAGCTGAGTCAGCTACGGGCAAGCGGGCAGCTGAAGATGAT
GAGGATGACGATGTCGATACCAAGAAGCAGAAG 31 (0.000805%)

GGAAGATGGCCCTAAATTCTTGAAGTCTGGTGATGCTGCCATTGTTGATATGGTTCCTGGCAAGCCCAT
GTGTGTTGAGAGCTTCTCAGACTATCCACCT 183 (0.004752%)

GGAAGCAGCTGGCTTCACTGCTCAGGTGATTATCCTGAACCATCCAGGCCAAATAAGCGCCGGCTATGC
CCCTGTATTGGATTGCCACACGGCTCACATT 17 (0.000441%)

GGAAGCTCCGTAGTCACCGACGAGACCAGAAGTGGCATGATAAACAGTATAAGAAAGCTCATTTGGGC
ACAGCCCTAAAGGCCAACCCTTTTGGAGGTGC 21 (0.000545%)

GGAAGCTGAGTCAGCTACGGGCAAGCGGGCAGCTGAAGATGATGAGGATGACGATGTCGATACCAAGA
AGCAGAAGACCGACGAGGATGACTAGACAGCA 35 (0.000909%)

GGAAGTAAAATCTGTCGAAATGCACCATGAAGCTTTGAGTGAAGCTCTTCCTGGGGACAATGTGGGCTT
CAATGTCAAGAATGTGTCTGTCAAGGATGTT 19 (0.000493%)

GGACATTCAGACTGAGCGTGCCTACCAAAAGCAGCCGACCATCTTTCAAAACAAGAAGAGGGTCCTGC
TGGGAGAAACTGGCAAGGAGAAGCTCCCGCGG 985 (0.025575%)

GGACCCGCTATGGGGCCTCCCTCCGGAAAATGGTGAAGAAAATTGAAATCAGCCAGCACGCCAAGTAC
ACTTGCTCTTTCTGTGGCAAAACCAAGATGAA 10 (0.000260%)

GGACTTCAAACTCTACTCCCACTAATAGCTTTTGATGACTTCTAGCAAGCCTCGCTAACCTCGCCTTAC
CCCCCACTATTAACCTACTGGGAGAACTCT 9 (0.000234%)

GGAGAGCACGCCATGAAGGCCTCGGGCACGCTACGAGAGTACAAGGTAGTGGGTCGCTGCCTGCCCAC
CCCCAAATGCCACACGCCGCCCCTCTACCGCA 22 (0.000571%)

GGAGATGAAGATGAGGAAGCTGAGTCAGCTACGGGCAAGCGGGCAGCTGAAGATGATGAGGATGACG
ATGTCGATACCAAGAAGCAGAAGACCGACGAGG 16 (0.000415%)

GGAGCACCCCGTGCTGCTGACCGAGGCCCCCCTGAACCCCAAGGCCAACCGCGAGAAGATGACCCAGA
TCATGTTTGAGACCTTCAACACCCCAGCCATG 5 (0.000130%)

GGAGCTAGGAGTGGGAATAGCTTTGCGAAAAATGGGCGCAATGGCCAAGCCAGATTGTATCATCACTT
GTGATGGTAAAAACCTCACCATAAAAACTGAG 5 (0.000130%)

GGAGCTGGCAAGGTCACCAAGTCTGCCCAGAAAGCTCAGAAGGCTAAATGAATATTATCCCTAATACCT
GCCACCCCACTCTTAATCAGTGGTGGAAGAA 9 (0.000234%)

GGAGGCTGCTGAGATGGGAAAGGGCTCCTTCAAGTATGCCTGGGTCTTGGATAAACTGAAAGCTGAGC
GTGAACGTGGTATCACCATTGATATCTCCTTG 872 (0.022641%)

GGATACATAGGTATGGTCTGAGCTATGATATCAATTGGCTTCCTAGGGTTTATCGTGTGAGCACACCAT
ATATTTACAGTAGGAATAGACGTAGACACAC 8 (0.000208%)

GGATCTCCTTCATCCCTCTCCAGAAGAGGAGAAGAGGAAACACAAGAAGAAACGCCTGGTGCAGAGCC
CCAATTCCTACTTCATGGATGTGAAATGCCCA 4 (0.000104%)

GGATGAGGTTTTGAAGATTATGCCAGTGCAGAAGCAGACCCGTGCCGGCCAGCGCACCAGGTTCAAGG
CATTTGTTGCTATCGGGGACTACAATGGCCAC 6 (0.000156%)

GGATGCCCGTCACCAAGTTGGGCCGCTTGGTCAAGGACATGAAGATCAAGTCCCTGGAGGAGATCTATC
TCTTCTCCCTGCCCATTAAGGAATCAGAGAT 52 (0.001350%)

GGATGGAGATGAAGATGAGGAAGCTGAGTCAGCTACGGGCAAGCGGGCAGCTGAAGATGATGAGGAT
GACGATGTCGATACCAAGAAGCAGAAGACCGAC 4 (0.000104%)

GGATGTTCGTCGTGGCAACGTTGCTGGTGACAGCAAAAATGACCCACCAATGGAAGCAGCTGGCTTCAC
TGCTCAGGTGATTATCCTGAACCATCCAGGC 35 (0.000909%)

GGCAACGTTGCTGGTGACAGCAAAAATGACCCACCAATGGAAGCAGCTGGCTTCACTGCTCAGGTGATT
ATCCTGAACCATCCAGGCCAAATAAGCGCCG 34 (0.000883%)

GGCAAGGTCACCAAGTCTGCCCAGAAAGCTCAGAAGGCTAAATGAATATTATCCCTAATACCTGCCACC
CCACTCTTAATCAGTGGTGGAAGAACGGTCT 28 (0.000727%)

GGCAAGTCCACCACTACTGGCCATCTGATCTATAAATGCGGTGGCATCGACAAAAGAACCATTGAAAA

ATTTGAGAAGGAGGCTGCTGAGATGGGAAAGG 525 (0.013632%)

GGCACATACTTCCTATTCTACACCCTAGTAGGCTCCCTTCCCCTACTCATCGCACTGATTTACACTCACA
ACACCCTAGGCTCACTAAACATTCTACTAC 4 (0.000104%)

GGCACGCTACGAGAGTACAAGGTAGTGGGTCGCTGCCTGCCCACCCCCAAATGCCACACGCCGCCCCTC
TACCGCATGCGAATCTTTGCGCCTAATCATG 4 (0.000104%)

GGCAGAGGTCCAAGTAAACCGCTAGCTTGTTGCACCGTGGAGGCCACAGGAGCAGAAACATGGAATGC
CAGACGCTGGGGATGCTGGTACAAGTTGTGGG 48 (0.001246%)

GGCCCTAAATTCTTGAAGTCTGGTGATGCTGCCATTGTTGATATGGTTCCTGGCAAGCCCATGTGTGTTG
AGAGCTTCTCAGACTATCCACCTTTGGGTC 8 (0.000208%)

GGCCCTCTCAGCCCTCCTAATGACCTCCGGCCTAGCCATGTGATTTCACTTCCACTCCATAACGCTCCTC
ATACTAGGCCTACTAACCAACACACTAACC 9 (0.000234%)

GGCCGCTTGGTCAAGGACATGAAGATCAAGTCCCTGGAGGAGATCTATCTCTTCTCCCTGCCCATTAAG
GAATCAGAGATCATTGATTTCTTCCTGGGGG 22 (0.000571%)

GGCCGTTCTGCCATCAACGAAGTGGTAACCCGAGAATACACCATCAACATTCACAAGCGCATCCATGGA
GTGGGCTTCAAGAAGCGTGCACCTCGGGCAC 37 (0.000961%)

GGCCTCTCTCAAGGATGAGGTTTTGAAGATTATGCCAGTGCAGAAGCAGACCCGTGCCGGCCAGCGCAC
CAGGTTCAAGGCATTTGTTGCTATCGGGGAC 4 (0.000104%)

GGCGCACTGCGAGCAGTAGCCCAAACAATCTCATATGAAGTCACCCTAGCCATCATTCTACTATCAACA
TTACTAATAAGTGGCTCCTTTAACCTCTCCA 3 (0.000078%)

GGCGTCCCATGCCTCCATCTAGAAGAGATTATGATGATATGAGCCCTCGTCGAGGACCACCTCCCCCTC
CTCCCGGACGAGGCGGCCGGGGTGGTAGCAG 4 (0.000104%)

GGCTAAATGAATATTATCCCTAATACCTGCCACCCCACTCTTAATCAGTGGTGGAAGAACGGTCTCAGA
ACTGTTTGTTTCAATTGGCCATTTAAGTTTA 25 (0.000649%)

GGCTCACTCCTTGGCGCCTGCCTGATCCTCCAAATCACCACAGGACTATTCCTAGCCATGCACTACTCAC
CAGACGCCTCAACCGCCTTTTCATCAATCG 3 (0.000078%)

GGCTGCTGAGATGGGAAAGGGCTCCTTCAAGTATGCCTGGGTCTTGGATAAACTGAAAGCTGAGCGTG
AACGTGGTATCACCATTGATATCTCCTTGTGG 24 (0.000623%)

GGCTGCTGGAGCTGGCAAGGTCACCAAGTCTGCCCAGAAAGCTCAGAAGGCTAAATGAATATTATCCCT
AATACCTGCCACCCCACTCTTAATCAGTGGT 86 (0.002233%)

GGCTTCACTGCTCAGGTGATTATCCTGAACCATCCAGGCCAAATAAGCGCCGGCTATGCCCCTGTATTG
GATTGCCACACGGCTCACATTGCATGCAAGT 10 (0.000260%)

GGCTTCCTGCCTCGGAAGCGCAGCAGCAGGCATCGTGGGAAGGTGAAGAGCTTCCCTAAGGATGACCC
GTCCAAGCCGGTCCACCTCACAGCCTTCCTGG 7 (0.000182%)

GGGAAAGGAAAAGACTCATATCAACATTGTCGTCATTGGACACGTAGATTCGGGCAAGTCCACCACTA
CTGGCCATCTGATCTATAAATGCGGTGGCATC 36 (0.000935%)

GGGAAAGGGCTCCTTCAAGTATGCCTGGGTCTTGGATAAACTGAAAGCTGAGCGTGAACGTGGTATCAC
CATTGATATCTCCTTGTGGAAATTTGAGACC 94 (0.002441%)

GGGAAATTTTCACAATGTCCGGAGCCCTTGATGTCCTGCAAATGAAGGAGGAGGATGTCCTTAAGTTCC
TTGCAGCAGGAACCCACTTAGGTGGCACCAA 7 (0.000182%)

GGGACCCGCTATGGGGCCTCCCTCCGGAAAATGGTGAAGAAAATTGAAATCAGCCAGCACGCCAAGTACACTTGCTCTTTCTGTGGCAAAACCAAGATGA 15 (0.000389%)

GGGCACGCTACGAGAGTACAAGGTAGTGGGTCGCTGCCTGCCCACCCCCAAATGCCACACGCCGCCCCTCTACCGCATGCGAATCTTTGCGCCTAATCAT 8 (0.000208%)

GGGCCCTCTCAGCCCTCCTAATGACCTCCGGCCTAGCCATGTGATTTCACTTCCACTCCATAACGCTCCTCATACTAGGCCTACTAACCAACACACTAAC 28 (0.000727%)

GGGCCGCTTGGTCAAGGACATGAAGATCAAGTCCCTGGAGGAGATCTATCTCTTCTCCCTGCCCATTAAGGAATCAGAGATCATTGATTTCTTCCTGGGG 5 (0.000130%)

GGGCCGTTCTGCCATCAACGAAGTGGTAACCCGAGAATACACCATCAACATTCACAAGCGCATCCATGGAGTGGGCTTCAAGAAGCGTGCACCTCGGGCA 8 (0.000208%)

GGGCGTCCCATGCCTCCATCTAGAAGAGATTATGATGATATGAGCCCTCGTCGAGGACCACCTCCCCCTCCTCCCGGACGAGGCGGCCGGGGTGGTAGCA 3 (0.000078%)

GGGGCCCTCTCAGCCCTCCTAATGACCTCCGGCCTAGCCATGTGATTTCACTTCCACTCCATAACGCTCCTCATACTAGGCCTACTAACCAACACACTAA 35 (0.000909%)

GGGGCCTCTCTCAAGGATGAGGTTTTGAAGATTATGCCAGTGCAGAAGCAGACCCGTGCCGGCCAGCGCACCAGGTTCAAGGCATTTGTTGCTATCGGGG 6 (0.000156%)

GGGGGCCTCTCTCAAGGATGAGGTTTTGAAGATTATGCCAGTGCAGAAGCAGACCCGTGCCGGCCAGCGCACCAGGTTCAAGGCATTTGTTGCTATCGGG 7 (0.000182%)

GGGGGGGGGGGGGGGGGGGGGGGGGGGGGGGGGGGGGGGGGGGGGGGGGGGGGGGGGGGGGGGGGGGGGGGGGGGGGGGGGGGGGGGGGGGGGGGGGGGGGG 6369 (0.165370%)

GGGGGGGGGGGGGGGGGGGGGGGGGGGGGGGGGGGGGGGGGGGGGGGGGGGGGGGGGGGGGGGGGGGGGGGGGGGGGGGGGGGGGGGGGGGGGGGGGGGGGGGGGGGGGGGGGGGGGGGGGGGGGGGGGGGGGGGGGGGGGGGGGGGGGGGGGGG 5344 (0.206747%)

GGGTCCCTCGGCTTCCTGCCTCGGAAGCGCAGCAGCAGGCATCGTGGGAAGGTGAAGAGCTTCCCTAAGGATGACCCGTCCAAGCCGGTCCACCTCACAG3 (0.000078%)

GGGTGGCGAGAAGAAAAAGGGCCGTTCTGCCATCAACGAAGTGGTAACCCGAGAATACACCATCAACATTCACAAGCGCATCCATGGAGTGGGCTTCAAG5 (0.000130%)

GGGTGGGCGTCCCATGCCTCCATCTAGAAGAGATTATGATGATATGAGCCCTCGTCGAGGACCACCTCCCCCTCCTCCCGGACGAGGCGGCCGGGGTGGT 80 (0.002077%)

GGTAAAAAGCTGGAAGATGGCCCTAAATTCTTGAAGTCTGGTGATGCTGCCATTGTTGATATGGTTCCTGGCAAGCCCATGTGTGTTGAGAGCTTCTCAG 9 (0.000234%)

GGTATGGTCTGAGCTATGATATCAATTGGCTTCCTAGGGTTTATCGTGTGAGCACACCATATATTTACAGTAGGAATAGACGTAGACACACGAGCATATT 7 (0.000182%)

GGTCACCAAGTCTGCCCAGAAAGCTCAGAAGGCTAAATGAATATTATCCCTAATACCTGCCACCCCACTCTTAATCAGTGGTGGAAGAACGGTCTCAGAA 10 (0.000260%)

GGTCCAAGTAAACCGCTAGCTTGTTGCACCGTGGAGGCCACAGGAGCAGAAACATGGAATGCCAGACGCTGGGGATGCTGGTACAAGTTGTGGGACTGCA4 (0.000104%)

GGTCCCTCGGCTTCCTGCCTCGGAAGCGCAGCAGCAGGCATCGTGGGAAGGTGAAGAGCTTCCCTAAGGATGACCCGTCCAAGCCGGTCCACCTCACAGC10 (0.000260%)

GGTCTGAGCTATGATATCAATTGGCTTCCTAGGGTTTATCGTGTGAGCACACCATATATTTACAGTAGGA
ATAGACGTAGACACACGAGCATATTTCACC 4 (0.000104%)

GGTGAGGAAGAGGATGGAGATGAAGATGAGGAAGCTGAGTCAGCTACGGGCAAGCGGGCAGCTGAAG
ATGATGAGGATGACGATGTCGATACCAAGAAGC 20 (0.000519%)

GGTGATGGTGAGGAAGAGGATGGAGATGAAGATGAGGAAGCTGAGTCAGCTACGGGCAAGCGGGCAG
CTGAAGATGATGAGGATGACGATGTCGATACCA 11 (0.000286%)

GGTGATTATCCTGAACCATCCAGGCCAAATAAGCGCCGGCTATGCCCCTGTATTGGATTGCCACACGGC
TCACATTGCATGCAAGTTTGCTGAGCTGAAG 14 (0.000364%)

GGTGCAACTTCCTTCGGTCGTCCCGAATCCGGGTTCATCCGACACCAGCCGCCTCCACCATGCCGCCGA
AGTTCGACCCCAACGAGATCAAAGTCGTATA 5 (0.000130%)

GGTGGCGAGAAGAAAAAGGGCCGTTCTGCCATCAACGAAGTGGTAACCCGAGAATACACCATCAACAT
TCACAAGCGCATCCATGGAGTGGGCTTCAAGA 4 (0.000104%)

GGTGGGCGTCCCATGCCTCCATCTAGAAGAGATTATGATGATATGAGCCCTCGTCGAGGACCACCTCCC
CCTCCTCCCGGACGAGGCGGCCGGGGTGGTA 3 (0.000078%)

GGTGTCGTGAAAACTACCCCTAAAAGCCAAAATGGGAAAGGAAAAGACTCATATCAACATTGTCGTCA
TTGGACACGTAGATTCGGGCAAGTCCACCACT 53 (0.001376%)

GGTTAACGTCCCTAAAACCCGCCGGACTTTCTGTAAGAAGTGTGGCAAGCACCAACCCCATAAAGTGAC
ACAGTACAAGAAGGGCAAGGATTCTCTGTAC 18 (0.000467%)

GTAAAAAGCTGGAAGATGGCCCTAAATTCTTGAAGTCTGGTGATGCTGCCATTGTTGATATGGTTCCTG
GCAAGCCCATGTGTGTTGAGAGCTTCTCAGA 57 (0.001480%)

GTAAAACCCAGCCCATGACCCCTAACAGGGGCCCTCTCAGCCCTCCTAATGACCTCCGGCCTAGCCATG
TGATTTCACTTCCACTCCATAACGCTCCTCA 233 (0.006050%)

GTAAAATCTGTCGAAATGCACCATGAAGCTTTGAGTGAAGCTCTTCCTGGGGACAATGTGGGCTTCAAT
GTCAAGAATGTGTCTGTCAAGGATGTTCGTC 99 (0.002571%)

GTAATAATCTTCTTCATAGTAATACCCATCATAATCGGAGGCTTTGGCAACTGACTAGTTCCCCTAATAA
TCGGTGCCCCCGATATGGCGTTTCCCCGCA 3 (0.000078%)

GTACAAGGTAGTGGGTCGCTGCCTGCCCACCCCCAAATGCCACACGCCGCCCCTCTACCGCATGCGAAT
CTTTGCGCCTAATCATGTCGTCGCCAAGTCC 29 (0.000753%)

GTACTGCTAGGAAGCTCCGTAGTCACCGACGAGACCAGAAGTGGCATGATAAACAGTATAAGAAAGCT
CATTTGGGCACAGCCCTAAAGGCCAACCCTTT 5 (0.000130%)

GTATGGTCTGAGCTATGATATCAATTGGCTTCCTAGGGTTTATCGTGTGAGCACACCATATATTTACAGT
AGGAATAGACGTAGACACACGAGCATATTT 20 (0.000519%)

GTCAACGTTACAACGGAAGTAAAATCTGTCGAAATGCACCATGAAGCTTTGAGTGAAGCTCTTCCTGGG
GACAATGTGGGCTTCAATGTCAAGAATGTGT 11 (0.000286%)

GTCAAGAATGTGTCTGTCAAGGATGTTCGTCGTGGCAACGTTGCTGGTGACAGCAAAAATGACCCACCA
ATGGAAGCAGCTGGCTTCACTGCTCAGGTGA 23 (0.000597%)

GTCAAGGATGTTCGTCGTGGCAACGTTGCTGGTGACAGCAAAAATGACCCACCAATGGAAGCAGCTGG
CTTCACTGCTCAGGTGATTATCCTGAACCATC 9 (0.000234%)

GTCACCAAGTCTGCCCAGAAAGCTCAGAAGGCTAAATGAATATTATCCCTAATACCTGCCACCCCACTC
TTAATCAGTGGTGGAAGAACGGTCTCAGAAC 10 (0.000260%)

GTCACCAAGTTGGGCCGCTTGGTCAAGGACATGAAGATCAAGTCCCTGGAGGAGATCTATCTCTTCTCC
CTGCCCATTAAGGAATCAGAGATCATTGATT 15 (0.000389%)

GTCCAAGTAAACCGCTAGCTTGTTGCACCGTGGAGGCCACAGGAGCAGAAACATGGAATGCCAGACGC
TGGGGATGCTGGTACAAGTTGTGGGACTGCAT 12 (0.000312%)

GTCCACCACTACTGGCCATCTGATCTATAAATGCGGTGGCATCGACAAAAGAACCATTGAAAAATTTGA
GAAGGAGGCTGCTGAGATGGGAAAGGGCTCC 35 (0.000909%)

GTCCCTAAAACCCGCCGGACTTTCTGTAAGAAGTGTGGCAAGCACCAACCCCATAAAGTGACACAGTAC
AAGAAGGGCAAGGATTCTCTGTACGCCCAGG 3 (0.000078%)

GTCCCTCGGCTTCCTGCCTCGGAAGCGCAGCAGCAGGCATCGTGGGAAGGTGAAGAGCTTCCCTAAGG
ATGACCCGTCCAAGCCGGTCCACCTCACAGCC 3 (0.000078%)

GTCCTAATCACAGCAGTCCTACTTCTCCTATCTCTCCCAGTCCTAGCTGCTGGCATCACTATACTACTAA
CAGACCGCAACCTCAACACCACCTTCTTCG 19 (0.000493%)

GTCGAAATGCACCATGAAGCTTTGAGTGAAGCTCTTCCTGGGGACAATGTGGGCTTCAATGTCAAGAAT
GTGTCTGTCAAGGATGTTCGTCGTGGCAACG 91 (0.002363%)

GTCGTCCCGAATCCGGGTTCATCCGACACCAGCCGCCTCCACCATGCCGCCGAAGTTCGACCCCAACGA
GATCAAAGTCGTATACCTGAGGTGCACCGGA 6 (0.000156%)

GTCGTGAAAACTACCCCTAAAAGCCAAAATGGGAAAGGAAAAGACTCATATCAACATTGTCGTCATTG
GACACGTAGATTCGGGCAAGTCCACCACTACT 22 (0.000571%)

GTCGTGGCAACGTTGCTGGTGACAGCAAAAATGACCCACCAATGGAAGCAGCTGGCTTCACTGCTCAG
GTGATTATCCTGAACCATCCAGGCCAAATAAG 41 (0.001065%)

GTCTGAGCTATGATATCAATTGGCTTCCTAGGGTTTATCGTGTGAGCACACCATATATTTACAGTAGGAA
TAGACGTAGACACACGAGCATATTTCACCT 16 (0.000415%)

GTCTGATCCGTCCTAATCACAGCAGTCCTACTTCTCCTATCTCTCCCAGTCCTAGCTGCTGGCATCACTA
TACTACTAACAGACCGCAACCTCAACACCA 28 (0.000727%)

GTCTGCCCAGAAAGCTCAGAAGGCTAAATGAATATTATCCCTAATACCTGCCACCCCACTCTTAATCAG
TGGTGGAAGAACGGTCTCAGAACTGTTTGTT 29 (0.000753%)

GTCTGTCAAGGATGTTCGTCGTGGCAACGTTGCTGGTGACAGCAAAAATGACCCACCAATGGAAGCAG
CTGGCTTCACTGCTCAGGTGATTATCCTGAAC 8 (0.000208%)

GTGAAAACTACCCCTAAAAGCCAAAATGGGAAAGGAAAAGACTCATATCAACATTGTCGTCATTGGAC
ACGTAGATTCGGGCAAGTCCACCACTACTGGC 344 (0.008932%)

GTGAATTTGAAGCTGGTATCTCCAAGAATGGGCAGACCCGAGAGCATGCCCTTCTGGCTTACACACTGG
GTGTGAAACAACTAATTGTCGGTGTTAACAA 773 (0.020071%)

GTGACCTATTCACCCTCCACTTCCCGTCTCAGAATCTAAACGTGGTCACCTTCGAGTAGAGAGGCCCGC
CCGCCCACCGTGGGCAGTGCCACCCGCAGAT 6 (0.000156%)

GTGACTTCCGACCGAAGCAAGAATCGCAAAAGGCATTTCAATGCACCTTCCCACATTCGAAGGAAGATT
ATGTCTTCCCCTCTTTCCAAAGAGCTGAGAC 5 (0.000130%)

GTGAGGAAGAGGATGGAGATGAAGATGAGGAAGCTGAGTCAGCTACGGGCAAGCGGGCAGCTGAAGA

TGATGAGGATGACGATGTCGATACCAAGAAGCA 34 (0.000883%)

GTGATGGTGAGGAAGAGGATGGAGATGAAGATGAGGAAGCTGAGTCAGCTACGGGCAAGCGGGCAGC
TGAAGATGATGAGGATGACGATGTCGATACCAA 47 (0.001220%)

GTGATTATCCTGAACCATCCAGGCCAAATAAGCGCCGGCTATGCCCCTGTATTGGATTGCCACACGGCT
CACATTGCATGCAAGTTTGCTGAGCTGAAGG 7 (0.000182%)

GTGCAACTTCCTTCGGTCGTCCCGAATCCGGGTTCATCCGACACCAGCCGCCTCCACCATGCCGCCGAA
GTTCGACCCCAACGAGATCAAAGTCGTATAC 56 (0.001454%)

GTGCCTACCAAAAGCAGCCGACCATCTTTCAAAACAAGAAGAGGGTCCTGCTGGGAGAAACTGGCAAG
GAGAAGCTCCCGCGGTACTACAAGAACATCGG 5 (0.000130%)

GTGGATGCCCGTCACCAAGTTGGGCCGCTTGGTCAAGGACATGAAGATCAAGTCCCTGGAGGAGATCT
ATCTCTTCTCCCTGCCCATTAAGGAATCAGAG 659 (0.017111%)

GTGGCAACGTTGCTGGTGACAGCAAAAATGACCCACCAATGGAAGCAGCTGGCTTCACTGCTCAGGTG
ATTATCCTGAACCATCCAGGCCAAATAAGCGC 56 (0.001454%)

GTGGCGAGAAGAAAAAGGGCCGTTCTGCCATCAACGAAGTGGTAACCCGAGAATACACCATCAACATT
CACAAGCGCATCCATGGAGTGGGCTTCAAGAA 4 (0.000104%)

GTGGTTGCATTGTGGATGCAAATCTGAGCGTTCTCAACTTGGTTATTGTAAAAAAAGGAGAGAAGGATA
TTCCTGGACTGACTGATACTACAGTGCCTCG 4 (0.000104%)

GTGTCGTGAAAACTACCCCTAAAAGCCAAAATGGGAAAGGAAAAGACTCATATCAACATTGTCGTCAT
TGGACACGTAGATTCGGGCAAGTCCACCACTA 34 (0.000883%)

GTGTCTGTCAAGGATGTTCGTCGTGGCAACGTTGCTGGTGACAGCAAAAATGACCCACCAATGGAAGCA
GCTGGCTTCACTGCTCAGGTGATTATCCTGA 15 (0.000389%)

GTTAACGTCCCTAAAACCCGCCGGACTTTCTGTAAGAAGTGTGGCAAGCACCAACCCCATAAAGTGACA
CAGTACAAGAAGGGCAAGGATTCTCTGTACG 6 (0.000156%)

GTTACAACGGAAGTAAAATCTGTCGAAATGCACCATGAAGCTTTGAGTGAAGCTCTTCCTGGGGACAAT
GTGGGCTTCAATGTCAAGAATGTGTCTGTCA 35 (0.000909%)

GTTCATCCGACACCAGCCGCCTCCACCATGCCGCCGAAGTTCGACCCCAACGAGATCAAAGTCGTATAC
CTGAGGTGCACCGGAGGTGAAGTCGGTGCCA 6 (0.000156%)

GTTCGTCGTGGCAACGTTGCTGGTGACAGCAAAAATGACCCACCAATGGAAGCAGCTGGCTTCACTGCT
CAGGTGATTATCCTGAACCATCCAGGCCAAA 46 (0.001194%)

GTTCGTGGTTGCATTGTGGATGCAAATCTGAGCGTTCTCAACTTGGTTATTGTAAAAAAAGGAGAGAAG
GATATTCCTGGACTGACTGATACTACAGTGC 404 (0.010490%)

GTTCTCCGCTCCCAGACATGGGTCCCTCGGCTTCCTGCCTCGGAAGCGCAGCAGCAGGCATCGTGGGAA
GGTGAAGAGCTTCCCTAAGGATGACCCGTCC 6 (0.000156%)

GTTCTGCCATCAACGAAGTGGTAACCCGAGAATACACCATCAACATTCACAAGCGCATCCATGGAGTGG
GCTTCAAGAAGCGTGCACCTCGGGCACTCAA 11 (0.000286%)

GTTCTGGTAAAAAGCTGGAAGATGGCCCTAAATTCTTGAAGTCTGGTGATGCTGCCATTGTTGATATGG
TTCCTGGCAAGCCCATGTGTGTTGAGAGCTT 52 (0.001350%)

GTTGCACCGTGGAGGCCACAGGAGCAGAAACATGGAATGCCAGACGCTGGGGATGCTGGTACAAGTTG
TGGGACTGCATGCTACTGTCTAGAGCTTGTCT 5 (0.000130%)

GTTGCATTGTGGATGCAAATCTGAGCGTTCTCAACTTGGTTATTGTAAAAAAAGGAGAGAAGGATATTC
CTGGACTGACTGATACTACAGTGCCTCGCCG 7 (0.000182%)

GTTGCTGGTGACAGCAAAAATGACCCACCAATGGAAGCAGCTGGCTTCACTGCTCAGGTGATTATCCTG
AACCATCCAGGCCAAATAAGCGCCGGCTATG 16 (0.000415%)

GTTGGGCCGCTTGGTCAAGGACATGAAGATCAAGTCCCTGGAGGAGATCTATCTCTTCTCCCTGCCCAT
TAAGGAATCAGAGATCATTGATTTCTTCCTG 16 (0.000415%)

GTTTAATCCCTTTGTGACTTCCGACCGAAGCAAGAATCGCAAAAGGCATTTCAATGCACCTTCCCACATT
CGAAGGAAGATTATGTCTTCCCCTCTTTCC 79 (0.002051%)

GTTTTGAAGATTATGCCAGTGCAGAAGCAGACCCGTGCCGGCCAGCGCACCAGGTTCAAGGCATTTGTT
GCTATCGGGGACTACAATGGCCACGTCGGTC 7 (0.000182%)

TAAAAAATTATAACAAACCCTGAGAACCAAAATGAACGAAAATCTGTTCGCTTCATTCATTGCCCCCAC
AATCCTAGGCCTACCCGCCGCAGTACTGATC 5 (0.000130%)

TAAAAAGCTGGAAGATGGCCCTAAATTCTTGAAGTCTGGTGATGCTGCCATTGTTGATATGGTTCCTGG
CAAGCCCATGTGTGTTGAGAGCTTCTCAGAC 31 (0.000805%)

TAAAAATAAAAAATTATAACAAACCCTGAGAACCAAAATGAACGAAAATCTGTTCGCTTCATTCATTGC
CCCCACAATCCTAGGCCTACCCGCCGCAGTA 55 (0.001428%)

TAAAACCCAGCCCATGACCCCTAACAGGGGCCCTCTCAGCCCTCCTAATGACCTCCGGCCTAGCCATGT
GATTTCACTTCCACTCCATAACGCTCCTCAT 24 (0.000623%)

TAAAACCCGCCGGACTTTCTGTAAGAAGTGTGGCAAGCACCAACCCCATAAAGTGACACAGTACAAGA
AGGGCAAGGATTCTCTGTACGCCCAGGGAAAG 11 (0.000286%)

TAAAAGCCAAAATGGGAAAGGAAAAGACTCATATCAACATTGTCGTCATTGGACACGTAGATTCGGGC
AAGTCCACCACTACTGGCCATCTGATCTATAA 34 (0.000883%)

TAAAATCTGTCGAAATGCACCATGAAGCTTTGAGTGAAGCTCTTCCTGGGGACAATGTGGGCTTCAATG
TCAAGAATGTGTCTGTCAAGGATGTTCGTCG 10 (0.000260%)

TAAACCGCTAGCTTGTTGCACCGTGGAGGCCACAGGAGCAGAAACATGGAATGCCAGACGCTGGGGAT
GCTGGTACAAGTTGTGGGACTGCATGCTACTG 52 (0.001350%)

TAAAGGACGAACCTGATCTCTTATACTAGTATCCTTAATCATTTTTATTGCCACAACTAACCTCCTCGGA
CTCCTGCCTCACTCATTTACACCAACCACC 34 (0.000883%)

TAAAGGGAAATTTTCACAATGTCCGGAGCCCTTGATGTCCTGCAAATGAAGGAGGAGGATGTCCTTAAG
TTCCTTGCAGCAGGAACCCACTTAGGTGGCA 563 (0.014618%)

TAAATTCTTGAAGTCTGGTGATGCTGCCATTGTTGATATGGTTCCTGGCAAGCCCATGTGTGTTGAGAGC
TTCTCAGACTATCCACCTTTGGGTCGCTTT 20 (0.000519%)

TAACAAACCCTGAGAACCAAAATGAACGAAAATCTGTTCGCTTCATTCATTGCCCCCACAATCCTAGGC
CTACCCGCCGCAGTACTGATCATTCTATTTC 7 (0.000182%)

TAACACTCACAACAAAACTAACTAATACTAACATCTCAGACGCTCAGGAAATAGAAACCGTCTGAACT
ATCCTGCCCGCCATCATCCTAGTCCTCATCGC 11 (0.000286%)

TAACAGGGGCCCTCTCAGCCCTCCTAATGACCTCCGGCCTAGCCATGTGATTTCACTTCCACTCCATAAC
GCTCCTCATACTAGGCCTACTAACCAACAC 31 (0.000805%)

TAACCATACACAACACTAAAGGACGAACCTGATCTCTTATACTAGTATCCTTAATCATTTTTATTGCCAC AACTAACCTCCTCGGACTCCTGCCTCACTC 14 (0.000364%)

TAACCGCTAACATTACTGCAGGCCACCTACTCATGCACCTAATTGGAAGCGCCACCCTAGCAATATCAA CCATTAACCTTCCCTCTACACTTATCATCTT 5 (0.000130%)

TAACCTCAAAACAAATGATAACCATACACAACACTAAAGGACGAACCTGATCTCTTATACTAGTATCCT TAATCATTTTTATTGCCACAACTAACCTCCT 5 (0.000130%)

TAATAATCTTCTTCATAGTAATACCCATCATAATCGGAGGCTTTGGCAACTGACTAGTTCCCCTAATAAT CGGTGCCCCCGATATGGCGTTTCCCCGCAT 11 (0.000286%)

TAATACCTGCCACCCCACTCTTAATCAGTGGTGGAAGAACGGTCTCAGAACTGTTTGTTTCAATTGGCCA TTTAAGTTTAGTAGTAAAAGACTGGTTAAT 4 (0.000104%)

TAATAGCTTTTTGATGACTTCTAGCAAGCCTCGCTAACCTCGCCTTACCCCCCACTATTAACCTACTGGG AGAACTCTCTGTGCTAGTAACCACGTTCTC 3 (0.000078%)

TAATCACAGCAGTCCTACTTCTCCTATCTCTCCCAGTCCTAGCTGCTGGCATCACTATACTACTAACAGA CCGCAACCTCAACACCACCTTCTTCGACCC 6 (0.000156%)

TAATCCCTTTGTGACTTCCGACCGAAGCAAGAATCGCAAAAGGCATTTCAATGCACCTTCCCACATTCG AAGGAAGATTATGTCTTCCCCTCTTTCCAAA 3 (0.000078%)

TAATCTTCTTCATAGTAATACCCATCATAATCGGAGGCTTTGGCAACTGACTAGTTCCCCTAATAATCGG TGCCCCCGATATGGCGTTTCCCCGCATAAA 4 (0.000104%)

TAATGACCTCCGGCCTAGCCATGTGATTTCACTTCCACTCCATAACGCTCCTCATACTAGGCCTACTAAC CAACACACTAACCATATACCAATGATGGCG 6 (0.000156%)

TACAACGGAAGTAAAATCTGTCGAAATGCACCATGAAGCTTTGAGTGAAGCTCTTCCTGGGGACAATGT GGGCTTCAATGTCAAGAATGTGTCTGTCAAG 8 (0.000208%)

TACAACGTTATCGTCACAGCCCATGCATTTGTAATAATCTTCTTCATAGTAATACCCATCATAATCGGAG GCTTTGGCAACTGACTAGTTCCCCTAATAA 10 (0.000260%)

TACACAACACTAAAGGACGAACCTGATCTCTTATACTAGTATCCTTAATCATTTTTATTGCCACAACTAA CCTCCTCGGACTCCTGCCTCACTCATTTAC 4 (0.000104%)

TACATAGGTATGGTCTGAGCTATGATATCAATTGGCTTCCTAGGGTTTATCGTGTGAGCACACCATATAT TTACAGTAGGAATAGACGTAGACACACGAG 8 (0.000208%)

TACCAAAAGCAGCCGACCATCTTTCAAAACAAGAAGAGGGTCCTGCTGGGAGAAACTGGCAAGGAGAA GCTCCCGCGGTACTACAAGAACATCGGTCTGG 3 (0.000078%)

TACCCCTAAAAGCCAAAATGGGAAAGGAAAAGACTCATATCAACATTGTCGTCATTGGACACGTAGAT TCGGGCAAGTCCACCACTACTGGCCATCTGAT 7 (0.000182%)

TACCTGCCACCCCACTCTTAATCAGTGGTGGAAGAACGGTCTCAGAACTGTTTGTTTCAATTGGCCATTT AAGTTTAGTAGTAAAAGACTGGTTAATGAT 7 (0.000182%)

TACGCACACGAGAACATGCCTCTCGCAAAGGATCTCCTTCATCCCTCTCCAGAAGAGGAGAAGAGGAA ACACAAGAAGAAACGCCTGGTGCAGAGCCCCA 6 (0.000156%)

TACGCCCTGATCGGCGCACTGCGAGCAGTAGCCCAAACAATCTCATATGAAGTCACCCTAGCCATCATT CTACTATCAACATTACTAATAAGTGGCTCCT 214 (0.005556%)

TAGCTTGTTGCACCGTGGAGGCCACAGGAGCAGAAACATGGAATGCCAGACGCTGGGGATGCTGGTAC

AAGTTGTGGGACTGCATGCTACTGTCTAGAGC 3 (0.000078%)

TAGGAAGCTCCGTAGTCACCGACGAGACCAGAAGTGGCATGATAAACAGTATAAGAAAGCTCATTTGG
GCACAGCCCTAAAGGCCAACCCTTTTGGAGGT 12 (0.000312%)

TAGGTATGGTCTGAGCTATGATATCAATTGGCTTCCTAGGGTTTATCGTGTGAGCACACCATATATTTAC
AGTAGGAATAGACGTAGACACACGAGCATA 3 (0.000078%)

TAGTAAAACCCAGCCCATGACCCCTAACAGGGGCCCTCTCAGCCCTCCTAATGACCTCCGGCCTAGCCA
TGTGATTTCACTTCCACTCCATAACGCTCCT 4 (0.000104%)

TATAACAAACCCTGAGAACCAAAATGAACGAAAATCTGTTCGCTTCATTCATTGCCCCCACAATCCTAG
GCCTACCCGCCGCAGTACTGATCATTCTATT 13 (0.000338%)

TATAGTAAAACCCAGCCCATGACCCCTAACAGGGGCCCTCTCAGCCCTCCTAATGACCTCCGGCCTAGC
CATGTGATTTCACTTCCACTCCATAACGCTC 787 (0.020434%)

TATCACCCTATAGAAGAACTAATGTTAGTATAAGTAACATGAAAACATTCTCCTCCGCATAAGCCTGCG
TCAGATTAAAACACTGAACTGACAATTAACA 19 (0.000493%)

TATCCCTAATACCTGCCACCCCACTCTTAATCAGTGGTGGAAGAACGGTCTCAGAACTGTTTGTTTCAAT
TGGCCATTTAAGTTTAGTAGTAAAAGACTG 16 (0.000415%)

TATGCTCATGTGGTGTTGAGGAAAGCAGACATTGACCTCACCAAGAGGGCGGGAGAACTCACTGAGGA
TGAGGTGGAACGTGTGATCACCATTATGCAGA 10 (0.000260%)

TATGGTCTGAGCTATGATATCAATTGGCTTCCTAGGGTTTATCGTGTGAGCACACCATATATTTACAGTA
GGAATAGACGTAGACACACGAGCATATTTC 14 (0.000364%)

TATTATCCCTAATACCTGCCACCCCACTCTTAATCAGTGGTGGAAGAACGGTCTCAGAACTGTTTGTTTC
AATTGGCCATTTAAGTTTAGTAGTAAAAGA 57 (0.001480%)

TCAAAACAAATGATAACCATACACAACACTAAAGGACGAACCTGATCTCTTATACTAGTATCCTTAATC
ATTTTTATTGCCACAACTAACCTCCTCGGAC 6 (0.000156%)

TCAAAACAAGAAGAGGGTCCTGCTGGGAGAAACTGGCAAGGAGAAGCTCCCGCGGTACTACAAGAAC
ATCGGTCTGGGCTTCAAGACACCCAAGGAGGCT 6 (0.000156%)

TCAAACTCTACTCCCACTAATAGCTTTTTGATGACTTCTAGCAAGCCTCGCTAACCTCGCCTTACCCCCC
ACTATTAACCTACTGGGAGAACTCTCTGTG 8 (0.000208%)

TCAAGAATGTGTCTGTCAAGGATGTTCGTCGTGGCAACGTTGCTGGTGACAGCAAAAATGACCCACCAA
TGGAAGCAGCTGGCTTCACTGCTCAGGTGAT 6 (0.000156%)

TCAAGGACTTCAAACTCTACTCCCACTAATAGCTTTTTGATGACTTCTAGCAAGCCTCGCTAACCTCGCC
TTACCCCCCACTATTAACCTACTGGGAGAA 5 (0.000130%)

TCAAGGATGAGGTTTTGAAGATTATGCCAGTGCAGAAGCAGACCCGTGCCGGCCAGCGCACCAGGTTC
AAGGCATTTGTTGCTATCGGGGACTACAATGG 3 (0.000078%)

TCAAGGATGTTCGTCGTGGCAACGTTGCTGGTGACAGCAAAAATGACCCACCAATGGAAGCAGCTGGC
TTCACTGCTCAGGTGATTATCCTGAACCATCC 3 (0.000078%)

TCAATGTCAAGAATGTGTCTGTCAAGGATGTTCGTCGTGGCAACGTTGCTGGTGACAGCAAAAATGACC
CACCAATGGAAGCAGCTGGCTTCACTGCTCA 9 (0.000234%)

TCACAACAAAACTAACTAATACTAACATCTCAGACGCTCAGGAAATAGAAACCGTCTGAACTATCCTGC
CCGCCATCATCCTAGTCCTCATCGCCCTCCC 33 (0.000857%)

TCACCAAAGCCCATAAAAATAAAAAATTATAACAAACCCTGAGAACCAAAATGAACGAAAATCTGTTC
GCTTCATTCATTGCCCCCACAATCCTAGGCCT 5 (0.000130%)

TCACCAAGTTGGGCCGCTTGGTCAAGGACATGAAGATCAAGTCCCTGGAGGAGATCTATCTCTTCTCCC
TGCCCATTAAGGAATCAGAGATCATTGATTT 3 (0.000078%)

TCACCTTCCACCCTTACTACACAATCAAAGACGCCCTCGGCTTACTTCTCTTCATTCTCTCCTTAATGACA
TTAACACTATTCTCACCAGACCTCCTAGG 10 (0.000260%)

TCAGAAGGCTAAATGAATATTATCCCTAATACCTGCCACCCCACTCTTAATCAGTGGTGGAAGAACGGT
CTCAGAACTGTTTGTTTCAATTGGCCATTTA 8 (0.000208%)

TCAGACTGAGCGTGCCTACCAAAAGCAGCCGACCATCTTTCAAAACAAGAAGAGGGTCCTGCTGGGAG
AAACTGGCAAGGAGAAGCTCCCGCGGTACTAC 10 (0.000260%)

TCAGCCCTCCTAATGACCTCCGGCCTAGCCATGTGATTTCACTTCCACTCCATAACGCTCCTCATACTAG
GCCTACTAACCAACACACTAACCATATACC 3 (0.000078%)

TCAGGTGATTATCCTGAACCATCCAGGCCAAATAAGCGCCGGCTATGCCCCTGTATTGGATTGCCACAC
GGCTCACATTGCATGCAAGTTTGCTGAGCTG 5 (0.000130%)

TCATATCAACATTGTCGTCATTGGACACGTAGATTCGGGCAAGTCCACCACTACTGGCCATCTGATCTAT
AAATGCGGTGGCATCGACAAAAGAACCATT 4 (0.000104%)

TCATCCCTCTCCAGAAGAGGAGAAGAGGAAACACAAGAAGAAACGCCTGGTGCAGAGCCCCAATTCCT
ACTTCATGGATGTGAAATGCCCAGGATGCTAT 3 (0.000078%)

TCCAAGTAAACCGCTAGCTTGTTGCACCGTGGAGGCCACAGGAGCAGAAACATGGAATGCCAGACGCT
GGGGATGCTGGTACAAGTTGTGGGACTGCATG21 (0.000545%)

TCCACCACTACTGGCCATCTGATCTATAAATGCGGTGGCATCGACAAAAGAACCATTGAAAAATTTGAG
AAGGAGGCTGCTGAGATGGGAAAGGGCTCCT 10 (0.000260%)

TCCACCCTTACTACACAATCAAAGACGCCCTCGGCTTACTTCTCTTCATTCTCTCCTTAATGACATTAAC
ACTATTCTCACCAGACCTCCTAGGCGACCC 6 (0.000156%)

TCCAGTCAACGTTACAACGGAAGTAAAATCTGTCGAAATGCACCATGAAGCTTTGAGTGAAGCTCTTCC
TGGGGACAATGTGGGCTTCAATGTCAAGAAT 6 (0.000156%)

TCCCACTAATAGCTTTTTGATGACTTCTAGCAAGCCTCGCTAACCTCGCCTTACCCCCCACTATTAACCT
ACTGGGAGAACTCTCTGTGCTAGTAACCAC 4 (0.000104%)

TCCCTCACCAAAGCCCATAAAAATAAAAAATTATAACAAACCCTGAGAACCAAAATGAACGAAAATCT
GTTCGCTTCATTCATTGCCCCCACAATCCTAG 43 (0.001116%)

TCCCTCTCCAGAAGAGGAGAAGAGGAAACACAAGAAGAAACGCCTGGTGCAGAGCCCCAATTCCTACT
TCATGGATGTGAAATGCCCAGGATGCTATAAA3 (0.000078%)

TCCGACACCAGCCGCCTCCACCATGCCGCCGAAGTTCGACCCCAACGAGATCAAAGTCGTATACCTGAG
GTGCACCGGAGGTGAAGTCGGTGCCACTTCT 3 (0.000078%)

TCCGACCGAAGCAAGAATCGCAAAAGGCATTTCAATGCACCTTCCCACATTCGAAGGAAGATTATGTCT
TCCCCTCTTTCCAAAGAGCTGAGACAGAAGT 7 (0.000182%)

TCCGCATGATGAAACTTCGGCTCACTCCTTGGCGCCTGCCTGATCCTCCAAATCACCACAGGACTATTCC
TAGCCATGCACTACTCACCAGACGCCTCAA 3 (0.000078%)

TCCGCCTCCTTGCTCGCCGCAGCCGCCTCCGCCGCGCGCCTCCTCCGCCGCCGCGGACTCCGGCAGCTTT
ATCGCCAGAGTCCCTGAACTCTCGCTTTCT 581 (0.015086%)

TCCTAATCACAGCAGTCCTACTTCTCCTATCTCTCCCAGTCCTAGCTGCTGGCATCACTATACTACTAAC
AGACCGCAACCTCAACACCACCTTCTTCGA 9 (0.000234%)

TCCTAATGACCTCCGGCCTAGCCATGTGATTTCACTTCCACTCCATAACGCTCCTCATACTAGGCCTACT
AACCAACACACTAACCATATACCAATGATG 6 (0.000156%)

TCCTTCGGTCGTCCCGAATCCGGGTTCATCCGACACCAGCCGCCTCCACCATGCCGCCGAAGTTCGACC
CCAACGAGATCAAAGTCGTATACCTGAGGTG 4 (0.000104%)

TCGAAATGCACCATGAAGCTTTGAGTGAAGCTCTTCCTGGGGACAATGTGGGCTTCAATGTCAAGAATG
TGTCTGTCAAGGATGTTCGTCGTGGCAACGT 25 (0.000649%)

TCGATACGGGATAATCCTATTTATTACCTCAGAAGTTTTTTTCTTCGCAGGATTTTTCTGAGCCTTTTACC
ACTCCAGCCTAGCCCCTACCCCCCAATTA 4 (0.000104%)

TCGCAAAGGATCTCCTTCATCCCTCTCCAGAAGAGGAGAAGAGGAAACACAAGAAGAAACGCCTGGTG
CAGAGCCCCAATTCCTACTTCATGGATGTGAA 5 (0.000130%)

TCGCCGCAGCCGCCTCCGCCGCGCGCCTCCTCCGCCGCCGCGGACTCCGGCAGCTTTATCGCCAGAGTC
CCTGAACTCTCGCTTTCTTTTTAATCCCCTG 3 (0.000078%)

TCGCCGTTCTGGTAAAAAGCTGGAAGATGGCCCTAAATTCTTGAAGTCTGGTGATGCTGCCATTGTTGA
TATGGTTCCTGGCAAGCCCATGTGTGTTGAG 28 (0.000727%)

TCGGCGCACTGCGAGCAGTAGCCCAAACAATCTCATATGAAGTCACCCTAGCCATCATTCTACTATCAA
CATTACTAATAAGTGGCTCCTTTAACCTCTC 4 (0.000104%)

TCGGCTCACTCCTTGGCGCCTGCCTGATCCTCCAAATCACCACAGGACTATTCCTAGCCATGCACTACTC
ACCAGACGCCTCAACCGCCTTTTCATCAAT 3 (0.000078%)

TCGGCTTCCTGCCTCGGAAGCGCAGCAGCAGGCATCGTGGGAAGGTGAAGAGCTTCCCTAAGGATGAC
CCGTCCAAGCCGGTCCACCTCACAGCCTTCCT 9 (0.000234%)

TCGGGCACGCTACGAGAGTACAAGGTAGTGGGTCGCTGCCTGCCCACCCCCAAATGCCACACGCCGCCC
CTCTACCGCATGCGAATCTTTGCGCCTAATC 3 (0.000078%)

TCGTACTGCTAGGAAGCTCCGTAGTCACCGACGAGACCAGAAGTGGCATGATAAACAGTATAAGAAAG
CTCATTTGGGCACAGCCCTAAAGGCCAACCCT 8 (0.000208%)

TCGTCGTGGCAACGTTGCTGGTGACAGCAAAAATGACCCACCAATGGAAGCAGCTGGCTTCACTGCTCA
GGTGATTATCCTGAACCATCCAGGCCAAATA 3 (0.000078%)

TCGTCTGATCCGTCCTAATCACAGCAGTCCTACTTCTCCTATCTCTCCCAGTCCTAGCTGCTGGCATCACT
ATACTACTAACAGACCGCAACCTCAACAC 3 (0.000078%)

TCGTGGCAACGTTGCTGGTGACAGCAAAAATGACCCACCAATGGAAGCAGCTGGCTTCACTGCTCAGGT
GATTATCCTGAACCATCCAGGCCAAATAAGC 4 (0.000104%)

TCGTGGTTGCATTGTGGATGCAAATCTGAGCGTTCTCAACTTGGTTATTGTAAAAAAAGGAGAGAAGGA
TATTCCTGGACTGACTGATACTACAGTGCCT 3 (0.000078%)

TCTACTCCCACTAATAGCTTTTTGATGACTTCTAGCAAGCCTCGCTAACCTCGCCTTACCCCCCACTATT
AACCTACTGGGAGAACTCTCTGTGCTAGTA 9 (0.000234%)

TCTCAAGGATGAGGTTTTGAAGATTATGCCAGTGCAGAAGCAGACCCGTGCCGGCCAGCGCACCAGGTT

CAAGGCATTTGTTGCTATCGGGGACTACAAT 4 (0.000104%)

TCTCAGCCCTCCTAATGACCTCCGGCCTAGCCATGTGATTTCACTTCCACTCCATAACGCTCCTCATACT
AGGCCTACTAACCAACACACTAACCATATA 12 (0.000312%)

TCTCCTTCATCCCTCTCCAGAAGAGGAGAAGAGGAAACACAAGAAGAAACGCCTGGTGCAGAGCCCCA
ATTCCTACTTCATGGATGTGAAATGCCCAGGA 3 (0.000078%)

TCTCGCAAAGGATCTCCTTCATCCCTCTCCAGAAGAGGAGAAGAGGAAACACAAGAAGAAACGCCTGG
TGCAGAGCCCCAATTCCTACTTCATGGATGTG 5 (0.000130%)

TCTGATCCGTCCTAATCACAGCAGTCCTACTTCTCCTATCTCTCCCAGTCCTAGCTGCTGGCATCACTAT
ACTACTAACAGACCGCAACCTCAACACCAC 6 (0.000156%)

TCTGCCATCAACGAAGTGGTAACCCGAGAATACACCATCAACATTCACAAGCGCATCCATGGAGTGGG
CTTCAAGAAGCGTGCACCTCGGGCACTCAAAG 3 (0.000078%)

TCTGCCCAGAAAGCTCAGAAGGCTAAATGAATATTATCCCTAATACCTGCCACCCCACTCTTAATCAGT
GGTGGAAGAACGGTCTCAGAACTGTTTGTTT 12 (0.000312%)

TCTGGTAAAAAGCTGGAAGATGGCCCTAAATTCTTGAAGTCTGGTGATGCTGCCATTGTTGATATGGTT
CCTGGCAAGCCCATGTGTGTTGAGAGCTTCT 9 (0.000234%)

TCTGTCGAAATGCACCATGAAGCTTTGAGTGAAGCTCTTCCTGGGGACAATGTGGGCTTCAATGTCAAG
AATGTGTCTGTCAAGGATGTTCGTCGTGGCA 45 (0.001168%)

TCTTCCTGGGGGCCTCTCTCAAGGATGAGGTTTTGAAGATTATGCCAGTGCAGAAGCAGACCCGTGCCG
GCCAGCGCACCAGGTTCAAGGCATTTGTTGC 3 (0.000078%)

TCTTGAAGTCTGGTGATGCTGCCATTGTTGATATGGTTCCTGGCAAGCCCATGTGTGTTGAGAGCTTCTC
AGACTATCCACCTTTGGGTCGCTTTGCTGT35 (0.000909%)

TGAAAACTACCCCTAAAAGCCAAAATGGGAAAGGAAAAGACTCATATCAACATTGTCGTCATTGGACA
CGTAGATTCGGGCAAGTCCACCACTACTGGCC 90 (0.002337%)

TGAAACTTCGGCTCACTCCTTGGCGCCTGCCTGATCCTCCAAATCACCACAGGACTATTCCTAGCCATGC
ACTACTCACCAGACGCCTCAACCGCCTTTT 6 (0.000156%)

TGAACGCAGGCACATACTTCCTATTCTACACCCTAGTAGGCTCCCTTCCCCTACTCATCGCACTGATTTA
CACTCACAACACCCTAGGCTCACTAAACAT 10 (0.000260%)

TGAAGATGAGGAAGCTGAGTCAGCTACGGGCAAGCGGGCAGCTGAAGATGATGAGGATGACGATGTCG
ATACCAAGAAGCAGAAGACCGACGAGGATGAC 25 (0.000649%)

TGAAGATTATGCCAGTGCAGAAGCAGACCCGTGCCGGCCAGCGCACCAGGTTCAAGGCATTTGTTGCTA
TCGGGGACTACAATGGCCACGTCGGTCTGGG 7 (0.000182%)

TGAAGGCCTCGGGCACGCTACGAGAGTACAAGGTAGTGGGTCGCTGCCTGCCCACCCCCAAATGCCAC
ACGCCGCCCCTCTACCGCATGCGAATCTTTGC 13 (0.000338%)

TGAAGTCTGGTGATGCTGCCATTGTTGATATGGTTCCTGGCAAGCCCATGTGTGTTGAGAGCTTCTCAGA
CTATCCACCTTTGGGTCGCTTTGCTGTTCG 14 (0.000364%)

TGAAGTTTAATCCCTTTGTGACTTCCGACCGAAGCAAGAATCGCAAAAGGCATTTCAATGCACCTTCCC
ACATTCGAAGGAAGATTATGTCTTCCCCTCT 13 (0.000338%)

TGAATCCTGTGGAGCATCCTTTTGGAGGTGGCAACCACCAGCACATCGGCAAGCCCTCCACCATCCGCA
GAGATGCCCCTGCTGGCCGCAAAGTGGGTCT 4 (0.000104%)

TGACCCACCAATGGAAGCAGCTGGCTTCACTGCTCAGGTGATTATCCTGAACCATCCAGGCCAAATAAG
CGCCGGCTATGCCCCTGTATTGGATTGCCAC 3 (0.000078%)

TGACCCCTAACAGGGGCCCTCTCAGCCCTCCTAATGACCTCCGGCCTAGCCATGTGATTTCACTTCCACT
CCATAACGCTCCTCATACTAGGCCTACTAA 11 (0.000286%)

TGACTTCTAGCAAGCCTCGCTAACCTCGCCTTACCCCCCACTATTAACCTACTGGGAGAACTCTCTGTGC
TAGTAACCACGTTCTCCTGATCAAATATCA 5 (0.000130%)

TGAGATGGGAAAGGGCTCCTTCAAGTATGCCTGGGTCTTGGATAAACTGAAAGCTGAGCGTGAACGTG
GTATCACCATTGATATCTCCTTGTGGAAATTT 5 (0.000130%)

TGAGCGTGCCTACCAAAAGCAGCCGACCATCTTTCAAAACAAGAAGAGGGTCCTGCTGGGAGAAACTG
GCAAGGAGAAGCTCCCGCGGTACTACAAGAAC 9 (0.000234%)

TGAGGAAGAGGATGGAGATGAAGATGAGGAAGCTGAGTCAGCTACGGGCAAGCGGGCAGCTGAAGAT
GATGAGGATGACGATGTCGATACCAAGAAGCAG 6 (0.000156%)

TGAGGAAGCTGAGTCAGCTACGGGCAAGCGGGCAGCTGAAGATGATGAGGATGACGATGTCGATACCA
AGAAGCAGAAGACCGACGAGGATGACTAGACA 9 (0.000234%)

TGAGGCAGAGGTCCAAGTAAACCGCTAGCTTGTTGCACCGTGGAGGCCACAGGAGCAGAAACATGGAA
TGCCAGACGCTGGGGATGCTGGTACAAGTTGT4 (0.000104%)

TGATAACCATACACAACACTAAAGGACGAACCTGATCTCTTATACTAGTATCCTTAATCATTTTTATTGC
CACAACTAACCTCCTCGGACTCCTGCCTCA 18 (0.000467%)

TGATCGCCGTTCTGGTAAAAAGCTGGAAGATGGCCCTAAATTCTTGAAGTCTGGTGATGCTGCCATTGT
TGATATGGTTCCTGGCAAGCCCATGTGTGTT 7 (0.000182%)

TGATGACTTCTAGCAAGCCTCGCTAACCTCGCCTTACCCCCCACTATTAACCTACTGGGAGAACTCTCTG
TGCTAGTAACCACGTTCTCCTGATCAAATA 6 (0.000156%)

TGATGGTGAGGAAGAGGATGGAGATGAAGATGAGGAAGCTGAGTCAGCTACGGGCAAGCGGGCAGCT
GAAGATGATGAGGATGACGATGTCGATACCAAG 10 (0.000260%)

TGATTATCCTGAACCATCCAGGCCAAATAAGCGCCGGCTATGCCCCTGTATTGGATTGCCACACGGCTC
ACATTGCATGCAAGTTTGCTGAGCTGAAGGA 5 (0.000130%)

TGCAACTTCCTTCGGTCGTCCCGAATCCGGGTTCATCCGACACCAGCCGCCTCCACCATGCCGCCGAAG
TTCGACCCCAACGAGATCAAAGTCGTATACC 12 (0.000312%)

TGCATTGTGGATGCAAATCTGAGCGTTCTCAACTTGGTTATTGTAAAAAAAGGAGAGAAGGATATTCCT
GGACTGACTGATACTACAGTGCCTCGCCGCC 3 (0.000078%)

TGCATTTGTAATAATCTTCTTCATAGTAATACCCATCATAATCGGAGGCTTTGGCAACTGACTAGTTCCC
CTAATAATCGGTGCCCCCGATATGGCGTTT 14 (0.000364%)

TGCCCAGAAAGCTCAGAAGGCTAAATGAATATTATCCCTAATACCTGCCACCCCACTCTTAATCAGTGG
TGGAAGAACGGTCTCAGAACTGTTTGTTTCA 10 (0.000260%)

TGCCCGTCACCAAGTTGGGCCGCTTGGTCAAGGACATGAAGATCAAGTCCCTGGAGGAGATCTATCTCT
TCTCCCTGCCCATTAAGGAATCAGAGATCAT 5 (0.000130%)

TGCCCTTCTGGCTTACACACTGGGTGTGAAACAACTAATTGTCGGTGTTAACAAAATGGATTCCACTGA
GCCACCCTACAGCCAGAAGAGATATGAGGAA 8 (0.000208%)

TGCCTACCAAAAGCAGCCGACCATCTTTCAAAACAAGAAGAGGGTCCTGCTGGGAGAAACTGGCAAGG
AGAAGCTCCCGCGGTACTACAAGAACATCGGT 4 (0.000104%)

TGCCTCTCGCAAAGGATCTCCTTCATCCCTCTCCAGAAGAGGAGAAGAGGAAACACAAGAAGAAACGC
CTGGTGCAGAGCCCCAATTCCTACTTCATGGA 6 (0.000156%)

TGCGCAGGCTGAAGCGCAAAAGAAGAAAGATGAGGCAGAGGTCCAAGTAAACCGCTAGCTTGTTGCAC
CGTGGAGGCCACAGGAGCAGAAACATGGAATG 9 (0.000234%)

TGCGTGTGGCTCCCGAGGAGCACCCCGTGCTGCTGACCGAGGCCCCCCTGAACCCCAAGGCCAACCGCG
AGAAGATGACCCAGATCATGTTTGAGACCTT 3 (0.000078%)

TGCTAGGAAGCTCCGTAGTCACCGACGAGACCAGAAGTGGCATGATAAACAGTATAAGAAAGCTCATT
TGGGCACAGCCCTAAAGGCCAACCCTTTTGGA 4 (0.000104%)

TGCTCAGGTGATTATCCTGAACCATCCAGGCCAAATAAGCGCCGGCTATGCCCCTGTATTGGATTGCCA
CACGGCTCACATTGCATGCAAGTTTGCTGAG 7 (0.000182%)

TGCTCATGTGGTGTTGAGGAAAGCAGACATTGACCTCACCAAGAGGGCGGGAGAACTCACTGAGGATG
AGGTGGAACGTGTGATCACCATTATGCAGAAT 13 (0.000338%)

TGCTGAGATGGGAAAGGGCTCCTTCAAGTATGCCTGGGTCTTGGATAAACTGAAAGCTGAGCGTGAAC
GTGGTATCACCATTGATATCTCCTTGTGGAAA 44 (0.001142%)

TGCTGGAGCTGGCAAGGTCACCAAGTCTGCCCAGAAAGCTCAGAAGGCTAAATGAATATTATCCCTAAT
ACCTGCCACCCCACTCTTAATCAGTGGTGGA 17 (0.000441%)

TGCTGGTGACAGCAAAAATGACCCACCAATGGAAGCAGCTGGCTTCACTGCTCAGGTGATTATCCTGAA
CCATCCAGGCCAAATAAGCGCCGGCTATGCC 28 (0.000727%)

TGGAAGATGGCCCTAAATTCTTGAAGTCTGGTGATGCTGCCATTGTTGATATGGTTCCTGGCAAGCCCAT
GTGTGTTGAGAGCTTCTCAGACTATCCACC 64 (0.001662%)

TGGAAGCAGCTGGCTTCACTGCTCAGGTGATTATCCTGAACCATCCAGGCCAAATAAGCGCCGGCTATG
CCCCTGTATTGGATTGCCACACGGCTCACAT 10 (0.000260%)

TGGAGATGAAGATGAGGAAGCTGAGTCAGCTACGGGCAAGCGGGCAGCTGAAGATGATGAGGATGAC
GATGTCGATACCAAGAAGCAGAAGACCGACGAG 37 (0.000961%)

TGGAGCTGGCAAGGTCACCAAGTCTGCCCAGAAAGCTCAGAAGGCTAAATGAATATTATCCCTAATACC
TGCCACCCCACTCTTAATCAGTGGTGGAAGA 8 (0.000208%)

TGGATACATAGGTATGGTCTGAGCTATGATATCAATTGGCTTCCTAGGGTTTATCGTGTGAGCACACCAT
ATATTTACAGTAGGAATAGACGTAGACACA 27 (0.000701%)

TGGATGCCCGTCACCAAGTTGGGCCGCTTGGTCAAGGACATGAAGATCAAGTCCCTGGAGGAGATCTAT
CTCTTCTCCCTGCCCATTAAGGAATCAGAGA 33 (0.000857%)

TGGCAACGTTGCTGGTGACAGCAAAAATGACCCACCAATGGAAGCAGCTGGCTTCACTGCTCAGGTGAT
TATCCTGAACCATCCAGGCCAAATAAGCGCC 25 (0.000649%)

TGGCAAGGTCACCAAGTCTGCCCAGAAAGCTCAGAAGGCTAAATGAATATTATCCCTAATACCTGCCAC
CCCACTCTTAATCAGTGGTGGAAGAACGGTC 20 (0.000519%)

TGGCCCTAAATTCTTGAAGTCTGGTGATGCTGCCATTGTTGATATGGTTCCTGGCAAGCCCATGTGTGTT
GAGAGCTTCTCAGACTATCCACCTTTGGGT 27 (0.000701%)

TGGCGAGAAGAAAAAGGGCCGTTCTGCCATCAACGAAGTGGTAACCCGAGAATACACCATCAACATTC

ACAAGCGCATCCATGGAGTGGGCTTCAAGAAG 3 (0.000078%)

TGGCTTCACTGCTCAGGTGATTATCCTGAACCATCCAGGCCAAATAAGCGCCGGCTATGCCCCTGTATT
GGATTGCCACACGGCTCACATTGCATGCAAG 4 (0.000104%)

TGGGAAAGGAAAAGACTCATATCAACATTGTCGTCATTGGACACGTAGATTCGGGCAAGTCCACCACTA
CTGGCCATCTGATCTATAAATGCGGTGGCAT 24 (0.000623%)

TGGGAAAGGGCTCCTTCAAGTATGCCTGGGTCTTGGATAAACTGAAAGCTGAGCGTGAACGTGGTATCA
CCATTGATATCTCCTTGTGGAAATTTGAGAC 52 (0.001350%)

TGGGCCGCTTGGTCAAGGACATGAAGATCAAGTCCCTGGAGGAGATCTATCTCTTCTCCCTGCCCATTA
AGGAATCAGAGATCATTGATTTCTTCCTGGG 11 (0.000286%)

TGGGTCCCTCGGCTTCCTGCCTCGGAAGCGCAGCAGCAGGCATCGTGGGAAGGTGAAGAGCTTCCCTAA
GGATGACCCGTCCAAGCCGGTCCACCTCACA 4 (0.000104%)

TGGTAAAAAGCTGGAAGATGGCCCTAAATTCTTGAAGTCTGGTGATGCTGCCATTGTTGATATGGTTCC
TGGCAAGCCCATGTGTGTTGAGAGCTTCTCA 4 (0.000104%)

TGGTGAGGAAGAGGATGGAGATGAAGATGAGGAAGCTGAGTCAGCTACGGGCAAGCGGGCAGCTGAA
GATGATGAGGATGACGATGTCGATACCAAGAAG 11 (0.000286%)

TGGTTGCATTGTGGATGCAAATCTGAGCGTTCTCAACTTGGTTATTGTAAAAAAAGGAGAGAAGGATAT
TCCTGGACTGACTGATACTACAGTGCCTCGC 3 (0.000078%)

TGTCAAGAATGTGTCTGTCAAGGATGTTCGTCGTGGCAACGTTGCTGGTGACAGCAAAAATGACCCACC
AATGGAAGCAGCTGGCTTCACTGCTCAGGTG 11 (0.000286%)

TGTCAAGGATGTTCGTCGTGGCAACGTTGCTGGTGACAGCAAAAATGACCCACCAATGGAAGCAGCTG
GCTTCACTGCTCAGGTGATTATCCTGAACCAT 15 (0.000389%)

TGTCGAAATGCACCATGAAGCTTTGAGTGAAGCTCTTCCTGGGGACAATGTGGGCTTCAATGTCAAGAA
TGTGTCTGTCAAGGATGTTCGTCGTGGCAAC 34 (0.000883%)

TGTCGTGAAAACTACCCCTAAAAGCCAAAATGGGAAAGGAAAAGACTCATATCAACATTGTCGTCATT
GGACACGTAGATTCGGGCAAGTCCACCACTAC 12 (0.000312%)

TGTCTGTCAAGGATGTTCGTCGTGGCAACGTTGCTGGTGACAGCAAAAATGACCCACCAATGGAAGCAG
CTGGCTTCACTGCTCAGGTGATTATCCTGAA 27 (0.000701%)

TGTGACTTCCGACCGAAGCAAGAATCGCAAAAGGCATTTCAATGCACCTTCCCACATTCGAAGGAAGAT
TATGTCTTCCCCTCTTTCCAAAGAGCTGAGA 5 (0.000130%)

TGTGGCTCCCGAGGAGCACCCCGTGCTGCTGACCGAGGCCCCCCTGAACCCCAAGGCCAACCGCGAGA
AGATGACCCAGATCATGTTTGAGACCTTCAAC 3 (0.000078%)

TGTGTCTGTCAAGGATGTTCGTCGTGGCAACGTTGCTGGTGACAGCAAAAATGACCCACCAATGGAAGC
AGCTGGCTTCACTGCTCAGGTGATTATCCTG 18 (0.000467%)

TGTTCGTCGTGGCAACGTTGCTGGTGACAGCAAAAATGACCCACCAATGGAAGCAGCTGGCTTCACTGC
TCAGGTGATTATCCTGAACCATCCAGGCCAA 5 (0.000130%)

TTAATCCCTTTGTGACTTCCGACCGAAGCAAGAATCGCAAAAGGCATTTCAATGCACCTTCCCACATTC
GAAGGAAGATTATGTCTTCCCCTCTTTCCAA 5 (0.000130%)

TTACAACGGAAGTAAAATCTGTCGAAATGCACCATGAAGCTTTGAGTGAAGCTCTTCCTGGGGACAATG
TGGGCTTCAATGTCAAGAATGTGTCTGTCAA 19 (0.000493%)

TTATAACAAACCCTGAGAACCAAAATGAACGAAAATCTGTTCGCTTCATTCATTGCCCCCACAATCCTA
GGCCTACCCGCCGCAGTACTGATCATTCTAT 36 (0.000935%)

TTCAAAACAAGAAGAGGGTCCTGCTGGGAGAAACTGGCAAGGAGAAGCTCCCGCGGTACTACAAGAAC
ATCGGTCTGGGCTTCAAGACACCCAAGGAGGC 16 (0.000415%)

TTCAAACTCTACTCCCACTAATAGCTTTTTGATGACTTCTAGCAAGCCTCGCTAACCTCGCCTTACCCCC
CACTATTAACCTACTGGGAGAACTCTCTGT 23 (0.000597%)

TTCAATGTCAAGAATGTGTCTGTCAAGGATGTTCGTCGTGGCAACGTTGCTGGTGACAGCAAAAATGAC
CCACCAATGGAAGCAGCTGGCTTCACTGCTC 31 (0.000805%)

TTCACTGCTCAGGTGATTATCCTGAACCATCCAGGCCAAATAAGCGCCGGCTATGCCCCTGTATTGGATT
GCCACACGGCTCACATTGCATGCAAGTTTG 9 (0.000234%)

TTCAGACTGAGCGTGCCTACCAAAAGCAGCCGACCATCTTTCAAAACAAGAAGAGGGTCCTGCTGGGA
GAAACTGGCAAGGAGAAGCTCCCGCGGTACTA 57 (0.001480%)

TTCAGAGGAAACAAATGGTCATTGATGTCCTTCACCCCGGGAAGGCGACAGTGCCTAAGACAGAAATT
CGGGAAAAACTAGCCAAAATGTACAAGACCAC 4 (0.000104%)

TTCATCCCTCTCCAGAAGAGGAGAAGAGGAAACACAAGAAGAAACGCCTGGTGCAGAGCCCCAATTCC
TACTTCATGGATGTGAAATGCCCAGGATGCTA 9 (0.000234%)

TTCCACCCTTACTACACAATCAAAGACGCCCTCGGCTTACTTCTCTTCATTCTCTCCTTAATGACATTAAC
ACTATTCTCACCAGACCTCCTAGGCGACC 10 (0.000260%)

TTCCGACCGAAGCAAGAATCGCAAAAGGCATTTCAATGCACCTTCCCACATTCGAAGGAAGATTATGTC
TTCCCCTCTTTCCAAAGAGCTGAGACAGAAG 17 (0.000441%)

TTCCTTCGGTCGTCCCGAATCCGGGTTCATCCGACACCAGCCGCCTCCACCATGCCGCCGAAGTTCGACC
CCAACGAGATCAAAGTCGTATACCTGAGGT 7 (0.000182%)

TTCGATACGGGATAATCCTATTTATTACCTCAGAAGTTTTTTTCTTCGCAGGATTTTTCTGAGCCTTTTAC
CACTCCAGCCTAGCCCCTACCCCCCAATT 7 (0.000182%)

TTCGGCTCACTCCTTGGCGCCTGCCTGATCCTCCAAATCACCACAGGACTATTCCTAGCCATGCACTACT
CACCAGACGCCTCAACCGCCTTTTCATCAA 3 (0.000078%)

TTCGTACTGCTAGGAAGCTCCGTAGTCACCGACGAGACCAGAAGTGGCATGATAAACAGTATAAGAAA
GCTCATTTGGGCACAGCCCTAAAGGCCAACCC 5 (0.000130%)

TTCGTCGTGGCAACGTTGCTGGTGACAGCAAAAATGACCCACCAATGGAAGCAGCTGGCTTCACTGCTC
AGGTGATTATCCTGAACCATCCAGGCCAAAT 7 (0.000182%)

TTCGTCTGATCCGTCCTAATCACAGCAGTCCTACTTCTCCTATCTCTCCCAGTCCTAGCTGCTGGCATCAC
TATACTACTAACAGACCGCAACCTCAACA 6 (0.000156%)

TTCGTGGTTGCATTGTGGATGCAAATCTGAGCGTTCTCAACTTGGTTATTGTAAAAAAAGGAGAGAAGG
ATATTCCTGGACTGACTGATACTACAGTGCC 5 (0.000130%)

TTCTGGTAAAAAGCTGGAAGATGGCCCTAAATTCTTGAAGTCTGGTGATGCTGCCATTGTTGATATGGTT
CCTGGCAAGCCCATGTGTGTTGAGAGCTTC 24 (0.000623%)

TTCTTCCTGGGGGCCTCTCTCAAGGATGAGGTTTTGAAGATTATGCCAGTGCAGAAGCAGACCCGTGCC
GGCCAGCGCACCAGGTTCAAGGCATTTGTTG 247 (0.006413%)

TTCTTGAAGTCTGGTGATGCTGCCATTGTTGATATGGTTCCTGGCAAGCCCATGTGTGTTGAGAGCTTCT CAGACTATCCACCTTTGGGTCGCTTTGCTG 14 (0.000364%)

TTGAAGATTATGCCAGTGCAGAAGCAGACCCGTGCCGGCCAGCGCACCAGGTTCAAGGCATTTGTTGCT ATCGGGGACTACAATGGCCACGTCGGTCTGG 6 (0.000156%)

TTGAAGTCTGGTGATGCTGCCATTGTTGATATGGTTCCTGGCAAGCCCATGTGTGTTGAGAGCTTCTCAG ACTATCCACCTTTGGGTCGCTTTGCTGTTC 55 (0.001428%)

TTGATCGCCGTTCTGGTAAAAAGCTGGAAGATGGCCCTAAATTCTTGAAGTCTGGTGATGCTGCCATTG TTGATATGGTTCCTGGCAAGCCCATGTGTGT 25 (0.000649%)

TTGCTCCAGTCAACGTTACAACGGAAGTAAAATCTGTCGAAATGCACCATGAAGCTTTGAGTGAAGCTC TTCCTGGGGACAATGTGGGCTTCAATGTCAA 3 (0.000078%)

TTGCTGGTGACAGCAAAAATGACCCACCAATGGAAGCAGCTGGCTTCACTGCTCAGGTGATTATCCTGA ACCATCCAGGCCAAATAAGCGCCGGCTATGC 3 (0.000078%)

TTGGATACATAGGTATGGTCTGAGCTATGATATCAATTGGCTTCCTAGGGTTTATCGTGTGAGCACACCA TATATTTACAGTAGGAATAGACGTAGACAC 16 (0.000415%)

TTGGGCCGCTTGGTCAAGGACATGAAGATCAAGTCCCTGGAGGAGATCTATCTCTTCTCCCTGCCCATT AAGGAATCAGAGATCATTGATTTCTTCCTGG 9 (0.000234%)

TTGTAATAATCTTCTTCATAGTAATACCCATCATAATCGGAGGCTTTGGCAACTGACTAGTTCCCCTAAT AATCGGTGCCCCCGATATGGCGTTTCCCCG 7 (0.000182%)

TTGTGACTTCCGACCGAAGCAAGAATCGCAAAAGGCATTTCAATGCACCTTCCCACATTCGAAGGAAGA TTATGTCTTCCCCTCTTTCCAAAGAGCTGAG 3 (0.000078%)

TTTAATCCCTTTGTGACTTCCGACCGAAGCAAGAATCGCAAAAGGCATTTCAATGCACCTTCCCACATTC GAAGGAAGATTATGTCTTCCCCTCTTTCCA 8 (0.000208%)

TTTCAAAACAAGAAGAGGGTCCTGCTGGGAGAAACTGGCAAGGAGAAGCTCCCGCGGTACTACAAGAA CATCGGTCTGGGCTTCAAGACACCCAAGGAGG 28 (0.000727%)

TTTGAAGATTATGCCAGTGCAGAAGCAGACCCGTGCCGGCCAGCGCACCAGGTTCAAGGCATTTGTTGC TATCGGGGACTACAATGGCCACGTCGGTCTG 26 (0.000675%)

TTTGATGACTTCTAGCAAGCCTCGCTAACCTCGCCTTACCCCCCACTATTAACCTACTGGGAGAACTCTC TGTGCTAGTAACCACGTTCTCCTGATCAAA 3 (0.000078%)

TTTGCTCCAGTCAACGTTACAACGGAAGTAAAATCTGTCGAAATGCACCATGAAGCTTTGAGTGAAGCT CTTCCTGGGGACAATGTGGGCTTCAATGTCA 9 (0.000234%)

TTTGGATACATAGGTATGGTCTGAGCTATGATATCAATTGGCTTCCTAGGGTTTATCGTGTGAGCACACC ATATATTTACAGTAGGAATAGACGTAGACA 60 (0.001558%)

TTTGTGACTTCCGACCGAAGCAAGAATCGCAAAAGGCATTTCAATGCACCTTCCCACATTCGAAGGAAG ATTATGTCTTCCCCTCTTTCCAAAGAGCTGA 11 (0.000286%)

TTTTGAAGATTATGCCAGTGCAGAAGCAGACCCGTGCCGGCCAGCGCACCAGGTTCAAGGCATTTGTTG CTATCGGGGACTACAATGGCCACGTCGGTCT 4 (0.000104%)

TTTTGATGACTTCTAGCAAGCCTCGCTAACCTCGCCTTACCCCCCACTATTAACCTACTGGGAGAACTCT CTGTGCTAGTAACCACGTTCTCCTGATCAA 15 (0.000389%)

TTTTTGATGACTTCTAGCAAGCCTCGCTAACCTCGCCTTACCCCCCACTATTAACCTACTGGGAGAACTC

TCTGTGCTAGTAACCACGTTCTCCTGATCA 19 (0.000493%)

TTTTTTTTTTTTTTTTTTTTTTTTTTTTTTTTTTTTTTTTTTTTTTTTTTTTTTTTTTTTTTTTTTTTTTTTTTTTT
TTTTTTTTTTTTTTTTTTTTTTT 1707 (0.044322%)

fastp -i NG-A4833_RCH_ACV_siCTRa_2_libLAO1925_1.fastq.gz -I NG-A4833_RCH_ACV_siCTRa_2_libLAO1925_2.fastq.gz -o ../../trim/RCH-ACV_siCTRa_2.R1.fq.gz -O ../../trim/RCH-ACV_siCTRa_2.R2.fq.gz -q 20 -c -p --thread 4

fastp 0.23.4, at 2025-05-06 08:53:55

fastp report <https://github.com/OpenGene/fastp>
Summary
General
fastp version: 0.23.4 (https://github.com/OpenGene/fastp <https://
github.com/OpenGene/fastp>)
sequencing: paired end (151 cycles + 151 cycles)
mean length before filtering: 148bp, 148bp
mean length after filtering: 148bp, 148bp
duplication rate: 25.143310%
Insert size peak: 263

Before filtering
total reads: 89.427844 M
total bases: 13.309278 G
Q20 bases: 13.138391 G (98.716032%)
Q30 bases: 12.772440 G (95.966440%)
GC content: 47.056451%

After filtering
total reads: 88.368274 M
total bases: 13.137915 G
Q20 bases: 13.024215 G (99.134561%)
Q30 bases: 12.698525 G (96.655558%)
GC content: 47.007691%

Filtering result
reads passed filters: 88.368274 M (98.815168%)
reads corrected: 3.475553 M (3.886433%)
bases corrected: 6.272052 M (0.047125%)
reads with low quality: 964.186000 K (1.078172%)
reads with too many N: 95.384000 K (0.106660%)
reads too short: 0 (0.000000%)

Adapters
Adapter or bad ligation of read1
The input has little adapter percentage (~0.054304%), probably it's
trimmed before.
Sequence Occurrences
all adapter sequences 72161

Adapter or bad ligation of read2
The input has little adapter percentage (~0.140038%), probably it's
trimmed before.
Sequence Occurrences
AGATCGGAAGAGCGTCGTGGGGGGG 5946
AGATCGGAAGAGCGTCGTGGGGGGA 4574
other adapter sequences 225794

Insert size estimation
05010015020025000.10.20.3

Insert size distribution (53.815341% reads are with unknown length)Insert sizeRead percent (%)

<https://plot.ly/>
This estimation is based on paired-end overlap analysis, and there are
53.815341% reads found not overlapped.
The nonoverlapped read pairs may have insert size <30 or >272, or

contain too much sequencing errors to be detected as overlapped.
Before filtering
Before filtering: read1: quality
Value of each position will be shown on mouse over.
2040608010012014037.53838.53939.5

positionqualityATCGmean

<https://plot.ly/>
Before filtering: read1: base contents
Value of each position will be shown on mouse over.
2040608010012014000.10.20.30.40.50.60.7

positionbase content ratiosA(25.84%)T(27.49%)C(23.39%)G(23.17%)N(0.085%)GC(46.56%)

<https://plot.ly/>
Before filtering: read1: KMER counting
Darker background means larger counts. The count will be shown on mouse over.

AA AT AC AG TA TT TC TG CA CT CC CG GA GT GC GG
AAA AAAAA AAAAT AAAAC AAAAG AAATA AAATT AAATC AAATG AAACA AAACT
AAACC
AAACG AAAGA AAAGT AAAGC AAAGG
AAT AATAA AATAT AATAC AATAG AATTA AATTT AATTC AATTG AATCA AATCT
AATCC
AATCG AATGA AATGT AATGC AATGG
AAC AACAA AACAT AACAC AACAG AACTA AACTT AACTC AACTG AACCA AACCT
AACCC
AACCG AACGA AACGT AACGC AACGG
AAG AAGAA AAGAT AAGAC AAGAG AAGTA AAGTT AAGTC AAGTG AAGCA AAGCT
AAGCC
AAGCG AAGGA AAGGT AAGGC AAGGG
ATA ATAAA ATAAT ATAAC ATAAG ATATA ATATT ATATC ATATG ATACA ATACT
ATACC
ATACG ATAGA ATAGT ATAGC ATAGG
ATT ATTAA ATTAT ATTAC ATTAG ATTTA ATTTT ATTTC ATTTG ATTCA ATTCT ATTCC
ATTCG ATTGA ATTGT ATTGC ATTGG
ATC ATCAA ATCAT ATCAC ATCAG ATCTA ATCTT ATCTC ATCTG ATCCA ATCCT ATCCC
ATCCG ATCGA ATCGT ATCGC ATCGG
ATG ATGAA ATGAT ATGAC ATGAG ATGTA ATGTT ATGTC ATGTG ATGCA ATGCT
ATGCC
ATGCG ATGGA ATGGT ATGGC ATGGG
ACA ACAAA ACAAT ACAAC ACAAG ACATA ACATT ACATC ACATG ACACA ACACT
ACACC
ACACG ACAGA ACAGT ACAGC ACAGG
ACT ACTAA ACTAT ACTAC ACTAG ACTTA ACTTT ACTTC ACTTG ACTCA ACTCT ACTCC
ACTCG ACTGA ACTGT ACTGC ACTGG
ACC ACCAA ACCAT ACCAC ACCAG ACCTA ACCTT ACCTC ACCTG ACCCA ACCCT
ACCCC
ACCCG ACCGA ACCGT ACCGC ACCGG
ACG ACGAA ACGAT ACGAC ACGAG ACGTA ACGTT ACGTC ACGTG ACGCA ACGCT
ACGCC
ACGCG ACGGA ACGGT ACGGC ACGGG
AGA AGAAA AGAAT AGAAC AGAAG AGATA AGATT AGATC AGATG AGACA AGACT
AGACC
AGACG AGAGA AGAGT AGAGC AGAGG
AGT AGTAA AGTAT AGTAC AGTAG AGTTA AGTTT AGTTC AGTTG AGTCA AGTCT
AGTCC
AGTCG AGTGA AGTGT AGTGC AGTGG

AGC AGCAA AGCAT AGCAC AGCAG AGCTA AGCTT AGCTC AGCTG AGCCA AGCCT
AGCCC
AGCCG AGCGA AGCGT AGCGC AGCGG
AGG AGGAA AGGAT AGGAC AGGAG AGGTA AGGTT AGGTC AGGTG AGGCA AGGCT
AGGCC
AGGCG AGGGA AGGGT AGGGC AGGGG
TAA TAAAA TAAAT TAAAC TAAAG TAATA TAATT TAATC TAATG TAACA TAACT
TAACC
TAACG TAAGA TAAGT TAAGC TAAGG
TAT TATAA TATAT TATAC TATAG TATTA TATTT TATTC TATTG TATCA TATCT TATCC
TATCG TATGA TATGT TATGC TATGG
TAC TACAA TACAT TACAC TACAG TACTA TACTT TACTC TACTG TACCA TACCT TACCC
TACCG TACGA TACGT TACGC TACGG
TAG TAGAA TAGAT TAGAC TAGAG TAGTA TAGTT TAGTC TAGTG TAGCA TAGCT
TAGCC
TAGCG TAGGA TAGGT TAGGC TAGGG
TTA TTAAA TTAAT TTAAC TTAAG TTATA TTATT TTATC TTATG TTACA TTACT TTACC
TTACG TTAGA TTAGT TTAGC TTAGG
TTT TTTAA TTTAT TTTAC TTTAG TTTTA TTTTT TTTTC TTTTG TTTCA TTTCT TTTCC
TTTCG TTTGA TTTGT TTTGC TTTGG
TTC TTCAA TTCAT TTCAC TTCAG TTCTA TTCTT TTCTC TTCTG TTCCA TTCCT TTCCC
TTCCG TTCGA TTCGT TTCGC TTCGG
TTG TTGAA TTGAT TTGAC TTGAG TTGTA TTGTT TTGTC TTGTG TTGCA TTGCT TTGCC
TTGCG TTGGA TTGGT TTGGC TTGGG
TCA TCAAA TCAAT TCAAC TCAAG TCATA TCATT TCATC TCATG TCACA TCACT TCACC
TCACG TCAGA TCAGT TCAGC TCAGG
TCT TCTAA TCTAT TCTAC TCTAG TCTTA TCTTT TCTTC TCTTG TCTCA TCTCT TCTCC
TCTCG TCTGA TCTGT TCTGC TCTGG
TCC TCCAA TCCAT TCCAC TCCAG TCCTA TCCTT TCCTC TCCTG TCCCA TCCCT TCCCC
TCCCG TCCGA TCCGT TCCGC TCCGG
TCG TCGAA TCGAT TCGAC TCGAG TCGTA TCGTT TCGTC TCGTG TCGCA TCGCT TCGCC
TCGCG TCGGA TCGGT TCGGC TCGGG
TGA TGAAA TGAAT TGAAC TGAAG TGATA TGATT TGATC TGATG TGACA TGACT
TGACC
TGACG TGAGA TGAGT TGAGC TGAGG
TGT TGTAA TGTAT TGTAC TGTAG TGTTA TGTTT TGTTC TGTTG TGTCA TGTCT TGTCC
TGTCG TGTGA TGTGT TGTGC TGTGG
TGC TGCAA TGCAT TGCAC TGCAG TGCTA TGCTT TGCTC TGCTG TGCCA TGCCT TGCCC
TGCCG TGCGA TGCGT TGCGC TGCGG
TGG TGGAA TGGAT TGGAC TGGAG TGGTA TGGTT TGGTC TGGTG TGGCA TGGCT
TGGCC
TGGCG TGGGA TGGGT TGGGC TGGGG
CAA CAAAA CAAAT CAAAC CAAAG CAATA CAATT CAATC CAATG CAACA CAACT
CAACC
CAACG CAAGA CAAGT CAAGC CAAGG
CAT CATAA CATAT CATAC CATAG CATTA CATTT CATTC CATTG CATCA CATCT CATCC
CATCG CATGA CATGT CATGC CATGG
CAC CACAA CACAT CACAC CACAG CACTA CACTT CACTC CACTG CACCA CACCT
CACCC
CACCG CACGA CACGT CACGC CACGG
CAG CAGAA CAGAT CAGAC CAGAG CAGTA CAGTT CAGTC CAGTG CAGCA CAGCT
CAGCC
CAGCG CAGGA CAGGT CAGGC CAGGG
CTA CTAAA CTAAT CTAAC CTAAG CTATA CTATT CTATC CTATG CTACA CTACT CTACC
CTACG CTAGA CTAGT CTAGC CTAGG
CTT CTTAA CTTAT CTTAC CTTAG CTTTA CTTTT CTTTC CTTTG CTTCA CTTCT CTTCC
CTTCG CTTGA CTTGT CTTGC CTTGG
CTC CTCAA CTCAT CTCAC CTCAG CTCTA CTCTT CTCTC CTCTG CTCCA CTCCT CTCCC
CTCCG CTCGA CTCGT CTCGC CTCGG

CTG CTGAA CTGAT CTGAC CTGAG CTGTA CTGTT CTGTC CTGTG CTGCA CTGCT CTGCC
CTGCG CTGGA CTGGT CTGGC CTGGG
CCA CCAAA CCAAT CCAAC CCAAG CCATA CCATT CCATC CCATG CCACA CCACT
CCACC
CCACG CCAGA CCAGT CCAGC CCAGG
CCT CCTAA CCTAT CCTAC CCTAG CCTTA CCTTT CCTTC CCTTG CCTCA CCTCT CCTCC
CCTCG CCTGA CCTGT CCTGC CCTGG
CCC CCCAA CCCAT CCCAC CCCAG CCCTA CCCTT CCCTC CCCTG CCCCA CCCCT CCCCC
CCCCG CCCGA CCCGT CCCGC CCCGG
CCG CCGAA CCGAT CCGAC CCGAG CCGTA CCGTT CCGTC CCGTG CCGCA CCGCT
CCGCC
CCGCG CCGGA CCGGT CCGGC CCGGG
CGA CGAAA CGAAT CGAAC CGAAG CGATA CGATT CGATC CGATG CGACA CGACT
CGACC
CGACG CGAGA CGAGT CGAGC CGAGG
CGT CGTAA CGTAT CGTAC CGTAG CGTTA CGTTT CGTTC CGTTG CGTCA CGTCT CGTCC
CGTCG CGTGA CGTGT CGTGC CGTGG
CGC CGCAA CGCAT CGCAC CGCAG CGCTA CGCTT CGCTC CGCTG CGCCA CGCCT
CGCCC
CGCCG CGCGA CGCGT CGCGC CGCGG
CGG CGGAA CGGAT CGGAC CGGAG CGGTA CGGTT CGGTC CGGTG CGGCA CGGCT
CGGCC
CGGCG CGGGA CGGGT CGGGC CGGGG
GAA GAAAA GAAAT GAAAC GAAAG GAATA GAATT GAATC GAATG GAACA GAACT
GAACC
GAACG GAAGA GAAGT GAAGC GAAGG
GAT GATAA GATAT GATAC GATAG GATTA GATTT GATTC GATTG GATCA GATCT
GATCC
GATCG GATGA GATGT GATGC GATGG
GAC GACAA GACAT GACAC GACAG GACTA GACTT GACTC GACTG GACCA GACCT
GACCC
GACCG GACGA GACGT GACGC GACGG
GAG GAGAA GAGAT GAGAC GAGAG GAGTA GAGTT GAGTC GAGTG GAGCA GAGCT
GAGCC
GAGCG GAGGA GAGGT GAGGC GAGGG
GTA GTAAA GTAAT GTAAC GTAAG GTATA GTATT GTATC GTATG GTACA GTACT
GTACC
GTACG GTAGA GTAGT GTAGC GTAGG
GTT GTTAA GTTAT GTTAC GTTAG GTTTA GTTTT GTTTC GTTTG GTTCA GTTCT GTTCC
GTTCG GTTGA GTTGT GTTGC GTTGG
GTC GTCAA GTCAT GTCAC GTCAG GTCTA GTCTT GTCTC GTCTG GTCCA GTCCT GTCCC
GTCCG GTCGA GTCGT GTCGC GTCGG
GTG GTGAA GTGAT GTGAC GTGAG GTGTA GTGTT GTGTC GTGTG GTGCA GTGCT
GTGCC
GTGCG GTGGA GTGGT GTGGC GTGGG
GCA GCAAA GCAAT GCAAC GCAAG GCATA GCATT GCATC GCATG GCACA GCACT
GCACC
GCACG GCAGA GCAGT GCAGC GCAGG
GCT GCTAA GCTAT GCTAC GCTAG GCTTA GCTTT GCTTC GCTTG GCTCA GCTCT GCTCC
GCTCG GCTGA GCTGT GCTGC GCTGG
GCC GCCAA GCCAT GCCAC GCCAG GCCTA GCCTT GCCTC GCCTG GCCCA GCCCT
GCCCC
GCCCG GCCGA GCCGT GCCGC GCCGG
GCG GCGAA GCGAT GCGAC GCGAG GCGTA GCGTT GCGTC GCGTG GCGCA GCGCT
GCGCC
GCGCG GCGGA GCGGT GCGGC GCGGG
GGA GGAAA GGAAT GGAAC GGAAG GGATA GGATT GGATC GGATG GGACA GGACT
GGACC
GGACG GGAGA GGAGT GGAGC GGAGG

GGT GGTAA GGTAT GGTAC GGTAG GGTTA GGTTT GGTTC GGTTG GGTCA GGTCT GGTCC
GGTCG GGTGA GGTGT GGTGC GGTGG
GGC GGCAA GGCAT GGCAC GGCAG GGCTA GGCTT GGCTC GGCTG GGCCA GGCCT GGCCC
GGCCG GGCGA GGCGT GGCGC GGCGG
GGG GGGAA GGGAT GGGAC GGGAG GGGTA GGGTT GGGTC GGGTG GGGCA GGGCT GGGCC
GGGCG GGGGA GGGGT GGGGC GGGGG

Before filtering: read1: overrepresented sequences
Sampling rate: 1 / 20
overrepresented sequence count (% of bases) distribution: cycle 1 ~ cycle 151

AAAAAAAAAAAAAAAAAAAAAAAAAAAAAAAAAAAAAAAAAAAAAAAAAAAAAAAAAAAAAAAAAA
AAAAAAAAAAAAAAAAAAAAAAAAAAAAAAAAAA 1573 (0.047287%)

AAAAAAAAAAAAAAAAAAAAAAAAAAAAAAAAAAAAAAAAAAAAAAAAAAAAAAAAAAAAAAAAAA
AAAAAAAAAAAAAAAAAAAAAAAAAAAAAAAAAAAAAAAAAAAAAAAAAAAAAAAAAAAAAAAAAA
AAAAAAAAAAAAAAAAA 1075 (0.048151%)

AAAAAAGAAAACCAAAGTGGTCCACAAAACATTCTCCTTTCCTTCTGAAGGTTTTACGATGCATTGTTAT
CATTAACCAGTCTTTTACTACTAAACTTAA 36 (0.001082%)

AAAAACTAATAACTTAAAACTGCCACACGCAAAAAAGAAAACCAAAGTGGTCCACAAAACATTCTCCTT
TCCTTCTGAAGGTTTTACGATGCATTGTTAT 11 (0.000331%)

AAAAAGAAAACCAAAGTGGTCCACAAAACATTCTCCTTTCCTTCTGAAGGTTTTACGATGCATTGTTATC
ATTAACCAGTCTTTTACTACTAAACTTAAA 15 (0.000451%)

AAAAAGTACTGATTTTAAAAACTAATAACTTAAAACTGCCACACGCAAAAAAGAAAACCAAAGTGGTCC
ACAAAACATTCTCCTTTCCTTCTGAAGGTTT 23 (0.000691%)

AAAACCAAAGTGGTCCACAAAACATTCTCCTTTCCTTCTGAAGGTTTTACGATGCATTGTTATCATTAAC
CAGTCTTTTACTACTAAACTTAAATGGCCA 4 (0.000120%)

AAAACTAATAACTTAAAACTGCCACACGCAAAAAAGAAAACCAAAGTGGTCCACAAAACATTCTCCTTT
CCTTCTGAAGGTTTTACGATGCATTGTTATC 11 (0.000331%)

AAAAGAAAACCAAAGTGGTCCACAAAACATTCTCCTTTCCTTCTGAAGGTTTTACGATGCATTGTTATCA
TTAACCAGTCTTTTACTACTAAACTTAAAT 3 (0.000090%)

AAAAGAAAGATGAATCCTAGGGCTCAGAGCACTGCAGCAGATCATTTCATATTGCTTCCGTGGAGTGTG
GCGAGTCAGCTAAATACTTTGACGCCGGTGG 3 (0.000090%)

AAAAGGTTGGGGAACAGCTAAATAGGTTGTTGTTGATTTGGTTAAAAAATAGTAGAGGGATGATGCTAA
TAATTAGGCTGTGGGTGGTTGTGTTGATTCA 5 (0.000150%)

AAAAGTACTGATTTTAAAAACTAATAACTTAAAACTGCCACACGCAAAAAAGAAAACCAAAGTGGTCCA
CAAAACATTCTCCTTTCCTTCTGAAGGTTTT 8 (0.000240%)

AAAATAGAGACCCAGTAAAATTGTAATAAGCAGTGCTTGAATTATTTGGTTTCGGTTGTTTTCTATTAGA
CTATGGTGAGCTCAGGTGATTGATACTCCT 67 (0.002014%)

AAAATTGTAATAAGCAGTGCTTGAATTATTTGGTTTCGGTTGTTTTCTATTAGACTATGGTGAGCTCAGGT
GATTGATACTCCTGATGCGAGTAATACGG 16 (0.000481%)

AAACCAAAGTGGTCCACAAAACATTCTCCTTTCCTTCTGAAGGTTTTACGATGCATTGTTATCATTAACC

AGTCTTTTACTACTAAACTTAAATGGCCAA 7 (0.000210%)

AAACTAATAACTTAAAACTGCCACACGCAAAAAAGAAAACCAAAGTGGTCCACAAAACATTCTCCTTTC
CTTCTGAAGGTTTTACGATGCATTGTTATCA 6 (0.000180%)

AAAGAAAACCAAAGTGGTCCACAAAACATTCTCCTTTCCTTCTGAAGGTTTTACGATGCATTGTTATCAT
TAACCAGTCTTTTACTACTAAACTTAAATG 6 (0.000180%)

AAAGAAAGATGAATCCTAGGGCTCAGAGCACTGCAGCAGATCATTTCATATTGCTTCCGTGGAGTGTGG
CGAGTCAGCTAAATACTTTGACGCCGGTGGG 3 (0.000090%)

AAAGAGGTATCTTTACTATAAAAGCTATTGTGTAAGCTAGTCATATTAAGTTGTTGGCTCAGGAGTTTGA
TAGTTCTTGGGCAGTGAGAGTGAGTAGTAG 3 (0.000090%)

AAAGGTTGGGGAACAGCTAAATAGGTTGTTGTTGATTTGGTTAAAAAATAGTAGAGGGATGATGCTAAT
AATTAGGCTGTGGGTGGTTGTGTTGATTCAA 3 (0.000090%)

AAAGTACTGATTTTAAAAACTAATAACTTAAAACTGCCACACGCAAAAAAGAAAACCAAAGTGGTCCAC
AAAACATTCTCCTTTCCTTCTGAAGGTTTTA 5 (0.000150%)

AAATAGAGACCCAGTAAAATTGTAATAAGCAGTGCTTGAATTATTTGGTTTCGGTTGTTTTCTATTAGAC
TATGGTGAGCTCAGGTGATTGATACTCCTG 9 (0.000271%)

AAATGAGTGAGGCAGGAGTCCGAGGAGGTTAGTTGTGGCAATAAAAATGATTAAGGATACTAGTATAA
GAGATCAGGTTCGTCCTTTAGTGTTGTGTATG 67 (0.002014%)

AAATTCTGTGACAAATTTTTGGTCAAGTTGTTTCCATTAAAAAGTACTGATTTTAAAAACTAATAACTTA
AAACTGCCACACGCAAAAAAGAAAACCAAA 978 (0.029400%)

AAATTGATGGCCCCTAAGATAGAGGAGACACCTGCTAGGTGTAAGGAGAAGATGGTTAGGTCTACGGAG
GCTCCAGGGTGGGAGTAGTTCCCTGCTAAGG 10 (0.000301%)

AAATTGTAATAAGCAGTGCTTGAATTATTTGGTTTCGGTTGTTTTCTATTAGACTATGGTGAGCTCAGGTG
ATTGATACTCCTGATGCGAGTAATACGGA 9 (0.000271%)

AACAATCAGGACAGCACAGTCAGCCTGAGATGTCCCTGTAATCATGTTTTTGATAAAGTCTCTGTGTCCT
GGGGCATCAATGATAGTCACATAGTACTTG 3 (0.000090%)

AACAATTTCCTCATATCTCTTCTGGCTGTAGGGTGGCTCAGTGGAATCCATTTTGTTAACACCGACAATTA
GTTGTTTCACACCCAGTGTGTAAGCCAGA 12 (0.000361%)

AACACACATGGGCTTGCCAGGAACCATATCAACAATGGCAGCATCACCAGACTTCAAGAATTTAGGGCC
ATCTTCCAGCTTTTTACCAGAACGGCGATCA 5 (0.000150%)

AACATTGTTTCACACATACATCAAACAGGCCAAAAAAAATAAACAGCAACTTCATAGACAAAAAAGGA
AAAAAAAAGAAACCTTTTATCTTTGGCCTTTT 4 (0.000120%)

AACCAAAGTGGTCCACAAAACATTCTCCTTTCCTTCTGAAGGTTTTACGATGCATTGTTATCATTAACCA
GTCTTTTACTACTAAACTTAAATGGCCAAT 4 (0.000120%)

AACTAATAACTTAAAACTGCCACACGCAAAAAAGAAAACCAAAGTGGTCCACAAAACATTCTCCTTTCC
TTCTGAAGGTTTTACGATGCATTGTTATCAT 3 (0.000090%)

AAGAAAACCAAAGTGGTCCACAAAACATTCTCCTTTCCTTCTGAAGGTTTTACGATGCATTGTTATCATT
AACCAGTCTTTTACTACTAAACTTAAATGG 18 (0.000541%)

AAGAAAGTTAGATTTACGCCGATGAATATGATAGTGAAATGGATTTTGGCGTAGGTTTGGTCTAGGGTGT
AGCCTGAGAATAGGGGAAATCAGTGAATGA 28 (0.000842%)

AAGACCCAGGCATACTTGAAGGAGCCCTTTCCCATCTCAGCAGCCTCCTTCTCAAATTTTTCAATGGTTCT
TTTGTCGATGCCACCGCATTTATAGATCA 4 (0.000120%)

AAGACGTCTTGTGATGTAATTATTATACGAATGGGGGCTTCAATCGGGAGTACTACTCGATTGTCAACGT
CAAGGAGTCGCAGGTCGCCTGGTTCTAGGA 321 (0.009650%)

AAGAGCTTCACTCAAAGCTTCATGGTGCATTTCGACAGATTTTACTTCCGTTGTAACGTTGACTGGAGCA
AAGGTGACCACCATACCGGGTTTGAGAACA 32 (0.000962%)

AAGAGGTATCTTTACTATAAAAGCTATTGTGTAAGCTAGTCATATTAAGTTGTTGGCTCAGGAGTTTGAT
AGTTCTTGGGCAGTGAGAGTGAGTAGTAGA 3 (0.000090%)

AAGAGTGGGGTGGCAGGTATTAGGGATAATATTCATTTAGCCTTCTGAGCTTTCTGGGCAGACTTGGTGA
CCTTGCCAGCTCCAGCAGCCTTCTTGTCCA 4 (0.000120%)

AAGATAGAGGAGACACCTGCTAGGTGTAAGGAGAAGATGGTTAGGTCTACGGAGGCTCCAGGGTGGGA
GTAGTTCCCTGCTAAGGGAGGGTAGACTGTTC 3 (0.000090%)

AAGCAGCGTGGTTCCACTGGCATTGCCATCCTTACGGGTGACTTTCCATCCCTTGAACCAAGGCATGTTA
GCACTTGGCTCCAGCATGTTGTCACCATTC 7 (0.000210%)

AAGCCAGAAGGGCATGCTCTCGGGTCTGCCCATTCTTGGAGATACCAGCTTCAAATTCACCAACACCAG
CAGCAACAATCAGGACAGCACAGTCAGCCTG 5 (0.000150%)

AAGCCAGCTGCTTCCATTGGTGGGTCATTTTTGCTGTCACCAGCAACGTTGCCACGACGAACATCCTTGA
CAGACACATTCTTGACATTGAAGCCCACAT 3 (0.000090%)

AAGGAAGGGGTAGGCTATGTGTTTTGTCAGGGGGTTGAGAATGAGTGTGAGGCGTATTATACCATAGCC
GCCTAGTTTTAAGAGTACTGCGGCAAGTACT 4 (0.000120%)

AAGGAGAAGATGGTTAGGTCTACGGAGGCTCCAGGGTGGGAGTAGTTCCCTGCTAAGGGAGGGTAGACT
GTTCAACCTGTTCCTGCTCCGGCCTCCACTA 4 (0.000120%)

AAGGGCATGCTCTCGGGTCTGCCCATTCTTGGAGATACCAGCTTCAAATTCACCAACACCAGCAGCAAC
AATCAGGACAGCACAGTCAGCCTGAGATGTC 3 (0.000090%)

AAGGGCTTGTCAGTTGGACGAGTTGGTGGTAGGATGCAGTCCAGAGCCTCAAGCAGCGTGGTTCCACTG
GCATTGCCATCCTTACGGGTGACTTTCCATC 5 (0.000150%)

AAGTGGAGTCCGTAAAGAGGTATCTTTACTATAAAAGCTATTGTGTAAGCTAGTCATATTAAGTTGTTGG
CTCAGGAGTTTGATAGTTCTTGGGCAGTGA 4 (0.000120%)

AATAGAGACCCAGTAAAATTGTAATAAGCAGTGCTTGAATTATTTGGTTTCGGTTGTTTTCTATTAGACT
ATGGTGAGCTCAGGTGATTGATACTCCTGA 8 (0.000240%)

AATATTCATTTAGCCTTCTGAGCTTTCTGGGCAGACTTGGTGACCTTGCCAGCTCCAGCAGCCTTCTTGTC
CACTGCTTTGATGACACCCACCGCAACTG 3 (0.000090%)

AATCACCTGAGCAGTGAAGCCAGCTGCTTCCATTGGTGGGTCATTTTTGCTGTCACCAGCAACGTTGCCA
CGACGAACATCCTTGACAGACACATTCTTG 4 (0.000120%)

AATGAGTAGGCTGATGGTTTCGATAATAACTAGTATGGGGATAAGGGGTGTAGGTGTGCCTTGTGGTAA
GAAGTGGGCTAGGGCATTTTTAATCTTAGAG 6 (0.000180%)

AATGAGTGAGGCAGGAGTCCGAGGAGGTTAGTTGTGGCAATAAAAATGATTAAGGATACTAGTATAAG
AGATCAGGTTCGTCCTTTAGTGTTGTGTATGG 6 (0.000180%)

AATTATTATACGAATGGGGGCTTCAATCGGGAGTACTACTCGATTGTCAACGTCAAGGAGTCGCAGGTC
GCCTGGTTCTAGGAATAATGGGGGAAGTATG 111 (0.003337%)

AATTATTTGGTTTCGGTTGTTTTCTATTAGACTATGGTGAGCTCAGGTGATTGATACTCCTGATGCGAGTA ATACGGATGTGTTTAGGAGTGGGACTTCT 12 (0.000361%)

AATTCTGTGACAAATTTTTGGTCAAGTTGTTTCCATTAAAAAGTACTGATTTTAAAAACTAATAACTTAA AACTGCCACACGCAAAAAAGAAAACCAAAG 15 (0.000451%)

AATTGATGGCCCCTAAGATAGAGGAGACACCTGCTAGGTGTAAGGAGAAGATGGTTAGGTCTACGGAGG CTCCAGGGTGGGAGTAGTTCCCTGCTAAGGG 11 (0.000331%)

AATTGCATCTGTTTTTAAGCCTAATGTGGGGACAGCTCATGAGTGCAAGACGTCTTGTGATGTAATTATT ATACGAATGGGGGCTTCAATCGGGAGTACT 3 (0.000090%)

AATTGTAATAAGCAGTGCTTGAATTATTTGGTTTCGGTTGTTTTCTATTAGACTATGGTGAGCTCAGGTGA TTGATACTCCTGATGCGAGTAATACGGAT 12 (0.000361%)

AATTTCCTCATATCTCTTCTGGCTGTAGGGTGGCTCAGTGGAATCCATTTTGTTAACACCGACAATTAGTT GTTTCACACCCAGTGTGTAAGCCAGAAGG 15 (0.000451%)

AATTTTCTTAATGTAAGTGCTGACTTCCTTAACAATTTCCTCATATCTCTTCTGGCTGTAGGGTGGCTCAG TGGAATCCATTTTGTTAACACCGACAATT 28 (0.000842%)

AATTTTGTAGACATCCTGGAGAGGCAGGCGCAAGGGCTTGTCAGTTGGACGAGTTGGTGGTAGGATGCA GTCCAGAGCCTCAAGCAGCGTGGTTCCACTG 13 (0.000391%)

ACAAGGAAGGGGTAGGCTATGTGTTTTGTCAGGGGGTTGAGAATGAGTGTGAGGCGTATTATACCATAG CCGCCTAGTTTTAAGAGTACTGCGGCAAGTA 3 (0.000090%)

ACAATTTCCTCATATCTCTTCTGGCTGTAGGGTGGCTCAGTGGAATCCATTTTGTTAACACCGACAATTAG TTGTTTCACACCCAGTGTGTAAGCCAGAA 5 (0.000150%)

ACACACATGGGCTTGCCAGGAACCATATCAACAATGGCAGCATCACCAGACTTCAAGAATTTAGGGCCA TCTTCCAGCTTTTTACCAGAACGGCGATCAA 3 (0.000090%)

ACACATGAGTATTTGTCTAAAACATGTCTTCTTTGTAGCAGCTAGGCCCTGCCACCACTGTGCTTGGCTG AGTTCACAAATCTGTTGTAACCTGTAGCTT 3 (0.000090%)

ACACGCAAAAAAGAAAACCAAAGTGGTCCACAAAACATTCTCCTTTCCTTCTGAAGGTTTTACGATGCAT TGTTATCATTAACCAGTCTTTTACTACTAA 4 (0.000120%)

ACAGAACCACGAGCAGCAACTACCATGGGTGAGAGGATCTTTGAGGGGGTGTGACCCTTGAGGTCAGAA GAGGCCTCGAGAGGCCAGGCCTAACAGGGTTGGCAGCTGCACTAAAGCCTGGGGCAGCTCCCTTTCCAA AAGGACACTGC 2 (0.000090%)

ACATGGGCTTGCCAGGAACCATATCAACAATGGCAGCATCACCAGACTTCAAGAATTTAGGGCCATCTT CCAGCTTTTTACCAGAACGGCGATCAATCTT 3 (0.000090%)

ACATTGTCCCCAGGAAGAGCTTCACTCAAAGCTTCATGGTGCATTTCGACAGATTTTACTTCCGTTGTAA CGTTGACTGGAGCAAAGGTGACCACCATAC 13 (0.000391%)

ACCAAAGTGGTCCACAAAACATTCTCCTTTCCTTCTGAAGGTTTTACGATGCATTGTTATCATTAACCAGT CTTTTACTACTAAACTTAAATGGCCAATT 5 (0.000150%)

ACCCAGTGTGTAAGCCAGAAGGGCATGCTCTCGGGTCTGCCCATTCTTGGAGATACCAGCTTCAAATTCA CCAACACCAGCAGCAACAATCAGGACAGCA 3 (0.000090%)

ACCGTTCTTCCACCACTGATTAAGAGTGGGGTGGCAGGTATTAGGGATAATATTCATTTAGCCTTCTGAG CTTTCTGGGCAGACTTGGTGACCTTGCCAG 8 (0.000240%)

ACCTGAGCAGTGAAGCCAGCTGCTTCCATTGGTGGGTCATTTTTGCTGTCACCAGCAACGTTGCCACGAC
GAACATCCTTGACAGACACATTCTTGACAT 3 (0.000090%)

ACCTTTCTCTTTGGCTTCTTTCTTTTTCTGATCATTTTCCTTCACACGTTTCAGGAAGCTATCTCGGCTCTT
AGAGTGCTTAATGTGCTCAATACGCACA 3 (0.000090%)

ACGAGTTGGTGGTAGGATGCAGTCCAGAGCCTCAAGCAGCGTGGTTCCACTGGCATTGCCATCCTTACG
GGTGACTTTCCATCCCTTGAACCAAGGCATG 11 (0.000331%)

ACGCAAAAAAGAAAACCAAAGTGGTCCACAAAACATTCTCCTTTCCTTCTGAAGGTTTTACGATGCATTG
TTATCATTAACCAGTCTTTTACTACTAAAC12 (0.000361%)

ACTGATTTTAAAAACTAATAACTTAAAACTGCCACACGCAAAAAAGAAAACCAAAGTGGTCCACAAAAC
ATTCTCCTTTCCTTCTGAAGGTTTTACGATG 10 (0.000301%)

ACTGCCACACGCAAAAAAGAAAACCAAAGTGGTCCACAAAACATTCTCCTTTCCTTCTGAAGGTTTTACG
ATGCATTGTTATCATTAACCAGTCTTTTAC8 (0.000240%)

ACTTAAAACTGCCACACGCAAAAAAGAAAACCAAAGTGGTCCACAAAACATTCTCCTTTCCTTCTGAAG
GTTTTACGATGCATTGTTATCATTAACCAGT 3 (0.000090%)

ACTTCCTTAACAATTTCCTCATATCTCTTCTGGCTGTAGGGTGGCTCAGTGGAATCCATTTTGTTAACACC
GACAATTAGTTGTTTCACACCCAGTGTGT 4 (0.000120%)

ACTTGGTGACCTTGCCAGCTCCAGCAGCCTTCTTGTCCACTGCTTTGATGACACCCACCGCAACTGTCTGT
CTCATATCACGAACAGCAAAGCGACCCAA 5 (0.000150%)

AGAAAACCAAAGTGGTCCACAAAACATTCTCCTTTCCTTCTGAAGGTTTTACGATGCATTGTTATCATTA
ACCAGTCTTTTACTACTAAACTTAAATGGC 14 (0.000421%)

AGAAAGATGAATCCTAGGGCTCAGAGCACTGCAGCAGATCATTTCATATTGCTTCCGTGGAGTGTGGCG
AGTCAGCTAAATACTTTGACGCCGGTGGGGA 9 (0.000271%)

AGAAAGTTAGATTTACGCCGATGAATATGATAGTGAAATGGATTTTGGCGTAGGTTTGGTCTAGGGTGTA
GCCTGAGAATAGGGGAAATCAGTGAATGAA 21 (0.000631%)

AGAAGCTCTCAACACACATGGGCTTGCCAGGAACCATATCAACAATGGCAGCATCACCAGACTTCAAGA
ATTTAGGGCCATCTTCCAGCTTTTTACCAGA 11 (0.000331%)

AGAAGGGCATGCTCTCGGGTCTGCCCATTCTTGGAGATACCAGCTTCAAATTCACCAACACCAGCAGCA
ACAATCAGGACAGCACAGTCAGCCTGAGATG 27 (0.000812%)

AGAATGATGGCTAGGGTGACTTCATATGAGATTGTTTGGGCTACTGCTCGCAGTGCGCCGATCAGGGCGT
AGTTTGAGTTTGATGCTCACCCTGATCAGA 203 (0.006102%)

AGACATCCTGGAGAGGCAGGCGCAAGGGCTTGTCAGTTGGACGAGTTGGTGGTAGGATGCAGTCCAGAG
CCTCAAGCAGCGTGGTTCCACTGGCATTGCC 6 (0.000180%)

AGACCCAGTAAAATTGTAATAAGCAGTGCTTGAATTATTTGGTTTCGGTTGTTTTCTATTAGACTATGGTG
AGCTCAGGTGATTGATACTCCTGATGCGA 3 (0.000090%)

AGACCGTTCTTCCACCACTGATTAAGAGTGGGGTGGCAGGTATTAGGGATAATATTCATTTAGCCTTCTG
AGCTTTCTGGGCAGACTTGGTGACCTTGCC 5 (0.000150%)

AGACGTCTTGTGATGTAATTATTATACGAATGGGGGCTTCAATCGGGAGTACTACTCGATTGTCAACGTC
AAGGAGTCGCAGGTCGCCTGGTTCTAGGAA 3 (0.000090%)

AGACTTGGTGACCTTGCCAGCTCCAGCAGCCTTCTTGTCCACTGCTTTGATGACACCCACCGCAACTGTC
TGTCTCATATCACGAACAGCAAAGCGACCC 4 (0.000120%)

AGAGCACTGCAGCAGATCATTTCATATTGCTTCCGTGGAGTGTGGCGAGTCAGCTAAATACTTTGACGCC
GGTGGGGATAGCGATGATTATGGTAGCGGA 6 (0.000180%)

AGAGCTTCACTCAAAGCTTCATGGTGCATTTCGACAGATTTTACTTCCGTTGTAACGTTGACTGGAGCAA
AGGTGACCACCATACCGGGTTTGAGAACAC 12 (0.000361%)

AGAGGCAGGCGCAAGGGCTTGTCAGTTGGACGAGTTGGTGGTAGGATGCAGTCCAGAGCCTCAAGCAGC
GTGGTTCCACTGGCATTGCCATCCTTACGGG 6 (0.000180%)

AGAGGGAGTATAGGGCTGTGACTAGTATGTTGAGTCCTGTAAGTAGGAGAGTGATATTTGATCAGGAGA
ACGTGGTTACTAGCACAGAGAGTTCTCCCAG 4 (0.000120%)

AGAGGTGATCGGCGATCAGAGGGCGATGAAGTTCTAGATCCATTGAGACAAGCTCTAGACAGTAGCATG
CAGTCCCACAACTTGTACCAGCATCCCCAGC 441 (0.013257%)

AGAGTGGGGTGGCAGGTATTAGGGATAATATTCATTTAGCCTTCTGAGCTTTCTGGGCAGACTTGGTGAC
CTTGCCAGCTCCAGCAGCCTTCTTGTCCAC 5 (0.000150%)

AGATAGTTGGGTGGTTGGTGTAAATGAGTGAGGCAGGAGTCCGAGGAGGTTAGTTGTGGCAATAAAAAT
GATTAAGGATACTAGTATAAGAGATCAGGTT 22 (0.000661%)

AGCAACAATCAGGACAGCACAGTCAGCCTGAGATGTCCCTGTAATCATGTTTTTGATAAAGTCTCTGTGT
CCTGGGGCATCAATGATAGTCACATAGTAC 16 (0.000481%)

AGCACTGCAGCAGATCATTTCATATTGCTTCCGTGGAGTGTGGCGAGTCAGCTAAATACTTTGACGCCGG
TGGGGATAGCGATGATTATGGTAGCGGAGG 4 (0.000120%)

AGCAGATCATTTCATATTGCTTCCGTGGAGTGTGGCGAGTCAGCTAAATACTTTGACGCCGGTGGGGATA
GCGATGATTATGGTAGCGGAGGTGAAATAT 13 (0.000391%)

AGCAGCGTGGTTCCACTGGCATTGCCATCCTTACGGGTGACTTTCCATCCCTTGAACCAAGGCATGTTAG
CACTTGGCTCCAGCATGTTGTCACCATTCC 13 (0.000391%)

AGCCAATTTTCTTAATGTAAGTGCTGACTTCCTTAACAATTTCCTCATATCTCTTCTGGCTGTAGGGTGGC
TCAGTGGAATCCATTTTGTTAACACCGAC 6 (0.000180%)

AGCCTTCTGAGCTTTCTGGGCAGACTTGGTGACCTTGCCAGCTCCAGCAGCCTTCTTGTCCACTGCTTTGA
TGACACCCACCGCAACTGTCTGTCTCATA 11 (0.000331%)

AGCGGTTAGGCGTACGGCCAGGGCTATTGGTTGAATGAGTAGGCTGATGGTTTCGATAATAACTAGTAT
GGGGATAAGGGGTGTAGGTGTGCCTTGTGGT 11 (0.000331%)

AGCGTGGTTCCACTGGCATTGCCATCCTTACGGGTGACTTTCCATCCCTTGAACCAAGGCATGTTAGCAC
TTGGCTCCAGCATGTTGTCACCATTCCAAC 15 (0.000451%)

AGCTCCAGCAGCCTTCTTGTCCACTGCTTTGATGACACCCACCGCAACTGTCTGTCTCATATCACGAACA
GCAAAGCGACCCAAAGGTGGATAGTCTGAG 3 (0.000090%)

AGCTCTCAACACACATGGGCTTGCCAGGAACCATATCAACAATGGCAGCATCACCAGACTTCAAGAATT
TAGGGCCATCTTCCAGCTTTTTACCAGAACG 6 (0.000180%)

AGCTGCTTCCATTGGTGGGTCATTTTTGCTGTCACCAGCAACGTTGCCACGACGAACATCCTTGACAGAC
ACATTCTTGACATTGAAGCCCACATTGTCC 4 (0.000120%)

AGGAAAAGGTTGGGGAACAGCTAAATAGGTTGTTGTTGATTTGGTTAAAAAATAGTAGAGGGATGATGC
TAATAATTAGGCTGTGGGTGGTTGTGTTGAT 4 (0.000120%)

AGGAAGAGCTTCACTCAAAGCTTCATGGTGCATTTCGACAGATTTTACTTCCGTTGTAACGTTGACTGGA

GCAAAGGTGACCACCATACCGGGTTTGAGA 25 (0.000752%)

AGGAAGGGGTAGGCTATGTGTTTTGTCAGGGGGTTGAGAATGAGTGTGAGGCGTATTATACCATAGCCG
CCTAGTTTTAAGAGTACTGCGGCAAGTACTA 4 (0.000120%)

AGGAGAAGATGGTTAGGTCTACGGAGGCTCCAGGGTGGGAGTAGTTCCCTGCTAAGGGAGGGTAGACTG
TTCAACCTGTTCCTGCTCCGGCCTCCACTAT 4 (0.000120%)

AGGAGACACCTGCTAGGTGTAAGGAGAAGATGGTTAGGTCTACGGAGGCTCCAGGGTGGGAGTAGTTCC
CTGCTAAGGGAGGGTAGACTGTTCAACCTGT 6 (0.000180%)

AGGAGGGTAAAATAGAGACCCAGTAAAATTGTAATAAGCAGTGCTTGAATTATTTGGTTTCGGTTGTTTT
CTATTAGACTATGGTGAGCTCAGGTGATTG 189 (0.005682%)

AGGATAATCACCTGAGCAGTGAAGCCAGCTGCTTCCATTGGTGGGTCATTTTTGCTGTCACCAGCAACGT
TGCCACGACGAACATCCTTGACAGACACAT 12 (0.000361%)

AGGATGCAGTCCAGAGCCTCAAGCAGCGTGGTTCCACTGGCATTGCCATCCTTACGGGTGACTTTCCATC
CCTTGAACCAAGGCATGTTAGCACTTGGCT 6 (0.000180%)

AGGCCACCTACGGTGAAAAGAAAGATGAATCCTAGGGCTCAGAGCACTGCAGCAGATCATTTCATATTG
CTTCCGTGGAGTGTGGCGAGTCAGCTAAATA 16 (0.000481%)

AGGCGCAAGGGCTTGTCAGTTGGACGAGTTGGTGGTAGGATGCAGTCCAGAGCCTCAAGCAGCGTGGTT
CCACTGGCATTGCCATCCTTACGGGTGACTT 16 (0.000481%)

AGGCTTATGCGGAGGAGAATGTTTTCATGTTACTTATACTAACATTAGTTCTTCTATAGGGTGATAGATT
GGTCCAATTGGGTGTGAGGAGTTCAGTTAT 8 (0.000240%)

AGGGAGTCATAAGTGGAGTCCGTAAAGAGGTATCTTTACTATAAAAGCTATTGTGTAAGCTAGTCATATT
AAGTTGTTGGCTCAGGAGTTTGATAGTTCT 7 (0.000210%)

AGGGATAATATTCATTTAGCCTTCTGAGCTTTCTGGGCAGACTTGGTGACCTTGCCAGCTCCAGCAGCCT
TCTTGTCCACTGCTTTGATGACACCCACCG 4 (0.000120%)

AGGGATAGTACAAGGAAGGGGTAGGCTATGTGTTTTGTCAGGGGGTTGAGAATGAGTGTGAGGCGTATT
ATACCATAGCCGCCTAGTTTTAAGAGTACTG 9 (0.000271%)

AGGGCATGCTCTCGGGTCTGCCCATTCTTGGAGATACCAGCTTCAAATTCACCAACACCAGCAGCAACA
ATCAGGACAGCACAGTCAGCCTGAGATGTCC 6 (0.000180%)

AGGGCTATTGGTTGAATGAGTAGGCTGATGGTTTCGATAATAACTAGTATGGGGATAAGGGGTGTAGGT
GTGCCTTGTGGTAAGAAGTGGGCTAGGGCAT 3 (0.000090%)

AGGGCTCAGAGCACTGCAGCAGATCATTTCATATTGCTTCCGTGGAGTGTGGCGAGTCAGCTAAATACTT
TGACGCCGGTGGGGATAGCGATGATTATGG 17 (0.000511%)

AGGGCTTGTCAGTTGGACGAGTTGGTGGTAGGATGCAGTCCAGAGCCTCAAGCAGCGTGGTTCCACTGG
CATTGCCATCCTTACGGGTGACTTTCCATCC 4 (0.000120%)

AGGGGGTCGGAGGAAAAGGTTGGGGAACAGCTAAATAGGTTGTTGTTGATTTGGTTAAAAAATAGTAGA
GGGATGATGCTAATAATTAGGCTGTGGGTGG 5 (0.000150%)

AGGGTGACTTCATATGAGATTGTTTGGGCTACTGCTCGCAGTGCGCCGATCAGGGCGTAGTTTGAGTTTG
ATGCTCACCCTGATCAGAGGATTGAGTAAA 3 (0.000090%)

AGGTATTAGGGATAATATTCATTTAGCCTTCTGAGCTTTCTGGGCAGACTTGGTGACCTTGCCAGCTCCA
GCAGCCTTCTTGTCCACTGCTTTGATGACA 22 (0.000661%)

AGGTGATCGGCGATCAGAGGGCGATGAAGTTCTAGATCCATTGAGACAAGCTCTAGACAGTAGCATGCA
GTCCCACAACTTGTACCAGCATCCCCAGCGT 38 (0.001142%)

AGGTGCATGAGTAGGTGGCCTGCAGTAATGTTAGCGGTTAGGCGTACGGCCAGGGCTATTGGTTGAATG
AGTAGGCTGATGGTTTCGATAATAACTAGTA 4 (0.000120%)

AGGTGGCCTGCAGTAATGTTAGCGGTTAGGCGTACGGCCAGGGCTATTGGTTGAATGAGTAGGCTGATG
GTTTCGATAATAACTAGTATGGGGATAAGGG 14 (0.000421%)

AGTAATGTTAGCGGTTAGGCGTACGGCCAGGGCTATTGGTTGAATGAGTAGGCTGATGGTTTCGATAAT
AACTAGTATGGGGATAAGGGGTGTAGGTGTG 9 (0.000271%)

AGTACTACTCGATTGTCAACGTCAAGGAGTCGCAGGTCGCCTGGTTCTAGGAATAATGGGGGAAGTATG
TAGGAGTTGAAGATTAGTCCGCCGTAGTCGG 4 (0.000120%)

AGTACTCTGAGGCTTGTAGGAGGGTAAAATAGAGACCCAGTAAAATTGTAATAAGCAGTGCTTGAATTA
TTTGGTTTCGGTTGTTTCTATTAGACTATG 4 (0.000120%)

AGTAGGTGGCCTGCAGTAATGTTAGCGGTTAGGCGTACGGCCAGGGCTATTGGTTGAATGAGTAGGCTG
ATGGTTTCGATAATAACTAGTATGGGGATAA 44 (0.001323%)

AGTCAGGCCACCTACGGTGAAAAGAAAGATGAATCCTAGGGCTCAGAGCACTGCAGCAGATCATTTCAT
ATTGCTTCCGTGGAGTGTGGCGAGTCAGCTA 35 (0.001052%)

AGTGAAGCCAGCTGCTTCCATTGGTGGGTCATTTTTGCTGTCACCAGCAACGTTGCCACGACGAACATCC
TTGACAGACACATTCTTGACATTGAAGCCC 6 (0.000180%)

AGTGAGGCAGGAGTCCGAGGAGGTTAGTTGTGGCAATAAAAATGATTAAGGATACTAGTATAAGAGATC
AGGTTCGTCCTTTAGTGTTGTGTATGGTTAT 3 (0.000090%)

AGTGCTGACTTCCTTAACAATTTCCTCATATCTCTTCTGGCTGTAGGGTGGCTCAGTGGAATCCATTTTGT
TAACACCGACAATTAGTTGTTTCACACCC 3 (0.000090%)

AGTGGGGTGGCAGGTATTAGGGATAATATTCATTTAGCCTTCTGAGCTTTCTGGGCAGACTTGGTGACCT
TGCCAGCTCCAGCAGCCTTCTTGTCCACTG 5 (0.000150%)

AGTGTGTAAGCCAGAAGGGCATGCTCTCGGGTCTGCCCATTCTTGGAGATACCAGCTTCAAATTCACCAA
CACCAGCAGCAACAATCAGGACAGCACAGT 6 (0.000180%)

AGTTAGTATTAGGAGGGGGGTTGTTAGGGGGTCGGAGGAAAAGGTTGGGGAACAGCTAAATAGGTTGTT
GTTGATTTGGTTAAAAAATAGTAGAGGGATG 249 (0.007485%)

AGTTGGACGAGTTGGTGGTAGGATGCAGTCCAGAGCCTCAAGCAGCGTGGTTCCACTGGCATTGCCATC
CTTACGGGTGACTTTCCATCCCTTGAACCAA 12 (0.000361%)

AGTTGGGTGGTTGGTGTAAATGAGTGAGGCAGGAGTCCGAGGAGGTTAGTTGTGGCAATAAAAATGATT
AAGGATACTAGTATAAGAGATCAGGTTCGTC 3 (0.000090%)

AGTTGGTGGTAGGATGCAGTCCAGAGCCTCAAGCAGCGTGGTTCCACTGGCATTGCCATCCTTACGGGTG
ACTTTCCATCCCTTGAACCAAGGCATGTTA 7 (0.000210%)

ATAAGTGGAGTCCGTAAAGAGGTATCTTTACTATAAAAGCTATTGTGTAAGCTAGTCATATTAAGTTGTT
GGCTCAGGAGTTTGATAGTTCTTGGGCAGT 4 (0.000120%)

ATAATATTCATTTAGCCTTCTGAGCTTTCTGGGCAGACTTGGTGACCTTGCCAGCTCCAGCAGCCTTCTTG
TCCACTGCTTTGATGACACCCACCGCAAC 85 (0.002555%)

ATAATCACCTGAGCAGTGAAGCCAGCTGCTTCCATTGGTGGGTCATTTTTGCTGTCACCAGCAACGTTGC
CACGACGAACATCCTTGACAGACACATTCT 7 (0.000210%)

ATACAATGCCAGTCAGGCCACCTACGGTGAAAAGAAAGATGAATCCTAGGGCTCAGAGCACTGCAGCA GATCATTTCATATTGCTTCCGTGGAGTGTGGC 8 (0.000240%)

ATACACATGAGTATTTGTCTAAAACATGTCTTCTTTGTAGCAGCTAGGCCCTGCCACCACTGTGCTTGGCT GAGTTCACAAATCTGTTGTAACCTGTAGC 23 (0.000691%)

ATACGAATGGGGGCTTCAATCGGGAGTACTACTCGATTGTCAACGTCAAGGAGTCGCAGGTCGCCTGGT TCTAGGAATAATGGGGGAAGTATGTAGGAGT 12 (0.000361%)

ATAGAGACCCAGTAAAATTGTAATAAGCAGTGCTTGAATTATTTGGTTTCGGTTGTTTTCTATTAGACTAT GGTGAGCTCAGGTGATTGATACTCCTGAT 19 (0.000571%)

ATAGAGGAGACACCTGCTAGGTGTAAGGAGAAGATGGTTAGGTCTACGGAGGCTCCAGGGTGGGAGTA GTTCCCTGCTAAGGGAGGGTAGACTGTTCAAC 3 (0.000090%)

ATAGATAGTTGGGTGGTTGGTGTAAATGAGTGAGGCAGGAGTCCGAGGAGGTTAGTTGTGGCAATAAAA ATGATTAAGGATACTAGTATAAGAGATCAGG 16 (0.000481%)

ATAGGGATAGTACAAGGAAGGGGTAGGCTATGTGTTTTGTCAGGGGGTTGAGAATGAGTGTGAGGCGTA TTATACCATAGCCGCCTAGTTTTAAGAGTAC 6 (0.000180%)

ATATTCATTTAGCCTTCTGAGCTTTCTGGGCAGACTTGGTGACCTTGCCAGCTCCAGCAGCCTTCTTGTCC ACTGCTTTGATGACACCCACCGCAACTGT 12 (0.000361%)

ATCATGTTTTTGATAAAGTCTCTGTGTCCTGGGGCATCAATGATAGTCACATAGTACTTGCTGGTCTCAA ATTTCCACAAGGAGATATCAATGGTGATAC 4 (0.000120%)

ATCCTAGGGCTCAGAGCACTGCAGCAGATCATTTCATATTGCTTCCGTGGAGTGTGGCGAGTCAGCTAAA TACTTTGACGCCGGTGGGGATAGCGATGAT 3 (0.000090%)

ATCGGGAGTACTACTCGATTGTCAACGTCAAGGAGTCGCAGGTCGCCTGGTTCTAGGAATAATGGGGGA AGTATGTAGGAGTTGAAGATTAGTCCGCCGT 7 (0.000210%)

ATGAAATTGATGGCCCCTAAGATAGAGGAGACACCTGCTAGGTGTAAGGAGAAGATGGTTAGGTCTACG GAGGCTCCAGGGTGGGAGTAGTTCCCTGCTA 270 (0.008117%)

ATGAATCCTAGGGCTCAGAGCACTGCAGCAGATCATTTCATATTGCTTCCGTGGAGTGTGGCGAGTCAGC TAAATACTTTGACGCCGGTGGGGATAGCGA 4 (0.000120%)

ATGAGTATTTGTCTAAAACATGTCTTCTTTGTAGCAGCTAGGCCCTGCCACCACTGTGCTTGGCTGAGTTC ACAAATCTGTTGTAACCTGTAGCTTCCCT 4 (0.000120%)

ATGAGTGAGGCAGGAGTCCGAGGAGGTTAGTTGTGGCAATAAAAATGATTAAGGATACTAGTATAAGA GATCAGGTTCGTCCTTTAGTGTTGTGTATGGT 3 (0.000090%)

ATGATGGCTAGGGTGACTTCATATGAGATTGTTTGGGCTACTGCTCGCAGTGCGCCGATCAGGGCGTAGT TTGAGTTTGATGCTCACCCTGATCAGAGGA 5 (0.000150%)

ATGCAGTCCAGAGCCTCAAGCAGCGTGGTTCCACTGGCATTGCCATCCTTACGGGTGACTTTCCATCCCT TGAACCAAGGCATGTTAGCACTTGGCTCCA 20 (0.000601%)

ATGCTACTTGTCCAATGATGGTAAAAGGGTAGCTTACTGGTTGTCCTCCGATTCAGGTTAGAATGAGGAG GTCTGCGGCTAGGAGTCAATAAAGTGATTG 4 (0.000120%)

ATGCTCTCGGGTCTGCCCATTCTTGGAGATACCAGCTTCAAATTCACCAACACCAGCAGCAACAATCAGG ACAGCACAGTCAGCCTGAGATGTCCCTGTA 11 (0.000331%)

ATGGACACCAGTTTTAGCCAACATAGCATAGTACTCTATTTCAGATTTCCTCAAAGCTGGGCAGTTGTTA

GCGAGAATGACCAATTTCGCTTTGCCTTGT 10 (0.000301%)

ATGGCCCCTAAGATAGAGGAGACACCTGCTAGGTGTAAGGAGAAGATGGTTAGGTCTACGGAGGCTCCA
GGGTGGGAGTAGTTCCCTGCTAAGGGAGGGT 7 (0.000210%)

ATGGCTAGGTTTATAGATAGTTGGGTGGTTGGTGTAAATGAGTGAGGCAGGAGTCCGAGGAGGTTAGTT
GTGGCAATAAAAATGATTAAGGATACTAGTA 10 (0.000301%)

ATGGGCTTGCCAGGAACCATATCAACAATGGCAGCATCACCAGACTTCAAGAATTTAGGGCCATCTTCC
AGCTTTTTACCAGAACGGCGATCAATCTTTT 4 (0.000120%)

ATGGGCTTTAGGGAGTCATAAGTGGAGTCCGTAAAGAGGTATCTTTACTATAAAAGCTATTGTGTAAGCT
AGTCATATTAAGTTGTTGGCTCAGGAGTTT 11 (0.000331%)

ATGTAAGTGCTGACTTCCTTAACAATTTCCTCATATCTCTTCTGGCTGTAGGGTGGCTCAGTGGAATCCAT
TTTGTTAACACCGACAATTAGTTGTTTCA 3 (0.000090%)

ATGTTAGCGGTTAGGCGTACGGCCAGGGCTATTGGTTGAATGAGTAGGCTGATGGTTTCGATAATAACTA
GTATGGGGATAAGGGGTGTAGGTGTGCCTT 3 (0.000090%)

ATGTTTTTGATAAAGTCTCTGTGTCCTGGGGCATCAATGATAGTCACATAGTACTTGCTGGTCTCAAATTT
CCACAAGGAGATATCAATGGTGATACCAC 3 (0.000090%)

ATTAAAAAGTACTGATTTTAAAAACTAATAACTTAAAACTGCCACACGCAAAAAAGAAAACCAAAGTGG
TCCACAAAACATTCTCCTTTCCTTCTGAAGG 24 (0.000721%)

ATTAAGAGTGGGGTGGCAGGTATTAGGGATAATATTCATTTAGCCTTCTGAGCTTTCTGGGCAGACTTGG
TGACCTTGCCAGCTCCAGCAGCCTTCTTGT 13 (0.000391%)

ATTAGACTATGGTGAGCTCAGGTGATTGATACTCCTGATGCGAGTAATACGGATGTGTTTAGGAGTGGG
ACTTCTAGGGGATTTAGCGGGGTGATGCCTG 17 (0.000511%)

ATTAGGGATAATATTCATTTAGCCTTCTGAGCTTTCTGGGCAGACTTGGTGACCTTGCCAGCTCCAGCAG
CCTTCTTGTCCACTGCTTTGATGACACCCA45 (0.001353%)

ATTAGGTGCATGAGTAGGTGGCCTGCAGTAATGTTAGCGGTTAGGCGTACGGCCAGGGCTATTGGTTGA
ATGAGTAGGCTGATGGTTTCGATAATAACTA 113 (0.003397%)

ATTATACGAATGGGGGCTTCAATCGGGAGTACTACTCGATTGTCAACGTCAAGGAGTCGCAGGTCGCCT
GGTTCTAGGAATAATGGGGGAAGTATGTAGG 10 (0.000301%)

ATTATTATACGAATGGGGGCTTCAATCGGGAGTACTACTCGATTGTCAACGTCAAGGAGTCGCAGGTCG
CCTGGTTCTAGGAATAATGGGGGAAGTATGT 4 (0.000120%)

ATTATTTGGTTTCGGTTGTTTTCTATTAGACTATGGTGAGCTCAGGTGATTGATACTCCTGATGCGAGTAA
TACGGATGTGTTTAGGAGTGGGACTTCTA 6 (0.000180%)

ATTCATTTAGCCTTCTGAGCTTTCTGGGCAGACTTGGTGACCTTGCCAGCTCCAGCAGCCTTCTTGTCCAC
TGCTTTGATGACACCCACCGCAACTGTCT 191 (0.005742%)

ATTCTCATGGTTTGGGTTGGGTGGTGGAGAGCGCGTGTCATCTGCGGGTGGCACTGCCCACGGTGGGCG
GGCGGGCCTCTCTACTCGAAGGTGACCACGT 3 (0.000090%)

ATTCTGTGACAAATTTTTGGTCAAGTTGTTTCCATTAAAAAGTACTGATTTTAAAAACTAATAACTTAAA
ACTGCCACACGCAAAAAAGAAAACCAAAGT 7 (0.000210%)

ATTGATGGCCCCTAAGATAGAGGAGACACCTGCTAGGTGTAAGGAGAAGATGGTTAGGTCTACGGAGGC
TCCAGGGTGGGAGTAGTTCCCTGCTAAGGGA 10 (0.000301%)

ATTGCATCTGTTTTTAAGCCTAATGTGGGGACAGCTCATGAGTGCAAGACGTCTTGTGATGTAATTATTA
TACGAATGGGGGCTTCAATCGGGAGTACTA 16 (0.000481%)

ATTGCTAGGGTGGCGCTTCCAATTAGGTGCATGAGTAGGTGGCCTGCAGTAATGTTAGCGGTTAGGCGTA
CGGCCAGGGCTATTGGTTGAATGAGTAGGC 7 (0.000210%)

ATTGGTTGAATGAGTAGGCTGATGGTTTCGATAATAACTAGTATGGGGATAAGGGGTGTAGGTGTGCCTT
GTGGTAAGAAGTGGGCTAGGGCATTTTTAA 9 (0.000271%)

ATTGTAATAAGCAGTGCTTGAATTATTTGGTTTCGGTTGTTTTCTATTAGACTATGGTGAGCTCAGGTGAT
TGATACTCCTGATGCGAGTAATACGGATG 5 (0.000150%)

ATTGTCCCCAGGAAGAGCTTCACTCAAAGCTTCATGGTGCATTTCGACAGATTTTACTTCCGTTGTAACG
TTGACTGGAGCAAAGGTGACCACCATACCG 31 (0.000932%)

ATTTACGCCGATGAATATGATAGTGAAATGGATTTTGGCGTAGGTTTGGTCTAGGGTGTAGCCTGAGAAT
AGGGGAAATCAGTGAATGAAGCCTCCTATG 10 (0.000301%)

ATTTAGCCTTCTGAGCTTTCTGGGCAGACTTGGTGACCTTGCCAGCTCCAGCAGCCTTCTTGTCCACTGCT
TTGATGACACCCACCGCAACTGTCTGTCT 68 (0.002044%)

ATTTCCTCATATCTCTTCTGGCTGTAGGGTGGCTCAGTGGAATCCATTTTGTTAACACCGACAATTAGTTG
TTTCACACCCAGTGTGTAAGCCAGAAGGG 18 (0.000541%)

ATTTGTCTAAAACATGTCTTCTTTGTAGCAGCTAGGCCCTGCCACCACTGTGCTTGGCTGAGTTCACAAAT
CTGTTGTAACCTGTAGCTTCCCTGTCACT 8 (0.000240%)

ATTTTAAAAACTAATAACTTAAAACTGCCACACGCAAAAAAGAAAACCAAAGTGGTCCACAAAACATTC
TCCTTTCCTTCTGAAGGTTTTACGATGCATT 13 (0.000391%)

ATTTTCTTAATGTAAGTGCTGACTTCCTTAACAATTTCCTCATATCTCTTCTGGCTGTAGGGTGGCTCAGT
GGAATCCATTTTGTTAACACCGACAATTA 24 (0.000721%)

ATTTTGTAGACATCCTGGAGAGGCAGGCGCAAGGGCTTGTCAGTTGGACGAGTTGGTGGTAGGATGCAG
TCCAGAGCCTCAAGCAGCGTGGTTCCACTGG 24 (0.000721%)

ATTTTTGCTGTCACCAGCAACGTTGCCACGACGAACATCCTTGACAGACACATTCTTGACATTGAAGCCC
ACATTGTCCCCAGGAAGAGCTTCACTCAAA 42 (0.001263%)

CAAAAAAGAAAACCAAAGTGGTCCACAAAACATTCTCCTTTCCTTCTGAAGGTTTTACGATGCATTGTTA
TCATTAACCAGTCTTTTACTACTAAACTTA53 (0.001593%)

CAAATTCTCATGGTTTGGGTTGGGTGGTGGAGAGCGCGTGTCATCTGCGGGTGGCACTGCCCACGGTGG
GCGGGCGGGCCTCTCTACTCGAAGGTGACCA 10 (0.000301%)

CAACAATCAGGACAGCACAGTCAGCCTGAGATGTCCCTGTAATCATGTTTTTGATAAAGTCTCTGTGTCC
TGGGGCATCAATGATAGTCACATAGTACTT 53 (0.001593%)

CAACACACATGGGCTTGCCAGGAACCATATCAACAATGGCAGCATCACCAGACTTCAAGAATTTAGGGC
CATCTTCCAGCTTTTTACCAGAACGGCGATC 7 (0.000210%)

CAACATTGTTTCACACATACATCAAACAGGCCAAAAAAAATAAACAGCAACTTCATAGACAAAAAAGG
AAAAAAAAAGAAACCTTTTATCTTTGGCCTTT 310 (0.009319%)

CAAGACCCAGGCATACTTGAAGGAGCCCTTTCCCATCTCAGCAGCCTCCTTCTCAAATTTTTCAATGGTT
CTTTTGTCGATGCCACCGCATTTATAGATC3 (0.000090%)

CAAGCAGCGTGGTTCCACTGGCATTGCCATCCTTACGGGTGACTTTCCATCCCTTGAACCAAGGCATGTT
AGCACTTGGCTCCAGCATGTTGTCACCATT 10 (0.000301%)

CAAGGAAGGGGTAGGCTATGTGTTTTGTCAGGGGGTTGAGAATGAGTGTGAGGCGTATTATACCATAGC
CGCCTAGTTTTAAGAGTACTGCGGCAAGTAC 14 (0.000421%)

CAAGGGCTTGTCAGTTGGACGAGTTGGTGGTAGGATGCAGTCCAGAGCCTCAAGCAGCGTGGTTCCACT
GGCATTGCCATCCTTACGGGTGACTTTCCAT 13 (0.000391%)

CAATCGGGAGTACTACTCGATTGTCAACGTCAAGGAGTCGCAGGTCGCCTGGTTCTAGGAATAATGGGG
GAAGTATGTAGGAGTTGAAGATTAGTCCGCC 5 (0.000150%)

CAATGCCAGTCAGGCCACCTACGGTGAAAAGAAAGATGAATCCTAGGGCTCAGAGCACTGCAGCAGATC
ATTTCATATTGCTTCCGTGGAGTGTGGCGAG 3 (0.000090%)

CAATTTCCTCATATCTCTTCTGGCTGTAGGGTGGCTCAGTGGAATCCATTTTGTTAACACCGACAATTAGT
TGTTTCACACCCAGTGTGTAAGCCAGAAG 12 (0.000361%)

CAATTTTCTTAATGTAAGTGCTGACTTCCTTAACAATTTCCTCATATCTCTTCTGGCTGTAGGGTGGCTCA
GTGGAATCCATTTTGTTAACACCGACAAT 43 (0.001293%)

CAATTTTGTAGACATCCTGGAGAGGCAGGCGCAAGGGCTTGTCAGTTGGACGAGTTGGTGGTAGGATGC
AGTCCAGAGCCTCAAGCAGCGTGGTTCCACT 475 (0.014279%)

CACACATGGGCTTGCCAGGAACCATATCAACAATGGCAGCATCACCAGACTTCAAGAATTTAGGGCCAT
CTTCCAGCTTTTTACCAGAACGGCGATCAAT 6 (0.000180%)

CACACCCAGTGTGTAAGCCAGAAGGGCATGCTCTCGGGTCTGCCCATTCTTGGAGATACCAGCTTCAAAT
TCACCAACACCAGCAGCAACAATCAGGACA 7 (0.000210%)

CACACGCAAAAAAGAAAACCAAAGTGGTCCACAAAACATTCTCCTTTCCTTCTGAAGGTTTTACGATGC
ATTGTTATCATTAACCAGTCTTTTACTACTA 48 (0.001443%)

CACACTCCTTTGCTACTGGTCCTGTAATGGCAGAACCTTTCATCTCGCCTTTATTGTTCACTATGACTCCT
GCATTATCTTCAAAATAAAGAAACACGCC 4 (0.000120%)

CACATCTACAAAATGCCAGTATCAGGCGGCGGCTTCGAAGCCAAAGTGATGTTTGGATGTAAAGTGAAA
TATTAGTTGGCGGATGAAGCAGATAGTGAGG 342 (0.010281%)

CACATGAGTATTTGTCTAAAACATGTCTTCTTTGTAGCAGCTAGGCCCTGCCACCACTGTGCTTGGCTGA
GTTCACAAATCTGTTGTAACCTGTAGCTTC 4 (0.000120%)

CACATGGGCTTGCCAGGAACCATATCAACAATGGCAGCATCACCAGACTTCAAGAATTTAGGGCCATCT
TCCAGCTTTTTACCAGAACGGCGATCAATCT 4 (0.000120%)

CACATTGTCCCCAGGAAGAGCTTCACTCAAAGCTTCATGGTGCATTTCGACAGATTTTACTTCCGTTGTA
ACGTTGACTGGAGCAAAGGTGACCACCATA 22 (0.000661%)

CACCACTGATTAAGAGTGGGGTGGCAGGTATTAGGGATAATATTCATTTAGCCTTCTGAGCTTTCTGGGC
AGACTTGGTGACCTTGCCAGCTCCAGCAGC 5 (0.000150%)

CACCAGTTTTAGCCAACATAGCATAGTACTCTATTTCAGATTTCCTCAAAGCTGGGCAGTTGTTAGCGAG
AATGACCAATTTCGCTTTGCCTTGTCTGAT6 (0.000180%)

CACCTACGGTGAAAAGAAAGATGAATCCTAGGGCTCAGAGCACTGCAGCAGATCATTTCATATTGCTTC
CGTGGAGTGTGGCGAGTCAGCTAAATACTTT 6 (0.000180%)

CACCTGAGCAGTGAAGCCAGCTGCTTCCATTGGTGGGTCATTTTTGCTGTCACCAGCAACGTTGCCACGA
CGAACATCCTTGACAGACACATTCTTGACA 6 (0.000180%)

CACGCAAAAAAGAAAACCAAAGTGGTCCACAAAACATTCTCCTTTCCTTCTGAAGGTTTTACGATGCATT

GTTATCATTAACCAGTCTTTACTACTAAA27 (0.000812%)

CACTCCTTTGCTACTGGTCCTGTAATGGCAGAACCTTTCATCTCGCCTTTATTGTTCACTATGACTCCTGC
ATTATCTTCAAAATAAAGAAACACGCCAT 5 (0.000150%)

CACTGATTAAGAGTGGGGTGGCAGGTATTAGGGATAATATTCATTTAGCCTTCTGAGCTTTCTGGGCAGA
CTTGGTGACCTTGCCAGCTCCAGCAGCCTT 7 (0.000210%)

CACTGCAGCAGATCATTTCATATTGCTTCCGTGGAGTGTGGCGAGTCAGCTAAATACTTTGACGCCGGTG
GGGATAGCGATGATTATGGTAGCGGAGGTG 3 (0.000090%)

CAGAACTACTGCCTTCACCATGAAGCTCCATGAGCTTTCCCAATTCAAACTTGGGCTTCTTCAGCATTTTT
ACTTTTCTAACGAAGACATCATGGAGAGG 4 (0.000120%)

CAGAAGGGCATGCTCTCGGGTCTGCCCATTCTTGGAGATACCAGCTTCAAATTCACCAACACCAGCAGC
AACAATCAGGACAGCACAGTCAGCCTGAGAT 38 (0.001142%)

CAGACTTGGTGACCTTGCCAGCTCCAGCAGCCTTCTTGTCCACTGCTTTGATGACACCCACCGCAACTGT
CTGTCTCATATCACGAACAGCAAAGCGACC 28 (0.000842%)

CAGAGCACTGCAGCAGATCATTTCATATTGCTTCCGTGGAGTGTGGCGAGTCAGCTAAATACTTTGACGC
CGGTGGGGATAGCGATGATTATGGTAGCGG 14 (0.000421%)

CAGATCATTTCATATTGCTTCCGTGGAGTGTGGCGAGTCAGCTAAATACTTTGACGCCGGTGGGGATAGC
GATGATTATGGTAGCGGAGGTGAAATATGC 4 (0.000120%)

CAGCAACAATCAGGACAGCACAGTCAGCCTGAGATGTCCCTGTAATCATGTTTTTGATAAAGTCTCTGTG
TCCTGGGGCATCAATGATAGTCACATAGTA 16 (0.000481%)

CAGCAGATCATTTCATATTGCTTCCGTGGAGTGTGGCGAGTCAGCTAAATACTTTGACGCCGGTGGGGAT
AGCGATGATTATGGTAGCGGAGGTGAAATA 17 (0.000511%)

CAGCGTGGTTCCACTGGCATTGCCATCCTTACGGGTGACTTTCCATCCCTTGAACCAAGGCATGTTAGCA
CTTGGCTCCAGCATGTTGTCACCATTCCAA 7 (0.000210%)

CAGCTCCAGCAGCCTTCTTGTCCACTGCTTTGATGACACCCACCGCAACTGTCTGTCTCATATCACGAAC
AGCAAAGCGACCCAAAGGTGGATAGTCTGA 7 (0.000210%)

CAGCTGCTTCCATTGGTGGGTCATTTTTGCTGTCACCAGCAACGTTGCCACGACGAACATCCTTGACAGA
CACATTCTTGACATTGAAGCCCACATTGTC 11 (0.000331%)

CAGGAAGAGCTTCACTCAAAGCTTCATGGTGCATTTCGACAGATTTTACTTCCGTTGTAACGTTGACTGG
AGCAAAGGTGACCACCATACCGGGTTTGAG 44 (0.001323%)

CAGGATAATCACCTGAGCAGTGAAGCCAGCTGCTTCCATTGGTGGGTCATTTTTGCTGTCACCAGCAACG
TTGCCACGACGAACATCCTTGACAGACACA 1112 (0.033428%)

CAGGCCACCTACGGTGAAAAGAAAGATGAATCCTAGGGCTCAGAGCACTGCAGCAGATCATTTCATATT
GCTTCCGTGGAGTGTGGCGAGTCAGCTAAAT 26 (0.000782%)

CAGGCGCAAGGGCTTGTCAGTTGGACGAGTTGGTGGTAGGATGCAGTCCAGAGCCTCAAGCAGCGTGGT
TCCACTGGCATTGCCATCCTTACGGGTGACT 25 (0.000752%)

CAGGCTTATGCGGAGGAGAATGTTTTCATGTTACTTATACTAACATTAGTTCTTCTATAGGGTGATAGAT
TGGTCCAATTGGGTGTGAGGAGTTCAGTTA 18 (0.000541%)

CAGGGCTATTGGTTGAATGAGTAGGCTGATGGTTTCGATAATAACTAGTATGGGGATAAGGGGTGTAGG
TGTGCCTTGTGGTAAGAAGTGGGCTAGGGCA 21 (0.000631%)

CAGGTACCTTTCTCTTTGGCTTCTTTCTTTTTCTGATCATTTTCCTTCACACGTTTCAGGAAGCTATCTCGG
CTCTTAGAGTGCTTAATGTGCTCAATAC 3 (0.000090%)

CAGGTATTAGGGATAATATTCATTTAGCCTTCTGAGCTTTCTGGGCAGACTTGGTGACCTTGCCAGCTCC
AGCAGCCTTCTTGTCCACTGCTTTGATGAC 119 (0.003577%)

CAGGTCCCGGTATTCCCGGTACATGTTGTGGGTGCCGCTCCGGGAGTCATAGCGCAGCCAGATCCCGAA
GTTCTTCACCCGCAGGGGGGACTTCTCAAAC 5 (0.000150%)

CAGTAATGTTAGCGGTTAGGCGTACGGCCAGGGCTATTGGTTGAATGAGTAGGCTGATGGTTTCGATAAT
AACTAGTATGGGGATAAGGGGTGTAGGTGT 35 (0.001052%)

CAGTCAGGCCACCTACGGTGAAAAGAAAGATGAATCCTAGGGCTCAGAGCACTGCAGCAGATCATTTCA
TATTGCTTCCGTGGAGTGTGGCGAGTCAGCT 3 (0.000090%)

CAGTGAAGCCAGCTGCTTCCATTGGTGGGTCATTTTTGCTGTCACCAGCAACGTTGCCACGACGAACATC
CTTGACAGACACATTCTTGACATTGAAGCC 12 (0.000361%)

CAGTGTGTAAGCCAGAAGGGCATGCTCTCGGGTCTGCCCATTCTTGGAGATACCAGCTTCAAATTCACCA
ACACCAGCAGCAACAATCAGGACAGCACAG 3 (0.000090%)

CAGTGTTTTAATCTGACGCAGGCTTATGCGGAGGAGAATGTTTTCATGTTACTTATACTAACATTAGTTCT
TCTATAGGGTGATAGATTGGTCCAATTGG 6 (0.000180%)

CATAAGTGGAGTCCGTAAAGAGGTATCTTTACTATAAAAGCTATTGTGTAAGCTAGTCATATTAAGTTGT
TGGCTCAGGAGTTTGATAGTTCTTGGGCAG 17 (0.000511%)

CATACACATGAGTATTTGTCTAAAACATGTCTTCTTTGTAGCAGCTAGGCCCTGCCACCACTGTGCTTGG
CTGAGTTCACAAATCTGTTGTAACCTGTAG 3 (0.000090%)

CATAGGGATAGTACAAGGAAGGGGTAGGCTATGTGTTTTGTCAGGGGGTTGAGAATGAGTGTGAGGCGT
ATTATACCATAGCCGCCTAGTTTTAAGAGTA 6 (0.000180%)

CATATCTCTTCTGGCTGTAGGGTGGCTCAGTGGAATCCATTTTGTTAACACCGACAATTAGTTGTTTCACA
CCCAGTGTGTAAGCCAGAAGGGCATGCTC23 (0.000691%)

CATCCTGGAGAGGCAGGCGCAAGGGCTTGTCAGTTGGACGAGTTGGTGGTAGGATGCAGTCCAGAGCCT
CAAGCAGCGTGGTTCCACTGGCATTGCCATC 4 (0.000120%)

CATGGCTAGGTTTATAGATAGTTGGGTGGTTGGTGTAAATGAGTGAGGCAGGAGTCCGAGGAGGTTAGT
TGTGGCAATAAAAATGATTAAGGATACTAGT 28 (0.000842%)

CATGGGCTTGCCAGGAACCATATCAACAATGGCAGCATCACCAGACTTCAAGAATTTAGGGCCATCTTC
CAGCTTTTTACCAGAACGGCGATCAATCTTT 8 (0.000240%)

CATGGTTTGGGTTGGGTGGTGGAGAGCGCGTGTCATCTGCGGGTGGCACTGCCCACGGTGGGCGGGCGG
GCCTCTCTACTCGAAGGTGACCACGTTTAGA 11 (0.000331%)

CATGTTTTTGATAAAGTCTCTGTGTCCTGGGGCATCAATGATAGTCACATAGTACTTGCTGGTCTCAAATT
TCCACAAGGAGATATCAATGGTGATACCA12 (0.000361%)

CATTAAAAAGTACTGATTTTAAAAACTAATAACTTAAAACTGCCACACGCAAAAAAGAAAACCAAAGTG
GTCCACAAAACATTCTCCTTTCCTTCTGAAG 3 (0.000090%)

CATTGCCATCCTTACGGGTGACTTTCCATCCCTTGAACCAAGGCATGTTAGCACTTGGCTCCAGCATGTT
GTCACCATTCCAACCAGAAATTGGCACAAA 7 (0.000210%)

CATTGGTGGGTCATTTTTGCTGTCACCAGCAACGTTGCCACGACGAACATCCTTGACAGACACATTCTTG
ACATTGAAGCCCACATTGTCCCCAGGAAGA 4 (0.000120%)

CATTGTCCCCAGGAAGAGCTTCACTCAAAGCTTCATGGTGCATTTCGACAGATTTTACTTCCGTTGTAAC
GTTGACTGGAGCAAAGGTGACCACCATACC 10 (0.000301%)

CATTTAGCCTTCTGAGCTTTCTGGGCAGACTTGGTGACCTTGCCAGCTCCAGCAGCCTTCTTGTCCACTGC
TTTGATGACACCCACCGCAACTGTCTGTC 14 (0.000421%)

CATTTTTGCTGTCACCAGCAACGTTGCCACGACGAACATCCTTGACAGACACATTCTTGACATTGAAGCC
CACATTGTCCCCAGGAAGAGCTTCACTCAA 49 (0.001473%)

CCAAAGTGGTCCACAAAACATTCTCCTTTCCTTCTGAAGGTTTTACGATGCATTGTTATCATTAACCAGTC
TTTTACTACTAAACTTAAATGGCCAATTG 8 (0.000240%)

CCAAGACCCAGGCATACTTGAAGGAGCCCTTTCCCATCTCAGCAGCCTCCTTCTCAAATTTTTCAATGGT
TCTTTTGTCGATGCCACCGCATTTATAGAT13 (0.000391%)

CCAATTTTCTTAATGTAAGTGCTGACTTCCTTAACAATTTCCTCATATCTCTTCTGGCTGTAGGGTGGCTC
AGTGGAATCCATTTTGTTAACACCGACAA 6 (0.000180%)

CCACACGCAAAAAAGAAAACCAAAGTGGTCCACAAAACATTCTCCTTTCCTTCTGAAGGTTTTACGATGC
ATTGTTATCATTAACCAGTCTTTTACTACT 42 (0.001263%)

CCACATTGTCCCCAGGAAGAGCTTCACTCAAAGCTTCATGGTGCATTTCGACAGATTTTACTTCCGTTGT
AACGTTGACTGGAGCAAAGGTGACCACCAT 533 (0.016023%)

CCACCACTGATTAAGAGTGGGGTGGCAGGTATTAGGGATAATATTCATTTAGCCTTCTGAGCTTTCTGGG
CAGACTTGGTGACCTTGCCAGCTCCAGCAG 10 (0.000301%)

CCACTGATTAAGAGTGGGGTGGCAGGTATTAGGGATAATATTCATTTAGCCTTCTGAGCTTTCTGGGCAG
ACTTGGTGACCTTGCCAGCTCCAGCAGCCT 3 (0.000090%)

CCAGAACTACTGCCTTCACCATGAAGCTCCATGAGCTTTCCCAATTCAAACTTGGGCTTCTTCAGCATTTT
TACTTTTCTAACGAAGACATCATGGAGAG 4 (0.000120%)

CCAGAAGGGCATGCTCTCGGGTCTGCCCATTCTTGGAGATACCAGCTTCAAATTCACCAACACCAGCAGC
AACAATCAGGACAGCACAGTCAGCCTGAGA 4 (0.000120%)

CCAGCTCCAGCAGCCTTCTTGTCCACTGCTTTGATGACACCCACCGCAACTGTCTGTCTCATATCACGAA
CAGCAAAGCGACCCAAAGGTGGATAGTCTG 7 (0.000210%)

CCAGCTGCTTCCATTGGTGGGTCATTTTTGCTGTCACCAGCAACGTTGCCACGACGAACATCCTTGACAG
ACACATTCTTGACATTGAAGCCCACATTGT 5 (0.000150%)

CCAGGAAGAGCTTCACTCAAAGCTTCATGGTGCATTTCGACAGATTTTACTTCCGTTGTAACGTTGACTG
GAGCAAAGGTGACCACCATACCGGGTTTGA 62 (0.001864%)

CCAGGGCTATTGGTTGAATGAGTAGGCTGATGGTTTCGATAATAACTAGTATGGGGATAAGGGGTGTAG
GTGTGCCTTGTGGTAAGAAGTGGGCTAGGGC 5 (0.000150%)

CCAGGTACCTTTCTCTTTGGCTTCTTTCTTTTTCTGATCATTTTCCTTCACACGTTTCAGGAAGCTATCTCG
GCTCTTAGAGTGCTTAATGTGCTCAATA 6 (0.000180%)

CCAGTCAGGCCACCTACGGTGAAAAGAAAGATGAATCCTAGGGCTCAGAGCACTGCAGCAGATCATTTC
ATATTGCTTCCGTGGAGTGTGGCGAGTCAGC 6 (0.000180%)

CCAGTGTGTAAGCCAGAAGGGCATGCTCTCGGGTCTGCCCATTCTTGGAGATACCAGCTTCAAATTCACC
AACACCAGCAGCAACAATCAGGACAGCACA 36 (0.001082%)

CCAGTGTTACTTTAATTGGACTGCCTTCGTAATTCATTGCCTCTGCTTCAACAATGTGCAACTCATCCTTT

GCACCAGCCCCTAAACTGACCGTTCTTAA 3 (0.000090%)

CCAGTTTTAGCCAACATAGCATAGTACTCTATTTCAGATTTCCTCAAAGCTGGGCAGTTGTTAGCGAGAA
TGACCAATTTCGCTTTGCCTTGTCTGATCA 7 (0.000210%)

CCATGGCTAGGTTTATAGATAGTTGGGTGGTTGGTGTAAATGAGTGAGGCAGGAGTCCGAGGAGGTTAG
TTGTGGCAATAAAAATGATTAAGGATACTAG 21 (0.000631%)

CCATTAAAAAGTACTGATTTTAAAAACTAATAACTTAAAACTGCCACACGCAAAAAAGAAAACCAAAGT
GGTCCACAAAACATTCTCCTTTCCTTCTGAA 14 (0.000421%)

CCATTGGTGGGTCATTTTTGCTGTCACCAGCAACGTTGCCACGACGAACATCCTTGACAGACACATTCTT
GACATTGAAGCCCACATTGTCCCCAGGAAG 6 (0.000180%)

CCCAGGAAGAGCTTCACTCAAAGCTTCATGGTGCATTTCGACAGATTTTACTTCCGTTGTAACGTTGACT
GGAGCAAAGGTGACCACCATACCGGGTTTG 97 (0.002916%)

CCCAGGTACCTTTCTCTTTGGCTTCTTTCTTTTTCTGATCATTTTCCTTCACACGTTTCAGGAAGCTATCTC
GGCTCTTAGAGTGCTTAATGTGCTCAAT 13 (0.000391%)

CCCAGTAAAATTGTAATAAGCAGTGCTTGAATTATTTGGTTTCGGTTGTTTTCTATTAGACTATGGTGAGC
TCAGGTGATTGATACTCCTGATGCGAGTA 11 (0.000331%)

CCCAGTGTGTAAGCCAGAAGGGCATGCTCTCGGGTCTGCCCATTCTTGGAGATACCAGCTTCAAATTCAC
CAACACCAGCAGCAACAATCAGGACAGCAC 11 (0.000331%)

CCCATACACATGAGTATTTGTCTAAAACATGTCTTCTTTGTAGCAGCTAGGCCCTGCCACCACTGTGCTTG
GCTGAGTTCACAAATCTGTTGTAACCTGT 10 (0.000301%)

CCCCAGGAAGAGCTTCACTCAAAGCTTCATGGTGCATTTCGACAGATTTTACTTCCGTTGTAACGTTGAC
TGGAGCAAAGGTGACCACCATACCGGGTTT 30 (0.000902%)

CCCCTGTTGCAAATTCTCATGGTTTGGGTTGGGTGGTGGAGAGCGCGTGTCATCTGCGGGTGGCACTGCC
CACGGTGGGCGGGCGGGCCTCTCTACTCGA 9 (0.000271%)

CCCCTTTCATTTTATAATAGAAAACCTTGGACTCGCCAGTGTTAGCTGCTGGAATGAGGTGTTTGTCCAG
TACATCCAGAATGTCACAACAGATTAACTT 4 (0.000120%)

CCCTAAGATAGAGGAGACACCTGCTAGGTGTAAGGAGAAGATGGTTAGGTCTACGGAGGCTCCAGGGTG
GGAGTAGTTCCCTGCTAAGGGAGGGTAGACT 4 (0.000120%)

CCCTGTTGCAAATTCTCATGGTTTGGGTTGGGTGGTGGAGAGCGCGTGTCATCTGCGGGTGGCACTGCCC
ACGGTGGGCGGGCGGGCCTCTCTACTCGAA 19 (0.000571%)

CCGAAGCCTGGTAGGATAAGAATATAAACTTCAGGGTGACCGAAAAATCAGAATAGGTGTTGGTATAGA
ATGGGGTCTCCTCCTCCGGCGGGGTCGAAGA 31 (0.000932%)

CCGCGGGGCTCCCCGCCGCCTCGATGCGGCGGCTTGGGGTGGCCGCCAGGCGTACTTAGGCCCGGGCCG
CCACCTGGCTTCGGCCCGCCAGGCATTTTGC 22 (0.000661%)

CCGGTATTCCCGGTACATGTTGTGGGTGCCGCTCCGGGAGTCATAGCGCAGCCAGATCCCGAAGTTCTTC
ACCCGCAGGGGGGACTTCTCAAACACCTGC 22 (0.000661%)

CCGTTCTTCCACCACTGATTAAGAGTGGGGTGGCAGGTATTAGGGATAATATTCATTTAGCCTTCTGAGC
TTTCTGGGCAGACTTGGTGACCTTGCCAGC 33 (0.000992%)

CCTACGGTGAAAAGAAAGATGAATCCTAGGGCTCAGAGCACTGCAGCAGATCATTTCATATTGCTTCCG
TGGAGTGTGGCGAGTCAGCTAAATACTTTGA 3 (0.000090%)

CCTAGGGCTCAGAGCACTGCAGCAGATCATTTCATATTGCTTCCGTGGAGTGTGGCGAGTCAGCTAAATA CTTTGACGCCGGTGGGGATAGCGATGATTA 10 (0.000301%)

CCTCAAGCAGCGTGGTTCCACTGGCATTGCCATCCTTACGGGTGACTTTCCATCCCTTGAACCAAGGCAT GTTAGCACTTGGCTCCAGCATGTTGTCACC 29 (0.000872%)

CCTCATATCTCTTCTGGCTGTAGGGTGGCTCAGTGGAATCCATTTTGTTAACACCGACAATTAGTTGTTTC ACACCCAGTGTGTAAGCCAGAAGGGCATG 41 (0.001233%)

CCTCCCCTGTTGCAAATTCTCATGGTTTGGGTTGGGTGGTGGAGAGCGCGTGTCATCTGCGGGTGGCACT GCCCACGGTGGGCGGGCGGGCCTCTCTACT 9 (0.000271%)

CCTCTCCTGCTAAGCTTTGTTTCCTAATTAAAATCTTCTGCCACTGCCATAGCTACTGCTGCTGCTGGAAC CGCCATAGCCACCTTGGTTTCGTGGTTTT 5 (0.000150%)

CCTGAGCAGTGAAGCCAGCTGCTTCCATTGGTGGGTCATTTTTGCTGTCACCAGCAACGTTGCCACGACG AACATCCTTGACAGACACATTCTTGACATT 7 (0.000210%)

CCTGCAGTAATGTTAGCGGTTAGGCGTACGGCCAGGGCTATTGGTTGAATGAGTAGGCTGATGGTTTCGA TAATAACTAGTATGGGGATAAGGGGTGTAG 8 (0.000240%)

CCTGCTAGGTGTAAGGAGAAGATGGTTAGGTCTACGGAGGCTCCAGGGTGGGAGTAGTTCCCTGCTAAG GGAGGGTAGACTGTTCAACCTGTTCCTGCTC 3 (0.000090%)

CCTGGAGAGGCAGGCGCAAGGGCTTGTCAGTTGGACGAGTTGGTGGTAGGATGCAGTCCAGAGCCTCAA GCAGCGTGGTTCCACTGGCATTGCCATCCTT 25 (0.000752%)

CCTGTGGCAAATTCTGCCAGATACCTGTGGTAGTCCCCTTTCATTTTATAATAGAAAACCTTGGACTCGC CAGTGTTAGCTGCTGGAATGAGGTGTTTGT 70 (0.002104%)

CCTGTGGTAGTCCCCTTTCATTTTATAATAGAAAACCTTGGACTCGCCAGTGTTAGCTGCTGGAATGAGG TGTTTGTCCAGTACATCCAGAATGTCACAA 5 (0.000150%)

CCTGTTGCAAATTCTCATGGTTTGGGTTGGGTGGTGGAGAGCGCGTGTCATCTGCGGGTGGCACTGCCCA CGGTGGGCGGGCGGGCCTCTCTACTCGAAG 12 (0.000361%)

CCTTAACAATTTCCTCATATCTCTTCTGGCTGTAGGGTGGCTCAGTGGAATCCATTTTGTTAACACCGACA ATTAGTTGTTTCACACCCAGTGTGTAAGC 19 (0.000571%)

CCTTGCCAGCTCCAGCAGCCTTCTTGTCCACTGCTTTGATGACACCCACCGCAACTGTCTGTCTCATATCA CGAACAGCAAAGCGACCCAAAGGTGGATA 7 (0.000210%)

CGAAGCCTGGTAGGATAAGAATATAAACTTCAGGGTGACCGAAAAATCAGAATAGGTGTTGGTATAGAA TGGGGTCTCCTCCTCCGGCGGGGTCGAAGAA 10 (0.000301%)

CGAAGTACTCTGAGGCTTGTAGGAGGGTAAAATAGAGACCCAGTAAAATTGTAATAAGCAGTGCTTGAA TTATTTGGTTTCGGTTGTTTTCTATTAGACT 10 (0.000301%)

CGAATGGGGGCTTCAATCGGGAGTACTACTCGATTGTCAACGTCAAGGAGTCGCAGGTCGCCTGGTTCT AGGAATAATGGGGGAAGTATGTAGGAGTTGA 19 (0.000571%)

CGACATGGGCTTTAGGGAGTCATAAGTGGAGTCCGTAAAGAGGTATCTTTACTATAAAAGCTATTGTGTA AGCTAGTCATATTAAGTTGTTGGCTCAGGA 8 (0.000240%)

CGACCCAAAGGTGGATAGTCTGAGAAGCTCTCAACACACATGGGCTTGCCAGGAACCATATCAACAATG GCAGCATCACCAGACTTCAAGAATTTAGGGC 803 (0.024139%)

CGAGTTGGTGGTAGGATGCAGTCCAGAGCCTCAAGCAGCGTGGTTCCACTGGCATTGCCATCCTTACGG GTGACTTTCCATCCCTTGAACCAAGGCATGT 17 (0.000511%)

CGCAAAAAAGAAAACCAAAGTGGTCCACAAAACATTCTCCTTTCCTTCTGAAGGTTTTACGATGCATTGT
TATCATTAACCAGTCTTTTACTACTAAACT22 (0.000661%)

CGCAAGGGCTTGTCAGTTGGACGAGTTGGTGGTAGGATGCAGTCCAGAGCCTCAAGCAGCGTGGTTCCA
CTGGCATTGCCATCCTTACGGGTGACTTTCC 54 (0.001623%)

CGCAGGCTTATGCGGAGGAGAATGTTTTCATGTTACTTATACTAACATTAGTTCTTCTATAGGGTGATAG
ATTGGTCCAATTGGGTGTGAGGAGTTCAGT 16 (0.000481%)

CGGAGGAAAAGGTTGGGGAACAGCTAAATAGGTTGTTGTTGATTTGGTTAAAAAATAGTAGAGGGATGA
TGCTAATAATTAGGCTGTGGGTGGTTGTGTT 13 (0.000391%)

CGGATGCTACTTGTCCAATGATGGTAAAAGGGTAGCTTACTGGTTGTCCTCCGATTCAGGTTAGAATGAG
GAGGTCTGCGGCTAGGAGTCAATAAAGTGA 360 (0.010822%)

CGGCCAGGGCTATTGGTTGAATGAGTAGGCTGATGGTTTCGATAATAACTAGTATGGGGATAAGGGGTG
TAGGTGTGCCTTGTGGTAAGAAGTGGGCTAG 3 (0.000090%)

CGGGAGTACTACTCGATTGTCAACGTCAAGGAGTCGCAGGTCGCCTGGTTCTAGGAATAATGGGGGAAG
TATGTAGGAGTTGAAGATTAGTCCGCCGTAG 49 (0.001473%)

CGGGTTTTAGGGGCTCTTTGGTGAAGAGTTTTATGGCGTCAGCGAAGGGTTGTAGTAGCCCGTAGGGGCC
TACAACGTTGGGGCCTTTGCGTAGTTGTAT 4 (0.000120%)

CGGTCTGTTAGTAGTATAGTGATGCCAGCAGCTAGGACTGGGAGAGATAGGAGAAGTAGGACTGCTGTG
ATTAGGACGGATCAGACGAAGAGGGGCGTTT 7 (0.000210%)

CGGTGGTCAGGTCCCGGTATTCCCGGTACATGTTGTGGGTGCCGCTCCGGGAGTCATAGCGCAGCCAGAT
CCCGAAGTTCTTCACCCGCAGGGGGGACTT 12 (0.000361%)

CGTACGGCCAGGGCTATTGGTTGAATGAGTAGGCTGATGGTTTCGATAATAACTAGTATGGGGATAAGG
GGTGTAGGTGTGCCTTGTGGTAAGAAGTGGG 13 (0.000391%)

CGTTCTTCCACCACTGATTAAGAGTGGGGTGGCAGGTATTAGGGATAATATTCATTTAGCCTTCTGAGCT
TTCTGGGCAGACTTGGTGACCTTGCCAGCT 93 (0.002796%)

CTAAGATAGAGGAGACACCTGCTAGGTGTAAGGAGAAGATGGTTAGGTCTACGGAGGCTCCAGGGTGG
GAGTAGTTCCCTGCTAAGGGAGGGTAGACTGT17 (0.000511%)

CTAATAACTTAAAACTGCCACACGCAAAAAAGAAAACCAAAGTGGTCCACAAAACATTCTCCTTTCCTT
CTGAAGGTTTTACGATGCATTGTTATCATTA 8 (0.000240%)

CTAATACAATGCCAGTCAGGCCACCTACGGTGAAAAGAAAGATGAATCCTAGGGCTCAGAGCACTGCAG
CAGATCATTTCATATTGCTTCCGTGGAGTGT 7 (0.000210%)

CTACGGTGAAAAGAAAGATGAATCCTAGGGCTCAGAGCACTGCAGCAGATCATTTCATATTGCTTCCGT
GGAGTGTGGCGAGTCAGCTAAATACTTTGAC 10 (0.000301%)

CTAGGGCTCAGAGCACTGCAGCAGATCATTTCATATTGCTTCCGTGGAGTGTGGCGAGTCAGCTAAATAC
TTTGACGCCGGTGGGGATAGCGATGATTAT 15 (0.000451%)

CTAGGGTGACTTCATATGAGATTGTTTGGGCTACTGCTCGCAGTGCGCCGATCAGGGCGTAGTTTGAGTT
TGATGCTCACCCTGATCAGAGGATTGAGTA 5 (0.000150%)

CTAGGGTGGCGCTTCCAATTAGGTGCATGAGTAGGTGGCCTGCAGTAATGTTAGCGGTTAGGCGTACGG
CCAGGGCTATTGGTTGAATGAGTAGGCTGAT 17 (0.000511%)

CTAGGTTTATAGATAGTTGGGTGGTTGGTGTAAATGAGTGAGGCAGGAGTCCGAGGAGGTTAGTTGTGG

CAATAAAAATGATTAAGGATACTAGTATAAG 49 (0.001473%)

CTATTAGACTATGGTGAGCTCAGGTGATTGATACTCCTGATGCGAGTAATACGGATGTGTTTAGGAGTGG
GACTTCTAGGGGATTTAGCGGGGTGATGCC 10 (0.000301%)

CTCAAAGCTTCATGGTGCATTTCGACAGATTTTACTTCCGTTGTAACGTTGACTGGAGCAAAGGTGACCA
CCATACCGGGTTTGAGAACACCAGTCTCCA 8 (0.000240%)

CTCAACACACATGGGCTTGCCAGGAACCATATCAACAATGGCAGCATCACCAGACTTCAAGAATTTAGG
GCCATCTTCCAGCTTTTTACCAGAACGGCGA 17 (0.000511%)

CTCAAGCAGCGTGGTTCCACTGGCATTGCCATCCTTACGGGTGACTTTCCATCCCTTGAACCAAGGCATG
TTAGCACTTGGCTCCAGCATGTTGTCACCA 35 (0.001052%)

CTCAGAGCACTGCAGCAGATCATTTCATATTGCTTCCGTGGAGTGTGGCGAGTCAGCTAAATACTTTGAC
GCCGGTGGGGATAGCGATGATTATGGTAGC 48 (0.001443%)

CTCAGTGGAATCCATTTTGTTAACACCGACAATTAGTTGTTTCACACCCAGTGTGTAAGCCAGAAGGGCA
TGCTCTCGGGTCTGCCCATTCTTGGAGATA 33 (0.000992%)

CTCATAGGGATAGTACAAGGAAGGGGTAGGCTATGTGTTTTGTCAGGGGGTTGAGAATGAGTGTGAGGC
GTATTATACCATAGCCGCCTAGTTTTAAGAG 234 (0.007034%)

CTCATATCTCTTCTGGCTGTAGGGTGGCTCAGTGGAATCCATTTTGTTAACACCGACAATTAGTTGTTTCA
CACCCAGTGTGTAAGCCAGAAGGGCATGC 83 (0.002495%)

CTCATGGTTTGGGTTGGGTGGTGGAGAGCGCGTGTCATCTGCGGGTGGCACTGCCCACGGTGGGCGGGC
GGGCCTCTCTACTCGAAGGTGACCACGTTTA 21 (0.000631%)

CTCCCCTGTTGCAAATTCTCATGGTTTGGGTTGGGTGGTGGAGAGCGCGTGTCATCTGCGGGTGGCACTG
CCCACGGTGGGCGGGCGGGCCTCTCTACTC 52 (0.001563%)

CTCCTTTGCTACTGGTCCTGTAATGGCAGAACCTTTCATCTCGCCTTTATTGTTCACTATGACTCCTGCATT
ATCTTCAAAATAAAGAAACACGCCATCT 22 (0.000661%)

CTCGAAGTACTCTGAGGCTTGTAGGAGGGTAAAATAGAGACCCAGTAAAATTGTAATAAGCAGTGCTTG
AATTATTTGGTTTCGGTTGTTTCTATTAGA 595 (0.017887%)

CTCGGGTCTGCCCATTCTTGGAGATACCAGCTTCAAATTCACCAACACCAGCAGCAACAATCAGGACAG
CACAGTCAGCCTGAGATGTCCCTGTAATCATGTTTTTGATAAAGTCTCTGTGTCCTGGGGCATCAATGAT
AGTCACATAG 373 (0.016707%)

CTCTCAACACACATGGGCTTGCCAGGAACCATATCAACAATGGCAGCATCACCAGACTTCAAGAATTTA
GGGCCATCTTCCAGCTTTTTACCAGAACGGC 10 (0.000301%)

CTCTCGGGTCTGCCCATTCTTGGAGATACCAGCTTCAAATTCACCAACACCAGCAGCAACAATCAGGACA
GCACAGTCAGCCTGAGATGTCCCTGTAATC 27 (0.000812%)

CTCTTCTGGCTGTAGGGTGGCTCAGTGGAATCCATTTTGTTAACACCGACAATTAGTTGTTTCACACCCA
GTGTGTAAGCCAGAAGGGCATGCTCTCGGG 36 (0.001082%)

CTGACGCAGGCTTATGCGGAGGAGAATGTTTTCATGTTACTTATACTAACATTAGTTCTTCTATAGGGTG
ATAGATTGGTCCAATTGGGTGTGAGGAGTT 3 (0.000090%)

CTGACTTCCTTAACAATTTCCTCATATCTCTTCTGGCTGTAGGGTGGCTCAGTGGAATCCATTTTGTTAAC
ACCGACAATTAGTTGTTTCACACCCAGTG 49 (0.001473%)

CTGAGAAGCTCTCAACACACATGGGCTTGCCAGGAACCATATCAACAATGGCAGCATCACCAGACTTCA
AGAATTTAGGGCCATCTTCCAGCTTTTTACC 336 (0.010101%)

CTGAGCAGTGAAGCCAGCTGCTTCCATTGGTGGGTCATTTTGCTGTCACCAGCAACGTTGCCACGACGA
ACATCCTTGACAGACACATTCTTGACATTG 6 (0.000180%)

CTGAGCTTTCTGGGCAGACTTGGTGACCTTGCCAGCTCCAGCAGCCTTCTTGTCCACTGCTTTGATGACAC
CCACCGCAACTGTCTGTCTCATATCACGA 24 (0.000721%)

CTGATTAAGAGTGGGGTGGCAGGTATTAGGGATAATATTCATTTAGCCTTCTGAGCTTTCTGGGCAGACT
TGGTGACCTTGCCAGCTCCAGCAGCCTTCT 8 (0.000240%)

CTGATTTTAAAAACTAATAACTTAAAACTGCCACACGCAAAAAAGAAAACCAAAGTGGTCCACAAAACA
TTCTCCTTTCCTTCTGAAGGTTTTACGATGC 90 (0.002706%)

CTGCACACTCCTTTGCTACTGGTCCTGTAATGGCAGAACCTTTCATCTCGCCTTTATTGTTCACTATGACT
CCTGCATTATCTTCAAAATAAAGAAACAC 244 (0.007335%)

CTGCAGCAGATCATTTCATATTGCTTCCGTGGAGTGTGGCGAGTCAGCTAAATACTTTGACGCCGGTGGG
GATAGCGATGATTATGGTAGCGGAGGTGAA 12 (0.000361%)

CTGCAGTAATGTTAGCGGTTAGGCGTACGGCCAGGGCTATTGGTTGAATGAGTAGGCTGATGGTTTCGAT
AATAACTAGTATGGGGATAAGGGGTGTAGG 43 (0.001293%)

CTGCCACACGCAAAAAAGAAAACCAAAGTGGTCCACAAAACATTCTCCTTTCCTTCTGAAGGTTTTACGA
TGCATTGTTATCATTAACCAGTCTTTTACT 32 (0.000962%)

CTGCTTCCATTGGTGGGTCATTTTGCTGTCACCAGCAACGTTGCCACGACGAACATCCTTGACAGACAC
ATTCTTGACATTGAAGCCCACATTGTCCCC 8 (0.000240%)

CTGGAGAGGCAGGCGCAAGGGCTTGTCAGTTGGACGAGTTGGTGGTAGGATGCAGTCCAGAGCCTCAAG
CAGCGTGGTTCCACTGGCATTGCCATCCTTA 25 (0.000752%)

CTGGAGTGGTAAAAGGCTCAGAAAAATCCTGCGAAGAAAAAAACTTCTGAGGTAATAAATAGGATTATC
CCGTATCGAAGGCCTTTTTGGACAGGTGGTG 36 (0.001082%)

CTGGCTGTAGGGTGGCTCAGTGGAATCCATTTTGTTAACACCGACAATTAGTTGTTTCACACCCAGTGTG
TAAGCCAGAAGGGCATGCTCTCGGGTCTGC 31 (0.000932%)

CTGGGCAGACTTGGTGACCTTGCCAGCTCCAGCAGCCTTCTTGTCCACTGCTTTGATGACACCCACCGCA
ACTGTCTGTCTCATATCACGAACAGCAAAG 26 (0.000782%)

CTGTAGGGTGGCTCAGTGGAATCCATTTTGTTAACACCGACAATTAGTTGTTTCACACCCAGTGTGTAAG
CCAGAAGGGCATGCTCTCGGGTCTGCCCAT 14 (0.000421%)

CTGTAGTGATGGACACCAGTTTTAGCCAACATAGCATAGTACTCTATTTCAGATTTCCTCAAAGCTGGGC
AGTTGTTAGCGAGAATGACCAATTTCGCTT 350 (0.010521%)

CTGTGACAAATTTTTGGTCAAGTTGTTTCCATTAAAAAGTACTGATTTTAAAAACTAATAACTTAAAACT
GCCACACGCAAAAAAGAAAACCAAAGTGGT 26 (0.000782%)

CTGTGGTAGTCCCCTTTCATTTTATAATAGAAAACCTTGGACTCGCCAGTGTTAGCTGCTGGAATGAGGT
GTTTGTCCAGTACATCCAGAATGTCACAAC 5 (0.000150%)

CTGTTAGTAGTATAGTGATGCCAGCAGCTAGGACTGGGAGAGATAGGAGAAGTAGGACTGCTGTGATTA
GGACGGATCAGACGAAGAGGGGCGTTTGGTA 5 (0.000150%)

CTGTTTTTAAGCCTAATGTGGGGACAGCTCATGAGTGCAAGACGTCTTGTGATGTAATTATTATACGAAT
GGGGGCTTCAATCGGGAGTACTACTCGATT 24 (0.000721%)

CTTAAAACTGCCACACGCAAAAAAGAAAACCAAAGTGGTCCACAAAACATTCTCCTTTCCTTCTGAAGG

TTTTACGATGCATTGTTATCATTAACCAGTC 7 (0.000210%)

CTTAACAATTTCCTCATATCTCTTCTGGCTGTAGGGTGGCTCAGTGGAATCCATTTTGTTAACACCGACAA
TTAGTTGTTTCACACCCAGTGTGTAAGCC 15 (0.000451%)

CTTAATGTAAGTGCTGACTTCCTTAACAATTTCCTCATATCTCTTCTGGCTGTAGGGTGGCTCAGTGGAAT
CCATTTTGTTAACACCGACAATTAGTTGT 12 (0.000361%)

CTTATGCGGAGGAGAATGTTTTCATGTTACTTATACTAACATTAGTTCTTCTATAGGGTGATAGATTGGTC
CAATTGGGTGTGAGGAGTTCAGTTATATG 8 (0.000240%)

CTTCAATCGGGAGTACTACTCGATTGTCAACGTCAAGGAGTCGCAGGTCGCCTGGTTCTAGGAATAATGG
GGGAAGTATGTAGGAGTTGAAGATTAGTCC 77 (0.002315%)

CTTCACTCAAAGCTTCATGGTGCATTTCGACAGATTTTACTTCCGTTGTAACGTTGACTGGAGCAAAGGT
GACCACCATACCGGGTTTGAGAACACCAGT 46 (0.001383%)

CTTCATATGAGATTGTTTGGGCTACTGCTCGCAGTGCGCCGATCAGGGCGTAGTTTGAGTTTGATGCTCA
CCCTGATCAGAGGATTGAGTAAACGGCTAG 15 (0.000451%)

CTTCCACCACTGATTAAGAGTGGGGTGGCAGGTATTAGGGATAATATTCATTTAGCCTTCTGAGCTTTCT
GGGCAGACTTGGTGACCTTGCCAGCTCCAG 23 (0.000691%)

CTTCCATTGGTGGGTCATTTTTGCTGTCACCAGCAACGTTGCCACGACGAACATCCTTGACAGACACATT
CTTGACATTGAAGCCCACATTGTCCCCAGG 36 (0.001082%)

CTTCCTTAACAATTTCCTCATATCTCTTCTGGCTGTAGGGTGGCTCAGTGGAATCCATTTTGTTAACACCG
ACAATTAGTTGTTTCACACCCAGTGTGTA 56 (0.001683%)

CTTCTGAGCTTTCTGGGCAGACTTGGTGACCTTGCCAGCTCCAGCAGCCTTCTTGTCCACTGCTTTGATGA
CACCCACCGCAACTGTCTGTCTCATATCA 13 (0.000391%)

CTTCTGGCTGTAGGGTGGCTCAGTGGAATCCATTTTGTTAACACCGACAATTAGTTGTTTCACACCCAGT
GTGTAAGCCAGAAGGGCATGCTCTCGGGTC 10 (0.000301%)

CTTGCCAGCTCCAGCAGCCTTCTTGTCCACTGCTTTGATGACACCCACCGCAACTGTCTGTCTCATATCAC
GAACAGCAAAGCGACCCAAAGGTGGATAG 33 (0.000992%)

CTTGGTGACCTTGCCAGCTCCAGCAGCCTTCTTGTCCACTGCTTTGATGACACCCACCGCAACTGTCTGTC
TCATATCACGAACAGCAAAGCGACCCAAA 14 (0.000421%)

CTTGTCAGTTGGACGAGTTGGTGGTAGGATGCAGTCCAGAGCCTCAAGCAGCGTGGTTCCACTGGCATTG
CCATCCTTACGGGTGACTTTCCATCCCTTG65 (0.001954%)

CTTGTCCAATGATGGTAAAAGGGTAGCTTACTGGTTGTCCTCCGATTCAGGTTAGAATGAGGAGGTCTGC
GGCTAGGAGTCAATAAAGTGATTGGCTTAG 4 (0.000120%)

CTTTAATTGGACTGCCTTCGTAATTCATTGCCTCTGCTTCAACAATGTGCAACTCATCCTTTGCACCAGCC
CCTAAACTGACCGTTCTTAAAGATAACTG 17 (0.000511%)

CTTTACTATAAAAGCTATTGTGTAAGCTAGTCATATTAAGTTGTTGGCTCAGGAGTTTGATAGTTCTTGGG
CAGTGAGAGTGAGTAGTAGAATGTTTAGT15 (0.000451%)

CTTTCTGGGCAGACTTGGTGACCTTGCCAGCTCCAGCAGCCTTCTTGTCCACTGCTTTGATGACACCCACC
GCAACTGTCTGTCTCATATCACGAACAGC 3 (0.000090%)

CTTTGATGACACCCACCGCAACTGTCTGTCTCATATCACGAACAGCAAAGCGACCCAAAGGTGGATAGT
CTGAGAAGCTCTCAACACACATGGGCTTGCC 315 (0.009469%)

CTTTGCTACTGGTCCTGTAATGGCAGAACCTTTCATCTCGCCTTTATTGTTCACTATGACTCCTGCATTAT
CTTCAAAATAAAGAAACACGCCATCTTTT 17 (0.000511%)

GAAAACCAAAGTGGTCCACAAAACATTCTCCTTTCCTTCTGAAGGTTTTACGATGCATTGTTATCATTAA
CCAGTCTTTTACTACTAAACTTAAATGGCC 9 (0.000271%)

GAAAAGAAAGATGAATCCTAGGGCTCAGAGCACTGCAGCAGATCATTTCATATTGCTTCCGTGGAGTGT
GGCGAGTCAGCTAAATACTTTGACGCCGGTG 10 (0.000301%)

GAAAAGGTTGGGGAACAGCTAAATAGGTTGTTGTTGATTTGGTTAAAAAATAGTAGAGGGATGATGCTA
ATAATTAGGCTGTGGGTGGTTGTGTTGATTC 6 (0.000180%)

GAAAGATGAATCCTAGGGCTCAGAGCACTGCAGCAGATCATTTCATATTGCTTCCGTGGAGTGTGGCGA
GTCAGCTAAATACTTTGACGCCGGTGGGGAT 8 (0.000240%)

GAAAGTTAGATTTACGCCGATGAATATGATAGTGAAATGGATTTTGGCGTAGGTTTGGTCTAGGGTGTAG
CCTGAGAATAGGGGAAATCAGTGAATGAAG 14 (0.000421%)

GAAATTGATGGCCCCTAAGATAGAGGAGACACCTGCTAGGTGTAAGGAGAAGATGGTTAGGTCTACGGA
GGCTCCAGGGTGGGAGTAGTTCCCTGCTAAG 14 (0.000421%)

GAAGAAAGTTAGATTTACGCCGATGAATATGATAGTGAAATGGATTTTGGCGTAGGTTTGGTCTAGGGT
GTAGCCTGAGAATAGGGGAAATCAGTGAATG 22 (0.000661%)

GAAGAGCTTCACTCAAAGCTTCATGGTGCATTTCGACAGATTTTACTTCCGTTGTAACGTTGACTGGAGC
AAAGGTGACCACCATACCGGGTTTGAGAAC 30 (0.000902%)

GAAGCCAGCTGCTTCCATTGGTGGGTCATTTTTGCTGTCACCAGCAACGTTGCCACGACGAACATCCTTG
ACAGACACATTCTTGACATTGAAGCCCACA 7 (0.000210%)

GAAGCCTGGTAGGATAAGAATATAAACTTCAGGGTGACCGAAAAATCAGAATAGGTGTTGGTATAGAAT
GGGGTCTCCTCCTCCGGCGGGGTCGAAGAAG 13 (0.000391%)

GAAGCTCTCAACACACATGGGCTTGCCAGGAACCATATCAACAATGGCAGCATCACCAGACTTCAAGAA
TTTAGGGCCATCTTCCAGCTTTTTACCAGAA 11 (0.000331%)

GAAGGGCATGCTCTCGGGTCTGCCCATTCTTGGAGATACCAGCTTCAAATTCACCAACACCAGCAGCAA
CAATCAGGACAGCACAGTCAGCCTGAGATGT 11 (0.000331%)

GAAGGGGTAGGCTATGTGTTTTGTCAGGGGGTTGAGAATGAGTGTGAGGCGTATTATACCATAGCCGCC
TAGTTTTAAGAGTACTGCGGCAAGTACTATT 7 (0.000210%)

GAAGTACTCTGAGGCTTGTAGGAGGGTAAAATAGAGACCCAGTAAAATTGTAATAAGCAGTGCTTGAAT
TATTTGGTTTCGGTTGTTTTCTATTAGACTA 3 (0.000090%)

GAATCCTAGGGCTCAGAGCACTGCAGCAGATCATTTCATATTGCTTCCGTGGAGTGTGGCGAGTCAGCTA
AATACTTTGACGCCGGTGGGGATAGCGATG 8 (0.000240%)

GAATGAGTAGGCTGATGGTTTCGATAATAACTAGTATGGGGATAAGGGGTGTAGGTGTGCCTTGTGGTA
AGAAGTGGGCTAGGGCATTTTTAATCTTAGA 17 (0.000511%)

GAATGATGGCTAGGGTGACTTCATATGAGATTGTTTGGGCTACTGCTCGCAGTGCGCCGATCAGGGCGTA
GTTTGAGTTTGATGCTCACCCTGATCAGAG 4 (0.000120%)

GAATTATTTGGTTTCGGTTGTTTTCTATTAGACTATGGTGAGCTCAGGTGATTGATACTCCTGATGCGAGT
AATACGGATGTGTTTAGGAGTGGGACTTC 175 (0.005261%)

GACACCAGTTTTAGCCAACATAGCATAGTACTCTATTTCAGATTTCCTCAAAGCTGGGCAGTTGTTAGCG
AGAATGACCAATTTCGCTTTGCCTTGTCTG 6 (0.000180%)

GACACCTGCTAGGTGTAAGGAGAAGATGGTTAGGTCTACGGAGGCTCCAGGGTGGGAGTAGTTCCCTGC
TAAGGGAGGGTAGACTGTTCAACCTGTTCCT 3 (0.000090%)

GACATCCTGGAGAGGCAGGCGCAAGGGCTTGTCAGTTGGACGAGTTGGTGGTAGGATGCAGTCCAGAGC
CTCAAGCAGCGTGGTTCCACTGGCATTGCCA 10 (0.000301%)

GACCGTTCTTCCACCACTGATTAAGAGTGGGGTGGCAGGTATTAGGGATAATATTCATTTAGCCTTCTGA
GCTTTCTGGGCAGACTTGGTGACCTTGCCA 27 (0.000812%)

GACCTTGCCAGCTCCAGCAGCCTTCTTGTCCACTGCTTTGATGACACCCACCGCAACTGTCTGTCTCATAT
CACGAACAGCAAAGCGACCCAAAGGTGGA 7 (0.000210%)

GACGAGTTGGTGGTAGGATGCAGTCCAGAGCCTCAAGCAGCGTGGTTCCACTGGCATTGCCATCCTTAC
GGGTGACTTTCCATCCCTTGAACCAAGGCAT 29 (0.000872%)

GACTTCCTTAACAATTTCCTCATATCTCTTCTGGCTGTAGGGTGGCTCAGTGGAATCCATTTTGTTAACAC
CGACAATTAGTTGTTTCACACCCAGTGTG 13 (0.000391%)

GACTTGGTGACCTTGCCAGCTCCAGCAGCCTTCTTGTCCACTGCTTTGATGACACCCACCGCAACTGTCT
GTCTCATATCACGAACAGCAAAGCGACCCA 10 (0.000301%)

GAGAAGATGGTTAGGTCTACGGAGGCTCCAGGGTGGGAGTAGTTCCCTGCTAAGGGAGGGTAGACTGTT
CAACCTGTTCCTGCTCCGGCCTCCACTATAG 9 (0.000271%)

GAGAAGCTCTCAACACACATGGGCTTGCCAGGAACCATATCAACAATGGCAGCATCACCAGACTTCAAG
AATTTAGGGCCATCTTCCAGCTTTTTACCAG 5 (0.000150%)

GAGACACCTGCTAGGTGTAAGGAGAAGATGGTTAGGTCTACGGAGGCTCCAGGGTGGGAGTAGTTCCCT
GCTAAGGGAGGGTAGACTGTTCAACCTGTTC 6 (0.000180%)

GAGACCCAGTAAAATTGTAATAAGCAGTGCTTGAATTATTTGGTTTCGGTTGTTTTCTATTAGACTATGGT
GAGCTCAGGTGATTGATACTCCTGATGCG 4 (0.000120%)

GAGAGGCAGGCGCAAGGGCTTGTCAGTTGGACGAGTTGGTGGTAGGATGCAGTCCAGAGCCTCAAGCA
GCGTGGTTCCACTGGCATTGCCATCCTTACGG 19 (0.000571%)

GAGCACTGCAGCAGATCATTTCATATTGCTTCCGTGGAGTGTGGCGAGTCAGCTAAATACTTTGACGCCG
GTGGGGATAGCGATGATTATGGTAGCGGAG 20 (0.000601%)

GAGCAGTGAAGCCAGCTGCTTCCATTGGTGGGTCATTTTTGCTGTCACCAGCAACGTTGCCACGACGAAC
ATCCTTGACAGACACATTCTTGACATTGAA 9 (0.000271%)

GAGCTTCACTCAAAGCTTCATGGTGCATTTCGACAGATTTTACTTCCGTTGTAACGTTGACTGGAGCAAA
GGTGACCACCATACCGGGTTTGAGAACACC 5 (0.000150%)

GAGCTTTCTGGGCAGACTTGGTGACCTTGCCAGCTCCAGCAGCCTTCTTGTCCACTGCTTTGATGACACC
CACCGCAACTGTCTGTCTCATATCACGAAC 15 (0.000451%)

GAGGAAAAGGTTGGGGAACAGCTAAATAGGTTGTTGTTGATTTGGTTAAAAAATAGTAGAGGGATGATG
CTAATAATTAGGCTGTGGGTGGTTGTGTTGA 3 (0.000090%)

GAGGAGACACCTGCTAGGTGTAAGGAGAAGATGGTTAGGTCTACGGAGGCTCCAGGGTGGGAGTAGTTC
CCTGCTAAGGGAGGGTAGACTGTTCAACCTG 3 (0.000090%)

GAGGCAGGCGCAAGGGCTTGTCAGTTGGACGAGTTGGTGGTAGGATGCAGTCCAGAGCCTCAAGCAGCG
TGGTTCCACTGGCATTGCCATCCTTACGGGT 13 (0.000391%)

GAGGGTAAAATAGAGACCCAGTAAAATTGTAATAAGCAGTGCTTGAATTATTTGGTTTCGGTTGTTTTCT

ATTAGACTATGGTGAGCTCAGGTGATTGAT 3 (0.000090%)

GAGGTGATCGGCGATCAGAGGGCGATGAAGTTCTAGATCCATTGAGACAAGCTCTAGACAGTAGCATGC
AGTCCCACAACTTGTACCAGCATCCCCAGCG 4 (0.000120%)

GAGTATTTGTCTAAAACATGTCTTCTTTGTAGCAGCTAGGCCCTGCCACCACTGTGCTTGGCTGAGTTCAC
AAATCTGTTGTAACCTGTAGCTTCCCTGT 5 (0.000150%)

GAGTCATAAGTGGAGTCCGTAAAGAGGTATCTTTACTATAAAAGCTATTGTGTAAGCTAGTCATATTAAG
TTGTTGGCTCAGGAGTTTGATAGTTCTTGG 3 (0.000090%)

GAGTGAGGCAGGAGTCCGAGGAGGTTAGTTGTGGCAATAAAAATGATTAAGGATACTAGTATAAGAGA
TCAGGTTCGTCCTTTAGTGTTGTGTATGGTTA 7 (0.000210%)

GAGTTGGTGGTAGGATGCAGTCCAGAGCCTCAAGCAGCGTGGTTCCACTGGCATTGCCATCCTTACGGGT
GACTTTCCATCCCTTGAACCAAGGCATGTT 3 (0.000090%)

GATAATATTCATTTAGCCTTCTGAGCTTTCTGGGCAGACTTGGTGACCTTGCCAGCTCCAGCAGCCTTCTT
GTCCACTGCTTTGATGACACCCACCGCAA 18 (0.000541%)

GATAGTACAAGGAAGGGGTAGGCTATGTGTTTTGTCAGGGGGTTGAGAATGAGTGTGAGGCGTATTATA
CCATAGCCGCCTAGTTTTAAGAGTACTGCGG 4 (0.000120%)

GATAGTTGGGTGGTTGGTGTAAATGAGTGAGGCAGGAGTCCGAGGAGGTTAGTTGTGGCAATAAAAATG
ATTAAGGATACTAGTATAAGAGATCAGGTTC 4 (0.000120%)

GATATTGCTAGGGTGGCGCTTCCAATTAGGTGCATGAGTAGGTGGCCTGCAGTAATGTTAGCGGTTAGGC
GTACGGCCAGGGCTATTGGTTGAATGAGTA 11 (0.000331%)

GATGAATCCTAGGGCTCAGAGCACTGCAGCAGATCATTTCATATTGCTTCCGTGGAGTGTGGCGAGTCAG
CTAAATACTTTGACGCCGGTGGGGATAGCG 17 (0.000511%)

GATGCAGTCCAGAGCCTCAAGCAGCGTGGTTCCACTGGCATTGCCATCCTTACGGGTGACTTTCCATCCC
TTGAACCAAGGCATGTTAGCACTTGGCTCC 7 (0.000210%)

GATGCTACTTGTCCAATGATGGTAAAAGGGTAGCTTACTGGTTGTCCTCCGATTCAGGTTAGAATGAGGA
GGTCTGCGGCTAGGAGTCAATAAAGTGATT 7 (0.000210%)

GATGGACACCAGTTTTAGCCAACATAGCATAGTACTCTATTTCAGATTTCCTCAAAGCTGGGCAGTTGTT
AGCGAGAATGACCAATTTCGCTTTGCCTTG 6 (0.000180%)

GATGGCTAGGGTGACTTCATATGAGATTGTTTGGGCTACTGCTCGCAGTGCGCCGATCAGGGCGTAGTTT
GAGTTTGATGCTCACCCTGATCAGAGGATT 7 (0.000210%)

GATGTGGCGGGTTTTAGGGGCTCTTTGGTGAAGAGTTTTATGGCGTCAGCGAAGGGTTGTAGTAGCCCGT
AGGGGCCTACAACGTTGGGGCCTTTGCGTA 3 (0.000090%)

GATTAAGAGTGGGGTGGCAGGTATTAGGGATAATATTCATTTAGCCTTCTGAGCTTTCTGGGCAGACTTG
GTGACCTTGCCAGCTCCAGCAGCCTTCTTG 10 (0.000301%)

GATTTACGCCGATGAATATGATAGTGAAATGGATTTTGGCGTAGGTTTGGTCTAGGGTGTAGCCTGAGAA
TAGGGGAAATCAGTGAATGAAGCCTCCTAT 7 (0.000210%)

GATTTTAAAAACTAATAACTTAAAACTGCCACACGCAAAAAAGAAAACCAAAGTGGTCCACAAAACATT
CTCCTTTCCTTCTGAAGGTTTTACGATGCAT 18 (0.000541%)

GCAAAAAAGAAAACCAAAGTGGTCCACAAAACATTCTCCTTTCCTTCTGAAGGTTTTACGATGCATTGTT
ATCATTAACCAGTCTTTTACTACTAAACTT 41 (0.001233%)

GCAAATTCTCATGGTTTGGGTTGGGTGGTGGAGAGCGCGTGTCATCTGCGGGTGGCACTGCCCACGGTG
GGCGGGCGGGCCTCTCTACTCGAAGGTGACC 30 (0.000902%)

GCAACAATCAGGACAGCACAGTCAGCCTGAGATGTCCCTGTAATCATGTTTTTGATAAAGTCTCTGTGTC
CTGGGGCATCAATGATAGTCACATAGTACT 26 (0.000782%)

GCAAGGGCTTGTCAGTTGGACGAGTTGGTGGTAGGATGCAGTCCAGAGCCTCAAGCAGCGTGGTTCCAC
TGGCATTGCCATCCTTACGGGTGACTTTCCA 33 (0.000992%)

GCACACTCCTTTGCTACTGGTCCTGTAATGGCAGAACCTTTCATCTCGCCTTTATTGTTCACTATGACTCC
TGCATTATCTTCAAAATAAAGAAACACGC 6 (0.000180%)

GCACTGCAGCAGATCATTTCATATTGCTTCCGTGGAGTGTGGCGAGTCAGCTAAATACTTTGACGCCGGT
GGGGATAGCGATGATTATGGTAGCGGAGGT 12 (0.000361%)

GCAGACTTGGTGACCTTGCCAGCTCCAGCAGCCTTCTTGTCCACTGCTTTGATGACACCCACCGCAACTG
TCTGTCTCATATCACGAACAGCAAAGCGAC 55 (0.001653%)

GCAGATCATTTCATATTGCTTCCGTGGAGTGTGGCGAGTCAGCTAAATACTTTGACGCCGGTGGGGATAG
CGATGATTATGGTAGCGGAGGTGAAATATG 19 (0.000571%)

GCAGCAACAATCAGGACAGCACAGTCAGCCTGAGATGTCCCTGTAATCATGTTTTTGATAAAGTCTCTGT
GTCCTGGGGCATCAATGATAGTCACATAGT 923 (0.027747%)

GCAGCAGATCATTTCATATTGCTTCCGTGGAGTGTGGCGAGTCAGCTAAATACTTTGACGCCGGTGGGGA
TAGCGATGATTATGGTAGCGGAGGTGAAAT 9 (0.000271%)

GCAGCGTGGTTCCACTGGCATTGCCATCCTTACGGGTGACTTTCCATCCCTTGAACCAAGGCATGTTAGC
ACTTGGCTCCAGCATGTTGTCACCATTCCA 22 (0.000661%)

GCAGGCGCAAGGGCTTGTCAGTTGGACGAGTTGGTGGTAGGATGCAGTCCAGAGCCTCAAGCAGCGTGG
TTCCACTGGCATTGCCATCCTTACGGGTGAC 82 (0.002465%)

GCAGGCTTATGCGGAGGAGAATGTTTTCATGTTACTTATACTAACATTAGTTCTTCTATAGGGTGATAGA
TTGGTCCAATTGGGTGTGAGGAGTTCAGTT 30 (0.000902%)

GCAGGTATTAGGGATAATATTCATTTAGCCTTCTGAGCTTTCTGGGCAGACTTGGTGACCTTGCCAGCTC
CAGCAGCCTTCTTGTCCACTGCTTTGATGA 103 (0.003096%)

GCAGTAATGTTAGCGGTTAGGCGTACGGCCAGGGCTATTGGTTGAATGAGTAGGCTGATGGTTTCGATA
ATAACTAGTATGGGGATAAGGGGTGTAGGTG 9 (0.000271%)

GCAGTCCAGAGCCTCAAGCAGCGTGGTTCCACTGGCATTGCCATCCTTACGGGTGACTTTCCATCCCTTG
AACCAAGGCATGTTAGCACTTGGCTCCAGC 35 (0.001052%)

GCAGTGAAGCCAGCTGCTTCCATTGGTGGGTCATTTTTGCTGTCACCAGCAACGTTGCCACGACGAACAT
CCTTGACAGACACATTCTTGACATTGAAGC 3 (0.000090%)

GCATCTGTTTTTAAGCCTAATGTGGGGACAGCTCATGAGTGCAAGACGTCTTGTGATGTAATTATTATAC
GAATGGGGGCTTCAATCGGGAGTACTACTC 4 (0.000120%)

GCATGAGTAGGTGGCCTGCAGTAATGTTAGCGGTTAGGCGTACGGCCAGGGCTATTGGTTGAATGAGTA
GGCTGATGGTTTCGATAATAACTAGTATGGG 21 (0.000631%)

GCATGCTCTCGGGTCTGCCCATTCTTGGAGATACCAGCTTCAAATTCACCAACACCAGCAGCAACAATCA
GGACAGCACAGTCAGCCTGAGATGTCCCTG 28 (0.000842%)

GCATTGCCATCCTTACGGGTGACTTTCCATCCCTTGAACCAAGGCATGTTAGCACTTGGCTCCAGCATGT
TGTCACCATTCCAACCAGAAATTGGCACAA 6 (0.000180%)

GCCAATTTTCTTAATGTAAGTGCTGACTTCCTTAACAATTTCCTCATATCTCTTCTGGCTGTAGGGTGGCT
CAGTGGAATCCATTTTGTTAACACCGACA 35 (0.001052%)

GCCACACGCAAAAAAGAAAACCAAAGTGGTCCACAAAACATTCTCCTTTCCTTCTGAAGGTTTTACGAT
GCATTGTTATCATTAACCAGTCTTTTACTAC 126 (0.003788%)

GCCACCTACGGTGAAAAGAAAGATGAATCCTAGGGCTCAGAGCACTGCAGCAGATCATTTCATATTGCT
TCCGTGGAGTGTGGCGAGTCAGCTAAATACT 27 (0.000812%)

GCCAGAAGGGCATGCTCTCGGGTCTGCCCATTCTTGGAGATACCAGCTTCAAATTCACCAACACCAGCA
GCAACAATCAGGACAGCACAGTCAGCCTGAG 40 (0.001202%)

GCCAGCTCCAGCAGCCTTCTTGTCCACTGCTTTGATGACACCCACCGCAACTGTCTGTCTCATATCACGA
ACAGCAAAGCGACCCAAAGGTGGATAGTCT 45 (0.001353%)

GCCAGCTGCTTCCATTGGTGGGTCATTTTTGCTGTCACCAGCAACGTTGCCACGACGAACATCCTTGACA
GACACATTCTTGACATTGAAGCCCACATTG 16 (0.000481%)

GCCAGGGCTATTGGTTGAATGAGTAGGCTGATGGTTTCGATAATAACTAGTATGGGGATAAGGGGTGTA
GGTGTGCCTTGTGGTAAGAAGTGGGCTAGGG 27 (0.000812%)

GCCAGTCAGGCCACCTACGGTGAAAAGAAAGATGAATCCTAGGGCTCAGAGCACTGCAGCAGATCATTT
CATATTGCTTCCGTGGAGTGTGGCGAGTCAG 25 (0.000752%)

GCCAGTGTTACTTTAATTGGACTGCCTTCGTAATTCATTGCCTCTGCTTCAACAATGTGCAACTCATCCTT
TGCACCAGCCCCTAAACTGACCGTTCTTA 138 (0.004148%)

GCCATGGCTAGGTTTATAGATAGTTGGGTGGTTGGTGTAAATGAGTGAGGCAGGAGTCCGAGGAGGTTA
GTTGTGGCAATAAAAATGATTAAGGATACTA 386 (0.011604%)

GCCCATACACATGAGTATTTGTCTAAAACATGTCTTCTTTGTAGCAGCTAGGCCCTGCCACCACTGTGCTT
GGCTGAGTTCACAAATCTGTTGTAACCTG 11 (0.000331%)

GCCTCAAGCAGCGTGGTTCCACTGGCATTGCCATCCTTACGGGTGACTTTCCATCCCTTGAACCAAGGCA
TGTTAGCACTTGGCTCCAGCATGTTGTCAC 186 (0.005591%)

GCCTGCAGTAATGTTAGCGGTTAGGCGTACGGCCAGGGCTATTGGTTGAATGAGTAGGCTGATGGTTTCG
ATAATAACTAGTATGGGGATAAGGGGTGTA 36 (0.001082%)

GCCTTCTGAGCTTTCTGGGCAGACTTGGTGACCTTGCCAGCTCCAGCAGCCTTCTTGTCCACTGCTTTGAT
GACACCCACCGCAACTGTCTGTCTCATAT 44 (0.001323%)

GCGCAAGGGCTTGTCAGTTGGACGAGTTGGTGGTAGGATGCAGTCCAGAGCCTCAAGCAGCGTGGTTCC
ACTGGCATTGCCATCCTTACGGGTGACTTTC 19 (0.000571%)

GCGCTTCCAATTAGGTGCATGAGTAGGTGGCCTGCAGTAATGTTAGCGGTTAGGCGTACGGCCAGGGCT
ATTGGTTGAATGAGTAGGCTGATGGTTTCGA 14 (0.000421%)

GCGGGTTTTAGGGGCTCTTTGGTGAAGAGTTTTATGGCGTCAGCGAAGGGTTGTAGTAGCCCGTAGGGG
CCTACAACGTTGGGGCCTTTGCGTAGTTGTA 25 (0.000752%)

GCGGTCTGTTAGTAGTATAGTGATGCCAGCAGCTAGGACTGGGAGAGATAGGAGAAGTAGGACTGCTGT
GATTAGGACGGATCAGACGAAGAGGGGCGTT 415 (0.012475%)

GCGGTGGTCAGGTCCCGGTATTCCCGGTACATGTTGTGGGTGCCGCTCCGGGAGTCATAGCGCAGCCAG
ATCCCGAAGTTCTTCACCCGCAGGGGGGACT 307 (0.009229%)

GCGGTTAGGCGTACGGCCAGGGCTATTGGTTGAATGAGTAGGCTGATGGTTTCGATAATAACTAGTATG

GGGATAAGGGGTGTAGGTGTGCCTTGTGGTA 35 (0.001052%)

GCGTACGGCCAGGGCTATTGGTTGAATGAGTAGGCTGATGGTTTCGATAATAACTAGTATGGGGATAAG
GGGTGTAGGTGTGCCTTGTGGTAAGAAGTGG 7 (0.000210%)

GCTAATACAATGCCAGTCAGGCCACCTACGGTGAAAAGAAAGATGAATCCTAGGGCTCAGAGCACTGCA
GCAGATCATTTCATATTGCTTCCGTGGAGTG 17 (0.000511%)

GCTACTGGTCCTGTAATGGCAGAACCTTTCATCTCGCCTTTATTGTTCACTATGACTCCTGCATTATCTTC
AAAATAAAGAAACACGCCATCTTTTCTAC 4 (0.000120%)

GCTACTTGTCCAATGATGGTAAAAGGGTAGCTTACTGGTTGTCCTCCGATTCAGGTTAGAATGAGGAGGT
CTGCGGCTAGGAGTCAATAAAGTGATTGGC 6 (0.000180%)

GCTAGGGTGACTTCATATGAGATTGTTTGGGCTACTGCTCGCAGTGCGCCGATCAGGGCGTAGTTTGAGT
TTGATGCTCACCCTGATCAGAGGATTGAGT 9 (0.000271%)

GCTAGGGTGGCGCTTCCAATTAGGTGCATGAGTAGGTGGCCTGCAGTAATGTTAGCGGTTAGGCGTACG
GCCAGGGCTATTGGTTGAATGAGTAGGCTGA 26 (0.000782%)

GCTAGGTGTAAGGAGAAGATGGTTAGGTCTACGGAGGCTCCAGGGTGGGAGTAGTTCCCTGCTAAGGGA
GGGTAGACTGTTCAACCTGTTCCTGCTCCGG 22 (0.000661%)

GCTAGGTTTATAGATAGTTGGGTGGTTGGTGTAAATGAGTGAGGCAGGAGTCCGAGGAGGTTAGTTGTG
GCAATAAAAATGATTAAGGATACTAGTATAA 44 (0.001323%)

GCTATTGGTTGAATGAGTAGGCTGATGGTTTCGATAATAACTAGTATGGGGATAAGGGGTGTAGGTGTG
CCTTGTGGTAAGAAGTGGGCTAGGGCATTTT 12 (0.000361%)

GCTCAGAGCACTGCAGCAGATCATTTCATATTGCTTCCGTGGAGTGTGGCGAGTCAGCTAAATACTTTGA
CGCCGGTGGGGATAGCGATGATTATGGTAG 56 (0.001683%)

GCTCAGTGGAATCCATTTTGTTAACACCGACAATTAGTTGTTTCACACCCAGTGTGTAAGCCAGAAGGGC
ATGCTCTCGGGTCTGCCCATTCTTGGAGAT 43 (0.001293%)

GCTCTCAACACACATGGGCTTGCCAGGAACCATATCAACAATGGCAGCATCACCAGACTTCAAGAATTT
AGGGCCATCTTCCAGCTTTTTACCAGAACGG 11 (0.000331%)

GCTCTCGGGTCTGCCCATTCTTGGAGATACCAGCTTCAAATTCACCAACACCAGCAGCAACAATCAGGAC
AGCACAGTCAGCCTGAGATGTCCCTGTAAT 10 (0.000301%)

GCTGACTTCCTTAACAATTTCCTCATATCTCTTCTGGCTGTAGGGTGGCTCAGTGGAATCCATTTTGTTAA
CACCGACAATTAGTTGTTTCACACCCAGT 85 (0.002555%)

GCTGCTTCCATTGGTGGGTCATTTTTGCTGTCACCAGCAACGTTGCCACGACGAACATCCTTGACAGACA
CATTCTTGACATTGAAGCCCACATTGTCCC 41 (0.001233%)

GCTGGAGTGGTAAAAGGCTCAGAAAAATCCTGCGAAGAAAAAAACTTCTGAGGTAATAAATAGGATTAT
CCCGTATCGAAGGCCTTTTTGGACAGGTGGT 12 (0.000361%)

GCTGTAGGGTGGCTCAGTGGAATCCATTTTGTTAACACCGACAATTAGTTGTTTCACACCCAGTGTGTAA
GCCAGAAGGGCATGCTCTCGGGTCTGCCCA 10 (0.000301%)

GCTTCACTCAAAGCTTCATGGTGCATTTCGACAGATTTTACTTCCGTTGTAACGTTGACTGGAGCAAAGG
TGACCACCATACCGGGTTTGAGAACACCAG 24 (0.000721%)

GCTTGTCAGTTGGACGAGTTGGTGGTAGGATGCAGTCCAGAGCCTCAAGCAGCGTGGTTCCACTGGCATT
GCCATCCTTACGGGTGACTTTCCATCCCTT14 (0.000421%)

GCTTTAGGGAGTCATAAGTGGAGTCCGTAAAGAGGTATCTTTACTATAAAAGCTATTGTGTAAGCTAGTC
ATATTAAGTTGTTGGCTCAGGAGTTTGATA 5 (0.000150%)

GCTTTCTGGGCAGACTTGGTGACCTTGCCAGCTCCAGCAGCCTTCTTGTCCACTGCTTTGATGACACCCAC
CGCAACTGTCTGTCTCATATCACGAACAG 16 (0.000481%)

GGAAAAGGTTGGGGAACAGCTAAATAGGTTGTTGTTGATTTGGTTAAAAAATAGTAGAGGGATGATGCT
AATAATTAGGCTGTGGGTGGTTGTGTTGATT 18 (0.000541%)

GGAACAGCTAAATAGGTTGTTGTTGATTTGGTTAAAAAATAGTAGAGGGATGATGCTAATAATTAGGCT
GTGGGTGGTTGTGTTGATTCAAATTATGTGT 12 (0.000361%)

GGAAGAAAGTTAGATTTACGCCGATGAATATGATAGTGAAATGGATTTTGGCGTAGGTTTGGTCTAGGG
TGTAGCCTGAGAATAGGGGAAATCAGTGAAT 3 (0.000090%)

GGAAGAGCTTCACTCAAAGCTTCATGGTGCATTTCGACAGATTTTACTTCCGTTGTAACGTTGACTGGAG
CAAAGGTGACCACCATACCGGGTTTGAGAA 124 (0.003728%)

GGAAGGGGTAGGCTATGTGTTTTGTCAGGGGGTTGAGAATGAGTGTGAGGCGTATTATACCATAGCCGC
CTAGTTTTAAGAGTACTGCGGCAAGTACTAT 38 (0.001142%)

GGAATTGCATCTGTTTTTAAGCCTAATGTGGGGACAGCTCATGAGTGCAAGACGTCTTGTGATGTAATTA
TTATACGAATGGGGGCTTCAATCGGGAGTA 6 (0.000180%)

GGACACCAGTTTTAGCCAACATAGCATAGTACTCTATTTCAGATTTCCTCAAAGCTGGGCAGTTGTTAGC
GAGAATGACCAATTTCGCTTTGCCTTGTCT 4 (0.000120%)

GGACGAGTTGGTGGTAGGATGCAGTCCAGAGCCTCAAGCAGCGTGGTTCCACTGGCATTGCCATCCTTA
CGGGTGACTTTCCATCCCTTGAACCAAGGCA 40 (0.001202%)

GGAGAAGATGGTTAGGTCTACGGAGGCTCCAGGGTGGGAGTAGTTCCCTGCTAAGGGAGGGTAGACTGT
TCAACCTGTTCCTGCTCCGGCCTCCACTATA 45 (0.001353%)

GGAGAGGCAGGCGCAAGGGCTTGTCAGTTGGACGAGTTGGTGGTAGGATGCAGTCCAGAGCCTCAAGC
AGCGTGGTTCCACTGGCATTGCCATCCTTACG 45 (0.001353%)

GGAGGAAAAGGTTGGGGAACAGCTAAATAGGTTGTTGTTGATTTGGTTAAAAAATAGTAGAGGGATGAT
GCTAATAATTAGGCTGTGGGTGGTTGTGTTG 8 (0.000240%)

GGAGGGGGGTTGTTAGGGGGTCGGAGGAAAAGGTTGGGGAACAGCTAAATAGGTTGTTGTTGATTTGGT
TAAAAAATAGTAGAGGGATGATGCTAATAAT 26 (0.000782%)

GGAGGGTAAAATAGAGACCCAGTAAAATTGTAATAAGCAGTGCTTGAATTATTTGGTTTCGGTTGTTTTC
TATTAGACTATGGTGAGCTCAGGTGATTGA 41 (0.001233%)

GGAGTACTACTCGATTGTCAACGTCAAGGAGTCGCAGGTCGCCTGGTTCTAGGAATAATGGGGGAAGTA
TGTAGGAGTTGAAGATTAGTCCGCCGTAGTC 12 (0.000361%)

GGAGTCATAAGTGGAGTCCGTAAAGAGGTATCTTTACTATAAAAGCTATTGTGTAAGCTAGTCATATTAA
GTTGTTGGCTCAGGAGTTTGATAGTTCTTG 18 (0.000541%)

GGAGTCCGTAAAGAGGTATCTTTACTATAAAAGCTATTGTGTAAGCTAGTCATATTAAGTTGTTGGCTCA
GGAGTTTGATAGTTCTTGGGCAGTGAGAGT 22 (0.000661%)

GGATAATATTCATTTAGCCTTCTGAGCTTTCTGGGCAGACTTGGTGACCTTGCCAGCTCCAGCAGCCTTCT
TGTCCACTGCTTTGATGACACCCACCGCA 164 (0.004930%)

GGATAATCACCTGAGCAGTGAAGCCAGCTGCTTCCATTGGTGGGTCATTTTTGCTGTCACCAGCAACGTT
GCCACGACGAACATCCTTGACAGACACATT 31 (0.000932%)

GGATAGTACAAGGAAGGGGTAGGCTATGTGTTTTGTCAGGGGGTTGAGAATGAGTGTGAGGCGTATTAT
ACCATAGCCGCCTAGTTTTAAGAGTACTGCG 46 (0.001383%)

GGATGCAGTCCAGAGCCTCAAGCAGCGTGGTTCCACTGGCATTGCCATCCTTACGGGTGACTTTCCATCC
CTTGAACCAAGGCATGTTAGCACTTGGCTC 25 (0.000752%)

GGATGCTACTTGTCCAATGATGGTAAAAGGGTAGCTTACTGGTTGTCCTCCGATTCAGGTTAGAATGAGG
AGGTCTGCGGCTAGGAGTCAATAAAGTGAT 17 (0.000511%)

GGCAAATTCTGCCAGATACCTGTGGTAGTCCCCTTTCATTTTATAATAGAAAACCTTGGACTCGCCAGTG
TTAGCTGCTGGAATGAGGTGTTTGTCCAGT 8 (0.000240%)

GGCAGACTTGGTGACCTTGCCAGCTCCAGCAGCCTTCTTGTCCACTGCTTTGATGACACCCACCGCAACT
GTCTGTCTCATATCACGAACAGCAAAGCGA 87 (0.002615%)

GGCAGGCGCAAGGGCTTGTCAGTTGGACGAGTTGGTGGTAGGATGCAGTCCAGAGCCTCAAGCAGCGTG
GTTCCACTGGCATTGCCATCCTTACGGGTGA 85 (0.002555%)

GGCAGGTATTAGGGATAATATTCATTTAGCCTTCTGAGCTTTCTGGGCAGACTTGGTGACCTTGCCAGCT
CCAGCAGCCTTCTTGTCCACTGCTTTGATG 118 (0.003547%)

GGCATGCTCTCGGGTCTGCCCATTCTTGGAGATACCAGCTTCAAATTCACCAACACCAGCAGCAACAATC
AGGACAGCACAGTCAGCCTGAGATGTCCCT 28 (0.000842%)

GGCATTGCCATCCTTACGGGTGACTTTCCATCCCTTGAACCAAGGCATGTTAGCACTTGGCTCCAGCATG
TTGTCACCATTCCAACCAGAAATTGGCACA 21 (0.000631%)

GGCCACCTACGGTGAAAAGAAAGATGAATCCTAGGGCTCAGAGCACTGCAGCAGATCATTTCATATTGC
TTCCGTGGAGTGTGGCGAGTCAGCTAAATAC 13 (0.000391%)

GGCCAGGGCTATTGGTTGAATGAGTAGGCTGATGGTTTCGATAATAACTAGTATGGGGATAAGGGGTGT
AGGTGTGCCTTGTGGTAAGAAGTGGGCTAGG 13 (0.000391%)

GGCCCCTAAGATAGAGGAGACACCTGCTAGGTGTAAGGAGAAGATGGTTAGGTCTACGGAGGCTCCAG
GGTGGGAGTAGTTCCCTGCTAAGGGAGGGTAG 4 (0.000120%)

GGCCTGCAGTAATGTTAGCGGTTAGGCGTACGGCCAGGGCTATTGGTTGAATGAGTAGGCTGATGGTTTC
GATAATAACTAGTATGGGGATAAGGGGTGT 10 (0.000301%)

GGCGCAAGGGCTTGTCAGTTGGACGAGTTGGTGGTAGGATGCAGTCCAGAGCCTCAAGCAGCGTGGTTC
CACTGGCATTGCCATCCTTACGGGTGACTTT 47 (0.001413%)

GGCGCTTCCAATTAGGTGCATGAGTAGGTGGCCTGCAGTAATGTTAGCGGTTAGGCGTACGGCCAGGGC
TATTGGTTGAATGAGTAGGCTGATGGTTTCG 92 (0.002766%)

GGCGGGTTTTAGGGGCTCTTTGGTGAAGAGTTTTATGGCGTCAGCGAAGGGTTGTAGTAGCCCGTAGGG
GCCTACAACGTTGGGGCCTTTGCGTAGTTGT 4 (0.000120%)

GGCGTACGGCCAGGGCTATTGGTTGAATGAGTAGGCTGATGGTTTCGATAATAACTAGTATGGGGATAA
GGGGTGTAGGTGTGCCTTGTGGTAAGAAGTG 18 (0.000541%)

GGCTAGGGTGACTTCATATGAGATTGTTTGGGCTACTGCTCGCAGTGCGCCGATCAGGGCGTAGTTTGAG
TTTGATGCTCACCCTGATCAGAGGATTGAG 7 (0.000210%)

GGCTAGGTTTATAGATAGTTGGGTGGTTGGTGTAAATGAGTGAGGCAGGAGTCCGAGGAGGTTAGTTGT
GGCAATAAAAATGATTAAGGATACTAGTATA 27 (0.000812%)

GGCTATTGGTTGAATGAGTAGGCTGATGGTTTCGATAATAACTAGTATGGGGATAAGGGGTGTAGGTGT

GCCTTGTGGTAAGAAGTGGGCTAGGGCATTT 9 (0.000271%)

GGCTCAGAGCACTGCAGCAGATCATTTCATATTGCTTCCGTGGAGTGTGGCGAGTCAGCTAAATACTTTG
ACGCCGGTGGGGATAGCGATGATTATGGTA 5 (0.000150%)

GGCTCAGTGGAATCCATTTTGTTAACACCGACAATTAGTTGTTTCACACCCAGTGTGTAAGCCAGAAGGG
CATGCTCTCGGGTCTGCCCATTCTTGGAGA 20 (0.000601%)

GGCTGGAGTGGTAAAAGGCTCAGAAAAATCCTGCGAAGAAAAAAACTTCTGAGGTAATAAATAGGATT
ATCCCGTATCGAAGGCCTTTTTGGACAGGTGG 450 (0.013528%)

GGCTGTAGGGTGGCTCAGTGGAATCCATTTTGTTAACACCGACAATTAGTTGTTTCACACCCAGTGTGTA
AGCCAGAAGGGCATGCTCTCGGGTCTGCCC 5 (0.000150%)

GGCTTATGCGGAGGAGAATGTTTTCATGTTACTTATACTAACATTAGTTCTTCTATAGGGTGATAGATTG
GTCCAATTGGGTGTGAGGAGTTCAGTTATA 9 (0.000271%)

GGCTTCAATCGGGAGTACTACTCGATTGTCAACGTCAAGGAGTCGCAGGTCGCCTGGTTCTAGGAATAAT
GGGGGAAGTATGTAGGAGTTGAAGATTAGT 12 (0.000361%)

GGCTTGTCAGTTGGACGAGTTGGTGGTAGGATGCAGTCCAGAGCCTCAAGCAGCGTGGTTCCACTGGCA
TTGCCATCCTTACGGGTGACTTTCCATCCCT 14 (0.000421%)

GGGAAGAAAGTTAGATTTACGCCGATGAATATGATAGTGAAATGGATTTTGGCGTAGGTTTGGTCTAGG
GTGTAGCCTGAGAATAGGGGAAATCAGTGAA 8 (0.000240%)

GGGAATTGCATCTGTTTTTAAGCCTAATGTGGGGACAGCTCATGAGTGCAAGACGTCTTGTGATGTAATT
ATTATACGAATGGGGGCTTCAATCGGGAGT 776 (0.023328%)

GGGAGTACTACTCGATTGTCAACGTCAAGGAGTCGCAGGTCGCCTGGTTCTAGGAATAATGGGGGAAGT
ATGTAGGAGTTGAAGATTAGTCCGCCGTAGT 32 (0.000962%)

GGGAGTCATAAGTGGAGTCCGTAAAGAGGTATCTTTACTATAAAAGCTATTGTGTAAGCTAGTCATATTA
AGTTGTTGGCTCAGGAGTTTGATAGTTCTT 42 (0.001263%)

GGGATAATATTCATTTAGCCTTCTGAGCTTTCTGGGCAGACTTGGTGACCTTGCCAGCTCCAGCAGCCTT
CTTGTCCACTGCTTTGATGACACCCACCGC 31 (0.000932%)

GGGATAGTACAAGGAAGGGGTAGGCTATGTGTTTGTCAGGGGGTTGAGAATGAGTGTGAGGCGTATTA
TACCATAGCCGCCTAGTTTTAAGAGTACTGC 7 (0.000210%)

GGGCAGACTTGGTGACCTTGCCAGCTCCAGCAGCCTTCTTGTCCACTGCTTTGATGACACCCACCGCAAC
TGTCTGTCTCATATCACGAACAGCAAAGCG 21 (0.000631%)

GGGCATGCTCTCGGGTCTGCCCATTCTTGGAGATACCAGCTTCAAATTCACCAACACCAGCAGCAACAAT
CAGGACAGCACAGTCAGCCTGAGATGTCCC 19 (0.000571%)

GGGCTATTGGTTGAATGAGTAGGCTGATGGTTTCGATAATAACTAGTATGGGGATAAGGGGTGTAGGTG
TGCCTTGTGGTAAGAAGTGGGCTAGGGCATT 43 (0.001293%)

GGGCTCAGAGCACTGCAGCAGATCATTTCATATTGCTTCCGTGGAGTGTGGCGAGTCAGCTAAATACTTT
GACGCCGGTGGGGATAGCGATGATTATGGT 32 (0.000962%)

GGGCTTCAATCGGGAGTACTACTCGATTGTCAACGTCAAGGAGTCGCAGGTCGCCTGGTTCTAGGAATA
ATGGGGGAAGTATGTAGGAGTTGAAGATTAG 9 (0.000271%)

GGGCTTGTCAGTTGGACGAGTTGGTGGTAGGATGCAGTCCAGAGCCTCAAGCAGCGTGGTTCCACTGGC
ATTGCCATCCTTACGGGTGACTTTCCATCCC 36 (0.001082%)

GGGCTTTAGGGAGTCATAAGTGGAGTCCGTAAAGAGGTATCTTTACTATAAAAGCTATTGTGTAAGCTA
GTCATATTAAGTTGTTGGCTCAGGAGTTTGA 5 (0.000150%)

GGGGAACAGCTAAATAGGTTGTTGTTGATTTGGTTAAAAAATAGTAGAGGGATGATGCTAATAATTAGG
CTGTGGGTGGTTGTGTTGATTCAAATTATGT 8 (0.000240%)

GGGGCTTCAATCGGGAGTACTACTCGATTGTCAACGTCAAGGAGTCGCAGGTCGCCTGGTTCTAGGAAT
AATGGGGGAAGTATGTAGGAGTTGAAGATTA 41 (0.001233%)

GGGGGCTTCAATCGGGAGTACTACTCGATTGTCAACGTCAAGGAGTCGCAGGTCGCCTGGTTCTAGGAA
TAATGGGGGAAGTATGTAGGAGTTGAAGATT 59 (0.001774%)
GGGGGGGGGGGGGGGGGGGGGGGGGGGGGGGGGGGGGG 3497 (0.042050%)

GGGGGGTTGTTAGGGGGTCGGAGGAAAAGGTTGGGGAACAGCTAAATAGGTTGTTGTTGATTTGGTTAA
AAAATAGTAGAGGGATGATGCTAATAATTAG 5 (0.000150%)

GGGGGTCGGAGGAAAAGGTTGGGGAACAGCTAAATAGGTTGTTGTTGATTTGGTTAAAAAATAGTAGAG
GGATGATGCTAATAATTAGGCTGTGGGTGGT 8 (0.000240%)

GGGGGTTGTTAGGGGGTCGGAGGAAAAGGTTGGGGAACAGCTAAATAGGTTGTTGTTGATTTGGTTAAA
AAATAGTAGAGGGATGATGCTAATAATTAGG 16 (0.000481%)

GGGGTAGGCTATGTGTTTTGTCAGGGGGTTGAGAATGAGTGTGAGGCGTATTATACCATAGCCGCCTAGT
TTTAAGAGTACTGCGGCAAGTACTATTGAC 19 (0.000571%)

GGGGTGGCAGGTATTAGGGATAATATTCATTTAGCCTTCTGAGCTTTCTGGGCAGACTTGGTGACCTTGC
CAGCTCCAGCAGCCTTCTTGTCCACTGCTT 11 (0.000331%)

GGGGTTGTTAGGGGGTCGGAGGAAAAGGTTGGGGAACAGCTAAATAGGTTGTTGTTGATTTGGTTAAAA
AATAGTAGAGGGATGATGCTAATAATTAGGC 8 (0.000240%)

GGGTAAAATAGAGACCCAGTAAAATTGTAATAAGCAGTGCTTGAATTATTTGGTTTCGGTTGTTTTCTAT
TAGACTATGGTGAGCTCAGGTGATTGATAC 21 (0.000631%)

GGGTAGGCTATGTGTTTTGTCAGGGGGTTGAGAATGAGTGTGAGGCGTATTATACCATAGCCGCCTAGTT
TTAAGAGTACTGCGGCAAGTACTATTGACC 29 (0.000872%)

GGGTCATTTTTGCTGTCACCAGCAACGTTGCCACGACGAACATCCTTGACAGACACATTCTTGACATTGA
AGCCCACATTGTCCCCAGGAAGAGCTTCAC 36 (0.001082%)

GGGTGACTTCATATGAGATTGTTTGGGCTACTGCTCGCAGTGCGCCGATCAGGGCGTAGTTTGAGTTTGA
TGCTCACCCTGATCAGAGGATTGAGTAAAC 12 (0.000361%)

GGGTGGCGCTTCCAATTAGGTGCATGAGTAGGTGGCCTGCAGTAATGTTAGCGGTTAGGCGTACGGCCA
GGGCTATTGGTTGAATGAGTAGGCTGATGGT 3 (0.000090%)

GGGTGGCTCAGTGGAATCCATTTTGTTAACACCGACAATTAGTTGTTTCACACCCAGTGTGTAAGCCAGA
AGGGCATGCTCTCGGGTCTGCCCATTCTTG 52 (0.001563%)

GGGTGGTTGGTGTAAATGAGTGAGGCAGGAGTCCGAGGAGGTTAGTTGTGGCAATAAAAATGATTAAGG
ATACTAGTATAAGAGATCAGGTTCGTCCTTT 16 (0.000481%)

GGGTTGGGTGGTGGAGAGCGCGTGTCATCTGCGGGTGGCACTGCCCACGGTGGGCGGGCGGGCCTCTCT
ACTCGAAGGTGACCACGTTTAGATTCTGAGA 6 (0.000180%)

GGGTTGTTAGGGGGTCGGAGGAAAAGGTTGGGGAACAGCTAAATAGGTTGTTGTTGATTTGGTTAAAAA
ATAGTAGAGGGATGATGCTAATAATTAGGCT 3 (0.000090%)

GGGTTTTAGGGGCTCTTTGGTGAAGAGTTTTATGGCGTCAGCGAAGGGTTGTAGTAGCCCGTAGGGGCCT

ACAACGTTGGGGCCTTTGCGTAGTTGTATA 3 (0.000090%)

GGTAAAATAGAGACCCAGTAAAATTGTAATAAGCAGTGCTTGAATTATTTGGTTTCGGTTGTTTTCTATT
AGACTATGGTGAGCTCAGGTGATTGATACT 11 (0.000331%)

GGTACCTTTCTCTTTGGCTTCTTTCTTTTTCTGATCATTTTCCTTCACACGTTTCAGGAAGCTATCTCGGCT
CTTAGAGTGCTTAATGTGCTCAATACGC 9 (0.000271%)

GGTAGATGTGGCGGGTTTTAGGGGCTCTTTGGTGAAGAGTTTTATGGCGTCAGCGAAGGGTTGTAGTAGC
CCGTAGGGGCCTACAACGTTGGGGCCTTTG 118 (0.003547%)

GGTAGGATGCAGTCCAGAGCCTCAAGCAGCGTGGTTCCACTGGCATTGCCATCCTTACGGGTGACTTTCC
ATCCCTTGAACCAAGGCATGTTAGCACTTG 15 (0.000451%)

GGTAGTCCCCTTTCATTTTATAATAGAAAACCTTGGACTCGCCAGTGTTAGCTGCTGGAATGAGGTGTTT
GTCCAGTACATCCAGAATGTCACAACAGAT 3 (0.000090%)

GGTATCTTTACTATAAAAGCTATTGTGTAAGCTAGTCATATTAAGTTGTTGGCTCAGGAGTTTGATAGTTC
TTGGGCAGTGAGAGTGAGTAGTAGAATGT5 (0.000150%)

GGTATTAGGGATAATATTCATTTAGCCTTCTGAGCTTTCTGGGCAGACTTGGTGACCTTGCCAGCTCCAG
CAGCCTTCTTGTCCACTGCTTTGATGACAC 27 (0.000812%)

GGTCAGGTCCCGGTATTCCCGGTACATGTTGTGGGTGCCGCTCCGGGAGTCATAGCGCAGCCAGATCCCG
AAGTTCTTCACCCGCAGGGGGGACTTCTCA 7 (0.000210%)

GGTCATTTTTGCTGTCACCAGCAACGTTGCCACGACGAACATCCTTGACAGACACATTCTTGACATTGAA
GCCCACATTGTCCCCAGGAAGAGCTTCACT 27 (0.000812%)

GGTCGGAGGAAAAGGTTGGGGAACAGCTAAATAGGTTGTTGTTGATTTGGTTAAAAAATAGTAGAGGGA
TGATGCTAATAATTAGGCTGTGGGTGGTTGT 3 (0.000090%)

GGTCTGTTAGTAGTATAGTGATGCCAGCAGCTAGGACTGGGAGAGATAGGAGAAGTAGGACTGCTGTGA
TTAGGACGGATCAGACGAAGAGGGGCGTTTG 3 (0.000090%)

GGTGACCTTGCCAGCTCCAGCAGCCTTCTTGTCCACTGCTTTGATGACACCCACCGCAACTGTCTGTCTCA
TATCACGAACAGCAAAGCGACCCAAAGGT 20 (0.000601%)

GGTGACTTCATATGAGATTGTTTGGGCTACTGCTCGCAGTGCGCCGATCAGGGCGTAGTTTGAGTTTGAT
GCTCACCCTGATCAGAGGATTGAGTAAACG 7 (0.000210%)

GGTGATCGGCGATCAGAGGGCGATGAAGTTCTAGATCCATTGAGACAAGCTCTAGACAGTAGCATGCAG
TCCCACAACTTGTACCAGCATCCCCAGCGTC 6 (0.000180%)

GGTGCATGAGTAGGTGGCCTGCAGTAATGTTAGCGGTTAGGCGTACGGCCAGGGCTATTGGTTGAATGA
GTAGGCTGATGGTTTCGATAATAACTAGTAT 15 (0.000451%)

GGTGGCAGGTATTAGGGATAATATTCATTTAGCCTTCTGAGCTTTCTGGGCAGACTTGGTGACCTTGCCA
GCTCCAGCAGCCTTCTTGTCCACTGCTTTG3 (0.000090%)

GGTGGCCTGCAGTAATGTTAGCGGTTAGGCGTACGGCCAGGGCTATTGGTTGAATGAGTAGGCTGATGG
TTTCGATAATAACTAGTATGGGGATAAGGGG 8 (0.000240%)

GGTGGCGCTTCCAATTAGGTGCATGAGTAGGTGGCCTGCAGTAATGTTAGCGGTTAGGCGTACGGCCAG
GGCTATTGGTTGAATGAGTAGGCTGATGGTT 12 (0.000361%)

GGTGGCTCAGTGGAATCCATTTGTTAACACCGACAATTAGTTGTTTCACACCCAGTGTGTAAGCCAGAA
GGGCATGCTCTCGGGTCTGCCCATTCTTGG 24 (0.000721%)

GGTGGGTCATTTTTGCTGTCACCAGCAACGTTGCCACGACGAACATCCTTGACAGACACATTCTTGACAT
TGAAGCCCACATTGTCCCCAGGAAGAGCTT 24 (0.000721%)

GGTGGTAGGATGCAGTCCAGAGCCTCAAGCAGCGTGGTTCCACTGGCATTGCCATCCTTACGGGTGACTT
TCCATCCCTTGAACCAAGGCATGTTAGCAC 21 (0.000631%)

GGTGGTTGGTGTAAATGAGTGAGGCAGGAGTCCGAGGAGGTTAGTTGTGGCAATAAAAATGATTAAGGA
TACTAGTATAAGAGATCAGGTTCGTCCTTTA 7 (0.000210%)

GGTGTAAATGAGTGAGGCAGGAGTCCGAGGAGGTTAGTTGTGGCAATAAAAATGATTAAGGATACTAGT
ATAAGAGATCAGGTTCGTCCTTTAGTGTTGT 7 (0.000210%)

GGTGTAAGGAGAAGATGGTTAGGTCTACGGAGGCTCCAGGGTGGGAGTAGTTCCCTGCTAAGGGAGGGT
AGACTGTTCAACCTGTTCCTGCTCCGGCCTC 3 (0.000090%)

GGTTAGGCGTACGGCCAGGGCTATTGGTTGAATGAGTAGGCTGATGGTTTCGATAATAACTAGTATGGG
GATAAGGGGTGTAGGTGTGCCTTGTGGTAAG 12 (0.000361%)

GGTTGAATGAGTAGGCTGATGGTTTCGATAATAACTAGTATGGGGATAAGGGGTGTAGGTGTGCCTTGT
GGTAAGAAGTGGGCTAGGGCATTTTTAATCT 31 (0.000932%)

GGTTGGGTGGTGGAGAGCGCGTGTCATCTGCGGGTGGCACTGCCCACGGTGGGCGGGCGGGCCTCTCTA
CTCGAAGGTGACCACGTTTAGATTCTGAGAC 3 (0.000090%)

GGTTGGTGTAAATGAGTGAGGCAGGAGTCCGAGGAGGTTAGTTGTGGCAATAAAAATGATTAAGGATAC
TAGTATAAGAGATCAGGTTCGTCCTTTAGTG 40 (0.001202%)

GGTTGTTAGGGGGTCGGAGGAAAAGGTTGGGGAACAGCTAAATAGGTTGTTGTTGATTTGGTTAAAAAA
TAGTAGAGGGATGATGCTAATAATTAGGCTG 12 (0.000361%)

GGTTTATAGATAGTTGGGTGGTTGGTGTAAATGAGTGAGGCAGGAGTCCGAGGAGGTTAGTTGTGGCAA
TAAAAATGATTAAGGATACTAGTATAAGAGA 27 (0.000812%)

GGTTTGGGTTGGGTGGTGGAGAGCGCGTGTCATCTGCGGGTGGCACTGCCCACGGTGGGCGGGCGGGCC
TCTCTACTCGAAGGTGACCACGTTTAGATTC 8 (0.000240%)

GTAAAATAGAGACCCAGTAAAATTGTAATAAGCAGTGCTTGAATTATTTGGTTTCGGTTGTTTTCTATTA
GACTATGGTGAGCTCAGGTGATTGATACTC 66 (0.001984%)

GTAAAATTGTAATAAGCAGTGCTTGAATTATTTGGTTTCGGTTGTTTTCTATTAGACTATGGTGAGCTCAG
GTGATTGATACTCCTGATGCGAGTAATAC 85 (0.002555%)

GTAAAGAGGTATCTTTACTATAAAAGCTATTGTGTAAGCTAGTCATATTAAGTTGTTGGCTCAGGAGTTT
GATAGTTCTTGGGCAGTGAGAGTGAGTAGT 42 (0.001263%)

GTAAATGAGTGAGGCAGGAGTCCGAGGAGGTTAGTTGTGGCAATAAAAATGATTAAGGATACTAGTATA
AGAGATCAGGTTCGTCCTTTAGTGTTGTGTA 33 (0.000992%)

GTAAGCCAGAAGGGCATGCTCTCGGGTCTGCCCATTCTTGGAGATACCAGCTTCAAATTCACCAACACCA
GCAGCAACAATCAGGACAGCACAGTCAGCC 29 (0.000872%)

GTAAGGAGAAGATGGTTAGGTCTACGGAGGCTCCAGGGTGGGAGTAGTTCCCTGCTAAGGGAGGGTAG
ACTGTTCAACCTGTTCCTGCTCCGGCCTCCAC 16 (0.000481%)

GTAAGTGCTGACTTCCTTAACAATTTCCTCATATCTCTTCTGGCTGTAGGGTGGCTCAGTGGAATCCATTT
TGTTAACACCGACAATTAGTTGTTTCACA 5 (0.000150%)

GTAATCATGTTTTTGATAAAGTCTCTGTGTCCTGGGGCATCAATGATAGTCACATAGTACTTGCTGGTCTC
AAATTTCCACAAGGAGATATCAATGGTGA18 (0.000541%)

GTAATGTTAGCGGTTAGGCGTACGGCCAGGGCTATTGGTTGAATGAGTAGGCTGATGGTTTCGATAATA
ACTAGTATGGGGATAAGGGGTGTAGGTGTGC 34 (0.001022%)

GTACAAGGAAGGGGTAGGCTATGTGTTTTGTCAGGGGGTTGAGAATGAGTGTGAGGCGTATTATACCAT
AGCCGCCTAGTTTTAAGAGTACTGCGGCAAG 13 (0.000391%)

GTACCTTTCTCTTTGGCTTCTTTCTTTTTCTGATCATTTTCCTTCACACGTTTCAGGAAGCTATCTCGGCTC
TTAGAGTGCTTAATGTGCTCAATACGCA 10 (0.000301%)

GTACGGCCAGGGCTATTGGTTGAATGAGTAGGCTGATGGTTTCGATAATAACTAGTATGGGGATAAGGG
GTGTAGGTGTGCCTTGTGGTAAGAAGTGGGC 23 (0.000691%)

GTACTACTCGATTGTCAACGTCAAGGAGTCGCAGGTCGCCTGGTTCTAGGAATAATGGGGGAAGTATGT
AGGAGTTGAAGATTAGTCCGCCGTAGTCGGT 4 (0.000120%)

GTACTCTGAGGCTTGTAGGAGGGTAAAATAGAGACCCAGTAAAATTGTAATAAGCAGTGCTTGAATTAT
TTGGTTTCGGTTGTTTTCTATTAGACTATGG 4 (0.000120%)

GTAGACATCCTGGAGAGGCAGGCGCAAGGGCTTGTCAGTTGGACGAGTTGGTGGTAGGATGCAGTCCAG
AGCCTCAAGCAGCGTGGTTCCACTGGCATTG 14 (0.000421%)

GTAGAGGGAGTATAGGGCTGTGACTAGTATGTTGAGTCCTGTAAGTAGGAGAGTGATATTTGATCAGGA
GAACGTGGTTACTAGCACAGAGAGTTCTCCC 8 (0.000240%)

GTAGATGTGGCGGGTTTTAGGGGCTCTTTGGTGAAGAGTTTTATGGCGTCAGCGAAGGGTTGTAGTAGCC
CGTAGGGGCCTACAACGTTGGGGCCTTTGC 7 (0.000210%)

GTAGCCAATTTTCTTAATGTAAGTGCTGACTTCCTTAACAATTTCCTCATATCTCTTCTGGCTGTAGGGTG
GCTCAGTGGAATCCATTTTGTTAACACCG 15 (0.000451%)

GTAGGATGCAGTCCAGAGCCTCAAGCAGCGTGGTTCCACTGGCATTGCCATCCTTACGGGTGACTTTCCA
TCCCTTGAACCAAGGCATGTTAGCACTTGG 56 (0.001683%)

GTAGGGTGGCTCAGTGGAATCCATTTTGTTAACACCGACAATTAGTTGTTTCACACCCAGTGTGTAAGCC
AGAAGGGCATGCTCTCGGGTCTGCCCATTC 24 (0.000721%)

GTAGGTGGCCTGCAGTAATGTTAGCGGTTAGGCGTACGGCCAGGGCTATTGGTTGAATGAGTAGGCTGA
TGGTTTCGATAATAACTAGTATGGGGATAAG 43 (0.001293%)

GTAGTCCCCTTTCATTTTATAATAGAAAACCTTGGACTCGCCAGTGTTAGCTGCTGGAATGAGGTGTTTG
TCCAGTACATCCAGAATGTCACAACAGATT 4 (0.000120%)

GTAGTGATGGACACCAGTTTTAGCCAACATAGCATAGTACTCTATTTCAGATTTCCTCAAAGCTGGGCAG
TTGTTAGCGAGAATGACCAATTTCGCTTTG 9 (0.000271%)

GTATTAGGGATAATATTCATTTAGCCTTCTGAGCTTTCTGGGCAGACTTGGTGACCTTGCCAGCTCCAGC
AGCCTTCTTGTCCACTGCTTTGATGACACC 14 (0.000421%)

GTATTTCTTTGCAACTTCTGTCTGGGCATGGGCGGCATCGCAAAGTGAATACAGATGGGCCATCTCCATT
TCCGATTTGGCCAGGTTAATAGCTTTGTTGAACATGTCAATGGCTTTCTCCATGTTTCCTCTTTGTACTTC
AATAGTTC 2 (0.000090%)

GTATTTGTCTAAAACATGTCTTCTTTGTAGCAGCTAGGCCCTGCCACCACTGTGCTTGGCTGAGTTCACAA
ATCTGTTGTAACCTGTAGCTTCCCTGTCA 4 (0.000120%)

GTCAGGCCACCTACGGTGAAAAGAAAGATGAATCCTAGGGCTCAGAGCACTGCAGCAGATCATTTCATA
TTGCTTCCGTGGAGTGTGGCGAGTCAGCTAA 13 (0.000391%)

GTCAGGTCCCGGTATTCCCGGTACATGTTGTGGGTGCCGCTCCGGGAGTCATAGCGCAGCCAGATCCCGA
AGTTCTTCACCCGCAGGGGGGACTTCTCAA 21 (0.000631%)

GTCAGTTCAGTGTTTTAATCTGACGCAGGCTTATGCGGAGGAGAATGTTTTCATGTTACTTATACTAACA
TTAGTTCTTCTATAGGGTGATAGATTGGTC 345 (0.010371%)

GTCAGTTGGACGAGTTGGTGGTAGGATGCAGTCCAGAGCCTCAAGCAGCGTGGTTCCACTGGCATTGCC
ATCCTTACGGGTGACTTTCCATCCCTTGAAC 11 (0.000331%)

GTCATAAGTGGAGTCCGTAAAGAGGTATCTTTACTATAAAAGCTATTGTGTAAGCTAGTCATATTAAGTT
GTTGGCTCAGGAGTTTGATAGTTCTTGGGC 12 (0.000361%)

GTCATTTTTGCTGTCACCAGCAACGTTGCCACGACGAACATCCTTGACAGACACATTCTTGACATTGAAG
CCCACATTGTCCCCAGGAAGAGCTTCACTC 71 (0.002134%)

GTCCAATGATGGTAAAAGGGTAGCTTACTGGTTGTCCTCCGATTCAGGTTAGAATGAGGAGGTCTGCGG
CTAGGAGTCAATAAAGTGATTGGCTTAGTGG 9 (0.000271%)

GTCCCCAGGAAGAGCTTCACTCAAAGCTTCATGGTGCATTTCGACAGATTTTACTTCCGTTGTAACGTTG
ACTGGAGCAAAGGTGACCACCATACCGGGT 8 (0.000240%)

GTCCCCTTTCATTTTATAATAGAAAACCTTGGACTCGCCAGTGTTAGCTGCTGGAATGAGGTGTTTGTCC
AGTACATCCAGAATGTCACAACAGATTAAC 7 (0.000210%)

GTCCCGGTATTCCCGGTACATGTTGTGGGTGCCGCTCCGGGAGTCATAGCGCAGCCAGATCCCGAAGTTC
TTCACCCGCAGGGGGGACTTCTCAAACACC 3 (0.000090%)

GTCGGAGGAAAAGGTTGGGGAACAGCTAAATAGGTTGTTGTTGATTTGGTTAAAAAATAGTAGAGGGAT
GATGCTAATAATTAGGCTGTGGGTGGTTGTG 12 (0.000361%)

GTCTGTTAGTAGTATAGTGATGCCAGCAGCTAGGACTGGGAGAGATAGGAGAAGTAGGACTGCTGTGAT
TAGGACGGATCAGACGAAGAGGGGCGTTTGG 18 (0.000541%)

GTGAAAAGAAAGATGAATCCTAGGGCTCAGAGCACTGCAGCAGATCATTTCATATTGCTTCCGTGGAGT
GTGGCGAGTCAGCTAAATACTTTGACGCCGG 14 (0.000421%)

GTGAAGCCAGCTGCTTCCATTGGTGGGTCATTTTTGCTGTCACCAGCAACGTTGCCACGACGAACATCCT
TGACAGACACATTCTTGACATTGAAGCCCA 18 (0.000541%)

GTGACCTTGCCAGCTCCAGCAGCCTTCTTGTCCACTGCTTTGATGACACCCACCGCAACTGTCTGTCTCAT
ATCACGAACAGCAAAGCGACCCAAAGGTG 14 (0.000421%)

GTGACTTCATATGAGATTGTTTGGGCTACTGCTCGCAGTGCGCCGATCAGGGCGTAGTTTGAGTTTGATG
CTCACCCTGATCAGAGGATTGAGTAAACGG 16 (0.000481%)

GTGATCGGCGATCAGAGGGCGATGAAGTTCTAGATCCATTGAGACAAGCTCTAGACAGTAGCATGCAGT
CCCACAACTTGTACCAGCATCCCCAGCGTCT 6 (0.000180%)

GTGATGGACACCAGTTTTAGCCAACATAGCATAGTACTCTATTTCAGATTTCCTCAAAGCTGGGCAGTTG
TTAGCGAGAATGACCAATTTCGCTTTGCCT 6 (0.000180%)

GTGCATGAGTAGGTGGCCTGCAGTAATGTTAGCGGTTAGGCGTACGGCCAGGGCTATTGGTTGAATGAG
TAGGCTGATGGTTTCGATAATAACTAGTATG 11 (0.000331%)

GTGCTGACTTCCTTAACAATTTCCTCATATCTCTTCTGGCTGTAGGGTGGCTCAGTGGAATCCATTTTGTT
AACACCGACAATTAGTTGTTTCACACCCA 32 (0.000962%)

GTGGAGTCCGTAAAGAGGTATCTTTACTATAAAAGCTATTGTGTAAGCTAGTCATATTAAGTTGTTGGCT
CAGGAGTTTGATAGTTCTTGGGCAGTGAGA 16 (0.000481%)

GTGGCAGGTATTAGGGATAATATTCATTTAGCCTTCTGAGCTTTCTGGGCAGACTTGGTGACCTTGCCAG
CTCCAGCAGCCTTCTTGTCCACTGCTTTGA3 (0.000090%)

GTGGCCTGCAGTAATGTTAGCGGTTAGGCGTACGGCCAGGGCTATTGGTTGAATGAGTAGGCTGATGGT
TTCGATAATAACTAGTATGGGGATAAGGGGT 15 (0.000451%)

GTGGCCTTGGTATGTGCTTTCTCGTGTTACATCGCGCCATCATTGGTATATGGTTAGTGTGTTGGTTAGTA
GGCCTAGTATGAGGAGCGTTATGGAGTGG93 (0.002796%)

GTGGCGCTTCCAATTAGGTGCATGAGTAGGTGGCCTGCAGTAATGTTAGCGGTTAGGCGTACGGCCAGG
GCTATTGGTTGAATGAGTAGGCTGATGGTTT 6 (0.000180%)

GTGGCTACAAAAAATGTTGAGCCGTAGATGCCGTCGGAAATGGTGAAGGGAGACTCGAAGTACTCTGAG
GCTTGTAGGAGGGTAAAATAGAGACCCAGTA 14 (0.000421%)

GTGGCTCAGTGGAATCCATTTTGTTAACACCGACAATTAGTTGTTTCACACCCAGTGTGTAAGCCAGAAG
GGCATGCTCTCGGGTCTGCCCATTCTTGGA 16 (0.000481%)

GTGGGAAGAAAGTTAGATTTACGCCGATGAATATGATAGTGAAATGGATTTTGGCGTAGGTTTGGTCTA
GGGTGTAGCCTGAGAATAGGGGAAATCAGTG 413 (0.012415%)

GTGGGGTGGCAGGTATTAGGGATAATATTCATTTAGCCTTCTGAGCTTTCTGGGCAGACTTGGTGACCTT
GCCAGCTCCAGCAGCCTTCTTGTCCACTGC 6 (0.000180%)

GTGGGTCATTTTTGCTGTCACCAGCAACGTTGCCACGACGAACATCCTTGACAGACACATTCTTGACATT
GAAGCCCACATTGTCCCCAGGAAGAGCTTC 7 (0.000210%)

GTGGTAGGATGCAGTCCAGAGCCTCAAGCAGCGTGGTTCCACTGGCATTGCCATCCTTACGGGTGACTTT
CCATCCCTTGAACCAAGGCATGTTAGCACT 42 (0.001263%)

GTGGTAGTCCCCTTTCATTTTATAATAGAAAACCTTGGACTCGCCAGTGTTAGCTGCTGGAATGAGGTGT
TTGTCCAGTACATCCAGAATGTCACAACAG 6 (0.000180%)

GTGGTCAGGTCCCGGTATTCCCGGTACATGTTGTGGGTGCCGCTCCGGGAGTCATAGCGCAGCCAGATCC
CGAAGTTCTTCACCCGCAGGGGGGACTTCT 5 (0.000150%)

GTGGTTGGTGTAAATGAGTGAGGCAGGAGTCCGAGGAGGTTAGTTGTGGCAATAAAAATGATTAAGGAT
ACTAGTATAAGAGATCAGGTTCGTCCTTTAG 5 (0.000150%)

GTGTAAATGAGTGAGGCAGGAGTCCGAGGAGGTTAGTTGTGGCAATAAAAATGATTAAGGATACTAGTA
TAAGAGATCAGGTTCGTCCTTTAGTGTTGTG 44 (0.001323%)

GTGTAAGCCAGAAGGGCATGCTCTCGGGTCTGCCCATTCTTGGAGATACCAGCTTCAAATTCACCAACAC
CAGCAGCAACAATCAGGACAGCACAGTCAG 7 (0.000210%)

GTGTGTAAGCCAGAAGGGCATGCTCTCGGGTCTGCCCATTCTTGGAGATACCAGCTTCAAATTCACCAAC
ACCAGCAGCAACAATCAGGACAGCACAGTC 4 (0.000120%)

GTGTTACTTTAATTGGACTGCCTTCGTAATTCATTGCCTCTGCTTCAACAATGTGCAACTCATCCTTTGCA
CCAGCCCCTAAACTGACCGTTCTTAAAGA 3 (0.000090%)

GTTAGATTTACGCCGATGAATATGATAGTGAAATGGATTTTGGCGTAGGTTTGGTCTAGGGTGTAGCCTG
AGAATAGGGGAAATCAGTGAATGAAGCCTC 48 (0.001443%)

GTTAGCGGTTAGGCGTACGGCCAGGGCTATTGGTTGAATGAGTAGGCTGATGGTTTCGATAATAACTAGT
ATGGGGATAAGGGGTGTAGGTGTGCCTTGT 13 (0.000391%)

GTTAGGCGTACGGCCAGGGCTATTGGTTGAATGAGTAGGCTGATGGTTTCGATAATAACTAGTATGGGG

ATAAGGGGTGTAGGTGTGCCTTGTGGTAAGA 28 (0.000842%)

GTTAGGGGGTCGGAGGAAAAGGTTGGGGAACAGCTAAATAGGTTGTTGTTGATTTGGTTAAAAAATAGT
AGAGGGATGATGCTAATAATTAGGCTGTGGG 13 (0.000391%)

GTTAGTATTAGGAGGGGGGTTGTTAGGGGGTCGGAGGAAAAGGTTGGGGAACAGCTAAATAGGTTGTTG
TTGATTTGGTTAAAAAATAGTAGAGGGATGA 13 (0.000391%)

GTTCAGTGTTTTAATCTGACGCAGGCTTATGCGGAGGAGAATGTTTTCATGTTACTTATACTAACATTAGT
TCTTCTATAGGGTGATAGATTGGTCCAAT 7 (0.000210%)

GTTCTTCCACCACTGATTAAGAGTGGGGTGGCAGGTATTAGGGATAATATTCATTTAGCCTTCTGAGCTT
TCTGGGCAGACTTGGTGACCTTGCCAGCTC 30 (0.000902%)

GTTGAACCCAGGTACCTTTCTCTTTGGCTTCTTTCTTTTTCTGATCATTTTCCTTCACACGTTTCAGGAAGC
TATCTCGGCTCTTAGAGTGCTTAATGTG 204 (0.006133%)

GTTGAATGAGTAGGCTGATGGTTTCGATAATAACTAGTATGGGGATAAGGGGTGTAGGTGTGCCTTGTG
GTAAGAAGTGGGCTAGGGCATTTTTAATCTT 19 (0.000571%)

GTTGATATTGCTAGGGTGGCGCTTCCAATTAGGTGCATGAGTAGGTGGCCTGCAGTAATGTTAGCGGTTA
GGCGTACGGCCAGGGCTATTGGTTGAATGA 649 (0.019510%)

GTTGCAAATTCTCATGGTTTGGGTTGGGTGGTGGAGAGCGCGTGTCATCTGCGGGTGGCACTGCCCACGG
TGGGCGGGCGGGCCTCTCTACTCGAAGGTG 6 (0.000180%)

GTTGGACGAGTTGGTGGTAGGATGCAGTCCAGAGCCTCAAGCAGCGTGGTTCCACTGGCATTGCCATCCT
TACGGGTGACTTTCCATCCCTTGAACCAAG 18 (0.000541%)

GTTGGGGAACAGCTAAATAGGTTGTTGTTGATTTGGTTAAAAAATAGTAGAGGGATGATGCTAATAATT
AGGCTGTGGGTGGTTGTGTTGATTCAAATTA 4 (0.000120%)

GTTGGGTGGTGGAGAGCGCGTGTCATCTGCGGGTGGCACTGCCCACGGTGGGCGGGCGGGCCTCTCTAC
TCGAAGGTGACCACGTTTAGATTCTGAGACG 8 (0.000240%)

GTTGGGTGGTTGGTGTAAATGAGTGAGGCAGGAGTCCGAGGAGGTTAGTTGTGGCAATAAAAATGATTA
AGGATACTAGTATAAGAGATCAGGTTCGTCC 14 (0.000421%)

GTTGGTGGTAGGATGCAGTCCAGAGCCTCAAGCAGCGTGGTTCCACTGGCATTGCCATCCTTACGGGTGA
CTTTCCATCCCTTGAACCAAGGCATGTTAG 22 (0.000661%)

GTTGGTGTAAATGAGTGAGGCAGGAGTCCGAGGAGGTTAGTTGTGGCAATAAAAATGATTAAGGATACT
AGTATAAGAGATCAGGTTCGTCCTTTAGTGT 10 (0.000301%)

GTTGTAGCCAATTTTCTTAATGTAAGTGCTGACTTCCTTAACAATTTCCTCATATCTCTTCTGGCTGTAGG
GTGGCTCAGTGGAATCCATTTTGTTAACA 1127 (0.033879%)

GTTGTTAGGGGGTCGGAGGAAAAGGTTGGGGAACAGCTAAATAGGTTGTTGTTGATTTGGTTAAAAAAT
AGTAGAGGGATGATGCTAATAATTAGGCTGT 7 (0.000210%)

GTTGTTTCACACCCAGTGTGTAAGCCAGAAGGGCATGCTCTCGGGTCTGCCCATTCTTGGAGATACCAGC
TTCAAATTCACCAACACCAGCAGCAACAAT 572 (0.017195%)

GTTTATAGATAGTTGGGTGGTTGGTGTAAATGAGTGAGGCAGGAGTCCGAGGAGGTTAGTTGTGGCAAT
AAAAATGATTAAGGATACTAGTATAAGAGAT 84 (0.002525%)

GTTTCACACCCAGTGTGTAAGCCAGAAGGGCATGCTCTCGGGTCTGCCCATTCTTGGAGATACCAGCTTC
AAATTCACCAACACCAGCAGCAACAATCAG 10 (0.000301%)

GTTTGCTAATACAATGCCAGTCAGGCCACCTACGGTGAAAAGAAAGATGAATCCTAGGGCTCAGAGCAC
TGCAGCAGATCATTTCATATTGCTTCCGTGG 525 (0.015782%)

GTTTGGGTTGGGTGGTGGAGAGCGCGTGTCATCTGCGGGTGGCACTGCCCACGGTGGGCGGGCGGGCCT
CTCTACTCGAAGGTGACCACGTTTAGATTCT 30 (0.000902%)

GTTTTAATCTGACGCAGGCTTATGCGGAGGAGAATGTTTTCATGTTACTTATACTAACATTAGTTCTTCTA
TAGGGTGATAGATTGGTCCAATTGGGTGT 6 (0.000180%)

GTTTTAGCCAACATAGCATAGTACTCTATTTCAGATTTCCTCAAAGCTGGGCAGTTGTTAGCGAGAATGA
CCAATTTCGCTTTGCCTTGTCTGATCATCT 5 (0.000150%)

GTTTTCTATTAGACTATGGTGAGCTCAGGTGATTGATACTCCTGATGCGAGTAATACGGATGTGTTTAGG
AGTGGGACTTCTAGGGGATTTAGCGGGGTG 72 (0.002164%)

GTTTTTAAGCCTAATGTGGGGACAGCTCATGAGTGCAAGACGTCTTGTGATGTAATTATTATACGAATGG
GGGCTTCAATCGGGAGTACTACTCGATTGT 16 (0.000481%)

GTTTTTGATAAAGTCTCTGTGTCCTGGGGCATCAATGATAGTCACATAGTACTTGCTGGTCTCAAATTTCC
ACAAGGAGATATCAATGGTGATACCACGT41 (0.001233%)

TAAAAACTAATAACTTAAAACTGCCACACGCAAAAAAGAAAACCAAAGTGGTCCACAAAACATTCTCCT
TTCCTTCTGAAGGTTTTACGATGCATTGTTA 17 (0.000511%)

TAAAAAGTACTGATTTTAAAAACTAATAACTTAAAACTGCCACACGCAAAAAAGAAAACCAAAGTGGTC
CACAAAACATTCTCCTTTCCTTCTGAAGGTT 19 (0.000571%)

TAAAATAGAGACCCAGTAAAATTGTAATAAGCAGTGCTTGAATTATTTGGTTTCGGTTGTTTTCTATTAG
ACTATGGTGAGCTCAGGTGATTGATACTCC 8 (0.000240%)

TAAAATTGTAATAAGCAGTGCTTGAATTATTTGGTTTCGGTTGTTTTCTATTAGACTATGGTGAGCTCAGG
TGATTGATACTCCTGATGCGAGTAATACG 17 (0.000511%)

TAAAGAGGTATCTTTACTATAAAAGCTATTGTGTAAGCTAGTCATATTAAGTTGTTGGCTCAGGAGTTTG
ATAGTTCTTGGGCAGTGAGAGTGAGTAGTA 10 (0.000301%)

TAAATGAGTGAGGCAGGAGTCCGAGGAGGTTAGTTGTGGCAATAAAAATGATTAAGGATACTAGTATAA
GAGATCAGGTTCGTCCTTTAGTGTTGTGTAT 8 (0.000240%)

TAACAATTTCCTCATATCTCTTCTGGCTGTAGGGTGGCTCAGTGGAATCCATTTTGTTAACACCGACAATT
AGTTGTTTCACACCCAGTGTGTAAGCCAG 14 (0.000421%)

TAAGAGTGGGGTGGCAGGTATTAGGGATAATATTCATTTAGCCTTCTGAGCTTTCTGGGCAGACTTGGTG
ACCTTGCCAGCTCCAGCAGCCTTCTTGTCC 4 (0.000120%)

TAAGATAGAGGAGACACCTGCTAGGTGTAAGGAGAAGATGGTTAGGTCTACGGAGGCTCCAGGGTGGG
AGTAGTTCCCTGCTAAGGGAGGGTAGACTGTT 3 (0.000090%)

TAAGCCTAATGTGGGGACAGCTCATGAGTGCAAGACGTCTTGTGATGTAATTATTATACGAATGGGGGC
TTCAATCGGGAGTACTACTCGATTGTCAACG 3 (0.000090%)

TAAGGAGAAGATGGTTAGGTCTACGGAGGCTCCAGGGTGGGAGTAGTTCCCTGCTAAGGGAGGGTAGAC
TGTTCAACCTGTTCCTGCTCCGGCCTCCACT 3 (0.000090%)

TAAGTGCTGACTTCCTTAACAATTTCCTCATATCTCTTCTGGCTGTAGGGTGGCTCAGTGGAATCCATTTT
GTTAACACCGACAATTAGTTGTTTCACAC 7 (0.000210%)

TAATAACTTAAAACTGCCACACGCAAAAAAGAAAACCAAAGTGGTCCACAAAACATTCTCCTTTCCTTCT
GAAGGTTTTACGATGCATTGTTATCATTAA 12 (0.000361%)

TAATACAATGCCAGTCAGGCCACCTACGGTGAAAAGAAAGATGAATCCTAGGGCTCAGAGCACTGCAGC AGATCATTTCATATTGCTTCCGTGGAGTGTG 6 (0.000180%)

TAATATTCATTTAGCCTTCTGAGCTTTCTGGGCAGACTTGGTGACCTTGCCAGCTCCAGCAGCCTTCTTGT CCACTGCTTTGATGACACCCACCGCAACT 22 (0.000661%)

TAATCACCTGAGCAGTGAAGCCAGCTGCTTCCATTGGTGGGTCATTTTTGCTGTCACCAGCAACGTTGCC ACGACGAACATCCTTGACAGACACATTCTT 8 (0.000240%)

TAATCATGTTTTTGATAAAGTCTCTGTGTCCTGGGGCATCAATGATAGTCACATAGTACTTGCTGGTCTCA AATTTCCACAAGGAGATATCAATGGTGAT 5 (0.000150%)

TACCTTTCTCTTTGGCTTCTTTCTTTTTCTGATCATTTTCCTTCACACGTTTCAGGAAGCTATCTCGGCTCTT AGAGTGCTTAATGTGCTCAATACGCAC 4 (0.000120%)

TACGAATGGGGGCTTCAATCGGGAGTACTACTCGATTGTCAACGTCAAGGAGTCGCAGGTCGCCTGGTT CTAGGAATAATGGGGGAAGTATGTAGGAGTT 8 (0.000240%)

TACTACTCGATTGTCAACGTCAAGGAGTCGCAGGTCGCCTGGTTCTAGGAATAATGGGGGAAGTATGTA GGAGTTGAAGATTAGTCCGCCGTAGTCGGTG 3 (0.000090%)

TACTGATTTTAAAAACTAATAACTTAAAACTGCCACACGCAAAAAAGAAAACCAAAGTGGTCCACAAAA CATTCTCCTTTCCTTCTGAAGGTTTTACGAT 6 (0.000180%)

TAGACATCCTGGAGAGGCAGGCGCAAGGGCTTGTCAGTTGGACGAGTTGGTGGTAGGATGCAGTCCAGA GCCTCAAGCAGCGTGGTTCCACTGGCATTGC 9 (0.000271%)

TAGAGACCCAGTAAAATTGTAATAAGCAGTGCTTGAATTATTTGGTTTCGGTTGTTTTCTATTAGACTATG GTGAGCTCAGGTGATTGATACTCCTGATG 28 (0.000842%)

TAGAGGAGACACCTGCTAGGTGTAAGGAGAAGATGGTTAGGTCTACGGAGGCTCCAGGGTGGGAGTAG TTCCCTGCTAAGGGAGGGTAGACTGTTCAACC 3 (0.000090%)

TAGATAGTTGGGTGGTTGGTGTAAATGAGTGAGGCAGGAGTCCGAGGAGGTTAGTTGTGGCAATAAAAA TGATTAAGGATACTAGTATAAGAGATCAGGT 5 (0.000150%)

TAGATTTACGCCGATGAATATGATAGTGAAATGGATTTTGGCGTAGGTTTGGTCTAGGGTGTAGCCTGAG AATAGGGGAAATCAGTGAATGAAGCCTCCT 11 (0.000331%)

TAGCCAATTTTCTTAATGTAAGTGCTGACTTCCTTAACAATTTCCTCATATCTCTTCTGGCTGTAGGGTGG CTCAGTGGAATCCATTTTGTTAACACCGA 3 (0.000090%)

TAGCCTTCTGAGCTTTCTGGGCAGACTTGGTGACCTTGCCAGCTCCAGCAGCCTTCTTGTCCACTGCTTTG ATGACACCCACCGCAACTGTCTGTCTCAT 18 (0.000541%)

TAGCGGTTAGGCGTACGGCCAGGGCTATTGGTTGAATGAGTAGGCTGATGGTTTCGATAATAACTAGTAT GGGGATAAGGGGTGTAGGTGTGCCTTGTGG 10 (0.000301%)

TAGGATGCAGTCCAGAGCCTCAAGCAGCGTGGTTCCACTGGCATTGCCATCCTTACGGGTGACTTTCCAT CCCTTGAACCAAGGCATGTTAGCACTTGGC 6 (0.000180%)

TAGGCGTACGGCCAGGGCTATTGGTTGAATGAGTAGGCTGATGGTTTCGATAATAACTAGTATGGGGAT AAGGGGTGTAGGTGTGCCTTGTGGTAAGAAG 5 (0.000150%)

TAGGGAGTCATAAGTGGAGTCCGTAAAGAGGTATCTTTACTATAAAAGCTATTGTGTAAGCTAGTCATAT TAAGTTGTTGGCTCAGGAGTTTGATAGTTC 3 (0.000090%)

TAGGGATAATATTCATTTAGCCTTCTGAGCTTTCTGGGCAGACTTGGTGACCTTGCCAGCTCCAGCAGCC

TTCTTGTCCACTGCTTTGATGACACCCACC30 (0.000902%)

TAGGGATAGTACAAGGAAGGGGTAGGCTATGTGTTTTGTCAGGGGGTTGAGAATGAGTGTGAGGCGTAT
TATACCATAGCCGCCTAGTTTTAAGAGTACT 3 (0.000090%)

TAGGGCTCAGAGCACTGCAGCAGATCATTTCATATTGCTTCCGTGGAGTGTGGCGAGTCAGCTAAATACT
TTGACGCCGGTGGGGATAGCGATGATTATG 17 (0.000511%)

TAGGGGGTCGGAGGAAAAGGTTGGGGAACAGCTAAATAGGTTGTTGTTGATTTGGTTAAAAAATAGTAG
AGGGATGATGCTAATAATTAGGCTGTGGGTG 7 (0.000210%)

TAGGTGGCCTGCAGTAATGTTAGCGGTTAGGCGTACGGCCAGGGCTATTGGTTGAATGAGTAGGCTGAT
GGTTTCGATAATAACTAGTATGGGGATAAGG 6 (0.000180%)

TAGGTTTATAGATAGTTGGGTGGTTGGTGTAAATGAGTGAGGCAGGAGTCCGAGGAGGTTAGTTGTGGC
AATAAAAATGATTAAGGATACTAGTATAAGA 23 (0.000691%)

TAGTACAAGGAAGGGGTAGGCTATGTGTTTTGTCAGGGGGTTGAGAATGAGTGTGAGGCGTATTATACC
ATAGCCGCCTAGTTTTAAGAGTACTGCGGCA 3 (0.000090%)

TAGTGATGGACACCAGTTTTAGCCAACATAGCATAGTACTCTATTTCAGATTTCCTCAAAGCTGGGCAGT
TGTTAGCGAGAATGACCAATTTCGCTTTGC 3 (0.000090%)

TATACGAATGGGGGCTTCAATCGGGAGTACTACTCGATTGTCAACGTCAAGGAGTCGCAGGTCGCCTGG
TTCTAGGAATAATGGGGGAAGTATGTAGGAG 7 (0.000210%)

TATAGATAGTTGGGTGGTTGGTGTAAATGAGTGAGGCAGGAGTCCGAGGAGGTTAGTTGTGGCAATAAA
AATGATTAAGGATACTAGTATAAGAGATCAG 22 (0.000661%)

TATCCAAGACCCAGGCATACTTGAAGGAGCCCTTTCCCATCTCAGCAGCCTCCTTCTCAAATTTTTCAAT
GGTTCTTTTGTCGATGCCACCGCATTTATA3 (0.000090%)

TATTAGACTATGGTGAGCTCAGGTGATTGATACTCCTGATGCGAGTAATACGGATGTGTTTAGGAGTGGG
ACTTCTAGGGGATTTAGCGGGGTGATGCCT 7 (0.000210%)

TATTAGGGATAATATTCATTTAGCCTTCTGAGCTTTCTGGGCAGACTTGGTGACCTTGCCAGCTCCAGCA
GCCTTCTTGTCCACTGCTTTGATGACACCC3 (0.000090%)

TATTATACGAATGGGGGCTTCAATCGGGAGTACTACTCGATTGTCAACGTCAAGGAGTCGCAGGTCGCCT
GGTTCTAGGAATAATGGGGGAAGTATGTAG 4 (0.000120%)

TATTCATTTAGCCTTCTGAGCTTTCTGGGCAGACTTGGTGACCTTGCCAGCTCCAGCAGCCTTCTTGTCCA
CTGCTTTGATGACACCCACCGCAACTGTC 34 (0.001022%)

TATTGCTAGGGTGGCGCTTCCAATTAGGTGCATGAGTAGGTGGCCTGCAGTAATGTTAGCGGTTAGGCGT
ACGGCCAGGGCTATTGGTTGAATGAGTAGG 7 (0.000210%)

TATTGGTTGAATGAGTAGGCTGATGGTTTCGATAATAACTAGTATGGGGATAAGGGGTGTAGGTGTGCCT
TGTGGTAAGAAGTGGGCTAGGGCATTTTTA 22 (0.000661%)

TATTTGTCTAAAACATGTCTTCTTTGTAGCAGCTAGGCCCTGCCACCACTGTGCTTGGCTGAGTTCACAAA
TCTGTTGTAACCTGTAGCTTCCCTGTCAC 3 (0.000090%)

TCAACACACATGGGCTTGCCAGGAACCATATCAACAATGGCAGCATCACCAGACTTCAAGAATTTAGGG
CCATCTTCCAGCTTTTTACCAGAACGGCGAT 8 (0.000240%)

TCAAGCAGCGTGGTTCCACTGGCATTGCCATCCTTACGGGTGACTTTCCATCCCTTGAACCAAGGCATGT
TAGCACTTGGCTCCAGCATGTTGTCACCAT 3 (0.000090%)

TCAATCGGGAGTACTACTCGATTGTCAACGTCAAGGAGTCGCAGGTCGCCTGGTTCTAGGAATAATGGG
GGAAGTATGTAGGAGTTGAAGATTAGTCCGC 7 (0.000210%)

TCACTCAAAGCTTCATGGTGCATTTCGACAGATTTTACTTCCGTTGTAACGTTGACTGGAGCAAAGGTGA
CCACCATACCGGGTTTGAGAACACCAGTCT 3 (0.000090%)

TCAGAGCACTGCAGCAGATCATTTCATATTGCTTCCGTGGAGTGTGGCGAGTCAGCTAAATACTTTGACG
CCGGTGGGGATAGCGATGATTATGGTAGCG 4 (0.000120%)

TCAGTGGAATCCATTTTGTTAACACCGACAATTAGTTGTTTCACACCCAGTGTGTAAGCCAGAAGGGCAT
GCTCTCGGGTCTGCCCATTCTTGGAGATAC 4 (0.000120%)

TCATAGGGATAGTACAAGGAAGGGGTAGGCTATGTGTTTTGTCAGGGGGTTGAGAATGAGTGTGAGGCG
TATTATACCATAGCCGCCTAGTTTTAAGAGT 4 (0.000120%)

TCATGTTTTTGATAAAGTCTCTGTGTCCTGGGGCATCAATGATAGTCACATAGTACTTGCTGGTCTCAAAT
TTCCACAAGGAGATATCAATGGTGATACC 3 (0.000090%)

TCCAATGATGGTAAAAGGGTAGCTTACTGGTTGTCCTCCGATTCAGGTTAGAATGAGGAGGTCTGCGGCT
AGGAGTCAATAAAGTGATTGGCTTAGTGGG 3 (0.000090%)

TCCACCACTGATTAAGAGTGGGGTGGCAGGTATTAGGGATAATATTCATTTAGCCTTCTGAGCTTTCTGG
GCAGACTTGGTGACCTTGCCAGCTCCAGCA 3 (0.000090%)

TCCAGAACTACTGCCTTCACCATGAAGCTCCATGAGCTTTCCCAATTCAAACTTGGGCTTCTTCAGCATTT
TTACTTTTCTAACGAAGACATCATGGAGA 382 (0.011483%)

TCCATTAAAAAGTACTGATTTTAAAAACTAATAACTTAAAACTGCCACACGCAAAAAAGAAAACCAAAG
TGGTCCACAAAACATTCTCCTTTCCTTCTGA 14 (0.000421%)

TCCCCAGGAAGAGCTTCACTCAAAGCTTCATGGTGCATTTCGACAGATTTTACTTCCGTTGTAACGTTGA
CTGGAGCAAAGGTGACCACCATACCGGGTT 3 (0.000090%)

TCCCCTGTTGCAAATTCTCATGGTTTGGGTTGGGTGGTGGAGAGCGCGTGTCATCTGCGGGTGGCACTGC
CCACGGTGGGCGGGCGGGCCTCTCTACTCG 3 (0.000090%)

TCCCGGTATTCCCGGTACATGTTGTGGGTGCCGCTCCGGGAGTCATAGCGCAGCCAGATCCCGAAGTTCT
TCACCCGCAGGGGGGACTTCTCAAACACCT 5 (0.000150%)

TCCGAAGCCTGGTAGGATAAGAATATAAACTTCAGGGTGACCGAAAAATCAGAATAGGTGTTGGTATAG
AATGGGGTCTCCTCCTCCGGCGGGGTCGAAG 667 (0.020051%)

TCCTCATATCTCTTCTGGCTGTAGGGTGGCTCAGTGGAATCCATTTTGTTAACACCGACAATTAGTTGTTT
CACACCCAGTGTGTAAGCCAGAAGGGCAT 18 (0.000541%)

TCCTCTCCTGCTAAGCTTTGTTTCCTAATTAAAATCTTCTGCCACTGCCATAGCTACTGCTGCTGCTGGAA
CCGCCATAGCCACCTTGGTTTCGTGGTTT 252 (0.007575%)

TCCTGGAGAGGCAGGCGCAAGGGCTTGTCAGTTGGACGAGTTGGTGGTAGGATGCAGTCCAGAGCCTCA
AGCAGCGTGGTTCCACTGGCATTGCCATCCT 5 (0.000150%)

TCCTTAACAATTTCCTCATATCTCTTCTGGCTGTAGGGTGGCTCAGTGGAATCCATTTTGTTAACACCGAC
AATTAGTTGTTTCACACCCAGTGTGTAAG 5 (0.000150%)

TCGAAGTACTCTGAGGCTTGTAGGAGGGTAAAATAGAGACCCAGTAAAATTGTAATAAGCAGTGCTTGA
ATTATTTGGTTTCGGTTGTTTTCTATTAGAC 5 (0.000150%)

TCGACATGGGCTTTAGGGAGTCATAAGTGGAGTCCGTAAAGAGGTATCTTTACTATAAAAGCTATTGTGT
AAGCTAGTCATATTAAGTTGTTGGCTCAGG 9 (0.000271%)

TCTCAACACACATGGGCTTGCCAGGAACCATATCAACAATGGCAGCATCACCAGACTTCAAGAATTTAG
GGCCATCTTCCAGCTTTTTACCAGAACGGCG 13 (0.000391%)

TCTCGGGTCTGCCCATTCTTGGAGATACCAGCTTCAAATTCACCAACACCAGCAGCAACAATCAGGACA
GCACAGTCAGCCTGAGATGTCCCTGTAATCA 50 (0.001503%)

TCTGAGCTTTCTGGGCAGACTTGGTGACCTTGCCAGCTCCAGCAGCCTTCTTGTCCACTGCTTTGATGACA
CCCACCGCAACTGTCTGTCTCATATCACG 6 (0.000180%)

TCTGGCTGTAGGGTGGCTCAGTGGAATCCATTTTGTTAACACCGACAATTAGTTGTTTCACACCCAGTGT
GTAAGCCAGAAGGGCATGCTCTCGGGTCTG 31 (0.000932%)

TCTGGGCAGACTTGGTGACCTTGCCAGCTCCAGCAGCCTTCTTGTCCACTGCTTTGATGACACCCACCGC
AACTGTCTGTCTCATATCACGAACAGCAAA 6 (0.000180%)

TCTGTGACAAATTTTTGGTCAAGTTGTTTCCATTAAAAAGTACTGATTTTAAAAACTAATAACTTAAAAC
TGCCACACGCAAAAAAGAAAACCAAAGTGG 18 (0.000541%)

TCTGTTAGTAGTATAGTGATGCCAGCAGCTAGGACTGGGAGAGATAGGAGAAGTAGGACTGCTGTGATT
AGGACGGATCAGACGAAGAGGGGCGTTTGGT 7 (0.000210%)

TCTGTTTTTAAGCCTAATGTGGGGACAGCTCATGAGTGCAAGACGTCTTGTGATGTAATTATTATACGAA
TGGGGGCTTCAATCGGGAGTACTACTCGAT 3 (0.000090%)

TCTTAATGTAAGTGCTGACTTCCTTAACAATTTCCTCATATCTCTTCTGGCTGTAGGGTGGCTCAGTGGAA
TCCATTTTGTTAACACCGACAATTAGTTG 24 (0.000721%)

TCTTCCACCACTGATTAAGAGTGGGGTGGCAGGTATTAGGGATAATATTCATTTAGCCTTCTGAGCTTTC
TGGGCAGACTTGGTGACCTTGCCAGCTCCA 15 (0.000451%)

TGAAAAGAAAGATGAATCCTAGGGCTCAGAGCACTGCAGCAGATCATTTCATATTGCTTCCGTGGAGTG
TGGCGAGTCAGCTAAATACTTTGACGCCGGT 13 (0.000391%)

TGAAATTGATGGCCCCTAAGATAGAGGAGACACCTGCTAGGTGTAAGGAGAAGATGGTTAGGTCTACGG
AGGCTCCAGGGTGGGAGTAGTTCCCTGCTAA 22 (0.000661%)

TGAAGCCAGCTGCTTCCATTGGTGGGTCATTTTTGCTGTCACCAGCAACGTTGCCACGACGAACATCCTT
GACAGACACATTCTTGACATTGAAGCCCAC 7 (0.000210%)

TGAATCCTAGGGCTCAGAGCACTGCAGCAGATCATTTCATATTGCTTCCGTGGAGTGTGGCGAGTCAGCT
AAATACTTTGACGCCGGTGGGGATAGCGAT 4 (0.000120%)

TGAATGAGTAGGCTGATGGTTTCGATAATAACTAGTATGGGGATAAGGGGTGTAGGTGTGCCTTGTGGT
AAGAAGTGGGCTAGGGCATTTTTAATCTTAG 35 (0.001052%)

TGACCTTGCCAGCTCCAGCAGCCTTCTTGTCCACTGCTTTGATGACACCCACCGCAACTGTCTGTCTCATA
TCACGAACAGCAAAGCGACCCAAAGGTGG 6 (0.000180%)

TGACTTCATATGAGATTGTTTGGGCTACTGCTCGCAGTGCGCCGATCAGGGCGTAGTTTGAGTTTGATGC
TCACCCTGATCAGAGGATTGAGTAAACGGC 4 (0.000120%)

TGACTTCCTTAACAATTTCCTCATATCTCTTCTGGCTGTAGGGTGGCTCAGTGGAATCCATTTTGTTAACA
CCGACAATTAGTTGTTTCACACCCAGTGT 21 (0.000631%)

TGAGAAGCTCTCAACACACATGGGCTTGCCAGGAACCATATCAACAATGGCAGCATCACCAGACTTCAA
GAATTTAGGGCCATCTTCCAGCTTTTTACCA 31 (0.000932%)

TGAGACCGTTCTTCCACCACTGATTAAGAGTGGGGTGGCAGGTATTAGGGATAATATTCATTTAGCCTTC

TGAGCTTTCTGGGCAGACTTGGTGACCTTG 938 (0.028198%)

TGAGCAGTGAAGCCAGCTGCTTCCATTGGTGGGTCATTTTGCTGTCACCAGCAACGTTGCCACGACGAA
CATCCTTGACAGACACATTCTTGACATTGA 7 (0.000210%)

TGAGCTTTCTGGGCAGACTTGGTGACCTTGCCAGCTCCAGCAGCCTTCTTGTCCACTGCTTTGATGACACC
CACCGCAACTGTCTGTCTCATATCACGAA 22 (0.000661%)

TGAGTATTTGTCTAAAACATGTCTTCTTTGTAGCAGCTAGGCCCTGCCACCACTGTGCTTGGCTGAGTTCA
CAAATCTGTTGTAACCTGTAGCTTCCCTG 10 (0.000301%)

TGAGTGAGGCAGGAGTCCGAGGAGGTTAGTTGTGGCAATAAAAATGATTAAGGATACTAGTATAAGAG
ATCAGGTTCGTCCTTTAGTGTTGTGTATGGTT 6 (0.000180%)

TGATATTGCTAGGGTGGCGCTTCCAATTAGGTGCATGAGTAGGTGGCCTGCAGTAATGTTAGCGGTTAGG
CGTACGGCCAGGGCTATTGGTTGAATGAGT 27 (0.000812%)

TGATGGCCCCTAAGATAGAGGAGACACCTGCTAGGTGTAAGGAGAAGATGGTTAGGTCTACGGAGGCTC
CAGGGTGGGAGTAGTTCCCTGCTAAGGGAGG 7 (0.000210%)

TGATGGCTAGGGTGACTTCATATGAGATTGTTTGGGCTACTGCTCGCAGTGCGCCGATCAGGGCGTAGTT
TGAGTTTGATGCTCACCCTGATCAGAGGAT 7 (0.000210%)

TGATTAAGAGTGGGGTGGCAGGTATTAGGGATAATATTCATTTAGCCTTCTGAGCTTTCTGGGCAGACTT
GGTGACCTTGCCAGCTCCAGCAGCCTTCTT 4 (0.000120%)

TGATTTTAAAAACTAATAACTTAAAACTGCCACACGCAAAAAAGAAAACCAAAGTGGTCCACAAAACAT
TCTCCTTTCCTTCTGAAGGTTTTACGATGCA 38 (0.001142%)

TGCAAATTCTCATGGTTTGGGTTGGGTGGTGGAGAGCGCGTGTCATCTGCGGGTGGCACTGCCCACGGTG
GGCGGGCGGGCCTCTCTACTCGAAGGTGAC 32 (0.000962%)

TGCACACTCCTTTGCTACTGGTCCTGTAATGGCAGAACCTTTCATCTCGCCTTTATTGTTCACTATGACTC
CTGCATTATCTTCAAAATAAAGAAACACG 4 (0.000120%)

TGCAGCAGATCATTTCATATTGCTTCCGTGGAGTGTGGCGAGTCAGCTAAATACTTTGACGCCGGTGGGG
ATAGCGATGATTATGGTAGCGGAGGTGAAA 4 (0.000120%)

TGCAGTAATGTTAGCGGTTAGGCGTACGGCCAGGGCTATTGGTTGAATGAGTAGGCTGATGGTTTCGATA
ATAACTAGTATGGGGATAAGGGGTGTAGGT 13 (0.000391%)

TGCAGTCCAGAGCCTCAAGCAGCGTGGTTCCACTGGCATTGCCATCCTTACGGGTGACTTTCCATCCCTT
GAACCAAGGCATGTTAGCACTTGGCTCCAG 12 (0.000361%)

TGCATCTGTTTTTAAGCCTAATGTGGGGACAGCTCATGAGTGCAAGACGTCTTGTGATGTAATTATTATA
CGAATGGGGGCTTCAATCGGGAGTACTACT 10 (0.000301%)

TGCATGAGTAGGTGGCCTGCAGTAATGTTAGCGGTTAGGCGTACGGCCAGGGCTATTGGTTGAATGAGT
AGGCTGATGGTTTCGATAATAACTAGTATGG 37 (0.001112%)

TGCCACACGCAAAAAAGAAAACCAAAGTGGTCCACAAAACATTCTCCTTTCCTTCTGAAGGTTTTACGAT
GCATTGTTATCATTAACCAGTCTTTTACTA 37 (0.001112%)

TGCCAGCTCCAGCAGCCTTCTTGTCCACTGCTTTGATGACACCCACCGCAACTGTCTGTCTCATATCACGA
ACAGCAAAGCGACCCAAAGGTGGATAGTC 6 (0.000180%)

TGCCAGTCAGGCCACCTACGGTGAAAAGAAAGATGAATCCTAGGGCTCAGAGCACTGCAGCAGATCATT
TCATATTGCTTCCGTGGAGTGTGGCGAGTCA 10 (0.000301%)

TGCCATCCTTACGGGTGACTTTCCATCCCTTGAACCAAGGCATGTTAGCACTTGGCTCCAGCATGTTGTC
ACCATTCCAACCAGAAATTGGCACAAATGC 10 (0.000301%)

TGCCCATACACATGAGTATTTGTCTAAAACATGTCTTCTTTGTAGCAGCTAGGCCCTGCCACCACTGTGCT
TGGCTGAGTTCACAAATCTGTTGTAACCT 8 (0.000240%)

TGCTAATACAATGCCAGTCAGGCCACCTACGGTGAAAAGAAAGATGAATCCTAGGGCTCAGAGCACTGC
AGCAGATCATTTCATATTGCTTCCGTGGAGT 38 (0.001142%)

TGCTACTTGTCCAATGATGGTAAAAGGGTAGCTTACTGGTTGTCCTCCGATTCAGGTTAGAATGAGGAGG
TCTGCGGCTAGGAGTCAATAAAGTGATTGG 5 (0.000150%)

TGCTAGGGTGGCGCTTCCAATTAGGTGCATGAGTAGGTGGCCTGCAGTAATGTTAGCGGTTAGGCGTAC
GGCCAGGGCTATTGGTTGAATGAGTAGGCTG 4 (0.000120%)

TGCTGACTTCCTTAACAATTTCCTCATATCTCTTCTGGCTGTAGGGTGGCTCAGTGGAATCCATTTGTTA
ACACCGACAATTAGTTGTTTCACACCCAG 32 (0.000962%)

TGCTTCCATTGGTGGGTCATTTTTGCTGTCACCAGCAACGTTGCCACGACGAACATCCTTGACAGACACA
TTCTTGACATTGAAGCCCACATTGTCCCCA 6 (0.000180%)

TGGACACCAGTTTTAGCCAACATAGCATAGTACTCTATTTCAGATTTCCTCAAAGCTGGGCAGTTGTTAG
CGAGAATGACCAATTTCGCTTTGCCTTGTC 16 (0.000481%)

TGGACGAGTTGGTGGTAGGATGCAGTCCAGAGCCTCAAGCAGCGTGGTTCCACTGGCATTGCCATCCTTA
CGGGTGACTTTCCATCCCTTGAACCAAGGC 28 (0.000842%)

TGGAGAGGCAGGCGCAAGGGCTTGTCAGTTGGACGAGTTGGTGGTAGGATGCAGTCCAGAGCCTCAAGC
AGCGTGGTTCCACTGGCATTGCCATCCTTAC 28 (0.000842%)

TGGAGTGGTAAAAGGCTCAGAAAAATCCTGCGAAGAAAAAAACTTCTGAGGTAATAAATAGGATTATCC
CGTATCGAAGGCCTTTTTGGACAGGTGGTGT 23 (0.000691%)

TGGCAGGTATTAGGGATAATATTCATTTAGCCTTCTGAGCTTTCTGGGCAGACTTGGTGACCTTGCCAGC
TCCAGCAGCCTTCTTGTCCACTGCTTTGAT 7 (0.000210%)

TGGCATTGCCATCCTTACGGGTGACTTTCCATCCCTTGAACCAAGGCATGTTAGCACTTGGCTCCAGCAT
GTTGTCACCATTCCAACCAGAAATTGGCAC 241 (0.007245%)

TGGCGCTTCCAATTAGGTGCATGAGTAGGTGGCCTGCAGTAATGTTAGCGGTTAGGCGTACGGCCAGGG
CTATTGGTTGAATGAGTAGGCTGATGGTTTC 10 (0.000301%)

TGGCTAGGTTTATAGATAGTTGGGTGGTTGGTGTAAATGAGTGAGGCAGGAGTCCGAGGAGGTTAGTTG
TGGCAATAAAAATGATTAAGGATACTAGTAT 10 (0.000301%)

TGGCTGTAGGGTGGCTCAGTGGAATCCATTTGTTAACACCGACAATTAGTTGTTTCACACCCAGTGTGT
AAGCCAGAAGGGCATGCTCTCGGGTCTGCC 13 (0.000391%)

TGGGAAGAAAGTTAGATTTACGCCGATGAATATGATAGTGAAATGGATTTTGGCGTAGGTTTGGTCTAG
GGTGTAGCCTGAGAATAGGGGAAATCAGTGA 10 (0.000301%)

TGGGCAGACTTGGTGACCTTGCCAGCTCCAGCAGCCTTCTTGTCCACTGCTTTGATGACACCCACCGCAA
CTGTCTGTCTCATATCACGAACAGCAAAGC 3 (0.000090%)

TGGGCTTTAGGGAGTCATAAGTGGAGTCCGTAAAGAGGTATCTTTACTATAAAAGCTATTGTGTAAGCTA
GTCATATTAAGTTGTTGGCTCAGGAGTTTG 10 (0.000301%)

TGGGGAACAGCTAAATAGGTTGTTGTTGATTTGGTTAAAAAATAGTAGAGGGATGATGCTAATAATTAG
GCTGTGGGTGGTTGTGTTGATTCAAATTATG 4 (0.000120%)

TGGGGGCTTCAATCGGGAGTACTACTCGATTGTCAACGTCAAGGAGTCGCAGGTCGCCTGGTTCTAGGA
ATAATGGGGGAAGTATGTAGGAGTTGAAGAT 6 (0.000180%)

TGGGGTGGCAGGTATTAGGGATAATATTCATTTAGCCTTCTGAGCTTTCTGGGCAGACTTGGTGACCTTG
CCAGCTCCAGCAGCCTTCTTGTCCACTGCT 6 (0.000180%)

TGGGTCATTTTTGCTGTCACCAGCAACGTTGCCACGACGAACATCCTTGACAGACACATTCTTGACATTG
AAGCCCACATTGTCCCCAGGAAGAGCTTCA 8 (0.000240%)

TGGGTGGTTGGTGTAAATGAGTGAGGCAGGAGTCCGAGGAGGTTAGTTGTGGCAATAAAAATGATTAAG
GATACTAGTATAAGAGATCAGGTTCGTCCTT 16 (0.000481%)

TGGTAGGATGCAGTCCAGAGCCTCAAGCAGCGTGGTTCCACTGGCATTGCCATCCTTACGGGTGACTTTC
CATCCCTTGAACCAAGGCATGTTAGCACTT 35 (0.001052%)

TGGTAGTCCCCTTTCATTTTATAATAGAAAACCTTGGACTCGCCAGTGTTAGCTGCTGGAATGAGGTGTT
TGTCCAGTACATCCAGAATGTCACAACAGA 3 (0.000090%)

TGGTGACCTTGCCAGCTCCAGCAGCCTTCTTGTCCACTGCTTTGATGACACCCACCGCAACTGTCTGTCTC
ATATCACGAACAGCAAAGCGACCCAAAGG 15 (0.000451%)

TGGTGGGTCATTTTTGCTGTCACCAGCAACGTTGCCACGACGAACATCCTTGACAGACACATTCTTGACA
TTGAAGCCCACATTGTCCCCAGGAAGAGCT 21 (0.000631%)

TGGTGGTAGGATGCAGTCCAGAGCCTCAAGCAGCGTGGTTCCACTGGCATTGCCATCCTTACGGGTGACT
TTCCATCCCTTGAACCAAGGCATGTTAGCA 11 (0.000331%)

TGGTTGAATGAGTAGGCTGATGGTTTCGATAATAACTAGTATGGGGATAAGGGGTGTAGGTGTGCCTTGT
GGTAAGAAGTGGGCTAGGGCATTTTTAATC 3 (0.000090%)

TGGTTTGGGTTGGGTGGTGGAGAGCGCGTGTCATCTGCGGGTGGCACTGCCCACGGTGGGCGGGCGGGC
CTCTCTACTCGAAGGTGACCACGTTTAGATT 5 (0.000150%)

TGTAAATGAGTGAGGCAGGAGTCCGAGGAGGTTAGTTGTGGCAATAAAAATGATTAAGGATACTAGTAT
AAGAGATCAGGTTCGTCCTTTAGTGTTGTGT 14 (0.000421%)

TGTAAGGAGAAGATGGTTAGGTCTACGGAGGCTCCAGGGTGGGAGTAGTTCCCTGCTAAGGGAGGGTAG
ACTGTTCAACCTGTTCCTGCTCCGGCCTCCA 3 (0.000090%)

TGTAAGTGCTGACTTCCTTAACAATTTCCTCATATCTCTTCTGGCTGTAGGGTGGCTCAGTGGAATCCATT
TTGTTAACACCGACAATTAGTTGTTTCAC 4 (0.000120%)

TGTAATCATGTTTTTGATAAAGTCTCTGTGTCCTGGGGCATCAATGATAGTCACATAGTACTTGCTGGTCT
CAAATTTCCACAAGGAGATATCAATGGTG 363 (0.010912%)

TGTAGACATCCTGGAGAGGCAGGCGCAAGGGCTTGTCAGTTGGACGAGTTGGTGGTAGGATGCAGTCCA
GAGCCTCAAGCAGCGTGGTTCCACTGGCATT 18 (0.000541%)

TGTAGAGGGAGTATAGGGCTGTGACTAGTATGTTGAGTCCTGTAAGTAGGAGAGTGATATTTGATCAGG
AGAACGTGGTTACTAGCACAGAGAGTTCTCC 354 (0.010642%)

TGTAGCCAATTTTCTTAATGTAAGTGCTGACTTCCTTAACAATTTCCTCATATCTCTTCTGGCTGTAGGGT
GGCTCAGTGGAATCCATTTTGTTAACACC 22 (0.000661%)

TGTAGGGTGGCTCAGTGGAATCCATTTTGTTAACACCGACAATTAGTTGTTTCACACCCAGTGTGTAAGC
CAGAAGGGCATGCTCTCGGGTCTGCCCATT 17 (0.000511%)

TGTCAGTTGGACGAGTTGGTGGTAGGATGCAGTCCAGAGCCTCAAGCAGCGTGGTTCCACTGGCATTGC

CATCCTTACGGGTGACTTTCCATCCCTTGAA 6 (0.000180%)

TGTGACAAATTTTTGGTCAAGTTGTTTCCATTAAAAAGTACTGATTTTAAAAACTAATAACTTAAAACTG
CCACACGCAAAAAAGAAAACCAAAGTGGTC 6 (0.000180%)

TGTGGCTACAAAAAATGTTGAGCCGTAGATGCCGTCGGAAATGGTGAAGGGAGACTCGAAGTACTCTGA
GGCTTGTAGGAGGGTAAAATAGAGACCCAGT 514 (0.015452%)

TGTTACTTTAATTGGACTGCCTTCGTAATTCATTGCCTCTGCTTCAACAATGTGCAACTCATCCTTTGCAC
CAGCCCCTAAACTGACCGTTCTTAAAGAT 6 (0.000180%)

TGTTGCAAATTCTCATGGTTTGGGTTGGGTGGTGGAGAGCGCGTGTCATCTGCGGGTGGCACTGCCCACG
GTGGGCGGGCGGGCCTCTCTACTCGAAGGT 4 (0.000120%)

TGTTTTTAAGCCTAATGTGGGGACAGCTCATGAGTGCAAGACGTCTTGTGATGTAATTATTATACGAATG
GGGGCTTCAATCGGGAGTACTACTCGATTG 11 (0.000331%)

TTAAAAACTAATAACTTAAAACTGCCACACGCAAAAAAGAAAACCAAAGTGGTCCACAAAACATTCTCC
TTTCCTTCTGAAGGTTTTACGATGCATTGTT 55 (0.001653%)

TTAAAAAGTACTGATTTTAAAAACTAATAACTTAAAACTGCCACACGCAAAAAAGAAAACCAAAGTGGT
CCACAAAACATTCTCCTTTCCTTCTGAAGGT 32 (0.000962%)

TTAACAATTTCCTCATATCTCTTCTGGCTGTAGGGTGGCTCAGTGGAATCCATTTTGTTAACACCGACAAT
TAGTTGTTTCACACCCAGTGTGTAAGCCA 6 (0.000180%)

TTACTTTAATTGGACTGCCTTCGTAATTCATTGCCTCTGCTTCAACAATGTGCAACTCATCCTTTGCACCA
GCCCCTAAACTGACCGTTCTTAAAGATAA 3 (0.000090%)

TTAGATTTACGCCGATGAATATGATAGTGAAATGGATTTTGGCGTAGGTTTGGTCTAGGGTGTAGCCTGA
GAATAGGGGAAATCAGTGAATGAAGCCTCC 3 (0.000090%)

TTAGCCTTCTGAGCTTTCTGGGCAGACTTGGTGACCTTGCCAGCTCCAGCAGCCTTCTTGTCCACTGCTTT
GATGACACCCACCGCAACTGTCTGTCTCA 46 (0.001383%)

TTAGCGGTTAGGCGTACGGCCAGGGCTATTGGTTGAATGAGTAGGCTGATGGTTTCGATAATAACTAGTA
TGGGGATAAGGGGTGTAGGTGTGCCTTGTG 5 (0.000150%)

TTAGGGAGTCATAAGTGGAGTCCGTAAAGAGGTATCTTTACTATAAAAGCTATTGTGTAAGCTAGTCATA
TTAAGTTGTTGGCTCAGGAGTTTGATAGTT 4 (0.000120%)

TTAGGGATAATATTCATTTAGCCTTCTGAGCTTTCTGGGCAGACTTGGTGACCTTGCCAGCTCCAGCAGC
CTTCTTGTCCACTGCTTTGATGACACCCAC3 (0.000090%)

TTAGGTGCATGAGTAGGTGGCCTGCAGTAATGTTAGCGGTTAGGCGTACGGCCAGGGCTATTGGTTGAA
TGAGTAGGCTGATGGTTTCGATAATAACTAG 3 (0.000090%)

TTAGTATTAGGAGGGGGGTTGTTAGGGGGTCGGAGGAAAAGGTTGGGGAACAGCTAAATAGGTTGTTGT
TGATTTGGTTAAAAAATAGTAGAGGGATGAT 6 (0.000180%)

TTATACGAATGGGGGCTTCAATCGGGAGTACTACTCGATTGTCAACGTCAAGGAGTCGCAGGTCGCCTG
GTTCTAGGAATAATGGGGGAAGTATGTAGGA 6 (0.000180%)

TTATAGATAGTTGGGTGGTTGGTGTAAATGAGTGAGGCAGGAGTCCGAGGAGGTTAGTTGTGGCAATAA
AAATGATTAAGGATACTAGTATAAGAGATCA 18 (0.000541%)

TTATCCAAGACCCAGGCATACTTGAAGGAGCCCTTTCCCATCTCAGCAGCCTCCTTCTCAAATTTTTCAAT
GGTTCTTTTGTCGATGCCACCGCATTTAT 415 (0.012475%)

TTATTATACGAATGGGGGCTTCAATCGGGAGTACTACTCGATTGTCAACGTCAAGGAGTCGCAGGTCGCC TGGTTCTAGGAATAATGGGGGAAGTATGTA 4 (0.000120%)

TTCACACCCAGTGTGTAAGCCAGAAGGGCATGCTCTCGGGTCTGCCCATTCTTGGAGATACCAGCTTCAA ATTCACCAACACCAGCAGCAACAATCAGGA 16 (0.000481%)

TTCACTCAAAGCTTCATGGTGCATTTCGACAGATTTTACTTCCGTTGTAACGTTGACTGGAGCAAAGGTG ACCACCATACCGGGTTTGAGAACACCAGTC 8 (0.000240%)

TTCATTTAGCCTTCTGAGCTTTCTGGGCAGACTTGGTGACCTTGCCAGCTCCAGCAGCCTTCTTGTCCACT GCTTTGATGACACCCACCGCAACTGTCTG 19 (0.000571%)

TTCCACCACTGATTAAGAGTGGGGTGGCAGGTATTAGGGATAATATTCATTTAGCCTTCTGAGCTTTCTG GGCAGACTTGGTGACCTTGCCAGCTCCAGC 13 (0.000391%)

TTCCATTAAAAAGTACTGATTTTAAAAACTAATAACTTAAAACTGCCACACGCAAAAAAGAAAACCAAA GTGGTCCACAAAACATTCTCCTTTCCTTCTG 63 (0.001894%)

TTCCATTGGTGGGTCATTTTTGCTGTCACCAGCAACGTTGCCACGACGAACATCCTTGACAGACACATTC TTGACATTGAAGCCCACATTGTCCCCAGGA 14 (0.000421%)

TTCCTCATATCTCTTCTGGCTGTAGGGTGGCTCAGTGGAATCCATTTTGTTAACACCGACAATTAGTTGTT TCACACCCAGTGTGTAAGCCAGAAGGGCA 21 (0.000631%)

TTCCTCCCCTGTTGCAAATTCTCATGGTTTGGGTTGGGTGGTGGAGAGCGCGTGTCATCTGCGGGTGGCA CTGCCCACGGTGGGCGGGCGGGCCTCTCTA 4 (0.000120%)

TTCCTTAACAATTTCCTCATATCTCTTCTGGCTGTAGGGTGGCTCAGTGGAATCCATTTTGTTAACACCGA CAATTAGTTGTTTCACACCCAGTGTGTAA 21 (0.000631%)

TTCGACATGGGCTTTAGGGAGTCATAAGTGGAGTCCGTAAAGAGGTATCTTTACTATAAAAGCTATTGTG TAAGCTAGTCATATTAAGTTGTTGGCTCAG 389 (0.011694%)

TTCGGTTGTTTTCTATTAGACTATGGTGAGCTCAGGTGATTGATACTCCTGATGCGAGTAATACGGATGT GTTTAGGAGTGGGACTTCTAGGGGATTTAG 92 (0.002766%)

TTCTATTAGACTATGGTGAGCTCAGGTGATTGATACTCCTGATGCGAGTAATACGGATGTGTTTAGGAGT GGGACTTCTAGGGGATTTAGCGGGGTGATG 12 (0.000361%)

TTCTGAGCTTTCTGGGCAGACTTGGTGACCTTGCCAGCTCCAGCAGCCTTCTTGTCCACTGCTTTGATGAC ACCCACCGCAACTGTCTGTCTCATATCAC 18 (0.000541%)

TTCTGGCTGTAGGGTGGCTCAGTGGAATCCATTTTGTTAACACCGACAATTAGTTGTTTCACACCCAGTG TGTAAGCCAGAAGGGCATGCTCTCGGGTCT 13 (0.000391%)

TTCTGGGCAGACTTGGTGACCTTGCCAGCTCCAGCAGCCTTCTTGTCCACTGCTTTGATGACACCCACCG CAACTGTCTGTCTCATATCACGAACAGCAA 10 (0.000301%)

TTCTGTGACAAATTTTTGGTCAAGTTGTTTCCATTAAAAAGTACTGATTTTAAAAACTAATAACTTAAAA CTGCCACACGCAAAAAAGAAAACCAAAGTG 6 (0.000180%)

TTCTTAATGTAAGTGCTGACTTCCTTAACAATTTCCTCATATCTCTTCTGGCTGTAGGGTGGCTCAGTGGA ATCCATTTTGTTAACACCGACAATTAGTT 7 (0.000210%)

TTGATATTGCTAGGGTGGCGCTTCCAATTAGGTGCATGAGTAGGTGGCCTGCAGTAATGTTAGCGGTTAG GCGTACGGCCAGGGCTATTGGTTGAATGAG 6 (0.000180%)

TTGATGACACCCACCGCAACTGTCTGTCTCATATCACGAACAGCAAAGCGACCCAAAGGTGGATAGTCT GAGAAGCTCTCAACACACATGGGCTTGCCAG 4 (0.000120%)

TTGATGGCCCCTAAGATAGAGGAGACACCTGCTAGGTGTAAGGAGAAGATGGTTAGGTCTACGGAGGCT
CCAGGGTGGGAGTAGTTCCCTGCTAAGGGAG 5 (0.000150%)

TTGCAAATTCTCATGGTTTGGGTTGGGTGGTGGAGAGCGCGTGTCATCTGCGGGTGGCACTGCCCACGGT
GGGCGGGCGGGCCTCTCTACTCGAAGGTGA 3 (0.000090%)

TTGCCAGCTCCAGCAGCCTTCTTGTCCACTGCTTTGATGACACCCACCGCAACTGTCTGTCTCATATCACG
AACAGCAAAGCGACCCAAAGGTGGATAGT 6 (0.000180%)

TTGCCATCCTTACGGGTGACTTTCCATCCCTTGAACCAAGGCATGTTAGCACTTGGCTCCAGCATGTTGTC
ACCATTCCAACCAGAAATTGGCACAAATG3 (0.000090%)

TTGCCCATACACATGAGTATTTGTCTAAAACATGTCTTCTTTGTAGCAGCTAGGCCCTGCCACCACTGTGC
TTGGCTGAGTTCACAAATCTGTTGTAACC 6 (0.000180%)

TTGCTAATACAATGCCAGTCAGGCCACCTACGGTGAAAAGAAAGATGAATCCTAGGGCTCAGAGCACTG
CAGCAGATCATTTCATATTGCTTCCGTGGAG 8 (0.000240%)

TTGGGGAACAGCTAAATAGGTTGTTGTTGATTTGGTTAAAAAATAGTAGAGGGATGATGCTAATAATTA
GGCTGTGGGTGGTTGTGTTGATTCAAATTAT 5 (0.000150%)

TTGGGTTGGGTGGTGGAGAGCGCGTGTCATCTGCGGGTGGCACTGCCCACGGTGGGCGGGCGGGCCTCT
CTACTCGAAGGTGACCACGTTTAGATTCTGA 7 (0.000210%)

TTGGTGACCTTGCCAGCTCCAGCAGCCTTCTTGTCCACTGCTTTGATGACACCCACCGCAACTGTCTGTCT
CATATCACGAACAGCAAAGCGACCCAAAG 7 (0.000210%)

TTGGTGGGTCATTTTTGCTGTCACCAGCAACGTTGCCACGACGAACATCCTTGACAGACACATTCTTGAC
ATTGAAGCCCACATTGTCCCCAGGAAGAGC 3 (0.000090%)

TTGGTGTAAATGAGTGAGGCAGGAGTCCGAGGAGGTTAGTTGTGGCAATAAAAATGATTAAGGATACTA
GTATAAGAGATCAGGTTCGTCCTTTAGTGTT 5 (0.000150%)

TTGGTTGAATGAGTAGGCTGATGGTTTCGATAATAACTAGTATGGGGATAAGGGGTGTAGGTGTGCCTTG
TGGTAAGAAGTGGGCTAGGGCATTTTTAAT 5 (0.000150%)

TTGTAATAAGCAGTGCTTGAATTATTTGGTTTCGGTTGTTTTCTATTAGACTATGGTGAGCTCAGGTGATT
GATACTCCTGATGCGAGTAATACGGATGT 3 (0.000090%)

TTGTAGACATCCTGGAGAGGCAGGCGCAAGGGCTTGTCAGTTGGACGAGTTGGTGGTAGGATGCAGTCC
AGAGCCTCAAGCAGCGTGGTTCCACTGGCAT 11 (0.000331%)

TTGTAGCCAATTTTCTTAATGTAAGTGCTGACTTCCTTAACAATTTCCTCATATCTCTTCTGGCTGTAGGG
TGGCTCAGTGGAATCCATTTTGTTAACAC 6 (0.000180%)

TTGTCAGTTGGACGAGTTGGTGGTAGGATGCAGTCCAGAGCCTCAAGCAGCGTGGTTCCACTGGCATTGC
CATCCTTACGGGTGACTTTCCATCCCTTGA 7 (0.000210%)

TTGTCCCCAGGAAGAGCTTCACTCAAAGCTTCATGGTGCATTTCGACAGATTTTACTTCCGTTGTAACGTT
GACTGGAGCAAAGGTGACCACCATACCGG 3 (0.000090%)

TTGTTTCACACCCAGTGTGTAAGCCAGAAGGGCATGCTCTCGGGTCTGCCCATTCTTGGAGATACCAGCT
TCAAATTCACCAACACCAGCAGCAACAATC 5 (0.000150%)

TTGTTTTCTATTAGACTATGGTGAGCTCAGGTGATTGATACTCCTGATGCGAGTAATACGGATGTGTTTA
GGAGTGGGACTTCTAGGGGATTTAGCGGGG 89 (0.002675%)

TTTAAAAACTAATAACTTAAAACTGCCACACGCAAAAAAGAAAACCAAAGTGGTCCACAAAACATTCTC

CTTTCCTTCTGAAGGTTTTACGATGCATTGT 41 (0.001233%)

TTTAAGCCTAATGTGGGGACAGCTCATGAGTGCAAGACGTCTTGTGATGTAATTATTATACGAATGGGGG
CTTCAATCGGGAGTACTACTCGATTGTCAA 5 (0.000150%)

TTTAATCTGACGCAGGCTTATGCGGAGGAGAATGTTTTCATGTTACTTATACTAACATTAGTTCTTCTATA
GGGTGATAGATTGGTCCAATTGGGTGTGA 3 (0.000090%)

TTTAATTGGACTGCCTTCGTAATTCATTGCCTCTGCTTCAACAATGTGCAACTCATCCTTTGCACCAGCCC
CTAAACTGACCGTTCTTAAAGATAACTGG 4 (0.000120%)

TTTACGCCGATGAATATGATAGTGAAATGGATTTTGGCGTAGGTTTGGTCTAGGGTGTAGCCTGAGAATA
GGGGAAATCAGTGAATGAAGCCTCCTATGA 5 (0.000150%)

TTTAGCCTTCTGAGCTTTCTGGGCAGACTTGGTGACCTTGCCAGCTCCAGCAGCCTTCTTGTCCACTGCTT
TGATGACACCCACCGCAACTGTCTGTCTC 22 (0.000661%)

TTTAGGGAGTCATAAGTGGAGTCCGTAAAGAGGTATCTTTACTATAAAAGCTATTGTGTAAGCTAGTCAT
ATTAAGTTGTTGGCTCAGGAGTTTGATAGT 10 (0.000301%)

TTTATAGATAGTTGGGTGGTTGGTGTAAATGAGTGAGGCAGGAGTCCGAGGAGGTTAGTTGTGGCAATA
AAAATGATTAAGGATACTAGTATAAGAGATC 50 (0.001503%)

TTTCACACCCAGTGTGTAAGCCAGAAGGGCATGCTCTCGGGTCTGCCCATTCTTGGAGATACCAGCTTCA
AATTCACCAACACCAGCAGCAACAATCAGG 5 (0.000150%)

TTTCCATTAAAAAGTACTGATTTTAAAAACTAATAACTTAAAACTGCCACACGCAAAAAAGAAAACCAA
AGTGGTCCACAAAACATTCTCCTTTCCTTCT 399 (0.011994%)

TTTCCTCATATCTCTTCTGGCTGTAGGGTGGCTCAGTGGAATCCATTTTGTTAACACCGACAATTAGTTGT
TTCACACCCAGTGTGTAAGCCAGAAGGGC12 (0.000361%)

TTTCTATTAGACTATGGTGAGCTCAGGTGATTGATACTCCTGATGCGAGTAATACGGATGTGTTTAGGAG
TGGGACTTCTAGGGGATTTAGCGGGGTGAT 38 (0.001142%)

TTTCTGGGCAGACTTGGTGACCTTGCCAGCTCCAGCAGCCTTCTTGTCCACTGCTTTGATGACACCCACCG
CAACTGTCTGTCTCATATCACGAACAGCA 9 (0.000271%)

TTTCTTAATGTAAGTGCTGACTTCCTTAACAATTTCCTCATATCTCTTCTGGCTGTAGGGTGGCTCAGTGG
AATCCATTTTGTTAACACCGACAATTAGT 10 (0.000301%)

TTTGATGACACCCACCGCAACTGTCTGTCTCATATCACGAACAGCAAAGCGACCCAAAGGTGGATAGTC
TGAGAAGCTCTCAACACACATGGGCTTGCCA 30 (0.000902%)

TTTGCTAATACAATGCCAGTCAGGCCACCTACGGTGAAAAGAAAGATGAATCCTAGGGCTCAGAGCACT
GCAGCAGATCATTTCATATTGCTTCCGTGGA 16 (0.000481%)

TTTGCTACTGGTCCTGTAATGGCAGAACCTTTCATCTCGCCTTTATTGTTCACTATGACTCCTGCATTATCT
TCAAAATAAAGAAACACGCCATCTTTTC 3 (0.000090%)

TTTGGACAGGTGGTGTGTGGTGGCCTTGGTATGTGCTTTCTCGTGTTACATCGCGCCATCATTGGTATATG
GTTAGTGTGTTGGTTAGTAGGCCTAGTAT 9 (0.000271%)

TTTGGGTTGGGTGGTGGAGAGCGCGTGTCATCTGCGGGTGGCACTGCCCACGGTGGGCGGGCGGGCCTC
TCTACTCGAAGGTGACCACGTTTAGATTCTG 10 (0.000301%)

TTTGTAGACATCCTGGAGAGGCAGGCGCAAGGGCTTGTCAGTTGGACGAGTTGGTGGTAGGATGCAGTC
CAGAGCCTCAAGCAGCGTGGTTCCACTGGCA 23 (0.000691%)

TTTGTCTAAAACATGTCTTCTTTGTAGCAGCTAGGCCCTGCCACCACTGTGCTTGGCTGAGTTCACAAATC
TGTTGTAACCTGTAGCTTCCCTGTCACTT 5 (0.000150%)

TTTTAAAAACTAATAACTTAAAACTGCCACACGCAAAAAAGAAAACCAAAGTGGTCCACAAAACATTCT
CCTTTCCTTCTGAAGGTTTTACGATGCATTG 7 (0.000210%)

TTTTAAGCCTAATGTGGGGACAGCTCATGAGTGCAAGACGTCTTGTGATGTAATTATTATACGAATGGGG
GCTTCAATCGGGAGTACTACTCGATTGTCA 21 (0.000631%)

TTTTAATCTGACGCAGGCTTATGCGGAGGAGAATGTTTTCATGTTACTTATACTAACATTAGTTCTTCTAT
AGGGTGATAGATTGGTCCAATTGGGTGTG 10 (0.000301%)

TTTTCCTCCCCTGTTGCAAATTCTCATGGTTTGGGTTGGGTGGTGGAGAGCGCGTGTCATCTGCGGGTGG
CACTGCCCACGGTGGGCGGGCGGGCCTCTC 4 (0.000120%)

TTTTCTTAATGTAAGTGCTGACTTCCTTAACAATTTCCTCATATCTCTTCTGGCTGTAGGGTGGCTCAGTG
GAATCCATTTTGTTAACACCGACAATTAG 23 (0.000691%)

TTTTGCCCATACACATGAGTATTTGTCTAAAACATGTCTTCTTTGTAGCAGCTAGGCCCTGCCACCACTGT
GCTTGGCTGAGTTCACAAATCTGTTGTAA 4 (0.000120%)

TTTTGCTGTCACCAGCAACGTTGCCACGACGAACATCCTTGACAGACACATTCTTGACATTGAAGCCCAC
ATTGTCCCCAGGAAGAGCTTCACTCAAAGC 80 (0.002405%)

TTTTGGACAGGTGGTGTGTGGTGGCCTTGGTATGTGCTTTCTCGTGTTACATCGCGCCATCATTGGTATAT
GGTTAGTGTGTTGGTTAGTAGGCCTAGTA 23 (0.000691%)

TTTTGTAGACATCCTGGAGAGGCAGGCGCAAGGGCTTGTCAGTTGGACGAGTTGGTGGTAGGATGCAGT
CCAGAGCCTCAAGCAGCGTGGTTCCACTGGC 4 (0.000120%)

TTTTTCCTCCCCTGTTGCAAATTCTCATGGTTTGGGTTGGGTGGTGGAGAGCGCGTGTCATCTGCGGGTGG
CACTGCCCACGGTGGGCGGGCGGGCCTCT 473 (0.014219%)

TTTTTGATAAAGTCTCTGTGTCCTGGGGCATCAATGATAGTCACATAGTACTTGCTGGTCTCAAATTTCCA
CAAGGAGATATCAATGGTGATACCACGTT 5 (0.000150%)

TTTTTGCCCATACACATGAGTATTTGTCTAAAACATGTCTTCTTTGTAGCAGCTAGGCCCTGCCACCACTG
TGCTTGGCTGAGTTCACAAATCTGTTGTA 7 (0.000210%)

TTTTTGCTGTCACCAGCAACGTTGCCACGACGAACATCCTTGACAGACACATTCTTGACATTGAAGCCCA
CATTGTCCCCAGGAAGAGCTTCACTCAAAG 14 (0.000421%)

TTTTTGGACAGGTGGTGTGTGGTGGCCTTGGTATGTGCTTTCTCGTGTTACATCGCGCCATCATTGGTATA
TGGTTAGTGTGTTGGTTAGTAGGCCTAGT 346 (0.010401%)

TTTTTTGCCCATACACATGAGTATTTGTCTAAAACATGTCTTCTTTGTAGCAGCTAGGCCCTGCCACCACT
GTGCTTGGCTGAGTTCACAAATCTGTTGT 300 (0.009018%)

TTTTTTTTTTTTTTTTTTTTTTTTTTTTTTTTTTTTTTTTTTTTTTTTTTTTTTTTTTTTTTTTTTTTTTTTTTTTTT
TTTTTTTTTTTTTTTTTTTTTT 1151 (0.034601%)

Before filtering: read2: quality
Value of each position will be shown on mouse over.
2040608010012014037.53838.53939.5

positionqualityATCGmean

<https://plot.ly/>
Before filtering: read2: base contents

Value of each position will be shown on mouse over.
2040608010012014000.10.20.30.40.50.6

positionbase content ratiosA(26.79%)T(25.58%)C(23.50%)G(24.03%)N(0.076%)GC(47.54%)

<https://plot.ly/>
Before filtering: read2: KMER counting
Darker background means larger counts. The count will be shown on mouse over.

AA AT AC AG TA TT TC TG CA CT CC CG GA GT GC GG
AAA AAAAA AAAAT AAAAC AAAAG AAATA AAATT AAATC AAATG AAACA AAACT AAACC
AAACG AAAGA AAAGT AAAGC AAAGG
AAT AATAA AATAT AATAC AATAG AATTA AATTT AATTC AATTG AATCA AATCT AATCC
AATCGAATGA AATGT AATGC AATGG
AAC AACAA AACAT AACAC AACAG AACTA AACTT AACTC AACTG AACCA AACCT AACCC
AACCG AACGA AACGT AACGC AACGG
AAG AAGAA AAGAT AAGAC AAGAG AAGTA AAGTT AAGTC AAGTG AAGCA AAGCT AAGCC
AAGCG AAGGA AAGGT AAGGC AAGGG
ATA ATAAA ATAAT ATAAC ATAAG ATATA ATATT ATATC ATATG ATACA ATACT ATACC
ATACGATAGA ATAGT ATAGC ATAGG
ATT ATTAA ATTAT ATTAC ATTAG ATTTA ATTTT ATTTC ATTTG ATTCA ATTCT ATTCC
ATTCG ATTGA ATTGT ATTGC ATTGG
ATC ATCAA ATCAT ATCAC ATCAG ATCTA ATCTT ATCTC ATCTG ATCCA ATCCT ATCCC
ATCCG ATCGA ATCGT ATCGC ATCGG
ATG ATGAA ATGAT ATGAC ATGAG ATGTA ATGTT ATGTC ATGTG ATGCA ATGCT ATGCC
ATGCGATGGA ATGGT ATGGC ATGGG
ACA ACAAA ACAAT ACAAC ACAAG ACATA ACATT ACATC ACATG ACACA ACACT ACACC
ACACG ACAGA ACAGT ACAGC ACAGG
ACT ACTAA ACTAT ACTAC ACTAG ACTTA ACTTT ACTTC ACTTG ACTCA ACTCT ACTCC
ACTCGACTGA ACTGT ACTGC ACTGG
ACC ACCAA ACCAT ACCAC ACCAG ACCTA ACCTT ACCTC ACCTG ACCCA ACCCT ACCCC
ACCCGACCGA ACCGT ACCGC ACCGG
ACG ACGAA ACGAT ACGAC ACGAG ACGTA ACGTT ACGTC ACGTG ACGCA ACGCT ACGCC
ACGCG ACGGA ACGGT ACGGC ACGGG
AGA AGAAA AGAAT AGAAC AGAAG AGATA AGATT AGATC AGATG AGACA AGACT AGACC
AGACG AGAGA AGAGT AGAGC AGAGG
AGT AGTAA AGTAT AGTAC AGTAG AGTTA AGTTT AGTTC AGTTG AGTCA AGTCT AGTCC
AGTCGAGTGA AGTGT AGTGC AGTGG
AGC AGCAA AGCAT AGCAC AGCAG AGCTA AGCTT AGCTC AGCTG AGCCA AGCCT AGCCC
AGCCG AGCGA AGCGT AGCGC AGCGG
AGG AGGAA AGGAT AGGAC AGGAG AGGTA AGGTT AGGTC AGGTG AGGCA AGGCT AGGCC
AGGCG AGGGA AGGGT AGGGC AGGGG
TAA TAAAA TAAAT TAAAC TAAAG TAATA TAATT TAATC TAATG TAACA TAACT TAACC
TAACGTAAGA TAAGT TAAGC TAAGG
TAT TATAA TATAT TATAC TATAG TATTA TATTT TATTC TATTG TATCA TATCT TATCC
TATCG TATGA TATGT TATGC TATGG
TAC TACAA TACAT TACAC TACAG TACTA TACTT TACTC TACTG TACCA TACCT TACCC
TACCG TACGA TACGT TACGC TACGG
TAG TAGAA TAGAT TAGAC TAGAG TAGTA TAGTT TAGTC TAGTG TAGCA TAGCT TAGCC
TAGCGTAGGA TAGGT TAGGC TAGGG
TTA TTAAA TTAAT TTAAC TTAAG TTATA TTATT TTATC TTATG TTACA TTACT TTACC
TTACG TTAGA TTAGT TTAGC TTAGG
TTT TTTAA TTTAT TTTAC TTTAG TTTTA TTTTT TTTTC TTTTG TTTCA TTTCT TTTCC
TTTCG TTTGA TTTGT TTTGC TTTGG
TTC TTCAA TTCAT TTCAC TTCAG TTCTA TTCTT TTCTC TTCTG TTCCA TTCCT TTCCC
TTCCG TTCGA TTCGT TTCGC TTCGG
TTG TTGAA TTGAT TTGAC TTGAG TTGTA TTGTT TTGTC TTGTG TTGCA TTGCT TTGCC
TTGCG TTGGA TTGGT TTGGC TTGGG
TCA TCAAA TCAAT TCAAC TCAAG TCATA TCATT TCATC TCATG TCACA TCACT TCACC

TCACG TCAGA TCAGT TCAGC TCAGG
TCT TCTAA TCTAT TCTAC TCTAG TCTTA TCTTT TCTTC TCTTG TCTCA TCTCT TCTCC
TCTCG TCTGA TCTGT TCTGC TCTGG
TCC TCCAA TCCAT TCCAC TCCAG TCCTA TCCTT TCCTC TCCTG TCCCA TCCCT TCCCC
TCCCG TCCGA TCCGT TCCGC TCCGG
TCG TCGAA TCGAT TCGAC TCGAG TCGTA TCGTT TCGTC TCGTG TCGCA TCGCT TCGCC
TCGCG TCGGA TCGGT TCGGC TCGGG
TGA TGAAA TGAAT TGAAC TGAAG TGATA TGATT TGATC TGATG TGACA TGACT TGACC
TGACG TGAGA TGAGT TGAGC TGAGG
TGT TGTAA TGTAT TGTAC TGTAG TGTTA TGTTT TGTTC TGTTG TGTCA TGTCT TGTCC
TGTCG TGTGA TGTGT TGTGC TGTGG
TGC TGCAA TGCAT TGCAC TGCAG TGCTA TGCTT TGCTC TGCTG TGCCA TGCCT TGCCC
TGCCG TGCGA TGCGT TGCGC TGCGG
TGG TGGAA TGGAT TGGAC TGGAG TGGTA TGGTT TGGTC TGGTG TGGCA TGGCT
TGGCC
TGGCG TGGGA TGGGT TGGGC TGGGG
CAA CAAAA CAAAT CAAAC CAAAG CAATA CAATT CAATC CAATG CAACA CAACT
CAACC
CAACG CAAGA CAAGT CAAGC CAAGG
CAT CATAA CATAT CATAC CATAG CATTA CATTT CATTC CATTG CATCA CATCT CATCC
CATCG CATGA CATGT CATGC CATGG
CAC CACAA CACAT CACAC CACAG CACTA CACTT CACTC CACTG CACCA CACCT
CACCC
CACCG CACGA CACGT CACGC CACGG
CAG CAGAA CAGAT CAGAC CAGAG CAGTA CAGTT CAGTC CAGTG CAGCA CAGCT
CAGCC
CAGCG CAGGA CAGGT CAGGC CAGGG
CTA CTAAA CTAAT CTAAC CTAAG CTATA CTATT CTATC CTATG CTACA CTACT CTACC
CTACG CTAGA CTAGT CTAGC CTAGG
CTT CTTAA CTTAT CTTAC CTTAG CTTTA CTTTT CTTTC CTTTG CTTCA CTTCT CTTCC
CTTCG CTTGA CTTGT CTTGC CTTGG
CTC CTCAA CTCAT CTCAC CTCAG CTCTA CTCTT CTCTC CTCTG CTCCA CTCCT CTCCC
CTCCG CTCGA CTCGT CTCGC CTCGG
CTG CTGAA CTGAT CTGAC CTGAG CTGTA CTGTT CTGTC CTGTG CTGCA CTGCT CTGCC
CTGCG CTGGA CTGGT CTGGC CTGGG
CCA CCAAA CCAAT CCAAC CCAAG CCATA CCATT CCATC CCATG CCACA CCACT CCACC
CCACGCCAGA CCAGT CCAGC CCAGG
CCT CCTAA CCTAT CCTAC CCTAG CCTTA CCTTT CCTTC CCTTG CCTCA CCTCT CCTCC
CCTCG CCTGA CCTGT CCTGC CCTGG
CCC CCCAA CCCAT CCCAC CCCAG CCCTA CCCTT CCCTC CCCTG CCCCA CCCCT CCCCC
CCCCG CCCGA CCCGT CCCGC CCCGG
CCG CCGAA CCGAT CCGAC CCGAG CCGTA CCGTT CCGTC CCGTG CCGCA CCGCT CCGCC
CCGCGCCGGA CCGGT CCGGC CCGGG
CGA CGAAA CGAAT CGAAC CGAAG CGATA CGATT CGATC CGATG CGACA CGACT CGACC
CGACG CGAGA CGAGT CGAGC CGAGG
CGT CGTAA CGTAT CGTAC CGTAG CGTTA CGTTT CGTTC CGTTG CGTCA CGTCT CGTCC
CGTCG CGTGA CGTGT CGTGC CGTGG
CGC CGCAA CGCAT CGCAC CGCAG CGCTA CGCTT CGCTC CGCTG CGCCA CGCCT CGCCC
CGCCGCGCGA CGCGT CGCGC CGCGG
CGG CGGAA CGGAT CGGAC CGGAG CGGTA CGGTT CGGTC CGGTG CGGCA CGGCT CGGCC
CGGCG CGGGA CGGGT CGGGC CGGGG
GAA GAAAA GAAAT GAAAC GAAAG GAATA GAATT GAATC GAATG GAACA GAACT GAACC
GAACG GAAGA GAAGT GAAGC GAAGG
GAT GATAA GATAT GATAC GATAG GATTA GATTT GATTC GATTG GATCA GATCT GATCC
GATCGGATGA GATGT GATGC GATGG
GAC GACAA GACAT GACAC GACAG GACTA GACTT GACTC GACTG GACCA GACCT GACCC
GACCG GACGA GACGT GACGC GACGG
GAG GAGAA GAGAT GAGAC GAGAG GAGTA GAGTT GAGTC GAGTG GAGCA GAGCT GAGCC
GAGCG GAGGA GAGGT GAGGC GAGGG

GTA GTAAA GTAAT GTAAC GTAAG GTATA GTATT GTATC GTATG GTACA GTACT GTACC
GTACGGTAGA GTAGT GTAGC GTAGG
GTT GTTAA GTTAT GTTAC GTTAG GTTTA GTTTT GTTTC GTTTG GTTCA GTTCT GTTCC
GTTCG GTTGA GTTGT GTTGC GTTGG
GTC GTCAA GTCAT GTCAC GTCAG GTCTA GTCTT GTCTC GTCTG GTCCA GTCCT GTCCC
GTCCG GTCGA GTCGT GTCGC GTCGG
GTG GTGAA GTGAT GTGAC GTGAG GTGTA GTGTT GTGTC GTGTG GTGCA GTGCT GTGCC
GTGCGGTGGA GTGGT GTGGC GTGGG
GCA GCAAA GCAAT GCAAC GCAAG GCATA GCATT GCATC GCATG GCACA GCACT GCACC
GCACG GCAGA GCAGT GCAGC GCAGG
GCT GCTAA GCTAT GCTAC GCTAG GCTTA GCTTT GCTTC GCTTG GCTCA GCTCT GCTCC
GCTCG GCTGA GCTGT GCTGC GCTGG
GCC GCCAA GCCAT GCCAC GCCAG GCCTA GCCTT GCCTC GCCTG GCCCA GCCCT GCCCC
GCCCGGCCGA GCCGT GCCGC GCCGG
GCG GCGAA GCGAT GCGAC GCGAG GCGTA GCGTT GCGTC GCGTG GCGCA GCGCT GCGCC
GCGCG GCGGA GCGGT GCGGC GCGGG
GGA GGAAA GGAAT GGAAC GGAAG GGATA GGATT GGATC GGATG GGACA GGACT GGACC
GGACG GGAGA GGAGT GGAGC GGAGG
GGT GGTAA GGTAT GGTAC GGTAG GGTTA GGTTT GGTTC GGTTG GGTCA GGTCT GGTCC
GGTCGGGTGA GGTGT GGTGC GGTGG
GGC GGCAA GGCAT GGCAC GGCAG GGCTA GGCTT GGCTC GGCTG GGCCA GGCCT GGCCC
GGCCG GGCGA GGCGT GGCGC GGCGG
GGG GGGAA GGGAT GGGAC GGGAG GGGTA GGGTT GGGTC GGGTG GGGCA GGGCT GGGCC
GGGCG GGGGA GGGGT GGGGC GGGGG

Before filtering: read2: overrepresented sequences
Sampling rate: 1 / 20
overrepresented sequence count (% of bases) distribution: cycle 1 ~
cycle 151
AAAAAAAAAAAAAAAAAAAA 6926 (0.041621%)

AAAAAAAAAAAGGCCGCCGTGACCTATTCACCCTCCACTTCCCGTCTCAGAATCTAAACGTGGTCACCTT
CGAGTAGAGAGGCCCGCCCGCCCACCGTGG 408 (0.012259%)

AAAAACATGATTACAGGGACATCTCAGGCTGACTGTGCTGTCCTGATTGTTGCTGCTGGTGTTGGTGAAT
TTGAAGCTGGTATCTCCAAGAATGGGCAGA 30 (0.000901%)

AAAAAGCTGGAAGATGGCCCTAAATTCTTGAAGTCTGGTGATGCTGCCATTGTTGATATGGTTCCTGGCA
AGCCCATGTGTGTTGAGAGCTTCTCAGACT 15 (0.000451%)

AAAAAGGCCTTCGATACGGGATAATCCTATTTATTACCTCAGAAGTTTTTTCTTCGCAGGATTTTTCTGA
GCCTTTTACCACTCCAGCCTAGCCCCTAC 28 (0.000841%)

AAAAATAAAAAATTATAACAAACCCTGAGAACCAAAATGAACGAAAATCTGTTCGCTTCATTCATTGCC
CCCACAATCCTAGGCCTACCCGCCGCAGTAC 20 (0.000601%)

AAAACATGATTACAGGGACATCTCAGGCTGACTGTGCTGTCCTGATTGTTGCTGCTGGTGTTGGTGAATT
TGAAGCTGGTATCTCCAAGAATGGGCAGAC 13 (0.000391%)

AAAACCCAGCCCATGACCCCTAACAGGGGCCCTCTCAGCCCTCCTAATGACCTCCGGCCTAGCCATGTGA
TTTCACTTCCACTCCATAACGCTCCTCATA41 (0.001232%)

AAAAGAAGAAAGATGAGGCAGAGGTCCAAGTAAACCGCTAGCTTGTTGCACCGTGGAGGCCACAGGAG
CAGAAACATGGAATGCCAGACGCTGGGGATGC 5 (0.000150%)

AAAAGCTGGAAGATGGCCCTAAATTCTTGAAGTCTGGTGATGCTGCCATTGTTGATATGGTTCCTGGCAA
GCCCATGTGTGTTGAGAGCTTCTCAGACTA 27 (0.000811%)

AAAAGGCCTTCGATACGGGATAATCCTATTTATTACCTCAGAAGTTTTTTCTTCGCAGGATTTTTCTGAG

CCTTTTACCACTCCAGCCTAGCCCCTACC 20 (0.000601%)

AAAATCACCTTCCACCCTTACTACACAATCAAAGACGCCCTCGGCTTACTTCTCTTCATTCTCTCCTTAAT
GACATTAACACTATTCTCACCAGACCTCC 4 (0.000120%)

AAAATCTGTTCGCTTCATTCATTGCCCCCACAATCCTAGGCCTACCCGCCGCAGTACTGATCATTCTATTT
CCCCCTCTATTGATCCCCACCTCCAAATA 34 (0.001022%)

AAACAACTAATTGTCGGTGTTAACAAAATGGATTCCACTGAGCCACCCTACAGCCAGAAGAGATATGAG
GAAATTGTTAAGGAAGTCAGCACTTACATTA 12 (0.000361%)

AAACATATAACTGAACTCCTCACACCCAATTGGACCAATCTATCACCCTATAGAAGAACTAATGTTAGTA
TAAGTAACATGAAAACATTCTCCTCCGCAT 9 (0.000270%)

AAACATGATTACAGGGACATCTCAGGCTGACTGTGCTGTCCTGATTGTTGCTGCTGGTGTTGGTGAATTT
GAAGCTGGTATCTCCAAGAATGGGCAGACC 11 (0.000331%)

AAACCCGGTATGGTGGTCACCTTTGCTCCAGTCAACGTTACAACGGAAGTAAAATCTGTCGAAATGCAC
CATGAAGCTTTGAGTGAAGCTCTTCCTGGGG 8 (0.000240%)

AAACCGTCTGAACTATCCTGCCCGCCATCATCCTAGTCCTCATCGCCCTCCCATCCCTACGCATCCTTTAC
ATAACAGACGAGGTCAACGATCCCTCCCT 9 (0.000270%)

AAACTGAAAGCTGAGCGTGAACGTGGTATCACCATTGATATCTCCTTGTGGAAATTTGAGACCAGCAAG
TACTATGTGACTATCATTGATGCCCCAGGAC 12 (0.000361%)

AAAGAAGAAAGATGAGGCAGAGGTCCAAGTAAACCGCTAGCTTGTTGCACCGTGGAGGCCACAGGAGC
AGAAACATGGAATGCCAGACGCTGGGGATGCT 3 (0.000090%)

AAAGAGGAGAGGCACCCGATATATGTTCTCTAGGCCTTTTAGAAAACATGGAGTTGTTCCTTTGGCCACA
TATATGCGAATCTATAAGAAAGGTGATATT 529 (0.015895%)

AAAGCAGTGGACAAGAAGGCTGCTGGAGCTGGCAAGGTCACCAAGTCTGCCCAGAAAGCTCAGAAGGC
TAAATGAATATTATCCCTAATACCTGCCACCC 47 (0.001412%)

AAAGCCCATAAAAATAAAAAATTATAACAAACCCTGAGAACCAAAATGAACGAAAATCTGTTCGCTTCA
TTCATTGCCCCCACAATCCTAGGCCTACCCG 23 (0.000691%)

AAAGCGGTCTGCCCCTGGAGGTGGTAGCAAGGTTCCACAGAAAAAAGTAAAACTTGCTGCTGATGAAGA
TGATGACGATGATGATGAAGAGGATGATGAT 3 (0.000090%)

AAAGCTGAGCGTGAACGTGGTATCACCATTGATATCTCCTTGTGGAAATTTGAGACCAGCAAGTACTATG
TGACTATCATTGATGCCCCAGGACACAGAG 10 (0.000300%)

AAAGCTGGAAGATGGCCCTAAATTCTTGAAGTCTGGTGATGCTGCCATTGTTGATATGGTTCCTGGCAAG
CCCATGTGTGTTGAGAGCTTCTCAGACTAT 13 (0.000391%)

AAAGGCCTTCGATACGGGATAATCCTATTTATTACCTCAGAAGTTTTTTTCTTCGCAGGATTTTTCTGAGC
CTTTTACCACTCCAGCCTAGCCCCTACCC 6 (0.000180%)

AAAGGCGCTGCGCGCCTGCGCGCCGTGGATTTCGCTGAGCGGCACGGCTACATCAAGGGCATCGTCAAG
GACATCATCCACGACCCGGGCCGCGGCGCGC 3 (0.000090%)

AAAGGGCTCCTTCAAGTATGCCTGGGTCTTGGATAAACTGAAAGCTGAGCGTGAACGTGGTATCACCAT
TGATATCTCCTTGTGGAAATTTGAGACCAGC 19 (0.000571%)

AAAGTTCTCCGCTCCCAGACATGGGTCCCTCGGCTTCCTGCCTCGGAAGCGCAGCAGCAGGCATCGTGG
GAAGGTGAAGAGCTTCCCTAAGGATGACCCG 3 (0.000090%)

AAATAAAAAATTATAACAAACCCTGAGAACCAAAATGAACGAAAATCTGTTCGCTTCATTCATTGCCCC
CACAATCCTAGGCCTACCCGCCGCAGTACTG 4 (0.000120%)

AAATAATTCAAGCACTGCTTATTACAATTTTACTGGGTCTCTATTTTACCCTCCTACAAGCCTCAGAGTAC
TTCGAGTCTCCCTTCACCATTTCCGACGG 44 (0.001322%)

AAATCACCTTCCACCCTTACTACACAATCAAAGACGCCCTCGGCTTACTTCTCTTCATTCTCTCCTTAATG
ACATTAACACTATTCTCACCAGACCTCCT 5 (0.000150%)

AAATCTGTTCGCTTCATTCATTGCCCCCACAATCCTAGGCCTACCCGCCGCAGTACTGATCATTCTATTTC
CCCCTCTATTGATCCCCACCTCCAAATAT 6 (0.000180%)

AAATGCGGTGGCATCGACAAAAGAACCATTGAAAAATTTGAGAAGGAGGCTGCTGAGATGGGAAAGGG
CTCCTTCAAGTATGCCTGGGTCTTGGATAAAC 6 (0.000180%)

AAATTCTTGAAGTCTGGTGATGCTGCCATTGTTGATATGGTTCCTGGCAAGCCCATGTGTGTTGAGAGCT
TCTCAGACTATCCACCTTTGGGTCGCTTTG78 (0.002344%)

AACACAGGTGTCGTGAAAACTACCCCTAAAAGCCAAAATGGGAAAGGAAAAGACTCATATCAACATTGT
CGTCATTGGACACGTAGATTCGGGCAAGTCC 24 (0.000721%)

AACATGATTACAGGGACATCTCAGGCTGACTGTGCTGTCCTGATTGTTGCTGCTGGTGTTGGTGAATTTG
AAGCTGGTATCTCCAAGAATGGGCAGACCC 6 (0.000180%)

AACCAAATAATTCAAGCACTGCTTATTACAATTTTACTGGGTCTCTATTTTACCCTCCTACAAGCCTCAGA
GTACTTCGAGTCTCCCTTCACCATTTCCG 271 (0.008143%)

AACCACCCAACTATCTATAAACCTAGCCATGGCCATCCCCTTATGAGCGGGCGCAGTGATTATAGGCTTT
CGCTCTAAGATTAAAAATGCCCTAGCCCAC 12 (0.000361%)

AACCCAGCCCATGACCCCTAACAGGGGCCCTCTCAGCCCTCCTAATGACCTCCGGCCTAGCCATGTGATT
TCACTTCCACTCCATAACGCTCCTCATACT5 (0.000150%)

AACCGACTAATCACCACCCAACAATGACTAATCAAACTAACCTCAAAACAAATGATAACCATACACAAC
ACTAAAGGACGAACCTGATCTCTTATACTAG 9 (0.000270%)

AACCGTCTGAACTATCCTGCCCGCCATCATCCTAGTCCTCATCGCCCTCCCATCCCTACGCATCCTTTACA
TAACAGACGAGGTCAACGATCCCTCCCTT 5 (0.000150%)

AACCTTTGACTGCTTCCATGTTGGCATCTGCCCCTCCTCAAGAGCAAAAGCAAATGTTGGGTGAACGGCT
GTTTCCTCTTATTCAAGCCATGCACCCTAC3 (0.000090%)

AACGCAGGCACATACTTCCTATTCTACACCCTAGTAGGCTCCCTTCCCCTACTCATCGCACTGATTTACAC
TCACAACACCCTAGGCTCACTAAACATTC 3 (0.000090%)

AACGCCTGAACGCAGGCACATACTTCCTATTCTACACCCTAGTAGGCTCCCTTCCCCTACTCATCGCACT
GATTTACACTCACAACACCCTAGGCTCACT 20 (0.000601%)

AACGTGGTATCACCATTGATATCTCCTTGTGGAAATTTGAGACCAGCAAGTACTATGTGACTATCATTGA
TGCCCCAGGACACAGAGACTTTATCAAAAA 3 (0.000090%)

AACTAACCTCCTCGGACTCCTGCCTCACTCATTTACACCAACCACCCAACTATCTATAAACCTAGCCATG
GCCATCCCCTTATGAGCGGGCGCAGTGATT 11 (0.000331%)

AACTATCTATAAACCTAGCCATGGCCATCCCCTTATGAGCGGGCGCAGTGATTATAGGCTTTCGCTCTAA
GATTAAAAATGCCCTAGCCCACTTCTTACC 4 (0.000120%)

AACTGAAAGCTGAGCGTGAACGTGGTATCACCATTGATATCTCCTTGTGGAAATTTGAGACCAGCAAGT
ACTATGTGACTATCATTGATGCCCCAGGACA 7 (0.000210%)

AACTGAACTCCTCACACCCAATTGGACCAATCTATCACCCTATAGAAGAACTAATGTTAGTATAAGTAAC
ATGAAAACATTCTCCTCCGCATAAGCCTGC 14 (0.000421%)

AACTGACTAGTTCCCCTAATAATCGGTGCCCCCGATATGGCGTTTCCCCGCATAAACAACATAAGCTTCT
GACTCTTACCTCCCTCTCTCCTACTCCTGC 4 (0.000120%)

AACTTACTACTCCGGAAAAAAAGAACCATTTGGATACATAGGTATGGTCTGAGCTATGATATCAATTGG
CTTCCTAGGGTTTATCGTGTGAGCACACCAT 3 (0.000090%)

AACTTCACCCGTAACCCACCGCCATGGCCGAGGAAGGCATTGCTGCTGGAGGTGTAATGGACGTTAATA
CTGCTTTACAAGAGGTTCTGAAGACTGCCCT 5 (0.000150%)

AAGAAGGCTGCTGGAGCTGGCAAGGTCACCAAGTCTGCCCAGAAAGCTCAGAAGGCTAAATGAATATTA
TCCCTAATACCTGCCACCCCACTCTTAATCA 22 (0.000661%)

AAGAATGTGTCTGTCAAGGATGTTCGTCGTGGCAACGTTGCTGGTGACAGCAAAAATGACCCACCAATG
GAAGCAGCTGGCTTCACTGCTCAGGTGATTA 5 (0.000150%)

AAGACTACCGATGGTTACTTGCTTCGTCTGTTCTGTGTTGGTTTTACTAAAAAACGCAACAATCAGATAC
GGAAGACCTCTTATGCTCAGCACCAACAGG 3 (0.000090%)

AAGATGGCCCTAAATTCTTGAAGTCTGGTGATGCTGCCATTGTTGATATGGTTCCTGGCAAGCCCATGTG
TGTTGAGAGCTTCTCAGACTATCCACCTTT5 (0.000150%)

AAGCAATTTTGGAGGTGGTGGAAGCTACAATGATTTTGGGAATTACAACAATCAGTCTTCAAATTTTGGA
CCCATGAAGGGAGGAAATTTTGGAGGCAGA 5 (0.000150%)

AAGCACTGCTTATTACAATTTTACTGGGTCTCTATTTTACCCTCCTACAAGCCTCAGAGTACTTCGAGTCT
CCCTTCACCATTTCCGACGGCATCTACGG 6 (0.000180%)

AAGCAGTGGACAAGAAGGCTGCTGGAGCTGGCAAGGTCACCAAGTCTGCCCAGAAAGCTCAGAAGGCT
AAATGAATATTATCCCTAATACCTGCCACCCC 14 (0.000421%)

AAGCCCATAAAAATAAAAAATTATAACAAACCCTGAGAACCAAAATGAACGAAAATCTGTTCGCTTCAT
TCATTGCCCCCACAATCCTAGGCCTACCCGC 4 (0.000120%)

AAGCCCATGTGTGTTGAGAGCTTCTCAGACTATCCACCTTTGGGTCGCTTTGCTGTTCGTGATATGAGAC
AGACAGTTGCGGTGGGTGTCATCAAAGCAG 3 (0.000090%)

AAGCGCAAAAGAAGAAAGATGAGGCAGAGGTCCAAGTAAACCGCTAGCTTGTTGCACCGTGGAGGCCA
CAGGAGCAGAAACATGGAATGCCAGACGCTGG 3 (0.000090%)

AAGCGGTCTGCCCCTGGAGGTGGTAGCAAGGTTCCACAGAAAAAAGTAAAACTTGCTGCTGATGAAGAT
GATGACGATGATGATGAAGAGGATGATGATG 8 (0.000240%)

AAGCTACAATGATTTTGGGAATTACAACAATCAGTCTTCAAATTTTGGACCCATGAAGGGAGGAAATTTT
GGAGGCAGAAGCTCTGGCCCCTATGGCGGT 8 (0.000240%)

AAGCTGAGCGTGAACGTGGTATCACCATTGATATCTCCTTGTGGAAATTTGAGACCAGCAAGTACTATGT
GACTATCATTGATGCCCCAGGACACAGAGA 7 (0.000210%)

AAGCTGGAAGATGGCCCTAAATTCTTGAAGTCTGGTGATGCTGCCATTGTTGATATGGTTCCTGGCAAGC
CCATGTGTGTTGAGAGCTTCTCAGACTATC 10 (0.000300%)

AAGGAGGCTGCTGAGATGGGAAAGGGCTCCTTCAAGTATGCCTGGGTCTTGGATAAACTGAAAGCTGAG
CGTGAACGTGGTATCACCATTGATATCTCCT 4 (0.000120%)

AAGGCCTTCGATACGGGATAATCCTATTTATTACCTCAGAAGTTTTTTCTTCGCAGGATTTTTCTGAGCC

TTTTACCACTCCAGCCTAGCCCCTACCCC 6 (0.000180%)

AAGGCTGCTGGAGCTGGCAAGGTCACCAAGTCTGCCCAGAAAGCTCAGAAGGCTAAATGAATATTATCC
CTAATACCTGCCACCCCACTCTTAATCAGTG 7 (0.000210%)

AAGGGATGGAAAGTCACCCGTAAGGATGGCAATGCCAGTGGAACCACGCTGCTTGAGGCTCTGGACTGC
ATCCTACCACCAACTCGTCCAACTGACAAGC 4 (0.000120%)

AAGGGCGCCGGGTCTGTGTTCCGCGCGCACGTGAAGCACCGTAAAGGCGCTGCGCGCCTGCGCGCCGTG
GATTTCGCTGAGCGGCACGGCTACATCAAGG 5 (0.000150%)

AAGGGCTCCTTCAAGTATGCCTGGGTCTTGGATAAACTGAAAGCTGAGCGTGAACGTGGTATCACCATT
GATATCTCCTTGTGGAAATTTGAGACCAGCA 34 (0.001022%)

AAGTATGCCTGGGTCTTGGATAAACTGAAAGCTGAGCGTGAACGTGGTATCACCATTGATATCTCCTTGT
GGAAATTTGAGACCAGCAAGTACTATGTGA 7 (0.000210%)

AAGTCCACCACTACTGGCCATCTGATCTATAAATGCGGTGGCATCGACAAAAGAACCATTGAAAAATTT
GAGAAGGAGGCTGCTGAGATGGGAAAGGGCT 4 (0.000120%)

AAGTCTGGTGATGCTGCCATTGTTGATATGGTTCCTGGCAAGCCCATGTGTGTTGAGAGCTTCTCAGACT
ATCCACCTTTGGGTCGCTTTGCTGTTCGTG 5 (0.000150%)

AAGTTTTTTTCTTCGCAGGATTTTTCTGAGCCTTTTACCACTCCAGCCTAGCCCCTACCCCCCAATTAGGA
GGGCACTGGCCCCCAACAGGCATCACCCC 3 (0.000090%)

AATAATCGGTGCCCCCGATATGGCGTTTCCCCGCATAAACAACATAAGCTTCTGACTCTTACCTCCCTCT
CTCCTACTCCTGCTCGCATCTGCTATAGTG 9 (0.000270%)

AATAATCTCCCATATTGTAACTTACTACTCCGGAAAAAAAGAACCATTTGGATACATAGGTATGGTCTGA
GCTATGATATCAATTGGCTTCCTAGGGTTT 11 (0.000331%)

AATAATTCAAGCACTGCTTATTACAATTTTACTGGGTCTCTATTTTACCCTCCTACAAGCCTCAGAGTACT
TCGAGTCTCCCTTCACCATTTCCGACGGC 35 (0.001052%)

AATCACCTCCCATTCCGATAAAATCACCTTCCACCCTTACTACACAATCAAAGACGCCCTCGGCTTACTT
CTCTTCATTCTCTCCTTAATGACATTAACA 5 (0.000150%)

AATCACCTTCCACCCTTACTACACAATCAAAGACGCCCTCGGCTTACTTCTCTTCATTCTCTCCTTAATGA
CATTAACACTATTCTCACCAGACCTCCTA 5 (0.000150%)

AATCGGTGCCCCCGATATGGCGTTTCCCCGCATAAACAACATAAGCTTCTGACTCTTACCTCCCTCTCTCC
TACTCCTGCTCGCATCTGCTATAGTGGAG 5 (0.000150%)

AATCTATCACCCTATAGAAGAACTAATGTTAGTATAAGTAACATGAAAACATTCTCCTCCGCATAAGCCT
GCGTCAGATTAAAACACTGAACTGACAATT 13 (0.000391%)

AATCTCCCATATTGTAACTTACTACTCCGGAAAAAAAGAACCATTTGGATACATAGGTATGGTCTGAGCT
ATGATATCAATTGGCTTCCTAGGGTTTATC 8 (0.000240%)

AATCTGTTCGCTTCATTCATTGCCCCCACAATCCTAGGCCTACCCGCCGCAGTACTGATCATTCTATTTCC
CCCTCTATTGATCCCCACCTCCAAATATC 7 (0.000210%)

AATGACCTCCGGCCTAGCCATGTGATTTCACTTCCACTCCATAACGCTCCTCATACTAGGCCTACTAACC
AACACACTAACCATATACCAATGATGGCGC 10 (0.000300%)

AATGATTTTGGGAATTACAACAATCAGTCTTCAAATTTTGGACCCATGAAGGGAGGAAATTTTGGAGGC
AGAAGCTCTGGCCCCTATGGCGGTGGAGGCC 6 (0.000180%)

AATGCGCAGGCTGAAGCGCAAAAGAAGAAAGATGAGGCAGAGGTCCAAGTAAACCGCTAGCTTGTTGC
ACCGTGGAGGCCACAGGAGCAGAAACATGGAA 3 (0.000090%)

AATGCGGTGGCATCGACAAAAGAACCATTGAAAAATTTGAGAAGGAGGCTGCTGAGATGGGAAAGGGC
TCCTTCAAGTATGCCTGGGTCTTGGATAAACT 5 (0.000150%)

AATGTCAAGAATGTGTCTGTCAAGGATGTTCGTCGTGGCAACGTTGCTGGTGACAGCAAAAATGACCCA
CCAATGGAAGCAGCTGGCTTCACTGCTCAGG 5 (0.000150%)

AATGTGTCTGTCAAGGATGTTCGTCGTGGCAACGTTGCTGGTGACAGCAAAAATGACCCACCAATGGAA
GCAGCTGGCTTCACTGCTCAGGTGATTATCC 8 (0.000240%)

AATTCAAGCACTGCTTATTACAATTTTACTGGGTCTCTATTTTACCCTCCTACAAGCCTCAGAGTACTTCG
AGTCTCCCTTCACCATTTCCGACGGCATC 8 (0.000240%)

AATTCTTGAAGTCTGGTGATGCTGCCATTGTTGATATGGTTCCTGGCAAGCCCATGTGTGTTGAGAGCTT
CTCAGACTATCCACCTTTGGGTCGCTTTGC 31 (0.000931%)

AATTGGACCAATCTATCACCCTATAGAAGAACTAATGTTAGTATAAGTAACATGAAAACATTCTCCTCCG
CATAAGCCTGCGTCAGATTAAAACACTGAA 7 (0.000210%)

AATTTTGGAGGTGGTGGAAGCTACAATGATTTTGGGAATTACAACAATCAGTCTTCAAATTTTGGACCCA
TGAAGGGAGGAAATTTTGGAGGCAGAAGCT 5 (0.000150%)

ACAACTAATTGTCGGTGTTAACAAAATGGATTCCACTGAGCCACCCTACAGCCAGAAGAGATATGAGGA
AATTGTTAAGGAAGTCAGCACTTACATTAAG 4 (0.000120%)

ACAAGAAGGCTGCTGGAGCTGGCAAGGTCACCAAGTCTGCCCAGAAAGCTCAGAAGGCTAAATGAATA
TTATCCCTAATACCTGCCACCCCACTCTTAAT 12 (0.000361%)

ACACAGGTGTCGTGAAAACTACCCCTAAAAGCCAAAATGGGAAAGGAAAAGACTCATATCAACATTGTC
GTCATTGGACACGTAGATTCGGGCAAGTCCA 14 (0.000421%)

ACAGAAAGTTCTCCGCTCCCAGACATGGGTCCCTCGGCTTCCTGCCTCGGAAGCGCAGCAGCAGGCATC
GTGGGAAGGTGAAGAGCTTCCCTAAGGATGA 3 (0.000090%)

ACAGACAGTTGCGGTGGGTGTCATCAAAGCAGTGGACAAGAAGGCTGCTGGAGCTGGCAAGGTCACCA
AGTCTGCCCAGAAAGCTCAGAAGGCTAAATGA 3 (0.000090%)

ACAGAGACTTTATCAAAAACATGATTACAGGGACATCTCAGGCTGACTGTGCTGTCCTGATTGTTGCTGC
TGGTGTTGGTGAATTTGAAGCTGGTATCTC 6 (0.000180%)

ACAGGGGCCCTCTCAGCCCTCCTAATGACCTCCGGCCTAGCCATGTGATTTCACTTCCACTCCATAACGC
TCCTCATACTAGGCCTACTAACCAACACAC 4 (0.000120%)

ACAGGTGTCGTGAAAACTACCCCTAAAAGCCAAAATGGGAAAGGAAAAGACTCATATCAACATTGTCGT
CATTGGACACGTAGATTCGGGCAAGTCCACC 14 (0.000421%)

ACATAACAGACGAGGTCAACGATCCCTCCCTTACCATCAAATCAATTGGCCACCAATGGTACTGAACCT
ACGAGTACACCGACTACGGCGGACTAATCTT 4 (0.000120%)

ACATGATTACAGGGACATCTCAGGCTGACTGTGCTGTCCTGATTGTTGCTGCTGGTGTTGGTGAATTTGA
AGCTGGTATCTCCAAGAATGGGCAGACCCG 10 (0.000300%)

ACCAAAGCCCATAAAAATAAAAAATTATAACAAACCCTGAGAACCAAAATGAACGAAAATCTGTTCGCT
TCATTCATTGCCCCCACAATCCTAGGCCTAC 8 (0.000240%)

ACCAACCACCCAACTATCTATAAACCTAGCCATGGCCATCCCCTTATGAGCGGGCGCAGTGATTATAGGC
TTTCGCTCTAAGATTAAAAATGCCCTAGCC 3 (0.000090%)

ACCACCCAACTATCTATAAACCTAGCCATGGCCATCCCCTTATGAGCGGGCGCAGTGATTATAGGCTTTC
GCTCTAAGATTAAAAATGCCCTAGCCCACT 4 (0.000120%)

ACCAGGCGACCTGCGACTCCTTGACGTTGACAATCGAGTAGTACTCCCGATTGAAGCCCCCATTCGTATA
ATAATTACATCACAAGACGTCTTGCACTCA 4 (0.000120%)

ACCAGGCTTCGGAATAATCTCCCATATTGTAACTTACTACTCCGGAAAAAAAGAACCATTTGGATACATA
GGTATGGTCTGAGCTATGATATCAATTGGC 6 (0.000180%)

ACCATTGATATCTCCTTGTGGAAATTTGAGACCAGCAAGTACTATGTGACTATCATTGATGCCCCAGGAC
ACAGAGACTTTATCAAAAACATGATTACAG 8 (0.000240%)

ACCCAACTATCTATAAACCTAGCCATGGCCATCCCCTTATGAGCGGGCGCAGTGATTATAGGCTTTCGCT
CTAAGATTAAAAATGCCCTAGCCCACTTCT 4 (0.000120%)

ACCCAGCCCATGACCCCTAACAGGGGCCCTCTCAGCCCTCCTAATGACCTCCGGCCTAGCCATGTGATTT
CACTTCCACTCCATAACGCTCCTCATACTA 9 (0.000270%)

ACCCCATTCTATACCAACACCTATTCTGATTTTTCGGTCACCCTGAAGTTTATATTCTTATCCTACCAGGC
TTCGGAATAATCTCCCATATTGTAACTTA 4 (0.000120%)

ACCCCTAACAGGGGCCCTCTCAGCCCTCCTAATGACCTCCGGCCTAGCCATGTGATTTCACTTCCACTCC
ATAACGCTCCTCATACTAGGCCTACTAACC 4 (0.000120%)

ACCCGATATATGTTCTCTAGGCCTTTTAGAAAACATGGAGTTGTTCCTTTGGCCACATATATGCGAATCT
ATAAGAAAGGTGATATTGTAGACATCAAGG 4 (0.000120%)

ACCCGTAACCCACCGCCATGGCCGAGGAAGGCATTGCTGCTGGAGGTGTAATGGACGTTAATACTGCTT
TACAAGAGGTTCTGAAGACTGCCCTCATCCA 3 (0.000090%)

ACCGACTAATCACCACCCAACAATGACTAATCAAACTAACCTCAAAACAAATGATAACCATACACAACA
CTAAAGGACGAACCTGATCTCTTATACTAGT 4 (0.000120%)

ACCGTCTGAACTATCCTGCCCGCCATCATCCTAGTCCTCATCGCCCTCCCATCCCTACGCATCCTTTACAT
AACAGACGAGGTCAACGATCCCTCCCTTA 6 (0.000180%)

ACCTTTGCTCCAGTCAACGTTACAACGGAAGTAAAATCTGTCGAAATGCACCATGAAGCTTTGAGTGAA
GCTCTTCCTGGGGACAATGTGGGCTTCAATG 5 (0.000150%)

ACCTTTGGGTCGCTTTGCTGTTCGTGATATGAGACAGACAGTTGCGGTGGGTGTCATCAAAGCAGTGGAC
AAGAAGGCTGCTGGAGCTGGCAAGGTCACC 4 (0.000120%)

ACGGGATAATCCTATTTATTACCTCAGAAGTTTTTTTCTTCGCAGGATTTTTCTGAGCCTTTTACCACTCC
AGCCTAGCCCCTACCCCCCAATTAGGAGG 10 (0.000300%)

ACGGGCACTGGGCGACTCTGTGCCTCGCTGAGGAAAAATAACTAAACATGGGCAAAGGAGATCCTAAG
AAGCCGAGAGGCAAAATGTCATCATATGCATT3 (0.000090%)

ACGTGGTATCACCATTGATATCTCCTTGTGGAAATTTGAGACCAGCAAGTACTATGTGACTATCATTGAT
GCCCCAGGACACAGAGACTTTATCAAAAAC 8 (0.000240%)

ACTAGTTCCCCTAATAATCGGTGCCCCCGATATGGCGTTTCCCCGCATAAACAACATAAGCTTCTGACTC
TTACCTCCCTCTCTCCTACTCCTGCTCGCA 10 (0.000300%)

ACTATCCACCTTTGGGTCGCTTTGCTGTTCGTGATATGAGACAGACAGTTGCGGTGGGTGTCATCAAAGC
AGTGGACAAGAAGGCTGCTGGAGCTGGCAA 3 (0.000090%)

ACTATCTATAAACCTAGCCATGGCCATCCCCTTATGAGCGGGCGCAGTGATTATAGGCTTTCGCTCTAAG

ATTAAAAATGCCCTAGCCCACTTCTTACCA 10 (0.000300%)

ACTATGTGACTATCATTGATGCCCCAGGACACAGAGACTTTATCAAAAACATGATTACAGGGACATCTC
AGGCTGACTGTGCTGTCCTGATTGTTGCTGC 4 (0.000120%)

ACTCATTTACACCAACCACCCAACTATCTATAAACCTAGCCATGGCCATCCCCTTATGAGCGGGCGCAGT
GATTATAGGCTTTCGCTCTAAGATTAAAAA 3 (0.000090%)

ACTCCTCACACCCAATTGGACCAATCTATCACCCTATAGAAGAACTAATGTTAGTATAAGTAACATGAAA
ACATTCTCCTCCGCATAAGCCTGCGTCAGA 13 (0.000391%)

ACTGAAAGCTGAGCGTGAACGTGGTATCACCATTGATATCTCCTTGTGGAAATTTGAGACCAGCAAGTA
CTATGTGACTATCATTGATGCCCCAGGACAC 6 (0.000180%)

ACTGAACTCCTCACACCCAATTGGACCAATCTATCACCCTATAGAAGAACTAATGTTAGTATAAGTAACA
TGAAAACATTCTCCTCCGCATAAGCCTGCG 4 (0.000120%)

ACTTACTACTCCGGAAAAAAAGAACCATTTGGATACATAGGTATGGTCTGAGCTATGATATCAATTGGCT
TCCTAGGGTTTATCGTGTGAGCACACCATA 5 (0.000150%)

ACTTCCCCCATTATTCCTAGAACCAGGCGACCTGCGACTCCTTGACGTTGACAATCGAGTAGTACTCCCG
ATTGAAGCCCCCATTCGTATAATAATTACA 4 (0.000120%)

AGAAACCGTCTGAACTATCCTGCCCGCCATCATCCTAGTCCTCATCGCCCTCCCATCCCTACGCATCCTTT
ACATAACAGACGAGGTCAACGATCCCTCC12 (0.000361%)

AGAAAGTTCTCCGCTCCCAGACATGGGTCCCTCGGCTTCCTGCCTCGGAAGCGCAGCAGCAGGCATCGT
GGGAAGGTGAAGAGCTTCCCTAAGGATGACC 13 (0.000391%)

AGAACACAGGTGTCGTGAAAACTACCCCTAAAAGCCAAAATGGGAAAGGAAAAGACTCATATCAACAT
TGTCGTCATTGGACACGTAGATTCGGGCAAGT 167 (0.005018%)

AGAACGAGAAGCTGAACTTGGAGCTAGGGCAAAAGAATTCACCAATGTTTACATCAAGAATTTTGGAGA
AGACATGGATGATGAGCGCCTTAAGGATCTC 136 (0.004086%)

AGAAGAGCTACGAGCTGCCTGACGGCCAGGTCATCACCATTGGCAATGAGCGGTTCCGCTGCCCTGAGG
CACTCTTCCAGCCTTCCTTCCTGGGCATGGA 8 (0.000240%)

AGAAGGAGGCTGCTGAGATGGGAAAGGGCTCCTTCAAGTATGCCTGGGTCTTGGATAAACTGAAAGCTG
AGCGTGAACGTGGTATCACCATTGATATCTC 14 (0.000421%)

AGAAGGCTGCTGGAGCTGGCAAGGTCACCAAGTCTGCCCAGAAAGCTCAGAAGGCTAAATGAATATTAT
CCCTAATACCTGCCACCCCACTCTTAATCAG 6 (0.000180%)

AGAATGTGTCTGTCAAGGATGTTCGTCGTGGCAACGTTGCTGGTGACAGCAAAAATGACCCACCAATGG
AAGCAGCTGGCTTCACTGCTCAGGTGATTAT 12 (0.000361%)

AGACAGACAGTTGCGGTGGGTGTCATCAAAGCAGTGGACAAGAAGGCTGCTGGAGCTGGCAAGGTCAC
CAAGTCTGCCCAGAAAGCTCAGAAGGCTAAAT 9 (0.000270%)

AGACAGTTGCGGTGGGTGTCATCAAAGCAGTGGACAAGAAGGCTGCTGGAGCTGGCAAGGTCACCAAG
TCTGCCCAGAAAGCTCAGAAGGCTAAATGAAT 30 (0.000901%)

AGACCAGCAAGTACTATGTGACTATCATTGATGCCCCAGGACACAGAGACTTTATCAAAAACATGATTA
CAGGGACATCTCAGGCTGACTGTGCTGTCCT 6 (0.000180%)

AGACCCCATTCTATACCAACACCTATTCTGATTTTTCGGTCACCCTGAAGTTTATATTCTTATCCTACCAG
GCTTCGGAATAATCTCCCATATTGTAACT 4 (0.000120%)

AGACCCGAGAGCATGCCCTTCTGGCTTACACACTGGGTGTGAAACAACTAATTGTCGGTGTTAACAAAA
TGGATTCCACTGAGCCACCCTACAGCCAGAA 6 (0.000180%)

AGACTACCGATGGTTACTTGCTTCGTCTGTTCTGTGTTGGTTTTACTAAAAAACGCAACAATCAGATACG
GAAGACCTCTTATGCTCAGCACCAACAGGT 3 (0.000090%)

AGACTATCCACCTTTGGGTCGCTTTGCTGTTCGTGATATGAGACAGACAGTTGCGGTGGGTGTCATCAAA
GCAGTGGACAAGAAGGCTGCTGGAGCTGGC 28 (0.000841%)

AGAGCATGCCCTTCTGGCTTACACACTGGGTGTGAAACAACTAATTGTCGGTGTTAACAAAATGGATTCC
ACTGAGCCACCCTACAGCCAGAAGAGATAT 17 (0.000511%)

AGAGCTTCTCAGACTATCCACCTTTGGGTCGCTTTGCTGTTCGTGATATGAGACAGACAGTTGCGGTGGG
TGTCATCAAAGCAGTGGACAAGAAGGCTGC 10 (0.000300%)

AGAGGAAGGGCGCCGGGTCTGTGTTCCGCGCGCACGTGAAGCACCGTAAAGGCGCTGCGCGCCTGCGCG
CCGTGGATTTCGCTGAGCGGCACGGCTACAT 4 (0.000120%)

AGAGGCACCCGATATATGTTCTCTAGGCCTTTTAGAAAACATGGAGTTGTTCCTTTGGCCACATATATGC
GAATCTATAAGAAAGGTGATATTGTAGACA 3 (0.000090%)

AGATGGCCCTAAATTCTTGAAGTCTGGTGATGCTGCCATTGTTGATATGGTTCCTGGCAAGCCCATGTGT
GTTGAGAGCTTCTCAGACTATCCACCTTTG 10 (0.000300%)

AGCAAGTACTATGTGACTATCATTGATGCCCCAGGACACAGAGACTTTATCAAAAACATGATTACAGGG
ACATCTCAGGCTGACTGTGCTGTCCTGATTG 11 (0.000331%)

AGCAATTTTGGAGGTGGTGGAAGCTACAATGATTTTGGGAATTACAACAATCAGTCTTCAAATTTTGGAC
CCATGAAGGGAGGAAATTTTGGAGGCAGAA 5 (0.000150%)

AGCACCGTAAAGGCGCTGCGCGCCTGCGCGCCGTGGATTTCGCTGAGCGGCACGGCTACATCAAGGGCA
TCGTCAAGGACATCATCCACGACCCGGGCCG 3 (0.000090%)

AGCACTGCTTATTACAATTTTACTGGGTCTCTATTTTACCCTCCTACAAGCCTCAGAGTACTTCGAGTCTC
CCTTCACCATTTCCGACGGCATCTACGGC 4 (0.000120%)

AGCAGCACCAGCGGTGGCAGAGACCCCAGACATCAAGCTCTTTGGGAAGTGGAGCACCGATGATGTGCA
GATCAATGACATTTCCCTGCAGGATTACATT 9 (0.000270%)

AGCATGCCCTTCTGGCTTACACACTGGGTGTGAAACAACTAATTGTCGGTGTTAACAAAATGGATTCCAC
TGAGCCACCCTACAGCCAGAAGAGATATGA 5 (0.000150%)

AGCATTTGTGCCAATTTCTGGTTGGAATGGTGACAACATGCTGGAGCCAAGTGCTAACATGCCTTGGTTC
AAGGGATGGAAAGTCACCCGTAAGGATGGC 607 (0.018239%)

AGCCAAGTGCTAACATGCCTTGGTTCAAGGGATGGAAAGTCACCCGTAAGGATGGCAATGCCAGTGGAA
CCACGCTGCTTGAGGCTCTGGACTGCATCCT 6 (0.000180%)

AGCCAGGCAACCTTCTAGGTAACGACCACATCTACAACGTTATCGTCACAGCCCATGCATTTGTAATAAT
CTTCTTCATAGTAATACCCATCATAATCGG 70 (0.002103%)

AGCCCATAAAAATAAAAAATTATAACAAACCCTGAGAACCAAAATGAACGAAAATCTGTTCGCTTCATT
CATTGCCCCCACAATCCTAGGCCTACCCGCC 9 (0.000270%)

AGCCCATGACCCCTAACAGGGGCCCTCTCAGCCCTCCTAATGACCTCCGGCCTAGCCATGTGATTTCACT
TCCACTCCATAACGCTCCTCATACTAGGCC 13 (0.000391%)

AGCCCATGTGTGTTGAGAGCTTCTCAGACTATCCACCTTTGGGTCGCTTTGCTGTTCGTGATATGAGACA
GACAGTTGCGGTGGGTGTCATCAAAGCAGT 3 (0.000090%)

AGCGAATGCGCAGGCTGAAGCGCAAAAGAAGAAAGATGAGGCAGAGGTCCAAGTAAACCGCTAGCTTG
TTGCACCGTGGAGGCCACAGGAGCAGAAACAT 936 (0.028124%)

AGCGCAAAAGAAGAAAGATGAGGCAGAGGTCCAAGTAAACCGCTAGCTTGTTGCACCGTGGAGGCCAC
AGGAGCAGAAACATGGAATGCCAGACGCTGGG 4 (0.000120%)

AGCGTGAACGTGGTATCACCATTGATATCTCCTTGTGGAAATTTGAGACCAGCAAGTACTATGTGACTAT
CATTGATGCCCCAGGACACAGAGACTTTAT 5 (0.000150%)

AGCTACAATGATTTTGGGAATTACAACAATCAGTCTTCAAATTTTGGACCCATGAAGGGAGGAAATTTTG
GAGGCAGAAGCTCTGGCCCCTATGGCGGTG 3 (0.000090%)

AGCTGAGCGTGAACGTGGTATCACCATTGATATCTCCTTGTGGAAATTTGAGACCAGCAAGTACTATGTG
ACTATCATTGATGCCCCAGGACACAGAGAC 9 (0.000270%)

AGCTGGAAGATGGCCCTAAATTCTTGAAGTCTGGTGATGCTGCCATTGTTGATATGGTTCCTGGCAAGCC
CATGTGTGTTGAGAGCTTCTCAGACTATCC 7 (0.000210%)

AGCTGGCAAGGTCACCAAGTCTGCCCAGAAAGCTCAGAAGGCTAAATGAATATTATCCCTAATACCTGC
CACCCCACTCTTAATCAGTGGTGGAAGAACG 3 (0.000090%)

AGCTTCTCAGACTATCCACCTTTGGGTCGCTTTGCTGTTCGTGATATGAGACAGACAGTTGCGGTGGGTG
TCATCAAAGCAGTGGACAAGAAGGCTGCTG 11 (0.000331%)

AGGAAGGGCGCCGGGTCTGTGTTCCGCGCGCACGTGAAGCACCGTAAAGGCGCTGCGCGCCTGCGCGCC
GTGGATTTCGCTGAGCGGCACGGCTACATCA 8 (0.000240%)

AGGACACAGAGACTTTATCAAAAACATGATTACAGGGACATCTCAGGCTGACTGTGCTGTCCTGATTGTT
GCTGCTGGTGTTGGTGAATTTGAAGCTGGT 25 (0.000751%)

AGGAGACCCCATTCTATACCAACACCTATTCTGATTTTTCGGTCACCCTGAAGTTTATATTCTTATCCTAC
CAGGCTTCGGAATAATCTCCCATATTGTA 16 (0.000481%)

AGGAGAGGCACCCGATATATGTTCTCTAGGCCTTTTAGAAAACATGGAGTTGTTCCTTTGGCCACATATA
TGCGAATCTATAAGAAAGGTGATATTGTAG 12 (0.000361%)

AGGAGGAGACCCCATTCTATACCAACACCTATTCTGATTTTTCGGTCACCCTGAAGTTTATATTCTTATCC
TACCAGGCTTCGGAATAATCTCCCATATT 586 (0.017608%)

AGGAGGCTGCTGAGATGGGAAAGGGCTCCTTCAAGTATGCCTGGGTCTTGGATAAACTGAAAGCTGAGC
GTGAACGTGGTATCACCATTGATATCTCCTT 8 (0.000240%)

AGGCAACCTTCTAGGTAACGACCACATCTACAACGTTATCGTCACAGCCCATGCATTTGTAATAATCTTC
TTCATAGTAATACCCATCATAATCGGAGGC 17 (0.000511%)

AGGCACAGCTCTAAGCCTCCTTATTCGAGCCGAGCTGGGCCAGCCAGGCAACCTTCTAGGTAACGACCA
CATCTACAACGTTATCGTCACAGCCCATGCA 4 (0.000120%)

AGGCACCCGATATATGTTCTCTAGGCCTTTTAGAAAACATGGAGTTGTTCCTTTGGCCACATATATGCGA
ATCTATAAGAAAGGTGATATTGTAGACATC 10 (0.000300%)

AGGCCATCTCCTGGGCCGCCTGGCGGCCATCGTGGCTAAACAGGTACTGCTGGGCCGGAAGGTGGTGGT
CGTACGCTGTGAAGGCATCAACATTTCTGGC 16 (0.000481%)

AGGCCTTCGATACGGGATAATCCTATTTATTACCTCAGAAGTTTTTTTCTTCGCAGGATTTTTCTGAGCCT
TTTACCACTCCAGCCTAGCCCCTACCCCC 5 (0.000150%)

AGGCTGACTGTGCTGTCCTGATTGTTGCTGCTGGTGTTGGTGAATTTGAAGCTGGTATCTCCAAGAATGG

GCAGACCCGAGAGCATGCCCTTCTGGCTTA 11 (0.000331%)

AGGCTGCTGGAGCTGGCAAGGTCACCAAGTCTGCCCAGAAAGCTCAGAAGGCTAAATGAATATTATCCC
TAATACCTGCCACCCCACTCTTAATCAGTGG 18 (0.000541%)

AGGGATTGTGTTTAAAGTAGTGCTTCTACCAACATGTCCCGTGGTTCCAGCGCCGGTTTTGACCGCCACA
TTACCATTTTTTCACCCGAGGGTCGGCTCT4 (0.000120%)

AGGGCGCCGGGTCTGTGTTCCGCGCGCACGTGAAGCACCGTAAAGGCGCTGCGCGCCTGCGCGCCGTGG
ATTTCGCTGAGCGGCACGGCTACATCAAGGG 3 (0.000090%)

AGGGCTCCTTCAAGTATGCCTGGGTCTTGGATAAACTGAAAGCTGAGCGTGAACGTGGTATCACCATTG
ATATCTCCTTGTGGAAATTTGAGACCAGCAA 11 (0.000331%)

AGGGGCCCTCTCAGCCCTCCTAATGACCTCCGGCCTAGCCATGTGATTTCACTTCCACTCCATAACGCTC
CTCATACTAGGCCTACTAACCAACACACTA 8 (0.000240%)

AGGTCAACGATCCCTCCCTTACCATCAAATCAATTGGCCACCAATGGTACTGAACCTACGAGTACACCGA
CTACGGCGGACTAATCTTCAACTCCTACAT 3 (0.000090%)

AGGTGTCGTGAAAACTACCCCTAAAAGCCAAAATGGGAAAGGAAAAGACTCATATCAACATTGTCGTCA
TTGGACACGTAGATTCGGGCAAGTCCACCAC 6 (0.000180%)

AGTAGGCTCCCTTCCCCTACTCATCGCACTGATTTACACTCACAACACCCTAGGCTCACTAAACATTCTA
CTACTCACTCTCACTGCCCAAGAACTATCA 3 (0.000090%)

AGTCCTAGCTGCTGGCATCACTATACTACTAACAGACCGCAACCTCAACACCACCTTCTTCGACCCCGCC
GGAGGAGGAGACCCCATTCTATACCAACAC 3 (0.000090%)

AGTCCTCATCGCCCTCCCATCCCTACGCATCCTTTACATAACAGACGAGGTCAACGATCCCTCCCTTACC
ATCAAATCAATTGGCCACCAATGGTACTGA 4 (0.000120%)

AGTCTGGTGATGCTGCCATTGTTGATATGGTTCCTGGCAAGCCCATGTGTGTTGAGAGCTTCTCAGACTA
TCCACCTTTGGGTCGCTTTGCTGTTCGTGA 19 (0.000571%)

AGTGGACAAGAAGGCTGCTGGAGCTGGCAAGGTCACCAAGTCTGCCCAGAAAGCTCAGAAGGCTAAAT
GAATATTATCCCTAATACCTGCCACCCCACTC 10 (0.000300%)

AGTGGGAGACAGCAGCACCAGCGGTGGCAGAGACCCCAGACATCAAGCTCTTTGGGAAGTGGAGCACC
GATGATGTGCAGATCAATGACATTTCCCTGCA 220 (0.006610%)

AGTTCCCCTAATAATCGGTGCCCCCGATATGGCGTTTCCCCGCATAAACAACATAAGCTTCTGACTCTTA
CCTCCCTCTCTCCTACTCCTGCTCGCATCT 5 (0.000150%)

AGTTGCGGTGGGTGTCATCAAAGCAGTGGACAAGAAGGCTGCTGGAGCTGGCAAGGTCACCAAGTCTGC
CCAGAAAGCTCAGAAGGCTAAATGAATATTA 4 (0.000120%)

AGTTTTTTTCTTCGCAGGATTTTTCTGAGCCTTTTACCACTCCAGCCTAGCCCCTACCCCCCAATTAGGAG
GGCACTGGCCCCCAACAGGCATCACCCCG3 (0.000090%)

ATAAAAAATTATAACAAACCCTGAGAACCAAAATGAACGAAAATCTGTTCGCTTCATTCATTGCCCCCA
CAATCCTAGGCCTACCCGCCGCAGTACTGAT 3 (0.000090%)

ATAAAAATAAAAAATTATAACAAACCCTGAGAACCAAAATGAACGAAAATCTGTTCGCTTCATTCATTG
CCCCCACAATCCTAGGCCTACCCGCCGCAGT 23 (0.000691%)

ATAAAATCACCTTCCACCCTTACTACACAATCAAAGACGCCCTCGGCTTACTTCTCTTCATTCTCTCCTTA
ATGACATTAACACTATTCTCACCAGACCT 59 (0.001773%)

ATAAACCTAGCCATGGCCATCCCCTTATGAGCGGGCGCAGTGATTATAGGCTTTCGCTCTAAGATTAAAA
ATGCCCTAGCCCACTTCTTACCACAAGGCA 15 (0.000451%)

ATAAACTGAAAGCTGAGCGTGAACGTGGTATCACCATTGATATCTCCTTGTGGAAATTTGAGACCAGCA
AGTACTATGTGACTATCATTGATGCCCCAGG 50 (0.001502%)

ATAAATGCGGTGGCATCGACAAAAGAACCATTGAAAAATTTGAGAAGGAGGCTGCTGAGATGGGAAAG
GGCTCCTTCAAGTATGCCTGGGTCTTGGATAA 5 (0.000150%)

ATAACTGAACTCCTCACACCCAATTGGACCAATCTATCACCCTATAGAAGAACTAATGTTAGTATAAGTA
ACATGAAAACATTCTCCTCCGCATAAGCCT 6 (0.000180%)

ATAATCCTATTTATTACCTCAGAAGTTTTTTTCTTCGCAGGATTTTTCTGAGCCTTTTACCACTCCAGCCTA
GCCCCTACCCCCCAATTAGGAGGGCACT 4 (0.000120%)

ATAATCGGTGCCCCCGATATGGCGTTTCCCCGCATAAACAACATAAGCTTCTGACTCTTACCTCCCTCTCT
CCTACTCCTGCTCGCATCTGCTATAGTGG 7 (0.000210%)

ATAATCTCCCATATTGTAACTTACTACTCCGGAAAAAAAGAACCATTTGGATACATAGGTATGGTCTGAG
CTATGATATCAATTGGCTTCCTAGGGTTTA 15 (0.000451%)

ATAATTCAAGCACTGCTTATTACAATTTTACTGGGTCTCTATTTTACCCTCCTACAAGCCTCAGAGTACTT
CGAGTCTCCCTTCACCATTTCCGACGGCA 7 (0.000210%)

ATACGGGATAATCCTATTTATTACCTCAGAAGTTTTTTTCTTCGCAGGATTTTTCTGAGCCTTTTACCACT
CCAGCCTAGCCCCTACCCCCCAATTAGGA 6 (0.000180%)

ATACTTCCCCCATTATTCCTAGAACCAGGCGACCTGCGACTCCTTGACGTTGACAATCGAGTAGTACTCC
CGATTGAAGCCCCCATTCGTATAATAATTA 5 (0.000150%)

ATACTTCCTATTCTACACCCTAGTAGGCTCCCTTCCCCTACTCATCGCACTGATTTACACTCACAACACCC
TAGGCTCACTAAACATTCTACTACTCACT 4 (0.000120%)

ATAGAAACCGTCTGAACTATCCTGCCCGCCATCATCCTAGTCCTCATCGCCCTCCCATCCCTACGCATCCT
TTACATAACAGACGAGGTCAACGATCCCT 645 (0.019380%)

ATATAACTGAACTCCTCACACCCAATTGGACCAATCTATCACCCTATAGAAGAACTAATGTTAGTATAAG
TAACATGAAAACATTCTCCTCCGCATAAGC 28 (0.000841%)

ATATATGTTCTCTAGGCCTTTTAGAAAACATGGAGTTGTTCCTTTGGCCACATATATGCGAATCTATAAG
AAAGGTGATATTGTAGACATCAAGGGAATG 4 (0.000120%)

ATATCTCCTTGTGGAAATTTGAGACCAGCAAGTACTATGTGACTATCATTGATGCCCCAGGACACAGAGA
CTTTATCAAAAACATGATTACAGGGACATC 5 (0.000150%)

ATATCTGGAAAGCGGTCTGCCCCTGGAGGTGGTAGCAAGGTTCCACAGAAAAAAGTAAAACTTGCTGCT
GATGAAGATGATGACGATGATGATGAAGAGG 289 (0.008684%)

ATATGAGACAGACAGTTGCGGTGGGTGTCATCAAAGCAGTGGACAAGAAGGCTGCTGGAGCTGGCAAG
GTCACCAAGTCTGCCCAGAAAGCTCAGAAGGC 4 (0.000120%)

ATATGTTCTCTAGGCCTTTTAGAAAACATGGAGTTGTTCCTTTGGCCACATATATGCGAATCTATAAGAA
AGGTGATATTGTAGACATCAAGGGAATGGG 3 (0.000090%)

ATCAAAAACATGATTACAGGGACATCTCAGGCTGACTGTGCTGTCCTGATTGTTGCTGCTGGTGTTGGTG
AATTTGAAGCTGGTATCTCCAAGAATGGGC 22 (0.000661%)

ATCAAAGCAGTGGACAAGAAGGCTGCTGGAGCTGGCAAGGTCACCAAGTCTGCCCAGAAAGCTCAGAA
GGCTAAATGAATATTATCCCTAATACCTGCCA 40 (0.001202%)

ATCACCATTGATATCTCCTTGTGGAAATTTGAGACCAGCAAGTACTATGTGACTATCATTGATGCCCCAG
GACACAGAGACTTTATCAAAAACATGATTA 37 (0.001112%)

ATCACCCTATAGAAGAACTAATGTTAGTATAAGTAACATGAAAACATTCTCCTCCGCATAAGCCTGCGTC
AGATTAAAACACTGAACTGACAATTAACAG 8 (0.000240%)

ATCACCTCCCATTCCGATAAAATCACCTTCCACCCTTACTACACAATCAAAGACGCCCTCGGCTTACTTCT
CTTCATTCTCTCCTTAATGACATTAACAC 6 (0.000180%)

ATCACCTTCCACCCTTACTACACAATCAAAGACGCCCTCGGCTTACTTCTCTTCATTCTCTCCTTAATGAC
ATTAACACTATTCTCACCAGACCTCCTAG 10 (0.000300%)

ATCATCCTAGTCCTCATCGCCCTCCCATCCCTACGCATCCTTTACATAACAGACGAGGTCAACGATCCCT
CCCTTACCATCAAATCAATTGGCCACCAAT 4 (0.000120%)

ATCATTGATGCCCCAGGACACAGAGACTTTATCAAAAACATGATTACAGGGACATCTCAGGCTGACTGT
GCTGTCCTGATTGTTGCTGCTGGTGTTGGTG 8 (0.000240%)

ATCATTTTTATTGCCACAACTAACCTCCTCGGACTCCTGCCTCACTCATTTACACCAACCACCCAACTATC
TATAAACCTAGCCATGGCCATCCCCTTAT 3 (0.000090%)

ATCCACCTTTGGGTCGCTTTGCTGTTCGTGATATGAGACAGACAGTTGCGGTGGGTGTCATCAAAGCAGT
GGACAAGAAGGCTGCTGGAGCTGGCAAGGT 9 (0.000270%)

ATCCCTACGCATCCTTTACATAACAGACGAGGTCAACGATCCCTCCCTTACCATCAAATCAATTGGCCAC
CAATGGTACTGAACCTACGAGTACACCGAC 3 (0.000090%)

ATCCTACCAGGCTTCGGAATAATCTCCCATATTGTAACTTACTACTCCGGAAAAAAAGAACCATTTGGAT
ACATAGGTATGGTCTGAGCTATGATATCAA 502 (0.015084%)

ATCCTGCCCGCCATCATCCTAGTCCTCATCGCCCTCCCATCCCTACGCATCCTTTACATAACAGACGAGGT
CAACGATCCCTCCCTTACCATCAAATCAA 5 (0.000150%)

ATCGACAAAAGAACCATTGAAAAATTTGAGAAGGAGGCTGCTGAGATGGGAAAGGGCTCCTTCAAGTAT
GCCTGGGTCTTGGATAAACTGAAAGCTGAGC 4 (0.000120%)

ATCGCCCTCCCATCCCTACGCATCCTTTACATAACAGACGAGGTCAACGATCCCTCCCTTACCATCAAAT
CAATTGGCCACCAATGGTACTGAACCTACG 7 (0.000210%)

ATCGGTGCCCCCGATATGGCGTTTCCCCGCATAAACAACATAAGCTTCTGACTCTTACCTCCCTCTCTCCT
ACTCCTGCTCGCATCTGCTATAGTGGAGG 4 (0.000120%)

ATCTATAAACCTAGCCATGGCCATCCCCTTATGAGCGGGCGCAGTGATTATAGGCTTTCGCTCTAAGATT
AAAAATGCCCTAGCCCACTTCTTACCACAA 5 (0.000150%)

ATCTATAAATGCGGTGGCATCGACAAAAGAACCATTGAAAAATTTGAGAAGGAGGCTGCTGAGATGGG
AAAGGGCTCCTTCAAGTATGCCTGGGTCTTGG 253 (0.007602%)

ATCTATCACCCTATAGAAGAACTAATGTTAGTATAAGTAACATGAAAACATTCTCCTCCGCATAAGCCTG
CGTCAGATTAAAACACTGAACTGACAATTA 5 (0.000150%)

ATCTCAGGCTGACTGTGCTGTCCTGATTGTTGCTGCTGGTGTTGGTGAATTTGAAGCTGGTATCTCCAAG
AATGGGCAGACCCGAGAGCATGCCCTTCTG 4 (0.000120%)

ATCTCCTGGGCCGCCTGGCGGCCATCGTGGCTAAACAGGTACTGCTGGGCCGGAAGGTGGTGGTCGTAC
GCTGTGAAGGCATCAACATTTCTGGCAATTT 4 (0.000120%)

ATCTCCTTGTGGAAATTTGAGACCAGCAAGTACTATGTGACTATCATTGATGCCCCAGGACACAGAGACT

TTATCAAAAACATGATTACAGGGACATCTC 11 (0.000331%)

ATCTCTCCCAGTCCTAGCTGCTGGCATCACTATACTACTAACAGACCGCAACCTCAACACCACCTTCTTC
GACCCCGCCGGAGGAGGAGACCCCATTCTA 3 (0.000090%)

ATCTGTTCGCTTCATTCATTGCCCCCACAATCCTAGGCCTACCCGCCGCAGTACTGATCATTCTATTTCCC
CCTCTATTGATCCCCACCTCCAAATATCT 6 (0.000180%)

ATGACCCCTAACAGGGGCCCTCTCAGCCCTCCTAATGACCTCCGGCCTAGCCATGTGATTTCACTTCCAC
TCCATAACGCTCCTCATACTAGGCCTACTA 24 (0.000721%)

ATGACCTCCGGCCTAGCCATGTGATTTCACTTCCACTCCATAACGCTCCTCATACTAGGCCTACTAACCA
ACACACTAACCATATACCAATGATGGCGCG 4 (0.000120%)

ATGAGACAGACAGTTGCGGTGGGTGTCATCAAAGCAGTGGACAAGAAGGCTGCTGGAGCTGGCAAGGT
CACCAAGTCTGCCCAGAAAGCTCAGAAGGCTA 7 (0.000210%)

ATGATTACAGGGACATCTCAGGCTGACTGTGCTGTCCTGATTGTTGCTGCTGGTGTTGGTGAATTTGAAG
CTGGTATCTCCAAGAATGGGCAGACCCGAG 8 (0.000240%)

ATGATTTTGGGAATTACAACAATCAGTCTTCAAATTTTGGACCCATGAAGGGAGGAAATTTTGGAGGCA
GAAGCTCTGGCCCCTATGGCGGTGGAGGCCA 9 (0.000270%)

ATGCCCTTCTGGCTTACACACTGGGTGTGAAACAACTAATTGTCGGTGTTAACAAAATGGATTCCACTGA
GCCACCCTACAGCCAGAAGAGATATGAGGA 6 (0.000180%)

ATGCCTGGGTCTTGGATAAACTGAAAGCTGAGCGTGAACGTGGTATCACCATTGATATCTCCTTGTGGAA
ATTTGAGACCAGCAAGTACTATGTGACTAT 4 (0.000120%)

ATGCGCAGGCTGAAGCGCAAAAGAAGAAAGATGAGGCAGAGGTCCAAGTAAACCGCTAGCTTGTTGCA
CCGTGGAGGCCACAGGAGCAGAAACATGGAAT 3 (0.000090%)

ATGCGGTGGCATCGACAAAAGAACCATTGAAAAATTTGAGAAGGAGGCTGCTGAGATGGGAAAGGGCT
CCTTCAAGTATGCCTGGGTCTTGGATAAACTG 5 (0.000150%)

ATGGAAAGTCACCCGTAAGGATGGCAATGCCAGTGGAACCACGCTGCTTGAGGCTCTGGACTGCATCCT
ACCACCAACTCGTCCAACTGACAAGCCCTTG 12 (0.000361%)

ATGGCCCTAAATTCTTGAAGTCTGGTGATGCTGCCATTGTTGATATGGTTCCTGGCAAGCCCATGTGTGTT
GAGAGCTTCTCAGACTATCCACCTTTGGG 7 (0.000210%)

ATGGCGTTTCCCCGCATAAACAACATAAGCTTCTGACTCTTACCTCCCTCTCTCCTACTCCTGCTCGCATC
TGCTATAGTGGAGGCCGGAGCAGGAACAG 3 (0.000090%)

ATGGTGGTCACCTTTGCTCCAGTCAACGTTACAACGGAAGTAAAATCTGTCGAAATGCACCATGAAGCTT
TGAGTGAAGCTCTTCCTGGGGACAATGTGG 9 (0.000270%)

ATGGTTCCTGGCAAGCCCATGTGTGTTGAGAGCTTCTCAGACTATCCACCTTTGGGTCGCTTTGCTGTTCG
TGATATGAGACAGACAGTTGCGGTGGGTG7 (0.000210%)

ATGTGACTATCATTGATGCCCCAGGACACAGAGACTTTATCAAAAACATGATTACAGGGACATCTCAGG
CTGACTGTGCTGTCCTGATTGTTGCTGCTGG 7 (0.000210%)

ATGTGTCTGTCAAGGATGTTCGTCGTGGCAACGTTGCTGGTGACAGCAAAAATGACCCACCAATGGAAG
CAGCTGGCTTCACTGCTCAGGTGATTATCCT 3 (0.000090%)

ATGTTCTCTAGGCCTTTTAGAAAACATGGAGTTGTTCCTTTGGCCACATATATGCGAATCTATAAGAAAG
GTGATATTGTAGACATCAAGGGAATGGGTA 7 (0.000210%)

ATTACCTCAGAAGTTTTTTTCTTCGCAGGATTTTTCTGAGCCTTTTACCACTCCAGCCTAGCCCCTACCCC
CCAATTAGGAGGGCACTGGCCCCCAACAG 38 (0.001142%)

ATTCAACTTCACCCGTAACCCACCGCCATGGCCGAGGAAGGCATTGCTGCTGGAGGTGTAATGGACGTT
AATACTGCTTTACAAGAGGTTCTGAAGACTG 11 (0.000331%)

ATTCAAGCACTGCTTATTACAATTTTACTGGGTCTCTATTTTACCCTCCTACAAGCCTCAGAGTACTTCGA
GTCTCCCTTCACCATTTCCGACGGCATCT 26 (0.000781%)

ATTCACCCTCCACTTCCCGTCTCAGAATCTAAACGTGGTCACCTTCGAGTAGAGAGGCCCGCCCGCCCAC
CGTGGGCAGTGCCACCCGCAGATGACACGC 252 (0.007572%)

ATTCATTGCCCCCACAATCCTAGGCCTACCCGCCGCAGTACTGATCATTCTATTTCCCCCTCTATTGATCC
CCACCTCCAAATATCTCATCAACAACCGA 7 (0.000210%)

ATTCCTAGAACCAGGCGACCTGCGACTCCTTGACGTTGACAATCGAGTAGTACTCCCGATTGAAGCCCCC
ATTCGTATAATAATTACATCACAAGACGTC 3 (0.000090%)

ATTCGAGCCGAGCTGGGCCAGCCAGGCAACCTTCTAGGTAACGACCACATCTACAACGTTATCGTCACA
GCCCATGCATTTGTAATAATCTTCTTCATAG 14 (0.000421%)

ATTCGGGCAAGTCCACCACTACTGGCCATCTGATCTATAAATGCGGTGGCATCGACAAAAGAACCATTG
AAAAATTTGAGAAGGAGGCTGCTGAGATGGG 37 (0.001112%)

ATTCTACACCCTAGTAGGCTCCCTTCCCCTACTCATCGCACTGATTTACACTCACAACACCCTAGGCTCAC
TAAACATTCTACTACTCACTCTCACTGCC 9 (0.000270%)

ATTCTTGAAGTCTGGTGATGCTGCCATTGTTGATATGGTTCCTGGCAAGCCCATGTGTGTTGAGAGCTTCT
CAGACTATCCACCTTTGGGTCGCTTTGCT 16 (0.000481%)

ATTGATATCTCCTTGTGGAAATTTGAGACCAGCAAGTACTATGTGACTATCATTGATGCCCCAGGACACA
GAGACTTTATCAAAAACATGATTACAGGGA 23 (0.000691%)

ATTGATGCCCCAGGACACAGAGACTTTATCAAAAACATGATTACAGGGACATCTCAGGCTGACTGTGCT
GTCCTGATTGTTGCTGCTGGTGTTGGTGAAT 23 (0.000691%)

ATTGCCACACGGCTCACATTGCATGCAAGTTTGCTGAGCTGAAGGAAAAGATTGATCGCCGTTCTGGTAA
AAAGCTGGAAGATGGCCCTAAATTCTTGAA 18 (0.000541%)

ATTGGACACGTAGATTCGGGCAAGTCCACCACTACTGGCCATCTGATCTATAAATGCGGTGGCATCGAC
AAAAGAACCATTGAAAAATTTGAGAAGGAGG 10 (0.000300%)

ATTGGACCAATCTATCACCCTATAGAAGAACTAATGTTAGTATAAGTAACATGAAAACATTCTCCTCCGC
ATAAGCCTGCGTCAGATTAAAACACTGAAC 14 (0.000421%)

ATTGTAACTTACTACTCCGGAAAAAAAGAACCATTTGGATACATAGGTATGGTCTGAGCTATGATATCAA
TTGGCTTCCTAGGGTTTATCGTGTGAGCAC 19 (0.000571%)

ATTGTCGTCATTGGACACGTAGATTCGGGCAAGTCCACCACTACTGGCCATCTGATCTATAAATGCGGTG
GCATCGACAAAAGAACCATTGAAAAATTTG 16 (0.000481%)

ATTGTTGATATGGTTCCTGGCAAGCCCATGTGTGTTGAGAGCTTCTCAGACTATCCACCTTTGGGTCGCTT
TGCTGTTCGTGATATGAGACAGACAGTTG 11 (0.000331%)

ATTTATTACCTCAGAAGTTTTTTTCTTCGCAGGATTTTTCTGAGCCTTTTACCACTCCAGCCTAGCCCCTAC
CCCCCAATTAGGAGGGCACTGGCCCCCA 30 (0.000901%)

ATTTGAGAAGGAGGCTGCTGAGATGGGAAAGGGCTCCTTCAAGTATGCCTGGGTCTTGGATAAACTGAA
AGCTGAGCGTGAACGTGGTATCACCATTGAT 548 (0.016466%)

ATTTGAGACCAGCAAGTACTATGTGACTATCATTGATGCCCCAGGACACAGAGACTTTATCAAAAACAT
GATTACAGGGACATCTCAGGCTGACTGTGCT 150 (0.004507%)

ATTTGTGCCAATTTCTGGTTGGAATGGTGACAACATGCTGGAGCCAAGTGCTAACATGCCTTGGTTCAAG
GGATGGAAAGTCACCCGTAAGGATGGCAAT 55 (0.001653%)

ATTTTAACATGGCTGCTGACATCGATCCTCAGAATGCAGATGTTTATCACCACCGAGGACAGCTGAAAAT
ACTCCTTGATCAAGTTGAAGAAGCAGTGGCAGATTTTGATGAATGTATTAGGTTAAGACCTGAGTCTGCT
CTGGCACAA 2 (0.000090%)

ATTTTGGAGGTGGTGGAAGCTACAATGATTTTGGGAATTACAACAATCAGTCTTCAAATTTTGGACCCAT
GAAGGGAGGAAATTTTGGAGGCAGAAGCTC 9 (0.000270%)

ATTTTGGGAATTACAACAATCAGTCTTCAAATTTTGGACCCATGAAGGGAGGAAATTTTGGAGGCAGAA
GCTCTGGCCCCTATGGCGGTGGAGGCCAATA 6 (0.000180%)

ATTTTTATTGCCACAACTAACCTCCTCGGACTCCTGCCTCACTCATTTACACCAACCACCCAACTATCTAT
AAACCTAGCCATGGCCATCCCCTTATGAG 11 (0.000331%)

CAAAAACATGATTACAGGGACATCTCAGGCTGACTGTGCTGTCCTGATTGTTGCTGCTGGTGTTGGTGAA
TTTGAAGCTGGTATCTCCAAGAATGGGCAG 98 (0.002945%)

CAAAAAGGCCTTCGATACGGGATAATCCTATTTATTACCTCAGAAGTTTTTTCTTCGCAGGATTTTTCTG
AGCCTTTTACCACTCCAGCCTAGCCCCTA 718 (0.021574%)

CAAAACCAAGATGAAGAGACGAGCTGTGGGGATCTGGCACTGTGGTTCCTGCATGAAGACAGTGGCTGG
CGGTGCCTGGACGTACAATACCACTTCCGCT 215 (0.006460%)

CAAACATATAACTGAACTCCTCACACCCAATTGGACCAATCTATCACCCTATAGAAGAACTAATGTTAGT
ATAAGTAACATGAAAACATTCTCCTCCGCA 71 (0.002133%)

CAAACCCGGTATGGTGGTCACCTTTGCTCCAGTCAACGTTACAACGGAAGTAAAATCTGTCGAAATGCA
CCATGAAGCTTTGAGTGAAGCTCTTCCTGGG 14 (0.000421%)

CAAAGCAGTGGACAAGAAGGCTGCTGGAGCTGGCAAGGTCACCAAGTCTGCCCAGAAAGCTCAGAAGG
CTAAATGAATATTATCCCTAATACCTGCCACC 27 (0.000811%)

CAAAGCCCATAAAAATAAAAAATTATAACAAACCCTGAGAACCAAAATGAACGAAAATCTGTTCGCTTC
ATTCATTGCCCCCACAATCCTAGGCCTACCC 17 (0.000511%)

CAAAGGATCTCCTTCATCCCTCTCCAGAAGAGGAGAAGAGGAAACACAAGAAGAAACGCCTGGTGCAG
AGCCCCAATTCCTACTTCATGGATGTGAAATG 3 (0.000090%)

CAAATAATTCAAGCACTGCTTATTACAATTTTACTGGGTCTCTATTTTACCCTCCTACAAGCCTCAGAGTA
CTTCGAGTCTCCCTTCACCATTTCCGACG 3 (0.000090%)

CAACAACCGACTAATCACCACCCAACAATGACTAATCAAACTAACCTCAAAACAAATGATAACCATACA
CAACACTAAAGGACGAACCTGATCTCTTATA 788 (0.023677%)

CAACCACCCAACTATCTATAAACCTAGCCATGGCCATCCCCTTATGAGCGGGCGCAGTGATTATAGGCTT
TCGCTCTAAGATTAAAAATGCCCTAGCCCA 52 (0.001562%)

CAACCGACTAATCACCACCCAACAATGACTAATCAAACTAACCTCAAAACAAATGATAACCATACACAA
CACTAAAGGACGAACCTGATCTCTTATACTA 25 (0.000751%)

CAACCTTCTAGGTAACGACCACATCTACAACGTTATCGTCACAGCCCATGCATTTGTAATAATCTTCTTC
ATAGTAATACCCATCATAATCGGAGGCTTT 11 (0.000331%)

CAACGTTGTAGGCCCCTACGGGCTACTACAACCCTTCGCTGACGCCATAAAACTCTTCACCAAAGAGCCC
CTAAAACCCGCCACATCTACCATCACCCTC 19 (0.000571%)

CAACTAACCTCCTCGGACTCCTGCCTCACTCATTTACACCAACCACCCAACTATCTATAAACCTAGCCAT
GGCCATCCCCTTATGAGCGGGCGCAGTGAT 8 (0.000240%)

CAACTAATTGTCGGTGTTAACAAAATGGATTCCACTGAGCCACCCTACAGCCAGAAGAGATATGAGGAA
ATTGTTAAGGAAGTCAGCACTTACATTAAGA 5 (0.000150%)

CAACTATCTATAAACCTAGCCATGGCCATCCCCTTATGAGCGGGCGCAGTGATTATAGGCTTTCGCTCTA
AGATTAAAAATGCCCTAGCCCACTTCTTAC 40 (0.001202%)

CAACTGACTAGTTCCCCTAATAATCGGTGCCCCCGATATGGCGTTTCCCCGCATAAACAACATAAGCTTC
TGACTCTTACCTCCCTCTCTCCTACTCCTG 338 (0.010156%)

CAACTTCACCCGTAACCCACCGCCATGGCCGAGGAAGGCATTGCTGCTGGAGGTGTAATGGACGTTAAT
ACTGCTTTACAAGAGGTTCTGAAGACTGCCC 13 (0.000391%)

CAAGAAGGCTGCTGGAGCTGGCAAGGTCACCAAGTCTGCCCAGAAAGCTCAGAAGGCTAAATGAATATT
ATCCCTAATACCTGCCACCCCACTCTTAATC 101 (0.003035%)

CAAGAATGTGTCTGTCAAGGATGTTCGTCGTGGCAACGTTGCTGGTGACAGCAAAAATGACCCACCAAT
GGAAGCAGCTGGCTTCACTGCTCAGGTGATT 76 (0.002284%)

CAAGACTACCGATGGTTACTTGCTTCGTCTGTTCTGTGTTGGTTTTACTAAAAAACGCAACAATCAGATA
CGGAAGACCTCTTATGCTCAGCACCAACAG 5 (0.000150%)

CAAGATTCAACTTCACCCGTAACCCACCGCCATGGCCGAGGAAGGCATTGCTGCTGGAGGTGTAATGGA
CGTTAATACTGCTTTACAAGAGGTTCTGAAG 713 (0.021424%)

CAAGCACTGCTTATTACAATTTTACTGGGTCTCTATTTTACCCTCCTACAAGCCTCAGAGTACTTCGAGTC
TCCCTTCACCATTTCCGACGGCATCTACG 12 (0.000361%)

CAAGCCCATGTGTGTTGAGAGCTTCTCAGACTATCCACCTTTGGGTCGCTTTGCTGTTCGTGATATGAGA
CAGACAGTTGCGGTGGGTGTCATCAAAGCA 11 (0.000331%)

CAAGGGATGGAAAGTCACCCGTAAGGATGGCAATGCCAGTGGAACCACGCTGCTTGAGGCTCTGGACTG
CATCCTACCACCAACTCGTCCAACTGACAAG 242 (0.007271%)

CAAGTACTATGTGACTATCATTGATGCCCCAGGACACAGAGACTTTATCAAAAACATGATTACAGGGAC
ATCTCAGGCTGACTGTGCTGTCCTGATTGTT 16 (0.000481%)

CAAGTATGCCTGGGTCTTGGATAAACTGAAAGCTGAGCGTGAACGTGGTATCACCATTGATATCTCCTTG
TGGAAATTTGAGACCAGCAAGTACTATGTG 26 (0.000781%)

CAAGTCCACCACTACTGGCCATCTGATCTATAAATGCGGTGGCATCGACAAAAGAACCATTGAAAAATT
TGAGAAGGAGGCTGCTGAGATGGGAAAGGGC 8 (0.000240%)

CAAGTGCTAACATGCCTTGGTTCAAGGGATGGAAAGTCACCCGTAAGGATGGCAATGCCAGTGGAACCA
CGCTGCTTGAGGCTCTGGACTGCATCCTACC 10 (0.000300%)

CAATCTATCACCCTATAGAAGAACTAATGTTAGTATAAGTAACATGAAAACATTCTCCTCCGCATAAGCC
TGCGTCAGATTAAAACACTGAACTGACAAT 4 (0.000120%)

CAATGATTTTGGGAATTACAACAATCAGTCTTCAAATTTTGGACCCATGAAGGGAGGAAATTTTGGAGG
CAGAAGCTCTGGCCCCTATGGCGGTGGAGGC 16 (0.000481%)

CAATGTCAAGAATGTGTCTGTCAAGGATGTTCGTCGTGGCAACGTTGCTGGTGACAGCAAAAATGACCC
ACCAATGGAAGCAGCTGGCTTCACTGCTCAG 14 (0.000421%)

CAATTTTGGAGGTGGTGGAAGCTACAATGATTTTGGGAATTACAACAATCAGTCTTCAAATTTTGGACCC
ATGAAGGGAGGAAATTTTGGAGGCAGAAGC 29 (0.000871%)

CACAACTAACCTCCTCGGACTCCTGCCTCACTCATTTACACCAACCACCCAACTATCTATAAACCTAGCC
ATGGCCATCCCCTTATGAGCGGGCGCAGTG 8 (0.000240%)

CACACCCAATTGGACCAATCTATCACCCTATAGAAGAACTAATGTTAGTATAAGTAACATGAAAACATT
CTCCTCCGCATAAGCCTGCGTCAGATTAAAA 4 (0.000120%)

CACACTGGGTGTGAAACAACTAATTGTCGGTGTTAACAAAATGGATTCCACTGAGCCACCCTACAGCCA
GAAGAGATATGAGGAAATTGTTAAGGAAGTC 5 (0.000150%)

CACAGAAAGTTCTCCGCTCCCAGACATGGGTCCCTCGGCTTCCTGCCTCGGAAGCGCAGCAGCAGGCAT
CGTGGGAAGGTGAAGAGCTTCCCTAAGGATG 301 (0.009044%)

CACAGAGACTTTATCAAAAACATGATTACAGGGACATCTCAGGCTGACTGTGCTGTCCTGATTGTTGCTG
CTGGTGTTGGTGAATTTGAAGCTGGTATCT 12 (0.000361%)

CACAGCTCTAAGCCTCCTTATTCGAGCCGAGCTGGGCCAGCCAGGCAACCTTCTAGGTAACGACCACATC
TACAACGTTATCGTCACAGCCCATGCATTT 20 (0.000601%)

CACAGGTGTCGTGAAAACTACCCCTAAAAGCCAAAATGGGAAAGGAAAAGACTCATATCAACATTGTCG
TCATTGGACACGTAGATTCGGGCAAGTCCAC 152 (0.004567%)

CACATACTTCCTATTCTACACCCTAGTAGGCTCCCTTCCCCTACTCATCGCACTGATTTACACTCACAACA
CCCTAGGCTCACTAAACATTCTACTACTC 5 (0.000150%)

CACCAAAGCCCATAAAAATAAAAAATTATAACAAACCCTGAGAACCAAAATGAACGAAAATCTGTTCGC
TTCATTCATTGCCCCCACAATCCTAGGCCTA 9 (0.000270%)

CACCAACAGCAGCAACAGCCACCACCGCAGCAGCCACCGCCGCAGCAGCCGCCACCGCATCAGCCGCC
GCCGCATCCACAGCCGCATCAGCAGCAGCAGC 25 (0.000751%)

CACCAACCACCCAACTATCTATAAACCTAGCCATGGCCATCCCCTTATGAGCGGGCGCAGTGATTATAGG
CTTTCGCTCTAAGATTAAAAATGCCCTAGC 9 (0.000270%)

CACCACTACTGGCCATCTGATCTATAAATGCGGTGGCATCGACAAAAGAACCATTGAAAAATTTGAGAA
GGAGGCTGCTGAGATGGGAAAGGGCTCCTTC 17 (0.000511%)

CACCATATATTTACAGTAGGAATAGACGTAGACACACGAGCATATTTCACCTCCGCTACCATAATCATCG
CTATCCCCACCGGCGTCAAAGTATTTAGCT 430 (0.012920%)

CACCATTGATATCTCCTTGTGGAAATTTGAGACCAGCAAGTACTATGTGACTATCATTGATGCCCCAGGA
CACAGAGACTTTATCAAAAACATGATTACA 11 (0.000331%)

CACCCAACTATCTATAAACCTAGCCATGGCCATCCCCTTATGAGCGGGCGCAGTGATTATAGGCTTTCGC
TCTAAGATTAAAAATGCCCTAGCCCACTTC 10 (0.000300%)

CACCCGTAACCCACCGCCATGGCCGAGGAAGGCATTGCTGCTGGAGGTGTAATGGACGTTAATACTGCT
TTACAAGAGGTTCTGAAGACTGCCCTCATCC 9 (0.000270%)

CACCTCCCATTCCGATAAAATCACCTTCCACCCTTACTACACAATCAAAGACGCCCTCGGCTTACTTCTCT
TCATTCTCTCCTTAATGACATTAACACTA 3 (0.000090%)

CACCTTCCACCCTTACTACACAATCAAAGACGCCCTCGGCTTACTTCTCTTCATTCTCTCCTTAATGACAT
TAACACTATTCTCACCAGACCTCCTAGGC 5 (0.000150%)

CACCTTTGCTCCAGTCAACGTTACAACGGAAGTAAAATCTGTCGAAATGCACCATGAAGCTTTGAGTGA

AGCTCTTCCTGGGGACAATGTGGGCTTCAAT 23 (0.000691%)

CACTACTGGCCATCTGATCTATAAATGCGGTGGCATCGACAAAAGAACCATTGAAAAATTTGAGAAGGA
GGCTGCTGAGATGGGAAAGGGCTCCTTCAAG 14 (0.000421%)

CACTCATTTACACCAACCACCCAACTATCTATAAACCTAGCCATGGCCATCCCCTTATGAGCGGGCGCAG
TGATTATAGGCTTTCGCTCTAAGATTAAAA 6 (0.000180%)

CAGAAAGTTCTCCGCTCCCAGACATGGGTCCCTCGGCTTCCTGCCTCGGAAGCGCAGCAGCAGGCATCGT
GGGAAGGTGAAGAGCTTCCCTAAGGATGAC 13 (0.000391%)

CAGAACACAGGTGTCGTGAAAACTACCCCTAAAAGCCAAAATGGGAAAGGAAAAGACTCATATCAACA
TTGTCGTCATTGGACACGTAGATTCGGGCAAG 176 (0.005288%)

CAGAACGCCTGAACGCAGGCACATACTTCCTATTCTACACCCTAGTAGGCTCCCTTCCCCTACTCATCGC
ACTGATTTACACTCACAACACCCTAGGCTC 5 (0.000150%)

CAGAAGTTTTTTCTTCGCAGGATTTTTCTGAGCCTTTTACCACTCCAGCCTAGCCCCTACCCCCCAATTA
GGAGGGCACTGGCCCCCAACAGGCATCAC 6 (0.000180%)

CAGACAGTTGCGGTGGGTGTCATCAAAGCAGTGGACAAGAAGGCTGCTGGAGCTGGCAAGGTCACCAA
GTCTGCCCAGAAAGCTCAGAAGGCTAAATGAA 153 (0.004597%)

CAGACATGGGTCCCTCGGCTTCCTGCCTCGGAAGCGCAGCAGCAGGCATCGTGGGAAGGTGAAGAGCTT
CCCTAAGGATGACCCGTCCAAGCCGGTCCAC 8 (0.000240%)

CAGACCCGAGAGCATGCCCTTCTGGCTTACACACTGGGTGTGAAACAACTAATTGTCGGTGTTAACAAA
ATGGATTCCACTGAGCCACCCTACAGCCAGA 10 (0.000300%)

CAGACTATCCACCTTTGGGTCGCTTTGCTGTTCGTGATATGAGACAGACAGTTGCGGTGGGTGTCATCAA
AGCAGTGGACAAGAAGGCTGCTGGAGCTGG 9 (0.000270%)

CAGAGACTTTATCAAAAACATGATTACAGGGACATCTCAGGCTGACTGTGCTGTCCTGATTGTTGCTGCT
GGTGTTGGTGAATTTGAAGCTGGTATCTCC 22 (0.000661%)

CAGAGGAAGGGCGCCGGGTCTGTGTTCCGCGCGCACGTGAAGCACCGTAAAGGCGCTGCGCGCCTGCGC
GCCGTGGATTTCGCTGAGCGGCACGGCTACA 3 (0.000090%)

CAGCAAGTACTATGTGACTATCATTGATGCCCCAGGACACAGAGACTTTATCAAAAACATGATTACAGG
GACATCTCAGGCTGACTGTGCTGTCCTGATT 11 (0.000331%)

CAGCACCAGCGGTGGCAGAGACCCCAGACATCAAGCTCTTTGGGAAGTGGAGCACCGATGATGTGCAGA
TCAATGACATTTCCCTGCAGGATTACATTGC 7 (0.000210%)

CAGCAGCACCAGCGGTGGCAGAGACCCCAGACATCAAGCTCTTTGGGAAGTGGAGCACCGATGATGTGC
AGATCAATGACATTTCCCTGCAGGATTACAT 48 (0.001442%)

CAGCCCATGACCCCTAACAGGGGCCCTCTCAGCCCTCCTAATGACCTCCGGCCTAGCCATGTGATTTCAC
TTCCACTCCATAACGCTCCTCATACTAGGC 24 (0.000721%)

CAGCCCTCCTAATGACCTCCGGCCTAGCCATGTGATTTCACTTCCACTCCATAACGCTCCTCATACTAGGC
CTACTAACCAACACACTAACCATATACCA 13 (0.000391%)

CAGCTCCTCCCTGGAGAAGAGCTACGAGCTGCCTGACGGCCAGGTCATCACCATTGGCAATGAGCGGTT
CCGCTGCCCTGAGGCACTCTTCCAGCCTTCC 37 (0.001112%)

CAGCTCTAAGCCTCCTTATTCGAGCCGAGCTGGGCCAGCCAGGCAACCTTCTAGGTAACGACCACATCTA
CAACGTTATCGTCACAGCCCATGCATTTGT 23 (0.000691%)

CAGGACACAGAGACTTTATCAAAAACATGATTACAGGGACATCTCAGGCTGACTGTGCTGTCCTGATTGT
TGCTGCTGGTGTTGGTGAATTTGAAGCTGG 9 (0.000270%)

CAGGCACATACTTCCTATTCTACACCCTAGTAGGCTCCCTTCCCCTACTCATCGCACTGATTTACACTCAC
AACACCCTAGGCTCACTAAACATTCTACT 13 (0.000391%)

CAGGCTGAAGCGCAAAAGAAGAAAGATGAGGCAGAGGTCCAAGTAAACCGCTAGCTTGTTGCACCGTG
GAGGCCACAGGAGCAGAAACATGGAATGCCAG 11 (0.000331%)

CAGGCTGACTGTGCTGTCCTGATTGTTGCTGCTGGTGTTGGTGAATTTGAAGCTGGTATCTCCAAGAATG
GGCAGACCCGAGAGCATGCCCTTCTGGCTT 12 (0.000361%)

CAGGCTTCGGAATAATCTCCCATATTGTAACTTACTACTCCGGAAAAAAAGAACCATTTGGATACATAGG
TATGGTCTGAGCTATGATATCAATTGGCTT 3 (0.000090%)

CAGGGATTGTGTTTAAAGTAGTGCTTCTACCAACATGTCCCGTGGTTCCAGCGCCGGTTTTGACCGCCAC
ATTACCATTTTTTCACCCGAGGGTCGGCTC 16 (0.000481%)

CAGGGGCCCTCTCAGCCCTCCTAATGACCTCCGGCCTAGCCATGTGATTTCACTTCCACTCCATAACGCT
CCTCATACTAGGCCTACTAACCAACACACT 14 (0.000421%)

CAGGTGTCGTGAAAACTACCCCTAAAAGCCAAAATGGGAAAGGAAAAGACTCATATCAACATTGTCGTC
ATTGGACACGTAGATTCGGGCAAGTCCACCA 70 (0.002103%)

CAGTCCTAGCTGCTGGCATCACTATACTACTAACAGACCGCAACCTCAACACCACCTTCTTCGACCCCGC
CGGAGGAGGAGACCCCATTCTATACCAACA 8 (0.000240%)

CAGTGGACAAGAAGGCTGCTGGAGCTGGCAAGGTCACCAAGTCTGCCCAGAAAGCTCAGAAGGCTAAA
TGAATATTATCCCTAATACCTGCCACCCCACT 14 (0.000421%)

CAGTTGCGGTGGGTGTCATCAAAGCAGTGGACAAGAAGGCTGCTGGAGCTGGCAAGGTCACCAAGTCTG
CCCAGAAAGCTCAGAAGGCTAAATGAATATT 10 (0.000300%)

CATAAAAATAAAAAATTATAACAAACCCTGAGAACCAAAATGAACGAAAATCTGTTCGCTTCATTCATT
GCCCCCACAATCCTAGGCCTACCCGCCGCAG 4 (0.000120%)

CATACTTCCCCCATTATTCCTAGAACCAGGCGACCTGCGACTCCTTGACGTTGACAATCGAGTAGTACTC
CCGATTGAAGCCCCCATTCGTATAATAATT 9 (0.000270%)

CATATATTTACAGTAGGAATAGACGTAGACACACGAGCATATTTCACCTCCGCTACCATAATCATCGCTA
TCCCCACCGGCGTCAAAGTATTTAGCTGAC 7 (0.000210%)

CATATTGTAACTTACTACTCCGGAAAAAAAGAACCATTTGGATACATAGGTATGGTCTGAGCTATGATAT
CAATTGGCTTCCTAGGGTTTATCGTGTGAG 13 (0.000391%)

CATCAAAGCAGTGGACAAGAAGGCTGCTGGAGCTGGCAAGGTCACCAAGTCTGCCCAGAAAGCTCAGA
AGGCTAAATGAATATTATCCCTAATACCTGCC 32 (0.000962%)

CATCATCCTAGTCCTCATCGCCCTCCCATCCCTACGCATCCTTTACATAACAGACGAGGTCAACGATCCC
TCCCTTACCATCAAATCAATTGGCCACCAA 8 (0.000240%)

CATCCCTACGCATCCTTTACATAACAGACGAGGTCAACGATCCCTCCCTTACCATCAAATCAATTGGCCA
CCAATGGTACTGAACCTACGAGTACACCGA 12 (0.000361%)

CATCCCTCTCCAGAAGAGGAGAAGAGGAAACACAAGAAGAAACGCCTGGTGCAGAGCCCCAATTCCTA
CTTCATGGATGTGAAATGCCCAGGATGCTATA 4 (0.000120%)

CATCCTAGTCCTCATCGCCCTCCCATCCCTACGCATCCTTTACATAACAGACGAGGTCAACGATCCCTCC
CTTACCATCAAATCAATTGGCCACCAATGG 5 (0.000150%)

CATCGACAAAAGAACCATTGAAAAATTTGAGAAGGAGGCTGCTGAGATGGAAAGGGCTCCTTCAAGT ATGCCTGGGTCTTGGATAAACTGAAAGCTGAG11 (0.000331%)

CATCGCCCTCCCATCCCTACGCATCCTTTACATAACAGACGAGGTCAACGATCCCTCCCTTACCATCAAA TCAATTGGCCACCAATGGTACTGAACCTAC 9 (0.000270%)

CATGACCCCTAACAGGGGCCCTCTCAGCCCTCCTAATGACCTCCGGCCTAGCCATGTGATTTCACTTCCA CTCCATAACGCTCCTCATACTAGGCCTACT 20 (0.000601%)

CATGATTACAGGGACATCTCAGGCTGACTGTGCTGTCCTGATTGTTGCTGCTGGTGTTGGTGAATTTGAA GCTGGTATCTCCAAGAATGGGCAGACCCGA 36 (0.001082%)

CATGCCCTTCTGGCTTACACACTGGGTGTGAAACAACTAATTGTCGGTGTTAACAAAATGGATTCCACTG AGCCACCCTACAGCCAGAAGAGATATGAGG 3 (0.000090%)

CATTCATTGCCCCCACAATCCTAGGCCTACCCGCCGCAGTACTGATCATTCTATTTCCCCCTCTATTGATC CCCACCTCCAAATATCTCATCAACAACCG 4 (0.000120%)

CATTGATATCTCCTTGTGGAAATTTGAGACCAGCAAGTACTATGTGACTATCATTGATGCCCCAGGACAC AGAGACTTTATCAAAAACATGATTACAGGG 16 (0.000481%)

CATTGATGCCCCAGGACACAGAGACTTTATCAAAAACATGATTACAGGGACATCTCAGGCTGACTGTGC TGTCCTGATTGTTGCTGCTGGTGTTGGTGAA 25 (0.000751%)

CATTGGACACGTAGATTCGGGCAAGTCCACCACTACTGGCCATCTGATCTATAAATGCGGTGGCATCGAC AAAAGAACCATTGAAAAATTTGAGAAGGAG 4 (0.000120%)

CATTGTCGTCATTGGACACGTAGATTCGGGCAAGTCCACCACTACTGGCCATCTGATCTATAAATGCGGT GGCATCGACAAAAGAACCATTGAAAAATTT 1198 (0.035996%)

CATTGTTGATATGGTTCCTGGCAAGCCCATGTGTGTTGAGAGCTTCTCAGACTATCCACCTTTGGGTCGCT TTGCTGTTCGTGATATGAGACAGACAGTT 9 (0.000270%)

CATTTGTGCCAATTTCTGGTTGGAATGGTGACAACATGCTGGAGCCAAGTGCTAACATGCCTTGGTTCAA GGGATGGAAAGTCACCCGTAAGGATGGCAA 4 (0.000120%)

CCAAACATATAACTGAACTCCTCACACCCAATTGGACCAATCTATCACCCTATAGAAGAACTAATGTTAG TATAAGTAACATGAAAACATTCTCCTCCGC 8 (0.000240%)

CCAAAGCCCATAAAAATAAAAAATTATAACAAACCCTGAGAACCAAAATGAACGAAAATCTGTTCGCTT CATTCATTGCCCCCACAATCCTAGGCCTACC 14 (0.000421%)

CCAACAGCAGCAACAGCCACCACCGCAGCAGCCACCGCCGCAGCAGCCGCCACCGCATCAGCCGCCGCC GCATCCACAGCCGCATCAGCAGCAGCAGCCG 3 (0.000090%)

CCAACTATCTATAAACCTAGCCATGGCCATCCCCTTATGAGCGGGCGCAGTGATTATAGGCTTTCGCTCT AAGATTAAAAATGCCCTAGCCCACTTCTTA 3 (0.000090%)

CCAAGTGCTAACATGCCTTGGTTCAAGGGATGGAAAGTCACCCGTAAGGATGGCAATGCCAGTGGAACC ACGCTGCTTGAGGCTCTGGACTGCATCCTAC 3 (0.000090%)

CCAATCTATCACCCTATAGAAGAACTAATGTTAGTATAAGTAACATGAAAACATTCTCCTCCGCATAAGC CTGCGTCAGATTAAAACACTGAACTGACAA 3 (0.000090%)

CCAATTGGACCAATCTATCACCCTATAGAAGAACTAATGTTAGTATAAGTAACATGAAAACATTCTCCTC CGCATAAGCCTGCGTCAGATTAAAACACTG 7 (0.000210%)

CCACAACTAACCTCCTCGGACTCCTGCCTCACTCATTTACACCAACCACCCAACTATCTATAAACCTAGC

CATGGCCATCCCCTTATGAGCGGGCGCAGT 11 (0.000331%)

CCACCACTACTGGCCATCTGATCTATAAATGCGGTGGCATCGACAAAAGAACCATTGAAAAATTTGAGA
AGGAGGCTGCTGAGATGGGAAAGGGCTCCTT 6 (0.000180%)

CCACCCAACTATCTATAAACCTAGCCATGGCCATCCCCTTATGAGCGGGCGCAGTGATTATAGGCTTTCG
CTCTAAGATTAAAAATGCCCTAGCCCACTT 5 (0.000150%)

CCACCTTTGGGTCGCTTTGCTGTTCGTGATATGAGACAGACAGTTGCGGTGGGTGTCATCAAAGCAGTGG
ACAAGAAGGCTGCTGGAGCTGGCAAGGTCA 10 (0.000300%)

CCACTACTGGCCATCTGATCTATAAATGCGGTGGCATCGACAAAAGAACCATTGAAAAATTTGAGAAGG
AGGCTGCTGAGATGGGAAAGGGCTCCTTCAA 23 (0.000691%)

CCAGAACACAGGTGTCGTGAAAACTACCCCTAAAAGCCAAAATGGGAAAGGAAAAGACTCATATCAAC
ATTGTCGTCATTGGACACGTAGATTCGGGCAA 277 (0.008323%)

CCAGAACGCCTGAACGCAGGCACATACTTCCTATTCTACACCCTAGTAGGCTCCCTTCCCCTACTCATCG
CACTGATTTACACTCACAACACCCTAGGCT 6 (0.000180%)

CCAGACATGGGTCCCTCGGCTTCCTGCCTCGGAAGCGCAGCAGCAGGCATCGTGGGAAGGTGAAGAGCT
TCCCTAAGGATGACCCGTCCAAGCCGGTCCA 3 (0.000090%)

CCAGCAAGTACTATGTGACTATCATTGATGCCCCAGGACACAGAGACTTTATCAAAAACATGATTACAG
GGACATCTCAGGCTGACTGTGCTGTCCTGAT 12 (0.000361%)

CCAGCCCATGACCCCTAACAGGGGCCCTCTCAGCCCTCCTAATGACCTCCGGCCTAGCCATGTGATTTCA
CTTCCACTCCATAACGCTCCTCATACTAGG 15 (0.000451%)

CCAGGACACAGAGACTTTATCAAAAACATGATTACAGGGACATCTCAGGCTGACTGTGCTGTCCTGATT
GTTGCTGCTGGTGTTGGTGAATTTGAAGCTG 14 (0.000421%)

CCAGGCGACCTGCGACTCCTTGACGTTGACAATCGAGTAGTACTCCCGATTGAAGCCCCCATTCGTATAA
TAATTACATCACAAGACGTCTTGCACTCAT 7 (0.000210%)

CCAGGCTTCGGAATAATCTCCCATATTGTAACTTACTACTCCGGAAAAAAAGAACCATTTGGATACATAG
GTATGGTCTGAGCTATGATATCAATTGGCT 5 (0.000150%)

CCAGTCCTAGCTGCTGGCATCACTATACTACTAACAGACCGCAACCTCAACACCACCTTCTTCGACCCCG
CCGGAGGAGGAGACCCCATTCTATACCAAC 7 (0.000210%)

CCATAAAAATAAAAAATTATAACAAACCCTGAGAACCAAAATGAACGAAAATCTGTTCGCTTCATTCAT
TGCCCCCACAATCCTAGGCCTACCCGCCGCA 3 (0.000090%)

CCATATTGTAACTTACTACTCCGGAAAAAAAGAACCATTTGGATACATAGGTATGGTCTGAGCTATGATA
TCAATTGGCTTCCTAGGGTTTATCGTGTGA 28 (0.000841%)

CCATCCCTACGCATCCTTTACATAACAGACGAGGTCAACGATCCCTCCCTTACCATCAAATCAATTGGCC
ACCAATGGTACTGAACCTACGAGTACACCG 4 (0.000120%)

CCATGACCCCTAACAGGGGCCCTCTCAGCCCTCCTAATGACCTCCGGCCTAGCCATGTGATTTCACTTCC
ACTCCATAACGCTCCTCATACTAGGCCTAC 15 (0.000451%)

CCATGTTGGCATCTGCCCCTCCTCAAGAGCAAAAGCAAATGTTGGGTGAACGGCTGTTTCCTCTTATTCA
AGCCATGCACCCTACTCTTGCTGGTAAAAT 7 (0.000210%)

CCATTGTTGATATGGTTCCTGGCAAGCCCATGTGTGTTGAGAGCTTCTCAGACTATCCACCTTTGGGTCGC
TTTGCTGTTCGTGATATGAGACAGACAGT 4 (0.000120%)

CCCAAACATATAACTGAACTCCTCACACCCAATTGGACCAATCTATCACCCTATAGAAGAACTAATGTTA
GTATAAGTAACATGAAAACATTCTCCTCCG 581 (0.017457%)

CCCAACTATCTATAAACCTAGCCATGGCCATCCCCTTATGAGCGGGCGCAGTGATTATAGGCTTTCGCTC
TAAGATTAAAAATGCCCTAGCCCACTTCTT 29 (0.000871%)

CCCAATTGGACCAATCTATCACCCTATAGAAGAACTAATGTTAGTATAAGTAACATGAAAACATTCTCCT
CCGCATAAGCCTGCGTCAGATTAAAACACT 19 (0.000571%)

CCCAGACATGGGTCCCTCGGCTTCCTGCCTCGGAAGCGCAGCAGCAGGCATCGTGGAAGGTGAAGAGC
TTCCCTAAGGATGACCCGTCCAAGCCGGTCC 5 (0.000150%)

CCCAGCCCATGACCCCTAACAGGGGCCCTCTCAGCCCTCCTAATGACCTCCGGCCTAGCCATGTGATTTC
ACTTCCACTCCATAACGCTCCTCATACTAG 32 (0.000962%)

CCCAGGACACAGAGACTTTATCAAAAACATGATTACAGGGACATCTCAGGCTGACTGTGCTGTCCTGATT
GTTGCTGCTGGTGTTGGTGAATTTGAAGCT 5 (0.000150%)

CCCAGGGATTGTGTTTAAAGTAGTGCTTCTACCAACATGTCCCGTGGTTCCAGCGCCGGTTTTGACCGCC
ACATTACCATTTTTTCACCCGAGGGTCGGC 3 (0.000090%)

CCCAGTCCTAGCTGCTGGCATCACTATACTACTAACAGACCGCAACCTCAACACCACCTTCTTCGACCCC
GCCGGAGGAGGAGACCCCATTCTATACCAA 24 (0.000721%)

CCCATATTGTAACTTACTACTCCGGAAAAAAAGAACCATTTGGATACATAGGTATGGTCTGAGCTATGAT
ATCAATTGGCTTCCTAGGGTTTATCGTGTG 25 (0.000751%)

CCCATCCCTACGCATCCTTTACATAACAGACGAGGTCAACGATCCCTCCCTTACCATCAAATCAATTGGC
CACCAATGGTACTGAACCTACGAGTACACC 24 (0.000721%)

CCCATGACCCCTAACAGGGGCCCTCTCAGCCCTCCTAATGACCTCCGGCCTAGCCATGTGATTTCACTTC
CACTCCATAACGCTCCTCATACTAGGCCTA 26 (0.000781%)

CCCATGTGTGTTGAGAGCTTCTCAGACTATCCACCTTTGGGTCGCTTTGCTGTTCGTGATATGAGACAGA
CAGTTGCGGTGGGTGTCATCAAAGCAGTGG 9 (0.000270%)

CCCCAACGTTGTAGGCCCCTACGGGCTACTACAACCCTTCGCTGACGCCATAAAACTCTTCACCAAAGAG
CCCCTAAAACCCGCCACATCTACCATCACC 27 (0.000811%)

CCCCATTCTATACCAACACCTATTCTGATTTTTCGGTCACCCTGAAGTTTATATTCTTATCCTACCAGGCT
TCGGAATAATCTCCCATATTGTAACTTAC 33 (0.000992%)

CCCCCCCCCCCCCCCCCCCCCCCCCCCCCCCCCCCCCCCCCCCCCCCCCCCCCCCCCCCCCCCCCCCCCC
CCCCCCCCCCCCCCCCCCCCCCCCCCCCCC 76 (0.002284%)

CCCCGATATGGCGTTTCCCCGCATAAACAACATAAGCTTCTGACTCTTACCTCCCTCTCTCCTACTCCTGC
TCGCATCTGCTATAGTGGAGGCCGGAGCA 9 (0.000270%)

CCCCTAACAGGGGCCCTCTCAGCCCTCCTAATGACCTCCGGCCTAGCCATGTGATTTCACTTCCACTCCAT
AACGCTCCTCATACTAGGCCTACTAACCA 8 (0.000240%)

CCCGAGAGCATGCCCTTCTGGCTTACACACTGGGTGTGAAACAACTAATTGTCGGTGTTAACAAAATGG
ATTCCACTGAGCCACCCTACAGCCAGAAGAG 22 (0.000661%)

CCCGATATATGTTCTCTAGGCCTTTTAGAAAACATGGAGTTGTTCCTTTGGCCACATATATGCGAATCTAT
AAGAAAGGTGATATTGTAGACATCAAGGG 6 (0.000180%)

CCCGATATGGCGTTTCCCCGCATAAACAACATAAGCTTCTGACTCTTACCTCCCTCTCTCCTACTCCTGCT
CGCATCTGCTATAGTGGAGGCCGGAGCAG 27 (0.000811%)

CCCGCCATCATCCTAGTCCTCATCGCCCTCCCATCCCTACGCATCCTTTACATAACAGACGAGGTCAACG ATCCCTCCCTTACCATCAAATCAATTGGCC 6 (0.000180%)

CCCGGTATGGTGGTCACCTTTGCTCCAGTCAACGTTACAACGGAAGTAAAATCTGTCGAAATGCACCATG AAGCTTTGAGTGAAGCTCTTCCTGGGGACA 15 (0.000451%)

CCCTAAATTCTTGAAGTCTGGTGATGCTGCCATTGTTGATATGGTTCCTGGCAAGCCCATGTGTGTTGAG AGCTTCTCAGACTATCCACCTTTGGGTCGC 3 (0.000090%)

CCCTAACAGGGGCCCTCTCAGCCCTCCTAATGACCTCCGGCCTAGCCATGTGATTTCACTTCCACTCCAT AACGCTCCTCATACTAGGCCTACTAACCAA 34 (0.001022%)

CCCTAATAATCGGTGCCCCCGATATGGCGTTTCCCCGCATAAACAACATAAGCTTCTGACTCTTACCTCC CTCTCTCCTACTCCTGCTCGCATCTGCTAT 10 (0.000300%)

CCCTACGCATCCTTTACATAACAGACGAGGTCAACGATCCCTCCCTTACCATCAAATCAATTGGCCACCA ATGGTACTGAACCTACGAGTACACCGACTA 5 (0.000150%)

CCCTAGTAGGCTCCCTTCCCCTACTCATCGCACTGATTTACACTCACAACACCCTAGGCTCACTAAACATT CTACTACTCACTCTCACTGCCCAAGAACT 6 (0.000180%)

CCCTCACCAAAGCCCATAAAAATAAAAAATTATAACAAACCCTGAGAACCAAAATGAACGAAAATCTGT TCGCTTCATTCATTGCCCCCACAATCCTAGG 10 (0.000300%)

CCCTCCACTTCCCGTCTCAGAATCTAAACGTGGTCACCTTCGAGTAGAGAGGCCCGCCCGCCCACCGTGG GCAGTGCCACCCGCAGATGACACGCGCTCT 3 (0.000090%)

CCCTCCCATCCCTACGCATCCTTTACATAACAGACGAGGTCAACGATCCCTCCCTTACCATCAAATCAAT TGGCCACCAATGGTACTGAACCTACGAGTA 8 (0.000240%)

CCCTCTCAGCCCTCCTAATGACCTCCGGCCTAGCCATGTGATTTCACTTCCACTCCATAACGCTCCTCATA CTAGGCCTACTAACCAACACACTAACCAT 7 (0.000210%)

CCCTTCTGGCTTACACACTGGGTGTGAAACAACTAATTGTCGGTGTTAACAAAATGGATTCCACTGAGCC ACCCTACAGCCAGAAGAGATATGAGGAAAT 7 (0.000210%)

CCGACTAATCACCACCCAACAATGACTAATCAAACTAACCTCAAAACAAATGATAACCATACACAACAC TAAAGGACGAACCTGATCTCTTATACTAGTA 11 (0.000331%)

CCGAGAGCATGCCCTTCTGGCTTACACACTGGGTGTGAAACAACTAATTGTCGGTGTTAACAAAATGGAT TCCACTGAGCCACCCTACAGCCAGAAGAGA 51 (0.001532%)

CCGATATATGTTCTCTAGGCCTTTTAGAAAACATGGAGTTGTTCCTTTGGCCACATATATGCGAATCTATA AGAAAGGTGATATTGTAGACATCAAGGGA 20 (0.000601%)

CCGATGGTTACTTGCTTCGTCTGTTCTGTGTTGGTTTTACTAAAAAACGCAACAATCAGATACGGAAGAC CTCTTATGCTCAGCACCAACAGGTCCGCCA 6 (0.000180%)

CCGCAAACTCTGTCTCAACATCTGTGTTGGGGAGAGTGGAGACAGACTGACGCGAGCAGCCAAGGTGTT GGAGCAGCTCACAGGGCAGACCCCTGTGTTT 3 (0.000090%)

CCGCCATCATCCTAGTCCTCATCGCCCTCCCATCCCTACGCATCCTTTACATAACAGACGAGGTCAACGA TCCCTCCCTTACCATCAAATCAATTGGCCA 22 (0.000661%)

CCGCGCGCACGTGAAGCACCGTAAAGGCGCTGCGCGCCTGCGCGCCGTGGATTTCGCTGAGCGGCACGG CTACATCAAGGGCATCGTCAAGGACATCATC 7 (0.000210%)

CCGCTCCCAGACATGGGTCCCTCGGCTTCCTGCCTCGGAAGCGCAGCAGCAGGCATCGTGGGAAGGTGA

AGAGCTTCCCTAAGGATGACCCGTCCAAGCC 5 (0.000150%)

CCGGACGGGCACTGGGCGACTCTGTGCCTCGCTGAGGAAAAATAACTAAACATGGGCAAAGGAGATCCT
AAGAAGCCGAGAGGCAAAATGTCATCATATG 9 (0.000270%)

CCGGGTCTGTGTTCCGCGCGCACGTGAAGCACCGTAAAGGCGCTGCGCGCCTGCGCGCCGTGGATTTCG
CTGAGCGGCACGGCTACATCAAGGGCATCGT 9 (0.000270%)

CCGGTATGGTGGTCACCTTTGCTCCAGTCAACGTTACAACGGAAGTAAAATCTGTCGAAATGCACCATGA
AGCTTTGAGTGAAGCTCTTCCTGGGGACAA 31 (0.000931%)

CCGTAAAGGCGCTGCGCGCCTGCGCGCCGTGGATTTCGCTGAGCGGCACGGCTACATCAAGGGCATCGT
CAAGGACATCATCCACGACCCGGGCCGCGGC 6 (0.000180%)

CCGTCTGAACTATCCTGCCCGCCATCATCCTAGTCCTCATCGCCCTCCCATCCCTACGCATCCTTTACATA
ACAGACGAGGTCAACGATCCCTCCCTTAC 3 (0.000090%)

CCGTGGACAGAGGAAGGGCGCCGGGTCTGTGTTCCGCGCGCACGTGAAGCACCGTAAAGGCGCTGCGCG
CCTGCGCGCCGTGGATTTCGCTGAGCGGCAC 6 (0.000180%)

CCGTTCTGGTAAAAAGCTGGAAGATGGCCCTAAATTCTTGAAGTCTGGTGATGCTGCCATTGTTGATATG
GTTCCTGGCAAGCCCATGTGTGTTGAGAGC 1156 (0.034734%)

CCTAAATTCTTGAAGTCTGGTGATGCTGCCATTGTTGATATGGTTCCTGGCAAGCCCATGTGTGTTGAGA
GCTTCTCAGACTATCCACCTTTGGGTCGCT 39 (0.001172%)

CCTAACAGGGGCCCTCTCAGCCCTCCTAATGACCTCCGGCCTAGCCATGTGATTTCACTTCCACTCCATA
ACGCTCCTCATACTAGGCCTACTAACCAAC 16 (0.000481%)

CCTAATAATCGGTGCCCCCGATATGGCGTTTCCCCGCATAAACAACATAAGCTTCTGACTCTTACCTCCC
TCTCTCCTACTCCTGCTCGCATCTGCTATA 7 (0.000210%)

CCTAATGACCTCCGGCCTAGCCATGTGATTTCACTTCCACTCCATAACGCTCCTCATACTAGGCCTACTAA
CCAACACACTAACCATATACCAATGATGG 5 (0.000150%)

CCTACATACTTCCCCCATTATTCCTAGAACCAGGCGACCTGCGACTCCTTGACGTTGACAATCGAGTAGT
ACTCCCGATTGAAGCCCCCATTCGTATAAT 459 (0.013792%)

CCTACGCATCCTTTACATAACAGACGAGGTCAACGATCCCTCCCTTACCATCAAATCAATTGGCCACCAA
TGGTACTGAACCTACGAGTACACCGACTAC 16 (0.000481%)

CCTAGAACCAGGCGACCTGCGACTCCTTGACGTTGACAATCGAGTAGTACTCCCGATTGAAGCCCCCATT
CGTATAATAATTACATCACAAGACGTCTTG 34 (0.001022%)

CCTAGCTGCTGGCATCACTATACTACTAACAGACCGCAACCTCAACACCACCTTCTTCGACCCCGCCGGA
GGAGGAGACCCCATTCTATACCAACACCTA 14 (0.000421%)

CCTAGTAGGCTCCCTTCCCCTACTCATCGCACTGATTTACACTCACAACACCCTAGGCTCACTAAACATTC
TACTACTCACTCTCACTGCCCAAGAACTA 3 (0.000090%)

CCTAGTCCTCATCGCCCTCCCATCCCTACGCATCCTTTACATAACAGACGAGGTCAACGATCCCTCCCTTA
CCATCAAATCAATTGGCCACCAATGGTAC 12 (0.000361%)

CCTATCTCTCCCAGTCCTAGCTGCTGGCATCACTATACTACTAACAGACCGCAACCTCAACACCACCTTC
TTCGACCCCGCCGGAGGAGGAGACCCCATT 15 (0.000451%)

CCTATTCTACACCCTAGTAGGCTCCCTTCCCCTACTCATCGCACTGATTTACACTCACAACACCCTAGGCT
CACTAAACATTCTACTACTCACTCTCACT 3 (0.000090%)

CCTATTTATTACCTCAGAAGTTTTTTCTTCGCAGGATTTTCTGAGCCTTTTACCACTCCAGCCTAGCCCC
TACCCCCCAATTAGGAGGGCACTGGCCC 8 (0.000240%)

CCTCACCAAAGCCCATAAAAATAAAAAATTATAACAAACCCTGAGAACCAAAATGAACGAAAATCTGTT
CGCTTCATTCATTGCCCCCACAATCCTAGGC 45 (0.001352%)

CCTCACTCATTTACACCAACCACCCAACTATCTATAAACCTAGCCATGGCCATCCCCTTATGAGCGGGCG
CAGTGATTATAGGCTTTCGCTCTAAGATTA 7 (0.000210%)

CCTCAGAAGTTTTTTCTTCGCAGGATTTTCTGAGCCTTTTACCACTCCAGCCTAGCCCCTACCCCCCAA
TTAGGAGGGCACTGGCCCCCAACAGGCAT 5 (0.000150%)

CCTCATCGCCCTCCCATCCCTACGCATCCTTTACATAACAGACGAGGTCAACGATCCCTCCCTTACCATC
AAATCAATTGGCCACCAATGGTACTGAACC 19 (0.000571%)

CCTCCCATCCCTACGCATCCTTTACATAACAGACGAGGTCAACGATCCCTCCCTTACCATCAAATCAATT
GGCCACCAATGGTACTGAACCTACGAGTAC 3 (0.000090%)

CCTCCCTGGAGAAGAGCTACGAGCTGCCTGACGGCCAGGTCATCACCATTGGCAATGAGCGGTTCCGCT
GCCCTGAGGCACTCTTCCAGCCTTCCTTCCT 3 (0.000090%)

CCTCCTAATGACCTCCGGCCTAGCCATGTGATTTCACTTCCACTCCATAACGCTCCTCATACTAGGCCTAC
TAACCAACACACTAACCATATACCAATGA 4 (0.000120%)

CCTCCTTATTCGAGCCGAGCTGGGCCAGCCAGGCAACCTTCTAGGTAACGACCACATCTACAACGTTATC
GTCACAGCCCATGCATTTGTAATAATCTTC 8 (0.000240%)

CCTCGCTGAGGAAAAATAACTAAACATGGGCAAAGGAGATCCTAAGAAGCCGAGAGGCAAAATGTCAT
CATATGCATTTTTTGTGCAAACTTGTCGGGAG 3 (0.000090%)

CCTCGGACTCCTGCCTCACTCATTTACACCAACCACCCAACTATCTATAAACCTAGCCATGGCCATCCCC
TTATGAGCGGGCGCAGTGATTATAGGCTTT 25 (0.000751%)

CCTCGGCTTCCTGCCTCGGAAGCGCAGCAGCAGGCATCGTGGGAAGGTGAAGAGCTTCCCTAAGGATGA
CCCGTCCAAGCCGGTCCACCTCACAGCCTTC 6 (0.000180%)

CCTCTCGCAAAGGATCTCCTTCATCCCTCTCCAGAAGAGGAGAAGAGGAAACACAAGAAGAAACGCCTG
GTGCAGAGCCCCAATTCCTACTTCATGGATG 19 (0.000571%)

CCTGAACGCAGGCACATACTTCCTATTCTACACCCTAGTAGGCTCCCTTCCCCTACTCATCGCACTGATTT
ACACTCACAACACCCTAGGCTCACTAAAC 33 (0.000992%)

CCTGATTGTTGCTGCTGGTGTTGGTGAATTTGAAGCTGGTATCTCCAAGAATGGGCAGACCCGAGAGCAT
GCCCTTCTGGCTTACACACTGGGTGTGAAA 40 (0.001202%)

CCTGCCCGCCATCATCCTAGTCCTCATCGCCCTCCCATCCCTACGCATCCTTTACATAACAGACGAGGTC
AACGATCCCTCCCTTACCATCAAATCAATT 4 (0.000120%)

CCTGCCTCACTCATTTACACCAACCACCCAACTATCTATAAACCTAGCCATGGCCATCCCCTTATGAGCG
GGCGCAGTGATTATAGGCTTTCGCTCTAAG 17 (0.000511%)

CCTGGCAAGCCCATGTGTGTTGAGAGCTTCTCAGACTATCCACCTTTGGGTCGCTTTGCTGTTCGTGATAT
GAGACAGACAGTTGCGGTGGGTGTCATCA31 (0.000931%)

CCTGGGTCTTGGATAAACTGAAAGCTGAGCGTGAACGTGGTATCACCATTGATATCTCCTTGTGGAAATT
TGAGACCAGCAAGTACTATGTGACTATCAT 39 (0.001172%)

CCTTAATCATTTTTATTGCCACAACTAACCTCCTCGGACTCCTGCCTCACTCATTTACACCAACCACCCAA
CTATCTATAAACCTAGCCATGGCCATCCC 344 (0.010336%)

CCTTATTCGAGCCGAGCTGGGCCAGCCAGGCAACCTTCTAGGTAACGACCACATCTACAACGTTATCGTC
ACAGCCCATGCATTTGTAATAATCTTCTTC11 (0.000331%)

CCTTCAAGTATGCCTGGGTCTTGGATAAACTGAAAGCTGAGCGTGAACGTGGTATCACCATTGATATCTC
CTTGTGGAAATTTGAGACCAGCAAGTACTA 16 (0.000481%)

CCTTCATCCCTCTCCAGAAGAGGAGAAGAGGAAACACAAGAAGAAACGCCTGGTGCAGAGCCCCAATTC
CTACTTCATGGATGTGAAATGCCCAGGATGC 7 (0.000210%)

CCTTCTGGCTTACACACTGGGTGTGAAACAACTAATTGTCGGTGTTAACAAAATGGATTCCACTGAGCCA
CCCTACAGCCAGAAGAGATATGAGGAAATT 3 (0.000090%)

CCTTGTGGAAATTTGAGACCAGCAAGTACTATGTGACTATCATTGATGCCCCAGGACACAGAGACTTTAT
CAAAAACATGATTACAGGGACATCTCAGGC 12 (0.000361%)

CCTTTACATAACAGACGAGGTCAACGATCCCTCCCTTACCATCAAATCAATTGGCCACCAATGGTACTGA
ACCTACGAGTACACCGACTACGGCGGACTA 14 (0.000421%)

CCTTTGACTGCTTCCATGTTGGCATCTGCCCCTCCTCAAGAGCAAAAGCAAATGTTGGGTGAACGGCTGT
TTCCTCTTATTCAAGCCATGCACCCTACTC5 (0.000150%)

CGAAAATCTGTTCGCTTCATTCATTGCCCCCACAATCCTAGGCCTACCCGCCGCAGTACTGATCATTCTAT
TTCCCCCTCTATTGATCCCCACCTCCAAA 695 (0.020883%)

CGAACCTGATCTCTTATACTAGTATCCTTAATCATTTTTATTGCCACAACTAACCTCCTCGGACTCCTGCC
TCACTCATTTACACCAACCACCCAACTAT 738 (0.022175%)

CGAATGCGCAGGCTGAAGCGCAAAAGAAGAAAGATGAGGCAGAGGTCCAAGTAAACCGCTAGCTTGTT
GCACCGTGGAGGCCACAGGAGCAGAAACATGG 46 (0.001382%)

CGACAAAAGAACCATTGAAAAATTTGAGAAGGAGGCTGCTGAGATGGGAAAGGGCTCCTTCAAGTATG
CCTGGGTCTTGGATAAACTGAAAGCTGAGCGT47 (0.001412%)

CGACTAATCACCACCCAACAATGACTAATCAAACTAACCTCAAAACAAATGATAACCATACACAACACT
AAAGGACGAACCTGATCTCTTATACTAGTAT 39 (0.001172%)

CGACTCTGTGCCTCGCTGAGGAAAAATAACTAAACATGGGCAAAGGAGATCCTAAGAAGCCGAGAGGC
AAAATGTCATCATATGCATTTTTGTGCAAAC 11 (0.000331%)

CGAGAGCATGCCCTTCTGGCTTACACACTGGGTGTGAAACAACTAATTGTCGGTGTTAACAAAATGGATT
CCACTGAGCCACCCTACAGCCAGAAGAGAT 93 (0.002794%)

CGAGGCCATCTCCTGGGCCGCCTGGCGGCCATCGTGGCTAAACAGGTACTGCTGGGCCGGAAGGTGGTG
GTCGTACGCTGTGAAGGCATCAACATTTCTG 26 (0.000781%)

CGAGGTCAACGATCCCTCCCTTACCATCAAATCAATTGGCCACCAATGGTACTGAACCTACGAGTACACC
GACTACGGCGGACTAATCTTCAACTCCTAC 84 (0.002524%)

CGAGTGGAGACTGGTGTTCTCAAACCCGGTATGGTGGTCACCTTTGCTCCAGTCAACGTTACAACGGAAG
TAAAATCTGTCGAAATGCACCATGAAGCTT 18 (0.000541%)

CGATATATGTTCTCTAGGCCTTTTAGAAAACATGGAGTTGTTCCTTTGGCCACATATATGCGAATCTATA
AGAAAGGTGATATTGTAGACATCAAGGGAA 3 (0.000090%)

CGATATGGCGTTTCCCCGCATAAACAACATAAGCTTCTGACTCTTACCTCCCTCTCTCCTACTCCTGCTCG
CATCTGCTATAGTGGAGGCCGGAGCAGGA 40 (0.001202%)

CGATGGTTACTTGCTTCGTCTGTTCTGTGTTGGTTTACTAAAAAACGCAACAATCAGATACGGAAGACC

TCTTATGCTCAGCACCAACAGGTCCGCCAA 5 (0.000150%)

CGCAAAAGAAGAAAGATGAGGCAGAGGTCCAAGTAAACCGCTAGCTTGTTGCACCGTGGAGGCCACAG
GAGCAGAAACATGGAATGCCAGACGCTGGGGA 18 (0.000541%)

CGCAAACTCTGTCTCAACATCTGTGTTGGGGAGAGTGGAGACAGACTGACGCGAGCAGCCAAGGTGTTG
GAGCAGCTCACAGGGCAGACCCCTGTGTTTT 4 (0.000120%)

CGCAAAGGATCTCCTTCATCCCTCTCCAGAAGAGGAGAAGAGGAAACACAAGAAGAAACGCCTGGTGC
AGAGCCCCAATTCCTACTTCATGGATGTGAAA 5 (0.000150%)

CGCACGTGAAGCACCGTAAAGGCGCTGCGCGCCTGCGCGCCGTGGATTTCGCTGAGCGGCACGGCTACA
TCAAGGGCATCGTCAAGGACATCATCCACGA 5 (0.000150%)

CGCAGGCACATACTTCCTATTCTACACCCTAGTAGGCTCCCTTCCCCTACTCATCGCACTGATTTACACTC
ACAACACCCTAGGCTCACTAAACATTCTA 13 (0.000391%)

CGCAGGCTGAAGCGCAAAAGAAGAAAGATGAGGCAGAGGTCCAAGTAAACCGCTAGCTTGTTGCACCG
TGGAGGCCACAGGAGCAGAAACATGGAATGCC 54 (0.001623%)

CGCATCCGCAAACTCTGTCTCAACATCTGTGTTGGGGAGAGTGGAGACAGACTGACGCGAGCAGCCAAG
GTGTTGGAGCAGCTCACAGGGCAGACCCCTG 5 (0.000150%)

CGCATCCTTTACATAACAGACGAGGTCAACGATCCCTCCCTTACCATCAAATCAATTGGCCACCAATGGT
ACTGAACCTACGAGTACACCGACTACGGCG 32 (0.000962%)

CGCCATCATCCTAGTCCTCATCGCCCTCCCATCCCTACGCATCCTTTACATAACAGACGAGGTCAACGAT
CCCTCCCTTACCATCAAATCAATTGGCCAC 26 (0.000781%)

CGCCCTCCCATCCCTACGCATCCTTTACATAACAGACGAGGTCAACGATCCCTCCCTTACCATCAAATCA
ATTGGCCACCAATGGTACTGAACCTACGAG 11 (0.000331%)

CGCCGGGTCTGTGTTCCGCGCGCACGTGAAGCACCGTAAAGGCGCTGCGCGCCTGCGCGCCGTGGATTT
CGCTGAGCGGCACGGCTACATCAAGGGCATC 17 (0.000511%)

CGCCTGAACGCAGGCACATACTTCCTATTCTACACCCTAGTAGGCTCCCTTCCCCTACTCATCGCACTGAT
TTACACTCACAACACCCTAGGCTCACTAA 9 (0.000270%)

CGCGCACGTGAAGCACCGTAAAGGCGCTGCGCGCCTGCGCGCCGTGGATTTCGCTGAGCGGCACGGCTA
CATCAAGGGCATCGTCAAGGACATCATCCAC 4 (0.000120%)

CGCGCGCACGTGAAGCACCGTAAAGGCGCTGCGCGCCTGCGCGCCGTGGATTTCGCTGAGCGGCACGGC
TACATCAAGGGCATCGTCAAGGACATCATCC 12 (0.000361%)

CGCTCCCAGACATGGGTCCCTCGGCTTCCTGCCTCGGAAGCGCAGCAGCAGGCATCGTGGGAAGGTGAA
GAGCTTCCCTAAGGATGACCCGTCCAAGCCG 7 (0.000210%)

CGCTGAGGAAAAATAACTAAACATGGGCAAAGGAGATCCTAAGAAGCCGAGAGGCAAAATGTCATCAT
ATGCATTTTTTGTGCAAACTTGTCGGGAGGAG 9 (0.000270%)

CGCTGCGCGCCTGCGCGCCGTGGATTTCGCTGAGCGGCACGGCTACATCAAGGGCATCGTCAAGGACAT
CATCCACGACCCGGGCCGCGGCGCGCCCCTC 3 (0.000090%)

CGCTTCATTCATTGCCCCCACAATCCTAGGCCTACCCGCCGCAGTACTGATCATTCTATTTCCCCCTCTAT
TGATCCCCACCTCCAAATATCTCATCAAC 7 (0.000210%)

CGGAATAATCTCCCATATTGTAACTTACTACTCCGGAAAAAAAGAACCATTTGGATACATAGGTATGGTC
TGAGCTATGATATCAATTGGCTTCCTAGGG 3 (0.000090%)

CGGACGGGCACTGGGCGACTCTGTGCCTCGCTGAGGAAAAATAACTAAACATGGGCAAAGGAGATCCTA
AGAAGCCGAGAGGCAAAATGTCATCATATGC 51 (0.001532%)

CGGACTCCTGCCTCACTCATTTACACCAACCACCCAACTATCTATAAACCTAGCCATGGCCATCCCCTTA
TGAGCGGGCGCAGTGATTATAGGCTTTCGC 25 (0.000751%)

CGGCTGCTTCCAGCTCCTCCCTGGAGAAGAGCTACGAGCTGCCTGACGGCCAGGTCATCACCATTGGCA
ATGAGCGGTTCCGCTGCCCTGAGGCACTCTT 218 (0.006550%)

CGGGATAATCCTATTTATTACCTCAGAAGTTTTTTTCTTCGCAGGATTTTTCTGAGCCTTTTACCACTCCA
GCCTAGCCCCTACCCCCCAATTAGGAGGG 22 (0.000661%)

CGGGCAAGTCCACCACTACTGGCCATCTGATCTATAAATGCGGTGGCATCGACAAAAGAACCATTGAAA
AATTTGAGAAGGAGGCTGCTGAGATGGGAAA 38 (0.001142%)

CGGGCACTGGGCGACTCTGTGCCTCGCTGAGGAAAAATAACTAAACATGGGCAAAGGAGATCCTAAGA
AGCCGAGAGGCAAAATGTCATCATATGCATTT 44 (0.001322%)

CGGGGCCCAGGGATTGTGTTTAAAGTAGTGCTTCTACCAACATGTCCCGTGGTTCCAGCGCCGGTTTTGA
CCGCCACATTACCATTTTTTCACCCGAGGG 11 (0.000331%)

CGGGTCTGTGTTCCGCGCGCACGTGAAGCACCGTAAAGGCGCTGCGCGCCTGCGCGCCGTGGATTTCGCT
GAGCGGCACGGCTACATCAAGGGCATCGTC 19 (0.000571%)

CGGTGCCCCCGATATGGCGTTTCCCCGCATAAACAACATAAGCTTCTGACTCTTACCTCCCTCTCTCCTAC
TCCTGCTCGCATCTGCTATAGTGGAGGCC 13 (0.000391%)

CGGTGGCATCGACAAAAGAACCATTGAAAAATTTGAGAAGGAGGCTGCTGAGATGGGAAAGGGCTCCT
TCAAGTATGCCTGGGTCTTGGATAAACTGAAA 3 (0.000090%)

CGTAAAGGCGCTGCGCGCCTGCGCGCCGTGGATTTCGCTGAGCGGCACGGCTACATCAAGGGCATCGTC
AAGGACATCATCCACGACCCGGGCCGCGGCG 3 (0.000090%)

CGTCATTGGACACGTAGATTCGGGCAAGTCCACCACTACTGGCCATCTGATCTATAAATGCGGTGGCATC
GACAAAAGAACCATTGAAAAATTTGAGAAG 15 (0.000451%)

CGTCTGAACTATCCTGCCCGCCATCATCCTAGTCCTCATCGCCCTCCCATCCCTACGCATCCTTTACATAA
CAGACGAGGTCAACGATCCCTCCCTTACC 3 (0.000090%)

CGTGAAAACTACCCCTAAAAGCCAAAATGGGAAAGGAAAAGACTCATATCAACATTGTCGTCATTGGAC
ACGTAGATTCGGGCAAGTCCACCACTACTGG 13 (0.000391%)

CGTGAACGTGGTATCACCATTGATATCTCCTTGTGGAAATTTGAGACCAGCAAGTACTATGTGACTATCA
TTGATGCCCCAGGACACAGAGACTTTATCA 27 (0.000811%)

CGTGAAGCACCGTAAAGGCGCTGCGCGCCTGCGCGCCGTGGATTTCGCTGAGCGGCACGGCTACATCAA
GGGCATCGTCAAGGACATCATCCACGACCCG 3 (0.000090%)

CGTGATATGAGACAGACAGTTGCGGTGGGTGTCATCAAAGCAGTGGACAAGAAGGCTGCTGGAGCTGGC
AAGGTCACCAAGTCTGCCCAGAAAGCTCAGA 134 (0.004026%)

CGTGGACAGAGGAAGGGCGCCGGGTCTGTGTTCCGCGCGCACGTGAAGCACCGTAAAGGCGCTGCGCGC
CTGCGCGCCGTGGATTTCGCTGAGCGGCACG 39 (0.001172%)

CGTGGTATCACCATTGATATCTCCTTGTGGAAATTTGAGACCAGCAAGTACTATGTGACTATCATTGATG
CCCCAGGACACAGAGACTTTATCAAAAACA 34 (0.001022%)

CGTTCTGGTAAAAAGCTGGAAGATGGCCCTAAATTCTTGAAGTCTGGTGATGCTGCCATTGTTGATATGG
TTCCTGGCAAGCCCATGTGTGTTGAGAGCT 12 (0.000361%)

CGTTGATGTCAAGACTACCGATGGTTACTTGCTTCGTCTGTTCTGTGTTGGTTTTACTAAAAAACGCAACA
ATCAGATACGGAAGACCTCTTATGCTCAG 292 (0.008774%)

CGTTGTAGGCCCCTACGGGCTACTACAACCCTTCGCTGACGCCATAAAACTCTTCACCAAAGAGCCCCTA
AAACCCGCCACATCTACCATCACCCTCTAC 3 (0.000090%)

CGTTTCCCCGCATAAACAACATAAGCTTCTGACTCTTACCTCCCTCTCTCCTACTCCTGCTCGCATCTGCT
ATAGTGGAGGCCGGAGCAGGAACAGGTTG 5 (0.000150%)

CTAAATTCTTGAAGTCTGGTGATGCTGCCATTGTTGATATGGTTCCTGGCAAGCCCATGTGTGTTGAGAG
CTTCTCAGACTATCCACCTTTGGGTCGCTT 76 (0.002284%)

CTAACAGGGGCCCTCTCAGCCCTCCTAATGACCTCCGGCCTAGCCATGTGATTTCACTTCCACTCCATAA
CGCTCCTCATACTAGGCCTACTAACCAACA 22 (0.000661%)

CTAAGCCTCCTTATTCGAGCCGAGCTGGGCCAGCCAGGCAACCTTCTAGGTAACGACCACATCTACAAC
GTTATCGTCACAGCCCATGCATTTGTAATAA 4 (0.000120%)

CTAATAATCGGTGCCCCCGATATGGCGTTTCCCCGCATAAACAACATAAGCTTCTGACTCTTACCTCCCT
CTCTCCTACTCCTGCTCGCATCTGCTATAG 3 (0.000090%)

CTAATGACCTCCGGCCTAGCCATGTGATTTCACTTCCACTCCATAACGCTCCTCATACTAGGCCTACTAAC
CAACACACTAACCATATACCAATGATGGC 3 (0.000090%)

CTACAATGATTTTGGGAATTACAACAATCAGTCTTCAAATTTTGGACCCATGAAGGGAGGAAATTTTGGA
GGCAGAAGCTCTGGCCCCTATGGCGGTGGA 17 (0.000511%)

CTACACCCTAGTAGGCTCCCTTCCCCTACTCATCGCACTGATTTACACTCACAACACCCTAGGCTCACTA
AACATTCTACTACTCACTCTCACTGCCCAA 4 (0.000120%)

CTACATACTTCCCCCATTATTCCTAGAACCAGGCGACCTGCGACTCCTTGACGTTGACAATCGAGTAGTA
CTCCCGATTGAAGCCCCCATTCGTATAATA 4 (0.000120%)

CTACGCATCCTTTACATAACAGACGAGGTCAACGATCCCTCCCTTACCATCAAATCAATTGGCCACCAAT
GGTACTGAACCTACGAGTACACCGACTACG 5 (0.000150%)

CTAGAACCAGGCGACCTGCGACTCCTTGACGTTGACAATCGAGTAGTACTCCCGATTGAAGCCCCCATTC
GTATAATAATTACATCACAAGACGTCTTGC 25 (0.000751%)

CTAGGAATCACCTCCCATTCCGATAAAATCACCTTCCACCCTTACTACACAATCAAAGACGCCCTCGGCT
TACTTCTCTTCATTCTCTCCTTAATGACAT 227 (0.006821%)

CTAGTAGGCTCCCTTCCCCTACTCATCGCACTGATTTACACTCACAACACCCTAGGCTCACTAAACATTCT
ACTACTCACTCTCACTGCCCAAGAACTAT 6 (0.000180%)

CTAGTCCTCATCGCCCTCCCATCCCTACGCATCCTTTACATAACAGACGAGGTCAACGATCCCTCCCTTAC
CATCAAATCAATTGGCCACCAATGGTACT 27 (0.000811%)

CTAGTTCCCCTAATAATCGGTGCCCCCGATATGGCGTTTCCCCGCATAAACAACATAAGCTTCTGACTCT
TACCTCCCTCTCTCCTACTCCTGCTCGCAT 3 (0.000090%)

CTATAAACCTAGCCATGGCCATCCCCTTATGAGCGGGCGCAGTGATTATAGGCTTTCGCTCTAAGATTAA
AAATGCCCTAGCCCACTTCTTACCACAAGG 14 (0.000421%)

CTATAAATGCGGTGGCATCGACAAAAGAACCATTGAAAAATTTGAGAAGGAGGCTGCTGAGATGGGAA
AGGGCTCCTTCAAGTATGCCTGGGTCTTGGAT 8 (0.000240%)

CTATCACCCTATAGAAGAACTAATGTTAGTATAAGTAACATGAAAACATTCTCCTCCGCATAAGCCTGCG

TCAGATTAAAACACTGAACTGACAATTAAC 14 (0.000421%)

CTATCATTGATGCCCCAGGACACAGAGACTTTATCAAAAACATGATTACAGGGACATCTCAGGCTGACT
GTGCTGTCCTGATTGTTGCTGCTGGTGTTGG 26 (0.000781%)

CTATCCTGCCCGCCATCATCCTAGTCCTCATCGCCCTCCCATCCCTACGCATCCTTTACATAACAGACGAG
GTCAACGATCCCTCCCTTACCATCAAATC 15 (0.000451%)

CTATCTATAAACCTAGCCATGGCCATCCCCTTATGAGCGGGCGCAGTGATTATAGGCTTTCGCTCTAAGA
TTAAAAATGCCCTAGCCCACTTCTTACCAC 10 (0.000300%)

CTATGTGACTATCATTGATGCCCCAGGACACAGAGACTTTATCAAAAACATGATTACAGGGACATCTCA
GGCTGACTGTGCTGTCCTGATTGTTGCTGCT 3 (0.000090%)

CTATTCTACACCCTAGTAGGCTCCCTTCCCCTACTCATCGCACTGATTTACACTCACAACACCCTAGGCTC
ACTAAACATTCTACTACTCACTCTCACTG 12 (0.000361%)

CTATTTATTACCTCAGAAGTTTTTTTCTTCGCAGGATTTTTCTGAGCCTTTTACCACTCCAGCCTAGCCCCT
ACCCCCCAATTAGGAGGGCACTGGCCCC 12 (0.000361%)

CTCAAACCCGGTATGGTGGTCACCTTTGCTCCAGTCAACGTTACAACGGAAGTAAAATCTGTCGAAATGC
ACCATGAAGCTTTGAGTGAAGCTCTTCCTG 92 (0.002764%)

CTCACACCCAATTGGACCAATCTATCACCCTATAGAAGAACTAATGTTAGTATAAGTAACATGAAAACA
TTCTCCTCCGCATAAGCCTGCGTCAGATTAA 33 (0.000992%)

CTCACCAAAGCCCATAAAAATAAAAAATTATAACAAACCCTGAGAACCAAAATGAACGAAAATCTGTTC
GCTTCATTCATTGCCCCCACAATCCTAGGCC 23 (0.000691%)

CTCACTCATTTACACCAACCACCCAACTATCTATAAACCTAGCCATGGCCATCCCCTTATGAGCGGGCGC
AGTGATTATAGGCTTTCGCTCTAAGATTAA 12 (0.000361%)

CTCAGAAGTTTTTTTCTTCGCAGGATTTTTCTGAGCCTTTTACCACTCCAGCCTAGCCCCTACCCCCCAAT
TAGGAGGGCACTGGCCCCCAACAGGCATC 30 (0.000901%)

CTCAGACTATCCACCTTTGGGTCGCTTTGCTGTTCGTGATATGAGACAGACAGTTGCGGTGGGTGTCATC
AAAGCAGTGGACAAGAAGGCTGCTGGAGCT 83 (0.002494%)

CTCAGCCCTCCTAATGACCTCCGGCCTAGCCATGTGATTTCACTTCCACTCCATAACGCTCCTCATACTAG
GCCTACTAACCAACACACTAACCATATAC 18 (0.000541%)

CTCAGGCTGACTGTGCTGTCCTGATTGTTGCTGCTGGTGTTGGTGAATTTGAAGCTGGTATCTCCAAGAA
TGGGCAGACCCGAGAGCATGCCCTTCTGGC 67 (0.002013%)

CTCATCGCCCTCCCATCCCTACGCATCCTTTACATAACAGACGAGGTCAACGATCCCTCCCTTACCATCA
AATCAATTGGCCACCAATGGTACTGAACCT 15 (0.000451%)

CTCATTTACACCAACCACCCAACTATCTATAAACCTAGCCATGGCCATCCCCTTATGAGCGGGCGCAGTG
ATTATAGGCTTTCGCTCTAAGATTAAAAAT 31 (0.000931%)

CTCCCAGTCCTAGCTGCTGGCATCACTATACTACTAACAGACCGCAACCTCAACACCACCTTCTTCGACC
CCGCCGGAGGAGGAGACCCCATTCTATACC 5 (0.000150%)

CTCCCATATTGTAACTTACTACTCCGGAAAAAAAGAACCATTTGGATACATAGGTATGGTCTGAGCTATG
ATATCAATTGGCTTCCTAGGGTTTATCGTG 58 (0.001743%)

CTCCCATCCCTACGCATCCTTTACATAACAGACGAGGTCAACGATCCCTCCCTTACCATCAAATCAATTG
GCCACCAATGGTACTGAACCTACGAGTACA 4 (0.000120%)

CTCCGCTCCCAGACATGGGTCCCTCGGCTTCCTGCCTCGGAAGCGCAGCAGCAGGCATCGTGGGAAGGT
GAAGAGCTTCCCTAAGGATGACCCGTCCAAG 14 (0.000421%)

CTCCTAATGACCTCCGGCCTAGCCATGTGATTTCACTTCCACTCCATAACGCTCCTCATACTAGGCCTACT
AACCAACACACTAACCATATACCAATGAT 13 (0.000391%)

CTCCTATCTCTCCCAGTCCTAGCTGCTGGCATCACTATACTACTAACAGACCGCAACCTCAACACCACCT
TCTTCGACCCCGCCGGAGGAGGAGACCCCA 25 (0.000751%)

CTCCTCACACCCAATTGGACCAATCTATCACCCTATAGAAGAACTAATGTTAGTATAAGTAACATGAAAA
CATTCTCCTCCGCATAAGCCTGCGTCAGAT 7 (0.000210%)

CTCCTGCCTCACTCATTTACACCAACCACCCAACTATCTATAAACCTAGCCATGGCCATCCCCTTATGAG
CGGGCGCAGTGATTATAGGCTTTCGCTCTA 12 (0.000361%)

CTCCTGGGCCGCCTGGCGGCCATCGTGGCTAAACAGGTACTGCTGGGCCGGAAGGTGGTGGTCGTACGC
TGTGAAGGCATCAACATTTCTGGCAATTTCT 6 (0.000180%)

CTCCTTCAAGTATGCCTGGGTCTTGGATAAACTGAAAGCTGAGCGTGAACGTGGTATCACCATTGATATC
TCCTTGTGGAAATTTGAGACCAGCAAGTAC 38 (0.001142%)

CTCCTTCATCCCTCTCCAGAAGAGGAGAAGAGGAAACACAAGAAGAAACGCCTGGTGCAGAGCCCCAAT
TCCTACTTCATGGATGTGAAATGCCCAGGAT 10 (0.000300%)

CTCCTTGTGGAAATTTGAGACCAGCAAGTACTATGTGACTATCATTGATGCCCCAGGACACAGAGACTTT
ATCAAAAACATGATTACAGGGACATCTCAG 7 (0.000210%)

CTCGCAAAGGATCTCCTTCATCCCTCTCCAGAAGAGGAGAAGAGGAAACACAAGAAGAAACGCCTGGTG
CAGAGCCCCAATTCCTACTTCATGGATGTGA 12 (0.000361%)

CTCGGACTCCTGCCTCACTCATTTACACCAACCACCCAACTATCTATAAACCTAGCCATGGCCATCCCCTT
ATGAGCGGGCGCAGTGATTATAGGCTTTC 49 (0.001472%)

CTCGGCTTCCTGCCTCGGAAGCGCAGCAGCAGGCATCGTGGGAAGGTGAAGAGCTTCCCTAAGGATGAC
CCGTCCAAGCCGGTCCACCTCACAGCCTTCC 11 (0.000331%)

CTCTAAGCCTCCTTATTCGAGCCGAGCTGGGCCAGCCAGGCAACCTTCTAGGTAACGACCACATCTACAA
CGTTATCGTCACAGCCCATGCATTTGTAAT 14 (0.000421%)

CTCTAGGCCTTTTAGAAAACATGGAGTTGTTCCTTTGGCCACATATATGCGAATCTATAAGAAAGGTGAT
ATTGTAGACATCAAGGGAATGGGTACTGTT 14 (0.000421%)

CTCTCAGCCCTCCTAATGACCTCCGGCCTAGCCATGTGATTTCACTTCCACTCCATAACGCTCCTCATACT
AGGCCTACTAACCAACACACTAACCATAT 5 (0.000150%)

CTCTCCCAGTCCTAGCTGCTGGCATCACTATACTACTAACAGACCGCAACCTCAACACCACCTTCTTCGA
CCCCGCCGGAGGAGGAGACCCCATTCTATA 16 (0.000481%)

CTCTCGCAAAGGATCTCCTTCATCCCTCTCCAGAAGAGGAGAAGAGGAAACACAAGAAGAAACGCCTGG
TGCAGAGCCCCAATTCCTACTTCATGGATGT 13 (0.000391%)

CTCTGTGCCTCGCTGAGGAAAAATAACTAAACATGGGCAAAGGAGATCCTAAGAAGCCGAGAGGCAAA
ATGTCATCATATGCATTTTTTGTGCAAACTTG 10 (0.000300%)

CTGAAAGCTGAGCGTGAACGTGGTATCACCATTGATATCTCCTTGTGGAAATTTGAGACCAGCAAGTACT
ATGTGACTATCATTGATGCCCCAGGACACA 95 (0.002854%)

CTGAACGCAGGCACATACTTCCTATTCTACACCCTAGTAGGCTCCCTTCCCCTACTCATCGCACTGATTTA
CACTCACAACACCCTAGGCTCACTAAACA 15 (0.000451%)

CTGAACTATCCTGCCCGCCATCATCCTAGTCCTCATCGCCCTCCCATCCCTACGCATCCTTTACATAACAG
ACGAGGTCAACGATCCCTCCCTTACCATC 59 (0.001773%)

CTGAACTCCTCACACCCAATTGGACCAATCTATCACCCTATAGAAGAACTAATGTTAGTATAAGTAACAT
GAAAACATTCTCCTCCGCATAAGCCTGCGT 43 (0.001292%)

CTGAAGCGCAAAAGAAGAAAGATGAGGCAGAGGTCCAAGTAAACCGCTAGCTTGTTGCACCGTGGAGG
CCACAGGAGCAGAAACATGGAATGCCAGACGC 3 (0.000090%)

CTGACTAGTTCCCCTAATAATCGGTGCCCCCGATATGGCGTTTCCCCGCATAAACAACATAAGCTTCTGA
CTCTTACCTCCCTCTCTCCTACTCCTGCTC 10 (0.000300%)

CTGACTGTGCTGTCCTGATTGTTGCTGCTGGTGTTGGTGAATTTGAAGCTGGTATCTCCAAGAATGGGCA
GACCCGAGAGCATGCCCTTCTGGCTTACAC 4 (0.000120%)

CTGAGCGTGAACGTGGTATCACCATTGATATCTCCTTGTGGAAATTTGAGACCAGCAAGTACTATGTGAC
TATCATTGATGCCCCAGGACACAGAGACTT 26 (0.000781%)

CTGAGCTCACCATAGTCTAATAGAAAACAACCGAAACCAAATAATTCAAGCACTGCTTATTACAATTTTA
CTGGGTCTCTATTTTACCCTCCTACAAGCC444 (0.013341%)

CTGCCATTGTTGATATGGTTCCTGGCAAGCCCATGTGTGTTGAGAGCTTCTCAGACTATCCACCTTTGGGT
CGCTTTGCTGTTCGTGATATGAGACAGAC 18 (0.000541%)

CTGCCCGCCATCATCCTAGTCCTCATCGCCCTCCCATCCCTACGCATCCTTTACATAACAGACGAGGTCA
ACGATCCCTCCCTTACCATCAAATCAATTG 20 (0.000601%)

CTGCCTCACTCATTTACACCAACCACCCAACTATCTATAAACCTAGCCATGGCCATCCCCTTATGAGCGG
GCGCAGTGATTATAGGCTTTCGCTCTAAGA 8 (0.000240%)

CTGCGACTTGTGTTGGGACTGCTGATAGGAAGATGTCTTCAGGAAATGCTAAAATTGGGCACCCTGCCCC
CAACTTCAAAGCCACAGCTGTTATGCCAGA 382 (0.011478%)

CTGCTGGAGCTGGCAAGGTCACCAAGTCTGCCCAGAAAGCTCAGAAGGCTAAATGAATATTATCCCTAA
TACCTGCCACCCCACTCTTAATCAGTGGTGG 6 (0.000180%)

CTGCTTCCATGTTGGCATCTGCCCCTCCTCAAGAGCAAAAGCAAATGTTGGGTGAACGGCTGTTTCCTCT
TATTCAAGCCATGCACCCTACTCTTGCTGG 4 (0.000120%)

CTGGAAGATGGCCCTAAATTCTTGAAGTCTGGTGATGCTGCCATTGTTGATATGGTTCCTGGCAAGCCCA
TGTGTGTTGAGAGCTTCTCAGACTATCCAC 77 (0.002314%)

CTGGAGAAGAGCTACGAGCTGCCTGACGGCCAGGTCATCACCATTGGCAATGAGCGGTTCCGCTGCCCT
GAGGCACTCTTCCAGCCTTCCTTCCTGGGCA 4 (0.000120%)

CTGGAGCTGGCAAGGTCACCAAGTCTGCCCAGAAAGCTCAGAAGGCTAAATGAATATTATCCCTAATAC
CTGCCACCCCACTCTTAATCAGTGGTGGAAG 7 (0.000210%)

CTGGCAAGCCCATGTGTGTTGAGAGCTTCTCAGACTATCCACCTTTGGGTCGCTTTGCTGTTCGTGATATG
AGACAGACAGTTGCGGTGGGTGTCATCAA28 (0.000841%)

CTGGCTTACACACTGGGTGTGAAACAACTAATTGTCGGTGTTAACAAAATGGATTCCACTGAGCCACCCT
ACAGCCAGAAGAGATATGAGGAAATTGTTA 24 (0.000721%)

CTGGGCGACTCTGTGCCTCGCTGAGGAAAAATAACTAAACATGGGCAAAGGAGATCCTAAGAAGCCGA
GAGGCAAAATGTCATCATATGCATTTTTTGTG 6 (0.000180%)

CTGGGTCTTGGATAAACTGAAAGCTGAGCGTGAACGTGGTATCACCATTGATATCTCCTTGTGGAAATTT

GAGACCAGCAAGTACTATGTGACTATCATT 27 (0.000811%)

CTGGGTGTGAAACAACTAATTGTCGGTGTTAACAAAATGGATTCCACTGAGCCACCCTACAGCCAGAAG
AGATATGAGGAAATTGTTAAGGAAGTCAGCA 9 (0.000270%)

CTGGTAAAAAGCTGGAAGATGGCCCTAAATTCTTGAAGTCTGGTGATGCTGCCATTGTTGATATGGTTCC
TGGCAAGCCCATGTGTGTTGAGAGCTTCTC 20 (0.000601%)

CTGGTGATGCTGCCATTGTTGATATGGTTCCTGGCAAGCCCATGTGTGTTGAGAGCTTCTCAGACTATCC
ACCTTTGGGTCGCTTTGCTGTTCGTGATAT 24 (0.000721%)

CTGGTGTTCTCAAACCCGGTATGGTGGTCACCTTTGCTCCAGTCAACGTTACAACGGAAGTAAAATCTGT
CGAAATGCACCATGAAGCTTTGAGTGAAGC 61 (0.001833%)

CTGTCAAGGATGTTCGTCGTGGCAACGTTGCTGGTGACAGCAAAAATGACCCACCAATGGAAGCAGCTG
GCTTCACTGCTCAGGTGATTATCCTGAACCA 7 (0.000210%)

CTGTGCCTCGCTGAGGAAAAATAACTAAACATGGGCAAAGGAGATCCTAAGAAGCCGAGAGGCAAAAT
GTCATCATATGCATTTTTTGTGCAAACTTGTC 7 (0.000210%)

CTGTGCTGTCCTGATTGTTGCTGCTGGTGTTGGTGAATTTGAAGCTGGTATCTCCAAGAATGGGCAGACC
CGAGAGCATGCCCTTCTGGCTTACACACTG 9 (0.000270%)

CTGTGTTCCGCGCGCACGTGAAGCACCGTAAAGGCGCTGCGCGCCTGCGCGCCGTGGATTTCGCTGAGC
GGCACGGCTACATCAAGGGCATCGTCAAGGA 19 (0.000571%)

CTGTGTTGGTTTTACTAAAAAACGCAACAATCAGATACGGAAGACCTCTTATGCTCAGCACCAACAGGTC
CGCCAAATCCGGAAGAAGATGATGGAAATC 4 (0.000120%)

CTGTTCGCTTCATTCATTGCCCCCACAATCCTAGGCCTACCCGCCGCAGTACTGATCATTCTATTTCCCCC
TCTATTGATCCCCACCTCCAAATATCTCA 8 (0.000240%)

CTGTTCTGTGTTGGTTTTACTAAAAAACGCAACAATCAGATACGGAAGACCTCTTATGCTCAGCACCAAC
AGGTCCGCCAAATCCGGAAGAAGATGATGG 6 (0.000180%)

CTTAATCATTTTTATTGCCACAACTAACCTCCTCGGACTCCTGCCTCACTCATTTACACCAACCACCCAAC
TATCTATAAACCTAGCCATGGCCATCCCC 6 (0.000180%)

CTTACACACTGGGTGTGAAACAACTAATTGTCGGTGTTAACAAAATGGATTCCACTGAGCCACCCTACAG
CCAGAAGAGATATGAGGAAATTGTTAAGGA 18 (0.000541%)

CTTACTACTCCGGAAAAAAAGAACCATTTGGATACATAGGTATGGTCTGAGCTATGATATCAATTGGCTT
CCTAGGGTTTATCGTGTGAGCACACCATAT 20 (0.000601%)

CTTATTCGAGCCGAGCTGGGCCAGCCAGGCAACCTTCTAGGTAACGACCACATCTACAACGTTATCGTCA
CAGCCCATGCATTTGTAATAATCTTCTTCA9 (0.000270%)

CTTCAAGTATGCCTGGGTCTTGGATAAACTGAAAGCTGAGCGTGAACGTGGTATCACCATTGATATCTCC
TTGTGGAAATTTGAGACCAGCAAGTACTAT 70 (0.002103%)

CTTCAATGTCAAGAATGTGTCTGTCAAGGATGTTCGTCGTGGCAACGTTGCTGGTGACAGCAAAAATGAC
CCACCAATGGAAGCAGCTGGCTTCACTGCT 132 (0.003966%)

CTTCACCCGTAACCCACCGCCATGGCCGAGGAAGGCATTGCTGCTGGAGGTGTAATGGACGTTAATACT
GCTTTACAAGAGGTTCTGAAGACTGCCCTCA 22 (0.000661%)

CTTCATCCCTCTCCAGAAGAGGAGAAGAGGAAACACAAGAAGAAACGCCTGGTGCAGAGCCCCAATTCC
TACTTCATGGATGTGAAATGCCCAGGATGCT 17 (0.000511%)

CTTCATTCATTGCCCCCACAATCCTAGGCCTACCCGCCGCAGTACTGATCATTCTATTTCCCCCTCTATTG
ATCCCCACCTCCAAATATCTCATCAACAA 9 (0.000270%)

CTTCCACCCTTACTACACAATCAAAGACGCCCTCGGCTTACTTCTCTTCATTCTCTCCTTAATGACATTAA
CACTATTCTCACCAGACCTCCTAGGCGAC 8 (0.000240%)

CTTCCCCCATTATTCCTAGAACCAGGCGACCTGCGACTCCTTGACGTTGACAATCGAGTAGTACTCCCGA
TTGAAGCCCCCATTCGTATAATAATTACAT 14 (0.000421%)

CTTCGATACGGGATAATCCTATTTATTACCTCAGAAGTTTTTTCTTCGCAGGATTTTTCTGAGCCTTTTAC
CACTCCAGCCTAGCCCCTACCCCCCAAT 58 (0.001743%)

CTTCGCATCCGCAAACTCTGTCTCAACATCTGTGTTGGGGAGAGTGGAGACAGACTGACGCGAGCAGCC
AAGGTGTTGGAGCAGCTCACAGGGCAGACCC 11 (0.000331%)

CTTCGGAATAATCTCCCATATTGTAACTTACTACTCCGGAAAAAAAGAACCATTTGGATACATAGGTATG
GTCTGAGCTATGATATCAATTGGCTTCCTA 17 (0.000511%)

CTTCTCAGACTATCCACCTTTGGGTCGCTTTGCTGTTCGTGATATGAGACAGACAGTTGCGGTGGGTGTC
ATCAAAGCAGTGGACAAGAAGGCTGCTGGA 7 (0.000210%)

CTTCTCCTATCTCTCCCAGTCCTAGCTGCTGGCATCACTATACTACTAACAGACCGCAACCTCAACACCA
CCTTCTTCGACCCCGCCGGAGGAGGAGACC 10 (0.000300%)

CTTCTGGCTTACACACTGGGTGTGAAACAACTAATTGTCGGTGTTAACAAAATGGATTCCACTGAGCCAC
CCTACAGCCAGAAGAGATATGAGGAAATTG 23 (0.000691%)

CTTGAAGTCTGGTGATGCTGCCATTGTTGATATGGTTCCTGGCAAGCCCATGTGTGTTGAGAGCTTCTCA
GACTATCCACCTTTGGGTCGCTTTGCTGTT 178 (0.005348%)

CTTGATGGTCGAGGCCATCTCCTGGGCCGCCTGGCGGCCATCGTGGCTAAACAGGTACTGCTGGGCCGG
AAGGTGGTGGTCGTACGCTGTGAAGGCATCA 23 (0.000691%)

CTTGCTTCGTCTGTTCTGTGTTGGTTTACTAAAAAACGCAACAATCAGATACGGAAGACCTCTTATGCTC
AGCACCAACAGGTCCGCCAAATCCGGAAG 15 (0.000451%)

CTTGGATAAACTGAAAGCTGAGCGTGAACGTGGTATCACCATTGATATCTCCTTGTGGAAATTTGAGACC
AGCAAGTACTATGTGACTATCATTGATGCC 94 (0.002824%)

CTTGTGGAAATTTGAGACCAGCAAGTACTATGTGACTATCATTGATGCCCCAGGACACAGAGACTTTATC
AAAAACATGATTACAGGGACATCTCAGGCT 14 (0.000421%)

CTTTACATAACAGACGAGGTCAACGATCCCTCCCTTACCATCAAATCAATTGGCCACCAATGGTACTGAA
CCTACGAGTACACCGACTACGGCGGACTAA 15 (0.000451%)

CTTTATCAAAAACATGATTACAGGGACATCTCAGGCTGACTGTGCTGTCCTGATTGTTGCTGCTGGTGTT
GGTGAATTTGAAGCTGGTATCTCCAAGAAT 233 (0.007001%)

CTTTGACTGCTTCCATGTTGGCATCTGCCCCTCCTCAAGAGCAAAAGCAAATGTTGGGTGAACGGCTGTT
TCCTCTTATTCAAGCCATGCACCCTACTCT 12 (0.000361%)

CTTTGCTCCAGTCAACGTTACAACGGAAGTAAAATCTGTCGAAATGCACCATGAAGCTTTGAGTGAAGCT
CTTCCTGGGGACAATGTGGGCTTCAATGTC 106 (0.003185%)

CTTTGGGTCGCTTTGCTGTTCGTGATATGAGACAGACAGTTGCGGTGGGTGTCATCAAAGCAGTGGACAA
GAAGGCTGCTGGAGCTGGCAAGGTCACCAA 49 (0.001472%)

GAAAATCTGTTCGCTTCATTCATTGCCCCCACAATCCTAGGCCTACCCGCCGCAGTACTGATCATTCTATT
TCCCCCTCTATTGATCCCCACCTCCAAAT 35 (0.001052%)

GAAACAACTAATTGTCGGTGTTAACAAAATGGATTCCACTGAGCCACCCTACAGCCAGAAGAGATATGA
GGAAATTGTTAAGGAAGTCAGCACTTACATT 27 (0.000811%)

GAAACCGTCTGAACTATCCTGCCCGCCATCATCCTAGTCCTCATCGCCCTCCCATCCCTACGCATCCTTTA
CATAACAGACGAGGTCAACGATCCCTCCC 12 (0.000361%)

GAAAGCTGAGCGTGAACGTGGTATCACCATTGATATCTCCTTGTGGAAATTTGAGACCAGCAAGTACTAT
GTGACTATCATTGATGCCCCAGGACACAGA 38 (0.001142%)

GAAAGGGCTCCTTCAAGTATGCCTGGGTCTTGGATAAACTGAAAGCTGAGCGTGAACGTGGTATCACCA
TTGATATCTCCTTGTGGAAATTTGAGACCAG 643 (0.019320%)

GAAAGTTCTCCGCTCCCAGACATGGGTCCCTCGGCTTCCTGCCTCGGAAGCGCAGCAGCAGGCATCGTG
GGAAGGTGAAGAGCTTCCCTAAGGATGACCC 33 (0.000992%)

GAACACAGGTGTCGTGAAAACTACCCCTAAAAGCCAAAATGGGAAAGGAAAAGACTCATATCAACATT
GTCGTCATTGGACACGTAGATTCGGGCAAGTC 35 (0.001052%)

GAACCAGGCGACCTGCGACTCCTTGACGTTGACAATCGAGTAGTACTCCCGATTGAAGCCCCCATTCGTA
TAATAATTACATCACAAGACGTCTTGCACT 6 (0.000180%)

GAACCTGATCTCTTATACTAGTATCCTTAATCATTTTTATTGCCACAACTAACCTCCTCGGACTCCTGCCT
CACTCATTTACACCAACCACCCAACTATC 4 (0.000120%)

GAACCTTTGACTGCTTCCATGTTGGCATCTGCCCCTCCTCAAGAGCAAAAGCAAATGTTGGGTGAACGGC
TGTTTCCTCTTATTCAAGCCATGCACCCTA 104 (0.003125%)

GAACGCCTGAACGCAGGCACATACTTCCTATTCTACACCCTAGTAGGCTCCCTTCCCCTACTCATCGCAC
TGATTTACACTCACAACACCCTAGGCTCAC 13 (0.000391%)

GAACGTGGTATCACCATTGATATCTCCTTGTGGAAATTTGAGACCAGCAAGTACTATGTGACTATCATTG
ATGCCCCAGGACACAGAGACTTTATCAAAA 14 (0.000421%)

GAACTATCCTGCCCGCCATCATCCTAGTCCTCATCGCCCTCCCATCCCTACGCATCCTTTACATAACAGAC
GAGGTCAACGATCCCTCCCTTACCATCAA 26 (0.000781%)

GAACTCCTCACACCCAATTGGACCAATCTATCACCCTATAGAAGAACTAATGTTAGTATAAGTAACATGA
AAACATTCTCCTCCGCATAAGCCTGCGTCA 12 (0.000361%)

GAAGATCCTGGTGTCGCCATGGGCCGCCGCCCCGCCCGTTGTTACCGGTATTGTAAGAACAAGCCGTACC
CAAAGTCTCGCTTCTGCCGAGGTGTCCCTGATGCCAAGATTCGCATTTTTGACCTGGGGCGGAAAAAGGC
AAAAGTGGA 222 (0.009939%)

GAAGATGGCCCTAAATTCTTGAAGTCTGGTGATGCTGCCATTGTTGATATGGTTCCTGGCAAGCCCATGT
GTGTTGAGAGCTTCTCAGACTATCCACCTT 13 (0.000391%)

GAAGCAATTTTGGAGGTGGTGGAAGCTACAATGATTTTGGGAATTACAACAATCAGTCTTCAAATTTTGG
ACCCATGAAGGGAGGAAATTTTGGAGGCAG 5 (0.000150%)

GAAGCGCAAAAGAAGAAAGATGAGGCAGAGGTCCAAGTAAACCGCTAGCTTGTTGCACCGTGGAGGCC
ACAGGAGCAGAAACATGGAATGCCAGACGCTG 4 (0.000120%)

GAAGCTACAATGATTTTGGGAATTACAACAATCAGTCTTCAAATTTTGGACCCATGAAGGGAGGAAATT
TTGGAGGCAGAAGCTCTGGCCCCTATGGCGG 7 (0.000210%)

GAAGGAGGCTGCTGAGATGGGAAAGGGCTCCTTCAAGTATGCCTGGGTCTTGGATAAACTGAAAGCTGA
GCGTGAACGTGGTATCACCATTGATATCTCC 34 (0.001022%)

GAAGGCTGCTGGAGCTGGCAAGGTCACCAAGTCTGCCCAGAAAGCTCAGAAGGCTAAATGAATATTATC
CCTAATACCTGCCACCCCACTCTTAATCAGT 28 (0.000841%)

GAAGGGCGCCGGGTCTGTGTTCCGCGCGCACGTGAAGCACCGTAAAGGCGCTGCGCGCCTGCGCGCCGT
GGATTTCGCTGAGCGGCACGGCTACATCAAG 9 (0.000270%)

GAAGTCTGGTGATGCTGCCATTGTTGATATGGTTCCTGGCAAGCCCATGTGTGTTGAGAGCTTCTCAGAC
TATCCACCTTTGGGTCGCTTTGCTGTTCGT 6 (0.000180%)

GAAGTTTTTTCTTCGCAGGATTTTTCTGAGCCTTTTACCACTCCAGCCTAGCCCCTACCCCCCAATTAGG
AGGGCACTGGCCCCCAACAGGCATCACCC 3 (0.000090%)

GAATAATCTCCCATATTGTAACTTACTACTCCGGAAAAAAAGAACCATTTGGATACATAGGTATGGTCTG
AGCTATGATATCAATTGGCTTCCTAGGGTT 46 (0.001382%)

GAATCACCTCCCATTCCGATAAAATCACCTTCCACCCTTACTACACAATCAAAGACGCCCTCGGCTTACT
TCTCTTCATTCTCTCCTTAATGACATTAAC 9 (0.000270%)

GAATGCGCAGGCTGAAGCGCAAAAGAAGAAAGATGAGGCAGAGGTCCAAGTAAACCGCTAGCTTGTTG
CACCGTGGAGGCCACAGGAGCAGAAACATGGA 18 (0.000541%)

GAATGTGTCTGTCAAGGATGTTCGTCGTGGCAACGTTGCTGGTGACAGCAAAAATGACCCACCAATGGA
AGCAGCTGGCTTCACTGCTCAGGTGATTATC 12 (0.000361%)

GACAAGAAGGCTGCTGGAGCTGGCAAGGTCACCAAGTCTGCCCAGAAAGCTCAGAAGGCTAAATGAAT
ATTATCCCTAATACCTGCCACCCCACTCTTAA 15 (0.000451%)

GACACAGAGACTTTATCAAAAACATGATTACAGGGACATCTCAGGCTGACTGTGCTGTCCTGATTGTTGC
TGCTGGTGTTGGTGAATTTGAAGCTGGTAT 5 (0.000150%)

GACAGACAGTTGCGGTGGGTGTCATCAAAGCAGTGGACAAGAAGGCTGCTGGAGCTGGCAAGGTCACC
AAGTCTGCCCAGAAAGCTCAGAAGGCTAAATG 42 (0.001262%)

GACAGAGGAAGGGCGCCGGGTCTGTGTTCCGCGCGCACGTGAAGCACCGTAAAGGCGCTGCGCGCCTGC
GCGCCGTGGATTTCGCTGAGCGGCACGGCTA 11 (0.000331%)

GACAGTTGCGGTGGGTGTCATCAAAGCAGTGGACAAGAAGGCTGCTGGAGCTGGCAAGGTCACCAAGTC
TGCCCAGAAAGCTCAGAAGGCTAAATGAATA 14 (0.000421%)

GACATCTCAGGCTGACTGTGCTGTCCTGATTGTTGCTGCTGGTGTTGGTGAATTTGAAGCTGGTATCTCCA
AGAATGGGCAGACCCGAGAGCATGCCCTT 5 (0.000150%)

GACATGGGTCCCTCGGCTTCCTGCCTCGGAAGCGCAGCAGCAGGCATCGTGGGAAGGTGAAGAGCTTCC
CTAAGGATGACCCGTCCAAGCCGGTCCACCT 5 (0.000150%)

GACCAATCTATCACCCTATAGAAGAACTAATGTTAGTATAAGTAACATGAAAACATTCTCCTCCGCATAA
GCCTGCGTCAGATTAAAACACTGAACTGAC 5 (0.000150%)

GACCAGCAAGTACTATGTGACTATCATTGATGCCCCAGGACACAGAGACTTTATCAAAAACATGATTAC
AGGGACATCTCAGGCTGACTGTGCTGTCCTG 3 (0.000090%)

GACCCCTAACAGGGGCCCTCTCAGCCCTCCTAATGACCTCCGGCCTAGCCATGTGATTTCACTTCCACTC
CATAACGCTCCTCATACTAGGCCTACTAAC 10 (0.000300%)

GACCCGAGAGCATGCCCTTCTGGCTTACACACTGGGTGTGAAACAACTAATTGTCGGTGTTAACAAAAT
GGATTCCACTGAGCCACCCTACAGCCAGAAG 3 (0.000090%)

GACGGGCACTGGGCGACTCTGTGCCTCGCTGAGGAAAAATAACTAAACATGGGCAAAGGAGATCCTAA
GAAGCCGAGAGGCAAAATGTCATCATATGCAT 6 (0.000180%)

GACTAATCACCACCCAACAATGACTAATCAAACTAACCTCAAAACAAATGATAACCATACACAACACTA
AAGGACGAACCTGATCTCTTATACTAGTATC 8 (0.000240%)

GACTAGTTCCCCTAATAATCGGTGCCCCCGATATGGCGTTTCCCCGCATAAACAACATAAGCTTCTGACT
CTTACCTCCCTCTCTCCTACTCCTGCTCGC 8 (0.000240%)

GACTATCATTGATGCCCCAGGACACAGAGACTTTATCAAAAACATGATTACAGGGACATCTCAGGCTGA
CTGTGCTGTCCTGATTGTTGCTGCTGGTGTT 8 (0.000240%)

GACTATCCACCTTTGGGTCGCTTTGCTGTTCGTGATATGAGACAGACAGTTGCGGTGGGTGTCATCAAAG
CAGTGGACAAGAAGGCTGCTGGAGCTGGCA 3 (0.000090%)

GACTGGTGTTCTCAAACCCGGTATGGTGGTCACCTTTGCTCCAGTCAACGTTACAACGGAAGTAAAATCT
GTCGAAATGCACCATGAAGCTTTGAGTGAA 11 (0.000331%)

GACTGTGCTGTCCTGATTGTTGCTGCTGGTGTTGGTGAATTTGAAGCTGGTATCTCCAAGAATGGGCAGA
CCCGAGAGCATGCCCTTCTGGCTTACACAC 3 (0.000090%)

GAGAAGGAGGCTGCTGAGATGGGAAAGGGCTCCTTCAAGTATGCCTGGGTCTTGGATAAACTGAAAGCT
GAGCGTGAACGTGGTATCACCATTGATATCT 12 (0.000361%)

GAGACAGACAGTTGCGGTGGGTGTCATCAAAGCAGTGGACAAGAAGGCTGCTGGAGCTGGCAAGGTCA
CCAAGTCTGCCCAGAAAGCTCAGAAGGCTAAA 20 (0.000601%)

GAGACCAGCAAGTACTATGTGACTATCATTGATGCCCCAGGACACAGAGACTTTATCAAAAACATGATT
ACAGGGACATCTCAGGCTGACTGTGCTGTCC 5 (0.000150%)

GAGACCCCATTCTATACCAACACCTATTCTGATTTTTCGGTCACCCTGAAGTTTATATTCTTATCCTACCA
GGCTTCGGAATAATCTCCCATATTGTAAC 8 (0.000240%)

GAGACTGGTGTTCTCAAACCCGGTATGGTGGTCACCTTTGCTCCAGTCAACGTTACAACGGAAGTAAAAT
CTGTCGAAATGCACCATGAAGCTTTGAGTG 6 (0.000180%)

GAGAGCATGCCCTTCTGGCTTACACACTGGGTGTGAAACAACTAATTGTCGGTGTTAACAAAATGGATTC
CACTGAGCCACCCTACAGCCAGAAGAGATA 13 (0.000391%)

GAGAGCCGGACGGGCACTGGGCGACTCTGTGCCTCGCTGAGGAAAAATAACTAAACATGGGCAAAGGA
GATCCTAAGAAGCCGAGAGGCAAAATGTCATC 223 (0.006700%)

GAGAGCTTCTCAGACTATCCACCTTTGGGTCGCTTTGCTGTTCGTGATATGAGACAGACAGTTGCGGTGG
GTGTCATCAAAGCAGTGGACAAGAAGGCTG 10 (0.000300%)

GAGAGGCACCCGATATATGTTCTCTAGGCCTTTTAGAAAACATGGAGTTGTTCCTTTGGCCACATATATG
CGAATCTATAAGAAAGGTGATATTGTAGAC 8 (0.000240%)

GAGCATGCCCTTCTGGCTTACACACTGGGTGTGAAACAACTAATTGTCGGTGTTAACAAAATGGATTCCA
CTGAGCCACCCTACAGCCAGAAGAGATATG 7 (0.000210%)

GAGCCGGACGGGCACTGGGCGACTCTGTGCCTCGCTGAGGAAAAATAACTAAACATGGGCAAAGGAGA
TCCTAAGAAGCCGAGAGGCAAAATGTCATCAT 4 (0.000120%)

GAGCGTGAACGTGGTATCACCATTGATATCTCCTTGTGGAAATTTGAGACCAGCAAGTACTATGTGACTA
TCATTGATGCCCCAGGACACAGAGACTTTA 5 (0.000150%)

GAGCTACGAGCTGCCTGACGGCCAGGTCATCACCATTGGCAATGAGCGGTTCCGCTGCCCTGAGGCACT
CTTCCAGCCTTCCTTCCTGGGCATGGAGTCC 3 (0.000090%)

GAGCTGGCAAGGTCACCAAGTCTGCCCAGAAAGCTCAGAAGGCTAAATGAATATTATCCCTAATACCTG

CCACCCCACTCTTAATCAGTGGTGGAAGAAC 16 (0.000481%)

GAGCTTCTCAGACTATCCACCTTTGGGTCGCTTTGCTGTTCGTGATATGAGACAGACAGTTGCGGTGGGT
GTCATCAAAGCAGTGGACAAGAAGGCTGCT 9 (0.000270%)

GAGGAAGGGCGCCGGGTCTGTGTTCCGCGCGCACGTGAAGCACCGTAAAGGCGCTGCGCGCCTGCGCGC
CGTGGATTTCGCTGAGCGGCACGGCTACATC 3 (0.000090%)

GAGGAGACCCCATTCTATACCAACACCTATTCTGATTTTTCGGTCACCCTGAAGTTTATATTCTTATCCTA
CCAGGCTTCGGAATAATCTCCCATATTGT 19 (0.000571%)

GAGGCACCCGATATATGTTCTCTAGGCCTTTTAGAAAACATGGAGTTGTTCCTTTGGCCACATATATGCG
AATCTATAAGAAAGGTGATATTGTAGACAT 8 (0.000240%)

GAGGCCATCTCCTGGGCCGCCTGGCGGCCATCGTGGCTAAACAGGTACTGCTGGGCCGGAAGGTGGTGG
TCGTACGCTGTGAAGGCATCAACATTTCTGG 6 (0.000180%)

GAGGTGGTGGAAGCTACAATGATTTTGGGAATTACAACAATCAGTCTTCAAATTTTGGACCCATGAAGG
GAGGAAATTTTGGAGGCAGAAGCTCTGGCCC 3 (0.000090%)

GAGTGGAGACTGGTGTTCTCAAACCCGGTATGGTGGTCACCTTTGCTCCAGTCAACGTTACAACGGAAGT
AAAATCTGTCGAAATGCACCATGAAGCTTT 6 (0.000180%)

GATAAACTGAAAGCTGAGCGTGAACGTGGTATCACCATTGATATCTCCTTGTGGAAATTTGAGACCAGC
AAGTACTATGTGACTATCATTGATGCCCCAG 54 (0.001623%)

GATAATCCTATTTATTACCTCAGAAGTTTTTTTCTTCGCAGGATTTTTCTGAGCCTTTTACCACTCCAGCCT
AGCCCCTACCCCCCAATTAGGAGGGCAC 9 (0.000270%)

GATACGGGATAATCCTATTTATTACCTCAGAAGTTTTTTTCTTCGCAGGATTTTTCTGAGCCTTTTACCAC
TCCAGCCTAGCCCCTACCCCCCAATTAGG 4 (0.000120%)

GATATCTCCTTGTGGAAATTTGAGACCAGCAAGTACTATGTGACTATCATTGATGCCCCAGGACACAGAG
ACTTTATCAAAAACATGATTACAGGGACAT 15 (0.000451%)

GATATGAGACAGACAGTTGCGGTGGGTGTCATCAAAGCAGTGGACAAGAAGGCTGCTGGAGCTGGCAA
GGTCACCAAGTCTGCCCAGAAAGCTCAGAAGG 5 (0.000150%)

GATATGGTTCCTGGCAAGCCCATGTGTGTTGAGAGCTTCTCAGACTATCCACCTTTGGGTCGCTTTGCTGT
TCGTGATATGAGACAGACAGTTGCGGTGG9 (0.000270%)

GATCCGTGGACAGAGGAAGGGCGCCGGGTCTGTGTTCCGCGCGCACGTGAAGCACCGTAAAGGCGCTGC
GCGCCTGCGCGCCGTGGATTTCGCTGAGCGG 429 (0.012890%)

GATCTCCTTCATCCCTCTCCAGAAGAGGAGAAGAGGAAACACAAGAAGAAACGCCTGGTGCAGAGCCCC
AATTCCTACTTCATGGATGTGAAATGCCCAG 4 (0.000120%)

GATGCCCCAGGACACAGAGACTTTATCAAAAACATGATTACAGGGACATCTCAGGCTGACTGTGCTGTC
CTGATTGTTGCTGCTGGTGTTGGTGAATTTG 4 (0.000120%)

GATGCTGCCATTGTTGATATGGTTCCTGGCAAGCCCATGTGTGTTGAGAGCTTCTCAGACTATCCACCTTT
GGGTCGCTTTGCTGTTCGTGATATGAGAC 8 (0.000240%)

GATGGAAGCAATTTTGGAGGTGGTGGAAGCTACAATGATTTTGGGAATTACAACAATCAGTCTTCAAAT
TTTGGACCCATGAAGGGAGGAAATTTTGGAG 12 (0.000361%)

GATGGCCCTAAATTCTTGAAGTCTGGTGATGCTGCCATTGTTGATATGGTTCCTGGCAAGCCCATGTGTG
TTGAGAGCTTCTCAGACTATCCACCTTTGG 28 (0.000841%)

GATGGTTACTTGCTTCGTCTGTTCTGTGTTGGTTTACTAAAAAACGCAACAATCAGATACGGAAGACCT
CTTATGCTCAGCACCAACAGGTCCGCCAAA 5 (0.000150%)

GATTACAGGGACATCTCAGGCTGACTGTGCTGTCCTGATTGTTGCTGCTGGTGTTGGTGAATTTGAAGCT
GGTATCTCCAAGAATGGGCAGACCCGAGAG 6 (0.000180%)

GATTCAACTTCACCCGTAACCCACCGCCATGGCCGAGGAAGGCATTGCTGCTGGAGGTGTAATGGACGT
TAATACTGCTTTACAAGAGGTTCTGAAGACT 3 (0.000090%)

GATTCGGGCAAGTCCACCACTACTGGCCATCTGATCTATAAATGCGGTGGCATCGACAAAAGAACCATT
GAAAAATTTGAGAAGGAGGCTGCTGAGATGG 18 (0.000541%)

GATTGCCACACGGCTCACATTGCATGCAAGTTTGCTGAGCTGAAGGAAAAGATTGATCGCCGTTCTGGTA
AAAAGCTGGAAGATGGCCCTAAATTCTTGA 25 (0.000751%)

GATTTTGGGAATTACAACAATCAGTCTTCAAATTTTGGACCCATGAAGGGAGGAAATTTTGGAGGCAGA
AGCTCTGGCCCCTATGGCGGTGGAGGCCAAT 5 (0.000150%)

GCAAAAGAAGAAAGATGAGGCAGAGGTCCAAGTAAACCGCTAGCTTGTTGCACCGTGGAGGCCACAGG
AGCAGAAACATGGAATGCCAGACGCTGGGGAT 5 (0.000150%)

GCAAAGGATCTCCTTCATCCCTCTCCAGAAGAGGAGAAGAGGAAACACAAGAAGAAACGCCTGGTGCA
GAGCCCCAATTCCTACTTCATGGATGTGAAAT 3 (0.000090%)

GCAAAGGCCCCAACGTTGTAGGCCCCTACGGGCTACTACAACCCTTCGCTGACGCCATAAAACTCTTCAC
CAAAGAGCCCCTAAAACCCGCCACATCTAC 277 (0.008323%)

GCAACCTTCTAGGTAACGACCACATCTACAACGTTATCGTCACAGCCCATGCATTTGTAATAATCTTCTT
CATAGTAATACCCATCATAATCGGAGGCTT 12 (0.000361%)

GCAAGCCCATGTGTGTTGAGAGCTTCTCAGACTATCCACCTTTGGGTCGCTTTGCTGTTCGTGATATGAG
ACAGACAGTTGCGGTGGGTGTCATCAAAGC 15 (0.000451%)

GCAAGTACTATGTGACTATCATTGATGCCCCAGGACACAGAGACTTTATCAAAAACATGATTACAGGGA
CATCTCAGGCTGACTGTGCTGTCCTGATTGT 19 (0.000571%)

GCAAGTCCACCACTACTGGCCATCTGATCTATAAATGCGGTGGCATCGACAAAAGAACCATTGAAAAAT
TTGAGAAGGAGGCTGCTGAGATGGGAAAGGG 8 (0.000240%)

GCAATTTTGGAGGTGGTGGAAGCTACAATGATTTTGGGAATTACAACAATCAGTCTTCAAATTTTGGACC
CATGAAGGGAGGAAATTTTGGAGGCAGAAG 39 (0.001172%)

GCACAGCTCTAAGCCTCCTTATTCGAGCCGAGCTGGGCCAGCCAGGCAACCTTCTAGGTAACGACCACA
TCTACAACGTTATCGTCACAGCCCATGCATT 4 (0.000120%)

GCACATACTTCCTATTCTACACCCTAGTAGGCTCCCTTCCCCTACTCATCGCACTGATTTACACTCACAAC
ACCCTAGGCTCACTAAACATTCTACTACT 5 (0.000150%)

GCACCCGATATATGTTCTCTAGGCCTTTTAGAAAACATGGAGTTGTTCCTTTGGCCACATATATGCGAAT
CTATAAGAAAGGTGATATTGTAGACATCAA 5 (0.000150%)

GCACCGTAAAGGCGCTGCGCGCCTGCGCGCCGTGGATTTCGCTGAGCGGCACGGCTACATCAAGGGCAT
CGTCAAGGACATCATCCACGACCCGGGCCGC 5 (0.000150%)

GCAGACCCGAGAGCATGCCCTTCTGGCTTACACACTGGGTGTGAAACAACTAATTGTCGGTGTTAACAA
AATGGATTCCACTGAGCCACCCTACAGCCAG 15 (0.000451%)

GCAGCACCAGCGGTGGCAGAGACCCCAGACATCAAGCTCTTTGGGAAGTGGAGCACCGATGATGTGCAG
ATCAATGACATTTCCCTGCAGGATTACATTG 14 (0.000421%)

GCAGGCACATACTTCCTATTCTACACCCTAGTAGGCTCCCTTCCCCTACTCATCGCACTGATTTACACTCA CAACACCCTAGGCTCACTAAACATTCTAC 7 (0.000210%)

GCAGGCTGAAGCGCAAAAGAAGAAAGATGAGGCAGAGGTCCAAGTAAACCGCTAGCTTGTTGCACCGT GGAGGCCACAGGAGCAGAAACATGGAATGCCA 18 (0.000541%)

GCAGTGGACAAGAAGGCTGCTGGAGCTGGCAAGGTCACCAAGTCTGCCCAGAAAGCTCAGAAGGCTAA ATGAATATTATCCCTAATACCTGCCACCCCAC 23 (0.000691%)

GCATCCTTTACATAACAGACGAGGTCAACGATCCCTCCCTTACCATCAAATCAATTGGCCACCAATGGTA CTGAACCTACGAGTACACCGACTACGGCGG 21 (0.000631%)

GCATCGACAAAAGAACCATTGAAAAATTTGAGAAGGAGGCTGCTGAGATGGGAAAGGGCTCCTTCAAG TATGCCTGGGTCTTGGATAAACTGAAAGCTGA 7 (0.000210%)

GCATGCCCTTCTGGCTTACACACTGGGTGTGAAACAACTAATTGTCGGTGTTAACAAAATGGATTCCACT GAGCCACCCTACAGCCAGAAGAGATATGAG 13 (0.000391%)

GCATTTGTGCCAATTTCTGGTTGGAATGGTGACAACATGCTGGAGCCAAGTGCTAACATGCCTTGGTTCA AGGGATGGAAAGTCACCCGTAAGGATGGCA 3 (0.000090%)

GCCAAGTGCTAACATGCCTTGGTTCAAGGGATGGAAAGTCACCCGTAAGGATGGCAATGCCAGTGGAAC CACGCTGCTTGAGGCTCTGGACTGCATCCTA 23 (0.000691%)

GCCACAACTAACCTCCTCGGACTCCTGCCTCACTCATTTACACCAACCACCCAACTATCTATAAACCTAG CCATGGCCATCCCCTTATGAGCGGGCGCAG 15 (0.000451%)

GCCACACGGCTCACATTGCATGCAAGTTTGCTGAGCTGAAGGAAAAGATTGATCGCCGTTCTGGTAAAA AGCTGGAAGATGGCCCTAAATTCTTGAAGTC 19 (0.000571%)

GCCAGAACACAGGTGTCGTGAAAACTACCCCTAAAAGCCAAAATGGGAAAGGAAAAGACTCATATCAA CATTGTCGTCATTGGACACGTAGATTCGGGCA 526 (0.015805%)

GCCAGAACGCCTGAACGCAGGCACATACTTCCTATTCTACACCCTAGTAGGCTCCCTTCCCCTACTCATC GCACTGATTTACACTCACAACACCCTAGGC 392 (0.011778%)

GCCAGGCAACCTTCTAGGTAACGACCACATCTACAACGTTATCGTCACAGCCCATGCATTTGTAATAATC TTCTTCATAGTAATACCCATCATAATCGGA 13 (0.000391%)

GCCATCATCCTAGTCCTCATCGCCCTCCCATCCCTACGCATCCTTTACATAACAGACGAGGTCAACGATC CCTCCCTTACCATCAAATCAATTGGCCACC 5 (0.000150%)

GCCATCTCCTGGGCCGCCTGGCGGCCATCGTGGCTAAACAGGTACTGCTGGGCCGGAAGGTGGTGGTCG TACGCTGTGAAGGCATCAACATTTCTGGCAA 24 (0.000721%)

GCCATTGTTGATATGGTTCCTGGCAAGCCCATGTGTGTTGAGAGCTTCTCAGACTATCCACCTTTGGGTC GCTTTGCTGTTCGTGATATGAGACAGACAG 10 (0.000300%)

GCCCATAAAAATAAAAAATTATAACAAACCCTGAGAACCAAAATGAACGAAAATCTGTTCGCTTCATTC ATTGCCCCCACAATCCTAGGCCTACCCGCCG 9 (0.000270%)

GCCCATGACCCCTAACAGGGGCCCTCTCAGCCCTCCTAATGACCTCCGGCCTAGCCATGTGATTTCACTT CCACTCCATAACGCTCCTCATACTAGGCCT 27 (0.000811%)

GCCCATGTGTGTTGAGAGCTTCTCAGACTATCCACCTTTGGGTCGCTTTGCTGTTCGTGATATGAGACAG ACAGTTGCGGTGGGTGTCATCAAAGCAGTG 5 (0.000150%)

GCCCGCCATCATCCTAGTCCTCATCGCCCTCCCATCCCTACGCATCCTTTACATAACAGACGAGGTCAAC

GATCCCTCCCTTACCATCAAATCAATTGGC 10 (0.000300%)

GCCCTAAATTCTTGAAGTCTGGTGATGCTGCCATTGTTGATATGGTTCCTGGCAAGCCCATGTGTGTTGA
GAGCTTCTCAGACTATCCACCTTTGGGTCG 9 (0.000270%)

GCCCTCCCATCCCTACGCATCCTTTACATAACAGACGAGGTCAACGATCCCTCCCTTACCATCAAATCAA
TTGGCCACCAATGGTACTGAACCTACGAGT 9 (0.000270%)

GCCCTCTCAGCCCTCCTAATGACCTCCGGCCTAGCCATGTGATTTCACTTCCACTCCATAACGCTCCTCAT
ACTAGGCCTACTAACCAACACACTAACCA 14 (0.000421%)

GCCCTTCTGGCTTACACACTGGGTGTGAAACAACTAATTGTCGGTGTTAACAAAATGGATTCCACTGAGC
CACCCTACAGCCAGAAGAGATATGAGGAAA 9 (0.000270%)

GCCGAGTGGAGACTGGTGTTCTCAAACCCGGTATGGTGGTCACCTTTGCTCCAGTCAACGTTACAACGGA
AGTAAAATCTGTCGAAATGCACCATGAAGC 20 (0.000601%)

GCCGGACGGGCACTGGGCGACTCTGTGCCTCGCTGAGGAAAAATAACTAAACATGGGCAAAGGAGATC
CTAAGAAGCCGAGAGGCAAAATGTCATCATAT 22 (0.000661%)

GCCGGGTCTGTGTTCCGCGCGCACGTGAAGCACCGTAAAGGCGCTGCGCGCCTGCGCGCCGTGGATTTC
GCTGAGCGGCACGGCTACATCAAGGGCATCG 25 (0.000751%)

GCCTCACTCATTTACACCAACCACCCAACTATCTATAAACCTAGCCATGGCCATCCCCTTATGAGCGGGC
GCAGTGATTATAGGCTTTCGCTCTAAGATT 18 (0.000541%)

GCCTCTCGCAAAGGATCTCCTTCATCCCTCTCCAGAAGAGGAGAAGAGGAAACACAAGAAGAAACGCCT
GGTGCAGAGCCCCAATTCCTACTTCATGGAT 630 (0.018930%)

GCCTGAACGCAGGCACATACTTCCTATTCTACACCCTAGTAGGCTCCCTTCCCCTACTCATCGCACTGATT
TACACTCACAACACCCTAGGCTCACTAAA 13 (0.000391%)

GCCTGGGTCTTGGATAAACTGAAAGCTGAGCGTGAACGTGGTATCACCATTGATATCTCCTTGTGGAAAT
TTGAGACCAGCAAGTACTATGTGACTATCA 9 (0.000270%)

GCCTGGTGCGGGAGCTACGGGGCCCAGGGATTGTGTTTAAAGTAGTGCTTCTACCAACATGTCCCGTGGT
TCCAGCGCCGGTTTTGACCGCCACATTACC 6 (0.000180%)

GCCTTCGATACGGGATAATCCTATTTATTACCTCAGAAGTTTTTTTCTTCGCAGGATTTTTCTGAGCCTTTT
ACCACTCCAGCCTAGCCCCTACCCCCCA 18 (0.000541%)

GCGAATGCGCAGGCTGAAGCGCAAAAGAAGAAAGATGAGGCAGAGGTCCAAGTAAACCGCTAGCTTGT
TGCACCGTGGAGGCCACAGGAGCAGAAACATG 52 (0.001562%)

GCGACTCTGTGCCTCGCTGAGGAAAAATAACTAAACATGGGCAAAGGAGATCCTAAGAAGCCGAGAGG
CAAAATGTCATCATATGCATTTTTTGTGCAAA 9 (0.000270%)

GCGCAAAAGAAGAAAGATGAGGCAGAGGTCCAAGTAAACCGCTAGCTTGTTGCACCGTGGAGGCCACA
GGAGCAGAAACATGGAATGCCAGACGCTGGGG 6 (0.000180%)

GCGCCGGGTCTGTGTTCCGCGCGCACGTGAAGCACCGTAAAGGCGCTGCGCGCCTGCGCGCCGTGGATT
TCGCTGAGCGGCACGGCTACATCAAGGGCAT 5 (0.000150%)

GCGCGCACGTGAAGCACCGTAAAGGCGCTGCGCGCCTGCGCGCCGTGGATTTCGCTGAGCGGCACGGCT
ACATCAAGGGCATCGTCAAGGACATCATCCA 3 (0.000090%)

GCGCTGCGCGCCTGCGCGCCGTGGATTTCGCTGAGCGGCACGGCTACATCAAGGGCATCGTCAAGGACA
TCATCCACGACCCGGGCCGCGGCGCGCCCCT 4 (0.000120%)

GCGGGAGCTACGGGGCCCAGGGATTGTGTTTAAAGTAGTGCTTCTACCAACATGTCCCGTGGTTCCAGCG
CCGGTTTTGACCGCCACATTACCATTTTTT 5 (0.000150%)

GCGGTCTGCCCCTGGAGGTGGTAGCAAGGTTCCACAGAAAAAAGTAAAACTTGCTGCTGATGAAGATGA
TGACGATGATGATGAAGAGGATGATGATGAA 4 (0.000120%)

GCGGTGGCATCGACAAAAGAACCATTGAAAAATTTGAGAAGGAGGCTGCTGAGATGGGAAAGGGCTCC
TTCAAGTATGCCTGGGTCTTGGATAAACTGAA 14 (0.000421%)

GCGGTGGGTGTCATCAAAGCAGTGGACAAGAAGGCTGCTGGAGCTGGCAAGGTCACCAAGTCTGCCCAG
AAAGCTCAGAAGGCTAAATGAATATTATCCC 25 (0.000751%)

GCGTGAACGTGGTATCACCATTGATATCTCCTTGTGGAAATTTGAGACCAGCAAGTACTATGTGACTATC
ATTGATGCCCCAGGACACAGAGACTTTATC 20 (0.000601%)

GCGTTTCCCCGCATAAACAACATAAGCTTCTGACTCTTACCTCCCTCTCTCCTACTCCTGCTCGCATCTGC
TATAGTGGAGGCCGGAGCAGGAACAGGTT 7 (0.000210%)

GCTACAATGATTTTGGGAATTACAACAATCAGTCTTCAAATTTTGGACCCATGAAGGGAGGAAATTTTGG
AGGCAGAAGCTCTGGCCCCTATGGCGGTGG 43 (0.001292%)

GCTACGAGCTGCCTGACGGCCAGGTCATCACCATTGGCAATGAGCGGTTCCGCTGCCCTGAGGCACTCTT
CCAGCCTTCCTTCCTGGGCATGGAGTCCTG 6 (0.000180%)

GCTCCTCCCTGGAGAAGAGCTACGAGCTGCCTGACGGCCAGGTCATCACCATTGGCAATGAGCGGTTCC
GCTGCCCTGAGGCACTCTTCCAGCCTTCCTT 5 (0.000150%)

GCTCCTTCAAGTATGCCTGGGTCTTGGATAAACTGAAAGCTGAGCGTGAACGTGGTATCACCATTGATAT
CTCCTTGTGGAAATTTGAGACCAGCAAGTA 120 (0.003606%)

GCTGACTGTGCTGTCCTGATTGTTGCTGCTGGTGTTGGTGAATTTGAAGCTGGTATCTCCAAGAATGGGC
AGACCCGAGAGCATGCCCTTCTGGCTTACA 25 (0.000751%)

GCTGAGCGTGAACGTGGTATCACCATTGATATCTCCTTGTGGAAATTTGAGACCAGCAAGTACTATGTGA
CTATCATTGATGCCCCAGGACACAGAGACT 9 (0.000270%)

GCTGAGGAAAAATAACTAAACATGGGCAAAGGAGATCCTAAGAAGCCGAGAGGCAAAATGTCATCATA
TGCATTTTTTGTGCAAACTTGTCGGGAGGAGC 4 (0.000120%)

GCTGCCATTGTTGATATGGTTCCTGGCAAGCCCATGTGTGTTGAGAGCTTCTCAGACTATCCACCTTTGG
GTCGCTTTGCTGTTCGTGATATGAGACAGA 3 (0.000090%)

GCTGCTGGAGCTGGCAAGGTCACCAAGTCTGCCCAGAAAGCTCAGAAGGCTAAATGAATATTATCCCTA
ATACCTGCCACCCCACTCTTAATCAGTGGTG 21 (0.000631%)

GCTGGAAGATGGCCCTAAATTCTTGAAGTCTGGTGATGCTGCCATTGTTGATATGGTTCCTGGCAAGCCC
ATGTGTGTTGAGAGCTTCTCAGACTATCCA 7 (0.000210%)

GCTGGAGCTGGCAAGGTCACCAAGTCTGCCCAGAAAGCTCAGAAGGCTAAATGAATATTATCCCTAATA
CCTGCCACCCCACTCTTAATCAGTGGTGGAA 19 (0.000571%)

GCTGGCAAGGTCACCAAGTCTGCCCAGAAAGCTCAGAAGGCTAAATGAATATTATCCCTAATACCTGCC
ACCCCACTCTTAATCAGTGGTGGAAGAACGG 4 (0.000120%)

GCTGTCCTGATTGTTGCTGCTGGTGTTGGTGAATTTGAAGCTGGTATCTCCAAGAATGGGCAGACCCGAG
AGCATGCCCTTCTGGCTTACACACTGGGTG 8 (0.000240%)

GCTTACACACTGGGTGTGAAACAACTAATTGTCGGTGTTAACAAAATGGATTCCACTGAGCCACCCTACA
GCCAGAAGAGATATGAGGAAATTGTTAAGG 3 (0.000090%)

GCTTCAATGTCAAGAATGTGTCTGTCAAGGATGTTCGTCGTGGCAACGTTGCTGGTGACAGCAAAAATG
ACCCACCAATGGAAGCAGCTGGCTTCACTGC 11 (0.000331%)

GCTTCTCAGACTATCCACCTTTGGGTCGCTTTGCTGTTCGTGATATGAGACAGACAGTTGCGGTGGGTGT
CATCAAAGCAGTGGACAAGAAGGCTGCTGG 38 (0.001142%)

GCTTGATGGTCGAGGCCATCTCCTGGGCCGCCTGGCGGCCATCGTGGCTAAACAGGTACTGCTGGGCCG
GAAGGTGGTGGTCGTACGCTGTGAAGGCATC 1076 (0.032331%)

GGAAAGCGGTCTGCCCCTGGAGGTGGTAGCAAGGTTCCACAGAAAAAAGTAAAACTTGCTGCTGATGAA
GATGATGACGATGATGATGAAGAGGATGATG 3 (0.000090%)

GGAACTTCGCATCCGCAAACTCTGTCTCAACATCTGTGTTGGGGAGAGTGGAGACAGACTGACGCGAGC
AGCCAAGGTGTTGGAGCAGCTCACAGGGCAG 456 (0.013701%)

GGAAGATGGCCCTAAATTCTTGAAGTCTGGTGATGCTGCCATTGTTGATATGGTTCCTGGCAAGCCCATG
TGTGTTGAGAGCTTCTCAGACTATCCACCT 138 (0.004146%)

GGAAGCAATTTTGGAGGTGGTGGAAGCTACAATGATTTTGGGAATTACAACAATCAGTCTTCAAATTTTG
GACCCATGAAGGGAGGAAATTTTGGAGGCA 13 (0.000391%)

GGAAGCTACAATGATTTTGGGAATTACAACAATCAGTCTTCAAATTTTGGACCCATGAAGGGAGGAAAT
TTTGGAGGCAGAAGCTCTGGCCCCTATGGCG 65 (0.001953%)

GGAAGGGCGCCGGGTCTGTGTTCCGCGCGCACGTGAAGCACCGTAAAGGCGCTGCGCGCCTGCGCGCCG
TGGATTTCGCTGAGCGGCACGGCTACATCAA 23 (0.000691%)

GGAATAATCTCCCATATTGTAACTTACTACTCCGGAAAAAAAGAACCATTTGGATACATAGGTATGGTCT
GAGCTATGATATCAATTGGCTTCCTAGGGT 9 (0.000270%)

GGAATCACCTCCCATTCCGATAAAATCACCTTCCACCCTTACTACACAATCAAAGACGCCCTCGGCTTAC
TTCTCTTCATTCTCTCCTTAATGACATTAA 5 (0.000150%)

GGACAAGAAGGCTGCTGGAGCTGGCAAGGTCACCAAGTCTGCCCAGAAAGCTCAGAAGGCTAAATGAA
TATTATCCCTAATACCTGCCACCCCACTCTTA 81 (0.002434%)

GGACACAGAGACTTTATCAAAAACATGATTACAGGGACATCTCAGGCTGACTGTGCTGTCCTGATTGTTG
CTGCTGGTGTTGGTGAATTTGAAGCTGGTA 14 (0.000421%)

GGACACGTAGATTCGGGCAAGTCCACCACTACTGGCCATCTGATCTATAAATGCGGTGGCATCGACAAA
AGAACCATTGAAAAATTTGAGAAGGAGGCTG 17 (0.000511%)

GGACAGAGGAAGGGCGCCGGGTCTGTGTTCCGCGCGCACGTGAAGCACCGTAAAGGCGCTGCGCGCCTG
CGCGCCGTGGATTTCGCTGAGCGGCACGGCT 20 (0.000601%)

GGACATCTCAGGCTGACTGTGCTGTCCTGATTGTTGCTGCTGGTGTTGGTGAATTTGAAGCTGGTATCTCC
AAGAATGGGCAGACCCGAGAGCATGCCCT 32 (0.000962%)

GGACGGGCACTGGGCGACTCTGTGCCTCGCTGAGGAAAAATAACTAAACATGGGCAAAGGAGATCCTA
AGAAGCCGAGAGGCAAAATGTCATCATATGCA 14 (0.000421%)

GGACTCCTGCCTCACTCATTTACACCAACCACCCAACTATCTATAAACCTAGCCATGGCCATCCCCTTAT
GAGCGGGCGCAGTGATTATAGGCTTTCGCT 6 (0.000180%)

GGAGACCCCATTCTATACCAACACCTATTCTGATTTTTCGGTCACCCTGAAGTTTATATTCTTATCCTACC
AGGCTTCGGAATAATCTCCCATATTGTAA 78 (0.002344%)

GGAGACTGGTGTTCTCAAACCCGGTATGGTGGTCACCTTTGCTCCAGTCAACGTTACAACGGAAGTAAA

ATCTGTCGAAATGCACCATGAAGCTTTGAGT 40 (0.001202%)

GGAGAGGCACCCGATATATGTTCTCTAGGCCTTTTAGAAAACATGGAGTTGTTCCTTTGGCCACATATAT GCGAATCTATAAGAAAGGTGATATTGTAGA 9 (0.000270%)

GGAGCCAAGTGCTAACATGCCTTGGTTCAAGGGATGGAAAGTCACCCGTAAGGATGGCAATGCCAGTGG AACCACGCTGCTTGAGGCTCTGGACTGCATC 4 (0.000120%)

GGAGCTGGCAAGGTCACCAAGTCTGCCCAGAAAGCTCAGAAGGCTAAATGAATATTATCCCTAATACCT GCCACCCCACTCTTAATCAGTGGTGGAAGAA 7 (0.000210%)

GGAGGAGACCCCATTCTATACCAACACCTATTCTGATTTTTCGGTCACCCTGAAGTTTATATTCTTATCCT ACCAGGCTTCGGAATAATCTCCCATATTG 56 (0.001683%)

GGATAAACTGAAAGCTGAGCGTGAACGTGGTATCACCATTGATATCTCCTTGTGGAAATTTGAGACCAG CAAGTACTATGTGACTATCATTGATGCCCCA 14 (0.000421%)

GGATAATCCTATTTATTACCTCAGAAGTTTTTTTCTTCGCAGGATTTTTCTGAGCCTTTTACCACTCCAGC CTAGCCCCTACCCCCCAATTAGGAGGGCA 91 (0.002734%)

GGATCTCCTTCATCCCTCTCCAGAAGAGGAGAAGAGGAAACACAAGAAGAAACGCCTGGTGCAGAGCC CCAATTCCTACTTCATGGATGTGAAATGCCCA 4 (0.000120%)

GGATGGAAAGTCACCCGTAAGGATGGCAATGCCAGTGGAACCACGCTGCTTGAGGCTCTGGACTGCATC CTACCACCAACTCGTCCAACTGACAAGCCCT 4 (0.000120%)

GGATTGCCACACGGCTCACATTGCATGCAAGTTTGCTGAGCTGAAGGAAAAGATTGATCGCCGTTCTGGT AAAAAGCTGGAAGATGGCCCTAAATTCTTG 4 (0.000120%)

GGATTGTGTTTAAAGTAGTGCTTCTACCAACATGTCCCGTGGTTCCAGCGCCGGTTTTGACCGCCACATT ACCATTTTTTCACCCGAGGGTCGGCTCTAC 3 (0.000090%)

GGCAACCTTCTAGGTAACGACCACATCTACAACGTTATCGTCACAGCCCATGCATTTGTAATAATCTTCT TCATAGTAATACCCATCATAATCGGAGGCT 7 (0.000210%)

GGCAAGCCCATGTGTGTTGAGAGCTTCTCAGACTATCCACCTTTGGGTCGCTTTGCTGTTCGTGATATGA GACAGACAGTTGCGGTGGGTGTCATCAAAG 40 (0.001202%)

GGCAAGTCCACCACTACTGGCCATCTGATCTATAAATGCGGTGGCATCGACAAAAGAACCATTGAAAAA TTTGAGAAGGAGGCTGCTGAGATGGGAAAGG 72 (0.002163%)

GGCACAGCTCTAAGCCTCCTTATTCGAGCCGAGCTGGGCCAGCCAGGCAACCTTCTAGGTAACGACCAC ATCTACAACGTTATCGTCACAGCCCATGCAT 9 (0.000270%)

GGCACATACTTCCTATTCTACACCCTAGTAGGCTCCCTTCCCCTACTCATCGCACTGATTTACACTCACAA CACCCTAGGCTCACTAAACATTCTACTAC 4 (0.000120%)

GGCACCCGATATATGTTCTCTAGGCCTTTTAGAAAACATGGAGTTGTTCCTTTGGCCACATATATGCGAA TCTATAAGAAAGGTGATATTGTAGACATCA 9 (0.000270%)

GGCACTGGGCGACTCTGTGCCTCGCTGAGGAAAAATAACTAAACATGGGCAAAGGAGATCCTAAGAAG CCGAGAGGCAAAATGTCATCATATGCATTTTT 4 (0.000120%)

GGCAGACCCGAGAGCATGCCCTTCTGGCTTACACACTGGGTGTGAAACAACTAATTGTCGGTGTTAACA AAATGGATTCCACTGAGCCACCCTACAGCCA 22 (0.000661%)

GGCATCGACAAAAGAACCATTGAAAAATTTGAGAAGGAGGCTGCTGAGATGGGAAAGGGCTCCTTCAA GTATGCCTGGGTCTTGGATAAACTGAAAGCTG 8 (0.000240%)

GGCCATCTCCTGGGCCGCCTGGCGGCCATCGTGGCTAAACAGGTACTGCTGGGCCGGAAGGTGGTGGTC
GTACGCTGTGAAGGCATCAACATTTCTGGCA 5 (0.000150%)

GGCCCTAAATTCTTGAAGTCTGGTGATGCTGCCATTGTTGATATGGTTCCTGGCAAGCCCATGTGTGTTG
AGAGCTTCTCAGACTATCCACCTTTGGGTC 6 (0.000180%)

GGCCCTCTCAGCCCTCCTAATGACCTCCGGCCTAGCCATGTGATTTCACTTCCACTCCATAACGCTCCTCA
TACTAGGCCTACTAACCAACACACTAACC 8 (0.000240%)

GGCCGAGTGGAGACTGGTGTTCTCAAACCCGGTATGGTGGTCACCTTTGCTCCAGTCAACGTTACAACGG
AAGTAAAATCTGTCGAAATGCACCATGAAG 8 (0.000240%)

GGCCTTCGATACGGGATAATCCTATTTATTACCTCAGAAGTTTTTTCTTCGCAGGATTTTTCTGAGCCTT
TTACCACTCCAGCCTAGCCCCTACCCCCC 6 (0.000180%)

GGCGACTCTGTGCCTCGCTGAGGAAAAATAACTAAACATGGGCAAAGGAGATCCTAAGAAGCCGAGAG
GCAAAATGTCATCATATGCATTTTTGTGCAA 9 (0.000270%)

GGCGCCGGGTCTGTGTTCCGCGCGCACGTGAAGCACCGTAAAGGCGCTGCGCGCCTGCGCGCCGTGGAT
TTCGCTGAGCGGCACGGCTACATCAAGGGCA 6 (0.000180%)

GGCGCTGCGCGCCTGCGCGCCGTGGATTTCGCTGAGCGGCACGGCTACATCAAGGGCATCGTCAAGGAC
ATCATCCACGACCCGGGCCGCGGCGCGCCCC 3 (0.000090%)

GGCGTTTCCCCGCATAAACAACATAAGCTTCTGACTCTTACCTCCCTCTCTCCTACTCCTGCTCGCATCTG
CTATAGTGGAGGCCGGAGCAGGAACAGGT 25 (0.000751%)

GGCTCCCTTCCCCTACTCATCGCACTGATTTACACTCACAACACCCTAGGCTCACTAAACATTCTACTACT
CACTCTCACTGCCCAAGAACTATCAAACT 3 (0.000090%)

GGCTCCTTCAAGTATGCCTGGGTCTTGGATAAACTGAAAGCTGAGCGTGAACGTGGTATCACCATTGATA
TCTCCTTGTGGAAATTTGAGACCAGCAAGT 93 (0.002794%)

GGCTGAAGCGCAAAAGAAGAAAGATGAGGCAGAGGTCCAAGTAAACCGCTAGCTTGTTGCACCGTGGA
GGCCACAGGAGCAGAAACATGGAATGCCAGAC 3 (0.000090%)

GGCTGACTGTGCTGTCCTGATTGTTGCTGCTGGTGTTGGTGAATTTGAAGCTGGTATCTCCAAGAATGGG
CAGACCCGAGAGCATGCCCTTCTGGCTTAC 29 (0.000871%)

GGCTGCTGGAGCTGGCAAGGTCACCAAGTCTGCCCAGAAAGCTCAGAAGGCTAAATGAATATTATCCCT
AATACCTGCCACCCCACTCTTAATCAGTGGT 64 (0.001923%)

GGCTGCTTCCAGCTCCTCCCTGGAGAAGAGCTACGAGCTGCCTGACGGCCAGGTCATCACCATTGGCAAT
GAGCGGTTCCGCTGCCCTGAGGCACTCTTC 15 (0.000451%)

GGCTTACACACTGGGTGTGAAACAACTAATTGTCGGTGTTAACAAAATGGATTCCACTGAGCCACCCTAC
AGCCAGAAGAGATATGAGGAAATTGTTAAG 7 (0.000210%)

GGCTTCAATGTCAAGAATGTGTCTGTCAAGGATGTTCGTCGTGGCAACGTTGCTGGTGACAGCAAAAAT
GACCCACCAATGGAAGCAGCTGGCTTCACTG 19 (0.000571%)

GGGAATTACAACAATCAGTCTTCAAATTTTGGACCCATGAAGGGAGGAAATTTTGGAGGCAGAAGCTCT
GGCCCCTATGGCGGTGGAGGCCAATACTTTG 32 (0.000962%)

GGGACATCTCAGGCTGACTGTGCTGTCCTGATTGTTGCTGCTGGTGTTGGTGAATTTGAAGCTGGTATCT
CCAAGAATGGGCAGACCCGAGAGCATGCCC 165 (0.004958%)

GGGAGCTACGGGGCCCAGGGATTGTGTTTAAAGTAGTGCTTCTACCAACATGTCCCGTGGTTCCAGCGCC
GGTTTTGACCGCCACATTACCATTTTTTCA 3 (0.000090%)

GGGATAATCCTATTTATTACCTCAGAAGTTTTTTCTTCGCAGGATTTTCTGAGCCTTTTACCACTCCAG
CCTAGCCCCTACCCCCCAATTAGGAGGGC 5 (0.000150%)

GGGATGGAAAGTCACCCGTAAGGATGGCAATGCCAGTGGAACCACGCTGCTTGAGGCTCTGGACTGCAT
CCTACCACCAACTCGTCCAACTGACAAGCCC 12 (0.000361%)

GGGCAAGTCCACCACTACTGGCCATCTGATCTATAAATGCGGTGGCATCGACAAAAGAACCATTGAAAA
ATTTGAGAAGGAGGCTGCTGAGATGGGAAAG 31 (0.000931%)

GGGCACTGGGCGACTCTGTGCCTCGCTGAGGAAAAATAACTAAACATGGGCAAAGGAGATCCTAAGAA
GCCGAGAGGCAAAATGTCATCATATGCATTTT 19 (0.000571%)

GGGCAGACCCGAGAGCATGCCCTTCTGGCTTACACACTGGGTGTGAAACAACTAATTGTCGGTGTTAAC
AAAATGGATTCCACTGAGCCACCCTACAGCC 15 (0.000451%)

GGGCCCTCTCAGCCCTCCTAATGACCTCCGGCCTAGCCATGTGATTTCACTTCCACTCCATAACGCTCCTC
ATACTAGGCCTACTAACCAACACACTAAC 28 (0.000841%)

GGGCGACTCTGTGCCTCGCTGAGGAAAAATAACTAAACATGGGCAAAGGAGATCCTAAGAAGCCGAGA
GGCAAAATGTCATCATATGCATTTTTGTGCA 6 (0.000180%)

GGGCGCCGGGTCTGTGTTCCGCGCGCACGTGAAGCACCGTAAAGGCGCTGCGCGCCTGCGCGCCGTGGA
TTTCGCTGAGCGGCACGGCTACATCAAGGGC 8 (0.000240%)

GGGCTCCTTCAAGTATGCCTGGGTCTTGGATAAACTGAAAGCTGAGCGTGAACGTGGTATCACCATTGAT
ATCTCCTTGTGGAAATTTGAGACCAGCAAG 30 (0.000901%)

GGGCTTCAATGTCAAGAATGTGTCTGTCAAGGATGTTCGTCGTGGCAACGTTGCTGGTGACAGCAAAAA
TGACCCACCAATGGAAGCAGCTGGCTTCACT 40 (0.001202%)

GGGGCCCTCTCAGCCCTCCTAATGACCTCCGGCCTAGCCATGTGATTTCACTTCCACTCCATAACGCTCCT
CATACTAGGCCTACTAACCAACACACTAA 19 (0.000571%)

GGGGGGGGGGGGGGGGGGGGGGGGGGGGGGGGGGGGGGGGGGGGGGGGGGGGGGGGGGGGGGGGGG
GGGGGGGGGGGGGGGGGGGGGGGGGGGGGGGGGG 6293 (0.189086%)

GGGGGGGGGGGGGGGGGGGGGGGGGGGGGGGGGGGGGGGGGGGGGGGGGGGGGGGGGGGGGGGGGG
GGGGGGGGGGGGGGGGGGGGGGGGGGGGGGGGGGGGGGGGGGGGGGGGGGGGGGGGGGGGGGGGGG
GGGGGGGGGGGGGGGGG 5064 (0.226716%)

GGGTCCCTCGGCTTCCTGCCTCGGAAGCGCAGCAGCAGGCATCGTGGGAAGGTGAAGAGCTTCCCTAAG
GATGACCCGTCCAAGCCGGTCCACCTCACAG 3 (0.000090%)

GGGTCGCTTTGCTGTTCGTGATATGAGACAGACAGTTGCGGTGGGTGTCATCAAAGCAGTGGACAAGAA
GGCTGCTGGAGCTGGCAAGGTCACCAAGTCT 3 (0.000090%)

GGGTCTTGGATAAACTGAAAGCTGAGCGTGAACGTGGTATCACCATTGATATCTCCTTGTGGAAATTTGA
GACCAGCAAGTACTATGTGACTATCATTGA 8 (0.000240%)

GGGTGTCATCAAAGCAGTGGACAAGAAGGCTGCTGGAGCTGGCAAGGTCACCAAGTCTGCCCAGAAAG
CTCAGAAGGCTAAATGAATATTATCCCTAATA 9 (0.000270%)

GGTAAAAAGCTGGAAGATGGCCCTAAATTCTTGAAGTCTGGTGATGCTGCCATTGTTGATATGGTTCCTG
GCAAGCCCATGTGTGTTGAGAGCTTCTCAG 9 (0.000270%)

GGTAATGATGGAAGCAATTTTGGAGGTGGTGGAAGCTACAATGATTTTGGGAATTACAACAATCAGTCT
TCAAATTTTGGACCCATGAAGGGAGGAAATT 3 (0.000090%)

GGTATCACCATTGATATCTCCTTGTGGAAATTTGAGACCAGCAAGTACTATGTGACTATCATTGATGCCC
CAGGACACAGAGACTTTATCAAAAACATGA 26 (0.000781%)

GGTATGGTGGTCACCTTTGCTCCAGTCAACGTTACAACGGAAGTAAAATCTGTCGAAATGCACCATGAA
GCTTTGAGTGAAGCTCTTCCTGGGGACAATG 7 (0.000210%)

GGTCAACGATCCCTCCCTTACCATCAAATCAATTGGCCACCAATGGTACTGAACCTACGAGTACACCGAC
TACGGCGGACTAATCTTCAACTCCTACATA 20 (0.000601%)

GGTCACCTTTGCTCCAGTCAACGTTACAACGGAAGTAAAATCTGTCGAAATGCACCATGAAGCTTTGAGT
GAAGCTCTTCCTGGGGACAATGTGGGCTTC 66 (0.001983%)

GGTCCCTCGGCTTCCTGCCTCGGAAGCGCAGCAGCAGGCATCGTGGGAAGGTGAAGAGCTTCCCTAAGG
ATGACCCGTCCAAGCCGGTCCACCTCACAGC 5 (0.000150%)

GGTCGAGGCCATCTCCTGGGCCGCCTGGCGGCCATCGTGGCTAAACAGGTACTGCTGGGCCGGAAGGTG
GTGGTCGTACGCTGTGAAGGCATCAACATTT 9 (0.000270%)

GGTCTGTGTTCCGCGCGCACGTGAAGCACCGTAAAGGCGCTGCGCGCCTGCGCGCCGTGGATTTCGCTG
AGCGGCACGGCTACATCAAGGGCATCGTCAA 6 (0.000180%)

GGTCTTGGATAAACTGAAAGCTGAGCGTGAACGTGGTATCACCATTGATATCTCCTTGTGGAAATTTGAG
ACCAGCAAGTACTATGTGACTATCATTGAT 3 (0.000090%)

GGTGATGCTGCCATTGTTGATATGGTTCCTGGCAAGCCCATGTGTGTTGAGAGCTTCTCAGACTATCCAC
CTTTGGGTCGCTTTGCTGTTCGTGATATGA26 (0.000781%)

GGTGCCTTGATGGCCTCCTCTCCTCAAGACATCAAATTTCAAGATTTGGTCGTCTTCATTTTGGAGAAGA
AAATGGGAACCACCCGCAGAGCGTTCCTCA 116 (0.003485%)

GGTGGAAGCTACAATGATTTTGGGAATTACAACAATCAGTCTTCAAATTTTGGACCCATGAAGGGAGGA
AATTTTGGAGGCAGAAGCTCTGGCCCCTATG 4 (0.000120%)

GGTGGCATCGACAAAAGAACCATTGAAAAATTTGAGAAGGAGGCTGCTGAGATGGGAAAGGGCTCCTT
CAAGTATGCCTGGGTCTTGGATAAACTGAAAG3 (0.000090%)

GGTGGGTGTCATCAAAGCAGTGGACAAGAAGGCTGCTGGAGCTGGCAAGGTCACCAAGTCTGCCCAGA
AAGCTCAGAAGGCTAAATGAATATTATCCCTA 13 (0.000391%)

GGTGGTCACCTTTGCTCCAGTCAACGTTACAACGGAAGTAAAATCTGTCGAAATGCACCATGAAGCTTTG
AGTGAAGCTCTTCCTGGGGACAATGTGGGC 18 (0.000541%)

GGTGTCATCAAAGCAGTGGACAAGAAGGCTGCTGGAGCTGGCAAGGTCACCAAGTCTGCCCAGAAAGCT
CAGAAGGCTAAATGAATATTATCCCTAATAC 5 (0.000150%)

GGTGTCGTGAAAACTACCCCTAAAAGCCAAAATGGGAAAGGAAAAGACTCATATCAACATTGTCGTCAT
TGGACACGTAGATTCGGGCAAGTCCACCACT 45 (0.001352%)

GGTGTTCTCAAACCCGGTATGGTGGTCACCTTTGCTCCAGTCAACGTTACAACGGAAGTAAAATCTGTCG
AAATGCACCATGAAGCTTTGAGTGAAGCTC 22 (0.000661%)

GGTTCCTGGCAAGCCCATGTGTGTTGAGAGCTTCTCAGACTATCCACCTTTGGGTCGCTTTGCTGTTCGTG
ATATGAGACAGACAGTTGCGGTGGGTGTC5 (0.000150%)

GTAAAAAGCTGGAAGATGGCCCTAAATTCTTGAAGTCTGGTGATGCTGCCATTGTTGATATGGTTCCTGG
CAAGCCCATGTGTGTTGAGAGCTTCTCAGA 43 (0.001292%)

GTAAAACCCAGCCCATGACCCCTAACAGGGGCCCTCTCAGCCCTCCTAATGACCTCCGGCCTAGCCATGT
GATTTCACTTCCACTCCATAACGCTCCTCA 903 (0.027132%)

GTAAAGGCGCTGCGCGCCTGCGCGCCGTGGATTTCGCTGAGCGGCACGGCTACATCAAGGGCATCGTCA
AGGACATCATCCACGACCCGGGCCGCGGCGC 10 (0.000300%)

GTAACTTACTACTCCGGAAAAAAAGAACCATTTGGATACATAGGTATGGTCTGAGCTATGATATCAATTG
GCTTCCTAGGGTTTATCGTGTGAGCACACC 25 (0.000751%)

GTAATGATGGAAGCAATTTTGGAGGTGGTGGAAGCTACAATGATTTTGGGAATTACAACAATCAGTCTT
CAAATTTTGGACCCATGAAGGGAGGAAATTT 5 (0.000150%)

GTACAAGGTAGTGGGTCGCTGCCTGCCCACCCCCAAATGCCACACGCCGCCCCTCTACCGCATGCGAATC
TTTGCGCCTAATCATGTCGTCGCCAAGTCC 545 (0.016376%)

GTACTATGTGACTATCATTGATGCCCCAGGACACAGAGACTTTATCAAAAACATGATTACAGGGACATCT
CAGGCTGACTGTGCTGTCCTGATTGTTGCT 46 (0.001382%)

GTAGATTCGGGCAAGTCCACCACTACTGGCCATCTGATCTATAAATGCGGTGGCATCGACAAAAGAACC
ATTGAAAAATTTGAGAAGGAGGCTGCTGAGA 56 (0.001683%)

GTAGGCTCCCTTCCCCTACTCATCGCACTGATTTACACTCACAACACCCTAGGCTCACTAAACATTCTACT
ACTCACTCTCACTGCCCAAGAACTATCAA 7 (0.000210%)

GTATCACCATTGATATCTCCTTGTGGAAATTTGAGACCAGCAAGTACTATGTGACTATCATTGATGCCCC
AGGACACAGAGACTTTATCAAAAACATGAT 12 (0.000361%)

GTATGCCTGGGTCTTGGATAAACTGAAAGCTGAGCGTGAACGTGGTATCACCATTGATATCTCCTTGTGG
AAATTTGAGACCAGCAAGTACTATGTGACT 11 (0.000331%)

GTATGGTGGTCACCTTTGCTCCAGTCAACGTTACAACGGAAGTAAAATCTGTCGAAATGCACCATGAAG
CTTTGAGTGAAGCTCTTCCTGGGGACAATGT 6 (0.000180%)

GTCAAGAATGTGTCTGTCAAGGATGTTCGTCGTGGCAACGTTGCTGGTGACAGCAAAAATGACCCACCA
ATGGAAGCAGCTGGCTTCACTGCTCAGGTGA 15 (0.000451%)

GTCAAGACTACCGATGGTTACTTGCTTCGTCTGTTCTGTGTTGGTTTTACTAAAAAACGCAACAATCAGA
TACGGAAGACCTCTTATGCTCAGCACCAAC 10 (0.000300%)

GTCAAGGATGTTCGTCGTGGCAACGTTGCTGGTGACAGCAAAAATGACCCACCAATGGAAGCAGCTGGC
TTCACTGCTCAGGTGATTATCCTGAACCATC 20 (0.000601%)

GTCACCTTTGCTCCAGTCAACGTTACAACGGAAGTAAAATCTGTCGAAATGCACCATGAAGCTTTGAGTG
AAGCTCTTCCTGGGGACAATGTGGGCTTCA 77 (0.002314%)

GTCATCAAAGCAGTGGACAAGAAGGCTGCTGGAGCTGGCAAGGTCACCAAGTCTGCCCAGAAAGCTCA
GAAGGCTAAATGAATATTATCCCTAATACCTG 18 (0.000541%)

GTCATTGGACACGTAGATTCGGGCAAGTCCACCACTACTGGCCATCTGATCTATAAATGCGGTGGCATCG
ACAAAAGAACCATTGAAAAATTTGAGAAGG 6 (0.000180%)

GTCCAACTGACAAGCCCTTGCGCCTGCCTCTCCAGGATGTCTACAAAATTGGTGGTATTGGTACTGTTCC
TGTTGGCCGAGTGGAGACTGGTGTTCTCAA 512 (0.015384%)

GTCCACCACTACTGGCCATCTGATCTATAAATGCGGTGGCATCGACAAAAGAACCATTGAAAAATTTGA
GAAGGAGGCTGCTGAGATGGGAAAGGGCTCC 30 (0.000901%)

GTCCCTCGGCTTCCTGCCTCGGAAGCGCAGCAGCAGGCATCGTGGGAAGGTGAAGAGCTTCCCTAAGGA
TGACCCGTCCAAGCCGGTCCACCTCACAGCC 4 (0.000120%)

GTCCTAGCTGCTGGCATCACTATACTACTAACAGACCGCAACCTCAACACCACCTTCTTCGACCCCGCCG

GAGGAGGAGACCCCATTCTATACCAACACC 5 (0.000150%)

GTCCTCATCGCCCTCCCATCCCTACGCATCCTTTACATAACAGACGAGGTCAACGATCCCTCCCTTACCAT
CAAATCAATTGGCCACCAATGGTACTGAA 44 (0.001322%)

GTCCTGATTGTTGCTGCTGGTGTTGGTGAATTTGAAGCTGGTATCTCCAAGAATGGGCAGACCCGAGAGC
ATGCCCTTCTGGCTTACACACTGGGTGTGA 5 (0.000150%)

GTCGAGGCCATCTCCTGGGCCGCCTGGCGGCCATCGTGGCTAAACAGGTACTGCTGGGCCGGAAGGTGG
TGGTCGTACGCTGTGAAGGCATCAACATTTC 40 (0.001202%)

GTCGTCATTGGACACGTAGATTCGGGCAAGTCCACCACTACTGGCCATCTGATCTATAAATGCGGTGGCA
TCGACAAAAGAACCATTGAAAAATTTGAGA 7 (0.000210%)

GTCGTGAAAACTACCCCTAAAAGCCAAAATGGGAAAGGAAAAGACTCATATCAACATTGTCGTCATTGG
ACACGTAGATTCGGGCAAGTCCACCACTACT 23 (0.000691%)

GTCTGAACTATCCTGCCCGCCATCATCCTAGTCCTCATCGCCCTCCCATCCCTACGCATCCTTTACATAAC
AGACGAGGTCAACGATCCCTCCCTTACCA 84 (0.002524%)

GTCTGGTGATGCTGCCATTGTTGATATGGTTCCTGGCAAGCCCATGTGTGTTGAGAGCTTCTCAGACTAT
CCACCTTTGGGTCGCTTTGCTGTTCGTGAT 53 (0.001592%)

GTCTGTCAAGGATGTTCGTCGTGGCAACGTTGCTGGTGACAGCAAAAATGACCCACCAATGGAAGCAGC
TGGCTTCACTGCTCAGGTGATTATCCTGAAC 14 (0.000421%)

GTCTGTGTTCCGCGCGCACGTGAAGCACCGTAAAGGCGCTGCGCGCCTGCGCGCCGTGGATTTCGCTGA
GCGGCACGGCTACATCAAGGGCATCGTCAAG 24 (0.000721%)

GTCTGTTCTGTGTTGGTTTTACTAAAAAACGCAACAATCAGATACGGAAGACCTCTTATGCTCAGCACCA
ACAGGTCCGCCAAATCCGGAAGAAGATGAT 5 (0.000150%)

GTCTTGGATAAACTGAAAGCTGAGCGTGAACGTGGTATCACCATTGATATCTCCTTGTGGAAATTTGAGA
CCAGCAAGTACTATGTGACTATCATTGATG 11 (0.000331%)

GTGAAAACTACCCCTAAAAGCCAAAATGGGAAAGGAAAAGACTCATATCAACATTGTCGTCATTGGACA
CGTAGATTCGGGCAAGTCCACCACTACTGGCCATCTGATCTATAAATGCGGTGGCATCGACAAAAGAAC
CATTGAAAAAT 277 (0.012401%)

GTGAAACAACTAATTGTCGGTGTTAACAAAATGGATTCCACTGAGCCACCCTACAGCCAGAAGAGATAT
GAGGAAATTGTTAAGGAAGTCAGCACTTACA 4 (0.000120%)

GTGAACGTGGTATCACCATTGATATCTCCTTGTGGAAATTTGAGACCAGCAAGTACTATGTGACTATCAT
TGATGCCCCAGGACACAGAGACTTTATCAA 20 (0.000601%)

GTGACTATCATTGATGCCCCAGGACACAGAGACTTTATCAAAAACATGATTACAGGGACATCTCAGGCT
GACTGTGCTGTCCTGATTGTTGCTGCTGGTG 26 (0.000781%)

GTGATATGAGACAGACAGTTGCGGTGGGTGTCATCAAAGCAGTGGACAAGAAGGCTGCTGGAGCTGGC
AAGGTCACCAAGTCTGCCCAGAAAGCTCAGAA 28 (0.000841%)

GTGATGCTGCCATTGTTGATATGGTTCCTGGCAAGCCCATGTGTGTTGAGAGCTTCTCAGACTATCCACC
TTTGGGTCGCTTTGCTGTTCGTGATATGAG5 (0.000150%)

GTGCCTCGCTGAGGAAAAATAACTAAACATGGGCAAAGGAGATCCTAAGAAGCCGAGAGGCAAAATGT
CATCATATGCATTTTTTGTGCAAACTTGTCGG 4 (0.000120%)

GTGCCTGGTGCGGGAGCTACGGGGCCCAGGGATTGTGTTTAAAGTAGTGCTTCTACCAACATGTCCCGTG
GTTCCAGCGCCGGTTTTGACCGCCACATTA 42 (0.001262%)

GTGCTGTCCTGATTGTTGCTGCTGGTGTTGGTGAATTTGAAGCTGGTATCTCCAAGAATGGGCAGACCCG
AGAGCATGCCCTTCTGGCTTACACACTGGG 12 (0.000361%)

GTGGAAATTTGAGACCAGCAAGTACTATGTGACTATCATTGATGCCCCAGGACACAGAGACTTTATCAA
AAACATGATTACAGGGACATCTCAGGCTGAC 7 (0.000210%)

GTGGAAGCTACAATGATTTTGGGAATTACAACAATCAGTCTTCAAATTTTGGACCCATGAAGGGAGGAA
ATTTTGGAGGCAGAAGCTCTGGCCCCTATGG 5 (0.000150%)

GTGGACAAGAAGGCTGCTGGAGCTGGCAAGGTCACCAAGTCTGCCCAGAAAGCTCAGAAGGCTAAATG
AATATTATCCCTAATACCTGCCACCCCACTCT 30 (0.000901%)

GTGGACAGAGGAAGGGCGCCGGGTCTGTGTTCCGCGCGCACGTGAAGCACCGTAAAGGCGCTGCGCGCC
TGCGCGCCGTGGATTTCGCTGAGCGGCACGG 7 (0.000210%)

GTGGAGACTGGTGTTCTCAAACCCGGTATGGTGGTCACCTTTGCTCCAGTCAACGTTACAACGGAAGTAA
AATCTGTCGAAATGCACCATGAAGCTTTGA 18 (0.000541%)

GTGGCATCGACAAAAGAACCATTGAAAAATTTGAGAAGGAGGCTGCTGAGATGGGAAAGGGCTCCTTC
AAGTATGCCTGGGTCTTGGATAAACTGAAAGC12 (0.000361%)

GTGGGAGACAGCAGCACCAGCGGTGGCAGAGACCCCAGACATCAAGCTCTTTGGGAAGTGGAGCACCG
ATGATGTGCAGATCAATGACATTTCCCTGCAG 19 (0.000571%)

GTGGGCTTCAATGTCAAGAATGTGTCTGTCAAGGATGTTCGTCGTGGCAACGTTGCTGGTGACAGCAAA
AATGACCCACCAATGGAAGCAGCTGGCTTCA 844 (0.025360%)

GTGGGTGTCATCAAAGCAGTGGACAAGAAGGCTGCTGGAGCTGGCAAGGTCACCAAGTCTGCCCAGAA
AGCTCAGAAGGCTAAATGAATATTATCCCTAA28 (0.000841%)

GTGGTATCACCATTGATATCTCCTTGTGGAAATTTGAGACCAGCAAGTACTATGTGACTATCATTGATGC
CCCAGGACACAGAGACTTTATCAAAAACAT 24 (0.000721%)

GTGGTCACCTTTGCTCCAGTCAACGTTACAACGGAAGTAAAATCTGTCGAAATGCACCATGAAGCTTTGA
GTGAAGCTCTTCCTGGGGACAATGTGGGCT 53 (0.001592%)

GTGTCATCAAAGCAGTGGACAAGAAGGCTGCTGGAGCTGGCAAGGTCACCAAGTCTGCCCAGAAAGCTC
AGAAGGCTAAATGAATATTATCCCTAATACC 59 (0.001773%)

GTGTCGTGAAAACTACCCCTAAAAGCCAAAATGGGAAAGGAAAAGACTCATATCAACATTGTCGTCATT
GGACACGTAGATTCGGGCAAGTCCACCACTA 33 (0.000992%)

GTGTCTGTCAAGGATGTTCGTCGTGGCAACGTTGCTGGTGACAGCAAAAATGACCCACCAATGGAAGCA
GCTGGCTTCACTGCTCAGGTGATTATCCTGA 24 (0.000721%)

GTGTGAAACAACTAATTGTCGGTGTTAACAAAATGGATTCCACTGAGCCACCCTACAGCCAGAAGAGAT
ATGAGGAAATTGTTAAGGAAGTCAGCACTTA 16 (0.000481%)

GTGTGTTGAGAGCTTCTCAGACTATCCACCTTTGGGTCGCTTTGCTGTTCGTGATATGAGACAGACAGTT
GCGGTGGGTGTCATCAAAGCAGTGGACAAG 17 (0.000511%)

GTGTTCCGCGCGCACGTGAAGCACCGTAAAGGCGCTGCGCGCCTGCGCGCCGTGGATTTCGCTGAGCGG
CACGGCTACATCAAGGGCATCGTCAAGGACA 17 (0.000511%)

GTGTTCTCAAACCCGGTATGGTGGTCACCTTTGCTCCAGTCAACGTTACAACGGAAGTAAAATCTGTCGA
AATGCACCATGAAGCTTTGAGTGAAGCTCT 32 (0.000962%)

GTGTTGAGAGCTTCTCAGACTATCCACCTTTGGGTCGCTTTGCTGTTCGTGATATGAGACAGACAGTTGC

GGTGGGTGTCATCAAAGCAGTGGACAAGAA 3 (0.000090%)

GTTACTTGCTTCGTCTGTTCTGTGTTGGTTTTACTAAAAAACGCAACAATCAGATACGGAAGACCTCTTAT
GCTCAGCACCAACAGGTCCGCCAAATCCG3 (0.000090%)

GTTCCCCTAATAATCGGTGCCCCCGATATGGCGTTTCCCCGCATAAACAACATAAGCTTCTGACTCTTAC
CTCCCTCTCTCCTACTCCTGCTCGCATCTG 13 (0.000391%)

GTTCCGCGCGCACGTGAAGCACCGTAAAGGCGCTGCGCGCCTGCGCGCCGTGGATTTCGCTGAGCGGCA
CGGCTACATCAAGGGCATCGTCAAGGACATC 7 (0.000210%)

GTTCCTGGCAAGCCCATGTGTGTTGAGAGCTTCTCAGACTATCCACCTTTGGGTCGCTTTGCTGTTCGTGA
TATGAGACAGACAGTTGCGGTGGGTGTCA28 (0.000841%)

GTTCGCTTCATTCATTGCCCCCACAATCCTAGGCCTACCCGCCGCAGTACTGATCATTCTATTTCCCCCTC
TATTGATCCCCACCTCCAAATATCTCATC 39 (0.001172%)

GTTCTCAAACCCGGTATGGTGGTCACCTTTGCTCCAGTCAACGTTACAACGGAAGTAAAATCTGTCGAAA
TGCACCATGAAGCTTTGAGTGAAGCTCTTC 39 (0.001172%)

GTTCTCCGCTCCCAGACATGGGTCCCTCGGCTTCCTGCCTCGGAAGCGCAGCAGCAGGCATCGTGGGAA
GGTGAAGAGCTTCCCTAAGGATGACCCGTCC 6 (0.000180%)

GTTCTCTAGGCCTTTTAGAAAACATGGAGTTGTTCCTTTGGCCACATATATGCGAATCTATAAGAAAGGT
GATATTGTAGACATCAAGGGAATGGGTACT 20 (0.000601%)

GTTCTGGTAAAAAGCTGGAAGATGGCCCTAAATTCTTGAAGTCTGGTGATGCTGCCATTGTTGATATGGT
TCCTGGCAAGCCCATGTGTGTTGAGAGCTT 46 (0.001382%)

GTTCTGTGTTGGTTTTACTAAAAAACGCAACAATCAGATACGGAAGACCTCTTATGCTCAGCACCAACAG
GTCCGCCAAATCCGGAAGAAGATGATGGAA 6 (0.000180%)

GTTGAGAGCTTCTCAGACTATCCACCTTTGGGTCGCTTTGCTGTTCGTGATATGAGACAGACAGTTGCGG
TGGGTGTCATCAAAGCAGTGGACAAGAAGG 18 (0.000541%)

GTTGATATGGTTCCTGGCAAGCCCATGTGTGTTGAGAGCTTCTCAGACTATCCACCTTTGGGTCGCTTTGC
TGTTCGTGATATGAGACAGACAGTTGCGG 30 (0.000901%)

GTTGATGTCAAGACTACCGATGGTTACTTGCTTCGTCTGTTCTGTGTTGGTTTTACTAAAAAACGCAACA
ATCAGATACGGAAGACCTCTTATGCTCAGC 17 (0.000511%)

GTTGCGGTGGGTGTCATCAAAGCAGTGGACAAGAAGGCTGCTGGAGCTGGCAAGGTCACCAAGTCTGCC
CAGAAAGCTCAGAAGGCTAAATGAATATTAT 45 (0.001352%)

GTTGGCCGAGTGGAGACTGGTGTTCTCAAACCCGGTATGGTGGTCACCTTTGCTCCAGTCAACGTTACAA
CGGAAGTAAAATCTGTCGAAATGCACCATG 768 (0.023076%)

GTTGTAGGCCCCTACGGGCTACTACAACCCTTCGCTGACGCCATAAAACTCTTCACCAAAGAGCCCCTAA
AACCCGCCACATCTACCATCACCCTCTACA 9 (0.000270%)

GTTTCCCCGCATAAACAACATAAGCTTCTGACTCTTACCTCCCTCTCTCCTACTCCTGCTCGCATCTGCTA
TAGTGGAGGCCGGAGCAGGAACAGGTTGA 3 (0.000090%)

GTTTTTTTCTTCGCAGGATTTTTCTGAGCCTTTTACCACTCCAGCCTAGCCCCTACCCCCCAATTAGGAGG
GCACTGGCCCCCAACAGGCATCACCCCGC55 (0.001653%)

TAAAAAGCTGGAAGATGGCCCTAAATTCTTGAAGTCTGGTGATGCTGCCATTGTTGATATGGTTCCTGGC
AAGCCCATGTGTGTTGAGAGCTTCTCAGAC 23 (0.000691%)

TAAAAATAAAAAATTATAACAAACCCTGAGAACCAAAATGAACGAAAATCTGTTCGCTTCATTCATTGC
CCCCACAATCCTAGGCCTACCCGCCGCAGTA 46 (0.001382%)

TAAAACCCAGCCCATGACCCCTAACAGGGGCCCTCTCAGCCCTCCTAATGACCTCCGGCCTAGCCATGTG
ATTTCACTTCCACTCCATAACGCTCCTCAT 21 (0.000631%)

TAAACCTAGCCATGGCCATCCCCTTATGAGCGGGCGCAGTGATTATAGGCTTTCGCTCTAAGATTAAAAA
TGCCCTAGCCCACTTCTTACCACAAGGCAC 8 (0.000240%)

TAAACTGAAAGCTGAGCGTGAACGTGGTATCACCATTGATATCTCCTTGTGGAAATTTGAGACCAGCAA
GTACTATGTGACTATCATTGATGCCCCAGGA 12 (0.000361%)

TAAATGCGGTGGCATCGACAAAAGAACCATTGAAAAATTTGAGAAGGAGGCTGCTGAGATGGGAAAGG
GCTCCTTCAAGTATGCCTGGGTCTTGGATAAA 15 (0.000451%)

TAAATTCTTGAAGTCTGGTGATGCTGCCATTGTTGATATGGTTCCTGGCAAGCCCATGTGTGTTGAGAGC
TTCTCAGACTATCCACCTTTGGGTCGCTTT 8 (0.000240%)

TAACAGGGGCCCTCTCAGCCCTCCTAATGACCTCCGGCCTAGCCATGTGATTTCACTTCCACTCCATAAC
GCTCCTCATACTAGGCCTACTAACCAACAC 12 (0.000361%)

TAACTGAACTCCTCACACCCAATTGGACCAATCTATCACCCTATAGAAGAACTAATGTTAGTATAAGTAA
CATGAAAACATTCTCCTCCGCATAAGCCTG 4 (0.000120%)

TAATAATCGGTGCCCCCGATATGGCGTTTCCCCGCATAAACAACATAAGCTTCTGACTCTTACCTCCCTCT
CTCCTACTCCTGCTCGCATCTGCTATAGT 26 (0.000781%)

TAATCATTTTTATTGCCACAACTAACCTCCTCGGACTCCTGCCTCACTCATTTACACCAACCACCCAACTA
TCTATAAACCTAGCCATGGCCATCCCCTT 3 (0.000090%)

TAATCGGTGCCCCCGATATGGCGTTTCCCCGCATAAACAACATAAGCTTCTGACTCTTACCTCCCTCTCTC
CTACTCCTGCTCGCATCTGCTATAGTGGA 3 (0.000090%)

TAATCTCCCATATTGTAACTTACTACTCCGGAAAAAAAGAACCATTTGGATACATAGGTATGGTCTGAGC
TATGATATCAATTGGCTTCCTAGGGTTTAT8 (0.000240%)

TAATGACCTCCGGCCTAGCCATGTGATTTCACTTCCACTCCATAACGCTCCTCATACTAGGCCTACTAACC
AACACACTAACCATATACCAATGATGGCG6 (0.000180%)

TAATGATGGAAGCAATTTTGGAGGTGGTGGAAGCTACAATGATTTTGGGAATTACAACAATCAGTCTTC
AAATTTTGGACCCATGAAGGGAGGAAATTTT 12 (0.000361%)

TACAAGGTAGTGGGTCGCTGCCTGCCCACCCCCAAATGCCACACGCCGCCCCTCTACCGCATGCGAATCT
TTGCGCCTAATCATGTCGTCGCCAAGTCCC 5 (0.000150%)

TACAATGATTTTGGGAATTACAACAATCAGTCTTCAAATTTTGGACCCATGAAGGGAGGAAATTTTGGAG
GCAGAAGCTCTGGCCCCTATGGCGGTGGAG 3 (0.000090%)

TACATACTTCCCCCATTATTCCTAGAACCAGGCGACCTGCGACTCCTTGACGTTGACAATCGAGTAGTAC
TCCCGATTGAAGCCCCCATTCGTATAATAA 6 (0.000180%)

TACCAGGCTTCGGAATAATCTCCCATATTGTAACTTACTACTCCGGAAAAAAAGAACCATTTGGATACAT
AGGTATGGTCTGAGCTATGATATCAATTGG 6 (0.000180%)

TACCTCAGAAGTTTTTTTCTTCGCAGGATTTTTCTGAGCCTTTTACCACTCCAGCCTAGCCCCTACCCCCC
AATTAGGAGGGCACTGGCCCCCAACAGGC 3 (0.000090%)

TACGCATCCTTTACATAACAGACGAGGTCAACGATCCCTCCCTTACCATCAAATCAATTGGCCACCAATG
GTACTGAACCTACGAGTACACCGACTACGG 7 (0.000210%)

TACTATGTGACTATCATTGATGCCCCAGGACACAGAGACTTTATCAAAAACATGATTACAGGGACATCTC
AGGCTGACTGTGCTGTCCTGATTGTTGCTG 4 (0.000120%)

TACTTCTCCTATCTCTCCCAGTCCTAGCTGCTGGCATCACTATACTACTAACAGACCGCAACCTCAACACC
ACCTTCTTCGACCCCGCCGGAGGAGGAGA 684 (0.020552%)

TAGAAACCGTCTGAACTATCCTGCCCGCCATCATCCTAGTCCTCATCGCCCTCCCATCCCTACGCATCCTT
TACATAACAGACGAGGTCAACGATCCCTC 34 (0.001022%)

TAGAACCAGGCGACCTGCGACTCCTTGACGTTGACAATCGAGTAGTACTCCCGATTGAAGCCCCCATTCG
TATAATAATTACATCACAAGACGTCTTGCA 7 (0.000210%)

TAGGAATCACCTCCCATTCCGATAAAATCACCTTCCACCCTTACTACACAATCAAAGACGCCCTCGGCTT
ACTTCTCTTCATTCTCTCCTTAATGACATT 12 (0.000361%)

TAGGCACAGCTCTAAGCCTCCTTATTCGAGCCGAGCTGGGCCAGCCAGGCAACCTTCTAGGTAACGACC
ACATCTACAACGTTATCGTCACAGCCCATGC 720 (0.021634%)

TAGTAGGCTCCCTTCCCCTACTCATCGCACTGATTTACACTCACAACACCCTAGGCTCACTAAACATTCTA
CTACTCACTCTCACTGCCCAAGAACTATC 7 (0.000210%)

TAGTCCTCATCGCCCTCCCATCCCTACGCATCCTTTACATAACAGACGAGGTCAACGATCCCTCCCTTACC
ATCAAATCAATTGGCCACCAATGGTACTG 8 (0.000240%)

TATAAACCTAGCCATGGCCATCCCCTTATGAGCGGGCGCAGTGATTATAGGCTTTCGCTCTAAGATTAAA
AATGCCCTAGCCCACTTCTTACCACAAGGC 78 (0.002344%)

TATAAATGCGGTGGCATCGACAAAAGAACCATTGAAAAATTTGAGAAGGAGGCTGCTGAGATGGGAAA
GGGCTCCTTCAAGTATGCCTGGGTCTTGGATA 17 (0.000511%)

TATAACTGAACTCCTCACACCCAATTGGACCAATCTATCACCCTATAGAAGAACTAATGTTAGTATAAGT
AACATGAAAACATTCTCCTCCGCATAAGCC 9 (0.000270%)

TATATGTTCTCTAGGCCTTTTAGAAAACATGGAGTTGTTCCTTTGGCCACATATATGCGAATCTATAAGA
AAGGTGATATTGTAGACATCAAGGGAATGG 13 (0.000391%)

TATCAAAAACATGATTACAGGGACATCTCAGGCTGACTGTGCTGTCCTGATTGTTGCTGCTGGTGTTGGT
GAATTTGAAGCTGGTATCTCCAAGAATGGG 7 (0.000210%)

TATCACCCTATAGAAGAACTAATGTTAGTATAAGTAACATGAAAACATTCTCCTCCGCATAAGCCTGCGT
CAGATTAAAACACTGAACTGACAATTAACA 22 (0.000661%)

TATCATTGATGCCCCAGGACACAGAGACTTTATCAAAAACATGATTACAGGGACATCTCAGGCTGACTG
TGCTGTCCTGATTGTTGCTGCTGGTGTTGGT 12 (0.000361%)

TATCCACCTTTGGGTCGCTTTGCTGTTCGTGATATGAGACAGACAGTTGCGGTGGGTGTCATCAAAGCAG
TGGACAAGAAGGCTGCTGGAGCTGGCAAGG 5 (0.000150%)

TATCCTGCCCGCCATCATCCTAGTCCTCATCGCCCTCCCATCCCTACGCATCCTTTACATAACAGACGAGG
TCAACGATCCCTCCCTTACCATCAAATCA 4 (0.000120%)

TATCTATAAACCTAGCCATGGCCATCCCCTTATGAGCGGGCGCAGTGATTATAGGCTTTCGCTCTAAGAT
TAAAAATGCCCTAGCCCACTTCTTACCACA 41 (0.001232%)

TATCTCCTTGTGGAAATTTGAGACCAGCAAGTACTATGTGACTATCATTGATGCCCCAGGACACAGAGAC
TTTATCAAAAACATGATTACAGGGACATCT 12 (0.000361%)

TATGAGACAGACAGTTGCGGTGGGTGTCATCAAAGCAGTGGACAAGAAGGCTGCTGGAGCTGGCAAGG

TCACCAAGTCTGCCCAGAAAGCTCAGAAGGCT 9 (0.000270%)

TATGCCTGGGTCTTGGATAAACTGAAAGCTGAGCGTGAACGTGGTATCACCATTGATATCTCCTTGTGGA
AATTTGAGACCAGCAAGTACTATGTGACTA 3 (0.000090%)

TATGGCGTTTCCCCGCATAAACAACATAAGCTTCTGACTCTTACCTCCCTCTCTCCTACTCCTGCTCGCAT
CTGCTATAGTGGAGGCCGGAGCAGGAACA 6 (0.000180%)

TATGGTGGTCACCTTTGCTCCAGTCAACGTTACAACGGAAGTAAAATCTGTCGAAATGCACCATGAAGCT
TTGAGTGAAGCTCTTCCTGGGGACAATGTG 5 (0.000150%)

TATGGTTCCTGGCAAGCCCATGTGTGTTGAGAGCTTCTCAGACTATCCACCTTTGGGTCGCTTTGCTGTTC
GTGATATGAGACAGACAGTTGCGGTGGGT47 (0.001412%)

TATGTGACTATCATTGATGCCCCAGGACACAGAGACTTTATCAAAAACATGATTACAGGGACATCTCAG
GCTGACTGTGCTGTCCTGATTGTTGCTGCTG 9 (0.000270%)

TATGTTCTCTAGGCCTTTTAGAAAACATGGAGTTGTTCCTTTGGCCACATATATGCGAATCTATAAGAAA
GGTGATATTGTAGACATCAAGGGAATGGGT 4 (0.000120%)

TATTACCTCAGAAGTTTTTTTCTTCGCAGGATTTTTCTGAGCCTTTTACCACTCCAGCCTAGCCCCTACCC
CCCAATTAGGAGGGCACTGGCCCCCAACA 16 (0.000481%)

TATTCCTAGAACCAGGCGACCTGCGACTCCTTGACGTTGACAATCGAGTAGTACTCCCGATTGAAGCCCC
CATTCGTATAATAATTACATCACAAGACGT 48 (0.001442%)

TATTCTACACCCTAGTAGGCTCCCTTCCCCTACTCATCGCACTGATTTACACTCACAACACCCTAGGCTCA
CTAAACATTCTACTACTCACTCTCACTGC 3 (0.000090%)

TATTGCCACAACTAACCTCCTCGGACTCCTGCCTCACTCATTTACACCAACCACCCAACTATCTATAAAC
CTAGCCATGGCCATCCCCTTATGAGCGGGC 6 (0.000180%)

TATTTATTACCTCAGAAGTTTTTTTCTTCGCAGGATTTTTCTGAGCCTTTTACCACTCCAGCCTAGCCCCTA
CCCCCCAATTAGGAGGGCACTGGCCCCC 10 (0.000300%)

TCAAACCCGGTATGGTGGTCACCTTTGCTCCAGTCAACGTTACAACGGAAGTAAAATCTGTCGAAATGCA
CCATGAAGCTTTGAGTGAAGCTCTTCCTGG 7 (0.000210%)

TCAAAGCAGTGGACAAGAAGGCTGCTGGAGCTGGCAAGGTCACCAAGTCTGCCCAGAAAGCTCAGAAG
GCTAAATGAATATTATCCCTAATACCTGCCAC 10 (0.000300%)

TCAACTTCACCCGTAACCCACCGCCATGGCCGAGGAAGGCATTGCTGCTGGAGGTGTAATGGACGTTAA
TACTGCTTTACAAGAGGTTCTGAAGACTGCC 7 (0.000210%)

TCAAGAATGTGTCTGTCAAGGATGTTCGTCGTGGCAACGTTGCTGGTGACAGCAAAAATGACCCACCAA
TGGAAGCAGCTGGCTTCACTGCTCAGGTGAT 7 (0.000210%)

TCAAGACTACCGATGGTTACTTGCTTCGTCTGTTCTGTGTTGGTTTTACTAAAAAACGCAACAATCAGAT
ACGGAAGACCTCTTATGCTCAGCACCAACA 5 (0.000150%)

TCAAGCACTGCTTATTACAATTTTACTGGGTCTCTATTTTACCCTCCTACAAGCCTCAGAGTACTTCGAGT
CTCCCTTCACCATTTCCGACGGCATCTAC 5 (0.000150%)

TCAAGTATGCCTGGGTCTTGGATAAACTGAAAGCTGAGCGTGAACGTGGTATCACCATTGATATCTCCTT
GTGGAAATTTGAGACCAGCAAGTACTATGT 3 (0.000090%)

TCAATGTCAAGAATGTGTCTGTCAAGGATGTTCGTCGTGGCAACGTTGCTGGTGACAGCAAAAATGACC
CACCAATGGAAGCAGCTGGCTTCACTGCTCA 14 (0.000421%)

TCACACCCAATTGGACCAATCTATCACCCTATAGAAGAACTAATGTTAGTATAAGTAACATGAAAACATT
CTCCTCCGCATAAGCCTGCGTCAGATTAAA 5 (0.000150%)

TCACCAAAGCCCATAAAAATAAAAAATTATAACAAACCCTGAGAACCAAAATGAACGAAAATCTGTTCG
CTTCATTCATTGCCCCCACAATCCTAGGCCT 3 (0.000090%)

TCACCATTGATATCTCCTTGTGGAAATTTGAGACCAGCAAGTACTATGTGACTATCATTGATGCCCCAGG
ACACAGAGACTTTATCAAAAACATGATTAC 7 (0.000210%)

TCACCCTCCACTTCCCGTCTCAGAATCTAAACGTGGTCACCTTCGAGTAGAGAGGCCCGCCCGCCCACCG
TGGGCAGTGCCACCCGCAGATGACACGCGC 16 (0.000481%)

TCACCTTCCACCCTTACTACACAATCAAAGACGCCCTCGGCTTACTTCTCTTCATTCTCTCCTTAATGACA
TTAACACTATTCTCACCAGACCTCCTAGG 8 (0.000240%)

TCACCTTTGCTCCAGTCAACGTTACAACGGAAGTAAAATCTGTCGAAATGCACCATGAAGCTTTGAGTGA
AGCTCTTCCTGGGGACAATGTGGGCTTCAA 6 (0.000180%)

TCAGAAGTTTTTTTCTTCGCAGGATTTTTCTGAGCCTTTTACCACTCCAGCCTAGCCCCTACCCCCCAATT
AGGAGGGCACTGGCCCCCAACAGGCATCA 3 (0.000090%)

TCAGACTATCCACCTTTGGGTCGCTTTGCTGTTCGTGATATGAGACAGACAGTTGCGGTGGGTGTCATCA
AAGCAGTGGACAAGAAGGCTGCTGGAGCTG 8 (0.000240%)

TCAGCCCTCCTAATGACCTCCGGCCTAGCCATGTGATTTCACTTCCACTCCATAACGCTCCTCATACTAGG
CCTACTAACCAACACACTAACCATATACC 3 (0.000090%)

TCAGGCTGACTGTGCTGTCCTGATTGTTGCTGCTGGTGTTGGTGAATTTGAAGCTGGTATCTCCAAGAAT
GGGCAGACCCGAGAGCATGCCCTTCTGGCT 3 (0.000090%)

TCATCCTAGTCCTCATCGCCCTCCCATCCCTACGCATCCTTTACATAACAGACGAGGTCAACGATCCCTCC
CTTACCATCAAATCAATTGGCCACCAATG 7 (0.000210%)

TCATCGCCCTCCCATCCCTACGCATCCTTTACATAACAGACGAGGTCAACGATCCCTCCCTTACCATCAA
ATCAATTGGCCACCAATGGTACTGAACCTA 3 (0.000090%)

TCCACCACTACTGGCCATCTGATCTATAAATGCGGTGGCATCGACAAAAGAACCATTGAAAAATTTGAG
AAGGAGGCTGCTGAGATGGGAAAGGGCTCCT 5 (0.000150%)

TCCACCTTTGGGTCGCTTTGCTGTTCGTGATATGAGACAGACAGTTGCGGTGGGTGTCATCAAAGCAGTG
GACAAGAAGGCTGCTGGAGCTGGCAAGGTC 4 (0.000120%)

TCCCAGTCCTAGCTGCTGGCATCACTATACTACTAACAGACCGCAACCTCAACACCACCTTCTTCGACCC
CGCCGGAGGAGGAGACCCCATTCTATACCA 8 (0.000240%)

TCCCATCCCTACGCATCCTTTACATAACAGACGAGGTCAACGATCCCTCCCTTACCATCAAATCAATTGG
CCACCAATGGTACTGAACCTACGAGTACAC 4 (0.000120%)

TCCCTACGCATCCTTTACATAACAGACGAGGTCAACGATCCCTCCCTTACCATCAAATCAATTGGCCACC
AATGGTACTGAACCTACGAGTACACCGACT 3 (0.000090%)

TCCCTCACCAAAGCCCATAAAAATAAAAAATTATAACAAACCCTGAGAACCAAAATGAACGAAAATCTG
TTCGCTTCATTCATTGCCCCCACAATCCTAG 806 (0.024218%)

TCCGCTCCCAGACATGGGTCCCTCGGCTTCCTGCCTCGGAAGCGCAGCAGCAGGCATCGTGGGAAGGTG
AAGAGCTTCCCTAAGGATGACCCGTCCAAGC 4 (0.000120%)

TCCTAATGACCTCCGGCCTAGCCATGTGATTTCACTTCCACTCCATAACGCTCCTCATACTAGGCCTACTA
ACCAACACACTAACCATATACCAATGATG 7 (0.000210%)

TCCTACCAGGCTTCGGAATAATCTCCCATATTGTAACTTACTACTCCGGAAAAAAAGAACCATTTGGATA
CATAGGTATGGTCTGAGCTATGATATCAAT 3 (0.000090%)

TCCTAGTCCTCATCGCCCTCCCATCCCTACGCATCCTTTACATAACAGACGAGGTCAACGATCCCTCCCTT
ACCATCAAATCAATTGGCCACCAATGGTA 13 (0.000391%)

TCCTATCTCTCCCAGTCCTAGCTGCTGGCATCACTATACTACTAACAGACCGCAACCTCAACACCACCTT
CTTCGACCCCGCCGGAGGAGGAGACCCCAT 4 (0.000120%)

TCCTATTCTACACCCTAGTAGGCTCCCTTCCCCTACTCATCGCACTGATTTACACTCACAACACCCTAGGC
TCACTAAACATTCTACTACTCACTCTCAC 7 (0.000210%)

TCCTCACACCCAATTGGACCAATCTATCACCCTATAGAAGAACTAATGTTAGTATAAGTAACATGAAAAC
ATTCTCCTCCGCATAAGCCTGCGTCAGATT 4 (0.000120%)

TCCTCATCGCCCTCCCATCCCTACGCATCCTTTACATAACAGACGAGGTCAACGATCCCTCCCTTACCATC
AAATCAATTGGCCACCAATGGTACTGAAC 8 (0.000240%)

TCCTCGGACTCCTGCCTCACTCATTTACACCAACCACCCAACTATCTATAAACCTAGCCATGGCCATCCC
CTTATGAGCGGGCGCAGTGATTATAGGCTT 75 (0.002254%)

TCCTGATTGTTGCTGCTGGTGTTGGTGAATTTGAAGCTGGTATCTCCAAGAATGGGCAGACCCGAGAGCA
TGCCCTTCTGGCTTACACACTGGGTGTGAA 13 (0.000391%)

TCCTGCCTCACTCATTTACACCAACCACCCAACTATCTATAAACCTAGCCATGGCCATCCCCTTATGAGC
GGGCGCAGTGATTATAGGCTTTCGCTCTAA 7 (0.000210%)

TCCTGGCAAGCCCATGTGTGTTGAGAGCTTCTCAGACTATCCACCTTTGGGTCGCTTTGCTGTTCGTGATA
TGAGACAGACAGTTGCGGTGGGTGTCATC 11 (0.000331%)

TCCTGGGCCGCCTGGCGGCCATCGTGGCTAAACAGGTACTGCTGGGCCGGAAGGTGGTGGTCGTACGCT
GTGAAGGCATCAACATTTCTGGCAATTTCTA 5 (0.000150%)

TCCTTCAAGTATGCCTGGGTCTTGGATAAACTGAAAGCTGAGCGTGAACGTGGTATCACCATTGATATCT
CCTTGTGGAAATTTGAGACCAGCAAGTACT 4 (0.000120%)

TCCTTGTGGAAATTTGAGACCAGCAAGTACTATGTGACTATCATTGATGCCCCAGGACACAGAGACTTTA
TCAAAAACATGATTACAGGGACATCTCAGG 5 (0.000150%)

TCGAGGCCATCTCCTGGGCCGCCTGGCGGCCATCGTGGCTAAACAGGTACTGCTGGGCCGGAAGGTGGT
GGTCGTACGCTGTGAAGGCATCAACATTTCT 4 (0.000120%)

TCGCTTCATTCATTGCCCCCACAATCCTAGGCCTACCCGCCGCAGTACTGATCATTCTATTTCCCCCTCTA
TTGATCCCCACCTCCAAATATCTCATCAA 5 (0.000150%)

TCGGAATAATCTCCCATATTGTAACTTACTACTCCGGAAAAAAAGAACCATTTGGATACATAGGTATGGT
CTGAGCTATGATATCAATTGGCTTCCTAGG 4 (0.000120%)

TCGGACTCCTGCCTCACTCATTTACACCAACCACCCAACTATCTATAAACCTAGCCATGGCCATCCCCTT
ATGAGCGGGCGCAGTGATTATAGGCTTTCG 24 (0.000721%)

TCGGCTTCCTGCCTCGGAAGCGCAGCAGCAGGCATCGTGGGAAGGTGAAGAGCTTCCCTAAGGATGACC
CGTCCAAGCCGGTCCACCTCACAGCCTTCCT 5 (0.000150%)

TCGGGCAAGTCCACCACTACTGGCCATCTGATCTATAAATGCGGTGGCATCGACAAAAGAACCATTGAA
AAATTTGAGAAGGAGGCTGCTGAGATGGGAA 5 (0.000150%)

TCGGTGCCCCCGATATGGCGTTTCCCCGCATAAACAACATAAGCTTCTGACTCTTACCTCCCTCTCTCCTA

CTCCTGCTCGCATCTGCTATAGTGGAGGC 4 (0.000120%)

TCTAAGCCTCCTTATTCGAGCCGAGCTGGGCCAGCCAGGCAACCTTCTAGGTAACGACCACATCTACAAC
GTTATCGTCACAGCCCATGCATTTGTAATA 11 (0.000331%)

TCTACACCCTAGTAGGCTCCCTTCCCCTACTCATCGCACTGATTTACACTCACAACACCCTAGGCTCACTA
AACATTCTACTACTCACTCTCACTGCCCA 4 (0.000120%)

TCTAGGCCTTTTAGAAAACATGGAGTTGTTCCTTTGGCCACATATATGCGAATCTATAAGAAAGGTGATA
TTGTAGACATCAAGGGAATGGGTACTGTTC 5 (0.000150%)

TCTATAAATGCGGTGGCATCGACAAAAGAACCATTGAAAAATTTGAGAAGGAGGCTGCTGAGATGGGA
AAGGGCTCCTTCAAGTATGCCTGGGTCTTGGA 26 (0.000781%)

TCTCAAACCCGGTATGGTGGTCACCTTTGCTCCAGTCAACGTTACAACGGAAGTAAAATCTGTCGAAATG
CACCATGAAGCTTTGAGTGAAGCTCTTCCT 49 (0.001472%)

TCTCAGACTATCCACCTTTGGGTCGCTTTGCTGTTCGTGATATGAGACAGACAGTTGCGGTGGGTGTCAT
CAAAGCAGTGGACAAGAAGGCTGCTGGAGC 28 (0.000841%)

TCTCAGCCCTCCTAATGACCTCCGGCCTAGCCATGTGATTTCACTTCCACTCCATAACGCTCCTCATACTA
GGCCTACTAACCAACACACTAACCATATA 16 (0.000481%)

TCTCAGGCTGACTGTGCTGTCCTGATTGTTGCTGCTGGTGTTGGTGAATTTGAAGCTGGTATCTCCAAGA
ATGGGCAGACCCGAGAGCATGCCCTTCTGG 3 (0.000090%)

TCTCGCAAAGGATCTCCTTCATCCCTCTCCAGAAGAGGAGAAGAGGAAACACAAGAAGAAACGCCTGGT
GCAGAGCCCCAATTCCTACTTCATGGATGTG 4 (0.000120%)

TCTGAACTATCCTGCCCGCCATCATCCTAGTCCTCATCGCCCTCCCATCCCTACGCATCCTTTACATAACA
GACGAGGTCAACGATCCCTCCCTTACCAT 13 (0.000391%)

TCTGGCTTACACACTGGGTGTGAAACAACTAATTGTCGGTGTTAACAAAATGGATTCCACTGAGCCACCC
TACAGCCAGAAGAGATATGAGGAAATTGTT 6 (0.000180%)

TCTGGTAAAAAGCTGGAAGATGGCCCTAAATTCTTGAAGTCTGGTGATGCTGCCATTGTTGATATGGTTC
CTGGCAAGCCCATGTGTGTTGAGAGCTTCT 14 (0.000421%)

TCTGGTGATGCTGCCATTGTTGATATGGTTCCTGGCAAGCCCATGTGTGTTGAGAGCTTCTCAGACTATCC
ACCTTTGGGTCGCTTTGCTGTTCGTGATA 3 (0.000090%)

TCTGTCAAGGATGTTCGTCGTGGCAACGTTGCTGGTGACAGCAAAAATGACCCACCAATGGAAGCAGCT
GGCTTCACTGCTCAGGTGATTATCCTGAACC 3 (0.000090%)

TCTGTGCCTCGCTGAGGAAAAATAACTAAACATGGGCAAAGGAGATCCTAAGAAGCCGAGAGGCAAAA
TGTCATCATATGCATTTTTTGTGCAAACTTGT 7 (0.000210%)

TCTGTGTTCCGCGCGCACGTGAAGCACCGTAAAGGCGCTGCGCGCCTGCGCGCCGTGGATTTCGCTGAGC
GGCACGGCTACATCAAGGGCATCGTCAAGG 3 (0.000090%)

TCTGTGTTGGTTTTACTAAAAAACGCAACAATCAGATACGGAAGACCTCTTATGCTCAGCACCAACAGGT
CCGCCAAATCCGGAAGAAGATGATGGAAAT 11 (0.000331%)

TCTTGAAGTCTGGTGATGCTGCCATTGTTGATATGGTTCCTGGCAAGCCCATGTGTGTTGAGAGCTTCTCA
GACTATCCACCTTTGGGTCGCTTTGCTGT 34 (0.001022%)

TGAAAACTACCCCTAAAAGCCAAAATGGGAAAGGAAAAGACTCATATCAACATTGTCGTCATTGGACAC
GTAGATTCGGGCAAGTCCACCACTACTGGCCATCTGATCTATAAATGCGGTGGCATCGACAAAAGAACC
ATTGAAAAATT 55 (0.002462%)

TGAAAGCTGAGCGTGAACGTGGTATCACCATTGATATCTCCTTGTGGAAATTTGAGACCAGCAAGTACTA
TGTGACTATCATTGATGCCCCAGGACACAG 60 (0.001803%)

TGAACGCAGGCACATACTTCCTATTCTACACCCTAGTAGGCTCCCTTCCCCTACTCATCGCACTGATTTAC
ACTCACAACACCCTAGGCTCACTAAACAT 10 (0.000300%)

TGAACGTGGTATCACCATTGATATCTCCTTGTGGAAATTTGAGACCAGCAAGTACTATGTGACTATCATT
GATGCCCCAGGACACAGAGACTTTATCAAA 11 (0.000331%)

TGAACTATCCTGCCCGCCATCATCCTAGTCCTCATCGCCCTCCCATCCCTACGCATCCTTTACATAACAGA
CGAGGTCAACGATCCCTCCCTTACCATCA 7 (0.000210%)

TGAACTCCTCACACCCAATTGGACCAATCTATCACCCTATAGAAGAACTAATGTTAGTATAAGTAACATG
AAAACATTCTCCTCCGCATAAGCCTGCGTC 18 (0.000541%)

TGAAGTCTGGTGATGCTGCCATTGTTGATATGGTTCCTGGCAAGCCCATGTGTGTTGAGAGCTTCTCAGA
CTATCCACCTTTGGGTCGCTTTGCTGTTCG 17 (0.000511%)

TGACATCTGACGACGTGAAGGAGCAGATTTACAAACTGGCCAAGAAGGGCCTTACTCCTTCACAGATCG
GTGTAATCCTGAGAGATTCACATGGTGTTGC 163 (0.004898%)

TGACCCCTAACAGGGGCCCTCTCAGCCCTCCTAATGACCTCCGGCCTAGCCATGTGATTTCACTTCCACT
CCATAACGCTCCTCATACTAGGCCTACTAA 10 (0.000300%)

TGACCTCCGGCCTAGCCATGTGATTTCACTTCCACTCCATAACGCTCCTCATACTAGGCCTACTAACCAA
CACACTAACCATATACCAATGATGGCGCGA 14 (0.000421%)

TGACTAGTTCCCCTAATAATCGGTGCCCCCGATATGGCGTTTCCCCGCATAAACAACATAAGCTTCTGAC
TCTTACCTCCCTCTCTCCTACTCCTGCTCG 7 (0.000210%)

TGACTATCATTGATGCCCCAGGACACAGAGACTTTATCAAAAACATGATTACAGGGACATCTCAGGCTG
ACTGTGCTGTCCTGATTGTTGCTGCTGGTGT 9 (0.000270%)

TGAGAAGGAGGCTGCTGAGATGGGAAAGGGCTCCTTCAAGTATGCCTGGGTCTTGGATAAACTGAAAGC
TGAGCGTGAACGTGGTATCACCATTGATATC 14 (0.000421%)

TGAGACCAGCAAGTACTATGTGACTATCATTGATGCCCCAGGACACAGAGACTTTATCAAAAACATGAT
TACAGGGACATCTCAGGCTGACTGTGCTGTC 5 (0.000150%)

TGAGAGCTTCTCAGACTATCCACCTTTGGGTCGCTTTGCTGTTCGTGATATGAGACAGACAGTTGCGGTG
GGTGTCATCAAAGCAGTGGACAAGAAGGCT 8 (0.000240%)

TGAGCGTGAACGTGGTATCACCATTGATATCTCCTTGTGGAAATTTGAGACCAGCAAGTACTATGTGACT
ATCATTGATGCCCCAGGACACAGAGACTTT 23 (0.000691%)

TGAGCTCACCATAGTCTAATAGAAAACAACCGAAACCAAATAATTCAAGCACTGCTTATTACAATTTTAC
TGGGTCTCTATTTTACCCTCCTACAAGCCT 3 (0.000090%)

TGATATCTCCTTGTGGAAATTTGAGACCAGCAAGTACTATGTGACTATCATTGATGCCCCAGGACACAGA
GACTTTATCAAAAACATGATTACAGGGACA 8 (0.000240%)

TGATATGGTTCCTGGCAAGCCCATGTGTGTTGAGAGCTTCTCAGACTATCCACCTTTGGGTCGCTTTGCTG
TTCGTGATATGAGACAGACAGTTGCGGTG 16 (0.000481%)

TGATGCCCCAGGACACAGAGACTTTATCAAAAACATGATTACAGGGACATCTCAGGCTGACTGTGCTGT
CCTGATTGTTGCTGCTGGTGTTGGTGAATTT 27 (0.000811%)

TGATGCTGCCATTGTTGATATGGTTCCTGGCAAGCCCATGTGTGTTGAGAGCTTCTCAGACTATCCACCTT

TGGGTCGCTTTGCTGTTCGTGATATGAGA 17 (0.000511%)

TGATGGAAGCAATTTTGGAGGTGGTGGAAGCTACAATGATTTTGGGAATTACAACAATCAGTCTTCAAA
TTTTGGACCCATGAAGGGAGGAAATTTTGGA 3 (0.000090%)

TGATGGTCGAGGCCATCTCCTGGGCCGCCTGGCGGCCATCGTGGCTAAACAGGTACTGCTGGGCCGGAA
GGTGGTGGTCGTACGCTGTGAAGGCATCAAC 40 (0.001202%)

TGATTACAGGGACATCTCAGGCTGACTGTGCTGTCCTGATTGTTGCTGCTGGTGTTGGTGAATTTGAAGC
TGGTATCTCCAAGAATGGGCAGACCCGAGA 13 (0.000391%)

TGATTTTGGGAATTACAACAATCAGTCTTCAAATTTTGGACCCATGAAGGGAGGAAATTTTGGAGGCAG
AAGCTCTGGCCCCTATGGCGGTGGAGGCCAA 10 (0.000300%)

TGCCACACGGCTCACATTGCATGCAAGTTTGCTGAGCTGAAGGAAAAGATTGATCGCCGTTCTGGTAAA
AAGCTGGAAGATGGCCCTAAATTCTTGAAGT 22 (0.000661%)

TGCCATTGTTGATATGGTTCCTGGCAAGCCCATGTGTGTTGAGAGCTTCTCAGACTATCCACCTTTGGGTC
GCTTTGCTGTTCGTGATATGAGACAGACA 30 (0.000901%)

TGCCCGCCATCATCCTAGTCCTCATCGCCCTCCCATCCCTACGCATCCTTTACATAACAGACGAGGTCAA
CGATCCCTCCCTTACCATCAAATCAATTGG 4 (0.000120%)

TGCCCTTCTGGCTTACACACTGGGTGTGAAACAACTAATTGTCGGTGTTAACAAAATGGATTCCACTGAG
CCACCCTACAGCCAGAAGAGATATGAGGAA 13 (0.000391%)

TGCCTCGCTGAGGAAAAATAACTAAACATGGGCAAAGGAGATCCTAAGAAGCCGAGAGGCAAAATGTC
ATCATATGCATTTTTTGTGCAAACTTGTCGGG 5 (0.000150%)

TGCCTGGGTCTTGGATAAACTGAAAGCTGAGCGTGAACGTGGTATCACCATTGATATCTCCTTGTGGAAA
TTTGAGACCAGCAAGTACTATGTGACTATC 3 (0.000090%)

TGCGACTTGTGTTGGGACTGCTGATAGGAAGATGTCTTCAGGAAATGCTAAAATTGGGCACCCTGCCCCC
AACTTCAAAGCCACAGCTGTTATGCCAGATGGTCAGTTTAAAGATATCAGCCTGTCTGACTACAAAGGA
AAATATGTTG 96 (0.004298%)

TGCGGTGGCATCGACAAAAGAACCATTGAAAAATTTGAGAAGGAGGCTGCTGAGATGGGAAAGGGCTC
CTTCAAGTATGCCTGGGTCTTGGATAAACTGA 14 (0.000421%)

TGCGGTGGGTGTCATCAAAGCAGTGGACAAGAAGGCTGCTGGAGCTGGCAAGGTCACCAAGTCTGCCCA
GAAAGCTCAGAAGGCTAAATGAATATTATCC 14 (0.000421%)

TGCTGCCATTGTTGATATGGTTCCTGGCAAGCCCATGTGTGTTGAGAGCTTCTCAGACTATCCACCTTTGG
GTCGCTTTGCTGTTCGTGATATGAGACAG 28 (0.000841%)

TGCTGGAGCTGGCAAGGTCACCAAGTCTGCCCAGAAAGCTCAGAAGGCTAAATGAATATTATCCCTAAT
ACCTGCCACCCCACTCTTAATCAGTGGTGGA 14 (0.000421%)

TGCTGTCCTGATTGTTGCTGCTGGTGTTGGTGAATTTGAAGCTGGTATCTCCAAGAATGGGCAGACCCGA
GAGCATGCCCTTCTGGCTTACACACTGGGT 4 (0.000120%)

TGCTTCGTCTGTTCTGTGTTGGTTTTACTAAAAAACGCAACAATCAGATACGGAAGACCTCTTATGCTCA
GCACCAACAGGTCCGCCAAATCCGGAAGAA 3 (0.000090%)

TGGAAAGCGGTCTGCCCCTGGAGGTGGTAGCAAGGTTCCACAGAAAAAAGTAAAACTTGCTGCTGATGA
AGATGATGACGATGATGATGAAGAGGATGAT 6 (0.000180%)

TGGAAAGTCACCCGTAAGGATGGCAATGCCAGTGGAACCACGCTGCTTGAGGCTCTGGACTGCATCCTA
CCACCAACTCGTCCAACTGACAAGCCCTTGC 77 (0.002314%)

TGGAAGATGGCCCTAAATTCTTGAAGTCTGGTGATGCTGCCATTGTTGATATGGTTCCTGGCAAGCCCAT
GTGTGTTGAGAGCTTCTCAGACTATCCACC 54 (0.001623%)

TGGAAGCAATTTTGGAGGTGGTGGAAGCTACAATGATTTTGGGAATTACAACAATCAGTCTTCAAATTTT
GGACCCATGAAGGGAGGAAATTTTGGAGGC 8 (0.000240%)

TGGACAAGAAGGCTGCTGGAGCTGGCAAGGTCACCAAGTCTGCCCAGAAAGCTCAGAAGGCTAAATGA
ATATTATCCCTAATACCTGCCACCCCACTCTT 18 (0.000541%)

TGGACACGTAGATTCGGGCAAGTCCACCACTACTGGCCATCTGATCTATAAATGCGGTGGCATCGACAA
AAGAACCATTGAAAAATTTGAGAAGGAGGCT 36 (0.001082%)

TGGAGACTGGTGTTCTCAAACCCGGTATGGTGGTCACCTTTGCTCCAGTCAACGTTACAACGGAAGTAAA
ATCTGTCGAAATGCACCATGAAGCTTTGAG 28 (0.000841%)

TGGAGCCAAGTGCTAACATGCCTTGGTTCAAGGGATGGAAAGTCACCCGTAAGGATGGCAATGCCAGTG
GAACCACGCTGCTTGAGGCTCTGGACTGCAT 628 (0.018870%)

TGGAGCTGGCAAGGTCACCAAGTCTGCCCAGAAAGCTCAGAAGGCTAAATGAATATTATCCCTAATACC
TGCCACCCCACTCTTAATCAGTGGTGGAAGA 5 (0.000150%)

TGGAGGTGGTGGAAGCTACAATGATTTTGGGAATTACAACAATCAGTCTTCAAATTTTGGACCCATGAA
GGGAGGAAATTTTGGAGGCAGAAGCTCTGGC 3 (0.000090%)

TGGATAAACTGAAAGCTGAGCGTGAACGTGGTATCACCATTGATATCTCCTTGTGGAAATTTGAGACCA
GCAAGTACTATGTGACTATCATTGATGCCCC 4 (0.000120%)

TGGATTGCCACACGGCTCACATTGCATGCAAGTTTGCTGAGCTGAAGGAAAAGATTGATCGCCGTTCTGG
TAAAAAGCTGGAAGATGGCCCTAAATTCTT 626 (0.018809%)

TGGCAAGCCCATGTGTGTTGAGAGCTTCTCAGACTATCCACCTTTGGGTCGCTTTGCTGTTCGTGATATGA
GACAGACAGTTGCGGTGGGTGTCATCAAA44 (0.001322%)

TGGCCCTAAATTCTTGAAGTCTGGTGATGCTGCCATTGTTGATATGGTTCCTGGCAAGCCCATGTGTGTTG
AGAGCTTCTCAGACTATCCACCTTTGGGT 10 (0.000300%)

TGGCGTTTCCCCGCATAAACAACATAAGCTTCTGACTCTTACCTCCCTCTCTCCTACTCCTGCTCGCATCT
GCTATAGTGGAGGCCGGAGCAGGAACAGG 9 (0.000270%)

TGGCTTACACACTGGGTGTGAAACAACTAATTGTCGGTGTTAACAAAATGGATTCCACTGAGCCACCCTA
CAGCCAGAAGAGATATGAGGAAATTGTTAA 3 (0.000090%)

TGGGAATTACAACAATCAGTCTTCAAATTTTGGACCCATGAAGGGAGGAAATTTTGGAGGCAGAAGCTC
TGGCCCCTATGGCGGTGGAGGCCAATACTTT 10 (0.000300%)

TGGGCAGACCCGAGAGCATGCCCTTCTGGCTTACACACTGGGTGTGAAACAACTAATTGTCGGTGTTAAC
AAAATGGATTCCACTGAGCCACCCTACAGC 653 (0.019621%)

TGGGCTTCAATGTCAAGAATGTGTCTGTCAAGGATGTTCGTCGTGGCAACGTTGCTGGTGACAGCAAAA
ATGACCCACCAATGGAAGCAGCTGGCTTCAC 26 (0.000781%)

TGGGTCCCTCGGCTTCCTGCCTCGGAAGCGCAGCAGCAGGCATCGTGGGAAGGTGAAGAGCTTCCCTAA
GGATGACCCGTCCAAGCCGGTCCACCTCACA 3 (0.000090%)

TGGGTCGCTTTGCTGTTCGTGATATGAGACAGACAGTTGCGGTGGGTGTCATCAAAGCAGTGGACAAGA
AGGCTGCTGGAGCTGGCAAGGTCACCAAGTC 3 (0.000090%)

TGGGTCTTGGATAAACTGAAAGCTGAGCGTGAACGTGGTATCACCATTGATATCTCCTTGTGGAAATTTG

AGACCAGCAAGTACTATGTGACTATCATTG 24 (0.000721%)

TGGGTGTCATCAAAGCAGTGGACAAGAAGGCTGCTGGAGCTGGCAAGGTCACCAAGTCTGCCCAGAAA
GCTCAGAAGGCTAAATGAATATTATCCCTAAT 7 (0.000210%)

TGGTAAAAAGCTGGAAGATGGCCCTAAATTCTTGAAGTCTGGTGATGCTGCCATTGTTGATATGGTTCCT
GGCAAGCCCATGTGTGTTGAGAGCTTCTCA 9 (0.000270%)

TGGTAATGATGGAAGCAATTTTGGAGGTGGTGGAAGCTACAATGATTTTGGGAATTACAACAATCAGTC
TTCAAATTTTGGACCCATGAAGGGAGGAAAT 299 (0.008984%)

TGGTATCACCATTGATATCTCCTTGTGGAAATTTGAGACCAGCAAGTACTATGTGACTATCATTGATGCC
CCAGGACACAGAGACTTTATCAAAAACATG 3 (0.000090%)

TGGTCACCTTTGCTCCAGTCAACGTTACAACGGAAGTAAAATCTGTCGAAATGCACCATGAAGCTTTGAG
TGAAGCTCTTCCTGGGGACAATGTGGGCTT 27 (0.000811%)

TGGTCGAGGCCATCTCCTGGGCCGCCTGGCGGCCATCGTGGCTAAACAGGTACTGCTGGGCCGGAAGGT
GGTGGTCGTACGCTGTGAAGGCATCAACATT 4 (0.000120%)

TGGTGATGCTGCCATTGTTGATATGGTTCCTGGCAAGCCCATGTGTGTTGAGAGCTTCTCAGACTATCCA
CCTTTGGGTCGCTTTGCTGTTCGTGATATG 23 (0.000691%)

TGGTGCGGGAGCTACGGGGCCCAGGGATTGTGTTTAAAGTAGTGCTTCTACCAACATGTCCCGTGGTTCC
AGCGCCGGTTTTGACCGCCACATTACCATT 3 (0.000090%)

TGGTGGTCACCTTTGCTCCAGTCAACGTTACAACGGAAGTAAAATCTGTCGAAATGCACCATGAAGCTTT
GAGTGAAGCTCTTCCTGGGGACAATGTGGG 12 (0.000361%)

TGGTGTTCTCAAACCCGGTATGGTGGTCACCTTTGCTCCAGTCAACGTTACAACGGAAGTAAAATCTGTC
GAAATGCACCATGAAGCTTTGAGTGAAGCT 14 (0.000421%)

TGGTTCCTGGCAAGCCCATGTGTGTTGAGAGCTTCTCAGACTATCCACCTTTGGGTCGCTTTGCTGTTCGT
GATATGAGACAGACAGTTGCGGTGGGTGT5 (0.000150%)

TGTAACTTACTACTCCGGAAAAAAAGAACCATTTGGATACATAGGTATGGTCTGAGCTATGATATCAATT
GGCTTCCTAGGGTTTATCGTGTGAGCACAC 11 (0.000331%)

TGTAGGCCCCTACGGGCTACTACAACCCTTCGCTGACGCCATAAAACTCTTCACCAAAGAGCCCCTAAAA
CCCGCCACATCTACCATCACCCTCTACATC 6 (0.000180%)

TGTCAAGAATGTGTCTGTCAAGGATGTTCGTCGTGGCAACGTTGCTGGTGACAGCAAAAATGACCCACC
AATGGAAGCAGCTGGCTTCACTGCTCAGGTG 13 (0.000391%)

TGTCAAGGATGTTCGTCGTGGCAACGTTGCTGGTGACAGCAAAAATGACCCACCAATGGAAGCAGCTGG
CTTCACTGCTCAGGTGATTATCCTGAACCAT 14 (0.000421%)

TGTCATCAAAGCAGTGGACAAGAAGGCTGCTGGAGCTGGCAAGGTCACCAAGTCTGCCCAGAAAGCTCA
GAAGGCTAAATGAATATTATCCCTAATACCT 24 (0.000721%)

TGTCCTGATTGTTGCTGCTGGTGTTGGTGAATTTGAAGCTGGTATCTCCAAGAATGGGCAGACCCGAGAG
CATGCCCTTCTGGCTTACACACTGGGTGTG 3 (0.000090%)

TGTCGTCATTGGACACGTAGATTCGGGCAAGTCCACCACTACTGGCCATCTGATCTATAAATGCGGTGGC
ATCGACAAAAGAACCATTGAAAAATTTGAG 6 (0.000180%)

TGTCGTGAAAACTACCCCTAAAAGCCAAAATGGGAAAGGAAAAGACTCATATCAACATTGTCGTCATTG
GACACGTAGATTCGGGCAAGTCCACCACTAC 11 (0.000331%)

TGTCTGTCAAGGATGTTCGTCGTGGCAACGTTGCTGGTGACAGCAAAAATGACCCACCAATGGAAGCAG
CTGGCTTCACTGCTCAGGTGATTATCCTGAA 22 (0.000661%)

TGTGAAACAACTAATTGTCGGTGTTAACAAAATGGATTCCACTGAGCCACCCTACAGCCAGAAGAGATA
TGAGGAAATTGTTAAGGAAGTCAGCACTTAC 9 (0.000270%)

TGTGACTATCATTGATGCCCCAGGACACAGAGACTTTATCAAAAACATGATTACAGGGACATCTCAGGC
TGACTGTGCTGTCCTGATTGTTGCTGCTGGT 28 (0.000841%)

TGTGCCTCGCTGAGGAAAAATAACTAAACATGGGCAAAGGAGATCCTAAGAAGCCGAGAGGCAAAATG
TCATCATATGCATTTTTTGTGCAAACTTGTCG 5 (0.000150%)

TGTGCTGTCCTGATTGTTGCTGCTGGTGTTGGTGAATTTGAAGCTGGTATCTCCAAGAATGGGCAGACCC
GAGAGCATGCCCTTCTGGCTTACACACTGG 3 (0.000090%)

TGTGTCTGTCAAGGATGTTCGTCGTGGCAACGTTGCTGGTGACAGCAAAAATGACCCACCAATGGAAGC
AGCTGGCTTCACTGCTCAGGTGATTATCCTG 5 (0.000150%)

TGTGTGTTGAGAGCTTCTCAGACTATCCACCTTTGGGTCGCTTTGCTGTTCGTGATATGAGACAGACAGTT
GCGGTGGGTGTCATCAAAGCAGTGGACAA 8 (0.000240%)

TGTGTTTAAAGTAGTGCTTCTACCAACATGTCCCGTGGTTCCAGCGCCGGTTTTGACCGCCACATTACCAT
TTTTTCACCCGAGGGTCGGCTCTACCAAG 5 (0.000150%)

TGTTCTCAAACCCGGTATGGTGGTCACCTTTGCTCCAGTCAACGTTACAACGGAAGTAAAATCTGTCGAA
ATGCACCATGAAGCTTTGAGTGAAGCTCTT 16 (0.000481%)

TGTTGATATGGTTCCTGGCAAGCCCATGTGTGTTGAGAGCTTCTCAGACTATCCACCTTTGGGTCGCTTTG
CTGTTCGTGATATGAGACAGACAGTTGCG 65 (0.001953%)

TTAATCATTTTTATTGCCACAACTAACCTCCTCGGACTCCTGCCTCACTCATTTACACCAACCACCCAACT
ATCTATAAACCTAGCCATGGCCATCCCCT 3 (0.000090%)

TTACACACTGGGTGTGAAACAACTAATTGTCGGTGTTAACAAAATGGATTCCACTGAGCCACCCTACAGC
CAGAAGAGATATGAGGAAATTGTTAAGGAA 3 (0.000090%)

TTACACCAACCACCCAACTATCTATAAACCTAGCCATGGCCATCCCCTTATGAGCGGGCGCAGTGATTAT
AGGCTTTCGCTCTAAGATTAAAAATGCCCT 3 (0.000090%)

TTACATAACAGACGAGGTCAACGATCCCTCCCTTACCATCAAATCAATTGGCCACCAATGGTACTGAACC
TACGAGTACACCGACTACGGCGGACTAATC 4 (0.000120%)

TTACCTCAGAAGTTTTTTTCTTCGCAGGATTTTTCTGAGCCTTTTACCACTCCAGCCTAGCCCCTACCCCC
CAATTAGGAGGGCACTGGCCCCCAACAGG 7 (0.000210%)

TTACTACTCCGGAAAAAAAGAACCATTTGGATACATAGGTATGGTCTGAGCTATGATATCAATTGGCTTC
CTAGGGTTTATCGTGTGAGCACACCATATA 4 (0.000120%)

TTACTTGCTTCGTCTGTTCTGTGTTGGTTTTACTAAAAAACGCAACAATCAGATACGGAAGACCTCTTATG
CTCAGCACCAACAGGTCCGCCAAATCCGG3 (0.000090%)

TTATCAAAAACATGATTACAGGGACATCTCAGGCTGACTGTGCTGTCCTGATTGTTGCTGCTGGTGTTGG
TGAATTTGAAGCTGGTATCTCCAAGAATGG 3 (0.000090%)

TTATTACCTCAGAAGTTTTTTTCTTCGCAGGATTTTTCTGAGCCTTTTACCACTCCAGCCTAGCCCCTACCC
CCCAATTAGGAGGGCACTGGCCCCCAAC 28 (0.000841%)

TTCAACTTCACCCGTAACCCACCGCCATGGCCGAGGAAGGCATTGCTGCTGGAGGTGTAATGGACGTTA
ATACTGCTTTACAAGAGGTTCTGAAGACTGC 25 (0.000751%)

TTCAAGCACTGCTTATTACAATTTTACTGGGTCTCTATTTTACCCTCCTACAAGCCTCAGAGTACTTCGAG
TCTCCCTTCACCATTTCCGACGGCATCTA 15 (0.000451%)

TTCAAGTATGCCTGGGTCTTGGATAAACTGAAAGCTGAGCGTGAACGTGGTATCACCATTGATATCTCCT
TGTGGAAATTTGAGACCAGCAAGTACTATG 42 (0.001262%)

TTCAATGTCAAGAATGTGTCTGTCAAGGATGTTCGTCGTGGCAACGTTGCTGGTGACAGCAAAAATGACC
CACCAATGGAAGCAGCTGGCTTCACTGCTC 34 (0.001022%)

TTCACCCTCCACTTCCCGTCTCAGAATCTAAACGTGGTCACCTTCGAGTAGAGAGGCCCGCCCGCCCACC
GTGGGCAGTGCCACCCGCAGATGACACGCG 8 (0.000240%)

TTCATCCCTCTCCAGAAGAGGAGAAGAGGAAACACAAGAAGAAACGCCTGGTGCAGAGCCCCAATTCCT
ACTTCATGGATGTGAAATGCCCAGGATGCTA 3 (0.000090%)

TTCCACCCTTACTACACAATCAAAGACGCCCTCGGCTTACTTCTCTTCATTCTCTCCTTAATGACATTAAC
ACTATTCTCACCAGACCTCCTAGGCGACC 7 (0.000210%)

TTCCCCCATTATTCCTAGAACCAGGCGACCTGCGACTCCTTGACGTTGACAATCGAGTAGTACTCCCGAT
TGAAGCCCCCATTCGTATAATAATTACATC 5 (0.000150%)

TTCCTATTCTACACCCTAGTAGGCTCCCTTCCCCTACTCATCGCACTGATTTACACTCACAACACCCTAGG
CTCACTAAACATTCTACTACTCACTCTCA 13 (0.000391%)

TTCCTGGCAAGCCCATGTGTGTTGAGAGCTTCTCAGACTATCCACCTTTGGGTCGCTTTGCTGTTCGTGAT
ATGAGACAGACAGTTGCGGTGGGTGTCAT20 (0.000601%)

TTCGATACGGGATAATCCTATTTATTACCTCAGAAGTTTTTTTCTTCGCAGGATTTTTCTGAGCCTTTTACC
ACTCCAGCCTAGCCCCTACCCCCCAATT 8 (0.000240%)

TTCGCATCCGCAAACTCTGTCTCAACATCTGTGTTGGGGAGAGTGGAGACAGACTGACGCGAGCAGCCA
AGGTGTTGGAGCAGCTCACAGGGCAGACCCC 13 (0.000391%)

TTCGGAATAATCTCCCATATTGTAACTTACTACTCCGGAAAAAAAGAACCATTTGGATACATAGGTATGG
TCTGAGCTATGATATCAATTGGCTTCCTAG 5 (0.000150%)

TTCGGGCAAGTCCACCACTACTGGCCATCTGATCTATAAATGCGGTGGCATCGACAAAAGAACCATTGA
AAAATTTGAGAAGGAGGCTGCTGAGATGGGA 9 (0.000270%)

TTCTCAAACCCGGTATGGTGGTCACCTTTGCTCCAGTCAACGTTACAACGGAAGTAAAATCTGTCGAAAT
GCACCATGAAGCTTTGAGTGAAGCTCTTCC 9 (0.000270%)

TTCTCAGACTATCCACCTTTGGGTCGCTTTGCTGTTCGTGATATGAGACAGACAGTTGCGGTGGGTGTCA
TCAAAGCAGTGGACAAGAAGGCTGCTGGAG 7 (0.000210%)

TTCTGGCTTACACACTGGGTGTGAAACAACTAATTGTCGGTGTTAACAAAATGGATTCCACTGAGCCACC
CTACAGCCAGAAGAGATATGAGGAAATTGT 16 (0.000481%)

TTCTGGTAAAAAGCTGGAAGATGGCCCTAAATTCTTGAAGTCTGGTGATGCTGCCATTGTTGATATGGTT
CCTGGCAAGCCCATGTGTGTTGAGAGCTTC 16 (0.000481%)

TTCTTGAAGTCTGGTGATGCTGCCATTGTTGATATGGTTCCTGGCAAGCCCATGTGTGTTGAGAGCTTCTC
AGACTATCCACCTTTGGGTCGCTTTGCTG 7 (0.000210%)

TTGAAGTCTGGTGATGCTGCCATTGTTGATATGGTTCCTGGCAAGCCCATGTGTGTTGAGAGCTTCTCAG
ACTATCCACCTTTGGGTCGCTTTGCTGTTC 41 (0.001232%)

TTGAGAAGGAGGCTGCTGAGATGGGAAAGGGCTCCTTCAAGTATGCCTGGGTCTTGGATAAACTGAAAG

CTGAGCGTGAACGTGGTATCACCATTGATAT 33 (0.000992%)

TTGAGACCAGCAAGTACTATGTGACTATCATTGATGCCCCAGGACACAGAGACTTTATCAAAAACATGA
TTACAGGGACATCTCAGGCTGACTGTGCTGT 17 (0.000511%)

TTGAGAGCTTCTCAGACTATCCACCTTTGGGTCGCTTTGCTGTTCGTGATATGAGACAGACAGTTGCGGT
GGGTGTCATCAAAGCAGTGGACAAGAAGGC 5 (0.000150%)

TTGATATCTCCTTGTGGAAATTTGAGACCAGCAAGTACTATGTGACTATCATTGATGCCCCAGGACACAG
AGACTTTATCAAAAACATGATTACAGGGAC 34 (0.001022%)

TTGATATGGTTCCTGGCAAGCCCATGTGTGTTGAGAGCTTCTCAGACTATCCACCTTTGGGTCGCTTTGCT
GTTCGTGATATGAGACAGACAGTTGCGGT 8 (0.000240%)

TTGATGCCCCAGGACACAGAGACTTTATCAAAAACATGATTACAGGGACATCTCAGGCTGACTGTGCTG
TCCTGATTGTTGCTGCTGGTGTTGGTGAATT 7 (0.000210%)

TTGATGGTCGAGGCCATCTCCTGGGCCGCCTGGCGGCCATCGTGGCTAAACAGGTACTGCTGGGCCGGA
AGGTGGTGGTCGTACGCTGTGAAGGCATCAA 3 (0.000090%)

TTGCCACACGGCTCACATTGCATGCAAGTTTGCTGAGCTGAAGGAAAAGATTGATCGCCGTTCTGGTAAA
AAGCTGGAAGATGGCCCTAAATTCTTGAAG 12 (0.000361%)

TTGCGGTGGGTGTCATCAAAGCAGTGGACAAGAAGGCTGCTGGAGCTGGCAAGGTCACCAAGTCTGCCC
AGAAAGCTCAGAAGGCTAAATGAATATTATC 4 (0.000120%)

TTGGACACGTAGATTCGGGCAAGTCCACCACTACTGGCCATCTGATCTATAAATGCGGTGGCATCGACA
AAAGAACCATTGAAAAATTTGAGAAGGAGGC 7 (0.000210%)

TTGGACCAATCTATCACCCTATAGAAGAACTAATGTTAGTATAAGTAACATGAAAACATTCTCCTCCGCA
TAAGCCTGCGTCAGATTAAAACACTGAACT 5 (0.000150%)

TTGGATAAACTGAAAGCTGAGCGTGAACGTGGTATCACCATTGATATCTCCTTGTGGAAATTTGAGACCA
GCAAGTACTATGTGACTATCATTGATGCCC 4 (0.000120%)

TTGGCCGAGTGGAGACTGGTGTTCTCAAACCCGGTATGGTGGTCACCTTTGCTCCAGTCAACGTTACAAC
GGAAGTAAAATCTGTCGAAATGCACCATGA 6 (0.000180%)

TTGGGAATTACAACAATCAGTCTTCAAATTTTGGACCCATGAAGGGAGGAAATTTTGGAGGCAGAAGCT
CTGGCCCCTATGGCGGTGGAGGCCAATACTT 9 (0.000270%)

TTGTAACTTACTACTCCGGAAAAAAAGAACCATTTGGATACATAGGTATGGTCTGAGCTATGATATCAAT
TGGCTTCCTAGGGTTTATCGTGTGAGCACA 31 (0.000931%)

TTGTGCCAATTTCTGGTTGGAATGGTGACAACATGCTGGAGCCAAGTGCTAACATGCCTTGGTTCAAGGG
ATGGAAAGTCACCCGTAAGGATGGCAATGC 7 (0.000210%)

TTGTGTTTAAAGTAGTGCTTCTACCAACATGTCCCGTGGTTCCAGCGCCGGTTTTGACCGCCACATTACCA
TTTTTTCACCCGAGGGTCGGCTCTACCAA 3 (0.000090%)

TTTACACCAACCACCCAACTATCTATAAACCTAGCCATGGCCATCCCCTTATGAGCGGGCGCAGTGATTA
TAGGCTTTCGCTCTAAGATTAAAAATGCCC 46 (0.001382%)

TTTACATAACAGACGAGGTCAACGATCCCTCCCTTACCATCAAATCAATTGGCCACCAATGGTACTGAAC
CTACGAGTACACCGACTACGGCGGACTAAT 17 (0.000511%)

TTTATCAAAAACATGATTACAGGGACATCTCAGGCTGACTGTGCTGTCCTGATTGTTGCTGCTGGTGTTG
GTGAATTTGAAGCTGGTATCTCCAAGAATG 40 (0.001202%)

TTTATTACCTCAGAAGTTTTTTCTTCGCAGGATTTTTCTGAGCCTTTTACCACTCCAGCCTAGCCCCTACC CCCCAATTAGGAGGGCACTGGCCCCCAA 10 (0.000300%)

TTTATTGCCACAACTAACCTCCTCGGACTCCTGCCTCACTCATTTACACCAACCACCCAACTATCTATAAA CCTAGCCATGGCCATCCCCTTATGAGCGG 4 (0.000120%)

TTTGACTGCTTCCATGTTGGCATCTGCCCCTCCTCAAGAGCAAAAGCAAATGTTGGGTGAACGGCTGTTT CCTCTTATTCAAGCCATGCACCCTACTCTT 3 (0.000090%)

TTTGAGAAGGAGGCTGCTGAGATGGGAAAGGGCTCCTTCAAGTATGCCTGGGTCTTGGATAAACTGAAA GCTGAGCGTGAACGTGGTATCACCATTGATA 8 (0.000240%)

TTTGAGACCAGCAAGTACTATGTGACTATCATTGATGCCCCAGGACACAGAGACTTTATCAAAAACATG ATTACAGGGACATCTCAGGCTGACTGTGCTG 31 (0.000931%)

TTTGGAGGTGGTGGAAGCTACAATGATTTTGGGAATTACAACAATCAGTCTTCAAATTTTGGACCCATGA AGGGAGGAAATTTTGGAGGCAGAAGCTCTG 6 (0.000180%)

TTTGGGAATTACAACAATCAGTCTTCAAATTTTGGACCCATGAAGGGAGGAAATTTTGGAGGCAGAAGC TCTGGCCCCTATGGCGGTGGAGGCCAATACT 20 (0.000601%)

TTTGGGTCGCTTTGCTGTTCGTGATATGAGACAGACAGTTGCGGTGGGTGTCATCAAAGCAGTGGACAAG AAGGCTGCTGGAGCTGGCAAGGTCACCAAG 5 (0.000150%)

TTTGTGCCAATTTCTGGTTGGAATGGTGACAACATGCTGGAGCCAAGTGCTAACATGCCTTGGTTCAAGG GATGGAAAGTCACCCGTAAGGATGGCAATG 19 (0.000571%)

TTTTATTGCCACAACTAACCTCCTCGGACTCCTGCCTCACTCATTTACACCAACCACCCAACTATCTATAA ACCTAGCCATGGCCATCCCCTTATGAGCG 20 (0.000601%)

TTTTCTTCGCAGGATTTTTCTGAGCCTTTTACCACTCCAGCCTAGCCCCTACCCCCCAATTAGGAGGGCAC TGGCCCCCAACAGGCATCACCCCGCTAAA 11 (0.000331%)

TTTTGGAGGTGGTGGAAGCTACAATGATTTTGGGAATTACAACAATCAGTCTTCAAATTTTGGACCCATG AAGGGAGGAAATTTTGGAGGCAGAAGCTCT 4 (0.000120%)

TTTTGGGAATTACAACAATCAGTCTTCAAATTTTGGACCCATGAAGGGAGGAAATTTTGGAGGCAGAAG CTCTGGCCCCTATGGCGGTGGAGGCCAATAC 5 (0.000150%)

TTTTTATTGCCACAACTAACCTCCTCGGACTCCTGCCTCACTCATTTACACCAACCACCCAACTATCTATA AACCTAGCCATGGCCATCCCCTTATGAGC 11 (0.000331%)

TTTTTCTTCGCAGGATTTTTCTGAGCCTTTTACCACTCCAGCCTAGCCCCTACCCCCCAATTAGGAGGGCA CTGGCCCCCAACAGGCATCACCCCGCTAA 15 (0.000451%)

TTTTTTCTTCGCAGGATTTTTCTGAGCCTTTTACCACTCCAGCCTAGCCCCTACCCCCCAATTAGGAGGGC ACTGGCCCCCAACAGGCATCACCCCGCTA 17 (0.000511%)

TTTTTTTCTTCGCAGGATTTTTCTGAGCCTTTTACCACTCCAGCCTAGCCCCTACCCCCCAATTAGGAGGG CACTGGCCCCCAACAGGCATCACCCCGCT 39 (0.001172%)

TTTTTTTTTTTTTTTTTTTTTTTTTTTTTTTTTTTTTTTTTTTTTTTTTTTTTTTTTTTTTTTTTTTTTTTTTTTTT TTTTTTTTTTTTTTTTTTTTTT 2675 (0.080376%)

TTTTTTTTTTTTTTTTTTTTTTTTTTTTTTTTTTTTTTTTTTTTTTTTTTTTTTTTTTTTTTTTTTTTTTTTTTTTT TTTTTTTTTTTTTTTTTTTTTTTTTTTTTTTTTTTTTTTTTTTTTTTTTTTTTTTTTTTTTTTTTTTTTTTT 1914 (0.085690%)

After filtering

After filtering: read1: quality
Value of each position will be shown on mouse over.
20406080100120140383 8.53939.540

positionqualityATCGmean

<https://plot.ly/>
After filtering: read1: base contents
Value of each position will be shown on mouse over.
2040608010012014000.10.20.30.40.50.60.7

positionbase content ratiosA(25.88%)T(27.51%)C(23.36%)G(23.17%)N(0.059%)GC(46.54%)

<https://plot.ly/>
After filtering: read1: KMER counting
Darker background means larger counts. The count will be shown on mouse over.

AA AT AC AG TA TT TC TG CA CT CC CG GA GT GC GG
AAA AAAAA AAAAT AAAAC AAAAG AAATA AAATT AAATC AAATG AAACA AAACT AAACC AAACG AAAGA AAAGT AAAGC AAAGG
AAT AATAA AATAT AATAC AATAG AATTA AATTT AATTC AATTG AATCA AATCT AATCC AATCG AATGA AATGT AATGC AATGG
AAC AACAA AACAT AACAC AACAG AACTA AACTT AACTC AACTG AACCA AACCT AACCC AACCG AACGA AACGT AACGC AACGG
AAG AAGAA AAGAT AAGAC AAGAG AAGTA AAGTT AAGTC AAGTG AAGCA AAGCT AAGCC AAGCG AAGGA AAGGT AAGGC AAGGG
ATA ATAAA ATAAT ATAAC ATAAG ATATA ATATT ATATC ATATG ATACA ATACT ATACC ATACG ATAGA ATAGT ATAGC ATAGG
ATT ATTAA ATTAT ATTAC ATTAG ATTTA ATTTT ATTTC ATTTG ATTCA ATTCT ATTCC ATTCG ATTGA ATTGT ATTGC ATTGG
ATC ATCAA ATCAT ATCAC ATCAG ATCTA ATCTT ATCTC ATCTG ATCCA ATCCT ATCCC ATCCG ATCGA ATCGT ATCGC ATCGG
ATG ATGAA ATGAT ATGAC ATGAG ATGTA ATGTT ATGTC ATGTG ATGCA ATGCT ATGCC ATGCG ATGGA ATGGT ATGGC ATGGG
ACA ACAAA ACAAT ACAAC ACAAG ACATA ACATT ACATC ACATG ACACA ACACT ACACC ACACG ACAGA ACAGT ACAGC ACAGG
ACT ACTAA ACTAT ACTAC ACTAG ACTTA ACTTT ACTTC ACTTG ACTCA ACTCT ACTCC ACTCG ACTGA ACTGT ACTGC ACTGG
ACC ACCAA ACCAT ACCAC ACCAG ACCTA ACCTT ACCTC ACCTG ACCCA ACCCT ACCCC ACCCG ACCGA ACCGT ACCGC ACCGG
ACG ACGAA ACGAT ACGAC ACGAG ACGTA ACGTT ACGTC ACGTG ACGCA ACGCT ACGCC ACGCG ACGGA ACGGT ACGGC ACGGG
AGA AGAAA AGAAT AGAAC AGAAG AGATA AGATT AGATC AGATG AGACA AGACT AGACC AGACG AGAGA AGAGT AGAGC AGAGG
AGT AGTAA AGTAT AGTAC AGTAG AGTTA AGTTT AGTTC AGTTG AGTCA AGTCT AGTCC AGTCG AGTGA AGTGT AGTGC AGTGG
AGC AGCAA AGCAT AGCAC AGCAG AGCTA AGCTT AGCTC AGCTG AGCCA AGCCT AGCCC AGCCG AGCGA AGCGT AGCGC AGCGG
AGG AGGAA AGGAT AGGAC AGGAG AGGTA AGGTT AGGTC AGGTG AGGCA AGGCT AGGCC AGGCG AGGGA AGGGT AGGGC AGGGG
TAA TAAAA TAAAT TAAAC TAAAG TAATA TAATT TAATC TAATG TAACA TAACT TAACC TAACG TAAGA TAAGT TAAGC TAAGG
TAT TATAA TATAT TATAC TATAG TATTA TATTT TATTC TATTG TATCA TATCT TATCC TATCG TATGA TATGT TATGC TATGG
TAC TACAA TACAT TACAC TACAG TACTA TACTT TACTC TACTG TACCA TACCT TACCC TACCG TACGA TACGT TACGC TACGG
TAG TAGAA TAGAT TAGAC TAGAG TAGTA TAGTT TAGTC TAGTG TAGCA TAGCT TAGCC TAGCG TAGGA TAGGT TAGGC TAGGG
TTA TTAAA TTAAT TTAAC TTAAG TTATA TTATT TTATC TTATG TTACA TTACT TTACC

TTACG TTAGA TTAGT TTAGC TTAGG
TTT TTTAA TTTAT TTTAC TTTAG TTTTA TTTTT TTTTC TTTTG TTTCA TTTCT TTTCC
TTTCG TTTGA TTTGT TTTGC TTTGG
TTC TTCAA TTCAT TTCAC TTCAG TTCTA TTCTT TTCTC TTCTG TTCCA TTCCT TTCCC
TTCCG TTCGA TTCGT TTCGC TTCGG
TTG TTGAA TTGAT TTGAC TTGAG TTGTA TTGTT TTGTC TTGTG TTGCA TTGCT TTGCC
TTGCG TTGGA TTGGT TTGGC TTGGG
TCA TCAAA TCAAT TCAAC TCAAG TCATA TCATT TCATC TCATG TCACA TCACT TCACC
TCACGTCAGA TCAGT TCAGC TCAGG
TCT TCTAA TCTAT TCTAC TCTAG TCTTA TCTTT TCTTC TCTTG TCTCA TCTCT TCTCC
TCTCG TCTGA TCTGT TCTGC TCTGG
TCC TCCAA TCCAT TCCAC TCCAG TCCTA TCCTT TCCTC TCCTG TCCCA TCCCT TCCCC
TCCCG TCCGA TCCGT TCCGC TCCGG
TCG TCGAA TCGAT TCGAC TCGAG TCGTA TCGTT TCGTC TCGTG TCGCA TCGCT TCGCC
TCGCG TCGGA TCGGT TCGGC TCGGG
TGA TGAAA TGAAT TGAAC TGAAG TGATA TGATT TGATC TGATG TGACA TGACT TGACC
TGACGTGAGA TGAGT TGAGC TGAGG
TGT TGTAA TGTAT TGTAC TGTAG TGTTA TGTTT TGTTC TGTTG TGTCA TGTCT TGTCC
TGTCG TGTGA TGTGT TGTGC TGTGG
TGC TGCAA TGCAT TGCAC TGCAG TGCTA TGCTT TGCTC TGCTG TGCCA TGCCT TGCCC
TGCCG TGCGA TGCGT TGCGC TGCGG
TGG TGGAA TGGAT TGGAC TGGAG TGGTA TGGTT TGGTC TGGTG TGGCA TGGCT TGGCC
TGGCGTGGGA TGGGT TGGGC TGGGG
CAA CAAAA CAAAT CAAAC CAAAG CAATA CAATT CAATC CAATG CAACA CAACT CAACC
CAACG CAAGA CAAGT CAAGC CAAGG
CAT CATAA CATAT CATAC CATAG CATTA CATTT CATTC CATTG CATCA CATCT CATCC
CATCG CATGA CATGT CATGC CATGG
CAC CACAA CACAT CACAC CACAG CACTA CACTT CACTC CACTG CACCA CACCT CACCC
CACCGCACGA CACGT CACGC CACGG
CAG CAGAA CAGAT CAGAC CAGAG CAGTA CAGTT CAGTC CAGTG CAGCA CAGCT CAGCC
CAGCG CAGGA CAGGT CAGGC CAGGG
CTA CTAAA CTAAT CTAAC CTAAG CTATA CTATT CTATC CTATG CTACA CTACT CTACC
CTACG CTAGA CTAGT CTAGC CTAGG
CTT CTTAA CTTAT CTTAC CTTAG CTTTA CTTTT CTTTC CTTTG CTTCA CTTCT CTTCC
CTTCG CTTGA CTTGT CTTGC CTTGG
CTC CTCAA CTCAT CTCAC CTCAG CTCTA CTCTT CTCTC CTCTG CTCCA CTCCT CTCCC
CTCCG CTCGA CTCGT CTCGC CTCGG
CTG CTGAA CTGAT CTGAC CTGAG CTGTA CTGTT CTGTC CTGTG CTGCA CTGCT CTGCC
CTGCG CTGGA CTGGT CTGGC CTGGG
CCA CCAAA CCAAT CCAAC CCAAG CCATA CCATT CCATC CCATG CCACA CCACT CCACC
CCACGCCAGA CCAGT CCAGC CCAGG
CCT CCTAA CCTAT CCTAC CCTAG CCTTA CCTTT CCTTC CCTTG CCTCA CCTCT CCTCC
CCTCG CCTGA CCTGT CCTGC CCTGG
CCC CCCAA CCCAT CCCAC CCCAG CCCTA CCCTT CCCTC CCCTG CCCCA CCCCT CCCCC
CCCCG CCCGA CCCGT CCCGC CCCGG
CCG CCGAA CCGAT CCGAC CCGAG CCGTA CCGTT CCGTC CCGTG CCGCA CCGCT CCGCC
CCGCGCCGGA CCGGT CCGGC CCGGG
CGA CGAAA CGAAT CGAAC CGAAG CGATA CGATT CGATC CGATG CGACA CGACT CGACC
CGACG CGAGA CGAGT CGAGC CGAGG
CGT CGTAA CGTAT CGTAC CGTAG CGTTA CGTTT CGTTC CGTTG CGTCA CGTCT CGTCC
CGTCG CGTGA CGTGT CGTGC CGTGG
CGC CGCAA CGCAT CGCAC CGCAG CGCTA CGCTT CGCTC CGCTG CGCCA CGCCT CGCCC
CGCCGCGCGA CGCGT CGCGC CGCGG
CGG CGGAA CGGAT CGGAC CGGAG CGGTA CGGTT CGGTC CGGTG CGGCA CGGCT CGGCC
CGGCG CGGGA CGGGT CGGGC CGGGG
GAA GAAAA GAAAT GAAAC GAAAG GAATA GAATT GAATC GAATG GAACA GAACT GAACC
GAACG GAAGA GAAGT GAAGC GAAGG
GAT GATAA GATAT GATAC GATAG GATTA GATTT GATTC GATTG GATCA GATCT GATCC
GATCGGATGA GATGT GATGC GATGG

GAC GACAA GACAT GACAC GACAG GACTA GACTT GACTC GACTG GACCA GACCT GACCC
GACCG GACGA GACGT GACGC GACGG
GAG GAGAA GAGAT GAGAC GAGAG GAGTA GAGTT GAGTC GAGTG GAGCA GAGCT GAGCC
GAGCG GAGGA GAGGT GAGGC GAGGG
GTA GTAAA GTAAT GTAAC GTAAG GTATA GTATT GTATC GTATG GTACA GTACT GTACC
GTACGGTAGA GTAGT GTAGC GTAGG
GTT GTTAA GTTAT GTTAC GTTAG GTTTA GTTTT GTTTC GTTTG GTTCA GTTCT GTTCC
GTTCG GTTGA GTTGT GTTGC GTTGG
GTC GTCAA GTCAT GTCAC GTCAG GTCTA GTCTT GTCTC GTCTG GTCCA GTCCT GTCCC
GTCCG GTCGA GTCGT GTCGC GTCGG
GTG GTGAA GTGAT GTGAC GTGAG GTGTA GTGTT GTGTC GTGTG GTGCA GTGCT GTGCC
GTGCGGTGGA GTGGT GTGGC GTGGG
GCA GCAAA GCAAT GCAAC GCAAG GCATA GCATT GCATC GCATG GCACA GCACT GCACC
GCACG GCAGA GCAGT GCAGC GCAGG
GCT GCTAA GCTAT GCTAC GCTAG GCTTA GCTTT GCTTC GCTTG GCTCA GCTCT GCTCC
GCTCG GCTGA GCTGT GCTGC GCTGG
GCC GCCAA GCCAT GCCAC GCCAG GCCTA GCCTT GCCTC GCCTG GCCCA GCCCT GCCCC
GCCCGGCCGA GCCGT GCCGC GCCGG
GCG GCGAA GCGAT GCGAC GCGAG GCGTA GCGTT GCGTC GCGTG GCGCA GCGCT GCGCC
GCGCG GCGGA GCGGT GCGGC GCGGG
GGA GGAAA GGAAT GGAAC GGAAG GGATA GGATT GGATC GGATG GGACA GGACT GGACC
GGACG GGAGA GGAGT GGAGC GGAGG
GGT GGTAA GGTAT GGTAC GGTAG GGTTA GGTTT GGTTC GGTTG GGTCA GGTCT GGTCC
GGTCGGGTGA GGTGT GGTGC GGTGG
GGC GGCAA GGCAT GGCAC GGCAG GGCTA GGCTT GGCTC GGCTG GGCCA GGCCT GGCCC
GGCCG GGCGA GGCGT GGCGC GGCGG
GGG GGGAA GGGAT GGGAC GGGAG GGGTA GGGTT GGGTC GGGTG GGGCA GGGCT GGGCC
GGGCG GGGGA GGGGT GGGGC GGGGG

After filtering: read1: overrepresented sequences
Sampling rate: 1 / 20
overrepresented sequence count (% of bases) distribution: cycle 1 ~
cycle 151

AAAAAAAAAAAAAAAAAAAAAAAAAAAAAAAAAAAAAAAAAAAAAAAAAAAAAAAAAAAAAAAAAA
AAAAAAAAAAAAAAAAAAAAAAAAAAAAAAAAAA 1776 (0.054061%)

AAAAAAAAAAAAAAAAAAAAAAAAAAAAAAAAAAAAAAAAAAAAAAAAAAAAAAAAAAAAAAAAAA
AAAAAAAAAAAAAAAAAAAAAAAAAAAAAAAAAAAAAAAAAAAAAAAAAAAAAAAAAAAAAAAAAA
AAAAAAAAAAAAAAAAA 1418 (0.064314%)

AAAAAAGAAAACCAAAGTGGTCCACAAAACATTCTCCTTTCCTTCTGAAGGTTTTACGATGCATTGTTAT
CATTAACCAGTCTTTTACTACTAAACTTAA 46 (0.001400%)

AAAAACTAATAACTTAAAACTGCCACACGCAAAAAAGAAAACCAAAGTGGTCCACAAAACATTCTCCTT
TCCTTCTGAAGGTTTTACGATGCATTGTTAT 20 (0.000609%)

AAAAAGAAAACCAAAGTGGTCCACAAAACATTCTCCTTTCCTTCTGAAGGTTTTACGATGCATTGTTATC
ATTAACCAGTCTTTTACTACTAAACTTAAA 8 (0.000244%)

AAAAAGTACTGATTTTAAAAACTAATAACTTAAAACTGCCACACGCAAAAAAGAAAACCAAAGTGGTCC
ACAAAACATTCTCCTTTCCTTCTGAAGGTTT 27 (0.000822%)

AAAACTAATAACTTAAAACTGCCACACGCAAAAAAGAAAACCAAAGTGGTCCACAAAACATTCTCCTTT
CCTTCTGAAGGTTTTACGATGCATTGTTATC 24 (0.000731%)

AAAAGAAAACCAAAGTGGTCCACAAAACATTCTCCTTTCCTTCTGAAGGTTTTACGATGCATTGTTATCA
TTAACCAGTCTTTTACTACTAAACTTAAAT7 (0.000213%)

AAAAGAAAGATGAATCCTAGGGCTCAGAGCACTGCAGCAGATCATTTCATATTGCTTCCGTGGAGTGTG
GCGAGTCAGCTAAATACTTTGACGCCGGTGG 5 (0.000152%)

AAAAGGTTGGGGAACAGCTAAATAGGTTGTTGTTGATTTGGTTAAAAAATAGTAGAGGGATGATGCTAA
TAATTAGGCTGTGGGTGGTTGTGTTGATTCA 9 (0.000274%)

AAAAGTACTGATTTTAAAAACTAATAACTTAAAACTGCCACACGCAAAAAAGAAAACCAAAGTGGTCCA
CAAAACATTCTCCTTTCCTTCTGAAGGTTTT 3 (0.000091%)

AAAATAGAGACCCAGTAAAATTGTAATAAGCAGTGCTTGAATTATTTGGTTTCGGTTGTTTTCTATTAGA
CTATGGTGAGCTCAGGTGATTGATACTCCT 73 (0.002222%)

AAAATTGTAATAAGCAGTGCTTGAATTATTTGGTTTCGGTTGTTTTCTATTAGACTATGGTGAGCTCAGGT
GATTGATACTCCTGATGCGAGTAATACGG 5 (0.000152%)

AAAGAAAACCAAAGTGGTCCACAAAACATTCTCCTTTCCTTCTGAAGGTTTTACGATGCATTGTTATCAT
TAACCAGTCTTTTACTACTAAACTTAAATG 3 (0.000091%)

AAAGAAAGATGAATCCTAGGGCTCAGAGCACTGCAGCAGATCATTTCATATTGCTTCCGTGGAGTGTGG
CGAGTCAGCTAAATACTTTGACGCCGGTGGG 6 (0.000183%)

AAAGAGGTATCTTTACTATAAAAGCTATTGTGTAAGCTAGTCATATTAAGTTGTTGGCTCAGGAGTTTGA
TAGTTCTTGGGCAGTGAGAGTGAGTAGTAG 4 (0.000122%)

AAAGTACTGATTTTAAAAACTAATAACTTAAAACTGCCACACGCAAAAAAGAAAACCAAAGTGGTCCAC
AAAACATTCTCCTTTCCTTCTGAAGGTTTTA 6 (0.000183%)

AAATAGAGACCCAGTAAAATTGTAATAAGCAGTGCTTGAATTATTTGGTTTCGGTTGTTTTCTATTAGAC
TATGGTGAGCTCAGGTGATTGATACTCCTG 11 (0.000335%)

AAATGAGTGAGGCAGGAGTCCGAGGAGGTTAGTTGTGGCAATAAAAATGATTAAGGATACTAGTATAA
GAGATCAGGTTCGTCCTTTAGTGTTGTGTATG 72 (0.002192%)

AAATTCTGTGACAAATTTTTGGTCAAGTTGTTTCCATTAAAAAGTACTGATTTTAAAAACTAATAACTTA
AAACTGCCACACGCAAAAAAGAAAACCAAA 982 (0.029892%)

AAATTGATGGCCCCTAAGATAGAGGAGACACCTGCTAGGTGTAAGGAGAAGATGGTTAGGTCTACGGAG
GCTCCAGGGTGGGAGTAGTTCCCTGCTAAGG 16 (0.000487%)

AAATTGTAATAAGCAGTGCTTGAATTATTTGGTTTCGGTTGTTTTCTATTAGACTATGGTGAGCTCAGGTG
ATTGATACTCCTGATGCGAGTAATACGGA 3 (0.000091%)

AACAATCAGGACAGCACAGTCAGCCTGAGATGTCCCTGTAATCATGTTTTTGATAAAGTCTCTGTGTCCT
GGGGCATCAATGATAGTCACATAGTACTTG 4 (0.000122%)

AACAATTTCCTCATATCTCTTCTGGCTGTAGGGTGGCTCAGTGGAATCCATTTTGTTAACACCGACAATTA
GTTGTTTCACACCCAGTGTGTAAGCCAGA 10 (0.000304%)

AACACACATGGGCTTGCCAGGAACCATATCAACAATGGCAGCATCACCAGACTTCAAGAATTTAGGGCC
ATCTTCCAGCTTTTTACCAGAACGGCGATCA 5 (0.000152%)

AACCAAAGTGGTCCACAAAACATTCTCCTTTCCTTCTGAAGGTTTTACGATGCATTGTTATCATTAACCA
GTCTTTTACTACTAAACTTAAATGGCCAAT 3 (0.000091%)

AACTTAAAACTGCCACACGCAAAAAAGAAAACCAAAGTGGTCCACAAAACATTCTCCTTTCCTTCTGAA
GGTTTTACGATGCATTGTTATCATTAACCAG 3 (0.000091%)

AAGAAAACCAAAGTGGTCCACAAAACATTCTCCTTTCCTTCTGAAGGTTTTACGATGCATTGTTATCATT
AACCAGTCTTTTACTACTAAACTTAAATGG 13 (0.000396%)

AAGAAAGATGAATCCTAGGGCTCAGAGCACTGCAGCAGATCATTTCATATTGCTTCCGTGGAGTGTGGC
GAGTCAGCTAAATACTTTGACGCCGGTGGGG 5 (0.000152%)

AAGAAAGTTAGATTTACGCCGATGAATATGATAGTGAAATGGATTTTGGCGTAGGTTTGGTCTAGGGTGT
AGCCTGAGAATAGGGGAAATCAGTGAATGA 34 (0.001035%)

AAGACGTCTTGTGATGTAATTATTATACGAATGGGGGCTTCAATCGGGAGTACTACTCGATTGTCAACGT
CAAGGAGTCGCAGGTCGCCTGGTTCTAGGA 353 (0.010745%)

AAGAGCTTCACTCAAAGCTTCATGGTGCATTTCGACAGATTTTACTTCCGTTGTAACGTTGACTGGAGCA
AAGGTGACCACCATACCGGGTTTGAGAACA 44 (0.001339%)

AAGAGGTATCTTTACTATAAAAGCTATTGTGTAAGCTAGTCATATTAAGTTGTTGGCTCAGGAGTTTGAT
AGTTCTTGGGCAGTGAGAGTGAGTAGTAGA 3 (0.000091%)

AAGATGAATCCTAGGGCTCAGAGCACTGCAGCAGATCATTTCATATTGCTTCCGTGGAGTGTGGCGAGTC
AGCTAAATACTTTGACGCCGGTGGGGATAG 5 (0.000152%)

AAGCAGCGTGGTTCCACTGGCATTGCCATCCTTACGGGTGACTTTCCATCCCTTGAACCAAGGCATGTTA
GCACTTGGCTCCAGCATGTTGTCACCATTC 5 (0.000152%)

AAGCTCTCAACACACATGGGCTTGCCAGGAACCATATCAACAATGGCAGCATCACCAGACTTCAAGAAT
TTAGGGCCATCTTCCAGCTTTTTACCAGAAC 3 (0.000091%)

AAGGAAGGGGTAGGCTATGTGTTTTGTCAGGGGGTTGAGAATGAGTGTGAGGCGTATTATACCATAGCC
GCCTAGTTTTAAGAGTACTGCGGCAAGTACT 5 (0.000152%)

AAGGAGAAGATGGTTAGGTCTACGGAGGCTCCAGGGTGGGAGTAGTTCCCTGCTAAGGGAGGGTAGACT
GTTCAACCTGTTCCTGCTCCGGCCTCCACTA 6 (0.000183%)

AAGGGCTTGTCAGTTGGACGAGTTGGTGGTAGGATGCAGTCCAGAGCCTCAAGCAGCGTGGTTCCACTG
GCATTGCCATCCTTACGGGTGACTTTCCATC 3 (0.000091%)

AAGTACTGATTTTAAAAACTAATAACTTAAAACTGCCACACGCAAAAAAGAAAACCAAAGTGGTCCACA
AAACATTCTCCTTTCCTTCTGAAGGTTTTAC 3 (0.000091%)

AAGTGCTGACTTCCTTAACAATTTCCTCATATCTCTTCTGGCTGTAGGGTGGCTCAGTGGAATCCATTTTG
TTAACACCGACAATTAGTTGTTTCACACC 5 (0.000152%)

AAGTGGAGTCCGTAAAGAGGTATCTTTACTATAAAAGCTATTGTGTAAGCTAGTCATATTAAGTTGTTGG
CTCAGGAGTTTGATAGTTCTTGGGCAGTGA 10 (0.000304%)

AATACAATGCCAGTCAGGCCACCTACGGTGAAAAGAAAGATGAATCCTAGGGCTCAGAGCACTGCAGC
AGATCATTTCATATTGCTTCCGTGGAGTGTGG 5 (0.000152%)

AATAGAGACCCAGTAAAATTGTAATAAGCAGTGCTTGAATTATTTGGTTTCGGTTGTTTTCTATTAGACT
ATGGTGAGCTCAGGTGATTGATACTCCTGA 7 (0.000213%)

AATATTCATTTAGCCTTCTGAGCTTTCTGGGCAGACTTGGTGACCTTGCCAGCTCCAGCAGCCTTCTTGTC
CACTGCTTTGATGACACCCACCGCAACTG 9 (0.000274%)

AATCATGTTTTTGATAAAGTCTCTGTGTCCTGGGGCATCAATGATAGTCACATAGTACTTGCTGGTCTCA
AATTTCCACAAGGAGATATCAATGGTGATA 5 (0.000152%)

AATGAGTAGGCTGATGGTTTCGATAATAACTAGTATGGGGATAAGGGGTGTAGGTGTGCCTTGTGGTAA
GAAGTGGGCTAGGGCATTTTTAATCTTAGAG 7 (0.000213%)

AATGAGTGAGGCAGGAGTCCGAGGAGGTTAGTTGTGGCAATAAAAATGATTAAGGATACTAGTATAAG

AGATCAGGTTCGTCCTTTAGTGTTGTGTATGG 12 (0.000365%)

AATGTTAGCGGTTAGGCGTACGGCCAGGGCTATTGGTTGAATGAGTAGGCTGATGGTTTCGATAATAACT
AGTATGGGGATAAGGGGTGTAGGTGTGCCT 4 (0.000122%)

AATTATTATACGAATGGGGGCTTCAATCGGGAGTACTACTCGATTGTCAACGTCAAGGAGTCGCAGGTC
GCCTGGTTCTAGGAATAATGGGGGAAGTATG 93 (0.002831%)

AATTATTTGGTTTCGGTTGTTTTCTATTAGACTATGGTGAGCTCAGGTGATTGATACTCCTGATGCGAGTA
ATACGGATGTGTTTAGGAGTGGGACTTCT 17 (0.000517%)

AATTCTGTGACAAATTTTTGGTCAAGTTGTTTCCATTAAAAAGTACTGATTTTAAAAACTAATAACTTAA
AACTGCCACACGCAAAAAAGAAAACCAAAG 15 (0.000457%)

AATTGATGGCCCCTAAGATAGAGGAGACACCTGCTAGGTGTAAGGAGAAGATGGTTAGGTCTACGGAGG
CTCCAGGGTGGGAGTAGTTCCCTGCTAAGGG 13 (0.000396%)

AATTGCATCTGTTTTTAAGCCTAATGTGGGGACAGCTCATGAGTGCAAGACGTCTTGTGATGTAATTATT
ATACGAATGGGGGCTTCAATCGGGAGTACT 3 (0.000091%)

AATTGGACTGCCTTCGTAATTCATTGCCTCTGCTTCAACAATGTGCAACTCATCCTTTGCACCAGCCCCTA
AACTGACCGTTCTTAAAGATAACTGGTGC 3 (0.000091%)

AATTGTAATAAGCAGTGCTTGAATTATTTGGTTTCGGTTGTTTTCTATTAGACTATGGTGAGCTCAGGTGA
TTGATACTCCTGATGCGAGTAATACGGAT 11 (0.000335%)

AATTTCCTCATATCTCTTCTGGCTGTAGGGTGGCTCAGTGGAATCCATTTTGTTAACACCGACAATTAGTT
GTTTCACACCCAGTGTGTAAGCCAGAAGG 17 (0.000517%)

AATTTTCTTAATGTAAGTGCTGACTTCCTTAACAATTTCCTCATATCTCTTCTGGCTGTAGGGTGGCTCAG
TGGAATCCATTTTGTTAACACCGACAATT 29 (0.000883%)

AATTTTGTAGACATCCTGGAGAGGCAGGCGCAAGGGCTTGTCAGTTGGACGAGTTGGTGGTAGGATGCA
GTCCAGAGCCTCAAGCAGCGTGGTTCCACTG 6 (0.000183%)

ACAAGGAAGGGGTAGGCTATGTGTTTTGTCAGGGGGTTGAGAATGAGTGTGAGGCGTATTATACCATAG
CCGCCTAGTTTTAAGAGTACTGCGGCAAGTA 4 (0.000122%)

ACAATTTCCTCATATCTCTTCTGGCTGTAGGGTGGCTCAGTGGAATCCATTTTGTTAACACCGACAATTAG
TTGTTTCACACCCAGTGTGTAAGCCAGAA 5 (0.000152%)

ACACGCAAAAAAGAAAACCAAAGTGGTCCACAAAACATTCTCCTTTCCTTCTGAAGGTTTTACGATGCAT
TGTTATCATTAACCAGTCTTTTACTACTAA 3 (0.000091%)

ACATCCTGGAGAGGCAGGCGCAAGGGCTTGTCAGTTGGACGAGTTGGTGGTAGGATGCAGTCCAGAGCC
TCAAGCAGCGTGGTTCCACTGGCATTGCCAT 4 (0.000122%)

ACATGGGCTTGCCAGGAACCATATCAACAATGGCAGCATCACCAGACTTCAAGAATTTAGGGCCATCTT
CCAGCTTTTTACCAGAACGGCGATCAATCTT 4 (0.000122%)

ACATTGTCCCCAGGAAGAGCTTCACTCAAAGCTTCATGGTGCATTTCGACAGATTTTACTTCCGTTGTAA
CGTTGACTGGAGCAAAGGTGACCACCATAC 11 (0.000335%)

ACCAAAGTGGTCCACAAAACATTCTCCTTTCCTTCTGAAGGTTTTACGATGCATTGTTATCATTAACCAGT
CTTTTACTACTAAACTTAAATGGCCAATT 5 (0.000152%)

ACCAGTTTTAGCCAACATAGCATAGTACTCTATTTCAGATTTCCTCAAAGCTGGGCAGTTGTTAGCGAGA
ATGACCAATTTCGCTTTGCCTTGTCTGATC 5 (0.000152%)

ACCGTTCTTCCACCACTGATTAAGAGTGGGGTGGCAGGTATTAGGGATAATATTCATTTAGCCTTCTGAG
CTTTCTGGGCAGACTTGGTGACCTTGCCAG 6 (0.000183%)

ACCTGAGCAGTGAAGCCAGCTGCTTCCATTGGTGGGTCATTTTTGCTGTCACCAGCAACGTTGCCACGAC
GAACATCCTTGACAGACACATTCTTGACAT 6 (0.000183%)

ACGAATGGGGGCTTCAATCGGGAGTACTACTCGATTGTCAACGTCAAGGAGTCGCAGGTCGCCTGGTTC
TAGGAATAATGGGGGAAGTATGTAGGAGTTG 3 (0.000091%)

ACGAGTTGGTGGTAGGATGCAGTCCAGAGCCTCAAGCAGCGTGGTTCCACTGGCATTGCCATCCTTACG
GGTGACTTTCCATCCCTTGAACCAAGGCATG 12 (0.000365%)

ACGCAGGCTTATGCGGAGGAGAATGTTTTCATGTTACTTATACTAACATTAGTTCTTCTATAGGGTGATA
GATTGGTCCAATTGGGTGTGAGGAGTTCAG 4 (0.000122%)

ACTCAAAGCTTCATGGTGCATTTCGACAGATTTTACTTCCGTTGTAACGTTGACTGGAGCAAAGGTGACC
ACCATACCGGGTTTGAGAACACCAGTCTCC 3 (0.000091%)

ACTCCTTTGCTACTGGTCCTGTAATGGCAGAACCTTTCATCTCGCCTTTATTGTTCACTATGACTCCTGCA
TTATCTTCAAAATAAAGAAACACGCCATC 3 (0.000091%)

ACTGATTAAGAGTGGGGTGGCAGGTATTAGGGATAATATTCATTTAGCCTTCTGAGCTTTCTGGGCAGAC
TTGGTGACCTTGCCAGCTCCAGCAGCCTTC 4 (0.000122%)

ACTGATTTTAAAAACTAATAACTTAAAACTGCCACACGCAAAAAAGAAAACCAAAGTGGTCCACAAAAC
ATTCTCCTTTCCTTCTGAAGGTTTTACGATG 7 (0.000213%)

ACTGCCACACGCAAAAAAGAAAACCAAAGTGGTCCACAAAACATTCTCCTTTCCTTCTGAAGGTTTTACG
ATGCATTGTTATCATTAACCAGTCTTTTAC 8 (0.000244%)

ACTTCCTTAACAATTTCCTCATATCTCTTCTGGCTGTAGGGTGGCTCAGTGGAATCCATTTTGTTAACACC
GACAATTAGTTGTTTCACACCCAGTGTGT 5 (0.000152%)

ACTTGGTGACCTTGCCAGCTCCAGCAGCCTTCTTGTCCACTGCTTTGATGACACCCACCGCAACTGTCTGT
CTCATATCACGAACAGCAAAGCGACCCAA 6 (0.000183%)

AGAAAACCAAAGTGGTCCACAAAACATTCTCCTTTCCTTCTGAAGGTTTTACGATGCATTGTTATCATTA
ACCAGTCTTTTACTACTAAACTTAAATGGC 11 (0.000335%)

AGAAAGATGAATCCTAGGGCTCAGAGCACTGCAGCAGATCATTTCATATTGCTTCCGTGGAGTGTGGCG
AGTCAGCTAAATACTTTGACGCCGGTGGGGA 11 (0.000335%)

AGAAAGTTAGATTTACGCCGATGAATATGATAGTGAAATGGATTTTGGCGTAGGTTTGGTCTAGGGTGTA
GCCTGAGAATAGGGGAAATCAGTGAATGAA 20 (0.000609%)

AGAACTACTGCCTTCACCATGAAGCTCCATGAGCTTTCCCAATTCAAACTTGGGCTTCTTCAGCATTTTTA
CTTTTCTAACGAAGACATCATGGAGAGGA 4 (0.000122%)

AGAAGCTCTCAACACACATGGGCTTGCCAGGAACCATATCAACAATGGCAGCATCACCAGACTTCAAGA
ATTTAGGGCCATCTTCCAGCTTTTTACCAGA 11 (0.000335%)

AGAAGGGCATGCTCTCGGGTCTGCCCATTCTTGGAGATACCAGCTTCAAATTCACCAACACCAGCAGCA
ACAATCAGGACAGCACAGTCAGCCTGAGATG 16 (0.000487%)

AGAATGATGGCTAGGGTGACTTCATATGAGATTGTTTGGGCTACTGCTCGCAGTGCGCCGATCAGGGCGT
AGTTTGAGTTTGATGCTCACCCTGATCAGA 222 (0.006758%)

AGACATCCTGGAGAGGCAGGCGCAAGGGCTTGTCAGTTGGACGAGTTGGTGGTAGGATGCAGTCCAGAG
CCTCAAGCAGCGTGGTTCCACTGGCATTGCC 5 (0.000152%)

AGACCCAGTAAAATTGTAATAAGCAGTGCTTGAATTATTTGGTTTCGGTTGTTTTCTATTAGACTATGGTG
AGCTCAGGTGATTGATACTCCTGATGCGA 5 (0.000152%)

AGACCGTTCTTCCACCACTGATTAAGAGTGGGGTGGCAGGTATTAGGGATAATATTCATTTAGCCTTCTG
AGCTTTCTGGGCAGACTTGGTGACCTTGCC 4 (0.000122%)

AGACTTGGTGACCTTGCCAGCTCCAGCAGCCTTCTTGTCCACTGCTTTGATGACACCCACCGCAACTGTC
TGTCTCATATCACGAACAGCAAAGCGACCC 7 (0.000213%)

AGAGACCCAGTAAAATTGTAATAAGCAGTGCTTGAATTATTTGGTTTCGGTTGTTTTCTATTAGACTATG
GTGAGCTCAGGTGATTGATACTCCTGATGC 5 (0.000152%)

AGAGCACTGCAGCAGATCATTTCATATTGCTTCCGTGGAGTGTGGCGAGTCAGCTAAATACTTTGACGCC
GGTGGGGATAGCGATGATTATGGTAGCGGA 10 (0.000304%)

AGAGCTTCACTCAAAGCTTCATGGTGCATTTCGACAGATTTTACTTCCGTTGTAACGTTGACTGGAGCAA
AGGTGACCACCATACCGGGTTTGAGAACAC 11 (0.000335%)

AGAGGAGACACCTGCTAGGTGTAAGGAGAAGATGGTTAGGTCTACGGAGGCTCCAGGGTGGGAGTAGT
TCCCTGCTAAGGGAGGGTAGACTGTTCAACCT 4 (0.000122%)

AGAGGCAGGCGCAAGGGCTTGTCAGTTGGACGAGTTGGTGGTAGGATGCAGTCCAGAGCCTCAAGCAGC
GTGGTTCCACTGGCATTGCCATCCTTACGGG 9 (0.000274%)

AGAGGGAGTATAGGGCTGTGACTAGTATGTTGAGTCCTGTAAGTAGGAGAGTGATATTTGATCAGGAGA
ACGTGGTTACTAGCACAGAGAGTTCTCCCAG 9 (0.000274%)

AGAGGTGATCGGCGATCAGAGGGCGATGAAGTTCTAGATCCATTGAGACAAGCTCTAGACAGTAGCATG
CAGTCCCACAACTTGTACCAGCATCCCCAGC 429 (0.013059%)

AGAGTGGGGTGGCAGGTATTAGGGATAATATTCATTTAGCCTTCTGAGCTTTCTGGGCAGACTTGGTGAC
CTTGCCAGCTCCAGCAGCCTTCTTGTCCAC 3 (0.000091%)

AGATACCTGTGGTAGTCCCCTTTCATTTTATAATAGAAAACCTTGGACTCGCCAGTGTTAGCTGCTGGAA
TGAGGTGTTTGTCCAGTACATCCAGAATGT 3 (0.000091%)

AGATAGAGGAGACACCTGCTAGGTGTAAGGAGAAGATGGTTAGGTCTACGGAGGCTCCAGGGTGGGAG
TAGTTCCCTGCTAAGGGAGGGTAGACTGTTCA 6 (0.000183%)

AGATAGTTGGGTGGTTGGTGTAAATGAGTGAGGCAGGAGTCCGAGGAGGTTAGTTGTGGCAATAAAAAT
GATTAAGGATACTAGTATAAGAGATCAGGTT 22 (0.000670%)

AGATTTACGCCGATGAATATGATAGTGAAATGGATTTTGGCGTAGGTTTGGTCTAGGGTGTAGCCTGAGA
ATAGGGGAAATCAGTGAATGAAGCCTCCTA 4 (0.000122%)

AGCAACAATCAGGACAGCACAGTCAGCCTGAGATGTCCCTGTAATCATGTTTTTGATAAAGTCTCTGTGT
CCTGGGGCATCAATGATAGTCACATAGTAC 13 (0.000396%)

AGCACTGCAGCAGATCATTTCATATTGCTTCCGTGGAGTGTGGCGAGTCAGCTAAATACTTTGACGCCGG
TGGGGATAGCGATGATTATGGTAGCGGAGG 7 (0.000213%)

AGCAGATCATTTCATATTGCTTCCGTGGAGTGTGGCGAGTCAGCTAAATACTTTGACGCCGGTGGGGATA
GCGATGATTATGGTAGCGGAGGTGAAATAT 15 (0.000457%)

AGCAGCGTGGTTCCACTGGCATTGCCATCCTTACGGGTGACTTTCCATCCCTTGAACCAAGGCATGTTAG
CACTTGGCTCCAGCATGTTGTCACCATTCC 10 (0.000304%)

AGCCAATTTTCTTAATGTAAGTGCTGACTTCCTTAACAATTTCCTCATATCTCTTCTGGCTGTAGGGTGGC

TCAGTGGAATCCATTTTGTTAACACCGAC 17 (0.000517%)

AGCCAGAAGGGCATGCTCTCGGGTCTGCCCATTCTTGGAGATACCAGCTTCAAATTCACCAACACCAGC
AGCAACAATCAGGACAGCACAGTCAGCCTGA 4 (0.000122%)

AGCCTTCTGAGCTTTCTGGGCAGACTTGGTGACCTTGCCAGCTCCAGCAGCCTTCTTGTCCACTGCTTTGA
TGACACCCACCGCAACTGTCTGTCTCATA 13 (0.000396%)

AGCGGTTAGGCGTACGGCCAGGGCTATTGGTTGAATGAGTAGGCTGATGGTTTCGATAATAACTAGTAT
GGGGATAAGGGGTGTAGGTGTGCCTTGTGGT 5 (0.000152%)

AGCGTGGTTCCACTGGCATTGCCATCCTTACGGGTGACTTTCCATCCCTTGAACCAAGGCATGTTAGCAC
TTGGCTCCAGCATGTTGTCACCATTCCAAC 15 (0.000457%)

AGCTCCAGCAGCCTTCTTGTCCACTGCTTTGATGACACCCACCGCAACTGTCTGTCTCATATCACGAACA
GCAAAGCGACCCAAAGGTGGATAGTCTGAG 4 (0.000122%)

AGCTGCTTCCATTGGTGGGTCATTTTTGCTGTCACCAGCAACGTTGCCACGACGAACATCCTTGACAGAC
ACATTCTTGACATTGAAGCCCACATTGTCC 5 (0.000152%)

AGGAAGAGCTTCACTCAAAGCTTCATGGTGCATTTCGACAGATTTTACTTCCGTTGTAACGTTGACTGGA
GCAAAGGTGACCACCATACCGGGTTTGAGA 29 (0.000883%)

AGGAGAAGATGGTTAGGTCTACGGAGGCTCCAGGGTGGGAGTAGTTCCCTGCTAAGGGAGGGTAGACTG
TTCAACCTGTTCCTGCTCCGGCCTCCACTAT 4 (0.000122%)

AGGAGGGTAAAATAGAGACCCAGTAAAATTGTAATAAGCAGTGCTTGAATTATTTGGTTTCGGTTGTTTT
CTATTAGACTATGGTGAGCTCAGGTGATTG 174 (0.005297%)

AGGATAATCACCTGAGCAGTGAAGCCAGCTGCTTCCATTGGTGGGTCATTTTTGCTGTCACCAGCAACGT
TGCCACGACGAACATCCTTGACAGACACAT 11 (0.000335%)

AGGATGCAGTCCAGAGCCTCAAGCAGCGTGGTTCCACTGGCATTGCCATCCTTACGGGTGACTTTCCATC
CCTTGAACCAAGGCATGTTAGCACTTGGCT 9 (0.000274%)

AGGCAGGCGCAAGGGCTTGTCAGTTGGACGAGTTGGTGGTAGGATGCAGTCCAGAGCCTCAAGCAGCGT
GGTTCCACTGGCATTGCCATCCTTACGGGTG 3 (0.000091%)

AGGCCACCTACGGTGAAAAGAAAGATGAATCCTAGGGCTCAGAGCACTGCAGCAGATCATTTCATATTG
CTTCCGTGGAGTGTGGCGAGTCAGCTAAATA 26 (0.000791%)

AGGCGCAAGGGCTTGTCAGTTGGACGAGTTGGTGGTAGGATGCAGTCCAGAGCCTCAAGCAGCGTGGTT
CCACTGGCATTGCCATCCTTACGGGTGACTT 12 (0.000365%)

AGGCGTACGGCCAGGGCTATTGGTTGAATGAGTAGGCTGATGGTTTCGATAATAACTAGTATGGGGATA
AGGGGTGTAGGTGTGCCTTGTGGTAAGAAGT 7 (0.000213%)

AGGGAGTCATAAGTGGAGTCCGTAAAGAGGTATCTTTACTATAAAAGCTATTGTGTAAGCTAGTCATATT
AAGTTGTTGGCTCAGGAGTTTGATAGTTCT 8 (0.000244%)

AGGGATAATATTCATTTAGCCTTCTGAGCTTTCTGGGCAGACTTGGTGACCTTGCCAGCTCCAGCAGCCT
TCTTGTCCACTGCTTTGATGACACCCACCG 3 (0.000091%)

AGGGCATGCTCTCGGGTCTGCCCATTCTTGGAGATACCAGCTTCAAATTCACCAACACCAGCAGCAACA
ATCAGGACAGCACAGTCAGCCTGAGATGTCC 8 (0.000244%)

AGGGCTATTGGTTGAATGAGTAGGCTGATGGTTTCGATAATAACTAGTATGGGGATAAGGGGTGTAGGT
GTGCCTTGTGGTAAGAAGTGGGCTAGGGCAT 3 (0.000091%)

AGGGCTCAGAGCACTGCAGCAGATCATTTCATATTGCTTCCGTGGAGTGTGGCGAGTCAGCTAAATACTT
TGACGCCGGTGGGGATAGCGATGATTATGG 11 (0.000335%)

AGGGCTTGTCAGTTGGACGAGTTGGTGGTAGGATGCAGTCCAGAGCCTCAAGCAGCGTGGTTCCACTGG
CATTGCCATCCTTACGGGTGACTTTCCATCC 9 (0.000274%)

AGGGGGTCGGAGGAAAAGGTTGGGGAACAGCTAAATAGGTTGTTGTTGATTTGGTTAAAAAATAGTAGA
GGGATGATGCTAATAATTAGGCTGTGGGTGG 5 (0.000152%)

AGGGTGGCTCAGTGGAATCCATTTTGTTAACACCGACAATTAGTTGTTTCACACCCAGTGTGTAAGCCAG
AAGGGCATGCTCTCGGGTCTGCCCATTCTT 8 (0.000244%)

AGGTACCTTTCTCTTTGGCTTCTTTCTTTTTCTGATCATTTTCCTTCACACGTTTCAGGAAGCTATCTCGGC
TCTTAGAGTGCTTAATGTGCTCAATACG 4 (0.000122%)

AGGTATTAGGGATAATATTCATTTAGCCTTCTGAGCTTTCTGGGCAGACTTGGTGACCTTGCCAGCTCCA
GCAGCCTTCTTGTCCACTGCTTTGATGACA 21 (0.000639%)

AGGTGATCGGCGATCAGAGGGCGATGAAGTTCTAGATCCATTGAGACAAGCTCTAGACAGTAGCATGCA
GTCCCACAACTTGTACCAGCATCCCCAGCGT 36 (0.001096%)

AGGTGCATGAGTAGGTGGCCTGCAGTAATGTTAGCGGTTAGGCGTACGGCCAGGGCTATTGGTTGAATG
AGTAGGCTGATGGTTTCGATAATAACTAGTA 5 (0.000152%)

AGGTGGCCTGCAGTAATGTTAGCGGTTAGGCGTACGGCCAGGGCTATTGGTTGAATGAGTAGGCTGATG
GTTTCGATAATAACTAGTATGGGGATAAGGG 12 (0.000365%)

AGTAATGTTAGCGGTTAGGCGTACGGCCAGGGCTATTGGTTGAATGAGTAGGCTGATGGTTTCGATAAT
AACTAGTATGGGGATAAGGGGTGTAGGTGTG 13 (0.000396%)

AGTACTGATTTTAAAAACTAATAACTTAAAACTGCCACACGCAAAAAAGAAAACCAAAGTGGTCCACAA
AACATTCTCCTTTCCTTCTGAAGGTTTTACG 3 (0.000091%)

AGTAGGTGGCCTGCAGTAATGTTAGCGGTTAGGCGTACGGCCAGGGCTATTGGTTGAATGAGTAGGCTG
ATGGTTTCGATAATAACTAGTATGGGGATAA 51 (0.001552%)

AGTCAGGCCACCTACGGTGAAAAGAAAGATGAATCCTAGGGCTCAGAGCACTGCAGCAGATCATTTCAT
ATTGCTTCCGTGGAGTGTGGCGAGTCAGCTA 18 (0.000548%)

AGTGAAGCCAGCTGCTTCCATTGGTGGGTCATTTTTGCTGTCACCAGCAACGTTGCCACGACGAACATCC
TTGACAGACACATTCTTGACATTGAAGCCC 6 (0.000183%)

AGTGCTGACTTCCTTAACAATTTCCTCATATCTCTTCTGGCTGTAGGGTGGCTCAGTGGAATCCATTTTGT
TAACACCGACAATTAGTTGTTTCACACCC 3 (0.000091%)

AGTGTGTAAGCCAGAAGGGCATGCTCTCGGGTCTGCCCATTCTTGGAGATACCAGCTTCAAATTCACCAA
CACCAGCAGCAACAATCAGGACAGCACAGT 3 (0.000091%)

AGTTAGTATTAGGAGGGGGGTTGTTAGGGGGTCGGAGGAAAAGGTTGGGGAACAGCTAAATAGGTTGTT
GTTGATTTGGTTAAAAAATAGTAGAGGGATG 301 (0.009162%)

AGTTGGACGAGTTGGTGGTAGGATGCAGTCCAGAGCCTCAAGCAGCGTGGTTCCACTGGCATTGCCATC
CTTACGGGTGACTTTCCATCCCTTGAACCAA 11 (0.000335%)

AGTTGGGTGGTTGGTGTAAATGAGTGAGGCAGGAGTCCGAGGAGGTTAGTTGTGGCAATAAAAATGATT
AAGGATACTAGTATAAGAGATCAGGTTCGTC 4 (0.000122%)

AGTTGGTGGTAGGATGCAGTCCAGAGCCTCAAGCAGCGTGGTTCCACTGGCATTGCCATCCTTACGGGTG
ACTTTCCATCCCTTGAACCAAGGCATGTTA 3 (0.000091%)

ATAACTTAAAACTGCCACACGCAAAAAAGAAAACCAAAGTGGTCCACAAAACATTCTCCTTTCCTTCTG AAGGTTTTACGATGCATTGTTATCATTAACC 7 (0.000213%)

ATAAGTGGAGTCCGTAAAGAGGTATCTTTACTATAAAAGCTATTGTGTAAGCTAGTCATATTAAGTTGTT GGCTCAGGAGTTTGATAGTTCTTGGGCAGT 8 (0.000244%)

ATAATATTCATTTAGCCTTCTGAGCTTTCTGGGCAGACTTGGTGACCTTGCCAGCTCCAGCAGCCTTCTTG TCCACTGCTTTGATGACACCCACCGCAAC 99 (0.003014%)

ATAATCACCTGAGCAGTGAAGCCAGCTGCTTCCATTGGTGGGTCATTTTTGCTGTCACCAGCAACGTTGC CACGACGAACATCCTTGACAGACACATTCT 5 (0.000152%)

ATACAATGCCAGTCAGGCCACCTACGGTGAAAAGAAAGATGAATCCTAGGGCTCAGAGCACTGCAGCA GATCATTTCATATTGCTTCCGTGGAGTGTGGC 7 (0.000213%)

ATACACATGAGTATTTGTCTAAAACATGTCTTCTTTGTAGCAGCTAGGCCCTGCCACCACTGTGCTTGGCT GAGTTCACAAATCTGTTGTAACCTGTAGC 24 (0.000731%)

ATACGAATGGGGGCTTCAATCGGGAGTACTACTCGATTGTCAACGTCAAGGAGTCGCAGGTCGCCTGGT TCTAGGAATAATGGGGGAAGTATGTAGGAGT 12 (0.000365%)

ATAGAGACCCAGTAAAATTGTAATAAGCAGTGCTTGAATTATTTGGTTTCGGTTGTTTTCTATTAGACTAT GGTGAGCTCAGGTGATTGATACTCCTGAT 13 (0.000396%)

ATAGAGGAGACACCTGCTAGGTGTAAGGAGAAGATGGTTAGGTCTACGGAGGCTCCAGGGTGGGAGTA GTTCCCTGCTAAGGGAGGGTAGACTGTTCAAC 7 (0.000213%)

ATAGATAGTTGGGTGGTTGGTGTAAATGAGTGAGGCAGGAGTCCGAGGAGGTTAGTTGTGGCAATAAAA ATGATTAAGGATACTAGTATAAGAGATCAGG 23 (0.000700%)

ATAGGGATAGTACAAGGAAGGGGTAGGCTATGTGTTTTGTCAGGGGGTTGAGAATGAGTGTGAGGCGTA TTATACCATAGCCGCCTAGTTTTAAGAGTAC 4 (0.000122%)

ATAGTACAAGGAAGGGGTAGGCTATGTGTTTTGTCAGGGGGTTGAGAATGAGTGTGAGGCGTATTATAC CATAGCCGCCTAGTTTTAAGAGTACTGCGGC 3 (0.000091%)

ATATTCATTTAGCCTTCTGAGCTTTCTGGGCAGACTTGGTGACCTTGCCAGCTCCAGCAGCCTTCTTGTCC ACTGCTTTGATGACACCCACCGCAACTGT 5 (0.000152%)

ATATTGCTAGGGTGGCGCTTCCAATTAGGTGCATGAGTAGGTGGCCTGCAGTAATGTTAGCGGTTAGGCG TACGGCCAGGGCTATTGGTTGAATGAGTAG 3 (0.000091%)

ATCACCTGAGCAGTGAAGCCAGCTGCTTCCATTGGTGGGTCATTTTTGCTGTCACCAGCAACGTTGCCAC GACGAACATCCTTGACAGACACATTCTTGA 3 (0.000091%)

ATCATGTTTTTGATAAAGTCTCTGTGTCCTGGGGCATCAATGATAGTCACATAGTACTTGCTGGTCTCAA ATTTCCACAAGGAGATATCAATGGTGATAC 4 (0.000122%)

ATCCAAGACCCAGGCATACTTGAAGGAGCCCTTTCCCATCTCAGCAGCCTCCTTCTCAAATTTTTCAATG GTTCTTTTGTCGATGCCACCGCATTTATAG6 (0.000183%)

ATCGGGAGTACTACTCGATTGTCAACGTCAAGGAGTCGCAGGTCGCCTGGTTCTAGGAATAATGGGGGA AGTATGTAGGAGTTGAAGATTAGTCCGCCGT 3 (0.000091%)

ATGAAATTGATGGCCCCTAAGATAGAGGAGACACCTGCTAGGTGTAAGGAGAAGATGGTTAGGTCTACG GAGGCTCCAGGGTGGGAGTAGTTCCCTGCTA 309 (0.009406%)

ATGAATCCTAGGGCTCAGAGCACTGCAGCAGATCATTTCATATTGCTTCCGTGGAGTGTGGCGAGTCAGC

TAAATACTTTGACGCCGGTGGGGATAGCGA 5 (0.000152%)

ATGAGTAGGCTGATGGTTTCGATAATAACTAGTATGGGGATAAGGGGTGTAGGTGTGCCTTGTGGTAAG
AAGTGGGCTAGGGCATTTTTAATCTTAGAGC 3 (0.000091%)

ATGAGTATTTGTCTAAAACATGTCTTCTTTGTAGCAGCTAGGCCCTGCCACCACTGTGCTTGGCTGAGTTC
ACAAATCTGTTGTAACCTGTAGCTTCCCT 4 (0.000122%)

ATGCAGTCCAGAGCCTCAAGCAGCGTGGTTCCACTGGCATTGCCATCCTTACGGGTGACTTTCCATCCCT
TGAACCAAGGCATGTTAGCACTTGGCTCCA 21 (0.000639%)

ATGCTACTTGTCCAATGATGGTAAAAGGGTAGCTTACTGGTTGTCCTCCGATTCAGGTTAGAATGAGGAG
GTCTGCGGCTAGGAGTCAATAAAGTGATTG 5 (0.000152%)

ATGCTCTCGGGTCTGCCCATTCTTGGAGATACCAGCTTCAAATTCACCAACACCAGCAGCAACAATCAGG
ACAGCACAGTCAGCCTGAGATGTCCCTGTA 9 (0.000274%)

ATGGACACCAGTTTTAGCCAACATAGCATAGTACTCTATTTCAGATTTCCTCAAAGCTGGGCAGTTGTTA
GCGAGAATGACCAATTTCGCTTTGCCTTGT 14 (0.000426%)

ATGGCCCCTAAGATAGAGGAGACACCTGCTAGGTGTAAGGAGAAGATGGTTAGGTCTACGGAGGCTCCA
GGGTGGGAGTAGTTCCCTGCTAAGGGAGGGT 5 (0.000152%)

ATGGCTAGGTTTATAGATAGTTGGGTGGTTGGTGTAAATGAGTGAGGCAGGAGTCCGAGGAGGTTAGTT
GTGGCAATAAAAATGATTAAGGATACTAGTA 9 (0.000274%)

ATGGGCTTGCCAGGAACCATATCAACAATGGCAGCATCACCAGACTTCAAGAATTTAGGGCCATCTTCC
AGCTTTTTACCAGAACGGCGATCAATCTTTT 5 (0.000152%)

ATGGGCTTTAGGGAGTCATAAGTGGAGTCCGTAAAGAGGTATCTTTACTATAAAAGCTATTGTGTAAGCT
AGTCATATTAAGTTGTTGGCTCAGGAGTTT 7 (0.000213%)

ATGGTTTGGGTTGGGTGGTGGAGAGCGCGTGTCATCTGCGGGTGGCACTGCCCACGGTGGGCGGGCGGG
CCTCTCTACTCGAAGGTGACCACGTTTAGAT 3 (0.000091%)

ATGTTTTTGATAAAGTCTCTGTGTCCTGGGGCATCAATGATAGTCACATAGTACTTGCTGGTCTCAAATTT
CCACAAGGAGATATCAATGGTGATACCAC 3 (0.000091%)

ATTAAAAAGTACTGATTTTAAAAACTAATAACTTAAAACTGCCACACGCAAAAAAGAAAACCAAAGTGG
TCCACAAAACATTCTCCTTTCCTTCTGAAGG 25 (0.000761%)

ATTAAGAGTGGGGTGGCAGGTATTAGGGATAATATTCATTTAGCCTTCTGAGCTTTCTGGGCAGACTTGG
TGACCTTGCCAGCTCCAGCAGCCTTCTTGT 9 (0.000274%)

ATTAGACTATGGTGAGCTCAGGTGATTGATACTCCTGATGCGAGTAATACGGATGTGTTTAGGAGTGGG
ACTTCTAGGGGATTTAGCGGGGTGATGCCTG 23 (0.000700%)

ATTAGGGATAATATTCATTTAGCCTTCTGAGCTTTCTGGGCAGACTTGGTGACCTTGCCAGCTCCAGCAG
CCTTCTTGTCCACTGCTTTGATGACACCCA42 (0.001278%)

ATTAGGTGCATGAGTAGGTGGCCTGCAGTAATGTTAGCGGTTAGGCGTACGGCCAGGGCTATTGGTTGA
ATGAGTAGGCTGATGGTTTCGATAATAACTA 80 (0.002435%)

ATTATACGAATGGGGGCTTCAATCGGGAGTACTACTCGATTGTCAACGTCAAGGAGTCGCAGGTCGCCT
GGTTCTAGGAATAATGGGGGAAGTATGTAGG 10 (0.000304%)

ATTATTTGGTTTCGGTTGTTTCTATTAGACTATGGTGAGCTCAGGTGATTGATACTCCTGATGCGAGTAA
TACGGATGTGTTTAGGAGTGGGACTTCTA 9 (0.000274%)

ATTCATTTAGCCTTCTGAGCTTTCTGGGCAGACTTGGTGACCTTGCCAGCTCCAGCAGCCTTCTTGTCCAC
TGCTTTGATGACACCCACCGCAACTGTCT 190 (0.005784%)

ATTCTCATGGTTTGGGTTGGGTGGTGGAGAGCGCGTGTCATCTGCGGGTGGCACTGCCCACGGTGGGCG
GGCGGGCCTCTCTACTCGAAGGTGACCACGT 4 (0.000122%)

ATTCTGTGACAAATTTTTGGTCAAGTTGTTTCCATTAAAAAGTACTGATTTTAAAAACTAATAACTTAAA
ACTGCCACACGCAAAAAAGAAAACCAAAGT 15 (0.000457%)

ATTGATGGCCCCTAAGATAGAGGAGACACCTGCTAGGTGTAAGGAGAAGATGGTTAGGTCTACGGAGGC
TCCAGGGTGGGAGTAGTTCCCTGCTAAGGGA 13 (0.000396%)

ATTGCATCTGTTTTTAAGCCTAATGTGGGGACAGCTCATGAGTGCAAGACGTCTTGTGATGTAATTATTA
TACGAATGGGGGCTTCAATCGGGAGTACTA 7 (0.000213%)

ATTGCCATCCTTACGGGTGACTTTCCATCCCTTGAACCAAGGCATGTTAGCACTTGGCTCCAGCATGTTGT
CACCATTCCAACCAGAAATTGGCACAAAT5 (0.000152%)

ATTGCTAGGGTGGCGCTTCCAATTAGGTGCATGAGTAGGTGGCCTGCAGTAATGTTAGCGGTTAGGCGTA
CGGCCAGGGCTATTGGTTGAATGAGTAGGC 8 (0.000244%)

ATTGGTGGGTCATTTTTGCTGTCACCAGCAACGTTGCCACGACGAACATCCTTGACAGACACATTCTTGA
CATTGAAGCCCACATTGTCCCCAGGAAGAG 5 (0.000152%)

ATTGGTTGAATGAGTAGGCTGATGGTTTCGATAATAACTAGTATGGGGATAAGGGGTGTAGGTGTGCCTT
GTGGTAAGAAGTGGGCTAGGGCATTTTTAA 7 (0.000213%)

ATTGTAATAAGCAGTGCTTGAATTATTTGGTTTCGGTTGTTTCTATTAGACTATGGTGAGCTCAGGTGAT
TGATACTCCTGATGCGAGTAATACGGATG 8 (0.000244%)

ATTGTCCCCAGGAAGAGCTTCACTCAAAGCTTCATGGTGCATTTCGACAGATTTTACTTCCGTTGTAACG
TTGACTGGAGCAAAGGTGACCACCATACCG 18 (0.000548%)

ATTTACGCCGATGAATATGATAGTGAAATGGATTTTGGCGTAGGTTTGGTCTAGGGTGTAGCCTGAGAAT
AGGGGAAATCAGTGAATGAAGCCTCCTATG 6 (0.000183%)

ATTTAGCCTTCTGAGCTTTCTGGGCAGACTTGGTGACCTTGCCAGCTCCAGCAGCCTTCTTGTCCACTGCT
TTGATGACACCCACCGCAACTGTCTGTCT 75 (0.002283%)

ATTTCCTCATATCTCTTCTGGCTGTAGGGTGGCTCAGTGGAATCCATTTTGTTAACACCGACAATTAGTTG
TTTCACACCCAGTGTGTAAGCCAGAAGGG31 (0.000944%)

ATTTGTCTAAAACATGTCTTCTTTGTAGCAGCTAGGCCCTGCCACCACTGTGCTTGGCTGAGTTCACAAAT
CTGTTGTAACCTGTAGCTTCCCTGTCACT 12 (0.000365%)

ATTTTAAAAACTAATAACTTAAAACTGCCACACGCAAAAAAGAAAACCAAAGTGGTCCACAAAACATTC
TCCTTTCCTTCTGAAGGTTTTACGATGCATT 9 (0.000274%)

ATTTTCTTAATGTAAGTGCTGACTTCCTTAACAATTTCCTCATATCTCTTCTGGCTGTAGGGTGGCTCAGT
GGAATCCATTTTGTTAACACCGACAATTA 20 (0.000609%)

ATTTTGTAGACATCCTGGAGAGGCAGGCGCAAGGGCTTGTCAGTTGGACGAGTTGGTGGTAGGATGCAG
TCCAGAGCCTCAAGCAGCGTGGTTCCACTGG 20 (0.000609%)

ATTTTTGCTGTCACCAGCAACGTTGCCACGACGAACATCCTTGACAGACACATTCTTGACATTGAAGCCC
ACATTGTCCCCAGGAAGAGCTTCACTCAAA 37 (0.001126%)

CAAAAAAGAAAACCAAAGTGGTCCACAAAACATTCTCCTTTCCTTCTGAAGGTTTTACGATGCATTGTTA
TCATTAACCAGTCTTTTACTACTAAACTTA49 (0.001492%)

CAAATTCTCATGGTTTGGGTTGGGTGGTGGAGAGCGCGTGTCATCTGCGGGTGGCACTGCCCACGGTGG
GCGGGCGGGCCTCTCTACTCGAAGGTGACCA 10 (0.000304%)

CAACAATCAGGACAGCACAGTCAGCCTGAGATGTCCCTGTAATCATGTTTTGATAAAGTCTCTGTGTCC
TGGGGCATCAATGATAGTCACATAGTACTT 32 (0.000974%)

CAACACACATGGGCTTGCCAGGAACCATATCAACAATGGCAGCATCACCAGACTTCAAGAATTTAGGGC
CATCTTCCAGCTTTTTACCAGAACGGCGATC 4 (0.000122%)

CAACATTGTTTCACACATACATCAAACAGGCCAAAAAAAATAAACAGCAACTTCATAGACAAAAAAGG
AAAAAAAAAGAAACCTTTTATCTTTGGCCTTT 331 (0.010076%)

CAAGACCCAGGCATACTTGAAGGAGCCCTTTCCCATCTCAGCAGCCTCCTTCTCAAATTTTTCAATGGTT
CTTTTGTCGATGCCACCGCATTTATAGATC5 (0.000152%)

CAAGCAGCGTGGTTCCACTGGCATTGCCATCCTTACGGGTGACTTTCCATCCCTTGAACCAAGGCATGTT
AGCACTTGGCTCCAGCATGTTGTCACCATT 10 (0.000304%)

CAAGGAAGGGGTAGGCTATGTGTTTTGTCAGGGGGTTGAGAATGAGTGTGAGGCGTATTATACCATAGC
CGCCTAGTTTTAAGAGTACTGCGGCAAGTAC 14 (0.000426%)

CAAGGGCTTGTCAGTTGGACGAGTTGGTGGTAGGATGCAGTCCAGAGCCTCAAGCAGCGTGGTTCCACT
GGCATTGCCATCCTTACGGGTGACTTTCCAT 11 (0.000335%)

CAATCGGGAGTACTACTCGATTGTCAACGTCAAGGAGTCGCAGGTCGCCTGGTTCTAGGAATAATGGGG
GAAGTATGTAGGAGTTGAAGATTAGTCCGCC 3 (0.000091%)

CAATTTCCTCATATCTCTTCTGGCTGTAGGGTGGCTCAGTGGAATCCATTTTGTTAACACCGACAATTAGT
TGTTTCACACCCAGTGTGTAAGCCAGAAG 19 (0.000578%)

CAATTTTCTTAATGTAAGTGCTGACTTCCTTAACAATTTCCTCATATCTCTTCTGGCTGTAGGGTGGCTCA
GTGGAATCCATTTTGTTAACACCGACAAT 36 (0.001096%)

CAATTTTGTAGACATCCTGGAGAGGCAGGCGCAAGGGCTTGTCAGTTGGACGAGTTGGTGGTAGGATGC
AGTCCAGAGCCTCAAGCAGCGTGGTTCCACT 476 (0.014489%)

CACACATGGGCTTGCCAGGAACCATATCAACAATGGCAGCATCACCAGACTTCAAGAATTTAGGGCCAT
CTTCCAGCTTTTTACCAGAACGGCGATCAAT 8 (0.000244%)

CACACCCAGTGTGTAAGCCAGAAGGGCATGCTCTCGGGTCTGCCCATTCTTGGAGATACCAGCTTCAAAT
TCACCAACACCAGCAGCAACAATCAGGACA 5 (0.000152%)

CACACGCAAAAAAGAAAACCAAAGTGGTCCACAAAACATTCTCCTTTCCTTCTGAAGGTTTTACGATGC
ATTGTTATCATTAACCAGTCTTTTACTACTA 34 (0.001035%)

CACACTCCTTTGCTACTGGTCCTGTAATGGCAGAACCTTTCATCTCGCCTTTATTGTTCACTATGACTCCT
GCATTATCTTCAAAATAAAGAAACACGCC4 (0.000122%)

CACATCTACAAAATGCCAGTATCAGGCGGCGGCTTCGAAGCCAAAGTGATGTTTGGATGTAAAGTGAAA
TATTAGTTGGCGGATGAAGCAGATAGTGAGG 394 (0.011993%)

CACATGAGTATTTGTCTAAAACATGTCTTCTTTGTAGCAGCTAGGCCCTGCCACCACTGTGCTTGGCTGA
GTTCACAAATCTGTTGTAACCTGTAGCTTC 7 (0.000213%)

CACATGGGCTTGCCAGGAACCATATCAACAATGGCAGCATCACCAGACTTCAAGAATTTAGGGCCATCT
TCCAGCTTTTTACCAGAACGGCGATCAATCT 3 (0.000091%)

CACATTGTCCCCAGGAAGAGCTTCACTCAAAGCTTCATGGTGCATTTCGACAGATTTTACTTCCGTTGTA

ACGTTGACTGGAGCAAAGGTGACCACCATA 29 (0.000883%)

CACCACTGATTAAGAGTGGGGTGGCAGGTATTAGGGATAATATTCATTTAGCCTTCTGAGCTTTCTGGGC
AGACTTGGTGACCTTGCCAGCTCCAGCAGC 9 (0.000274%)

CACCAGTTTTAGCCAACATAGCATAGTACTCTATTTCAGATTTCCTCAAAGCTGGGCAGTTGTTAGCGAG
AATGACCAATTTCGCTTTGCCTTGTCTGAT3 (0.000091%)

CACCCAGTGTGTAAGCCAGAAGGGCATGCTCTCGGGTCTGCCCATTCTTGGAGATACCAGCTTCAAATTC
ACCAACACCAGCAGCAACAATCAGGACAGC 6 (0.000183%)

CACCTGAGCAGTGAAGCCAGCTGCTTCCATTGGTGGGTCATTTTTGCTGTCACCAGCAACGTTGCCACGA
CGAACATCCTTGACAGACACATTCTTGACA 8 (0.000244%)

CACGCAAAAAAGAAAACCAAAGTGGTCCACAAAACATTCTCCTTTCCTTCTGAAGGTTTTACGATGCATT
GTTATCATTAACCAGTCTTTTACTACTAAA25 (0.000761%)

CACTCAAAGCTTCATGGTGCATTTCGACAGATTTTACTTCCGTTGTAACGTTGACTGGAGCAAAGGTGAC
CACCATACCGGGTTTGAGAACACCAGTCTC 3 (0.000091%)

CACTCCTTTGCTACTGGTCCTGTAATGGCAGAACCTTTCATCTCGCCTTTATTGTTCACTATGACTCCTGC
ATTATCTTCAAAATAAAGAAACACGCCAT 8 (0.000244%)

CACTGATTAAGAGTGGGGTGGCAGGTATTAGGGATAATATTCATTTAGCCTTCTGAGCTTTCTGGGCAGA
CTTGGTGACCTTGCCAGCTCCAGCAGCCTT 8 (0.000244%)

CACTGCAGCAGATCATTTCATATTGCTTCCGTGGAGTGTGGCGAGTCAGCTAAATACTTTGACGCCGGTG
GGGATAGCGATGATTATGGTAGCGGAGGTG 3 (0.000091%)

CAGAACTACTGCCTTCACCATGAAGCTCCATGAGCTTTCCCAATTCAAACTTGGGCTTCTTCAGCATTTTT
ACTTTTCTAACGAAGACATCATGGAGAGG 8 (0.000244%)

CAGAAGGGCATGCTCTCGGGTCTGCCCATTCTTGGAGATACCAGCTTCAAATTCACCAACACCAGCAGC
AACAATCAGGACAGCACAGTCAGCCTGAGAT 42 (0.001278%)

CAGACTTGGTGACCTTGCCAGCTCCAGCAGCCTTCTTGTCCACTGCTTTGATGACACCCACCGCAACTGT
CTGTCTCATATCACGAACAGCAAAGCGACC 31 (0.000944%)

CAGAGCACTGCAGCAGATCATTTCATATTGCTTCCGTGGAGTGTGGCGAGTCAGCTAAATACTTTGACGC
CGGTGGGGATAGCGATGATTATGGTAGCGG 9 (0.000274%)

CAGATCATTTCATATTGCTTCCGTGGAGTGTGGCGAGTCAGCTAAATACTTTGACGCCGGTGGGGATAGC
GATGATTATGGTAGCGGAGGTGAAATATGC 5 (0.000152%)

CAGCAACAATCAGGACAGCACAGTCAGCCTGAGATGTCCCTGTAATCATGTTTTTGATAAAGTCTCTGTG
TCCTGGGGCATCAATGATAGTCACATAGTA 16 (0.000487%)

CAGCAGATCATTTCATATTGCTTCCGTGGAGTGTGGCGAGTCAGCTAAATACTTTGACGCCGGTGGGGAT
AGCGATGATTATGGTAGCGGAGGTGAAATA 19 (0.000578%)

CAGCGTGGTTCCACTGGCATTGCCATCCTTACGGGTGACTTTCCATCCCTTGAACCAAGGCATGTTAGCA
CTTGGCTCCAGCATGTTGTCACCATTCCAA 7 (0.000213%)

CAGCTCCAGCAGCCTTCTTGTCCACTGCTTTGATGACACCCACCGCAACTGTCTGTCTCATATCACGAAC
AGCAAAGCGACCCAAAGGTGGATAGTCTGA 8 (0.000244%)

CAGCTGCTTCCATTGGTGGGTCATTTTTGCTGTCACCAGCAACGTTGCCACGACGAACATCCTTGACAGA
CACATTCTTGACATTGAAGCCCACATTGTC 15 (0.000457%)

CAGGAAGAGCTTCACTCAAAGCTTCATGGTGCATTTCGACAGATTTTACTTCCGTTGTAACGTTGACTGG
AGCAAAGGTGACCACCATACCGGGTTTGAG 42 (0.001278%)

CAGGATAATCACCTGAGCAGTGAAGCCAGCTGCTTCCATTGGTGGGTCATTTTTGCTGTCACCAGCAACG
TTGCCACGACGAACATCCTTGACAGACACA 1110 (0.033788%)

CAGGCCACCTACGGTGAAAAGAAAGATGAATCCTAGGGCTCAGAGCACTGCAGCAGATCATTTCATATT
GCTTCCGTGGAGTGTGGCGAGTCAGCTAAAT 17 (0.000517%)

CAGGCGCAAGGGCTTGTCAGTTGGACGAGTTGGTGGTAGGATGCAGTCCAGAGCCTCAAGCAGCGTGGT
TCCACTGGCATTGCCATCCTTACGGGTGACT 24 (0.000731%)

CAGGCTTATGCGGAGGAGAATGTTTTCATGTTACTTATACTAACATTAGTTCTTCTATAGGGTGATAGAT
TGGTCCAATTGGGTGTGAGGAGTTCAGTTA 19 (0.000578%)

CAGGGCTATTGGTTGAATGAGTAGGCTGATGGTTTCGATAATAACTAGTATGGGGATAAGGGGTGTAGG
TGTGCCTTGTGGTAAGAAGTGGGCTAGGGCA 30 (0.000913%)

CAGGTACCTTTCTCTTTGGCTTCTTTCTTTTTCTGATCATTTTCCTTCACACGTTTCAGGAAGCTATCTCGG
CTCTTAGAGTGCTTAATGTGCTCAATAC 7 (0.000213%)

CAGGTATTAGGGATAATATTCATTTAGCCTTCTGAGCTTTCTGGGCAGACTTGGTGACCTTGCCAGCTCC
AGCAGCCTTCTTGTCCACTGCTTTGATGAC 147 (0.004475%)

CAGGTCCCGGTATTCCCGGTACATGTTGTGGGTGCCGCTCCGGGAGTCATAGCGCAGCCAGATCCCGAA
GTTCTTCACCCGCAGGGGGGACTTCTCAAAC 11 (0.000335%)

CAGTAAAATTGTAATAAGCAGTGCTTGAATTATTTGGTTTCGGTTGTTTTCTATTAGACTATGGTGAGCTC
AGGTGATTGATACTCCTGATGCGAGTAAT 6 (0.000183%)

CAGTAATGTTAGCGGTTAGGCGTACGGCCAGGGCTATTGGTTGAATGAGTAGGCTGATGGTTTCGATAAT
AACTAGTATGGGGATAAGGGGTGTAGGTGT 41 (0.001248%)

CAGTCAGGCCACCTACGGTGAAAAGAAAGATGAATCCTAGGGCTCAGAGCACTGCAGCAGATCATTTCA
TATTGCTTCCGTGGAGTGTGGCGAGTCAGCT 3 (0.000091%)

CAGTCCAGAGCCTCAAGCAGCGTGGTTCCACTGGCATTGCCATCCTTACGGGTGACTTTCCATCCCTTGA
ACCAAGGCATGTTAGCACTTGGCTCCAGCA 4 (0.000122%)

CAGTGAAGCCAGCTGCTTCCATTGGTGGGTCATTTTTGCTGTCACCAGCAACGTTGCCACGACGAACATC
CTTGACAGACACATTCTTGACATTGAAGCC 7 (0.000213%)

CAGTGTGTAAGCCAGAAGGGCATGCTCTCGGGTCTGCCCATTCTTGGAGATACCAGCTTCAAATTCACCA
ACACCAGCAGCAACAATCAGGACAGCACAG 6 (0.000183%)

CAGTGTTTTAATCTGACGCAGGCTTATGCGGAGGAGAATGTTTTCATGTTACTTATACTAACATTAGTTCT
TCTATAGGGTGATAGATTGGTCCAATTGG 3 (0.000091%)

CAGTTCAGTGTTTTAATCTGACGCAGGCTTATGCGGAGGAGAATGTTTTCATGTTACTTATACTAACATT
AGTTCTTCTATAGGGTGATAGATTGGTCCA 3 (0.000091%)

CATAAGTGGAGTCCGTAAAGAGGTATCTTTACTATAAAAGCTATTGTGTAAGCTAGTCATATTAAGTTGT
TGGCTCAGGAGTTTGATAGTTCTTGGGCAG 12 (0.000365%)

CATAGGGATAGTACAAGGAAGGGGTAGGCTATGTGTTTTGTCAGGGGGTTGAGAATGAGTGTGAGGCGT
ATTATACCATAGCCGCCTAGTTTTAAGAGTA 5 (0.000152%)

CATATCTCTTCTGGCTGTAGGGTGGCTCAGTGGAATCCATTTTGTTAACACCGACAATTAGTTGTTTCACA
CCCAGTGTGTAAGCCAGAAGGGCATGCTC21 (0.000639%)

CATCCTGGAGAGGCAGGCGCAAGGGCTTGTCAGTTGGACGAGTTGGTGGTAGGATGCAGTCCAGAGCCT CAAGCAGCGTGGTTCCACTGGCATTGCCATC 6 (0.000183%)

CATGCTCTCGGGTCTGCCCATTCTTGGAGATACCAGCTTCAAATTCACCAACACCAGCAGCAACAATCAG GACAGCACAGTCAGCCTGAGATGTCCCTGT 5 (0.000152%)

CATGGCTAGGTTTATAGATAGTTGGGTGGTTGGTGTAAATGAGTGAGGCAGGAGTCCGAGGAGGTTAGT TGTGGCAATAAAAATGATTAAGGATACTAGT 24 (0.000731%)

CATGGGCTTGCCAGGAACCATATCAACAATGGCAGCATCACCAGACTTCAAGAATTTAGGGCCATCTTC CAGCTTTTTACCAGAACGGCGATCAATCTTT 7 (0.000213%)

CATGGGCTTTAGGGAGTCATAAGTGGAGTCCGTAAAGAGGTATCTTTACTATAAAAGCTATTGTGTAAGC TAGTCATATTAAGTTGTTGGCTCAGGAGTT 3 (0.000091%)

CATGGTTTGGGTTGGGTGGTGGAGAGCGCGTGTCATCTGCGGGTGGCACTGCCCACGGTGGGCGGGCGG GCCTCTCTACTCGAAGGTGACCACGTTTAGA 11 (0.000335%)

CATGTTTTTGATAAAGTCTCTGTGTCCTGGGGCATCAATGATAGTCACATAGTACTTGCTGGTCTCAAATT TCCACAAGGAGATATCAATGGTGATACCA9 (0.000274%)

CATTAAAAAGTACTGATTTTAAAAACTAATAACTTAAAACTGCCACACGCAAAAAAGAAAACCAAAGTG GTCCACAAAACATTCTCCTTTCCTTCTGAAG 4 (0.000122%)

CATTGCCATCCTTACGGGTGACTTTCCATCCCTTGAACCAAGGCATGTTAGCACTTGGCTCCAGCATGTT GTCACCATTCCAACCAGAAATTGGCACAAA 7 (0.000213%)

CATTGTCCCCAGGAAGAGCTTCACTCAAAGCTTCATGGTGCATTTCGACAGATTTTACTTCCGTTGTAAC GTTGACTGGAGCAAAGGTGACCACCATACC 14 (0.000426%)

CATTTAGCCTTCTGAGCTTTCTGGGCAGACTTGGTGACCTTGCCAGCTCCAGCAGCCTTCTTGTCCACTGC TTTGATGACACCCACCGCAACTGTCTGTC 12 (0.000365%)

CATTTTTGCTGTCACCAGCAACGTTGCCACGACGAACATCCTTGACAGACACATTCTTGACATTGAAGCC CACATTGTCCCCAGGAAGAGCTTCACTCAA 51 (0.001552%)

CCAAAGTGGTCCACAAAACATTCTCCTTTCCTTCTGAAGGTTTACGATGCATTGTTATCATTAACCAGTC TTTTACTACTAAACTTAAATGGCCAATTG 8 (0.000244%)

CCAAGACCCAGGCATACTTGAAGGAGCCCTTTCCCATCTCAGCAGCCTCCTTCTCAAATTTTTCAATGGT TCTTTTGTCGATGCCACCGCATTTATAGAT 16 (0.000487%)

CCAATTTTCTTAATGTAAGTGCTGACTTCCTTAACAATTTCCTCATATCTCTTCTGGCTGTAGGGTGGCTC AGTGGAATCCATTTTGTTAACACCGACAA 8 (0.000244%)

CCACACGCAAAAAAGAAAACCAAAGTGGTCCACAAAACATTCTCCTTTCCTTCTGAAGGTTTTACGATGC ATTGTTATCATTAACCAGTCTTTTACTACT 40 (0.001218%)

CCACATTGTCCCCAGGAAGAGCTTCACTCAAAGCTTCATGGTGCATTTCGACAGATTTTACTTCCGTTGT AACGTTGACTGGAGCAAAGGTGACCACCAT 563 (0.017138%)

CCACCACTGATTAAGAGTGGGGTGGCAGGTATTAGGGATAATATTCATTTAGCCTTCTGAGCTTTCTGGG CAGACTTGGTGACCTTGCCAGCTCCAGCAG 7 (0.000213%)

CCAGAACTACTGCCTTCACCATGAAGCTCCATGAGCTTTCCCAATTCAAACTTGGGCTTCTTCAGCATTTT TACTTTTCTAACGAAGACATCATGGAGAG 5 (0.000152%)

CCAGAAGGGCATGCTCTCGGGTCTGCCCATTCTTGGAGATACCAGCTTCAAATTCACCAACACCAGCAGC

AACAATCAGGACAGCACAGTCAGCCTGAGA 12 (0.000365%)

CCAGCTCCAGCAGCCTTCTTGTCCACTGCTTTGATGACACCCACCGCAACTGTCTGTCTCATATCACGAA
CAGCAAAGCGACCCAAAGGTGGATAGTCTG 3 (0.000091%)

CCAGCTGCTTCCATTGGTGGGTCATTTTTGCTGTCACCAGCAACGTTGCCACGACGAACATCCTTGACAG
ACACATTCTTGACATTGAAGCCCACATTGT 8 (0.000244%)

CCAGGAAGAGCTTCACTCAAAGCTTCATGGTGCATTTCGACAGATTTTACTTCCGTTGTAACGTTGACTG
GAGCAAAGGTGACCACCATACCGGGTTTGA 69 (0.002100%)

CCAGGGCTATTGGTTGAATGAGTAGGCTGATGGTTTCGATAATAACTAGTATGGGGATAAGGGGTGTAG
GTGTGCCTTGTGGTAAGAAGTGGGCTAGGGC 7 (0.000213%)

CCAGGTACCTTTCTCTTTGGCTTCTTTCTTTTTCTGATCATTTTCCTTCACACGTTTCAGGAAGCTATCTCG
GCTCTTAGAGTGCTTAATGTGCTCAATA 3 (0.000091%)

CCAGTAAAATTGTAATAAGCAGTGCTTGAATTATTTGGTTTCGGTTGTTTTCTATTAGACTATGGTGAGCT
CAGGTGATTGATACTCCTGATGCGAGTAA 3 (0.000091%)

CCAGTCAGGCCACCTACGGTGAAAAGAAAGATGAATCCTAGGGCTCAGAGCACTGCAGCAGATCATTTC
ATATTGCTTCCGTGGAGTGTGGCGAGTCAGC 4 (0.000122%)

CCAGTGTGTAAGCCAGAAGGGCATGCTCTCGGGTCTGCCCATTCTTGGAGATACCAGCTTCAAATTCACC
AACACCAGCAGCAACAATCAGGACAGCACA 30 (0.000913%)

CCAGTGTTACTTTAATTGGACTGCCTTCGTAATTCATTGCCTCTGCTTCAACAATGTGCAACTCATCCTTT
GCACCAGCCCCTAAACTGACCGTTCTTAA 4 (0.000122%)

CCAGTTTTAGCCAACATAGCATAGTACTCTATTTCAGATTTCCTCAAAGCTGGGCAGTTGTTAGCGAGAA
TGACCAATTTCGCTTTGCCTTGTCTGATCA 9 (0.000274%)

CCATACACATGAGTATTTGTCTAAAACATGTCTTCTTTGTAGCAGCTAGGCCCTGCCACCACTGTGCTTG
GCTGAGTTCACAAATCTGTTGTAACCTGTA 4 (0.000122%)

CCATGGCTAGGTTTATAGATAGTTGGGTGGTTGGTGTAAATGAGTGAGGCAGGAGTCCGAGGAGGTTAG
TTGTGGCAATAAAAATGATTAAGGATACTAG 42 (0.001278%)

CCATTAAAAAGTACTGATTTTAAAAACTAATAACTTAAAACTGCCACACGCAAAAAAGAAAACCAAAGT
GGTCCACAAAACATTCTCCTTTCCTTCTGAA 12 (0.000365%)

CCATTGGTGGGTCATTTTTGCTGTCACCAGCAACGTTGCCACGACGAACATCCTTGACAGACACATTCTT
GACATTGAAGCCCACATTGTCCCCAGGAAG 5 (0.000152%)

CCCAGGAAGAGCTTCACTCAAAGCTTCATGGTGCATTTCGACAGATTTTACTTCCGTTGTAACGTTGACT
GGAGCAAAGGTGACCACCATACCGGGTTTG 89 (0.002709%)

CCCAGGTACCTTTCTCTTTGGCTTCTTTCTTTTTCTGATCATTTTCCTTCACACGTTTCAGGAAGCTATCTC
GGCTCTTAGAGTGCTTAATGTGCTCAAT 10 (0.000304%)

CCCAGTAAAATTGTAATAAGCAGTGCTTGAATTATTTGGTTTCGGTTGTTTTCTATTAGACTATGGTGAGC
TCAGGTGATTGATACTCCTGATGCGAGTA 15 (0.000457%)

CCCAGTGTGTAAGCCAGAAGGGCATGCTCTCGGGTCTGCCCATTCTTGGAGATACCAGCTTCAAATTCAC
CAACACCAGCAGCAACAATCAGGACAGCAC 9 (0.000274%)

CCCATACACATGAGTATTTGTCTAAAACATGTCTTCTTTGTAGCAGCTAGGCCCTGCCACCACTGTGCTTG
GCTGAGTTCACAAATCTGTTGTAACCTGT 14 (0.000426%)

CCCCAGGAAGAGCTTCACTCAAAGCTTCATGGTGCATTTCGACAGATTTTACTTCCGTTGTAACGTTGAC
TGGAGCAAAGGTGACCACCATACCGGGTTT 25 (0.000761%)

CCCCTGTTGCAAATTCTCATGGTTTGGGTTGGGTGGTGGAGAGCGCGTGTCATCTGCGGGTGGCACTGCC
CACGGTGGGCGGGCGGGCCTCTCTACTCGA 7 (0.000213%)

CCCCTTTCATTTTATAATAGAAAACCTTGGACTCGCCAGTGTTAGCTGCTGGAATGAGGTGTTTGTCCAG
TACATCCAGAATGTCACAACAGATTAACTT 3 (0.000091%)

CCCGGTATTCCCGGTACATGTTGTGGGTGCCGCTCCGGGAGTCATAGCGCAGCCAGATCCCGAAGTTCTT
CACCCGCAGGGGGGACTTCTCAAACACCTG 5 (0.000152%)

CCCTGTTGCAAATTCTCATGGTTTGGGTTGGGTGGTGGAGAGCGCGTGTCATCTGCGGGTGGCACTGCCC
ACGGTGGGCGGGCGGGCCTCTCTACTCGAA 23 (0.000700%)

CCGAAGCCTGGTAGGATAAGAATATAAACTTCAGGGTGACCGAAAAATCAGAATAGGTGTTGGTATAGA
ATGGGGTCTCCTCCTCCGGCGGGGTCGAAGA 24 (0.000731%)

CCGCGGGGCTCCCCGCCGCCTCGATGCGGCGGCTTGGGGTGGCCGCCAGGCGTACTTAGGCCCGGGCCG
CCACCTGGCTTCGGCCCGCCAGGCATTTTGC 19 (0.000578%)

CCGGTATTCCCGGTACATGTTGTGGGTGCCGCTCCGGGAGTCATAGCGCAGCCAGATCCCGAAGTTCTTC
ACCCGCAGGGGGGACTTCTCAAACACCTGC 12 (0.000365%)

CCGTTCTTCCACCACTGATTAAGAGTGGGGTGGCAGGTATTAGGGATAATATTCATTTAGCCTTCTGAGC
TTTCTGGGCAGACTTGGTGACCTTGCCAGC 35 (0.001065%)

CCTACGGTGAAAAGAAAGATGAATCCTAGGGCTCAGAGCACTGCAGCAGATCATTTCATATTGCTTCCG
TGGAGTGTGGCGAGTCAGCTAAATACTTTGA 8 (0.000244%)

CCTAGGGCTCAGAGCACTGCAGCAGATCATTTCATATTGCTTCCGTGGAGTGTGGCGAGTCAGCTAAATA
CTTTGACGCCGGTGGGGATAGCGATGATTA 11 (0.000335%)

CCTCAAGCAGCGTGGTTCCACTGGCATTGCCATCCTTACGGGTGACTTTCCATCCCTTGAACCAAGGCAT
GTTAGCACTTGGCTCCAGCATGTTGTCACC 26 (0.000791%)

CCTCATATCTCTTCTGGCTGTAGGGTGGCTCAGTGGAATCCATTTTGTTAACACCGACAATTAGTTGTTTC
ACACCCAGTGTGTAAGCCAGAAGGGCATG 35 (0.001065%)

CCTCCCCTGTTGCAAATTCTCATGGTTTGGGTTGGGTGGTGGAGAGCGCGTGTCATCTGCGGGTGGCACT
GCCCACGGTGGGCGGGCGGGCCTCTCTACT 4 (0.000122%)

CCTCTCCTGCTAAGCTTTGTTTCCTAATTAAAATCTTCTGCCACTGCCATAGCTACTGCTGCTGCTGGAAC
CGCCATAGCCACCTTGGTTTCGTGGTTTT 5 (0.000152%)

CCTGAGCAGTGAAGCCAGCTGCTTCCATTGGTGGGTCATTTTTGCTGTCACCAGCAACGTTGCCACGACG
AACATCCTTGACAGACACATTCTTGACATT 5 (0.000152%)

CCTGCAGTAATGTTAGCGGTTAGGCGTACGGCCAGGGCTATTGGTTGAATGAGTAGGCTGATGGTTTCGA
TAATAACTAGTATGGGGATAAGGGGTGTAG 5 (0.000152%)

CCTGGAGAGGCAGGCGCAAGGGCTTGTCAGTTGGACGAGTTGGTGGTAGGATGCAGTCCAGAGCCTCAA
GCAGCGTGGTTCCACTGGCATTGCCATCCTT 24 (0.000731%)

CCTGTGGCAAATTCTGCCAGATACCTGTGGTAGTCCCCTTTCATTTTATAATAGAAAACCTTGGACTCGC
CAGTGTTAGCTGCTGGAATGAGGTGTTTGT 60 (0.001826%)

CCTGTTGCAAATTCTCATGGTTTGGGTTGGGTGGTGGAGAGCGCGTGTCATCTGCGGGTGGCACTGCCCA
CGGTGGGCGGGCGGGCCTCTCTACTCGAAG 16 (0.000487%)

CCTTAACAATTTCCTCATATCTCTTCTGGCTGTAGGGTGGCTCAGTGGAATCCATTTTGTTAACACCGACA ATTAGTTGTTTCACACCCAGTGTGTAAGC 11 (0.000335%)

CCTTCTGAGCTTTCTGGGCAGACTTGGTGACCTTGCCAGCTCCAGCAGCCTTCTTGTCCACTGCTTTGATG ACACCCACCGCAACTGTCTGTCTCATATC 4 (0.000122%)

CCTTGCCAGCTCCAGCAGCCTTCTTGTCCACTGCTTTGATGACACCCACCGCAACTGTCTGTCTCATATCA CGAACAGCAAAGCGACCCAAAGGTGGATA 10 (0.000304%)

CCTTTCTCTTTGGCTTCTTTCTTTTTCTGATCATTTTCCTTCACACGTTTCAGGAAGCTATCTCGGCTCTTA GAGTGCTTAATGTGCTCAATACGCACAT 6 (0.000183%)

CGAAGCCTGGTAGGATAAGAATATAAACTTCAGGGTGACCGAAAAATCAGAATAGGTGTTGGTATAGAA TGGGGTCTCCTCCTCCGGCGGGGTCGAAGAA 14 (0.000426%)

CGAAGTACTCTGAGGCTTGTAGGAGGGTAAAATAGAGACCCAGTAAAATTGTAATAAGCAGTGCTTGAA TTATTTGGTTTCGGTTGTTTCTATTAGACT 8 (0.000244%)

CGAATGGGGGCTTCAATCGGGAGTACTACTCGATTGTCAACGTCAAGGAGTCGCAGGTCGCCTGGTTCT AGGAATAATGGGGGAAGTATGTAGGAGTTGA 30 (0.000913%)

CGACCCAAAGGTGGATAGTCTGAGAAGCTCTCAACACACATGGGCTTGCCAGGAACCATATCAACAATG GCAGCATCACCAGACTTCAAGAATTTAGGGC 781 (0.023774%)

CGAGTTGGTGGTAGGATGCAGTCCAGAGCCTCAAGCAGCGTGGTTCCACTGGCATTGCCATCCTTACGG GTGACTTTCCATCCCTTGAACCAAGGCATGT 17 (0.000517%)

CGCAAAAAAGAAAACCAAAGTGGTCCACAAAACATTCTCCTTTCCTTCTGAAGGTTTTACGATGCATTGT TATCATTAACCAGTCTTTTACTACTAAACT 33 (0.001005%)

CGCAAGGGCTTGTCAGTTGGACGAGTTGGTGGTAGGATGCAGTCCAGAGCCTCAAGCAGCGTGGTTCCA CTGGCATTGCCATCCTTACGGGTGACTTTCC 38 (0.001157%)

CGCAGGCTTATGCGGAGGAGAATGTTTTCATGTTACTTATACTAACATTAGTTCTTCTATAGGGTGATAG ATTGGTCCAATTGGGTGTGAGGAGTTCAGT 19 (0.000578%)

CGGAGGAAAAGGTTGGGGAACAGCTAAATAGGTTGTTGTTGATTTGGTTAAAAAATAGTAGAGGGATGA TGCTAATAATTAGGCTGTGGGTGGTTGTGTT 19 (0.000578%)

CGGATGCTACTTGTCCAATGATGGTAAAAGGGTAGCTTACTGGTTGTCCTCCGATTCAGGTTAGAATGAG GAGGTCTGCGGCTAGGAGTCAATAAAGTGA 352 (0.010715%)

CGGCCAGGGCTATTGGTTGAATGAGTAGGCTGATGGTTTCGATAATAACTAGTATGGGGATAAGGGGTG TAGGTGTGCCTTGTGGTAAGAAGTGGGCTAG 6 (0.000183%)

CGGGAGTACTACTCGATTGTCAACGTCAAGGAGTCGCAGGTCGCCTGGTTCTAGGAATAATGGGGGAAG TATGTAGGAGTTGAAGATTAGTCCGCCGTAG 51 (0.001552%)

CGGTCTGTTAGTAGTATAGTGATGCCAGCAGCTAGGACTGGGAGAGATAGGAGAAGTAGGACTGCTGTG ATTAGGACGGATCAGACGAAGAGGGGCGTTT 5 (0.000152%)

CGGTGGTCAGGTCCCGGTATTCCCGGTACATGTTGTGGGTGCCGCTCCGGGAGTCATAGCGCAGCCAGAT CCCGAAGTTCTTCACCCGCAGGGGGGACTT 12 (0.000365%)

CGGTTAGGCGTACGGCCAGGGCTATTGGTTGAATGAGTAGGCTGATGGTTTCGATAATAACTAGTATGG GGATAAGGGGTGTAGGTGTGCCTTGTGGTAA 3 (0.000091%)

CGTACGGCCAGGGCTATTGGTTGAATGAGTAGGCTGATGGTTTCGATAATAACTAGTATGGGGATAAGG

GGTGTAGGTGTGCCTTGTGGTAAGAAGTGGG 15 (0.000457%)

CGTTCTTCCACCACTGATTAAGAGTGGGGTGGCAGGTATTAGGGATAATATTCATTTAGCCTTCTGAGCTTTCTGGGCAGACTTGGTGACCTTGCCAGCT 83 (0.002527%)

CTAAGATAGAGGAGACACCTGCTAGGTGTAAGGAGAAGATGGTTAGGTCTACGGAGGCTCCAGGGTGGGAGTAGTTCCCTGCTAAGGGAGGGTAGACTGT 20 (0.000609%)

CTAATAACTTAAAACTGCCACACGCAAAAAAGAAAACCAAAGTGGTCCACAAAACATTCTCCTTTCCTTCTGAAGGTTTTACGATGCATTGTTATCATTA 9 (0.000274%)

CTAATACAATGCCAGTCAGGCCACCTACGGTGAAAAGAAAGATGAATCCTAGGGCTCAGAGCACTGCAGCAGATCATTTCATATTGCTTCCGTGGAGTGT 13 (0.000396%)

CTACGGTGAAAAGAAAGATGAATCCTAGGGCTCAGAGCACTGCAGCAGATCATTTCATATTGCTTCCGTGGAGTGTGGCGAGTCAGCTAAATACTTTGAC 9 (0.000274%)

CTAGGGCTCAGAGCACTGCAGCAGATCATTTCATATTGCTTCCGTGGAGTGTGGCGAGTCAGCTAAATACTTTGACGCCGGTGGGGATAGCGATGATTAT 16 (0.000487%)

CTAGGGTGGCGCTTCCAATTAGGTGCATGAGTAGGTGGCCTGCAGTAATGTTAGCGGTTAGGCGTACGGCCAGGGCTATTGGTTGAATGAGTAGGCTGAT 4 (0.000122%)

CTAGGTTTATAGATAGTTGGGTGGTTGGTGTAAATGAGTGAGGCAGGAGTCCGAGGAGGTTAGTTGTGGCAATAAAAATGATTAAGGATACTAGTATAAG 60 (0.001826%)

CTATTAGACTATGGTGAGCTCAGGTGATTGATACTCCTGATGCGAGTAATACGGATGTGTTTAGGAGTGGGACTTCTAGGGGATTTAGCGGGGTGATGCC 8 (0.000244%)

CTCAAAGCTTCATGGTGCATTTCGACAGATTTTACTTCCGTTGTAACGTTGACTGGAGCAAAGGTGACCACCATACCGGGTTTGAGAACACCAGTCTCCA 13 (0.000396%)

CTCAACACACATGGGCTTGCCAGGAACCATATCAACAATGGCAGCATCACCAGACTTCAAGAATTTAGGGCCATCTTCCAGCTTTTTACCAGAACGGCGA 23 (0.000700%)

CTCAAGCAGCGTGGTTCCACTGGCATTGCCATCCTTACGGGTGACTTTCCATCCCTTGAACCAAGGCATGTTAGCACTTGGCTCCAGCATGTTGTCACCA 27 (0.000822%)

CTCAGAGCACTGCAGCAGATCATTTCATATTGCTTCCGTGGAGTGTGGCGAGTCAGCTAAATACTTTGACGCCGGTGGGGATAGCGATGATTATGGTAGC 48 (0.001461%)

CTCAGTGGAATCCATTTTGTTAACACCGACAATTAGTTGTTTCACACCCAGTGTGTAAGCCAGAAGGGCATGCTCTCGGGTCTGCCCATTCTTGGAGATA 31 (0.000944%)

CTCATAGGGATAGTACAAGGAAGGGGTAGGCTATGTGTTTTGTCAGGGGGTTGAGAATGAGTGTGAGGCGTATTATACCATAGCCGCCTAGTTTTAAGAG 285 (0.008675%)

CTCATATCTCTTCTGGCTGTAGGGTGGCTCAGTGGAATCCATTTTGTTAACACCGACAATTAGTTGTTTCACACCCAGTGTGTAAGCCAGAAGGGCATGC 95 (0.002892%)

CTCATGGTTTGGGTTGGGTGGTGGAGAGCGCGTGTCATCTGCGGGTGGCACTGCCCACGGTGGGCGGGCGGGCCTCTCTACTCGAAGGTGACCACGTTTA 14 (0.000426%)

CTCCCCTGTTGCAAATTCTCATGGTTTGGGTTGGGTGGTGGAGAGCGCGTGTCATCTGCGGGTGGCACTGCCCACGGTGGGCGGGCGGGCCTCTCTACTC 34 (0.001035%)

CTCCTTTGCTACTGGTCCTGTAATGGCAGAACCTTTCATCTCGCCTTTATTGTTCACTATGACTCCTGCATTATCTTCAAAATAAAGAAACACGCCATCT 10 (0.000304%)

CTCGAAGTACTCTGAGGCTTGTAGGAGGGTAAAATAGAGACCCAGTAAAATTGTAATAAGCAGTGCTTG
AATTATTTGGTTTCGGTTGTTTCTATTAGA 584 (0.017777%)

CTCGGGTCTGCCCATTCTTGGAGATACCAGCTTCAAATTCACCAACACCAGCAGCAACAATCAGGACAG
CACAGTCAGCCTGAGATGTCCCTGTAATCATGTTTTGATAAAGTCTCTGTGTCCTGGGGCATCAATGAT
AGTCACATAG 386 (0.017507%)

CTCTCAACACACATGGGCTTGCCAGGAACCATATCAACAATGGCAGCATCACCAGACTTCAAGAATTTA
GGGCCATCTTCCAGCTTTTTACCAGAACGGC 12 (0.000365%)

CTCTCGGGTCTGCCCATTCTTGGAGATACCAGCTTCAAATTCACCAACACCAGCAGCAACAATCAGGACA
GCACAGTCAGCCTGAGATGTCCCTGTAATC 37 (0.001126%)

CTCTTCTGGCTGTAGGGTGGCTCAGTGGAATCCATTTTGTTAACACCGACAATTAGTTGTTTCACACCCA
GTGTGTAAGCCAGAAGGGCATGCTCTCGGG 55 (0.001674%)

CTGACTTCCTTAACAATTTCCTCATATCTCTTCTGGCTGTAGGGTGGCTCAGTGGAATCCATTTTGTTAAC
ACCGACAATTAGTTGTTTCACACCCAGTG 36 (0.001096%)

CTGAGAAGCTCTCAACACACATGGGCTTGCCAGGAACCATATCAACAATGGCAGCATCACCAGACTTCA
AGAATTTAGGGCCATCTTCCAGCTTTTTACC 311 (0.009467%)

CTGAGCAGTGAAGCCAGCTGCTTCCATTGGTGGGTCATTTTTGCTGTCACCAGCAACGTTGCCACGACGA
ACATCCTTGACAGACACATTCTTGACATTG 6 (0.000183%)

CTGAGCTTTCTGGGCAGACTTGGTGACCTTGCCAGCTCCAGCAGCCTTCTTGTCCACTGCTTTGATGACAC
CCACCGCAACTGTCTGTCTCATATCACGA 24 (0.000731%)

CTGATTAAGAGTGGGGTGGCAGGTATTAGGGATAATATTCATTTAGCCTTCTGAGCTTTCTGGGCAGACT
TGGTGACCTTGCCAGCTCCAGCAGCCTTCT 16 (0.000487%)

CTGATTTTAAAAACTAATAACTTAAAACTGCCACACGCAAAAAAGAAAACCAAAGTGGTCCACAAAACA
TTCTCCTTTCCTTCTGAAGGTTTTACGATGC 86 (0.002618%)

CTGCACACTCCTTTGCTACTGGTCCTGTAATGGCAGAACCTTTCATCTCGCCTTTATTGTTCACTATGACT
CCTGCATTATCTTCAAAATAAAGAAACAC 259 (0.007884%)

CTGCAGCAGATCATTTCATATTGCTTCCGTGGAGTGTGGCGAGTCAGCTAAATACTTTGACGCCGGTGGG
GATAGCGATGATTATGGTAGCGGAGGTGAA 15 (0.000457%)

CTGCAGTAATGTTAGCGGTTAGGCGTACGGCCAGGGCTATTGGTTGAATGAGTAGGCTGATGGTTTCGAT
AATAACTAGTATGGGGATAAGGGGTGTAGG 54 (0.001644%)

CTGCCACACGCAAAAAAGAAAACCAAAGTGGTCCACAAAACATTCTCCTTTCCTTCTGAAGGTTTTACGA
TGCATTGTTATCATTAACCAGTCTTTTACT 30 (0.000913%)

CTGCTTCCATTGGTGGGTCATTTTTGCTGTCACCAGCAACGTTGCCACGACGAACATCCTTGACAGACAC
ATTCTTGACATTGAAGCCCACATTGTCCCC 3 (0.000091%)

CTGGAGAGGCAGGCGCAAGGGCTTGTCAGTTGGACGAGTTGGTGGTAGGATGCAGTCCAGAGCCTCAAG
CAGCGTGGTTCCACTGGCATTGCCATCCTTA 18 (0.000548%)

CTGGAGTGGTAAAAGGCTCAGAAAAATCCTGCGAAGAAAAAAACTTCTGAGGTAATAAATAGGATTATC
CCGTATCGAAGGCCTTTTTGGACAGGTGGTG 34 (0.001035%)

CTGGCTGTAGGGTGGCTCAGTGGAATCCATTTTGTTAACACCGACAATTAGTTGTTTCACACCCAGTGTG
TAAGCCAGAAGGGCATGCTCTCGGGTCTGC 32 (0.000974%)

CTGGGCAGACTTGGTGACCTTGCCAGCTCCAGCAGCCTTCTTGTCCACTGCTTTGATGACACCCACCGCA

ACTGTCTGTCTCATATCACGAACAGCAAAG 17 (0.000517%)

CTGGTCCTGTAATGGCAGAACCTTTCATCTCGCCTTTATTGTTCACTATGACTCCTGCATTATCTTCAAAA
TAAAGAAACACGCCATCTTTTCTACGGTA 3 (0.000091%)

CTGTAGGGTGGCTCAGTGGAATCCATTTTGTTAACACCGACAATTAGTTGTTTCACACCCAGTGTGTAAG
CCAGAAGGGCATGCTCTCGGGTCTGCCCAT 18 (0.000548%)

CTGTAGTGATGGACACCAGTTTTAGCCAACATAGCATAGTACTCTATTTCAGATTTCCTCAAAGCTGGGC
AGTTGTTAGCGAGAATGACCAATTTCGCTT 344 (0.010471%)

CTGTGACAAATTTTTGGTCAAGTTGTTTCCATTAAAAAGTACTGATTTTAAAAACTAATAACTTAAAACT
GCCACACGCAAAAAAGAAAACCAAAGTGGT 39 (0.001187%)

CTGTTAGTAGTATAGTGATGCCAGCAGCTAGGACTGGGAGAGATAGGAGAAGTAGGACTGCTGTGATTA
GGACGGATCAGACGAAGAGGGGCGTTTGGTA 3 (0.000091%)

CTGTTGCAAATTCTCATGGTTTGGGTTGGGTGGTGGAGAGCGCGTGTCATCTGCGGGTGGCACTGCCCAC
GGTGGGCGGGCGGGCCTCTCTACTCGAAGG 7 (0.000213%)

CTGTTTTTAAGCCTAATGTGGGGACAGCTCATGAGTGCAAGACGTCTTGTGATGTAATTATTATACGAAT
GGGGGCTTCAATCGGGAGTACTACTCGATT 27 (0.000822%)

CTTAAAACTGCCACACGCAAAAAAGAAAACCAAAGTGGTCCACAAAACATTCTCCTTTCCTTCTGAAGG
TTTTACGATGCATTGTTATCATTAACCAGTC 8 (0.000244%)

CTTAACAATTTCCTCATATCTCTTCTGGCTGTAGGGTGGCTCAGTGGAATCCATTTTGTTAACACCGACAA
TTAGTTGTTTCACACCCAGTGTGTAAGCC 24 (0.000731%)

CTTAATGTAAGTGCTGACTTCCTTAACAATTTCCTCATATCTCTTCTGGCTGTAGGGTGGCTCAGTGGAAT
CCATTTTGTTAACACCGACAATTAGTTGT 13 (0.000396%)

CTTATGCGGAGGAGAATGTTTTCATGTTACTTATACTAACATTAGTTCTTCTATAGGGTGATAGATTGGTC
CAATTGGGTGTGAGGAGTTCAGTTATATG 5 (0.000152%)

CTTCAATCGGGAGTACTACTCGATTGTCAACGTCAAGGAGTCGCAGGTCGCCTGGTTCTAGGAATAATGG
GGGAAGTATGTAGGAGTTGAAGATTAGTCC 93 (0.002831%)

CTTCACTCAAAGCTTCATGGTGCATTTCGACAGATTTTACTTCCGTTGTAACGTTGACTGGAGCAAAGGT
GACCACCATACCGGGTTTGAGAACACCAGT 51 (0.001552%)

CTTCATATGAGATTGTTTGGGCTACTGCTCGCAGTGCGCCGATCAGGGCGTAGTTTGAGTTTGATGCTCA
CCCTGATCAGAGGATTGAGTAAACGGCTAG 27 (0.000822%)

CTTCCACCACTGATTAAGAGTGGGGTGGCAGGTATTAGGGATAATATTCATTTAGCCTTCTGAGCTTTCT
GGGCAGACTTGGTGACCTTGCCAGCTCCAG 20 (0.000609%)

CTTCCATTGGTGGGTCATTTTTGCTGTCACCAGCAACGTTGCCACGACGAACATCCTTGACAGACACATT
CTTGACATTGAAGCCCACATTGTCCCCAGG 31 (0.000944%)

CTTCCTTAACAATTTCCTCATATCTCTTCTGGCTGTAGGGTGGCTCAGTGGAATCCATTTTGTTAACACCG
ACAATTAGTTGTTTCACACCCAGTGTGTA 52 (0.001583%)

CTTCTGAGCTTTCTGGGCAGACTTGGTGACCTTGCCAGCTCCAGCAGCCTTCTTGTCCACTGCTTTGATGA
CACCCACCGCAACTGTCTGTCTCATATCA 21 (0.000639%)

CTTCTGGCTGTAGGGTGGCTCAGTGGAATCCATTTTGTTAACACCGACAATTAGTTGTTTCACACCCAGT
GTGTAAGCCAGAAGGGCATGCTCTCGGGTC 18 (0.000548%)

CTTGCCAGCTCCAGCAGCCTTCTTGTCCACTGCTTTGATGACACCCACCGCAACTGTCTGTCTCATATCAC
GAACAGCAAAGCGACCCAAAGGTGGATAG 16 (0.000487%)

CTTGGTGACCTTGCCAGCTCCAGCAGCCTTCTTGTCCACTGCTTTGATGACACCCACCGCAACTGTCTGTC
TCATATCACGAACAGCAAAGCGACCCAAA 12 (0.000365%)

CTTGTCAGTTGGACGAGTTGGTGGTAGGATGCAGTCCAGAGCCTCAAGCAGCGTGGTTCCACTGGCATTG
CCATCCTTACGGGTGACTTTCCATCCCTTG 57 (0.001735%)

CTTGTCCAATGATGGTAAAAGGGTAGCTTACTGGTTGTCCTCCGATTCAGGTTAGAATGAGGAGGTCTGC
GGCTAGGAGTCAATAAAGTGATTGGCTTAG 4 (0.000122%)

CTTTAATTGGACTGCCTTCGTAATTCATTGCCTCTGCTTCAACAATGTGCAACTCATCCTTTGCACCAGCC
CCTAAACTGACCGTTCTTAAAGATAACTG 12 (0.000365%)

CTTTACTATAAAAGCTATTGTGTAAGCTAGTCATATTAAGTTGTTGGCTCAGGAGTTTGATAGTTCTTGGG
CAGTGAGAGTGAGTAGTAGAATGTTTAGT 16 (0.000487%)

CTTTAGGGAGTCATAAGTGGAGTCCGTAAAGAGGTATCTTTACTATAAAAGCTATTGTGTAAGCTAGTCA
TATTAAGTTGTTGGCTCAGGAGTTTGATAG 5 (0.000152%)

CTTTGATGACACCCACCGCAACTGTCTGTCTCATATCACGAACAGCAAAGCGACCCAAAGGTGGATAGT
CTGAGAAGCTCTCAACACACATGGGCTTGCC 354 (0.010776%)

CTTTGCTACTGGTCCTGTAATGGCAGAACCTTTCATCTCGCCTTTATTGTTCACTATGACTCCTGCATTAT
CTTCAAAATAAAGAAACACGCCATCTTTT 17 (0.000517%)

GAAAACCAAAGTGGTCCACAAAACATTCTCCTTTCCTTCTGAAGGTTTTACGATGCATTGTTATCATTAA
CCAGTCTTTTACTACTAAACTTAAATGGCC 11 (0.000335%)

GAAAAGAAAGATGAATCCTAGGGCTCAGAGCACTGCAGCAGATCATTTCATATTGCTTCCGTGGAGTGT
GGCGAGTCAGCTAAATACTTTGACGCCGGTG 4 (0.000122%)

GAAAAGGTTGGGGAACAGCTAAATAGGTTGTTGTTGATTTGGTTAAAAAATAGTAGAGGGATGATGCTA
ATAATTAGGCTGTGGGTGGTTGTGTTGATTC 11 (0.000335%)

GAAAGATGAATCCTAGGGCTCAGAGCACTGCAGCAGATCATTTCATATTGCTTCCGTGGAGTGTGGCGA
GTCAGCTAAATACTTTGACGCCGGTGGGGAT 10 (0.000304%)

GAAAGTTAGATTTACGCCGATGAATATGATAGTGAAATGGATTTTGGCGTAGGTTTGGTCTAGGGTGTAG
CCTGAGAATAGGGGAAATCAGTGAATGAAG 17 (0.000517%)

GAAATTGATGGCCCCTAAGATAGAGGAGACACCTGCTAGGTGTAAGGAGAAGATGGTTAGGTCTACGGA
GGCTCCAGGGTGGGAGTAGTTCCCTGCTAAG 23 (0.000700%)

GAAGAAAGTTAGATTTACGCCGATGAATATGATAGTGAAATGGATTTTGGCGTAGGTTTGGTCTAGGGT
GTAGCCTGAGAATAGGGGAAATCAGTGAATG 21 (0.000639%)

GAAGAGCTTCACTCAAAGCTTCATGGTGCATTTCGACAGATTTTACTTCCGTTGTAACGTTGACTGGAGC
AAAGGTGACCACCATACCGGGTTTGAGAAC 29 (0.000883%)

GAAGCCAGCTGCTTCCATTGGTGGGTCATTTTTGCTGTCACCAGCAACGTTGCCACGACGAACATCCTTG
ACAGACACATTCTTGACATTGAAGCCCACA 6 (0.000183%)

GAAGCCTGGTAGGATAAGAATATAAACTTCAGGGTGACCGAAAAATCAGAATAGGTGTTGGTATAGAAT
GGGGTCTCCTCCTCCGGCGGGGTCGAAGAAG 5 (0.000152%)

GAAGCTCTCAACACACATGGGCTTGCCAGGAACCATATCAACAATGGCAGCATCACCAGACTTCAAGAA
TTTAGGGCCATCTTCCAGCTTTTTACCAGAA 17 (0.000517%)

GAAGGGCATGCTCTCGGGTCTGCCCATTCTTGGAGATACCAGCTTCAAATTCACCAACACCAGCAGCAA
CAATCAGGACAGCACAGTCAGCCTGAGATGT 8 (0.000244%)

GAAGGGGTAGGCTATGTGTTTTGTCAGGGGGTTGAGAATGAGTGTGAGGCGTATTATACCATAGCCGCC
TAGTTTTAAGAGTACTGCGGCAAGTACTATT 7 (0.000213%)

GAATGAGTAGGCTGATGGTTTCGATAATAACTAGTATGGGGATAAGGGGTGTAGGTGTGCCTTGTGGTA
AGAAGTGGGCTAGGGCATTTTTAATCTTAGA 22 (0.000670%)

GAATGATGGCTAGGGTGACTTCATATGAGATTGTTTGGGCTACTGCTCGCAGTGCGCCGATCAGGGCGTA
GTTTGAGTTTGATGCTCACCCTGATCAGAG 3 (0.000091%)

GAATTATTTGGTTTCGGTTGTTTTCTATTAGACTATGGTGAGCTCAGGTGATTGATACTCCTGATGCGAGT
AATACGGATGTGTTTAGGAGTGGGACTTC 167 (0.005083%)

GACATCCTGGAGAGGCAGGCGCAAGGGCTTGTCAGTTGGACGAGTTGGTGGTAGGATGCAGTCCAGAGC
CTCAAGCAGCGTGGTTCCACTGGCATTGCCA 4 (0.000122%)

GACCCAGTAAAATTGTAATAAGCAGTGCTTGAATTATTTGGTTTCGGTTGTTTTCTATTAGACTATGGTGA
GCTCAGGTGATTGATACTCCTGATGCGAG 7 (0.000213%)

GACCGTTCTTCCACCACTGATTAAGAGTGGGGTGGCAGGTATTAGGGATAATATTCATTTAGCCTTCTGA
GCTTTCTGGGCAGACTTGGTGACCTTGCCA 29 (0.000883%)

GACCTTGCCAGCTCCAGCAGCCTTCTTGTCCACTGCTTTGATGACACCCACCGCAACTGTCTGTCTCATAT
CACGAACAGCAAAGCGACCCAAAGGTGGA 9 (0.000274%)

GACGAGTTGGTGGTAGGATGCAGTCCAGAGCCTCAAGCAGCGTGGTTCCACTGGCATTGCCATCCTTAC
GGGTGACTTTCCATCCCTTGAACCAAGGCAT 32 (0.000974%)

GACTTCCTTAACAATTTCCTCATATCTCTTCTGGCTGTAGGGTGGCTCAGTGGAATCCATTTTGTTAACAC
CGACAATTAGTTGTTTCACACCCAGTGTG 10 (0.000304%)

GACTTGGTGACCTTGCCAGCTCCAGCAGCCTTCTTGTCCACTGCTTTGATGACACCCACCGCAACTGTCT
GTCTCATATCACGAACAGCAAAGCGACCCA 16 (0.000487%)

GAGAAGATGGTTAGGTCTACGGAGGCTCCAGGGTGGGAGTAGTTCCCTGCTAAGGGAGGGTAGACTGTT
CAACCTGTTCCTGCTCCGGCCTCCACTATAG 13 (0.000396%)

GAGAAGCTCTCAACACACATGGGCTTGCCAGGAACCATATCAACAATGGCAGCATCACCAGACTTCAAG
AATTTAGGGCCATCTTCCAGCTTTTTACCAG 13 (0.000396%)

GAGACCCAGTAAAATTGTAATAAGCAGTGCTTGAATTATTTGGTTTCGGTTGTTTTCTATTAGACTATGGT
GAGCTCAGGTGATTGATACTCCTGATGCG 5 (0.000152%)

GAGACCGTTCTTCCACCACTGATTAAGAGTGGGGTGGCAGGTATTAGGGATAATATTCATTTAGCCTTCT
GAGCTTTCTGGGCAGACTTGGTGACCTTGC 8 (0.000244%)

GAGAGGCAGGCGCAAGGGCTTGTCAGTTGGACGAGTTGGTGGTAGGATGCAGTCCAGAGCCTCAAGCA
GCGTGGTTCCACTGGCATTGCCATCCTTACGG 22 (0.000670%)

GAGCACTGCAGCAGATCATTTCATATTGCTTCCGTGGAGTGTGGCGAGTCAGCTAAATACTTTGACGCCG
GTGGGGATAGCGATGATTATGGTAGCGGAG 18 (0.000548%)

GAGCAGTGAAGCCAGCTGCTTCCATTGGTGGGTCATTTTTGCTGTCACCAGCAACGTTGCCACGACGAAC
ATCCTTGACAGACACATTCTTGACATTGAA 4 (0.000122%)

GAGCTTCACTCAAAGCTTCATGGTGCATTTCGACAGATTTTACTTCCGTTGTAACGTTGACTGGAGCAAA

GGTGACCACCATACCGGGTTTGAGAACACC 10 (0.000304%)

GAGCTTTCTGGGCAGACTTGGTGACCTTGCCAGCTCCAGCAGCCTTCTTGTCCACTGCTTTGATGACACC
CACCGCAACTGTCTGTCTCATATCACGAAC 14 (0.000426%)

GAGGAAAAGGTTGGGGAACAGCTAAATAGGTTGTTGTTGATTTGGTTAAAAAATAGTAGAGGGATGATG
CTAATAATTAGGCTGTGGGTGGTTGTGTTGA 3 (0.000091%)

GAGGAGACACCTGCTAGGTGTAAGGAGAAGATGGTTAGGTCTACGGAGGCTCCAGGGTGGGAGTAGTTC
CCTGCTAAGGGAGGGTAGACTGTTCAACCTG 4 (0.000122%)

GAGGCAGGCGCAAGGGCTTGTCAGTTGGACGAGTTGGTGGTAGGATGCAGTCCAGAGCCTCAAGCAGCG
TGGTTCCACTGGCATTGCCATCCTTACGGGT 17 (0.000517%)

GAGGGTAAAATAGAGACCCAGTAAAATTGTAATAAGCAGTGCTTGAATTATTTGGTTTCGGTTGTTTTCT
ATTAGACTATGGTGAGCTCAGGTGATTGAT 5 (0.000152%)

GAGGTGATCGGCGATCAGAGGGCGATGAAGTTCTAGATCCATTGAGACAAGCTCTAGACAGTAGCATGC
AGTCCCACAACTTGTACCAGCATCCCCAGCG 5 (0.000152%)

GAGTACTACTCGATTGTCAACGTCAAGGAGTCGCAGGTCGCCTGGTTCTAGGAATAATGGGGGAAGTAT
GTAGGAGTTGAAGATTAGTCCGCCGTAGTCG 7 (0.000213%)

GAGTCATAAGTGGAGTCCGTAAAGAGGTATCTTTACTATAAAAGCTATTGTGTAAGCTAGTCATATTAAG
TTGTTGGCTCAGGAGTTTGATAGTTCTTGG 4 (0.000122%)

GAGTGAGGCAGGAGTCCGAGGAGGTTAGTTGTGGCAATAAAAATGATTAAGGATACTAGTATAAGAGA
TCAGGTTCGTCCTTTAGTGTTGTGTATGGTTA 9 (0.000274%)

GAGTTGGTGGTAGGATGCAGTCCAGAGCCTCAAGCAGCGTGGTTCCACTGGCATTGCCATCCTTACGGGT
GACTTTCCATCCCTTGAACCAAGGCATGTT 7 (0.000213%)

GATAATATTCATTTAGCCTTCTGAGCTTTCTGGGCAGACTTGGTGACCTTGCCAGCTCCAGCAGCCTTCTT
GTCCACTGCTTTGATGACACCCACCGCAA 20 (0.000609%)

GATAATCACCTGAGCAGTGAAGCCAGCTGCTTCCATTGGTGGGTCATTTTTGCTGTCACCAGCAACGTTG
CCACGACGAACATCCTTGACAGACACATTC 3 (0.000091%)

GATAGAGGAGACACCTGCTAGGTGTAAGGAGAAGATGGTTAGGTCTACGGAGGCTCCAGGGTGGGAGT
AGTTCCCTGCTAAGGGAGGGTAGACTGTTCAA 3 (0.000091%)

GATAGTTGGGTGGTTGGTGTAAATGAGTGAGGCAGGAGTCCGAGGAGGTTAGTTGTGGCAATAAAAATG
ATTAAGGATACTAGTATAAGAGATCAGGTTC 5 (0.000152%)

GATATTGCTAGGGTGGCGCTTCCAATTAGGTGCATGAGTAGGTGGCCTGCAGTAATGTTAGCGGTTAGGC
GTACGGCCAGGGCTATTGGTTGAATGAGTA 9 (0.000274%)

GATGAATCCTAGGGCTCAGAGCACTGCAGCAGATCATTTCATATTGCTTCCGTGGAGTGTGGCGAGTCAG
CTAAATACTTTGACGCCGGTGGGGATAGCG 18 (0.000548%)

GATGCTACTTGTCCAATGATGGTAAAAGGGTAGCTTACTGGTTGTCCTCCGATTCAGGTTAGAATGAGGA
GGTCTGCGGCTAGGAGTCAATAAAGTGATT 3 (0.000091%)

GATGGACACCAGTTTTAGCCAACATAGCATAGTACTCTATTTCAGATTTCCTCAAAGCTGGGCAGTTGTT
AGCGAGAATGACCAATTTCGCTTTGCCTTG 5 (0.000152%)

GATGGCCCCTAAGATAGAGGAGACACCTGCTAGGTGTAAGGAGAAGATGGTTAGGTCTACGGAGGCTCC
AGGGTGGGAGTAGTTCCCTGCTAAGGGAGGG 3 (0.000091%)

GATGGCTAGGGTGACTTCATATGAGATTGTTTGGGCTACTGCTCGCAGTGCGCCGATCAGGGCGTAGTTT
GAGTTTGATGCTCACCCTGATCAGAGGATT 8 (0.000244%)

GATTAAGAGTGGGGTGGCAGGTATTAGGGATAATATTCATTTAGCCTTCTGAGCTTTCTGGGCAGACTTG
GTGACCTTGCCAGCTCCAGCAGCCTTCTTG 3 (0.000091%)

GATTTTAAAAACTAATAACTTAAAACTGCCACACGCAAAAAAGAAAACCAAAGTGGTCCACAAAACATT
CTCCTTTCCTTCTGAAGGTTTTACGATGCAT 15 (0.000457%)

GCAAAAAAGAAAACCAAAGTGGTCCACAAAACATTCTCCTTTCCTTCTGAAGGTTTTACGATGCATTGTT
ATCATTAACCAGTCTTTTACTACTAAACTT44 (0.001339%)

GCAAATTCTCATGGTTTGGGTTGGGTGGTGGAGAGCGCGTGTCATCTGCGGGTGGCACTGCCCACGGTG
GGCGGGCGGGCCTCTCTACTCGAAGGTGACC 23 (0.000700%)

GCAAATTCTGCCAGATACCTGTGGTAGTCCCCTTTCATTTTATAATAGAAAACCTTGGACTCGCCAGTGT
TAGCTGCTGGAATGAGGTGTTTGTCCAGTA 4 (0.000122%)

GCAACAATCAGGACAGCACAGTCAGCCTGAGATGTCCCTGTAATCATGTTTTTGATAAAGTCTCTGTGTC
CTGGGGCATCAATGATAGTCACATAGTACT 33 (0.001005%)

GCAAGGGCTTGTCAGTTGGACGAGTTGGTGGTAGGATGCAGTCCAGAGCCTCAAGCAGCGTGGTTCCAC
TGGCATTGCCATCCTTACGGGTGACTTTCCA 29 (0.000883%)

GCACACTCCTTTGCTACTGGTCCTGTAATGGCAGAACCTTTCATCTCGCCTTTATTGTTCACTATGACTCC
TGCATTATCTTCAAAATAAAGAAACACGC 11 (0.000335%)

GCACTGCAGCAGATCATTTCATATTGCTTCCGTGGAGTGTGGCGAGTCAGCTAAATACTTTGACGCCGGT
GGGGATAGCGATGATTATGGTAGCGGAGGT 17 (0.000517%)

GCAGACTTGGTGACCTTGCCAGCTCCAGCAGCCTTCTTGTCCACTGCTTTGATGACACCCACCGCAACTG
TCTGTCTCATATCACGAACAGCAAAGCGAC 56 (0.001705%)

GCAGATCATTTCATATTGCTTCCGTGGAGTGTGGCGAGTCAGCTAAATACTTTGACGCCGGTGGGGATAG
CGATGATTATGGTAGCGGAGGTGAAATATG 9 (0.000274%)

GCAGCAACAATCAGGACAGCACAGTCAGCCTGAGATGTCCCTGTAATCATGTTTTTGATAAAGTCTCTGT
GTCCTGGGGCATCAATGATAGTCACATAGT 975 (0.029679%)

GCAGCAGATCATTTCATATTGCTTCCGTGGAGTGTGGCGAGTCAGCTAAATACTTTGACGCCGGTGGGGA
TAGCGATGATTATGGTAGCGGAGGTGAAAT 12 (0.000365%)

GCAGCGTGGTTCCACTGGCATTGCCATCCTTACGGGTGACTTTCCATCCCTTGAACCAAGGCATGTTAGC
ACTTGGCTCCAGCATGTTGTCACCATTCCA 23 (0.000700%)

GCAGGCGCAAGGGCTTGTCAGTTGGACGAGTTGGTGGTAGGATGCAGTCCAGAGCCTCAAGCAGCGTGG
TTCCACTGGCATTGCCATCCTTACGGGTGAC 63 (0.001918%)

GCAGGCTTATGCGGAGGAGAATGTTTTCATGTTACTTATACTAACATTAGTTCTTCTATAGGGTGATAGA
TTGGTCCAATTGGGTGTGAGGAGTTCAGTT 44 (0.001339%)

GCAGGTATTAGGGATAATATTCATTTAGCCTTCTGAGCTTTCTGGGCAGACTTGGTGACCTTGCCAGCTC
CAGCAGCCTTCTTGTCCACTGCTTTGATGA 114 (0.003470%)

GCAGTAATGTTAGCGGTTAGGCGTACGGCCAGGGCTATTGGTTGAATGAGTAGGCTGATGGTTTCGATA
ATAACTAGTATGGGGATAAGGGGTGTAGGTG 10 (0.000304%)

GCAGTCCAGAGCCTCAAGCAGCGTGGTTCCACTGGCATTGCCATCCTTACGGGTGACTTTCCATCCCTTG
AACCAAGGCATGTTAGCACTTGGCTCCAGC 33 (0.001005%)

GCAGTGAAGCCAGCTGCTTCCATTGGTGGGTCATTTTTGCTGTCACCAGCAACGTTGCCACGACGAACAT
CCTTGACAGACACATTCTTGACATTGAAGC 7 (0.000213%)

GCATCTGTTTTTAAGCCTAATGTGGGGACAGCTCATGAGTGCAAGACGTCTTGTGATGTAATTATTATAC
GAATGGGGGCTTCAATCGGGAGTACTACTC 4 (0.000122%)

GCATGAGTAGGTGGCCTGCAGTAATGTTAGCGGTTAGGCGTACGGCCAGGGCTATTGGTTGAATGAGTA
GGCTGATGGTTTCGATAATAACTAGTATGGG 25 (0.000761%)

GCATGCTCTCGGGTCTGCCCATTCTTGGAGATACCAGCTTCAAATTCACCAACACCAGCAGCAACAATCA
GGACAGCACAGTCAGCCTGAGATGTCCCTG 29 (0.000883%)

GCATTGCCATCCTTACGGGTGACTTTCCATCCCTTGAACCAAGGCATGTTAGCACTTGGCTCCAGCATGT
TGTCACCATTCCAACCAGAAATTGGCACAA 5 (0.000152%)

GCCAATTTTCTTAATGTAAGTGCTGACTTCCTTAACAATTTCCTCATATCTCTTCTGGCTGTAGGGTGGCT
CAGTGGAATCCATTTTGTTAACACCGACA 40 (0.001218%)

GCCACACGCAAAAAAGAAAACCAAAGTGGTCCACAAAACATTCTCCTTTCCTTCTGAAGGTTTTACGAT
GCATTGTTATCATTAACCAGTCTTTTACTAC 132 (0.004018%)

GCCACCTACGGTGAAAAGAAAGATGAATCCTAGGGCTCAGAGCACTGCAGCAGATCATTTCATATTGCT
TCCGTGGAGTGTGGCGAGTCAGCTAAATACT 21 (0.000639%)

GCCAGAAGGGCATGCTCTCGGGTCTGCCCATTCTTGGAGATACCAGCTTCAAATTCACCAACACCAGCA
GCAACAATCAGGACAGCACAGTCAGCCTGAG 48 (0.001461%)

GCCAGATACCTGTGGTAGTCCCCTTTCATTTTATAATAGAAAACCTTGGACTCGCCAGTGTTAGCTGCTG
GAATGAGGTGTTTGTCCAGTACATCCAGAA 3 (0.000091%)

GCCAGCTCCAGCAGCCTTCTTGTCCACTGCTTTGATGACACCCACCGCAACTGTCTGTCTCATATCACGA
ACAGCAAAGCGACCCAAAGGTGGATAGTCT 47 (0.001431%)

GCCAGCTGCTTCCATTGGTGGGTCATTTTTGCTGTCACCAGCAACGTTGCCACGACGAACATCCTTGACA
GACACATTCTTGACATTGAAGCCCACATTG 15 (0.000457%)

GCCAGGGCTATTGGTTGAATGAGTAGGCTGATGGTTTCGATAATAACTAGTATGGGGATAAGGGGTGTA
GGTGTGCCTTGTGGTAAGAAGTGGGCTAGGG 28 (0.000852%)

GCCAGTCAGGCCACCTACGGTGAAAAGAAAGATGAATCCTAGGGCTCAGAGCACTGCAGCAGATCATTT
CATATTGCTTCCGTGGAGTGTGGCGAGTCAG 18 (0.000548%)

GCCAGTGTTACTTTAATTGGACTGCCTTCGTAATTCATTGCCTCTGCTTCAACAATGTGCAACTCATCCTT
TGCACCAGCCCCTAAACTGACCGTTCTTA 145 (0.004414%)

GCCATGGCTAGGTTTATAGATAGTTGGGTGGTTGGTGTAAATGAGTGAGGCAGGAGTCCGAGGAGGTTA
GTTGTGGCAATAAAAATGATTAAGGATACTA 391 (0.011902%)

GCCCATACACATGAGTATTTGTCTAAAACATGTCTTCTTTGTAGCAGCTAGGCCCTGCCACCACTGTGCTT
GGCTGAGTTCACAAATCTGTTGTAACCTG 8 (0.000244%)

GCCCCTAAGATAGAGGAGACACCTGCTAGGTGTAAGGAGAAGATGGTTAGGTCTACGGAGGCTCCAGG
GTGGGAGTAGTTCCCTGCTAAGGGAGGGTAGA 3 (0.000091%)

GCCTCAAGCAGCGTGGTTCCACTGGCATTGCCATCCTTACGGGTGACTTTCCATCCCTTGAACCAAGGCA
TGTTAGCACTTGGCTCCAGCATGTTGTCAC 175 (0.005327%)

GCCTGCAGTAATGTTAGCGGTTAGGCGTACGGCCAGGGCTATTGGTTGAATGAGTAGGCTGATGGTTTCG

ATAATAACTAGTATGGGGATAAGGGGTGTA 32 (0.000974%)

GCCTTCTGAGCTTTCTGGGCAGACTTGGTGACCTTGCCAGCTCCAGCAGCCTTCTTGTCCACTGCTTTGAT
GACACCCACCGCAACTGTCTGTCTCATAT 33 (0.001005%)

GCGCAAGGGCTTGTCAGTTGGACGAGTTGGTGGTAGGATGCAGTCCAGAGCCTCAAGCAGCGTGGTTCC
ACTGGCATTGCCATCCTTACGGGTGACTTTC 18 (0.000548%)

GCGCTTCCAATTAGGTGCATGAGTAGGTGGCCTGCAGTAATGTTAGCGGTTAGGCGTACGGCCAGGGCT
ATTGGTTGAATGAGTAGGCTGATGGTTTCGA 16 (0.000487%)

GCGGGTTTTAGGGGCTCTTTGGTGAAGAGTTTTATGGCGTCAGCGAAGGGTTGTAGTAGCCCGTAGGGG
CCTACAACGTTGGGGCCTTTGCGTAGTTGTA 28 (0.000852%)

GCGGTCTGTTAGTAGTATAGTGATGCCAGCAGCTAGGACTGGGAGAGATAGGAGAAGTAGGACTGCTGT
GATTAGGACGGATCAGACGAAGAGGGGCGTT 442 (0.013454%)

GCGGTGGTCAGGTCCCGGTATTCCCGGTACATGTTGTGGGTGCCGCTCCGGGAGTCATAGCGCAGCCAG
ATCCCGAAGTTCTTCACCCGCAGGGGGGACT 298 (0.009071%)

GCGGTTAGGCGTACGGCCAGGGCTATTGGTTGAATGAGTAGGCTGATGGTTTCGATAATAACTAGTATG
GGGATAAGGGGTGTAGGTGTGCCTTGTGGTA 38 (0.001157%)

GCGTACGGCCAGGGCTATTGGTTGAATGAGTAGGCTGATGGTTTCGATAATAACTAGTATGGGGATAAG
GGGTGTAGGTGTGCCTTGTGGTAAGAAGTGG 8 (0.000244%)

GCTAATACAATGCCAGTCAGGCCACCTACGGTGAAAAGAAAGATGAATCCTAGGGCTCAGAGCACTGCA
GCAGATCATTTCATATTGCTTCCGTGGAGTG 16 (0.000487%)

GCTACTTGTCCAATGATGGTAAAAGGGTAGCTTACTGGTTGTCCTCCGATTCAGGTTAGAATGAGGAGGT
CTGCGGCTAGGAGTCAATAAAGTGATTGGC 11 (0.000335%)

GCTAGGGTGACTTCATATGAGATTGTTTGGGCTACTGCTCGCAGTGCGCCGATCAGGGCGTAGTTTGAGT
TTGATGCTCACCCTGATCAGAGGATTGAGT 17 (0.000517%)

GCTAGGGTGGCGCTTCCAATTAGGTGCATGAGTAGGTGGCCTGCAGTAATGTTAGCGGTTAGGCGTACG
GCCAGGGCTATTGGTTGAATGAGTAGGCTGA 26 (0.000791%)

GCTAGGTGTAAGGAGAAGATGGTTAGGTCTACGGAGGCTCCAGGGTGGGAGTAGTTCCCTGCTAAGGGA
GGGTAGACTGTTCAACCTGTTCCTGCTCCGG 25 (0.000761%)

GCTAGGTTTATAGATAGTTGGGTGGTTGGTGTAAATGAGTGAGGCAGGAGTCCGAGGAGGTTAGTTGTG
GCAATAAAAATGATTAAGGATACTAGTATAA 44 (0.001339%)

GCTATTGGTTGAATGAGTAGGCTGATGGTTTCGATAATAACTAGTATGGGGATAAGGGGTGTAGGTGTG
CCTTGTGGTAAGAAGTGGGCTAGGGCATTTT 12 (0.000365%)

GCTCAGAGCACTGCAGCAGATCATTTCATATTGCTTCCGTGGAGTGTGGCGAGTCAGCTAAATACTTTGA
CGCCGGTGGGGATAGCGATGATTATGGTAG 59 (0.001796%)

GCTCAGTGGAATCCATTTTGTTAACACCGACAATTAGTTGTTTCACACCCAGTGTGTAAGCCAGAAGGGC
ATGCTCTCGGGTCTGCCCATTCTTGGAGAT 55 (0.001674%)

GCTCTCAACACACACATGGGCTTGCCAGGAACCATATCAACAATGGCAGCATCACCAGACTTCAAGAATTT
AGGGCCATCTTCCAGCTTTTTACCAGAACGG 11 (0.000335%)

GCTCTCGGGTCTGCCCATTCTTGGAGATACCAGCTTCAAATTCACCAACACCAGCAGCAACAATCAGGAC
AGCACAGTCAGCCTGAGATGTCCCTGTAAT 11 (0.000335%)

GCTGACTTCCTTAACAATTTCCTCATATCTCTTCTGGCTGTAGGGTGGCTCAGTGGAATCCATTTTGTTAA CACCGACAATTAGTTGTTTCACACCCAGT 73 (0.002222%)

GCTGCTTCCATTGGTGGGTCATTTTTGCTGTCACCAGCAACGTTGCCACGACGAACATCCTTGACAGACA CATTCTTGACATTGAAGCCCACATTGTCCC 39 (0.001187%)

GCTGGAGTGGTAAAAGGCTCAGAAAAATCCTGCGAAGAAAAAAACTTCTGAGGTAATAAATAGGATTAT CCCGTATCGAAGGCCTTTTTGGACAGGTGGT 16 (0.000487%)

GCTGTAGGGTGGCTCAGTGGAATCCATTTTGTTAACACCGACAATTAGTTGTTTCACACCCAGTGTGTAA GCCAGAAGGGCATGCTCTCGGGTCTGCCCA 11 (0.000335%)

GCTTATGCGGAGGAGAATGTTTTCATGTTACTTATACTAACATTAGTTCTTCTATAGGGTGATAGATTGGT CCAATTGGGTGTGAGGAGTTCAGTTATAT 4 (0.000122%)

GCTTCAATCGGGAGTACTACTCGATTGTCAACGTCAAGGAGTCGCAGGTCGCCTGGTTCTAGGAATAATG GGGGAAGTATGTAGGAGTTGAAGATTAGTC 5 (0.000152%)

GCTTCACTCAAAGCTTCATGGTGCATTTCGACAGATTTTACTTCCGTTGTAACGTTGACTGGAGCAAAGG TGACCACCATACCGGGTTTGAGAACACCAG 24 (0.000731%)

GCTTGTCAGTTGGACGAGTTGGTGGTAGGATGCAGTCCAGAGCCTCAAGCAGCGTGGTTCCACTGGCATT GCCATCCTTACGGGTGACTTTCCATCCCTT11 (0.000335%)

GCTTTAGGGAGTCATAAGTGGAGTCCGTAAAGAGGTATCTTTACTATAAAAGCTATTGTGTAAGCTAGTC ATATTAAGTTGTTGGCTCAGGAGTTTGATA 9 (0.000274%)

GCTTTCTGGGCAGACTTGGTGACCTTGCCAGCTCCAGCAGCCTTCTTGTCCACTGCTTTGATGACACCCAC CGCAACTGTCTGTCTCATATCACGAACAG 10 (0.000304%)

GGAAAAGGTTGGGGAACAGCTAAATAGGTTGTTGTTGATTTGGTTAAAAAATAGTAGAGGGATGATGCT AATAATTAGGCTGTGGGTGGTTGTGTTGATT 18 (0.000548%)

GGAACAGCTAAATAGGTTGTTGTTGATTTGGTTAAAAAATAGTAGAGGGATGATGCTAATAATTAGGCT GTGGGTGGTTGTGTTGATTCAAATTATGTGT 11 (0.000335%)

GGAAGAAAGTTAGATTTACGCCGATGAATATGATAGTGAAATGGATTTTGGCGTAGGTTTGGTCTAGGG TGTAGCCTGAGAATAGGGGAAATCAGTGAAT 4 (0.000122%)

GGAAGAGCTTCACTCAAAGCTTCATGGTGCATTTCGACAGATTTTACTTCCGTTGTAACGTTGACTGGAG CAAAGGTGACCACCATACCGGGTTTGAGAA 137 (0.004170%)

GGAAGGGGTAGGCTATGTGTTTTGTCAGGGGGTTGAGAATGAGTGTGAGGCGTATTATACCATAGCCGC CTAGTTTTAAGAGTACTGCGGCAAGTACTAT 50 (0.001522%)

GGAATTGCATCTGTTTTTAAGCCTAATGTGGGGACAGCTCATGAGTGCAAGACGTCTTGTGATGTAATTA TTATACGAATGGGGGCTTCAATCGGGAGTA 6 (0.000183%)

GGACACCAGTTTTAGCCAACATAGCATAGTACTCTATTTCAGATTTCCTCAAAGCTGGGCAGTTGTTAGC GAGAATGACCAATTTCGCTTTGCCTTGTCT 17 (0.000517%)

GGACGAGTTGGTGGTAGGATGCAGTCCAGAGCCTCAAGCAGCGTGGTTCCACTGGCATTGCCATCCTTA CGGGTGACTTTCCATCCCTTGAACCAAGGCA 36 (0.001096%)

GGAGAAGATGGTTAGGTCTACGGAGGCTCCAGGGTGGGAGTAGTTCCCTGCTAAGGGAGGGTAGACTGT TCAACCTGTTCCTGCTCCGGCCTCCACTATA 37 (0.001126%)

GGAGACACCTGCTAGGTGTAAGGAGAAGATGGTTAGGTCTACGGAGGCTCCAGGGTGGGAGTAGTTCCC TGCTAAGGGAGGGTAGACTGTTCAACCTGTT 10 (0.000304%)

GGAGAGGCAGGCGCAAGGGCTTGTCAGTTGGACGAGTTGGTGGTAGGATGCAGTCCAGAGCCTCAAGC
AGCGTGGTTCCACTGGCATTGCCATCCTTACG 42 (0.001278%)

GGAGGAAAAGGTTGGGGAACAGCTAAATAGGTTGTTGTTGATTTGGTTAAAAAATAGTAGAGGGATGAT
GCTAATAATTAGGCTGTGGGTGGTTGTGTTG 4 (0.000122%)

GGAGGGGGGTTGTTAGGGGGTCGGAGGAAAAGGTTGGGGAACAGCTAAATAGGTTGTTGTTGATTTGGT
TAAAAAATAGTAGAGGGATGATGCTAATAAT 27 (0.000822%)

GGAGGGTAAAATAGAGACCCAGTAAAATTGTAATAAGCAGTGCTTGAATTATTTGGTTTCGGTTGTTTTC
TATTAGACTATGGTGAGCTCAGGTGATTGA 32 (0.000974%)

GGAGTACTACTCGATTGTCAACGTCAAGGAGTCGCAGGTCGCCTGGTTCTAGGAATAATGGGGGAAGTA
TGTAGGAGTTGAAGATTAGTCCGCCGTAGTC 12 (0.000365%)

GGAGTCATAAGTGGAGTCCGTAAAGAGGTATCTTTACTATAAAAGCTATTGTGTAAGCTAGTCATATTAA
GTTGTTGGCTCAGGAGTTTGATAGTTCTTG 13 (0.000396%)

GGAGTCCGTAAAGAGGTATCTTTACTATAAAAGCTATTGTGTAAGCTAGTCATATTAAGTTGTTGGCTCA
GGAGTTTGATAGTTCTTGGGCAGTGAGAGT 20 (0.000609%)

GGATAATATTCATTTAGCCTTCTGAGCTTTCTGGGCAGACTTGGTGACCTTGCCAGCTCCAGCAGCCTTCT
TGTCCACTGCTTTGATGACACCCACCGCA 189 (0.005753%)

GGATAATCACCTGAGCAGTGAAGCCAGCTGCTTCCATTGGTGGGTCATTTTTGCTGTCACCAGCAACGTT
GCCACGACGAACATCCTTGACAGACACATT 31 (0.000944%)

GGATAGTACAAGGAAGGGGTAGGCTATGTGTTTTGTCAGGGGGTTGAGAATGAGTGTGAGGCGTATTAT
ACCATAGCCGCCTAGTTTTAAGAGTACTGCG 45 (0.001370%)

GGATGCAGTCCAGAGCCTCAAGCAGCGTGGTTCCACTGGCATTGCCATCCTTACGGGTGACTTTCCATCC
CTTGAACCAAGGCATGTTAGCACTTGGCTC 27 (0.000822%)

GGATGCTACTTGTCCAATGATGGTAAAAGGGTAGCTTACTGGTTGTCCTCCGATTCAGGTTAGAATGAGG
AGGTCTGCGGCTAGGAGTCAATAAAGTGAT 20 (0.000609%)

GGCAAATTCTGCCAGATACCTGTGGTAGTCCCCTTTCATTTTATAATAGAAAACCTTGGACTCGCCAGTG
TTAGCTGCTGGAATGAGGTGTTTGTCCAGT 3 (0.000091%)

GGCAGACTTGGTGACCTTGCCAGCTCCAGCAGCCTTCTTGTCCACTGCTTTGATGACACCCACCGCAACT
GTCTGTCTCATATCACGAACAGCAAAGCGA 79 (0.002405%)

GGCAGGCGCAAGGGCTTGTCAGTTGGACGAGTTGGTGGTAGGATGCAGTCCAGAGCCTCAAGCAGCGTG
GTTCCACTGGCATTGCCATCCTTACGGGTGA 86 (0.002618%)

GGCAGGTATTAGGGATAATATTCATTTAGCCTTCTGAGCTTTCTGGGCAGACTTGGTGACCTTGCCAGCT
CCAGCAGCCTTCTTGTCCACTGCTTTGATG 117 (0.003561%)

GGCATGCTCTCGGGTCTGCCCATTCTTGGAGATACCAGCTTCAAATTCACCAACACCAGCAGCAACAATC
AGGACAGCACAGTCAGCCTGAGATGTCCCT 36 (0.001096%)

GGCATTGCCATCCTTACGGGTGACTTTCCATCCCTTGAACCAAGGCATGTTAGCACTTGGCTCCAGCATG
TTGTCACCATTCCAACCAGAAATTGGCACA 15 (0.000457%)

GGCCACCTACGGTGAAAAGAAAGATGAATCCTAGGGCTCAGAGCACTGCAGCAGATCATTTCATATTGC
TTCCGTGGAGTGTGGCGAGTCAGCTAAATAC 18 (0.000548%)

GGCCAGGGCTATTGGTTGAATGAGTAGGCTGATGGTTTCGATAATAACTAGTATGGGGATAAGGGGTGT

AGGTGTGCCTTGTGGTAAGAAGTGGGCTAGG 5 (0.000152%)

GGCCCCTAAGATAGAGGAGACACCTGCTAGGTGTAAGGAGAAGATGGTTAGGTCTACGGAGGCTCCAG
GGTGGGAGTAGTTCCCTGCTAAGGGAGGGTAG 5 (0.000152%)

GGCCTGCAGTAATGTTAGCGGTTAGGCGTACGGCCAGGGCTATTGGTTGAATGAGTAGGCTGATGGTTTC
GATAATAACTAGTATGGGGATAAGGGGTGT 10 (0.000304%)

GGCGCAAGGGCTTGTCAGTTGGACGAGTTGGTGGTAGGATGCAGTCCAGAGCCTCAAGCAGCGTGGTTC
CACTGGCATTGCCATCCTTACGGGTGACTTT 46 (0.001400%)

GGCGCTTCCAATTAGGTGCATGAGTAGGTGGCCTGCAGTAATGTTAGCGGTTAGGCGTACGGCCAGGGC
TATTGGTTGAATGAGTAGGCTGATGGTTTCG 86 (0.002618%)

GGCGGGTTTTAGGGGCTCTTTGGTGAAGAGTTTTATGGCGTCAGCGAAGGGTTGTAGTAGCCCGTAGGG
GCCTACAACGTTGGGGCCTTTGCGTAGTTGT 3 (0.000091%)

GGCGTACGGCCAGGGCTATTGGTTGAATGAGTAGGCTGATGGTTTCGATAATAACTAGTATGGGGATAA
GGGGTGTAGGTGTGCCTTGTGGTAAGAAGTG 24 (0.000731%)

GGCTAGGGTGACTTCATATGAGATTGTTTGGGCTACTGCTCGCAGTGCGCCGATCAGGGCGTAGTTTGAG
TTTGATGCTCACCCTGATCAGAGGATTGAG 4 (0.000122%)

GGCTAGGTTTATAGATAGTTGGGTGGTTGGTGTAAATGAGTGAGGCAGGAGTCCGAGGAGGTTAGTTGT
GGCAATAAAAATGATTAAGGATACTAGTATA 17 (0.000517%)

GGCTATTGGTTGAATGAGTAGGCTGATGGTTTCGATAATAACTAGTATGGGGATAAGGGGTGTAGGTGT
GCCTTGTGGTAAGAAGTGGGCTAGGGCATTT 16 (0.000487%)

GGCTCAGAGCACTGCAGCAGATCATTTCATATTGCTTCCGTGGAGTGTGGCGAGTCAGCTAAATACTTTG
ACGCCGGTGGGGATAGCGATGATTATGGTA 7 (0.000213%)

GGCTCAGTGGAATCCATTTTGTTAACACCGACAATTAGTTGTTTCACACCCAGTGTGTAAGCCAGAAGGG
CATGCTCTCGGGTCTGCCCATTCTTGGAGA 9 (0.000274%)

GGCTGGAGTGGTAAAAGGCTCAGAAAAATCCTGCGAAGAAAAAAACTTCTGAGGTAATAAATAGGATT
ATCCCGTATCGAAGGCCTTTTTGGACAGGTGG 430 (0.013089%)

GGCTGTAGGGTGGCTCAGTGGAATCCATTTTGTTAACACCGACAATTAGTTGTTTCACACCCAGTGTGTA
AGCCAGAAGGGCATGCTCTCGGGTCTGCCC 6 (0.000183%)

GGCTTATGCGGAGGAGAATGTTTTCATGTTACTTATACTAACATTAGTTCTTCTATAGGGTGATAGATTG
GTCCAATTGGGTGTGAGGAGTTCAGTTATA 10 (0.000304%)

GGCTTCAATCGGGAGTACTACTCGATTGTCAACGTCAAGGAGTCGCAGGTCGCCTGGTTCTAGGAATAAT
GGGGGAAGTATGTAGGAGTTGAAGATTAGT 7 (0.000213%)

GGCTTGTCAGTTGGACGAGTTGGTGGTAGGATGCAGTCCAGAGCCTCAAGCAGCGTGGTTCCACTGGCA
TTGCCATCCTTACGGGTGACTTTCCATCCCT 11 (0.000335%)

GGGAACAGCTAAATAGGTTGTTGTTGATTTGGTTAAAAAATAGTAGAGGGATGATGCTAATAATTAGGC
TGTGGGTGGTTGTGTTGATTCAAATTATGTG 7 (0.000213%)

GGGAAGAAAGTTAGATTTACGCCGATGAATATGATAGTGAAATGGATTTTGGCGTAGGTTTGGTCTAGG
GTGTAGCCTGAGAATAGGGGAAATCAGTGAA 7 (0.000213%)

GGGAATTGCATCTGTTTTTAAGCCTAATGTGGGGACAGCTCATGAGTGCAAGACGTCTTGTGATGTAATT
ATTATACGAATGGGGGCTTCAATCGGGAGT 791 (0.024078%)

GGGAGTACTACTCGATTGTCAACGTCAAGGAGTCGCAGGTCGCCTGGTTCTAGGAATAATGGGGGAAGT
ATGTAGGAGTTGAAGATTAGTCCGCCGTAGT 39 (0.001187%)

GGGAGTCATAAGTGGAGTCCGTAAAGAGGTATCTTTACTATAAAAGCTATTGTGTAAGCTAGTCATATTA
AGTTGTTGGCTCAGGAGTTTGATAGTTCTT 42 (0.001278%)

GGGATAATATTCATTTAGCCTTCTGAGCTTTCTGGGCAGACTTGGTGACCTTGCCAGCTCCAGCAGCCTT
CTTGTCCACTGCTTTGATGACACCCACCGC 35 (0.001065%)

GGGATAGTACAAGGAAGGGGTAGGCTATGTGTTTGTCAGGGGGTTGAGAATGAGTGTGAGGCGTATTA
TACCATAGCCGCCTAGTTTTAAGAGTACTGC 13 (0.000396%)

GGGCAGACTTGGTGACCTTGCCAGCTCCAGCAGCCTTCTTGTCCACTGCTTTGATGACACCCACCGCAAC
TGTCTGTCTCATATCACGAACAGCAAAGCG 22 (0.000670%)

GGGCATGCTCTCGGGTCTGCCCATTCTTGGAGATACCAGCTTCAAATTCACCAACACCAGCAGCAACAAT
CAGGACAGCACAGTCAGCCTGAGATGTCCC 20 (0.000609%)

GGGCTATTGGTTGAATGAGTAGGCTGATGGTTTCGATAATAACTAGTATGGGGATAAGGGGTGTAGGTG
TGCCTTGTGGTAAGAAGTGGGCTAGGGCATT 45 (0.001370%)

GGGCTCAGAGCACTGCAGCAGATCATTTCATATTGCTTCCGTGGAGTGTGGCGAGTCAGCTAAATACTTT
GACGCCGGTGGGGATAGCGATGATTATGGT 37 (0.001126%)

GGGCTTCAATCGGGAGTACTACTCGATTGTCAACGTCAAGGAGTCGCAGGTCGCCTGGTTCTAGGAATA
ATGGGGGAAGTATGTAGGAGTTGAAGATTAG 20 (0.000609%)

GGGCTTGTCAGTTGGACGAGTTGGTGGTAGGATGCAGTCCAGAGCCTCAAGCAGCGTGGTTCCACTGGC
ATTGCCATCCTTACGGGTGACTTTCCATCCC 35 (0.001065%)

GGGCTTTAGGGAGTCATAAGTGGAGTCCGTAAAGAGGTATCTTTACTATAAAAGCTATTGTGTAAGCTA
GTCATATTAAGTTGTTGGCTCAGGAGTTTGA 4 (0.000122%)

GGGGAACAGCTAAATAGGTTGTTGTTGATTTGGTTAAAAAATAGTAGAGGGATGATGCTAATAATTAGG
CTGTGGGTGGTTGTGTTGATTCAAATTATGT 9 (0.000274%)

GGGGCTTCAATCGGGAGTACTACTCGATTGTCAACGTCAAGGAGTCGCAGGTCGCCTGGTTCTAGGAAT
AATGGGGGAAGTATGTAGGAGTTGAAGATTA 42 (0.001278%)

GGGGGCTTCAATCGGGAGTACTACTCGATTGTCAACGTCAAGGAGTCGCAGGTCGCCTGGTTCTAGGAA
TAATGGGGGAAGTATGTAGGAGTTGAAGATT 67 (0.002039%)
GGGGGGGGGGGGGGGGGGGGGGGGGGGGGGGGGGGGGG 3151 (0.038366%)

GGGGGGTTGTTAGGGGGTCGGAGGAAAAGGTTGGGGAACAGCTAAATAGGTTGTTGTTGATTTGGTTAA
AAAATAGTAGAGGGATGATGCTAATAATTAG 5 (0.000152%)

GGGGGTCGGAGGAAAAGGTTGGGGAACAGCTAAATAGGTTGTTGTTGATTTGGTTAAAAAATAGTAGAG
GGATGATGCTAATAATTAGGCTGTGGGTGGT 9 (0.000274%)

GGGGGTTGTTAGGGGGTCGGAGGAAAAGGTTGGGGAACAGCTAAATAGGTTGTTGTTGATTTGGTTAAA
AAATAGTAGAGGGATGATGCTAATAATTAGG 17 (0.000517%)

GGGGTAGGCTATGTGTTTGTCAGGGGGTTGAGAATGAGTGTGAGGCGTATTATACCATAGCCGCCTAGT
TTTAAGAGTACTGCGGCAAGTACTATTGAC 18 (0.000548%)

GGGGTCGGAGGAAAAGGTTGGGGAACAGCTAAATAGGTTGTTGTTGATTTGGTTAAAAAATAGTAGAGG
GATGATGCTAATAATTAGGCTGTGGGTGGTT 3 (0.000091%)

GGGGTGGCAGGTATTAGGGATAATATTCATTTAGCCTTCTGAGCTTTCTGGGCAGACTTGGTGACCTTGC

CAGCTCCAGCAGCCTTCTTGTCCACTGCTT 6 (0.000183%)

GGGGTTGTTAGGGGGTCGGAGGAAAAGGTTGGGGAACAGCTAAATAGGTTGTTGTTGATTTGGTTAAAA
AATAGTAGAGGGATGATGCTAATAATTAGGC 12 (0.000365%)

GGGTAAAATAGAGACCCAGTAAAATTGTAATAAGCAGTGCTTGAATTATTTGGTTTCGGTTGTTTTCTAT
TAGACTATGGTGAGCTCAGGTGATTGATAC 9 (0.000274%)

GGGTAGGCTATGTGTTTTGTCAGGGGGTTGAGAATGAGTGTGAGGCGTATTATACCATAGCCGCCTAGTT
TTAAGAGTACTGCGGCAAGTACTATTGACC 28 (0.000852%)

GGGTCATTTTTGCTGTCACCAGCAACGTTGCCACGACGAACATCCTTGACAGACACATTCTTGACATTGA
AGCCCACATTGTCCCCAGGAAGAGCTTCAC 39 (0.001187%)

GGGTGACTTCATATGAGATTGTTTGGGCTACTGCTCGCAGTGCGCCGATCAGGGCGTAGTTTGAGTTTGA
TGCTCACCCTGATCAGAGGATTGAGTAAAC 9 (0.000274%)

GGGTGGCAGGTATTAGGGATAATATTCATTTAGCCTTCTGAGCTTTCTGGGCAGACTTGGTGACCTTGCC
AGCTCCAGCAGCCTTCTTGTCCACTGCTTT4 (0.000122%)

GGGTGGCGCTTCCAATTAGGTGCATGAGTAGGTGGCCTGCAGTAATGTTAGCGGTTAGGCGTACGGCCA
GGGCTATTGGTTGAATGAGTAGGCTGATGGT 5 (0.000152%)

GGGTGGCTCAGTGGAATCCATTTTGTTAACACCGACAATTAGTTGTTTCACACCCAGTGTGTAAGCCAGA
AGGGCATGCTCTCGGGTCTGCCCATTCTTG 41 (0.001248%)

GGGTGGTTGGTGTAAATGAGTGAGGCAGGAGTCCGAGGAGGTTAGTTGTGGCAATAAAAATGATTAAGG
ATACTAGTATAAGAGATCAGGTTCGTCCTTT 8 (0.000244%)

GGGTTGTTAGGGGGTCGGAGGAAAAGGTTGGGGAACAGCTAAATAGGTTGTTGTTGATTTGGTTAAAAA
ATAGTAGAGGGATGATGCTAATAATTAGGCT 3 (0.000091%)

GGTAAAATAGAGACCCAGTAAAATTGTAATAAGCAGTGCTTGAATTATTTGGTTTCGGTTGTTTTCTATT
AGACTATGGTGAGCTCAGGTGATTGATACT 12 (0.000365%)

GGTACCTTTCTCTTTGGCTTCTTTCTTTTTCTGATCATTTTCCTTCACACGTTTCAGGAAGCTATCTCGGCT
CTTAGAGTGCTTAATGTGCTCAATACGC 7 (0.000213%)

GGTAGATGTGGCGGGTTTTAGGGGCTCTTTGGTGAAGAGTTTTATGGCGTCAGCGAAGGGTTGTAGTAGC
CCGTAGGGGCCTACAACGTTGGGGCCTTTG 135 (0.004109%)

GGTAGGATGCAGTCCAGAGCCTCAAGCAGCGTGGTTCCACTGGCATTGCCATCCTTACGGGTGACTTTCC
ATCCCTTGAACCAAGGCATGTTAGCACTTG 11 (0.000335%)

GGTATCTTTACTATAAAAGCTATTGTGTAAGCTAGTCATATTAAGTTGTTGGCTCAGGAGTTTGATAGTTC
TTGGGCAGTGAGAGTGAGTAGTAGAATGT9 (0.000274%)

GGTATTAGGGATAATATTCATTTAGCCTTCTGAGCTTTCTGGGCAGACTTGGTGACCTTGCCAGCTCCAG
CAGCCTTCTTGTCCACTGCTTTGATGACAC 35 (0.001065%)

GGTCAGGTCCCGGTATTCCCGGTACATGTTGTGGGTGCCGCTCCGGGAGTCATAGCGCAGCCAGATCCCG
AAGTTCTTCACCCGCAGGGGGGACTTCTCA 3 (0.000091%)

GGTCATTTTTGCTGTCACCAGCAACGTTGCCACGACGAACATCCTTGACAGACACATTCTTGACATTGAA
GCCCACATTGTCCCCAGGAAGAGCTTCACT 19 (0.000578%)

GGTCGGAGGAAAAGGTTGGGGAACAGCTAAATAGGTTGTTGTTGATTTGGTTAAAAAATAGTAGAGGGA
TGATGCTAATAATTAGGCTGTGGGTGGTTGT 4 (0.000122%)

GGTCTGTTAGTAGTATAGTGATGCCAGCAGCTAGGACTGGGAGAGATAGGAGAAGTAGGACTGCTGTGA
TTAGGACGGATCAGACGAAGAGGGGCGTTTG 4 (0.000122%)

GGTGACCTTGCCAGCTCCAGCAGCCTTCTTGTCCACTGCTTTGATGACACCCACCGCAACTGTCTGTCTCA
TATCACGAACAGCAAAGCGACCCAAAGGT 14 (0.000426%)

GGTGACTTCATATGAGATTGTTTGGGCTACTGCTCGCAGTGCGCCGATCAGGGCGTAGTTTGAGTTTGAT
GCTCACCCTGATCAGAGGATTGAGTAAACG 3 (0.000091%)

GGTGATCGGCGATCAGAGGGCGATGAAGTTCTAGATCCATTGAGACAAGCTCTAGACAGTAGCATGCAG
TCCCACAACTTGTACCAGCATCCCCAGCGTC 5 (0.000152%)

GGTGCATGAGTAGGTGGCCTGCAGTAATGTTAGCGGTTAGGCGTACGGCCAGGGCTATTGGTTGAATGA
GTAGGCTGATGGTTTCGATAATAACTAGTAT 13 (0.000396%)

GGTGGCAGGTATTAGGGATAATATTCATTTAGCCTTCTGAGCTTTCTGGGCAGACTTGGTGACCTTGCCA
GCTCCAGCAGCCTTCTTGTCCACTGCTTTG3 (0.000091%)

GGTGGCCTGCAGTAATGTTAGCGGTTAGGCGTACGGCCAGGGCTATTGGTTGAATGAGTAGGCTGATGG
TTTCGATAATAACTAGTATGGGGATAAGGGG 3 (0.000091%)

GGTGGCGCTTCCAATTAGGTGCATGAGTAGGTGGCCTGCAGTAATGTTAGCGGTTAGGCGTACGGCCAG
GGCTATTGGTTGAATGAGTAGGCTGATGGTT 10 (0.000304%)

GGTGGCTCAGTGGAATCCATTTTGTTAACACCGACAATTAGTTGTTTCACACCCAGTGTGTAAGCCAGAA
GGGCATGCTCTCGGGTCTGCCCATTCTTGG 26 (0.000791%)

GGTGGGTCATTTTTGCTGTCACCAGCAACGTTGCCACGACGAACATCCTTGACAGACACATTCTTGACAT
TGAAGCCCACATTGTCCCCAGGAAGAGCTT 24 (0.000731%)

GGTGGTAGGATGCAGTCCAGAGCCTCAAGCAGCGTGGTTCCACTGGCATTGCCATCCTTACGGGTGACTT
TCCATCCCTTGAACCAAGGCATGTTAGCAC 21 (0.000639%)

GGTGGTCAGGTCCCGGTATTCCCGGTACATGTTGTGGGTGCCGCTCCGGGAGTCATAGCGCAGCCAGATC
CCGAAGTTCTTCACCCGCAGGGGGGACTTC 3 (0.000091%)

GGTGGTTGGTGTAAATGAGTGAGGCAGGAGTCCGAGGAGGTTAGTTGTGGCAATAAAAATGATTAAGGA
TACTAGTATAAGAGATCAGGTTCGTCCTTTA 5 (0.000152%)

GGTGTAAATGAGTGAGGCAGGAGTCCGAGGAGGTTAGTTGTGGCAATAAAAATGATTAAGGATACTAGT
ATAAGAGATCAGGTTCGTCCTTTAGTGTTGT 3 (0.000091%)

GGTTAGGCGTACGGCCAGGGCTATTGGTTGAATGAGTAGGCTGATGGTTTCGATAATAACTAGTATGGG
GATAAGGGGTGTAGGTGTGCCTTGTGGTAAG 7 (0.000213%)

GGTTGAATGAGTAGGCTGATGGTTTCGATAATAACTAGTATGGGGATAAGGGGTGTAGGTGTGCCTTGT
GGTAAGAAGTGGGCTAGGGCATTTTTAATCT 31 (0.000944%)

GGTTGGTGTAAATGAGTGAGGCAGGAGTCCGAGGAGGTTAGTTGTGGCAATAAAAATGATTAAGGATAC
TAGTATAAGAGATCAGGTTCGTCCTTTAGTG 32 (0.000974%)

GGTTGTTAGGGGGTCGGAGGAAAAGGTTGGGGAACAGCTAAATAGGTTGTTGTTGATTTGGTTAAAAAA
TAGTAGAGGGATGATGCTAATAATTAGGCTG 10 (0.000304%)

GGTTTATAGATAGTTGGGTGGTTGGTGTAAATGAGTGAGGCAGGAGTCCGAGGAGGTTAGTTGTGGCAA
TAAAAATGATTAAGGATACTAGTATAAGAGA 26 (0.000791%)

GGTTTGGGTTGGGTGGTGGAGAGCGCGTGTCATCTGCGGGTGGCACTGCCCACGGTGGGCGGGCGGGCC
TCTCTACTCGAAGGTGACCACGTTTAGATTC 9 (0.000274%)

GTAAAATAGAGACCCAGTAAAATTGTAATAAGCAGTGCTTGAATTATTTGGTTTCGGTTGTTTTCTATTA
GACTATGGTGAGCTCAGGTGATTGATACTC 78 (0.002374%)

GTAAAATTGTAATAAGCAGTGCTTGAATTATTTGGTTTCGGTTGTTTTCTATTAGACTATGGTGAGCTCAG
GTGATTGATACTCCTGATGCGAGTAATAC 86 (0.002618%)

GTAAAGAGGTATCTTTACTATAAAAGCTATTGTGTAAGCTAGTCATATTAAGTTGTTGGCTCAGGAGTTT
GATAGTTCTTGGGCAGTGAGAGTGAGTAGT 40 (0.001218%)

GTAAATGAGTGAGGCAGGAGTCCGAGGAGGTTAGTTGTGGCAATAAAAATGATTAAGGATACTAGTATA
AGAGATCAGGTTCGTCCTTTAGTGTTGTGTA 45 (0.001370%)

GTAAGCCAGAAGGGCATGCTCTCGGGTCTGCCCATTCTTGGAGATACCAGCTTCAAATTCACCAACACCA
GCAGCAACAATCAGGACAGCACAGTCAGCC 21 (0.000639%)

GTAAGGAGAAGATGGTTAGGTCTACGGAGGCTCCAGGGTGGGAGTAGTTCCCTGCTAAGGGAGGGTAG
ACTGTTCAACCTGTTCCTGCTCCGGCCTCCAC 8 (0.000244%)

GTAAGTGCTGACTTCCTTAACAATTTCCTCATATCTCTTCTGGCTGTAGGGTGGCTCAGTGGAATCCATTT
TGTTAACACCGACAATTAGTTGTTTCACA 8 (0.000244%)

GTAATCATGTTTTTGATAAAGTCTCTGTGTCCTGGGGCATCAATGATAGTCACATAGTACTTGCTGGTCTC
AAATTTCCACAAGGAGATATCAATGGTGA17 (0.000517%)

GTAATGTTAGCGGTTAGGCGTACGGCCAGGGCTATTGGTTGAATGAGTAGGCTGATGGTTTCGATAATA
ACTAGTATGGGGATAAGGGGTGTAGGTGTGC 38 (0.001157%)

GTACAAGGAAGGGGTAGGCTATGTGTTTTGTCAGGGGGTTGAGAATGAGTGTGAGGCGTATTATACCAT
AGCCGCCTAGTTTTAAGAGTACTGCGGCAAG 12 (0.000365%)

GTACCTTTCTCTTTGGCTTCTTTCTTTTTCTGATCATTTTCCTTCACACGTTTCAGGAAGCTATCTCGGCTC
TTAGAGTGCTTAATGTGCTCAATACGCA 7 (0.000213%)

GTACGGCCAGGGCTATTGGTTGAATGAGTAGGCTGATGGTTTCGATAATAACTAGTATGGGGATAAGGG
GTGTAGGTGTGCCTTGTGGTAAGAAGTGGGC 22 (0.000670%)

GTACTACTCGATTGTCAACGTCAAGGAGTCGCAGGTCGCCTGGTTCTAGGAATAATGGGGGAAGTATGT
AGGAGTTGAAGATTAGTCCGCCGTAGTCGGT 5 (0.000152%)

GTAGACATCCTGGAGAGGCAGGCGCAAGGGCTTGTCAGTTGGACGAGTTGGTGGTAGGATGCAGTCCAG
AGCCTCAAGCAGCGTGGTTCCACTGGCATTG 14 (0.000426%)

GTAGAGGGAGTATAGGGCTGTGACTAGTATGTTGAGTCCTGTAAGTAGGAGAGTGATATTTGATCAGGA
GAACGTGGTTACTAGCACAGAGAGTTCTCCC 6 (0.000183%)

GTAGATGTGGCGGGTTTTAGGGGCTCTTTGGTGAAGAGTTTTATGGCGTCAGCGAAGGGTTGTAGTAGCC
CGTAGGGGCCTACAACGTTGGGGCCTTTGC 9 (0.000274%)

GTAGCCAATTTTCTTAATGTAAGTGCTGACTTCCTTAACAATTTCCTCATATCTCTTCTGGCTGTAGGGTG
GCTCAGTGGAATCCATTTTGTTAACACCG 18 (0.000548%)

GTAGGATGCAGTCCAGAGCCTCAAGCAGCGTGGTTCCACTGGCATTGCCATCCTTACGGGTGACTTTCCA
TCCCTTGAACCAAGGCATGTTAGCACTTGG 75 (0.002283%)

GTAGGGTGGCTCAGTGGAATCCATTTTGTTAACACCGACAATTAGTTGTTTCACACCCAGTGTGTAAGCC
AGAAGGGCATGCTCTCGGGTCTGCCCATTC 16 (0.000487%)

GTAGGTGGCCTGCAGTAATGTTAGCGGTTAGGCGTACGGCCAGGGCTATTGGTTGAATGAGTAGGCTGA

TGGTTTCGATAATAACTAGTATGGGGATAAG 38 (0.001157%)

GTAGTGATGGACACCAGTTTTAGCCAACATAGCATAGTACTCTATTTCAGATTTCCTCAAAGCTGGGCAG
TTGTTAGCGAGAATGACCAATTTCGCTTTG 5 (0.000152%)

GTATTAGGGATAATATTCATTTAGCCTTCTGAGCTTTCTGGGCAGACTTGGTGACCTTGCCAGCTCCAGC
AGCCTTCTTGTCCACTGCTTTGATGACACC 9 (0.000274%)

GTCAGGCCACCTACGGTGAAAAGAAAGATGAATCCTAGGGCTCAGAGCACTGCAGCAGATCATTTCATA
TTGCTTCCGTGGAGTGTGGCGAGTCAGCTAA 14 (0.000426%)

GTCAGGTCCCGGTATTCCCGGTACATGTTGTGGGTGCCGCTCCGGGAGTCATAGCGCAGCCAGATCCCGA
AGTTCTTCACCCGCAGGGGGGACTTCTCAA 11 (0.000335%)

GTCAGTTCAGTGTTTTAATCTGACGCAGGCTTATGCGGAGGAGAATGTTTCATGTTACTTATACTAACA
TTAGTTCTTCTATAGGGTGATAGATTGGTC 359 (0.010928%)

GTCAGTTGGACGAGTTGGTGGTAGGATGCAGTCCAGAGCCTCAAGCAGCGTGGTTCCACTGGCATTGCC
ATCCTTACGGGTGACTTTCCATCCCTTGAAC 13 (0.000396%)

GTCATAAGTGGAGTCCGTAAAGAGGTATCTTTACTATAAAAGCTATTGTGTAAGCTAGTCATATTAAGTT
GTTGGCTCAGGAGTTTGATAGTTCTTGGGC 17 (0.000517%)

GTCATTTTTGCTGTCACCAGCAACGTTGCCACGACGAACATCCTTGACAGACACATTCTTGACATTGAAG
CCCACATTGTCCCCAGGAAGAGCTTCACTC 73 (0.002222%)

GTCCAATGATGGTAAAAGGGTAGCTTACTGGTTGTCCTCCGATTCAGGTTAGAATGAGGAGGTCTGCGG
CTAGGAGTCAATAAAGTGATTGGCTTAGTGG 5 (0.000152%)

GTCCCCAGGAAGAGCTTCACTCAAAGCTTCATGGTGCATTTCGACAGATTTTACTTCCGTTGTAACGTTG
ACTGGAGCAAAGGTGACCACCATACCGGGT 11 (0.000335%)

GTCCCCTTTCATTTTATAATAGAAAACCTTGGACTCGCCAGTGTTAGCTGCTGGAATGAGGTGTTTGTCC
AGTACATCCAGAATGTCACAACAGATTAAC 4 (0.000122%)

GTCCCGGTATTCCCGGTACATGTTGTGGGTGCCGCTCCGGGAGTCATAGCGCAGCCAGATCCCGAAGTTC
TTCACCCGCAGGGGGGACTTCTCAAACACC 3 (0.000091%)

GTCTGTTAGTAGTATAGTGATGCCAGCAGCTAGGACTGGGAGAGATAGGAGAAGTAGGACTGCTGTGAT
TAGGACGGATCAGACGAAGAGGGGCGTTTGG 13 (0.000396%)

GTGAAAAGAAAGATGAATCCTAGGGCTCAGAGCACTGCAGCAGATCATTTCATATTGCTTCCGTGGAGT
GTGGCGAGTCAGCTAAATACTTTGACGCCGG 9 (0.000274%)

GTGAAGCCAGCTGCTTCCATTGGTGGGTCATTTTTGCTGTCACCAGCAACGTTGCCACGACGAACATCCT
TGACAGACACATTCTTGACATTGAAGCCCA 15 (0.000457%)

GTGACCTTGCCAGCTCCAGCAGCCTTCTTGTCCACTGCTTTGATGACACCCACCGCAACTGTCTGTCTCAT
ATCACGAACAGCAAAGCGACCCAAAGGTG 14 (0.000426%)

GTGACTTCATATGAGATTGTTTGGGCTACTGCTCGCAGTGCGCCGATCAGGGCGTAGTTTGAGTTTGATG
CTCACCCTGATCAGAGGATTGAGTAAACGG 13 (0.000396%)

GTGATCGGCGATCAGAGGGCGATGAAGTTCTAGATCCATTGAGACAAGCTCTAGACAGTAGCATGCAGT
CCCACAACTTGTACCAGCATCCCCAGCGTCT 5 (0.000152%)

GTGATGGACACCAGTTTTAGCCAACATAGCATAGTACTCTATTTCAGATTTCCTCAAAGCTGGGCAGTTG
TTAGCGAGAATGACCAATTTCGCTTTGCCT 5 (0.000152%)

GTGCATGAGTAGGTGGCCTGCAGTAATGTTAGCGGTTAGGCGTACGGCCAGGGCTATTGGTTGAATGAG
TAGGCTGATGGTTTCGATAATAACTAGTATG 9 (0.000274%)

GTGCTGACTTCCTTAACAATTTCCTCATATCTCTTCTGGCTGTAGGGTGGCTCAGTGGAATCCATTTTGTT
AACACCGACAATTAGTTGTTTCACACCCA 27 (0.000822%)

GTGGAGTCCGTAAAGAGGTATCTTTACTATAAAAGCTATTGTGTAAGCTAGTCATATTAAGTTGTTGGCT
CAGGAGTTTGATAGTTCTTGGGCAGTGAGA 11 (0.000335%)

GTGGCAGGTATTAGGGATAATATTCATTTAGCCTTCTGAGCTTTCTGGGCAGACTTGGTGACCTTGCCAG
CTCCAGCAGCCTTCTTGTCCACTGCTTTGA5 (0.000152%)

GTGGCCTGCAGTAATGTTAGCGGTTAGGCGTACGGCCAGGGCTATTGGTTGAATGAGTAGGCTGATGGT
TTCGATAATAACTAGTATGGGGATAAGGGGT 20 (0.000609%)

GTGGCCTTGGTATGTGCTTTCTCGTGTTACATCGCGCCATCATTGGTATATGGTTAGTGTGTTGGTTAGTA
GGCCTAGTATGAGGAGCGTTATGGAGTGG88 (0.002679%)

GTGGCGCTTCCAATTAGGTGCATGAGTAGGTGGCCTGCAGTAATGTTAGCGGTTAGGCGTACGGCCAGG
GCTATTGGTTGAATGAGTAGGCTGATGGTTT 8 (0.000244%)

GTGGCTACAAAAAATGTTGAGCCGTAGATGCCGTCGGAAATGGTGAAGGGAGACTCGAAGTACTCTGAG
GCTTGTAGGAGGGTAAAATAGAGACCCAGTA 14 (0.000426%)

GTGGCTCAGTGGAATCCATTTTGTTAACACCGACAATTAGTTGTTTCACACCCAGTGTGTAAGCCAGAAG
GGCATGCTCTCGGGTCTGCCCATTCTTGGA 11 (0.000335%)

GTGGGAAGAAAGTTAGATTTACGCCGATGAATATGATAGTGAAATGGATTTTGGCGTAGGTTTGGTCTA
GGGTGTAGCCTGAGAATAGGGGAAATCAGTG 418 (0.012724%)

GTGGGGTGGCAGGTATTAGGGATAATATTCATTTAGCCTTCTGAGCTTTCTGGGCAGACTTGGTGACCTT
GCCAGCTCCAGCAGCCTTCTTGTCCACTGC 5 (0.000152%)

GTGGGTCATTTTTGCTGTCACCAGCAACGTTGCCACGACGAACATCCTTGACAGACACATTCTTGACATT
GAAGCCCACATTGTCCCCAGGAAGAGCTTC 14 (0.000426%)

GTGGTAGGATGCAGTCCAGAGCCTCAAGCAGCGTGGTTCCACTGGCATTGCCATCCTTACGGGTGACTTT
CCATCCCTTGAACCAAGGCATGTTAGCACT 50 (0.001522%)

GTGGTAGTCCCCTTTCATTTTATAATAGAAAACCTTGGACTCGCCAGTGTTAGCTGCTGGAATGAGGTGT
TTGTCCAGTACATCCAGAATGTCACAACAG 6 (0.000183%)

GTGGTTGGTGTAAATGAGTGAGGCAGGAGTCCGAGGAGGTTAGTTGTGGCAATAAAAATGATTAAGGAT
ACTAGTATAAGAGATCAGGTTCGTCCTTTAG 5 (0.000152%)

GTGTAAATGAGTGAGGCAGGAGTCCGAGGAGGTTAGTTGTGGCAATAAAAATGATTAAGGATACTAGTA
TAAGAGATCAGGTTCGTCCTTTAGTGTTGTG 49 (0.001492%)

GTGTAAGCCAGAAGGGCATGCTCTCGGGTCTGCCCATTCTTGGAGATACCAGCTTCAAATTCACCAACAC
CAGCAGCAACAATCAGGACAGCACAGTCAG 11 (0.000335%)

GTGTGTAAGCCAGAAGGGCATGCTCTCGGGTCTGCCCATTCTTGGAGATACCAGCTTCAAATTCACCAAC
ACCAGCAGCAACAATCAGGACAGCACAGTC 8 (0.000244%)

GTGTTACTTTAATTGGACTGCCTTCGTAATTCATTGCCTCTGCTTCAACAATGTGCAACTCATCCTTTGCA
CCAGCCCCTAAACTGACCGTTCTTAAAGA 3 (0.000091%)

GTTACTTTAATTGGACTGCCTTCGTAATTCATTGCCTCTGCTTCAACAATGTGCAACTCATCCTTTGCACC
AGCCCCTAAACTGACCGTTCTTAAAGATA 6 (0.000183%)

GTTAGATTTACGCCGATGAATATGATAGTGAAATGGATTTTGGCGTAGGTTTGGTCTAGGGTGTAGCCTG
AGAATAGGGGAAATCAGTGAATGAAGCCTC 51 (0.001552%)

GTTAGCGGTTAGGCGTACGGCCAGGGCTATTGGTTGAATGAGTAGGCTGATGGTTTCGATAATAACTAGT
ATGGGGATAAGGGGTGTAGGTGTGCCTTGT 17 (0.000517%)

GTTAGGCGTACGGCCAGGGCTATTGGTTGAATGAGTAGGCTGATGGTTTCGATAATAACTAGTATGGGG
ATAAGGGGTGTAGGTGTGCCTTGTGGTAAGA 20 (0.000609%)

GTTAGGGGGTCGGAGGAAAAGGTTGGGGAACAGCTAAATAGGTTGTTGTTGATTTGGTTAAAAAATAGT
AGAGGGATGATGCTAATAATTAGGCTGTGGG 6 (0.000183%)

GTTAGTATTAGGAGGGGGGTTGTTAGGGGGTCGGAGGAAAAGGTTGGGGAACAGCTAAATAGGTTGTTG
TTGATTTGGTTAAAAAATAGTAGAGGGATGA 12 (0.000365%)

GTTCAGTGTTTTAATCTGACGCAGGCTTATGCGGAGGAGAATGTTTTCATGTTACTTATACTAACATTAGT
TCTTCTATAGGGTGATAGATTGGTCCAAT 6 (0.000183%)

GTTCTTCCACCACTGATTAAGAGTGGGGTGGCAGGTATTAGGGATAATATTCATTTAGCCTTCTGAGCTT
TCTGGGCAGACTTGGTGACCTTGCCAGCTC 29 (0.000883%)

GTTGAACCCAGGTACCTTTCTCTTTGGCTTCTTTCTTTTTCTGATCATTTTCCTTCACACGTTTCAGGAAGC
TATCTCGGCTCTTAGAGTGCTTAATGTG 226 (0.006879%)

GTTGAATGAGTAGGCTGATGGTTTCGATAATAACTAGTATGGGGATAAGGGGTGTAGGTGTGCCTTGTG
GTAAGAAGTGGGCTAGGGCATTTTTAATCTT 15 (0.000457%)

GTTGATATTGCTAGGGTGGCGCTTCCAATTAGGTGCATGAGTAGGTGGCCTGCAGTAATGTTAGCGGTTA
GGCGTACGGCCAGGGCTATTGGTTGAATGA 722 (0.021978%)

GTTGCAAATTCTCATGGTTTGGGTTGGGTGGTGGAGAGCGCGTGTCATCTGCGGGTGGCACTGCCCACGG
TGGGCGGGCGGGCCTCTCTACTCGAAGGTG 3 (0.000091%)

GTTGGACGAGTTGGTGGTAGGATGCAGTCCAGAGCCTCAAGCAGCGTGGTTCCACTGGCATTGCCATCCT
TACGGGTGACTTTCCATCCCTTGAACCAAG 12 (0.000365%)

GTTGGGGAACAGCTAAATAGGTTGTTGTTGATTTGGTTAAAAAATAGTAGAGGGATGATGCTAATAATT
AGGCTGTGGGTGGTTGTGTTGATTCAAATTA 7 (0.000213%)

GTTGGGTGGTGGAGAGCGCGTGTCATCTGCGGGTGGCACTGCCCACGGTGGGCGGGCGGGCCTCTCTAC
TCGAAGGTGACCACGTTTAGATTCTGAGACG 8 (0.000244%)

GTTGGGTGGTTGGTGTAAATGAGTGAGGCAGGAGTCCGAGGAGGTTAGTTGTGGCAATAAAAATGATTA
AGGATACTAGTATAAGAGATCAGGTTCGTCC 11 (0.000335%)

GTTGGTGGTAGGATGCAGTCCAGAGCCTCAAGCAGCGTGGTTCCACTGGCATTGCCATCCTTACGGGTGA
CTTTCCATCCCTTGAACCAAGGCATGTTAG 24 (0.000731%)

GTTGGTGTAAATGAGTGAGGCAGGAGTCCGAGGAGGTTAGTTGTGGCAATAAAAATGATTAAGGATACT
AGTATAAGAGATCAGGTTCGTCCTTTAGTGT 13 (0.000396%)

GTTGTAGCCAATTTTCTTAATGTAAGTGCTGACTTCCTTAACAATTTCCTCATATCTCTTCTGGCTGTAGG
GTGGCTCAGTGGAATCCATTTTGTTAACA 1081 (0.032906%)

GTTGTTTCACACCCAGTGTGTAAGCCAGAAGGGCATGCTCTCGGGTCTGCCCATTCTTGGAGATACCAGC
TTCAAATTCACCAACACCAGCAGCAACAAT 553 (0.016833%)

GTTTATAGATAGTTGGGTGGTTGGTGTAAATGAGTGAGGCAGGAGTCCGAGGAGGTTAGTTGTGGCAAT

AAAAATGATTAAGGATACTAGTATAAGAGAT 88 (0.002679%)

GTTTCACACCCAGTGTGTAAGCCAGAAGGGCATGCTCTCGGGTCTGCCCATTCTTGGAGATACCAGCTTC
AAATTCACCAACACCAGCAGCAACAATCAG 7 (0.000213%)

GTTTGCTAATACAATGCCAGTCAGGCCACCTACGGTGAAAAGAAAGATGAATCCTAGGGCTCAGAGCAC
TGCAGCAGATCATTTCATATTGCTTCCGTGG 569 (0.017320%)

GTTTGGGTTGGGTGGTGGAGAGCGCGTGTCATCTGCGGGTGGCACTGCCCACGGTGGGCGGGCGGGCCT
CTCTACTCGAAGGTGACCACGTTTAGATTCT 35 (0.001065%)

GTTTTAGCCAACATAGCATAGTACTCTATTTCAGATTTCCTCAAAGCTGGGCAGTTGTTAGCGAGAATGA
CCAATTTCGCTTTGCCTTGTCTGATCATCT 11 (0.000335%)

GTTTTCTATTAGACTATGGTGAGCTCAGGTGATTGATACTCCTGATGCGAGTAATACGGATGTGTTTAGG
AGTGGGACTTCTAGGGGATTTAGCGGGGTG 72 (0.002192%)

GTTTTTAAGCCTAATGTGGGGACAGCTCATGAGTGCAAGACGTCTTGTGATGTAATTATTATACGAATGG
GGGCTTCAATCGGGAGTACTACTCGATTGT 23 (0.000700%)

GTTTTTGATAAAGTCTCTGTGTCCTGGGGCATCAATGATAGTCACATAGTACTTGCTGGTCTCAAATTTCC
ACAAGGAGATATCAATGGTGATACCACGT49 (0.001492%)

TAAAAACTAATAACTTAAAACTGCCACACGCAAAAAAGAAAACCAAAGTGGTCCACAAAACATTCTCCT
TTCCTTCTGAAGGTTTTACGATGCATTGTTA 11 (0.000335%)

TAAAAAGTACTGATTTTAAAAACTAATAACTTAAAACTGCCACACGCAAAAAAGAAAACCAAAGTGGTC
CACAAAACATTCTCCTTTCCTTCTGAAGGTT 10 (0.000304%)

TAAAATAGAGACCCAGTAAAATTGTAATAAGCAGTGCTTGAATTATTTGGTTTCGGTTGTTTTCTATTAG
ACTATGGTGAGCTCAGGTGATTGATACTCC 8 (0.000244%)

TAAAATTGTAATAAGCAGTGCTTGAATTATTTGGTTTCGGTTGTTTTCTATTAGACTATGGTGAGCTCAGG
TGATTGATACTCCTGATGCGAGTAATACG 12 (0.000365%)

TAAAGAGGTATCTTTACTATAAAAGCTATTGTGTAAGCTAGTCATATTAAGTTGTTGGCTCAGGAGTTTG
ATAGTTCTTGGGCAGTGAGAGTGAGTAGTA 12 (0.000365%)

TAAATGAGTGAGGCAGGAGTCCGAGGAGGTTAGTTGTGGCAATAAAAATGATTAAGGATACTAGTATAA
GAGATCAGGTTCGTCCTTTAGTGTTGTGTAT 11 (0.000335%)

TAACAATTTCCTCATATCTCTTCTGGCTGTAGGGTGGCTCAGTGGAATCCATTTTGTTAACACCGACAATT
AGTTGTTTCACACCCAGTGTGTAAGCCAG 26 (0.000791%)

TAAGAGTGGGGTGGCAGGTATTAGGGATAATATTCATTTAGCCTTCTGAGCTTTCTGGGCAGACTTGGTG
ACCTTGCCAGCTCCAGCAGCCTTCTTGTCC 3 (0.000091%)

TAAGATAGAGGAGACACCTGCTAGGTGTAAGGAGAAGATGGTTAGGTCTACGGAGGCTCCAGGGTGGG
AGTAGTTCCCTGCTAAGGGAGGGTAGACTGTT 4 (0.000122%)

TAATAACTTAAAACTGCCACACGCAAAAAAGAAAACCAAAGTGGTCCACAAAACATTCTCCTTTCCTTCT
GAAGGTTTTACGATGCATTGTTATCATTAA 12 (0.000365%)

TAATACAATGCCAGTCAGGCCACCTACGGTGAAAAGAAAGATGAATCCTAGGGCTCAGAGCACTGCAGC
AGATCATTTCATATTGCTTCCGTGGAGTGTG 3 (0.000091%)

TAATATTCATTTAGCCTTCTGAGCTTTCTGGGCAGACTTGGTGACCTTGCCAGCTCCAGCAGCCTTCTTGT
CCACTGCTTTGATGACACCCACCGCAACT 13 (0.000396%)

TAATCACCTGAGCAGTGAAGCCAGCTGCTTCCATTGGTGGGTCATTTTGCTGTCACCAGCAACGTTGCC
ACGACGAACATCCTTGACAGACACATTCTT 8 (0.000244%)

TAATCATGTTTTTGATAAAGTCTCTGTGTCCTGGGGCATCAATGATAGTCACATAGTACTTGCTGGTCTCA
AATTTCCACAAGGAGATATCAATGGTGAT 3 (0.000091%)

TAATGTAAGTGCTGACTTCCTTAACAATTTCCTCATATCTCTTCTGGCTGTAGGGTGGCTCAGTGGAATCC
ATTTTGTTAACACCGACAATTAGTTGTTT 3 (0.000091%)

TACAAGGAAGGGGTAGGCTATGTGTTTTGTCAGGGGGTTGAGAATGAGTGTGAGGCGTATTATACCATA
GCCGCCTAGTTTTAAGAGTACTGCGGCAAGT 3 (0.000091%)

TACACATGAGTATTTGTCTAAAACATGTCTTCTTTGTAGCAGCTAGGCCCTGCCACCACTGTGCTTGGCTG
AGTTCACAAATCTGTTGTAACCTGTAGCT 6 (0.000183%)

TACCTTTCTCTTTGGCTTCTTTCTTTTTCTGATCATTTTCCTTCACACGTTTCAGGAAGCTATCTCGGCTCTT
AGAGTGCTTAATGTGCTCAATACGCAC 3 (0.000091%)

TACGAATGGGGGCTTCAATCGGGAGTACTACTCGATTGTCAACGTCAAGGAGTCGCAGGTCGCCTGGTT
CTAGGAATAATGGGGGAAGTATGTAGGAGTT 8 (0.000244%)

TACGGTGAAAAGAAAGATGAATCCTAGGGCTCAGAGCACTGCAGCAGATCATTTCATATTGCTTCCGTG
GAGTGTGGCGAGTCAGCTAAATACTTTGACG 3 (0.000091%)

TACTGATTTTAAAAACTAATAACTTAAAACTGCCACACGCAAAAAAGAAAACCAAAGTGGTCCACAAAA
CATTCTCCTTTCCTTCTGAAGGTTTTACGAT 3 (0.000091%)

TAGACATCCTGGAGAGGCAGGCGCAAGGGCTTGTCAGTTGGACGAGTTGGTGGTAGGATGCAGTCCAGA
GCCTCAAGCAGCGTGGTTCCACTGGCATTGC 19 (0.000578%)

TAGAGACCCAGTAAAATTGTAATAAGCAGTGCTTGAATTATTTGGTTTCGGTTGTTTTCTATTAGACTATG
GTGAGCTCAGGTGATTGATACTCCTGATG 26 (0.000791%)

TAGATAGTTGGGTGGTTGGTGTAAATGAGTGAGGCAGGAGTCCGAGGAGGTTAGTTGTGGCAATAAAAA
TGATTAAGGATACTAGTATAAGAGATCAGGT 4 (0.000122%)

TAGATTTACGCCGATGAATATGATAGTGAAATGGATTTTGGCGTAGGTTTGGTCTAGGGTGTAGCCTGAG
AATAGGGGAAATCAGTGAATGAAGCCTCCT 7 (0.000213%)

TAGCCAATTTTCTTAATGTAAGTGCTGACTTCCTTAACAATTTCCTCATATCTCTTCTGGCTGTAGGGTGG
CTCAGTGGAATCCATTTTGTTAACACCGA 3 (0.000091%)

TAGCCTTCTGAGCTTTCTGGGCAGACTTGGTGACCTTGCCAGCTCCAGCAGCCTTCTTGTCCACTGCTTTG
ATGACACCCACCGCAACTGTCTGTCTCAT 19 (0.000578%)

TAGGCGTACGGCCAGGGCTATTGGTTGAATGAGTAGGCTGATGGTTTCGATAATAACTAGTATGGGGAT
AAGGGGTGTAGGTGTGCCTTGTGGTAAGAAG 11 (0.000335%)

TAGGGAGTCATAAGTGGAGTCCGTAAAGAGGTATCTTTACTATAAAAGCTATTGTGTAAGCTAGTCATAT
TAAGTTGTTGGCTCAGGAGTTTGATAGTTC 5 (0.000152%)

TAGGGATAATATTCATTTAGCCTTCTGAGCTTTCTGGGCAGACTTGGTGACCTTGCCAGCTCCAGCAGCC
TTCTTGTCCACTGCTTTGATGACACCCACC20 (0.000609%)

TAGGGATAGTACAAGGAAGGGGTAGGCTATGTGTTTTGTCAGGGGGTTGAGAATGAGTGTGAGGCGTAT
TATACCATAGCCGCCTAGTTTTAAGAGTACT 10 (0.000304%)

TAGGGCTCAGAGCACTGCAGCAGATCATTTCATATTGCTTCCGTGGAGTGTGGCGAGTCAGCTAAATACT
TTGACGCCGGTGGGGATAGCGATGATTATG 13 (0.000396%)

TAGGTGGCCTGCAGTAATGTTAGCGGTTAGGCGTACGGCCAGGGCTATTGGTTGAATGAGTAGGCTGAT
GGTTTCGATAATAACTAGTATGGGGATAAGG 13 (0.000396%)

TAGGTTTATAGATAGTTGGGTGGTTGGTGTAAATGAGTGAGGCAGGAGTCCGAGGAGGTTAGTTGTGGC
AATAAAAATGATTAAGGATACTAGTATAAGA 17 (0.000517%)

TAGTTGGGTGGTTGGTGTAAATGAGTGAGGCAGGAGTCCGAGGAGGTTAGTTGTGGCAATAAAAATGAT
TAAGGATACTAGTATAAGAGATCAGGTTCGT 3 (0.000091%)

TATACGAATGGGGGCTTCAATCGGGAGTACTACTCGATTGTCAACGTCAAGGAGTCGCAGGTCGCCTGG
TTCTAGGAATAATGGGGGAAGTATGTAGGAG 6 (0.000183%)

TATAGATAGTTGGGTGGTTGGTGTAAATGAGTGAGGCAGGAGTCCGAGGAGGTTAGTTGTGGCAATAAA
AATGATTAAGGATACTAGTATAAGAGATCAG 23 (0.000700%)

TATCTTTACTATAAAAGCTATTGTGTAAGCTAGTCATATTAAGTTGTTGGCTCAGGAGTTTGATAGTTCTT
GGGCAGTGAGAGTGAGTAGTAGAATGTTT4 (0.000122%)

TATTAGACTATGGTGAGCTCAGGTGATTGATACTCCTGATGCGAGTAATACGGATGTGTTTAGGAGTGGG
ACTTCTAGGGGATTTAGCGGGGTGATGCCT 9 (0.000274%)

TATTATACGAATGGGGGCTTCAATCGGGAGTACTACTCGATTGTCAACGTCAAGGAGTCGCAGGTCGCCT
GGTTCTAGGAATAATGGGGGAAGTATGTAG 10 (0.000304%)

TATTCATTTAGCCTTCTGAGCTTTCTGGGCAGACTTGGTGACCTTGCCAGCTCCAGCAGCCTTCTTGTCCA
CTGCTTTGATGACACCCACCGCAACTGTC 37 (0.001126%)

TATTGCTAGGGTGGCGCTTCCAATTAGGTGCATGAGTAGGTGGCCTGCAGTAATGTTAGCGGTTAGGCGT
ACGGCCAGGGCTATTGGTTGAATGAGTAGG 12 (0.000365%)

TATTGGTTGAATGAGTAGGCTGATGGTTTCGATAATAACTAGTATGGGGATAAGGGGTGTAGGTGTGCCT
TGTGGTAAGAAGTGGGCTAGGGCATTTTTA 18 (0.000548%)

TATTTGTCTAAAACATGTCTTCTTTGTAGCAGCTAGGCCCTGCCACCACTGTGCTTGGCTGAGTTCACAAA
TCTGTTGTAACCTGTAGCTTCCCTGTCAC 5 (0.000152%)

TCAACACACATGGGCTTGCCAGGAACCATATCAACAATGGCAGCATCACCAGACTTCAAGAATTTAGGG
CCATCTTCCAGCTTTTTACCAGAACGGCGAT 3 (0.000091%)

TCAATCGGGAGTACTACTCGATTGTCAACGTCAAGGAGTCGCAGGTCGCCTGGTTCTAGGAATAATGGG
GGAAGTATGTAGGAGTTGAAGATTAGTCCGC 6 (0.000183%)

TCACACCCAGTGTGTAAGCCAGAAGGGCATGCTCTCGGGTCTGCCCATTCTTGGAGATACCAGCTTCAAA
TTCACCAACACCAGCAGCAACAATCAGGAC 5 (0.000152%)

TCACTCAAAGCTTCATGGTGCATTTCGACAGATTTTACTTCCGTTGTAACGTTGACTGGAGCAAAGGTGA
CCACCATACCGGGTTTGAGAACACCAGTCT 3 (0.000091%)

TCAGAGCACTGCAGCAGATCATTTCATATTGCTTCCGTGGAGTGTGGCGAGTCAGCTAAATACTTTGACG
CCGGTGGGGATAGCGATGATTATGGTAGCG 6 (0.000183%)

TCAGTGGAATCCATTTTGTTAACACCGACAATTAGTTGTTTCACACCCAGTGTGTAAGCCAGAAGGGCAT
GCTCTCGGGTCTGCCCATTCTTGGAGATAC 9 (0.000274%)

TCATATCTCTTCTGGCTGTAGGGTGGCTCAGTGGAATCCATTTTGTTAACACCGACAATTAGTTGTTTCAC
ACCCAGTGTGTAAGCCAGAAGGGCATGCT 6 (0.000183%)

TCATGGTTTGGGTTGGGTGGTGGAGAGCGCGTGTCATCTGCGGGTGGCACTGCCCACGGTGGGCGGGCG

GGCCTCTCTACTCGAAGGTGACCACGTTTAG 3 (0.000091%)

TCATGTTTTTGATAAAGTCTCTGTGTCCTGGGGCATCAATGATAGTCACATAGTACTTGCTGGTCTCAAAT
TTCCACAAGGAGATATCAATGGTGATACC 4 (0.000122%)

TCATTTAGCCTTCTGAGCTTTCTGGGCAGACTTGGTGACCTTGCCAGCTCCAGCAGCCTTCTTGTCCACTG
CTTTGATGACACCCACCGCAACTGTCTGT 5 (0.000152%)

TCATTTTTGCTGTCACCAGCAACGTTGCCACGACGAACATCCTTGACAGACACATTCTTGACATTGAAGC
CCACATTGTCCCCAGGAAGAGCTTCACTCA 4 (0.000122%)

TCCAAGACCCAGGCATACTTGAAGGAGCCCTTTCCCATCTCAGCAGCCTCCTTCTCAAATTTTTCAATGG
TTCTTTTGTCGATGCCACCGCATTTATAGA5 (0.000152%)

TCCAGAACTACTGCCTTCACCATGAAGCTCCATGAGCTTTCCCAATTCAAACTTGGGCTTCTTCAGCATTT
TTACTTTTCTAACGAAGACATCATGGAGA 422 (0.012846%)

TCCATTAAAAAGTACTGATTTTAAAAACTAATAACTTAAAACTGCCACACGCAAAAAAGAAAACCAAAG
TGGTCCACAAAACATTCTCCTTTCCTTCTGA 12 (0.000365%)

TCCATTGGTGGGTCATTTTTGCTGTCACCAGCAACGTTGCCACGACGAACATCCTTGACAGACACATTCT
TGACATTGAAGCCCACATTGTCCCCAGGAA 3 (0.000091%)

TCCCCAGGAAGAGCTTCACTCAAAGCTTCATGGTGCATTTCGACAGATTTTACTTCCGTTGTAACGTTGA
CTGGAGCAAAGGTGACCACCATACCGGGTT 3 (0.000091%)

TCCCGGTATTCCCGGTACATGTTGTGGGTGCCGCTCCGGGAGTCATAGCGCAGCCAGATCCCGAAGTTCT
TCACCCGCAGGGGGGACTTCTCAAACACCT 3 (0.000091%)

TCCGAAGCCTGGTAGGATAAGAATATAAACTTCAGGGTGACCGAAAAATCAGAATAGGTGTTGGTATAG
AATGGGGTCTCCTCCTCCGGCGGGGTCGAAG 678 (0.020638%)

TCCTCATATCTCTTCTGGCTGTAGGGTGGCTCAGTGGAATCCATTTTGTTAACACCGACAATTAGTTGTTT
CACACCCAGTGTGTAAGCCAGAAGGGCAT 22 (0.000670%)

TCCTCTCCTGCTAAGCTTTGTTTCCTAATTAAAATCTTCTGCCACTGCCATAGCTACTGCTGCTGCTGGAA
CCGCCATAGCCACCTTGGTTTCGTGGTTT 226 (0.006879%)

TCCTGGAGAGGCAGGCGCAAGGGCTTGTCAGTTGGACGAGTTGGTGGTAGGATGCAGTCCAGAGCCTCA
AGCAGCGTGGTTCCACTGGCATTGCCATCCT 12 (0.000365%)

TCCTTAACAATTTCCTCATATCTCTTCTGGCTGTAGGGTGGCTCAGTGGAATCCATTTTGTTAACACCGAC
AATTAGTTGTTTCACACCCAGTGTGTAAG 8 (0.000244%)

TCCTTTGCTACTGGTCCTGTAATGGCAGAACCTTTCATCTCGCCTTTATTGTTCACTATGACTCCTGCATT
ATCTTCAAAATAAAGAAACACGCCATCTT 6 (0.000183%)

TCGACATGGGCTTTAGGGAGTCATAAGTGGAGTCCGTAAAGAGGTATCTTTACTATAAAAGCTATTGTGT
AAGCTAGTCATATTAAGTTGTTGGCTCAGG 5 (0.000152%)

TCGGAGGAAAAGGTTGGGGAACAGCTAAATAGGTTGTTGTTGATTTGGTTAAAAAATAGTAGAGGGATG
ATGCTAATAATTAGGCTGTGGGTGGTTGTGT 5 (0.000152%)

TCGGGAGTACTACTCGATTGTCAACGTCAAGGAGTCGCAGGTCGCCTGGTTCTAGGAATAATGGGGGAA
GTATGTAGGAGTTGAAGATTAGTCCGCCGTA 3 (0.000091%)

TCTCAACACACATGGGCTTGCCAGGAACCATATCAACAATGGCAGCATCACCAGACTTCAAGAATTTAG
GGCCATCTTCCAGCTTTTTACCAGAACGGCG 28 (0.000852%)

TCTCGGGTCTGCCCATTCTTGGAGATACCAGCTTCAAATTCACCAACACCAGCAGCAACAATCAGGACA
GCACAGTCAGCCTGAGATGTCCCTGTAATCA 40 (0.001218%)

TCTGAGCTTTCTGGGCAGACTTGGTGACCTTGCCAGCTCCAGCAGCCTTCTTGTCCACTGCTTTGATGACA
CCCACCGCAACTGTCTGTCTCATATCACG 8 (0.000244%)

TCTGGCTGTAGGGTGGCTCAGTGGAATCCATTTTGTTAACACCGACAATTAGTTGTTTCACACCCAGTGT
GTAAGCCAGAAGGGCATGCTCTCGGGTCTG 30 (0.000913%)

TCTGGGCAGACTTGGTGACCTTGCCAGCTCCAGCAGCCTTCTTGTCCACTGCTTTGATGACACCCACCGC
AACTGTCTGTCTCATATCACGAACAGCAAA 6 (0.000183%)

TCTGTGACAAATTTTTGGTCAAGTTGTTTCCATTAAAAAGTACTGATTTTAAAAACTAATAACTTAAAAC
TGCCACACGCAAAAAAGAAAACCAAAGTGG 21 (0.000639%)

TCTGTTAGTAGTATAGTGATGCCAGCAGCTAGGACTGGGAGAGATAGGAGAAGTAGGACTGCTGTGATT
AGGACGGATCAGACGAAGAGGGGCGTTTGGT 11 (0.000335%)

TCTGTTTTTAAGCCTAATGTGGGGACAGCTCATGAGTGCAAGACGTCTTGTGATGTAATTATTATACGAA
TGGGGGCTTCAATCGGGAGTACTACTCGAT 7 (0.000213%)

TCTTAATGTAAGTGCTGACTTCCTTAACAATTTCCTCATATCTCTTCTGGCTGTAGGGTGGCTCAGTGGAA
TCCATTTTGTTAACACCGACAATTAGTTG 20 (0.000609%)

TCTTCCACCACTGATTAAGAGTGGGGTGGCAGGTATTAGGGATAATATTCATTTAGCCTTCTGAGCTTTC
TGGGCAGACTTGGTGACCTTGCCAGCTCCA 6 (0.000183%)

TCTTCTGGCTGTAGGGTGGCTCAGTGGAATCCATTTTGTTAACACCGACAATTAGTTGTTTCACACCCAGT
GTGTAAGCCAGAAGGGCATGCTCTCGGGT4 (0.000122%)

TGAAAAGAAAGATGAATCCTAGGGCTCAGAGCACTGCAGCAGATCATTTCATATTGCTTCCGTGGAGTG
TGGCGAGTCAGCTAAATACTTTGACGCCGGT 11 (0.000335%)

TGAAATTGATGGCCCCTAAGATAGAGGAGACACCTGCTAGGTGTAAGGAGAAGATGGTTAGGTCTACGG
AGGCTCCAGGGTGGGAGTAGTTCCCTGCTAA 25 (0.000761%)

TGAAGCCAGCTGCTTCCATTGGTGGGTCATTTTTGCTGTCACCAGCAACGTTGCCACGACGAACATCCTT
GACAGACACATTCTTGACATTGAAGCCCAC 5 (0.000152%)

TGAATCCTAGGGCTCAGAGCACTGCAGCAGATCATTTCATATTGCTTCCGTGGAGTGTGGCGAGTCAGCT
AAATACTTTGACGCCGGTGGGGATAGCGAT 3 (0.000091%)

TGAATGAGTAGGCTGATGGTTTCGATAATAACTAGTATGGGGATAAGGGGTGTAGGTGTGCCTTGTGGT
AAGAAGTGGGCTAGGGCATTTTTAATCTTAG 38 (0.001157%)

TGACCTTGCCAGCTCCAGCAGCCTTCTTGTCCACTGCTTTGATGACACCCACCGCAACTGTCTGTCTCATA
TCACGAACAGCAAAGCGACCCAAAGGTGG 4 (0.000122%)

TGACTTCATATGAGATTGTTTGGGCTACTGCTCGCAGTGCGCCGATCAGGGCGTAGTTTGAGTTTGATGC
TCACCCTGATCAGAGGATTGAGTAAACGGC 4 (0.000122%)

TGACTTCCTTAACAATTTCCTCATATCTCTTCTGGCTGTAGGGTGGCTCAGTGGAATCCATTTTGTTAACA
CCGACAATTAGTTGTTTCACACCCAGTGT 13 (0.000396%)

TGAGAAGCTCTCAACACACATGGGCTTGCCAGGAACCATATCAACAATGGCAGCATCACCAGACTTCAA
GAATTTAGGGCCATCTTCCAGCTTTTTACCA 26 (0.000791%)

TGAGACCGTTCTTCCACCACTGATTAAGAGTGGGGTGGCAGGTATTAGGGATAATATTCATTTAGCCTTC
TGAGCTTTCTGGGCAGACTTGGTGACCTTG 1026 (0.031231%)

TGAGCAGTGAAGCCAGCTGCTTCCATTGGTGGGTCATTTTGCTGTCACCAGCAACGTTGCCACGACGAA
CATCCTTGACAGACACATTCTTGACATTGA 12 (0.000365%)

TGAGCTTTCTGGGCAGACTTGGTGACCTTGCCAGCTCCAGCAGCCTTCTTGTCCACTGCTTTGATGACACC
CACCGCAACTGTCTGTCTCATATCACGAA 23 (0.000700%)

TGAGTATTTGTCTAAAACATGTCTTCTTTGTAGCAGCTAGGCCCTGCCACCACTGTGCTTGGCTGAGTTCA
CAAATCTGTTGTAACCTGTAGCTTCCCTG 14 (0.000426%)

TGAGTGAGGCAGGAGTCCGAGGAGGTTAGTTGTGGCAATAAAAATGATTAAGGATACTAGTATAAGAG
ATCAGGTTCGTCCTTTAGTGTTGTGTATGGTT 5 (0.000152%)

TGATATTGCTAGGGTGGCGCTTCCAATTAGGTGCATGAGTAGGTGGCCTGCAGTAATGTTAGCGGTTAGG
CGTACGGCCAGGGCTATTGGTTGAATGAGT 22 (0.000670%)

TGATCGGCGATCAGAGGGCGATGAAGTTCTAGATCCATTGAGACAAGCTCTAGACAGTAGCATGCAGTC
CCACAACTTGTACCAGCATCCCCAGCGTCTG 7 (0.000213%)

TGATGGCCCCTAAGATAGAGGAGACACCTGCTAGGTGTAAGGAGAAGATGGTTAGGTCTACGGAGGCTC
CAGGGTGGGAGTAGTTCCCTGCTAAGGGAGG 7 (0.000213%)

TGATGGCTAGGGTGACTTCATATGAGATTGTTTGGGCTACTGCTCGCAGTGCGCCGATCAGGGCGTAGTT
TGAGTTTGATGCTCACCCTGATCAGAGGAT 3 (0.000091%)

TGATTTTAAAAACTAATAACTTAAAACTGCCACACGCAAAAAAGAAAACCAAAGTGGTCCACAAAACAT
TCTCCTTTCCTTCTGAAGGTTTTACGATGCA 27 (0.000822%)

TGCAAATTCTCATGGTTTGGGTTGGGTGGTGGAGAGCGCGTGTCATCTGCGGGTGGCACTGCCCACGGTG
GGCGGGCGGGCCTCTCTACTCGAAGGTGAC 35 (0.001065%)

TGCACACTCCTTTGCTACTGGTCCTGTAATGGCAGAACCTTTCATCTCGCCTTTATTGTTCACTATGACTC
CTGCATTATCTTCAAAATAAAGAAACACG 3 (0.000091%)

TGCAGCAGATCATTTCATATTGCTTCCGTGGAGTGTGGCGAGTCAGCTAAATACTTTGACGCCGGTGGGG
ATAGCGATGATTATGGTAGCGGAGGTGAAA 7 (0.000213%)

TGCAGTAATGTTAGCGGTTAGGCGTACGGCCAGGGCTATTGGTTGAATGAGTAGGCTGATGGTTTCGATA
ATAACTAGTATGGGGATAAGGGGTGTAGGT 16 (0.000487%)

TGCAGTCCAGAGCCTCAAGCAGCGTGGTTCCACTGGCATTGCCATCCTTACGGGTGACTTTCCATCCCTT
GAACCAAGGCATGTTAGCACTTGGCTCCAG 14 (0.000426%)

TGCATCTGTTTTTAAGCCTAATGTGGGGACAGCTCATGAGTGCAAGACGTCTTGTGATGTAATTATTATA
CGAATGGGGGCTTCAATCGGGAGTACTACT 14 (0.000426%)

TGCATGAGTAGGTGGCCTGCAGTAATGTTAGCGGTTAGGCGTACGGCCAGGGCTATTGGTTGAATGAGT
AGGCTGATGGTTTCGATAATAACTAGTATGG 38 (0.001157%)

TGCCACACGCAAAAAAGAAAACCAAAGTGGTCCACAAAACATTCTCCTTTCCTTCTGAAGGTTTTACGAT
GCATTGTTATCATTAACCAGTCTTTTACTA 21 (0.000639%)

TGCCAGCTCCAGCAGCCTTCTTGTCCACTGCTTTGATGACACCCACCGCAACTGTCTGTCTCATATCACGA
ACAGCAAAGCGACCCAAAGGTGGATAGTC 5 (0.000152%)

TGCCAGTCAGGCCACCTACGGTGAAAAGAAAGATGAATCCTAGGGCTCAGAGCACTGCAGCAGATCATT
TCATATTGCTTCCGTGGAGTGTGGCGAGTCA 7 (0.000213%)

TGCCATCCTTACGGGTGACTTTCCATCCCTTGAACCAAGGCATGTTAGCACTTGGCTCCAGCATGTTGTC

ACCATTCCAACCAGAAATTGGCACAAATGC 8 (0.000244%)

TGCCCATACACATGAGTATTTGTCTAAAACATGTCTTCTTTGTAGCAGCTAGGCCCTGCCACCACTGTGCT
TGGCTGAGTTCACAAATCTGTTGTAACCT 11 (0.000335%)

TGCTAATACAATGCCAGTCAGGCCACCTACGGTGAAAAGAAAGATGAATCCTAGGGCTCAGAGCACTGC
AGCAGATCATTTCATATTGCTTCCGTGGAGT 41 (0.001248%)

TGCTACTTGTCCAATGATGGTAAAAGGGTAGCTTACTGGTTGTCCTCCGATTCAGGTTAGAATGAGGAGG
TCTGCGGCTAGGAGTCAATAAAGTGATTGG 4 (0.000122%)

TGCTAGGGTGGCGCTTCCAATTAGGTGCATGAGTAGGTGGCCTGCAGTAATGTTAGCGGTTAGGCGTAC
GGCCAGGGCTATTGGTTGAATGAGTAGGCTG 3 (0.000091%)

TGCTAGGTGTAAGGAGAAGATGGTTAGGTCTACGGAGGCTCCAGGGTGGGAGTAGTTCCCTGCTAAGGG
AGGGTAGACTGTTCAACCTGTTCCTGCTCCG 6 (0.000183%)

TGCTCTCGGGTCTGCCCATTCTTGGAGATACCAGCTTCAAATTCACCAACACCAGCAGCAACAATCAGGA
CAGCACAGTCAGCCTGAGATGTCCCTGTAA 9 (0.000274%)

TGCTGACTTCCTTAACAATTTCCTCATATCTCTTCTGGCTGTAGGGTGGCTCAGTGGAATCCATTTTGTTA
ACACCGACAATTAGTTGTTTCACACCCAG 36 (0.001096%)

TGCTTCCATTGGTGGGTCATTTTTGCTGTCACCAGCAACGTTGCCACGACGAACATCCTTGACAGACACA
TTCTTGACATTGAAGCCCACATTGTCCCCA 9 (0.000274%)

TGGACACCAGTTTTAGCCAACATAGCATAGTACTCTATTTCAGATTTCCTCAAAGCTGGGCAGTTGTTAG
CGAGAATGACCAATTTCGCTTTGCCTTGTC 16 (0.000487%)

TGGACGAGTTGGTGGTAGGATGCAGTCCAGAGCCTCAAGCAGCGTGGTTCCACTGGCATTGCCATCCTTA
CGGGTGACTTTCCATCCCTTGAACCAAGGC 31 (0.000944%)

TGGAGAGGCAGGCGCAAGGGCTTGTCAGTTGGACGAGTTGGTGGTAGGATGCAGTCCAGAGCCTCAAGC
AGCGTGGTTCCACTGGCATTGCCATCCTTAC 31 (0.000944%)

TGGAGTGGTAAAAGGCTCAGAAAAATCCTGCGAAGAAAAAAACTTCTGAGGTAATAAATAGGATTATCC
CGTATCGAAGGCCTTTTTGGACAGGTGGTGT 15 (0.000457%)

TGGCAGGTATTAGGGATAATATTCATTTAGCCTTCTGAGCTTTCTGGGCAGACTTGGTGACCTTGCCAGC
TCCAGCAGCCTTCTTGTCCACTGCTTTGAT 9 (0.000274%)

TGGCATTGCCATCCTTACGGGTGACTTTCCATCCCTTGAACCAAGGCATGTTAGCACTTGGCTCCAGCAT
GTTGTCACCATTCCAACCAGAAATTGGCAC 243 (0.007397%)

TGGCCCCTAAGATAGAGGAGACACCTGCTAGGTGTAAGGAGAAGATGGTTAGGTCTACGGAGGCTCCAG
GGTGGGAGTAGTTCCCTGCTAAGGGAGGGTA 3 (0.000091%)

TGGCCTGCAGTAATGTTAGCGGTTAGGCGTACGGCCAGGGCTATTGGTTGAATGAGTAGGCTGATGGTTT
CGATAATAACTAGTATGGGGATAAGGGGTG 3 (0.000091%)

TGGCGCTTCCAATTAGGTGCATGAGTAGGTGGCCTGCAGTAATGTTAGCGGTTAGGCGTACGGCCAGGG
CTATTGGTTGAATGAGTAGGCTGATGGTTTC 7 (0.000213%)

TGGCTAGGTTTATAGATAGTTGGGTGGTTGGTGTAAATGAGTGAGGCAGGAGTCCGAGGAGGTTAGTTG
TGGCAATAAAAATGATTAAGGATACTAGTAT 11 (0.000335%)

TGGCTGTAGGGTGGCTCAGTGGAATCCATTTGTTAACACCGACAATTAGTTGTTTCACACCCAGTGTGT
AAGCCAGAAGGGCATGCTCTCGGGTCTGCC 16 (0.000487%)

TGGGAAGAAAGTTAGATTTACGCCGATGAATATGATAGTGAAATGGATTTTGGCGTAGGTTTGGTCTAG
GGTGTAGCCTGAGAATAGGGGAAATCAGTGA 5 (0.000152%)

TGGGCAGACTTGGTGACCTTGCCAGCTCCAGCAGCCTTCTTGTCCACTGCTTTGATGACACCCACCGCAA
CTGTCTGTCTCATATCACGAACAGCAAAGC 7 (0.000213%)

TGGGCTTTAGGGAGTCATAAGTGGAGTCCGTAAAGAGGTATCTTTACTATAAAAGCTATTGTGTAAGCTA
GTCATATTAAGTTGTTGGCTCAGGAGTTTG 16 (0.000487%)

TGGGGAACAGCTAAATAGGTTGTTGTTGATTTGGTTAAAAAATAGTAGAGGGATGATGCTAATAATTAG
GCTGTGGGTGGTTGTGTTGATTCAAATTATG 5 (0.000152%)

TGGGGGCTTCAATCGGGAGTACTACTCGATTGTCAACGTCAAGGAGTCGCAGGTCGCCTGGTTCTAGGA
ATAATGGGGGAAGTATGTAGGAGTTGAAGAT 11 (0.000335%)

TGGGGTGGCAGGTATTAGGGATAATATTCATTTAGCCTTCTGAGCTTTCTGGGCAGACTTGGTGACCTTG
CCAGCTCCAGCAGCCTTCTTGTCCACTGCT 4 (0.000122%)

TGGGTCATTTTTGCTGTCACCAGCAACGTTGCCACGACGAACATCCTTGACAGACACATTCTTGACATTG
AAGCCCACATTGTCCCCAGGAAGAGCTTCA 10 (0.000304%)

TGGGTGGTTGGTGTAAATGAGTGAGGCAGGAGTCCGAGGAGGTTAGTTGTGGCAATAAAAATGATTAAG
GATACTAGTATAAGAGATCAGGTTCGTCCTT 11 (0.000335%)

TGGGTTGGGTGGTGGAGAGCGCGTGTCATCTGCGGGTGGCACTGCCCACGGTGGGCGGGCGGGCCTCTC
TACTCGAAGGTGACCACGTTTAGATTCTGAG 3 (0.000091%)

TGGTAGGATGCAGTCCAGAGCCTCAAGCAGCGTGGTTCCACTGGCATTGCCATCCTTACGGGTGACTTTC
CATCCCTTGAACCAAGGCATGTTAGCACTT 31 (0.000944%)

TGGTAGTCCCCTTTCATTTTATAATAGAAAACCTTGGACTCGCCAGTGTTAGCTGCTGGAATGAGGTGTT
TGTCCAGTACATCCAGAATGTCACAACAGA 5 (0.000152%)

TGGTGACCTTGCCAGCTCCAGCAGCCTTCTTGTCCACTGCTTTGATGACACCCACCGCAACTGTCTGTCTC
ATATCACGAACAGCAAAGCGACCCAAAGG 8 (0.000244%)

TGGTGGGTCATTTTTGCTGTCACCAGCAACGTTGCCACGACGAACATCCTTGACAGACACATTCTTGACA
TTGAAGCCCACATTGTCCCCAGGAAGAGCT 17 (0.000517%)

TGGTGGTAGGATGCAGTCCAGAGCCTCAAGCAGCGTGGTTCCACTGGCATTGCCATCCTTACGGGTGACT
TTCCATCCCTTGAACCAAGGCATGTTAGCA 10 (0.000304%)

TGGTTGAATGAGTAGGCTGATGGTTTCGATAATAACTAGTATGGGGATAAGGGGTGTAGGTGTGCCTTGT
GGTAAGAAGTGGGCTAGGGCATTTTTAATC 5 (0.000152%)

TGGTTGGTGTAAATGAGTGAGGCAGGAGTCCGAGGAGGTTAGTTGTGGCAATAAAAATGATTAAGGATA
CTAGTATAAGAGATCAGGTTCGTCCTTTAGT 5 (0.000152%)

TGGTTTGGGTTGGGTGGTGGAGAGCGCGTGTCATCTGCGGGTGGCACTGCCCACGGTGGGCGGGCGGGC
CTCTCTACTCGAAGGTGACCACGTTTAGATT 4 (0.000122%)

TGTAAATGAGTGAGGCAGGAGTCCGAGGAGGTTAGTTGTGGCAATAAAAATGATTAAGGATACTAGTAT
AAGAGATCAGGTTCGTCCTTTAGTGTTGTGT 15 (0.000457%)

TGTAATCATGTTTTTGATAAAGTCTCTGTGTCCTGGGGCATCAATGATAGTCACATAGTACTTGCTGGTCT
CAAATTTCCACAAGGAGATATCAATGGTG 385 (0.011719%)

TGTAGACATCCTGGAGAGGCAGGCGCAAGGGCTTGTCAGTTGGACGAGTTGGTGGTAGGATGCAGTCCA
GAGCCTCAAGCAGCGTGGTTCCACTGGCATT 8 (0.000244%)

TGTAGAGGGAGTATAGGGCTGTGACTAGTATGTTGAGTCCTGTAAGTAGGAGAGTGATATTTGATCAGG
AGAACGTGGTTACTAGCACAGAGAGTTCTCC 398 (0.012115%)

TGTAGCCAATTTTCTTAATGTAAGTGCTGACTTCCTTAACAATTTCCTCATATCTCTTCTGGCTGTAGGGT
GGCTCAGTGGAATCCATTTTGTTAACACC 31 (0.000944%)

TGTAGGGTGGCTCAGTGGAATCCATTTTGTTAACACCGACAATTAGTTGTTTCACACCCAGTGTGTAAGC
CAGAAGGGCATGCTCTCGGGTCTGCCCATT 18 (0.000548%)

TGTCAGTTGGACGAGTTGGTGGTAGGATGCAGTCCAGAGCCTCAAGCAGCGTGGTTCCACTGGCATTGC
CATCCTTACGGGTGACTTTCCATCCCTTGAA 10 (0.000304%)

TGTCCCCAGGAAGAGCTTCACTCAAAGCTTCATGGTGCATTTCGACAGATTTTACTTCCGTTGTAACGTT
GACTGGAGCAAAGGTGACCACCATACCGGG 7 (0.000213%)

TGTGACAAATTTTTGGTCAAGTTGTTTCCATTAAAAAGTACTGATTTTAAAAACTAATAACTTAAAACTG
CCACACGCAAAAAAGAAAACCAAAGTGGTC 4 (0.000122%)

TGTGGCTACAAAAAATGTTGAGCCGTAGATGCCGTCGGAAATGGTGAAGGGAGACTCGAAGTACTCTGA
GGCTTGTAGGAGGGTAAAATAGAGACCCAGT 552 (0.016803%)

TGTTACTTTAATTGGACTGCCTTCGTAATTCATTGCCTCTGCTTCAACAATGTGCAACTCATCCTTTGCAC
CAGCCCCTAAACTGACCGTTCTTAAAGAT 3 (0.000091%)

TGTTGCAAATTCTCATGGTTTGGGTTGGGTGGTGGAGAGCGCGTGTCATCTGCGGGTGGCACTGCCCACG
GTGGGCGGGCGGGCCTCTCTACTCGAAGGT 3 (0.000091%)

TGTTTCACACCCAGTGTGTAAGCCAGAAGGGCATGCTCTCGGGTCTGCCCATTCTTGGAGATACCAGCTT
CAAATTCACCAACACCAGCAGCAACAATCA 3 (0.000091%)

TGTTTTTAAGCCTAATGTGGGGACAGCTCATGAGTGCAAGACGTCTTGTGATGTAATTATTATACGAATG
GGGGCTTCAATCGGGAGTACTACTCGATTG 7 (0.000213%)

TTAAAAACTAATAACTTAAAACTGCCACACGCAAAAAAGAAAACCAAAGTGGTCCACAAAACATTCTCC
TTTCCTTCTGAAGGTTTTACGATGCATTGTT 49 (0.001492%)

TTAAAAAGTACTGATTTTAAAAACTAATAACTTAAAACTGCCACACGCAAAAAAGAAAACCAAAGTGGT
CCACAAAACATTCTCCTTTCCTTCTGAAGGT 42 (0.001278%)

TTAAAACTGCCACACGCAAAAAAGAAAACCAAAGTGGTCCACAAAACATTCTCCTTTCCTTCTGAAGGT
TTTACGATGCATTGTTATCATTAACCAGTCT 3 (0.000091%)

TTAACAATTTCCTCATATCTCTTCTGGCTGTAGGGTGGCTCAGTGGAATCCATTTTGTTAACACCGACAAT
TAGTTGTTTCACACCCAGTGTGTAAGCCA 8 (0.000244%)

TTAAGAGTGGGGTGGCAGGTATTAGGGATAATATTCATTTAGCCTTCTGAGCTTTCTGGGCAGACTTGGT
GACCTTGCCAGCTCCAGCAGCCTTCTTGTC 5 (0.000152%)

TTAATGTAAGTGCTGACTTCCTTAACAATTTCCTCATATCTCTTCTGGCTGTAGGGTGGCTCAGTGGAATC
CATTTTGTTAACACCGACAATTAGTTGTT 4 (0.000122%)

TTAGCCTTCTGAGCTTTCTGGGCAGACTTGGTGACCTTGCCAGCTCCAGCAGCCTTCTTGTCCACTGCTTT
GATGACACCCACCGCAACTGTCTGTCTCA 41 (0.001248%)

TTAGGGAGTCATAAGTGGAGTCCGTAAAGAGGTATCTTTACTATAAAAGCTATTGTGTAAGCTAGTCATA
TTAAGTTGTTGGCTCAGGAGTTTGATAGTT 5 (0.000152%)

TTAGGGATAATATTCATTTAGCCTTCTGAGCTTTCTGGGCAGACTTGGTGACCTTGCCAGCTCCAGCAGC

CTTCTTGTCCACTGCTTTGATGACACCCAC 5 (0.000152%)

TTAGGGGGTCGGAGGAAAAGGTTGGGGAACAGCTAAATAGGTTGTTGTTGATTTGGTTAAAAAATAGTA
GAGGGATGATGCTAATAATTAGGCTGTGGGT 3 (0.000091%)

TTAGTATTAGGAGGGGGGTTGTTAGGGGGTCGGAGGAAAAGGTTGGGGAACAGCTAAATAGGTTGTTGT
TGATTTGGTTAAAAAATAGTAGAGGGATGAT 5 (0.000152%)

TTATACGAATGGGGGCTTCAATCGGGAGTACTACTCGATTGTCAACGTCAAGGAGTCGCAGGTCGCCTG
GTTCTAGGAATAATGGGGGAAGTATGTAGGA 3 (0.000091%)

TTATAGATAGTTGGGTGGTTGGTGTAAATGAGTGAGGCAGGAGTCCGAGGAGGTTAGTTGTGGCAATAA
AAATGATTAAGGATACTAGTATAAGAGATCA 13 (0.000396%)

TTATCCAAGACCCAGGCATACTTGAAGGAGCCCTTTCCCATCTCAGCAGCCTCCTTCTCAAATTTTTCAAT
GGTTCTTTTGTCGATGCCACCGCATTTAT 469 (0.014276%)

TTATTATACGAATGGGGGCTTCAATCGGGAGTACTACTCGATTGTCAACGTCAAGGAGTCGCAGGTCGCC
TGGTTCTAGGAATAATGGGGGAAGTATGTA 5 (0.000152%)

TTATTTGGTTTCGGTTGTTTTCTATTAGACTATGGTGAGCTCAGGTGATTGATACTCCTGATGCGAGTAAT
ACGGATGTGTTTAGGAGTGGGACTTCTAG 3 (0.000091%)

TTCAATCGGGAGTACTACTCGATTGTCAACGTCAAGGAGTCGCAGGTCGCCTGGTTCTAGGAATAATGG
GGGAAGTATGTAGGAGTTGAAGATTAGTCCG 9 (0.000274%)

TTCACACCCAGTGTGTAAGCCAGAAGGGCATGCTCTCGGGTCTGCCCATTCTTGGAGATACCAGCTTCAA
ATTCACCAACACCAGCAGCAACAATCAGGA 33 (0.001005%)

TTCACTCAAAGCTTCATGGTGCATTTCGACAGATTTTACTTCCGTTGTAACGTTGACTGGAGCAAAGGTG
ACCACCATACCGGGTTTGAGAACACCAGTC 9 (0.000274%)

TTCATATGAGATTGTTTGGGCTACTGCTCGCAGTGCGCCGATCAGGGCGTAGTTTGAGTTTGATGCTCAC
CCTGATCAGAGGATTGAGTAAACGGCTAGG 5 (0.000152%)

TTCATTTAGCCTTCTGAGCTTTCTGGGCAGACTTGGTGACCTTGCCAGCTCCAGCAGCCTTCTTGTCCACT
GCTTTGATGACACCCACCGCAACTGTCTG 22 (0.000670%)

TTCCACCACTGATTAAGAGTGGGGTGGCAGGTATTAGGGATAATATTCATTTAGCCTTCTGAGCTTTCTG
GGCAGACTTGGTGACCTTGCCAGCTCCAGC 13 (0.000396%)

TTCCATTAAAAAGTACTGATTTTAAAAACTAATAACTTAAAACTGCCACACGCAAAAAAGAAAACCAAA
GTGGTCCACAAAACATTCTCCTTTCCTTCTG 69 (0.002100%)

TTCCATTGGTGGGTCATTTTTGCTGTCACCAGCAACGTTGCCACGACGAACATCCTTGACAGACACATTC
TTGACATTGAAGCCCACATTGTCCCCAGGA 12 (0.000365%)

TTCCTCATATCTCTTCTGGCTGTAGGGTGGCTCAGTGGAATCCATTTTGTTAACACCGACAATTAGTTGTT
TCACACCCAGTGTGTAAGCCAGAAGGGCA 24 (0.000731%)

TTCCTCCCCTGTTGCAAATTCTCATGGTTTGGGTTGGGTGGTGGAGAGCGCGTGTCATCTGCGGGTGGCA
CTGCCCACGGTGGGCGGGCGGGCCTCTCTA 4 (0.000122%)

TTCCTTAACAATTTCCTCATATCTCTTCTGGCTGTAGGGTGGCTCAGTGGAATCCATTTTGTTAACACCGA
CAATTAGTTGTTTCACACCCAGTGTGTAA 18 (0.000548%)

TTCGACATGGGCTTTAGGGAGTCATAAGTGGAGTCCGTAAAGAGGTATCTTTACTATAAAAGCTATTGTG
TAAGCTAGTCATATTAAGTTGTTGGCTCAG 447 (0.013607%)

TTCGGTTGTTTCTATTAGACTATGGTGAGCTCAGGTGATTGATACTCCTGATGCGAGTAATACGGATGT
GTTTAGGAGTGGGACTTCTAGGGGATTTAG 99 (0.003014%)

TTCTATTAGACTATGGTGAGCTCAGGTGATTGATACTCCTGATGCGAGTAATACGGATGTGTTTAGGAGT
GGGACTTCTAGGGGATTTAGCGGGGTGATG 6 (0.000183%)

TTCTGAGCTTTCTGGGCAGACTTGGTGACCTTGCCAGCTCCAGCAGCCTTCTTGTCCACTGCTTTGATGAC
ACCCACCGCAACTGTCTGTCTCATATCAC 9 (0.000274%)

TTCTGGCTGTAGGGTGGCTCAGTGGAATCCATTTTGTTAACACCGACAATTAGTTGTTTCACACCCAGTG
TGTAAGCCAGAAGGGCATGCTCTCGGGTCT 10 (0.000304%)

TTCTGGGCAGACTTGGTGACCTTGCCAGCTCCAGCAGCCTTCTTGTCCACTGCTTTGATGACACCCACCG
CAACTGTCTGTCTCATATCACGAACAGCAA 9 (0.000274%)

TTCTGTGACAAATTTTTGGTCAAGTTGTTTCCATTAAAAAGTACTGATTTTAAAAACTAATAACTTAAAA
CTGCCACACGCAAAAAAGAAAACCAAAGTG 15 (0.000457%)

TTCTTAATGTAAGTGCTGACTTCCTTAACAATTTCCTCATATCTCTTCTGGCTGTAGGGTGGCTCAGTGGA
ATCCATTTTGTTAACACCGACAATTAGTT 13 (0.000396%)

TTCTTCCACCACTGATTAAGAGTGGGGTGGCAGGTATTAGGGATAATATTCATTTAGCCTTCTGAGCTTT
CTGGGCAGACTTGGTGACCTTGCCAGCTCC 4 (0.000122%)

TTGATGACACCCACCGCAACTGTCTGTCTCATATCACGAACAGCAAAGCGACCCAAAGGTGGATAGTCT
GAGAAGCTCTCAACACACATGGGCTTGCCAG 6 (0.000183%)

TTGATGGCCCCTAAGATAGAGGAGACACCTGCTAGGTGTAAGGAGAAGATGGTTAGGTCTACGGAGGCT
CCAGGGTGGGAGTAGTTCCCTGCTAAGGGAG 12 (0.000365%)

TTGCAAATTCTCATGGTTTGGGTTGGGTGGTGGAGAGCGCGTGTCATCTGCGGGTGGCACTGCCCACGGT
GGGCGGGCGGGCCTCTCTACTCGAAGGTGA 4 (0.000122%)

TTGCATCTGTTTTTAAGCCTAATGTGGGGACAGCTCATGAGTGCAAGACGTCTTGTGATGTAATTATTAT
ACGAATGGGGGCTTCAATCGGGAGTACTAC 4 (0.000122%)

TTGCCATCCTTACGGGTGACTTTCCATCCCTTGAACCAAGGCATGTTAGCACTTGGCTCCAGCATGTTGTC
ACCATTCCAACCAGAAATTGGCACAAATG 5 (0.000152%)

TTGCCCATACACATGAGTATTTGTCTAAAACATGTCTTCTTTGTAGCAGCTAGGCCCTGCCACCACTGTGC
TTGGCTGAGTTCACAAATCTGTTGTAACC 5 (0.000152%)

TTGCTAATACAATGCCAGTCAGGCCACCTACGGTGAAAAGAAAGATGAATCCTAGGGCTCAGAGCACTG
CAGCAGATCATTTCATATTGCTTCCGTGGAG 11 (0.000335%)

TTGGACGAGTTGGTGGTAGGATGCAGTCCAGAGCCTCAAGCAGCGTGGTTCCACTGGCATTGCCATCCTT
ACGGGTGACTTTCCATCCCTTGAACCAAGG 3 (0.000091%)

TTGGGGAACAGCTAAATAGGTTGTTGTTGATTTGGTTAAAAAATAGTAGAGGGATGATGCTAATAATTA
GGCTGTGGGTGGTTGTGTTGATTCAAATTAT 4 (0.000122%)

TTGGGTTGGGTGGTGGAGAGCGCGTGTCATCTGCGGGTGGCACTGCCCACGGTGGGCGGGCGGGCCTCT
CTACTCGAAGGTGACCACGTTTAGATTCTGA 11 (0.000335%)

TTGGTGACCTTGCCAGCTCCAGCAGCCTTCTTGTCCACTGCTTTGATGACACCCACCGCAACTGTCTGTCT
CATATCACGAACAGCAAAGCGACCCAAAG 5 (0.000152%)

TTGGTGGGTCATTTTTGCTGTCACCAGCAACGTTGCCACGACGAACATCCTTGACAGACACATTCTTGAC
ATTGAAGCCCACATTGTCCCCAGGAAGAGC 4 (0.000122%)

TTGGTGTAAATGAGTGAGGCAGGAGTCCGAGGAGGTTAGTTGTGGCAATAAAAATGATTAAGGATACTA GTATAAGAGATCAGGTTCGTCCTTTAGTGTT 5 (0.000152%)

TTGGTTGAATGAGTAGGCTGATGGTTTCGATAATAACTAGTATGGGGATAAGGGGTGTAGGTGTGCCTTG TGGTAAGAAGTGGGCTAGGGCATTTTTAAT 3 (0.000091%)

TTGTAGACATCCTGGAGAGGCAGGCGCAAGGGCTTGTCAGTTGGACGAGTTGGTGGTAGGATGCAGTCC AGAGCCTCAAGCAGCGTGGTTCCACTGGCAT 12 (0.000365%)

TTGTAGCCAATTTTCTTAATGTAAGTGCTGACTTCCTTAACAATTTCCTCATATCTCTTCTGGCTGTAGGG TGGCTCAGTGGAATCCATTTTGTTAACAC 9 (0.000274%)

TTGTCAGTTGGACGAGTTGGTGGTAGGATGCAGTCCAGAGCCTCAAGCAGCGTGGTTCCACTGGCATTGC CATCCTTACGGGTGACTTTCCATCCCTTGA 3 (0.000091%)

TTGTTTCACACCCAGTGTGTAAGCCAGAAGGGCATGCTCTCGGGTCTGCCCATTCTTGGAGATACCAGCT TCAAATTCACCAACACCAGCAGCAACAATC 5 (0.000152%)

TTGTTTTCTATTAGACTATGGTGAGCTCAGGTGATTGATACTCCTGATGCGAGTAATACGGATGTGTTTA GGAGTGGGACTTCTAGGGGATTTAGCGGGG 92 (0.002800%)

TTTAAAAACTAATAACTTAAAACTGCCACACGCAAAAAAGAAAACCAAAGTGGTCCACAAAACATTCTC CTTTCCTTCTGAAGGTTTTACGATGCATTGT 36 (0.001096%)

TTTAAGCCTAATGTGGGGACAGCTCATGAGTGCAAGACGTCTTGTGATGTAATTATTATACGAATGGGGG CTTCAATCGGGAGTACTACTCGATTGTCAA 7 (0.000213%)

TTTAATCTGACGCAGGCTTATGCGGAGGAGAATGTTTTCATGTTACTTATACTAACATTAGTTCTTCTATA GGGTGATAGATTGGTCCAATTGGGTGTGA 3 (0.000091%)

TTTAATTGGACTGCCTTCGTAATTCATTGCCTCTGCTTCAACAATGTGCAACTCATCCTTTGCACCAGCCC CTAAACTGACCGTTCTTAAAGATAACTGG 4 (0.000122%)

TTTAGCCTTCTGAGCTTTCTGGGCAGACTTGGTGACCTTGCCAGCTCCAGCAGCCTTCTTGTCCACTGCTT TGATGACACCCACCGCAACTGTCTGTCTC 14 (0.000426%)

TTTAGGGAGTCATAAGTGGAGTCCGTAAAGAGGTATCTTTACTATAAAAGCTATTGTGTAAGCTAGTCAT ATTAAGTTGTTGGCTCAGGAGTTTGATAGT 3 (0.000091%)

TTTATAGATAGTTGGGTGGTTGGTGTAAATGAGTGAGGCAGGAGTCCGAGGAGGTTAGTTGTGGCAATA AAAATGATTAAGGATACTAGTATAAGAGATC 37 (0.001126%)

TTTCACACCCAGTGTGTAAGCCAGAAGGGCATGCTCTCGGGTCTGCCCATTCTTGGAGATACCAGCTTCA AATTCACCAACACCAGCAGCAACAATCAGG 4 (0.000122%)

TTTCCATTAAAAAGTACTGATTTTAAAAACTAATAACTTAAAACTGCCACACGCAAAAAAGAAAACCAA AGTGGTCCACAAAACATTCTCCTTTCCTTCT 402 (0.012237%)

TTTCCTCATATCTCTTCTGGCTGTAGGGTGGCTCAGTGGAATCCATTTTGTTAACACCGACAATTAGTTGT TTCACACCCAGTGTGTAAGCCAGAAGGGC 12 (0.000365%)

TTTCTATTAGACTATGGTGAGCTCAGGTGATTGATACTCCTGATGCGAGTAATACGGATGTGTTTAGGAG TGGGACTTCTAGGGGATTTAGCGGGGTGAT 48 (0.001461%)

TTTCTGGGCAGACTTGGTGACCTTGCCAGCTCCAGCAGCCTTCTTGTCCACTGCTTTGATGACACCCACCG CAACTGTCTGTCTCATATCACGAACAGCA 4 (0.000122%)

TTTCTTAATGTAAGTGCTGACTTCCTTAACAATTTCCTCATATCTCTTCTGGCTGTAGGGTGGCTCAGTGG

AATCCATTTTGTTAACACCGACAATTAGT 10 (0.000304%)

TTTGATGACACCCACCGCAACTGTCTGTCTCATATCACGAACAGCAAAGCGACCCAAAGGTGGATAGTC
TGAGAAGCTCTCAACACACATGGGCTTGCCA 25 (0.000761%)

TTTGCTAATACAATGCCAGTCAGGCCACCTACGGTGAAAAGAAAGATGAATCCTAGGGCTCAGAGCACT
GCAGCAGATCATTTCATATTGCTTCCGTGGA 12 (0.000365%)

TTTGCTACTGGTCCTGTAATGGCAGAACCTTTCATCTCGCCTTTATTGTTCACTATGACTCCTGCATTATCT
TCAAAATAAAGAAACACGCCATCTTTTC 5 (0.000152%)

TTTGGACAGGTGGTGTGTGGTGGCCTTGGTATGTGCTTTCTCGTGTTACATCGCGCCATCATTGGTATATG
GTTAGTGTGTTGGTTAGTAGGCCTAGTAT 6 (0.000183%)

TTTGGGTTGGGTGGTGGAGAGCGCGTGTCATCTGCGGGTGGCACTGCCCACGGTGGGCGGGCGGGCCTC
TCTACTCGAAGGTGACCACGTTTAGATTCTG 9 (0.000274%)

TTTGTAGACATCCTGGAGAGGCAGGCGCAAGGGCTTGTCAGTTGGACGAGTTGGTGGTAGGATGCAGTC
CAGAGCCTCAAGCAGCGTGGTTCCACTGGCA 16 (0.000487%)

TTTGTCTAAAACATGTCTTCTTTGTAGCAGCTAGGCCCTGCCACCACTGTGCTTGGCTGAGTTCACAAATC
TGTTGTAACCTGTAGCTTCCCTGTCACTT 6 (0.000183%)

TTTTAAAAACTAATAACTTAAAACTGCCACACGCAAAAAAGAAAACCAAAGTGGTCCACAAAACATTCT
CCTTTCCTTCTGAAGGTTTTACGATGCATTG 3 (0.000091%)

TTTTAAGCCTAATGTGGGGACAGCTCATGAGTGCAAGACGTCTTGTGATGTAATTATTATACGAATGGGG
GCTTCAATCGGGAGTACTACTCGATTGTCA 19 (0.000578%)

TTTTAATCTGACGCAGGCTTATGCGGAGGAGAATGTTTTCATGTTACTTATACTAACATTAGTTCTTCTAT
AGGGTGATAGATTGGTCCAATTGGGTGTG 10 (0.000304%)

TTTTCTATTAGACTATGGTGAGCTCAGGTGATTGATACTCCTGATGCGAGTAATACGGATGTGTTTAGGA
GTGGGACTTCTAGGGGATTTAGCGGGGTGA 4 (0.000122%)

TTTTCTTAATGTAAGTGCTGACTTCCTTAACAATTTCCTCATATCTCTTCTGGCTGTAGGGTGGCTCAGTG
GAATCCATTTTGTTAACACCGACAATTAG 20 (0.000609%)

TTTTGCCCATACACATGAGTATTTGTCTAAAACATGTCTTCTTTGTAGCAGCTAGGCCCTGCCACCACTGT
GCTTGGCTGAGTTCACAAATCTGTTGTAA 3 (0.000091%)

TTTTGCTGTCACCAGCAACGTTGCCACGACGAACATCCTTGACAGACACATTCTTGACATTGAAGCCCAC
ATTGTCCCCAGGAAGAGCTTCACTCAAAGC 87 (0.002648%)

TTTTGGACAGGTGGTGTGTGGTGGCCTTGGTATGTGCTTTCTCGTGTTACATCGCGCCATCATTGGTATAT
GGTTAGTGTGTTGGTTAGTAGGCCTAGTA 20 (0.000609%)

TTTTTAAGCCTAATGTGGGGACAGCTCATGAGTGCAAGACGTCTTGTGATGTAATTATTATACGAATGGG
GGCTTCAATCGGGAGTACTACTCGATTGTC 3 (0.000091%)

TTTTTCCTCCCCTGTTGCAAATTCTCATGGTTTGGGTTGGGTGGTGGAGAGCGCGTGTCATCTGCGGGTGG
CACTGCCCACGGTGGGCGGGCGGGCCTCT 505 (0.015372%)

TTTTTGATAAAGTCTCTGTGTCCTGGGGCATCAATGATAGTCACATAGTACTTGCTGGTCTCAAATTTCCA
CAAGGAGATATCAATGGTGATACCACGTT 5 (0.000152%)

TTTTTGCCCATACACATGAGTATTTGTCTAAAACATGTCTTCTTTGTAGCAGCTAGGCCCTGCCACCACTG
TGCTTGGCTGAGTTCACAAATCTGTTGTA 6 (0.000183%)

TTTTTGCTGTCACCAGCAACGTTGCCACGACGAACATCCTTGACAGACACATTCTTGACATTGAAGCCCA
CATTGTCCCCAGGAAGAGCTTCACTCAAAG 14 (0.000426%)

TTTTTGGACAGGTGGTGTGTGGTGGCCTTGGTATGTGCTTTCTCGTGTTACATCGCGCCATCATTGGTATA
TGGTTAGTGTGTTGGTTAGTAGGCCTAGT 364 (0.011080%)

TTTTTTGCCCATACACATGAGTATTTGTCTAAAACATGTCTTCTTTGTAGCAGCTAGGCCCTGCCACCACT
GTGCTTGGCTGAGTTCACAAATCTGTTGT 322 (0.009802%)

TTTTTTTTTTTTTTTTTTTTTTTTTTTTTTTTTTTTTTTTTTTTTTTTTTTTTTTTTTTTTTTTTTTTTTTTTTT
TTTTTTTTTTTTTTTTTTTTTT 1169 (0.035584%)

After filtering: read2: quality
Value of each position will be shown on mouse over.
2040608010012014038 38.53939.540

positionqualityATCGmean

<https://plot.ly/>
After filtering: read2: base contents
Value of each position will be shown on mouse over.
20406080100120140 00.10.20.30.40.50.6

positionbase content ratiosA(26.86%)T(25.60%)C(23.44%)G(24.02%)N(0.053%)GC(47.46%)

<https://plot.ly/>
After filtering: read2: KMER counting
Darker background means larger counts. The count will be shown on mouse over.

	AA	AT	AC	AG	TA	TT	TC	TG	CA	CT	CC	CG	GA	GT	GC	GG
AAA	AAAAA	AAAAT	AAAAC	AAAAG	AAATA	AAATT	AAATC	AAATG	AAACA	AAACT	AAACC	AAACG	AAAGA	AAAGT	AAAGC	AAAGG
AAT	AATAA	AATAT	AATAC	AATAG	AATTA	AATTT	AATTC	AATTG	AATCA	AATCT	AATCC	AATCG	AATGA	AATGT	AATGC	AATGG
AAC	AACAA	AACAT	AACAC	AACAG	AACTA	AACTT	AACTC	AACTG	AACCA	AACCT	AACCC	AACCG	AACGA	AACGT	AACGC	AACGG
AAG	AAGAA	AAGAT	AAGAC	AAGAG	AAGTA	AAGTT	AAGTC	AAGTG	AAGCA	AAGCT	AAGCC	AAGCG	AAGGA	AAGGT	AAGGC	AAGGG
ATA	ATAAA	ATAAT	ATAAC	ATAAG	ATATA	ATATT	ATATC	ATATG	ATACA	ATACT	ATACC	ATACG	ATAGA	ATAGT	ATAGC	ATAGG
ATT	ATTAA	ATTAT	ATTAC	ATTAG	ATTTA	ATTTT	ATTTC	ATTTG	ATTCA	ATTCT	ATTCC	ATTCG	ATTGA	ATTGT	ATTGC	ATTGG
ATC	ATCAA	ATCAT	ATCAC	ATCAG	ATCTA	ATCTT	ATCTC	ATCTG	ATCCA	ATCCT	ATCCC	ATCCG	ATCGA	ATCGT	ATCGC	ATCGG
ATG	ATGAA	ATGAT	ATGAC	ATGAG	ATGTA	ATGTT	ATGTC	ATGTG	ATGCA	ATGCT	ATGCC	ATGCG	ATGGA	ATGGT	ATGGC	ATGGG
ACA	ACAAA	ACAAT	ACAAC	ACAAG	ACATA	ACATT	ACATC	ACATG	ACACA	ACACT	ACACC	ACACG	ACAGA	ACAGT	ACAGC	ACAGG
ACT	ACTAA	ACTAT	ACTAC	ACTAG	ACTTA	ACTTT	ACTTC	ACTTG	ACTCA	ACTCT	ACTCC	ACTCG	ACTGA	ACTGT	ACTGC	ACTGG
ACC	ACCAA	ACCAT	ACCAC	ACCAG	ACCTA	ACCTT	ACCTC	ACCTG	ACCCA	ACCCT	ACCCC	ACCCG	ACCGA	ACCGT	ACCGC	ACCGG
ACG	ACGAA	ACGAT	ACGAC	ACGAG	ACGTA	ACGTT	ACGTC	ACGTG	ACGCA	ACGCT	ACGCC	ACGCG	ACGGA	ACGGT	ACGGC	ACGGG
AGA	AGAAA	AGAAT	AGAAC	AGAAG	AGATA	AGATT	AGATC	AGATG	AGACA	AGACT	AGACC	AGACG	AGAGA	AGAGT	AGAGC	AGAGG
AGT	AGTAA	AGTAT	AGTAC	AGTAG	AGTTA	AGTTT	AGTTC	AGTTG	AGTCA	AGTCT	AGTCC	AGTCG	AGTGA	AGTGT	AGTGC	AGTGG
AGC	AGCAA	AGCAT	AGCAC	AGCAG	AGCTA	AGCTT	AGCTC	AGCTG	AGCCA	AGCCT	AGCCC					

AGCCG AGCGA AGCGT AGCGC AGCGG
AGG AGGAA AGGAT AGGAC AGGAG AGGTA AGGTT AGGTC AGGTG AGGCA AGGCT AGGCC
AGGCG AGGGA AGGGT AGGGC AGGGG
TAA TAAAA TAAAT TAAAC TAAAG TAATA TAATT TAATC TAATG TAACA TAACT TAACC
TAACG TAAGA TAAGT TAAGC TAAGG
TAT TATAA TATAT TATAC TATAG TATTA TATTT TATTC TATTG TATCA TATCT TATCC
TATCG TATGA TATGT TATGC TATGG
TAC TACAA TACAT TACAC TACAG TACTA TACTT TACTC TACTG TACCA TACCT TACCC
TACCG TACGA TACGT TACGC TACGG
TAG TAGAA TAGAT TAGAC TAGAG TAGTA TAGTT TAGTC TAGTG TAGCA TAGCT TAGCC
TAGCG TAGGA TAGGT TAGGC TAGGG
TTA TTAAA TTAAT TTAAC TTAAG TTATA TTATT TTATC TTATG TTACA TTACT TTACC
TTACG TTAGA TTAGT TTAGC TTAGG
TTT TTTAA TTTAT TTTAC TTTAG TTTTA TTTTT TTTTC TTTTG TTTCA TTTCT TTTCC
TTTCG TTTGA TTTGT TTTGC TTTGG
TTC TTCAA TTCAT TTCAC TTCAG TTCTA TTCTT TTCTC TTCTG TTCCA TTCCT TTCCC
TTCCG TTCGA TTCGT TTCGC TTCGG
TTG TTGAA TTGAT TTGAC TTGAG TTGTA TTGTT TTGTC TTGTG TTGCA TTGCT TTGCC
TTGCG TTGGA TTGGT TTGGC TTGGG
TCA TCAAA TCAAT TCAAC TCAAG TCATA TCATT TCATC TCATG TCACA TCACT TCACC
TCACG TCAGA TCAGT TCAGC TCAGG
TCT TCTAA TCTAT TCTAC TCTAG TCTTA TCTTT TCTTC TCTTG TCTCA TCTCT TCTCC
TCTCG TCTGA TCTGT TCTGC TCTGG
TCC TCCAA TCCAT TCCAC TCCAG TCCTA TCCTT TCCTC TCCTG TCCCA TCCCT TCCCC
TCCCG TCCGA TCCGT TCCGC TCCGG
TCG TCGAA TCGAT TCGAC TCGAG TCGTA TCGTT TCGTC TCGTG TCGCA TCGCT TCGCC
TCGCG TCGGA TCGGT TCGGC TCGGG
TGA TGAAA TGAAT TGAAC TGAAG TGATA TGATT TGATC TGATG TGACA TGACT TGACC
TGACG TGAGA TGAGT TGAGC TGAGG
TGT TGTAA TGTAT TGTAC TGTAG TGTTA TGTTT TGTTC TGTTG TGTCA TGTCT TGTCC
TGTCG TGTGA TGTGT TGTGC TGTGG
TGC TGCAA TGCAT TGCAC TGCAG TGCTA TGCTT TGCTC TGCTG TGCCA TGCCT TGCCC
TGCCG TGCGA TGCGT TGCGC TGCGG
TGG TGGAA TGGAT TGGAC TGGAG TGGTA TGGTT TGGTC TGGTG TGGCA TGGCT TGGCC
TGGCG TGGGA TGGGT TGGGC TGGGG
CAA CAAAA CAAAT CAAAC CAAAG CAATA CAATT CAATC CAATG CAACA CAACT CAACC
CAACG CAAGA CAAGT CAAGC CAAGG
CAT CATAA CATAT CATAC CATAG CATTA CATTT CATTC CATTG CATCA CATCT CATCC
CATCG CATGA CATGT CATGC CATGG
CAC CACAA CACAT CACAC CACAG CACTA CACTT CACTC CACTG CACCA CACCT CACCC
CACCG CACGA CACGT CACGC CACGG
CAG CAGAA CAGAT CAGAC CAGAG CAGTA CAGTT CAGTC CAGTG CAGCA CAGCT CAGCC
CAGCG CAGGA CAGGT CAGGC CAGGG
CTA CTAAA CTAAT CTAAC CTAAG CTATA CTATT CTATC CTATG CTACA CTACT CTACC
CTACG CTAGA CTAGT CTAGC CTAGG
CTT CTTAA CTTAT CTTAC CTTAG CTTTA CTTTT CTTTC CTTTG CTTCA CTTCT CTTCC
CTTCG CTTGA CTTGT CTTGC CTTGG
CTC CTCAA CTCAT CTCAC CTCAG CTCTA CTCTT CTCTC CTCTG CTCCA CTCCT CTCCC
CTCCG CTCGA CTCGT CTCGC CTCGG
CTG CTGAA CTGAT CTGAC CTGAG CTGTA CTGTT CTGTC CTGTG CTGCA CTGCT CTGCC
CTGCG CTGGA CTGGT CTGGC CTGGG
CCA CCAAA CCAAT CCAAC CCAAG CCATA CCATT CCATC CCATG CCACA CCACT CCACC
CCACG CCAGA CCAGT CCAGC CCAGG
CCT CCTAA CCTAT CCTAC CCTAG CCTTA CCTTT CCTTC CCTTG CCTCA CCTCT CCTCC
CCTCG CCTGA CCTGT CCTGC CCTGG
CCC CCCAA CCCAT CCCAC CCCAG CCCTA CCCTT CCCTC CCCTG CCCCA CCCCT CCCCC
CCCCG CCCGA CCCGT CCCGC CCCGG
CCG CCGAA CCGAT CCGAC CCGAG CCGTA CCGTT CCGTC CCGTG CCGCA CCGCT CCGCC
CCGCG CCGGA CCGGT CCGGC CCGGG

CGA CGAAA CGAAT CGAAC CGAAG CGATA CGATT CGATC CGATG CGACA CGACT CGACC
CGACG CGAGA CGAGT CGAGC CGAGG
CGT CGTAA CGTAT CGTAC CGTAG CGTTA CGTTT CGTTC CGTTG CGTCA CGTCT CGTCC
CGTCG CGTGA CGTGT CGTGC CGTGG
CGC CGCAA CGCAT CGCAC CGCAG CGCTA CGCTT CGCTC CGCTG CGCCA CGCCT CGCCC
CGCCG CGCGA CGCGT CGCGC CGCGG
CGG CGGAA CGGAT CGGAC CGGAG CGGTA CGGTT CGGTC CGGTG CGGCA CGGCT CGGCC
CGGCG CGGGA CGGGT CGGGC CGGGG
GAA GAAAA GAAAT GAAAC GAAAG GAATA GAATT GAATC GAATG GAACA GAACT GAACC
GAACG GAAGA GAAGT GAAGC GAAGG
GAT GATAA GATAT GATAC GATAG GATTA GATTT GATTC GATTG GATCA GATCT GATCC
GATCG GATGA GATGT GATGC GATGG
GAC GACAA GACAT GACAC GACAG GACTA GACTT GACTC GACTG GACCA GACCT GACCC
GACCG GACGA GACGT GACGC GACGG
GAG GAGAA GAGAT GAGAC GAGAG GAGTA GAGTT GAGTC GAGTG GAGCA GAGCT GAGCC
GAGCG GAGGA GAGGT GAGGC GAGGG
GTA GTAAA GTAAT GTAAC GTAAG GTATA GTATT GTATC GTATG GTACA GTACT GTACC
GTACG GTAGA GTAGT GTAGC GTAGG
GTT GTTAA GTTAT GTTAC GTTAG GTTTA GTTTT GTTTC GTTTG GTTCA GTTCT GTTCC
GTTCG GTTGA GTTGT GTTGC GTTGG
GTC GTCAA GTCAT GTCAC GTCAG GTCTA GTCTT GTCTC GTCTG GTCCA GTCCT GTCCC
GTCCG GTCGA GTCGT GTCGC GTCGG
GTG GTGAA GTGAT GTGAC GTGAG GTGTA GTGTT GTGTC GTGTG GTGCA GTGCT GTGCC
GTGCG GTGGA GTGGT GTGGC GTGGG
GCA GCAAA GCAAT GCAAC GCAAG GCATA GCATT GCATC GCATG GCACA GCACT GCACC
GCACG GCAGA GCAGT GCAGC GCAGG
GCT GCTAA GCTAT GCTAC GCTAG GCTTA GCTTT GCTTC GCTTG GCTCA GCTCT GCTCC
GCTCG GCTGA GCTGT GCTGC GCTGG
GCC GCCAA GCCAT GCCAC GCCAG GCCTA GCCTT GCCTC GCCTG GCCCA GCCCT GCCCC
GCCCG GCCGA GCCGT GCCGC GCCGG
GCG GCGAA GCGAT GCGAC GCGAG GCGTA GCGTT GCGTC GCGTG GCGCA GCGCT GCGCC
GCGCG GCGGA GCGGT GCGGC GCGGG
GGA GGAAA GGAAT GGAAC GGAAG GGATA GGATT GGATC GGATG GGACA GGACT GGACC
GGACG GGAGA GGAGT GGAGC GGAGG
GGT GGTAA GGTAT GGTAC GGTAG GGTTA GGTTT GGTTC GGTTG GGTCA GGTCT GGTCC
GGTCG GGTGA GGTGT GGTGC GGTGG
GGC GGCAA GGCAT GGCAC GGCAG GGCTA GGCTT GGCTC GGCTG GGCCA GGCCT GGCCC
GGCCG GGCGA GGCGT GGCGC GGCGG
GGG GGGAA GGGAT GGGAC GGGAG GGGTA GGGTT GGGTC GGGTG GGGCA GGGCT GGGCC
GGGCG GGGGA GGGGT GGGGC GGGGG

After filtering: read2: overrepresented sequences
Sampling rate: 1 / 20
overrepresented sequence count (% of bases) distribution: cycle 1 ~
cycle 151
AAAAAAAAAAAAAAAAAAAA 6646 (0.040478%)

AAAAAAAAAAAGGCCGCCGTGACCTATTCACCCTCCACTTCCCGTCTCAGAATCTAAACGTGGTCACCTT
CGAGTAGAGAGGCCCGCCCGCCCACCGTGG 429 (0.013064%)

AAAAACATGATTACAGGGACATCTCAGGCTGACTGTGCTGTCCTGATTGTTGCTGCTGGTGTTGGTGAAT
TTGAAGCTGGTATCTCCAAGAATGGCAGA 35 (0.001066%)

AAAAAGCTGGAAGATGGCCCTAAATTCTTGAAGTCTGGTGATGCTGCCATTGTTGATATGGTTCCTGGCA
AGCCCATGTGTGTTGAGAGCTTCTCAGACT 16 (0.000487%)

AAAAAGGCCTTCGATACGGGATAATCCTATTTATTACCTCAGAAGTTTTTTCTTCGCAGGATTTTTCTGA
GCCTTTTACCACTCCAGCCTAGCCCCTAC 27 (0.000822%)

AAAAATAAAAAATTATAACAAACCCTGAGAACCAAAATGAACGAAAATCTGTTCGCTTCATTCATTGCC
CCCACAATCCTAGGCCTACCCGCCGCAGTAC 19 (0.000579%)

AAAACATGATTACAGGGACATCTCAGGCTGACTGTGCTGTCCTGATTGTTGCTGCTGGTGTTGGTGAATT
TGAAGCTGGTATCTCCAAGAATGGGCAGAC 15 (0.000457%)

AAAACCCAGCCCATGACCCCTAACAGGGGCCCTCTCAGCCCTCCTAATGACCTCCGGCCTAGCCATGTGA
TTTCACTTCCACTCCATAACGCTCCTCATA 41 (0.001249%)

AAAAGAAGAAAGATGAGGCAGAGGTCCAAGTAAACCGCTAGCTTGTTGCACCGTGGAGGCCACAGGAG
CAGAAACATGGAATGCCAGACGCTGGGGATGC 5 (0.000152%)

AAAAGCTGGAAGATGGCCCTAAATTCTTGAAGTCTGGTGATGCTGCCATTGTTGATATGGTTCCTGGCAA
GCCCATGTGTGTTGAGAGCTTCTCAGACTA 29 (0.000883%)

AAAAGGCCTTCGATACGGGATAATCCTATTTATTACCTCAGAAGTTTTTTTCTTCGCAGGATTTTTCTGAG
CCTTTTACCACTCCAGCCTAGCCCCTACC 26 (0.000792%)

AAAATAAAAAATTATAACAAACCCTGAGAACCAAAATGAACGAAAATCTGTTCGCTTCATTCATTGCCC
CCACAATCCTAGGCCTACCCGCCGCAGTACT 8 (0.000244%)

AAAATCTGTTCGCTTCATTCATTGCCCCCACAATCCTAGGCCTACCCGCCGCAGTACTGATCATTCTATTT
CCCCCTCTATTGATCCCCACCTCCAAATA 32 (0.000974%)

AAACAACTAATTGTCGGTGTTAACAAAATGGATTCCACTGAGCCACCCTACAGCCAGAAGAGATATGAG
GAAATTGTTAAGGAAGTCAGCACTTACATTA 14 (0.000426%)

AAACATATAACTGAACTCCTCACACCCAATTGGACCAATCTATCACCCTATAGAAGAACTAATGTTAGTA
TAAGTAACATGAAAACATTCTCCTCCGCAT 7 (0.000213%)

AAACATGATTACAGGGACATCTCAGGCTGACTGTGCTGTCCTGATTGTTGCTGCTGGTGTTGGTGAATTT
GAAGCTGGTATCTCCAAGAATGGGCAGACC 12 (0.000365%)

AAACCCAGCCCATGACCCCTAACAGGGGCCCTCTCAGCCCTCCTAATGACCTCCGGCCTAGCCATGTGAT
TTCACTTCCACTCCATAACGCTCCTCATAC3 (0.000091%)

AAACCCGGTATGGTGGTCACCTTTGCTCCAGTCAACGTTACAACGGAAGTAAAATCTGTCGAAATGCAC
CATGAAGCTTTGAGTGAAGCTCTTCCTGGGG 8 (0.000244%)

AAACCGTCTGAACTATCCTGCCCGCCATCATCCTAGTCCTCATCGCCCTCCCATCCCTACGCATCCTTTAC
ATAACAGACGAGGTCAACGATCCCTCCCT 5 (0.000152%)

AAACTGAAAGCTGAGCGTGAACGTGGTATCACCATTGATATCTCCTTGTGGAAATTTGAGACCAGCAAG
TACTATGTGACTATCATTGATGCCCCAGGAC 12 (0.000365%)

AAAGAAGAAAGATGAGGCAGAGGTCCAAGTAAACCGCTAGCTTGTTGCACCGTGGAGGCCACAGGAGC
AGAAACATGGAATGCCAGACGCTGGGGATGCT 7 (0.000213%)

AAAGAGGAGAGGCACCCGATATATGTTCTCTAGGCCTTTTAGAAAACATGGAGTTGTTCCTTTGGCCACA
TATATGCGAATCTATAAGAAAGGTGATATT 582 (0.017723%)

AAAGCAGTGGACAAGAAGGCTGCTGGAGCTGGCAAGGTCACCAAGTCTGCCCAGAAAGCTCAGAAGGC
TAAATGAATATTATCCCTAATACCTGCCACCC 54 (0.001644%)

AAAGCCCATAAAAATAAAAAATTATAACAAACCCTGAGAACCAAAATGAACGAAAATCTGTTCGCTTCA
TTCATTGCCCCCACAATCCTAGGCCTACCCG 19 (0.000579%)

AAAGCGGTCTGCCCCTGGAGGTGGTAGCAAGGTTCCACAGAAAAAAGTAAAACTTGCTGCTGATGAAGA
TGATGACGATGATGATGAAGAGGATGATGAT 3 (0.000091%)

AAAGCTGAGCGTGAACGTGGTATCACCATTGATATCTCCTTGTGGAAATTTGAGACCAGCAAGTACTATG
TGACTATCATTGATGCCCCAGGACACAGAG 5 (0.000152%)

AAAGCTGGAAGATGGCCCTAAATTCTTGAAGTCTGGTGATGCTGCCATTGTTGATATGGTTCCTGGCAAG
CCCATGTGTGTTGAGAGCTTCTCAGACTAT 15 (0.000457%)

AAAGGATCTCCTTCATCCCTCTCCAGAAGAGGAGAAGAGGAAACACAAGAAGAAACGCCTGGTGCAGA
GCCCCAATTCCTACTTCATGGATGTGAAATGC 5 (0.000152%)

AAAGGCCTTCGATACGGGATAATCCTATTTATTACCTCAGAAGTTTTTTTCTTCGCAGGATTTTTCTGAGC
CTTTTACCACTCCAGCCTAGCCCCTACCC 6 (0.000183%)

AAAGGGCTCCTTCAAGTATGCCTGGGTCTTGGATAAACTGAAAGCTGAGCGTGAACGTGGTATCACCAT
TGATATCTCCTTGTGGAAATTTGAGACCAGC 14 (0.000426%)

AAAGTTCTCCGCTCCCAGACATGGGTCCCTCGGCTTCCTGCCTCGGAAGCGCAGCAGCAGGCATCGTGG
GAAGGTGAAGAGCTTCCCTAAGGATGACCCG 4 (0.000122%)

AAATAAAAAATTATAACAAACCCTGAGAACCAAAATGAACGAAAATCTGTTCGCTTCATTCATTGCCCC
CACAATCCTAGGCCTACCCGCCGCAGTACTG 3 (0.000091%)

AAATAATTCAAGCACTGCTTATTACAATTTTACTGGGTCTCTATTTTACCCTCCTACAAGCCTCAGAGTAC
TTCGAGTCTCCCTTCACCATTTCCGACGG 57 (0.001736%)

AAATCACCTTCCACCCTTACTACACAATCAAAGACGCCCTCGGCTTACTTCTCTTCATTCTCTCCTTAATG
ACATTAACACTATTCTCACCAGACCTCCT 6 (0.000183%)

AAATCTGTTCGCTTCATTCATTGCCCCCACAATCCTAGGCCTACCCGCCGCAGTACTGATCATTCTATTTC
CCCCTCTATTGATCCCCACCTCCAAATAT 8 (0.000244%)

AAATGCGGTGGCATCGACAAAAGAACCATTGAAAAATTTGAGAAGGAGGCTGCTGAGATGGGAAAGGG
CTCCTTCAAGTATGCCTGGGTCTTGGATAAAC 9 (0.000274%)

AAATTCTTGAAGTCTGGTGATGCTGCCATTGTTGATATGGTTCCTGGCAAGCCCATGTGTGTTGAGAGCT
TCTCAGACTATCCACCTTTGGGTCGCTTTG69 (0.002101%)

AACAACCGACTAATCACCACCCAACAATGACTAATCAAACTAACCTCAAAACAAATGATAACCATACAC
AACACTAAAGGACGAACCTGATCTCTTATAC 3 (0.000091%)

AACACAGGTGTCGTGAAAACTACCCCTAAAAGCCAAAATGGGAAAGGAAAAGACTCATATCAACATTGT
CGTCATTGGACACGTAGATTCGGGCAAGTCC 20 (0.000609%)

AACAGGGGCCCTCTCAGCCCTCCTAATGACCTCCGGCCTAGCCATGTGATTTCACTTCCACTCCATAACG
CTCCTCATACTAGGCCTACTAACCAACACA 6 (0.000183%)

AACATATAACTGAACTCCTCACACCCAATTGGACCAATCTATCACCCTATAGAAGAACTAATGTTAGTAT
AAGTAACATGAAAACATTCTCCTCCGCATA 5 (0.000152%)

AACATGATTACAGGGACATCTCAGGCTGACTGTGCTGTCCTGATTGTTGCTGCTGGTGTTGGTGAATTTG
AAGCTGGTATCTCCAAGAATGGGCAGACCC 5 (0.000152%)

AACCAAATAATTCAAGCACTGCTTATTACAATTTTACTGGGTCTCTATTTTACCCTCCTACAAGCCTCAGA
GTACTTCGAGTCTCCCTTCACCATTTCCG 274 (0.008344%)

AACCACCCAACTATCTATAAACCTAGCCATGGCCATCCCCTTATGAGCGGGCGCAGTGATTATAGGCTTT
CGCTCTAAGATTAAAAATGCCCTAGCCCAC 16 (0.000487%)

AACCAGGCGACCTGCGACTCCTTGACGTTGACAATCGAGTAGTACTCCCGATTGAAGCCCCCATTCGTAT

AATAATTACATCACAAGACGTCTTGCACTC 15 (0.000457%)

AACCCAGCCCATGACCCCTAACAGGGGCCCTCTCAGCCCTCCTAATGACCTCCGGCCTAGCCATGTGATT
TCACTTCCACTCCATAACGCTCCTCATACT6 (0.000183%)

AACCCGGTATGGTGGTCACCTTTGCTCCAGTCAACGTTACAACGGAAGTAAAATCTGTCGAAATGCACC
ATGAAGCTTTGAGTGAAGCTCTTCCTGGGGA 5 (0.000152%)

AACCGACTAATCACCACCCAACAATGACTAATCAAACTAACCTCAAAACAAATGATAACCATACACAAC
ACTAAAGGACGAACCTGATCTCTTATACTAG 6 (0.000183%)

AACCGTCTGAACTATCCTGCCCGCCATCATCCTAGTCCTCATCGCCCTCCCATCCCTACGCATCCTTTACA
TAACAGACGAGGTCAACGATCCCTCCCTT 10 (0.000305%)

AACCTAGCCATGGCCATCCCCTTATGAGCGGGCGCAGTGATTATAGGCTTTCGCTCTAAGATTAAAAATG
CCCTAGCCCACTTCTTACCACAAGGCACAC 6 (0.000183%)

AACGCCTGAACGCAGGCACATACTTCCTATTCTACACCCTAGTAGGCTCCCTTCCCCTACTCATCGCACT
GATTTACACTCACAACACCCTAGGCTCACT 24 (0.000731%)

AACGTGGTATCACCATTGATATCTCCTTGTGGAAATTTGAGACCAGCAAGTACTATGTGACTATCATTGA
TGCCCCAGGACACAGAGACTTTATCAAAAA 5 (0.000152%)

AACGTTGTAGGCCCCTACGGGCTACTACAACCCTTCGCTGACGCCATAAAACTCTTCACCAAAGAGCCCC
TAAAACCCGCCACATCTACCATCACCCTCT 3 (0.000091%)

AACTAACCTCCTCGGACTCCTGCCTCACTCATTTACACCAACCACCCAACTATCTATAAACCTAGCCATG
GCCATCCCCTTATGAGCGGGCGCAGTGATT 11 (0.000335%)

AACTATCCTGCCCGCCATCATCCTAGTCCTCATCGCCCTCCCATCCCTACGCATCCTTTACATAACAGACG
AGGTCAACGATCCCTCCCTTACCATCAAA 7 (0.000213%)

AACTATCTATAAACCTAGCCATGGCCATCCCCTTATGAGCGGGCGCAGTGATTATAGGCTTTCGCTCTAA
GATTAAAAATGCCCTAGCCCACTTCTTACC 5 (0.000152%)

AACTGAACTCCTCACACCCAATTGGACCAATCTATCACCCTATAGAAGAACTAATGTTAGTATAAGTAAC
ATGAAAACATTCTCCTCCGCATAAGCCTGC 7 (0.000213%)

AACTGACTAGTTCCCCTAATAATCGGTGCCCCCGATATGGCGTTTCCCCGCATAAACAACATAAGCTTCT
GACTCTTACCTCCCTCTCTCCTACTCCTGC 3 (0.000091%)

AACTTACTACTCCGGAAAAAAAGAACCATTTGGATACATAGGTATGGTCTGAGCTATGATATCAATTGG
CTTCCTAGGGTTTATCGTGTGAGCACACCAT 3 (0.000091%)

AACTTCACCCGTAACCCACCGCCATGGCCGAGGAAGGCATTGCTGCTGGAGGTGTAATGGACGTTAATA
CTGCTTTACAAGAGGTTCTGAAGACTGCCCT 4 (0.000122%)

AAGAAGGCTGCTGGAGCTGGCAAGGTCACCAAGTCTGCCCAGAAAGCTCAGAAGGCTAAATGAATATTA
TCCCTAATACCTGCCACCCCACTCTTAATCA 30 (0.000914%)

AAGAATGTGTCTGTCAAGGATGTTCGTCGTGGCAACGTTGCTGGTGACAGCAAAAATGACCCACCAATG
GAAGCAGCTGGCTTCACTGCTCAGGTGATTA 9 (0.000274%)

AAGAGCTACGAGCTGCCTGACGGCCAGGTCATCACCATTGGCAATGAGCGGTTCCGCTGCCCTGAGGCA
CTCTTCCAGCCTTCCTTCCTGGGCATGGAGT 6 (0.000183%)

AAGATGGCCCTAAATTCTTGAAGTCTGGTGATGCTGCCATTGTTGATATGGTTCCTGGCAAGCCCATGTG
TGTTGAGAGCTTCTCAGACTATCCACCTTT4 (0.000122%)

AAGATTCAACTTCACCCGTAACCCACCGCCATGGCCGAGGAAGGCATTGCTGCTGGAGGTGTAATGGAC
GTTAATACTGCTTTACAAGAGGTTCTGAAGA 4 (0.000122%)

AAGCAATTTTGGAGGTGGTGGAAGCTACAATGATTTTGGGAATTACAACAATCAGTCTTCAAATTTTGGA
CCCATGAAGGGAGGAAATTTTGGAGGCAGA 6 (0.000183%)

AAGCACTGCTTATTACAATTTTACTGGGTCTCTATTTTACCCTCCTACAAGCCTCAGAGTACTTCGAGTCT
CCCTTCACCATTTCCGACGGCATCTACGG 5 (0.000152%)

AAGCAGTGGACAAGAAGGCTGCTGGAGCTGGCAAGGTCACCAAGTCTGCCCAGAAAGCTCAGAAGGCT
AAATGAATATTATCCCTAATACCTGCCACCCC 13 (0.000396%)

AAGCCCATAAAAATAAAAAATTATAACAAACCCTGAGAACCAAAATGAACGAAAATCTGTTCGCTTCAT
TCATTGCCCCCACAATCCTAGGCCTACCCGC 6 (0.000183%)

AAGCTACAATGATTTTGGGAATTACAACAATCAGTCTTCAAATTTTGGACCCATGAAGGGAGGAAATTTT
GGAGGCAGAAGCTCTGGCCCCTATGGCGGT 7 (0.000213%)

AAGCTGAGCGTGAACGTGGTATCACCATTGATATCTCCTTGTGGAAATTTGAGACCAGCAAGTACTATGT
GACTATCATTGATGCCCCAGGACACAGAGA 12 (0.000365%)

AAGCTGGAAGATGGCCCTAAATTCTTGAAGTCTGGTGATGCTGCCATTGTTGATATGGTTCCTGGCAAGC
CCATGTGTGTTGAGAGCTTCTCAGACTATC 5 (0.000152%)

AAGGAGGCTGCTGAGATGGGAAAGGGCTCCTTCAAGTATGCCTGGGTCTTGGATAAACTGAAAGCTGAG
CGTGAACGTGGTATCACCATTGATATCTCCT 7 (0.000213%)

AAGGCCTTCGATACGGGATAATCCTATTTATTACCTCAGAAGTTTTTTTCTTCGCAGGATTTTTCTGAGCC
TTTTACCACTCCAGCCTAGCCCCTACCCC 4 (0.000122%)

AAGGCTGCTGGAGCTGGCAAGGTCACCAAGTCTGCCCAGAAAGCTCAGAAGGCTAAATGAATATTATCC
CTAATACCTGCCACCCCACTCTTAATCAGTG 7 (0.000213%)

AAGGGATGGAAAGTCACCCGTAAGGATGGCAATGCCAGTGGAACCACGCTGCTTGAGGCTCTGGACTGC
ATCCTACCACCAACTCGTCCAACTGACAAGC 3 (0.000091%)

AAGGGCGCCGGGTCTGTGTTCCGCGCGCACGTGAAGCACCGTAAAGGCGCTGCGCGCCTGCGCGCCGTG
GATTTCGCTGAGCGGCACGGCTACATCAAGG 7 (0.000213%)

AAGGGCTCCTTCAAGTATGCCTGGGTCTTGGATAAACTGAAAGCTGAGCGTGAACGTGGTATCACCATT
GATATCTCCTTGTGGAAATTTGAGACCAGCA 43 (0.001309%)

AAGTATGCCTGGGTCTTGGATAAACTGAAAGCTGAGCGTGAACGTGGTATCACCATTGATATCTCCTTGT
GGAAATTTGAGACCAGCAAGTACTATGTGA 9 (0.000274%)

AAGTCTGGTGATGCTGCCATTGTTGATATGGTTCCTGGCAAGCCCATGTGTGTTGAGAGCTTCTCAGACT
ATCCACCTTTGGGTCGCTTTGCTGTTCGTG 4 (0.000122%)

AAGTGCTAACATGCCTTGGTTCAAGGGATGGAAAGTCACCCGTAAGGATGGCAATGCCAGTGGAACCAC
GCTGCTTGAGGCTCTGGACTGCATCCTACCA 3 (0.000091%)

AAGTTCTCCGCTCCCAGACATGGGTCCCTCGGCTTCCTGCCTCGGAAGCGCAGCAGCAGGCATCGTGGG
AAGGTGAAGAGCTTCCCTAAGGATGACCCGT 3 (0.000091%)

AATAATCGGTGCCCCCGATATGGCGTTTCCCCGCATAAACAACATAAGCTTCTGACTCTTACCTCCCTCT
CTCCTACTCCTGCTCGCATCTGCTATAGTG 5 (0.000152%)

AATAATCTCCCATATTGTAACTTACTACTCCGGAAAAAAAGAACCATTTGGATACATAGGTATGGTCTGA
GCTATGATATCAATTGGCTTCCTAGGGTTT 10 (0.000305%)

AATAATTCAAGCACTGCTTATTACAATTTTACTGGGTCTCTATTTTACCCTCCTACAAGCCTCAGAGTACT
TCGAGTCTCCCTTCACCATTTCCGACGGC 34 (0.001035%)

AATCATTTTTATTGCCACAACTAACCTCCTCGGACTCCTGCCTCACTCATTTACACCAACCACCCAACTAT
CTATAAACCTAGCCATGGCCATCCCCTTA 5 (0.000152%)

AATCCTATTTATTACCTCAGAAGTTTTTTTCTTCGCAGGATTTTTCTGAGCCTTTTACCACTCCAGCCTAGC
CCCTACCCCCCAATTAGGAGGGCACTGG 3 (0.000091%)

AATCTATCACCCTATAGAAGAACTAATGTTAGTATAAGTAACATGAAAACATTCTCCTCCGCATAAGCCT
GCGTCAGATTAAAACACTGAACTGACAATT 21 (0.000640%)

AATCTCCCATATTGTAACTTACTACTCCGGAAAAAAAGAACCATTTGGATACATAGGTATGGTCTGAGCT
ATGATATCAATTGGCTTCCTAGGGTTTATC 8 (0.000244%)

AATCTGTTCGCTTCATTCATTGCCCCCACAATCCTAGGCCTACCCGCCGCAGTACTGATCATTCTATTTCC
CCCTCTATTGATCCCCACCTCCAAATATC 12 (0.000365%)

AATGACCTCCGGCCTAGCCATGTGATTTCACTTCCACTCCATAACGCTCCTCATACTAGGCCTACTAACC
AACACACTAACCATATACCAATGATGGCGC 8 (0.000244%)

AATGATGGAAGCAATTTTGGAGGTGGTGGAAGCTACAATGATTTTGGGAATTACAACAATCAGTCTTCA
AATTTTGGACCCATGAAGGGAGGAAATTTTG 3 (0.000091%)

AATGATTTTGGGAATTACAACAATCAGTCTTCAAATTTTGGACCCATGAAGGGAGGAAATTTTGGAGGC
AGAAGCTCTGGCCCCTATGGCGGTGGAGGCC 11 (0.000335%)

AATGCGCAGGCTGAAGCGCAAAAGAAGAAAGATGAGGCAGAGGTCCAAGTAAACCGCTAGCTTGTTGC
ACCGTGGAGGCCACAGGAGCAGAAACATGGAA 5 (0.000152%)

AATGCGGTGGCATCGACAAAAGAACCATTGAAAAATTTGAGAAGGAGGCTGCTGAGATGGGAAAGGGC
TCCTTCAAGTATGCCTGGGTCTTGGATAAACT 5 (0.000152%)

AATGTCAAGAATGTGTCTGTCAAGGATGTTCGTCGTGGCAACGTTGCTGGTGACAGCAAAAATGACCCA
CCAATGGAAGCAGCTGGCTTCACTGCTCAGG 4 (0.000122%)

AATGTGTCTGTCAAGGATGTTCGTCGTGGCAACGTTGCTGGTGACAGCAAAAATGACCCACCAATGGAA
GCAGCTGGCTTCACTGCTCAGGTGATTATCC 5 (0.000152%)

AATTCAAGCACTGCTTATTACAATTTTACTGGGTCTCTATTTTACCCTCCTACAAGCCTCAGAGTACTTCG
AGTCTCCCTTCACCATTTCCGACGGCATC 4 (0.000122%)

AATTCTTGAAGTCTGGTGATGCTGCCATTGTTGATATGGTTCCTGGCAAGCCCATGTGTGTTGAGAGCTT
CTCAGACTATCCACCTTTGGGTCGCTTTGC 20 (0.000609%)

AATTGGACCAATCTATCACCCTATAGAAGAACTAATGTTAGTATAAGTAACATGAAAACATTCTCCTCCG
CATAAGCCTGCGTCAGATTAAAACACTGAA 6 (0.000183%)

AATTTTGGAGGTGGTGGAAGCTACAATGATTTTGGGAATTACAACAATCAGTCTTCAAATTTTGGACCCA
TGAAGGGAGGAAATTTTGGAGGCAGAAGCT 3 (0.000091%)

ACAAGAAGGCTGCTGGAGCTGGCAAGGTCACCAAGTCTGCCCAGAAAGCTCAGAAGGCTAAATGAATA
TTATCCCTAATACCTGCCACCCCACTCTTAAT 15 (0.000457%)

ACACAGGTGTCGTGAAAACTACCCCTAAAAGCCAAAATGGGAAAGGAAAAGACTCATATCAACATTGTC
GTCATTGGACACGTAGATTCGGGCAAGTCCA 10 (0.000305%)

ACAGACAGTTGCGGTGGGTGTCATCAAAGCAGTGGACAAGAAGGCTGCTGGAGCTGGCAAGGTCACCA

AGTCTGCCCAGAAAGCTCAGAAGGCTAAATGA 7 (0.000213%)

ACAGAGACTTTATCAAAAACATGATTACAGGGACATCTCAGGCTGACTGTGCTGTCCTGATTGTTGCTGC
TGGTGTTGGTGAATTTGAAGCTGGTATCTC 6 (0.000183%)

ACAGAGGAAGGGCGCCGGGTCTGTGTTCCGCGCGCACGTGAAGCACCGTAAAGGCGCTGCGCGCCTGCG
CGCCGTGGATTTCGCTGAGCGGCACGGCTAC 3 (0.000091%)

ACAGCTCTAAGCCTCCTTATTCGAGCCGAGCTGGGCCAGCCAGGCAACCTTCTAGGTAACGACCACATCT
ACAACGTTATCGTCACAGCCCATGCATTTG 7 (0.000213%)

ACAGGGGCCCTCTCAGCCCTCCTAATGACCTCCGGCCTAGCCATGTGATTTCACTTCCACTCCATAACGC
TCCTCATACTAGGCCTACTAACCAACACAC 3 (0.000091%)

ACAGGTGTCGTGAAAACTACCCCTAAAAGCCAAAATGGGAAAGGAAAAGACTCATATCAACATTGTCGT
CATTGGACACGTAGATTCGGGCAAGTCCACC 14 (0.000426%)

ACAGTTGCGGTGGGTGTCATCAAAGCAGTGGACAAGAAGGCTGCTGGAGCTGGCAAGGTCACCAAGTCT
GCCCAGAAAGCTCAGAAGGCTAAATGAATAT 3 (0.000091%)

ACATATAACTGAACTCCTCACACCCAATTGGACCAATCTATCACCCTATAGAAGAACTAATGTTAGTATA
AGTAACATGAAAACATTCTCCTCCGCATAA 5 (0.000152%)

ACATGATTACAGGGACATCTCAGGCTGACTGTGCTGTCCTGATTGTTGCTGCTGGTGTTGGTGAATTTGA
AGCTGGTATCTCCAAGAATGGGCAGACCCG 7 (0.000213%)

ACCAAAGCCCATAAAAATAAAAAATTATAACAAACCCTGAGAACCAAAATGAACGAAAATCTGTTCGCT
TCATTCATTGCCCCCACAATCCTAGGCCTAC 10 (0.000305%)

ACCAGCAAGTACTATGTGACTATCATTGATGCCCCAGGACACAGAGACTTTATCAAAAACATGATTACA
GGGACATCTCAGGCTGACTGTGCTGTCCTGA 5 (0.000152%)

ACCAGCGGTGGCAGAGACCCCAGACATCAAGCTCTTTGGGAAGTGGAGCACCGATGATGTGCAGATCAA
TGACATTTCCCTGCAGGATTACATTGCAGTG 3 (0.000091%)

ACCAGGCGACCTGCGACTCCTTGACGTTGACAATCGAGTAGTACTCCCGATTGAAGCCCCCATTCGTATA
ATAATTACATCACAAGACGTCTTGCACTCA 7 (0.000213%)

ACCATTGATATCTCCTTGTGGAAATTTGAGACCAGCAAGTACTATGTGACTATCATTGATGCCCCAGGAC
ACAGAGACTTTATCAAAAACATGATTACAG 7 (0.000213%)

ACCCAACTATCTATAAACCTAGCCATGGCCATCCCCTTATGAGCGGGCGCAGTGATTATAGGCTTTCGCT
CTAAGATTAAAAATGCCCTAGCCCACTTCT 6 (0.000183%)

ACCCAGCCCATGACCCCTAACAGGGGCCCTCTCAGCCCTCCTAATGACCTCCGGCCTAGCCATGTGATTT
CACTTCCACTCCATAACGCTCCTCATACTA 5 (0.000152%)

ACCCCATTCTATACCAACACCTATTCTGATTTTTCGGTCACCCTGAAGTTTATATTCTTATCCTACCAGGC
TTCGGAATAATCTCCCATATTGTAACTTA 3 (0.000091%)

ACCCGATATATGTTCTCTAGGCCTTTTAGAAAACATGGAGTTGTTCCTTTGGCCACATATATGCGAATCT
ATAAGAAAGGTGATATTGTAGACATCAAGG 5 (0.000152%)

ACCCGGTATGGTGGTCACCTTTGCTCCAGTCAACGTTACAACGGAAGTAAAATCTGTCGAAATGCACCAT
GAAGCTTTGAGTGAAGCTCTTCCTGGGGAC 4 (0.000122%)

ACCGACTAATCACCACCCAACAATGACTAATCAAACTAACCTCAAAACAAATGATAACCATACACAACA
CTAAAGGACGAACCTGATCTCTTATACTAGT 3 (0.000091%)

ACCGTAAAGGCGCTGCGCGCCTGCGCGCCGTGGATTTCGCTGAGCGGCACGGCTACATCAAGGGCATCG
TCAAGGACATCATCCACGACCCGGGCCGCGG 5 (0.000152%)

ACCGTCTGAACTATCCTGCCCGCCATCATCCTAGTCCTCATCGCCCTCCCATCCCTACGCATCCTTTACAT
AACAGACGAGGTCAACGATCCCTCCCTTA 8 (0.000244%)

ACCTCAGAAGTTTTTTTCTTCGCAGGATTTTTCTGAGCCTTTTACCACTCCAGCCTAGCCCCTACCCCCCA
ATTAGGAGGGCACTGGCCCCCAACAGGCA 4 (0.000122%)

ACCTTTGGGTCGCTTTGCTGTTCGTGATATGAGACAGACAGTTGCGGTGGGTGTCATCAAAGCAGTGGAC
AAGAAGGCTGCTGGAGCTGGCAAGGTCACC 4 (0.000122%)

ACGCAGGCACATACTTCCTATTCTACACCCTAGTAGGCTCCCTTCCCCTACTCATCGCACTGATTTACACT
CACAACACCCTAGGCTCACTAAACATTCT 3 (0.000091%)

ACGCCTGAACGCAGGCACATACTTCCTATTCTACACCCTAGTAGGCTCCCTTCCCCTACTCATCGCACTG
ATTTACACTCACAACACCCTAGGCTCACTA 3 (0.000091%)

ACGGGATAATCCTATTTATTACCTCAGAAGTTTTTTTCTTCGCAGGATTTTTCTGAGCCTTTTACCACTCC
AGCCTAGCCCCTACCCCCCAATTAGGAGG 3 (0.000091%)

ACGTGGTATCACCATTGATATCTCCTTGTGGAAATTTGAGACCAGCAAGTACTATGTGACTATCATTGAT
GCCCCAGGACACAGAGACTTTATCAAAAAC 7 (0.000213%)

ACTAGTTCCCCTAATAATCGGTGCCCCCGATATGGCGTTTCCCCGCATAAACAACATAAGCTTCTGACTC
TTACCTCCCTCTCTCCTACTCCTGCTCGCA 10 (0.000305%)

ACTATCCTGCCCGCCATCATCCTAGTCCTCATCGCCCTCCCATCCCTACGCATCCTTTACATAACAGACGA
GGTCAACGATCCCTCCCTTACCATCAAAT 6 (0.000183%)

ACTATCTATAAACCTAGCCATGGCCATCCCCTTATGAGCGGGCGCAGTGATTATAGGCTTTCGCTCTAAG
ATTAAAAATGCCCTAGCCCACTTCTTACCA 13 (0.000396%)

ACTATGTGACTATCATTGATGCCCCAGGACACAGAGACTTTATCAAAAACATGATTACAGGGACATCTC
AGGCTGACTGTGCTGTCCTGATTGTTGCTGC 4 (0.000122%)

ACTCATTTACACCAACCACCCAACTATCTATAAACCTAGCCATGGCCATCCCCTTATGAGCGGGCGCAGT
GATTATAGGCTTTCGCTCTAAGATTAAAAA 3 (0.000091%)

ACTCCTCACACCCAATTGGACCAATCTATCACCCTATAGAAGAACTAATGTTAGTATAAGTAACATGAAA
ACATTCTCCTCCGCATAAGCCTGCGTCAGA 11 (0.000335%)

ACTGAAAGCTGAGCGTGAACGTGGTATCACCATTGATATCTCCTTGTGGAAATTTGAGACCAGCAAGTA
CTATGTGACTATCATTGATGCCCCAGGACAC 4 (0.000122%)

ACTGAACTCCTCACACCCAATTGGACCAATCTATCACCCTATAGAAGAACTAATGTTAGTATAAGTAACA
TGAAAACATTCTCCTCCGCATAAGCCTGCG 8 (0.000244%)

ACTGACTAGTTCCCCTAATAATCGGTGCCCCCGATATGGCGTTTCCCCGCATAAACAACATAAGCTTCTG
ACTCTTACCTCCCTCTCTCCTACTCCTGCT 5 (0.000152%)

ACTGGGTGTGAAACAACTAATTGTCGGTGTTAACAAAATGGATTCCACTGAGCCACCCTACAGCCAGAA
GAGATATGAGGAAATTGTTAAGGAAGTCAGC 4 (0.000122%)

ACTTCACCCGTAACCCACCGCCATGGCCGAGGAAGGCATTGCTGCTGGAGGTGTAATGGACGTTAATAC
TGCTTTACAAGAGGTTCTGAAGACTGCCCTC 4 (0.000122%)

ACTTCCCCCATTATTCCTAGAACCAGGCGACCTGCGACTCCTTGACGTTGACAATCGAGTAGTACTCCCG
ATTGAAGCCCCCATTCGTATAATAATTACA 3 (0.000091%)

ACTTCGCATCCGCAAACTCTGTCTCAACATCTGTGTTGGGGAGAGTGGAGACAGACTGACGCGAGCAGC
CAAGGTGTTGGAGCAGCTCACAGGGCAGACC 5 (0.000152%)

ACTTCTCCTATCTCTCCCAGTCCTAGCTGCTGGCATCACTATACTACTAACAGACCGCAACCTCAACACC
ACCTTCTTCGACCCCGCCGGAGGAGGAGAC 4 (0.000122%)

ACTTGCTTCGTCTGTTCTGTGTTGGTTTTACTAAAAAACGCAACAATCAGATACGGAAGACCTCTTATGC
TCAGCACCAACAGGTCCGCCAAATCCGGAA 3 (0.000091%)

AGAAACCGTCTGAACTATCCTGCCCGCCATCATCCTAGTCCTCATCGCCCTCCCATCCCTACGCATCCTTT
ACATAACAGACGAGGTCAACGATCCCTCC10 (0.000305%)

AGAAAGTTCTCCGCTCCCAGACATGGGTCCCTCGGCTTCCTGCCTCGGAAGCGCAGCAGCAGGCATCGT
GGGAAGGTGAAGAGCTTCCCTAAGGATGACC 13 (0.000396%)

AGAACACAGGTGTCGTGAAAACTACCCCTAAAAGCCAAAATGGGAAAGGAAAAGACTCATATCAACAT
TGTCGTCATTGGACACGTAGATTCGGGCAAGT 150 (0.004568%)

AGAACGAGAAGCTGAACTTGGAGCTAGGGCAAAAGAATTCACCAATGTTTACATCAAGAATTTTGGAGA
AGACATGGATGATGAGCGCCTTAAGGATCTC 127 (0.003867%)

AGAACGCCTGAACGCAGGCACATACTTCCTATTCTACACCCTAGTAGGCTCCCTTCCCCTACTCATCGCA
CTGATTTACACTCACAACACCCTAGGCTCA 3 (0.000091%)

AGAAGAGCTACGAGCTGCCTGACGGCCAGGTCATCACCATTGGCAATGAGCGGTTCCGCTGCCCTGAGG
CACTCTTCCAGCCTTCCTTCCTGGGCATGGA 3 (0.000091%)

AGAAGGAGGCTGCTGAGATGGGAAAGGGCTCCTTCAAGTATGCCTGGGTCTTGGATAAACTGAAAGCTG
AGCGTGAACGTGGTATCACCATTGATATCTC 9 (0.000274%)

AGAAGGCTGCTGGAGCTGGCAAGGTCACCAAGTCTGCCCAGAAAGCTCAGAAGGCTAAATGAATATTAT
CCCTAATACCTGCCACCCCACTCTTAATCAG 3 (0.000091%)

AGAAGTTTTTTTCTTCGCAGGATTTTTCTGAGCCTTTTACCACTCCAGCCTAGCCCCTACCCCCCAATTAG
GAGGGCACTGGCCCCCAACAGGCATCACC 3 (0.000091%)

AGAATGTGTCTGTCAAGGATGTTCGTCGTGGCAACGTTGCTGGTGACAGCAAAAATGACCCACCAATGG
AAGCAGCTGGCTTCACTGCTCAGGTGATTAT 7 (0.000213%)

AGACAGACAGTTGCGGTGGGTGTCATCAAAGCAGTGGACAAGAAGGCTGCTGGAGCTGGCAAGGTCAC
CAAGTCTGCCCAGAAAGCTCAGAAGGCTAAAT 10 (0.000305%)

AGACAGTTGCGGTGGGTGTCATCAAAGCAGTGGACAAGAAGGCTGCTGGAGCTGGCAAGGTCACCAAG
TCTGCCCAGAAAGCTCAGAAGGCTAAATGAAT 33 (0.001005%)

AGACCAGCAAGTACTATGTGACTATCATTGATGCCCCAGGACACAGAGACTTTATCAAAAACATGATTA
CAGGGACATCTCAGGCTGACTGTGCTGTCCT 4 (0.000122%)

AGACCCCATTCTATACCAACACCTATTCTGATTTTTCGGTCACCCTGAAGTTTATATTCTTATCCTACCAG
GCTTCGGAATAATCTCCCATATTGTAACT 5 (0.000152%)

AGACTATCCACCTTTGGGTCGCTTTGCTGTTCGTGATATGAGACAGACAGTTGCGGTGGGTGTCATCAAA
GCAGTGGACAAGAAGGCTGCTGGAGCTGGC 40 (0.001218%)

AGAGCATGCCCTTCTGGCTTACACACTGGGTGTGAAACAACTAATTGTCGGTGTTAACAAAATGGATTCC
ACTGAGCCACCCTACAGCCAGAAGAGATAT 17 (0.000518%)

AGAGCTACGAGCTGCCTGACGGCCAGGTCATCACCATTGGCAATGAGCGGTTCCGCTGCCCTGAGGCAC

TCTTCCAGCCTTCCTTCCTGGGCATGGAGTC 4 (0.000122%)

AGAGCTTCTCAGACTATCCACCTTTGGGTCGCTTTGCTGTTCGTGATATGAGACAGACAGTTGCGGTGGG
TGTCATCAAAGCAGTGGACAAGAAGGCTGC 8 (0.000244%)

AGAGGAAGGGCGCCGGGTCTGTGTTCCGCGCGCACGTGAAGCACCGTAAAGGCGCTGCGCGCCTGCGCG
CCGTGGATTTCGCTGAGCGGCACGGCTACAT 8 (0.000244%)

AGATGGCCCTAAATTCTTGAAGTCTGGTGATGCTGCCATTGTTGATATGGTTCCTGGCAAGCCCATGTGT
GTTGAGAGCTTCTCAGACTATCCACCTTTG 14 (0.000426%)

AGCAAGTACTATGTGACTATCATTGATGCCCCAGGACACAGAGACTTTATCAAAAACATGATTACAGGG
ACATCTCAGGCTGACTGTGCTGTCCTGATTG 10 (0.000305%)

AGCAATTTTGGAGGTGGTGGAAGCTACAATGATTTTGGGAATTACAACAATCAGTCTTCAAATTTTGGAC
CCATGAAGGGAGGAAATTTTGGAGGCAGAA 4 (0.000122%)

AGCAGCACCAGCGGTGGCAGAGACCCCAGACATCAAGCTCTTTGGGAAGTGGAGCACCGATGATGTGCA
GATCAATGACATTTCCCTGCAGGATTACATT 7 (0.000213%)

AGCATGCCCTTCTGGCTTACACACTGGGTGTGAAACAACTAATTGTCGGTGTTAACAAAATGGATTCCAC
TGAGCCACCCTACAGCCAGAAGAGATATGA 3 (0.000091%)

AGCATTTGTGCCAATTTCTGGTTGGAATGGTGACAACATGCTGGAGCCAAGTGCTAACATGCCTTGGTTC
AAGGGATGGAAAGTCACCCGTAAGGATGGC 614 (0.018698%)

AGCCAGGCAACCTTCTAGGTAACGACCACATCTACAACGTTATCGTCACAGCCCATGCATTTGTAATAAT
CTTCTTCATAGTAATACCCATCATAATCGG 85 (0.002588%)

AGCCCATAAAAATAAAAAATTATAACAAACCCTGAGAACCAAAATGAACGAAAATCTGTTCGCTTCATT
CATTGCCCCCACAATCCTAGGCCTACCCGCC 8 (0.000244%)

AGCCCATGACCCCTAACAGGGGCCCTCTCAGCCCTCCTAATGACCTCCGGCCTAGCCATGTGATTTCACT
TCCACTCCATAACGCTCCTCATACTAGGCC 13 (0.000396%)

AGCCCATGTGTGTTGAGAGCTTCTCAGACTATCCACCTTTGGGTCGCTTTGCTGTTCGTGATATGAGACA
GACAGTTGCGGTGGGTGTCATCAAAGCAGT 5 (0.000152%)

AGCGAATGCGCAGGCTGAAGCGCAAAAGAAGAAAGATGAGGCAGAGGTCCAAGTAAACCGCTAGCTTG
TTGCACCGTGGAGGCCACAGGAGCAGAAACAT 909 (0.027681%)

AGCGTGAACGTGGTATCACCATTGATATCTCCTTGTGGAAATTTGAGACCAGCAAGTACTATGTGACTAT
CATTGATGCCCCAGGACACAGAGACTTTAT 8 (0.000244%)

AGCTACAATGATTTTGGGAATTACAACAATCAGTCTTCAAATTTTGGACCCATGAAGGGAGGAAATTTTG
GAGGCAGAAGCTCTGGCCCCTATGGCGGTG 5 (0.000152%)

AGCTCCTCCCTGGAGAAGAGCTACGAGCTGCCTGACGGCCAGGTCATCACCATTGGCAATGAGCGGTTC
CGCTGCCCTGAGGCACTCTTCCAGCCTTCCT 4 (0.000122%)

AGCTGAGCGTGAACGTGGTATCACCATTGATATCTCCTTGTGGAAATTTGAGACCAGCAAGTACTATGTG
ACTATCATTGATGCCCCAGGACACAGAGAC 16 (0.000487%)

AGCTGGAAGATGGCCCTAAATTCTTGAAGTCTGGTGATGCTGCCATTGTTGATATGGTTCCTGGCAAGCC
CATGTGTGTTGAGAGCTTCTCAGACTATCC 3 (0.000091%)

AGCTGGCAAGGTCACCAAGTCTGCCCAGAAAGCTCAGAAGGCTAAATGAATATTATCCCTAATACCTGC
CACCCCACTCTTAATCAGTGGTGGAAGAACG 10 (0.000305%)

AGCTTCTCAGACTATCCACCTTTGGGTCGCTTTGCTGTTCGTGATATGAGACAGACAGTTGCGGTGGGTG
TCATCAAAGCAGTGGACAAGAAGGCTGCTG 6 (0.000183%)

AGGAAGGGCGCCGGGTCTGTGTTCCGCGCGCACGTGAAGCACCGTAAAGGCGCTGCGCGCCTGCGCGCC
GTGGATTTCGCTGAGCGGCACGGCTACATCA 5 (0.000152%)

AGGAATCACCTCCCATTCCGATAAAATCACCTTCCACCCTTACTACACAATCAAAGACGCCCTCGGCTTA
CTTCTCTTCATTCTCTCCTTAATGACATTA 4 (0.000122%)

AGGACACAGAGACTTTATCAAAAACATGATTACAGGGACATCTCAGGCTGACTGTGCTGTCCTGATTGTT
GCTGCTGGTGTTGGTGAATTTGAAGCTGGT 25 (0.000761%)

AGGAGACCCCATTCTATACCAACACCTATTCTGATTTTTCGGTCACCCTGAAGTTTATATTCTTATCCTAC
CAGGCTTCGGAATAATCTCCCATATTGTA 18 (0.000548%)

AGGAGAGGCACCCGATATATGTTCTCTAGGCCTTTTAGAAAACATGGAGTTGTTCCTTTGGCCACATATA
TGCGAATCTATAAGAAAGGTGATATTGTAG 8 (0.000244%)

AGGAGGAGACCCCATTCTATACCAACACCTATTCTGATTTTTCGGTCACCCTGAAGTTTATATTCTTATCC
TACCAGGCTTCGGAATAATCTCCCATATT 613 (0.018667%)

AGGAGGCTGCTGAGATGGGAAAGGGCTCCTTCAAGTATGCCTGGGTCTTGGATAAACTGAAAGCTGAGC
GTGAACGTGGTATCACCATTGATATCTCCTT 4 (0.000122%)

AGGCAACCTTCTAGGTAACGACCACATCTACAACGTTATCGTCACAGCCCATGCATTTGTAATAATCTTC
TTCATAGTAATACCCATCATAATCGGAGGC 18 (0.000548%)

AGGCACAGCTCTAAGCCTCCTTATTCGAGCCGAGCTGGGCCAGCCAGGCAACCTTCTAGGTAACGACCA
CATCTACAACGTTATCGTCACAGCCCATGCA 3 (0.000091%)

AGGCACCCGATATATGTTCTCTAGGCCTTTTAGAAAACATGGAGTTGTTCCTTTGGCCACATATATGCGA
ATCTATAAGAAAGGTGATATTGTAGACATC 3 (0.000091%)

AGGCCATCTCCTGGGCCGCCTGGCGGCCATCGTGGCTAAACAGGTACTGCTGGGCCGGAAGGTGGTGGT
CGTACGCTGTGAAGGCATCAACATTTCTGGC 11 (0.000335%)

AGGCCTTCGATACGGGATAATCCTATTTATTACCTCAGAAGTTTTTTTCTTCGCAGGATTTTTCTGAGCCT
TTTACCACTCCAGCCTAGCCCCTACCCCC 5 (0.000152%)

AGGCTCCCTTCCCCTACTCATCGCACTGATTTACACTCACAACACCCTAGGCTCACTAAACATTCTACTAC
TCACTCTCACTGCCCAAGAACTATCAAAC 5 (0.000152%)

AGGCTGACTGTGCTGTCCTGATTGTTGCTGCTGGTGTTGGTGAATTTGAAGCTGGTATCTCCAAGAATGG
GCAGACCCGAGAGCATGCCCTTCTGGCTTA 12 (0.000365%)

AGGCTGCTGGAGCTGGCAAGGTCACCAAGTCTGCCCAGAAAGCTCAGAAGGCTAAATGAATATTATCCC
TAATACCTGCCACCCCACTCTTAATCAGTGG 19 (0.000579%)

AGGCTTCGGAATAATCTCCCATATTGTAACTTACTACTCCGGAAAAAAAGAACCATTTGGATACATAGGT
ATGGTCTGAGCTATGATATCAATTGGCTTC 3 (0.000091%)

AGGGATGGAAAGTCACCCGTAAGGATGGCAATGCCAGTGGAACCACGCTGCTTGAGGCTCTGGACTGCA
TCCTACCACCAACTCGTCCAACTGACAAGCC 3 (0.000091%)

AGGGATTGTGTTTAAAGTAGTGCTTCTACCAACATGTCCCGTGGTTCCAGCGCCGGTTTTGACCGCCACA
TTACCATTTTTTCACCCGAGGGTCGGCTCT3 (0.000091%)

AGGGCTCCTTCAAGTATGCCTGGGTCTTGGATAAACTGAAAGCTGAGCGTGAACGTGGTATCACCATTG
ATATCTCCTTGTGGAAATTTGAGACCAGCAA 10 (0.000305%)

AGGGGCCCTCTCAGCCCTCCTAATGACCTCCGGCCTAGCCATGTGATTTCACTTCCACTCCATAACGCTC
CTCATACTAGGCCTACTAACCAACACACTA 12 (0.000365%)

AGGTCAACGATCCCTCCCTTACCATCAAATCAATTGGCCACCAATGGTACTGAACCTACGAGTACACCGA
CTACGGCGGACTAATCTTCAACTCCTACAT 5 (0.000152%)

AGGTGTCGTGAAAACTACCCCTAAAAGCCAAAATGGGAAGGAAAAGACTCATATCAACATTGTCGTCA
TTGGACACGTAGATTCGGGCAAGTCCACCAC 10 (0.000305%)

AGTAGGCTCCCTTCCCCTACTCATCGCACTGATTTACACTCACAACACCCTAGGCTCACTAAACATTCTA
CTACTCACTCTCACTGCCCAAGAACTATCA 5 (0.000152%)

AGTCCTCATCGCCCTCCCATCCCTACGCATCCTTTACATAACAGACGAGGTCAACGATCCCTCCCTTACC
ATCAAATCAATTGGCCACCAATGGTACTGA 8 (0.000244%)

AGTCTGGTGATGCTGCCATTGTTGATATGGTTCCTGGCAAGCCCATGTGTGTTGAGAGCTTCTCAGACTA
TCCACCTTTGGGTCGCTTTGCTGTTCGTGA 19 (0.000579%)

AGTGGACAAGAAGGCTGCTGGAGCTGGCAAGGTCACCAAGTCTGCCCAGAAAGCTCAGAAGGCTAAAT
GAATATTATCCCTAATACCTGCCACCCCACTC 11 (0.000335%)

AGTGGAGACTGGTGTTCTCAAACCCGGTATGGTGGTCACCTTTGCTCCAGTCAACGTTACAACGGAAGTA
AAATCTGTCGAAATGCACCATGAAGCTTTG 5 (0.000152%)

AGTGGGAGACAGCAGCACCAGCGGTGGCAGAGACCCCAGACATCAAGCTCTTTGGGAAGTGGAGCACC
GATGATGTGCAGATCAATGACATTTCCCTGCA 232 (0.007065%)

AGTTCCCCTAATAATCGGTGCCCCCGATATGGCGTTTCCCCGCATAAACAACATAAGCTTCTGACTCTTA
CCTCCCTCTCTCCTACTCCTGCTCGCATCT 8 (0.000244%)

AGTTGCGGTGGGTGTCATCAAAGCAGTGGACAAGAAGGCTGCTGGAGCTGGCAAGGTCACCAAGTCTGC
CCAGAAAGCTCAGAAGGCTAAATGAATATTA 4 (0.000122%)

AGTTTTTTTCTTCGCAGGATTTTTCTGAGCCTTTTACCACTCCAGCCTAGCCCCTACCCCCCAATTAGGAG
GGCACTGGCCCCCAACAGGCATCACCCCG4 (0.000122%)

ATAAAAAATTATAACAAACCCTGAGAACCAAAATGAACGAAAATCTGTTCGCTTCATTCATTGCCCCCA
CAATCCTAGGCCTACCCGCCGCAGTACTGAT 5 (0.000152%)

ATAAAAATAAAAAATTATAACAAACCCTGAGAACCAAAATGAACGAAAATCTGTTCGCTTCATTCATTG
CCCCCACAATCCTAGGCCTACCCGCCGCAGT 18 (0.000548%)

ATAAAATCACCTTCCACCCTTACTACACAATCAAAGACGCCCTCGGCTTACTTCTCTTCATTCTCTCCTTA
ATGACATTAACACTATTCTCACCAGACCT 64 (0.001949%)

ATAAACCTAGCCATGGCCATCCCCTTATGAGCGGGCGCAGTGATTATAGGCTTTCGCTCTAAGATTAAAA
ATGCCCTAGCCCACTTCTTACCACAAGGCA 13 (0.000396%)

ATAAACTGAAAGCTGAGCGTGAACGTGGTATCACCATTGATATCTCCTTGTGGAAATTTGAGACCAGCA
AGTACTATGTGACTATCATTGATGCCCCAGG 50 (0.001523%)

ATAAATGCGGTGGCATCGACAAAAGAACCATTGAAAAATTTGAGAAGGAGGCTGCTGAGATGGGAAAG
GGCTCCTTCAAGTATGCCTGGGTCTTGGATAA 3 (0.000091%)

ATAACTGAACTCCTCACACCCAATTGGACCAATCTATCACCCTATAGAAGAACTAATGTTAGTATAAGTA
ACATGAAAACATTCTCCTCCGCATAAGCCT 7 (0.000213%)

ATAATCGGTGCCCCCGATATGGCGTTTCCCCGCATAAACAACATAAGCTTCTGACTCTTACCTCCCTCTCT

CCTACTCCTGCTCGCATCTGCTATAGTGG 6 (0.000183%)

ATAATCTCCCATATTGTAACTTACTACTCCGGAAAAAAAGAACCATTTGGATACATAGGTATGGTCTGAG
CTATGATATCAATTGGCTTCCTAGGGTTTA 15 (0.000457%)

ATAATTCAAGCACTGCTTATTACAATTTTACTGGGTCTCTATTTTACCCTCCTACAAGCCTCAGAGTACTT
CGAGTCTCCCTTCACCATTTCCGACGGCA 5 (0.000152%)

ATACGGGATAATCCTATTTATTACCTCAGAAGTTTTTTTCTTCGCAGGATTTTTCTGAGCCTTTTACCACT
CCAGCCTAGCCCCTACCCCCCAATTAGGA 6 (0.000183%)

ATACTTCCCCCATTATTCCTAGAACCAGGCGACCTGCGACTCCTTGACGTTGACAATCGAGTAGTACTCC
CGATTGAAGCCCCCATTCGTATAATAATTA 8 (0.000244%)

ATAGAAACCGTCTGAACTATCCTGCCCGCCATCATCCTAGTCCTCATCGCCCTCCCATCCCTACGCATCCT
TTACATAACAGACGAGGTCAACGATCCCT 643 (0.019581%)

ATATAACTGAACTCCTCACACCCAATTGGACCAATCTATCACCCTATAGAAGAACTAATGTTAGTATAAG
TAACATGAAAACATTCTCCTCCGCATAAGC 23 (0.000700%)

ATATCTCCTTGTGGAAATTTGAGACCAGCAAGTACTATGTGACTATCATTGATGCCCCAGGACACAGAGA
CTTTATCAAAAACATGATTACAGGGACATC 5 (0.000152%)

ATATCTGGAAAGCGGTCTGCCCCTGGAGGTGGTAGCAAGGTTCCACAGAAAAAAGTAAAACTTGCTGCT
GATGAAGATGATGACGATGATGATGAAGAGG 270 (0.008222%)

ATCAAAAACATGATTACAGGGACATCTCAGGCTGACTGTGCTGTCCTGATTGTTGCTGCTGGTGTTGGTG
AATTTGAAGCTGGTATCTCCAAGAATGGGC 13 (0.000396%)

ATCAAAGCAGTGGACAAGAAGGCTGCTGGAGCTGGCAAGGTCACCAAGTCTGCCCAGAAAGCTCAGAA
GGCTAAATGAATATTATCCCTAATACCTGCCA 42 (0.001279%)

ATCACCATTGATATCTCCTTGTGGAAATTTGAGACCAGCAAGTACTATGTGACTATCATTGATGCCCCAG
GACACAGAGACTTTATCAAAAACATGATTA 29 (0.000883%)

ATCACCCTATAGAAGAACTAATGTTAGTATAAGTAACATGAAAACATTCTCCTCCGCATAAGCCTGCGTC
AGATTAAAACACTGAACTGACAATTAACAG 5 (0.000152%)

ATCACCTCCCATTCCGATAAAATCACCTTCCACCCTTACTACACAATCAAAGACGCCCTCGGCTTACTTCT
CTTCATTCTCTCCTTAATGACATTAACAC 4 (0.000122%)

ATCACCTTCCACCCTTACTACACAATCAAAGACGCCCTCGGCTTACTTCTCTTCATTCTCTCCTTAATGAC
ATTAACACTATTCTCACCAGACCTCCTAG 17 (0.000518%)

ATCATCCTAGTCCTCATCGCCCTCCCATCCCTACGCATCCTTTACATAACAGACGAGGTCAACGATCCCT
CCCTTACCATCAAATCAATTGGCCACCAAT 8 (0.000244%)

ATCATTGATGCCCCAGGACACAGAGACTTTATCAAAAACATGATTACAGGGACATCTCAGGCTGACTGT
GCTGTCCTGATTGTTGCTGCTGGTGTTGGTG 8 (0.000244%)

ATCCACCTTTGGGTCGCTTTGCTGTTCGTGATATGAGACAGACAGTTGCGGTGGGTGTCATCAAAGCAGT
GGACAAGAAGGCTGCTGGAGCTGGCAAGGT 5 (0.000152%)

ATCCCTACGCATCCTTTACATAACAGACGAGGTCAACGATCCCTCCCTTACCATCAAATCAATTGGCCAC
CAATGGTACTGAACCTACGAGTACACCGAC 3 (0.000091%)

ATCCGTGGACAGAGGAAGGGCGCCGGGTCTGTGTTCCGCGCGCACGTGAAGCACCGTAAAGGCGCTGCG
CGCCTGCGCGCCGTGGATTTCGCTGAGCGGC 4 (0.000122%)

ATCCTACCAGGCTTCGGAATAATCTCCCATATTGTAACTTACTACTCCGGAAAAAAAGAACCATTTGGAT
ACATAGGTATGGTCTGAGCTATGATATCAA 524 (0.015957%)

ATCCTAGTCCTCATCGCCCTCCCATCCCTACGCATCCTTTACATAACAGACGAGGTCAACGATCCCTCCCT
TACCATCAAATCAATTGGCCACCAATGGT 4 (0.000122%)

ATCGACAAAAGAACCATTGAAAAATTTGAGAAGGAGGCTGCTGAGATGGGAAAGGGCTCCTTCAAGTAT
GCCTGGGTCTTGGATAAACTGAAAGCTGAGC 4 (0.000122%)

ATCGCCCTCCCATCCCTACGCATCCTTTACATAACAGACGAGGTCAACGATCCCTCCCTTACCATCAAAT
CAATTGGCCACCAATGGTACTGAACCTACG 11 (0.000335%)

ATCGGTGCCCCCGATATGGCGTTTCCCCGCATAAACAACATAAGCTTCTGACTCTTACCTCCCTCTCTCCT
ACTCCTGCTCGCATCTGCTATAGTGGAGG 10 (0.000305%)

ATCTATAAACCTAGCCATGGCCATCCCCTTATGAGCGGGCGCAGTGATTATAGGCTTTCGCTCTAAGATT
AAAAATGCCCTAGCCCACTTCTTACCACAA 7 (0.000213%)

ATCTATAAATGCGGTGGCATCGACAAAAGAACCATTGAAAAATTTGAGAAGGAGGCTGCTGAGATGGG
AAAGGGCTCCTTCAAGTATGCCTGGGTCTTGG 249 (0.007583%)

ATCTATCACCCTATAGAAGAACTAATGTTAGTATAAGTAACATGAAAACATTCTCCTCCGCATAAGCCTG
CGTCAGATTAAAACACTGAACTGACAATTA 8 (0.000244%)

ATCTCAGGCTGACTGTGCTGTCCTGATTGTTGCTGCTGGTGTTGGTGAATTTGAAGCTGGTATCTCCAAG
AATGGGCAGACCCGAGAGCATGCCCTTCTG 5 (0.000152%)

ATCTCCTTCATCCCTCTCCAGAAGAGGAGAAGAGGAAACACAAGAAGAAACGCCTGGTGCAGAGCCCCA
ATTCCTACTTCATGGATGTGAAATGCCCAGG 4 (0.000122%)

ATCTCCTTGTGGAAATTTGAGACCAGCAAGTACTATGTGACTATCATTGATGCCCCAGGACACAGAGACT
TTATCAAAAACATGATTACAGGGACATCTC 12 (0.000365%)

ATCTCTCCCAGTCCTAGCTGCTGGCATCACTATACTACTAACAGACCGCAACCTCAACACCACCTTCTTC
GACCCCGCCGGAGGAGGAGACCCCATTCTA 3 (0.000091%)

ATCTGTTCGCTTCATTCATTGCCCCCACAATCCTAGGCCTACCCGCCGCAGTACTGATCATTCTATTTCCC
CCTCTATTGATCCCCACCTCCAAATATCT 4 (0.000122%)

ATGACCCCTAACAGGGGCCCTCTCAGCCCTCCTAATGACCTCCGGCCTAGCCATGTGATTTCACTTCCAC
TCCATAACGCTCCTCATACTAGGCCTACTA 25 (0.000761%)

ATGACCTCCGGCCTAGCCATGTGATTTCACTTCCACTCCATAACGCTCCTCATACTAGGCCTACTAACCA
ACACACTAACCATATACCAATGATGGCGCG 8 (0.000244%)

ATGAGACAGACAGTTGCGGTGGGTGTCATCAAAGCAGTGGACAAGAAGGCTGCTGGAGCTGGCAAGGT
CACCAAGTCTGCCCAGAAAGCTCAGAAGGCTA 15 (0.000457%)

ATGATTACAGGGACATCTCAGGCTGACTGTGCTGTCCTGATTGTTGCTGCTGGTGTTGGTGAATTTGAAG
CTGGTATCTCCAAGAATGGGCAGACCCGAG 7 (0.000213%)

ATGATTTTGGGAATTACAACAATCAGTCTTCAAATTTTGGACCCATGAAGGGAGGAAATTTTGGAGGCA
GAAGCTCTGGCCCCTATGGCGGTGGAGGCCA 4 (0.000122%)

ATGCCCTTCTGGCTTACACACTGGGTGTGAAACAACTAATTGTCGGTGTTAACAAAATGGATTCCACTGA
GCCACCCTACAGCCAGAAGAGATATGAGGA 7 (0.000213%)

ATGCGGTGGCATCGACAAAAGAACCATTGAAAAATTTGAGAAGGAGGCTGCTGAGATGGGAAAGGGCT
CCTTCAAGTATGCCTGGGTCTTGGATAAACTG 3 (0.000091%)

ATGGAAAGTCACCCGTAAGGATGGCAATGCCAGTGGAACCACGCTGCTTGAGGCTCTGGACTGCATCCT ACCACCAACTCGTCCAACTGACAAGCCCTTG 11 (0.000335%)

ATGGCCCTAAATTCTTGAAGTCTGGTGATGCTGCCATTGTTGATATGGTTCCTGGCAAGCCCATGTGTGTT GAGAGCTTCTCAGACTATCCACCTTTGGG 11 (0.000335%)

ATGGCGTTTCCCCGCATAAACAACATAAGCTTCTGACTCTTACCTCCCTCTCTCCTACTCCTGCTCGCATC TGCTATAGTGGAGGCCGGAGCAGGAACAG 5 (0.000152%)

ATGGTCGAGGCCATCTCCTGGGCCGCCTGGCGGCCATCGTGGCTAAACAGGTACTGCTGGGCCGGAAGG TGGTGGTCGTACGCTGTGAAGGCATCAACAT 4 (0.000122%)

ATGGTGGTCACCTTTGCTCCAGTCAACGTTACAACGGAAGTAAAATCTGTCGAAATGCACCATGAAGCTT TGAGTGAAGCTCTTCCTGGGGACAATGTGG 11 (0.000335%)

ATGGTTACTTGCTTCGTCTGTTCTGTGTTGGTTTTACTAAAAAACGCAACAATCAGATACGGAAGACCTC TTATGCTCAGCACCAACAGGTCCGCCAAAT 3 (0.000091%)

ATGGTTCCTGGCAAGCCCATGTGTGTTGAGAGCTTCTCAGACTATCCACCTTTGGGTCGCTTTGCTGTTCG TGATATGAGACAGACAGTTGCGGTGGGTG10 (0.000305%)

ATGTGACTATCATTGATGCCCCAGGACACAGAGACTTTATCAAAAACATGATTACAGGGACATCTCAGG CTGACTGTGCTGTCCTGATTGTTGCTGCTGG 10 (0.000305%)

ATGTTCTCTAGGCCTTTTAGAAAACATGGAGTTGTTCCTTTGGCCACATATATGCGAATCTATAAGAAAG GTGATATTGTAGACATCAAGGGAATGGGTA 7 (0.000213%)

ATTACCTCAGAAGTTTTTTTCTTCGCAGGATTTTTCTGAGCCTTTTACCACTCCAGCCTAGCCCCTACCCC CCAATTAGGAGGGCACTGGCCCCCAACAG 36 (0.001096%)

ATTCAACTTCACCCGTAACCCACCGCCATGGCCGAGGAAGGCATTGCTGCTGGAGGTGTAATGGACGTT AATACTGCTTTACAAGAGGTTCTGAAGACTG 15 (0.000457%)

ATTCAAGCACTGCTTATTACAATTTTACTGGGTCTCTATTTTACCCTCCTACAAGCCTCAGAGTACTTCGA GTCTCCCTTCACCATTTCCGACGGCATCT 44 (0.001340%)

ATTCACCCTCCACTTCCCGTCTCAGAATCTAAACGTGGTCACCTTCGAGTAGAGAGGCCCGCCCGCCCAC CGTGGGCAGTGCCACCCGCAGATGACACGC 251 (0.007644%)

ATTCATTGCCCCCACAATCCTAGGCCTACCCGCCGCAGTACTGATCATTCTATTTCCCCCTCTATTGATCC CCACCTCCAAATATCTCATCAACAACCGA 8 (0.000244%)

ATTCCTAGAACCAGGCGACCTGCGACTCCTTGACGTTGACAATCGAGTAGTACTCCCGATTGAAGCCCCC ATTCGTATAATAATTACATCACAAGACGTC 4 (0.000122%)

ATTCGAGCCGAGCTGGGCCAGCCAGGCAACCTTCTAGGTAACGACCACATCTACAACGTTATCGTCACA GCCCATGCATTTGTAATAATCTTCTTCATAG 14 (0.000426%)

ATTCGGGCAAGTCCACCACTACTGGCCATCTGATCTATAAATGCGGTGGCATCGACAAAAGAACCATTG AAAAATTTGAGAAGGAGGCTGCTGAGATGGG 36 (0.001096%)

ATTCTACACCCTAGTAGGCTCCCTTCCCCTACTCATCGCACTGATTTACACTCACAACACCCTAGGCTCAC TAAACATTCTACTACTCACTCTCACTGCC 6 (0.000183%)

ATTCTTGAAGTCTGGTGATGCTGCCATTGTTGATATGGTTCCTGGCAAGCCCATGTGTGTTGAGAGCTTCT CAGACTATCCACCTTTGGGTCGCTTTGCT 20 (0.000609%)

ATTGATATCTCCTTGTGGAAATTTGAGACCAGCAAGTACTATGTGACTATCATTGATGCCCCAGGACACA

GAGACTTTATCAAAAACATGATTACAGGGA 38 (0.001157%)

ATTGATGCCCCAGGACACAGAGACTTTATCAAAAACATGATTACAGGGACATCTCAGGCTGACTGTGCT
GTCCTGATTGTTGCTGCTGGTGTTGGTGAAT 31 (0.000944%)

ATTGCCACAACTAACCTCCTCGGACTCCTGCCTCACTCATTTACACCAACCACCCAACTATCTATAAACC
TAGCCATGGCCATCCCCTTATGAGCGGGCG 3 (0.000091%)

ATTGCCACACGGCTCACATTGCATGCAAGTTTGCTGAGCTGAAGGAAAAGATTGATCGCCGTTCTGGTAA
AAAGCTGGAAGATGGCCCTAAATTCTTGAA 11 (0.000335%)

ATTGGACACGTAGATTCGGGCAAGTCCACCACTACTGGCCATCTGATCTATAAATGCGGTGGCATCGAC
AAAAGAACCATTGAAAAATTTGAGAAGGAGG 6 (0.000183%)

ATTGGACCAATCTATCACCCTATAGAAGAACTAATGTTAGTATAAGTAACATGAAAACATTCTCCTCCGC
ATAAGCCTGCGTCAGATTAAAACACTGAAC 8 (0.000244%)

ATTGTAACTTACTACTCCGGAAAAAAAGAACCATTTGGATACATAGGTATGGTCTGAGCTATGATATCAA
TTGGCTTCCTAGGGTTTATCGTGTGAGCAC 24 (0.000731%)

ATTGTCGTCATTGGACACGTAGATTCGGGCAAGTCCACCACTACTGGCCATCTGATCTATAAATGCGGTG
GCATCGACAAAAGAACCATTGAAAAATTTG 13 (0.000396%)

ATTGTGTTTAAAGTAGTGCTTCTACCAACATGTCCCGTGGTTCCAGCGCCGGTTTTGACCGCCACATTACC
ATTTTTTCACCCGAGGGTCGGCTCTACCA 6 (0.000183%)

ATTGTTGATATGGTTCCTGGCAAGCCCATGTGTGTTGAGAGCTTCTCAGACTATCCACCTTTGGGTCGCTT
TGCTGTTCGTGATATGAGACAGACAGTTG 11 (0.000335%)

ATTTATTACCTCAGAAGTTTTTTTCTTCGCAGGATTTTTCTGAGCCTTTTACCACTCCAGCCTAGCCCCTAC
CCCCCAATTAGGAGGGCACTGGCCCCCA 15 (0.000457%)

ATTTGAGAAGGAGGCTGCTGAGATGGGAAAGGGCTCCTTCAAGTATGCCTGGGTCTTGGATAAACTGAA
AGCTGAGCGTGAACGTGGTATCACCATTGAT 528 (0.016079%)

ATTTGAGACCAGCAAGTACTATGTGACTATCATTGATGCCCCAGGACACAGAGACTTTATCAAAAACAT
GATTACAGGGACATCTCAGGCTGACTGTGCT 153 (0.004659%)

ATTTGTGCCAATTTCTGGTTGGAATGGTGACAACATGCTGGAGCCAAGTGCTAACATGCCTTGGTTCAAG
GGATGGAAAGTCACCCGTAAGGATGGCAAT 55 (0.001675%)

ATTTTTATTGCCACAACTAACCTCCTCGGACTCCTGCCTCACTCATTTACACCAACCACCCAACTATCTAT
AAACCTAGCCATGGCCATCCCCTTATGAG 8 (0.000244%)

CAAAAACATGATTACAGGGACATCTCAGGCTGACTGTGCTGTCCTGATTGTTGCTGCTGGTGTTGGTGAA
TTTGAAGCTGGTATCTCCAAGAATGGGCAG 103 (0.003137%)

CAAAAAGGCCTTCGATACGGGATAATCCTATTTATTACCTCAGAAGTTTTTTTCTTCGCAGGATTTTTCTG
AGCCTTTTACCACTCCAGCCTAGCCCCTA 728 (0.022169%)

CAAAACCAAGATGAAGAGACGAGCTGTGGGGATCTGGCACTGTGGTTCCTGCATGAAGACAGTGGCTGG
CGGTGCCTGGACGTACAATACCACTTCCGCT 223 (0.006791%)

CAAACATATAACTGAACTCCTCACACCCAATTGGACCAATCTATCACCCTATAGAAGAACTAATGTTAGT
ATAAGTAACATGAAAACATTCTCCTCCGCA 66 (0.002010%)

CAAACCCGGTATGGTGGTCACCTTTGCTCCAGTCAACGTTACAACGGAAGTAAAATCTGTCGAAATGCA
CCATGAAGCTTTGAGTGAAGCTCTTCCTGGG 14 (0.000426%)

CAAAGCAGTGGACAAGAAGGCTGCTGGAGCTGGCAAGGTCACCAAGTCTGCCCAGAAAGCTCAGAAGG
CTAAATGAATATTATCCCTAATACCTGCCACC 24 (0.000731%)

CAAAGCCCATAAAAATAAAAAATTATAACAAACCCTGAGAACCAAAATGAACGAAAATCTGTTCGCTTC
ATTCATTGCCCCCACAATCCTAGGCCTACCC 18 (0.000548%)

CAAAGGATCTCCTTCATCCCTCTCCAGAAGAGGAGAAGAGGAAACACAAGAAGAAACGCCTGGTGCAG
AGCCCCAATTCCTACTTCATGGATGTGAAATG 5 (0.000152%)

CAAATAATTCAAGCACTGCTTATTACAATTTTACTGGGTCTCTATTTTACCCTCCTACAAGCCTCAGAGTA
CTTCGAGTCTCCCTTCACCATTTCCGACG 3 (0.000091%)

CAACAACCGACTAATCACCACCCAACAATGACTAATCAAACTAACCTCAAAACAAATGATAACCATACA
CAACACTAAAGGACGAACCTGATCTCTTATA 813 (0.024758%)

CAACCACCCAACTATCTATAAACCTAGCCATGGCCATCCCCTTATGAGCGGGCGCAGTGATTATAGGCTT
TCGCTCTAAGATTAAAAATGCCCTAGCCCA 76 (0.002314%)

CAACCGACTAATCACCACCCAACAATGACTAATCAAACTAACCTCAAAACAAATGATAACCATACACAA
CACTAAAGGACGAACCTGATCTCTTATACTA 18 (0.000548%)

CAACCTTCTAGGTAACGACCACATCTACAACGTTATCGTCACAGCCCATGCATTTGTAATAATCTTCTTC
ATAGTAATACCCATCATAATCGGAGGCTTT 10 (0.000305%)

CAACGTTGTAGGCCCCTACGGGCTACTACAACCCTTCGCTGACGCCATAAAACTCTTCACCAAAGAGCCC
CTAAAACCCGCCACATCTACCATCACCCTC 26 (0.000792%)

CAACTAACCTCCTCGGACTCCTGCCTCACTCATTTACACCAACCACCCAACTATCTATAAACCTAGCCAT
GGCCATCCCCTTATGAGCGGGCGCAGTGAT 9 (0.000274%)

CAACTAATTGTCGGTGTTAACAAAATGGATTCCACTGAGCCACCCTACAGCCAGAAGAGATATGAGGAA
ATTGTTAAGGAAGTCAGCACTTACATTAAGA 8 (0.000244%)

CAACTATCTATAAACCTAGCCATGGCCATCCCCTTATGAGCGGGCGCAGTGATTATAGGCTTTCGCTCTA
AGATTAAAAATGCCCTAGCCCACTTCTTAC 58 (0.001766%)

CAACTGACTAGTTCCCCTAATAATCGGTGCCCCCGATATGGCGTTTCCCCGCATAAACAACATAAGCTTC
TGACTCTTACCTCCCTCTCTCCTACTCCTG 362 (0.011024%)

CAACTTCACCCGTAACCCACCGCCATGGCCGAGGAAGGCATTGCTGCTGGAGGTGTAATGGACGTTAAT
ACTGCTTTACAAGAGGTTCTGAAGACTGCCC 3 (0.000091%)

CAAGAAGGCTGCTGGAGCTGGCAAGGTCACCAAGTCTGCCCAGAAAGCTCAGAAGGCTAAATGAATATT
ATCCCTAATACCTGCCACCCCACTCTTAATC 95 (0.002893%)

CAAGAATGTGTCTGTCAAGGATGTTCGTCGTGGCAACGTTGCTGGTGACAGCAAAAATGACCCACCAAT
GGAAGCAGCTGGCTTCACTGCTCAGGTGATT 76 (0.002314%)

CAAGACTACCGATGGTTACTTGCTTCGTCTGTTCTGTGTTGGTTTTACTAAAAAACGCAACAATCAGATA
CGGAAGACCTCTTATGCTCAGCACCAACAG 6 (0.000183%)

CAAGATTCAACTTCACCCGTAACCCACCGCCATGGCCGAGGAAGGCATTGCTGCTGGAGGTGTAATGGA
CGTTAATACTGCTTTACAAGAGGTTCTGAAG 744 (0.022657%)

CAAGCACTGCTTATTACAATTTTACTGGGTCTCTATTTTACCCTCCTACAAGCCTCAGAGTACTTCGAGTC
TCCCTTCACCATTTCCGACGGCATCTACG 11 (0.000335%)

CAAGCCCATGTGTGTTGAGAGCTTCTCAGACTATCCACCTTTGGGTCGCTTTGCTGTTCGTGATATGAGA
CAGACAGTTGCGGTGGGTGTCATCAAAGCA 6 (0.000183%)

CAAGGGATGGAAAGTCACCCGTAAGGATGGCAATGCCAGTGGAACCACGCTGCTTGAGGCTCTGGACTG
CATCCTACCACCAACTCGTCCAACTGACAAG 252 (0.007674%)

CAAGTACTATGTGACTATCATTGATGCCCCAGGACACAGAGACTTTATCAAAAACATGATTACAGGGAC
ATCTCAGGCTGACTGTGCTGTCCTGATTGTT 20 (0.000609%)

CAAGTATGCCTGGGTCTTGGATAAACTGAAAGCTGAGCGTGAACGTGGTATCACCATTGATATCTCCTTG
TGGAAATTTGAGACCAGCAAGTACTATGTG 20 (0.000609%)

CAAGTCCACCACTACTGGCCATCTGATCTATAAATGCGGTGGCATCGACAAAAGAACCATTGAAAAATT
TGAGAAGGAGGCTGCTGAGATGGGAAAGGGC 6 (0.000183%)

CAAGTGCTAACATGCCTTGGTTCAAGGGATGGAAAGTCACCCGTAAGGATGGCAATGCCAGTGGAACCA
CGCTGCTTGAGGCTCTGGACTGCATCCTACC 12 (0.000365%)

CAATCTATCACCCTATAGAAGAACTAATGTTAGTATAAGTAACATGAAAACATTCTCCTCCGCATAAGCC
TGCGTCAGATTAAAACACTGAACTGACAAT 10 (0.000305%)

CAATGATTTTGGGAATTACAACAATCAGTCTTCAAATTTTGGACCCATGAAGGGAGGAAATTTTGGAGG
CAGAAGCTCTGGCCCCTATGGCGGTGGAGGC 13 (0.000396%)

CAATGTCAAGAATGTGTCTGTCAAGGATGTTCGTCGTGGCAACGTTGCTGGTGACAGCAAAAATGACCC
ACCAATGGAAGCAGCTGGCTTCACTGCTCAG 7 (0.000213%)

CAATTTTGGAGGTGGTGGAAGCTACAATGATTTTGGGAATTACAACAATCAGTCTTCAAATTTTGGACCC
ATGAAGGGAGGAAATTTTGGAGGCAGAAGC 23 (0.000700%)

CACAACTAACCTCCTCGGACTCCTGCCTCACTCATTTACACCAACCACCCAACTATCTATAAACCTAGCC
ATGGCCATCCCCTTATGAGCGGGCGCAGTG 13 (0.000396%)

CACAGAAAGTTCTCCGCTCCCAGACATGGGTCCCTCGGCTTCCTGCCTCGGAAGCGCAGCAGCAGGCAT
CGTGGGAAGGTGAAGAGCTTCCCTAAGGATG 294 (0.008953%)

CACAGAGACTTTATCAAAAACATGATTACAGGGACATCTCAGGCTGACTGTGCTGTCCTGATTGTTGCTG
CTGGTGTTGGTGAATTTGAAGCTGGTATCT 8 (0.000244%)

CACAGCTCTAAGCCTCCTTATTCGAGCCGAGCTGGGCCAGCCAGGCAACCTTCTAGGTAACGACCACATC
TACAACGTTATCGTCACAGCCCATGCATTT 28 (0.000853%)

CACAGGTGTCGTGAAAACTACCCCTAAAAGCCAAAATGGGAAAGGAAAAGACTCATATCAACATTGTCG
TCATTGGACACGTAGATTCGGGCAAGTCCAC 173 (0.005268%)

CACATACTTCCTATTCTACACCCTAGTAGGCTCCCTTCCCCTACTCATCGCACTGATTTACACTCACAACA
CCCTAGGCTCACTAAACATTCTACTACTC 4 (0.000122%)

CACCAAAGCCCATAAAAATAAAAAATTATAACAAACCCTGAGAACCAAAATGAACGAAAATCTGTTCGC
TTCATTCATTGCCCCCACAATCCTAGGCCTA 9 (0.000274%)

CACCAACAGCAGCAACAGCCACCACCGCAGCAGCCACCGCCGCAGCAGCCGCCACCGCATCAGCCGCC
GCCGCATCCACAGCCGCATCAGCAGCAGCAGC 9 (0.000274%)

CACCAACCACCCAACTATCTATAAACCTAGCCATGGCCATCCCCTTATGAGCGGGCGCAGTGATTATAGG
CTTTCGCTCTAAGATTAAAAATGCCCTAGC 20 (0.000609%)

CACCACTACTGGCCATCTGATCTATAAATGCGGTGGCATCGACAAAAGAACCATTGAAAAATTTGAGAA
GGAGGCTGCTGAGATGGGAAAGGGCTCCTTC 23 (0.000700%)

CACCATATATTTACAGTAGGAATAGACGTAGACACACGAGCATATTTCACCTCCGCTACCATAATCATCG

CTATCCCCACCGGCGTCAAAGTATTTAGCT 434 (0.013216%)

CACCATTGATATCTCCTTGTGGAAATTTGAGACCAGCAAGTACTATGTGACTATCATTGATGCCCCAGGA
CACAGAGACTTTATCAAAAACATGATTACA 7 (0.000213%)

CACCCAACTATCTATAAACCTAGCCATGGCCATCCCCTTATGAGCGGGCGCAGTGATTATAGGCTTTCGC
TCTAAGATTAAAAATGCCCTAGCCCACTTC 7 (0.000213%)

CACCCGTAACCCACCGCCATGGCCGAGGAAGGCATTGCTGCTGGAGGTGTAATGGACGTTAATACTGCT
TTACAAGAGGTTCTGAAGACTGCCCTCATCC 11 (0.000335%)

CACCCTAGTAGGCTCCCTTCCCCTACTCATCGCACTGATTTACACTCACAACACCCTAGGCTCACTAAAC
ATTCTACTACTCACTCTCACTGCCCAAGAA 4 (0.000122%)

CACCCTCCACTTCCCGTCTCAGAATCTAAACGTGGTCACCTTCGAGTAGAGAGGCCCGCCCGCCCACCGT
GGGCAGTGCCACCCGCAGATGACACGCGCT 4 (0.000122%)

CACCTTCCACCCTTACTACACAATCAAAGACGCCCTCGGCTTACTTCTCTTCATTCTCTCCTTAATGACAT
TAACACTATTCTCACCAGACCTCCTAGGC 6 (0.000183%)

CACCTTTGCTCCAGTCAACGTTACAACGGAAGTAAAATCTGTCGAAATGCACCATGAAGCTTTGAGTGA
AGCTCTTCCTGGGGACAATGTGGGCTTCAAT 26 (0.000792%)

CACCTTTGGGTCGCTTTGCTGTTCGTGATATGAGACAGACAGTTGCGGTGGGTGTCATCAAAGCAGTGGA
CAAGAAGGCTGCTGGAGCTGGCAAGGTCAC 6 (0.000183%)

CACTACTGGCCATCTGATCTATAAATGCGGTGGCATCGACAAAAGAACCATTGAAAAATTTGAGAAGGA
GGCTGCTGAGATGGGAAAGGGCTCCTTCAAG 12 (0.000365%)

CACTCATTTACACCAACCACCCAACTATCTATAAACCTAGCCATGGCCATCCCCTTATGAGCGGGCGCAG
TGATTATAGGCTTTCGCTCTAAGATTAAAA 6 (0.000183%)

CACTGGGCGACTCTGTGCCTCGCTGAGGAAAAATAACTAAACATGGGCAAAGGAGATCCTAAGAAGCCG
AGAGGCAAAATGTCATCATATGCATTTTTG 6 (0.000183%)

CACTGGGTGTGAAACAACTAATTGTCGGTGTTAACAAAATGGATTCCACTGAGCCACCCTACAGCCAGA
AGAGATATGAGGAAATTGTTAAGGAAGTCAG 3 (0.000091%)

CAGAAAGTTCTCCGCTCCCAGACATGGGTCCCTCGGCTTCCTGCCTCGGAAGCGCAGCAGCAGGCATCGT
GGGAAGGTGAAGAGCTTCCCTAAGGATGAC 9 (0.000274%)

CAGAACACAGGTGTCGTGAAAACTACCCCTAAAAGCCAAAATGGGAAAGGAAAAGACTCATATCAACA
TTGTCGTCATTGGACACGTAGATTCGGGCAAG 170 (0.005177%)

CAGAACGCCTGAACGCAGGCACATACTTCCTATTCTACACCCTAGTAGGCTCCCTTCCCCTACTCATCGC
ACTGATTTACACTCACAACACCCTAGGCTC 8 (0.000244%)

CAGAAGTTTTTTTCTTCGCAGGATTTTTCTGAGCCTTTTACCACTCCAGCCTAGCCCCTACCCCCCAATTA
GGAGGGCACTGGCCCCCAACAGGCATCAC 5 (0.000152%)

CAGACAGTTGCGGTGGGTGTCATCAAAGCAGTGGACAAGAAGGCTGCTGGAGCTGGCAAGGTCACCAA
GTCTGCCCAGAAAGCTCAGAAGGCTAAATGAA 118 (0.003593%)

CAGACATGGGTCCCTCGGCTTCCTGCCTCGGAAGCGCAGCAGCAGGCATCGTGGGAAGGTGAAGAGCTT
CCCTAAGGATGACCCGTCCAAGCCGGTCCAC 9 (0.000274%)

CAGACCCGAGAGCATGCCCTTCTGGCTTACACACTGGGTGTGAAACAACTAATTGTCGGTGTTAACAAA
ATGGATTCCACTGAGCCACCCTACAGCCAGA 11 (0.000335%)

CAGACTATCCACCTTTGGGTCGCTTTGCTGTTCGTGATATGAGACAGACAGTTGCGGTGGGTGTCATCAA
AGCAGTGGACAAGAAGGCTGCTGGAGCTGG 6 (0.000183%)

CAGAGACTTTATCAAAAACATGATTACAGGGACATCTCAGGCTGACTGTGCTGTCCTGATTGTTGCTGCT
GGTGTTGGTGAATTTGAAGCTGGTATCTCC 22 (0.000670%)

CAGAGGAAGGGCGCCGGGTCTGTGTTCCGCGCGCACGTGAAGCACCGTAAAGGCGCTGCGCGCCTGCGC
GCCGTGGATTTCGCTGAGCGGCACGGCTACA 5 (0.000152%)

CAGCAAGTACTATGTGACTATCATTGATGCCCCAGGACACAGAGACTTTATCAAAAACATGATTACAGG
GACATCTCAGGCTGACTGTGCTGTCCTGATT 15 (0.000457%)

CAGCACCAGCGGTGGCAGAGACCCCAGACATCAAGCTCTTTGGGAAGTGGAGCACCGATGATGTGCAGA
TCAATGACATTTCCCTGCAGGATTACATTGC 4 (0.000122%)

CAGCAGCACCAGCGGTGGCAGAGACCCCAGACATCAAGCTCTTTGGGAAGTGGAGCACCGATGATGTGC
AGATCAATGACATTTCCCTGCAGGATTACAT 54 (0.001644%)

CAGCCCATGACCCCTAACAGGGGCCCTCTCAGCCCTCCTAATGACCTCCGGCCTAGCCATGTGATTTCAC
TTCCACTCCATAACGCTCCTCATACTAGGC 37 (0.001127%)

CAGCCCTCCTAATGACCTCCGGCCTAGCCATGTGATTTCACTTCCACTCCATAACGCTCCTCATACTAGGC
CTACTAACCAACACACTAACCATATACCA 10 (0.000305%)

CAGCTCCTCCCTGGAGAAGAGCTACGAGCTGCCTGACGGCCAGGTCATCACCATTGGCAATGAGCGGTT
CCGCTGCCCTGAGGCACTCTTCCAGCCTTCC 29 (0.000883%)

CAGCTCTAAGCCTCCTTATTCGAGCCGAGCTGGGCCAGCCAGGCAACCTTCTAGGTAACGACCACATCTA
CAACGTTATCGTCACAGCCCATGCATTTGT 14 (0.000426%)

CAGGACACAGAGACTTTATCAAAAACATGATTACAGGGACATCTCAGGCTGACTGTGCTGTCCTGATTGT
TGCTGCTGGTGTTGGTGAATTTGAAGCTGG 14 (0.000426%)

CAGGCAACCTTCTAGGTAACGACCACATCTACAACGTTATCGTCACAGCCCATGCATTTGTAATAATCTT
CTTCATAGTAATACCCATCATAATCGGAGG 4 (0.000122%)

CAGGCACATACTTCCTATTCTACACCCTAGTAGGCTCCCTTCCCCTACTCATCGCACTGATTTACACTCAC
AACACCCTAGGCTCACTAAACATTCTACT 12 (0.000365%)

CAGGCTGAAGCGCAAAAGAAGAAAGATGAGGCAGAGGTCCAAGTAAACCGCTAGCTTGTTGCACCGTG
GAGGCCACAGGAGCAGAAACATGGAATGCCAG 13 (0.000396%)

CAGGCTGACTGTGCTGTCCTGATTGTTGCTGCTGGTGTTGGTGAATTTGAAGCTGGTATCTCCAAGAATG
GGCAGACCCGAGAGCATGCCCTTCTGGCTT 4 (0.000122%)

CAGGCTTCGGAATAATCTCCCATATTGTAACTTACTACTCCGGAAAAAAAGAACCATTTGGATACATAGG
TATGGTCTGAGCTATGATATCAATTGGCTT 6 (0.000183%)

CAGGGATTGTGTTTAAAGTAGTGCTTCTACCAACATGTCCCGTGGTTCCAGCGCCGGTTTTGACCGCCAC
ATTACCATTTTTTCACCCGAGGGTCGGCTC 15 (0.000457%)

CAGGGGCCCTCTCAGCCCTCCTAATGACCTCCGGCCTAGCCATGTGATTTCACTTCCACTCCATAACGCT
CCTCATACTAGGCCTACTAACCAACACACT 18 (0.000548%)

CAGGTGTCGTGAAAACTACCCCTAAAAGCCAAAATGGGAAAGGAAAAGACTCATATCAACATTGTCGTC
ATTGGACACGTAGATTCGGGCAAGTCCACCA 67 (0.002040%)

CAGTCCTAGCTGCTGGCATCACTATACTACTAACAGACCGCAACCTCAACACCACCTTCTTCGACCCCGC
CGGAGGAGGAGACCCCATTCTATACCAACA 13 (0.000396%)

CAGTGGACAAGAAGGCTGCTGGAGCTGGCAAGGTCACCAAGTCTGCCCAGAAAGCTCAGAAGGCTAAA TGAATATTATCCCTAATACCTGCCACCCCACT 13 (0.000396%)

CAGTTGCGGTGGGTGTCATCAAAGCAGTGGACAAGAAGGCTGCTGGAGCTGGCAAGGTCACCAAGTCTG CCCAGAAAGCTCAGAAGGCTAAATGAATATT 9 (0.000274%)

CATAAAAATAAAAAATTATAACAAACCCTGAGAACCAAAATGAACGAAAATCTGTTCGCTTCATTCATT GCCCCCACAATCCTAGGCCTACCCGCCGCAG 6 (0.000183%)

CATACTTCCCCCATTATTCCTAGAACCAGGCGACCTGCGACTCCTTGACGTTGACAATCGAGTAGTACTC CCGATTGAAGCCCCCATTCGTATAATAATT 5 (0.000152%)

CATACTTCCTATTCTACACCCTAGTAGGCTCCCTTCCCCTACTCATCGCACTGATTTACACTCACAACACC CTAGGCTCACTAAACATTCTACTACTCAC 10 (0.000305%)

CATATATTTACAGTAGGAATAGACGTAGACACACGAGCATATTTCACCTCCGCTACCATAATCATCGCTA TCCCCACCGGCGTCAAAGTATTTAGCTGAC 5 (0.000152%)

CATATTGTAACTTACTACTCCGGAAAAAAAGAACCATTTGGATACATAGGTATGGTCTGAGCTATGATAT CAATTGGCTTCCTAGGGTTTATCGTGTGAG 10 (0.000305%)

CATCAAAGCAGTGGACAAGAAGGCTGCTGGAGCTGGCAAGGTCACCAAGTCTGCCCAGAAAGCTCAGA AGGCTAAATGAATATTATCCCTAATACCTGCC 36 (0.001096%)

CATCATCCTAGTCCTCATCGCCCTCCCATCCCTACGCATCCTTTACATAACAGACGAGGTCAACGATCCC TCCCTTACCATCAAATCAATTGGCCACCAA 9 (0.000274%)

CATCCCTACGCATCCTTTACATAACAGACGAGGTCAACGATCCCTCCCTTACCATCAAATCAATTGGCCA CCAATGGTACTGAACCTACGAGTACACCGA 7 (0.000213%)

CATCCCTCTCCAGAAGAGGAGAAGAGGAAACACAAGAAGAAACGCCTGGTGCAGAGCCCCAATTCCTA CTTCATGGATGTGAAATGCCCAGGATGCTATA 4 (0.000122%)

CATCCTAGTCCTCATCGCCCTCCCATCCCTACGCATCCTTTACATAACAGACGAGGTCAACGATCCCTCC CTTACCATCAAATCAATTGGCCACCAATGG 5 (0.000152%)

CATCCTTTACATAACAGACGAGGTCAACGATCCCTCCCTTACCATCAAATCAATTGGCCACCAATGGTAC TGAACCTACGAGTACACCGACTACGGCGGA 3 (0.000091%)

CATCGACAAAAGAACCATTGAAAAATTTGAGAAGGAGGCTGCTGAGATGGGAAAGGGCTCCTTCAAGT ATGCCTGGGTCTTGGATAAACTGAAAGCTGAG 14 (0.000426%)

CATCGCCCTCCCATCCCTACGCATCCTTTACATAACAGACGAGGTCAACGATCCCTCCCTTACCATCAAA TCAATTGGCCACCAATGGTACTGAACCTAC 7 (0.000213%)

CATCTCCTGGGCCGCCTGGCGGCCATCGTGGCTAAACAGGTACTGCTGGGCCGGAAGGTGGTGGTCGTA CGCTGTGAAGGCATCAACATTTCTGGCAATT 4 (0.000122%)

CATGACCCCTAACAGGGGCCCTCTCAGCCCTCCTAATGACCTCCGGCCTAGCCATGTGATTTCACTTCCA CTCCATAACGCTCCTCATACTAGGCCTACT 22 (0.000670%)

CATGATTACAGGGACATCTCAGGCTGACTGTGCTGTCCTGATTGTTGCTGCTGGTGTTGGTGAATTTGAA GCTGGTATCTCCAAGAATGGGCAGACCCGA 29 (0.000883%)

CATTGATATCTCCTTGTGGAAATTTGAGACCAGCAAGTACTATGTGACTATCATTGATGCCCCAGGACAC AGAGACTTTATCAAAAACATGATTACAGGG 19 (0.000579%)

CATTGATGCCCCAGGACACAGAGACTTTATCAAAAACATGATTACAGGGACATCTCAGGCTGACTGTGC

TGTCCTGATTGTTGCTGCTGGTGTTGGTGAA 26 (0.000792%)

CATTGGACACGTAGATTCGGGCAAGTCCACCACTACTGGCCATCTGATCTATAAATGCGGTGGCATCGAC AAAAGAACCATTGAAAAATTTGAGAAGGAG 8 (0.000244%)

CATTGTCGTCATTGGACACGTAGATTCGGGCAAGTCCACCACTACTGGCCATCTGATCTATAAATGCGGT GGCATCGACAAAAGAACCATTGAAAAATTT 1259 (0.038340%)

CATTGTTGATATGGTTCCTGGCAAGCCCATGTGTGTTGAGAGCTTCTCAGACTATCCACCTTTGGGTCGCT TTGCTGTTCGTGATATGAGACAGACAGTT 8 (0.000244%)

CCAAAGCCCATAAAAATAAAAAATTATAACAAACCCTGAGAACCAAAATGAACGAAAATCTGTTCGCTT CATTCATTGCCCCCACAATCCTAGGCCTACC 18 (0.000548%)

CCAACTATCTATAAACCTAGCCATGGCCATCCCCTTATGAGCGGGCGCAGTGATTATAGGCTTTCGCTCT AAGATTAAAAATGCCCTAGCCCACTTCTTA 8 (0.000244%)

CCAAGATGAAGAGACGAGCTGTGGGGATCTGGCACTGTGGTTCCTGCATGAAGACAGTGGCTGGCGGTG CCTGGACGTACAATACCACTTCCGCTGTCAC 4 (0.000122%)

CCAAGTGCTAACATGCCTTGGTTCAAGGGATGGAAAGTCACCCGTAAGGATGGCAATGCCAGTGGAACC ACGCTGCTTGAGGCTCTGGACTGCATCCTAC 7 (0.000213%)

CCAATCTATCACCCTATAGAAGAACTAATGTTAGTATAAGTAACATGAAAACATTCTCCTCCGCATAAGC CTGCGTCAGATTAAAACACTGAACTGACAA 5 (0.000152%)

CCAATTGGACCAATCTATCACCCTATAGAAGAACTAATGTTAGTATAAGTAACATGAAAACATTCTCCTC CGCATAAGCCTGCGTCAGATTAAAACACTG 7 (0.000213%)

CCACAACTAACCTCCTCGGACTCCTGCCTCACTCATTTACACCAACCACCCAACTATCTATAAACCTAGC CATGGCCATCCCCTTATGAGCGGGCGCAGT 13 (0.000396%)

CCACCTTTGGGTCGCTTTGCTGTTCGTGATATGAGACAGACAGTTGCGGTGGGTGTCATCAAAGCAGTGG ACAAGAAGGCTGCTGGAGCTGGCAAGGTCA 6 (0.000183%)

CCACTACTGGCCATCTGATCTATAAATGCGGTGGCATCGACAAAAGAACCATTGAAAAATTTGAGAAGG AGGCTGCTGAGATGGGAAAGGGCTCCTTCAA 29 (0.000883%)

CCAGAACACAGGTGTCGTGAAAACTACCCCTAAAAGCCAAAATGGGAAAGGAAAAGACTCATATCAAC ATTGTCGTCATTGGACACGTAGATTCGGGCAA 258 (0.007857%)

CCAGAACGCCTGAACGCAGGCACATACTTCCTATTCTACACCCTAGTAGGCTCCCTTCCCCTACTCATCG CACTGATTTACACTCACAACACCCTAGGCT 8 (0.000244%)

CCAGACATGGGTCCCTCGGCTTCCTGCCTCGGAAGCGCAGCAGCAGGCATCGTGGGAAGGTGAAGAGCT TCCCTAAGGATGACCCGTCCAAGCCGGTCCA 6 (0.000183%)

CCAGCAAGTACTATGTGACTATCATTGATGCCCCAGGACACAGAGACTTTATCAAAAACATGATTACAG GGACATCTCAGGCTGACTGTGCTGTCCTGAT 14 (0.000426%)

CCAGCCCATGACCCCTAACAGGGGCCCTCTCAGCCCTCCTAATGACCTCCGGCCTAGCCATGTGATTTCA CTTCCACTCCATAACGCTCCTCATACTAGG 22 (0.000670%)

CCAGGACACAGAGACTTTATCAAAAACATGATTACAGGGACATCTCAGGCTGACTGTGCTGTCCTGATT GTTGCTGCTGGTGTTGGTGAATTTGAAGCTG 12 (0.000365%)

CCAGGCGACCTGCGACTCCTTGACGTTGACAATCGAGTAGTACTCCCGATTGAAGCCCCCATTCGTATAA TAATTACATCACAAGACGTCTTGCACTCAT 8 (0.000244%)

CCAGGGATTGTGTTTAAAGTAGTGCTTCTACCAACATGTCCCGTGGTTCCAGCGCCGGTTTTGACCGCCA
CATTACCATTTTTTCACCCGAGGGTCGGCT 4 (0.000122%)

CCAGTCCTAGCTGCTGGCATCACTATACTACTAACAGACCGCAACCTCAACACCACCTTCTTCGACCCCG
CCGGAGGAGGAGACCCCATTCTATACCAAC 3 (0.000091%)

CCATAAAAATAAAAAATTATAACAAACCCTGAGAACCAAAATGAACGAAAATCTGTTCGCTTCATTCAT
TGCCCCCACAATCCTAGGCCTACCCGCCGCA 3 (0.000091%)

CCATATTGTAACTTACTACTCCGGAAAAAAAGAACCATTTGGATACATAGGTATGGTCTGAGCTATGATA
TCAATTGGCTTCCTAGGGTTTATCGTGTGA 26 (0.000792%)

CCATCATCCTAGTCCTCATCGCCCTCCCATCCCTACGCATCCTTTACATAACAGACGAGGTCAACGATCC
CTCCCTTACCATCAAATCAATTGGCCACCA 7 (0.000213%)

CCATCCCTACGCATCCTTTACATAACAGACGAGGTCAACGATCCCTCCCTTACCATCAAATCAATTGGCC
ACCAATGGTACTGAACCTACGAGTACACCG 6 (0.000183%)

CCATGACCCCTAACAGGGGCCCTCTCAGCCCTCCTAATGACCTCCGGCCTAGCCATGTGATTTCACTTCC
ACTCCATAACGCTCCTCATACTAGGCCTAC 15 (0.000457%)

CCATGTTGGCATCTGCCCCTCCTCAAGAGCAAAAGCAAATGTTGGGTGAACGGCTGTTTCCTCTTATTCA
AGCCATGCACCCTACTCTTGCTGGTAAAAT 4 (0.000122%)

CCATTGATATCTCCTTGTGGAAATTTGAGACCAGCAAGTACTATGTGACTATCATTGATGCCCCAGGACA
CAGAGACTTTATCAAAAACATGATTACAGG 5 (0.000152%)

CCATTGTTGATATGGTTCCTGGCAAGCCCATGTGTGTTGAGAGCTTCTCAGACTATCCACCTTTGGGTCGC
TTTGCTGTTCGTGATATGAGACAGACAGT 8 (0.000244%)

CCCAAACATATAACTGAACTCCTCACACCCAATTGGACCAATCTATCACCCTATAGAAGAACTAATGTTA
GTATAAGTAACATGAAAACATTCTCCTCCG 571 (0.017388%)

CCCAACTATCTATAAACCTAGCCATGGCCATCCCCTTATGAGCGGGCGCAGTGATTATAGGCTTTCGCTC
TAAGATTAAAAATGCCCTAGCCCACTTCTT 27 (0.000822%)

CCCAATTGGACCAATCTATCACCCTATAGAAGAACTAATGTTAGTATAAGTAACATGAAAACATTCTCCT
CCGCATAAGCCTGCGTCAGATTAAAACACT 15 (0.000457%)

CCCAGCCCATGACCCCTAACAGGGGCCCTCTCAGCCCTCCTAATGACCTCCGGCCTAGCCATGTGATTTC
ACTTCCACTCCATAACGCTCCTCATACTAG 40 (0.001218%)

CCCAGGGATTGTGTTTAAAGTAGTGCTTCTACCAACATGTCCCGTGGTTCCAGCGCCGGTTTTGACCGCC
ACATTACCATTTTTTCACCCGAGGGTCGGC 4 (0.000122%)

CCCAGTCCTAGCTGCTGGCATCACTATACTACTAACAGACCGCAACCTCAACACCACCTTCTTCGACCCC
GCCGGAGGAGGAGACCCCATTCTATACCAA 15 (0.000457%)

CCCATAAAAATAAAAAATTATAACAAACCCTGAGAACCAAAATGAACGAAAATCTGTTCGCTTCATTCA
TTGCCCCCACAATCCTAGGCCTACCCGCCGC 7 (0.000213%)

CCCATATTGTAACTTACTACTCCGGAAAAAAAGAACCATTTGGATACATAGGTATGGTCTGAGCTATGAT
ATCAATTGGCTTCCTAGGGTTTATCGTGTG 28 (0.000853%)

CCCATCCCTACGCATCCTTTACATAACAGACGAGGTCAACGATCCCTCCCTTACCATCAAATCAATTGGC
CACCAATGGTACTGAACCTACGAGTACACC 18 (0.000548%)

CCCATGACCCCTAACAGGGGCCCTCTCAGCCCTCCTAATGACCTCCGGCCTAGCCATGTGATTTCACTTC
CACTCCATAACGCTCCTCATACTAGGCCTA 24 (0.000731%)

CCCATGTGTGTTGAGAGCTTCTCAGACTATCCACCTTTGGGTCGCTTTGCTGTTCGTGATATGAGACAGA
CAGTTGCGGTGGGTGTCATCAAAGCAGTGG 11 (0.000335%)

CCCCAACGTTGTAGGCCCCTACGGGCTACTACAACCCTTCGCTGACGCCATAAAACTCTTCACCAAAGAG
CCCCTAAAACCCGCCACATCTACCATCACC 21 (0.000640%)

CCCCAGGACACAGAGACTTTATCAAAAACATGATTACAGGGACATCTCAGGCTGACTGTGCTGTCCTGA
TTGTTGCTGCTGGTGTTGGTGAATTTGAAGC 7 (0.000213%)

CCCCATTCTATACCAACACCTATTCTGATTTTTCGGTCACCCTGAAGTTTATATTCTTATCCTACCAGGCT
TCGGAATAATCTCCCATATTGTAACTTAC 46 (0.001401%)

CCCCCCCCCCCCCCCCCCCCCCCCCCCCCCCCCCCCCCCCCCCCCCCCCCCCCCCCCCCCCCCCCCCCCCC
CCCCCCCCCCCCCCCCCCCCCCCCCCCCC 69 (0.002101%)

CCCCGATATGGCGTTTCCCCGCATAAACAACATAAGCTTCTGACTCTTACCTCCCTCTCTCCTACTCCTGC
TCGCATCTGCTATAGTGGAGGCCGGAGCA 6 (0.000183%)

CCCCTAACAGGGGCCCTCTCAGCCCTCCTAATGACCTCCGGCCTAGCCATGTGATTTCACTTCCACTCCAT
AACGCTCCTCATACTAGGCCTACTAACCA 11 (0.000335%)

CCCGAGAGCATGCCCTTCTGGCTTACACACTGGGTGTGAAACAACTAATTGTCGGTGTTAACAAAATGG
ATTCCACTGAGCCACCCTACAGCCAGAAGAG 16 (0.000487%)

CCCGATATATGTTCTCTAGGCCTTTTAGAAAACATGGAGTTGTTCCTTTGGCCACATATATGCGAATCTAT
AAGAAAGGTGATATTGTAGACATCAAGGG 7 (0.000213%)

CCCGATATGGCGTTTCCCCGCATAAACAACATAAGCTTCTGACTCTTACCTCCCTCTCTCCTACTCCTGCT
CGCATCTGCTATAGTGGAGGCCGGAGCAG 14 (0.000426%)

CCCGCCATCATCCTAGTCCTCATCGCCCTCCCATCCCTACGCATCCTTTACATAACAGACGAGGTCAACG
ATCCCTCCCTTACCATCAAATCAATTGGCC 3 (0.000091%)

CCCGGTATGGTGGTCACCTTTGCTCCAGTCAACGTTACAACGGAAGTAAAATCTGTCGAAATGCACCATG
AAGCTTTGAGTGAAGCTCTTCCTGGGGACA 22 (0.000670%)

CCCTAACAGGGGCCCTCTCAGCCCTCCTAATGACCTCCGGCCTAGCCATGTGATTTCACTTCCACTCCAT
AACGCTCCTCATACTAGGCCTACTAACCAA 47 (0.001431%)

CCCTAATAATCGGTGCCCCCGATATGGCGTTTCCCCGCATAAACAACATAAGCTTCTGACTCTTACCTCC
CTCTCTCCTACTCCTGCTCGCATCTGCTAT 7 (0.000213%)

CCCTACGCATCCTTTACATAACAGACGAGGTCAACGATCCCTCCCTTACCATCAAATCAATTGGCCACCA
ATGGTACTGAACCTACGAGTACACCGACTA 8 (0.000244%)

CCCTAGTAGGCTCCCTTCCCCTACTCATCGCACTGATTTACACTCACAACACCCTAGGCTCACTAAACATT
CTACTACTCACTCTCACTGCCCAAGAACT 6 (0.000183%)

CCCTCACCAAAGCCCATAAAAATAAAAAATTATAACAAACCCTGAGAACCAAAATGAACGAAAATCTGT
TCGCTTCATTCATTGCCCCCACAATCCTAGG 17 (0.000518%)

CCCTCCCATCCCTACGCATCCTTTACATAACAGACGAGGTCAACGATCCCTCCCTTACCATCAAATCAAT
TGGCCACCAATGGTACTGAACCTACGAGTA 5 (0.000152%)

CCCTCCTAATGACCTCCGGCCTAGCCATGTGATTTCACTTCCACTCCATAACGCTCCTCATACTAGGCCTA
CTAACCAACACACTAACCATATACCAATG 6 (0.000183%)

CCCTTCTGGCTTACACACTGGGTGTGAAACAACTAATTGTCGGTGTTAACAAAATGGATTCCACTGAGCC

ACCCTACAGCCAGAAGAGATATGAGGAAAT 8 (0.000244%)

CCGACTAATCACCACCCAACAATGACTAATCAAACTAACCTCAAAACAAATGATAACCATACACAACAC
TAAAGGACGAACCTGATCTCTTATACTAGTA 13 (0.000396%)

CCGAGAGCATGCCCTTCTGGCTTACACACTGGGTGTGAAACAACTAATTGTCGGTGTTAACAAAATGGAT
TCCACTGAGCCACCCTACAGCCAGAAGAGA 40 (0.001218%)

CCGATATATGTTCTCTAGGCCTTTTAGAAAACATGGAGTTGTTCCTTTGGCCACATATATGCGAATCTATA
AGAAAGGTGATATTGTAGACATCAAGGGA 24 (0.000731%)

CCGATGGTTACTTGCTTCGTCTGTTCTGTGTTGGTTTTACTAAAAAACGCAACAATCAGATACGGAAGAC
CTCTTATGCTCAGCACCAACAGGTCCGCCA 9 (0.000274%)

CCGCAAACTCTGTCTCAACATCTGTGTTGGGGAGAGTGGAGACAGACTGACGCGAGCAGCCAAGGTGTT
GGAGCAGCTCACAGGGCAGACCCCTGTGTTT 4 (0.000122%)

CCGCCATCATCCTAGTCCTCATCGCCCTCCCATCCCTACGCATCCTTTACATAACAGACGAGGTCAACGA
TCCCTCCCTTACCATCAAATCAATTGGCCA 23 (0.000700%)

CCGCGCGCACGTGAAGCACCGTAAAGGCGCTGCGCGCCTGCGCGCCGTGGATTTCGCTGAGCGGCACGG
CTACATCAAGGGCATCGTCAAGGACATCATC 5 (0.000152%)

CCGCTCCCAGACATGGGTCCCTCGGCTTCCTGCCTCGGAAGCGCAGCAGCAGGCATCGTGGGAAGGTGA
AGAGCTTCCCTAAGGATGACCCGTCCAAGCC 5 (0.000152%)

CCGGGTCTGTGTTCCGCGCGCACGTGAAGCACCGTAAAGGCGCTGCGCGCCTGCGCGCCGTGGATTTCG
CTGAGCGGCACGGCTACATCAAGGGCATCGT 12 (0.000365%)

CCGGTATGGTGGTCACCTTTGCTCCAGTCAACGTTACAACGGAAGTAAAATCTGTCGAAATGCACCATGA
AGCTTTGAGTGAAGCTCTTCCTGGGGACAA 33 (0.001005%)

CCGTAAAGGCGCTGCGCGCCTGCGCGCCGTGGATTTCGCTGAGCGGCACGGCTACATCAAGGGCATCGT
CAAGGACATCATCCACGACCCGGGCCGCGGC 6 (0.000183%)

CCGTCTGAACTATCCTGCCCGCCATCATCCTAGTCCTCATCGCCCTCCCATCCCTACGCATCCTTTACATA
ACAGACGAGGTCAACGATCCCTCCCTTAC 5 (0.000152%)

CCGTGGACAGAGGAAGGGCGCCGGGTCTGTGTTCCGCGCGCACGTGAAGCACCGTAAAGGCGCTGCGCG
CCTGCGCGCCGTGGATTTCGCTGAGCGGCAC 7 (0.000213%)

CCGTTCTGGTAAAAAGCTGGAAGATGGCCCTAAATTCTTGAAGTCTGGTGATGCTGCCATTGTTGATATG
GTTCCTGGCAAGCCCATGTGTGTTGAGAGC 1154 (0.035142%)

CCTAAATTCTTGAAGTCTGGTGATGCTGCCATTGTTGATATGGTTCCTGGCAAGCCCATGTGTGTTGAGA
GCTTCTCAGACTATCCACCTTTGGGTCGCT 28 (0.000853%)

CCTAACAGGGGCCCTCTCAGCCCTCCTAATGACCTCCGGCCTAGCCATGTGATTTCACTTCCACTCCATA
ACGCTCCTCATACTAGGCCTACTAACCAAC 11 (0.000335%)

CCTAATAATCGGTGCCCCCGATATGGCGTTTCCCCGCATAAACAACATAAGCTTCTGACTCTTACCTCCC
TCTCTCCTACTCCTGCTCGCATCTGCTATA 9 (0.000274%)

CCTAATGACCTCCGGCCTAGCCATGTGATTTCACTTCCACTCCATAACGCTCCTCATACTAGGCCTACTAA
CCAACACACTAACCATATACCAATGATGG 4 (0.000122%)

CCTACATACTTCCCCCATTATTCCTAGAACCAGGCGACCTGCGACTCCTTGACGTTGACAATCGAGTAGT
ACTCCCGATTGAAGCCCCCATTCGTATAAT 465 (0.014160%)

CCTACGCATCCTTTACATAACAGACGAGGTCAACGATCCCTCCCTTACCATCAAATCAATTGGCCACCAA
TGGTACTGAACCTACGAGTACACCGACTAC 18 (0.000548%)

CCTAGAACCAGGCGACCTGCGACTCCTTGACGTTGACAATCGAGTAGTACTCCCGATTGAAGCCCCCATT
CGTATAATAATTACATCACAAGACGTCTTG 39 (0.001188%)

CCTAGCTGCTGGCATCACTATACTACTAACAGACCGCAACCTCAACACCACCTTCTTCGACCCCGCCGGA
GGAGGAGACCCCATTCTATACCAACACCTA 17 (0.000518%)

CCTAGTCCTCATCGCCCTCCCATCCCTACGCATCCTTTACATAACAGACGAGGTCAACGATCCCTCCCTTA
CCATCAAATCAATTGGCCACCAATGGTAC 9 (0.000274%)

CCTATCTCTCCCAGTCCTAGCTGCTGGCATCACTATACTACTAACAGACCGCAACCTCAACACCACCTTC
TTCGACCCCGCCGGAGGAGGAGACCCCATT 15 (0.000457%)

CCTATTTATTACCTCAGAAGTTTTTTCTTCGCAGGATTTTTCTGAGCCTTTTACCACTCCAGCCTAGCCCC
TACCCCCCAATTAGGAGGGCACTGGCCC 11 (0.000335%)

CCTCACCAAAGCCCATAAAAATAAAAAATTATAACAAACCCTGAGAACCAAAATGAACGAAAATCTGTT
CGCTTCATTCATTGCCCCCACAATCCTAGGC 41 (0.001249%)

CCTCACTCATTTACACCAACCACCCAACTATCTATAAACCTAGCCATGGCCATCCCCTTATGAGCGGGCG
CAGTGATTATAGGCTTTCGCTCTAAGATTA 11 (0.000335%)

CCTCAGAAGTTTTTTCTTCGCAGGATTTTTCTGAGCCTTTTACCACTCCAGCCTAGCCCCTACCCCCCAA
TTAGGAGGGCACTGGCCCCCAACAGGCAT 9 (0.000274%)

CCTCATCGCCCTCCCATCCCTACGCATCCTTTACATAACAGACGAGGTCAACGATCCCTCCCTTACCATC
AAATCAATTGGCCACCAATGGTACTGAACC 19 (0.000579%)

CCTCCCATCCCTACGCATCCTTTACATAACAGACGAGGTCAACGATCCCTCCCTTACCATCAAATCAATT
GGCCACCAATGGTACTGAACCTACGAGTAC 4 (0.000122%)

CCTCCTAATGACCTCCGGCCTAGCCATGTGATTTCACTTCCACTCCATAACGCTCCTCATACTAGGCCTAC
TAACCAACACACTAACCATATACCAATGA 4 (0.000122%)

CCTCGCTGAGGAAAAATAACTAAACATGGGCAAAGGAGATCCTAAGAAGCCGAGAGGCAAAATGTCAT
CATATGCATTTTTTGTGCAAACTTGTCGGGAG 4 (0.000122%)

CCTCGGACTCCTGCCTCACTCATTTACACCAACCACCCAACTATCTATAAACCTAGCCATGGCCATCCCC
TTATGAGCGGGCGCAGTGATTATAGGCTTT 24 (0.000731%)

CCTCGGCTTCCTGCCTCGGAAGCGCAGCAGCAGGCATCGTGGGAAGGTGAAGAGCTTCCCTAAGGATGA
CCCGTCCAAGCCGGTCCACCTCACAGCCTTC 5 (0.000152%)

CCTCTCAGCCCTCCTAATGACCTCCGGCCTAGCCATGTGATTTCACTTCCACTCCATAACGCTCCTCATAC
TAGGCCTACTAACCAACACACTAACCATA 5 (0.000152%)

CCTCTCGCAAAGGATCTCCTTCATCCCTCTCCAGAAGAGGAGAAGAGGAAACACAAGAAGAAACGCCTG
GTGCAGAGCCCCAATTCCTACTTCATGGATG 6 (0.000183%)

CCTGAACGCAGGCACATACTTCCTATTCTACACCCTAGTAGGCTCCCTTCCCCTACTCATCGCACTGATTT
ACACTCACAACACCCTAGGCTCACTAAAC 29 (0.000883%)

CCTGATTGTTGCTGCTGGTGTTGGTGAATTTGAAGCTGGTATCTCCAAGAATGGGCAGACCCGAGAGCAT
GCCCTTCTGGCTTACACACTGGGTGTGAAA 34 (0.001035%)

CCTGCCCGCCATCATCCTAGTCCTCATCGCCCTCCCATCCCTACGCATCCTTTACATAACAGACGAGGTC
AACGATCCCTCCCTTACCATCAAATCAATT 5 (0.000152%)

CCTGCCTCACTCATTTACACCAACCACCCAACTATCTATAAACCTAGCCATGGCCATCCCCTTATGAGCG
GGCGCAGTGATTATAGGCTTTCGCTCTAAG 16 (0.000487%)

CCTGGCAAGCCCATGTGTGTTGAGAGCTTCTCAGACTATCCACCTTTGGGTCGCTTTGCTGTTCGTGATAT
GAGACAGACAGTTGCGGTGGGTGTCATCA25 (0.000761%)

CCTGGGTCTTGGATAAACTGAAAGCTGAGCGTGAACGTGGTATCACCATTGATATCTCCTTGTGGAAATT
TGAGACCAGCAAGTACTATGTGACTATCAT 31 (0.000944%)

CCTTAATCATTTTTATTGCCACAACTAACCTCCTCGGACTCCTGCCTCACTCATTTACACCAACCACCCAA
CTATCTATAAACCTAGCCATGGCCATCCC 376 (0.011450%)

CCTTATTCGAGCCGAGCTGGGCCAGCCAGGCAACCTTCTAGGTAACGACCACATCTACAACGTTATCGTC
ACAGCCCATGCATTTGTAATAATCTTCTTC19 (0.000579%)

CCTTCAAGTATGCCTGGGTCTTGGATAAACTGAAAGCTGAGCGTGAACGTGGTATCACCATTGATATCTC
CTTGTGGAAATTTGAGACCAGCAAGTACTA 7 (0.000213%)

CCTTCATCCCTCTCCAGAAGAGGAGAAGAGGAAACACAAGAAGAAACGCCTGGTGCAGAGCCCCAATTC
CTACTTCATGGATGTGAAATGCCCAGGATGC 7 (0.000213%)

CCTTCGATACGGGATAATCCTATTTATTACCTCAGAAGTTTTTTTCTTCGCAGGATTTTTCTGAGCCTTTTA
CCACTCCAGCCTAGCCCCTACCCCCCAA 3 (0.000091%)

CCTTGTGGAAATTTGAGACCAGCAAGTACTATGTGACTATCATTGATGCCCCAGGACACAGAGACTTTAT
CAAAAACATGATTACAGGGACATCTCAGGC 6 (0.000183%)

CCTTTACATAACAGACGAGGTCAACGATCCCTCCCTTACCATCAAATCAATTGGCCACCAATGGTACTGA
ACCTACGAGTACACCGACTACGGCGGACTA 10 (0.000305%)

CGAAAATCTGTTCGCTTCATTCATTGCCCCCACAATCCTAGGCCTACCCGCCGCAGTACTGATCATTCTAT
TTCCCCCTCTATTGATCCCCACCTCCAAA 718 (0.021865%)

CGAACCTGATCTCTTATACTAGTATCCTTAATCATTTTTATTGCCACAACTAACCTCCTCGGACTCCTGCC
TCACTCATTTACACCAACCACCCAACTAT 722 (0.021987%)

CGAATGCGCAGGCTGAAGCGCAAAAGAAGAAAGATGAGGCAGAGGTCCAAGTAAACCGCTAGCTTGTT
GCACCGTGGAGGCCACAGGAGCAGAAACATGG 63 (0.001919%)

CGACAAAAGAACCATTGAAAAATTTGAGAAGGAGGCTGCTGAGATGGGAAAGGGCTCCTTCAAGTATG
CCTGGGTCTTGGATAAACTGAAAGCTGAGCGT 55 (0.001675%)

CGACTAATCACCACCCAACAATGACTAATCAAACTAACCTCAAAACAAATGATAACCATACACAACACT
AAAGGACGAACCTGATCTCTTATACTAGTAT 47 (0.001431%)

CGACTCTGTGCCTCGCTGAGGAAAAATAACTAAACATGGGCAAAGGAGATCCTAAGAAGCCGAGAGGC
AAAATGTCATCATATGCATTTTTTGTGCAAAC 6 (0.000183%)

CGAGAGCATGCCCTTCTGGCTTACACACTGGGTGTGAAACAACTAATTGTCGGTGTTAACAAAATGGATT
CCACTGAGCCACCCTACAGCCAGAAGAGAT 81 (0.002467%)

CGAGGCCATCTCCTGGGCCGCCTGGCGGCCATCGTGGCTAAACAGGTACTGCTGGGCCGGAAGGTGGTG
GTCGTACGCTGTGAAGGCATCAACATTTCTG 9 (0.000274%)

CGAGGTCAACGATCCCTCCCTTACCATCAAATCAATTGGCCACCAATGGTACTGAACCTACGAGTACACC
GACTACGGCGGACTAATCTTCAACTCCTAC 85 (0.002588%)

CGAGTGGAGACTGGTGTTCTCAAACCCGGTATGGTGGTCACCTTTGCTCCAGTCAACGTTACAACGGAAG

TAAAATCTGTCGAAATGCACCATGAAGCTT 23 (0.000700%)

CGATATATGTTCTCTAGGCCTTTTAGAAAACATGGAGTTGTTCCTTTGGCCACATATATGCGAATCTATA
AGAAAGGTGATATTGTAGACATCAAGGGAA 4 (0.000122%)

CGATATGGCGTTTCCCCGCATAAACAACATAAGCTTCTGACTCTTACCTCCCTCTCTCCTACTCCTGCTCG
CATCTGCTATAGTGGAGGCCGGAGCAGGA 43 (0.001309%)

CGATGGTTACTTGCTTCGTCTGTTCTGTGTTGGTTTACTAAAAAACGCAACAATCAGATACGGAAGACC
TCTTATGCTCAGCACCAACAGGTCCGCCAA 5 (0.000152%)

CGCAAAAGAAGAAAGATGAGGCAGAGGTCCAAGTAAACCGCTAGCTTGTTGCACCGTGGAGGCCACAG
GAGCAGAAACATGGAATGCCAGACGCTGGGGA 16 (0.000487%)

CGCAAACTCTGTCTCAACATCTGTGTTGGGGAGAGTGGAGACAGACTGACGCGAGCAGCCAAGGTGTTG
GAGCAGCTCACAGGGCAGACCCCTGTGTTTT 4 (0.000122%)

CGCAAAGGATCTCCTTCATCCCTCTCCAGAAGAGGAGAAGAGGAAACACAAGAAGAAACGCCTGGTGC
AGAGCCCCAATTCCTACTTCATGGATGTGAAA 8 (0.000244%)

CGCACGTGAAGCACCGTAAAGGCGCTGCGCGCCTGCGCGCCGTGGATTTCGCTGAGCGGCACGGCTACA
TCAAGGGCATCGTCAAGGACATCATCCACGA 6 (0.000183%)

CGCAGGCACATACTTCCTATTCTACACCCTAGTAGGCTCCCTTCCCCTACTCATCGCACTGATTTACACTC
ACAACACCCTAGGCTCACTAAACATTCTA 15 (0.000457%)

CGCAGGCTGAAGCGCAAAAGAAGAAAGATGAGGCAGAGGTCCAAGTAAACCGCTAGCTTGTTGCACCG
TGGAGGCCACAGGAGCAGAAACATGGAATGCC 66 (0.002010%)

CGCATCCTTTACATAACAGACGAGGTCAACGATCCCTCCCTTACCATCAAATCAATTGGCCACCAATGGT
ACTGAACCTACGAGTACACCGACTACGGCG 42 (0.001279%)

CGCCATCATCCTAGTCCTCATCGCCCTCCCATCCCTACGCATCCTTTACATAACAGACGAGGTCAACGAT
CCCTCCCTTACCATCAAATCAATTGGCCAC 31 (0.000944%)

CGCCCTCCCATCCCTACGCATCCTTTACATAACAGACGAGGTCAACGATCCCTCCCTTACCATCAAATCA
ATTGGCCACCAATGGTACTGAACCTACGAG 7 (0.000213%)

CGCCGGGTCTGTGTTCCGCGCGCACGTGAAGCACCGTAAAGGCGCTGCGCGCCTGCGCGCCGTGGATTT
CGCTGAGCGGCACGGCTACATCAAGGGCATC 18 (0.000548%)

CGCCTGAACGCAGGCACATACTTCCTATTCTACACCCTAGTAGGCTCCCTTCCCCTACTCATCGCACTGAT
TTACACTCACAACACCCTAGGCTCACTAA 11 (0.000335%)

CGCGCGCACGTGAAGCACCGTAAAGGCGCTGCGCGCCTGCGCGCCGTGGATTTCGCTGAGCGGCACGGC
TACATCAAGGGCATCGTCAAGGACATCATCC 9 (0.000274%)

CGCTCCCAGACATGGGTCCCTCGGCTTCCTGCCTCGGAAGCGCAGCAGCAGGCATCGTGGGAAGGTGAA
GAGCTTCCCTAAGGATGACCCGTCCAAGCCG 9 (0.000274%)

CGCTGAGGAAAAATAACTAAACATGGGCAAAGGAGATCCTAAGAAGCCGAGAGGCAAAATGTCATCAT
ATGCATTTTTTGTGCAAACTTGTCGGGAGGAG 10 (0.000305%)

CGCTTCATTCATTGCCCCCACAATCCTAGGCCTACCCGCCGCAGTACTGATCATTCTATTTCCCCCTCTAT
TGATCCCCACCTCCAAATATCTCATCAAC 8 (0.000244%)

CGGAATAATCTCCCATATTGTAACTTACTACTCCGGAAAAAAAGAACCATTTGGATACATAGGTATGGTC
TGAGCTATGATATCAATTGGCTTCCTAGGG 6 (0.000183%)

CGGACGGGCACTGGGCGACTCTGTGCCTCGCTGAGGAAAAATAACTAAACATGGGCAAAGGAGATCCTA
AGAAGCCGAGAGGCAAAATGTCATCATATGC 66 (0.002010%)

CGGACTCCTGCCTCACTCATTTACACCAACCACCCAACTATCTATAAACCTAGCCATGGCCATCCCCTTA
TGAGCGGGCGCAGTGATTATAGGCTTTCGC 26 (0.000792%)

CGGCTGCTTCCAGCTCCTCCCTGGAGAAGAGCTACGAGCTGCCTGACGGCCAGGTCATCACCATTGGCA
ATGAGCGGTTCCGCTGCCCTGAGGCACTCTT 221 (0.006730%)

CGGGATAATCCTATTTATTACCTCAGAAGTTTTTTTCTTCGCAGGATTTTTCTGAGCCTTTTACCACTCCA
GCCTAGCCCCTACCCCCCAATTAGGAGGG 20 (0.000609%)

CGGGCAAGTCCACCACTACTGGCCATCTGATCTATAAATGCGGTGGCATCGACAAAAGAACCATTGAAA
AATTTGAGAAGGAGGCTGCTGAGATGGGAAA 34 (0.001035%)

CGGGCACTGGGCGACTCTGTGCCTCGCTGAGGAAAAATAACTAAACATGGGCAAAGGAGATCCTAAGA
AGCCGAGAGGCAAAATGTCATCATATGCATTT 26 (0.000792%)

CGGGTCTGTGTTCCGCGCGCACGTGAAGCACCGTAAAGGCGCTGCGCGCCTGCGCGCCGTGGATTTCGCT
GAGCGGCACGGCTACATCAAGGGCATCGTC 13 (0.000396%)

CGGTATGGTGGTCACCTTTGCTCCAGTCAACGTTACAACGGAAGTAAAATCTGTCGAAATGCACCATGA
AGCTTTGAGTGAAGCTCTTCCTGGGGACAAT 7 (0.000213%)

CGGTGCCCCCGATATGGCGTTTCCCCGCATAAACAACATAAGCTTCTGACTCTTACCTCCCTCTCTCCTAC
TCCTGCTCGCATCTGCTATAGTGGAGGCC 11 (0.000335%)

CGTAAAGGCGCTGCGCGCCTGCGCGCCGTGGATTTCGCTGAGCGGCACGGCTACATCAAGGGCATCGTC
AAGGACATCATCCACGACCCGGGCCGCGGCG 7 (0.000213%)

CGTCATTGGACACGTAGATTCGGGCAAGTCCACCACTACTGGCCATCTGATCTATAAATGCGGTGGCATC
GACAAAAGAACCATTGAAAAATTTGAGAAG 16 (0.000487%)

CGTCTGAACTATCCTGCCCGCCATCATCCTAGTCCTCATCGCCCTCCCATCCCTACGCATCCTTTACATAA
CAGACGAGGTCAACGATCCCTCCCTTACC 4 (0.000122%)

CGTGAAAACTACCCCTAAAAGCCAAAATGGGAAAGGAAAAGACTCATATCAACATTGTCGTCATTGGAC
ACGTAGATTCGGGCAAGTCCACCACTACTGG 12 (0.000365%)

CGTGAACGTGGTATCACCATTGATATCTCCTTGTGGAAATTTGAGACCAGCAAGTACTATGTGACTATCA
TTGATGCCCCAGGACACAGAGACTTTATCA 18 (0.000548%)

CGTGATATGAGACAGACAGTTGCGGTGGGTGTCATCAAAGCAGTGGACAAGAAGGCTGCTGGAGCTGGC
AAGGTCACCAAGTCTGCCCAGAAAGCTCAGA 113 (0.003441%)

CGTGGACAGAGGAAGGGCGCCGGGTCTGTGTTCCGCGCGCACGTGAAGCACCGTAAAGGCGCTGCGCGC
CTGCGCGCCGTGGATTTCGCTGAGCGGCACG 33 (0.001005%)

CGTGGTATCACCATTGATATCTCCTTGTGGAAATTTGAGACCAGCAAGTACTATGTGACTATCATTGATG
CCCCAGGACACAGAGACTTTATCAAAAACA 29 (0.000883%)

CGTTCTGGTAAAAAGCTGGAAGATGGCCCTAAATTCTTGAAGTCTGGTGATGCTGCCATTGTTGATATGG
TTCCTGGCAAGCCCATGTGTGTTGAGAGCT 14 (0.000426%)

CGTTGATGTCAAGACTACCGATGGTTACTTGCTTCGTCTGTTCTGTGTTGGTTTTACTAAAAAACGCAACA
ATCAGATACGGAAGACCTCTTATGCTCAG 268 (0.008161%)

CGTTTCCCCGCATAAACAACATAAGCTTCTGACTCTTACCTCCCTCTCTCCTACTCCTGCTCGCATCTGCT
ATAGTGGAGGCCGGAGCAGGAACAGGTTG 4 (0.000122%)

CTAAATTCTTGAAGTCTGGTGATGCTGCCATTGTTGATATGGTTCCTGGCAAGCCCATGTGTGTTGAGAG
CTTCTCAGACTATCCACCTTTGGGTCGCTT 70 (0.002132%)

CTAACAGGGGCCCTCTCAGCCCTCCTAATGACCTCCGGCCTAGCCATGTGATTTCACTTCCACTCCATAA
CGCTCCTCATACTAGGCCTACTAACCAACA 36 (0.001096%)

CTAAGCCTCCTTATTCGAGCCGAGCTGGGCCAGCCAGGCAACCTTCTAGGTAACGACCACATCTACAAC
GTTATCGTCACAGCCCATGCATTTGTAATAA 5 (0.000152%)

CTAATAATCGGTGCCCCCGATATGGCGTTTCCCCGCATAAACAACATAAGCTTCTGACTCTTACCTCCCT
CTCTCCTACTCCTGCTCGCATCTGCTATAG 3 (0.000091%)

CTACAATGATTTTGGGAATTACAACAATCAGTCTTCAAATTTTGGACCCATGAAGGGAGGAAATTTTGGA
GGCAGAAGCTCTGGCCCCTATGGCGGTGGA 16 (0.000487%)

CTACACCCTAGTAGGCTCCCTTCCCCTACTCATCGCACTGATTTACACTCACAACACCCTAGGCTCACTA
AACATTCTACTACTCACTCTCACTGCCCAA 10 (0.000305%)

CTACATACTTCCCCCATTATTCCTAGAACCAGGCGACCTGCGACTCCTTGACGTTGACAATCGAGTAGTA
CTCCCGATTGAAGCCCCCATTCGTATAATA 5 (0.000152%)

CTACGCATCCTTTACATAACAGACGAGGTCAACGATCCCTCCCTTACCATCAAATCAATTGGCCACCAAT
GGTACTGAACCTACGAGTACACCGACTACG 5 (0.000152%)

CTAGAACCAGGCGACCTGCGACTCCTTGACGTTGACAATCGAGTAGTACTCCCGATTGAAGCCCCCATTC
GTATAATAATTACATCACAAGACGTCTTGC 25 (0.000761%)

CTAGGAATCACCTCCCATTCCGATAAAATCACCTTCCACCCTTACTACACAATCAAAGACGCCCTCGGCT
TACTTCTCTTCATTCTCTCCTTAATGACAT 210 (0.006395%)

CTAGTAGGCTCCCTTCCCCTACTCATCGCACTGATTTACACTCACAACACCCTAGGCTCACTAAACATTCT
ACTACTCACTCTCACTGCCCAAGAACTAT 3 (0.000091%)

CTAGTCCTCATCGCCCTCCCATCCCTACGCATCCTTTACATAACAGACGAGGTCAACGATCCCTCCCTTAC
CATCAAATCAATTGGCCACCAATGGTACT 23 (0.000700%)

CTAGTTCCCCTAATAATCGGTGCCCCCGATATGGCGTTTCCCCGCATAAACAACATAAGCTTCTGACTCT
TACCTCCCTCTCTCCTACTCCTGCTCGCAT 6 (0.000183%)

CTATAAACCTAGCCATGGCCATCCCCTTATGAGCGGGCGCAGTGATTATAGGCTTTCGCTCTAAGATTAA
AAATGCCCTAGCCCACTTCTTACCACAAGG 20 (0.000609%)

CTATAAATGCGGTGGCATCGACAAAAGAACCATTGAAAAATTTGAGAAGGAGGCTGCTGAGATGGGAA
AGGGCTCCTTCAAGTATGCCTGGGTCTTGGAT 15 (0.000457%)

CTATCACCCTATAGAAGAACTAATGTTAGTATAAGTAACATGAAAACATTCTCCTCCGCATAAGCCTGCG
TCAGATTAAAACACTGAACTGACAATTAAC 14 (0.000426%)

CTATCATTGATGCCCCAGGACACAGAGACTTTATCAAAAACATGATTACAGGGACATCTCAGGCTGACT
GTGCTGTCCTGATTGTTGCTGCTGGTGTTGG 40 (0.001218%)

CTATCCACCTTTGGGTCGCTTTGCTGTTCGTGATATGAGACAGACAGTTGCGGTGGGTGTCATCAAAGCA
GTGGACAAGAAGGCTGCTGGAGCTGGCAAG 3 (0.000091%)

CTATCCTGCCCGCCATCATCCTAGTCCTCATCGCCCTCCCATCCCTACGCATCCTTTACATAACAGACGAG
GTCAACGATCCCTCCCTTACCATCAAATC 11 (0.000335%)

CTATCTATAAACCTAGCCATGGCCATCCCCTTATGAGCGGGCGCAGTGATTATAGGCTTTCGCTCTAAGA

TTAAAAATGCCCTAGCCCACTTCTTACCAC 16 (0.000487%)

CTATGTGACTATCATTGATGCCCCAGGACACAGAGACTTTATCAAAAACATGATTACAGGGACATCTCA
GGCTGACTGTGCTGTCCTGATTGTTGCTGCT 4 (0.000122%)

CTATTCTACACCCTAGTAGGCTCCCTTCCCCTACTCATCGCACTGATTTACACTCACAACACCCTAGGCTC
ACTAAACATTCTACTACTCACTCTCACTG 14 (0.000426%)

CTATTTATTACCTCAGAAGTTTTTTTCTTCGCAGGATTTTTCTGAGCCTTTTACCACTCCAGCCTAGCCCCT
ACCCCCCAATTAGGAGGGCACTGGCCCC 9 (0.000274%)

CTCAAACCCGGTATGGTGGTCACCTTTGCTCCAGTCAACGTTACAACGGAAGTAAAATCTGTCGAAATGC
ACCATGAAGCTTTGAGTGAAGCTCTTCCTG 111 (0.003380%)

CTCACACCCAATTGGACCAATCTATCACCCTATAGAAGAACTAATGTTAGTATAAGTAACATGAAAACA
TTCTCCTCCGCATAAGCCTGCGTCAGATTAA 30 (0.000914%)

CTCACCAAAGCCCATAAAAATAAAAAATTATAACAAACCCTGAGAACCAAAATGAACGAAAATCTGTTC
GCTTCATTCATTGCCCCCACAATCCTAGGCC 27 (0.000822%)

CTCACTCATTTACACCAACCACCCAACTATCTATAAACCTAGCCATGGCCATCCCCTTATGAGCGGGCGC
AGTGATTATAGGCTTTCGCTCTAAGATTAA 14 (0.000426%)

CTCAGAAGTTTTTTTCTTCGCAGGATTTTTCTGAGCCTTTTACCACTCCAGCCTAGCCCCTACCCCCCAAT
TAGGAGGGCACTGGCCCCCAACAGGCATC 22 (0.000670%)

CTCAGACTATCCACCTTTGGGTCGCTTTGCTGTTCGTGATATGAGACAGACAGTTGCGGTGGGTGTCATC
AAAGCAGTGGACAAGAAGGCTGCTGGAGCT 88 (0.002680%)

CTCAGCCCTCCTAATGACCTCCGGCCTAGCCATGTGATTTCACTTCCACTCCATAACGCTCCTCATACTAG
GCCTACTAACCAACACACTAACCATATAC 16 (0.000487%)

CTCAGGCTGACTGTGCTGTCCTGATTGTTGCTGCTGGTGTTGGTGAATTTGAAGCTGGTATCTCCAAGAA
TGGGCAGACCCGAGAGCATGCCCTTCTGGC 44 (0.001340%)

CTCATCGCCCTCCCATCCCTACGCATCCTTTACATAACAGACGAGGTCAACGATCCCTCCCTTACCATCA
AATCAATTGGCCACCAATGGTACTGAACCT 12 (0.000365%)

CTCATTTACACCAACCACCCAACTATCTATAAACCTAGCCATGGCCATCCCCTTATGAGCGGGCGCAGTG
ATTATAGGCTTTCGCTCTAAGATTAAAAAT 33 (0.001005%)

CTCCCATATTGTAACTTACTACTCCGGAAAAAAAGAACCATTTGGATACATAGGTATGGTCTGAGCTATG
ATATCAATTGGCTTCCTAGGGTTTATCGTG 62 (0.001888%)

CTCCCATCCCTACGCATCCTTTACATAACAGACGAGGTCAACGATCCCTCCCTTACCATCAAATCAATTG
GCCACCAATGGTACTGAACCTACGAGTACA 4 (0.000122%)

CTCCGCTCCCAGACATGGGTCCCTCGGCTTCCTGCCTCGGAAGCGCAGCAGCAGGCATCGTGGGAAGGT
GAAGAGCTTCCCTAAGGATGACCCGTCCAAG 11 (0.000335%)

CTCCTAATGACCTCCGGCCTAGCCATGTGATTTCACTTCCACTCCATAACGCTCCTCATACTAGGCCTACT
AACCAACACACTAACCATATACCAATGAT 16 (0.000487%)

CTCCTATCTCTCCCAGTCCTAGCTGCTGGCATCACTATACTACTAACAGACCGCAACCTCAACACCACCT
TCTTCGACCCCGCCGGAGGAGGAGACCCCA 17 (0.000518%)

CTCCTCACACCCAATTGGACCAATCTATCACCCTATAGAAGAACTAATGTTAGTATAAGTAACATGAAAA
CATTCTCCTCCGCATAAGCCTGCGTCAGAT 22 (0.000670%)

CTCCTGCCTCACTCATTTACACCAACCACCCAACTATCTATAAACCTAGCCATGGCCATCCCCTTATGAG
CGGGCGCAGTGATTATAGGCTTTCGCTCTA 6 (0.000183%)

CTCCTGGGCCGCCTGGCGGCCATCGTGGCTAAACAGGTACTGCTGGGCCGGAAGGTGGTGGTCGTACGC
TGTGAAGGCATCAACATTTCTGGCAATTTCT 6 (0.000183%)

CTCCTTCAAGTATGCCTGGGTCTTGGATAAACTGAAAGCTGAGCGTGAACGTGGTATCACCATTGATATC
TCCTTGTGGAAATTTGAGACCAGCAAGTAC 37 (0.001127%)

CTCCTTCATCCCTCTCCAGAAGAGGAGAAGAGGAAACACAAGAAGAAACGCCTGGTGCAGAGCCCCAAT
TCCTACTTCATGGATGTGAAATGCCCAGGAT 13 (0.000396%)

CTCCTTGTGGAAATTTGAGACCAGCAAGTACTATGTGACTATCATTGATGCCCCAGGACACAGAGACTTT
ATCAAAAACATGATTACAGGGACATCTCAG 6 (0.000183%)

CTCGCAAAGGATCTCCTTCATCCCTCTCCAGAAGAGGAGAAGAGGAAACACAAGAAGAAACGCCTGGTG
CAGAGCCCCAATTCCTACTTCATGGATGTGA 8 (0.000244%)

CTCGGACTCCTGCCTCACTCATTTACACCAACCACCCAACTATCTATAAACCTAGCCATGGCCATCCCCTT
ATGAGCGGGCGCAGTGATTATAGGCTTTC 46 (0.001401%)

CTCGGCTTCCTGCCTCGGAAGCGCAGCAGCAGGCATCGTGGGAAGGTGAAGAGCTTCCCTAAGGATGAC
CCGTCCAAGCCGGTCCACCTCACAGCCTTCC 13 (0.000396%)

CTCTAAGCCTCCTTATTCGAGCCGAGCTGGGCCAGCCAGGCAACCTTCTAGGTAACGACCACATCTACAA
CGTTATCGTCACAGCCCATGCATTTGTAAT 19 (0.000579%)

CTCTAGGCCTTTTAGAAAACATGGAGTTGTTCCTTTGGCCACATATATGCGAATCTATAAGAAAGGTGAT
ATTGTAGACATCAAGGGAATGGGTACTGTT 13 (0.000396%)

CTCTCAGCCCTCCTAATGACCTCCGGCCTAGCCATGTGATTTCACTTCCACTCCATAACGCTCCTCATACT
AGGCCTACTAACCAACACACTAACCATAT 12 (0.000365%)

CTCTCCCAGTCCTAGCTGCTGGCATCACTATACTACTAACAGACCGCAACCTCAACACCACCTTCTTCGA
CCCCGCCGGAGGAGGAGACCCCATTCTATA 8 (0.000244%)

CTCTCGCAAAGGATCTCCTTCATCCCTCTCCAGAAGAGGAGAAGAGGAAACACAAGAAGAAACGCCTGG
TGCAGAGCCCCAATTCCTACTTCATGGATGT 15 (0.000457%)

CTCTGTGCCTCGCTGAGGAAAAATAACTAAACATGGGCAAAGGAGATCCTAAGAAGCCGAGAGGCAAA
ATGTCATCATATGCATTTTTTGTGCAAACTTG 12 (0.000365%)

CTGAAAGCTGAGCGTGAACGTGGTATCACCATTGATATCTCCTTGTGGAAATTTGAGACCAGCAAGTACT
ATGTGACTATCATTGATGCCCCAGGACACA 109 (0.003319%)

CTGAACGCAGGCACATACTTCCTATTCTACACCCTAGTAGGCTCCCTTCCCCTACTCATCGCACTGATTTA
CACTCACAACACCCTAGGCTCACTAAACA 12 (0.000365%)

CTGAACTATCCTGCCCGCCATCATCCTAGTCCTCATCGCCCTCCCATCCCTACGCATCCTTTACATAACAG
ACGAGGTCAACGATCCCTCCCTTACCATC 49 (0.001492%)

CTGAACTCCTCACACCCAATTGGACCAATCTATCACCCTATAGAAGAACTAATGTTAGTATAAGTAACAT
GAAAACATTCTCCTCCGCATAAGCCTGCGT 57 (0.001736%)

CTGACTAGTTCCCCTAATAATCGGTGCCCCCGATATGGCGTTTCCCCGCATAAACAACATAAGCTTCTGA
CTCTTACCTCCCTCTCTCCTACTCCTGCTC 11 (0.000335%)

CTGAGCGTGAACGTGGTATCACCATTGATATCTCCTTGTGGAAATTTGAGACCAGCAAGTACTATGTGAC
TATCATTGATGCCCCAGGACACAGAGACTT 23 (0.000700%)

CTGAGCTCACCATAGTCTAATAGAAAACAACCGAAACCAAATAATTCAAGCACTGCTTATTACAATTTTA
CTGGGTCTCTATTTACCCTCCTACAAGCC481 (0.014648%)

CTGCCATTGTTGATATGGTTCCTGGCAAGCCCATGTGTGTTGAGAGCTTCTCAGACTATCCACCTTTGGGT
CGCTTTGCTGTTCGTGATATGAGACAGAC 27 (0.000822%)

CTGCCCGCCATCATCCTAGTCCTCATCGCCCTCCCATCCCTACGCATCCTTTACATAACAGACGAGGTCA
ACGATCCCTCCCTTACCATCAAATCAATTG 19 (0.000579%)

CTGCCTCACTCATTTACACCAACCACCCAACTATCTATAAACCTAGCCATGGCCATCCCCTTATGAGCGG
GCGCAGTGATTATAGGCTTTCGCTCTAAGA 6 (0.000183%)

CTGCGACTTGTGTTGGGACTGCTGATAGGAAGATGTCTTCAGGAAATGCTAAAATTGGGCACCCTGCCCC
CAACTTCAAAGCCACAGCTGTTATGCCAGA 420 (0.012790%)

CTGCTGGAGCTGGCAAGGTCACCAAGTCTGCCCAGAAAGCTCAGAAGGCTAAATGAATATTATCCCTAA
TACCTGCCACCCCACTCTTAATCAGTGGTGG 8 (0.000244%)

CTGGAAAGCGGTCTGCCCCTGGAGGTGGTAGCAAGGTTCCACAGAAAAAGTAAAACTTGCTGCTGATG
AAGATGATGACGATGATGATGAAGAGGATGA 3 (0.000091%)

CTGGAAGATGGCCCTAAATTCTTGAAGTCTGGTGATGCTGCCATTGTTGATATGGTTCCTGGCAAGCCCA
TGTGTGTTGAGAGCTTCTCAGACTATCCAC 69 (0.002101%)

CTGGAGAAGAGCTACGAGCTGCCTGACGGCCAGGTCATCACCATTGGCAATGAGCGGTTCCGCTGCCCT
GAGGCACTCTTCCAGCCTTCCTTCCTGGGCA 3 (0.000091%)

CTGGAGCTGGCAAGGTCACCAAGTCTGCCCAGAAAGCTCAGAAGGCTAAATGAATATTATCCCTAATAC
CTGCCACCCCACTCTTAATCAGTGGTGGAAG 12 (0.000365%)

CTGGCAAGCCCATGTGTGTTGAGAGCTTCTCAGACTATCCACCTTTGGGTCGCTTTGCTGTTCGTGATATG
AGACAGACAGTTGCGGTGGGTGTCATCAA40 (0.001218%)

CTGGCTTACACACTGGGTGTGAAACAACTAATTGTCGGTGTTAACAAAATGGATTCCACTGAGCCACCCT
ACAGCCAGAAGAGATATGAGGAAATTGTTA 26 (0.000792%)

CTGGGCGACTCTGTGCCTCGCTGAGGAAAAATAACTAAACATGGGCAAAGGAGATCCTAAGAAGCCGA
GAGGCAAAATGTCATCATATGCATTTTTTGTG 9 (0.000274%)

CTGGGTCTTGGATAAACTGAAAGCTGAGCGTGAACGTGGTATCACCATTGATATCTCCTTGTGGAAATTT
GAGACCAGCAAGTACTATGTGACTATCATT 23 (0.000700%)

CTGGGTGTGAAACAACTAATTGTCGGTGTTAACAAAATGGATTCCACTGAGCCACCCTACAGCCAGAAG
AGATATGAGGAAATTGTTAAGGAAGTCAGCA 10 (0.000305%)

CTGGTAAAAAGCTGGAAGATGGCCCTAAATTCTTGAAGTCTGGTGATGCTGCCATTGTTGATATGGTTCC
TGGCAAGCCCATGTGTGTTGAGAGCTTCTC 22 (0.000670%)

CTGGTGATGCTGCCATTGTTGATATGGTTCCTGGCAAGCCCATGTGTGTTGAGAGCTTCTCAGACTATCC
ACCTTTGGGTCGCTTTGCTGTTCGTGATAT18 (0.000548%)

CTGGTGTTCTCAAACCCGGTATGGTGGTCACCTTTGCTCCAGTCAACGTTACAACGGAAGTAAAATCTGT
CGAAATGCACCATGAAGCTTTGAGTGAAGC 59 (0.001797%)

CTGTCAAGGATGTTCGTCGTGGCAACGTTGCTGGTGACAGCAAAAATGACCCACCAATGGAAGCAGCTG
GCTTCACTGCTCAGGTGATTATCCTGAACCA 7 (0.000213%)

CTGTCCTGATTGTTGCTGCTGGTGTTGGTGAATTTGAAGCTGGTATCTCCAAGAATGGGCAGACCCGAGA

GCATGCCCTTCTGGCTTACACACTGGGTGT 3 (0.000091%)

CTGTGCCTCGCTGAGGAAAAATAACTAAACATGGGCAAAGGAGATCCTAAGAAGCCGAGAGGCAAAAT
GTCATCATATGCATTTTTTGTGCAAACTTGTC 8 (0.000244%)

CTGTGCTGTCCTGATTGTTGCTGCTGGTGTTGGTGAATTTGAAGCTGGTATCTCCAAGAATGGGCAGACC
CGAGAGCATGCCCTTCTGGCTTACACACTG 9 (0.000274%)

CTGTGTTCCGCGCGCACGTGAAGCACCGTAAAGGCGCTGCGCGCCTGCGCGCCGTGGATTTCGCTGAGC
GGCACGGCTACATCAAGGGCATCGTCAAGGA 16 (0.000487%)

CTGTGTTGGTTTTACTAAAAAACGCAACAATCAGATACGGAAGACCTCTTATGCTCAGCACCAACAGGTC
CGCCAAATCCGGAAGAAGATGATGGAAATC 3 (0.000091%)

CTGTTCGCTTCATTCATTGCCCCCACAATCCTAGGCCTACCCGCCGCAGTACTGATCATTCTATTTCCCCC
TCTATTGATCCCCACCTCCAAATATCTCA 10 (0.000305%)

CTGTTCTGTGTTGGTTTTACTAAAAAACGCAACAATCAGATACGGAAGACCTCTTATGCTCAGCACCAAC
AGGTCCGCCAAATCCGGAAGAAGATGATGG 3 (0.000091%)

CTTAATCATTTTTATTGCCACAACTAACCTCCTCGGACTCCTGCCTCACTCATTTACACCAACCACCCAAC
TATCTATAAACCTAGCCATGGCCATCCCC 13 (0.000396%)

CTTACACACTGGGTGTGAAACAACTAATTGTCGGTGTTAACAAAATGGATTCCACTGAGCCACCCTACAG
CCAGAAGAGATATGAGGAAATTGTTAAGGA 11 (0.000335%)

CTTACTACTCCGGAAAAAAAGAACCATTTGGATACATAGGTATGGTCTGAGCTATGATATCAATTGGCTT
CCTAGGGTTTATCGTGTGAGCACACCATAT 10 (0.000305%)

CTTATTCGAGCCGAGCTGGGCCAGCCAGGCAACCTTCTAGGTAACGACCACATCTACAACGTTATCGTCA
CAGCCCATGCATTTGTAATAATCTTCTTCA4 (0.000122%)

CTTCAAGTATGCCTGGGTCTTGGATAAACTGAAAGCTGAGCGTGAACGTGGTATCACCATTGATATCTCC
TTGTGGAAATTTGAGACCAGCAAGTACTAT 67 (0.002040%)

CTTCAATGTCAAGAATGTGTCTGTCAAGGATGTTCGTCGTGGCAACGTTGCTGGTGACAGCAAAAATGAC
CCACCAATGGAAGCAGCTGGCTTCACTGCT 142 (0.004324%)

CTTCACCCGTAACCCACCGCCATGGCCGAGGAAGGCATTGCTGCTGGAGGTGTAATGGACGTTAATACT
GCTTTACAAGAGGTTCTGAAGACTGCCCTCA 24 (0.000731%)

CTTCATCCCTCTCCAGAAGAGGAGAAGAGGAAACACAAGAAGAAACGCCTGGTGCAGAGCCCCAATTCC
TACTTCATGGATGTGAAATGCCCAGGATGCT 15 (0.000457%)

CTTCATTCATTGCCCCCACAATCCTAGGCCTACCCGCCGCAGTACTGATCATTCTATTTCCCCCTCTATTG
ATCCCCACCTCCAAATATCTCATCAACAA 12 (0.000365%)

CTTCCACCCTTACTACACAATCAAAGACGCCCTCGGCTTACTTCTCTTCATTCTCTCCTTAATGACATTAA
CACTATTCTCACCAGACCTCCTAGGCGAC 4 (0.000122%)

CTTCCCCCATTATTCCTAGAACCAGGCGACCTGCGACTCCTTGACGTTGACAATCGAGTAGTACTCCCGA
TTGAAGCCCCCATTCGTATAATAATTACAT 15 (0.000457%)

CTTCGATACGGGATAATCCTATTTATTACCTCAGAAGTTTTTTTCTTCGCAGGATTTTTCTGAGCCTTTTAC
CACTCCAGCCTAGCCCCTACCCCCCAAT 67 (0.002040%)

CTTCGCATCCGCAAACTCTGTCTCAACATCTGTGTTGGGGAGAGTGGAGACAGACTGACGCGAGCAGCC
AAGGTGTTGGAGCAGCTCACAGGGCAGACCC 4 (0.000122%)

CTTCGGAATAATCTCCCATATTGTAACTTACTACTCCGGAAAAAAAGAACCATTTGGATACATAGGTATG
GTCTGAGCTATGATATCAATTGGCTTCCTA 13 (0.000396%)

CTTCTCCTATCTCTCCCAGTCCTAGCTGCTGGCATCACTATACTACTAACAGACCGCAACCTCAACACCA
CCTTCTTCGACCCCGCCGGAGGAGGAGACC 10 (0.000305%)

CTTCTGGCTTACACACTGGGTGTGAAACAACTAATTGTCGGTGTTAACAAAATGGATTCCACTGAGCCAC
CCTACAGCCAGAAGAGATATGAGGAAATTG 23 (0.000700%)

CTTGAAGTCTGGTGATGCTGCCATTGTTGATATGGTTCCTGGCAAGCCCATGTGTGTTGAGAGCTTCTCA
GACTATCCACCTTTGGGTCGCTTTGCTGTT 235 (0.007156%)

CTTGATGGTCGAGGCCATCTCCTGGGCCGCCTGGCGGCCATCGTGGCTAAACAGGTACTGCTGGGCCGG
AAGGTGGTGGTCGTACGCTGTGAAGGCATCA 30 (0.000914%)

CTTGCTTCGTCTGTTCTGTGTTGGTTTACTAAAAAACGCAACAATCAGATACGGAAGACCTCTTATGCTC
AGCACCAACAGGTCCGCCAAATCCGGAAG 9 (0.000274%)

CTTGGATAAACTGAAAGCTGAGCGTGAACGTGGTATCACCATTGATATCTCCTTGTGGAAATTTGAGACC
AGCAAGTACTATGTGACTATCATTGATGCC 80 (0.002436%)

CTTGTGGAAATTTGAGACCAGCAAGTACTATGTGACTATCATTGATGCCCCAGGACACAGAGACTTTATC
AAAAACATGATTACAGGGACATCTCAGGCT 19 (0.000579%)

CTTTACATAACAGACGAGGTCAACGATCCCTCCCTTACCATCAAATCAATTGGCCACCAATGGTACTGAA
CCTACGAGTACACCGACTACGGCGGACTAA 13 (0.000396%)

CTTTATCAAAAACATGATTACAGGGACATCTCAGGCTGACTGTGCTGTCCTGATTGTTGCTGCTGGTGTT
GGTGAATTTGAAGCTGGTATCTCCAAGAAT 226 (0.006882%)

CTTTGACTGCTTCCATGTTGGCATCTGCCCCTCCTCAAGAGCAAAAGCAAATGTTGGGTGAACGGCTGTT
TCCTCTTATTCAAGCCATGCACCCTACTCT 6 (0.000183%)

CTTTGCTCCAGTCAACGTTACAACGGAAGTAAAATCTGTCGAAATGCACCATGAAGCTTTGAGTGAAGCT
CTTCCTGGGGACAATGTGGGCTTCAATGTC 92 (0.002802%)

CTTTGGGTCGCTTTGCTGTTCGTGATATGAGACAGACAGTTGCGGTGGGTGTCATCAAAGCAGTGGACAA
GAAGGCTGCTGGAGCTGGCAAGGTCACCAA 55 (0.001675%)

GAAAATCTGTTCGCTTCATTCATTGCCCCCACAATCCTAGGCCTACCCGCCGCAGTACTGATCATTCTATT
TCCCCCTCTATTGATCCCCACCTCCAAAT 35 (0.001066%)

GAAACAACTAATTGTCGGTGTTAACAAAATGGATTCCACTGAGCCACCCTACAGCCAGAAGAGATATGA
GGAAATTGTTAAGGAAGTCAGCACTTACATT 36 (0.001096%)

GAAACCGTCTGAACTATCCTGCCCGCCATCATCCTAGTCCTCATCGCCCTCCCATCCCTACGCATCCTTTA
CATAACAGACGAGGTCAACGATCCCTCCC 14 (0.000426%)

GAAAGCTGAGCGTGAACGTGGTATCACCATTGATATCTCCTTGTGGAAATTTGAGACCAGCAAGTACTAT
GTGACTATCATTGATGCCCCAGGACACAGA 36 (0.001096%)

GAAAGGGCTCCTTCAAGTATGCCTGGGTCTTGGATAAACTGAAAGCTGAGCGTGAACGTGGTATCACCA
TTGATATCTCCTTGTGGAAATTTGAGACCAG 653 (0.019886%)

GAAAGTTCTCCGCTCCCAGACATGGGTCCCTCGGCTTCCTGCCTCGGAAGCGCAGCAGCAGGCATCGTG
GGAAGGTGAAGAGCTTCCCTAAGGATGACCC 25 (0.000761%)

GAACACAGGTGTCGTGAAAACTACCCCTAAAAGCCAAAATGGGAAAGGAAAAGACTCATATCAACATT
GTCGTCATTGGACACGTAGATTCGGGCAAGTC 46 (0.001401%)

GAACCTTTGACTGCTTCCATGTTGGCATCTGCCCCTCCTCAAGAGCAAAAGCAAATGTTGGGTGAACGGC
TGTTTCCTCTTATTCAAGCCATGCACCCTA 108 (0.003289%)

GAACGAGAAGCTGAACTTGGAGCTAGGGCAAAAGAATTCACCAATGTTTACATCAAGAATTTTGGAGAA
GACATGGATGATGAGCGCCTTAAGGATCTCT 3 (0.000091%)

GAACGCAGGCACATACTTCCTATTCTACACCCTAGTAGGCTCCCTTCCCCTACTCATCGCACTGATTTACA
CTCACAACACCCTAGGCTCACTAAACATT 6 (0.000183%)

GAACGCCTGAACGCAGGCACATACTTCCTATTCTACACCCTAGTAGGCTCCCTTCCCCTACTCATCGCAC
TGATTTACACTCACAACACCCTAGGCTCAC 14 (0.000426%)

GAACGTGGTATCACCATTGATATCTCCTTGTGGAAATTTGAGACCAGCAAGTACTATGTGACTATCATTG
ATGCCCCAGGACACAGAGACTTTATCAAAA 15 (0.000457%)

GAACTATCCTGCCCGCCATCATCCTAGTCCTCATCGCCCTCCCATCCCTACGCATCCTTTACATAACAGAC
GAGGTCAACGATCCCTCCCTTACCATCAA 34 (0.001035%)

GAACTCCTCACACCCAATTGGACCAATCTATCACCCTATAGAAGAACTAATGTTAGTATAAGTAACATGA
AAACATTCTCCTCCGCATAAGCCTGCGTCA 12 (0.000365%)

GAAGATCCTGGTGTCGCCATGGGCCGCCGCCCCGCCCGTTGTTACCGGTATTGTAAGAACAAGCCGTACC
CAAAGTCTCGCTTCTGCCGAGGTGTCCCTGATGCCAAGATTCGCATTTTTGACCTGGGGCGGAAAAAGGC
AAAAGTGGA 207 (0.009392%)

GAAGATGGCCCTAAATTCTTGAAGTCTGGTGATGCTGCCATTGTTGATATGGTTCCTGGCAAGCCCATGT
GTGTTGAGAGCTTCTCAGACTATCCACCTT 10 (0.000305%)

GAAGCTACAATGATTTTGGGAATTACAACAATCAGTCTTCAAATTTTGGACCCATGAAGGGAGGAAATT
TTGGAGGCAGAAGCTCTGGCCCCTATGGCGG 7 (0.000213%)

GAAGGAGGCTGCTGAGATGGGAAAGGGCTCCTTCAAGTATGCCTGGGTCTTGGATAAACTGAAAGCTGA
GCGTGAACGTGGTATCACCATTGATATCTCC 52 (0.001584%)

GAAGGCTGCTGGAGCTGGCAAGGTCACCAAGTCTGCCCAGAAAGCTCAGAAGGCTAAATGAATATTATC
CCTAATACCTGCCACCCCACTCTTAATCAGT 31 (0.000944%)

GAAGGGCGCCGGGTCTGTGTTCCGCGCGCACGTGAAGCACCGTAAAGGCGCTGCGCGCCTGCGCGCCGT
GGATTTCGCTGAGCGGCACGGCTACATCAAG 6 (0.000183%)

GAAGTCTGGTGATGCTGCCATTGTTGATATGGTTCCTGGCAAGCCCATGTGTGTTGAGAGCTTCTCAGAC
TATCCACCTTTGGGTCGCTTTGCTGTTCGT 7 (0.000213%)

GAAGTTTTTTTCTTCGCAGGATTTTTCTGAGCCTTTTACCACTCCAGCCTAGCCCCTACCCCCCAATTAGG
AGGGCACTGGCCCCCAACAGGCATCACCC 5 (0.000152%)

GAATAATCTCCCATATTGTAACTTACTACTCCGGAAAAAAAGAACCATTTGGATACATAGGTATGGTCTG
AGCTATGATATCAATTGGCTTCCTAGGGTT 47 (0.001431%)

GAATGCGCAGGCTGAAGCGCAAAAGAAGAAAGATGAGGCAGAGGTCCAAGTAAACCGCTAGCTTGTTG
CACCGTGGAGGCCACAGGAGCAGAAACATGGA 8 (0.000244%)

GAATGTGTCTGTCAAGGATGTTCGTCGTGGCAACGTTGCTGGTGACAGCAAAAATGACCCACCAATGGA
AGCAGCTGGCTTCACTGCTCAGGTGATTATC 12 (0.000365%)

GACAAGAAGGCTGCTGGAGCTGGCAAGGTCACCAAGTCTGCCCAGAAAGCTCAGAAGGCTAAATGAAT
ATTATCCCTAATACCTGCCACCCCACTCTTAA 15 (0.000457%)

GACACAGAGACTTTATCAAAAACATGATTACAGGGACATCTCAGGCTGACTGTGCTGTCCTGATTGTTGC
TGCTGGTGTTGGTGAATTTGAAGCTGGTAT 9 (0.000274%)

GACAGACAGTTGCGGTGGGTGTCATCAAAGCAGTGGACAAGAAGGCTGCTGGAGCTGGCAAGGTCACC
AAGTCTGCCCAGAAAGCTCAGAAGGCTAAATG 44 (0.001340%)

GACAGAGGAAGGGCGCCGGGTCTGTGTTCCGCGCGCACGTGAAGCACCGTAAAGGCGCTGCGCGCCTGC
GCGCCGTGGATTTCGCTGAGCGGCACGGCTA 9 (0.000274%)

GACAGTTGCGGTGGGTGTCATCAAAGCAGTGGACAAGAAGGCTGCTGGAGCTGGCAAGGTCACCAAGTC
TGCCCAGAAAGCTCAGAAGGCTAAATGAATA 21 (0.000640%)

GACATCTCAGGCTGACTGTGCTGTCCTGATTGTTGCTGCTGGTGTTGGTGAATTTGAAGCTGGTATCTCCA
AGAATGGGCAGACCCGAGAGCATGCCCTT 5 (0.000152%)

GACATGGGTCCCTCGGCTTCCTGCCTCGGAAGCGCAGCAGCAGGCATCGTGGGAAGGTGAAGAGCTTCC
CTAAGGATGACCCGTCCAAGCCGGTCCACCT 5 (0.000152%)

GACCAATCTATCACCCTATAGAAGAACTAATGTTAGTATAAGTAACATGAAAACATTCTCCTCCGCATAA
GCCTGCGTCAGATTAAAACACTGAACTGAC 5 (0.000152%)

GACCAGCAAGTACTATGTGACTATCATTGATGCCCCAGGACACAGAGACTTTATCAAAAACATGATTAC
AGGGACATCTCAGGCTGACTGTGCTGTCCTG 3 (0.000091%)

GACCCCTAACAGGGGCCCTCTCAGCCCTCCTAATGACCTCCGGCCTAGCCATGTGATTTCACTTCCACTC
CATAACGCTCCTCATACTAGGCCTACTAAC 8 (0.000244%)

GACGGGCACTGGGCGACTCTGTGCCTCGCTGAGGAAAAATAACTAAACATGGGCAAAGGAGATCCTAA
GAAGCCGAGAGGCAAAATGTCATCATATGCAT 5 (0.000152%)

GACTAATCACCACCCAACAATGACTAATCAAACTAACCTCAAAACAAATGATAACCATACACAACACTA
AAGGACGAACCTGATCTCTTATACTAGTATC 16 (0.000487%)

GACTAGTTCCCCTAATAATCGGTGCCCCCGATATGGCGTTTCCCCGCATAAACAACATAAGCTTCTGACT
CTTACCTCCCTCTCTCCTACTCCTGCTCGC 12 (0.000365%)

GACTATCATTGATGCCCCAGGACACAGAGACTTTATCAAAAACATGATTACAGGGACATCTCAGGCTGA
CTGTGCTGTCCTGATTGTTGCTGCTGGTGTT 8 (0.000244%)

GACTATCCACCTTTGGGTCGCTTTGCTGTTCGTGATATGAGACAGACAGTTGCGGTGGGTGTCATCAAAG
CAGTGGACAAGAAGGCTGCTGGAGCTGGCA 7 (0.000213%)

GACTCTGTGCCTCGCTGAGGAAAAATAACTAAACATGGGCAAAGGAGATCCTAAGAAGCCGAGAGGCA
AAATGTCATCATATGCATTTTTTGTGCAAACT 3 (0.000091%)

GACTGGTGTTCTCAAACCCGGTATGGTGGTCACCTTTGCTCCAGTCAACGTTACAACGGAAGTAAAATCT
GTCGAAATGCACCATGAAGCTTTGAGTGAA 13 (0.000396%)

GACTGTGCTGTCCTGATTGTTGCTGCTGGTGTTGGTGAATTTGAAGCTGGTATCTCCAAGAATGGGCAGA
CCCGAGAGCATGCCCTTCTGGCTTACACAC 4 (0.000122%)

GAGAAGGAGGCTGCTGAGATGGGAAAGGGCTCCTTCAAGTATGCCTGGGTCTTGGATAAACTGAAAGCT
GAGCGTGAACGTGGTATCACCATTGATATCT 13 (0.000396%)

GAGACAGACAGTTGCGGTGGGTGTCATCAAAGCAGTGGACAAGAAGGCTGCTGGAGCTGGCAAGGTCA
CCAAGTCTGCCCAGAAAGCTCAGAAGGCTAAA 29 (0.000883%)

GAGACCAGCAAGTACTATGTGACTATCATTGATGCCCCAGGACACAGAGACTTTATCAAAAACATGATT
ACAGGGACATCTCAGGCTGACTGTGCTGTCC 6 (0.000183%)

GAGACCCCATTCTATACCAACACCTATTCTGATTTTTCGGTCACCCTGAAGTTTATATTCTTATCCTACCA
GGCTTCGGAATAATCTCCCATATTGTAAC 8 (0.000244%)

GAGACTGGTGTTCTCAAACCCGGTATGGTGGTCACCTTTGCTCCAGTCAACGTTACAACGGAAGTAAAAT
CTGTCGAAATGCACCATGAAGCTTTGAGTG 4 (0.000122%)

GAGAGCATGCCCTTCTGGCTTACACACTGGGTGTGAAACAACTAATTGTCGGTGTTAACAAAATGGATTC
CACTGAGCCACCCTACAGCCAGAAGAGATA 15 (0.000457%)

GAGAGCCGGACGGGCACTGGGCGACTCTGTGCCTCGCTGAGGAAAAATAACTAAACATGGGCAAAGGA
GATCCTAAGAAGCCGAGAGGCAAAATGTCATC 213 (0.006486%)

GAGAGCTTCTCAGACTATCCACCTTTGGGTCGCTTTGCTGTTCGTGATATGAGACAGACAGTTGCGGTGG
GTGTCATCAAAGCAGTGGACAAGAAGGCTG 15 (0.000457%)

GAGCATGCCCTTCTGGCTTACACACTGGGTGTGAAACAACTAATTGTCGGTGTTAACAAAATGGATTCCA
CTGAGCCACCCTACAGCCAGAAGAGATATG 11 (0.000335%)

GAGCCAAGTGCTAACATGCCTTGGTTCAAGGGATGGAAAGTCACCCGTAAGGATGGCAATGCCAGTGGA
ACCACGCTGCTTGAGGCTCTGGACTGCATCC 3 (0.000091%)

GAGCCGGACGGGCACTGGGCGACTCTGTGCCTCGCTGAGGAAAAATAACTAAACATGGGCAAAGGAGA
TCCTAAGAAGCCGAGAGGCAAAATGTCATCAT 3 (0.000091%)

GAGCTACGAGCTGCCTGACGGCCAGGTCATCACCATTGGCAATGAGCGGTTCCGCTGCCCTGAGGCACT
CTTCCAGCCTTCCTTCCTGGGCATGGAGTCC 3 (0.000091%)

GAGCTGGCAAGGTCACCAAGTCTGCCCAGAAAGCTCAGAAGGCTAAATGAATATTATCCCTAATACCTG
CCACCCCACTCTTAATCAGTGGTGGAAGAAC 16 (0.000487%)

GAGCTTCTCAGACTATCCACCTTTGGGTCGCTTTGCTGTTCGTGATATGAGACAGACAGTTGCGGTGGGT
GTCATCAAAGCAGTGGACAAGAAGGCTGCT 7 (0.000213%)

GAGGAAGGGCGCCGGGTCTGTGTTCCGCGCGCACGTGAAGCACCGTAAAGGCGCTGCGCGCCTGCGCGC
CGTGGATTTCGCTGAGCGGCACGGCTACATC 4 (0.000122%)

GAGGAGACCCCATTCTATACCAACACCTATTCTGATTTTTCGGTCACCCTGAAGTTTATATTCTTATCCTA
CCAGGCTTCGGAATAATCTCCCATATTGT 16 (0.000487%)

GAGGCACCCGATATATGTTCTCTAGGCCTTTTAGAAAACATGGAGTTGTTCCTTTGGCCACATATATGCG
AATCTATAAGAAAGGTGATATTGTAGACAT 7 (0.000213%)

GAGGTCAACGATCCCTCCCTTACCATCAAATCAATTGGCCACCAATGGTACTGAACCTACGAGTACACCG
ACTACGGCGGACTAATCTTCAACTCCTACA 7 (0.000213%)

GATAAACTGAAAGCTGAGCGTGAACGTGGTATCACCATTGATATCTCCTTGTGGAAATTTGAGACCAGC
AAGTACTATGTGACTATCATTGATGCCCCAG 71 (0.002162%)

GATAATCCTATTTATTACCTCAGAAGTTTTTTTCTTCGCAGGATTTTTCTGAGCCTTTTACCACTCCAGCCT
AGCCCCTACCCCCCAATTAGGAGGGCAC 11 (0.000335%)

GATATATGTTCTCTAGGCCTTTTAGAAAACATGGAGTTGTTCCTTTGGCCACATATATGCGAATCTATAA
GAAAGGTGATATTGTAGACATCAAGGGAAT 7 (0.000213%)

GATATCTCCTTGTGGAAATTTGAGACCAGCAAGTACTATGTGACTATCATTGATGCCCCAGGACACAGAG
ACTTTATCAAAAACATGATTACAGGGACAT 16 (0.000487%)

GATATGGTTCCTGGCAAGCCCATGTGTGTTGAGAGCTTCTCAGACTATCCACCTTTGGGTCGCTTTGCTGT

TCGTGATATGAGACAGACAGTTGCGGTGG9 (0.000274%)

GATCCGTGGACAGAGGAAGGGCGCCGGGTCTGTGTTCCGCGCGCACGTGAAGCACCGTAAAGGCGCTGC
GCGCCTGCGCGCCGTGGATTTCGCTGAGCGG 416 (0.012668%)

GATCTCCTTCATCCCTCTCCAGAAGAGGAGAAGAGGAAACACAAGAAGAAACGCCTGGTGCAGAGCCCC
AATTCCTACTTCATGGATGTGAAATGCCCAG 5 (0.000152%)

GATGCCCCAGGACACAGAGACTTTATCAAAAACATGATTACAGGGACATCTCAGGCTGACTGTGCTGTC
CTGATTGTTGCTGCTGGTGTTGGTGAATTTG 4 (0.000122%)

GATGCTGCCATTGTTGATATGGTTCCTGGCAAGCCCATGTGTGTTGAGAGCTTCTCAGACTATCCACCTTT
GGGTCGCTTTGCTGTTCGTGATATGAGAC 9 (0.000274%)

GATGGAAAGTCACCCGTAAGGATGGCAATGCCAGTGGAACCACGCTGCTTGAGGCTCTGGACTGCATCC
TACCACCAACTCGTCCAACTGACAAGCCCTT 3 (0.000091%)

GATGGAAGCAATTTTGGAGGTGGTGGAAGCTACAATGATTTTGGGAATTACAACAATCAGTCTTCAAAT
TTTGGACCCATGAAGGGAGGAAATTTTGGAG 6 (0.000183%)

GATGGCCCTAAATTCTTGAAGTCTGGTGATGCTGCCATTGTTGATATGGTTCCTGGCAAGCCCATGTGTG
TTGAGAGCTTCTCAGACTATCCACCTTTGG 20 (0.000609%)

GATGGTCGAGGCCATCTCCTGGGCCGCCTGGCGGCCATCGTGGCTAAACAGGTACTGCTGGGCCGGAAG
GTGGTGGTCGTACGCTGTGAAGGCATCAACA 6 (0.000183%)

GATGGTTACTTGCTTCGTCTGTTCTGTGTTGGTTTTACTAAAAAACGCAACAATCAGATACGGAAGACCT
CTTATGCTCAGCACCAACAGGTCCGCCAAA 5 (0.000152%)

GATTACAGGGACATCTCAGGCTGACTGTGCTGTCCTGATTGTTGCTGCTGGTGTTGGTGAATTTGAAGCT
GGTATCTCCAAGAATGGGCAGACCCGAGAG 4 (0.000122%)

GATTCGGGCAAGTCCACCACTACTGGCCATCTGATCTATAAATGCGGTGGCATCGACAAAAGAACCATT
GAAAAATTTGAGAAGGAGGCTGCTGAGATGG 7 (0.000213%)

GATTGCCACACGGCTCACATTGCATGCAAGTTTGCTGAGCTGAAGGAAAAGATTGATCGCCGTTCTGGTA
AAAAGCTGGAAGATGGCCCTAAATTCTTGA 24 (0.000731%)

GATTTTGGGAATTACAACAATCAGTCTTCAAATTTTGGACCCATGAAGGGAGGAAATTTTGGAGGCAGA
AGCTCTGGCCCCTATGGCGGTGGAGGCCAAT 4 (0.000122%)

GCAAAAGAAGAAAGATGAGGCAGAGGTCCAAGTAAACCGCTAGCTTGTTGCACCGTGGAGGCCACAGG
AGCAGAAACATGGAATGCCAGACGCTGGGGAT 7 (0.000213%)

GCAAAGGATCTCCTTCATCCCTCTCCAGAAGAGGAGAAGAGGAAACACAAGAAGAAACGCCTGGTGCA
GAGCCCCAATTCCTACTTCATGGATGTGAAAT 3 (0.000091%)

GCAAAGGCCCCAACGTTGTAGGCCCCTACGGGCTACTACAACCCTTCGCTGACGCCATAAAACTCTTCAC
CAAAGAGCCCCTAAAACCCGCCACATCTAC 300 (0.009136%)

GCAACCTTCTAGGTAACGACCACATCTACAACGTTATCGTCACAGCCCATGCATTTGTAATAATCTTCTT
CATAGTAATACCCATCATAATCGGAGGCTT 17 (0.000518%)

GCAAGCCCATGTGTGTTGAGAGCTTCTCAGACTATCCACCTTTGGGTCGCTTTGCTGTTCGTGATATGAG
ACAGACAGTTGCGGTGGGTGTCATCAAAGC 15 (0.000457%)

GCAAGTACTATGTGACTATCATTGATGCCCCAGGACACAGAGACTTTATCAAAAACATGATTACAGGGA
CATCTCAGGCTGACTGTGCTGTCCTGATTGT 16 (0.000487%)

GCAAGTCCACCACTACTGGCCATCTGATCTATAAATGCGGTGGCATCGACAAAAGAACCATTGAAAAAT
TTGAGAAGGAGGCTGCTGAGATGGGAAAGGG 6 (0.000183%)

GCAATTTTGGAGGTGGTGGAAGCTACAATGATTTTGGGAATTACAACAATCAGTCTTCAAATTTTGGACC
CATGAAGGGAGGAAATTTTGGAGGCAGAAG 41 (0.001249%)

GCACCAGCGGTGGCAGAGACCCCAGACATCAAGCTCTTTGGGAAGTGGAGCACCGATGATGTGCAGATC
AATGACATTTCCCTGCAGGATTACATTGCAG 4 (0.000122%)

GCACCCGATATATGTTCTCTAGGCCTTTTAGAAAACATGGAGTTGTTCCTTTGGCCACATATATGCGAAT
CTATAAGAAAGGTGATATTGTAGACATCAA 3 (0.000091%)

GCACCGTAAAGGCGCTGCGCGCCTGCGCGCCGTGGATTTCGCTGAGCGGCACGGCTACATCAAGGGCAT
CGTCAAGGACATCATCCACGACCCGGGCCGC 4 (0.000122%)

GCACTGCTTATTACAATTTTACTGGGTCTCTATTTTACCCTCCTACAAGCCTCAGAGTACTTCGAGTCTCC
CTTCACCATTTCCGACGGCATCTACGGCT 8 (0.000244%)

GCAGACCCGAGAGCATGCCCTTCTGGCTTACACACTGGGTGTGAAACAACTAATTGTCGGTGTTAACAA
AATGGATTCCACTGAGCCACCCTACAGCCAG 17 (0.000518%)

GCAGCACCAGCGGTGGCAGAGACCCCAGACATCAAGCTCTTTGGGAAGTGGAGCACCGATGATGTGCAG
ATCAATGACATTTCCCTGCAGGATTACATTG 17 (0.000518%)

GCAGCAGCCACCGCCGCAGCAGCCGCCACCGCATCAGCCGCCGCCGCATCCACAGCCGCATCAGCAGCA
GCAGCCGCCGCCACCGCCGCAGGACTCTTCC 4 (0.000122%)

GCAGGCACATACTTCCTATTCTACACCCTAGTAGGCTCCCTTCCCCTACTCATCGCACTGATTTACACTCA
CAACACCCTAGGCTCACTAAACATTCTAC 3 (0.000091%)

GCAGGCTGAAGCGCAAAAGAAGAAAGATGAGGCAGAGGTCCAAGTAAACCGCTAGCTTGTTGCACCGT
GGAGGCCACAGGAGCAGAAACATGGAATGCCA 16 (0.000487%)

GCAGTGGACAAGAAGGCTGCTGGAGCTGGCAAGGTCACCAAGTCTGCCCAGAAAGCTCAGAAGGCTAA
ATGAATATTATCCCTAATACCTGCCACCCCAC 20 (0.000609%)

GCATCCGCAAACTCTGTCTCAACATCTGTGTTGGGGAGAGTGGAGACAGACTGACGCGAGCAGCCAAGG
TGTTGGAGCAGCTCACAGGGCAGACCCCTGT 3 (0.000091%)

GCATCCTTTACATAACAGACGAGGTCAACGATCCCTCCCTTACCATCAAATCAATTGGCCACCAATGGTA
CTGAACCTACGAGTACACCGACTACGGCGG 12 (0.000365%)

GCATGCCCTTCTGGCTTACACACTGGGTGTGAAACAACTAATTGTCGGTGTTAACAAAATGGATTCCACT
GAGCCACCCTACAGCCAGAAGAGATATGAG 13 (0.000396%)

GCATTTGTGCCAATTTCTGGTTGGAATGGTGACAACATGCTGGAGCCAAGTGCTAACATGCCTTGGTTCA
AGGGATGGAAAGTCACCCGTAAGGATGGCA 8 (0.000244%)

GCCAAGTGCTAACATGCCTTGGTTCAAGGGATGGAAAGTCACCCGTAAGGATGGCAATGCCAGTGGAAC
CACGCTGCTTGAGGCTCTGGACTGCATCCTA 21 (0.000640%)

GCCACAACTAACCTCCTCGGACTCCTGCCTCACTCATTTACACCAACCACCCAACTATCTATAAACCTAG
CCATGGCCATCCCCTTATGAGCGGGCGCAG 13 (0.000396%)

GCCACACGGCTCACATTGCATGCAAGTTTGCTGAGCTGAAGGAAAAGATTGATCGCCGTTCTGGTAAAA
AGCTGGAAGATGGCCCTAAATTCTTGAAGTC 23 (0.000700%)

GCCAGAACACAGGTGTCGTGAAAACTACCCCTAAAAGCCAAAATGGGAAAGGAAAAGACTCATATCAA
CATTGTCGTCATTGGACACGTAGATTCGGGCA 526 (0.016018%)

GCCAGAACGCCTGAACGCAGGCACATACTTCCTATTCTACACCCTAGTAGGCTCCCTTCCCCTACTCATC
GCACTGATTTACACTCACAACACCCTAGGC 397 (0.012090%)

GCCAGGCAACCTTCTAGGTAACGACCACATCTACAACGTTATCGTCACAGCCCATGCATTTGTAATAATC
TTCTTCATAGTAATACCCATCATAATCGGA 15 (0.000457%)

GCCATCTCCTGGGCCGCCTGGCGGCCATCGTGGCTAAACAGGTACTGCTGGGCCGGAAGGTGGTGGTCG
TACGCTGTGAAGGCATCAACATTTCTGGCAA 26 (0.000792%)

GCCATTGTTGATATGGTTCCTGGCAAGCCCATGTGTGTTGAGAGCTTCTCAGACTATCCACCTTTGGGTC
GCTTTGCTGTTCGTGATATGAGACAGACAG 15 (0.000457%)

GCCCATAAAAATAAAAAATTATAACAAACCCTGAGAACCAAAATGAACGAAAATCTGTTCGCTTCATTC
ATTGCCCCCACAATCCTAGGCCTACCCGCCG 7 (0.000213%)

GCCCATGACCCCTAACAGGGGCCCTCTCAGCCCTCCTAATGACCTCCGGCCTAGCCATGTGATTTCACTT
CCACTCCATAACGCTCCTCATACTAGGCCT 17 (0.000518%)

GCCCATGTGTGTTGAGAGCTTCTCAGACTATCCACCTTTGGGTCGCTTTGCTGTTCGTGATATGAGACAG
ACAGTTGCGGTGGGTGTCATCAAAGCAGTG 7 (0.000213%)

GCCCCCGATATGGCGTTTCCCCGCATAAACAACATAAGCTTCTGACTCTTACCTCCCTCTCTCCTACTCCT
GCTCGCATCTGCTATAGTGGAGGCCGGAG 3 (0.000091%)

GCCCGCCATCATCCTAGTCCTCATCGCCCTCCCATCCCTACGCATCCTTTACATAACAGACGAGGTCAAC
GATCCCTCCCTTACCATCAAATCAATTGGC 7 (0.000213%)

GCCCTAAATTCTTGAAGTCTGGTGATGCTGCCATTGTTGATATGGTTCCTGGCAAGCCCATGTGTGTTGA
GAGCTTCTCAGACTATCCACCTTTGGGTCG 12 (0.000365%)

GCCCTCCCATCCCTACGCATCCTTTACATAACAGACGAGGTCAACGATCCCTCCCTTACCATCAAATCAA
TTGGCCACCAATGGTACTGAACCTACGAGT 11 (0.000335%)

GCCCTCCTAATGACCTCCGGCCTAGCCATGTGATTTCACTTCCACTCCATAACGCTCCTCATACTAGGCCT
ACTAACCAACACACTAACCATATACCAAT 4 (0.000122%)

GCCCTCTCAGCCCTCCTAATGACCTCCGGCCTAGCCATGTGATTTCACTTCCACTCCATAACGCTCCTCAT
ACTAGGCCTACTAACCAACACACTAACCA 21 (0.000640%)

GCCCTTCTGGCTTACACACTGGGTGTGAAACAACTAATTGTCGGTGTTAACAAAATGGATTCCACTGAGC
CACCCTACAGCCAGAAGAGATATGAGGAAA 17 (0.000518%)

GCCGAGTGGAGACTGGTGTTCTCAAACCCGGTATGGTGGTCACCTTTGCTCCAGTCAACGTTACAACGGA
AGTAAAATCTGTCGAAATGCACCATGAAGC 20 (0.000609%)

GCCGGACGGGCACTGGGCGACTCTGTGCCTCGCTGAGGAAAAATAACTAAACATGGGCAAAGGAGATC
CTAAGAAGCCGAGAGGCAAAATGTCATCATAT 24 (0.000731%)

GCCGGGTCTGTGTTCCGCGCGCACGTGAAGCACCGTAAAGGCGCTGCGCGCCTGCGCGCCGTGGATTTC
GCTGAGCGGCACGGCTACATCAAGGGCATCG 21 (0.000640%)

GCCTCACTCATTTACACCAACCACCCAACTATCTATAAACCTAGCCATGGCCATCCCCTTATGAGCGGGC
GCAGTGATTATAGGCTTTCGCTCTAAGATT 8 (0.000244%)

GCCTCGCTGAGGAAAAATAACTAAACATGGGCAAAGGAGATCCTAAGAAGCCGAGAGGCAAAATGTCA
TCATATGCATTTTTTGTGCAAACTTGTCGGGA 3 (0.000091%)

GCCTCTCGCAAAGGATCTCCTTCATCCCTCTCCAGAAGAGGAGAAGAGGAAACACAAGAAGAAACGCCT

GGTGCAGAGCCCCAATTCCTACTTCATGGAT 629 (0.019155%)

GCCTGAACGCAGGCACATACTTCCTATTCTACACCCTAGTAGGCTCCCTTCCCCTACTCATCGCACTGATT
TACACTCACAACACCCTAGGCTCACTAAA 7 (0.000213%)

GCCTGGGTCTTGGATAAACTGAAAGCTGAGCGTGAACGTGGTATCACCATTGATATCTCCTTGTGGAAAT
TTGAGACCAGCAAGTACTATGTGACTATCA 16 (0.000487%)

GCCTGGTGCGGGAGCTACGGGGCCCAGGGATTGTGTTTAAAGTAGTGCTTCTACCAACATGTCCCGTGGT
TCCAGCGCCGGTTTTGACCGCCACATTACC 3 (0.000091%)

GCCTTCGATACGGGATAATCCTATTTATTACCTCAGAAGTTTTTTTCTTCGCAGGATTTTTCTGAGCCTTTT
ACCACTCCAGCCTAGCCCCTACCCCCCA 22 (0.000670%)

GCGAATGCGCAGGCTGAAGCGCAAAAGAAGAAAGATGAGGCAGAGGTCCAAGTAAACCGCTAGCTTGT
TGCACCGTGGAGGCCACAGGAGCAGAAACATG 57 (0.001736%)

GCGACTCTGTGCCTCGCTGAGGAAAAATAACTAAACATGGGCAAAGGAGATCCTAAGAAGCCGAGAGG
CAAAATGTCATCATATGCATTTTTTGTGCAAA 5 (0.000152%)

GCGCAAAAGAAGAAAGATGAGGCAGAGGTCCAAGTAAACCGCTAGCTTGTTGCACCGTGGAGGCCACA
GGAGCAGAAACATGGAATGCCAGACGCTGGGG 4 (0.000122%)

GCGCACGTGAAGCACCGTAAAGGCGCTGCGCGCCTGCGCGCCGTGGATTTCGCTGAGCGGCACGGCTAC
ATCAAGGGCATCGTCAAGGACATCATCCACG 3 (0.000091%)

GCGCCGGGTCTGTGTTCCGCGCGCACGTGAAGCACCGTAAAGGCGCTGCGCGCCTGCGCGCCGTGGATT
TCGCTGAGCGGCACGGCTACATCAAGGGCAT 10 (0.000305%)

GCGCGCACGTGAAGCACCGTAAAGGCGCTGCGCGCCTGCGCGCCGTGGATTTCGCTGAGCGGCACGGCT
ACATCAAGGGCATCGTCAAGGACATCATCCA 3 (0.000091%)

GCGGGAGCTACGGGGCCCAGGGATTGTGTTTAAAGTAGTGCTTCTACCAACATGTCCCGTGGTTCCAGCG
CCGGTTTTGACCGCCACATTACCATTTTTT 3 (0.000091%)

GCGGTCTGCCCCTGGAGGTGGTAGCAAGGTTCCACAGAAAAAAGTAAAACTTGCTGCTGATGAAGATGA
TGACGATGATGATGAAGAGGATGATGATGAA 7 (0.000213%)

GCGGTGGCATCGACAAAAGAACCATTGAAAAATTTGAGAAGGAGGCTGCTGAGATGGGAAAGGGCTCC
TTCAAGTATGCCTGGGTCTTGGATAAACTGAA 15 (0.000457%)

GCGGTGGGTGTCATCAAAGCAGTGGACAAGAAGGCTGCTGGAGCTGGCAAGGTCACCAAGTCTGCCCAG
AAAGCTCAGAAGGCTAAATGAATATTATCCC 27 (0.000822%)

GCGTGAACGTGGTATCACCATTGATATCTCCTTGTGGAAATTTGAGACCAGCAAGTACTATGTGACTATC
ATTGATGCCCCAGGACACAGAGACTTTATC 23 (0.000700%)

GCTACAATGATTTTGGGAATTACAACAATCAGTCTTCAAATTTTGGACCCATGAAGGGAGGAAATTTTGG
AGGCAGAAGCTCTGGCCCCTATGGCGGTGG 44 (0.001340%)

GCTACGGGGCCCAGGGATTGTGTTTAAAGTAGTGCTTCTACCAACATGTCCCGTGGTTCCAGCGCCGGTT
TTGACCGCCACATTACCATTTTTTCACCCG4 (0.000122%)

GCTCCTCCCTGGAGAAGAGCTACGAGCTGCCTGACGGCCAGGTCATCACCATTGGCAATGAGCGGTTCC
GCTGCCCTGAGGCACTCTTCCAGCCTTCCTT 3 (0.000091%)

GCTCCTTCAAGTATGCCTGGGTCTTGGATAAACTGAAAGCTGAGCGTGAACGTGGTATCACCATTGATAT
CTCCTTGTGGAAATTTGAGACCAGCAAGTA 150 (0.004568%)

GCTGAAGCGCAAAAGAAGAAAGATGAGGCAGAGGTCCAAGTAAACCGCTAGCTTGTTGCACCGTGGAG
GCCACAGGAGCAGAAACATGGAATGCCAGACG 3 (0.000091%)

GCTGACTGTGCTGTCCTGATTGTTGCTGCTGGTGTTGGTGAATTTGAAGCTGGTATCTCCAAGAATGGGC
AGACCCGAGAGCATGCCCTTCTGGCTTACA 13 (0.000396%)

GCTGAGCGTGAACGTGGTATCACCATTGATATCTCCTTGTGGAAATTTGAGACCAGCAAGTACTATGTGA
CTATCATTGATGCCCCAGGACACAGAGACT 7 (0.000213%)

GCTGAGGAAAAATAACTAAACATGGGCAAAGGAGATCCTAAGAAGCCGAGAGGCAAAATGTCATCATA
TGCATTTTTTGTGCAAACTTGTCGGGAGGAGC 3 (0.000091%)

GCTGCCATTGTTGATATGGTTCCTGGCAAGCCCATGTGTGTTGAGAGCTTCTCAGACTATCCACCTTTGG
GTCGCTTTGCTGTTCGTGATATGAGACAGA 12 (0.000365%)

GCTGCTGGAGCTGGCAAGGTCACCAAGTCTGCCCAGAAAGCTCAGAAGGCTAAATGAATATTATCCCTA
ATACCTGCCACCCCACTCTTAATCAGTGGTG 12 (0.000365%)

GCTGGAAGATGGCCCTAAATTCTTGAAGTCTGGTGATGCTGCCATTGTTGATATGGTTCCTGGCAAGCCC
ATGTGTGTTGAGAGCTTCTCAGACTATCCA 10 (0.000305%)

GCTGGAGCTGGCAAGGTCACCAAGTCTGCCCAGAAAGCTCAGAAGGCTAAATGAATATTATCCCTAATA
CCTGCCACCCCACTCTTAATCAGTGGTGGAA 22 (0.000670%)

GCTGGCAAGGTCACCAAGTCTGCCCAGAAAGCTCAGAAGGCTAAATGAATATTATCCCTAATACCTGCC
ACCCCACTCTTAATCAGTGGTGGAAGAACGG 5 (0.000152%)

GCTGTCCTGATTGTTGCTGCTGGTGTTGGTGAATTTGAAGCTGGTATCTCCAAGAATGGGCAGACCCGAG
AGCATGCCCTTCTGGCTTACACACTGGGTG 12 (0.000365%)

GCTTCAATGTCAAGAATGTGTCTGTCAAGGATGTTCGTCGTGGCAACGTTGCTGGTGACAGCAAAAATG
ACCCACCAATGGAAGCAGCTGGCTTCACTGC 16 (0.000487%)

GCTTCATTCATTGCCCCCACAATCCTAGGCCTACCCGCCGCAGTACTGATCATTCTATTTCCCCCTCTATT
GATCCCCACCTCCAAATATCTCATCAACA 5 (0.000152%)

GCTTCTCAGACTATCCACCTTTGGGTCGCTTTGCTGTTCGTGATATGAGACAGACAGTTGCGGTGGGTGT
CATCAAAGCAGTGGACAAGAAGGCTGCTGG 43 (0.001309%)

GCTTGATGGTCGAGGCCATCTCCTGGGCCGCCTGGCGGCCATCGTGGCTAAACAGGTACTGCTGGGCCG
GAAGGTGGTGGTCGTACGCTGTGAAGGCATC 1056 (0.032158%)

GGAACTTCGCATCCGCAAACTCTGTCTCAACATCTGTGTTGGGGAGAGTGGAGACAGACTGACGCGAGC
AGCCAAGGTGTTGGAGCAGCTCACAGGGCAG 487 (0.014830%)

GGAAGATGGCCCTAAATTCTTGAAGTCTGGTGATGCTGCCATTGTTGATATGGTTCCTGGCAAGCCCATG
TGTGTTGAGAGCTTCTCAGACTATCCACCT 158 (0.004812%)

GGAAGCAATTTTGGAGGTGGTGGAAGCTACAATGATTTTGGGAATTACAACAATCAGTCTTCAAATTTTG
GACCCATGAAGGGAGGAAATTTTGGAGGCA 10 (0.000305%)

GGAAGCTACAATGATTTTGGGAATTACAACAATCAGTCTTCAAATTTTGGACCCATGAAGGGAGGAAAT
TTTGGAGGCAGAAGCTCTGGCCCCTATGGCG 86 (0.002619%)

GGAAGGGCGCCGGGTCTGTGTTCCGCGCGCACGTGAAGCACCGTAAAGGCGCTGCGCGCCTGCGCGCCG
TGGATTTCGCTGAGCGGCACGGCTACATCAA 20 (0.000609%)

GGAATAATCTCCCATATTGTAACTTACTACTCCGGAAAAAAAGAACCATTTGGATACATAGGTATGGTCT
GAGCTATGATATCAATTGGCTTCCTAGGGT 5 (0.000152%)

GGAATCACCTCCCATTCCGATAAAATCACCTTCCACCCTTACTACACAATCAAAGACGCCCTCGGCTTAC TTCTCTTCATTCTCTCCTTAATGACATTAA 4 (0.000122%)

GGACAAGAAGGCTGCTGGAGCTGGCAAGGTCACCAAGTCTGCCCAGAAAGCTCAGAAGGCTAAATGAA TATTATCCCTAATACCTGCCACCCCACTCTTA 72 (0.002193%)

GGACACAGAGACTTTATCAAAAACATGATTACAGGGACATCTCAGGCTGACTGTGCTGTCCTGATTGTTG CTGCTGGTGTTGGTGAATTTGAAGCTGGTA 15 (0.000457%)

GGACACGTAGATTCGGGCAAGTCCACCACTACTGGCCATCTGATCTATAAATGCGGTGGCATCGACAAA AGAACCATTGAAAAATTTGAGAAGGAGGCTG 12 (0.000365%)

GGACAGAGGAAGGGCGCCGGGTCTGTGTTCCGCGCGCACGTGAAGCACCGTAAAGGCGCTGCGCGCCTG CGCGCCGTGGATTTCGCTGAGCGGCACGGCT 24 (0.000731%)

GGACATCTCAGGCTGACTGTGCTGTCCTGATTGTTGCTGCTGGTGTTGGTGAATTTGAAGCTGGTATCTCC AAGAATGGGCAGACCCGAGAGCATGCCCT 32 (0.000974%)

GGACGGGCACTGGGCGACTCTGTGCCTCGCTGAGGAAAAATAACTAAACATGGGCAAAGGAGATCCTA AGAAGCCGAGAGGCAAAATGTCATCATATGCA 24 (0.000731%)

GGACTCCTGCCTCACTCATTTACACCAACCACCCAACTATCTATAAACCTAGCCATGGCCATCCCCTTAT GAGCGGGCGCAGTGATTATAGGCTTTCGCT 7 (0.000213%)

GGAGACCCCATTCTATACCAACACCTATTCTGATTTTTCGGTCACCCTGAAGTTTATATTCTTATCCTACC AGGCTTCGGAATAATCTCCCATATTGTAA 69 (0.002101%)

GGAGACTGGTGTTCTCAAACCCGGTATGGTGGTCACCTTTGCTCCAGTCAACGTTACAACGGAAGTAAA ATCTGTCGAAATGCACCATGAAGCTTTGAGT 43 (0.001309%)

GGAGAGGCACCCGATATATGTTCTCTAGGCCTTTTAGAAAACATGGAGTTGTTCCTTTGGCCACATATAT GCGAATCTATAAGAAAGGTGATATTGTAGA 11 (0.000335%)

GGAGCCAAGTGCTAACATGCCTTGGTTCAAGGGATGGAAAGTCACCCGTAAGGATGGCAATGCCAGTGG AACCACGCTGCTTGAGGCTCTGGACTGCATC 4 (0.000122%)

GGAGCTGGCAAGGTCACCAAGTCTGCCCAGAAAGCTCAGAAGGCTAAATGAATATTATCCCTAATACCT GCCACCCCACTCTTAATCAGTGGTGGAAGAA 9 (0.000274%)

GGAGGAGACCCCATTCTATACCAACACCTATTCTGATTTTTCGGTCACCCTGAAGTTTATATTCTTATCCT ACCAGGCTTCGGAATAATCTCCCATATTG 47 (0.001431%)

GGATAAACTGAAAGCTGAGCGTGAACGTGGTATCACCATTGATATCTCCTTGTGGAAATTTGAGACCAG CAAGTACTATGTGACTATCATTGATGCCCCA 15 (0.000457%)

GGATAATCCTATTTATTACCTCAGAAGTTTTTTCTTCGCAGGATTTTTCTGAGCCTTTTACCACTCCAGC CTAGCCCCTACCCCCCAATTAGGAGGGCA 85 (0.002588%)

GGATCTCCTTCATCCCTCTCCAGAAGAGGAGAAGAGGAAACACAAGAAGAAACGCCTGGTGCAGAGCC CCAATTCCTACTTCATGGATGTGAAATGCCCA 3 (0.000091%)

GGATGGAAAGTCACCCGTAAGGATGGCAATGCCAGTGGAACCACGCTGCTTGAGGCTCTGGACTGCATC CTACCACCAACTCGTCCAACTGACAAGCCCT 4 (0.000122%)

GGATTGTGTTTAAAGTAGTGCTTCTACCAACATGTCCCGTGGTTCCAGCGCCGGTTTTGACCGCCACATT ACCATTTTTTCACCCGAGGGTCGGCTCTAC 4 (0.000122%)

GGCAACCTTCTAGGTAACGACCACATCTACAACGTTATCGTCACAGCCCATGCATTTGTAATAATCTTCT

TCATAGTAATACCCATCATAATCGGAGGCT 4 (0.000122%)

GGCAAGCCCATGTGTGTTGAGAGCTTCTCAGACTATCCACCTTTGGGTCGCTTTGCTGTTCGTGATATGA
GACAGACAGTTGCGGTGGGTGTCATCAAAG 31 (0.000944%)

GGCAAGTCCACCACTACTGGCCATCTGATCTATAAATGCGGTGGCATCGACAAAAGAACCATTGAAAAA
TTTGAGAAGGAGGCTGCTGAGATGGGAAAGG 74 (0.002253%)

GGCACAGCTCTAAGCCTCCTTATTCGAGCCGAGCTGGGCCAGCCAGGCAACCTTCTAGGTAACGACCAC
ATCTACAACGTTATCGTCACAGCCCATGCAT 7 (0.000213%)

GGCACCCGATATATGTTCTCTAGGCCTTTTAGAAAACATGGAGTTGTTCCTTTGGCCACATATATGCGAA
TCTATAAGAAAGGTGATATTGTAGACATCA 5 (0.000152%)

GGCAGACCCGAGAGCATGCCCTTCTGGCTTACACACTGGGTGTGAAACAACTAATTGTCGGTGTTAACA
AAATGGATTCCACTGAGCCACCCTACAGCCA 20 (0.000609%)

GGCATCGACAAAAGAACCATTGAAAAATTTGAGAAGGAGGCTGCTGAGATGGGAAAGGGCTCCTTCAA
GTATGCCTGGGTCTTGGATAAACTGAAAGCTG 6 (0.000183%)

GGCCATCTCCTGGGCCGCCTGGCGGCCATCGTGGCTAAACAGGTACTGCTGGGCCGGAAGGTGGTGGTC
GTACGCTGTGAAGGCATCAACATTTCTGGCA 6 (0.000183%)

GGCCCTCTCAGCCCTCCTAATGACCTCCGGCCTAGCCATGTGATTTCACTTCCACTCCATAACGCTCCTCA
TACTAGGCCTACTAACCAACACACTAACC 5 (0.000152%)

GGCGACTCTGTGCCTCGCTGAGGAAAAATAACTAAACATGGGCAAAGGAGATCCTAAGAAGCCGAGAG
GCAAAATGTCATCATATGCATTTTTGTGCAA 16 (0.000487%)

GGCGCCGGGTCTGTGTTCCGCGCGCACGTGAAGCACCGTAAAGGCGCTGCGCGCCTGCGCGCCGTGGAT
TTCGCTGAGCGGCACGGCTACATCAAGGGCA 8 (0.000244%)

GGCGCTGCGCGCCTGCGCGCCGTGGATTTCGCTGAGCGGCACGGCTACATCAAGGGCATCGTCAAGGAC
ATCATCCACGACCCGGGCCGCGGCGCGCCCC 4 (0.000122%)

GGCGTTTCCCCGCATAAACAACATAAGCTTCTGACTCTTACCTCCCTCTCTCCTACTCCTGCTCGCATCTG
CTATAGTGGAGGCCGGAGCAGGAACAGGT 32 (0.000974%)

GGCTCCTTCAAGTATGCCTGGGTCTTGGATAAACTGAAAGCTGAGCGTGAACGTGGTATCACCATTGATA
TCTCCTTGTGGAAATTTGAGACCAGCAAGT 110 (0.003350%)

GGCTGAAGCGCAAAAGAAGAAAGATGAGGCAGAGGTCCAAGTAAACCGCTAGCTTGTTGCACCGTGGA
GGCCACAGGAGCAGAAACATGGAATGCCAGAC 9 (0.000274%)

GGCTGACTGTGCTGTCCTGATTGTTGCTGCTGGTGTTGGTGAATTTGAAGCTGGTATCTCCAAGAATGGG
CAGACCCGAGAGCATGCCCTTCTGGCTTAC 25 (0.000761%)

GGCTGCTGGAGCTGGCAAGGTCACCAAGTCTGCCCAGAAAGCTCAGAAGGCTAAATGAATATTATCCCT
AATACCTGCCACCCCACTCTTAATCAGTGGT 71 (0.002162%)

GGCTGCTTCCAGCTCCTCCCTGGAGAAGAGCTACGAGCTGCCTGACGGCCAGGTCATCACCATTGGCAAT
GAGCGGTTCCGCTGCCCTGAGGCACTCTTC 15 (0.000457%)

GGCTTACACACTGGGTGTGAAACAACTAATTGTCGGTGTTAACAAAATGGATTCCACTGAGCCACCCTAC
AGCCAGAAGAGATATGAGGAAATTGTTAAG 13 (0.000396%)

GGCTTCAATGTCAAGAATGTGTCTGTCAAGGATGTTCGTCGTGGCAACGTTGCTGGTGACAGCAAAAAT
GACCCACCAATGGAAGCAGCTGGCTTCACTG 13 (0.000396%)

GGGAATTACAACAATCAGTCTTCAAATTTTGGACCCATGAAGGGAGGAAATTTTGGAGGCAGAAGCTCT
GGCCCCTATGGCGGTGGAGGCCAATACTTTG 22 (0.000670%)

GGGACATCTCAGGCTGACTGTGCTGTCCTGATTGTTGCTGCTGGTGTTGGTGAATTTGAAGCTGGTATCT
CCAAGAATGGGCAGACCCGAGAGCATGCCC 163 (0.004964%)

GGGAGCTACGGGGCCCAGGGATTGTGTTTAAAGTAGTGCTTCTACCAACATGTCCCGTGGTTCCAGCGCC
GGTTTTGACCGCCACATTACCATTTTTTCA 3 (0.000091%)

GGGATAATCCTATTTATTACCTCAGAAGTTTTTTTCTTCGCAGGATTTTTCTGAGCCTTTTACCACTCCAG
CCTAGCCCCTACCCCCCAATTAGGAGGGC 5 (0.000152%)

GGGATGGAAAGTCACCCGTAAGGATGGCAATGCCAGTGGAACCACGCTGCTTGAGGCTCTGGACTGCAT
CCTACCACCAACTCGTCCAACTGACAAGCCC 11 (0.000335%)

GGGCAAGTCCACCACTACTGGCCATCTGATCTATAAATGCGGTGGCATCGACAAAAGAACCATTGAAAA
ATTTGAGAAGGAGGCTGCTGAGATGGGAAAG 21 (0.000640%)

GGGCACTGGGCGACTCTGTGCCTCGCTGAGGAAAAATAACTAAACATGGGCAAAGGAGATCCTAAGAA
GCCGAGAGGCAAAATGTCATCATATGCATTTT 16 (0.000487%)

GGGCAGACCCGAGAGCATGCCCTTCTGGCTTACACACTGGGTGTGAAACAACTAATTGTCGGTGTTAAC
AAAATGGATTCCACTGAGCCACCCTACAGCC 16 (0.000487%)

GGGCCCTCTCAGCCCTCCTAATGACCTCCGGCCTAGCCATGTGATTTCACTTCCACTCCATAACGCTCCTC
ATACTAGGCCTACTAACCAACACACTAAC 15 (0.000457%)

GGGCGACTCTGTGCCTCGCTGAGGAAAAATAACTAAACATGGGCAAAGGAGATCCTAAGAAGCCGAGA
GGCAAAATGTCATCATATGCATTTTTGTGCA 9 (0.000274%)

GGGCGCCGGGTCTGTGTTCCGCGCGCACGTGAAGCACCGTAAAGGCGCTGCGCGCCTGCGCGCCGTGGA
TTTCGCTGAGCGGCACGGCTACATCAAGGGC 3 (0.000091%)

GGGCTCCTTCAAGTATGCCTGGGTCTTGGATAAACTGAAAGCTGAGCGTGAACGTGGTATCACCATTGAT
ATCTCCTTGTGGAAATTTGAGACCAGCAAG 21 (0.000640%)

GGGCTTCAATGTCAAGAATGTGTCTGTCAAGGATGTTCGTCGTGGCAACGTTGCTGGTGACAGCAAAAA
TGACCCACCAATGGAAGCAGCTGGCTTCACT 43 (0.001309%)

GGGGCCCTCTCAGCCCTCCTAATGACCTCCGGCCTAGCCATGTGATTTCACTTCCACTCCATAACGCTCCT
CATACTAGGCCTACTAACCAACACACTAA 26 (0.000792%)

GGGGGGGGGGGGGGGGGGGGGGGGGGGGGGGGGGGGGGGGGGGGGGGGGGGGGGGGGGGGGGGGGG
GGGGGGGGGGGGGGGGGGGGGGGGGGGGGGGGG 5839 (0.177812%)

GGGGGGGGGGGGGGGGGGGGGGGGGGGGGGGGGGGGGGGGGGGGGGGGGGGGGGGGGGGGGGGGGG
GGGGGGGGGGGGGGGGGGGGGGGGGGGGGGGGGGGGGGGGGGGGGGGGGGGGGGGGGGGGGGGGGG
GGGGGGGGGGGGGGGGG 4812 (0.218341%)

GGGTCTTGGATAAACTGAAAGCTGAGCGTGAACGTGGTATCACCATTGATATCTCCTTGTGGAAATTTGA
GACCAGCAAGTACTATGTGACTATCATTGA 11 (0.000335%)

GGGTGTCATCAAAGCAGTGGACAAGAAGGCTGCTGGAGCTGGCAAGGTCACCAAGTCTGCCCAGAAAG
CTCAGAAGGCTAAATGAATATTATCCCTAATA 5 (0.000152%)

GGGTGTGAAACAACTAATTGTCGGTGTTAACAAAATGGATTCCACTGAGCCACCCTACAGCCAGAAGAG
ATATGAGGAAATTGTTAAGGAAGTCAGCACT 3 (0.000091%)

GGTAAAAAGCTGGAAGATGGCCCTAAATTCTTGAAGTCTGGTGATGCTGCCATTGTTGATATGGTTCCTG

GCAAGCCCATGTGTGTTGAGAGCTTCTCAG 7 (0.000213%)

GGTAATGATGGAAGCAATTTTGGAGGTGGTGGAAGCTACAATGATTTTGGGAATTACAACAATCAGTCT
TCAAATTTTGGACCCATGAAGGGAGGAAATT 4 (0.000122%)

GGTATCACCATTGATATCTCCTTGTGGAAATTTGAGACCAGCAAGTACTATGTGACTATCATTGATGCCC
CAGGACACAGAGACTTTATCAAAAACATGA 24 (0.000731%)

GGTATGGTGGTCACCTTTGCTCCAGTCAACGTTACAACGGAAGTAAAATCTGTCGAAATGCACCATGAA
GCTTTGAGTGAAGCTCTTCCTGGGGACAATG 9 (0.000274%)

GGTCAACGATCCCTCCCTTACCATCAAATCAATTGGCCACCAATGGTACTGAACCTACGAGTACACCGAC
TACGGCGGACTAATCTTCAACTCCTACATA 15 (0.000457%)

GGTCACCTTTGCTCCAGTCAACGTTACAACGGAAGTAAAATCTGTCGAAATGCACCATGAAGCTTTGAGT
GAAGCTCTTCCTGGGGACAATGTGGGCTTC 63 (0.001919%)

GGTCCCTCGGCTTCCTGCCTCGGAAGCGCAGCAGCAGGCATCGTGGGAAGGTGAAGAGCTTCCCTAAGG
ATGACCCGTCCAAGCCGGTCCACCTCACAGC 10 (0.000305%)

GGTCGAGGCCATCTCCTGGGCCGCCTGGCGGCCATCGTGGCTAAACAGGTACTGCTGGGCCGGAAGGTG
GTGGTCGTACGCTGTGAAGGCATCAACATTT 6 (0.000183%)

GGTCTGTGTTCCGCGCGCACGTGAAGCACCGTAAAGGCGCTGCGCGCCTGCGCGCCGTGGATTTCGCTG
AGCGGCACGGCTACATCAAGGGCATCGTCAA 11 (0.000335%)

GGTCTTGGATAAACTGAAAGCTGAGCGTGAACGTGGTATCACCATTGATATCTCCTTGTGGAAATTTGAG
ACCAGCAAGTACTATGTGACTATCATTGAT 3 (0.000091%)

GGTGATGCTGCCATTGTTGATATGGTTCCTGGCAAGCCCATGTGTGTTGAGAGCTTCTCAGACTATCCAC
CTTTGGGTCGCTTTGCTGTTCGTGATATGA 17 (0.000518%)

GGTGCCCCCGATATGGCGTTTCCCCGCATAAACAACATAAGCTTCTGACTCTTACCTCCCTCTCTCCTACT
CCTGCTCGCATCTGCTATAGTGGAGGCCG 4 (0.000122%)

GGTGCCTTGATGGCCTCCTCTCCTCAAGACATCAAATTTCAAGATTTGGTCGTCTTCATTTTGGAGAAGA
AAATGGGAACCACCCGCAGAGCGTTCCTCA 101 (0.003076%)

GGTGGAAGCTACAATGATTTTGGGAATTACAACAATCAGTCTTCAAATTTTGGACCCATGAAGGGAGGA
AATTTTGGAGGCAGAAGCTCTGGCCCCTATG 3 (0.000091%)

GGTGGGTGTCATCAAAGCAGTGGACAAGAAGGCTGCTGGAGCTGGCAAGGTCACCAAGTCTGCCCAGA
AAGCTCAGAAGGCTAAATGAATATTATCCCTA 13 (0.000396%)

GGTGGTCACCTTTGCTCCAGTCAACGTTACAACGGAAGTAAAATCTGTCGAAATGCACCATGAAGCTTTG
AGTGAAGCTCTTCCTGGGGACAATGTGGGC 24 (0.000731%)

GGTGGTGGAAGCTACAATGATTTTGGGAATTACAACAATCAGTCTTCAAATTTTGGACCCATGAAGGGA
GGAAATTTTGGAGGCAGAAGCTCTGGCCCCT 5 (0.000152%)

GGTGTCATCAAAGCAGTGGACAAGAAGGCTGCTGGAGCTGGCAAGGTCACCAAGTCTGCCCAGAAAGCT
CAGAAGGCTAAATGAATATTATCCCTAATAC 11 (0.000335%)

GGTGTCGTGAAAACTACCCCTAAAAGCCAAAATGGGAAAGGAAAAGACTCATATCAACATTGTCGTCAT
TGGACACGTAGATTCGGGCAAGTCCACCACT 41 (0.001249%)

GGTGTTCTCAAACCCGGTATGGTGGTCACCTTTGCTCCAGTCAACGTTACAACGGAAGTAAAATCTGTCG
AAATGCACCATGAAGCTTTGAGTGAAGCTC 11 (0.000335%)

GGTTACTTGCTTCGTCTGTTCTGTGTTGGTTTACTAAAAACGCAACAATCAGATACGGAAGACCTCTT
ATGCTCAGCACCAACAGGTCCGCCAAATCC 4 (0.000122%)

GGTTCCTGGCAAGCCCATGTGTGTTGAGAGCTTCTCAGACTATCCACCTTTGGGTCGCTTTGCTGTTCGTG
ATATGAGACAGACAGTTGCGGTGGGTGTC 4 (0.000122%)

GTAAAAAGCTGGAAGATGGCCCTAAATTCTTGAAGTCTGGTGATGCTGCCATTGTTGATATGGTTCCTGG
CAAGCCCATGTGTGTTGAGAGCTTCTCAGA 40 (0.001218%)

GTAAAACCCAGCCCATGACCCCTAACAGGGGCCCTCTCAGCCCTCCTAATGACCTCCGGCCTAGCCATGT
GATTTCACTTCCACTCCATAACGCTCCTCA 824 (0.025093%)

GTAAAGGCGCTGCGCGCCTGCGCGCCGTGGATTTCGCTGAGCGGCACGGCTACATCAAGGGCATCGTCA
AGGACATCATCCACGACCCGGGCCGCGGCGC 9 (0.000274%)

GTAACTTACTACTCCGGAAAAAAAGAACCATTTGGATACATAGGTATGGTCTGAGCTATGATATCAATTG
GCTTCCTAGGGTTTATCGTGTGAGCACACC 28 (0.000853%)

GTAATGATGGAAGCAATTTTGGAGGTGGTGGAAGCTACAATGATTTTGGGAATTACAACAATCAGTCTT
CAAATTTTGGACCCATGAAGGGAGGAAATTT 13 (0.000396%)

GTACAAGGTAGTGGGTCGCTGCCTGCCCACCCCCAAATGCCACACGCCGCCCCTCTACCGCATGCGAATC
TTTGCGCCTAATCATGTCGTCGCCAAGTCC 584 (0.017784%)

GTACTATGTGACTATCATTGATGCCCCAGGACACAGAGACTTTATCAAAAACATGATTACAGGGACATCT
CAGGCTGACTGTGCTGTCCTGATTGTTGCT 41 (0.001249%)

GTAGATTCGGGCAAGTCCACCACTACTGGCCATCTGATCTATAAATGCGGTGGCATCGACAAAAGAACC
ATTGAAAAATTTGAGAAGGAGGCTGCTGAGA 65 (0.001979%)

GTAGGCTCCCTTCCCCTACTCATCGCACTGATTTACACTCACAACACCCTAGGCTCACTAAACATTCTACT
ACTCACTCTCACTGCCCAAGAACTATCAA 6 (0.000183%)

GTATCACCATTGATATCTCCTTGTGGAAATTTGAGACCAGCAAGTACTATGTGACTATCATTGATGCCCC
AGGACACAGAGACTTTATCAAAAACATGAT 13 (0.000396%)

GTATGCCTGGGTCTTGGATAAACTGAAAGCTGAGCGTGAACGTGGTATCACCATTGATATCTCCTTGTGG
AAATTTGAGACCAGCAAGTACTATGTGACT 14 (0.000426%)

GTATGGTGGTCACCTTTGCTCCAGTCAACGTTACAACGGAAGTAAAATCTGTCGAAATGCACCATGAAG
CTTTGAGTGAAGCTCTTCCTGGGGACAATGT 7 (0.000213%)

GTCAAGAATGTGTCTGTCAAGGATGTTCGTCGTGGCAACGTTGCTGGTGACAGCAAAAATGACCCACCA
ATGGAAGCAGCTGGCTTCACTGCTCAGGTGA 9 (0.000274%)

GTCAAGACTACCGATGGTTACTTGCTTCGTCTGTTCTGTGTTGGTTTACTAAAAACGCAACAATCAGA
TACGGAAGACCTCTTATGCTCAGCACCAAC 10 (0.000305%)

GTCAAGGATGTTCGTCGTGGCAACGTTGCTGGTGACAGCAAAAATGACCCACCAATGGAAGCAGCTGGC
TTCACTGCTCAGGTGATTATCCTGAACCATC 16 (0.000487%)

GTCACCTTTGCTCCAGTCAACGTTACAACGGAAGTAAAATCTGTCGAAATGCACCATGAAGCTTTGAGTG
AAGCTCTTCCTGGGGACAATGTGGGCTTCA 89 (0.002710%)

GTCATCAAAGCAGTGGACAAGAAGGCTGCTGGAGCTGGCAAGGTCACCAAGTCTGCCCAGAAAGCTCA
GAAGGCTAAATGAATATTATCCCTAATACCTG 15 (0.000457%)

GTCATTGGACACGTAGATTCGGGCAAGTCCACCACTACTGGCCATCTGATCTATAAATGCGGTGGCATCG
ACAAAAGAACCATTGAAAAATTTGAGAAGG 4 (0.000122%)

GTCCAACTGACAAGCCCTTGCGCCTGCCTCTCCAGGATGTCTACAAAATTGGTGGTATTGGTACTGTTCC
TGTTGGCCGAGTGGAGACTGGTGTTCTCAA 532 (0.016201%)

GTCCACCACTACTGGCCATCTGATCTATAAATGCGGTGGCATCGACAAAAGAACCATTGAAAAATTTGA
GAAGGAGGCTGCTGAGATGGGAAAGGGCTCC 32 (0.000974%)

GTCCTAGCTGCTGGCATCACTATACTACTAACAGACCGCAACCTCAACACCACCTTCTTCGACCCCGCCG
GAGGAGGAGACCCCATTCTATACCAACACC 3 (0.000091%)

GTCCTCATCGCCCTCCCATCCCTACGCATCCTTTACATAACAGACGAGGTCAACGATCCCTCCCTTACCAT
CAAATCAATTGGCCACCAATGGTACTGAA 37 (0.001127%)

GTCCTGATTGTTGCTGCTGGTGTTGGTGAATTTGAAGCTGGTATCTCCAAGAATGGGCAGACCCGAGAGC
ATGCCCTTCTGGCTTACACACTGGGTGTGA 7 (0.000213%)

GTCGAGGCCATCTCCTGGGCCGCCTGGCGGCCATCGTGGCTAAACAGGTACTGCTGGGCCGGAAGGTGG
TGGTCGTACGCTGTGAAGGCATCAACATTTC 47 (0.001431%)

GTCGTCATTGGACACGTAGATTCGGGCAAGTCCACCACTACTGGCCATCTGATCTATAAATGCGGTGGCA
TCGACAAAAGAACCATTGAAAAATTTGAGA 8 (0.000244%)

GTCGTGAAAACTACCCCTAAAAGCCAAAATGGGAAAGGAAAAGACTCATATCAACATTGTCGTCATTGG
ACACGTAGATTCGGGCAAGTCCACCACTACT 24 (0.000731%)

GTCTGAACTATCCTGCCCGCCATCATCCTAGTCCTCATCGCCCTCCCATCCCTACGCATCCTTTACATAAC
AGACGAGGTCAACGATCCCTCCCTTACCA 86 (0.002619%)

GTCTGGTGATGCTGCCATTGTTGATATGGTTCCTGGCAAGCCCATGTGTGTTGAGAGCTTCTCAGACTAT
CCACCTTTGGGTCGCTTTGCTGTTCGTGAT 48 (0.001462%)

GTCTGTCAAGGATGTTCGTCGTGGCAACGTTGCTGGTGACAGCAAAAATGACCCACCAATGGAAGCAGC
TGGCTTCACTGCTCAGGTGATTATCCTGAAC 10 (0.000305%)

GTCTGTGTTCCGCGCGCACGTGAAGCACCGTAAAGGCGCTGCGCGCCTGCGCGCCGTGGATTTCGCTGA
GCGGCACGGCTACATCAAGGGCATCGTCAAG 13 (0.000396%)

GTCTGTTCTGTGTTGGTTTTACTAAAAAACGCAACAATCAGATACGGAAGACCTCTTATGCTCAGCACCA
ACAGGTCCGCCAAATCCGGAAGAAGATGAT 5 (0.000152%)

GTCTTGGATAAACTGAAAGCTGAGCGTGAACGTGGTATCACCATTGATATCTCCTTGTGGAAATTTGAGA
CCAGCAAGTACTATGTGACTATCATTGATG 8 (0.000244%)

GTGAAAACTACCCCTAAAAGCCAAAATGGGAAAGGAAAAGACTCATATCAACATTGTCGTCATTGGACA
CGTAGATTCGGGCAAGTCCACCACTACTGGCCATCTGATCTATAAATGCGGTGGCATCGACAAAAGAAC
CATTGAAAAAT 276 (0.012523%)

GTGAAACAACTAATTGTCGGTGTTAACAAAATGGATTCCACTGAGCCACCCTACAGCCAGAAGAGATAT
GAGGAAATTGTTAAGGAAGTCAGCACTTACA 7 (0.000213%)

GTGAACGTGGTATCACCATTGATATCTCCTTGTGGAAATTTGAGACCAGCAAGTACTATGTGACTATCAT
TGATGCCCCAGGACACAGAGACTTTATCAA 20 (0.000609%)

GTGACTATCATTGATGCCCCAGGACACAGAGACTTTATCAAAAACATGATTACAGGGACATCTCAGGCT
GACTGTGCTGTCCTGATTGTTGCTGCTGGTG 21 (0.000640%)

GTGATATGAGACAGACAGTTGCGGTGGGTGTCATCAAAGCAGTGGACAAGAAGGCTGCTGGAGCTGGC
AAGGTCACCAAGTCTGCCCAGAAAGCTCAGAA 31 (0.000944%)

GTGATGCTGCCATTGTTGATATGGTTCCTGGCAAGCCCATGTGTGTTGAGAGCTTCTCAGACTATCCACC
TTTGGGTCGCTTTGCTGTTCGTGATATGAG 7 (0.000213%)

GTGCCCCCGATATGGCGTTTCCCCGCATAAACAACATAAGCTTCTGACTCTTACCTCCCTCTCTCCTACTC
CTGCTCGCATCTGCTATAGTGGAGGCCGG 3 (0.000091%)

GTGCCTCGCTGAGGAAAAATAACTAAACATGGGCAAAGGAGATCCTAAGAAGCCGAGAGGCAAAATGT
CATCATATGCATTTTTTGTGCAAACTTGTCGG 6 (0.000183%)

GTGCCTGGTGCGGGAGCTACGGGGCCCAGGGATTGTGTTTAAAGTAGTGCTTCTACCAACATGTCCCGTG
GTTCCAGCGCCGGTTTTGACCGCCACATTA 41 (0.001249%)

GTGCTGTCCTGATTGTTGCTGCTGGTGTTGGTGAATTTGAAGCTGGTATCTCCAAGAATGGGCAGACCCG
AGAGCATGCCCTTCTGGCTTACACACTGGG 22 (0.000670%)

GTGGAAATTTGAGACCAGCAAGTACTATGTGACTATCATTGATGCCCCAGGACACAGAGACTTTATCAA
AAACATGATTACAGGGACATCTCAGGCTGAC 7 (0.000213%)

GTGGAAGCTACAATGATTTTGGGAATTACAACAATCAGTCTTCAAATTTTGGACCCATGAAGGGAGGAA
ATTTTGGAGGCAGAAGCTCTGGCCCCTATGG 6 (0.000183%)

GTGGACAAGAAGGCTGCTGGAGCTGGCAAGGTCACCAAGTCTGCCCAGAAAGCTCAGAAGGCTAAATG
AATATTATCCCTAATACCTGCCACCCCACTCT 24 (0.000731%)

GTGGACAGAGGAAGGGCGCCGGGTCTGTGTTCCGCGCGCACGTGAAGCACCGTAAAGGCGCTGCGCGCC
TGCGCGCCGTGGATTTCGCTGAGCGGCACGG 8 (0.000244%)

GTGGAGACTGGTGTTCTCAAACCCGGTATGGTGGTCACCTTTGCTCCAGTCAACGTTACAACGGAAGTAA
AATCTGTCGAAATGCACCATGAAGCTTTGA 20 (0.000609%)

GTGGCATCGACAAAAGAACCATTGAAAAATTTGAGAAGGAGGCTGCTGAGATGGGAAAGGGCTCCTTC
AAGTATGCCTGGGTCTTGGATAAACTGAAAGC 7 (0.000213%)

GTGGGAGACAGCAGCACCAGCGGTGGCAGAGACCCCAGACATCAAGCTCTTTGGGAAGTGGAGCACCG
ATGATGTGCAGATCAATGACATTTCCCTGCAG 14 (0.000426%)

GTGGGCTTCAATGTCAAGAATGTGTCTGTCAAGGATGTTCGTCGTGGCAACGTTGCTGGTGACAGCAAA
AATGACCCACCAATGGAAGCAGCTGGCTTCA 818 (0.024910%)

GTGGGTGTCATCAAAGCAGTGGACAAGAAGGCTGCTGGAGCTGGCAAGGTCACCAAGTCTGCCCAGAA
AGCTCAGAAGGCTAAATGAATATTATCCCTAA 19 (0.000579%)

GTGGTATCACCATTGATATCTCCTTGTGGAAATTTGAGACCAGCAAGTACTATGTGACTATCATTGATGC
CCCAGGACACAGAGACTTTATCAAAAACAT 24 (0.000731%)

GTGGTCACCTTTGCTCCAGTCAACGTTACAACGGAAGTAAAATCTGTCGAAATGCACCATGAAGCTTTGA
GTGAAGCTCTTCCTGGGGACAATGTGGGCT 58 (0.001766%)

GTGTCATCAAAGCAGTGGACAAGAAGGCTGCTGGAGCTGGCAAGGTCACCAAGTCTGCCCAGAAAGCTC
AGAAGGCTAAATGAATATTATCCCTAATACC 66 (0.002010%)

GTGTCGTGAAAACTACCCCTAAAAGCCAAAATGGGAAAGGAAAAGACTCATATCAACATTGTCGTCATT
GGACACGTAGATTCGGGCAAGTCCACCACTA 32 (0.000974%)

GTGTCTGTCAAGGATGTTCGTCGTGGCAACGTTGCTGGTGACAGCAAAAATGACCCACCAATGGAAGCA
GCTGGCTTCACTGCTCAGGTGATTATCCTGA 23 (0.000700%)

GTGTGAAACAACTAATTGTCGGTGTTAACAAAATGGATTCCACTGAGCCACCCTACAGCCAGAAGAGAT
ATGAGGAAATTGTTAAGGAAGTCAGCACTTA 12 (0.000365%)

GTGTGTTGAGAGCTTCTCAGACTATCCACCTTTGGGTCGCTTTGCTGTTCGTGATATGAGACAGACAGTT
GCGGTGGGTGTCATCAAAGCAGTGGACAAG 13 (0.000396%)

GTGTTCCGCGCGCACGTGAAGCACCGTAAAGGCGCTGCGCGCCTGCGCGCCGTGGATTTCGCTGAGCGG
CACGGCTACATCAAGGGCATCGTCAAGGACA 9 (0.000274%)

GTGTTCTCAAACCCGGTATGGTGGTCACCTTTGCTCCAGTCAACGTTACAACGGAAGTAAAATCTGTCGA
AATGCACCATGAAGCTTTGAGTGAAGCTCT 33 (0.001005%)

GTTACTTGCTTCGTCTGTTCTGTGTTGGTTTTACTAAAAAACGCAACAATCAGATACGGAAGACCTCTTAT
GCTCAGCACCAACAGGTCCGCCAAATCCG 5 (0.000152%)

GTTCCCCTAATAATCGGTGCCCCCGATATGGCGTTTCCCCGCATAAACAACATAAGCTTCTGACTCTTAC
CTCCCTCTCTCCTACTCCTGCTCGCATCTG 16 (0.000487%)

GTTCCGCGCGCACGTGAAGCACCGTAAAGGCGCTGCGCGCCTGCGCGCCGTGGATTTCGCTGAGCGGCA
CGGCTACATCAAGGGCATCGTCAAGGACATC 9 (0.000274%)

GTTCCTGGCAAGCCCATGTGTGTTGAGAGCTTCTCAGACTATCCACCTTTGGGTCGCTTTGCTGTTCGTGA
TATGAGACAGACAGTTGCGGTGGGTGTCA 41 (0.001249%)

GTTCGCTTCATTCATTGCCCCCACAATCCTAGGCCTACCCGCCGCAGTACTGATCATTCTATTTCCCCCTC
TATTGATCCCCACCTCCAAATATCTCATC 27 (0.000822%)

GTTCTCAAACCCGGTATGGTGGTCACCTTTGCTCCAGTCAACGTTACAACGGAAGTAAAATCTGTCGAAA
TGCACCATGAAGCTTTGAGTGAAGCTCTTC 29 (0.000883%)

GTTCTCCGCTCCCAGACATGGGTCCCTCGGCTTCCTGCCTCGGAAGCGCAGCAGCAGGCATCGTGGGAA
GGTGAAGAGCTTCCCTAAGGATGACCCGTCC 5 (0.000152%)

GTTCTCTAGGCCTTTTAGAAAACATGGAGTTGTTCCTTTGGCCACATATATGCGAATCTATAAGAAAGGT
GATATTGTAGACATCAAGGGAATGGGTACT 20 (0.000609%)

GTTCTGGTAAAAAGCTGGAAGATGGCCCTAAATTCTTGAAGTCTGGTGATGCTGCCATTGTTGATATGGT
TCCTGGCAAGCCCATGTGTGTTGAGAGCTT 48 (0.001462%)

GTTCTGTGTTGGTTTTACTAAAAAACGCAACAATCAGATACGGAAGACCTCTTATGCTCAGCACCAACAG
GTCCGCCAAATCCGGAAGAAGATGATGGAA 4 (0.000122%)

GTTGAGAGCTTCTCAGACTATCCACCTTTGGGTCGCTTTGCTGTTCGTGATATGAGACAGACAGTTGCGG
TGGGTGTCATCAAAGCAGTGGACAAGAAGG 32 (0.000974%)

GTTGATATGGTTCCTGGCAAGCCCATGTGTGTTGAGAGCTTCTCAGACTATCCACCTTTGGGTCGCTTTGC
TGTTCGTGATATGAGACAGACAGTTGCGG 41 (0.001249%)

GTTGATGTCAAGACTACCGATGGTTACTTGCTTCGTCTGTTCTGTGTTGGTTTTACTAAAAAACGCAACA
ATCAGATACGGAAGACCTCTTATGCTCAGC 19 (0.000579%)

GTTGCGGTGGGTGTCATCAAAGCAGTGGACAAGAAGGCTGCTGGAGCTGGCAAGGTCACCAAGTCTGCC
CAGAAAGCTCAGAAGGCTAAATGAATATTAT 49 (0.001492%)

GTTGGCCGAGTGGAGACTGGTGTTCTCAAACCCGGTATGGTGGTCACCTTTGCTCCAGTCAACGTTACAA
CGGAAGTAAAATCTGTCGAAATGCACCATG 827 (0.025184%)

GTTGTAGGCCCCTACGGGCTACTACAACCCTTCGCTGACGCCATAAAACTCTTCACCAAAGAGCCCCTAA
AACCCGCCACATCTACCATCACCCTCTACA 12 (0.000365%)

GTTTCCCCGCATAAACAACATAAGCTTCTGACTCTTACCTCCCTCTCTCCTACTCCTGCTCGCATCTGCTA

TAGTGGAGGCCGGAGCAGGAACAGGTTGA 3 (0.000091%)

GTTTTTTTCTTCGCAGGATTTTTCTGAGCCTTTTACCACTCCAGCCTAGCCCCTACCCCCCAATTAGGAGG
GCACTGGCCCCCAACAGGCATCACCCCGC 62 (0.001888%)

TAAAAAGCTGGAAGATGGCCCTAAATTCTTGAAGTCTGGTGATGCTGCCATTGTTGATATGGTTCCTGGC
AAGCCCATGTGTGTTGAGAGCTTCTCAGAC 26 (0.000792%)

TAAAAATAAAAAATTATAACAAACCCTGAGAACCAAAATGAACGAAAATCTGTTCGCTTCATTCATTGC
CCCCACAATCCTAGGCCTACCCGCCGCAGTA 38 (0.001157%)

TAAAACCCAGCCCATGACCCCTAACAGGGGCCCTCTCAGCCCTCCTAATGACCTCCGGCCTAGCCATGTG
ATTTCACTTCCACTCCATAACGCTCCTCAT 18 (0.000548%)

TAAACCTAGCCATGGCCATCCCCTTATGAGCGGGCGCAGTGATTATAGGCTTTCGCTCTAAGATTAAAAA
TGCCCTAGCCCACTTCTTACCACAAGGCAC 12 (0.000365%)

TAAACTGAAAGCTGAGCGTGAACGTGGTATCACCATTGATATCTCCTTGTGGAAATTTGAGACCAGCAA
GTACTATGTGACTATCATTGATGCCCCAGGA 16 (0.000487%)

TAAATGCGGTGGCATCGACAAAAGAACCATTGAAAAATTTGAGAAGGAGGCTGCTGAGATGGGAAAGG
GCTCCTTCAAGTATGCCTGGGTCTTGGATAAA 10 (0.000305%)

TAAATTCTTGAAGTCTGGTGATGCTGCCATTGTTGATATGGTTCCTGGCAAGCCCATGTGTGTTGAGAGC
TTCTCAGACTATCCACCTTTGGGTCGCTTT 11 (0.000335%)

TAACAGGGGCCCTCTCAGCCCTCCTAATGACCTCCGGCCTAGCCATGTGATTTCACTTCCACTCCATAAC
GCTCCTCATACTAGGCCTACTAACCAACAC 16 (0.000487%)

TAACTGAACTCCTCACACCCAATTGGACCAATCTATCACCCTATAGAAGAACTAATGTTAGTATAAGTAA
CATGAAAACATTCTCCTCCGCATAAGCCTG 5 (0.000152%)

TAACTTACTACTCCGGAAAAAAAGAACCATTTGGATACATAGGTATGGTCTGAGCTATGATATCAATTGG
CTTCCTAGGGTTTATCGTGTGAGCACACCA 5 (0.000152%)

TAATAATCGGTGCCCCCGATATGGCGTTTCCCCGCATAAACAACATAAGCTTCTGACTCTTACCTCCCTCT
CTCCTACTCCTGCTCGCATCTGCTATAGT 35 (0.001066%)

TAATCGGTGCCCCCGATATGGCGTTTCCCCGCATAAACAACATAAGCTTCTGACTCTTACCTCCCTCTCTC
CTACTCCTGCTCGCATCTGCTATAGTGGA 6 (0.000183%)

TAATCTCCCATATTGTAACTTACTACTCCGGAAAAAAAGAACCATTTGGATACATAGGTATGGTCTGAGC
TATGATATCAATTGGCTTCCTAGGGTTTAT 8 (0.000244%)

TAATGACCTCCGGCCTAGCCATGTGATTTCACTTCCACTCCATAACGCTCCTCATACTAGGCCTACTAACC
AACACACTAACCATATACCAATGATGGCG 5 (0.000152%)

TAATGATGGAAGCAATTTTGGAGGTGGTGGAAGCTACAATGATTTTGGGAATTACAACAATCAGTCTTC
AAATTTTGGACCCATGAAGGGAGGAAATTTT 16 (0.000487%)

TACAAGGTAGTGGGTCGCTGCCTGCCCACCCCCAAATGCCACACGCCGCCCCTCTACCGCATGCGAATCT
TTGCGCCTAATCATGTCGTCGCCAAGTCCC 5 (0.000152%)

TACAATGATTTTGGGAATTACAACAATCAGTCTTCAAATTTTGGACCCATGAAGGGAGGAAATTTTGGAG
GCAGAAGCTCTGGCCCCTATGGCGGTGGAG 3 (0.000091%)

TACACACTGGGTGTGAAACAACTAATTGTCGGTGTTAACAAAATGGATTCCACTGAGCCACCCTACAGC
CAGAAGAGATATGAGGAAATTGTTAAGGAAG 5 (0.000152%)

TACACCAACCACCCAACTATCTATAAACCTAGCCATGGCCATCCCCTTATGAGCGGGCGCAGTGATTATA
GGCTTTCGCTCTAAGATTAAAAATGCCCTA 6 (0.000183%)

TACATACTTCCCCCATTATTCCTAGAACCAGGCGACCTGCGACTCCTTGACGTTGACAATCGAGTAGTAC
TCCCGATTGAAGCCCCCATTCGTATAATAA 4 (0.000122%)

TACGCATCCTTTACATAACAGACGAGGTCAACGATCCCTCCCTTACCATCAAATCAATTGGCCACCAATG
GTACTGAACCTACGAGTACACCGACTACGG 9 (0.000274%)

TACTTCTCCTATCTCTCCCAGTCCTAGCTGCTGGCATCACTATACTACTAACAGACCGCAACCTCAACACC
ACCTTCTTCGACCCCGCCGGAGGAGGAGA 664 (0.020220%)

TAGAAACCGTCTGAACTATCCTGCCCGCCATCATCCTAGTCCTCATCGCCCTCCCATCCCTACGCATCCTT
TACATAACAGACGAGGTCAACGATCCCTC 23 (0.000700%)

TAGAACCAGGCGACCTGCGACTCCTTGACGTTGACAATCGAGTAGTACTCCCGATTGAAGCCCCCATTCG
TATAATAATTACATCACAAGACGTCTTGCA 5 (0.000152%)

TAGATTCGGGCAAGTCCACCACTACTGGCCATCTGATCTATAAATGCGGTGGCATCGACAAAAGAACCA
TTGAAAAATTTGAGAAGGAGGCTGCTGAGAT 4 (0.000122%)

TAGGAATCACCTCCCATTCCGATAAAATCACCTTCCACCCTTACTACACAATCAAAGACGCCCTCGGCTT
ACTTCTCTTCATTCTCTCCTTAATGACATT 10 (0.000305%)

TAGGCACAGCTCTAAGCCTCCTTATTCGAGCCGAGCTGGGCCAGCCAGGCAACCTTCTAGGTAACGACC
ACATCTACAACGTTATCGTCACAGCCCATGC 816 (0.024849%)

TAGGCCTTTTAGAAAACATGGAGTTGTTCCTTTGGCCACATATATGCGAATCTATAAGAAAGGTGATATT
GTAGACATCAAGGGAATGGGTACTGTTCAA 3 (0.000091%)

TAGTAGGCTCCCTTCCCCTACTCATCGCACTGATTTACACTCACAACACCCTAGGCTCACTAAACATTCTA
CTACTCACTCTCACTGCCCAAGAACTATC 8 (0.000244%)

TAGTCCTCATCGCCCTCCCATCCCTACGCATCCTTTACATAACAGACGAGGTCAACGATCCCTCCCTTACC
ATCAAATCAATTGGCCACCAATGGTACTG 3 (0.000091%)

TATAAACCTAGCCATGGCCATCCCCTTATGAGCGGGCGCAGTGATTATAGGCTTTCGCTCTAAGATTAAA
AATGCCCTAGCCCACTTCTTACCACAAGGC 91 (0.002771%)

TATAAATGCGGTGGCATCGACAAAAGAACCATTGAAAAATTTGAGAAGGAGGCTGCTGAGATGGGAAA
GGGCTCCTTCAAGTATGCCTGGGTCTTGGATA 17 (0.000518%)

TATAACTGAACTCCTCACACCCAATTGGACCAATCTATCACCCTATAGAAGAACTAATGTTAGTATAAGT
AACATGAAAACATTCTCCTCCGCATAAGCC 11 (0.000335%)

TATATGTTCTCTAGGCCTTTTAGAAAACATGGAGTTGTTCCTTTGGCCACATATATGCGAATCTATAAGA
AAGGTGATATTGTAGACATCAAGGGAATGG 6 (0.000183%)

TATCAAAAACATGATTACAGGGACATCTCAGGCTGACTGTGCTGTCCTGATTGTTGCTGCTGGTGTTGGT
GAATTTGAAGCTGGTATCTCCAAGAATGGG 8 (0.000244%)

TATCACCCTATAGAAGAACTAATGTTAGTATAAGTAACATGAAAACATTCTCCTCCGCATAAGCCTGCGT
CAGATTAAAACACTGAACTGACAATTAACA 16 (0.000487%)

TATCATTGATGCCCCAGGACACAGAGACTTTATCAAAAACATGATTACAGGGACATCTCAGGCTGACTG
TGCTGTCCTGATTGTTGCTGCTGGTGTTGGT 11 (0.000335%)

TATCCTGCCCGCCATCATCCTAGTCCTCATCGCCCTCCCATCCCTACGCATCCTTTACATAACAGACGAGG
TCAACGATCCCTCCCTTACCATCAAATCA 5 (0.000152%)

TATCTATAAACCTAGCCATGGCCATCCCCTTATGAGCGGGCGCAGTGATTATAGGCTTTCGCTCTAAGAT
TAAAAATGCCCTAGCCCACTTCTTACCACA 33 (0.001005%)

TATCTCCTTGTGGAAATTTGAGACCAGCAAGTACTATGTGACTATCATTGATGCCCCAGGACACAGAGAC
TTTATCAAAAACATGATTACAGGGACATCT 9 (0.000274%)

TATGAGACAGACAGTTGCGGTGGGTGTCATCAAAGCAGTGGACAAGAAGGCTGCTGGAGCTGGCAAGG
TCACCAAGTCTGCCCAGAAAGCTCAGAAGGCT 7 (0.000213%)

TATGCCTGGGTCTTGGATAAACTGAAAGCTGAGCGTGAACGTGGTATCACCATTGATATCTCCTTGTGGA
AATTTGAGACCAGCAAGTACTATGTGACTA 11 (0.000335%)

TATGGCGTTTCCCCGCATAAACAACATAAGCTTCTGACTCTTACCTCCCTCTCTCCTACTCCTGCTCGCAT
CTGCTATAGTGGAGGCCGGAGCAGGAACA 4 (0.000122%)

TATGGTGGTCACCTTTGCTCCAGTCAACGTTACAACGGAAGTAAAATCTGTCGAAATGCACCATGAAGCT
TTGAGTGAAGCTCTTCCTGGGGACAATGTG 6 (0.000183%)

TATGGTTCCTGGCAAGCCCATGTGTGTTGAGAGCTTCTCAGACTATCCACCTTTGGGTCGCTTTGCTGTTC
GTGATATGAGACAGACAGTTGCGGTGGGT48 (0.001462%)

TATGTGACTATCATTGATGCCCCAGGACACAGAGACTTTATCAAAAACATGATTACAGGGACATCTCAG
GCTGACTGTGCTGTCCTGATTGTTGCTGCTG 7 (0.000213%)

TATTACCTCAGAAGTTTTTTTCTTCGCAGGATTTTTCTGAGCCTTTTACCACTCCAGCCTAGCCCCTACCC
CCCAATTAGGAGGGCACTGGCCCCCAACA 8 (0.000244%)

TATTCCTAGAACCAGGCGACCTGCGACTCCTTGACGTTGACAATCGAGTAGTACTCCCGATTGAAGCCCC
CATTCGTATAATAATTACATCACAAGACGT 33 (0.001005%)

TATTCTACACCCTAGTAGGCTCCCTTCCCCTACTCATCGCACTGATTTACACTCACAACACCCTAGGCTCA
CTAAACATTCTACTACTCACTCTCACTGC 6 (0.000183%)

TATTGCCACAACTAACCTCCTCGGACTCCTGCCTCACTCATTTACACCAACCACCCAACTATCTATAAAC
CTAGCCATGGCCATCCCCTTATGAGCGGGC 6 (0.000183%)

TATTTATTACCTCAGAAGTTTTTTTCTTCGCAGGATTTTTCTGAGCCTTTTACCACTCCAGCCTAGCCCCTA
CCCCCCAATTAGGAGGGCACTGGCCCCC 11 (0.000335%)

TCAAAAACATGATTACAGGGACATCTCAGGCTGACTGTGCTGTCCTGATTGTTGCTGCTGGTGTTGGTGA
ATTTGAAGCTGGTATCTCCAAGAATGGGCA 6 (0.000183%)

TCAAACCCGGTATGGTGGTCACCTTTGCTCCAGTCAACGTTACAACGGAAGTAAAATCTGTCGAAATGCA
CCATGAAGCTTTGAGTGAAGCTCTTCCTGG 10 (0.000305%)

TCAAAGCAGTGGACAAGAAGGCTGCTGGAGCTGGCAAGGTCACCAAGTCTGCCCAGAAAGCTCAGAAG
GCTAAATGAATATTATCCCTAATACCTGCCAC 11 (0.000335%)

TCAAGAATGTGTCTGTCAAGGATGTTCGTCGTGGCAACGTTGCTGGTGACAGCAAAAATGACCCACCAA
TGGAAGCAGCTGGCTTCACTGCTCAGGTGAT 6 (0.000183%)

TCAAGACTACCGATGGTTACTTGCTTCGTCTGTTCTGTGTTGGTTTTACTAAAAAACGCAACAATCAGAT
ACGGAAGACCTCTTATGCTCAGCACCAACA 5 (0.000152%)

TCAAGTATGCCTGGGTCTTGGATAAACTGAAAGCTGAGCGTGAACGTGGTATCACCATTGATATCTCCTT
GTGGAAATTTGAGACCAGCAAGTACTATGT 5 (0.000152%)

TCAATGTCAAGAATGTGTCTGTCAAGGATGTTCGTCGTGGCAACGTTGCTGGTGACAGCAAAAATGACC

CACCAATGGAAGCAGCTGGCTTCACTGCTCA 9 (0.000274%)

TCACACCCAATTGGACCAATCTATCACCCTATAGAAGAACTAATGTTAGTATAAGTAACATGAAAACATT
CTCCTCCGCATAAGCCTGCGTCAGATTAAA 8 (0.000244%)

TCACCATTGATATCTCCTTGTGGAAATTTGAGACCAGCAAGTACTATGTGACTATCATTGATGCCCCAGG
ACACAGAGACTTTATCAAAAACATGATTAC 6 (0.000183%)

TCACCCTCCACTTCCCGTCTCAGAATCTAAACGTGGTCACCTTCGAGTAGAGAGGCCCGCCCGCCCACCG
TGGGCAGTGCCACCCGCAGATGACACGCGC 23 (0.000700%)

TCACCTCCCATTCCGATAAAATCACCTTCCACCCTTACTACACAATCAAAGACGCCCTCGGCTTACTTCTC
TTCATTCTCTCCTTAATGACATTAACACT 4 (0.000122%)

TCACCTTCCACCCTTACTACACAATCAAAGACGCCCTCGGCTTACTTCTCTTCATTCTCTCCTTAATGACA
TTAACACTATTCTCACCAGACCTCCTAGG 10 (0.000305%)

TCACCTTTGCTCCAGTCAACGTTACAACGGAAGTAAAATCTGTCGAAATGCACCATGAAGCTTTGAGTGA
AGCTCTTCCTGGGGACAATGTGGGCTTCAA 7 (0.000213%)

TCAGAAGTTTTTTTCTTCGCAGGATTTTTCTGAGCCTTTTACCACTCCAGCCTAGCCCCTACCCCCCAATT
AGGAGGGCACTGGCCCCCAACAGGCATCA 7 (0.000213%)

TCAGACTATCCACCTTTGGGTCGCTTTGCTGTTCGTGATATGAGACAGACAGTTGCGGTGGGTGTCATCA
AAGCAGTGGACAAGAAGGCTGCTGGAGCTG 11 (0.000335%)

TCATCCTAGTCCTCATCGCCCTCCCATCCCTACGCATCCTTTACATAACAGACGAGGTCAACGATCCCTCC
CTTACCATCAAATCAATTGGCCACCAATG 4 (0.000122%)

TCATTGATGCCCCAGGACACAGAGACTTTATCAAAAACATGATTACAGGGACATCTCAGGCTGACTGTG
CTGTCCTGATTGTTGCTGCTGGTGTTGGTGA 3 (0.000091%)

TCCACCACTACTGGCCATCTGATCTATAAATGCGGTGGCATCGACAAAAGAACCATTGAAAAATTTGAG
AAGGAGGCTGCTGAGATGGGAAAGGGCTCCT 9 (0.000274%)

TCCACCTTTGGGTCGCTTTGCTGTTCGTGATATGAGACAGACAGTTGCGGTGGGTGTCATCAAAGCAGTG
GACAAGAAGGCTGCTGGAGCTGGCAAGGTC 5 (0.000152%)

TCCCAGTCCTAGCTGCTGGCATCACTATACTACTAACAGACCGCAACCTCAACACCACCTTCTTCGACCC
CGCCGGAGGAGGAGACCCCATTCTATACCA 5 (0.000152%)

TCCCTCACCAAAGCCCATAAAAATAAAAAATTATAACAAACCCTGAGAACCAAAATGAACGAAAATCTG
TTCGCTTCATTCATTGCCCCCACAATCCTAG 800 (0.024362%)

TCCTAATGACCTCCGGCCTAGCCATGTGATTTCACTTCCACTCCATAACGCTCCTCATACTAGGCCTACTA
ACCAACACACTAACCATATACCAATGATG 4 (0.000122%)

TCCTACCAGGCTTCGGAATAATCTCCCATATTGTAACTTACTACTCCGGAAAAAAAGAACCATTTGGATA
CATAGGTATGGTCTGAGCTATGATATCAAT 4 (0.000122%)

TCCTAGTCCTCATCGCCCTCCCATCCCTACGCATCCTTTACATAACAGACGAGGTCAACGATCCCTCCCTT
ACCATCAAATCAATTGGCCACCAATGGTA 13 (0.000396%)

TCCTATCTCTCCCAGTCCTAGCTGCTGGCATCACTATACTACTAACAGACCGCAACCTCAACACCACCTT
CTTCGACCCCGCCGGAGGAGGAGACCCCAT 3 (0.000091%)

TCCTATTCTACACCCTAGTAGGCTCCCTTCCCCTACTCATCGCACTGATTTACACTCACAACACCCTAGGC
TCACTAAACATTCTACTACTCACTCTCAC 3 (0.000091%)

TCCTCACACCCAATTGGACCAATCTATCACCCTATAGAAGAACTAATGTTAGTATAAGTAACATGAAAAC
ATTCTCCTCCGCATAAGCCTGCGTCAGATT 4 (0.000122%)

TCCTCATCGCCCTCCCATCCCTACGCATCCTTTACATAACAGACGAGGTCAACGATCCCTCCCTTACCATC
AAATCAATTGGCCACCAATGGTACTGAAC 7 (0.000213%)

TCCTCGGACTCCTGCCTCACTCATTTACACCAACCACCCAACTATCTATAAACCTAGCCATGGCCATCCC
CTTATGAGCGGGCGCAGTGATTATAGGCTT 67 (0.002040%)

TCCTGATTGTTGCTGCTGGTGTTGGTGAATTTGAAGCTGGTATCTCCAAGAATGGGCAGACCCGAGAGCA
TGCCCTTCTGGCTTACACACTGGGTGTGAA 12 (0.000365%)

TCCTGCCCGCCATCATCCTAGTCCTCATCGCCCTCCCATCCCTACGCATCCTTTACATAACAGACGAGGTC
AACGATCCCTCCCTTACCATCAAATCAAT 3 (0.000091%)

TCCTGCCTCACTCATTTACACCAACCACCCAACTATCTATAAACCTAGCCATGGCCATCCCCTTATGAGC
GGGCGCAGTGATTATAGGCTTTCGCTCTAA 8 (0.000244%)

TCCTGGCAAGCCCATGTGTGTTGAGAGCTTCTCAGACTATCCACCTTTGGGTCGCTTTGCTGTTCGTGATA
TGAGACAGACAGTTGCGGTGGGTGTCATC 16 (0.000487%)

TCCTGGGCCGCCTGGCGGCCATCGTGGCTAAACAGGTACTGCTGGGCCGGAAGGTGGTGGTCGTACGCT
GTGAAGGCATCAACATTTCTGGCAATTTCTA 8 (0.000244%)

TCCTTCAAGTATGCCTGGGTCTTGGATAAACTGAAAGCTGAGCGTGAACGTGGTATCACCATTGATATCT
CCTTGTGGAAATTTGAGACCAGCAAGTACT 9 (0.000274%)

TCCTTGTGGAAATTTGAGACCAGCAAGTACTATGTGACTATCATTGATGCCCCAGGACACAGAGACTTTA
TCAAAAACATGATTACAGGGACATCTCAGG 7 (0.000213%)

TCGAGGCCATCTCCTGGGCCGCCTGGCGGCCATCGTGGCTAAACAGGTACTGCTGGGCCGGAAGGTGGT
GGTCGTACGCTGTGAAGGCATCAACATTTCT 3 (0.000091%)

TCGATACGGGATAATCCTATTTATTACCTCAGAAGTTTTTTTCTTCGCAGGATTTTTCTGAGCCTTTTACC
ACTCCAGCCTAGCCCCTACCCCCCAATTA 4 (0.000122%)

TCGCAAAGGATCTCCTTCATCCCTCTCCAGAAGAGGAGAAGAGGAAACACAAGAAGAAACGCCTGGTGC
AGAGCCCCAATTCCTACTTCATGGATGTGAA 4 (0.000122%)

TCGCCCTCCCATCCCTACGCATCCTTTACATAACAGACGAGGTCAACGATCCCTCCCTTACCATCAAATC
AATTGGCCACCAATGGTACTGAACCTACGA 3 (0.000091%)

TCGGAATAATCTCCCATATTGTAACTTACTACTCCGGAAAAAAAGAACCATTTGGATACATAGGTATGGT
CTGAGCTATGATATCAATTGGCTTCCTAGG 6 (0.000183%)

TCGGACTCCTGCCTCACTCATTTACACCAACCACCCAACTATCTATAAACCTAGCCATGGCCATCCCCTT
ATGAGCGGGCGCAGTGATTATAGGCTTTCG 17 (0.000518%)

TCGGCTTCCTGCCTCGGAAGCGCAGCAGCAGGCATCGTGGGAAGGTGAAGAGCTTCCCTAAGGATGACC
CGTCCAAGCCGGTCCACCTCACAGCCTTCCT 5 (0.000152%)

TCGGGCAAGTCCACCACTACTGGCCATCTGATCTATAAATGCGGTGGCATCGACAAAAGAACCATTGAA
AAATTTGAGAAGGAGGCTGCTGAGATGGGAA 6 (0.000183%)

TCGGTGCCCCCGATATGGCGTTTCCCCGCATAAACAACATAAGCTTCTGACTCTTACCTCCCTCTCTCCTA
CTCCTGCTCGCATCTGCTATAGTGGAGGC 11 (0.000335%)

TCGTCATTGGACACGTAGATTCGGGCAAGTCCACCACTACTGGCCATCTGATCTATAAATGCGGTGGCAT
CGACAAAAGAACCATTGAAAAATTTGAGAA 3 (0.000091%)

TCGTGAAAACTACCCCTAAAAGCCAAAATGGGAAAGGAAAAGACTCATATCAACATTGTCGTCATTGGA
CACGTAGATTCGGGCAAGTCCACCACTACTG 4 (0.000122%)

TCTAAGCCTCCTTATTCGAGCCGAGCTGGGCCAGCCAGGCAACCTTCTAGGTAACGACCACATCTACAAC
GTTATCGTCACAGCCCATGCATTTGTAATA 10 (0.000305%)

TCTACACCCTAGTAGGCTCCCTTCCCCTACTCATCGCACTGATTTACACTCACAACACCCTAGGCTCACTA
AACATTCTACTACTCACTCTCACTGCCCA 7 (0.000213%)

TCTAGGCCTTTTAGAAAACATGGAGTTGTTCCTTTGGCCACATATATGCGAATCTATAAGAAAGGTGATA
TTGTAGACATCAAGGGAATGGGTACTGTTC 4 (0.000122%)

TCTATAAACCTAGCCATGGCCATCCCCTTATGAGCGGGCGCAGTGATTATAGGCTTTCGCTCTAAGATTA
AAAATGCCCTAGCCCACTTCTTACCACAAG 3 (0.000091%)

TCTATAAATGCGGTGGCATCGACAAAAGAACCATTGAAAAATTTGAGAAGGAGGCTGCTGAGATGGGA
AAGGGCTCCTTCAAGTATGCCTGGGTCTTGGA 22 (0.000670%)

TCTCAAACCCGGTATGGTGGTCACCTTTGCTCCAGTCAACGTTACAACGGAAGTAAAATCTGTCGAAATG
CACCATGAAGCTTTGAGTGAAGCTCTTCCT 46 (0.001401%)

TCTCAGACTATCCACCTTTGGGTCGCTTTGCTGTTCGTGATATGAGACAGACAGTTGCGGTGGGTGTCAT
CAAAGCAGTGGACAAGAAGGCTGCTGGAGC 23 (0.000700%)

TCTCAGCCCTCCTAATGACCTCCGGCCTAGCCATGTGATTTCACTTCCACTCCATAACGCTCCTCATACTA
GGCCTACTAACCAACACACTAACCATATA 17 (0.000518%)

TCTCAGGCTGACTGTGCTGTCCTGATTGTTGCTGCTGGTGTTGGTGAATTTGAAGCTGGTATCTCCAAGA
ATGGGCAGACCCGAGAGCATGCCCTTCTGG 3 (0.000091%)

TCTCCCAGTCCTAGCTGCTGGCATCACTATACTACTAACAGACCGCAACCTCAACACCACCTTCTTCGAC
CCCGCCGGAGGAGGAGACCCCATTCTATAC 3 (0.000091%)

TCTCCTTGTGGAAATTTGAGACCAGCAAGTACTATGTGACTATCATTGATGCCCCAGGACACAGAGACTT
TATCAAAAACATGATTACAGGGACATCTCA 6 (0.000183%)

TCTGAACTATCCTGCCCGCCATCATCCTAGTCCTCATCGCCCTCCCATCCCTACGCATCCTTTACATAACA
GACGAGGTCAACGATCCCTCCCTTACCAT 17 (0.000518%)

TCTGGCTTACACACTGGGTGTGAAACAACTAATTGTCGGTGTTAACAAAATGGATTCCACTGAGCCACCC
TACAGCCAGAAGAGATATGAGGAAATTGTT 18 (0.000548%)

TCTGGTAAAAAGCTGGAAGATGGCCCTAAATTCTTGAAGTCTGGTGATGCTGCCATTGTTGATATGGTTC
CTGGCAAGCCCATGTGTGTTGAGAGCTTCT 12 (0.000365%)

TCTGGTGATGCTGCCATTGTTGATATGGTTCCTGGCAAGCCCATGTGTGTTGAGAGCTTCTCAGACTATCC
ACCTTTGGGTCGCTTTGCTGTTCGTGATA 6 (0.000183%)

TCTGTGCCTCGCTGAGGAAAAATAACTAAACATGGGCAAAGGAGATCCTAAGAAGCCGAGAGGCAAAA
TGTCATCATATGCATTTTTTGTGCAAACTTGT 5 (0.000152%)

TCTGTGTTGGTTTTACTAAAAAACGCAACAATCAGATACGGAAGACCTCTTATGCTCAGCACCAACAGGT
CCGCCAAATCCGGAAGAAGATGATGGAAAT 9 (0.000274%)

TCTGTTCGCTTCATTCATTGCCCCCACAATCCTAGGCCTACCCGCCGCAGTACTGATCATTCTATTTCCCC
CTCTATTGATCCCCACCTCCAAATATCTC 7 (0.000213%)

TCTTGAAGTCTGGTGATGCTGCCATTGTTGATATGGTTCCTGGCAAGCCCATGTGTGTTGAGAGCTTCTCA

GACTATCCACCTTTGGGTCGCTTTGCTGT 29 (0.000883%)

TCTTGGATAAACTGAAAGCTGAGCGTGAACGTGGTATCACCATTGATATCTCCTTGTGGAAATTTGAGAC
CAGCAAGTACTATGTGACTATCATTGATGC 5 (0.000152%)

TGAAAACTACCCCTAAAAGCCAAAATGGGAAAGGAAAAGACTCATATCAACATTGTCGTCATTGGACAC
GTAGATTCGGGCAAGTCCACCACTACTGGCCATCTGATCTATAAATGCGGTGGCATCGACAAAAGAACC
ATTGAAAAATT 75 (0.003403%)

TGAAAGCTGAGCGTGAACGTGGTATCACCATTGATATCTCCTTGTGGAAATTTGAGACCAGCAAGTACTA
TGTGACTATCATTGATGCCCCAGGACACAG 55 (0.001675%)

TGAACGCAGGCACATACTTCCTATTCTACACCCTAGTAGGCTCCCTTCCCCTACTCATCGCACTGATTTAC
ACTCACAACACCCTAGGCTCACTAAACAT 7 (0.000213%)

TGAACGTGGTATCACCATTGATATCTCCTTGTGGAAATTTGAGACCAGCAAGTACTATGTGACTATCATT
GATGCCCCAGGACACAGAGACTTTATCAAA 8 (0.000244%)

TGAACTATCCTGCCCGCCATCATCCTAGTCCTCATCGCCCTCCCATCCCTACGCATCCTTTACATAACAGA
CGAGGTCAACGATCCCTCCCTTACCATCA 3 (0.000091%)

TGAACTCCTCACACCCAATTGGACCAATCTATCACCCTATAGAAGAACTAATGTTAGTATAAGTAACATG
AAAACATTCTCCTCCGCATAAGCCTGCGTC 21 (0.000640%)

TGAAGCGCAAAAGAAGAAAGATGAGGCAGAGGTCCAAGTAAACCGCTAGCTTGTTGCACCGTGGAGGC
CACAGGAGCAGAAACATGGAATGCCAGACGCT 5 (0.000152%)

TGAAGTCTGGTGATGCTGCCATTGTTGATATGGTTCCTGGCAAGCCCATGTGTGTTGAGAGCTTCTCAGA
CTATCCACCTTTGGGTCGCTTTGCTGTTCG 12 (0.000365%)

TGACATCTGACGACGTGAAGGAGCAGATTTACAAACTGGCCAAGAAGGGCCTTACTCCTTCACAGATCG
GTGTAATCCTGAGAGATTCACATGGTGTTGC 164 (0.004994%)

TGACCCCTAACAGGGGCCCTCTCAGCCCTCCTAATGACCTCCGGCCTAGCCATGTGATTTCACTTCCACT
CCATAACGCTCCTCATACTAGGCCTACTAA 6 (0.000183%)

TGACCTCCGGCCTAGCCATGTGATTTCACTTCCACTCCATAACGCTCCTCATACTAGGCCTACTAACCAA
CACACTAACCATATACCAATGATGGCGCGA 11 (0.000335%)

TGACTAGTTCCCCTAATAATCGGTGCCCCCGATATGGCGTTTCCCCGCATAAACAACATAAGCTTCTGAC
TCTTACCTCCCTCTCTCCTACTCCTGCTCG 10 (0.000305%)

TGACTATCATTGATGCCCCAGGACACAGAGACTTTATCAAAAACATGATTACAGGGACATCTCAGGCTG
ACTGTGCTGTCCTGATTGTTGCTGCTGGTGT 10 (0.000305%)

TGACTGTGCTGTCCTGATTGTTGCTGCTGGTGTTGGTGAATTTGAAGCTGGTATCTCCAAGAATGGGCAG
ACCCGAGAGCATGCCCTTCTGGCTTACACA 4 (0.000122%)

TGAGAAGGAGGCTGCTGAGATGGGAAAGGGCTCCTTCAAGTATGCCTGGGTCTTGGATAAACTGAAAGC
TGAGCGTGAACGTGGTATCACCATTGATATC 21 (0.000640%)

TGAGAGCTTCTCAGACTATCCACCTTTGGGTCGCTTTGCTGTTCGTGATATGAGACAGACAGTTGCGGTG
GGTGTCATCAAAGCAGTGGACAAGAAGGCT 6 (0.000183%)

TGAGCGTGAACGTGGTATCACCATTGATATCTCCTTGTGGAAATTTGAGACCAGCAAGTACTATGTGACT
ATCATTGATGCCCCAGGACACAGAGACTTT 9 (0.000274%)

TGAGCTCACCATAGTCTAATAGAAAACAACCGAAACCAAATAATTCAAGCACTGCTTATTACAATTTTAC
TGGGTCTCTATTTTACCCTCCTACAAGCCT 8 (0.000244%)

TGATATCTCCTTGTGGAAATTTGAGACCAGCAAGTACTATGTGACTATCATTGATGCCCCAGGACACAGA GACTTTATCAAAAACATGATTACAGGGACA 4 (0.000122%)

TGATATGGTTCCTGGCAAGCCCATGTGTGTTGAGAGCTTCTCAGACTATCCACCTTTGGGTCGCTTTGCTG TTCGTGATATGAGACAGACAGTTGCGGTG 12 (0.000365%)

TGATGCCCCAGGACACAGAGACTTTATCAAAAACATGATTACAGGGACATCTCAGGCTGACTGTGCTGT CCTGATTGTTGCTGCTGGTGTTGGTGAATTT 32 (0.000974%)

TGATGCTGCCATTGTTGATATGGTTCCTGGCAAGCCCATGTGTGTTGAGAGCTTCTCAGACTATCCACCTT TGGGTCGCTTTGCTGTTCGTGATATGAGA 5 (0.000152%)

TGATGGAAGCAATTTTGGAGGTGGTGGAAGCTACAATGATTTTGGGAATTACAACAATCAGTCTTCAAA TTTTGGACCCATGAAGGGAGGAAATTTTGGA 3 (0.000091%)

TGATGGTCGAGGCCATCTCCTGGGCCGCCTGGCGGCCATCGTGGCTAAACAGGTACTGCTGGGCCGGAA GGTGGTGGTCGTACGCTGTGAAGGCATCAAC 38 (0.001157%)

TGATTACAGGGACATCTCAGGCTGACTGTGCTGTCCTGATTGTTGCTGCTGGTGTTGGTGAATTTGAAGC TGGTATCTCCAAGAATGGGCAGACCCGAGA 5 (0.000152%)

TGATTTTGGGAATTACAACAATCAGTCTTCAAATTTTGGACCCATGAAGGGAGGAAATTTTGGAGGCAG AAGCTCTGGCCCCTATGGCGGTGGAGGCCAA 6 (0.000183%)

TGCCACACGGCTCACATTGCATGCAAGTTTGCTGAGCTGAAGGAAAAGATTGATCGCCGTTCTGGTAAA AAGCTGGAAGATGGCCCTAAATTCTTGAAGT 23 (0.000700%)

TGCCATTGTTGATATGGTTCCTGGCAAGCCCATGTGTGTTGAGAGCTTCTCAGACTATCCACCTTTGGGTC GCTTTGCTGTTCGTGATATGAGACAGACA 27 (0.000822%)

TGCCCGCCATCATCCTAGTCCTCATCGCCCTCCCATCCCTACGCATCCTTTACATAACAGACGAGGTCAA CGATCCCTCCCTTACCATCAAATCAATTGG 4 (0.000122%)

TGCCCTTCTGGCTTACACACTGGGTGTGAAACAACTAATTGTCGGTGTTAACAAAATGGATTCCACTGAG CCACCCTACAGCCAGAAGAGATATGAGGAA 9 (0.000274%)

TGCGACTTGTGTTGGGACTGCTGATAGGAAGATGTCTTCAGGAAATGCTAAAATTGGGCACCCTGCCCCC AACTTCAAAGCCACAGCTGTTATGCCAGATGGTCAGTTTAAAGATATCAGCCTGTCTGACTACAAAGGA AAATATGTTG 91 (0.004129%)

TGCGGTGGCATCGACAAAAGAACCATTGAAAAATTTGAGAAGGAGGCTGCTGAGATGGGAAAGGGCTC CTTCAAGTATGCCTGGGTCTTGGATAAACTGA 10 (0.000305%)

TGCGGTGGGTGTCATCAAAGCAGTGGACAAGAAGGCTGCTGGAGCTGGCAAGGTCACCAAGTCTGCCCA GAAAGCTCAGAAGGCTAAATGAATATTATCC 12 (0.000365%)

TGCTGCCATTGTTGATATGGTTCCTGGCAAGCCCATGTGTGTTGAGAGCTTCTCAGACTATCCACCTTTGG GTCGCTTTGCTGTTCGTGATATGAGACAG 33 (0.001005%)

TGCTGGAGCTGGCAAGGTCACCAAGTCTGCCCAGAAAGCTCAGAAGGCTAAATGAATATTATCCCTAAT ACCTGCCACCCCACTCTTAATCAGTGGTGGA 10 (0.000305%)

TGCTGTCCTGATTGTTGCTGCTGGTGTTGGTGAATTTGAAGCTGGTATCTCCAAGAATGGGCAGACCCGA GAGCATGCCCTTCTGGCTTACACACTGGGT 3 (0.000091%)

TGGAAAGCGGTCTGCCCCTGGAGGTGGTAGCAAGGTTCCACAGAAAAAAGTAAAACTTGCTGCTGATGA AGATGATGACGATGATGATGAAGAGGATGAT 20 (0.000609%)

TGGAAAGTCACCCGTAAGGATGGCAATGCCAGTGGAACCACGCTGCTTGAGGCTCTGGACTGCATCCTA
CCACCAACTCGTCCAACTGACAAGCCCTTGC 83 (0.002528%)

TGGAAGATGGCCCTAAATTCTTGAAGTCTGGTGATGCTGCCATTGTTGATATGGTTCCTGGCAAGCCCAT
GTGTGTTGAGAGCTTCTCAGACTATCCACC 53 (0.001614%)

TGGAAGCAATTTTGGAGGTGGTGGAAGCTACAATGATTTTGGGAATTACAACAATCAGTCTTCAAATTTT
GGACCCATGAAGGGAGGAAATTTTGGAGGC 12 (0.000365%)

TGGAAGCTACAATGATTTTGGGAATTACAACAATCAGTCTTCAAATTTTGGACCCATGAAGGGAGGAAA
TTTTGGAGGCAGAAGCTCTGGCCCCTATGGC 3 (0.000091%)

TGGACAAGAAGGCTGCTGGAGCTGGCAAGGTCACCAAGTCTGCCCAGAAAGCTCAGAAGGCTAAATGA
ATATTATCCCTAATACCTGCCACCCCACTCTT 23 (0.000700%)

TGGACACGTAGATTCGGGCAAGTCCACCACTACTGGCCATCTGATCTATAAATGCGGTGGCATCGACAA
AAGAACCATTGAAAAATTTGAGAAGGAGGCT 23 (0.000700%)

TGGACCAATCTATCACCCTATAGAAGAACTAATGTTAGTATAAGTAACATGAAAACATTCTCCTCCGCAT
AAGCCTGCGTCAGATTAAAACACTGAACTG 5 (0.000152%)

TGGAGACTGGTGTTCTCAAACCCGGTATGGTGGTCACCTTTGCTCCAGTCAACGTTACAACGGAAGTAAA
ATCTGTCGAAATGCACCATGAAGCTTTGAG 28 (0.000853%)

TGGAGCCAAGTGCTAACATGCCTTGGTTCAAGGGATGGAAAGTCACCCGTAAGGATGGCAATGCCAGTG
GAACCACGCTGCTTGAGGCTCTGGACTGCAT 665 (0.020251%)

TGGAGCTGGCAAGGTCACCAAGTCTGCCCAGAAAGCTCAGAAGGCTAAATGAATATTATCCCTAATACC
TGCCACCCCACTCTTAATCAGTGGTGGAAGA 5 (0.000152%)

TGGAGGTGGTGGAAGCTACAATGATTTTGGGAATTACAACAATCAGTCTTCAAATTTTGGACCCATGAA
GGGAGGAAATTTTGGAGGCAGAAGCTCTGGC 5 (0.000152%)

TGGATAAACTGAAAGCTGAGCGTGAACGTGGTATCACCATTGATATCTCCTTGTGGAAATTTGAGACCA
GCAAGTACTATGTGACTATCATTGATGCCCC 8 (0.000244%)

TGGATTGCCACACGGCTCACATTGCATGCAAGTTTGCTGAGCTGAAGGAAAAGATTGATCGCCGTTCTGG
TAAAAAGCTGGAAGATGGCCCTAAATTCTT 646 (0.019672%)

TGGCAAGCCCATGTGTGTTGAGAGCTTCTCAGACTATCCACCTTTGGGTCGCTTTGCTGTTCGTGATATGA
GACAGACAGTTGCGGTGGGTGTCATCAAA34 (0.001035%)

TGGCCCTAAATTCTTGAAGTCTGGTGATGCTGCCATTGTTGATATGGTTCCTGGCAAGCCCATGTGTGTTG
AGAGCTTCTCAGACTATCCACCTTTGGGT 19 (0.000579%)

TGGCGTTTCCCCGCATAAACAACATAAGCTTCTGACTCTTACCTCCCTCTCTCCTACTCCTGCTCGCATCT
GCTATAGTGGAGGCCGGAGCAGGAACAGG 7 (0.000213%)

TGGCTTACACACTGGGTGTGAAACAACTAATTGTCGGTGTTAACAAAATGGATTCCACTGAGCCACCCTA
CAGCCAGAAGAGATATGAGGAAATTGTTAA 3 (0.000091%)

TGGGAATTACAACAATCAGTCTTCAAATTTTGGACCCATGAAGGGAGGAAATTTTGGAGGCAGAAGCTC
TGGCCCCTATGGCGGTGGAGGCCAATACTTT 4 (0.000122%)

TGGGCAGACCCGAGAGCATGCCCTTCTGGCTTACACACTGGGTGTGAAACAACTAATTGTCGGTGTTAAC
AAAATGGATTCCACTGAGCCACCCTACAGC 637 (0.019398%)

TGGGCTTCAATGTCAAGAATGTGTCTGTCAAGGATGTTCGTCGTGGCAACGTTGCTGGTGACAGCAAAA
ATGACCCACCAATGGAAGCAGCTGGCTTCAC 26 (0.000792%)

TGGGTCCCTCGGCTTCCTGCCTCGGAAGCGCAGCAGCAGGCATCGTGGGAAGGTGAAGAGCTTCCCTAA
GGATGACCCGTCCAAGCCGGTCCACCTCACA 4 (0.000122%)

TGGGTCGCTTTGCTGTTCGTGATATGAGACAGACAGTTGCGGTGGGTGTCATCAAAGCAGTGGACAAGA
AGGCTGCTGGAGCTGGCAAGGTCACCAAGTC 6 (0.000183%)

TGGGTCTTGGATAAACTGAAAGCTGAGCGTGAACGTGGTATCACCATTGATATCTCCTTGTGGAAATTTG
AGACCAGCAAGTACTATGTGACTATCATTG 14 (0.000426%)

TGGGTGTGAAACAACTAATTGTCGGTGTTAACAAAATGGATTCCACTGAGCCACCCTACAGCCAGAAGA
GATATGAGGAAATTGTTAAGGAAGTCAGCAC 3 (0.000091%)

TGGTAAAAAGCTGGAAGATGGCCCTAAATTCTTGAAGTCTGGTGATGCTGCCATTGTTGATATGGTTCCT
GGCAAGCCCATGTGTGTTGAGAGCTTCTCA 6 (0.000183%)

TGGTAATGATGGAAGCAATTTTGGAGGTGGTGGAAGCTACAATGATTTTGGGAATTACAACAATCAGTC
TTCAAATTTTGGACCCATGAAGGGAGGAAAT 301 (0.009166%)

TGGTCACCTTTGCTCCAGTCAACGTTACAACGGAAGTAAAATCTGTCGAAATGCACCATGAAGCTTTGAG
TGAAGCTCTTCCTGGGGACAATGTGGGCTT 39 (0.001188%)

TGGTCGAGGCCATCTCCTGGGCCGCCTGGCGGCCATCGTGGCTAAACAGGTACTGCTGGGCCGGAAGGT
GGTGGTCGTACGCTGTGAAGGCATCAACATT 5 (0.000152%)

TGGTGATGCTGCCATTGTTGATATGGTTCCTGGCAAGCCCATGTGTGTTGAGAGCTTCTCAGACTATCCA
CCTTTGGGTCGCTTTGCTGTTCGTGATATG 24 (0.000731%)

TGGTGGTCACCTTTGCTCCAGTCAACGTTACAACGGAAGTAAAATCTGTCGAAATGCACCATGAAGCTTT
GAGTGAAGCTCTTCCTGGGGACAATGTGGG 10 (0.000305%)

TGGTGTTCTCAAACCCGGTATGGTGGTCACCTTTGCTCCAGTCAACGTTACAACGGAAGTAAAATCTGTC
GAAATGCACCATGAAGCTTTGAGTGAAGCT 21 (0.000640%)

TGGTTACTTGCTTCGTCTGTTCTGTGTTGGTTTTACTAAAAAACGCAACAATCAGATACGGAAGACCTCTT
ATGCTCAGCACCAACAGGTCCGCCAAATC 6 (0.000183%)

TGGTTCCTGGCAAGCCCATGTGTGTTGAGAGCTTCTCAGACTATCCACCTTTGGGTCGCTTTGCTGTTCGT
GATATGAGACAGACAGTTGCGGTGGGTGT 5 (0.000152%)

TGTAACTTACTACTCCGGAAAAAAAGAACCATTTGGATACATAGGTATGGTCTGAGCTATGATATCAATT
GGCTTCCTAGGGTTTATCGTGTGAGCACAC 8 (0.000244%)

TGTCAAGAATGTGTCTGTCAAGGATGTTCGTCGTGGCAACGTTGCTGGTGACAGCAAAAATGACCCACC
AATGGAAGCAGCTGGCTTCACTGCTCAGGTG 12 (0.000365%)

TGTCAAGGATGTTCGTCGTGGCAACGTTGCTGGTGACAGCAAAAATGACCCACCAATGGAAGCAGCTGG
CTTCACTGCTCAGGTGATTATCCTGAACCAT 13 (0.000396%)

TGTCATCAAAGCAGTGGACAAGAAGGCTGCTGGAGCTGGCAAGGTCACCAAGTCTGCCCAGAAAGCTCA
GAAGGCTAAATGAATATTATCCCTAATACCT 30 (0.000914%)

TGTCGTCATTGGACACGTAGATTCGGGCAAGTCCACCACTACTGGCCATCTGATCTATAAATGCGGTGGC
ATCGACAAAAGAACCATTGAAAAATTTGAG 7 (0.000213%)

TGTCGTGAAAACTACCCCTAAAAGCCAAAATGGGAAAGGAAAAGACTCATATCAACATTGTCGTCATTG
GACACGTAGATTCGGGCAAGTCCACCACTAC 7 (0.000213%)

TGTCTGTCAAGGATGTTCGTCGTGGCAACGTTGCTGGTGACAGCAAAAATGACCCACCAATGGAAGCAG

CTGGCTTCACTGCTCAGGTGATTATCCTGAA 14 (0.000426%)

TGTGAAACAACTAATTGTCGGTGTTAACAAAATGGATTCCACTGAGCCACCCTACAGCCAGAAGAGATA
TGAGGAAATTGTTAAGGAAGTCAGCACTTAC 12 (0.000365%)

TGTGACTATCATTGATGCCCCAGGACACAGAGACTTTATCAAAAACATGATTACAGGGACATCTCAGGC
TGACTGTGCTGTCCTGATTGTTGCTGCTGGT 17 (0.000518%)

TGTGGAAATTTGAGACCAGCAAGTACTATGTGACTATCATTGATGCCCCAGGACACAGAGACTTTATCA
AAAACATGATTACAGGGACATCTCAGGCTGA 4 (0.000122%)

TGTGTCTGTCAAGGATGTTCGTCGTGGCAACGTTGCTGGTGACAGCAAAAATGACCCACCAATGGAAGC
AGCTGGCTTCACTGCTCAGGTGATTATCCTG 14 (0.000426%)

TGTGTGTTGAGAGCTTCTCAGACTATCCACCTTTGGGTCGCTTTGCTGTTCGTGATATGAGACAGACAGTT
GCGGTGGGTGTCATCAAAGCAGTGGACAA 10 (0.000305%)

TGTGTTGAGAGCTTCTCAGACTATCCACCTTTGGGTCGCTTTGCTGTTCGTGATATGAGACAGACAGTTG
CGGTGGGTGTCATCAAAGCAGTGGACAAGA 5 (0.000152%)

TGTGTTTAAAGTAGTGCTTCTACCAACATGTCCCGTGGTTCCAGCGCCGGTTTTGACCGCCACATTACCAT
TTTTTCACCCGAGGGTCGGCTCTACCAAG 3 (0.000091%)

TGTTCGCTTCATTCATTGCCCCCACAATCCTAGGCCTACCCGCCGCAGTACTGATCATTCTATTTCCCCCT
CTATTGATCCCCACCTCCAAATATCTCAT 5 (0.000152%)

TGTTCTCAAACCCGGTATGGTGGTCACCTTTGCTCCAGTCAACGTTACAACGGAAGTAAAATCTGTCGAA
ATGCACCATGAAGCTTTGAGTGAAGCTCTT 12 (0.000365%)

TGTTCTCTAGGCCTTTTAGAAAACATGGAGTTGTTCCTTTGGCCACATATATGCGAATCTATAAGAAAGG
TGATATTGTAGACATCAAGGGAATGGGTAC 4 (0.000122%)

TGTTGATATGGTTCCTGGCAAGCCCATGTGTGTTGAGAGCTTCTCAGACTATCCACCTTTGGGTCGCTTTG
CTGTTCGTGATATGAGACAGACAGTTGCG 57 (0.001736%)

TTACACACTGGGTGTGAAACAACTAATTGTCGGTGTTAACAAAATGGATTCCACTGAGCCACCCTACAGC
CAGAAGAGATATGAGGAAATTGTTAAGGAA 5 (0.000152%)

TTACACCAACCACCCAACTATCTATAAACCTAGCCATGGCCATCCCCTTATGAGCGGGCGCAGTGATTAT
AGGCTTTCGCTCTAAGATTAAAAATGCCCT 4 (0.000122%)

TTACATAACAGACGAGGTCAACGATCCCTCCCTTACCATCAAATCAATTGGCCACCAATGGTACTGAACC
TACGAGTACACCGACTACGGCGGACTAATC 5 (0.000152%)

TTACCTCAGAAGTTTTTTCTTCGCAGGATTTTTCTGAGCCTTTTACCACTCCAGCCTAGCCCCTACCCCC
CAATTAGGAGGGCACTGGCCCCCAACAGG 10 (0.000305%)

TTACTACTCCGGAAAAAAAGAACCATTTGGATACATAGGTATGGTCTGAGCTATGATATCAATTGGCTTC
CTAGGGTTTATCGTGTGAGCACACCATATA 5 (0.000152%)

TTATTACCTCAGAAGTTTTTTCTTCGCAGGATTTTTCTGAGCCTTTTACCACTCCAGCCTAGCCCCTACCC
CCCAATTAGGAGGGCACTGGCCCCCAAC 17 (0.000518%)

TTCAACTTCACCCGTAACCCACCGCCATGGCCGAGGAAGGCATTGCTGCTGGAGGTGTAATGGACGTTA
ATACTGCTTTACAAGAGGTTCTGAAGACTGC 13 (0.000396%)

TTCAAGCACTGCTTATTACAATTTTACTGGGTCTCTATTTTACCCTCCTACAAGCCTCAGAGTACTTCGAG
TCTCCCTTCACCATTTCCGACGGCATCTA 23 (0.000700%)

TTCAAGTATGCCTGGGTCTTGGATAAACTGAAAGCTGAGCGTGAACGTGGTATCACCATTGATATCTCCT
TGTGGAAATTTGAGACCAGCAAGTACTATG 43 (0.001309%)

TTCAATGTCAAGAATGTGTCTGTCAAGGATGTTCGTCGTGGCAACGTTGCTGGTGACAGCAAAAATGACC
CACCAATGGAAGCAGCTGGCTTCACTGCTC 27 (0.000822%)

TTCACCCTCCACTTCCCGTCTCAGAATCTAAACGTGGTCACCTTCGAGTAGAGAGGCCCGCCCGCCCACC
GTGGGCAGTGCCACCCGCAGATGACACGCG 11 (0.000335%)

TTCATCCCTCTCCAGAAGAGGAGAAGAGGAAACACAAGAAGAAACGCCTGGTGCAGAGCCCCAATTCCT
ACTTCATGGATGTGAAATGCCCAGGATGCTA 3 (0.000091%)

TTCATTGCCCCCACAATCCTAGGCCTACCCGCCGCAGTACTGATCATTCTATTTCCCCCTCTATTGATCCC
CACCTCCAAATATCTCATCAACAACCGAC 4 (0.000122%)

TTCCACCCTTACTACACAATCAAAGACGCCCTCGGCTTACTTCTCTTCATTCTCTCCTTAATGACATTAAC
ACTATTCTCACCAGACCTCCTAGGCGACC 7 (0.000213%)

TTCCCCCATTATTCCTAGAACCAGGCGACCTGCGACTCCTTGACGTTGACAATCGAGTAGTACTCCCGAT
TGAAGCCCCCATTCGTATAATAATTACATC 4 (0.000122%)

TTCCGCGCGCACGTGAAGCACCGTAAAGGCGCTGCGCGCCTGCGCGCCGTGGATTTCGCTGAGCGGCAC
GGCTACATCAAGGGCATCGTCAAGGACATCA 5 (0.000152%)

TTCCTATTCTACACCCTAGTAGGCTCCCTTCCCCTACTCATCGCACTGATTTACACTCACAACACCCTAGG
CTCACTAAACATTCTACTACTCACTCTCA 4 (0.000122%)

TTCCTGGCAAGCCCATGTGTGTTGAGAGCTTCTCAGACTATCCACCTTTGGGTCGCTTTGCTGTTCGTGAT
ATGAGACAGACAGTTGCGGTGGGTGTCAT22 (0.000670%)

TTCGATACGGGATAATCCTATTTATTACCTCAGAAGTTTTTTCTTCGCAGGATTTTTCTGAGCCTTTTACC
ACTCCAGCCTAGCCCCTACCCCCCAATT 6 (0.000183%)

TTCGCATCCGCAAACTCTGTCTCAACATCTGTGTTGGGGAGAGTGGAGACAGACTGACGCGAGCAGCCA
AGGTGTTGGAGCAGCTCACAGGGCAGACCCC 5 (0.000152%)

TTCGCTTCATTCATTGCCCCCACAATCCTAGGCCTACCCGCCGCAGTACTGATCATTCTATTTCCCCCTCT
ATTGATCCCCACCTCCAAATATCTCATCA 4 (0.000122%)

TTCGGAATAATCTCCCATATTGTAACTTACTACTCCGGAAAAAAAGAACCATTTGGATACATAGGTATGG
TCTGAGCTATGATATCAATTGGCTTCCTAG 6 (0.000183%)

TTCGGGCAAGTCCACCACTACTGGCCATCTGATCTATAAATGCGGTGGCATCGACAAAAGAACCATTGA
AAAATTTGAGAAGGAGGCTGCTGAGATGGGA 7 (0.000213%)

TTCTCAAACCCGGTATGGTGGTCACCTTTGCTCCAGTCAACGTTACAACGGAAGTAAAATCTGTCGAAAT
GCACCATGAAGCTTTGAGTGAAGCTCTTCC 8 (0.000244%)

TTCTCAGACTATCCACCTTTGGGTCGCTTTGCTGTTCGTGATATGAGACAGACAGTTGCGGTGGGTGTCA
TCAAAGCAGTGGACAAGAAGGCTGCTGGAG 7 (0.000213%)

TTCTGGCTTACACACTGGGTGTGAAACAACTAATTGTCGGTGTTAACAAAATGGATTCCACTGAGCCACC
CTACAGCCAGAAGAGATATGAGGAAATTGT 6 (0.000183%)

TTCTGGTAAAAAGCTGGAAGATGGCCCTAAATTCTTGAAGTCTGGTGATGCTGCCATTGTTGATATGGTT
CCTGGCAAGCCCATGTGTGTTGAGAGCTTC 22 (0.000670%)

TTCTTGAAGTCTGGTGATGCTGCCATTGTTGATATGGTTCCTGGCAAGCCCATGTGTGTTGAGAGCTTCTC
AGACTATCCACCTTTGGGTCGCTTTGCTG 4 (0.000122%)

TTGAAGTCTGGTGATGCTGCCATTGTTGATATGGTTCCTGGCAAGCCCATGTGTGTTGAGAGCTTCTCAG
ACTATCCACCTTTGGGTCGCTTTGCTGTTC 24 (0.000731%)

TTGAGAAGGAGGCTGCTGAGATGGGAAAGGGCTCCTTCAAGTATGCCTGGGTCTTGGATAAACTGAAAG
CTGAGCGTGAACGTGGTATCACCATTGATAT 27 (0.000822%)

TTGAGACCAGCAAGTACTATGTGACTATCATTGATGCCCCAGGACACAGAGACTTTATCAAAAACATGA
TTACAGGGACATCTCAGGCTGACTGTGCTGT 12 (0.000365%)

TTGAGAGCTTCTCAGACTATCCACCTTTGGGTCGCTTTGCTGTTCGTGATATGAGACAGACAGTTGCGGT
GGGTGTCATCAAAGCAGTGGACAAGAAGGC 8 (0.000244%)

TTGATATCTCCTTGTGGAAATTTGAGACCAGCAAGTACTATGTGACTATCATTGATGCCCCAGGACACAG
AGACTTTATCAAAAACATGATTACAGGGAC 38 (0.001157%)

TTGATATGGTTCCTGGCAAGCCCATGTGTGTTGAGAGCTTCTCAGACTATCCACCTTTGGGTCGCTTTGCT
GTTCGTGATATGAGACAGACAGTTGCGGT 9 (0.000274%)

TTGATGCCCCAGGACACAGAGACTTTATCAAAAACATGATTACAGGGACATCTCAGGCTGACTGTGCTG
TCCTGATTGTTGCTGCTGGTGTTGGTGAATT 7 (0.000213%)

TTGATGGTCGAGGCCATCTCCTGGGCCGCCTGGCGGCCATCGTGGCTAAACAGGTACTGCTGGGCCGGA
AGGTGGTGGTCGTACGCTGTGAAGGCATCAA 6 (0.000183%)

TTGATGTCAAGACTACCGATGGTTACTTGCTTCGTCTGTTCTGTGTTGGTTTTACTAAAAAACGCAACAAT
CAGATACGGAAGACCTCTTATGCTCAGCA 4 (0.000122%)

TTGCCACACGGCTCACATTGCATGCAAGTTTGCTGAGCTGAAGGAAAAGATTGATCGCCGTTCTGGTAAA
AAGCTGGAAGATGGCCCTAAATTCTTGAAG 11 (0.000335%)

TTGCGGTGGGTGTCATCAAAGCAGTGGACAAGAAGGCTGCTGGAGCTGGCAAGGTCACCAAGTCTGCCC
AGAAAGCTCAGAAGGCTAAATGAATATTATC 4 (0.000122%)

TTGGATAAACTGAAAGCTGAGCGTGAACGTGGTATCACCATTGATATCTCCTTGTGGAAATTTGAGACCA
GCAAGTACTATGTGACTATCATTGATGCCC 3 (0.000091%)

TTGGCCGAGTGGAGACTGGTGTTCTCAAACCCGGTATGGTGGTCACCTTTGCTCCAGTCAACGTTACAAC
GGAAGTAAAATCTGTCGAAATGCACCATGA 8 (0.000244%)

TTGGGAATTACAACAATCAGTCTTCAAATTTTGGACCCATGAAGGGAGGAAATTTTGGAGGCAGAAGCT
CTGGCCCCTATGGCGGTGGAGGCCAATACTT 7 (0.000213%)

TTGTAACTTACTACTCCGGAAAAAAAGAACCATTTGGATACATAGGTATGGTCTGAGCTATGATATCAAT
TGGCTTCCTAGGGTTTATCGTGTGAGCACA 23 (0.000700%)

TTGTCGTCATTGGACACGTAGATTCGGGCAAGTCCACCACTACTGGCCATCTGATCTATAAATGCGGTGG
CATCGACAAAAGAACCATTGAAAAATTTGA 3 (0.000091%)

TTGTGGAAATTTGAGACCAGCAAGTACTATGTGACTATCATTGATGCCCCAGGACACAGAGACTTTATCA
AAAACATGATTACAGGGACATCTCAGGCTG 4 (0.000122%)

TTGTGTTTAAAGTAGTGCTTCTACCAACATGTCCCGTGGTTCCAGCGCCGGTTTTGACCGCCACATTACCA
TTTTTTCACCCGAGGGTCGGCTCTACCAA 3 (0.000091%)

TTGTTGATATGGTTCCTGGCAAGCCCATGTGTGTTGAGAGCTTCTCAGACTATCCACCTTTGGGTCGCTTT
GCTGTTCGTGATATGAGACAGACAGTTGC 3 (0.000091%)

TTTACACCAACCACCCAACTATCTATAAACCTAGCCATGGCCATCCCCTTATGAGCGGGCGCAGTGATTA

TAGGCTTTCGCTCTAAGATTAAAAATGCCC 46 (0.001401%)

TTTACATAACAGACGAGGTCAACGATCCCTCCCTTACCATCAAATCAATTGGCCACCAATGGTACTGAAC
CTACGAGTACACCGACTACGGCGGACTAAT 21 (0.000640%)

TTTATCAAAAACATGATTACAGGGACATCTCAGGCTGACTGTGCTGTCCTGATTGTTGCTGCTGGTGTTG
GTGAATTTGAAGCTGGTATCTCCAAGAATG 34 (0.001035%)

TTTATTACCTCAGAAGTTTTTTCTTCGCAGGATTTTTCTGAGCCTTTTACCACTCCAGCCTAGCCCCTACC
CCCCAATTAGGAGGGCACTGGCCCCCAA 8 (0.000244%)

TTTGAGAAGGAGGCTGCTGAGATGGGAAAGGGCTCCTTCAAGTATGCCTGGGTCTTGGATAAACTGAAA
GCTGAGCGTGAACGTGGTATCACCATTGATA 9 (0.000274%)

TTTGAGACCAGCAAGTACTATGTGACTATCATTGATGCCCCAGGACACAGAGACTTTATCAAAAACATG
ATTACAGGGACATCTCAGGCTGACTGTGCTG 41 (0.001249%)

TTTGGAGGTGGTGGAAGCTACAATGATTTTGGGAATTACAACAATCAGTCTTCAAATTTTGGACCCATGA
AGGGAGGAAATTTTGGAGGCAGAAGCTCTG 6 (0.000183%)

TTTGGGAATTACAACAATCAGTCTTCAAATTTTGGACCCATGAAGGGAGGAAATTTTGGAGGCAGAAGC
TCTGGCCCCTATGGCGGTGGAGGCCAATACT 21 (0.000640%)

TTTGGGTCGCTTTGCTGTTCGTGATATGAGACAGACAGTTGCGGTGGGTGTCATCAAAGCAGTGGACAAG
AAGGCTGCTGGAGCTGGCAAGGTCACCAAG 9 (0.000274%)

TTTGTGCCAATTTCTGGTTGGAATGGTGACAACATGCTGGAGCCAAGTGCTAACATGCCTTGGTTCAAGG
GATGGAAAGTCACCCGTAAGGATGGCAATG 17 (0.000518%)

TTTTATTGCCACAACTAACCTCCTCGGACTCCTGCCTCACTCATTTACACCAACCACCCAACTATCTATAA
ACCTAGCCATGGCCATCCCCTTATGAGCG 14 (0.000426%)

TTTTCTTCGCAGGATTTTTCTGAGCCTTTTACCACTCCAGCCTAGCCCCTACCCCCCAATTAGGAGGGCAC
TGGCCCCCAACAGGCATCACCCCGCTAAA 8 (0.000244%)

TTTTGGAGGTGGTGGAAGCTACAATGATTTTGGGAATTACAACAATCAGTCTTCAAATTTTGGACCCATG
AAGGGAGGAAATTTTGGAGGCAGAAGCTCT 5 (0.000152%)

TTTTGGGAATTACAACAATCAGTCTTCAAATTTTGGACCCATGAAGGGAGGAAATTTTGGAGGCAGAAG
CTCTGGCCCCTATGGCGGTGGAGGCCAATAC 5 (0.000152%)

TTTTTATTGCCACAACTAACCTCCTCGGACTCCTGCCTCACTCATTTACACCAACCACCCAACTATCTATA
AACCTAGCCATGGCCATCCCCTTATGAGC 11 (0.000335%)

TTTTTCTTCGCAGGATTTTTCTGAGCCTTTTACCACTCCAGCCTAGCCCCTACCCCCCAATTAGGAGGGCA
CTGGCCCCCAACAGGCATCACCCCGCTAA 12 (0.000365%)

TTTTTTCTTCGCAGGATTTTTCTGAGCCTTTTACCACTCCAGCCTAGCCCCTACCCCCCAATTAGGAGGGC
ACTGGCCCCCAACAGGCATCACCCCGCTA 19 (0.000579%)

TTTTTTTCTTCGCAGGATTTTTCTGAGCCTTTTACCACTCCAGCCTAGCCCCTACCCCCCAATTAGGAGGG
CACTGGCCCCCAACAGGCATCACCCCGCT 39 (0.001188%)

TTTTTTTTTTTTTTTTTTTTTTTTTTTTTTTTTTTTTTTTTTTTTTTTTTTTTTTTTTTTTTTTTTTTTTTTTTTTT
TTTTTTTTTTTTTTTTTTTTTT 2708 (0.082465%)

TTTTTTTTTTTTTTTTTTTTTTTTTTTTTTTTTTTTTTTTTTTTTTTTTTTTTTTTTTTTTTTTTTTTTTTTTTTTTTT
TTTTTTTTTTTTTTTTTTTTTTTTTTTTTTTTTTTTTTTTTTTTTTTTTTTTTTTTTTTTTTTTTTTTTTTT 2093
(0.094968%)

fastp -i NG-A4833_RCH_ACV_siNKX6_3c_libLAO1928_1.fastq.gz -I NG-A4833_RCH_ACV_siNKX6_3c_libLAO1928_2.fastq.gz -o ../../trim/RCH-ACV_siNKX6_3c.R1.fq.gz -O ../../trim/RCH-ACV_siNKX6_3c.R2.fq.gz -q 20 -c -p --thread 4

fastp 0.23.4, at 2025-05-06 08:53:24
